# Association of high-risk sexual behaviour with diversity of the vaginal microbiota and abundance of *Lactobacillus*

**DOI:** 10.1371/journal.pone.0187612

**Published:** 2017-11-02

**Authors:** Jocelyn M. Wessels, Julie Lajoie, Danielle Vitali, Kenneth Omollo, Joshua Kimani, Julius Oyugi, Juliana Cheruiyot, Makubo Kimani, John N. Mungai, Maureen Akolo, Jennifer C. Stearns, Michael G. Surette, Keith R. Fowke, Charu Kaushic

**Affiliations:** 1 McMaster Immunology Research Centre, Michael G. DeGroote Centre for Learning and Discovery, McMaster University, Hamilton, Ontario, Canada; 2 Department of Pathology and Molecular Medicine, McMaster University, Hamilton, Ontario, Canada; 3 Department of Medical Microbiology and Infectious Diseases, University of Manitoba, Winnipeg, Manitoba, Canada; 4 Department of Medical Microbiology, University of Nairobi, Nairobi, Kenya; 5 Kenyan AIDS Control Program, University of Nairobi, Nairobi, Kenya; 6 Department of Medicine, McMaster University, Hamilton, Ontario, Canada; 7 Farncombe Family Digestive Health Institute, McMaster University, Hamilton, Ontario, Canada; 8 Department of Biochemistry and Biomedical Sciences, McMaster University, Hamilton, Ontario, Canada; Massachusetts General Hospital, UNITED STATES

## Abstract

**Objective:**

To compare the vaginal microbiota of women engaged in high-risk sexual behaviour (sex work) with women who are not engaged in high-risk sexual behaviour. Diverse vaginal microbiota, low in *Lactobacillus* species, like those in bacterial vaginosis (BV), are associated with increased prevalence of sexually transmitted infections (STIs) and human immunodeficiency virus (HIV) acquisition. Although high-risk sexual behaviour increases risk for STIs, the vaginal microbiota of sex workers is understudied.

**Methods:**

A retrospective cross-sectional study was conducted comparing vaginal microbiota of women who are not engaged in sex work (non-sex worker controls, NSW, N = 19) and women engaged in sex work (female sex workers, FSW, N = 48), using Illumina sequencing (16S rRNA, V3 region).

**Results:**

Bacterial richness and diversity were significantly less in controls, than FSW. Controls were more likely to have *Lactobacillus* as the most abundant genus (58% vs. 17%; P = 0.002) and composition of their vaginal microbiota differed from FSW (PERMANOVA, P = 0.001). Six microbiota clusters were detected, including a high diversity cluster with three sub-clusters, and 55% of women with low Nugent Scores fell within this cluster. High diversity was observed by 16S sequencing in FSW, regardless of Nugent Scores, suggesting that Nugent Score may not be capable of capturing the diversity present in the FSW vaginal microbiota.

**Conclusions:**

High-risk sexual behaviour is associated with diversity of the vaginal microbiota and lack of *Lactobacillus*. These factors could contribute to increased risk of STIs and HIV in women engaged in high-risk sexual behaviour.

## Introduction

The bacteria of the female genital tract (FGT) are increasingly implicated in reproductive health and disease [1]. While many factors including ethnicity, diet, and cultural background affect the vaginal microbiota [2–7], vaginal health includes a low diversity vaginal microbiota which is predominated by *Lactobacillus* species. Many lactobacilli produce antimicrobial agents including hydrogen peroxide [8] and bacteriocins [9], or co-aggregate generating a microenvironment that competitively excludes pathogens [10], thus contributing to host defense. Presently, the only clinical diagnosis related to the vaginal microbiota is bacterial vaginosis (BV), a polymicrobial condition characterized by low abundance of *Lactobacillus* and overgrowth of anaerobes. Understanding the contribution of BV and microbial diversity to the Human Immunodeficiency Virus (HIV)/AIDS epidemic is increasingly recognized as important. Epidemiologically, BV increases the risk of HIV acquisition in women by approximately 60% [11], and HIV-infected, BV+ women are three times more likely to transmit HIV to their partner [12] than BV-negative women. The mechanism by which BV and/or bacterial diversity affects acquisition and transmission of HIV likely involves a combination of factors including enhanced CD4+ HIV target cell recruitment and disruption of the vaginal epithelial barrier. Several studies reported elevated inflammatory cytokines including IL-1β, IL-1α and IL-8 in the vaginal fluids of BV+ women [13], and greater abundance of activated HIV target cells (CCR5+CD4+ T cells) in the endocervix, compared to women with low bacterial diversity [14]. Highly diverse bacterial communities, particularly those dominated by *Gardnerella vaginalis*, are also associated with proteomic signatures of epithelial barrier disruption [15]. Thus, diversity within the vaginal microbiota has the potential to influence immune responses and integrity of the epithelial barrier, which provides one of the first lines of defence against pathogens.

In addition to other factors [6, 16–19], sexual behaviour affects the vaginal microbiota. Women with more sexual partners, frequent vaginal intercourse and inconsistent condom use were more likely to have day-to-day variation in their vaginal microbiota [2, 20, 21] than controls. Sexual behaviour also modulates inflammatory factors within the genital microenvironment. In Africa, a geographical region with high HIV prevalence, women not engaged in sex work (Non-Sex Workers, NSW) had dampened inflammatory cytokines IL-1α and IL-6, compared to Female Sex Workers (FSW) [22]. Although we know certain sexual behaviours can change the vaginal microbiota and that sex work modifies inflammatory factors within the FGT, there is a paucity of data on how diversity and composition of the vaginal microbiota are affected by sex work. Thus, the aim of this retrospective cross-sectional study was to compare the vaginal microbiota of NSW and FSW living within the same community in Nairobi, Kenya. Herein, we describe bacterial genera in the vaginal microbiota, determine the prevalence of *Lactobacillus* dominant vaginal microbiota, compare alpha and beta diversity metrics between NSW controls and FSW, and examine correlations between bacterial diversity and Nugent Scores.

## Materials and methods

### Study participants

This retrospective cross-sectional study was performed on cervicovaginal lavage collected between January 2015 and April 2016 as part of ongoing longitudinal studies (manuscripts in preparation and, [23, 24]), from HIV negative NSW controls (N = 19) from the Pumwani Community clinics and HIV seronegative FSW (N = 48) enrolled in the Pumwani cohort with <3 years of sex work. The Pumwani Sex Worker cohort was established in 1984 as an open cohort to study sexually transmitted infections (STIs) [25]. Studies were approved by research ethics boards at the Universities of Manitoba and Nairobi/Kenyatta National Hospital. Every woman provided written informed consent, and basic demographic information Table 1. Women were included if they were >18 years, and excluded if pregnant, breastfeeding, or post-menopausal. Urine samples were tested for *Neisseria gonorrhoeae*, and *Chlamydia* species by PCR (Roche Amplicor kits, Hoffmann-La Roche Limited, Mississauga, ON, Canada). HIV serology was performed at the first and last visit for all participants using a rapid test (Determine, Inverness Medical, Japan), and HIV serostatus was confirmed by ELISA (Vironostika, bioMérieux Clinical Diagnostics, Saint-Laurent, QC, Canada). The FSW included in this study were asked to abstain from sex for at least 72 hours, and were tested for prostate-specific antigen to remove non-adherers. At each visit women underwent a gynaecological exam to obtain vaginal specimens for microscopy to assess BV by Nugent Score, yeast infection, and *Trichomonas vaginalis* [26]. Briefly, to determine the Nugent Score, vaginal swabs were smeared on a glass slide, air-dried, Gram stained, and visualized by light microscopy (100X with oil immersion). One technician read and scored all vaginal smears. Women positive for any STI were excluded from the present study and treated according to Kenyan treatment protocols. HIV+ women were referred to care for anti-retroviral therapy (ART).

**Table 1:** Characteristics of non-sex workers and female sex workers.

Total	Non-Sex Workers N = 19	Female Sex Workers N = 48	P
**Mean Age (Range), Years**	29.8 (19–45)	31.1 (21–45)	0.50
Unknown	1	4	
**Marital Status:**			***<0*.*001***
Married and Living with a Man	9 (47%)	0 (0%)	
Married but not Living with a Man	3 (16%)	7 (15%)	
Unmarried but Living with a Man	2 (11%)	0 (0%)	
Unmarried	4 (21%)	37 (77%)	
Unknown	0 (0%)	4 (8%)	
**Menstrual Cycle Stage:**			***0*.*03***
Proliferative	0 (0%)	3 (6%)	
Secretory	1 (5%)	17 (35%)	
Hormonal Contraceptive	8 (42%)	10 (21%)	
Unknown	10 (53%)	18 (38%)	
**Nugent Score (%)**			0.46
0–3	10 (53%)	24 (50%)	
4–6	6 (31%)	15 (31%)	
7–10	3 (16%)	9 (19%)	

### Cervicovaginal lavage collection

A gynecological exam was performed, the vaginal vault was swabbed for microscopy, and then the endocervix was washed with 2mL of sterile 1X phosphate buffered saline (PBS) from a 3mL PBS aliquot. Cervicovaginal lavage was collected from the posterior vaginal fornix, placed in a sterile tube on ice, and sent to the laboratory where it was centrifuged to remove cellular debris. Supernatants and the remaining 1mL of PBS (negative controls) were aliquoted in a biosafety cabinet, frozen, and stored at -80°C until shipped in a liquid nitrogen dry shipper to Winnipeg, Canada. Samples were then shipped on dry ice to Hamilton, Canada for analysis of the vaginal microbiota. The cervicovaginal lavage of NSW were compared with the vaginal microbiota of FSW from the same community.

### DNA extraction and 16S rRNA gene sequencing of the vaginal microbiota

DNA was extracted and purified as described [27], with modifications. Briefly, cervicovaginal lavage were thawed and inverted to mix. 250μl of cervicovaginal lavage was resuspended in ultrapure reagents: 800μl of 200mM monobasic NaPO_4_ (pH 8), 100μl of guanidine thiocyanate-ethylenediaminetetraacetic acid-Sarkosyl, and 0.2g of 0.1mm glass beads (Mo Bio Laboratories, Carlsbad, CA). Samples were mechanically homogenized by bead beater at 3000rpm for 3 minutes, then spun for 5 mins (max speed). DNA extraction was performed using the MagMAX Express-96 Deep Well Magnetic Particle Processor (ThermoFisher Scientific, Burlington, ON, Canada) and MagMax Express DNA Multi-sample Kit (ThermoFisher), following manufacturer’s protocol. Initial steps were modified: 360μl of isopropanol and 400μl of samples were added to the binding plate. Four 1 mL aliquots of PBS that remained following CVL collection (negative controls) were randomly selected and underwent genomic DNA extraction and PCR amplification of the 16S rRNA gene as below.

The hypervariable V3 region of the 16S rRNA gene was amplified by PCR as described [28], using modified 341F and 518R primers. Forward and reverse primers included a unique 6 base pair barcode, allowing for multiplexed amplification using the Illumina PCR platform (Illumina, San Diego, CA). Each reaction contained 5μl 10X PCR buffer (Life Technologies, Burlington, ON, Canada), 1.5μl 50mM MgCl_2_ (Life Technologies), 1μl dNTPs (New England Biolabs, Whitby, ON), 5μl 1μM V3F primer, 5μl 1μM V3R primer, 0.25μl Taq polymerase (Life Technologies), 30ng of template DNA, to a final volume of 50μl with RNase/DNase free water. The PCR consisted of a denaturation step for 2 minutes at 94°C followed by 30 cycles of 94°C, 30 seconds; 50°C, 30 seconds; 72°C, 30 seconds, and 1 cycle of 72°C for 10 minutes. PCR products were sequenced by the McMaster Genomics Facility (Hamilton, ON) using the Illumina MiSeq platform. The negative controls (4 random aliquots of 1mL PBS leftover from CVL collection) did not yield any PCR products for the 16S rRNA, and thus bacterial contamination during sample collection, handling, processing, extraction, and PCR was considered to be negligible.

### Data processing and statistical analysis

Sequences were processed using an in-house data pipeline (M.G.S.) [27]. Sequences surpassing the V3 region length were trimmed using Cutadapt [29], and paired-ends sequences were aligned using PANDAseq [30]. Sequences were clustered and binned at 97% similarity into operational taxonomic units (OTUs) using AbundantOTU+ [31], and taxonomy was assigned using the Ribosomal Database Project classifier [32] and GreenGenes (February 4, 2011) [33]. Quantitative Insights Into Microbial Ecology (QIIME) [34] was used to calculate alpha diversity (including singletons) and summarize OTU abundance tables to the genus level.

OTU counts, Shannon Diversity Index [35], and Chao1 Richness Estimates [36] were graphed and statistically analyzed using GraphPad Prism (GraphPad Software Inc., La Jolla, CA). Data are presented as mean ± standard error of measurement (SEM). Taxonomic bar charts by relative abundance of bacterial taxa, Bray-Curtis dissimilarity Principle Coordinate Analyses (PCoAs), and heatmaps were generated using phyloseq [37] in R version 3.2.3 (R Core Team, 2015). Permutational multivariate analysis of variance using distance matrices was performed using the adonis function, vegan package [38], to statistically compare microbial populations (β diversity) between groups [39]. The number of clusters present in the data was assessed using k-means clustering, clusGap function [40] in the cluster package [41]. A cluster dendrogram was constructed using the Bray-Curtis dissimilarity distance and hclust function in the stats package, and modified using FigTree v1.4.3 (http://tree.bio.ed.ac.uk/software/figtree/). In order to estimate species for the heatmap, the most abundant sequence per OTU was queried against NCBI’s 16S rRNA gene database using megaBLAST, as described by Hummelen et al., 2010 [42]. The highest scoring species (>97% identity and coverage) was selected as the putative identity of that OTU. Most of the scores displayed 100% identity and coverage (17/20), sequences were classified at the genus level if 100% identity and coverage was not met (2/20). As bacterial vaginosis associated bacteria 1 (BVAB1) has previously been misclassified during assignment of taxonomy as belonging to the *Shuttleworthia* genus [43, 44] we aligned our sequence for OTU 5 (“*Shuttleworthia*”) to two of the previously published BVAB-1 sequences (NCBI GenBank AY724739.1; AY959097.1) [45–47] using Clustal Omega (http://www.ebi.ac.uk/Tools/msa/clustalo/) and found 100% identity and coverage with both BVAB1 sequences (S1 Fig). We therefore re-classified OTU 5 as BVAB-1 for the remainder of the analyses.

Categorical variables (marital status, menstrual cycle stage, BV status, *Lactobacillus* as the most abundant genera, community state type (CST) [48]) were compared between groups by Fisher’s Exact Test or Chi-square (SigmaStat 3.5 Systat Software Inc., Chicago, IL, USA). Continuous variables (age, OTUs, Shannon Diversity Index, Chao1) were statistically compared across groups by student’s t-test for normal data, Mann-Whitney Rank Sum Test for data that were not normally distributed (Graphpad Software Inc.), or by one-way ANOVA (parametric) or Kruskal-Wallis one-way ANOVA (non-parametric). For all tests, P<0.05 was considered significant.

### Data

Metadata, relative abundance table (genus level, 0–1), and OTU table (count) can be found in S1 Table. Additional data provided includes sequence reads per sample S2 Table, observed species S3 Table, Chao1 Richness Estimates S4 Table, Shannon Diversity Index S5 Table and representative sequences by OTU (S2 Fig).

## Results

### Study sample

Cervicovaginal lavage was available for 67 women (19 NSW, 48 FSW). Average age (29.8 range 19–45 vs. 31.1, range 21–45; P = 0.502) and prevalence of BV (P = 0.455) did not differ between NSW and FSW Table 1. Menstrual cycle phase (P = 0.032) and marital status (P = <0.001) differed between groups. Multiple logistic regression was conducted using ‘*Lactobacillus* abundant’ or ‘not *Lactobacillus* abundant’ as the dependent variable and marital status and cycle stage as independent variables. In this model, neither marital status (P = 0.172) nor cycle stage (P = 0.183) were significantly associated with having *Lactobacillus* as the dominant genus in the vaginal microbiota.

### Non-Sex workers have less bacterial diversity in their vaginal microbiota than sex workers

**Fig 1:**
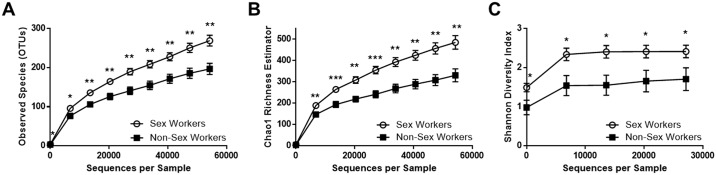
Non-Sex workers have less bacterial diversity in their vaginal microbiota than sex workers. Three alpha-diversity metrics were used to compare bacterial richness and diversity within the vaginal microbiota of Non-Sex Workers (NSW, N = 19) as compared to Female Sex Workers (FSW, N = 48) living in the same geographical location. NSW had a significantly less operational taxonomic units (OTUs, an approximation of the number of observed species) than FSW when sequences were rarefied to a depth of 10, 13572, 20353, 27134, 33915, 40696, 47477, and 54288 sequence reads (A). The Chao1 estimated bacterial richness was found to be significantly less in NSW than FSW at 6791, 13572, 20353, 27134, 33915, 40696, 47477, and 54288 sequence reads (B). The Shannon Diversity Index was also significantly lower in NSW than FSW at 10, 6791, 13572, 20353, and 27134 sequence reads (C). NSW (black squares), FSW (open circles). *: P<0.05, **: P< 0.01, *** P< 0.001. Data is presented as mean ± SEM.

Bacterial diversity was assessed using three alpha-diversity metrics: observed species (observed bacterial richness), Chao1 (estimated bacterial richness), and Shannon Diversity Index (estimated evenness and richness). The vaginal microbiota of NSW controls had significantly less observed and estimated (Chao1) OTUs than FSW, seen in rarefaction curves (Fig 1A and 1B). Bacterial diversity (Shannon Diversity Index), was also significantly less for the vaginal microbiota of NSW than FSW (Fig 1C).

### Non-Sex workers have significantly different vaginal microbiota than sex workers and are more likely to be *Lactobacillus* abundant

**Fig 2:**
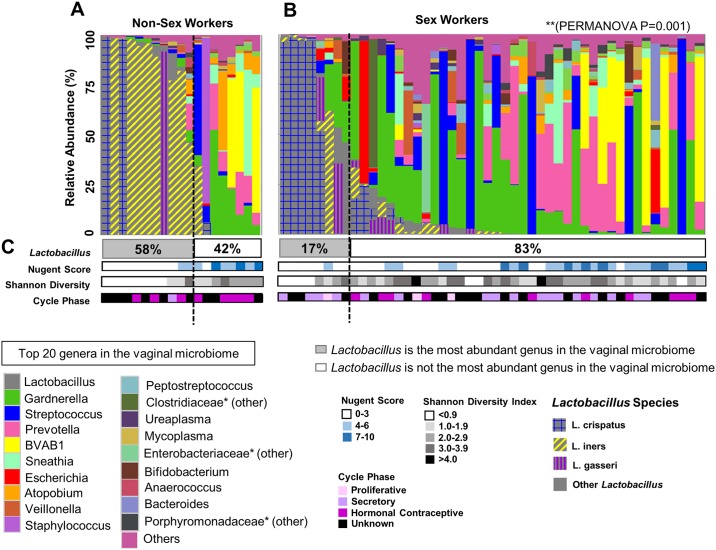
Non-Sex workers are more likely to have *Lactobacillus* as the most abundant genus in their vaginal microbiota. The top 20 bacterial genera in the vaginal microbiota were plotted by relative abundance as taxa bar charts and compared between Non-Sex Workers (NWS, N = 19) (A) and Female Sex Workers (FSW, N = 48) (B). Each bar represents the vaginal microbiota of one woman. Each colour represents a different genus of bacteria, as indicated in the legend. Species of *Lactobacillus* are indicated in grey/coloured patterns as per legend. Vaginal microbiota are ordered left to right in descending order of the relative abundance of *Lactobacillus*, and women to the left of the dashed lines have *Lactobacillus* as the most abundant genus in their vaginal microbiota (proportion listed as the percentage in the grey box) (C). NSW were significantly more likely (P = 0.002) to have *Lactobacillus* as the most abundant genus in their vaginal microbiota (A) as compared to FSW (B). Pairwise PERMANOVA revealed significant differences in the composition (β-diversity) of the NSW vs. FSW (PERMANOVA P = 0.001) vaginal microbiota (A vs. B). Nugent score, Shannon Diversity (at 6791 reads) and menstrual cycle stage are indicated below the taxa bar chart (C). None of the women with a Nugent Score 7–10 had *Lactobacillus* as the most abundant genus in their vaginal microbiota. *: Resolved to family level.

The top 20 genera were plotted as taxa bar charts. NSW (Fig 2A) were more likely (P = 0.002) to have *Lactobacillus* as the most abundant genus in their vaginal microbiota (11/19; 58%) compared with FSW (8/48; 17%) (Fig 2B). A nonparametric PERMANOVA [39] partitioned heterogeneity in microbial composition (β-diversity) between the vaginal microbiota of NSW and FSW. The composition of the vaginal microbiota was significantly different between NSW and FSW (PERMANOVA P = 0.001). Nugent Score, Shannon Diversity at 6791 reads (depth selected for maximal sample retention) and menstrual cycle stage are indicated below the taxa bar chart (Fig 2C). None of the women with a Nugent Score 7–10 had *Lactobacillus* as the most abundant genus in their vaginal microbiota Table 2.

**Table 2:** Proportion of women with *Lactobacillus* dominant or highly diverse vaginal microbiota (by Nugent Score) and proportion of women in each community state type (by group).

	All Women—Nugent Score			P
	0–3	4–6	7–10	
Lactobacillus Dominant (CST^#^ I, II, III)	15 (45%)	4 (18%)	0 (0%)	***0*.*005***
High Diversity Dominant (CST IV)	18 (55%)	18 (82%)	12 (100%)	
Total	33	22	12	
	**NSW**^%^	**FSW**^$^	**P**	
*L*. *crispatus* Dominant (CST I)	2 (10.5%)	5 (10.4%)	1.000	
*L*. *gasseri* Dominant (CST II)	1 (5.3%)	1 (2.1%)	0.490	
*L*. *iners* Dominant (CST III)	8 (42.1%)	1 (2.1%)	***<0*.*001***	
High Diversity Dominant (CST IV)	8 (42.1%)	41 (85.4%)	***<0*.*001***	
Total	19	48		

^#^CST: community state type

^$^FSW: female sex workers

^%^NSW: non-sex workers

### Vaginal microbiota cluster by relative abundance of *Lactobacillus*

**Fig 3:**
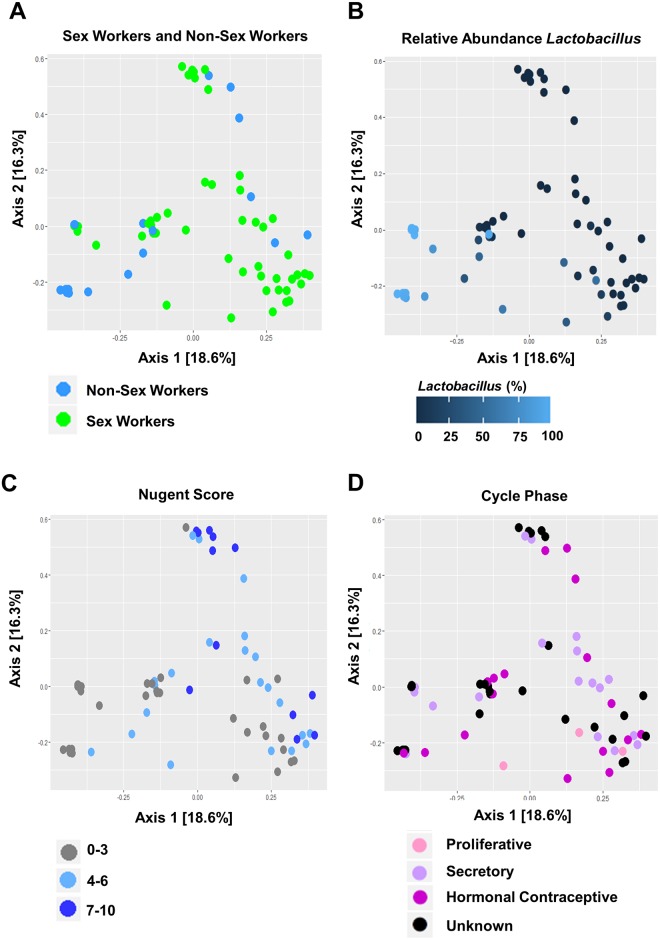
Principle coordinate analysis demonstrates clustering by relative abundance of *Lactobacillus*. PCoA plots demonstrate the beta-diversity of the vaginal microbiota of Non-Sex Workers (NSW, N = 19), and Female Sex Workers (FSW, N = 48). The vaginal microbiota did not appear to cluster by group (NSW vs. FSW; A). When coloured by the relative abundance of *Lactobacillus* one main cluster dominant in *Lactobacillus* were revealed (B). The stronger intensity of blue indicates greater relative abundance. Some clustering by Nugent Score (C) was also observed (0–3: grey, 4–6: light blue, 7–10: dark blue). The vaginal microbiota did not appear to strongly cluster by menstrual cycle phase (D) (Proliferative: light pink, Secretory: light purple, Hormonal Contraceptives: purple, Unknown: black). Axes = eigenvalues, a metric whose magnitude indicates the amount of variation captured in the PCoA axis.

**Fig 4:**
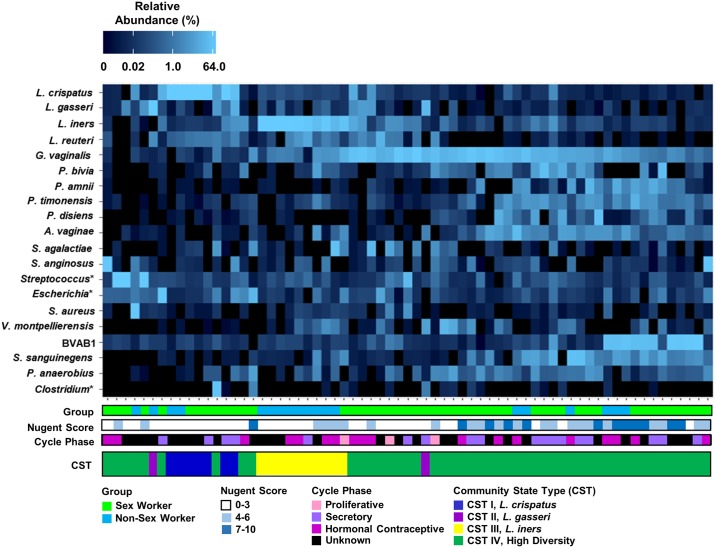
Heatmap reveals clustering patterns based on community state types. A heatmap of the top 20 species based on Bray-Curtis dissimilarity distance and PCoA ordination revealed that the vaginal microbiota (columns) of Non-Sex Workers (NSW), and Female Sex Workers (FSW) did not cluster independently, but clustered by community state type (CST). CSTI consisted of women who were *L*. *crispatus* dominant (blue, N = 7/67), CSTII were women who were *L*. *gasseri* dominant (purple, N = 2/67), CSTIII were *L*. *iners* dominant (yellow, N = 10/67), and CSTIV was women with highly diverse vaginal microbiota (green, N = 48/67). All of the women with Nugent Scores 7–10 (dark blue) clustered together in the most diverse CST, regardless of which group (NSW, or FSW) they belonged to. None of the women who were *L*. *crispatus*, or *L*. *gasseri* dominant had Nugent scores between 4 and 6, but 4 of the women who were *L*. *iners* dominant had Nugent scores between 4 and 6. Group, Nugent Score, menstrual cycle phase, and CST are listed below each column. *: resolved to bacterial genus.

Principle coordinate analysis (PCoA) (Fig 3) was performed to determine if vaginal microbiota clustered by group (Fig 3A), relative abundance of *Lactobacillus* (Fig 3B), Nugent Score (Fig 3C), or menstrual cycle phase (Fig 3D). Vaginal microbiota did not tightly cluster by group, nor did they appear to strongly cluster by menstrual cycle phase. Some clustering appeared to relate to the relative abundance of *Lactobacillus*, and Nugent Score. A non-hierarchial heatmap of the top 20 species, based on Bray-Curtis dissimilarity and PCOA ordination, was generated to resolve clustering patterns between the vaginal microbiota of NSW, and FSW (Fig 4). Unsupervised clustering revealed the vaginal microbiota of NSW and FSW did not cluster independently, but clustered by CST as previously reported [48].

### *Lactobacillus iners* is prevalent in Non-Sex workers while high diversity is prevalent in sex workers

CSTI consisted of women with *L*. *crispatus* dominant vaginal microbiota (N = 7/67), CSTII were *L*. *gasseri* dominant (N = 2/67), CSTIII were *L*. *iners* dominant (N = 10/67), and CSTIV had highly diverse vaginal microbiota (N = 48/67) (Fig 4). CSTV (*L*. *jensenii* dominant) was not observed in this study. In the heatmap, all of the BV+ women clustered in CSTIV, regardless of which group they belonged to. None of the *L*. *crispatus*, or *L*. *gasseri* dominant women had Nugent Scores between 4 and 6, but 4 of the *L*. *iners* dominant women had Nugent Scores between 4 and 6. The proportion of women in each CST was compared by group Table 2, and more FSW had highly diverse vaginal microbiota (CSTIV) than NSW (P<0.001), and *L*. *iners* was more prevalent in the vaginal microbiota of NSW than FSW (P<0.001).

### Six vaginal microbiota clusters identified

**Fig 5:**
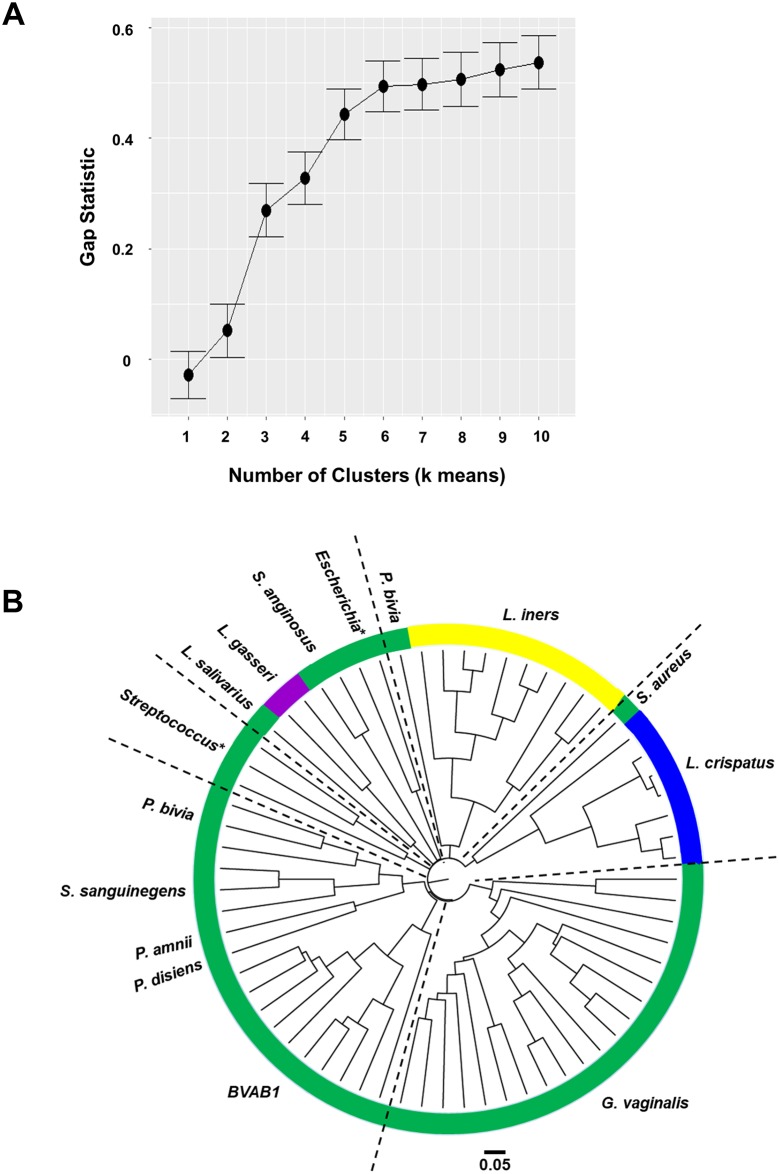
Six distinct vaginal microbiota clusters are found within this group of women. The gap statistic, which gives an estimation of how well ‘k’ (number of clusters) fits the data, was calculated (A). The number of clusters present in the data is indicated by the plateau in the gap statistic, which occurred at 6. A cluster dendrogram was created in order to better visualize the clusters (B). Three sub-clusters within community state type (CST) IV (green, high diversity) were identified. One sub-cluster had *Streptococcus* as the dominant genus, another had *G*. *vaginalis*, while the third had *Prevotella/Sneathia/BVAB1* and was more diverse in terms of the dominant species. In general, the previously described CSTs clustered together within the dendrogram. CSTI (blue): *L*. *cripatus* dominant, CSTII (purple): *L*. *gasseri* dominant, CSTIII (yellow): *L*. *iners* dominant, CSTIV (green): highly diverse. *: resolved to bacterial genus.

After finding clustering by CST, we mathematically validated the number of clusters within the data. The gap statistic, estimation of how well ‘k’ (number of clusters) fits the data, was calculated (Fig 5A). The number of clusters, indicated by a plateau in gap statistic, occurred at six. To visualize, a cluster dendrogram (Fig 5B), PCoA by CST and heatmap delineating the 6 clusters (S3 Fig) were created. Two *Lactobacillus* dominant clusters (*L*. *crispatus*, *L*. *iners*), one containing other *Lactobacillus* species and other genera (*L*. *gasseri*, *L*. *salivarius*, *S*.*anginosus*, *Escherichia*), and another high diversity CSTIV cluster consisting of three sub-clusters were identified. One sub-cluster was *Streptococcus* dominant, another *G*. *vaginalis* dominant, while the third (*Prevotella/Sneathia/BVAB1*) was more diverse. In general, previously described CSTs [48] clustered together within the dendrogram, and others have identified similar sub-clusters within CSTIV by hierarchical clustering [49].

### Diversity of the vaginal microbiota may not always be linked to Nugent Score

**Fig 6:**
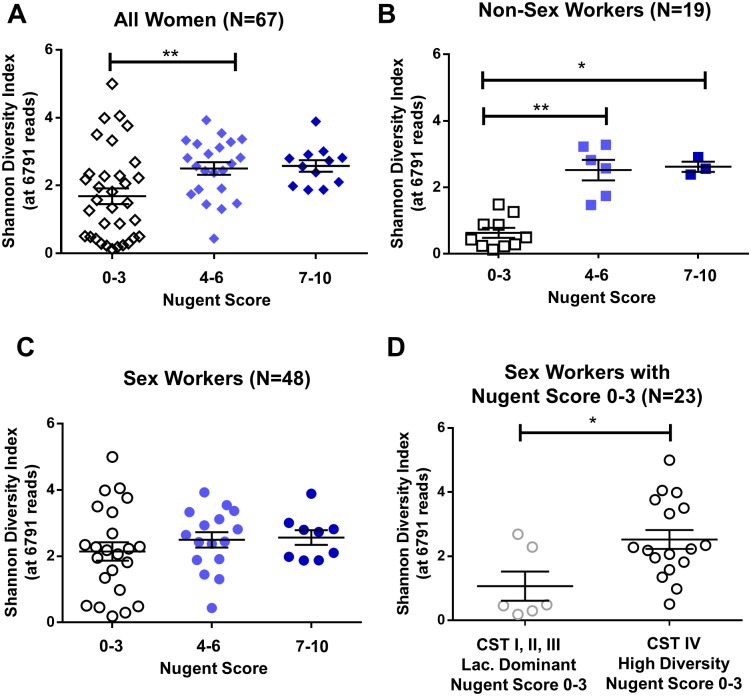
Diversity of the vaginal microbiota may not be linked to Nugent Score in FSW. There was significantly greater bacterial diversity (P = 0.0067) in the women with Nugent Scores of 4–6 as compared to those with Nugent Scores of 0–3 when the relationship between diversity and Nugent Score was examined including all 67 women (Non-Sex Workers (NSW) and Female Sex Workers (FSW)) (A). When only NSW (N = 19) were included in the analysis, similar relationships were found, where Shannon Diversity was significantly greater in NSW with Nugent Score of 4–6 and 7–10 versus those with scores of 0–3 (P = <0.0001) (B). However, when only FSW were included in the analysis (N = 48) there were no significant differences in the Shannon Diversity Index (6791 reads) between FSW with Nugent Scores of 0–3, 4–6, or 7–10 (P = 0.5051) (C). When FSW were stratified by community state type (CST) (*Lactobacillus* dominant vs. high diversity), the majority of the FSW with Nugent Scores 0–3 were in CSTIV and had significantly greater bacterial (17/23) diversity than FSW in CSTI, II, and III who also had Nugent Scores of 0–3 (6/23) (P = 0.0348) (D). BV: bacterial vaginosis, CST: community state type. *: P<0.05, **: P< 0.01. Data is presented as mean ± SEM.

As 55% of the women with low Nugent Scores (0–3) clustered within CSTIV Table 2, we questioned whether Nugent Score was always associated with bacterial diversity. When all 67 women were included in the analysis, we found women with Nugent Scores of 4–6 had greater diversity than women with Nugent Scores of 0–3 (2.502±0.18 vs. 1.686±0.23; N = 22, 33 respectively; P = 0.0067) (Fig 6A), and there was no difference between women with Nugent Scores 4–6 as compared to 7–10 (N = 12), as previously reported [50]. Similar relationships between Nugent Score and diversity were found in NSW (0.6322±0.15 Nugent Score 0–3 vs. 2.520±0.31 Nugent Score 4–6 vs. 2.620±0.15 Nugent Score 7–10; N = 3, 6, 10 respectively; P = <0.0001) (Fig 6B). Conversely, when only FSW were included (N = 48), no significant difference in Shannon Diversity was observed (2.144±0.28 Nugent Score 0–3 vs. 2.495±0.23 Nugent Score 4–6 vs. 2.564±0.22 Nugent Score 7–10; N = 23, 16, 9 respectively; P = 0.5051) (Fig 6C), suggesting the vaginal microbiota of FSW as described by 16S sequencing is diverse, regardless of Nugent Score. When FSW with Nugent Scores of 0–3 were stratified by CST we found 74% (17/23) were classified as highly diverse. Not surprisingly these FSW had greater bacterial diversity than the FSW with Nugent Scores 0–3 in CSTI-III (Fig 6D, 2.524±0.30 vs. 1.067±0.45; N = 17, 6 respectively; P = 0.0348). Thus, in these FSW, diversity of the vaginal microbiota was not linked to Nugent Score, and diversity appeared to exist in the absence of elevated Nugent Scores.

**Fig 7:**
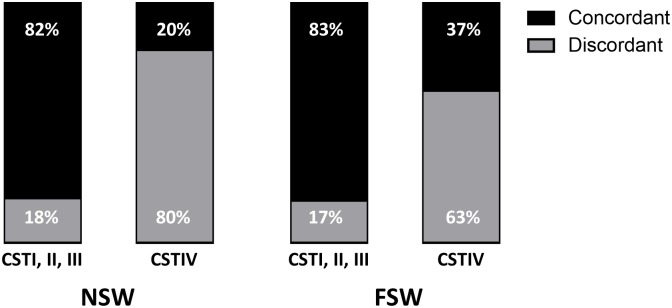
Concordance between bacterial morphotypes observed during Nugent Scoring and bacterial genera described by 16S sequencing. The top 5 bacterial genera for each woman were enumerated and compared to the raw bacterial morphotype scores (*Lactobacillus* morphotypes, *G*. *vaginalis/Bacteroides* morphotypes, *Mobiluncus* morphotypes) obtained during Nugent Scoring. The 16S and bacterial morphotypes were considered to be in complete agreement if the top 5 genera obtained by 16S sequencing included all organisms viewed by microscopy. Overall concordance rate for NSW was 63% (10/16) complete agreement, and 44% (18/41) complete agreement for FSW. After stratifying by Community State Type (CST) (*Lactobacillus* dominant vs. high diversity), concordance rates were found to be related to CST. Concordance rates were greater for women in CSTI, II, III as compared to CSTIV, regardless of whether they were NSW or FSW (9/11, 82% vs. 1/5, 20% in NSW and 5/6, 83% vs. 13/35, 37% in FSW). CST: community state type. FSW: Female Sex Worker. NSW: Non Sex Worker.

In order to better understand the apparent lack of correlation between bacterial diversity by 16S sequencing and Nugent Score, the top 5 bacterial genera for each woman were enumerated and compared to the raw bacterial morphotype scores (*Lactobacillus* morphotypes, *G*. *vaginalis/Bacteroides* morphotypes, *Mobiluncus* morphotypes) obtained during Nugent Scoring S1 Table, similar to methods employed in Hong et al., 2016 [51]. The 16S and bacterial morphotypes were considered to be in complete agreement if the top 5 genera obtained by 16S sequencing included all organisms viewed by microscopy (Agreement column, S1 Table). When all women were considered, there was 49% complete agreement between bacterial morphotypes as assessed by Nugent Score and the top 5 16S bacterial genera. However, when only NSW were considered there was a 63% (10/16) complete agreement, while there was a 44% (18/41) complete agreement for FSW. Our concordance rate of 63% for NSW was similar to the concordance rate reported by Hong et al., 2016 [51] where concordance between next generation sequencing (NGS) and bacterial cultures obtained from vaginal swabs was reported to be 73%. Upon closer examination of concordant and discordant pairs (Fig 7), we suspect that the diverse range of genera present in the high diversity dominant CSTIV is likely to be misclassified or missed during Nugent Scoring. This may in part explain the low concordance rate (44% (18/41) complete agreement between 16S and Nugent Scoring) in FSW, a group in which 85.4% of women were found to have vaginal microbiota belonging to CSTIV (high diversity).

## Discussion

Here we demonstrate that the vaginal microbiota of NSW is less rich in bacterial species and diversity than the vaginal microbiota of FSW. We found NSW were more likely to have *Lactobacillus* as the most abundant genus in their vaginal microbiota, important considering vaginal health centres around a low diversity, *Lactobacillus* dominant vaginal microbiota. Results also revealed six vaginal microbiota clusters based on the abundance of *Lactobacillus*, matching previously described CSTs [48]. Similar to recent studies [49], sub-clusters were identified within CSTIV. When women were divided by Nugent Score, NSW followed the conventional pattern where Nugent Score was associated with bacterial diversity, however FSW did not follow this pattern (Fig 6B vs. 6C). No significant difference in bacterial diversity was observed between FSW with Nugent Scores of 0–3, 4–6, or 7–10. Furthermore, the majority of FSW with Nugent Scores of 0–3 (17/23) fell into the high diversity CSTIV, while only few (6/23) had low diversity correlating with low Nugent Scores. These results indicate that in some women microbial diversity may not necessarily correlate to Nugent Score.

Unlike the highly diverse gut microbiota, the vaginal microbiota is typically low in microbial diversity, and abundant in *Lactobacillus* species. However, the dominant bacteria vary by ethnicity, where 80–90% of Caucasian and Asian women are *Lactobacillus* dominant compared to 60% of Black and Hispanic women [48]. This suggests 40% of Black and Hispanic women do not consistently match the current definition of a healthy vaginal microbiota, even though many are clinically asymptomatic for vaginal or reproductive disease. In the present study, 58% of Kenyan NSW had *Lactobacillus* as the most abundant genus in their vaginal microbiota, compared with only 17% of FSW. Furthermore, FSW had greater species richness and diversity than NSW of the same ethnic background, even though there was no difference in the proportion of women with Nugent Scores 7–10, 4–6, or 0–3 Table 1. It is increasingly evident that cervicovaginal bacteria regulate inflammation and inflammatory responses in the FGT [14, 52], and that diverse vaginal microbiota low in *Lactobacillus* species are associated with increased prevalence of STIs and risk of HIV acquisition [49, 52]. The proposed mechanism of enhanced STI/HIV susceptibility is via the induction of pro-inflammatory cytokines (MIP-1α, MIP-1β, IL-1α, IL-1β, TNF-α, IFN-γ, IL-10, IL-8, IL-23, and IL-17) and recruitment of HIV-1 target cells [14, 52], and recent clinical studies demonstrated a 17 fold increase in HIV target cells (CD4+CCR5+CD38+HLA-DR+) in the cervix of women with highly diverse vaginal microbiota, compared to *L*. *crispatus* dominant women [52]. Further, in a prospective study the rate of HIV acquisition was 4 times greater in South African women with high diversity of the vaginal microbiota as compared to *L*. *crispatus* dominant women [52]. Thus, this study demonstrating that FSW have increased diversity of the vaginal microbiota and decreased abundance of lactobacilli, indicates the possibility that diversity of the microbiota may confer additional risk of STI acquisition in these women who are already at a high-risk [21].

Additionally we found microbial diversity is consistently high in the vaginal microbiota of FSW, regardless of Nugent Score. These data indicate that in this specific and understudied group of women, FSW with Nugent Scores 7–10 tend to have high diversity of the vaginal microbiota, but that FSW with high microbial diversity in their vaginal microbiota do not necessarily have elevated Nugent Scores. This suggests that vaginal microbial diversity might exist in the absence of BV (as assessed by Nugent Score), or that the bacterial morphotypes of some of the bacterial genera present in the diverse vaginal microbiota might be being misidentified during Nugent Scoring, while they are found to be different genera by deep sequencing. While we demonstrate that in FSW diversity of the vaginal microbiota exists in the absence of elevated Nugent Scores, another group has described a similar phenomenon in a small proportion of NSW. In their examination of the temporal dynamics of the vaginal microbiota, Gajer et al., 2012 demonstrate that 6 of the 32 (19%) women in their study with vaginal microbiota belonging to the high diversity CST, had persistently low Nugent Scores [6]. Further, they highlight other women with persistently elevated Nugent Scores, but without symptoms of BV [6]. Additionally, several women in Srinivasan et al., 2012 are identified as BV- by Amsel and/or Nugent Scoring, but have high diversity in their bacterial taxonomic profiles [50]. Upon further examination of the women in our study whose bacterial morphotypes visualized during Nugent Scoring were discordant with those observed during 16S sequencing Fig 7 and S1 Table, we suggest that the diverse range of genera present in the high diversity dominant CSTIV is likely to be misclassified or missed during Nugent Scoring. This may in part explain the low concordance rate (44% (18/41) complete agreement between 16S and Nugent Scoring) in FSW, a group in which 85.4% of women were found to have vaginal microbiota belonging to CSTIV (high diversity). This hypothesis is supported by other groups who suggest that the Nugent Score should be refined and revised to include additional categories of bacteria particularly those associated with BV [53] or an ‘other morphotypes’ category, who have demonstrated that morphotypes are often hard to microscopically assign [53, 54], have shown that major discrepancies in scoring can occur depending on quantity of bacteria on the slide [53, 55], and have highlighted the fact that Gram negative rods are often misclassified as *Mobiluncus* morphotypes during Nugent Scoring and are more likely to be BVAB1 [56]. This was apparent in our dataset, where women with BVAB1 often had a score of 3 for *Mobiluncus* morphotypes even though *Mobiluncus* was not among the top 5 bacterial genera identified by 16S sequencing S1 Table. While high concordance between the presence of *L*. *crispatus* by culture and sequencing and *Lactobacillus* morphotypes observed following a Gram stain has been demonstrated, the association between *L*. *iners* and *Lactobacillus* morphotypes is not as clear; where *L*. *iners* has been shown to contribute to other categories of bacterial morphotypes during Nugent Scoring [56]. This is important considering the prevalence of *L*. *iners* in African women. Another study by Verhelst et al., suggests adding an additional category to the Nugent score, to account for the fact that a number of vaginal smears in their study were misclassified as being abundant in lactobacilli (Gram positive rods) during microscopy, due to the abundance of *Bifidobacteria* which are also Gram positive rods [53]. Thus, not only can lactobacilli be misclassified as other bacterial morphotypes, but other bacterial genera can be misclassified as lactobacilli. Our results demonstrating a lack of *Lactobacillus* dominance in FSW and the apparent inability of the Nugent Score to capture bacterial diversity in women calls into question how a healthy vaginal microbiota should be defined in these women. Considering only 17% of the FSW were found to have *Lactobacillus* as the most abundant genus in the vaginal microbiota future studies should examine measures of vaginal health in these women to determine if those with high diversity have vaginal symptoms or whether the diversity can exist in the absence of symptoms. Taken together, we suggest that there is a need to re-examine the relationship between bacterial diversity and BV in the FSW population, and further studies are needed to assess the effectiveness of the Nugent Score in assessing BV in this group. This will be useful as diversity of the FSW vaginal microbiota might affect their susceptibility to STIs.

In our cohort of Kenyan women, we identified six distinct vaginal microbiota clusters. Similar to other studies [6, 48, 49] we identified four of the previously identified vaginal community state types (CST) with 10% of the women having a vaginal microbiota dominated by *L*. *crispatus* (CST I), 3% *L*. *gasseri* (CST II) dominant, 13% *L*. *iners* dominant (CST III), and 74% were a diverse mix of bacterial taxa (CST IV), which contained three sub-clusters that generally grouped based on the dominant bacterial taxon present (*Streptococcus*, *G*. *vaginalis*, and *Prevotella/Sneathia/BVAB1*). In a few publications since Ravel et al., 2011, similar sub-clusters within the high diversity community state type (CST IV) have been identified by hierarchical clustering [49, 57, 58]. One of the interesting observations in the current study was that a vaginal microbiota with *L*. *iners* as the dominant bacterial species was more common among NSW controls than FSW in Kenya. Previous studies showed that vaginal microbiota dominant in *L*. *iners* tend to be more prevalent in African women and women of African descent [59–62], including those who are HIV+ [42, 60], and here we find this community state type to be more prevalent in Kenyan women who are not engaged in sex work. Vaginal microbiota dominant in *L*. *iners* have been shown to be more unstable and conducive to transitions towards abnormal vaginal flora and BV [63] than other species of lactobacilli. Additionally, *L*. *iners* can be found in women with BV, and has been shown to have protective features, similar to other lactobacilli, but also to have pathogenic features (reviewed in [64]). Perhaps what has occurred in the FSW cohort is that women whose vaginal microbiota were *L*. *iners* dominant prior to the initiation of sex work have transitioned to more diverse vaginal microbiota as they become exposed to sex partners and practices, while those who were initially dominated by the more stable *L*. *crispatus* remained so. Furthermore, the existence of diversity in the absence of elevated Nugent Scores within the FSW cohort leads us to speculate that the vaginal mucosal immune system might become tolerized to sex work and bacterial diversity over time. Prospective studies designed to conduct longitudinal profiling of the FSW vaginal microbiota as women initiate sex work and continue into full time sex work would be necessary to explore this hypothesis. In contrast to African women, the most common profile of vaginal microbiota in Caucasian women tends to be the *L*. *crispatus* dominant vaginal microbiota [48, 61], which has been associated with stability of the vaginal microflora [63]. It has been suggested that a vaginal microbiota rich in *L*. *crispatus* is protective against invading pathogens due to its production of lactic acid, anti-microbial and anti-inflammatory mediators [9, 22, 65–68], while *L*. *iners* appears to be less efficient in providing non-specific protection against pathogens, and might itself participate in cellular damage via synthesis of cytolysins [69]. In addition to reports that the presence of *L*. *iners* in the vaginal microbiota is linked with perturbations within the vaginal microbiota [70, 71], a recent study demonstrated that the prevalence of STIs in women who were *L*. *iners* dominant was higher than in those who were *L*. *crispatus* dominant [49]. *In vitro* studies suggest that *L*. *iners* induces the expression of multiple transcription factors, pro-inflammatory cytokines, and signalling via the pattern recognition receptors in a 3D culture of vaginal epithelial cells, while *L*. *crispatus* remains more immunologically inert [72]. This difference in dominant species of *Lactobacillus* may in part explain the biological susceptibility that African women and women of African origin appear to have in relation to the increased risk and prevalence of STIs and BV as compared to other ethnicities [73–75].

The present study was limited due to its retrospective nature. In this study we analyzed 67 cervicovaginal lavages which were not used in the original study. These samples were the only samples available for this study. Additionally, because of the retrospective nature of the study we were unable to obtain exact number of sex partners, condom use, and vaginal douching practices to correlate with these samples, since this information was not collected as part of the original study. These factors can influence the vaginal microbiota [2, 20], and may be different between NSW and FSW. Although there was no obvious correlation between hormonal contraceptive use and bacterial diversity, our study was not designed to examine this link. Furthermore, information regarding contraceptive use was not available for many women in the present study, making it difficult to draw conclusions regarding the effect of hormonal contraceptives on the vaginal microbiota. Future studies that are specifically designed to address this issue need to be conducted given the paucity of data in the literature currently surrounding the effects of hormonal contraceptives on the vaginal microbiota. Although we did not have the information on sex partners, condom use, and vaginal douching for these particular samples, other studies conducted on the same cohort are informative. Douching is common in Kenyan women, but a previous study in the Pumwani cohort found douching rates in NSW and FSW were equivalent (100%) [76]. Additionally, FSW are counselled on the importance of condom use in preventing STIs, thus condom use is close to 100% [24]. The mean number of clients for FSW was 4 (±3) and regular partners 2 (±2), while NSW had only an average of 1 (±2.1) regular partners [76]. Thus douching rates are equivalent, condom use and number of partners is high in this group of FSW. Another report found that recent sex (in the last 72 hours) decreased the presence of *L*. *crispatus*, *L*. *vaginalis*, and the *Lactobacillus* genus [62], which could affect the vaginal microbiota of FSW. However, the FSW included in this study were asked to abstain from sex for at least 72 hours, and were tested at sample collection for prostate-specific antigen to remove non-adherers. Thus, recent sex is not likely to be the sole explanation for the decreased proportion of *Lactobacillus* species seen in these FSWs as compared to NSW. As this was a retrospective study the samples from NSWs were not collected following 72 hours of abstinence. However, data collected in the study showed that NSW in this study had intercourse an average of 1.3±0.2 times in the week preceding sample collection. Furthermore, because recent sex was found to decrease *Lactobacillus* species [62], if anything, the difference in the proportion of women with *Lactobacillus* as the most abundant genus in their vaginal microbiota (58% NSW vs. 17% FSW) would be even greater if the NSW had been asked to abstain prior to sample collection. Taken together, it seems likely that the bacterial diversity observed in FSW is related to a greater number of sex partners. Additionally, due to its cross-sectional design, we are unable to discern whether alterations in the vaginal microbiota of FSW are permanent or transient in nature. Furthermore, while increased vaginal diversity in FSW suggests their vaginal tract might have an inflammatory microenvironment and greater susceptibility to STIs, we could not examine these correlations due to the retrospective study design. Longitudinal studies would address these important questions more comprehensively.

In conclusion, results indicate that the vaginal microbiota of the majority of FSW is richer in bacterial diversity and lacks *Lactobacillus* dominance as compared to NSW. Recent studies demonstrate that cervicovaginal bacteria regulate inflammation and inflammatory responses in the FGT [14, 52], and reveal an association between high diversity vaginal microbiota low in *Lactobacillus*, and the prevalence and acquisition of STIs including HIV [49, 52]. Thus, results suggest that microbial diversity and lack of *Lactobacillus* species may be the underlying factors that could contribute to STI susceptibility in FSW. Furthermore, results also indicate microbial diversity appears to be consistently high in FSW, and suggests the need to re-examine the ability of the Nugent Score to accurately capture bacterial diversity in the FSW cohort. Further studies are required to address these important leads. Finally, these results provide insight into the vaginal microbiota of NSW controls and FSW controls, and how this might relate to STI and HIV susceptibility.

## Supporting information

S1 Fig Multiple sequence alignment BVAB1. Clustal Omega was employed to align two Bacterial Vaginosis Associated Bacteria 1 (BVAB1) sequences found in the literature to OTU5 from our dataset. There was 100% consensus between our sequence and the two previously reported (AY724739.1 and AY959097.1).
(PDF)

S2 Fig Representative 16S sequences by OTU. List of representative 16S sequences, listed by OTU. len: length. tot-seq: total number of sequences.
(PDF)

S3 Fig Principle coordinate analysis demonstrates CSTs and sub-clusters. PCoA plot demonstrate the beta-diversity of the vaginal microbiota of Non-Sex Workers (NSW, N = 19), and Female Sex Workers (FSW, N = 48). The vaginal microbiota clustered by Community State Type (CST) which included 3 sub-clusters within CSTIV. Clusters are coloured by CST. Sub-clusters within CSTIV are circled in green. CSTI: blue circle, CSTII: purple circle, CSTIII: yellow circle, CSTIV: green circles. Axes = eigenvalues, a metric whose magnitude indicates the amount of variation captured in the PCoA axis.
(PDF)

S1 Table Metadata, relative abundance table, and OTU table. Data table containing sample metadata, list of top 5 bacterial genera identified by 16S sequencing of vaginal microbiota, bacterial morphotypes visualized during Nugent Scoring, and 16S sequencing results (relative abundance table at genus level, OTU table). BV: bacterial vaginosis, CST: community state type, FSW: female sex worker, KOH: potassium hydroxide, NSW: non-sex worker, WBC: white blood cells.
(XLSX)

S2 Table 16S sequences per sample. List of the number of 16S reads per sample obtained by Illumina sequencing.
(XLSX)

S3 Table Observed species. Table containing observed species rarefactions and iterations.
(XLSX)

S4 Table Chao1 Richness Estimates. Table containing Chao1 Richness rarefactions and iterations.
(XLSX)

S5 Table Shannon Diversity Index. Table containing Shannon Diversity rarefactions and iterations.
(XLSX)
