# Supplementary material for: Association of high-risk sexual behaviour with diversity of the vaginal microbiota and abundance of Lactobacillus
Source: PLoS One. 2017 Nov 2;12(11):e0187612. doi: 10.1371/journal.pone.0187612 (PMC5667760; doi:10.1371/journal.pone.0187612)
Supplement: S1 Fig — Clustal Omega was employed to align two Bacterial Vaginosis Associated Bacteria 1 (BVAB1) sequences found in the literature to OTU5 from our dataset. There was 100% consensus between our sequence and the two previously reported (AY724739.1 and AY959097.1). (PDF) [file pone.0187612.s001.pdf]

```
CLUSTAL O(1.2.4) multiple sequence alignment


AY724739.1      ------------------------------------------------------------ 0
AY959097.1      TTCGCCCTTAGAGTTTGATCCTGGCTCAGGATGAACGCTGGCGGCGTGCTTAACACATGC 60
OTU5            ------------------------------------------------------------ 0


AY724739.1      ------------------------------------------------------------ 0
AY959097.1      AAGTCGAACGAAGTGCTACGACGGAAGTTTTCGGACGGAAGATGTAGTTACTTAGTGGCG 120
OTU5            ------------------------------------------------------------ 0


AY724739.1      ------------------------------------------------------------ 0
AY959097.1      GACGGGTGAGTAACGCGTGGGGAACCTGCCCTGTACCGGGGGATAGCAGCCGGAAACGGC 180
OTU5            ------------------------------------------------------------ 0


AY724739.1      ------------------------------------------------------------ 0
AY959097.1      TGATAATACCGCATAAGCGCACGAATGTCGCATGACATGGTGTGAAAAACTCCGGTGGTA 240
OTU5            ------------------------------------------------------------ 0


AY724739.1      ------------------------------------------------------------ 0
AY959097.1      TAGGATGGACCCGCGTCTGATTAGCCGGTTGGTGGGGTAAAAGCCTACCAAAGCGAAGAT 300
OTU5            ------------------------------------------------------------ 0


AY724739.1      ------------------------------------------------------------ 0
AY959097.1      CAGTAGCCGAGTTGAGAGACTGACCGGCCACATTGGGACTGAGACACGGCCCAGACTCCT 360
OTU5            ------------------------------------------------------------ 0


AY724739.1      --------------TGGGGAATATTGCACAATGGGCGAAAGCCTGATGCAGCGACGCCGC 46
AY959097.1      ACGGGAGGCAGCAGTGGGGAATATTGCACAATGGGCGAAAGCCTGATGCAGCGACGCCGC 420
OTU5            --------------TGGGGAATATTGCACAATGGGCGAAAGCCTGATGCAGCGACGCCGC 46
                              **********************************************

AY724739.1      GTGAGCGAAGAAGTATTTCGGTATGTAAAGCTCTATCAGAAGGGAAGAAAATGACGGTAC 106
AY959097.1      GTGAGCGAAGAAGTATTTCGGTATGTAAAGCTCTATCAGAAGGGAAGAAAATGACGGTAC 480
OTU5            GTGAGCGAAGAAGTATTTCGGTATGTAAAGCTCTATCAGAAGGGAAGAAAATGACGGTA- 105
                *********************************************************** 

AY724739.1      CTTACTAAGAAGCTCCGGCTAAATACGTGCCAGCAGCCGCGGTAATACGTATGGAGCAAG 166
AY959097.1      CTTACTAAGAAGCTCCGGCTAAATACGTGCCAGCAGCCGCGGTAATACGTATGGAGCAAG 540
OTU5            ------------------------------------------------------------ 105


AY724739.1      CGTTATCCGGATTTACTGGGTGTAAAGGGAGTGTAGGCGGCACTATAAGTCTGATGTGAA 226
AY959097.1      CGTTATCCGGATTTACTGGGTGTAAAGGGAGTGTAGGCGGCACTATAAGTCTGATGTGAA 600
OTU5            ------------------------------------------------------------ 105


AY724739.1      AACCTAAGGCTTAACCATAGGATTGCATTGGAAACTGTAGAGCTGGAGTATCGGAGAGGC 286
AY959097.1      AACCTAAGGCTTAACCATAGGATTGCATTGGAAACTGTAGAGCTGGAGTATCGGAGAGGT 660
OTU5            ------------------------------------------------------------ 105


AY724739.1      AAGCGGAATTCCTGGTGTAGTGGTGAAATACGTAGATATCAGGAAGAACATCGGTGGCGA 346
AY959097.1      AAGCGGAATTCCTGGTGTAGTGGTGAAATACGTAGATATCAGGAAGAACATCGGTGGCGA 720
OTU5            ------------------------------------------------------------ 105
```

```
AY724739.1      AGGCGGCTTGCTGGACGATAACTGACGCTAAGGCTCGAAAGCGTGGGAAGCGAACAGGAT 406
AY959097.1      AGGCGGCTTGCTGGACGATAACTGACGCTAAGGCTCGAAAGCGTGGGAAGCGAACAGGAT 780
OTU5            ------------------------------------------------------------ 105

AY724739.1      TAGATACCCTGGTAGTCCACGCTGTAAACGATGAACACTAGGTGTTGGGAGGCTAAGCCT 466
AY959097.1      TAGATACCCTGGTAGTCCACGCTGTAAACGATGAACACTAGGTGTTGGGAGGCTAAGCCT 840
OTU5            ------------------------------------------------------------ 105

AY724739.1      TTCAGTGCCGCAGCAAACGCAATAAGTGTTCCACCTGGGGAGTACGTTCGCAAGAATGAA 526
AY959097.1      TTCAGTGCCGCAGCAAACGCAATAAGTGTTCCACCTGGGGAGTACGTTCGCAAGAATGAA 900
OTU5            ------------------------------------------------------------ 105

AY724739.1      ACTCAAAGGAATTGACGGGGACCCGCACAAGCGGTGGAGCATGTGGTTTAATTCGAAGCA 586
AY959097.1      ACTCAAAGGAATTGACGGGGACCCGCACAAGCGGTGGAGCATGTGGTTTAATTCGAAGCA 960
OTU5            ------------------------------------------------------------ 105

AY724739.1      ACGCGAAGAACCTTACCAAGTCTTGACATCCCTGTGACAGTATATGTAATGTATATTTTC 646
AY959097.1      ACGCGAAGAACCTTACCAAGTCTTGACATCCCTGTGACAGTATATGTAATGTATATTTTC 1020
OTU5            ------------------------------------------------------------ 105

AY724739.1      TACGGAACACAGGAGACAGGTGGTGCATGGTTGTCGTCAGCTCGTGTCGTGAGATGTTGG 706
AY959097.1      TACGGAACACAGGAGACAGGTGGTGCATGGTTGTCGTCAGCTCGTGTCGTGAGATGTTGG 1080
OTU5            ------------------------------------------------------------ 105

AY724739.1      GTTAAGTCCCGCAACGAGCGCAACCCTTGTACTTAGTAGCCAGCATTAAGGTGGGCACTC 766
AY959097.1      GTTAAGTCCCGCAACGAGCGCAACCCTTGTACTTAGTAGCCAGCATTAAGGTGGGCACTC 1140
OTU5            ------------------------------------------------------------ 105

AY724739.1      TAAGTAGACTGCCGGGGTGAACCCGGAGGAAGGTGGGGATGACGTCAAATCATCATGCCC 826
AY959097.1      TAAGTAGACTGCCGGGGTGAACCCGGAGGAAGGTGGGGACGACGTCAAATCATCATGCCC 1200
OTU5            ------------------------------------------------------------ 105

AY724739.1      CTTATGACTTGGGCTACACACGTGCTACAATGGCGTGAACAGAGGGAAGCGAAGGAGCGA 886
AY959097.1      CTTATGGCTTGGGCTACACACGTGCTACAATGGCGTGAACAGAGGGAAGCGAAGGAGCGA 1260
OTU5            ------------------------------------------------------------ 105

AY724739.1      TCCGGAGCAAATCTCATAAAGCACGTCCCAGTTCGGACTGCAGTCTGCAACTCGACTGCA 946
AY959097.1      TCCGGAGCAAATCTCATAAAGCACGTCCCAGTTCGGACTGCAGTCTGCAACTCGACTGCA 1320
OTU5            ------------------------------------------------------------ 105

AY724739.1      CGAAGCTGGAATCGCTAGTAATCGCAGATCATCATGCTGCGGTGAATACGTTCCCGGGTC 1006
AY959097.1      CGAAGCTGGAATCGCTAGTAATCGCAGATCATCATGCTGCGGTGAATACGTTCCCGGGTC 1380
OTU5            ------------------------------------------------------------ 105

AY724739.1      T----------------------------------------------------------- 1007
AY959097.1      TTGTACACACCGCCCGTCACACCATGGGAGTCTGGAATGCCCGAAGTCAGTGGCCAAACC 1440
OTU5            ------------------------------------------------------------ 105

AY724739.1      -------------------------------------------    1007
```

```
AY959097.1      GAAAGGATGGAGCTGCCGAAGGCAGGCCCGGTAACTGGGTGAA  1483
OTU5            -------------------------------------------  105
```
