# Supplementary material for: Association of high-risk sexual behaviour with diversity of the vaginal microbiota and abundance of Lactobacillus
Source: PLoS One. 2017 Nov 2;12(11):e0187612. doi: 10.1371/journal.pone.0187612 (PMC5667760; doi:10.1371/journal.pone.0187612)
Supplement: S3 Fig — PCoA plot demonstrate the beta-diversity of the vaginal microbiota of Non-Sex Workers (NSW, N = 19), and Female Sex Workers (FSW, N = 48). The vaginal microbiota clustered by Community State Type (CST) which included 3 sub-clusters within CSTIV. Clusters are coloured by CST. Sub-clusters within CSTIV are circled in green. CSTI: blue circle, CSTII: purple circle, CSTIII: yellow circle, CSTIV: green circles. Axes = eigenvalues, a metric whose magnitude indicates the amount of variation captured in the PCoA axis. (PDF) [file pone.0187612.s003.pdf]

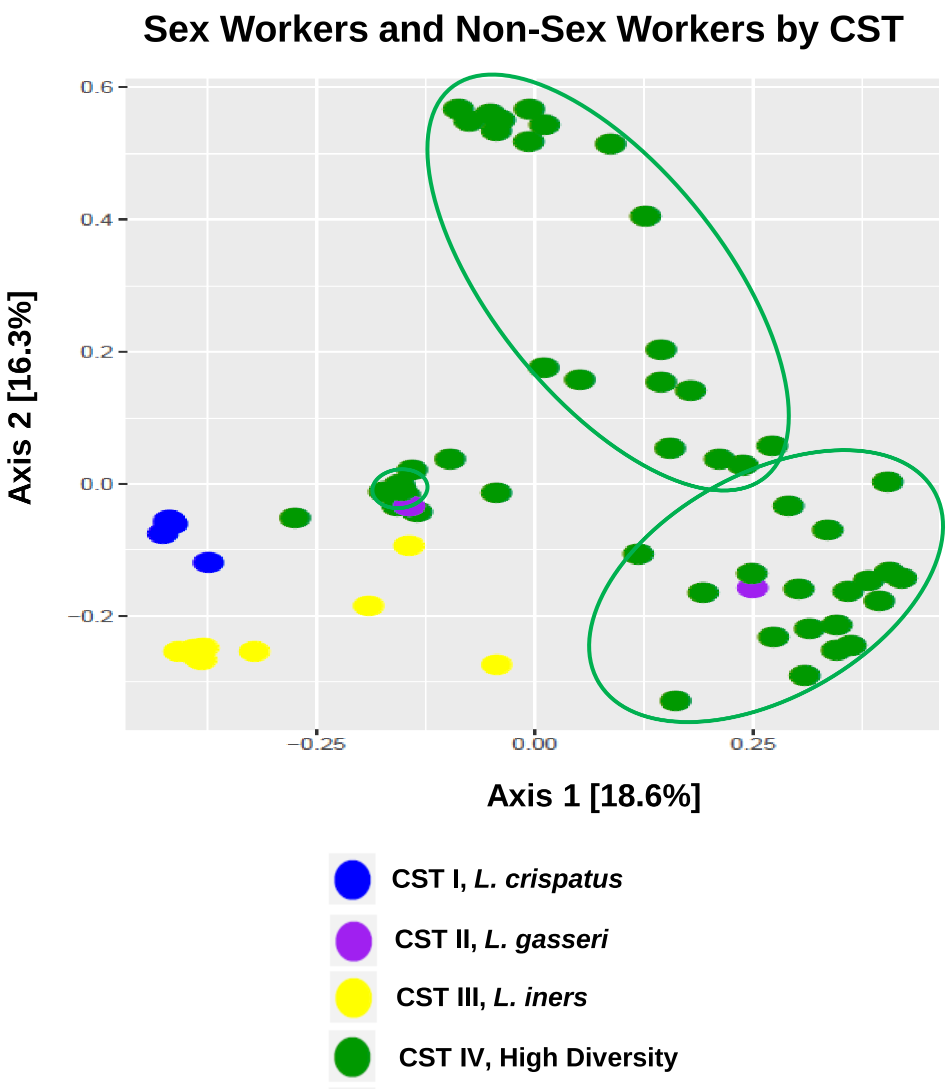

Sex Workers and Non-Sex Workers by CST
Axis 2 [16.3%]
0.6
0.4
0.2
0.0
−0.2
−0.25
0.00
0.25
Axis 1 [18.6%]
CST I, L. crispatus
CST II, L. gasseri
CST III, L. iners
CST IV, High Diversity

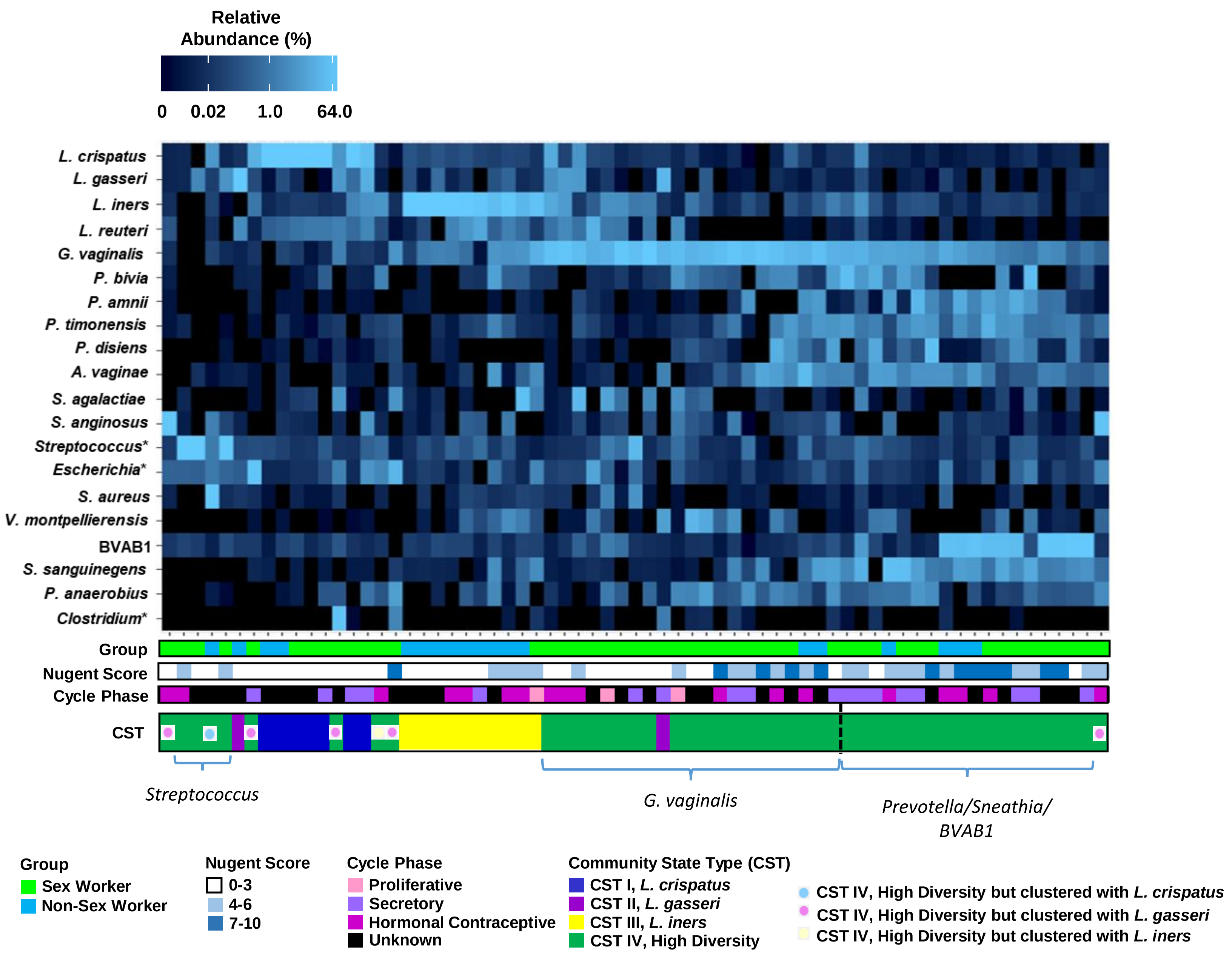

Relative Abundance (%)
0
0.02
1.0
64.0
L. crispatus
L. gasseri
L. iners
L. reuteri
G. vaginalis
P. bivia
P. amnii
P. timonensis
P. disiens
A. vaginae
S. agalactiae
S. anginosus
Streptococcus*
Escherichia*
S. aureus
V. montpellierensis
BVAB1
S. sanguinegens
P. anaerobius
Clostridium*
Group
Nugent Score
Cycle Phase
CST
Streptococcus
G. vaginalis
Prevotella/Sneathia/BVAB1
Group
Sex Worker
Non-Sex Worker
Nugent Score
0-3
4-6
7-10
Cycle Phase
Proliferative
Secretory
Hormonal Contraceptive
Unknown
Community State Type (CST)
CST I, L. crispatus
CST II, L. gasseri
CST III, L. iners
CST IV, High Diversity
CST IV, High Diversity but clustered with L. crispatus
CST IV, High Diversity but clustered with L. gasseri
CST IV, High Diversity but clustered with L. iners
