# Supplementary material for: Association of high-risk sexual behaviour with diversity of the vaginal microbiota and abundance of Lactobacillus
Source: PLoS One. 2017 Nov 2;12(11):e0187612. doi: 10.1371/journal.pone.0187612 (PMC5667760; doi:10.1371/journal.pone.0187612)
Supplement: S2 Fig — List of representative 16S sequences, listed by OTU. len: length. tot-seq: total number of sequences. (PDF) [file pone.0187612.s002.pdf]

>1 len=127 tot-seq=2525714

TAGGGAATCTTCCACAATGGACGCAAGTCTGATGGAGCAACGCCGCGTGAGTGAAGAAGG

TTTTCGGATCGTAAAGCTCTGTTGTTGGTGAAGAAGGATAGAGGTAGTAACTGGCCTTTA

TTTGACG

>2 len=152 tot-seq=2335314

TAGGGAATCTTCCACAATGGACGCAAGTCTGATGGAGCAACGCCGCGTGAGTGAAGAAGG

GTTTCGGCTCGTAAAGCTCTGTTGTTGGTGAAGAAGGACAGGGGTAGTAACTGACCTTTG

TTTGACGGTAATCAATTAGAAAGTCACGGCTA

>3 len=105 tot-seq=1937626

TGGGGAATATTGCGCAATGGGGGAAACCCTGACGCAGCGACGCCGCGTGCGGGATGAAGG

CCTTCGGGTTGTAAACCGCTTTTGATTGGGAGCAAGCCTTCGGGT

>4 len=218 tot-seq=629235

TAGGGAATCTTCGGCAATGGGGGCAACCCTGACCGAGCAACGCCGCGTGAGTGAAGAAGG

TTTTCGGATCGTAAAGCTCTGTTGTAAGAGAAGAACGTGTGTGAGAGTGGAAAGTTCACA

CAGTGACGGTAACTTACCAGAAAGGGACGGCTAACTACGTGGGGGGAGCCGCGGTAATAA

AAAGATCGGAAGAGCGTCGTGTAGGGAGGGAGGGAATC

>5 len=105 tot-seq=605715

TGGGGAATATTGCACAATGGGCGAAAGCCTGATGCAGCGACGCCGCGTGAGCGAAGAAGT

ATTTCGGTATGTAAAGCTCTATCAGAAGGGAAGAAAATGACGGTA

>6 len=134 tot-seq=364975

TGGGGAATATTGCACAATGGGCGCAAGCCTGATGCAGCCATGCCGCGTGTATGAAGAAGG

CCTTCGGGTTGTAAAGTACTTTCAGCGGGGAGGAAGGGAGTAAAGTTAATACCTTTGCTC

ATTGACGTTACCCG

>7 len=126 tot-seq=299437

TAGGGAATCTTCGGCAATGGACGGAAGTCTGACCGAGCAACGCCGCGTGAGTGAAGAAGG

TTTTCGGATCGTAAAGCTCTGTTGTTAGAGAAGAACGTTGGTAGGAGTGGAAAATCTACC

AAGTGA

>8 len=132 tot-seq=239129

TGAGGAATATTGGTCAATGGACGCAAGTCTGAACCAGCCAAGTAGCGTGCAGGATGACGG

CCCTATGGGTTGTAAACTGCTTTTATATGGGGATAAAGTGGGGAACGTGTTCCCTTTTGC

AGGTACCATATG

>9 len=105 tot-seq=213880

TGGGGAATCTTGCACAATGGGCGAAAGCCTGATGCAGCGACGCCGCGTGCGGGATGAAGG

CCTTCGGGTTGTAAACCGCTTTCAGCAGGGACGAGGCCGCAAGGT

>10 len=134 tot-seq=203233

TGAGGAATATTGGTCAATGGACGAGAGTCTGAACCAGCCAAGTAGCGTGCAGGATGACGG

CCCTATGGGTTGTAAACTGCTTTTGTTTGGGAATAATCGACATTACGTGTAATGTTTTGC

ATGTACCATTCGAA

>11 len=110 tot-seq=198189

GCGCGCAAATTACCCACTCCCGACCCGGGGAGGTAGTGACGAAAAATAACAATACAGGAC

TCTTTCGAGGCCCTGTAATTGGAATGAGTCCACTTTAAATCCTTTAACGA

>12 len=108 tot-seq=192408

TGGGGAATATTGGACAATGGAGGCAACTCTGATCCAGCAATTCTGTGTGTGTGAAGAAGG

TTTTAGGACTGTAAAACACTTTTAGTAGGGAAGAAAAAAATGACGGTA

>13 len=126 tot-seq=181581

TAGGGAATCTTCCACAATGGACGCAAGTCTGATGGAGCAACGCCGCGTGAGTGAAGAAGG

GTTTCGGCTCGTAAAGCTCTGTTGGTAGTGAAGAAAGATAGAGGTAGTAACTGGCCTTTA

TTTGAC

>14 len=131 tot-seq=173783

GGAATATTGGTCAATGGGCGAGAGCCTGAACCAGCCAAGTAGCGTGCAGGATGACGGCCC

TATGGGTTGTAAACTGCTTTTATATGGGAATAAAGTGAGGGACGTGTCCCTTATTGCATG

TACCATACGAA

>15 len=139 tot-seq=155421

TAGGGAATCTTCGGCAATGGGGGGAACCCTGACCGAGCAACGCCGCGTGAGTGAAGAAGG

TTTTCGGATCGTAAAGCTCTGTTGTTAAGGAAGAACGAGTGTGAGAATGGAAAGTTCATG

CTGTGACGGTACTTAACCA

>16 len=126 tot-seq=123819

TAGGGAATCTTCCGCAATGGGCGAAAGCCTGACGGAGCAACGCCGCGTGAGTGATGAAGG

TCTTCGGATCGTAAAACTCTGTTATTAGGGAAGAACATATGTGTAAGTAACTGTGCACAT

CTTGAC

>17 len=146 tot-seq=117396

TAGGGAATCTTCCACAATGGGCGCAAGCCTGATGGAGCAACACCGCGTGAGTGAAGAAGG

GTTTCGGCTCGTAAAGCTCTGTTGTTGGAGAAGAACGTGCGTGAGAGTAACTGTTCACGC

AGTGACGGTATCCAACCAGAAAGTCA

>18 len=161 tot-seq=99268

TAGGGAATCTTCGGCAATGGACGGAAGTCTGACCGAGCAACGCCGCGTGAGTGAAGAAGG

TTTTCGGATCGTAAAGCTCTGTTGTTAGAGAAGAACGAGTGTGAGAGTGGAAAGTTCATG

CTGTGACGGTACTTAACCAGAAAGGGACGGCTAACTACGTG

>19 len=229 tot-seq=96689

TGGGGAATATTGCACAATGGGCGCAAGCCTGATGCAGCCATGCCGCGTGTATGAAGAAGG

CCTTCGGGTTGTAAAGTACTTTCAGCGGGGAGGAAGGCGATAAGGTTAATAACCTTGTCG

ATTGACGTTACCCGCAGAAGAAGCACCGGCTAACTCCGTGCCAGCAGCCGCGGTAATCAC

AAGATCGGAAGAGCGTCGTGTTGGGGAAAAAAAAGGCCAATGTGTAGTT

>20 len=113 tot-seq=96512

TGGGGAATTTTGCACAATGGGGGAAACCCTGATGCAGCGACGCCGCGTGATTTAGAAGGC

CTTCGGGTTGTAAAAATCTTTTGTATGGGAAGAAAATGACAGTACCATACGAA

>21 len=201 tot-seq=89208

GTGACTGGAGTTCAGACGTGTGCTCTTCCGATCTCCTACGGGAGGCAGCTTTGAGGAATA

TTGGTCAATGGGCGAGAGCCTGAACCAGCCAAGTAGCGTGCAGGATGACGGCCCTATGGG

TTGTAAACTGCTTTTATGTGGGGATAAAGTGCGTGACGTGTCATGCATTGCAGGTACCAC

ATGAATAAGGACCGGCTAATT

>22 len=201 tot-seq=85270

TGGGGAATATTGCACAATGGGCGCAAGCCTGATGCAGCAACGCCGCGTGAACGATGAAGG

TCTTCGGATCGTAAAGTTCTGTTGCAGGGGAAGATAATGACGGTACCCTGTGAGGAAGCC

CCGGCTAACTACGTGGGGGGAGCCGCGGTAATACAAAGATCGGAAGAGCGTCGTGTAGGG

AAAGAGTGGGCCAATGTGTAG

>23 len=138 tot-seq=74266

TGGGGAATCTTCCGCAATGGACGAAAGTCTGACGGAGCAACGCCGCGTGAGTGATGACGG

TCTTCGGATTGTAAAGCTCTGTTAATCGGGACGAATGGTCGGTGTGCAAATAGTGCATGG

ACATGACGGTACCGGAAT

>24 len=241 tot-seq=66420

TAGGGAATTTTTCACAATGGGCGCAAGCCTTATGAAGCAATGCCGCGTGAACGATGAAGG

TCTTATAGATTGTAAAGTTCTTTTATATGGGAAGAAACGCTAAGATAGGAAATGATTTTA

GTTTGACTGTACCATTTGAATAAGTATCGGCTAACTATGTGGGGGGAGCCGCGGTAATAC

AAAGATCGGAAGAGCGTCGTGTAGGGAAAGAGTGGGGGAATGTGTAGATCTAGGGAATTT

T

>25 len=105 tot-seq=65137

TGGGGAATATTGCACAATGGGGGAAACCCTGATGCAGCAACGCCGCGTGAGTGATGAAGG

TTTTCGGATCGTAAAGCTCTGTCTTTGGGGAAGATAATGACGGTA

>26 len=139 tot-seq=61757

TAGGGAATCTTCCACAATGGACGCAAGTCTGATGGAGCAACGCCGCGTGAGTGAAGAAGG

TCTTCGGATCGTAAAACTCTGTTGTTAGAGAAGAACACGAGTGAGAGTAACTGTTCATTC

GATGACGGTATCTAACCAG

>27 len=134 tot-seq=56733

TGGGGAATATTGCACAATGGGGGAAACCCTGATGCAGCGACGCCGCGTGAGCGAAGAAGG

ATTTCGGTATGTAAAGCTCTATCAGCAGGGAAGAAAATGACGGTACCTGACTAAGAAGCC

CCGGCTAACTACGT

>28 len=128 tot-seq=54439

TGGGGAATCTTCCGCAATGGACGAAAGTCTGACGGAGCAACGCCGCGTGAGTGAAGAAGG

TCTTCGGATTGTAAAGCTCTGTTATACGGGACGAAAAAGACGGATGCCAACAGTATCCGT

CCGTGACG

>29 len=133 tot-seq=50754

TGAGGAATATTGGTCAATGGGCGGGAGCCTGAACCAGCCAAGTAGCGTGCAGGAAGACGG

CCCTATGGGTTGTAAACTGCTTTTATGCGGGGATAAAGGAGTCCACGTGTGGGCTTTTGC

AGGTATCGCATGA

>30 len=201 tot-seq=50450

GTGACTGGAGTTCAGACGTGTGCTCTTCCGATCTCCTACGGGAGGCAGCTTTGAGGAATA

TTGGTCAATGGGCGAGAGCCTGAACCAGCCAAGTCGCGTGAAGGAAGACTGCCCGCAAGG

GTTGTAAACTTCTTTTGTATGGGATTAAAGTCACCTACGTGTAGGTGTTTGCAGTTACCA

TACGAATAAGCATCGGCTAAC

>31 len=132 tot-seq=44894

TAGGGAATATTCCACAATGAGCGAAAGCTTGATGGAGCGACACAGCGTGCACGATGAAGG

TCTTCGGATTGTAAAGTGCTGTTATAAGGGAAGAACATTTGCAATAGGAAATGATTGCAG

ACTGACGGTACC

>32 len=226 tot-seq=40692

TAGGGAATCTTCCACAATGGGCGAAAGCCTGATGGAGCAACGCCGCGTGGGTGAAGAAGG

TCTTCGGATCGTAAAACCCTGTTGTTAGAGAAGAAAGTGCGTGAGAGTAACTGTTCACGT

TTCGACGGTATCTAACCAGAAAGCCACGGCTAACTACGTGGGGGGAGCCGCGGTAATATA

AAGATCGGAAGAGCGTCGTGTAGGGAAAGAGGGGGGCAATGTGTAG

>33 len=139 tot-seq=40552

TAGGGAATCTTCGGCAATGGACGGAAGTCTGACCGAGCAACGCCGCGTGAGTGAAGAAGG

TTTTCGGATCGTAAAGCTCTGTTGTAAGAGAAGAACGAGTGTGAGAGTGGAAAGTTCACA

CTGTGACGGTATCTTACCA

>34 len=137 tot-seq=39512

TGGGGAATCTTCCGCAATGGACGAAAGTCTGACGGAGCAACGCCGCGTGAGTGAAGACGG

TCTTCGGATTGTAAAGCTCTGTTATACGGGACGAAAGGCAAGGTGGTAAATAGCCATCAT

GAGTGACGGTACCGTAA

>35 len=132 tot-seq=38917

TGGGGAATATTGCACAATGGGGGAAACCCTGATGCAGCGACGCCGCGTGAAGGAAGAAGT

ATTTCGGTATGTAAACTTCTATCAGCAGGGAAGAAAATGACGGTACCTGACTAAGAAGCC

CCGGCTAACTAC

>36 len=133 tot-seq=33825

TGAGGAATATTGGTCAATGGGCGAGAGCCTGAACCAGCCAAGTAGCGTGCAGGATGACGG

CCCTATGGGTTGTAAACTGCTTTTACGCGGGGATAAAGTGCGTGACGTGTCTCGCATTGC

AGGTACCGCGTGA

>37 len=156 tot-seq=33479

TGAGGAATTTTTCACAATGGACGAAAGTCTGATGGAGCAATACCACGTGAACGATGAAGG

TCTTCTGATTGTAAAGTTCTTTTATTTAGGAAAAAAAGCGTGCTAGGAAATGAGCGCGCC

TTGATGGTACTAATTGAATAAGTGACAGCTAACTAT

>38 len=144 tot-seq=32265

TGAGGAATATTGGTCAATGGGCGCTAGCCTGAACCAGCCAAGTAGCGTGAAGGATGAAGG

CTCTATGGGTCGTAAACTTCTTTTATATAAGAATAAAGTGCAGTATGTATACTGTTTTGT

ATGTATTATATGAATAAGGATCGG

>39 len=143 tot-seq=30564

TGAGGAATATTGGTCAATGGACGGAAGTCTGAACCAGCCAAGTAGCGTGCAGGATGACGG

CCCTATGGGTTGTAAACTGCTTTTATATGGGGATAAAGTTAGGGACGTGTCCCTATTTGC

AGGTACCATACGAATAAGGACCG

>40 len=135 tot-seq=29699

TGGGGAATATTGCACAATGGGGGAAACCCTGATGCAGCGACGCCGCGTGAGGGAAGAAGG

CTTTCGAGTCGTAAAGCTCTGTCCTATGAGAAGATAATGACGGTATCATAGGAGGAAGCC

CTGGCTAAATACGTG

>41 len=125 tot-seq=27475

TGGGGAATATTGCACAATGGGCGCAAGCCTGATGCAGCGACGCCGCGTGAGGGATGGAGG

CCTTCGGGTTGTAAACCTCTTTTATCGGGGAGCAAGCGAGAGTGAGTTTACCCGTTGAAT

AAGCA

>42 len=135 tot-seq=25341

TGGGGAATATTGCACAATGGGGGAAACCCTGATGCAGCGACGCCGCGTGAGTGAAGAAGT

ATTTCGGTATGTAAAGCTCTATCAGCAGGGAAGAAAATGACGGTACCTGACTAAGAAGCC

CCGGCTAACTACGTG

>43 len=140 tot-seq=22723

TGGGGAATATTGCACAATGGGCGCAAGCCTGATGCAGCCATGCCGCGTGTGTGAAGAAGG

CCTTCGGGTTGTAAAGCACTTTCAGCGGGGAGGAAGGCGGTGAGGTTAATAACCTCATCG

ATTGACGTTACCCGCAGAAG

>44 len=132 tot-seq=22600

TGGGGAATATTGCACAATGGGGGAAACCCTGATGCAGCGACGCCGCGTGAGCGAAGAAGG

TATTCGTATCGTAAAGTTCTGTCCTATGGGAAGATAATGACAGTACCATAGAAGAAAGCT

CCGGCTAAATAC

>45 len=146 tot-seq=22453

TAGGGAATCTTCCGCAATGGACGCAAGTCTGACGGAGCAACGCCGCGTGAGTGAAGAAGG

ATTTCGGTTCGTAAAACTCTGTTGTTAGAGAAGAACAGCGCATAGAGTAACTGTTATGCG

TGTGACGGTATCTAACCAGAAAGCCA

>46 len=230 tot-seq=19937

TGGGGAATATTGCGCAATGGGGGGAACCCTGACGCAGCGACGCCGCGTGCGGGATGAAGG

CCTTCGGGTTGTAAACCGCTTTTGATTGGGAGCAAGCCTTTTGGGTGAGTGTACCTTTCG

AATAAGCGCCGGCTAACTACGTGCCAGCAGCCGCGGTAATCAAAAGATCGGAAGAGCGTC

GTGTAGGGAAAGAGTGGGGGAATGTGTAGATCTCGGGGGTTGGGGAATAT

>47 len=147 tot-seq=18199

TGGGGAATATTGCACAATGGGCGCAAGCCTGATGCAGCCATGCCGCGTGTATGAAGAAGG

CCTTCGGGTTGTAAAGTACTTTCAGCGGGGAGGAAGGCGTTGAGGTTAATAACCTCAGCA

ATTGACGTTACCCGCAGAAGAAGCACC

>48 len=150 tot-seq=16176

TGGGGAATATTGGACAATGGGCGAAAGCCTGATCCAGCCATGCCGCGTGTGTGAAGAAGG

TCTTCGGATTGTAAAGCACTTTAAGTTGGGAGGAAGGGCAGTAAATTAATACTTTGCTGT

TTTGACGTTACCGACAGAATAAGCACCGGC

>49 len=143 tot-seq=15631

TGGGGAATATTGGACAATGGGGGCAACCCTGATCCAGCCATGCCGCGTGTGTGAAGAAGG

CCCTCGGGTTGTAAAGCACTTTCAGTGAGGAAGAACGCCTGGTGGTTAATACCCATCAGG

AAAGACATCACTCACAGAAGAAG

>50 len=149 tot-seq=14895

TGGGGAATTTTGGACAATGGGCGCAAGCCTGATCCAGCCATGCCGCGTGTCTGAAGAAGG

CCTTCGGGTTGTAAAGGACTTTTGTCAGGGAAGAAAAGGCTGTTGCTAATACCAACAGCT

GATGACGGTACCTGAAGAATAAGCACCGG

>51 len=158 tot-seq=14739

TGGGGAATATTGGACAATGGGCGAAAGCCTGATCCAGCCATGCCGCGTGTGTGAAGAAGG

TCTTCGGATTGTAAAGCACTTTAAGTTGGGAGGAAGGGCAGTAGCTTAATACGTTACTGT

TTTGACGTTACCGACAGAATAAGCACCGGCTAACTCTG

>52 len=151 tot-seq=14585

TAGGGAATCTTCGGCAATGGACGAAAGTCTGACCGAGCAACGCCGCGTGAGTGAAGAAGG

TTTTCGGATCGTAAAACTCTGTTGTTAGAGAAGAACAAGGACGTTAGTAACTGAACGTCC

CCTGACGGTATCTAACCAGAAAGCCACGGCT

>53 len=135 tot-seq=14530

TGGGGAATATTGCACAATGGGGGAAACCCTGATGCAGCGACGCCGCGTGAAGGAAGAAGG

TATTCGGATTGTAAACTTCTGTCGTTGAGGAAGATAATGACGGTACTCAACAAGGAAGTG

ACGGCTAACTACGTG

>54 len=150 tot-seq=14218

TGGGGAATATTGGACAATGGGCGAAAGCCTGATCCAGCCATGCCGCGTGTGTGAAGAAGG

TCTTCGGATTGTAAAGCACTTTAAGTTGGGAGGAAGGGTTGTAGATTAATACTCTGCAAT

TTTGACGTTACCGACAGAATAAGCACCGGC

>55 len=118 tot-seq=13655

TGAGGAATATTCCACAATGGGCGAAAGCCTGATGGAGCAATGCCGCGTGCAGGATGAAGG

CCCTCGGGTCGTAAACTGCTTTTATTAGAGAAGAATATGACGGTAACTAATGAATAAG

>56 len=136 tot-seq=13590

TGGGGAATCTTCCGCAATGGGCGAAAGCCTGACGGAGCAACGCCGCGTGAGTGAAGACGG

CCTTCGGGTTGTAAAACTCTGTGATTCGGGACGAAAGATAAGTAGACGAATAATCTGCAT

AAGTGACGGTACCGAA

>57 len=137 tot-seq=13472

TGGGGAATCTTCCGCAATGGACGAAAGTCTGACGGAGCAACGCCGCGTGAGTGATGACGG

CCTTCGGGTTGTAAAGCTCTGTTATTCGGGACGAATGGTCTTTGTGTGAATAATGCAAAG

ATTTGACGGTACCGGAA

>58 len=145 tot-seq=13298

TAGGGAATCTTCCGCAATGGACGCAAGTCTGACGGAGCAACGCCGCGTGAGTGAAGAAGG

TTTTCGGATCGTAAAACTCTGTTGTAAGAGAAGAACAAATTGTAGAGTAACTGCTACAGT

CTTGACGGTATCTTACCAGAAAGCC

>59 len=139 tot-seq=12834

TGGGGAATATTGCACAATGGACGGAAGTTTGATGCAGCGACGCCGCGTGGAGGGTGTAGG

CCTTCGGGTTGTGAACTCCTTTTTCTCGTGAAAAAGGCATGCTTTTGGGTGTGTTGATGG

TAGCGGGGGAAGAAGCGCC

>60 len=145 tot-seq=12468

TGAGGAATATTGGTCAATGGACGAGAGTCTGAACCAGCCAAGTAGCGTGCAGGATGACGG

CCCTATGGGTTGTAAACTGCTTTTATAAGGGAATAAAGTGAGTCTCGTGAGACTTTTTGC

ATGTACCTTATGAATAAGGACCGGC

>61 len=201 tot-seq=12436

GACTGGAGTTCAGACGTGTGCTCTTCCGATCTCCTACGGGAGGCAGCTTTGAGGAATATT

GGTCAATGGACGAGAGTCTGAACCAGCCAAGTAGCGTGAAGGATGAAGGTTCTATGGATT

GTAAACTTCTTTTATACGGGAATAAACGAATCCACGTGTGGATTTTTGCATGTACCGTAT

GAATAAGGATCGGCTAACTCC

>62 len=148 tot-seq=11824

TGGGGAATCTTCCGCAATGGACGAAAGTCTGACGGAGCAACGCCGCGTGAGTGAAGACGG

CCTTCGGGTTGTAAAGCTCTGTTATTCGGGACGAAAGGCCATATGTGAATAATATATGGA

AATGACGGTACCGAAAAAGCAAGCCACG

>63 len=127 tot-seq=11734

TGGGGAATATTGCGCAATGGGGGAAACCCTGACGCAGCGACGCCGCGTGCGGGATGAAGG

CCTTCGGGTTGTAAACCGCTTTCAGCAGGGAAGACATCGACGGTACCTGCAGAAGAAGCC

CCGGCTA

>64 len=150 tot-seq=11164

TGGGGAATTTTGGACAATGGGCGCAAGCCTGATCCAGCAATGCCGCGTGAGTGAAGAAGG

CCCTCGGGTTGTAAAGCTCTTTTGTCAGGGAAGAAAAGGTGATCTCTAATACAGGTCACT

CATGACGGTACCTGAAGAATAAGCACCGGC

>65 len=142 tot-seq=10869

TGGGGAATATTGCACAATGGGCGCAAGCCTGATGCAGCGACGCCGCGTGAGGGATGACGG

CCTTCGGGTTGTAAACCTCTTTTGTTAGGGAGCAAGCCTTCGGGTGAGTGTACCTTTCGA

ATAAGCACCGGCTAACTACGTG

>66 len=146 tot-seq=10816

TAGGGAATCTTCCACAATGGACGAAAGTCTGATGGAGCAACGCCGCGTGAATGAAGAAGG

CCTTCGGGTCGTAAAATTCTGTTGTCAGAGAAGAACGTGCGTGAGAGTAACTGTTCACGT

ATTGACGGTATCTGACCAGAAAGCCA

>67 len=135 tot-seq=10781

TGGGGAATATTGCACAATGGGCGAAAGCCTGATGCAGCGACGCCGCGTGAGGGATGACGG

CCTTCGGGTTGTAAACCTCTTTCAGCAGGGACGAAGCGCAAGTGACGGTACCTGCAGAAG

AAGCACCGGCTAACT

>68 len=125 tot-seq=10264

TGGGGAATTTTGGACAATGGGCGCAAGCCTGATCCAGCAATGCCGCGTGTGTGAAGAAGG

CCTTCGGGTTGTAAAGCACTTTTGTCCGGAAAGAAAACGCCGTGGTTAATACCCGTGGCG

GATGA

>69 len=146 tot-seq=9792

TGGGGAATATTGCGCAATGGGGGGAACCCTGACGCAGCCATGCCGCGTGAATGAAGAAGG

CCTTAGGGTTGTAAAGTTCTTTCGGTATTGAGGAAGGTTGATGTGTTAATAGCACATCAA

ATTGACGTTAAATACAGAAGAAGCAC

>70 len=146 tot-seq=9685

TGGGGAATATTGCACAATGGGGGAAACCCTGATGCAGCGACGCCGCGTGAGTGAAGAAGT

ATTTCGGTATGTAAAGCTCTATCAGCAGGGAAGAAATAGCGACCTTTGGGTCGCCAGACG

GTACCTGATTAAGAAGCCCCGGCTAA

>71 len=146 tot-seq=9252

TGGGGAATTTTGGACAATGGGCGCAAGCCTGATCCAGCAATGCCGCGTGTGTGAAGAAGG

CCTTCGGGTTGTAAAGCACTTTTGTCCGGAAAGAAAACCTCCTGGTTAATACCTGGGGGG

GATGACGGTACCGGAAGAATAAGCAC

>72 len=135 tot-seq=8525

TGGGGAATATTGCACAATGGAGGGAACTCTGATGCAGCGACGCCGCGTGAACGAAGAAGG

CTTTCGAGTCGTAAAGTTCTTTTATATGGGAAGATAATGACGGTACCATAAGAAAAAGCC

CCGGCTAACTACGTG

>73 len=139 tot-seq=8515

TGGGGAATTTTCCGCAATGGGCGAAAGCCTGACGGAGCAATGCCGCGTGGAGGTGGAAGG

CCTACGGGTCGTCAACTTCTTTTCTCGGAGAAGAAACAATGACGGTATCTGAGGAATAAG

CATCGGCTAACTCTGTGGG

>74 len=229 tot-seq=8497

TGGGGAATATTGGGCAATGGGCGAAAGCCTGACCCAGCAACGCCGCGTGAGTGATGAAGG

CCTTCGGGTTGTAAAACTCTTTGGACAGGGACGAAGAAAGTGACGGTACCTGTAGAACAA

GCCACGGCTAACTACGTGGGGGGAGCCGGGTAATCGCAAGATCGGAAGAGCGTCGTGTAG

GGAAAGAGTGTGCCAATGGGGAATATTGGGCAATGGGCGAAAGCCTGAC

>75 len=149 tot-seq=8397

TAGGGAATCTTCCGCAATGGACGAAAGTCTGACGGAGCAACGCCGCGTGAGTGAAGAAGG

ATTTCGGTTCGTAAAACTCTGTTGTTAGGGAAGAAAAAATGTATAGTAACTATATACAAA

AGAGACGGTACCTAACCAGAAAGCCACGG

>76 len=140 tot-seq=8239

TGAGGAATATTGGTCAATGGGCGTAAGCCTGAACCAGCCAAGTCGCGTGAGGGATGAAGG

TTCTATGGATCGTAAACCTCTTTTATAAGGGAATAAAGTGCGGGACGTGTCCTGTTTTGT

ATGTACCTTATGAATAAGGA

>77 len=140 tot-seq=7986

TAGGGAATCTTCCACAATGGGCGCAAGCCTGATGGAGCAACACCGCGTGAGTGAAGAAGG

GTTTCGGCTCGTAAAGCTCTGTTGTTGGAGAAGAACGTATCTGAGAGTAACTGTTCAGGT

AGTGACGGTATCCAACCAGA

>78 len=148 tot-seq=7917

TGGGGAATATTGGACAATGGGCGCAAGCCTGATCCAGCCATACCGCGTGGGTGAAGAAGG

CCTTCGGGTTGTAAAGCCCTTTTGTTGGGAAAGAAATCCAGCCGGCTAATACCTGGTTGG

GATGACGGTACCCAAAGAATAAGCACCG

>79 len=182 tot-seq=7911

TGGGGAATATTGCACAATGGGGGAAACCCTGATGCAGCGACGCCGCGTGAGCGAAGAAGG

CCTTCGGGTCGTAAAGCTCTTTTATATGGGAAGATAATGACGGTACCATAAGAAAAAGCC

CCGGCTAACTACGTGGGGGGAGCCGCGGTAATACAAGATCGGAAGAGCGTCGTGTTGGGG

AA

>80 len=121 tot-seq=7652

TAGGGAATATTGCACAATGGAGGAAACTCTGATGCAGCCATGCCGCGTGTGTGAAGAAGG

CCTTCGGGTTGTAAAGCACTTTCGGAGGGGAGGAAAAAAATGACGTTACCCTCAGAAGAA

G

>81 len=144 tot-seq=7571

TGAGGAATATTGGTCAATGGGCGCTAGCCTGAACCAGCCAAGTAGCGTGCAGGATGACGG

CCCTATGGGTTGTAAACTGCTTTTATGCGGGGATAAAGTCACCCACGTGTGGGTGTTTGC

AGGTACCGCATGAATAAGGACCGG

>82 len=153 tot-seq=7245

TGGGGAATTTTGGACAATGGGGGCAACCCTGATCCAGCCATGCCGCGTGCAGGATGAAGG

TCTTCGGATTGTAAACTGCTTTTGTCAGGGAAGAAAAGGTCTGTGTTAATACCATAGACT

GCTGACGGTACCTGAAGAATAAGCACCGGCTAA

>83 len=147 tot-seq=6950

TGGGGAATCTTCCGCAATGGACGAAAGTCTGACGGAGCAACGCCGCGTGAACGATGACGG

CCTTCGGGTTGTAAAGTTCTGTTATATGGGACGAACAGGGCATCGGTTAATACCCGGTGT

CTTTGACGGTACCGTAAGAGAAAGCCA

>84 len=134 tot-seq=6760

TGGGGAATATTGGACAATGGGCGCAAGCCTGATCCAGCCATGCCGCGTGAGTGATGAAGG

CCTTAGGGTTGTAAAGCTCTTTCACCGGTGAAGATAATGACGGTAGCCGGAGAAGAAGCC

CCGGCTAACTTCGT

>85 len=139 tot-seq=6715

TGGGGAATATTGCACAATGGGCGGAAGCCTGATGCAGCAACGCCGCGTGCGGGATGACGG

CCTTCGGGTTGTAAACCGCTTTCGCCTGTGACGAAGCGTGAGTGACGGTAATGGGTAAAG

AAGCACCGGCTAACTACGT

>86 len=146 tot-seq=6693

TAGGGAATCTTCCACAATGGACGCAAGTCTGATGGAGCAACGCCGCGTGAGTGAAGAAGG

GTTTCGGCTCGTAAAGCTCTGTTGTTAAAGAAGAACGTGGGTAAGAGTAACTGTTTACCC

AGTGACGGTATTTAACCAGAAAGCCA

>87 len=144 tot-seq=6644

TGGGGAATCTTCCGCAATGGGCGCAAGCCTGACGGAGCAATGCCGCGTGAGTGAAGAAGG

CCTTCGGGTTGTAAAACTCTGTCCTCATCCAAGAGAAGGGAAGGTAGTAACTGACCTTTC

CAGGACGGTAGATGAGGAGGAAGC

>88 len=151 tot-seq=6502

TAGGGAATTTTCGTCAATGGGGGAAACCCTGAACGAGCAATGCCGCGTGAGTGAAGAAGG

TCTTCGGATCGTAAAGCTCTGTTGTAAGTGAAGAACGGCTCATAGAGGAAATGCTATGGG

AGTGACGGTAGCTTACCAGAAAGCCACGGCT

>89 len=216 tot-seq=6197

TAGGGAATTTTCGTCAATGGGCGCAAGCCTGAACGAGCGATGCCGCGTGAGTGAAGAAGG

CCTTCGGGTCGTAAAGCTCTGTTGCGGGGGAAAAAAGGCAGCATCAGGAAATGGGTGCTG

ACTGATGGTGCCCCGCCAGAAAGTCACGGCTAACTACGTGGGGGGAGCCGCGGTAATATC

GAGATCGGAAGAGCGTCGTGTAGGGGAAGAGTGTGC

>90 len=211 tot-seq=6160

TGAGGAATATTGGTCAATGGACGAGAGTCTGAACCAGCCAAGTAGCGTGAAGGATGACTG

CCCTATGGGTTGTAAACTTCTTTTATATGGGAATAAAGTTTTCCACGTGTGGAATTTTGT

ATGTACCATATGAATAAGGATCGGCTAACTCCGTGGGGGGAGCCGCGGTAATAAGAAGAT

CGGAAGAGCGTCGTGTAGGGAAAGAGGGGGC

>91 len=141 tot-seq=5976

TGAGGAATATTGGTCAATGGGAGAGATCCTGAACCAGCCAAGCCGCGTGAGGGAAGACGG

CCCTATGGGTTGTAAACCTCTTTTGTCGGAGAACAAAACCCGGGACGTGTCCCGGACTGC

GTGTATCCGAAGAAAAAGCAT

>92 len=126 tot-seq=5971

TGGGGAATATTGCACAATGGAGGGAACTCTGATGCAGCGACGCCGCGTGAGTGAAGAAGT

ATTTCGGTATGTAAAGCTCTATCAGCAGGGAAGAAGATGACGGTACCTGAATAAGAAGCC

CCGGCT

>93 len=134 tot-seq=5954

TGGGGAATATTGGACAATGGACCAAGAGTCTGATCCAGCAATTCTGTGTGCACGATGAAG

TTTTTCGGAATGTAAAGTGCTTTCAGTTGGGAAGAAAAAAATGACGGTACCAACAGAAGA

AGTGACGGCTAAAT

>94 len=147 tot-seq=5948

TGAGGAATATTGGTCAATGGACGAGAGTCTGAACCAGCCAAGTAGCGTGAAGGATGACTG

CCCTATGGGTTGTAAACTTCTTTTATACGGGAATAAAGTGAGGCACGTGTGCCTTTTTGT

ATGTACCGTATGAATAAGGATCGGCTA

>95 len=132 tot-seq=5898

TGGGGAATCTTCCGCAATGGACGAAAGTCTGACGGAGCAACGCCGCGTGAACGATGACGG

CCTTCGGGTTGTAAAGTTCTGTTATACGGGACGAATGGTACGACGGTCAATACCCGTCGT

AAGTGACGGTAC

>96 len=144 tot-seq=5697

TAGGGAATCTTCGGCAATGGACGAAAGTCTGACCGAGCAACGCCGCGTGAGTGAAGAAGG

TTTTCGGATCGTAAAACTCTGTTGGTAGAGAAGAACGTTGGTGAGAGTGGAAAGCTCATC

AAGTGACGGTAACTACCCAGAAAG

>97 len=156 tot-seq=5611

TGGGGAATCTTCCGCAATGGACGAAAGTCTGACGGAGCAACGCCGCGTGAACGATGACGG

CCTTCGGGTTGTAAAGTTCTGTTATACGGGACGAATGGCGCAACGGTCAATACCCGTTGC

GAGTGACGGTACCGTAAGAGAAAGCCACGGCTAACT

>98 len=183 tot-seq=5462

TGGGGAATATTGCACAATGGGGGAAACCCTGATGCAGCGACGCCGCGTGGAGGAAGAAGG

TCTTCGGATTGTAAACTCCTGTTGTTGAGGAAGATAATGACGGTACTCAACAAGGAAGTG

ACGGCTAACTACGTGGGGGGAGCCGCGGTAATACAAAGATCGGAAGAGCGTCGTGTTGGG

GAA

>99 len=129 tot-seq=5293

TCGAGAATCATTCACAATGGGGGAAACCCTGATGGTGCGACGCCGCGTGGGGGAATGAAG

GTCTTCGGATTGTAAACCCCTGTCATGTGGGAGCAAATTAAAAAGATAGTACCACAAGAG

GAAGAGACG

>100 len=134 tot-seq=5227

TAGGGAATCTTCGGCAATGGGGGCAACCCTGACCGAGCAACGCCGCGTGAGTGAAGAAGG

TTTTCGGATCGTAAAGCTCTGTTGTAAGTCAAGAACGAGTGTGAGAGTGGAAAGTTCACA

CTGTGACGGTAGCT

>101 len=144 tot-seq=5202

TGGGGAATATTGCACAATGGGCGCAAGCCTGATGCAGCGACGCCGCGTGAGGGATGGAGG

CCTTCGGGTTGTAAACCTCTTTTGTTAGGGAGCAAGGCACTTTGTGTTGAGTGTACCTTT

CGAATAAGCACCGGCTAACTACGT

>102 len=140 tot-seq=5042

TGAGGAATATTGGTCAATGGGCGATGGCCTGAACCAGCCAAGTAGCGTGAAGGATGACTG

CCCTATGGGTTGTAAACTTCTTTTATAAAGGAATAAAGTCGGGTATGCATACCCGTTTGC

ATGTACTTTATGAATAAGGA

>103 len=136 tot-seq=5018

TGGGGAATATTGCACAATGGACGAAAGTTTGATGCAGCGACGCCGCGTGGAGGGTGTAGG

CCTTCGGGTTGTGAACTCCTTTTTCTCGCGAAAAAGGCACAGTTTTGGCTGTGTTGATGG

TAGTGGGGGAAGAAGC

>104 len=134 tot-seq=4916

TGGGGGATATTGCACAATGGGGGAAACCCTGATGCAGCGACGCCGCGTGGAGGAAGAAGG

TTTTCGGATTGTAAACTCCTGTCGTTAGGGACGATAATGACGGTACCTAACAAGAAAGCA

CCGGCTAACTACGT

>105 len=129 tot-seq=4705

TGGGGAATATTGCACAATGGGGGAAACCCTGATGCAGCGACGCCGCGTGAGCGATGAAGG

CTTTCGAGTCGTAAAGCTCTGTCCTATGGGAAGATAATGACGGTACCATAGAAGAAAGCC

CCGGCTAAT

>106 len=158 tot-seq=4699

TGGGGAATCTTCCGCAATGGACGAAAGTCTGACGGAGCAACGCCGCGTGAGTGATGACGG

CCTTCGGGTTGTAAAGCTCTGTTAATCGGGACGAAAGGCCTTCTTGCGAATAGTTAGAAG

GATTGACGGTACCGGAATAGAAAGCCACGGCTAACTAC

>107 len=153 tot-seq=4565

TGGGGAATTTTGGACAATGGGGGCAACCCTGATCCAGCCATGCCGCGTGTCTGAAGAAGG

CCTTCGGGTTGTAAAGGACTTTTGTCCGGGAAGAAAAGCGCGATGTTAATACCATTGCGT

GCTGACGGTACCGGAAGAATAAGCACCGGCTAA

>108 len=157 tot-seq=4466

TAGGGAATCTTCGGCAATGGGCGAAAGCCTGACCGAGCAACGCCGCGTGAATGAAGAAGG

CCTTCGGGTTGTAAAATTCTGTTATAAGGGAAGAAAGGTGATAGGAGGAAATGACTATCA

ATTGACGGTACCTTATGAGAAAGCCACGGCTAACTAC

>109 len=188 tot-seq=4450

TGGGGAATCTTGGACAATGGGCGAAAGCCCGATCCAGCAATATCGCGTGAGTGAAGAAGG

GCAATGCCGCTTGTAAAGCTCTTTCGTCGAGTGCGCGATCATGACAGGACTCGAGGAAGA

AGCCCCGGCTAACTCCGTGGGGGGAGCCGCGGTAATAGAAGAAAGGGAGAGCGCGTGTAG

GGAAAGAG

>110 len=136 tot-seq=4417

TGGGGGATATTGGACAATGGGGGAAACCCTGATCCAGCGACGCCGCGTGAGTGAAGAAGT

ATTTCGGTATGTAAAGCTCTGTCAGCAGGGAAGAAAGAAATGACGGTACCTGACCAAGAA

GCCCCGGCTAACTACG

>111 len=130 tot-seq=4362

TGGGGAATATTGGGCAATGGGCGAAAGCCTGACCCAGCAACGCCGCGTGAAGTATGAAGG

CCTTCGGGTTGTAAACTTCTTTGATCAGGGAAGAAACAAATGACGGTACCTGAAAAACAA

GCCACGGCTA

>112 len=148 tot-seq=4082

TAGGGAATCTTCGGCAATGGACGAAAGTCTGACCGAGCAACGCCGCGTGAGTGAAGAAGG

TTTTCGGATCGTAAAACTCTGTTGTTAGAGAAGAACAAGGATGAGAGTAACTGTTCATCC

CTTGACGGTATCTAACCAGAAAGCCACG

>113 len=149 tot-seq=3970

TGGGGAATCTTCCGCAATGGACGAAAGTCTGACGGAGCAACGCCGCGTGAGTGATGAAGG

TCTTCGGATTGTAAAACTCTGTTGTCAGGGACGAATGTACTGATCTATAATACATTTCGG

TATTGACGGTACCTGACGAGGAAGCCACG

>114 len=135 tot-seq=3804

TGGGGAATATTGCACAATGGGGGAAACCCTGATGCAGCGACGCCGCGTGAGCGATGAAGT

ATTTCGGTATGTAAAGCTCTATCAGCAGGGAAGATAATGACGGTACCTGACTAAGAAGCT

CCGGCTAAATACGTG

>115 len=160 tot-seq=3793

TGGGGAATTTTGGACAATGGGCGCAAGCCTGATCCAGCCATGCCGCGTGTCTGAAGAAGG

CCTTCGGGTTGTAAAGGACTTTTGTCAGGGAAGAAAAGGGCGGGGTTAATACCCCTGTCT

GATGACGGTACCTGAAGAATAAGCACCGGCTAACTACGTG

>116 len=155 tot-seq=3753

TGAGGAATATTGGTCAATGGCCGGGAGGCTGAACCAGCCAAGTCGCGTGAGGGAAGACGG

TCCTATGGATTGTAAACCTCTTTTGTCAGGGAGCAAAAACTGCCACGTGTGGCAGGATGA

GAGTACCTGAAGAAAAAGCATCGGCTAACTCCGTG

>117 len=137 tot-seq=3730

TGGGGAATATTGGGCAATGGGCGCAAGCCTGACCCAGCAACGCCGCGTGAAGGAAGAAGG

CTTTCGGGTTGTAAACTTCTTTTGTCAGGGACGAAGCAAGTGACGGTACCTGACGAATAA

GCCACGGCTAACTACGT

>118 len=133 tot-seq=3707

TGGGGGATATTGCACAATGGAGGCAACTCTGATGCAGCAACGCCGCGTGAGTGAAGAAGG

CTTTCGAGTCGTAAAACTCTGTCGTATGGGAAGAAAAAAATGACGGTACCATACAAGAAA

GCCCCGGCTAACT

>119 len=153 tot-seq=3667

TGAGGAATATTGGACAATGGGCGGAAGCCTGATCCAGCCATGCCGCGTGAAGGAAGGAGG

TCCTATGGATCGTAAACTTCTTTTGACGCAGAGCAATAAAGTGTACGAGTACACTGATGA

GAGTATGCGTCGAATAAGCATCGGCTAACTCCG

>120 len=157 tot-seq=3627

TAGGGAATTTTCGGCAATGGGGGAAACCCTGACCGAGCAACGCCGCGTGAAGGAAGAAGT

AATTCGTTATGTAAACTTCTGTCATAGAGGAAGAACGGTGGATATAGGGAATGATATCCA

AGTGACGGTACTCTATAAGAAAGCCACGGCTAACTAC

>121 len=135 tot-seq=3613

TGGGGAATATTGCACAATGGGGGAAACCCTGATGCAGCGACGCCGCGTGAGCGAAGAAGT

ATTTCGGTATGTAAAGCTCTATCAGCAGGGAAGAAGAAATGACGGTACCTGACTAAGAAG

CACCGGCTAAATACG

>122 len=128 tot-seq=3607

TGGGGAATATTGCACAATGGGGGAAACCCTGATGCAGCAACGCCGCGTGAGTGAAGAAGG
TTTTCGGATTGTAAAGCTCTGTTAGCAGGGAAGAGGAAGGACGGTACCTGCAGAGGAAGC
CACGGCTA
>123 len=142 tot-seq=3465
TGAGGAATATTGGTCAATGGGCGCGAGCCTGAACCAGCCAAGTCGCGTGAGGGACGACGG
TCCTACGGATTGTAAACCTCTTTAGGCGGGGAGTAATGTGCTCTACGAGTAGAGTAGTGA
GAGTACCCGCAGAATAAGCATC
>124 len=153 tot-seq=3457
TGAGGAATATTGGTCAATGGGCGAGAGCCTGAACCAGCCAAGTAGCGTGAAGGATGAAGG
CTCTATGGGTCGTAAACTTCTTTTATATGGGAATAAAGTTTTCCACGTGTGGAATTTTGT
ATGTACCATATGAATAAGGATCGGCTAACTCCG
>125 len=139 tot-seq=3434
TGGGGAATATTGCACAATGGGGGAAACCCTGATGCAGCGACGCCGCGTGGGGGATGACGG
CCTTCGGGTTGTAAACCTCTTTCAGTAGGGAAGAAGCCTTCGGGTGACGGTACCTGCAGA
AGAAGTACCGGCTAACTAC
>126 len=149 tot-seq=3419
TGAGGAATATTGGTCAATGGGCGCGAGCCTGAACCAGCCAAGTCGCGTGAGGGAAGACGG
TCCTATGGATTGTAAACCTCTTTTGCCGGGGAGCAAGCTGTGTCACGTGTGACACAGCTG
AGAGTACCCGGAGAAAAAGCATCGGCTAA
>127 len=150 tot-seq=3409
TGAGGAATATTGGTCAATGGACGCAAGTCTGAACCAGCCATGCCGCGTGCAGGAAGACGG
CTCTATGAGTTGTAAACTGCTTTTGTACGAGGGTAAACGCATCTACGTGTAGGTGCCTGA
AAGTATCGTACGAATAAGGATCGGCTAACT
>128 len=133 tot-seq=3406
TGGGGAATATTGCACAATGGGCGAAAGCCTGATGCAGCAACGCCGCGTGAGCGATGAAGG
CCTTCGGGTCGTAAAGCTCTGTCCTCAAGGAAGATAATGACGGTACTTGAGGAGGAAGCC
CCGGCTAACTACG
>129 len=152 tot-seq=3331
TGGGGAATATTGCACAATGGGCGCAAGCCTGATGCAGCCATGCCGCGTGTATGAAGAAGG

CCTTCGGGTTGTAAAGTACTTTCAGCGGGGAGGAAGGGAGTGAGGTTAATAACCTTATTC

ATTGACGTTACCCGCAGAAGAAGCACCGGCTA

>130 len=158 tot-seq=3312

TAGGGAATCTTCCACAATGGGCGCAAGCCTGATGGAGCAACACCGCGTGAGTGAAGAAGG

GTTTCGGCTCGTAAAACTCTGTTGTTGAAGAAGAACGTGCGTGAGAGTAACTATTCACGC

AGTGACGGTATTCAACCAGAAAGTCACGGCTAACTACG

>131 len=160 tot-seq=3292

TGGGGAATATTGGACAATGGGCGAAAGCCTGATCCAGCCATGCCGCGTGTGTGAAGAAGG

TCTTCGGATTGTAAAGCACTTTAAGTTGGGAGGAAGGGCAGTTACCTAATACGTAATTGT

TTTGACGTTACCGACAGAATAAGCACCGGCTAACTCTGTG

>132 len=148 tot-seq=3281

TGGGGAATCTTCCGCAATGGGCGCAAGCCTGACGGAGCAACGCCGCGTGAGTGAAGAAGG

GTTTCGACTCGTAAAGCTCTGTTGTCGGGGACGAACGTGGAGACGGCGAATAGCCGTTTC

CAATGACGGTACCTGACGAGGAAGCCAC

>133 len=150 tot-seq=3122

TGAGGAATATTGGTCAATGGGCGAGAGCCTGAACCAGCCAAGTCGCGTGAAGGAAGACTG

CCCGCAAGGGTTGTAAACTTCTTTTGTATGGGATTAAAGTCGTCTACGTGTAGACGTTTG

CAGTTACTATACGAATAAGCATCGGCTAAC

>134 len=159 tot-seq=3084

TGGGGAATTTTGGACAATGGGGGAAACCCTGATCCAGCCATCCCGCGTGTGCGATGAAGG

CCTTCGGGTTGTAAAGCACTTTTGGCAGGAAAGAAACGTCGTGGGTTAATACCCCGCGAA

ACTGACGGTACCTGCAGAATAAGCACCGGCTAACTACGT

>135 len=151 tot-seq=3011

TGGGGAATATTGCACAATGGGGGAAACCCTGATGCAGCGACGCCGCGTGAGTGAAGAAGT

ATTTCGGTACGTAAAGCTCTATCAGCAGGGAAGAAAGAAAGTCCTTTGGACTTTGGACGG

TACCTGACCAAGAAGCCCCGGCTAACTACGT

>136 len=136 tot-seq=2929

GCATGCAAATTACCCACTCCCGACCCGAAGAGGTAGTGACGAAAAATAACAATACAGGAC

TCTTTTGAGGTCCTGTAATTGGAATGAGTCCACTTTAAATCCTTTAATGAGGATCCATTG

GAGGGCAAATCTAGTG

>137 len=140 tot-seq=2767

TGGGGAATATTGCACAATGGGCGCAAGCCTGATGCAGCGACGCCGCGTGGGGGATGACGG

CCTTCGGGTTGTAAACTCCTTTCGCCAGGGACGAAGCGTTTTGTGACGGTACCTGGATAA

GAAGCACCGGCTAACTACGT

>138 len=145 tot-seq=2724

TGAGGAATATTGGTCAATGGTCGGGAGACTGAACCAGCCAAGCCGCGTGAGGGAGGAAGG

TACAGAGTATCGTAAACCTCTTTTGTCAGGGAACAAAGGCGGGGACGTGTCCCCGGATGA

GTGTACCTGAAGAAAAAGCATCGGC

>139 len=134 tot-seq=2696

TGGGGAATATTGCACAATGGGCGGAAGCCTGATGCAGCGACGCCGCGTGAGGGATGACGG

CCTTCGGGTTGTAAACCTCTTTCAGCAGGGAAGAAGCCACAAGTGACGGTACCTGCAGAA

GAAGCGCCGGCTAA

>140 len=135 tot-seq=2633

TGGGGAATCTTGCACAATGGGGGAAACCCTGATGCAGCGACGCCGCGTGAGCGATGAAGG

TTTTCGAATCGTAAAGCTCTGTCCTATGGGAAGATAATGACGGTACCATAGGAGGAAGCC

CCGGCTAACTACGTG

>141 len=152 tot-seq=2610

TGGGGAATTTTGGACAATGGGCGCAAGCCTGATCCAGCAATGCCGCGTGTGTGAAGAAGG

CCTTCGGGTTGTAAAGCACTTTTGTCGGGAAAGAAAACCTCTGCCCTAATACGGTGGGGG

GATGACGGTACCGGAAGAATAAGCACCGGCTA

>142 len=133 tot-seq=2581

TGGGGAATATTGCACAATGGGGGAAACCCTGATGCAGCAACGCCGCGTGAGTGATGACGG

CCTTCGGGTTGTAAAGCTCTGTCTTCAGGGACGATAATGACGGTACCTGAGGAGGAAGCC

ACGGCTAACTACG

>143 len=160 tot-seq=2579

TAGGGAATCTTCCACAATGGGCGCAAGCCTGATGGAGCAACACCGCGTGAGTGAAGAAGG

GTTTCGGCTCGTAAAGCTCTGTTGTTAAAGAAGAACACGTATGAGAGTAACTGTTCATAC

GTTGACGGTATTTAACCAGAAAGTCACGGCTAACTACGTG

>144 len=137 tot-seq=2579

TGGGGAATATTGGGCAATGGGCGCAAGCCTGACCCAGCAACGCCGCGTGAAGGAAGAAGG

CTTTCGGGTTGTAAACTTCTTTTAAGAGGGAAGAGCAGAAGACGGTACCTCTTGAATAAG

CCACGGCTAACTACGTG

>145 len=176 tot-seq=2544

GTATCTCAAGCAGGGAGTCGAAGCCAAAGAGGGAGAGCTTCTGGAACTGGGGCTTTACTA

GGGCCCTCAGGCATGCTCCCTCAGGGGAGGTTGTGCATTGATTGGTTTAGAGGAAACACG

TGCAAAGGGGGACGTTATTGACAGGACTCTGGTGTTGATCATTAGGTTCAATCATG

>146 len=136 tot-seq=2543

GCATGCAAATTACCCACTCCCGACCCAAGGAGGTAGTGATGAAACATACCAATACAGGAC

TCTTTCGAGGTCCTGTAATTGGAATGAGTCCACTTTAAATCCTTTAACGAAGATCCATTG

GAGGGCAAGTCTAGTG

>147 len=135 tot-seq=2542

TGGGGAATATTGCACAATGGAGGGAACTCTGATGCAGCGACGCCGCGTGAGTGAAGAAGT

ATTTCGGTATGTAAAGCTCTATCAGTAGGGAAGATAATGACGGTACCTACATAAGAAGCC

CCGGCTAAATACGTG

>148 len=134 tot-seq=2484

TAGGGAATATTGCACAATGGGGGAAACCCTGATGCAGCGACGCCGCGTGAAGGATGAAGT

ATTTCGGTATGTAAACTTCTATCAGCAAGGAAGAAAATGACGGTACTTGACTAAGAAGCC

CCGGCTAAATACGT

>149 len=158 tot-seq=2469

TGGGGAATATTGCGCAATGGGGGGAACCCTGACGCAGCCATGCCGCGTGAATGAAGAAGG

CCTTCGGGTTGTAAAGTTCTTTCGGTAATGAGGAAGGGATGTTGTTAAATAGATAGCATC

ATTGACGTTAATTACAGAAGAAGCACCGGCTAACTCCG

>150 len=136 tot-seq=2416

TGGGGAATATTGCGCAATGGGGGAAACCCTGACGCAGCGACGCCGCGTGCGGGATGAAGG

CCTTCGGGTTGTAAACCGCTTTCAGCAGGGACGAGGCCGCAAGGTGACGGTACCTGCAGA

AGAAGCCCCGGCTAAC

>151 len=160 tot-seq=2410

TAGGGAATTTTCGGCAATGGGGGAAACCCTGACCGAGCAACGCCGCGTGAAGGAAGAAGG

TTTTCGGATTGTAAACTTCTGTTATAAAGGAAGAACGGCGGCTACAGGAAATGGTAGCCG

AGTGACGGTACTTTATTAGAAAGCCACGGCTAACTACGTG

>152 len=161 tot-seq=2392

TGGGGAATCTTCCGCAATGGACGAAAGTCTGACGGAGCAACGCCGCGTGAGTGAAGACGG

CCTTCGGGTTGTAAAGCTCTGTGATCCGGGACGAAAGAGCCTTGGGTAAATAGCCTAAGG

AAGTGACGGTACCGGAAAAGCAAGCCACGGCTAACTACGTG

>153 len=147 tot-seq=2329

TGAGGAATATTGGTCAATGGGCGAGAGCCTGAACCAGCCAAGTAGCGTGCAGGAAGACGG

CCCTATGGGTTGTAAACTGCTTTTATGCGGGGATAAAGTGCGCGACGTGTCGTGCATTGT

AGGTACCGCATGAATAAGGACCGGCTA

>154 len=156 tot-seq=2241

TGGGGAATATTGGACAATGGGCGAAAGCCTGATCCAGCCATGCCGCGTGTGTGAAGAAGG

CCTTTTGGTTGTAAAGCACTTTAAGTGGGGAGGAAAAGCTTATGGTTAATACCCATAAGC

CCTGACGTTACCCACAGAATAAGCACCGGCTAACTC

>155 len=144 tot-seq=2236

TGAGGAATATTGGTCAATGGCCGAGAGGCTGAACCAGCCAAGTCGCGTGAGGGAAGACGG

CCCTACGGGTTGTAAACCTCTTTTGTCAGGGAGCAAAGAACGTCACGTGTGGCGTCTCGA

GAGTACCTGAAGAAAAAGCATCGG

>156 len=134 tot-seq=2219

TGGGGAATATTGCACAATGGAGGAAACTCTGATGCAGCGACGCCGCGTGAGTGAAGAAGT

AGTTCGCTATGTAAAGCTCTATCAGCAGGGAAGATAGTGACGGTACCTGACTAAGAAGCT

CCGGCTAAATACGT

>157 len=214 tot-seq=2218

TGGGGAATTTTGGACAATGGGCGAAAGCCTGATCCAGCAATGCCGCGTGCAGGATGAAGG

CCTTCGGGTTGTAAACTGCTTTTGTACGGAACGAAAAAGCTTCTCCTAATACGAGAGGCC

CATGACGGTACCGTAAGAATAAGCACCGGCTAACTACGTGGGGGGAGCCGCGGTATCTGC

AGATCGGAAGGGGGTCGTGTAGGGAAAGAGGGGG

>158 len=159 tot-seq=2165

TGGGGAATTTTGGACAATGGGCGAAAGCCTGATCCAGCAATGCCGCGTGTGTGAAGAAGG

CCTTCGGGTTGTAAAGCACTTTTGTCCGGAAAGAAATCCTTGGCTCTAATACAGTCGGGG

GATGACGGTACCGGAAGAATAAGCACCGGCTAACTACGT

>159 len=146 tot-seq=2119

TGAGGAATATTGGTCAATGGGCGAGAGCCTGAACCAGCCAAGTCGCGTGAGGGAAGACGG

TCCTATGGATTGTAAACCTCTTTTGTCGGGGAGCAAAGAGGCCTCGTGAGGTCAAAAGTG

AGAGTACCCGAAGAAAAAGCATCGGC

>160 len=156 tot-seq=2038

TGAGGAATATTGGTCAATGGCCGAGAGGCTGAACCAGCCAAGTCGCGTGAGGGATGAAGG

TTCTATGGATCGTAAACCTCTTTTATAAGGGAATAAAGTGCGGGACGTGTCCCGTTTTGT

ATGTACCTTATGAATAAGGATCGGCTAACTCCGTGG

>161 len=152 tot-seq=2037

TAGGGAATTTTCGGCAATGGGCGAAAGCCTGACCGAGCAACGCCGCGTGAGTGATGACGG

CCTTCGGGTTGTAAAGCTCTGTTGTAAGAAAAGATAAACTAGTAGAGAATATTAGTTAGA

CATTATCTTACCAGAAAGCCACGGCTAACTAC

>162 len=160 tot-seq=2009

TAGGGAATCTTCCGCAATGGGCGAAAGCCTGACGGAGCAACGCCGCGTGAGTGAAGAAGG

ATTTCGGTTCGTAAAGCTCTGTTGTTAGGGAAGAATGATTGTGTAGTAACTATACACAGT

AGAGACGGTACCTAACCAGAAAGCCACGGCTAACTACGTG

>163 len=134 tot-seq=1919

GAATTTTCCGCAATGGGCGAAAGCCTGACGGAGCAATGCCGCGTGGAGGTAGAAGGCCCA

CGGGTCGTGAACTTCTTTTCCCGGAGAAGAAGCAATGACGGTATCTGGGGAATAAGCATC

GGCTAACTCTGTGG

>164 len=132 tot-seq=1909

TGGGGGATATTGCACAATGGGGGGAACCCTGATGCAGCGACGCCGCGTGAGTGAAGAAGT

AATTCGTTATGTAAAGCTCTATCAGCAGGGAAGAATAAATGACGGTACCTGAGTAAGAAG

CCCCGGCTAACT

>165 len=155 tot-seq=1893

TGGGGAATTTTGGACAATGGGCGCAAGCCTGATCCAGCAATGCCGCGTGCGGGAAGAAGG

CCTTCGGGTTGTAAACCGCTTTTGGCAGGAACGAAACGGTGTCCGCTAATACCGGGCGCT
AATGACGGTACCTGCAGAATAAGCACCGGCTAACT
>166 len=140 tot-seq=1887
TGGGGAATATTGCACAATGGGCGCAAGCCTGATGCAGCGACGCCGCGTGGGGGATGACGG
CCTTCGGGTTGTAAACTCCTTTCGTCAGGGACGAAGCGAAAGTGACGGTACCTGGATAAG
AAGCACCGGCTAACTACGTG
>167 len=135 tot-seq=1881
TGGGGGATATTGCGCAATGGGGGCAACCCTGACGCAGCAACGCCGCGTGAAGGATGAAGG
TTTTCGGATTGTAAACTTCTTTTATTAAGGACGAAAAATGACGGTACTTAATGAATAAGC
TCCGGCTAACTACGT
>168 len=132 tot-seq=1880
TGGGGAATATTGCACAATGGGGGGAACCCTGATGCAGCGACGCCGCGTGGGTGAGGAAGT
ACTTCGGTATGTAAAGCCCTATCAGCAGGGAAGAAAATGACGGTACCTGACTAAGAAGCC
CCGGCTAACTAC
>169 len=143 tot-seq=1800
TGGGGAATATTGCACAATGGGCGCAAGCCTGATGCAGCGACGCCGCGTGCGGGATGACGG
CCTTCGGGTTGTAAACCGCTTTTGACTGGGAGCAAGCCCTTCGGGGTGAGTGTACCTTTC
GAATAAGCACCGGCTAACTACGT
>170 len=159 tot-seq=1753
TGGGGAATATTGCACAATGGGCGCAAGCCTGATGCAGCCATGCCGCGTGTGTGAAGAAGG
CCTTCGGGTTGTAAAGCACTTTCAGTAAGGAGGAAAGGTTAAGTTTTAATAGAGCTTAGC
TGTGACGTTACTTACAGAAGAAGCACCGGCTAACTCCGT
>171 len=140 tot-seq=1723
TGGGGAATATTGCACAATGGGCGAAAGCCTGATGCAGCGACGCCGCGTGGGGGATGACGG
CCTTCGGGTTGTAAACCCCTTTCAGTAGGGAAGAAGCGAAAGTGACGGTACCTGCAGAAG
AAGCGCCGGCTAACTACGTG
>172 len=194 tot-seq=1719
TAGGGAATCTTCCACAATGGACGAAAGTCTGATGGAGCAATACCATGTGAACGATGAAGG
TCTTTTTGATTGTAAAGTTCTTTTATGAGGGATAACAACTGATAGTACCTCATGAATAAG

TGACAGCAAACTATGTGGGGGGAGCCGCGGTAATCAGCAGATCGGAAGAGCGTCGTGTAG

GGGGGGAGGGGAGA

>173 len=154 tot-seq=1695

TGAGGAATATTGGTCAATGGACGAGAGTCTGAACCAGCCAAGTAGCGTGCAGGATGACGG

CCCTATGGGTTGTAAACTGCTTTTGTTGGGGAATAACTGACATTACGTGTAATGTTTTGC

ATGTACCCTTCGAATAAGGACCGGCTAATTCCGT

>174 len=135 tot-seq=1686

TGGGGAATATTGCACAATGGGGGAAACCCTGATGCAGCGACGCCGCGTGAGTGAAGAAGT

ATTTCGGTATGTAAAGCTCTATCAGCAGGAACGAGACAAGACGGTACCTGACTAAGAAGC

CCCGGCTAACTACGT

>175 len=213 tot-seq=1673

TGGGGAATTTTGGACAATGGGGGAAACCCTGATCCAGCCATCCCGCGTGTATGATGAAGG

CCTTCGGGTTGTAAAGTACTTTTGGCAGAGAAGAAAAGGTACCTCCTAATACGAGGTACT

GCTGACGGTATCTGCAGAATAAGCACCGGCTAACTACGTGGGGGGAGCCGCGGTAATACG

AAGATCGGAAGAGCGTCGTGTAGGGAAAGAGTG

>176 len=139 tot-seq=1656

TGGGGAATATTGCACAATGGGGGAAACCCTGATGCAGCGACGCAGCGTGCGGGATGACGG

CCTTCGGGTTGTAAACCGCTTTCAGCAGGGAAGAAGCGCAAGTGACGGTACCTGCAGAAG

AAGTACCGGCTAACTACGT

>177 len=149 tot-seq=1559

TGGGGGATATTGCACAATGGGGGAAACCCTGATGCAGCGACGCCGCGTGAGTGAAGAAGT

ATTTCGGTACGTAAAGCTCTATCAGCAGGGAAGAAAAAAAGTCCACAGGACTTTGGACGG

TACCTGACTAAGAAGCCCCGGCTAACTAC

>178 len=160 tot-seq=1557

TGGGGAATATTGGACAATGGGCGAAAGCCTGATCCAGCCATGCCGCGTGTGTGAAGAAGG

TCTTCGGATTGTAAAGCACTTTAAGTTGGGAGGAAGGGCATTAACCTAATACGTTAGTGT

TTTGACGTTACCGACAGAATAAGCACCGGCTAACTTCGTG

>179 len=156 tot-seq=1547

TAGGGAATTTTCGTCAATGGGGGGAACCCTGAACGAGCAATGCCGCGTGAGTGAGGAAGG

TCTTCGGATCGTAAAGCTCTGTTGTAAGAGAAAAACGACATTCATAGGGAATGATGAGTG

AGTGATGGTATCTTACCAGAAAGTCACGGCTAACTA

>180 len=159 tot-seq=1533

TAGGGAATCTTCCGCAATGGGCGAAAGCCTGACGGAGCAACGCCGCGTGAGTGATGAAGG

TCTTCGGATCGTAAAACTCTGTTATTAGGGAAGAACAAATGTGTAAGTAACTATGCACGT

CTTGACGGTACCTAATCAGAAAGCCACGGCTAACTACGT

>181 len=138 tot-seq=1509

TGGGGAATATTGGGCAATGGAGGAAACTCTGACCCAGCAACGCCGCGTGAATGATGAAGG

TCTTCGGATTGTAAAGTTCTTTTCTAAGGGAAGAAGAAAGTGACGGTACCTTAGGAATAA

GCCTCGGCTAACTACGTG

>182 len=133 tot-seq=1497

TGGGGAATATTGGACAATGGGCGAAAGCCTGATCCAGCAATGCCGCGTGAGTGATGAAGG

CCTTAGGGTTGTAAAGCTCTTTTACCCGGGATGATAATGACAGTACCGGGAGAATAAGCT

CCGGCTAACTCCG

>183 len=134 tot-seq=1477

TGGGGAATATTGGACAATGGGCGCAAGCCTGATCCAGCCATGCCGCGTGAGTGATGAAGG

CCTTAGGGTTGTAAAGCTCTTTCGTCCGGGACGATAATGACGGTACCGGAAGAAGAAGCC

CCGGCTAACTTCGT

>184 len=162 tot-seq=1440

TGGGGAATTTTGGACAATGGGGGCAACCCTGATCCAGCAATGCCGCGTGTGTGAAGAAGG

CCTTCGGGTTGTAAAGCACTTTTGTCCGGAAAGAAATCGCGCTGGTTAATACCTGGCGTG

GATGACGGTACCGGAAGAATAAGCACCGGCTAACTACGTGGG

>185 len=157 tot-seq=1437

TGAGGAATATTGGTCAATGGGCGCGAGCCTGAACCAGCCAAGTCGCGTGAGGGATGACGG

TCCTACGGATTGTAAACCTCTTTTGCCGGGGAGCAAGCGTGCGTTCGTGAACGCGCGTCG

AGAGTACCCGGAGAAAAAGCATCGGCTAACTCCGTGG

>186 len=139 tot-seq=1421

TGGGGAATTTTGCACAATGGGCGGAAGCCTGATGCAGCGACGCCGTGTGGGGGATGAAGG

CCTTCGGGTTGTAAACTCCTTTCGTCAGGGACGAAGTTTGTTTGACGGTACCTGGATAAG

AAGCACCGGCTAACTACGT

>187 len=153 tot-seq=1404

TGAGGAATATTGGTCAATGGGCGAGAGCCTGAACCAGCCAAGTAGCGTGCAGGATGACGG

CCCTATGGGTTGTAAACTGCTTTTATAAGGGAATAAAGTGAGAGTCGTGACTCTTTTTGC

ATGTACCTTATGAATAAGGACCGGCTAATTCCG

>188 len=134 tot-seq=1401

TGGGGAATTTTGCACAATGGGGGAAACCCTGATGCAGCGACGCCGCGTGATTTAGAAGGC

CTTCGGGTTGTAAAAATCTTTTTACGGGATGAAAATGACAGTACCGTAAGAATAAGGTC

CGGCTAAATACGTG

>189 len=158 tot-seq=1380

TGGGGAATATTGCGCAATGGGGGCAACCCTGACGCAGCCATGCCGCGTGAATGAAGAAGG

CCTTCGGGTTGTAAAGTTCTTTCGGTAGCGAGGAAGGCATTTAGTTTAATAGACTAGGTG

ATTGACGTTAACTACAGAAGAAGCACCGGCTAACTCCG

>190 len=161 tot-seq=1363

TAGGGAATCTTCCACAATGGACGCAAGTCTGATGGAGCAACGCCGCGTGAGTGAAGAAGG

TTTTCGGATCGTAAAGCTCTGTTGTTAGAGAAGAACGTTGGTAGGAGTGGAAAATCTACC

AAGTGACGGTAACTAACCAGAAAGGGACGGCTAACTACGTG

>191 len=134 tot-seq=1354

TGGGGAATATTGCACAATGGGCGAAAGCCTGATGCAGCGACGCCGCGTGAGCGAAGAAGT

ATTTCGGTATGTAAAGCTCTATCAGCAGGGAAGAAAAAAATGACGGTACCTGACTAAGAA

GCCCCGGCTAACTA

>192 len=135 tot-seq=1353

TGGGGGATATTGCACAATGGGGGAAACCCTGATGCAGCGACGCCGCGTGTGGGAAGACGG

TCCTCTGGATTGTAAACCACTGTCCCCAGGGACGAAAATGACGGTACCTGGGGAGGAAGC

TCCGGCTAACTACGT

>193 len=135 tot-seq=1334

TGGGGAATATTGCACAATGGAGGGAACTCTGATGCAGCGACGCCGCGTGAGTGATGAAGG

AATTCGTTTCGTAAAACTCTGTTCTATGGGAAGAAAAGGACTGTACCATAGGAGAAAGCT

CCGGCTAAATACGTG

>194 len=135 tot-seq=1314

TGGGGAATATTGCACAATGGGGGAAACCCTGATGCAGCAACGCCGCGTGAGTGAAGAAGT

CATTCGTGATGTAAAGCTCTATCAGCAGGGAAGAAAATGACGGTACCTGAGTAAGAAGCC

CCGGCTAACTACGTG

>195 len=138 tot-seq=1296

TGGGGAATATTGGGCAATGGGCGCAAGCCTGACCCAGCAACGCCGCGTGAAGGAAGAAGG

CTTTCGGGTTGTAAACTTCTTTTGTCAGGGAAGAGTAGAAGACGGTACCTGACGAATAAG

CCACGGCTAACTACGTGG

>196 len=141 tot-seq=1260

TGGGGAATATTGCACAATGGGCGCAAGCCTGATGCAGCGACGCCGCGTGCGGGATGACGG

CCTTCGGGTTGTAAACCGCTTTTGATCGGGAGCAAGCCTTCGGGTGAGTGTACCTTTCGA

ATAAGCACCGGCTAACTACGT

>197 len=161 tot-seq=1228

TGGGGAATATTGGACAATGGGCGCAAGCCTGATCCAGCCATGCCGCGTGTGTGAAGAAGG

CCTTTTGGTTGTAAAGCACTTTAAGCGAGGAGGAGGCTACTTGGATTAATACTCTAGGAT

AGTGGACGTTACTCGCAGAATAAGCACCGGCTAACTCTGTG

>198 len=201 tot-seq=1226

TGGGGAATCTTGCACAATGGGCGAAAGCCTGATGCAGCGACGCCGCGTGCGGGATGAAGG

CCTTCGGGTTGTAAACCGCTTTTGATTGGGAGCAAGCCTTCGGGTGAGTGTACCTTTCGA

ATAAGCGCCGGCTAACTACGTGGGGGGAGCCGCGGTATGACCAGATCGGAAGAGCATCGT

GTAGGGAAAGAGTGGGCCAAT

>199 len=158 tot-seq=1219

TGGGGAATATTGCACAATGGGCGCAAGCCTGATGCAGCCATGCCGCGTGTATGAAGAAGG

CCTTCGGGTTGTAAAGTACTTTCAGCGAGGAGGAAGGCATTGTGGTTAATAACCGCAGTG

ATTGACGTTACTCGCAGAAGAAGCACCGGCTAACTCCG

>200 len=131 tot-seq=1215

GCACGCAAGTTACCCACTCCGGACCTGGGGATGTAGTGATGAAAAATAATACAGGACTCT

TTTGAGGGCCTGTAATTGGAATGAGTCCACTTTAAATCCTTTAACGAGGATCCATTGGAG

GGCAAGTCTGG

>201 len=154 tot-seq=1215

TGAGGAATATTGGTCAATGGGCGCTAGCCTGAACCAGCCAAGTAGCGTGAAGGATGACTG

CCCTATGGGTTGTAAACTTCTTTTATATGGGAATAAAGTGCAGTATGTATACTGCTTTGC

ATGTACCTTATGAATAAGGATCGGCTAACTCCGT

>202 len=158 tot-seq=1187

TGGGGAATTTTGGACAATGGGGGAAACCCTGATCCAGCCATGCCGCGTGCGGGATGAAGG

CCTTCGGGTTGTAAACCGCTTTTGTCAGAGACGAAAAGGGACGTACGAATAATACGTTCC

GCTGACGGTATCTGAAGAATAAGCACCGGCTAACTACG

>203 len=128 tot-seq=1186

TGGGGAATATTGCACAATGGGCGAAAGCCTGATGCAGCAACGCCGCGTGAAGGATGAAGG

GTTTCGGCTCGTAAACTTCTATCAATAGGGAAGAAACAAATGACGGTACCTAAATAAGAA

GCCCCGGC

>204 len=138 tot-seq=1172

TGGGGAATTTTCCGCAATGGGCGCAAGCCTGACGGAGCAATACCGCGTGAGGGATGACGG

CCTTTGGGTTGTAAACCTCTTTTATCAAGGAGGAAGTTCTGACGTGTACTTGATGAATAA

GCATCGGCTAACTCCGTG

>205 len=134 tot-seq=1170

TGGGGGATATTGCACAATGGGGGGAACCCTGATGCAGCGACGCCGCGTGGGCGATGGAGT

GCTTCGGCATGTAAAGCCCTATCAGCAGGGAAGAAAATGACGGTACCTGACTAAGAAGCC

CCGGCTAAATACGT

>206 len=135 tot-seq=1123

TGGGGAATATTGGACAATGGGGGCAACCCTGATCCAGCCATGCCGCGTGAGTGATGAAGG

CCCTAGGGTTGTAAAGCTCTTTTGTGCGGGAAGATAATGACGGTACCGCAAGAATAAGCC

CCGGCTAACTTCGTG

>207 len=160 tot-seq=1111

TGGGGAATCTTCCGCAATGGACGAAAGTCTGACGGAGCAACGCCGCGTGAGTGATGAAGG

TCTTCGGATTGTAAAGCTCTGTTAATCGGGACGAAAGAGTTCTGTGTGAATAATGCAGAA

AAGTGACGGTACCGGAATAGAAAGCCACGGCTAACTACGT

>208 len=134 tot-seq=1099

TGGGGAATCTTAGACAATGGGGGCAACCCTGATCTAGCCATGCCGCGTGAGTGATGAAGG

CCTTAGGGTTGTAAAGCTCTTTCAGCTGGGAAGATAATGACGGTACCAGCAGAAGAAGCC

CCGGCTAACTCCGT

>209 len=157 tot-seq=1089

TGAGGAATATTGGTCAATGGGCGCGAGCCTGAACCAGCCAAGTCGCGTGAGGGAAGACGG

TCCTATGGATTGTAAACCTCTTTTGTCGGGGAGCAATGGTCTTCACGTGTGGAGACTGTG

AGAGTACCCGAAGAAAAAGCATCGGCTAACTCCGTGG

>210 len=134 tot-seq=1086

TGGGGAATCTTGCGCAATGGGGGGAACCCTGACGCAGCGACGCCGCGTGCGGGACGGAGG

CCTTCGGGTCGTAAACCGCTTTCAGCAGGGAAGAGTCAAGACTGTACCTGCAGAAGAAGC

CCCGGCTAACTACG

>211 len=152 tot-seq=1061

TGAGGAATATTGGTCAATGGCCGAGAGGCTGAACCAGCCAAGTCGCGTGAGGGATGACGG

CCCTACGGGTTGTAAACCTCTTTTGTCAGGGAGCAAGGGGCGCCACGTGTGGCGCTGTGA

GAGTACCTGAAGAAAAAGCATCGGCTAACTCC

>212 len=157 tot-seq=1046

TGGGGAATATTGCACAATGGGGGGAACCCTGATGCAGCCATGCCGCGTGAATGAAGAAGG

CCTTCGGGTTGTAAAGTTCTTTCGGTGACGAGGAAGGTTGATAGGTTAATAGCCTATCAA

ATTGACGTTAATCACAGAAGAAGCACCGGCTAACTCC

>213 len=157 tot-seq=1039

TAGGGAATCTTCCGCAATGGGCGAAAGCCTGACGGAGCAACGCCGCGTGAGTGATGAAGG

GTTTCGGCTCGTAAAACTCTGTTATTAGGGAAGAACAAATGTGTAAGTAACTGTGCACAT

CTTGACGGTACCTAATCAGAAAGCCACGGCTAACTAC

>214 len=159 tot-seq=1039

TAGGGAATCTTCCGCAATGGACGCAAGTCTGACGGAGCAACGCCGCGTGAGTGAAGAAGG

TTTTCGGATCGTAAAACTCTGTTGTTAGAGAAGAACAAGTGCTAGAGTAACTGTTAGCGC

CTTGACGGTATCTAACCAGAAAGCCACGGCTAACTACGT

>215 len=130 tot-seq=990

GGTAGAGGAAAACAGGGGGCCCTAAACAGGGCTAAGTTCCAGCTTACCACTCATAAAAAT

AGTGGTACTTTTGGCCAGCTGGGTTGTTATGAAAAGTAGAAATAATAAATGTGAACAACC

TATCGTGATG

>216 len=158 tot-seq=990

TAGGGAATCTTCCACAATGGACGAAAGTCTGATGGAGCAACGCCGCGTGAACGATGAAGG

CCCTCGGGTCGTAAAGTTCTGTTGTAAGGGAAGAACAAGTGCCGCAGGCAATGGCGGCAC

CTTGACGGTACCTTGCGAGAAAGCCACGGCTAACTACG

>217 len=136 tot-seq=973

GCATGCAAATTACCCACTCCTGACTCGAGGAGGTAGTGATGAAACATAACAATACAGGAC

TCTTTCGAGGTCCTGTAATTGGAATGAGTCCACTTTAAATCCTTTAACGAAGATCCATTG

GAGGGCAAGTTTAGTG

>218 len=207 tot-seq=924

TAGGGAATTTTCGTCAATGGGGGAAACCCTGAACGAGCAATGCCGCGTGAGTGAAGAAGG

CCTTCGGGTCGTAAAGCTCTGTTGTAAGAGAAAAACGGTAATGATAGGGAATGATCATTA

AGTGATGGTATCTTACGAGGAAGCCACGGCTAACTACGTGGGGGGAGCCGCGGTAATATG

GAGATCGGAAGAGCGTCGTGTAGGGAA

>219 len=132 tot-seq=916

TGGGGGATATTGCACAATGGGGGGAACCCTGATGCAGCGACGCCGCGTGGGCGATGGAGC

GCCTCGGCGCGTAAAGCCCTATCAGCAGGGAAGAAGATGACGGTACCTGACTAAGAAGCC

CCGGCTAAATAC

>220 len=160 tot-seq=908

TGGGGAATATTGCACAATGGGCGCAAGCCTGATGCAGCCATGCCGCGTGTATGAAGAAGG

CCTTCGGGTTGTAAAGTACTTTCAGTCGTGAGGAAGGGGGTATCGTTAATAGCGGTATTC

TTTGACGTTAGCGACAGAAGAAGCACCGGCTAACTCCGTG

>221 len=160 tot-seq=907

TGGGGAATCTTCCGCAATGGACGAAAGTCTGACGGAGCAACGCCGCGTGAGTGATGACGG

CCTTCGGGTTGTAAAGCTCTGTTAATCGGGACGAATGGTTCTTGTGCGAATAGTGCGAGG

ATTTGACGGTACCGGAATAGAAAGCCACGGCTAACTACGT

>222 len=134 tot-seq=906

TGGGGAATATTGCACAATGGGGGAAACCCTGATGCAGCGACGCCGCGTGAGTGAAGAAGT

AATTCGTTATGTAAAGCTCTATCAGCAGGGAAGAAACAGACGGTACCTGAGTAAGAAGCC

CCGGCTAACTACGT

>223 len=156 tot-seq=902

TGAGGAATATTGGTCAATGGAGGAAACTCTGAACCAGCCAAGTCGCGTGAAGGAAGAATG

TCCTAAGGATTGTAAACTTCTTTAGCGAGTGAGTAAGGACTTCCACGCGTTGGGAGTTTG

AAAGTAGCTCGAGAATAAGTATCGGCTAACTCCGTG

>224 len=187 tot-seq=897

TCTCCTGGTACTCACTCCCTCATGCTACCTTAGCCTAGATCAACCCCCTCACCTTCCTCT

GTCAGGAGTCAGAGGGAGAGGCCCAACCTTCCCATGGCAGCTGCCATACTGGCCTGAGCC

ATACTCAGGCCATACTGAGTGCTCAGGCCATACTATGCCATACTGGCCCCCTTGACCCTG

CCTCCTT

>225 len=109 tot-seq=895

ATTATTAAAACAGGAAAAGCAAATTGGCTAAGTTTATGACAAACTACAGTACTGGGACAA

ACATATCTGTGCCTTTGAAAGCCCTAATACCCAAAGTGTGATCTGAAGA

>226 len=159 tot-seq=894

TGGGGAATCTTCCGCAATGGGCGCAAGCCTGACGGAGCAATGCCGCGTGAGTGAAGAAGG

CCTTCGGGTTGTAAAACTCTGTCCTCATCCAAGAGAGAGACCTGTAGTAACTGCCAGGTC

TAGGACGGTAGATGAGGAGGAAGCCCCGGCTAACTATGT

>227 len=159 tot-seq=885

TGGGGAATCTTCCGCAATGGGCGAAAGCCTGACGGAGCAACGCCGCGTGAGTGAAGACGG

CCTTCGGGTTGTAAAACTCTGTTATACGGGACGAATGTAGGCATGGTTAATACCCATGCG

TAGTGACGGTACCGTAAGAGAAAGCCACGGCTAACTACG

>228 len=137 tot-seq=876

TGGGGGATATTGCACAATGGGCGAAAGCCTGATGCAGCGACGCCGCGTGAGGGAAGACGG

CCTTCGGGTTGTAAACCTCTGTCATTCGGGACGAATTAGATGACGGTACCGAAGAAGGAA

GCTCCGGCTAACTACGT

>229 len=138 tot-seq=868

TGGGGAATATTGCGCAATGGGGGGAACCCTGACGCAGCAACGCCGCGTGAAGGAGGAAGT

ATTTCGGTATGTAAACTTCTATCAGCAGGGAAGAAGGAAGTGACGGTACCTGCATAAGAA

GCCCCGGCTAATTACGTG

>230 len=137 tot-seq=850

TGGGGAATATTGCACAATGGGGGAAACCCTGATGCAGCAACGCCGCGTGAAGGAAGACGG

TTTTCGGATTGTAAACTTCTATCAATAGGGACGAAAGAAATGACGGTACCTAAATAAGAA

GCCCCGGCTAACTACGT

>231 len=160 tot-seq=848

TAGGGAATTTTCGTCAATGGAGGGAACTCTGAACGAGCAATGCCGCGTGAGTGAAGAAGG

TCTTCGGATCGTAAAGCTCTGTTGCAAGAGAAAAACGACAAGCACAGGGAATGGTGATTG

AGTGATGGTATCTTGCGAGGAAGCCACGGCTAACTACGTG

>232 len=180 tot-seq=844

TGGGGAATCTTGCGCAATGGGCGAAAGCCTGACGCAGCCATGCCGCGTGAATGATGAAGG

TCTTAGGATTGTAAAATTCTTTCACCGGGGACGATAATGACGGTACCCGGAGAAGAAGCC

CCGGCTAACTTCGTGGGGGGAGCCGCGGTATAAGCAGATCGGAAGCGCGTCGTGTAGGGA

>233 len=155 tot-seq=843

TGAGGAATATTGGTCAATGGACGCAAGTCTGAACCAGCCATGCCGCGTGCAGGATGACGG

CTCTATGAGTTGTAAACTGCTTTTGTACGAGGGTAAACGCAGATACGTGTATCTGTCTGA

AAGTATCGTACGAATAAGGATCGGCTAACTCCGTG

>234 len=153 tot-seq=819

TGAGGAATATTGGTCAATGGGCGAGAGCCTGAACCAGCCAAGTCGCGTGAAGGATGACGG

CCCTACGGGTTGTAAACTTCTTTTGTCAGGGAGCAATTGAGTCCACGTGTGGGCTTAGCG

AGAGTACCTGAAGAAAAAGCATCGGCTAACTCC

>235 len=137 tot-seq=811

TGGGGAATATTGGGCAATGGAGGAAACTCTGACCCAGCAACGCCGCGTGGAGGAAGAAGG

TTTTCGGATCGTAAACTCCTGTCCTTGGAGACGAGTAGAAGACGGTATCCAAGGAGGAAG

CCCCGGCTAACTACGTG

>236 len=155 tot-seq=807

TGAGGAATATTGGTCAATGGGCGGAAGCCTGAACCAGCCAAGTAGCGTGCAGGATGACGG

CCCTACGGGTTGTAAACTGCTTTTATACGGGAATAACGCGCCTTACGTGTAGGGCTTTGC

ATGTACCGTATGAATAAGGACCGGCTAATTCCGTG

>237 len=161 tot-seq=801

TGGGGAATCTTCCGCAATGGGCGAAAGCCTGACGGAGCAACGCCGCGTGAGTGAAGACGG

CCTTCGGGTTGTAAAGCTCTGTTATACGGGACGAATAGTCTTATGGTTAATACCCATGAG

AAGTGACGGTACCGTAAGAGAAAGCCACGGCTAACTACGTG

>238 len=136 tot-seq=783

TGGGGAATTTTGCGCAATGGGGGCAACCCTGACGCAGCAACGCCGCGTGCGGGACGAAGG

CGTCCGCGTCGTAAACCGCTTTCAGCGGGGAACACCTAAACGAGGGTACCCGCAGAAGAA

GCCCCGGCTAAATACG

>239 len=134 tot-seq=762

TAGGGAATTTTGCGCAATGGGGGCAACCCTGACGCAGCAACGCCGCGTGATCGATTAAGC

CCTTCGGGGTGTAAAGATCTGTCAGTGGGGACGAAAGATGACGGTACCCACAGAGGAAGC

ACCGGCTAACTCCG

>240 len=155 tot-seq=743

TGAGGAATATTGGTCAATGGGCGAGAGCCTGAACCAGCCAAGTCGCGTGAAGGATGAAGG

ATCTATGGTTTGTAAACTTCTTTTATATGGGAATAAAGTGAGGAACGTGTTCCTTTTTGT

ATGTACCATATGAATAAGCATCGGCTAACTCCGTG

>241 len=171 tot-seq=742

TGAGGAATATTGGTCAATGGGCGTGAGCCTGAACCAGCCAAGTAGCGTGCAGGAAGACGG

CCCTATGGGTTGTAAACTGCTTTTATGCGGGGATAAAGGAGTCCACGTGTGGATTTTTGC

AGGTACCGCATGAATAAGGACCGGCTAATTCCGTGGGGGGAGACGGGGAAT

>242 len=135 tot-seq=726

TGAGGGATATTGGTCAATGGGGGAAACCCTGAACCAGCAACGCCGCGTGAGGGAAGACGG

TTTTCGGATTGTAAACCTCTGTCCTCTGTGAAGATAGTGACGGTAGCAGAGGAGGAAGCT

CCGGCTAACTACGTG

>243 len=153 tot-seq=713

TGAGGAATATTGGACAATGGGTTAGCGCCTGATCCAGCCATCCCGCGTGAAGGACGACGG

CCCTATGGGTTGTAAACTTCTTTTGTATAGGGATAAACCTACTCTCGTGAGAGTAGCTGA

AGGTACTATACGAATAAGCACCGGCTAACTCCG

>244 len=155 tot-seq=711

TGAGGAATATTGGTCAATGGGCGAGAGCCTGAACCAGCCAAGTAGCGTGCAGGATGACGG

CCCTATGGGTTGTAAACTGCTTTTATGCGGGGATAACCGGCCTCACGTGTGAGGCCCTGC

AGGTACCGCATGAATAAGGACCGGCTAATTCCGTG

>245 len=135 tot-seq=704

TGGGGAATTTTGCACAATGGAGGAAACTCTGATGCAGCGACGCCGCGTGAACGAAGAAGG

TCTTCGGATTGTAAAGTTCTGTCCTGGGTGAAGATAATGACGGTAACTCAGGAGGAAGCC

CCGGCTAACTACGTG

>246 len=160 tot-seq=698

TAGGGAATCTTCCGCAATGGACGCAAGTCTGACGGAGCAACGCCGCGTGAGTGATGAAGG

GTTTCGGCTCGTAAAACTCTGTTGTTAAGGAAGAACACTTGAGGTAGTAACTGGCCTTGA

GATGACGGTACTTAACCAGAAAGCCACGGCTAACTACGTG

>247 len=139 tot-seq=689

TGGGGAATATTGCACAATGGGCGGAAGCCTGATGCAGCGACGCCGCGTGGGGGATGACGG

CCTTCGGGTTGTAAACTCCTTTCACCATCGACGAAGGTTTTCTGACGGTAGATGGAGAAG

AAGCACCGGCTAACTACGT

>248 len=136 tot-seq=679

ACACTCAAATTACCCACTCCTGACACGGGGAGGTAGTGACGAAAAATAACAATACAGGAC

TCTTTCTAGGCCCTGTAATTGGAATGAGTGCACTTTAAATCTTTTAATGAGGATCAATTG

GAGGGCAAGTCTGGTG

>249 len=138 tot-seq=665

TGGGGAATATTGGGCAATGGGGGAAACCCTGACCCAGCAACGCCGCGTGAAGGAAGAAGG

CTTTCGGGTTGTAAACTTCTTTTACCAGGGACGAAGGACGTGACGGTACCTGGAGAAAAA

GCCACGGCTAACTACGTG

>250 len=160 tot-seq=648

TAGGGAATTTTCGTCAATGGGGGGAACCCTGAACGAGCAATGCCGCGTGTGTAAAGAAGG

TCTTCGGATCGTAAAGCACTGTTGTAAGTGAAGAATGCCACATAGAGGAAATGCTATGTG

GGTGACGGTAGCTTACCAGAAAGCCACGGCTAACTACGTG

>251 len=161 tot-seq=648

TAGGGAATCTTCCACAATGGACGCAAGTCTGATGGAGCAACGCCGCGTGAGTGAAGAAGG

TTTTCGGATCGTAAAGCTCTGTTGTAAGAGAAGAACGTGTGTGAGAGTGGAAAGTTCACA

CAGTGACGGTAACTTACCAGAAAGGGACGGCTAACTACGTG

>252 len=160 tot-seq=637

TAGGGAATTTTCGGCAATGGGGGAAACCCTGACCGAGCAACGCCGCGTGAGGGAAGAAGT

ATTTCGGTATGTAAACCTCTGTTATAAAGGAAGAACGGTATGAATAGGAAATGATTCATA

AGTGACGGTACTTTATGAGAAAGCCACGGCTAACTACGTG

>253 len=178 tot-seq=635

TAGGGAATCTTCGGCAATGGACGGAAGTCTGACCGAGCAACGCCGCGTGAGTGAAGAAGG

TTTTCGGATCGTAAAGCTCTGTTGTTGGTGAAGAAGGATAGAGGTAGTAACTGGCCTTTA

TTTGACGGTAATCAACCAGAAAGTCACGGCTAACTACGTGGGGGGAGCCGCGGAATGC

>254 len=140 tot-seq=618

TGGGGAATATTGCACAATGGGCGCAAGCCTGATGCAGCGACGCCGCGTGAGGGATGACGG

CCTTCGGGTTGTAAACCTCTGTTAGCATCGAAGAAGCGAAAGTGACGGTAGGTGCAGAGA

AAGCGCCGGCTAACTACGTG

>255 len=159 tot-seq=611

TAGGGAATTTTCGGCAATGGGCGAAAGCCTGACCGAGCAACGCCGCGTGAACGAAGAAGG

TCTTCGGATTGTAAAGTTCTGTTATAGAGGAAGAACGGTGTACGTAGGAAATGACGTACA

AGTGACGGTACTCTGTGAGGAAGCCACGGCTAACTACGT

>256 len=158 tot-seq=608

TGGGGAATTTTGGACAATGGGGGCAACCCTGATCCAGCCATGCCGCGTGCAGGATGAAGG

CCTTCGGGTTGTAAACTGCTTTTGTCAGGGACGAAAAGGACCGTGTTAATACCATGGTCT

GCTGACGGTACCTGAAGAATAAGCACCGGCTAACTACG

>257 len=136 tot-seq=603

GCACGCAAATTACCCACTCTCGACCCGGGGAGGTAGTGACGAAAAATAACAATACAGGAC

TCTTTTGAGGCCCTGTAATTGGAATGAGTCCACTTTAAATCCTTTAACAAGGATCCATTG

GAGGGCAAGTCTGGTG

>258 len=135 tot-seq=602

TAGGGAATATTGCGCAATGGGGGAAACCCTGACGCAGCAACGCCGCGTGGAGGATGACAC

TTTTCGGAGCGTAAACTCCTTTTATCGGGAAAGAATAATGACGGTACCCGATGAATAAGC

ACCGGCTAACTCCGT

>259 len=138 tot-seq=597

TGGGGGATATTGCGCAATGGGGGAAACCCTGACGCAGCAACGCCGCGTGAAGGAAGAAGG

TCTTCGGATTGTAAACTTCTTTTGTCAGGGACGAAGAAAGTGACGGTACCTGACGAATAA

GCTCCGGCTAACTACGTG

>260 len=177 tot-seq=592

TAGGGAATCTTCGGCAATGGGGGCAACCCTGACCGAGCAACGCCGCGTGAGTGAAGAAGG

TTTTCGGATCGTAAAGCTCTGTTGTAAGAGAAGAACGTGTGTGAGAGTGGAAAGTTCACA

CAGTGACGGTAACTTACCAGAAAGGGACGGCTAACTACGTGCCAGCAGCCGCGGTAA

>261 len=138 tot-seq=587

TGGGGAATATTGGGCAATGGGCGAAAGCCTGACCCAGCAACGCCGCGTGAGGGAAGAAGG

TTTTCGGATTGTAAACCTCTGTCCTTGGTGACGAAGGAAGTGACGGTAGCCAAGGAGGAA

GCCCCGGCTAACTACGTG

>262 len=135 tot-seq=586

TGGGGAATATTGCACAATGGGGGAAACCCTGATGCAGCAACGCCGCGTGGGTGAAGGAGC

GTTTCGGCGCGTAAAGCCCTGTCAGCGGGGAAGAAGAAAGACGGTACCCGACCAAGAAGC

CCCGGCTAACTACGT

>263 len=136 tot-seq=586

TAGGGAATATTGCTCAATGGGGGAAACCCTGAAGCAGCAACGCCGCGTGGAGGATGACAC

TTTTCGGAGCGTAAACTCCTTTTCTTTGGGAAGAATAATGACGGTACCAAAGGAATAAGC

ACCGGCTAACTCCGTG

>264 len=155 tot-seq=585

TGAGGAATATTGGTCAATGGGCGAGAGCCTGAACCAGCCAAGTAGCGTGAAGGATGACCG

CCCTATGGGTTGTAAACTTCTTTTATATGGGAATAAAGGGTGCCACGTGTGGCATTTTGT

ATGTACCATATGAATAAGGATCGGCTAACTCCGTG

>265 len=144 tot-seq=581

TGGGGAATATTGCACAATGGGCGCAAGCCTGATGCAGCGACGCCGCGTGGGGGATGAAGG

CCTTCGGGTTGTAAACTCCTTTCGCTATCGACGAAGCCACTTGTGGTGACGGTAGGTAGA

TAAGAAGCACCGGCTAACTACGTG

>266 len=139 tot-seq=577

TGGGGAATATTGGACAATGGACCAAAAGTCTGATCCAGCAATTCTGTGTGCACGATGACG

GTCTTAGGATTGTAAAGTGCTTTCAATCGGGAAAAAGAAAGTGATGGTACCGATAGAAGA

AGCGACGGCTAAATACGTG

>267 len=140 tot-seq=573

TGGGGAATATTGCACAATGGGCGCAAGCCTGATGCAGCGACGCCGCGTGAGGGATGACGG

CCTTCGGGTTGTAAACCTCTGTTAGCAGGGAAGAAGAGAGATTGACGGTACCTGCAGAGA

AAGCGCCGGCTAACTACGTG

>268 len=153 tot-seq=560

TGGGGAATATTGCACAATGGGCGAAAGCCTGATGCAGCGACGCCGCGTGAGGGATGGAGG

CCTTCGGGTTGTAAACCTCTTTCAGTAAGGGTGAAGGCTCTCTTTTGTGGGGGGTTGACA

GTACTTGCAGAAGAAGCGCCGGCTAACTACGTG

>269 len=135 tot-seq=554

TGGGGAATATTGCACAATGGGGGGAACCCTGATGCAGCAATGCCGCGTGGGTGAAGAAGC

ACTCCGGTGCGTAAAGCCCTGTCAGCAGGGAAGAAAATGACAGTACCTGGACAAGAAGCC

CCGGCTAACTACGTG

>270 len=159 tot-seq=551

TGGGGAATTTTGGACAATGGGCGCAAGCCTGATCCAGCAATGCCGCGTGAGTGAAGAAGG

CCTTCGGGTTGTAAAGCTCTTTTGTCAGGGAAGAAACGGTAGAGGCTAATATCCTTTGCT

AATGACGGTACCTGAAGAATAAGCACCGGCTAACTACGT

>271 len=136 tot-seq=546

TGGGGAATATTGCACAATGGGCGCAAGCCTGATGCAGCAACGCCGCGTGAACGAAGAAGG

CCTTCGGGTCGTAAAGTTCTGTCCATAGGGAAGAAACAAATGACGGTACCTATGGAGGAA

GCCCCGGCTAAATACG

>272 len=178 tot-seq=544

TAGGGAATCTTCCACAATGGGCGAAAGCCTGATGGAGCAACGCCGCGTGAGTGAAGAAGG

TTTTCGGATCGTAAAGCTCTGTTGTTGGTGAAGAAGGATAGAGGTAGTAACTGGCCTTTA

TTTGACGGTAATCAACCAGAAAGTCACGGCTAACTACGTGGGGGGAGCCGCGGTAATG

>273 len=141 tot-seq=540

TAGGGAATTTTCGGCAATGGGGGGAACCCTGACCGAGCAACGCCGCGTGAACGAAGAAGT
TATTCGTAATGTAAAGTTCTTTTATCAGGGAAGAAAGAGGAATTTGACGGTACTTGAGGA
ATAAGCTCCGGCTAACTACGT
>274 len=160 tot-seq=523
TAGGGAATTTTCGTCAATGGGCGCAAGCCTGAACGAGCAATGCCGCGTGAACGAGGAAGG
TCTTCGGATCGTAAAGTTCTGTTGAAGGGGAAAAAGAGCGGAGAGAGGAAATGGTCTCCG
ATTGATATTACCCTTCGAGGAAGTCACGGCTAACTACGTG
>275 len=138 tot-seq=522
TGGGGAATATTGCGCAATGGGGGAAACCCTGACGCAGCAACGCCGCGTGATTGAAGAAGG
CCTTCGGGTTGTAAAGATCTTTAATCAGGGACGAAACAAATGACGGTACCTGAAGAATAA
GCTCCGGCTAACTACGTG
>276 len=160 tot-seq=519
TGGGGAATATTGGACAATGGGGGGAACCCTGATCCAGCAATGCCGCGTGTGTGAAGAAGG
CCTGAGGGTTGTAAAGCACTTTCAGTGGGGAGGAGGATTGATAGGTTAAGAGCTGATTGA
TTGGACGTTACCCACAGAAGAAGCACCGGCTAACTCCGTG
>277 len=160 tot-seq=517
TGGGGAATATTGCACAATGGGCGCAAGCCTGATGCAGCCATGCCGCGTGTGTGAAGAAGG
CCTTCGGGTTGTAAAGCACTTTCAGCGGGGAGGAAGGCATTAAGGTTAATAACCTTAGTG
ATTGACGTTACCCGCAGAAGAAGCACCGGCTAACTCCGTG
>278 len=160 tot-seq=516
TGGGGAATTTTGGACAATGGGCGAAAGCCTGATCCAGCCATGCCGCGTGCAGGATGAAGG
CCTTCGGGTTGTAAACTGCTTTTGTACGGAACGAAAAGACTCTGGTTAATACCTGGGGTC
CATGACGGTACCGTAAGAATAAGCACCGGCTAACTACGTG
>279 len=137 tot-seq=513
TGGGGAATATTGGGCAATGGAGGAAACTCTGACCCAGCAATGCCGCGTGAATGAAGAAGG
TCTTCGGATTGTAAAGTTCTTTAGTCAGGGAAGAAGTCAATGACGGTACCTGAAGAATAA
GCTCCGGCTAACTACGT
>280 len=152 tot-seq=507
TGAGGAATATTGGTCAATGGGCGAGAGCCTGAACCAGCCAAGTAGCGTGCAGGATGACGG

CCCTCCGGGTTGTAAACTGCTTTTAGTTGGGAATAAAACAGCGGACTTGTCCGCTCTTGT

ATGTACCTTCAGAAAAAGGACCGGCTAATTCC

>281 len=160 tot-seq=504

TGGGGAATTTTGGACAATGGGCGAAAGCCTGATCCAGCAATGCCGCGTGTGTGAAGAAGG

CCTTCGGGTTGTAAAGCACTTTTGTCCGGAAAGAAATGGCTCTGGTTAATACCTGGGGTC

GATGACGGTACCGGAAGAATAAGGACCGGCTAACTACGTG

>282 len=142 tot-seq=502

TGGGGAATATTGCACAATGGACGGAAGTCTGATGCAGCGACGCCGCGTGGGGGATGAAGG

CTTTCGGGTTGTAAACTCCTTTCAGTACAGAAGAAGCTTTATTGTGACGGTATGTGCAGA

AGAAGCGCCGGCTAACTACGTG

>283 len=137 tot-seq=499

TGGGGAATATTGGGCAATGGACGCAAGTCTGACCCAGCAACGCCGCGTGAAGGAAGAAGG

CTTTCGGGTTGTAAACTTCTTTTAAGGGGGAAGAGTAGAAGACGGTACCCCTTGAATAAG

CCACGGCTAACTACGTG

>284 len=134 tot-seq=493

TGGGGGATATTGCACAATGGGGGGAACCCTGATGCAGCGACGCCGCGTGGGTGAAGGAGC

GTTTCGGCGCGTAAAGCCCTATCGGCAGGGAAGAAGGAGGACGGTACCTGACTAAGAAGC

CCCGGCTAACTACG

>285 len=159 tot-seq=491

TGGGGAATATTGCACAATGGGCGCAAGCCTGATGCAGCCATGCCGCGTGTGTGAAGAAGG

CCTTCGGGTTGTAAAGCACTTTCAGCGAGGAGGAAGGGTTCAGTGTTAATAGCACTGTTC

ATTGACGTTACTCGCAGAAGAAGCACCGGCTAACTCCGT

>286 len=140 tot-seq=485

TGGGGAATATTGCACAATGGGCGCAAGCCTGATGCAGCAACGCCGCGTGAGGGACGACGG

CCTTCGGGTTGTAAACCTCTTTTAGCAGGGAAGAAGCGAAAGTGACGGTACCTGCAGAAA

AAGCACCGGCTAACTACGTG

>287 len=135 tot-seq=484

TGGGGGATATTGCACAATGGGGGAAACCCTGATGCAGCAACGCCGCGTGTGGGAAGAAGG

TTTTCGGATTGTAAACCACTGTTCTTGGTGAAGATAATGACGGTAACCAAGGAGAAAGCT

CCGGCTAACTACGTG

>288 len=137 tot-seq=478

TGGGGAATATTGCACAATGGGGGGAACCCTGATGCAGCGACGCCGCGTGAGTGAAGAAGT

ATTTCGGTACGTAAAGCTCTATCAGCAGGGAAGAAAAAAGGACGGTACCTGAGTAAGAAG

CCCCGGCTAACTACGTG

>289 len=137 tot-seq=477

TGGGGAATATTGCACAATGGGCGAAAGCCTGATGCAGCAACGCCGCGTGAGGGAAGACGG

TTTTCGGATTGTAAACCTCTGTTTTCGGTGACGAACAAATGACGGTAACCGAGTAGGAAG

CCACGGCTAACTACGTG

>290 len=176 tot-seq=477

TAGGGAATCTTCCGCAATGGACGAAAGTCTGACGGAGCAACGCCGCGTGAGTGATGAAGG

ATTTCGGTTCGTAAAGCTCTGTTGTTAGGGAAGAACAAGTGCCGTTCGAATAGGTCGGCA

CCTTGACGGTACCTAACCAGAAAGCCACGGCTAACTACGTGGGGGGAGCCGAGGAA

>291 len=134 tot-seq=471

TGGGGAATATTGCACAATGGGCGAAAGCCTGATGCAGCGACGCCGCGTGAGTGAAGAAGT

ATCTCGGTATGTAAAGCTCTATCAGCAGGGAAGAAAATGACGGTACCTGACTAAGAAGCC

CCGGCTAACTACGT

>292 len=155 tot-seq=469

TGAGGAATATTGGTCAATGGGCGCGAGCCTGAACCAGCCAAGTAGCGTGAAGGATGACTG

CCCTACGGGTTGTAAACTTCTTTTATAAAGGAATAAAGTGAGGCACGTGTGCCTTTTTGT

ATGTACTTTATGAATAAGGATCGGCTAACTCCGTG

>293 len=135 tot-seq=468

TGGGGAATATTGCACAATGGAGGAAACTCTGATGCAGCGACGCCGCGTGAGCGATGAAGG

CTTTCGAGTCGTAAAGCTCTGTCCTATGGGAAGATAATGACGGTACCATAGGAGGAAGCC

CCGGCTAACTACGTG

>294 len=135 tot-seq=467

TCGGGAATATTGCGCAATGGAGGAAACTCTGACGCAGTGACGCCGCGTATGGGAAGAAGG

TTTTCGGATTGTAAACCATTTTAGACAAGGAAGAAACAAGACAGTACTTGTAGAATAAGC

TCCGGCTAACTACGT

>295 len=159 tot-seq=466

TAGGGAATTTTCGGCAATGGGGGAAACCCTGACCGAGCAATGCCGCGTGAGTGAAGACGG

CCTTCGGGTTGTAAAGCTCTGTTGTAAGGGAAGAACGGCATAGAGAGGGAATGCTCTATG

AGTGACGGTACCTTACCAGAAAGCCACGGCTAACTACGT

>296 len=158 tot-seq=461

TGGGGAATATTGCACAATGGGCGCAAGCCTGATGCAGCAACGCCGCGTGAACGATGAAGG

TCTTCGGATCGTAAAGTTCTGTTGCAGGGGAAGATAATGACGGTACCCTGTGAGGAAGCC

CCGGCTAACTACGTGCCAGCAGCCGCGGTAATCAGAAG

>297 len=160 tot-seq=449

TAGGGAATCTTCCACAATGGGCGCAAGCCTGATGGAGCAACACCGCGTGAGTGAAGAAGG

GTTTCGGCTCGTAAAGCTCTGTTGTTAGAGAAGAACGTGCGTGAGAGCAACTGTTCACGC

AGTGACGGTATCTAACCAGAAAGTCACGGCTAACTACGTG

>298 len=158 tot-seq=448

TAGGGAATCTTCCACAATGGACGAAAGTCTGATGGAGCAACGCCGCGTGAGTGATGAAGG

TTTTCGGATCGTAAAACTCTGTTGTAAGGGAAGAACACGTACGAGAGGAAATGCTCGTAC

CTTGACGGTACCTTACGAGAAAGCCACGGCTAACTACG

>299 len=135 tot-seq=447

TCGGGAATTTTGGGCAATGGGCGAAAGCCTGACCCAGCAACGCCGCGTGAAGGATGAAGT

TTTTCGGAATGTAAACTTCGTAAGAATAGGAAGAATAAATGACGGTACTATTTGTAAGGT

CCGGCTAACTACGTG

>300 len=156 tot-seq=446

TGAGGAATATTGGTCAATGGGCGTAAGCCTGAACCAGCCAAGTAGCGTGTAGGAATACAG

CCCTATGGGTTTTAAACTACTTTTGTTAGGGAATAAAGTGTGCCACGTGTTGGCATATTG

CATGTACCTATCGAATAAGGACCGGCTAATTCCGTG

>301 len=160 tot-seq=437

TAGGGAATCTTCCGCAATGGACGAAAGTCTGACGGAGCAACGCCGCGTGAGTGATGAAGG

CTTTCGGGTCGTAAAACTCTGTTGTTAGGGAAGAACAAGTACAAGAGTAACTGCTTGTAC

CTTGACGGTACCTAACCAGAAAGCCACGGCTAACTACGTG

>302 len=159 tot-seq=430

TGGGGAATTTTGGACAATGGGGGCAACCCTGATCCAGCCATGCCGCGTGCAGGATGAAGG

TCTTCGGATTGTAAACTGCTTTTGTCAGGGACGAAAAGGGATGCGATAACACCGCATTCC

GCTGACGGTACCTGAAGAATAAGCACCGGCTAACTACGT

>303 len=155 tot-seq=422

TGGGGAATATTGGACAATGGGCGAAAGCCTGATCCAGCAATTCCGCGTGTGTGAAGAAGG

CCTTCGGGTTGTAAAGCACTTTAGTGAGGGAAGAAACAGCTTGGCCACACCAAGCCTTGA

CGGTACCTCAAGAATAAGCACCGGCTAACTCTGTG

>304 len=156 tot-seq=421

TGAGGAATATTGGTCAATGGGCGTAAGCCTGAACCAGCCAAGTCGCGTGAGGGAAGACGG

TCCTACGGATTGTAAACCTCTTTTGCCGGGGAGCAACGGGGTCCTTGCGAGGGCCTTATG

AGAGTACCCGGAGAAAAAGCATCGGCTAACTCCGTG

>305 len=160 tot-seq=420

TGGGGAATTTTGGACAATGGGCGCAAGCCTGATCCAGCCATGCCGCGTGTCTGAAGAAGG

CCTTCGGGTTGTAAAGGACTTTTGTCGGGGAAGAAAAGGCTGTTGCTAATATCAGCGGCT

AATGACGGTACCCGAAGAATAAGCACCGGCTAACTACGTG

>306 len=155 tot-seq=415

TAGGGAATTTTCGGCAATGGGGGCAACCCTGACCGAGCAACGCCGCGTGAGTGAAGACGG

CCTTCGGGTTGTAAAGCTCTGTTGTAAGGGAAGAACGGTAGATAGAGAATATCTAAGTGA

CGGTACCTTACCAGAAAGCCACGGCTAACTACGTG

>307 len=160 tot-seq=411

TGGGGAATTTTGGACAATGGGGGCAACCCTGATCCAGCAATGCCGCGTGTGTGAAGAAGG

CCTTCGGGTTGTAAAGCACTTTTGTCCGGAAAGAAATCGCACTTACTAATATTAGGTGTG

GATGACGGTACCGGAAGAATAAGGACCGGCTAACTACGTG

>308 len=135 tot-seq=411

TGGGGAATATTGCACAATGGGGGAAACCCTGATGCAGCGACGCCGCGTGAGTGAAGAAGT

GATTCGTCACGTAAAGCTCTATCAGCAGGAAAGAAAATGACGGTACCTGACTAAGAAGCC

CCGGCTAACTACGTG

>309 len=145 tot-seq=410

AAGTCCTGTAGACCGCAGAAACCCAGGATGGCTGCATGGAGAAGAGGAGGAATCACCATG

CCTCTCCCACTTCATTCCCCTAATTAAGGTCAGCTCAGAACCAGAAGGAACTTCTCCCTG

TGGAGGAAAGGTAAAAAAGAGGACC

>310 len=135 tot-seq=408

TGGGGAATATTGCACAATGGGGGAAACCCTGATGCAGCAACGCCGCGTGAAGGAAGACGG

TTTTCGGATTGTAAACTTCTGTTCTTAGTGAAGAATAATGACGGTAGCTAAGGAGCAAGC

CACGGCTAACTACGT

>311 len=157 tot-seq=406

TAGGGAATCTTCCGCAATGGACGAAAGTCTGACGGAGCAACGCCGCGTGAGTGATGACGG

TCTTCGGATTGTAAAGCTCTGTTAATCGGGACGAATGGTTTGTGTGCAAATAGTGCATAG

ACATGACGGTACCGGAATAGAAAGCCACGGCTAACTA

>312 len=155 tot-seq=406

TGAGGAATATTGGTCAATGGACGAGAGTCTGAACCAGCCAAGTAGCGTGAAGGATGACTG

CCCTATGGGTTGTAAACTTCTTTTATATGGGAATAAAACAGGGTATGCATACCCTCTTGT

ATGTACCATATGAATAAGGATCGGCTAACTCCGTG

>313 len=160 tot-seq=405

TGGGGAATCTTCCGCAATGGGCGAAAGCCTGACGGAGCAACGCCGCGTGAGTGATGACGG

CCTTCGGGTTGTAAAACTCTGTGATCCGGGACGAAAAGGCAGAGTGCGAAGAACAAACTG

CATTGACGGTACCGGAAAAGCAAGCCACGGCTAACTACGT

>314 len=135 tot-seq=403

TGGGGAATATTGCACAATGGGGGAAACCCTGATGCAGCGACGCCGCGTGAGCGAAGAAGG

TATTCGTATCGTAAAGCTCTGTCCTATGGGAAGATAATGACGGTACCATAGGAGGAAGCT

CCGGCTAACTACGTG

>315 len=160 tot-seq=398

TAGGGAATTTTCGTCAATGGAGGGAACTCTGAACGAGCAATGCCGCGTGAGTGAAGAAGG

TCTTCGGATCGTAAAGCTCTGTTGTAAGAGAAAAACGGCACATAGAGGAAATGATATGTG

AGTGATGGTATCTTACCAGAAAGCCACGGCTAACTACGTG

>316 len=159 tot-seq=394

TGGGGAATATTGCACAATGGGCGCAAGCCTGATGCAGCCATGCCGCGTGTGTGAAGAAGG

CCTTCGGGTTGTAAAGCACTTTCAGCGAGGAGGAAGGCCAGTGACTTAATAAGTGACTGG

ATTGACGTTACTCGCAGAAGAAGCACCGGCTAACTCCGT

>317 len=131 tot-seq=387

ACACTCAAATTACCCACTCCTGACACGGGAGGTAGTGACAAAAAATAACAATACAGGACT

CTTTCAAGGCCCTGTAATTGGAATGAGTCCACTTTAAATCTTTTAATGAGGATCCATTGG

AGGGAAAGTCT

>318 len=156 tot-seq=384

TGAGGAATATTGGTCAATGGGCGAGAGCCTGAACCAGCCAAGTCGCGTGAAGGAAGACGG

TTCATAAGGATTGTAAACTTCTTTTGTAGAGGGGTAAAGGTGCGTACGTGTACGCATTTG

CAAGTACTCTACGAATAAGTATCGGCTAACTCCGTG

>319 len=135 tot-seq=382

TAGGGAATCTTTCACAATGGGCGAAAGCCTGATGGAGCAACGCCGCGTGCAGGATGAAGG

CCTTCGGGTTGTAAACTGCTTTTATAAGCGAGAAATATGATGGTAACTTATGAATAAGGA

TCGGCTAACTACGTG

>320 len=155 tot-seq=375

TGAGGAATATTGGTCAATGGCCGAGAGGCTGAACCAGCCAAGTCGCGTGAGGGATGACGG

CCCTACGGGTTGTAAACCTCTTTTGTCGGGGAGCAAAGAGAGTCACGTGTGACTCATTGA

GAGTACCCGAAGAAAAAGCATCGGCTAACTCCGTG

>321 len=138 tot-seq=373

TGGGGAATATTGCGCAATGGGGGAAACCCTGACGCAGCAACGCCGCGTGCAGGAAGAAGG

TCTTCGGATTGTAAACTGTTGTCGCAGGGGAAGAAGACAGTGACGGTACCCTGTGAGAAA

GTCACGGCTAACTACGTG

>322 len=160 tot-seq=371

TGGGGAATATTGCACAATGGGCGCAAGCCTGATGCAGCCATGCCGCGTGTGTGAAGAAGG

CCTTCGGGTTGTAAAGCACTTTCAGCGAGGAGGAAGGTGGTGAACTTAATACGTTCATCA

ATTGACGTTACTCGCAGAAGAAGCACCGGCTAACTCCGTG

>323 len=160 tot-seq=371

TAGGGAATCTTCCGCAATGGACGAAAGTCTGACGGAGCAACGCCGCGTGAACGAAGAAGG

CCTTCGGGTCGTAAAGTTCTGTTGTTAGGGAAGAACAAGTACCAGAGTAACTGCTGGTAC

CTTGACGGTACCTAACCAGAAAGCCACGGCTAACTACGTG

>324 len=161 tot-seq=369

TGGGGAATCTTCCGCAATGGGCGAAAGCCTGACGGAGCAATGCCGCGTGAGTGAAGAAGG

CCTTCGGGTTGTAAAACTCTGTCCTTATCGAAGAGAGGTAGGTATGTGAATAATGTACCT

ATAGGACGGTAGATAAGGAGGAAGCCCCGGCTAACTACGTG

>325 len=135 tot-seq=368

TGAGGAATCTTCCACAATGGGCGAAAGCCTGATGGAGCAACGCCGCGTGCAGGATGAAGG

CCTTAGGGTTGTAAACTGCTTTTATAAGTGAAGAATTTGACGGTAACTTATGAATAAGCA

CCGGCTAACTACGTG

>326 len=160 tot-seq=366

TAGGGAATCTTCCACAATGGACGCAAGTCTGATGGAGCAACGCCGCGTGTATGAAGAAGG

CCTTCGGGTTGTAAAGTACTTTCAGCGGGGAGGAAGGGAGTAAAGTTAATACCTTTGCTC

ATTGACGTTACCCGCAGAAGAAGCACCGGCTAACTCCGTG

>327 len=161 tot-seq=365

TGGGGAATCTTCCGCAATGGACGAAAGTCTGACGGAGCAACGCCGCGTGAGTGATGAAGG

ATTTCGGTCTGTAAAGCTCTGTTGTTTATGACGAACGTGCAGTGTGTGAACAATGCATTG

CAATGACGGTAGTAAACGAGGAAGCCACGGCTAACTACGTG

>328 len=142 tot-seq=360

TGGGGAATATTGCACAATGGGCGCAAGCCTGATGCAGCGACGCCGCGTGGGGGATGACGG

CCTTCGGGTTGTAAACTCCTTTCGCAACTGACGAAGCTTTTGTGTGACGGTAGGTTGAGA

AGAAGCACCGGCTAACTACGTG

>329 len=155 tot-seq=359

TGAGGAATATTGGTCAATGGGCGCGAGCCTGAACCAGCCAAGTCGCGTGAGGGAAGACGG

TCCTATGGATTGTAAACCTCTTTTGTCGGGGAGCAAAAGACGCCACGCGTGGCGTTCCGA

GAGTACCCGAAGAAAAAGCATCGGCTAACTCCGTG

>330 len=155 tot-seq=356

TGAGGAATATTGGTCAATGGACGCAAGTCTGAACCAGCCATGCCGCGTGCAGGAAGACGG

CTCTATGAGTTGTAAACTGCTTTTGTACTAGGGTAAACGCTCCTACGTGTAGGAGTCTGA

AAGTATAGTACGAATAAGGATCGGCTAACTCCGTG

>331 len=160 tot-seq=354

TGGGGAATTTTGGACAATGGGCGAAAGCCTGATCCAGCCATGCCGCGTGCAGGATGAAGG

CCTTCGGGTTGTAAACTGCTTTTGTACGGAACGAAAAGGCTCTTTCTAATAAAGAGGGCT

CATGACGGTACCGTAAGAATAAGCACCGGCTAACTACGTG

>332 len=135 tot-seq=353

TCGGGAATATTGCGCAATGGAGGAAACTCTGACGCAGTGACGCCGCGTATAGGAAGAAGG

TTTTCGGATTGTAAACTATTGTCGTTAGGGAAGAAAAAGACAGTACCTAAGGAGGAAGCT

CCGGCTAACTATGTG

>333 len=200 tot-seq=346

TAGGGAATTTTTCACAATGGGCGCAAGCCTTATGAAGCAATGCCGCGTGAACGATGAAGG

TCTTATAGATTGTAAAGTTCTTTTATATGGGAAGAAACGCTAAGATAGGAAATGATTTTA

GTTTGACTGTACCATTTGAATAAGTATCGGCTAACTATGTGCCAGCAGCCGCGGTAATAA

GAAGATCGGAAGAGCGTCGT

>334 len=210 tot-seq=346

TGAGGAATATTGGTCAATGGGCGCGAGCCTGAACCAGCCAAGTAGCGTGCAGGACGACGG

CCCTATGGGTTGTAAACTGCTTTTATACGGGGATAAAGTATGCCACGTGTGGTTTATTGC

AGGTACCGTATGAATAAGGACCGGCTAATTCCGTGGGGGGAGCCGGGTTCAGAGATCGGA

AGATCGTCGTGTAGGGAAAGAGTGTGCCAA

>335 len=159 tot-seq=340

TAGGGAATCTTCCACAATGGGTGCAAACCTGATGGAGCAATGCCGCGTGAGTGAAGAAGG

TCTTCGGATCGTAAAGCTCTGTTGTTAGAGAAGAACACGTGCTAGGTAACTACTAGCGCC

TTGACGGTATCTAACCAGAAAGTCACGGCTAACTACGTG

>336 len=134 tot-seq=335

TTGGGGAATATTGCACAATGGGGGAAACCCTGATGCAGCGACGCCGCGTGGGTGACGAAG

TATTTCGGTATGTAAAGCCCTATCAGCAGGGAAGAAGATGACAGTACCTGACTAAGAAGC

CCCGGCTAACTACG

>337 len=135 tot-seq=333

TGGGGAATATTGCACAATGGGGGGAACCCTGATGCAGCGACGCCGCGTGAAGGAAGAAGT

ATCTCGGTATGTAAACTTCTATCAGCAGGGAAGATAGTGACGGTACCTGACTAAGAAGCC

CCGGCTAACTACGTG

>338 len=215 tot-seq=329

TGGGGAATATTGCACAATGGGGGAAACCCTGACGCAGCGACGCCGCGTGCGGGATGAAGG

CCTTCGGGTTGTAAACCGCTTTTGATTGGGAGCAAGCTTTCGGGTGAGTGTACCTTTCGA

ATAAGCGCCGGCTAACTACGTGGGGGGAGCCGCGGTAATAAAGAGATCGGAAGGCGTCGT

GTAGGGAAAGAGTGTGCCAATGTGTAGATCTCGGT

>339 len=155 tot-seq=327

TGAGGAATATTGGTCAATGGGCGAGAGCCTGAACCAGCCAAGTCGCGTGAAGGATGAAGG

ATCTATGGTTCGTAAACTTCTTTTATATGGGAATAAAGTGCAGGACGTGTCCTGTTTTGT

ATGTACCGTATGAATAAGGATCGGCTAACTCCGTG

>340 len=135 tot-seq=326

TGGGGAATATTGCACAATGGGGGGAACCCTGATGCAGCGACGCCGCGTGAGCGAAGAAGG

TTTTCGAATCGTAAAGCTCTGTCCTATGAGAAGATAATGACGGTATCATAGGAGGAAGCC

CCGGCTAAATACGTG

>341 len=135 tot-seq=326

TGAGGAATATTGGGCAATGGAGGCAACTCTGACCCAGCCATGCCGCGTGAGTGAAGAAGG

TTTTCGGATTGTAAAGCTCTTTCGGATGTGACGATGATGACGGTAGCATCTAAAGAAGCC

CCGGCTAACTTCGTG

>342 len=154 tot-seq=322

TGAGGAATATTGGTCAATGGGCGGGAGCCTGAACCAGCCAAGCCGCGTGAGGGAATAAGG

CCCTATGGGTCGTAAACCTCTTTTGTCGGGGAACAAAAGCGGGGACGCGTCCCCGTCTGC

GTGTACCCGAAGAAAAAGCATCGGCTAACTCCGT

>343 len=135 tot-seq=320

TGGGGAATATTGCACAATGGGGGAAACCCTGATGCAGCGACGCCGCGTGAAGGACGACGT

ATTTCGGTATGTAAACTTCTATCAGCAAGGAAGATGATGACGGTACTTGACTAAGAAGCC

CCGGCTAACTACGTG

>344 len=135 tot-seq=316

TGGGGAATATTGGACAATGGGCGAAAGCCTGATCCAGCAATGCCGCGTGAGTGAAGAAGG

CCTTCGGGTTGTAAAGCTCTTTTACCCGGGAAGATAATGACTGTACCGGGAGAATAAGCC

CCGGCTAACTCCGTG

>345 len=160 tot-seq=316

TGGGGAATTTTGGACAATGGGGGCAACCCTGATCCAGCCATGCCGCGTGCAGGATGAAGG

TCTTCGGATTGTAAACTGCTTTTGTCAGGGAAGAAACGCAACGGGTCAACAACCCGTTGT

AATGACGGTACCTGAAGAATAAGCACCGGCTAACTACGTG

>346 len=212 tot-seq=313

TCCAGGTGGTGCAGGGACGACGGACCCCATCTTCCCCATCAGCAACATGCCGCTGCAGAG

GCCTCCACCCCCCCCGGGTGAGGAGGCTCAGGAAGGCCACGGCAGCCTGGCCGTGCATCC

CCAGACTCTCGTCCGCCCCCCTTTGTCTGCCTGGCGTTCTGGGTGGGCATGCGCAGCGAG

GCTGGCCCACCGCCAGCAGTTCTTCCTGCAGG

>347 len=160 tot-seq=311

TGGGGAATTTTGGACAATGGGCGCAAGCCTGATCCAGCCATGCCGCGTGCGGGAAGAAGG

CCTTCGGGTTGTAAACCGCTTTTGTCAGGGAAGAAAAGGTTCTGGCTAATACCTGGGACT

CATGACGGTACCTGAAGAATAAGCACCGGCTAACTACGTG

>348 len=136 tot-seq=310

TAGGGAATTTTGCGCAATGGGCGAAAGCCTGACGCAGCAACGCCGCGTGATTGATAAAGC

CCTTCGGGGTGTAAAGATCTGTCAGTGGGGACGAAACTTGACGGTACCCACAGAGGAAGC

ACCGGCTAACTCCGTG

>349 len=160 tot-seq=306

TGGGGAATTTTGGACAATGGGGGCAACCCTGATCCAGCCATGCCGCGTGAGTGAAGAAGG

CCTTCGGGTTGTAAAGCTCTTTCGGCCGGGAAGAAATCGCATAGGCTAATACCCTGTGTG

GATGACGGTACCGGAATAAGAAGCACCGGCTAACTACGTG

>350 len=161 tot-seq=306

TTAGGGAATCTTCCACAATGGACGCAAGTCTGATGGAGCAACGCCGCGTGAGTGAAGAAG

GTTTTCGGATCGTAAAGCTCTGTTGTTGGAGAAGAACGTGCGTGAGAGTAACTGTTCACG

CAGTGACGGTATCCAACCAGAAAGTCACGGCTAACTACGTG

>351 len=160 tot-seq=304

TAGGGAATTTTCGTCAATGGGGGGAACCCTGAACGAGCAATGCCGCGTGAGTGAAGAAGG

CCTTCGGGTCGTAAAGCTCTGTTGTAAGTGAAGAACGGTACTGACAGGGAATGGTCAGTA

AGTGACGGTAGCTTACCAGAAAGCCACGGCTAACTACGTG

>352 len=153 tot-seq=303

TGAGGAATATTGGTCAATGGACGAGAGTCTGAACCAGCCAAGTAGCGTGAAGGATGACTG

CCCTATGGGTTGTAAACTTCTTTTATATGGGAATAAAGTGAGCCACGTGTGGCTTTTTGT

ATGTACCATACGAATAAGGATCGGCTAACTCCG

>353 len=159 tot-seq=301

TAGGGAATCTTCCGCAATGGGCGAAAGCCTGACGGAGCAACGCCGCGTGAGTGAAGAAGG

ATTTCGGTTCGTAAAGCTCTGTTGTTAGGGAAGAATGGATATGTAGTAACTATACATATA

AGAGACGGTACCTAACCAGAAAGCCACGGCTAACTACGT

>354 len=154 tot-seq=300

TGAGGAATATTGGTCAATGGCCGCAGGGCTGAACCAGCCAAGTCGCGTGAGGGATGACGG

TCCTATGGATTGTAAACCTCTTTTGTCAGGGAGCAAAGGGCGCCACGTGTGGCGTTTTGC

GAGTACCTGAAGAAAAAGCATCGGCTAACTCCGT

>355 len=160 tot-seq=298

TGGGGAATATTGGACAATGGGGGCAACCCTGATCCAGCCATGCCGCGTGTGTGAAGAAGG

CCTTTTGGTTGTAAAGCACTTTAAGCAGGGAGGAGAGGCTAATGGTTAATACCCATTAGA

TTAGACGTTACCTGCAGAATAAGCACCGGCTAACTCTGTG

>356 len=199 tot-seq=297

GGATGGGGAAGCTATAGCCCTTGGGACTTGTTAGAAAAAAGGGAAGCAGGATCTAAGAGA

AAGGAGAAATTATTTGGTAGGAAAGGACAGAGACATTTCTGAGGTGGATAAAACAAGTCT

TAATGCTTTGCAACTCTTTCCCCCCTCCTGGGAACCTGTGACCTGTACCATCCTGCAAGC

TGTTGTGTTGCCTAGGTGA

>357 len=153 tot-seq=296

TGAGGAATATTGGTCAATGGACGAGAGTCTGAACCAGCCAAGTAGCGTGAAGGATGAAGG

TCCTACGGATTGTAAACTTCTTTTATAAGGGAATAAACCCTCCCACGTGTGGGAGCTTGT

ATGTACCTTATGAATAAGCATCGGCTAACTCCG

>358 len=135 tot-seq=294

TGGGGGATATTGCACAATGGGGGAAACCCTGATGCAGCAACGCCGCGTGAGGGAAGAAGG

TTTTCGGATTGTAAACCTCTGTTCTTAGTGACGATAATGACGGTAGCTAAGGAGAAAGCT

CCGGCTAACTACGTG

>359 len=135 tot-seq=294

TGGGGAATATTGCGCAATGGGGGCAACCCTGACGCAGCAACGCCGCGTGCGGGACGACGG

CCTTCGGGTTGTAAACCGCTTTCAGCAGGGAAGATAGTGACGGTACCTGCACAAGAAGCC

CCGGCTAACTACGTG

>360 len=140 tot-seq=294

TGGGGAATATTGCACAATGGGCGAAAGCCTGATGCAGCGACGCCGCGTGAGGGATGACGG

CCTTCGGGTTGTAAACCTCTTTCAGCACGGAAGAAGCGAAAGTGACGGTACGTGCAGAAG

AAGCGCCGGCTAACTACGTG

>361 len=205 tot-seq=291

CCAAGGCTCTGCAGGGTAACCCAGAGCCCTTGGTCTGCAACGTCCGGGGAACTGTGTGTT

CCTGGGCGTTCATGTGCCTGTGCACTCTTTCTCACGTGTATCCGTCTTTTTGTTCCCCTC

TCATGTTTGTTAGGGCATTGTTGATTGTTTATATCCTATGTATGGTGCAGTGGCTGAAGA

GTGGCCTCTCAAAAGATATGTCCAC

>362 len=155 tot-seq=291

TGAGGAATATTGGTCAATGGGCGGAAGCCTGAACCAGCCAAGTCGCGTGAGGGAAGACGG

CCCTACGGGTTGTAAACCTCTTTTGCCGGGGAGCAAAGGCGGGTATGTATACCCGGATGA

GAGTACCCGGAGAAAAAGCATCGGCTAACTCCGTG

>363 len=161 tot-seq=291

TAGGGAATCTTCCGCAATGGGCGAAAGCCTGACGGAGCAACGCCGCGTGAGTGAAGAAGG

TTTTCGGATCGTAAAGCTCTGTTGTAAGAGAAGAACGAGTGTGAGAGTGGAAAGTTCACA

CTGTGACGGTATCTTACCAGAAAGGGACGGCTAACTACGTG

>364 len=135 tot-seq=288

TGGGGAATATTGCACAATGGGGGAAACCCTGATGCAGCGACGCCGCGTGAGCGAAGAAGG

CCTTCGGGTCGTAAAGCTCTTTTATGAGGGAAGATAATGACGGTACCTCAAGAAAAAGCC

CCGGCTAACTACGTG

>365 len=135 tot-seq=288

TGGGGAATATTGCACAATGGGGGAAACCCTGATGCAGCGACGCCGCGTGGAGGAAGAAGG

TCTTCGGATTGTAAACTCCTGTCCCAGGGGACGATAATGACGGTACCCTGGGAGGAAGCA

CCGGCTAACTACGTG

>366 len=152 tot-seq=288

TGGGGAATATTGCACAATGGGCGAAAGCCTGATGCAGCGACGCCGCGTGAGGGATGGAGG

CCTTCGGGTTGTAAACCTCTTTCGCTCATGGTCAAGCCGCAACTGTGGTTGTGGTGAGGG

TAGTGGGTAAAGAAGCGCCGGCTAACTACGTG

>367 len=155 tot-seq=287

TGAGGAATATTGGTCAATGGGCGAGAGCCTGAACCAGCCAAGTAGCGTGCAGGATGACGG

CCCTATGGGTTGTAAACTGCTTTTATGCGGGGATAAAGTGAGGGACGTGTCCTTCATTGC

AGGTACCGCATGAATAAGGACCGGCTAATTCCGTG

>368 len=135 tot-seq=284

GCGCGCAAATTACCCACTCCCGACCCGGGGAGGTAGTGACGAAAAATAACAATACAGGAC

TCTTTCGAGGCCCTGTAATTGGAATGAGTCCACTTTAAATCCTTCCGCGAGGATCCATTG

GAGGGCAAGTCTGGT

>369 len=159 tot-seq=284

TAGGGAATCTTCGGCAATGGACGGAAGTCTGACCGAGCAACGCCGCGTGAGTGAAGAAGG

TTTTCGGATCGTAAAACCCTGTTGTTAGAGAAGAAAGTGCGTGAGAGTAACTGTTCACGT

TTCGACGGTATCTAACCAGAAAGCCACGGCTAACTACGT

>370 len=135 tot-seq=282

TGGGGGATATTGCACAATGGGGGCAACCCTGATGCAGCGACGCCGCGTGAGCGATGGAGT

ACTCCGGTATGTAAAGCTCTGTCAGCAGGGAAGAGAATGACGGTACCTGACCAAGAAGCC

CCGGCTAAATACGTG

>371 len=131 tot-seq=282

CCCCCGCCTGTGGCAATCTCTGTCTCCCTCTGGTGGTTTAAGCTCTCAAAATTACAGGTG

TTCTTTGTAAGTGACTCATACATTCCAGTACCGGGGTGAGTTTTTACTCTTTTGGCTCAT

GTCATTTCCTC

>372 len=201 tot-seq=281

TAGGGAATCTTCCACAATGGGCGAAAGCCTGATGGAGCAACGCCGCGTGGGTGAAGAAGG

TCTTCGGATCGTAAAACCCTGTTGTTAGAGAAGAAAGTGCGTGAGAGTAACTGTTCACGT

TTCGACGGTATCTAACCAGAAAGCCACGGCTAACTACGTGCCAGCAGCCGCGGTAATACA

AAGATCGGAAGAGCGTCGTGT

>373 len=211 tot-seq=279

GGGTGTGGGGGGGATTTAGTGATCCACTTCTAGCAAACAGAATAAAGTGGAAATGATGGC

ATGTGACTCCCAAGACTAGGTCCAAAGCTTTGACTTTGCTGTCTGTCTTGGATCACGCAC

TCTGGGGAAAGAGTTGCCATGTCATGAGGATGCTCAAGCAAGCCCAAGGAGAGGGTCCCG

TCGACAGAAAACAAAGCCCTGAGGCCTCTTG

>374 len=125 tot-seq=275

AACCTCAAAGGAAAAGTGCCAGTGTGTGTAGACCTGGCCGGGTTCCAAGCCGTTCTGCCC

CCACTGCCTGTGTGTCACCTGGAGCAAGCCATGGTGCTTCTCCAAGAACGGGGTGATCAG

GTGTG

>375 len=213 tot-seq=273

GTGAGTCAAGAAGTATTTCAAAGGCAGGTCACAATTGCTGTTTGTCATCTTGCAATGTGA

AGGTCAGGAGACAGAAGAAGGTGGGAGGAAGCAGAGAAAAGACCTGCTTGCAAGCAGATC

AACCATAGCCAGACAGGTCCTGCTCCATGGCTCTGACAGTGCCCAGGGAGCCCATGTAAT

TACTGCCTGAACTGACATCTGAGGCTTTCACCT

>376 len=156 tot-seq=272

TGAGGAATATTGGTCAATGGGCGAGAGCCTGAACCAGCCACGTCGCGTGAAGGAAGACTG

ACCTTACGGTTTGTAAACTTCTTTTGTAGAGGGGTAATAGCAGATACGTGTATTTGCGTG

AAAGTACTCTACGAATAAGCATCGGCTAACTCCGTG

>377 len=154 tot-seq=271

TGAGGAATATTGGTCAATGGGCGAGAGCCTGAACCAGCCAAGTCGCGTGAGGGAGTACTG

CCCTATGGGTTGTAAACCTCTTTTGTCGGGGAGCAAAAGCCGGACGTGTCCGTCTGTGAG

AGTACCCGAAGAAAAAGCATCGGCTAACTCCGTG

>378 len=155 tot-seq=270

TTCATATTTCAGTTCAATTGTTTCAGCCTTATCTAGCCAGTGTGGAATCTGCACCACACA

TGAAGTTCAAAGTCCAGCTTAGATTTAGGTAGATTTTATACATGGAATTTGGGGCTCCAC

TCTGTGGCTCACTCCTTTCAGAGACCCCACTCCTT

>379 len=136 tot-seq=270

GCATGCAAATTACCCACTCCCTACTCGGGGAGGTAGTGATGAAAAATAACAATACAGGAC

TCTTTCGAGGCCCTGTAATTGGAATGAGTCCACTTTAAATCCTTTAACGAGGATCCATTG

GAGGGCAAGTCTGGTG

>380 len=154 tot-seq=269

TGAGGAATATTGGTCAATGGCCGAGAGGCTGAACCAGCCAAGTCGCGTGAAGGAAGAAGG

ATCTATGGTTTGTAAACTTCTTTTATAGGGGAATAAAGTGGAGGACGTGTCCTTTTTTGT

ATGTACCCTATGAATAAGCATCGGCTAACTCCGT

>381 len=136 tot-seq=267

ACATGCAAATTACCCACTCCTGACACGGGGAGGTAGTGACGAAAAATAACAATACAGGAC

TCTTTCGAGGCCCTGTAATTGGAATGAGTCCACTTTAAATCTTTTAATGAGGATCCATTG

GAGGGCAAGTCTGGTG

>382 len=181 tot-seq=267

TGGGGGATATTGGACAATGGGGGAAACCCTGATCCAGCGATGCCGCGTGAGGGAAGAAGG

TTTTCGGATTGTAAACCTCTGTGGACAGGGACGATAATGACGGTACCTGTTTAGAAAGCC

ACGGCTAACTACGTGGGGGGAGCCGCGGAATACAGAGATCGGAAGAGGGGGGTGTAGGGA

A

>383 len=155 tot-seq=266

TGAGGAATATTGGTCAATGGGCGGGAGCCTGAACCAGCCAAGTAGCGTGAAGGATGACAG

CCCTATGGGTTTTAAACTTCTTTTATAAGGGAATAAAGTGCCCCACGTGTGGGGTTTTGT

ATGTACCTTATGAATAAGCATCGGCTAATTCCGTG

>384 len=190 tot-seq=264

TGGGGAATTTTGCACAATGGGCGCAAGCCTGATGCAGCGACGCCGCGTGAGGGATGAAGG

CCTTCGGGTTGTAAACCTTTTTCGCTCATGGTCAAGCCGCAGCTTTGGGTTGTGGTGAGG

GTAGTGGGTAAAGAAGCGCCGGCTAACTACGTGGGGGGCAGCGGGGAATCCCGAGATCGG

AAGAGCGTCG

>385 len=156 tot-seq=263

TGAGGAATATTGGTCAATGGGCGAGAGCCTGAACCAGCCAAGTCGCGTGAAGGAAGACGG

TTCATAAGGATTGTAAACTTCTTTTGCAGAGGAGTAACGATAGGCACGTGTGCCTGTGTG

AACGTACTCTGCGAATAAGTATCGGCTAACTCCGTG

>386 len=142 tot-seq=260

TAGGGAATTTTCGGCAATGGGGGGAACCCTGACCGAGCAACGCCGCGTGAACGAAGAAGT

AATTCGTTATGTAAAGTTCTTTTATTGGGGAAGAAAGCGCAAGTTGACGGTACCCAAGGA
ATAAGCTCCGGCTAACTACGTG
>387 len=136 tot-seq=259
TGGGGGATATTGGACAATGGGGGGAACCCTGATCCAGCGACGCCGCGTGAGTGAAGAAGT
ATCTCGGTATGTAAAGCTCTATCAGCAGGGAAGAAAGAAATGACGGTACCTGAGTAAGAA
GCCCCGGCTAACTACG
>388 len=177 tot-seq=259
GTAGGAACAGACAATGCGAAGGCCCTCACTGGGCTCACATTTCACCCCTTGACATCCGTC
TTTGCCCTAGCTTTACCCCTGTGATGTGGGGACATGGTATGGCAATCTGGAGGCCATGAG
GATTAAACCCTTAGTGATTGACAGAAGGTGGCAGGCTCCCAGGTTTTCTTTTGCACT
>389 len=205 tot-seq=259
TGGGGAATATTGGACAATGGGGGGAACCCTGATCCAGCCATGCCGCGTGTGTGAAGAAGG
CCTTATGGTTGTAAAGCACTTTAAGCGAGGAGGAGGCTACTGAGACTAATACTCTTGGAT
AGTGGACGTTACTCGCAGAATAAGCACCGGCTAACTCTGTGGGGGGAGCCGCGGAATTCA
CAGATCGGAAGAGCGTCGTGTATGG
>390 len=135 tot-seq=258
TGGGGAATATTGCACAATGGGGGAAACCCTGATGCAGCAACGCCGCGTGAGTGATGAAGG
TCTTCGGATCGTAAAGCTCTGTCTTCAGGGACGATAATGACGGTACCTGAGGAGGAAGCC
ACGGCTAACTACGTG
>391 len=138 tot-seq=252
TGGGGAATATTGGGCAATGGGCGCAAGCCTGACCCAGCAACGCCGCGTGAAGGAAGAAGG
CCTTCGGGTTGTAAACTTCTTTTCTTAGGGACGAAGAAAGTGACGGTACCTAAGGAATAA
GCCACGGCTAACTACGTG
>392 len=135 tot-seq=250
TGGGGAATATTGGACAATGGGCGAAAGCCTGATCCAGCCATGCCGCGTGAGTGATGAAGG
CCCTAGGGTTGTAAAGCTCTTTCAACGGTGAAGATAATGACGGTAACCGTAGAAGAAGCC
CCGGCTAACTTCGTG
>393 len=161 tot-seq=250
TGGGGAATATTGCGCAATGGGGGAAACCCTGACGCAGCGACGCCGCGTGAGTGAAGAAGG

TCTTCGGATTGTAAAGCTCTGTTATACGGGACGAAAAAGACGGATGCCAACAGTATCCGT

CCGTGACGGTACCGTAAGAGAAAGCCACGGCTAACTACGTG

>394 len=197 tot-seq=248

TGGGGAATATTGCACAATGGGCGCAAGCCTGATGCAGCGACGCCGCGTGAGGGATGGAGG

CCTTCGGGTTGTAAACCGCTTTTGATTGGGAGCAAGCTTTCGGGTGAGTGTACCTTTCGA

ATAAGCGCCGGCTAACTACGTGGGGGGCCCGCGGTAATGATAAGATCGGAAGAGCGTCGT

GTAGGGAAAGAGTGGGC

>395 len=155 tot-seq=248

TGAGGAATATTGGTCAATGGGCGGAAGCCTGAACCAGCCAAGTCGCGTGAGGGAAGAAGG

CCCTGCGGGTCGCAGACCTCTTTGGACGGGGAGCAAGGCCGCCGACGTGTCGGCGGAAGG

AGAGTACCCGTAGAACAAGCATCGGCTAACTCCGT

>396 len=160 tot-seq=245

TAGGGAATCTTCCGCAATGGACGAAAGTCTGACGGAGCAACGCCGCGTGAGTGATGAAGG

TTTTCGGATCGTAAAACTCTGTTGTCAGGGAAGAACAAGTGCCGGAGTAACTGCCGGCAC

CTTGACGGTACCTGACCAGAAAGCCACGGCTAACTACGTG

>397 len=135 tot-seq=243

TGGGGAATATTGCACAATGGAGGAAACTCTGATGCAGCGATGCCGCGTGAGGGAAGAAGG

TTTTCGGATTGTAAACCTCTGTCTTAAGGGACGATAATGACGGTACCTTAGGAGGAAGCT

CCGGCTAACTACGTG

>398 len=159 tot-seq=243

TGGGGAATCTTCCGCAATGGGCGAAAGCCTGACGGAGCAACGCCGCGTGATCGAATGAAG

GCCTTCGGGTTGTAAAGATCTGTTGACAGGGACGAATAAGCAATGCGAATAGTTTTGTGT

ATGACGGTACCTGTTTAGAAAGCTCCGGCTAACTACGTG

>399 len=133 tot-seq=242

TGGGGGATATTGGACAATGGGGGCAACCCTGATCCAGCGACGCCGCGTGAGGGAAGAAGG

TTTTCGGATTGTAAACCTCTGTTGACGGAGAAAAAAATGATGGTATCCGTTTAGAAAGCC

ACGGCTAACTACG

>400 len=240 tot-seq=238

CAGGAAATGTTTTCAGAATTCAGAAGCCAGAGCCATCAGGTGGGAGTTAGAACCAAAAAG

TGAGGGTTGCTCAGTGATAGATGGGACCACAGCCAGAAGAACCACTTGGGAGGACCTGGG

TCATGAAGGAGACCAAGCTGCTGACCTGCCAGAAAAGTTGTCGAAGTAGGAAAAGAGGGG

GCTCCTGGCTCTTCGTTTCCTTTCCAATCTTTTTCTACTAGTTGCTCCCCACTCGGAACC

>401 len=140 tot-seq=238

TGGGGAATATTGCACAATGGGCGGAAGCCTGATGCAGCGACGCCGCGTGGGGGATGAAGG

CCTTCGGGTTGTAAACTCCTTTCGACAGGGACGAAGCGTAAGTGACGGTACCTGTATAAG

AAGCACCGGCTAACTACGTG

>402 len=155 tot-seq=237

TGAGGAATATTGGTCAATGGGCGAGAGCCTGAACCAGCCAAGTAGCGTGCAGGATGACGG

CCCTATGGGTTGTAAACTGCTTTTGTATGGGGATAAAGTCAATCACGTGTGATTGTTTGC

AGGTACCATACGAATAAGGACCGGCTAATTCCGTG

>403 len=196 tot-seq=237

CCGGGAAAGCCAGGGTGCCATCAAGCCAGCAGGGGAAGCAGCTCCAGCTGCAGTGAGACC

CTCTTACAGGTGGGGAGGGGCTCCCCACGAGGGCCAGGGGTGACTGCAGCCCCCTGCCCT

GTTCCCGGAGGCAGCGGGGAGGGGGATGGGCAGCCCCTCGGTTTCTTGGAGCCCCCACCT

GGATTCGACTCCCCTC

>404 len=135 tot-seq=237

TGGGGAATATTGCACAATGGAGGAAACTCTGATGCAGCGATGCCGCGTGAGGGAAGAAGG

TTTTAGGATTGTAAACCTCTGTCTTCAGGGACGATAAAAACGGTACCTGAGGAGGAAGCT

CCGGCTAACTACGTG

>405 len=160 tot-seq=234

TAGGGAATCTTCCACAATGGACGAAAGTCTGATGGAGCAACGCCGCGTGAGCGATGAAGG

CCTTCGGGTCGTAAAGCTCTGTTGTTAGGGAAGAACAAGTGCGAGAGTAACTGCTCGCAC

CTTGACGGTACCTAACCAGAAAGCCACGGCTAACTACGTG

>406 len=160 tot-seq=233

TGGGGAATCTTCCGCAATGGGCGAAAGCCTGACGGAGCAATGCCGCGTGAGTGAAGAAGG

CCTTCGGGTTGTAAAACTCTGTCCTTATCCAAGAGAGCAAGATGTAGTAACTGACATTTT

GAGGACGGTAGATAAGGAGGAAGCCCCGGCTAACTATGTG

>407 len=155 tot-seq=230

TGAGGAATATTGGTCAATGGGCGAGAGCCTGAACCAGCCAAGTCGCGTGAAGGATGACTG

CCCTATGGGTTGTAAACTTCTTTTATGTGGGAATAAAAAGGGAACGTGTTCCCTATTGC

ATGTACCACTATGAATAAGCATCGGCTAACTCCGT

>408 len=138 tot-seq=228

TGGGGAATATTGGGCAATGGGCGAAAGCCTGACCCAGCAACGCCGCGTGAAGGAAGAAGG

TCTTCGGATTGTAAACTTCTTTTATCAGGGACGAAGTAAGTGACGGTACCTGATGAATAA

GCCACGGCTAACTACGTG

>409 len=159 tot-seq=227

TGGGGAATATTGCACAATGGGCGCAAGCCTGATGCAGCCATGCCGCGTGTATGAAGAAGG

CCTTCGGGTTGTAAAGTACTTTCAGTCGGGAGGAAGGTGGTAAGGTTAATAACCTTATCA

ATTGACGTTACCGACAGAAGAAGCACCGGCTAACTCCGT

>410 len=145 tot-seq=226

CAGTAGTCTCTCAGTCACCAAAACCCCAAAACAAGAGGCAATTTGAAAGAGTGGAGGAAA

CTTTTAGCAACCAATGTTCCCAAAACACATAGATCTTTGACAGAAAGGTAGGTTTGCTAC

CAAAGTAAAACGCTAGTTAGTAAGA

>411 len=135 tot-seq=226

TGAGGGATATTGGGCAATGGGGGCAACCCTGACCCAGCGACGCCGCGTGAGGGAAGACGG

TCTTCGGATTGTAAACCTCTGTCTTTGGGGACGAAAAAGGACGGTACCCAAGGAGGAAGC

TCCGGCTAACTACGT

>412 len=135 tot-seq=226

GCGCGCAAATTACCCAATCCTGACACAGGGAGGTAGTGACAATAAATAACGATACAGGGC

CCATTCGGGTCTTGTAATTGGAATGAGTACAATGTAAATACCTTAACGAGGAACAATTGG

AGGGCAAGTCTGGTG

>413 len=153 tot-seq=225

ACAATTTAAAGTAGAATTACCTCCCCCCCCAAACAAAACAACTATTGTAAGTTTGTAGAA

ATACTGCTTTTGTAAAATAAATGTTTTGACAGTCAGTCTAACGATTTCAATCATGAATGA

AGCACAATCACTCAGGAATCTGCCATTCACTCC

>414 len=161 tot-seq=225

TGGGGAATATTGGACAATGGGGGGAACCCTGATCCAGCCATGCCGCGTGTGTGAAGAAGG

CCTTTTGGTTGTAAAGCACTTTAAGTGGGGAGGAGGCTCTCTTGGTTAATACCTAAGAAG

AGTGGACGTTACCCACAGAATAAGCACCGGCTAACTCTGTG

>415 len=156 tot-seq=224

TGAGGAATATTGGTCAATGGCCGGAAGGCTGAACCAGCCAAGTCGCGTGAGGGAATAAGG

CCCTACGGGTCGTAAACCTCTTTTGCCAGGGAGCAATGCCGTCCACGTGTGGACGGAAGG

AGAGTACCTGGAGAAAAAGCATCGGCTAACTCCGTG

>416 len=161 tot-seq=223

TAGGGAATCTTCCGCAATGGACGAAAGTCTGACGGAGCAACGCCGCGTGAGCGAAGAAGG

TCTTCGGATCGTAAAGCTCTGTTGTTAGGGAAGAACAAGTACCGTTCAAATAGGGCGGTA

CCTTGACGGTACCTAACCAGAAAGCCACGGCTAACTACGTG

>417 len=138 tot-seq=222

TGGGGAATATTGCACAATGGGGGAAACCCTGATGCAGCAACGCCGCGTGAAGGATGACGG

TTTTCGGATTGTAAACTTCTTTTCTTAGTGACGAAGACAGTGACGGTAGCTAAGGAATAA

GCATCGGCTAACTACGTG

>418 len=155 tot-seq=222

TGAGGAATATTGGTCAATGGTCGGGAGACTGAACCAGCCAAGCCGCGTGAGGGAAGAAGG

TACAGCGTATCGTAAACCTCTTTTGCCGGGGAACAAGAGCTTCCACGAGTGGGAGTGTGA

GCGTACCCGGAGAAAAAGCATCGGCTAACTCCGTG

>419 len=205 tot-seq=221

TGGGGAATATTGGACAATGGGCGGAAGCCTGATCCAGCCATGCCGCGTGTGTGAAGAAGG

CCTTATGGTTGTAAAGCACTTTAAGCGAGGAGGAGGCTACTTTAGTTAATACCTAGAGAT

AGTGGACGTTACTCGCAGAATAAGCACCGGCTAACTCTGTGCCAGCACCGCGGTAATAAG

AAGATCGGAAGAGCGTCGTGTATGG

>420 len=160 tot-seq=221

TGGGGAATATTGGACAATGGGCGAAAGCCTGATCCAGCCATGCCGCGTGTGTGAAGAAGG

TCTTCGGATTGTAAAGCACTTTAAGTTGGGAGGAAGGGCTGCTGGTTAATACCCTGCAGT

TTTGACGTTACCAACAGAATAAGCACCGGCTAACTTCGTG

>421 len=159 tot-seq=221

TGGGGAATTTTGGACAATGGGCGCAAGCCTGATCCAGCCATGCCGCGTGTCTGAAGAAGG

CCTTCGGGTTGTAAAGGACTTTTGTCAGGGAAGAAAAGGGGCATGTTAATACCGTGCTCT

GATGACGGTACCTGAAGAATAAGCACCGGCTAACTACGT

>422 len=155 tot-seq=220

TGAGGAATATTGGTCAATGGGCGATAGCCTGAACCAGCCAAGTCGCGTGAAGGAAGACGG

CCTTACAGGTTGTAAACTTCTTTTATCGGAGAGTAAAGTGCACTACGTGTAGTGTATTGC

AAGTATCCGAAGAAAAAGCATCGGCTAACTCCGTG

>423 len=161 tot-seq=220

TAGGGAATCTTCCGCAATGGACGAAAGTCTGACGGAGCAACGCCGCGTGAGTGATGAAGG

CTTTCGGGTCGTAAAACTCTGTTGTTAGGGAAGAACAAGTGCTAGTTGAATAAGCTGGCA

CCTTGACGGTACCTAACCAGAAAGCCACGGCTAACTACGTG

>424 len=138 tot-seq=218

TGGGGAATATTGGGCAATGGAGGCAACTCTGACCCAGCAACGCCGCGTGAGTGATGAAGG

TCTTCGGATTGTAAAACTCTTTAAGTGGGGACGAAGAAAGTGACTGTACCCACAGAATAA

GCCTCGGCTAACTACGTG

>425 len=137 tot-seq=218

TGGGGAATTTTCTGCAATGGAAGAAAACCTGACGGAGCAATGCCGCATGAAGGCAAAAGG

CCCACGGGTCATGAACTTCTTTTCTCAGAGAAGAAAAAATGACGGTATCTAAGGAATAAG

CATCGGCTAACTCTGTG

>426 len=160 tot-seq=218

TGGGGAATATTGCACAATGGGGGAAACCCTGATGCAGCCATGCCGCGTGTGTGAAGAAGG

CCTTCGGGTTGTAAAGCACTTTCAGCGAGGAGGAAAGGTCAGTAGCTAATATCTGCTGGC

TGTGACGTTACTCGCAGAAGAAGCACCGGCTAACTCCGTG

>427 len=135 tot-seq=216

TGGGGAATATTGCACAATGGGGGGAACCCTGATGCAGCAACGCCGCGTGAGTGAAGAAGT

ACCTCGGTATGTAAAGCTCTATCAGCAGGGAAGAAAATGACGGTACCTGACCAAGAAGCA

CCGGCTAAATACGTG

>428 len=111 tot-seq=214

GCTCAGCATTCATCCCTCCCTTGACAGCAGCAGCAGAACTGTGGTCCCCAGGAAAATCAC

AAATGGAACCATTTGTTTGCAATAATAACCCATTTGTCAGGGTTCATCTCC

>429 len=161 tot-seq=214

TAGGGAATCTTCGGCAATGGGGGCAACCCTGACCGAGCAACGCCGCGTGAGTGAAGAAGG

TTTTCGGATCGTAAAGCTCTGTTGTAAGAGAAGAACGGGTGTGAGAGTGGAAAGTTCACA

CTGTGACGGTATCTTACCAGAAAGGGACGGCTAACTACGTG

>430 len=203 tot-seq=212

TGGGGAATATTGCGCAATGGGGGGAACCCTGACGCAGCCATGCCGCGTGAATGAAGAAGG

CCTTCGGGTTGTAAAGTTCTTTCGGTATTGAGGAAGGGATGTGTGCTAATAGTACATATC

ATTGACGTTAAATACAGAAGAAGCACCGGCTAACTCCGTGGGGGGAGCCGGGTAATTGCC

AGATCGGAAGAGCGTCGTGTTGG

>431 len=156 tot-seq=210

TGAGGAATCTTGGTCAATGGGCGCGAGCCTGAACCAGCCAAGTAGCGTGCAGGAAGACGG

CCCTATGGGTTGTAAACTGCTTTTTTATGGGAATAAAAGAGATCACGTGTTGATTTTTTG

CAGGTACCATACGAATAAGGACCGGCTAATTCCGTG

>432 len=161 tot-seq=210

TAGGGAATCTTCCGCAATGGACGAAAGTCTGACGGAGCAACGCCGCGTGAGCGATGAAGG

CCTTCGGGTCGTAAAGCTCTGTTGTCAGGGAAGAACAAGTGCCGTTCGAACAGGGCGGCA

CCTTGACGGTACCTGACCAGAAAGCCACGGCTAACTACGTG

>433 len=159 tot-seq=208

TGGGGAATTTTGGACAATGGGCGCAAGCCTGATCCAGCAATGCCGCGTGCAGGAAGAAGG

CCTTCGGGTTGTAAACTGCTTTTGTACGGAACGAAAAGCTCTGGGTTAATACCCTGGGGT

GATGACGGTACCGTAAGAATAAGCACCGGCTAACTACGT

>434 len=160 tot-seq=207

TGGGGAATTTTGGACAATGGGCGCAAGCCTGATCCAGCAATGCCGCGTGCAGGAAGAAGG

CCTTCGGGTTGTAAACTGCTTTTGTCAGGGAAGAAATCCTTTGGGTTAATACCTCGAAGG

GATGACGGTACCTGAAGAATAAGCACCGGCTAACTACGTG

>435 len=156 tot-seq=205

TGAGGAATATTGGTCAATGGGCGAGAGCCTGAACCAGCCAAGTAGCGTGCAGGATGACGG

CCCTATGGGTTGTAAACTGCTTTTATAGGGGAATAAAGTGAGCCACGTGTGGTTTTTTGC

ATGTACCCTATGAATAAGGACCGGCTAATTCCGTGG

>436 len=135 tot-seq=204

TCGGGAATATTGCGCAATGGAGGAAACTCTGACGCAGTGACGCCGCGTGCAGGAAGAAGG

TTTTCGGATTGTAAACTGCTTTAGACAGGGAAGAAAAAAGACAGTACCTGTAGAATAAGC

TCCGGCTAACTACGT

>437 len=160 tot-seq=203

TGGGGAATTTTGGACAATGGGGGCAACCCTGATCCAGCCATGCCGCGTGCGGGAAGAAGG

CCTTCGGGTTGTAAACCGCTTTTGTCAGGGACGAAAAGCTCCGGAGTAATATGCCGGAGT

GCTGACGGTACCTGAAGAATAAGCACCGGCTAACTACGTG

>438 len=138 tot-seq=203

TGGGGAATATTGGGCAATGGGCGCAAGCCTGACCCAGCAACGCCGCGTGAGGGAAGAAGG

TTTTCGGATTGTAAACCTCTGTCCTAAGGGAAGAAGGAAGTGACGGTACCTTAGGAGGAA

GCCCCGGCTAACTACGTG

>439 len=160 tot-seq=202

TAGGGAATCTTCCACAATGGGCGCAAGCCTGATGGAGCAACACCGCGTGAGTGAAGAAGG

GTTTCGGCTCGTAAAACTCTGTTGTTGGAGAAGAACGTGCGTAAGAGTAACTGTTTACGC

AGTGACGGTATCCAACCAGAAAGTCACGGCTAACTACGTG

>440 len=219 tot-seq=201

GCAGGTAAGGAAGAGAGAGAGAAAGCACGAGAGGCAGGGGGGTGGAGTGAGAGGCAAAAT

GGACTAGTCAAAACTGTCTGCAGATTCAATTATTCTACTAAACACGATGGGCAAATAGAA

AACAGCTAAGACGACACACATGGGCAGGCAGCATTCAGTGGGGAAAAGGGCCGTTCTCAT

CTCAGCTCCCCTCCTTTCAGCCCTGCGTGCACTCGAGAA

>441 len=155 tot-seq=198

TGAGGAATATTGGTCAATGGGCGGAAGCCTGAACCAGCCAAGTAGCGTGCAGGATGACGG

CCCTACGGGTTGTAAACTGCTTTTATGCGGGGATAAAGGGCAATACGCGTATTGCTTTGC

AGGTACCGCATGAATAAGGACCGGCTAATTCCGTG

>442 len=111 tot-seq=194

TCTGGGGCTTTGCTGGCCATTTGAAGGTGCAGAGACTAGTGTATATTAGGGCAGCCCTGG

TGTTTGCTCAGTATCACAGTGGGCCTTTCTCTTTGCTACAGGAAAATCCCA

>443 len=140 tot-seq=194

TGGGGAATATTGCACAATGGGCGGAAGCCTGATGCAGCGACGCCGCGTGAGGGATGACGG

CCTTCGGGTTGTAAACCTCTTTCAGCTCTGACGAAGCTTTTGTGACGGTAGGAGCAGAAG

AAGCACCGGCTAACTACGTG

>444 len=156 tot-seq=193

TGAGGAATATTGGTCAATGGGCGCGAGCCTGAACCAGCCAAGTCGCGTGAGGGATGACGG

CCCTACGGGTTGTAAACCTCTTTTGTCGGGGAGCAAATTCCGCCACGTGTGGCGGAGTCG

AGAGTACCCGAAGAAAAAGCATCGGCTAACTCCGTG

>445 len=160 tot-seq=192

TGGGGAATATTGCACAATGGGCGCAAGCCTGATGCAGCCATGCCGCGTGTATGAAGAAGG

CCTTCGGGTTGTAAAGTACTTTCAGCGGGGAGGAAGGCGTTGTGGTTAATAACCACAGCG

ATTGACGTTACCCGCAGAAGAAGCACCGGCTAACTCCGTG

>446 len=154 tot-seq=191

TGAGGAATATTGGTCAATGGCCGGAAGGCTGAACCAGCCAAGTCGCGTGAGGGAAGACGG

CCCTACGGGTTGTAAACCTCTTTTGTCGGGGAGCAAAGGCGGTCATGCGTGACCGGATGA

GAGTACCCGAAGAAAAAGCATCGGCTAACTCCGT

>447 len=156 tot-seq=190

TGAGGAATATTGGTCAATGGGCGGAAGCCTGAACCAGCCAAGTCGCGTGAGGGAATAAGG

CCCTACGGGTCGTAAACCTCTTTTGCCGGGGAGCAAGGGCGGGGACGTGTCCCGGCCCGG

AGAGTACCCGGAGAAAAAGCATCGGCTAACTCCGTG

>448 len=206 tot-seq=188

TGAGGAATATTGGTCAATGGGCGAGAGCCTGAACCAGCCAAGTAGCGTGCAGGATGACGG

CCCTATGGGTTGTAAACTGCTTTTATATGGGGATAAAGTGGGGAACGTGTTCCCTTTTGC

AGGTACCATATGAATAAGGACCGGCTAATTCCGTGGGGGGAGCCGCGGTAATCAGTAGAT

CGGAAGAGCGTCGTGTAGGGAACGAG

>449 len=133 tot-seq=188

TCGGGAATATTGCACAATGGAGGAAACTCTGATGCAGTGACACCGCGTATAGGAAGAAGG

CCTTAGGGTTGTAAGCTATTGTCGTGTGAGAAGAAAATGACTATCACAGGAGGAAGCCCT

GGCTAAATATGTG

>450 len=135 tot-seq=187

TGGGGAATATTGCACAATGGGGGAAACCCTGATGCAGCAACGCCGCGTGAGTGAAGAAGT

ATTTCGGTATGTAAAGCTCTATCAGCAGGGAAGATAATGACGGTACCTGACTAAGAAGCT

CCGGCTAAATACGTG

>451 len=142 tot-seq=186

TGGGGAATCTTGCACAATGGGCGGAAGCCTGATGCAGCGACGCCGCGTGCGGGACGAAGG

CCTTCGGGTCGTAAACCGCTTTCAGCAGGGACGAGGCCGCGAGGTGACGGTACCTGCAGA

AGAAGCCCCGGCTAACTACGTG

>452 len=160 tot-seq=186

TAGGGAATCTTCCACAATGGACGCAAGTCTGATGGAGCAACGCCGCGTGAGTGAAGAAGG

CTTTCGGGTCGTAAAACTCTGTTGTTGGAGAAGAATGGTCGGCAGAGTAACTGTTGTCGG

CGTGACGGTATCCAACCAGAAAGCCACGGCTAACTACGTG

>453 len=166 tot-seq=185

AGGGAGGAGAGGGTGGGAGAGGAAGAGCAGAGTGAGGGGATGGATTCTAGAGGAGGTGAC

AGAGATGACTCAGACACCTTAGAGGAGGCCTGGACTCAGCTCAAACAGAAGTGTCCTCTT

CGGGCCCTTCTAAAATGCCTGGCAGCAACCAATGTCCCATGACTGT

>454 len=138 tot-seq=184

TGGGGAATATTGGACAATGGGGGCAACCCTGATCCAGCAATTCTGTGTGCACGATGAAGG

TTTTCGGATTGTAAAGTGCTTTCAGCAGGGAAGAAAAAAATGACGGTACCTGCAGAAGAA

GCGACGGCTAAATACGTG

>455 len=160 tot-seq=184

TGGGGAATATTGCGCAATGGGGGAAACCCTGACGCAGCGACGCCGCGTGTATGAAGAAGG

CCTTCGGGTTGTAAAGTACTTTCAGCGGGGAGGAAGGGAGTAAAGTTAATACCTTTGCTC

ATTGACGTTACCCGCAGAAGAAGCACCGGCTAACTCCGTG

>456 len=160 tot-seq=183

TGGGGAATATTGCACAATGGGGGAAACCCTGATGCAGCAACGCCGCGTGAGTGAAGAAGG

TTTTCGGATCGTAAAGCTCTGTTGTTGGTGAAGAAGGATAGAGGTAGTAACTGGCCTTTA

TTTGACGGTAATCAACCAGAAAGTCACGGCTAACTACGTG

>457 len=142 tot-seq=182

TAGGGAATCTTCCACAATGGACGCAAGTCTGATGGAGCAACGCCGCGTGCGGGATGAAGG

CCTTCGGGTTGTAAACCGCTTTTGATTGGGAGCAAGCCTTCGGGTGAGTGTACCTTTCGA

ATAAGCGCCGGCTAACTACGTG

>458 len=160 tot-seq=181

TGGGGAATATTGCGCAATGGGGGAAACCCTGACGCAGCGACGCCGCGTGAGTGAAGAAGG

TTTTCGGATCGTAAAGCTCTGTTGTAAGAGAAGAACGAGTGTGAGAGTGGAAAGTTCACA

CTGTGACGGTATCTTACCAGAAAGGGACGGCTAACTACGT

>459 len=156 tot-seq=180

TGAGGAATATTGGTCAATGGGCTTACGCCTGAACCAGCCAAGTCGCGTGAGGGAAGACTG

CCCTATGGGTTGTAAACCTCTTTTGTCAGGGAGCAACGCCGGCTACGTGTAGCCGGACTG

AGAGTACCTGAAGAAAAAGCATCGGCTAACTCCGTG

>460 len=160 tot-seq=180

TAGGGAATCTTCCGCAATGGGCGAAAGCCTGACGGAGCAATGCCGCGTGAGTGAAGAAGG

CCTTCGGGTCGTAAAACTCTGTTATAAGAGAAGAACAAATTGTAGAGTAACTGCTACAGT

CTTGACGGTATCTTATCAGAAAGCCACGGCTAACTACGTG

>461 len=138 tot-seq=179

TGGGGAATCTTGGACAATGGGTGAAAGCCTGATCCAGCAATATCGCGTGAGTGAAGAAGG

GCAATGCCGCTTGTAAAGCTCTTTCGTCGAGTGAGCGCAAATGACAGGACTCGAGGAAGA

AGCCCCGGTTAACTCCAT

>462 len=171 tot-seq=177

TTCTGTCCAGGGAATTCCATCAGGGGAACGGGTGGGTCACAGTAGCCACGGGCTCATCCA

CAGTGCCACAGTGCACAGGGCACGTGGGCAAGGGCTGCAACTGCCAGCATCCTAGGCACA

CCCCGAGTCTCCAGGCAGGGGTGTGTGCACAGCCCTTCACAGTGGCAGCTC

>463 len=136 tot-seq=176

TGGGGAATATTGCACAATGGGGGAAACCCTGATGCAGCGACGCCGCGTGGGCGAGGAAGT

ACCTCGGTATGTAAAGCCCTATCAGCAGGGAAGAAACAGGACAGTACCTGACTAAGAAGC

CCCGGCTAACTACGTG

>464 len=160 tot-seq=176

TAGGGAATATTGCACAATGGAGGAAACTCTGATGCAGCCATGCCGCGTGTGTGAAGAAGG

CCTTCGGGTTGTAAAGTACTTTCAGCGGGGAGGAAGGGAGTAAAGTTAATACCTTTGCTC

ATTGACGTTACCCGCAGAAGAAGCACCGGCTAACTCCGTG

>465 len=160 tot-seq=174

TAGGGAATCTTCCACAATGGACGAAAGTCTGATGGAGCAACGCCGCGTGAGCGAAGAAGG

TTTTCGGATCGTAAAGCTCTGTTGCGAGGGAAGAACAAGTACGGGAGTAACTGCCCGTAC

CATGACGGTACCTCGTCAGAAAGCCACGGCTAACTACGTG

>466 len=141 tot-seq=174

GTACACGAGAGGGAAAATGCTGTGAGCAGTGAGACTTTGGCACAAAGATGAAAATAACCA

TGTTGGCAACAGATAATGGACAGCGAGGAGGGATGACTGTTCCTCAGCTCTGCCCCTTTG

CCTGTGCACGGAAACAGTGGT

>467 len=155 tot-seq=172

TAAGGAATATTGGACAATGGACGCAAGTCTGATCCAGCCATGCCGCGTGAAGGATGAATG

CCCTCTGGGTTGTAAACTTCTTTTACAGGGGAAGAAAGTTATCTTTTTTAGGATATTTGA

CGGTACCCTATGAATAAGCACCGGCTAACTCCGTG

>468 len=160 tot-seq=172

TAGGGAATCTTCCACAATGGGCGAAAGCCTGATGGAGCAACGCCGCGTGTGTGATGAAGG

CTTTAGGGTCGTAAAGCACTGTTGTATGGGAAGAAATGCTAGAATAGGGAATGATTCTAG

TTCGACGGTACCATACCAGAAAGGGACGGCTAAATACGTG

>469 len=215 tot-seq=171

GCCAACAGGAGCCACAACAGATGTGGGCATCTGGCAGGAGACACACACCCACAGACAGTT

GCTTCCTTTCTCTGCTTTCCACTTGCTGTCAGAGACATTCCCCAGCATCAAGGTCAGAAA

GGAGGCTGGGTAGGGGAGTGTCTGGAGCTAGGACTGTTCAGACAGGAGCTGGCAATGGCA

GAAGGGTCTGTGGGGTATCTAGATGTCATCCTTGT

>470 len=152 tot-seq=171

TGGGGGATATTGCACAATGGACGGAAGTCTGATGCAGCGACGTCGTGTGGGGGATGAAGG

CCTTCGGGTTGTAAACTCCTTTCGCCCGCGGAAAAGGCAGGGTTTTGGCCTTGTTGATGG

TAGTGGGTAAAGAAGCGCCGGCTAACTACGTG

>471 len=160 tot-seq=170

TGGGGAATTTTGGACAATGGGCGCAAGCCTGATCCAGCAATGCCGCGTGAGTGAAGAAGG

CCTTCGGGTTGTAAAGCTCTTTTGTCAGGGAAGAAACGGCTGCGGCTAATATCTGCGGCT

AATGACGGTACCTGAAGAATAAGCACCGGCTAACTACGTG

>472 len=137 tot-seq=170

TGGGGGATATTGCACAATGGGGGAAACCCTGATGCAGCGATGCCGCGTGGAGGAAGAAGG

TTTTCGGATTGTAAACTCCTGTCGTAAGGGACGAAGAAGTGACGGTACCTTACAAGAAAG

CTCCGGCTAACTACGTG

>473 len=161 tot-seq=169

TAGGGAATCTTCGGCAATGGACGAAAGTCTGACCGAGCAACGCCGCGTGAGTGATGAAGG

TTTTCGGATCGTAAAACTCTGTTGTTAGAGAAGAACAAGTACCGTTTGAATAAGGCGGTA

CCTTGACGGTACCTAACGAGAAAGCCCCGGCTAACTACGTG

>474 len=137 tot-seq=169

TGGGGAATATTGGACAATGGAGGAGACTCTGATCCAGCAATTCTGTGTGCACGAAGACGG

TTTTCGGATTGTAAAGTGCTTTCAGCAGGGAAGAAGAAAGTGACGGTACCTGCAGAAGAA

GCGACGGCTAAATACGT

>475 len=203 tot-seq=167

TAGGGAATCTTCGGCAATGGGGGCAACCCTGACCGAGCAACGCCGCGTGAGTGAAGAAGG

GTTTCGGCTCGTAAAGCTCTGTTGGTAGTGAAGAAAGATAGAGGTAGTAACTGGCCTTTA

TTTGACGGTAATTACTTAGAAAGTCACGGCTAACTACGTGGGGGGGAGCCGCGGTATCACA

AGATCGGAAGAGCGTCGTGTAGG

>476 len=161 tot-seq=166

TGGGGAATATTGCGCAATGGGCGAAAGCCTGACGCAGCGACGCCGCGTGAGGGATGAAGG

TTCTCGGATCGTAAACCTCTGTCAGGGGGGAAGAAACCCCCTCGTGTGAATAATGCGAGG

GCTTGACGGTACCCCCAAAGGAAGCACCGGCTAACTCCGTG

>477 len=135 tot-seq=166

TGGGGGATATTGCACAATGGGGGGAACCCTGATGCAGCGACGCCGCGTGGGTGAAGGAGT

GCTTCGGCATGTAAAGCCCTATCGGCAGGGAAGAAGCAAGACGGTACCTGACTAAGAAGC

CCCGGCTAACTACGT

>478 len=135 tot-seq=165

TGGGGAATATTGCACAATGGGGGAAACCCTGATGCAGCAACGCCGCGTGAGTGAAGAAGG

CTTTCGGGTCGTAAAACTCTGTCGCATAGGAAGAAAAGGACAGTACTATGCAAGGAAGTC

CCGGCTAACTACGTG

>479 len=156 tot-seq=163

TGAGGAATATTGGTCAATGGCCGGAAGGCTGAACCAGCCAAGTCGCGTGAGGGAATAAGG

CCCTATGGGTCGTAAACCTCTTTTGTCAGGGAGCAAAGGCGTCCACGTGTGGACGAGAGG

AGAGTACCTGAAGAAAAAGCATCGGCTAACTCCGTG

>480 len=203 tot-seq=163

TCAGCAGAGACTGTGGCAAAGAAGTGAAGTCTCTGGCGGACATCTGGACCTGCAAGCCAT

CCAGAGCATTGTCCTTGGCAGTGGCGAGATGGTCAGGTCAGCACCTGGGTCCTTCCCAGC

TTAGGCACACTTTTTGCCAGGATAGGAGCTTCTCAGGCCAGGGCCTCTCTTGCCAGTGCA

GGCAGTCATGCCTGGCATCAAGT

>481 len=154 tot-seq=163

TGAGGAATATTGGTCAATGGGCGAGAGCCTGAACCAGCCAAGTCGCGTGAGGGAAGACGG

TCCTACGGATTGTAAACCTCTTTTGTCAGGGAGCAAGGAGCGCCACGAGTGGTGCGGCGA

GAGTACCTGAAGAAAAAGCATCGGCTAACTCCGT

>482 len=172 tot-seq=163

TGGGGAATATTGCGCAATGGGGGAAACCCTGACGCAGCGACGCCGCGTGAGTGAAGAAGG

GTTTCGGCTCGTAAAGCTCTGTTGTTGGTGAAGAAGGACAGGGGTAGTAACTGACCTTTG

TTTGACGGTAATCAATTAGAAAGTCACGGCTAACTACGTGGGGGGAGCGCGG

>483 len=154 tot-seq=162

TGAGGAATATTGGTCAATGGACGAGAGTCTGAACCAGCCAAGTAGCGTGCAGGATGACGG

CCCTATGGGTTGTAAACTGCTTTTATAGGGGGATAAAGTGTGTCACGTGTGGCATATTGC

AGGTACCCTATGAATAAGGACCGGCTAATTCCGT

>484 len=135 tot-seq=161

TGGGGAATATTGCACAATGGGGGAAACCCTGATGCAGCGACGTCGCGTGAGCGAAGAAGG

CCTTCGGGTCGTAAAGCTCTGTCCTATGGGAAGAAAAGGACGGTACCATAGAAGGAAGCC

CCGGCTAACTACGTG

>485 len=140 tot-seq=161

TGGGGAATATTGCACAATGGGCGCAAGCCTGATGCAGCGACGCCGCGTGGGGGATGACGG

CCTTCGGGTTGTAAACTCCTTTCGCTAGGGACGAAGCGCAAGTGACGGTACCTAGAGAAG

AAGCACCGGCTAACTACGTG

>486 len=159 tot-seq=160

TAGGGAATCTTCCACAATGGACGCAAGTCTGACGGAGCAACGCCGCGTGAGTGATGACGG

CCTTCGGGTTGTAAAGCTCTGTTAATCGGGACGAATGGTCCTTGTGTGAATAATGCAAGG

ATTTGACGGTACCGGAATAGAAAGCCACGGCTAACTACG

>487 len=141 tot-seq=160

TGGGGAATCTTCCGCAATGGACGAAAGTCTGACGGAGCGACGCCGCGTGCGGGATGAAGG

CCTTCGGGTTGTAAACCGCTTTTGATTGGGAGCAAGCTTTCGGGTGAGTGTACCTTTCGA

ATAAGCGCCGGCTAACTACGT

>488 len=152 tot-seq=159

TAAGGAATATTGGACAATGGGCGAAAGCCTGATCCAGCCATGCCGCGTGAAGGATTAAGG

TCCTCTGGATTGTAAACTTCTTTTATTTGGGACGAAAAAAGGGAATTCTTTCCCACTTGA

CGGTACCAAGTGAATAAGCACCGGCTAACTCC

>489 len=155 tot-seq=158

TGAGGAATATTGGTCAATGGGCGGGAGCCTGAACCAGCCAAGTCGCGTGAGGGATGACGG

CCCTATGGGTTGTAAACCTCTTTTGCCGGGGAGCAAAGTGCCGCACGTGTGCGGTTTGGA

GAGTACCCGGAGAAAAAGCATCGGCTAACTCCGTG

>490 len=160 tot-seq=157

TGGGGAATTTTGGACAATGGGCGCAAGCCTGATCCAGCCATGCCGCGTGTCTGAAGAAGG

CCTTCGGGTTGTAAAGGACTTTTGTCAGGGAGGAAATCCCTAGCGCTAATATCGCTGGGG

GATGACAGTACCTGAAGAATAAGCACCGGCTAACTACGTG

>491 len=160 tot-seq=156

TGGGGAATTTTGGACAATGGGCGAAAGCCTGATCCAGCAATGCCGCGTGTGTGATGAAGG

CCTTCGGGTTGTAAAGCACTTTTGGCGGGAACGAAAAGGACTGTGCCAATACCACGGTTC

GATGACGGTACCCGCAGAATAAGCACCGGCTAACTACGTG

>492 len=160 tot-seq=156

TAGGGAATTTTCGGCAATGGGGGGAACCCTGACCGAGCAACGCCGCGTGAAGGAAGAAGG

AATTCGTTCTGTAAACTTCTGTTATAAAGGAAGAAAGACGGATGGAGGAAATGACATCCG

AGTGACGGTACTTTATGAGAAAGCCACGGCTAACTACGTG

>493 len=217 tot-seq=155

ACAGTGGCCCCCACTGTAGCTATCAATGCAATCCAGGCTCAACTGAGGTCACTCTGTCAC

TTGAGATTGAAATAAAATTGGCTTAACCCCCCTATCTTAAATCCATTGGGAAGACCAAGA

TTGTAGCACATTCCCAGGAACTGAAATTCTCAAGGCACTGCTGACTCCACAGTTTCAGGC

TGTCCCTCTCAATCCCTCACTCAGCTCTTCTGCTTCC

>494 len=136 tot-seq=153

TGGGGAATATTGGGCAATGGGCGAAAGCCTGACCCAGCAACGCCGCGTGAGGGAAGAAGG

GTTTCGGCTCGTAAACCTCTGTCCTATGGGACGAAAGAGATGACGGTACCATAGGAGGAA

GCCCCGGCTAACTACG

>495 len=161 tot-seq=152

TAGGGAATCTTCCACAATGGGCGAAAGCCTGATGGAGCAACGCCGCGTGGGTGAAGAAGG

TCTTCGGATCGTAAAACCCTGTTGTTAGAGAAGAACGTTGGTAGGAGTGGAAAATCTACC

AAGTGACGGTAACTAACCAGAAAGGGACGGCTAACTACGTG

>496 len=161 tot-seq=150

TGGGGAATCTTCCGCAATGGGCGAAAGCCTGACGGAGCAACGCCGCGTGAGTGATGACGG

CCTTCGGGTTGTAAAGCTCTGTGATCGGGGACGAACGGTCAGCAGACGAATACTCTGCTG

AAGTGACGGTACCCGAATAGCAAGCCACGGCTAACTACGTG

>497 len=214 tot-seq=150

TGAGGAATATTGGTCAATGGGCGGGAGCCTGAACCAGCCAAGTCGCGTGAGGGACGACGG

TCCTATGGATTGTAAACCTCTTTTGTCGGGGAACAAAGGCGTCCACGAGTGGGCGGATGA

GTGTACCCGAAGAAAAAGCATCGGCTAACTCCGTGCCAGCGCCGCGGAATTAAGAGATCG

GAAGAGCGTCGTGTAGGGAAAAGAGGAATATTGG

>498 len=213 tot-seq=150

TAGGGAATCTTCCACAATGGACGCAAGTCTGATGGAGCAACGCCGCGTGAGTGAAGAAGG

TTTTCGGATCGTAAAGCTCTGTTGTTAAGGAAGAACGAGTGTGAGAATGGAAAGTTCATG

CTGTGACGGTACTTAACCAGAAAGGGACGGCTAACTACGTGGGGGGAGCCCGCGGTAATA

GCTAGATCGGAAGAGCGTCGTGTAGGGATAGGG

>499 len=155 tot-seq=149

TGAGGAATATTGGTCAATGGACGCAAGTCTGAACCAGCCAAGTAGCGTGCAGGATGACGG

CCCTATGGGTTGTAAACTGCTTTTATGCGGGGATAAAGTGCGCGACGTGTCGTGCATTGC

AGGTACCGCATGAATAAGGACCGGCTAATTCCGTG

>500 len=141 tot-seq=149

TGGGGAATCTTGCGCAATGGGGGAAACCCTGACGCAGCGACGCCGCGTGCGGGATGAAGG

CCTTCGGGTTGTAAACCGCTTTCAGCAGGGACGAGGCCTTATGGTGACGGTACCTGCAGA

AGAAGCTCCGGCTAAATACGT

>501 len=141 tot-seq=146

TGGGGAATATTGCACAATGGGCGCAAGCCTGATGCAGCGACGCCGCGTGGGGGATGAAGG

CCTTCGGGTTGTAAACTCCTTTCGCTAGGGACGAAGCTTTTTGTGACGGTACCTGGAGAA

GAAGCACCGGCTAACTACGTG

>502 len=143 tot-seq=146

TAGGGAATCTTCCACAATGGGGGAAACCCTGACGCAGCGACGCCGCGTGCGGGATGAAGG

CCTTCGGGTTGTAAACCGCTTTTGATTGGGAGCAAGCCTTTTGGGTGAGTGTACCTTTCG

AATAAGCGCCGGCTAACTACGTG

>503 len=215 tot-seq=145

TGAGGAATATTGGACAATGGGCGGAAGCCTGATCCAGCCATGCCGCGTGAAGGACTAAGG

CCCTATGGGTCGTAAACTTCTTTTGACGCAGAGCAATAAGGACCACGCGTGGACCGATGA

GAGTATGCGTCGAATAAGCATCGGCTAACTCCGTGGGGGGAGCCGGGTAATACAAAGATC

GGAAGAGCGTCGTGTAGGGGGGGAGTGTGCCAATG

>504 len=135 tot-seq=145

TAGGGAATATTGCTCAATGGGGGAAACCCTGAAGCAGCAACGCCGCGTGGAGGATGAAGG

TTTTAGGATTGTAAACTCCTTTTGTGAGAGAAGATTATGACGGTATCTCACGAATAAGCA

CCGGCTAACTCCGTG

>505 len=135 tot-seq=145

TGGGGAATATTGCGCAATGGGGGAAACCCTGACGCAGCGACGCCGCGTGAGCGAAGAAGT

ATTTCGGTATGTAAAGCTCTATCAGAAGGGAAGAAAATGACGGTACCTTACTAAGAAGCT

CCGGCTAAATACGTG

>506 len=182 tot-seq=144

TGAGGAATATTGGTCAATGGGCGAGAGCCTGAACCAGCCAAGTAGCGTGCAGGAAGACGG

CCCTATGGGTTGTAAACTGCTTTTATGTGGGGATAAAGTGCGCGACGTGTCGTGCATTGC

AGGTACCACATGAATAAGGACCGGCTAATTCCGTGGGGGGAGCCGCGTAATACCAAGATC

GG

>507 len=161 tot-seq=144

TGGGGAATATTGCACAATGGGGGAAACCCTGATGCAGCAACGCCGCGTGAGTGAAGAAGG

TTTTCGGATCGTAAAGCTCTGTTGTTAGAGAAGAACGTTGGTAGGAGTGGAAAATCTACC

AAGTGACGGTAACTAACCAGAAAGGGACGGCTAACTACGTG

>508 len=135 tot-seq=142

TGGGGAATATTGCGCAATGGAGGAAACTCTGACGCAGCAACGCCGCGTGAATGATGAAGG

CCTTAGGGTTGTAAAGTTCTGTCTTCTGGGACGATAATGACGGTACCAGAGGAGGAAGCC

ACGGCTAACTACGTG

>509 len=135 tot-seq=142

TGGGGAATATTGCACAATGGGGGAAACCCTGATGCAGCGACGCCGCGTGAACGAAGAAGT

ACCTCGGTATGTAAAGTTCTATCAGCAGGGAAGAAAATGACGGTACCTGACTAAGAAGCC

CCGGCTAACTACGTG

>510 len=160 tot-seq=142

TAGGGAATATTGCACAATGGGGGAAACCCTGATGCAGCCATGCCGCGTGTGTGAAGAAGG

CCTTCGGGTTGTAAAGCACTTTCAGATGGGAAAAAAGTAGTAATTCGAATAGGGTTGCTA

TGTGATATTACTATCAGAAGAAGCACCGGCTAACTCCGTG

>511 len=161 tot-seq=142

TGGGGAATCTTCCGCAATGGACGAAAGTCTGACGGAGCAACGCCGCGTGAGTGAAGAAGG

CCTTCGGGTTGTAAAGCTCTGTTATTCGGGAAGAAAGATTCGTTTGTGAACAATGAACGG

AAGTGACGGTACCGAAAGAGAAAGCCACGGCTAACTACGTG

>512 len=135 tot-seq=140

TGGGGAATCTTCCGCAATGGACGAAAGTCTGACGGAGCAACGCCGCGTGAACGATGAAGG

TCTTCGGATCGTAAAGTTCTGTTGCAGGGGAAGATAATGACGGTACCCTGTGAGGAAGCC

CCGGCTAACTACGTG

>513 len=160 tot-seq=140

TAGGGAATCTTCGGCAATGGACGGAAGTCTGACCGAGCAACGCCGCGTGAGTGAAGAAGG

TTTTCGGATCGTAAAGCTCTGTTGTTGGAGAAGAACGTGCGTAAGAGTAACTGTTTACGC

AGTGACGGTATCCAACCAGAAAGTCACGGCTAACTACGTG

>514 len=160 tot-seq=138

TGGGGAATTTTGGACAATGGGCGCAAGCCTGATCCAGCTATTCCGCGTGTGGGATGAAGG

CCCTCGGGTTGTAAACCACTTTTGTAGAGAACGAAAAGACACCTTCGAATAAAGGGTGTT

GCTGACGGTACTCTAAGAATAAGCACCGGCTAACTACGTG

>515 len=212 tot-seq=137

GATCACGTGAGGCCAGGAGTTCAAGGACAGCCTGAGCAATATTGCAAGATCTTTCCTCTA

AAAAATTAATAAACTTAAAAAAAAAAAGAAATGGAACAGTCAACCCAACCCTCCTTCTCC

TATATTTACAGAGAGACCACATACTTAAACAAATATAAGCATGCTTAACAATGCAATGAG

ACAAGTAACCAGAGGGAAGGGTCTGTGGAAGT

>516 len=145 tot-seq=135

GAAAAAGATCATGCCCAGTAAGCTGCTTGAAGAAATGGGTAATGCTCCTATGCTAATTTC

ATGTTTCAATCCTTTATTGTATTTTCTTTAACAATCGAATTTCGCTTTCAAAATTAATGC

ATTAATTAAACACTATATCCTGTTC

>517 len=154 tot-seq=135

TGAGGAATATTGGTCAATGGGCGAGAGCCTGAACCAGCCAAGTCGCGTGAAGGATGACTG

TCTTATGGATTGTAAACTTCTTTTATACGGGAATAACAAGAGTCACGTGTGACTCCCTGC

ATGTACCGTATGAATAAGCATCGGCTAACTCCGT

>518 len=135 tot-seq=135

GCGCGCAAATTACCCAATCCTGACACGGGGAGGTAGTGACAATAAATAACAATACCGGGC

GCATTAGTGTCTGGTAATTGGAATGAGTACAATCTAAATCCCTTAACGAGGATCCATTGG

AGGGCAAGTCTGGTG

>519 len=160 tot-seq=134

TGGGGAATCTTCCGCAATGGGCGCAAGCCTGACGGAGCAACGCCGCGTGAGTGAAGAAGG

TTTTCGGATCGTAAAGCTCTGTTGAGAGGGACGAGAGGCAAGGCTAGGAAATGAGCTTTG

TAGGACGGTACCTTTCGAGGAAGCCACGGCTAACTACGTG

>520 len=138 tot-seq=133

GGGGGAATATTGGGCAATGGGGGGAACCCTGACCCAGCAATGCCGCGTGAAGGAAGAAGG

CCTTCGGGTTGTAAACTTCTGTGACGAGGGACGAAGGAAGTGACGGTACCTCGAGAGGAA

GCTCCGGCAAACTACGTG

>521 len=161 tot-seq=130

TAGGGAATCTTCGGCAATGGGGGGAACCCTGACCGAGCAACGCCGCGTGAGTGAAGAAGG

TTTTCGGATCGTAAAGCTCTGTTGTTAAGGAAGAACGTGTGTGAGAGTGGAAAGTTCACA

CAGTGACGGTACTTAACCAGAAAGGGACGGCTAACTACGTG

>522 len=135 tot-seq=129

TGGGGGATATTGCACAATGGGGGGAACCCTGATGCAGCGACGCCGCGTGGGTGAAGAAGC

GCCTCGGCGCGTAAAGCCCTGTCAGCAGGGAAGAAAATGACGGTACCTGAAGAAGAAGCC

CCGGCTAACTACGTG

>523 len=142 tot-seq=129

TGGGGAATCTTGCGCAATGGGCGAAAGCCTGACGCAGCGATGCCGCGTGCGGGATGAAGG

CCTTCGGGTCGTAAACCGCTTTCAGCAGGGACGAGGCCGCAAGGTGACGGTACCTGCAGA

AGAAGCCCCGGCTAACTACGTG

>524 len=138 tot-seq=128

TGGGGAATATTGCACAATGGGGGAAACCCTGATGCAGCAACGCCGCGTGAAGGAAGAAGG

TTTTCGGATCGTAAACTTCTATCAACAGGGACGAAGAAAGTGACGGTACCTGAATAAGAA

GCCCCGGCTAACTACGTG

>525 len=161 tot-seq=128

TGGGGAATATTGCACAATGGGGGAAACCCTGACCGAGCAACGCCGCGTGAGTGAAGAAGG

TTTTCGGATCGTAAAGCTCTGTTGTTAAGGAAGAACGAGTGTGAGAATGGAAAGTTCATG

CTGTGACGGTACTTAACCAGAAAGGGACGGCTAACTACGTG

>526 len=230 tot-seq=127

GGCGACATGCTCAGGACTTGATCAGGCGACATGCCCTTCCTGGCGTGGCCACCATGGGAC

AATGCATGCACAGCTTTCCATAGACCATCTCCTCAACACCACCACCCGCCCTCCCCCAAC

ACACACTCACATGCTTGCACACACACACGCACATAGGCACACACACCGAGAAAAGACAGC

AGGTCCAAGATGAATTATCTGAATCCAGCCCCAGCTCCCAAAGCATGGCC

>527 len=138 tot-seq=126

TGGGGAATTTTCCGCAATGGGCGAAAGCCTGACGGAGCAATACCGCGTGAGGGAGGAAGG

CTCTTGGGTTGTAAACCTCTTTTCTCAGGGAAGAATACAATGACGGTACCTGAGGAATAA

GCATCGGCTAACTCCGTG

>528 len=155 tot-seq=125

TGAGGAATATTGGTCAATGGGCGGGAGCCTGAACCAGCCAAGCCGCGTGAAGGAAGAAGG

CGCCAGGCGTCGTAAACTTCTTTTGTCGGGGAACAAAGGGCGCCACGTGTGGCGTTGTGA

GTGTACCCGAAGAAAAAGCATCGGCTAACTCCGTG

>529 len=122 tot-seq=125

TGGTTGGGGGAGTGGGGACCCTAGGGCAGGGTGCAGGCCTCCTGAGGCCTGGAGCTGCAG

GCCCTTCCAGGCACTGGACACTTAGGACTGTGGGAGCAGGCAGCAGATCAGCTTGTGTTC

AG

>530 len=135 tot-seq=124

TGGGGAATATTGCACAATGGAGGAAACTCTGATGCAGCGACGCCGCGTGAGTGAAGAAGT

ATTTCGGTATGTAAAGCTCTATCAGCAGGGAAGAAAATGACGGTACCTGACTAAGAAGCA

CCGGCTAAATACGTG

>531 len=142 tot-seq=124

TGGGGAATATTGCGCAATGGGGGAAACCCTGACGCAGCGACGCCGCGTGCGGGATGAAGG

CCTTCGGGTTGTAAACCGCTTTTGATTGGGAGCAAGCCTTTTGGGTGAGTGTACCTTTCG

AATAAGCGCCGGCTAACTACGT

>532 len=160 tot-seq=123

TGGGGAATTTTGGACAATGGGCGCAAGCCTGATCCAGCTATTCCGCGTGTGGGAAGACGG

CCTTCGGGTTGTAAACCACTTTTGTAGAGAACGAAAAGTCTATTGATAATACCGATAGAC

GATGACGGTACTCTAAGAATAAGCACCGGCTAACTACGTG

>533 len=160 tot-seq=122

TGGGGAATTTTGGACAATGGGCGCAAGCCTGATCCAGCAATGCCGCGTGCAGGAAGAAGG

CCTTCGGGTTGTAAACTGCTTTTGTCAGGGAAGAAATCTTCTGGGCTAATACCCCGGGAG

GATGACGGTACCTGAAGAATAAGCACCGGCTAACTACGTG

>534 len=154 tot-seq=122

TAAGGAATATTGGACAATGGGCGCAAGCCTGATCCAGCCATGCCGCGTGAAGGATTAAGG

TCCTCTGGATTGTAAACTTCTTTTATAAGGGACGAAAAAAGGGAATTCTTTCTCACTTGA

CGGTACCTTATGAATAAGCACCGGCTAACTCCGT

>535 len=136 tot-seq=122

TGGGGAATATTGCGCAATGGGGGAAACCCTGACGCAGCGACGCCGCGTGCGGGAAGGAGG

CCTTCGGGTCGTAAACCGCTTTCAGCAGGGAAGAAGCCTGACGGTACCTGCAGAAGAAGC

CCCGGCTAACTACGTG

>536 len=160 tot-seq=122

TAGGGAATCTTCCACAATGGACGCAAGTCTGATGGAGCAACGCCGCGTGAGTGATGAAGG

TCTTCGGATCGTAAAACTCTGTTATTAGGGAAGAACATATGTGTAAGTAACTGTGCACAT

CTTGACGGTACCTAATCAGAAAGCCACGGCTAACTACGTG

>537 len=142 tot-seq=121

TGAGGAATATTGGTCAATGGGCGAAAGCCTGATGCAGCGACGCCGCGTGCGGGATGAAGG

CCTTCGGGTTGTAAACCGCTTTCAGCAGGGACGAGGCCGCAAGGTGACGGTACCTGCAGA

AGAAGCCCCGGCTAACTACGTG

>538 len=135 tot-seq=120

AGGAAAGGATGATGTTCTTAGGCTTGGGTTTACCCCATGGGGGTGGAGAAGCAAGAGGAA

GTCAGAAATTTATTATGTGGGTGGGGAATCACTGTGTCACATGGAGAGAAGACAACTGCA

GGAATTGTTGGAATA

>539 len=159 tot-seq=120

TAGGGAATCTTCCACAATGGGTGAAAACCTGATGGAGCAATGCCGCGTGAATGAAGAAGG

TCTTCGGATCGTAAAGTTCTGTTATTAGAGAAGAACAAGTCAAGGAGTAACTGTCTTGAC

TTTGACGGTACCTAACCAGAAAGTCACGGCTAACTACGT

>540 len=154 tot-seq=120

TGAGGAATATTGGTCAATGGACGAAAGTCTGAACCAGCCAAGTAGCGTGCAGGATGACGG

CCCTCTGGGTTGTAAACTGCTTTTAGTTGGGAATAAAAAAGAGGACGTGTCCTCTATTGT

ATGTACCTTCAGAAAAAGGACCGGCTAATTCCGT

>541 len=135 tot-seq=120

TAGGGAATCTTCCACAATGGACGCAAGTCTGATGGAGCAACGCCGCGTGAGTGAAGAAGG

TTTTCGGATCGTAAAGCTCTGTCTTTGGGGAAGATAATGACGGTACCCAAGGAGGAAGCC

ACGGCTAACTACGTG

>542 len=154 tot-seq=119

TGAGGAATATTGGTCAATGGGCGAGAGCCTGAACCAGCCAAGTCGCGTGAGGGAAGAATG

GTCTATGGCCTGTAAACCTCTTTTGTCAAGGAAGAATAAAAGGTACGTGTACCTTCTTGC

CAGTACTTGACGAATAAGCATCGGCTAACTCCGT

>543 len=204 tot-seq=119

TTGCAGTAAATGAGACAGAATGTAGCTCAGGCAGCAGAATGACAGTCTAGGCTCCTGAGA

GCCAGACCCTGGCTCTTTGTAACCATGGTACGGAGTGGAGAGCACGTTCTGGTAACCTTG

GGCATGAGGTGGGGCCTGGGCAGTCAGGGCACCGTCCCTGAGAGGGTGAGAGCAAAAGCC

AGCCTCTGCCCTATGTGATGAAAC

>544 len=134 tot-seq=118

ACATGCACGGCACCACTCACACATGCACACACAAGGAAGCAGCAGACACGCACTGCCCTC

CTGCACACACACACACTCACACACCCTCACATACACTCACTGATGCATACAGCCCACGTC

AGAGTCCACTGGGC

>545 len=138 tot-seq=118

TGGGGAATATTGGGCAATGGGCGCAAGCCTGACCCAGCAACGCCGCGTGAAGGAAGAAGG

CTTTCGGGTTGTAAACTTCTTTTGTCGGGGACGAAACAAATGACGGTACCCGACGAATAA

GCCACGGCTAACTACGTG

>546 len=159 tot-seq=118

TAGGGAATTTTCGTCAATGGGGGGAACCCTGAACGAGCAATGCCGCGTGAGTGAAGAAGG

TCTTCGGATCGTAAAGCTCTGTTGTAAGAGAAAAAGGGCGCATATAGGAAATGATATGCG

ATTGATGGTATCTTACCAGAAAGCCACGGCTAACTACGT

>547 len=155 tot-seq=117

TGAGGAATATTGGTCAATGGGTGCAAGCCTGAACCAGCCATCCCGCGTGAAGGACGACTG

CCCTATGGGTTGTAAACTTCTTTTGTATAGGGATAAACCTACCCTCGTGAGGGTAGCTGA

AGGTACTATACGAATAAGCACCGGCTAACTCCGTG

>548 len=155 tot-seq=117

TGAGGGATATTGGTCAATGGGCGGAAGCCTGAACCAGCCATGCCGCGTGCCGGAAGACGG

CCCTACGGGCTGTAAACGGCTTTTCGCTCCGAGGAATAAGGGGGCCTCGGCCCCCGATGC

CAGTAGGGGCGGAATAAGGATCGGCTAACTCCGTG

>549 len=194 tot-seq=117

AAATCTTATAACAGATTACTACTTTGATCAGCATCAGAGGAAAAAAAAAAAGAGCCATAT

ATTTTGTCTGCATGGGAATGATGCATTCACCTGCCCGGACCTTTGGTTTTGCTCTACTGA

TAGAACGCAGGAGACACCCAAGGGTCACAGAGACACCCACAGTTCTGTGTTTTTCCACTC

CGAGCTTCCCTGTG

>550 len=154 tot-seq=115

TGAGGAATATTGGTCAATGGAGGAAACTCTGAACCAGCCAAGTCGCGTGAAGGATGACGG

TCCTATGGATTGTAAACTTCTTTAGTAGGTGAGTAAGGATAGGTACGTGTACCTATTAGA

AAGTAGCCTAAGAATAAGTATCGGCTAACTCCGT

>551 len=134 tot-seq=115

TGGGGAATATTGCACAATGGGCGAAAGCCTGATGCAGCAACGCCGCGTGCGGGATGACGG

CCTTCGGGTTGTAAACCGCTTTCACCAGGGGCGAATTTGACGGTACCTGGAGAAGAAGCA

CCGGCTAACTACGT

>552 len=154 tot-seq=115

TGAGGAATATTGGTCAATGGGCGAGAGCCTGAACCAGCCAAGTCGCGTGAGGGAAGACGG

TCCTATGGATTGTAAACCTCTTTTGCCGGGGAGCAAAAAGCGGTACGTGTACCGCATCGA

GAGTACCCGGAGAAAAAGCATCGGCTAACTCCGT

>553 len=156 tot-seq=112

TGAGGAATATTGGTCAATGGGCGAGAGCCTGAACCAGCCAAGTCGCGTGAGGGAAGACGG

CCCTACGGGTTGTAAACCTCTTTTGCCGGGGAGCAATGGCCGACACGCGTGTCGGCAGGG

AGAGTACCCGGAGAAAAAGCATCGGCTAACTCCGTG

>554 len=156 tot-seq=111

TGAGGAATATTGGTCAATGGGCGGGAGCCTGAACCAGCCAAGTCGCGTGAGGGAAGACGG

TCCTATGGATTGTAAACCTCTTTAGGCGGGGAGCAATGCCGGGCACGCGTGCCCGGAGGG

AGAGTACCCGCAGAATAAGCATCGGCTAACTCCGTG

>555 len=153 tot-seq=110

TGAGGAATATTGGTCAATGGACGCAAGTCTGAACCAGCCATGCCGCGTGCAGGATGACGG

CTCTATGAGTTGTAAACTGCTTTTGTACGAGGGTAAATCCCGATACGTGTATCGGGTTGA

AAGTATCGTACGAATAAGGATCGGCTAACTCCG

>556 len=155 tot-seq=110

TGAGGAATATTGGTCAATGGGCGCAAGCCTGAACCAGCCATGCCGCGTGCAGGATGAAGC

ATCTATGGTGTGTAAACTGCTTTTATACGAGAAGAAACAACGCTACGTGTAGCGTCTTGA

CGGTATCGTAAGAATAAGGACCGGCTAACTCCGTG

>557 len=140 tot-seq=110

TGGGGAATATTGCGCAATGGGGGAAACCCTGACGCAGCGACGCCGCGTGCGGGATGAAGG

CCTTCGGGTTGTAAACCTCTTTTATCGGGGAGCAAGCGAGAGTGAGTTTACCCGTTGAAT

AAGCACCGGCTAACTACGTG

>558 len=137 tot-seq=109

TGGGGAATATTGGGCAATGGGCGCAAGCCTGACCCAGCAACGCCGCGTGAAGGAAGAAGG

CCCTCGGGTTGTAAACTTCTTTTCTATAGGACGAAGAAGTGACGGTACTATAGGAATAAG

CCACGGCTAACTACGTG

>559 len=138 tot-seq=109

TGGGGAATATTGCGCAATGGGGGAAACTCTGATCCAGCAATTCTGTGTGTGTGAAGAAGG

TTTTAGGACTGTAAAACACTTTTAGTAGGGAAGAAAAAAATGACGGTACCTACAGAAGAA

GCAACGGCTAAATACGTG

>560 len=160 tot-seq=108

TGGGGAATATTGCACAATGGGGGAAACCCTGATGCAGCAACGCCGCGTGAGTGAAGAAGG

TCTTCGGATCGTAAAACTCTGTTGTTAGAGAAGAACACGAGTGAGAGTAACTGTTCATTC

GATGACGGTATCTAACCAGCAAGTCACGGCTAACTACGTG

>561 len=160 tot-seq=108

TAGGGAATTTTCGTCAATGGGGGCAACCCTGAACGAGCAATGCCGCGTGAGCGAAGAAGG

TCTTCGGATTGTAAAGCTCTGTTGCCGGGGAAAAAGGAAGGAAAGAGGAAATGCTTTTCT

TTGGATGGTACCCGGCCAGAAAGTCACGGCTAACTACGTG

>562 len=155 tot-seq=108

TGAGGAATATTGGTCAATGGACGGAAGTCTGAACCAGCCATGCCGCGTGCAGGAAGACGG

CTCTATGAGTTGTAAACTGCTTTTGTACAAGGGTAAACCTGAATACGTGTATTCAGCTGA

AAGTATTGTACGAATAAGGATCGGCTAACTCCGTG

>563 len=137 tot-seq=108

TGGGGAATTTGCCGCAATGGGCGAAAGCCTGACGGAGCAATTCCGCGTGGAGCTGGAAGG

CCTACAGGTCGTCAACTTGTTTTCTCGGAGAAGAAACAATGACGGTATCTGAGGAATAAG

CATCGGCTAACTCTGTG

>564 len=161 tot-seq=108

TGGGGAATATTGCGCAATGGGGGAAACCCTGACGCAGCGACGCCGCGTGAGTGATGACGG

TCTTCGGATTGTAAAGCTCTGTTAATCGGGACGAATGGTCGGTGTGCAAATAGTGCATGG

ACATGACGGTACCGGAATAGAAAGCCACGGCTAACTACGTG

>565 len=138 tot-seq=108

TGGGGGATATTGCACAATGGGGGAAACCCTGATGCAGCGACGCCGCGTGGGTGAAGAAGT

ATTTCGGTATGTAAAGCCCTATCAGCAGGGAAGAAAGAAGACGGTACCTGACTAAGAAGC

CCCGGCTAACTACGTGGG

>566 len=160 tot-seq=107

TGGGGAATTTTGGACAATGGGCGCAAGCCTGATCCAGCTATTCCGCGTGTGGGATGACGG

CCCTCGGGTTGTAAACCACTTTAGTAGAGAACGAAAAGGAGCTATCGAATAAATAGCTCT

GCTGACGGTACTCTAAGAATAAGCACCGGCTAACTACGTG

>567 len=161 tot-seq=107

TGAGGAATATTGGTCAATGGACGAAAGTCTGACGGAGCAACGCCGCGTGAGTGAAGACGG

TCTTCGGATTGTAAAGCTCTGTTATACGGGACGAACGGCAAGGTGGTAAATAGCCATCAT

GAGTGACGGTACCGTAAGAGAAAGCCACGGCTAACTACGTG

>568 len=113 tot-seq=107

CTTATGGAATGAATAGGTTGGTCGTAAGTGTTTGTCCTCAGGAAATGAGACAGAGAAAGA

GAAGACCGATGGGAGAGGAAGGGGGAGGGAAGAACTGAGAGGTGGCTGCCCAG

>569 len=137 tot-seq=106

CAAGGAATTTTCCGCAATGGGCGCAAGCCTGACGGAGCAACGCCGCGTGCGGGATGACGC

TTTTCGGAGTGTAAACCGCTGTTCGTGGGGACGAAGCACTGACGGTACCCACGGAGGAAG

GCCCGGCTAACTACGTG

>570 len=155 tot-seq=105

TGAGGAATATTGGTCAATGGGCGCGAGCCTGAACCAGCCAAGTAGCGTGAAGGATGACTG

CCCTATGGGTTGTAAACTTCTTTTATATTAGAATAAAGTGCAGTATGTATACTGTTTTGT

ATGTATAATATGAATAAGGATCGGCTAACTCCGTG

>571 len=161 tot-seq=105

TAGGGAATATTGCGCAATGGACGAAAGTCTGACGCAGCCACGCCGCGTGAGTGAGGAAGG

CCTTCGGGTTGTAAAGCTCTGTCATCCGGGAAAAACGGCTATCTGGGGAAAGTTCAGGTA

GCGTGATGGTACCGGAGGAGGAAGCACCGGCAAACTCTGTG

>572 len=155 tot-seq=104

TGAGGAATATTGGTCAATGGGCGCAAGCCTGAACCAGCCAAGTAGCGTGCAGGAAGACGG

CCCTATGGGTTGTAAACTGCTTTTATACGAGAATAATTTGATGCACGTGTGCGTTATTGC

ATGTATCGTATGAATAAGGACCGGCTAATTCCGTG

>573 len=160 tot-seq=104

TGGGGAATTTTGCGCAATGCTCGTAAGAGTGACGCAGCGACGCCGCGTGAATGACGAAGG

CCTTCGGGTCGTAAAGTTCTTTCGACAGGGAAGAAAATGCCTATAAGTAACTGTGTATGT

ATTGACGGTACCTGTATAAGCAGCCCCGGCTAACTCCGTG

>574 len=156 tot-seq=104

TGAGGAATATTGGTCAATGGGCGCAAGCCTGAACCAGCCAAGTCGCGTGAGGGAAGACGG

TCCTATGGATTGTAAACCTCTTTTGTCGGGGAGCAAAGCGCGGTACGAGTACCGTGAAGG

AGAGTACCCGAAGAAAAAGCATCGGCTAACTCCGTG

>575 len=135 tot-seq=103

TGGGGAATATTGCGCAATGGGGGAAACCCTGACGCAGCGACGCCGCGTGAACGATGAAGG

TCTTCGGATCGTAAAGTTCTGTTGCAGGGGAAGATAATGACGGTACCCTGTGAGGAAGCC

CCGGCTAACTACGTG

>576 len=204 tot-seq=102

CTCTCCGCTTTTGATCTTCCTAGCAGGTCCTGCGAGAGCCAGCATCAGCTCCCAGGGGGG

GCAGGAGGCTCTACATTCCATCGTCTCTGCAAAGCTCTTGGCACGGCCCTTGGCAGGCAG

CAGGCACACAATAAATGGCATTTCACAGCCATGTGGGAAAGACCACATGCCAGGTGGGAC

ACCTGGGGAGGCTGGGAAACTCGC

>577 len=135 tot-seq=102

TGGGGGATATTGCACAATGGGGGAAACCCTGATGCAGCGACGCCGCGTGAGTGAAGAAGT

ATTTCGGTATGTAAAGCTCTATCAGCAGGGAAGAAAATGACAGTACCTGACTAAGAAGCC

CCGGCTAACTACGTG

>578 len=139 tot-seq=101

TGGGGAATCTTGGACAATGGGCGAAAGCCCGATCTAGCAATATCGCGTGAGTGAAGAAGG

GCAATGCAGCTTATAAAGCTCTTTCATCGAGTGCGCGATCATGACAGGACTCGAGGAAGA

AGCCCTGGCTAACTCCGTG

>579 len=207 tot-seq=101

TCAGAACACAGCAACGACCCTTTACATTACTCAAGAGGTCCTTCGGAAGTTTTGATGTCT

GCCCTTTGCTTTCCTGGCCCGACCTCTCCCTCCCCTAGAACGGAAAAGCCCGTGGATGCG

CTGGAGCGTTCCAGGGTGAAACAAGCCTCCCTGCAACTTAAGATTCTTTATCACCCGCGC

AGGGGAATTCATTTCACGTTACTGGTA

>580 len=156 tot-seq=101

TGAGGAATATTGGTCAATGGAGGCAACTCTGAACCAGCCAAGTAGCGTGCAGGATGACGG

CCCTATGGGTTGTAAACTGCTTTTATGCGGGGATAAACTGAGCCACGTGTGGCTTATTGC

AGGTACCGCATGAATAAGGACCGGCTAATTCCGTGG

>581 len=135 tot-seq=99

TGGGGAATATTGGACAATGGGCGCAAGCCTGATCCAGCCATGCCGCGTGAGTGATGAAGG

CCTTAGGGTTGTAAAGCTCTTTTATCCGGGACGATAATGACGGTACCGGAGGAATAAGCC

CCGGCTAACTTCGTG

>582 len=225 tot-seq=99

CTGCAGCAGCGGCAACTGCTCGCATCCCAGCCGGGCTCGGGTTACCGCCTTCTTCTTCTA

GCATCTCCAGCCAGACAGGGAAAAGCTTGTTCAAGTTTGCAGCAGGACTCGGTCCCTGTG

CGTGCGAACCTGGGGCTCCGGGACGGGCCTGCTCCCCGGCGTCCCCACCTCCAGTGTTTA

AACAAGACCCGCAAACATTGCTTTCTCCCAGGACTCTGCTTGGAA

>583 len=175 tot-seq=99

TGGGGAATCTTGCGCAATGGGGGGAACCCTGACGCAGCGACGCCGCGTGCGGGACGAAGG

CCCTAGGGTCGTAAACCGCTTTCAGCAGGGAAGAGGCCGCGAGGTGACGGTACCTGCAGA

AGAAGCCCCGGCTAAATACGTGCCAGCAGCCGCGGTCCCAGATCGGAAGAGCGTC

>584 len=159 tot-seq=98

TGGGGAATATTGGACAATGGGCGCAAGCCTGATCCAGCAATGCCGCGTGTGTGAAGAAGG

CCTTCGGGTTGTAAAGCACTTTAAGTTGGGAAGAAAAAACTCGCTCTAACACAGTGAGTC

TTGACGGTACCGACAGAATAAGCACCGGCTAACTCTGTG

>585 len=161 tot-seq=98

TGGGGAATCTTCCGCAATGGACGAAAGTCTGACGGAGCAACGCCGCGTGAGTGATGACGG

CCTTCGGGTTGTAAAGCTCTGTGATCGGGGACGAATGGCTGGTATGCTAATACCATATCA

GAGTGACGGTACCCGAATAGCAAGCCACGGCTAACTACGTG

>586 len=174 tot-seq=97

CAGGTGCTACAAGGTAACTCAAGGTCACGGCAAGAACCCCATAGGGGCCTGAGATTATCT

GCTCACAAATGCTCTGTCTCACACACTGGCTGTCTGCTTTGCTCCAGGGGCTCATCCCAG

CGTTGGCCTCCCTCAGAGTCCCCTCCCTGCCACCCCATTCTCCTCCAACCCTAT

>587 len=156 tot-seq=97

TGAGGAATATTGGTCAATGGGCGGAAGCCTGAACCAGCCAAGTCGCGTGAGGGAAGACGG

TCCTATGGATTGTAAACCTCTTTTGCAGGGGAGCAAGGCGCGGTACGTGTACCGCGAAGG

AGAGTACCCTGAGAAAAAGCATCGGCTAACTCCGTG

>588 len=140 tot-seq=96

TGGGGAATATTGCACAATGGGCGCAAGCCTGATGCAGCGACGCCGCGTGAGGGATGACGG

CCTTCGGGTTGTAAACCTCTTTCAGCACAGACGAAGCGCAAGTGACGGTATGTGCAGAAG

AAGGACCGGCCAACTACGTG

>589 len=154 tot-seq=95

TGAGGAATATTGGTCAATGGACGCAAGTCTGAACCAGCCATGCCGCGTGCAGGAAGACGG

CTCTATGAGTTGTAAACTGCTTTTGTACGAGGGTAACAGCATCTACGCGTAGGTGCATGA

AAGTATCGTACGAATAAGGATCGGCTAACTCCGT

>590 len=135 tot-seq=95

TGGGGAATATTGGACAATGGAGGGAACTCTGATCCAGCAATTCTGTGTGTGTGAAGAAGG

TTTTAGGACTGTAAAACACTTTTAGTAGGGAAGAAAAAAATGACGGTACCTACAGAAGAA

GCAACGGCTAAATAC

>591 len=160 tot-seq=95

TGGGGAATATTGGACAATGGGCGAAAGCCTGATCCAGCCATGCCGCGTGTGTGAAGAAGG

TCTTCGGATTGTAAAGCACTTTAAGTTGGGAGGAAGGGCAGTAAGTTAATACCTTGCTGT

TTTGACGTTACCAACAGAATAAGCACCGGCTAACTTCGTG

>592 len=142 tot-seq=95

TAGGGAATCTTCCACAATGGACGCAAGTCTGATGGAGCGACGCCGCGTGCGGGATGAAGG

CCTTCGGGTTGTAAACCGCTTTCAGCAGGGACGAGGCCGCAAGGTGACGGTACCTGCAGA

AGAAGCCCCGGCTAACTACGTG

>593 len=160 tot-seq=95

TGGGGAATATTGCACAATGGGCGCAAGCCTGATGCAGCCATGCCGCGTGTGTGAAGAAGG

CCTTCGGGTTGTAAAGCACTTTCAGCGAGGAGGAAGGGCAGTGTGTTAATAGCACATTGC

ATTGACGTTACTCGCAGAAGAAGCACCGGCTAACTCCGTG

>594 len=137 tot-seq=95

TGGGGAATATTGGGCAATGGGCGCAAGCCTGACCCAGCAACGCCGCGTGAAGGAAGAAGG

CTTTCGGGTTGTAAACTTCTTTTCTCAGGGACGAACAGATGACGGTACCTGAGGAATAAG

CCACGGCTAACTACGTG

>595 len=138 tot-seq=95

TGGGGAATATTGCACAATGGGGGAAACCCTGATGCAGCGACGCCGCGTGAGTGAAGAAGT

ACCTCGGTACGTAAAGCTCTATCAGCAGGGAAGAAAGAAATGACGGTACCTGAGTAAGAA

GCCCCGGCTAACTACGTG

>596 len=161 tot-seq=94

TGGGGAATCTTCCGCAATGGACGAAAGTCTGACGGAGCAACGCCGCGTGAACGAAGAAGG

TCTTCGGATTGTAAAGTTCTGTGATTCGGGACGAAAGGGTTTGTGGTGAATAATCATAGA

CATTGACGGTACCGAAAAAGCAAGCCACGGCTAACTACGTG

>597 len=155 tot-seq=94

TGAGGAATATTGGTCAATGGACGGAAGTCTGAACCAGCCAAGTAGCGTGCAGGATGACGG

CCCTATGGGTTGTAAACTGCTTTTATACGGGGATAAAGTTAGGGACGTGTCCCTATTTGC

AGGTACCGTATGAATAAGGACCGGCTAATTCCGTG

>598 len=157 tot-seq=94

TGAGGAATATTGGTCAATGGGCGAGAGCCTGAACCAGCCAAGTAGCGTGCAGGATGACGG

CCCTATGGGTTGTAAACTGCTTTTATGTGGGGATAAAGTGAGCTACGTGTAGTTTATTGC

AGGTACCACATGAATAAGGACCGGCTAATTCCGTGGG

>599 len=161 tot-seq=93

TGGGGAATCTTCCGCAATGGGCGAAAGCCTGACGGAGCAACGCCGCGTGAGTGAAGAAGG

TCTTCGGATTGTAAAGCTCTGTTGTACATGACGAATGTGCCGGTTGTGAATAATGGCTGG

TAATGACGGTAGTGTACGAGGAAGCCACGGCTAACTACGTG

>600 len=160 tot-seq=93

TAGGGAATTTTCGGCAATGGGGGGAACCCTGACCGAGCAACGCCGCGTGAAGGAGGAAGG

TCTTCGGACTGTAAACTTCTGTTATAAAGGAAGAAAGGCGGATACAGGGAATGGTATCCG

AGTGACGGTACTTTATGAGGAAGCCACGGCTAACTACGTG

>601 len=155 tot-seq=92

TGAGGAATATTGGTCAATGGGCGGGAGCCTGAACCAGCCAAGTAGCGTGAAGGACGACGG

CCCTACGGGTTGTAAACTTCTTTTATAAGGGAATAAAGTTCGCCACGTGTGGTGTTTTGT

ATGTACCTTATGAATAAGCATCGGCTAATTCCGTG

>602 len=135 tot-seq=91

TGGGGAATATTGCACAATGGGCGAAAGCCTGATGCAGCAACGCCGCGTGAGGGAAGACGG

TTTTCGGATTGTAAACCTCTGTCTTTGGTGAAGATAATGACGGTAGCCAAGGAGGAAGCC

ACGGCTAACTACGTG

>603 len=161 tot-seq=91

TGGGGAATATTGCACAATGGGCGCAAGCCTGATGCAGCGACGCCGCGTGAGTGAAGAAGG

TTTTCGGATCGTAAAGCTCTGTTGTTAAGGAAGAACGAGTGTGAGAATGGAAAGTTCATG

CTGTGACGGTACTTAACCAGAAAGGGACGGCTAACTACGTG

>604 len=155 tot-seq=89

TGAGGAATATTGGTCAATGGGCGGGAGCCTGAACCAGCCAAGTCGCGTGAAGGATGACTG

CCCTACGGGTTGTAAACTTCTTTTATTGGGGAATAAAAAAGAGTATGTATACTCTATTGC

ATGTACCTTATGAATAAGCATCGGCTAACTCCGTG

>605 len=149 tot-seq=89

TGGGGGATATTGCACAATGGAGGAAACTCTGATGCAGCGACGCCGCGTGAGTGAAGAAGT

ATTTCGGTATGTAAAGCTCTATCAGCAGGGAAGAAAGGCTCGTAAGAGAGATGACGGTAC

CTGACTAAGAAGCCCCGGCTAACTACGTG

>606 len=138 tot-seq=89

TGGGGAATATTGGGCAATGGGGGAAACCCTGACCCAGCAACGCCGCGTGAGGGAAGAAGG

TTTTCGGATTGTAAACCTCTGTCCTTAGGGACGAAGAAAGTGACGGTACCTAAGGAGGAA

GCTCCGGCTAACTACGTG

>607 len=137 tot-seq=88

TGGGGAATATTGGGCAATGGGGGGAACCCTGACCCAGCAACGCCGCGTGGAGGAAGAAGG

TCTTCGGATCGTAAACTCCTGTCCTAAGAGACGAGGAGAAGACGGTATCTTAGGAGGAAG

CCCCGGCTAACTACGTG

>608 len=160 tot-seq=88

TGGGGAATATTGCGCAATGGGCGAAAGCCTGACGCAGCGACGCCGCGTGAGGGATGAAGG

TTTTCGGATCGTAAACCTCTGTCAGAAGGGAAGAAACTACGTTGTGCTAATCAGCAGCGT

ACTGACGGTACCTTCAAAGGAAGCACCGGCTAACTCCGTG

>609 len=155 tot-seq=88

TGAGGAATATTGGTCAATGGTCGGAAGACTGAACCAGCCAAGTAGCGTGAAGGATGAAGG

TTCTATGGATTGTAAACTTCTTTTATACGGGAATAAAACCTCCCACGTGTTGGAGCTTGT

ATGTACCGTATGAATAAGCATCGGCTAACTCCGTG

>610 len=152 tot-seq=88

TGGGGAATATTGCACAATGGGGGAAACCCTGATGCAGCGACGCCGCGTGAGTGAAGAAGT

ATTTCGGTACGTAAAGCTCTATCAGCAGGGAAGAAACACCGCCCTATGGGCGCTAGACGG

TACCTGACTAAGAAGCCCCGGCTAACTACGTG

>611 len=216 tot-seq=87

CCAAGTGCCCAGATTAAAGGTGTTTAATCCTGCCACCACACTTGGCTTATCTTAACTTCA

TAGAAGAATTTCAAAATTTTGCTAATTGCAGCTCAACAAATCATTTTTATAAGATAAGTA

TCTTAGACCGTTGGCCCTTGTAGAACTACCTAAGGCAGGATAACGTATGAGCTGTCGGAA

GTCCCGCAGGTTGGGTGGAGGTCCAGAGTAAAGGTC

>612 len=160 tot-seq=87

TAGGGAATCTTCGGCAATGGGCGAAAGCCTGACCGAGCAACGCCGCGTGAATGATGAAGG

CCTTCGGGTTGTAAAATTCTGTTATAAGGGAAGAACGACTTTAGTAGGAAATGGCTAGAG

TGTGACGGTACCTTATGAGAAAGCCACGGCTAACTACGTG

>613 len=160 tot-seq=87

TGGGGAATATTGCACAATGGGGGAAACCCTGATGCAGCAACGCCGCGTGAGTGAAGAAGG

GTTTCGGCTCGTAAAGCTCTGTTGGTAGTGAAGAAAGATAGAGGTAGTAACTGGCCTTTA

TTTGACGGTAATTACTTAGAAAGTCACGGCTAACTACGTG

>614 len=154 tot-seq=87

TGGGGAATATTGCGCAATGGGGGAAACCCTGACGCAGCGACGCCGCGTGCGGGATGAAGG

CCTTCGGGTTGTAAACTGCTTTTGTTTGGGAATAATCGACATTACGTGTAATGTTTTGCA

TGTACCATTCGAATAAGGACCGGCTAATTCCGTG

>615 len=155 tot-seq=87

TGAGGAATATTGGTCAATGGACGCAAGTCTGAACCAGCCAAGTAGCGTGCAGGATGACGG

CCCTATGGGTTGTAAACTGCTTTTATATGGGAATAAAGTGAGGGACGTGTCCCTTATTGC

ATGTACCATATGAATAAGGACCGGCTAATTCCGTG

>616 len=135 tot-seq=86

TGAGGAATATTGGTCAATGGGCGAGAGCCTGATGCAGCGACGCCGCGTGAGCGAAGAAGT

ATTTCGGTATGTAAAGCTCTATCAGAAGGGAAGAAAATGACGGTACCTTACTAAGAAGCT

CCGGCTAAATACGTG

>617 len=157 tot-seq=86

TGGGGAATATTGCACAATGGGCGCAAGCCTGATGCAGCGACGCCGCGTGAGGGATGGAGG

CCTTCGGGTTGTAAACCTCTTTCGCCAGTGAAGCAGGCCTGTCCCTTTTGTGGGTGGGTT

GACGGTAGCTGGATAAGAAGCGCCGGCTAACTACGTG

>618 len=135 tot-seq=86

TGGGGGATATTGCACAATGGGGGGAACCCTGATGCAGCGACGCCGCGTGAGTGAAGGAGT

ACTCCGGTATGTAAAGCTCTATCAGCAGGGAAGAAGCAAGACGGTACCTGACCAAGAAGC

CCCGGCTAACTACGT

>619 len=137 tot-seq=86

TGGGGAATATTGGGCAATGGGGGGAACCCTGACCCAGCAACGCCGCGTGAGGGAAGAAGG

TTTTCGGATTGTAAACCTCTGTCCTCCGGGACGAAGAAGTGACGGTACCGGGGGAGGAAG

CTCCGGCTAACTACGTG

>620 len=210 tot-seq=85

TGGGCCCCCTCCCTTCCCAAAATCTAGGTCCTTGTGAAACTGTACCCAATAAAGTACAAT

GGAAACGGCATAGATTTAAGAACCTGGGAGAATGGGAGTCTACTACTTACTTGGTGACTT

GTTCCCTGAACCTAGTCCGAAGACAGAAAGAAGCACCAAGACAACAGTAGACAAAAACAA

GGAGGGCAGGAGTTGTAATACTAAGCAAAG

>621 len=153 tot-seq=85

TGGTGGTGGTAGATGGGGAAGCCTCTCCTCAGGGCACATGCAAGTGCACTGTGCACCTGC

TACTCTGGAAGGTAAGGTTGCTGTCAGTGGCAGCAGTGTCACCAGGGCTGGTGTCACTGC

CAGTAGCTTGCACTTTATCTATAGCAGCAGCAT

>622 len=138 tot-seq=85

TGGGGAATATTGGACAATGGAGGCAACTCTGATCCAGCAATTCTGTGTGTGTGAAGAAGG

TTTTAGGACTGTAAAACACTTTTAGTAGGGAAGAAAGAAATGACGGTACCTACAGAAGAA

GCGACGGCTAAATACGTG

>623 len=216 tot-seq=85

GACCCCCCCCTTGTAGGCCCACCGAGGGCATGTGCACTGCTGGCTGCTCCCCATATCAGA

AACCTGAGACCCAAGGGGCTACTATCTTGGGAACAGGCGGTCAGCCAACCCTGATAAGAG

GTTCCTGCCCGGAGTGTCTCACAAACACCCTGTCATCAGAGGGCTGTGCTGGGTCACGAG

GCAGCCAAACCACTGCCCTGCAGGACTGGGGGTTGT

>624 len=155 tot-seq=85

TGAGGAATATTGGTCAATGGACGAGAGTCTGAACCAGCCAAGTAGCGTGAAGGATGACTG

CCCTATGGGTTGTAAACTTCTTTTATACGGGAATAAAGTGGAGTATGCATACTCCTTTGT

ATGTACCGTATGAATAAGGATCGGCTAACTCCGTG

>625 len=156 tot-seq=84

TGAGGAATATTGGTCAATGGACGAGAGTCTGAACCAGCCAAGTAGCGTGAGGGATGACTG

CCCTATGGGTTGTAAACCTCTTTTATATGGGAATAACAGAGATTACGTGTAGTCTTTTGC

ATGTACCATTATGAATAAGCATCGGCTAACTCCGTG

>626 len=167 tot-seq=83

TGAGGAATATTGGTCAATGGACGAGAGTCTGAACCAGCCAAGTAGCGTGCAGGATGACGG

CCCTATGGGTTGTAAACCGCTTTTGATTGGGAGCAAGCCTTCGGGTGAGTGTACCTTTCG

AATAAGCGCCGGCTAACTACGTGGGGGGAGCCGCGGAACGCGAGATT

>627 len=139 tot-seq=83

TGGGGAATATTGCACAATGGGCGAAAGCCTGATGCAGCGACGCCGCGTGAGGGATGACGG
CCTTCGGGTTGTAAACCTCTTTCAGTAGGGAAGAAGCGAAAGTGACGGTACCTGCAGAAG
AAGCACCGGCTAACTACGT
>628 len=242 tot-seq=83
GGAGGGGGCAGCGCCTGTCTCCCTGGAGAAGAAAGGCTCACCTGAGAAATTCCGTGTGGC
CAGCATGGTGGTGAGGATGGCTCCCTGGGGAGAGACACAGATAAAACCGGGGCTATCCCA
TCAGTGTTCCCTGCTGGCTTCTGGCCAGGATAAGACCCCTGTCCTCCTGCCCAGATGCTC
AGCCTCATTGTCATGAGGCTGACATGACCTCAGCCTTCCAGAGAGGCAAGAGCTCCAGGA
AG
>629 len=160 tot-seq=82
TGGGGAATATTGGACAATGGGCGCAAGCCTGATCCAGCCATACCGCGTGGGTGAAGAAGG
CCTTCGGGTTGTAAAGCCCTTTTGTTGGGAAAGAAAAGCAGTCGGTTAATACCCGGTTGT
TCTGACGGTACCCAAAGAATAAGCACCGGCTAACTTCGTG
>630 len=135 tot-seq=82
TGGGGAATATTGCACAATGGGGGAAACCCTGATGCAGCGATGCCGCGTGGAGGAAGAAGG
TTTTCGGATTGTAAACTCCTGTTGAAGAGGACGATAATGACGGTACTCTTTTAGAAAGCT
CCGGCTAACTACGTG
>631 len=138 tot-seq=82
TGGGGAATATTGCACAATGGGGGGAACCCTGATGCAGCAACGCCGCGTGAGTGAAGAAGG
CATTCGTGTCGTAAAACTCTGTAGTAGGGGAAGAAAGAAATGACAGTACCCTAAAAGAAA
GCCCCGGCTAACTACGTG
>632 len=161 tot-seq=82
TAGGGAATCTTCGGCAATGGGGGCAACCCTGACCGAGCAACGCCGCGTGAGTGAAGAAGG
TTTTCGGATCGTAAAGCTCTGTTGTTAGAGAAGAACGGTAATGGGAGTGGAAAATCCATT
ACGTGACGGTAACTAACCAGAAAGGGACGGCTAACTACGTG
>633 len=161 tot-seq=81
TGGGGAATCTTCCGCAATGGGCGAAAGCCTGACGGAGCAATGCCGCGTGAGTGATGAAGG
AATTCGTTCCGTAAAGCTCTTTTGTTTATGACGAATGTGCAGATTGTAAATAATGATTTG
TAATGACGGTAGTAAACGAATAAGCCACGGCTAACTACGTG

>634 len=132 tot-seq=80

TGGGGAATATTGCACAATGGGGGAAACCCTGATGCAGCGACGCCGCGTGATTTAGAAGGC

CTTCGGGTTGTAAAAATCTTTTCTATGGGAAGAAAATGACAGTACCATAGGAATAAGGAC

CGGCTAATTACG

>635 len=160 tot-seq=80

TGGGGAATTTTGGACAATGGGCGAAAGCCTGATCCAGCCATTCCGCGTGAGTGAAGAAGG

CCTTCGGGTTGTAAAGCTCTTTCGCAAGGAAAGAAAACTTACTTACTAATACTAGGTGAG

GATGACGGTACCTTGATAAGAAGCACCGGCTAACTACGTG

>636 len=155 tot-seq=80

TGAGGAATATTGGTCAATGGCCGAGAGGCTGAACCAGCCAAGTCGCGTGAGGGATGACGG

TCCTATGGATTGTAAACCTCTTTTGTCGGGGAGCAAAGGACGCCACGTGTGGTGTTTTGC

GAGTACCCGAAGAAAAAGCATCGGCTAACTCCGTG

>637 len=156 tot-seq=79

TGAGGAATATTGGTCAATGGGCGGAAGCCTGAACCAGCCAAGTCGCGTGAGGGATTAAGG

CCCTGCGGGTCGTAAACCTCTTTTGCCGGGGAGCAGTGGTCCGGACGCGTCCGGGCCGGG

AGAGTACCCGGAGAAAAAGCATCGGCTAACTCCGTG

>638 len=144 tot-seq=79

TGGGGAATATTGCACAATGGGCGCAAGCCTGATGCAGCGACGCCGCGTGCGGGATGGAGG

CCTTCGGGTTGTAAACCGCTTTTGATCGGGAGCAAGCCCTTCGGGGTGAGTGTACCCTTC

GAATAAGCACCGGCTAACTACGTG

>639 len=117 tot-seq=79

GTGCAAACCTCAGAGGTTGAACGCTTGGGAGCGTTTGGAGAAGGGCAGGGCCAGGACGCA

GGAAGGAATGAAGTGAGAACGTTGGTGAGGCGGGAAGGGAGACACAAAGCCTCATGC

>640 len=221 tot-seq=79

GTGTTAGTCGGGTCTGCAGCTGGGTAGTAACAGAGGAAGCGTTAGGGATGGAAAGCAAAC

TGTCTGCTTTCTAATGACTCAGTCACTGTGGAGAGGTCCTCCCAGTTGGACTGACCACAG

TGGGCTCGCACAGATACTCCTTTTGATTCTTTGTTTCCTGTTCTAGCTGCCGGACCACTG

GGAGAAAGCTGTCATCACTGTACAGCGGCCGGGGAAGATTC

>641 len=155 tot-seq=79

TAGGGAATCTTCCACAATGGACGCAAGTCTGAACCAGCCAAGTAGCGTGCAGGATGACGG

CCCTATGGGTTGTAAACTGCTTTTATATGGGGATAAAGTGGGGAACGTGTTCCCTTTTGC

AGGTACCATATGAATAAGGACCGGCTAATTCCGTG

>642 len=149 tot-seq=78

TCTTAAACAGATAATTTCATCTCTCTGGGACTCACTTTTCTCATTAAAAAAAAACTGGGG

TGGCATCTCCCTTGCTTAATAACAGCCAAAGGTTTCTTCGAGAATTCTGTCAGGTCATAA

ATGGACAAGCCATGTGAATAATGAATTAT

>643 len=160 tot-seq=77

TGGGGAATATTGCGCAATGGGGGGAACCCTGACGCAGCCATGCCGCGTGAATGAAGAAGG

CCTTCGGGTTGTAAAGTTCTTTCGGTATTGAGGAAGGGATGTGTGTTAATAACATACATT

ATTGACGTTAAATACAGAAGAAGCACCGGCTAACTCCGTG

>644 len=135 tot-seq=77

TGGGGAATATTGCGCAATGGGGGAAACCCTGACGCAGCAACGCCGCGTGAGTGAGGAAGT

ATCTCGGTATGTAAAGCTCTATCAGCGGGGAAGAAAGAGGACGGTACCCGACTAAGAAGC

CCCGGCTAACTACGT

>645 len=137 tot-seq=77

TGGGGAATATTGGGCAATGGGCGAAAGCCTGACCCAGCAACGCCGCGTGAAGGAAGAAGG

CCTTCGGGTTGTAAACTTCTTTTAAGAGGGACGAAGAAGTGACGGTACCTCTTGAATAAG

CCACGGCTAACTACGTG

>646 len=135 tot-seq=77

TGGGGAATATTGCACAATGGGGGAAACCCTGATGCAGCGACGCCGCGTGAGCGATGAAGT

ATCTCGGTATGTAAAGCTCTATCAGCAGGGAAGAAAACGACAGTACCTGACTAAGAAGCC

CCGGCTAACTACGTG

>647 len=160 tot-seq=76

TAGGGAATTTTCGGCAATGGGCGAAAGCCTGACCGAGCAATGCCGCGTGAATGAAGAAAT

TCTTCGGAATGTAAAATTCTGTGGTGAGGGAAGAAAGGTATTAGAAGGAAATGGCTAATA

TTTGACGGTACCTTACTAGAAAGCCCCGGCTAACTATGTG

>648 len=161 tot-seq=76

TAGGGAATCTTCGGCAATGGGGGCAACCCTGACCGAGCAACGCCGCGTGAGTGAAGAAGG

TTTTCGGATCGTAAAACTCTGTTGTTAGAGAAGAACGTTAAGTAGAGTGGAAAATTACTT

AAGTGACGGTATCTAACCAGAAAGGGACGGCTAACTACGTG

>649 len=140 tot-seq=76

TGGGGAATCTTGCACAATGGGCGAAAGCCTGATGCAGCGACGCCGCGTGCGGGACGAAGG

CCTTCGGGTTGTAAACCGCTTTCAGCAGGGACGAGGCGAAAGTGACGGTACCTGCAGAAG

AAGCCCCGGCTAACTACGTG

>650 len=142 tot-seq=75

TGGGGAATCTTCCGCAATGGACGAAAGTCTGACGGAGCAACGCCGCGTGAGTGATGACGG

TCTTCGGGTTGTAAACCGCTTTTGATTGGGAGCAAGCTTTCGGGTGAGTGTACCTTTCGA

ATAAGCGCCGGCTAACTACGTG

>651 len=135 tot-seq=75

TGGGGAATCTTCCGCAATGGACGAAAGTCTGACGGAGCAACGCCGCGTGAGCGAAGAAGT

ATTTCGGTATGTAAAGCTCTATCAGAAGGGAAGAAAATGACGGTACCTTACTAAGAAGCT

CCGGCTAAATACGTG

>652 len=155 tot-seq=75

TGAGGAATATTGGTCAATGGCCGTAAGGCTGAACCAGCCAAGTAGCGTGAGGGAAGACTG

CCCTATGGGTTGTAAACCTCTTTTATGCGGGGATAAAGGTGTCCACGTGTGGATGTTTGC

AGGTACCGCATGAATAAGGACCGGCTAATTCCGTG

>653 len=199 tot-seq=75

CTGCATTTGTTAGGGTTGAGCAGTATAAGGGAGGCTCCGTGAGGAAGTCCTTTGTCCCGG

GGAAGTGAAACCAGGAGTGGTTCTCAACTGAAGCTGGACAGAGGGACAGAATGTGAAGGC

GCCTTCGAATCTCTCCATGGCAAGCACATACACAGTGATAAGTAATTGCTAACGGAAGGA

GCCAATGAAAGGCCGACTC

>654 len=155 tot-seq=75

TGAGGAATATTGGTCAATGGGCGCAGGCCTGAACCAGCCAAGTAGCGTGAAGGATGACTG

CCCTATGGGTTGTAAACTTCTTTTATATGGGAATAAAGTTTTCCACGTGTGGAATCTTGT

ATGTACCATATGAATAAGGATCGGCTAACTCCGTG

>655 len=155 tot-seq=75

TGAGGAATATTGGTCAATGGGCGGGAGCCTGAACCAGCCAAGCCGCGTGAGGGAAGAAGG

TACAGCGTATCGTAAACCTCTTTTGTCAGGGAACAAAGGCGGGGACTAGTCCCCGGATGA

GGGTACCTGAAGAAAAAGCATCGGCTAACTCCGTG

>656 len=155 tot-seq=75

TGAGGAATATTGGTCAATGGCCGAGAGGCTGAACCAGCCAAGTCGCGTGAGGGAAGACGG

CCCTACGGGTTGTAAACCTCTTTTGTCGGGGAGCAAAGCGAACCACGTGTGGTTCATTGA

GAGTACCCGAAGAAAAAGCATCGGCTAACTCCGTG

>657 len=139 tot-seq=74

TGGGGAATATTGGACAATGGACCAAAAGTCTGATCCAGCAATTCTGTGTGCACGATGAAG

TTTTTCGGAATGTAAAGTGCTTTCAATTGGGAAGAAGAAAGTGACGGTACCAACAGAAGA

AGTGACGGCTAAATACGTG

>658 len=160 tot-seq=74

TGGGGAATCTTCCGCAATGGGCGAAAGCCTGACGGAGCAATGCCGCGTGAACGAAGAAGG

TCTTCGGATTGTAAAGTTCTGTCCTTATCGAAGAGAGGCAAATGGAGTAACTGCCATATG

CAGGACGGTAGATGAGGAGGAAGCCCCGGCTAACTACGTG

>659 len=135 tot-seq=74

TGGGGAATATTGCACAATGGGGGAAACCCTGATGCAGCGATGCCGCGTGGAGGAAGAAGG

TTTTCGGATTGTAAACTCCTGTCTTAAAGGACGATAATGACGGTACTTTAGGAGGAAGCT

CCGGCTAACTACGTG

>660 len=155 tot-seq=74

TAAGGAATATTGGTCAATGGACGAAAGTCTGAACCAGCCATGCCGCGTGGAGGATGAAGG

TCCTCTGGATTGTAAACTTCTTTTATTTGGGAAGAAACCCACGATTTCTATTGTGGTTGA

CGGTACCAGATGAATAAGCACCGGCTAACTCCGTG

>661 len=141 tot-seq=74

TGGGGAATATTGCACAATGGGCGCAAGCCTGATGCAGCAACGCCGCGTGCGGGATGACGG

CCTTCGGGTTGTAAACCGCTTTCAGCAGGGACGAAGCTTTTTGTGACGGTACCTGCAGAA

GAAGCACCGGCTAACTACGTG

>662 len=160 tot-seq=73

TGGGGAATATTGCACAATGGGCGCAAGCCTGATGCAGCCATGCCGCGTGTGTGAAGAAGG

CCTTCGGGTTGTAAAGCACTTTCAGCGGGGAGGAAGGCGCCAAGGTTAATAACCTTGGCG

ATTGACGTTACCCGCAGAAGAAGCACCGGCTAACTCCGTG

>663 len=160 tot-seq=73

TAGGGAATCTTCCACAATGGGCGCAAGCCTGATGGAGCAACGCCGCGTGAGTGAAGAAGG

TTTTCGGATCGTAAAACTCTGTTGTTAGAGAAGAACAAGGACGTTAGTAACTGAACGTCC

CCTGACGGTATCTAACCAGAAAGCCACGGCTAACTACGTG

>664 len=170 tot-seq=72

GAAGGCATTAAACCCCTTAGGAGTCTTCATCCAGACACAAATCCTGTACTAAGGATGATA

AAGGAGATCTGCACCCTGTACCTTAATAACGCACCAGTGTGGCACAGAGATACACCTATA

AAGCGGTGTGGGCACCTGCACGCACCCAGTGTGTTTACATCACACACAGT

>665 len=119 tot-seq=72

TTGCTTTTGTGCAATGGATGGAATGACTCAGCCTGCCTGTGATTACCCTTTCACTTTCTG

CCTTGCTTCTGAATCCAAGTTCACTTAATTGAGGTGTGTGGGCCCTTGCTCTCGACCCA

>666 len=161 tot-seq=72

TGGGGAATCTTCCGCAATGGACGAAAGTCTGACGGAGCAACGCCGCGTGAATGATGACGG

CCTTCGGGTTGTAAAGTTCTGTTATACGGGACGAATGGCGTAGCGGTCAATACCCGTTAC

GAGTGACGGTACCGTAAGAGAAAGCCACGGCTAACTACGTG

>667 len=138 tot-seq=72

TGGGGAATATTGGGCAATGGGGGCAACCCTGACCCAGCGACGCCGCGTGAGGGAAGACAG

CCTTCGGGTTGTAAACCTCTGTTGCAGGGGAAGAAGGACGTGACGGTACCCTGCGAGGAA

GCTCCGGCTAACTACGTG

>668 len=163 tot-seq=72

TAGGGTGAGGCCTCTGAGGTGGTGGGGAACAGTGTGGAAACCAGGAAGGAGTGCGGAGCT

TGCAGGTGAAGGGAGATCTCGTGTGGTCAGGGAGGGTGACTCGTGCGGGTGGTGGTGTCA

TGAACCGAGATCAATGGCAGGAAGTTCCCGTGTCTTTGTGCAG

>669 len=132 tot-seq=72

GAGGGGTCGGGAGGGGAAGACCGGGGCCAGCTGGAGGCAGAAGTCATCTGACCCAGTCTG

TGGCAAAAAGGGGACCAAGGTCAGCGAGAAAGGAGAGAAGAGGTATGGGATCCGGACAGC

CGAATCGAGAAC

>670 len=161 tot-seq=71

TGGGGAATCTTCCGCAATGGACGAAAGTCTGACGGAGCAACGCCGCGTGAGTGATGACGG

CCTTCGGGTTGTAAAGCTCTGTTAATCGGGACGAATGGTCTTTATGTGAATAATGTAAAG

ATTTGACGGTACCGGAATAGAAAGCCACGGCTAACTACGTG

>671 len=155 tot-seq=71

TGAGGAATATTGGACAATGGGTGAGAGCCTGATCCAGCCATCCCGCGTGAAGGATGACGG

TCCTATGGATTGTAAACTTCTTTTGTACAGGGATAAACCTACTCTCGTGAGGGTAGCTGA

AGGTACTGTACGAATAAGCACCGGCTAACTCCGTG

>672 len=137 tot-seq=71

TGGGGAATATTGGGCAATGGGGGAAACCCTGACCCAGCAACGCCGCGTGAAGGAAGAAGG

CCTTCGGGTTGTAAACTTCTTTTACCAGGGACGAATAAATGACGGTACCTGGAGAAAAAG

CAACGGCTAACTACGTG

>673 len=135 tot-seq=71

TGGGGAATATTGCACAATGGGGGAAACCCTGATGCAGCGACGCCGCGTGAGTGAAGAAGT

AATTCGTTACGTAAAGCTCTATCAGCAGGGAAGAAGAATGACGGTACCTGATTAAGAAGC

CCCGGCTAACTACGT

>674 len=160 tot-seq=70

TAGGGAATCTTCCGCAATGGGCGAAAGCCTGACGGAGCAACGCCGCGTGAGTGATGAAGG

TCTTCGGATCGTAAAACTCTGTTATCAGGGAAGAACAAACGTGTAAGTAACTGTGCACGT

CTTGACGGTACCTGATCAGAAAGCCACGGCTAACTACGTG

>675 len=137 tot-seq=70

TGGGGAATATTGCACAATGGGCGAAAGCCTGATGCAGCAACGCCGCGTGAAGGAAGAAGG

GTTTCGGCTCGTAAACTTCTATCAACAGGGACGAAAGAAATGACGGTACCTGAATAAGAA

GCCCCGGCTAACTACGT

>676 len=134 tot-seq=70

TAGGGAATCTTCGGCAATGGGGGGAACCCTGATGCAGCGACGCCGCGTGATTTAGAAGGC

CTTCGGGTTGTAAAAATCTTTTGTATGGGAAGAAAATGACAGTACCATACGAATAAGGAC

CGGCTAATTACGTG

>677 len=138 tot-seq=70

TGGGGAATATTGGACAATGGGGGAAACCCTGATCCAGCGACGCCGCGTGAGTGAAGAAGT

ATTTCGGTATGTAAAGCTCTATCAGCAGGGAAGAAAAAAATGACGGTACCTGACTAAGAA

GCCCCGGCTAACTACGTG

>678 len=137 tot-seq=68

TGAGGAATTTTCCGCAATGGGCGAGAGCCTGACGGAGCAATGCCGCGTGGAGGATGAAGG

CCCGTGGGTTGTAAACTCCTTTTCTTAGAGAAGAAGCCCTGACGGTATCTAAGGAATAAG

CATCGGCTAACTCCGTG

>679 len=162 tot-seq=68

GGTCCGTTTAACCCCAGCCTGACCTCCTTGTTCAACACCATCACCCGCCCCAGCTCCTAC

TTGGCCAAACTCACACTGGCACAGAGCGCAGTGGGGAAGGCTGGAGCGAAAACTGGAGAG

GCCAAGGAAGGCTTCCAGTGCTGCAGGCTTCTAGGATCTACA

>680 len=140 tot-seq=67

TGGGGAATATTGCACAATGGGCGAAAGCCTGATGCAGCGACGCCGCGTGGGGGATGACGG

TCTTCGGATTGTAAACCCCTTTCAGTAGGGACGAAGCGCAAGTGACGGTACCTGCAGAAG

AAGCACCGGCCAACTACGTG

>681 len=160 tot-seq=67

TGGGGAATCTTCCGCAATGGGCGAAAGCCTGACGGAGCAATGCCGCGTGAGTGAAGAAGG

CCTTCGGGTTGTAAAACTCTGTCCTAGACGAAGAGAGAGTATTGTAGTAACTGACAATAC

TAGGACGGTAGACTAGGAGGAAGCTCCGGCTAACTATGTG

>682 len=161 tot-seq=67

TAGGGAATTTTCCACAATGGACGAAAGTCTGATGGAGCAACGCCGCGTGAACGATGAAGG

TCTTCGGATTGTAAAGTTCTGTTGTCAGGGACGAACACGTGCCGTTCGAATAGGGCGGTA

CCTTGACGGTACCTGACGAGAAAGCCACGGCTAACTACGTG

>683 len=184 tot-seq=67

GAGGAGTCAGCACATCTGTGAGAGAGTGAAGATCCCATGCAGGTTGGAACGGCTGCTGCC

AAAAGTGAGCGGAGCTCAGAAGGGCAGGAATGCGCAGCCATGGCTGCCTCTTGAGGGCGA

GGGTAGGAGGCTGTGCATTTCTTCAGCAGCACACAGCGTGGCTGTGCGGTTCCCCCCACT

GCCA

>684 len=160 tot-seq=67

TGGGGAATATTGCGCAATGGGGGAAACCCTGACGCAGCGACGCCGCGTGCGGGATGAAGG

CCTTCGGGTTGTAAAGCTCTGTTGTTGGTGAAGAAGGACAGGGGTAGTAACTGACCTTTG

TTTGACGGTAATCAATTAGAAAGTCACGGCTAACTACGTG

>685 len=138 tot-seq=66

TGGGGAATATTGGACAATGGAGGTAACTCTGATCCAGCAATTCTGTGTGCACGAAGAAGG

TTTTCGGATTGTAAAGTGCTTTCAGTAGGGAAGAAGAAAGTGACGGTACCTACAGAAGAA

GCGACGGCTAAATACGTG

>686 len=107 tot-seq=66

TAGATGACACACTGGACGAGGTCCCAGAAGATCCCGTTGAGGCGCCAGGCTGTCCAGATG

TGGAGGCAGGTGCTGAGGTTGCATTAGCGGCTTCCCTCAGGCCACTC

>687 len=160 tot-seq=66

TGGGGAATATTGCACAATGGGCGCAAGCCTGATGCAGCCATGCCGCGTGTGTGAAGAAGG

CCTTCGGGTTGTAAAGCACTTTCAGTCAGGAGGAAAGCGTGGTGGTTAATACCCATCATG

TGTGACGTTACTGACAGAAGAAGCACCGGCTAACTCCGTG

>688 len=144 tot-seq=66

TGGGGAATATTGCACAATGGGCGCAAGCCTGATGCAGCGACGCCGCGTGGGGGATGAAGG

CCTTCGGGTTGTAAACTCCTTTCGCTAGGGAAGAAGCCACTTGTGGTGACGGTACCTGGA

TAAGAAGCACCGGCTAACTACGTG

>689 len=160 tot-seq=66

TGGGGAATTTTGGACAATGGGGGCAACCCTGATCCAGCCATGCCGCGTGCGGGAAGAAGG

CCTTCGGGTTGTAAACCGCTTTTGTCAGGGAAGAAATCCTTTGGGCTAATAACTCGGAGG

GATGACGGTACCTGAAGAATAAGCACCGGCTAACTACGTG

>690 len=143 tot-seq=65

TAGGGAATCTTCCACAATGGACGCAAGTCTGATGGAGCAACGCCGCGTGAGTGAAGAAGG

GTTTCGGGTTGTAAACCGCTTTTGATTGGGAGCAAGCTTTCGGGTGAGTGTACCTTTCGA

ATAAGCGCCGGCTAACTACGTGG

>691 len=161 tot-seq=65

TAGGGAATCTTCCGCAATGGACGAAAGTCTGACGGAGCGACGCCGCGTGAGCGAAGAAGG

TCTTCGGATCGTAAAGCTCTGTTGTTAGGGAAGAAGAAGTACCGTTCGAATAGGGCGGTA

CGGTGACGGTACCTAACGAGAAAGCCCCGGCTAACTACGTG

>692 len=160 tot-seq=65

TAGGGAATCTTCCGCAATGGACGAAAGTCTGACGGAGCAACGCCGCGTGAGCGAAGAAGG

CCTTCGGGTCGTAAAGCTCTGTTGTTAGGGAAGAACAAGTAACGCAGTAACTGGCGTTAC

CGTGACGGTACCTAACGAGAAAGCCACGGCTAACTACGTG

>693 len=138 tot-seq=65

TGGGGAATATTGGGCAATGGGCGCAAGCCTGACCCAGCAACGCCGCGTGAAGGAAGAAGG

CCCTCGGGTTGTAAACTTCTTTTATTCGGGACGAAACAAATGACGGTACCGAATGAATAA

GCCACGGCTAACTACGTG

>694 len=137 tot-seq=64

TGGGGAATATTGGGCAATGGAGGCAACTCTGACCCAGCAATGCCGCGTGAGTGATGAAGG

TAATTTTATTGTAAAGCTCTTTTCTTTGGAACGAATAAATGACGGTACCAAAGGAATAAG

CCCCGGCTAACTACGTG

>695 len=142 tot-seq=64

TGGGGAATTTTGGACAATGGGGGCAACCCTGATCCAGCCATGCCGCGTGCGGGATGAAGG

CCTTCGGGTTGTAAACCGCTTTTGATTGGGAGCAAGCTTTCGGGTGAGTGTACCTTTCGA

ATAAGCGCCGGCTAACTACGTG

>696 len=144 tot-seq=64

TGGGGAATATTGCACAATGGGCGCAAGCCTGATGCAGCGACGCCGCGTGAGGGATGACGG

CCTTCGGGTTGTAAACCTCTTTCAGCAGGGAAGCAGCCCCTCGGGGTGACGGTACCTGCA

GAAGAAGCGCCGGCTAACTACGTG

>697 len=155 tot-seq=64

TGAGGAATATTGGTCAATGGCCGAAGGGCTGAACCAGCCAAGTCGCGTGAGGGAAGACGG

CCCTACGGGTTGTAAACCTCTTTTGCCGGGGAGCAAAGGCGGTCACTGGTGACCGGATGA

GAGTACCCGGAGAAAAAGCATCGGCTAACTCCGTG

>698 len=160 tot-seq=63

TAGGGAATCTTCCGCAATGGGCGCAAGCCTGACGGAGCAACGCCGCGTGTGTGAAGAAGG

TCTTCGGATCGTAAAGCACTGTTGTTAGAGAAGAACGACCGCTAGAGTAACTGTTAGCGG

AGTGACGGTATCTAACCAGAAAGCCACGGCTAACTACGTG

>699 len=238 tot-seq=63

ACCTTACGGGGCCGGGGGGAAGCGGGGAGCAGTCAGGGCTGTCAGGGACACAGGCTCAAG

CAGAAGGTGCGGCTGACTGAGAGAGGGAGGACTTGGGGGCTGTGCCTTTATTGGGGTCCA

TGGGTGTTTTCCCCTAGGCCTTCCCTGTGCAGGTGTGGATGGGCGACTGTAAAGAAACCA

CGGCAAAGCGAGGAACTTTGTTATAGTTTCTGGTGCTGACCAGGAGGCTTCACCACCA

>700 len=191 tot-seq=62

GCCAGTGCTCTACCACTGTGCCCTACATTTCTAGCTCTCGGCCTCCCTTACAGGCTTCTG

TGGATCTCACATGCTCATAACAGCTCACGGCTAGCCACCTTTTAAAGGCAGAGATTGAGA

ATGTGAGAGTCAACCACTAGGTCACAAGAAGGCTGAATCCCAGCCGGGCGTGGTGGCGCA

CACCTTTAATC

>701 len=155 tot-seq=62

TGAGGAATATTGGTCAATGGGCGAGAGCCTGAACCAGCCAAGTAGCGTGAGGGAAGACTG

CCCTATGGGTTGTAAACCTCTTTTGTGCGGGGATAAAGTGAGGGACGTGTCCCTCATTGC

AGGTACCGCACGAATAAGGACCGGCTAATTCCGTG

>702 len=155 tot-seq=62

TGAGGAATATTGGTCAATGGGCGAGAGCCTGAACCAGCCAAGTCGCGTGAAGGATGACTG

CCCTATGGGTTGTAAACTTCTTTTACAGGGGAATAAAATGAGATACGTGTATTTTATTGC

ATGTACCTTGTGAATAAGCATCGGCTAACTCCGTG

>703 len=135 tot-seq=62

TGGGGAATATTGGACAATGGGCGCAAGCCTGATCCAGCCATGCCGCGTGAGTGATGACGG

CCTTAGGGTTGTAAAGCTCTTTCGACGGGGACGATAATGACGGTACCCGTAGAAGAAGCC

CCGGCTAACTTCGTG

>704 len=112 tot-seq=61

GCGTGGATTACATCTCCACCAGCCGCGCGCACCAACACAAGTGCGCGCTGCTCAGCCGCG

AGATCCCGGCCATCACCTACCGCGGTGGTGGCACCTATACCAAGGGCGCCTT

>705 len=222 tot-seq=61

ACGACCTGCTCTGGAATCGCAGAGGGAGCATCTAATGGAAGACCCTGGAGGGCTCCCTGT

AAGGTGGGCACTGAGGCTTTTGGTGCAGGGCACGGAGGGCGGGGCAGAGTGGAGCACAGG

CAGAGGGTATAGGTGAGCAGAGGCCGGAAGCCAGACCCTCCTGGATGCCCCTGGACATGC

CCAGACCTGGAGGTGTTGAGGGGGGCAAGAGAGGTGCCCCAG

>706 len=156 tot-seq=61

TGAGGAATATTGGTCAATGGACGAAAGTCTGAACCAGCCAAGTAGCGTGCAGGATGACGG

CCCTCCGGGTTGTAAACTGCTTTAGTTCGGGAATAACGACGGGGACGTGTCCCTGGAAAG

AATGTACCGAAAGAATAAGGACCGGCTAATTCCGTG

>707 len=127 tot-seq=61

CAAGGGCTAGCAGAGGGACACTGCCAGGAAGTGAGAACTTGGGGCAACAGGTGGCCCTGA

GTATGGGTAGAAATATCTGACCCCACCCAGGGCTGTGACCGAGGTCGGACGGAAGTGCAG

GGAAAGA

>708 len=161 tot-seq=61

TGGGGAATATTGCACAATGGGCGCAAGCCTGATGCAGCAACGCCGCGTGAGTGAAGAAGG

TTTTCGGATCGTAAAGCTCTGTTGTAAGAGAAGAACGAGTGTGAGAGTGGAAAGTTCACA

CTGTGACGGTATCTTACCAGAAAGGGACGGCTAACTACGTG

>709 len=160 tot-seq=61

TGGGGAATATTGCACAATGGGGGAAACCCTGATGCAGCCATGCCGCGTGTGTGAAGAAGG

CCTTCGGGTTGTAAAGCACTTTCAGCGAGGAGGAAAGGTTGATGCCTAATACGCATCAGC

TGTGACGTTACTCGCAGAAGAAGCACCGGCTAACTCCGTG

>710 len=135 tot-seq=61

TGGGGAATATTGGACAATGGGCGCAAGCCTGATCCAGCCATGCCGCGTGAGTGATGAAGG

CCCTAGGGTTGTAAAGCTCTTTTAACGGGGAAGATAATGACGGTACCCGTAGAATAAGCC

CCGGCTAACTTCGTG

>711 len=136 tot-seq=61

GGAAGAAAATTGGCCAATCCTAATTCAGGGAGCCAGTGACAAGAAATAGCAAGCTGGGAA

ACTTAGTTTCTACGGCATTGAAATGAGAAAAGTGTAAATCTCTTAGCGAGGAACAATTGG

AGGGCAAGTCATGGTG

>712 len=160 tot-seq=60

TGGGGAATATTCCACAATGGGCGCAAGCCTGATGGAGCGACGCCGCGTGAGTGAAGAAGG

GTTTCGGCTCGTAAAGCTCTGTTGTTGAAGAAGAACGTGCGTGAGAGCAACTGTTCACGC

AGTGACGGTATTCAACCAGAAAGTCACGGCTAACTACGTG

>713 len=134 tot-seq=60

GCGCGCAAATTACCCAATCCTGACACGGGGAGGTAGTGACAATAAATAACAATACCGGGC

TCTACGAGTCTGGTAATTGGAATGAGTACAATCTAAATCCCTTAACGAGGATCCATTGGA

GGGCAAGTCTGGTG

>714 len=135 tot-seq=60

GCGCGCAAATTACCCAATCCCGACACGGGGAGGTAGTGACAATAAATACTGATACAGGGC

TCTTTTGGGTCTTGTAATTGGAATGAGTACAATTTAAATCCCTTAACGAGGAACAATTGG

AGGGCAAGTCTGGTG

>715 len=120 tot-seq=60

CCACTTCTGACAGGGAGGTAGAGAGGAAGTTCCAAGTTTCCCAGCAGGCAAAGGTGGCCC

TGGGCTCACAGTCAAGAGCTGCCCTACTCAGGTCCTACCTGCACGATGTCCAACACCATG

>716 len=172 tot-seq=60

TAGGGAATCTTCCACAATGGACGCAAGTCTGATGGAGCAACGCCGCGTGAGTGAAGAAGG

GTTTCGGCTCGTAAAGCTCTGTTGTTGGTGAAGAAGGACAGGGGTAGTAACTGGCCTTTA

TTTGACGGTAATCAACCAGAAAGTCACGGCTAACTACGTGGGGGGGCCGCGG

>717 len=138 tot-seq=60

TGGGGAATATTGCACAATGGGGGAAACCCTGATGCAGCAACGCCGCGTGAACGATGAAGG

CCAAAAGGTCGTAAAGTTCTGTCCTTGGGGAAGAAACAAATGACGGTACCCTTGGAGGAA

GCCCCGGCTAAATACGTG

>718 len=160 tot-seq=59

TAGGGAATCTTCCACAATGGGCGCAAGCCTGATGGAGCAACACCGCGTGAGTGAAGAAGG

GTTTCGGCTCGTAAAACCCTGTTGTTAGAGAAGAAAGTGCGTGAGAGTAACTGTTCACGT

TTCGACGGTATCTAACCAGAAAGCCACGGCTAACTACGTG

>719 len=160 tot-seq=59

TAGGGAATCTTCCACAATGGGCGAAAGCCTGATGGAGCAACGCCGCGTGTGTGATGAAGG

CTTTCGGGTCGTAAAGCACTGTTGTATGGGAAGAACAGCTAGAATAGGAAATGATTTTAG

TTTGACGGTACCATACCAGAAAGGGACGGCTAAATACGTG

>720 len=135 tot-seq=59

TAGGGAATCTTCCACAATGGGCGCAAGCCTGATGCAGCAACGCCGCGTGAACGATGAAGG

TCTTCGGATCGTAAAGTTCTGTTGCAGGGGAAGATAATGACGGTACCCTGTGAGGAAGCC

CCGGCTAACTACGTG

>721 len=135 tot-seq=59

TGGGGAATATTGGACAATGGGCGAAAGCCTGATCCAGCGACGCCGCGTGAGCGATGAAGT

ATTTCGGTATGTAAAGCTCTATCAGCAGGGAAGAAAACGACGGTACCTGACTAAGAAGCC

CCGGCTAACTACGTG

>722 len=160 tot-seq=59

TGGGGAATTTTGGACAATGGGCGCAAGCCTGATCCAGCAATGCCGCGTGCAGGATGAAGG

CCTTCGGGTTGTAAACTGCTTTTGTACGGAACGAAACGGCGAGCTCTAATACAGTTTGCT

AATGACGGTACCGTAAGAATAAGCACCGGCTAACTACGTG

>723 len=180 tot-seq=59

GCGCGCAAATTACCCACTCCCGACCCGGGGAGGTAGTGACGAAAAATAATACAGGACTCT

TTCGAGGCCCTGTAATTGGAATGAGTCCACTTTAAATCCTTTAACGAGGATCCATTGGAG

GGCAAGTCTGGTGCCAGCAGCCGCGGTAAACATAGATCGGAAGACGTCGTGTAGGGAAAG

>724 len=135 tot-seq=59

TGGGGAATATTGCACAATGGGGGGAACCCTGATGCAGCGACGCCGCGTGAGCGAAGAAGG

GATTCGTTTCGTAAAGCTCTGTCCTATGGGAAGATAATGACGGTACCATAGAAGAAAGCC

CCGGCTAACTACGTG

>725 len=154 tot-seq=58

ATTTAATTATTTAACTCAGCTCTGAGTCAGAGATGGCCCAAGCATCAGAGCCCAAGCCAA

TGCTGTCAGGGGTGAAATTACAAAATGATCCCCTTAGGCCCTGACCACATCATCCCTGAG

TTATTAAGACCCAAAGCAGAAAATCAGAAATTCT

>726 len=157 tot-seq=58

TGAGGAATATTGGTCAATGGAGGAAACTCTGAACCAGCCACGCCGCGTGCAGGAAGAATG

CCTTACGGGTTGTAAACTGCTTTTGTTTAGGAATAAAAATTCTTACGTGATAAGAATTAT

GAATGTACTATACGAATAAGTGTCGGCAAACTCCGTG

>727 len=104 tot-seq=58

GAGCGGATGGAGTCGCTGAGGCCCATCCATTGCTGGTAGTCAGGGTACTCCCCACGCCGC

AGGAAGTACTGGTGGCCCTGGTAGTTGGGGCGCTCATAGATCAT

>728 len=210 tot-seq=58

TCAAAAAAAAAAAAAAAACAAATGAAAAAGAAACCCACTTCAATCCATCATGTGGCTGGG
AGCTCACATAGCAGCCTAGTCTCAGGCAGCATGACTTCCATGGCAGCAGGAATGAGTGAC
CTGTCTTTAGCTCCAGTCTCCAACTCAGGAATACCCATCTCAGACATTCAGTTTCATATG
TCCTGAGACAGCCTTTGCCCTGTATATTTC
>729 len=141 tot-seq=58
TGGGGAATATTGCACAATGGGCGGAAGCCTGATGCAGCGACGCCGCGTGGGGGATGACGG
CCTTCGGGTTGTAAACTCCTTTCGACAGGGACGAAGCTTTTTGTGACGGTACCTGTATAA
GAAGCACCGGCTAACTACGTG
>730 len=138 tot-seq=57
TAGGGAATCTTGCACAATGGGCGAAAGCCTGATGCAGCAACGCCGCGTGAAGGATGAAGG
CCTTCGGGTCGTAAACTTCTGTTCTTGGGGAAGAAACAAATGACGGTACCCAAGGAGCAA
GCCCCGGCTAACTACGTG
>731 len=160 tot-seq=57
TAGGGAATCTTCCACAATGGGCGAAAGCCTGATGGAGCAACGCCGCGTGGGTGAAGAAGG
TCTTCGGGTTGTAAAGTACTTTCAGCGGGGAGGAAGGGAGTAAAGTTAATACCTTTGCTC
ATTGACGTTACCCGCAGAAGAAGCACCGGCTAACTCCGTG
>732 len=137 tot-seq=57
TCGGGAATATTGCGCAATGGAGGAAACTCTGACGCAGTGACGCCGCGTATGGGAAGAAGG
TTTTCGGATTGTAAACCATTGTCGTTAGGGAAGAAGAAGTGACAGTACCTAAGGAGGAAG
CTCCGGCTAACTATGTG
>733 len=231 tot-seq=57
GGCGCAGCTGAGCGTTGGCAACGTCGCTGCCAATTAAAGGGCTCCCTGCTGTGGGGGCTG
ATCCAGGGTGGTTGTCGAGGGGGGCGGTGGAGAGAATGGAATCATTAAAATGGGAGAACA
AAGGAGGTAGGACAAGTTCAAATGAGATTAAGGCACAAAGGGAAAGGCTGCCTTTCTGGG
GCATTTATGCCTAGGGACAGCTGGGTGCCTCTGGCAAAATTTACCTGTAGC
>734 len=156 tot-seq=57
TGAGGAATATTGGTCAATGGGCGCGAGCCTGAACCAGCCAAGTCGCGTGAGGGAGGACGG
CCCTACGGGTTGTAAACCTCTTTTGCCGGGGAGCAACGGGCGCCACGTGTGGCGCCACTG
AGAGTACCCGGAGAAAAAGCATCGGCTAACTCCGTG

>735 len=137 tot-seq=57

TGGGGAATATTGGGCAATGGGCGCAAGCCTGACCCAGCAACGCCGCGTGAAGGAAGAAGG

CCCTCGGGTTGTAAACTTCTTTTATCAGGGACGAAGAAATGACGGTACCTGATGAATAAG

CCACGGCTAACTACGTG

>736 len=140 tot-seq=57

TGGGGAATATTGCACAATGGGCGAAAGCCTGATGCAGCGACGCCGCGTGAGGGATGACGG

CCTTCGGGTTGTAAACCTCTTTCAGCAGGGAAGAAGCTTTTGTGACGGTACCTGCAGAAG

AAGCACCGGCTAACTACGTG

>737 len=155 tot-seq=57

TGAGGAATATTGGTCAATGGGCGAGAGCCTGAACCAGCCAAGTAGCGTGCAGGATGACGG

CCCTATGGGTTGTAAACTGCTTTTATAAGGGAATAAAGTGGGTCACGTGTGACTTTTTGC

ATGTACCTTATGAATAAGGACCGGCTAATTCCGTG

>738 len=185 tot-seq=56

TGGGGGATATTGCACAATGGGGGGAACCCTGATGCAGCGATGCCGCGTGGAGGAAGAAGG

TTTTCGGATTGTAAACTCCTGTCCTGAAGGACGATAATGACGGTACTTCAGGAGGAAGCT

CCGGCTAACTATGTGGAAGGACGATAATGACGGTACTTCAGGAGGAAGCTCCGGCTAACT

ATGTG

>739 len=216 tot-seq=56

TGGGGAATATTGCACAATGGGCGAAAGCCTGACGCAGCAACGCCGCGTGAGTGAAGACGG

CCTTCGGGTTGTAAAACTCTGTGATTCGGGACGAAAGATAAGTAGACGAATAATCTGCAT

AAGTGACGGTACCGAAAAAGCAAGCCACGGCTAACTACGTGGGGGGAGCTGCGGTAATTC

CAAGATCGGAAGAGCGTCGTGTAGGGAAAGAGGTGG

>740 len=160 tot-seq=56

TAGGGAATTTTCGGCAATGGGCGCAAGCCTGACCGAGCAACGCCGCGTGAGTGAAGAAGG

CCTTCGGGTTGTAAAGCTCTGTTGTAAGGGAAGAACACTTAAAAGAGGGAATGCTTTTAA

GCTGACGGTACCTTACCAGAAAGCCACGGCTAACTACGTG

>741 len=156 tot-seq=56

TGAGGAATATTGGTCAATGGGCGGAAGCCTGAACCAGCCATGCCGCGTGCGGGAAGGAGG

CCCTATGGGCTGTGAACCGCTTTTGCCTGGGGGCAATAAGGGCGTCGCGCACGTCCGATG

AGAGTACCAGGCGAATAAGCATCGGCTAACTCCGTG

>742 len=180 tot-seq=56

AAGGCACAGTCAGCAAGAGGCCCAGCATCCAGGAGGAGGCCTGGACCACACGCACCCCAA

ATGTTCGATGATCTGCACCCTTCCAGGAGGGCCTGAAAGCCAGTAGGAAGAGGTCTCCGC

TGAGGCCGGTCAGGAGCAGCACACTGGCATACATAGACAGAATGGTGATGGAGGACAACA

>743 len=161 tot-seq=56

TGAGGAATATTGGTCAATGGACGGAAGTCTGACCGAGCAACGCCGCGTGAGTGAAGAAGG

TTTTCGGATCGTAAAGCTCTGTTGTAAGAGAAGAACGAGTGTGAGAGTGGAAAGTTCACA

CTGTGACGGTATCTTACCAGAAAGGGACGGCTAACTACGTG

>744 len=155 tot-seq=56

TGGGGAATATTGCGCAATGGGGGAAACCCTGACGCAGCCAAGTAGCGTGCAGGATGACGG

CCCTATGGGTTGTAAACTGCTTTTATATGGGAATAAAGTGAGGGACGTGTCCCTTATTGC

ATGTACCATACGAATAAGGACCGGCTAATTCCGTG

>745 len=180 tot-seq=55

TGGGGAATATTGCACAATGGGGGAAACCCTGATGCAGCAACGCCGCGTGAACGATGAAGG

CCTTTGGGTCGTAAAGTTCTGTTCTAGGTGATGAAAGCTGACAGTAACCTAGGAGAAAGC

CCCGGCTAACTCCGTGCCAGCAGCCGCGGTATCCTAAGATCGGAAGAGCGTCGTGTTGGG

>746 len=135 tot-seq=55

TAAGGAATATTCCACAATGGGGGAAACCCTGATGGAGCAATACCGCGTGCAGGAAGACGC

TTTTCGGAGTGTAAACTGCTTTTATCTACCAAGATTATGACGGTAGTAGATGAATAAGGA

CCGGCTAACTACGTG

>747 len=138 tot-seq=55

TGGGGAATATTGGGCAATGGGCGAAAGCCTGACCCAGCAACGCCGCGTGAGGGAAGAAGG

CCTTCGGGTTGTAAACCTCTGTCCTTAGTGACGAAGGAAGTGACGGTAGCTAAGGAGGAA

GCCCCGGCTAACTACGTG

>748 len=222 tot-seq=55

TCCTGCTGGGACACAGCCCAGGCGGAATCCCCCTTTCTTTCTCCTGGACCAATCAGATGT

GAGTCAGGGACCCTCTCATTAACAAATTTCCTCCCTTCCTCCTCCCCTGTTATGAGGCCA

GGAGCGGGCGTTGACCTACATGGAAGGGGAGGAGAGGTGGAACCAGTTGGAGCAAATACC

CAACCTGCCTCTTGCCTGGTAAGGTCACCCAGAAGCCTTGAG

>749 len=160 tot-seq=55

TGGGGAATATTGCACAATGGGCGCAAGCCTGATGCAGCAACGCCGCGTGAGTGAAGAAGG

GTTTCGGCTCGTAAAGCTCTGTTGTTGGTGAAGAAGGACAGGGGTAGTAACTGACCTTTG

TTTGACGGTAATCAATTAGAAAGTCACGGCTAACTACGTG

>750 len=156 tot-seq=54

CTGGAATTACCACAGGACCTGACTGTTGGTTGTAGGACCCAGATGGCAGAAGCCTGAAAA

TGTTTCTGGCAAATACCTCCACAAGCCGGAGTGTGTGTGTGTGTGTGTGTGCTAGCATGC

ATGCTCCTCCTTTTTATTGGCACTTTAGTTAAATGC

>751 len=224 tot-seq=54

AAGGTCCTCCAGAAAATAATAGCCTAGGCTACCCATCTGCCAAACTCATGAGCTGCCCCA

CACCTGTGCTTTTGGCTCTATGCAAACATTTAATACGATTCCAAGTTGGGTCAGGGGCAC

ATGCAAAAATTCAGGGCTCCATGGGCCTGCTGAAAGCAATTCAGGACGATCCCACAGCCT

GTAGTGCAGGCTCTTACAGGTCAGACAGGAAGGGAGGAAGAGCT

>752 len=141 tot-seq=54

TGGGGAATATTGCACAATGGGCGGAAGCCTGATGCAGCGACGCCGCGTGGGGGATGACGG

CCTTCGGGTTGTAAACCTCTTTCGCTAGGGACGAAGCTTTTGTGACGGTACCTAGATAAG

AAGCACCGGCTAACTACGTGG

>753 len=155 tot-seq=54

TGAGGAATATTGGTCAATGGGCGAAGGCCTGAACCAGCCAAGTAGCGTGCAGGATGACGG

CCCTATGGGTTGTAAACTGCTTTTATGCGGGGATAAATGACGCCACGTGTGGTGTTTTGC

AGGTACCGCATGAATAAGGACCGGCTAATTCCGTG

>754 len=160 tot-seq=54

TAGGGAATCTTCCACAATGGACGCAAGTCTGATGGAGCAACGCCGCGTGAGTGAAGAAGG

TTTTCGGATCGTAAAGCTCTGTTGTTAGAGAAGAAAGTGCGTGAGAGTAACTGTTCACGT

TTCGACGGTATCTAACCAGAAAGCCACGGCTAACTACGTG

>755 len=160 tot-seq=54

TAGGGAATCTTCCACAATGGACGCAAGTCTGATGGAGCAACGCCGCGTGAGCGAAGAAGG

TCTTCGGATCGTAAAACTCTGTTGTTAGAGAAGAACACGGGTGAGAGTAACTGTTCACCT

GTTGACGGTATCTAACCAGCAAGTCACGGCTAACTACGTG

>756 len=155 tot-seq=53

TGAGGAATATTGGTCAATGGACGAGAGTCTGAACCAGCCAAGTCGCGTGAAGGATGACTG

CCCTATGGGTTGTAAACTTCTTTTATGTGGGAATAAAAAGAGTATGTATACTCTATTGC

ATGTACCATAATGAATAAGCATCGGCTAACTCCGT

>757 len=135 tot-seq=53

TGGGGAATATTGCACAATGGGGGAAACCCTGATGCAGCGATGCCGCGTGGAGGAAGAAGG

TTTTCGGATTGTAAACTCCTTTTATCAGGGACGATAATGACGGTACCTGAAGAAAAAGCT

CCGGCTAACTACGTG

>758 len=155 tot-seq=53

TGAGGAATATTGGTCAATGGGCGGAAGCCTGAACCAGCCAAGTAGCGTGAAGGATGACTG

CCCTATGGGTTGTAAACTTCTTTTATAAAGGAATAAGATCACCTACGTGTAGGTGCGTGT

ATGTACTTTATGAATAAGCATCGGCTAACTCCGTG

>759 len=117 tot-seq=53

TAGGGAATCTTCCACAATGGACGCAAGCCTGATGGAGCAACGCCGCGTGAGTGAAGAAGG

TTTTCGGATCGTAAAGCTCTGTTGTTGGTGAAGAAGGATAGAGGTAGTAACTGGCCT

>760 len=138 tot-seq=53

TGGGGAATATTGGGCAATGGGCGCAAGCCTGACCCAGCAACGCCGCGTGAGGGAAGAAGG

TTTTCGGATTGTAAACCTCTGTCGCAGAAGACGAAGGAAGTGACGGTATTCTGTGAGGAA

GCCCCGGCTAACTACGTG

>761 len=155 tot-seq=53

TGAGGAATATTGGTCAATGGGAGAGATCCTGAACCAGCCAAGTCGCGTGAGGGAAGACGG

TCCTATGGATTGTAAACCTCTTTTGCCGGGGAGCAAGCTGTGCCACGTGTGGCGCAGCTG

AGAGTACCCGGAGAAAAAGCATCGGCTAACTCCGT

>762 len=160 tot-seq=53

TGGGGAATATTGGACAATGGGCGCAAGCCTGATCCAGCCATGCCGCGTGTGTGAAGAAGG

CCTTAGGGTTGTAAAGCACTTTCAGTGGGGAGGAAGGGTGTTGTGTTAATAGCACGGCAT

TTTGACGTTACCCACAGAAGAAGCACCGGCTAACTCCGTG

>763 len=155 tot-seq=53

TGAGGAATATTGGTCAATGGCCGGAAGGCTGAACCAGCCAAGCCGCGTGAGGGAGGAAGG

CGCAGAGCGTCGCAGACCTCTTTTGCCGGGGGACAAAAGGCCGGACTCGTCCGGTCCTGA

GGGTACCCGGAGAAAAAGCATCGGCTAACTCCGTG

>764 len=138 tot-seq=53

ATGGGTGTGAAATGTGGGCACCTTAGAGTGCTGGGTGCTTGGGGTGGGGCGAGCGCGAAC

AGCAGCTCGTGTCTGATCTCCCAGTAGTTTGCTTTGGAAACTGGGACCGTGTGTTGACTC

TGAACGCACCTTACGCGC

>765 len=160 tot-seq=53

TAGGGAATCTTCCGCAATGGACGAAAGTCTGACGGAGCAACGCCGCGTGAGTGAAGAAGG

TTTTCGGATCGTAAAACTCTGTTGTTGGAGAAGAACAAGTAGGAGAGTAACTGCTCTTAC

CTTGACGGTACCCAACCAGAAAGCCACGGCTAACTACGTG

>766 len=161 tot-seq=53

TAGGGAATCTTCGGCAATGGACGGAAGTCTGACCGAGCAACGCCGCGTGAGTGAAGAAGG

TTTTCGGATTGTAAAGCTCTGTTATACGGGACGAAAAAGACGGATGCCAACAGTATCCGT

CCGTGACGGTACCGTAAGAGAAAGCCACGGCTAACTACGTG

>767 len=135 tot-seq=52

TGGGGAATATTGCACAATGGAGGAAACTCTGATGCAGCGACGCCGCGTGAAGGATGAAGT

ATTTCGGTATGTAAACTTCTATCAGCAGGGAAGAAAATGACGGTACCTGACTAAGAAGCC

CCGGCTAACTACGTG

>768 len=154 tot-seq=52

TAAGGAATATTGGTCAATGGGCGCAAGCCTGAACCAGCCATGCCGCGTGGAGGATGAAGG

TCCTCTGGATTGTAAACTTCTTTTATTTGGGACGAAAAACGGGGATTCTTCCTCACTTGA

CGGTACCAGATGAATAAGCACCGGCTAACTCCGT

>769 len=137 tot-seq=52

TGGGGAATATTGGGCAATGGGCGAAAGCCTGACCCAGCGACGCCGCGTGAGGGAAGAAGG

TCTTCGGATTGTAAACCTTAGTTAGCAGGGAAGAAGAAAGTGACGGTACCTGCAGAGAAA

GCCACGGCTAACTACGT

>770 len=155 tot-seq=52

TGAGGAATATTGGTCAATGGGCGAGAGCCTGAACCAGCCAAGTAGCGTGCAGGATGACGG

CCCTATGGGTTGTAAACTGCTTTTATGCGGGGATAAAGTGCGTGACGTGTCACGCATTGC

AGGTACCGCATGAATAAGGACCGGCTAATTCCGTG

>771 len=136 tot-seq=52

TGGGGAATTTTGCGCAATGGGGGAAACCCTGACGCAGCAACGCCGCGTGCGGGACGACGG

CCTTCGGGTTGTAAACCGCTTTCAGCAGGGAAGAAATTCGACGGTACCTGCAGAAGAAGC

TCCGGCTAACTACGTG

>772 len=155 tot-seq=52

AATCTTCCACAATGGACGCAAGTCTGATGGAGCAACGCCGCGTGAGTGAAGAAGGGTTTC

GGCTCGTAAAGCTCTGTTGTTGGTGAAGAAGGACAGGGGTAGTAACTGACCTTTGTTTGA

CGGTAATCAATTAGAAAGTCACGGCTAACTACGTG

>773 len=145 tot-seq=52

TGGGGAATATTGCGCAATGGGGGAAACCCTGACGCAGCGACGCCGCGTGCGGGATGAAGG

CCTTCGGGTTGTAAACCTCTTTTGTTAGGGAGCAAGGCACTTTGTGTTGAGTGTACCTTT

CGAATAAGCACCGGCTAACTACGTG

>774 len=230 tot-seq=51

GAGGCCAGTGGTAGTGTCTGCATGGACCGTTGCTGGCTGTGGTCCTCTGCAGGGACCACT

GCTGTGTCTGTTTCCAGCCTCTCCTTTCCTAAACCAGGCCGGCCCTGTCAGCCTGGCTCC

AAACTTCACTCCCTGGCCTTTGGAGGGAAGACCCTCTGGTTCGGGCCTTCCTTCCTTCTC

AGGGCTTTGGAAGCCCCTCTTCTTTCCAAAGGGGCTTGGCTTTCTGTCAC

>775 len=155 tot-seq=51

TGAGGAATATTGGTCAATGGGCGAGAGCCTGAACCAGCCAAGTCGCGTGAAGGACGACGG

TTCTATGGATTGTAAACTTCTTTTGTAGAGGAATAATGGCAGCTACGTGTAGCTGAGATG

CATGTACTCTACGAATAAGTATCGGCTAACTCCGT

>776 len=155 tot-seq=51

TGGGGAATCTTCCGCAATGGACGAGAGTCTGAACCAGCCAAGTAGCGTGCAGGATGACGG

CCCTATGGGTTGTAAACTGCTTTTGTTTGGGAATAATCGACATTACGTGTAATGTTTTGC

ATGTACCATTCGAATAAGGACCGGCTAATTCCGTG

>777 len=160 tot-seq=51

CCAAAATCTCACTATTGCAGCCAAAGGAGATGTGTATTTCCCCTTCTCCACTCCTTGCAT

CTGGTCCATGCTCAAAGTAAGCCAAACCAGTGCTCTTGTGCAGAACCCTAAATCGAAAGG

GGGTGACACATAAATCCAGGCACAATGGAGACTACCAAAG

>778 len=160 tot-seq=51

TGGGGAATTTTGGACAATGGGGGCAACCCTGATCCAGCCATTCCGCGTGAGTGAAGAAGG

CCTTCGGGTTGTAAAGCTCTTTCGGACGGAACGAAACGGTTAGCGTGAATAACGCTGACT

AATGACGGTACCGTAAGAAGAAGCACCGGCTAACTACGTG

>779 len=126 tot-seq=50

TAGGGAATCTTCCACAATGGACGCAAGTCTGATGGAGCAACGCCGCGTGAGTGAAGAAGG

ATAGAGGTAGTAACTGGCCTTTATTTGACGGTAATCAACCAGAAAGTCACGGCTAACTAC

GTGGTG

>780 len=122 tot-seq=50

GAGGACCAGTGTGTAATAGGACAGCAGCAGAATCAAAGGGTTTATCATCTCTGCCAGCCA

GAGCCTGGATGGCAGTGGAGTTTCCAGGACACTGCGGTTGGTCCTGATGCATGCGATCAT

CT

>781 len=140 tot-seq=50

TGGGGAATATTGGACAATGGGCGGAAGCCTGATCCAGCAACGCCGCGTGAGGGATGACGG

CCTTCGGGTTGTAAACCTCTTTCAGCACCGACGAAGCGCAAGTGACGGTAGGTGCAGAAG

AAGGACCGGCCAACTACGTG

>782 len=149 tot-seq=50

CCCCCCCCCTCAGGGACTGCTCGTGTCCAGATCTTGGCCAGCATGGAAAACTCCAGATAG

TGGGGGCAGGGGTCCAGGTCATCTTTATTACGCCCCAGGTCAAGGGTTCTTTGTACAAAA

ATAGGTCTCCGTTTGCCAGCAGTGTCCCT

>783 len=144 tot-seq=50

TGGGGAATATTGCACAATGGGCGCAAGCCTGATGCAGCGACGCCGCGTGGGGGATGACGG

CCTTCGGGTTGTAAACTCCTTTCGCCCACGACGAAGCCCTTCGGGGTGACGGTAGTGGGA

TAAGAAGCACCGGCTAACTACGTG

>784 len=160 tot-seq=50

TGGGGAATATTGCACAATGGGCGAAAGCCTGATGCAGCGACGCCGCGTGAGTGAAGACGG

TCTTCGGATTGTAAAGCTCTGTTATACGGGACGAACGGCAAGGTGGTAAATAGCCATCAT

GAGTGACGGTACCGTAAGAGAAAGCCACGGCTAACTACGT

>785 len=135 tot-seq=50

TGGGGAATCTTGCACAATGGGCGAAAGCCTGATGCAGCGACGCCGCGTGCGGGATGAAGG

CCTTCGGGTTGTAAACCGCTTTCAGCAGGGAAGACATCGACGGTACCTGCAGAAGAAGCC

CCGGCTAACTACGTG

>786 len=135 tot-seq=49

TGGGGAATATTGGGCAATGGGCGCAAGCCTGACCCAGCAACGCCGCGTGAAGGAAGAAGG

TCTTCGGGTCGTAAACTTCTATCCTTGGTGAAGATAATGACGGTAGCCAAGAAGGAAGCC

CCGGCTAACTACGTG

>787 len=160 tot-seq=49

TAGGGAATTTTCGGCAATGGGGGAAACCCTGACCGAGCAACGCCGCGTGAGCGAAGAAGG

CCTTCGGGTCGTAAAGCTCTGTTGTAAAGGAAGAACGGCGCATACAGGAAATGGTATGCG

AGTGACGGTACTTTACCAGAAAGCCACGGCTAACTACGTG

>788 len=154 tot-seq=49

TGGGGAATATTGCGCAATGGGGGAAACCCTGACGCAGCGACGCCGCGTGCGGGATGAAGG

CCTTCGGGTTGTAAACTGCTTTTATATGGGAATAAAGTGAGGGACGTGTCCCTTATTGCA

TGTACCATACGAATAAGGACCGGCTAATTCCGTG

>789 len=135 tot-seq=48

TGGGGAATATTGCACAATGGGCGAAAGCCTGATGCAGCGACGCCGCGTGAGCGAAGAAGT

ATTTCGGTATGTAAAGCTCTATCAGCAGGGAAGATAATGACGGTACCTGACTAAGAAGCA

CCGGCTAAATACGTG

>790 len=155 tot-seq=48

TGAGGAATATTGGTCAATGGCCGTAAGGCTGAACCAGCCAAGTCGCGTGAGGGAAGACGG

CCCTACGGGTTGTAAACCTCTTTTGTCAGGGAGCAAGGGGCGGGTCGTGACCTGCTGTGA

GAGTACCTGAAGAAAAAGCATCGGCTAACTCCGTG

>791 len=135 tot-seq=48

TGGGGAATATTGGACAATGGGGGCAACCCTGATCCAGCCATGCCGCGTGAGTGATGAAGG

CCTTCGGGTTGTAAAGCTCTTTTGGCGGGGACGATGATGACGGTACCCGCAGAATAAGCC

CCGGCTAACTTCGTG

>792 len=189 tot-seq=48

TGGGGAATATTGCACAATGGGGGAAACCCTGATGCAGCGACGCCGCGTGGAGGAAGAAGG

TCTTCGGATTGTAAACTCCTGTTGTTGGGGAAGATAATGACGGTACCCAACAAGGAAGTG

ACGGCTAACTACGTGCCAGCAGCCGCGGTAAAACGTAGGTCACAAGCGTTGTCCGGAATT

ACTGGGTGT

>793 len=160 tot-seq=48

TGGGGAATATTGCACAATGGGGGAAACCCTGATGCAGCCATGCCGCGTGTGTGAAGAAGG

CCTTCGGGTTGTAAAGCACTTTCAGTAGGGAGGAAAGGGTGAGTCTTAATACGGCTCATC

TGTGACGTTACCTACAGAAGAAGGACCGGCTAACTCCGTG

>794 len=206 tot-seq=48

TGGGGAATATTGCGCAATGGACGAAAGTCTGATGGAGCAACGCCGCGTGAGTGAAGAAGG

TTTTCGGATCGTAAAGCTCTGTTGTTGGTGAAGAAGGATAGAGGTAGTAACTGGCCTTTA

TTTGACGGTAATCAACCAGAAAGTCACGGCTAACTACGTGCCAGCAGCCGCGTAATATTC

AGATCGGAAGAGCGTCGTGTTGGGGA

>795 len=142 tot-seq=47

TGGGGAATATTGCGCAATGGGGGAAACCCTGACGCAGCGACGCCGCGTGCGGGATGAAGG

CCTTCGGGTTGTAAACCGCTTTTGATTGGGAGCAAGCTTTCGGGTGAGTGTACCTTTCGA

ATAAGCGCCGGCTAACTACGTG

>796 len=135 tot-seq=47

TGGGGGATATTGCACAATGGGGGGAACCCTGATGCAGCGACGCCGCGTGAGCGAAGAAGT

ACCTCGGTATGTAAAGCTCTATCAGCAGGGAAGAAAATGACGGTACCTGACTAAGAAGCC

CCGGCTAAATACGTG

>797 len=101 tot-seq=47

TGACTTACAGTTCTGGTCGGAGTCTTCTATCAGTATCCTCAACTTCTGAACGCATAGCTG

AGAGGAGGAAAGCCACACTGGTCTGCACCCCACCACCATGC

>798 len=152 tot-seq=47

TGGGGAATCTTGCACAATGGGCGAAAGCCTGATGCAGCGACGCCGCGTGGAGGGTGTAGG

CCTTCGGGTTGTGAACTCCTTTTTCTCGTGAAAAAGGCATGCTTTTGGGTGTGTTGATGG

TAGCGGGGGAAGAAGCGCCGGCTAACTACGTG

>799 len=138 tot-seq=47

TGGGGAATATTGGGCAATGGGGGAAACCCTGACCCAGCAACGCCGCGTGAGGGAAGAAGG

CTTTCGAGTTGTAAACCTCTGTCCTATGGGAAGAAGAAAGTGACTGTACCATAGGAGGAA

GCTCCGGCTAACTACGTG

>800 len=212 tot-seq=47

GGGTTGGTGTAGCCTCATCTGTTGCCATGACAAGTAACCACAATACCCAGGGGAGAGAGG

GTTTGCGTGAACTTCCAAGCTGCACTCCACCATGGAGGGGAGTCCGGGCAGGAACTGGAG

CAGGAACAGGAAGACAGGCCTAGGTGTACTCCACACAGCACATCTCTGACTGAAAGGACG

GGTCTGAGTGTGTGCTAGCTCAGTCGGTCAGC

>801 len=223 tot-seq=47

TGGGGAATATTGCACAATGGGCGCAAGCCTGATGCAGCCATGCCGCGTGTATGAAGAAGG

CCTTCGGGTTGTAAAGTACTTTCAGCGGGGAGGAAGGCGATAAGGTTAATAACCTTGTCG

ATTGACGTTACCCGCAGAAGAAGCACCGGCTAACTCCGTGCCAGCAGCCGCGGTAATAAC

AAGATCGGAAGAGCGTCGTGTAGGGAAAGAGTGGGCCAATGTG

>802 len=136 tot-seq=46

TGGGGAATATTGCACAATGGGGGGAACCCTGATGCAGCGATGCCGCGTGGAGGAAGAAGG

TTTTCGGATTGTAAACTCCTGTCGACAGGAAAGAAAAAGGACTGTACCTGTCAAGAAAGC

TCCGGCTAACTACGTG

>803 len=155 tot-seq=46

TGAGGAATATTGGTCAATGGCCGTAAGGCTGAACCAGCCAAGTCGCGTGAAGGAAGAAGG

ATCTATGGTTTGTAAACTTCTTTTATAGGGGAATAAAGGTATCCACGTGTGGATATTTGC

ATGTACCCTATGAATAAGCATCGGCTAACTCCGTG

>804 len=160 tot-seq=46

TGGGGAATTTTGGACAATGGGCGCAAGCCTGATCCAGCCATGCCGCGTGCAGGATGAAGG

CCTTCGGGTTGTAAACTGCTTTTGTACGGAACGAAAAGACTCCTTCTAATAAAGGGGGTC

CATGACGGTACCGTAAGAATAAGCACCGGCTAACTACGTG

>805 len=155 tot-seq=46

TGAGGAATATTGGACAATGGGTGGAAGCCTGATCCAGCCATCCCGCGTGTAGGATGACGG

CCTTATGGGTTGTAAACTACTTTTATCTGGGGATAAACCTACTTACGTGTAAGTAGCTGA

AGGTACCAGAAGAATAAGCACCGGCTAACTCCGTG

>806 len=109 tot-seq=46

TAGGGAATTTTCGGCAATGGGGGAACCCTGACCGAGCAACGCCGCGTGAAGGAAGAAGG

AATTCGTTCTGTAAACTTCTGTTATAAAGGAAGAACGGCGGATATAGGG

>807 len=156 tot-seq=46

TGAGGAATATTGGTCAATGGGCGGAAGCCTGAACCAGCCAAGTCGCGTGAGGGAATAAGG

CCCTACGGGTCGTAAACCTCTTTTGTCGGGGAGCAAGGCCCGGGACGTGTCCCGGGAAGG

AGAGTACCCGAAGAAAAAGCATCGGCTAACTCCGTG

>808 len=155 tot-seq=46

TGAGGAATATTGGTCAATGGGCGCAGGCCTGAACCAGCCAAGTCGCGTGAGGGAAGACTG

CCCTATGGGTTGTAAACCTCTTTTATAAGGGAAGAATAATGACTACGTGTAGTCAGATGC

CCGTACCTTATGAATAAGCATCGGCTAACTCCGTG

>809 len=170 tot-seq=46

GAGGCTGTGTTCTAGAAAGGTCCCTGTAGCTGCCGTGTGGCAGTGGACTGATGGGTGCAG

GACTGGGGAGAAACAGGCTAAGGAGGAGGCTGCCACATGGGTTTAGTGGAAAGAGAGTAA

GGGCTTGCACCAGGACCCCCCAGCAGAGATGGAGAAGAAGGGACCTCATC

>810 len=137 tot-seq=46

TGGGGAATATTGGGCAATGGGGGAAACCCTGACCCAGCGACGCCGCGTGAGGGAAGAAGG

TTTTCGGATTGTAAACCTCTGTTCTTGGTGAAGAGGGGAAGACGGTAGCCAAGGAGAAAG

CCCCGGCTAACTACGTG

>811 len=160 tot-seq=46

TGGGGAATATTGCGCAATGGGGGAAACCCTGACGCAGCGACGCCGCGTGCGGGATGAAGG

CCTTCGGGTTGTAAAGCTCTGTTGTTGGTGAAGAAGGATAGAGGTAGTAACTGGCCTTTA

TTTGACGGTAATCAACCAGAAAGTCACGGCTAACTACGTG

>812 len=138 tot-seq=46

TGGGGGATATTGCGCAATGGGGGAAACCCTGACGCAGCAACGCCGCGTGAAGGATGAAGG

TCTTCGGATTGTAAACTTCTTTTATTAAGGACGAAGAAAGTGACGGTACTTAATGAATAA

GCTCCGGCTAACTACGTG

>813 len=135 tot-seq=45

TCGGGAATTTTGCGCAATGGACGAAAGTCTGACGCAGCAACGCCGCGTGAGGGATGACGG

CCTTCGGGTTGTAAACCTCTTTTATCAGGGAAGATAATGACGGTACCTGATGAATAAGTC

ACGGCTAACTACGTG

>814 len=155 tot-seq=45

TGAGGAATATTGGTCAATGGGCGCAAGCCTGAACCAGCCATGCCGCGTGCAGGATGACGG

TCCTATGGATTGTAAACTGCTTTTGTACAGGAAGAAACAGTTCTACGTGTAGAGCCTTGA

CGGTACTGTAAGAATAAGGATCGGCTAACTCCGTG

>815 len=160 tot-seq=45

TGGGGAATTTTGGACAATGGGGGCAACCCTGATCCAGCCATGCCGCGTGCGGGAAGAAGG

CCTTCGGGTTGTAAACCGCTTTTGTCAGGGACGAAAAGGGACGGGTTAAGAGCTAGTTCC

GCTGACGGTACCTGAAGAATAAGCACCGGCTAACTACGTG

>816 len=155 tot-seq=45

TGAGGAATATTGGACAATGGTCGGAAGACTGATCCAGCCATGCCGCGTGCAGGATGAAGG

TCCTATGGATTGTAAACTGCTTTTGTAAGGGAAGAATAAGGAGTACGTGTACTTTGATGA

CGGTACCTTACGAATAAGCATCGGCTAACTCCGTG

>817 len=135 tot-seq=45

TGGGGAATATTGCACAATGGACGGAAGTTTGATGCAGCGACGCCGCGTGAGCGAAGAAGT

ATTTCGGTATGTAAAGCTCTATCAGAAGGGAAGAAAATGACGGTACCTTACTAAGAAGCT

CCGGCTAAATACGTG

>818 len=138 tot-seq=45

TGGGGAATTTTACGCAATGGGGGAAACCCTGACGTAGCGACACCGCGTGAGCGAAGAAGC

CCTTTGGGGTGTAAAGCTCTGTCAGCTGGAACGAAAACAATGACGGTACCAGCAGAGGAA

GCATCGGCTAACTACGTG

>819 len=135 tot-seq=45

TGGGGAATATTGCACAATGGAGGAAACTCTGATGCAGCGACGCCGCGTGAGGGAAGAAGG

TCTTCGGATTGTAAACCTCTGTCTTCGGTGACGAAAATGACGGTAGCCGAGGAGGAAGCC

ACGGCTAACTACGTG

>820 len=135 tot-seq=45

TGGGGAATTTTGCACAATGGGGGAAACCCTGATGCAGCGACGCCGCGTGAGTGAAGACGG

TCTTCGGATTGTAAAGCTCTGTTTCGAGGGAAGAAAAGGACGGTACCTCGGGAGGAAGCC

CCGGCTAACTACGTG

>821 len=135 tot-seq=44

TGGGGAATATTGCACAATGGGGGGAACCCTGATGCAGCAATGCCGCGTGGGTGAAGAAGT

ACTCCGGTATGTAAAGCCCTATCAGCAGGGAAGAAAATGACGGTACCTGACTAAGAAGCC

CCGGCTAACTACGTG

>822 len=155 tot-seq=44

TGAGGAATATTGGTCAATGGACGAAAGTCTGAACCAGCCAAGTAGCGTGCAGGATGACGG

CCCTCTGGGTTGTAAACTGCTTTTAGTTGGGAATAAAAAGAGGGACGTGTCCCTTATTGT

ATGTACCATCAGAAAAAGGACCGGCTAATTCCGTG

>823 len=220 tot-seq=44

GACCTAAAACTGGGATTTGTAAGTTGATTACATGGAAAAAACTAGTGGATCTTAATTCAG

AAACTCTAATCCAGGATTAATGTGAACCTGATTGGACCATCTAAATCTGAAGACAGTCTT

CTGATCTCTGCTTCTCCTTTCTGTAGCTGGGCTGACTCGTGAGCAGCACCAATCAATCTA

GTGTCTCTGAGTTCATCTTCCTGGAACTCTCCAGGCAGCC

>824 len=161 tot-seq=44

TAGGGAATCTTCCACAATGGGCGCAAGCCTGATGGAGCAACACCGCGTGAGTGAAGAAGG

GTTTCGGCTCGTAAAACTCTGTTGTTAGAGAAGAACGTTGGTAGGAGTGGAAAATCTACC

AAGTGACGGTAACTAACCAGAAAGGGACGGCTAACTACGTG

>825 len=160 tot-seq=44

TGGGGAATATTGCGCAATGGACGAAAGTCTGACGCAGCGACGCCGCGTGAGGGATGAAGG

TTTTCGGATCGTAAACCTCTGTCAGGAGGGAAGAAAGCGCATGGCGCCAATCAGCCATGC

GTTGACGGTACCTCCAAAGGAAGCGCCGGCTAACTCCGTG

>826 len=165 tot-seq=44

TGCGCAAAATATGGCATTGGATTTTATTCTATGGGAAATAAGAAGTCATTGGTAAGGTTC

AGGCAGGGAAGTTACATGTTTCTAAGTAACTGCTGCTATGGTGAATGGCTGGTAGGTTGT

CAAGATTAGAAATCTGGAGACCGGTTGAAATGGTCTTGAAGTTGC

>827 len=137 tot-seq=44

TGGGGGATATTGCACAATGGGGGAAACCCTGATGCAGCGACGCCGCGTGAGGGAAGACGG

TTTTCGGATTGTAAACCTCTGTCTTTGGGGACGAACAAATGACGGTACCCAAGGAGGAAG

CCACGGCTAACTACGTG

>828 len=135 tot-seq=43

TGGGGAATATTGCACAATGGGGGAAACCCTGATGCAGCAACGCCGCGTGAAGGATGAAGG

CCTTTGGGTCGTAAACTTCTGTTCTAAGGGAAGATAGTGACGGTACCTTAGGAGCAAGTC

CCGGCTAACTACGTG

>829 len=160 tot-seq=43

TGGGGAATTTTGGACAATGGGCGAAAGCCTGATCCAGCAATGCCGCGTGCAGGATGAAGG

CCTTCGGGTTGTAAACTGCTTTTGTACGGAACGAAAAAGCTCCTTCTAATACAGGGGGCC

CATGACGGTACCGTAAGAATAAGCACCGGCTAACTACGTG

>830 len=160 tot-seq=43

CTAAGAATCTTCCGCAATGGGCGCAAGCCTGACGGAGCGACGCCGCGTGAATGAAGAAGG

CCGAAAGGTTGTAAAATTCTTTTAAGTTTGAAGAATAATCAGTATAGGGAATGATACTGA

GATGACGGTAAAACTTGAATAAGCCCCGGCTAATTACGTG

>831 len=161 tot-seq=43

TGGGGAATATTGCACAATGGGCGCAAGCCTGATGCAGCAACGCCGCGTGAGTGATGACGG

TCTTCGGATTGTAAAGCTCTGTTAATCGGGACGAATGGTCGGTGTGCAAATAGTGCATGG

ACATGACGGTACCGGAATAGAAAGCCACGGCTAACTACGTG

>832 len=137 tot-seq=43

TGGGGAATATTGCACAATGGGCGAAAGCCTGATGCAGCAACGCCGCGTGAAGGAAGAAGG

CCTTCGGGTCGTAAACTTCTGTCCTTGGGGAAGAAGAACTGACGGTACCCAAGGAGGAAG

CCCCGGCTAACTACGTG

>833 len=140 tot-seq=43

TGGGGAATATTGCACAATGGGCGCAAGCCTGATGCAGCGACGCCGCGTGAGGGATGACGG

CCTTCGGGTTGTAAACCTCTTTCAGCAGGGACGAAGCGAAAGTGACGGTACCTGCAGAAG

AAGGACCGGCCAACTACGTG

>834 len=160 tot-seq=43

TAGGGAATCTTCGGCAATGGACGGAAGTCTGACCGAGCAACGCCGCGTGAGTGAAGAAGG

TTTTCGGATCGTAAAGCTCTGTTGTTGGTGAAGAAGGACAGGGGTAGTAACTGACCTTTG

TTTGACGGTAATCAATTAGAAAGTCACGGCTAACTACGTG

>835 len=156 tot-seq=43

TGAGGAATATTGGTCAATGGGCGCAAGCCTGAACCAGCCAAGTCGCGTGAGGGAAGACGG

CCCTACGGGTTGTAAACCTCTTTTGCCGGGGAGCAAGCCTGAGCACGTGTGCCCAGGCGG

AGAGTACCCGGAGAAAAAGCATCGGCTAACTCCGTG

>836 len=159 tot-seq=43

TAGGGAATCTTCCGCAATGGGCGAAAGCCTGATGCAGCCATGCCGCGTGTATGAAGAAGG

CCTTCGGGTTGTAAAGTACTTTCAGCGGGGAGGAAGGGAGTAAAGTTAATACCTTTGCTC

ATTGACGTTACCCGCAGAAGAAGCACCGGCTAACTCCGT

>837 len=230 tot-seq=42

GCCTGATAAAATGGCACTGATGTATATGAACACATAGAGAAAAGAGGCCAACTGCAACAA

AATGCCAACAGACACAAAAGTCATGCCACCGGAAATGGAGCAGAAATGCTATTTGTGAAT

CTGCTTCATAAAGGACATTTTTTTTAAGCTAATAACATCTTTCTATACAATTTCACATGT

ACAATATCACTTAAGCCTCTTTTTGCTCCATATCTAAAGCTGCAGGAAGT

>838 len=155 tot-seq=42

TGAGGAATATTGGTCAATGGGCGTAAGCCTGAACCAGCCAAGTAGCGTGAAGGAAGACTG

CCCTACGGGTTGTAAACTTCTTTTATACGGGAATAAAGTGAGGCACGTGTGCCTTTTTGT

ATGTACCGTATGAATAAGCATCGGCTAATTCCGTG

>839 len=136 tot-seq=42

TGGGGGATATTGCACAATGGGGGGAACCCTGATGCAGCGACGCCGCGTGGGTGAAGAAGT

ATTTCGGTATGTAAAGCCCTATCGGCAGGGAAGAAATTGGACGGTACCTGACTAAGAAGC

CCCGGCTAACTACGTG

>840 len=221 tot-seq=42

TAATAGAGAGGCTTTTGCCGGGACGCTGACTGCCAAGTTTGGGAAGCCATAGTCCTAAGG

TGATAAAAAGCCCAACTTCAGGAACGTAAGTGTAGGGGAGCTTCCAGGCATCCAGCAGTC

CCCAATTCAGGGCAGGTCTTGCTAGTGTCAAACTCAGCTTCCGGGACTCTCCTGGTGCTC

AGAAGATGCTCTGTAAAAAAACTCCACCACCATTCCTCAGG

>841 len=155 tot-seq=42

TGGGGAATATTGCACAATGGGCGAGAGCCTGAACCAGCCAAGTAGCGTGCAGGATGACGG

CCCTATGGGTTGTAAACTGCTTTTACGCGGGGATAAAGTGCGTGACGTGTCGCGCATCGC

AGGTACCGCGTGAATAAGGACCGGCTAATTCCGTG

>842 len=135 tot-seq=42

TGGGGAATCTTGGACAATGGGGGCAACCCTGATCCAGCGACGCCGCGTGGGTGATGAAGG

CCTTCGGGTCGTAAAGCCCTTTTGCCGGGGAAGATGATGACGGTACCCGGAGAAGAAGCA

CCGGCTAACTCTGTG

>843 len=155 tot-seq=42

TGAGGAATATTGGTCAATGGCCGGGAGGCTGAACCAGCCAAGTCGCGTGAGGGAAGACGG

TCCTATGGGTTGTAAACCTCTTTTGTCGGAGAACAAAACCCGGGACGTGTCCCGGACTGC

GTGTATCCGAAGAAAAAGCATCGGCTAACTCCGTG

>844 len=135 tot-seq=42

TGGGGGATATTGCGCAATGGGGGAAACCCTGACGCAGCAACGCCGCGTGAAGGAAGAAGG

TCTTCGGATTGTAAACTTCTTTAAGTGTGGAAGAAGATGACGGTACACACAGAATAAGCC

ACGGCTAACTACGTG

>845 len=155 tot-seq=42

TGAGGAATATTGGTCAATGGGCGCGAGCCTGAACCAGCCAAGTCGCGTGAGGGATGAAGG

ATCTATGGTTCGTAAACCTCTTTTATAAGGGAATAAAGTGTAGGACGTGTCCTGTTTTGT

ATGTACCTTATGAATAAGGATCGGCTAACTCCGTG

>846 len=133 tot-seq=41

TCGGGAATATTGCACAATGGAGGAAACTCTGATGCAGTGACGCCGCGTATAGGAAGAAGG

TCTTAGGATTGTAAGCTATTGTCGTGCAAGAAGAAAATGACTATTGCAGGAGGAAGCCCT

GGCTAAATATGTG

>847 len=135 tot-seq=41

TGAGGAATCTTCCACAATGGGCGAAAGCCTGATGGAGCAACGCCGCGTGAAGGATGAAGG

CCTTCGGGTTGTAAACTTCTTTTATGAGTGAAGAATATGACGGTAACTCATGAATAAGCA

CCGGCTAACTACGTG

>848 len=136 tot-seq=41

TGGGGAATTTTGCGCAATGGGGGGAACCCTGACGCAGCAACGCCGCGTGCGGGACGAAGG

CCTTCGGGTTGTAAACCGCTTTCAGCAGGGAAGATTCAGACGGTACCTGCAGAAGAAGCT

CCGGCTAACTACGTGG

>849 len=226 tot-seq=41

GAGTTTTGTCTCTGCCAGATAATTAAGTTCAGAATGCTAACAAGCTTTCAAAACAAAACA

CAACTCAAGTACTCTTTGGATGGGATATAATAAAATGGTTCTATCTGAAATTGCAAACAG

CTTTGTTGCTGCAAACAAAGGCTCAGTTCTTTGTGCCTCTTTGGCGTGGAGGCCCAGAAG

TACGAGGGTGAAAAGGGTCAATGAAACTCTGAGGGTGTTGCTTCCC

>850 len=135 tot-seq=41

TAGGGAATATTGCACAATGGGCGAAAGCCTGATGCAGCGACGCCGCGTGAGTGATGAAGG

CCTTCGGGTCGTAAAACTCTGTGATATGTGAAGATAATGACGGTAACATATCAGAAAGGA

CCGGCTAACTTCGTG

>851 len=155 tot-seq=41

TGAGGAATATTGGTCAATGGGCGAGAGCCTGAACCAGCCAAGTAGCGTGAAGGATGAAGG

TCCTACGGATTGTAAACTTCTTTTATACGGGAATAAAGTTTCCTACGTGTAGGATTTTGT

ATGTACCGTATGAATAAGCATCGGCTAACTCCGTG

>852 len=161 tot-seq=41

TGGGGAATCTTCCGCAATGGACGAAAGTCTGACGGAGCAACGCCGCGTGAACGATGAAGG

TCTTCGGATTGTAAAGTTCTGTGATCCGGGACGAAGGCATTGATTGAGAATATTGATTGA

TGTTGACGGTACCGGAAAAGCAAGCCACGGCTAACTACGTG

>853 len=143 tot-seq=41

CATGTGGCCAGGGCGGCTCTTGCTTCTCCGGCATCTCAGAAATTTAAAACAGTAATCCCA

GGACTGAAATAGATGTGTTTGAAAGCATCTGTGGCATCTGGAGGCCGCTTCTAATGTATT

CCTGCACTTGAGGAAGAGCTGGT

>854 len=152 tot-seq=41

TGGGGAATATTGCACAATGGGCGCAAGCCTGATGCAGCGACGCCGCGTGCGGGATGGAGG

CCTTCGGGTTGTAAACCGCTTTTGTTCAAGGGCAAGGCACGGTTTCGGCCGTGTTGAGTG

GATTGTTCGAATAAGCACCGGCTAACTACGTG

>855 len=160 tot-seq=41

CTAAGAATATTCCGCAATGGACGAAAGTCTGACGGAGCGACGCCGCGTGGATGATGAAGG

CCGGAAGGTTGTAAAGTCCTTTTATCCGTGAAGAAGAAGATTAAGAGGGAATGCTTAATT

GGTGACGGTAGCGGATGAATAAGCACCGGCTAATTACGTG

>856 len=136 tot-seq=41

TGGGGAATATTGCACAATGGGCGCAAGCCTGATGCAGCAACGCCGCGTGAGGGAAGACGG

TTTTCGGATTGTAAACCTCTGTCTTTGGTGACGAAGAAGTGACGGTAGCCAAGGAGGAAG

CCACGGCTAACTACGT

>857 len=173 tot-seq=41

ACACGAGTCTATCCAGGGCAGCCAACAGGCTGGCTAGGAGAAGGAGGGACTAGGGAGTCT

AAGGAGTGAGAAGGCAGCTGGCAGCTGTTCGGATGGAATGTCAAAGAGGGAAAGAGAACA

GCCCCTACAGGAGCCCGTTCATGAGGTGAAGAGCAGGCCAGGAGTGGGTGGAG

>858 len=155 tot-seq=41

TGAGGAATATTGGACAATGGTCGGAAGACTGATCCGGCCATGCCGCGTGCAGGAAGACGG

CCTTATGGGTTGTAAACTGCTTTTGCAGGGGAAGAATAAGGACTACGAGTAGTTTGATGA

CGGTACTCTGCGAATAAGCATCGGCTAACTCCGTG

>859 len=140 tot-seq=40

TGGGGAATATTGCACAATGGGCGAAAGCCTGATGCAGCAACGCCGCGTGAGGGACGACGG

CCTTCGGGTTGTAAACCTCTTTTAGCAAGGAAGAAGCGAAAGTGACGGTACTTGCAGAAA

AAGCGCCGGCTAACTACGTG

>860 len=160 tot-seq=40

TGGGGAATCTTCCGCAATGGACGAAAGTCTGATGGAGCAACGCCGCGTGAGTGAAGAAGG

GTTTCGGCTCGTAAAGCTCTGTTGGTAGTGAAGAAAGATACAGGTAGTAACTGGCCTTTA

TTTGACGGTAATTACTTAGAAAGTCACGGCTAACTACGTG

>861 len=182 tot-seq=40

TAGGGAATCTTCCACAATGGGCGAAAGCCTGATGGAGCAACGCCGCGTGTGTGATGAAGG

GTTTCGGCTCGTAAAACACTGTTGTAAGAGAAGAATGACATTGAGAGTAACTGTTCAATG

TGTGACGGTATCTTACCAGAAAGGAACGGCTAAATACGTGCCAGCAGCCGCGGTCAGATC

GG

>862 len=198 tot-seq=40

TGGGGAATATTGGACAATGGGGGCAACCCTGATCCAGCAATTCTGTGTGCACGAAGAAGG

TTTTCGGATTGTAAAGTGCTTTCAGCAGGGAAGAAGGAAGTGACGGTACCTGCAGAAGAA

GCGACGGCTAAATACGTGCCAGCAGCCGCGGAATAGAGAGATCGGAAGAGCGTCGTGTAG

GGAAAGAGTGGGGAATAT

>863 len=131 tot-seq=40

GAGTTCAAAGTCATCTTTGGTTATACAACATGCTTGAGGCCAGCCTGGGCTACATGAGAC

TCTATTTCAAAACAACCACAAAAATAGTCATCCACAGAGTAGTCCATGCAACAGGTACAG

AACGCGTGTTC

>864 len=162 tot-seq=40

AAGAGAAGAAACCCCGGTGGTCCGGTTTAGAAGAGGGGTCCTGGAAGCAGCCTCCCCTCC

CTGAGACAAAGCAAAGGCCACAGGGCTCTTCGTGCGTGACGTCATCGCTGCAAAGCATGG

ACATTTAGAGCCAGCGGGGTAGGGCAGCTCACACCAGTGATC

>865 len=161 tot-seq=40

TGGGGAATCTTCCGCAATGGACGAAAGTCTGACGGAGCAACGCCGCGTGAGTGATGAAGG

TCTTCGGATTGTAAAACTCTGTTGTTAGGGACGAAAGCACCGTGTTCGAACAGGTCATGG

TGTTGACGGTACCTAACGAGGAAGCCACGGCTAACTACGTG

>866 len=161 tot-seq=40

TGGGGAATCTTCCGCAATGGACGAAAGTCTGACGGAGCAACGCCGCGTGAGTGATGACGG

CCTTCGGGTTGTAAAGCTCTGTTAATCGGGACGAAAGGTCCTCTTGCGAATAGTTAGAGG

AATTGACGGTACCGGAATAGAAAGCCACGGCTAACTACGTG

>867 len=155 tot-seq=40

TGAGGAATATTGGTCAATGGCCGAGAGGCTGAACCAGCCAAGTCGCGTGAGGGATGACGG

CCCTACGGGTTGTAAACCTCTTTTGTCAGGGAGCAAGGGGCATCACGAGTGATGCTGTGA

GAGTACCTGAAGAAAAAGCATCGGCTAACTCCGTG

>868 len=188 tot-seq=40

GCCGGGGCAGGGGTACACAGGTGGGCATGCCTGGATGCCAGCTCACCCTGGCGAGCTCAG

ATGTGCAGGCTTTTTAGGAGACTCTATGAAGTGGAAGTGGAAGATGAACTGAGTGGGGAA

AATGAAAAGGGGATCTTGGGATGAGGGTCTTACTCTTTGCAAATGCATTGCCTGTGGCAT

CTCCTTTC

>869 len=155 tot-seq=40

TGGGGAATATTGCACAATGGGCGAAAGCCTGAACCAGCCAAGTAGCGTGCAGGAAGACGG

CCCTATGGGTTGTAAACTGCTTTTATGTGGGGATAAAGTGCGTGACGTGTCATGCATTGC

AGGTACCACATGAATAAGGACCGGCTAATTCCGTG

>870 len=211 tot-seq=39

TGAGGAATATTGGTCAATGGGCGCAAGCCTGAACCAGCCATGCCGCGTGCAGGATGACGG

CTCTATGAGTTGTAAACTGCTTTTGTACGAGAATAACGGTGTTTACGTGTAGACATATGA

ATGTATCGTACGAATAAGGATCGGCTAACTCCGTGTGTTTACGTGTAGACATATGAATGT

ATCGTACGAATAAGGATCGGCTAACTCCGTG

>871 len=194 tot-seq=39

GTAAAACAGGCAGTGTTCTTTTTGCAGAGGCACCCTGCTACCAGCACAGTGACAGGACCC

AGGCTATGGGTTCTGCCCTAGATAGGTTATGTGTTCTCCCTAAAACATTGATAAATACAT

AAAATGTGCTATAGAGCTATTTCTGTAAGACTTTCTGGCCAGGCTTCATAGCACAAGATG

CCTATATCGTTCAT

>872 len=155 tot-seq=39

TGAGGAATATTGGTCAATGGGCGAGAGCCTGAACCAGCCAAGTAGCGTGCAGGATGACGG

CCCTATGGGTTGTAAACTGCTTTTGTATGGGATTAAAGTCACCTACGTGTAGGTGTTTGC

AGTTACCATACGAATAAGCATCGGCTAACTCCGTG

>873 len=136 tot-seq=39

TGGGGAATATTGCACAATGGAGGAAACTCTGATGCAGCGATGCCGCGTGAGGGAAGAAGG

TTTTAGGATTGTAAACCTCTGTCTTTGGGGACGAAAAATGACGGTACCCAAGGAGGAAGC

TCCGGCTAACTACGTG

>874 len=154 tot-seq=39

AGGATGTGCCATCTTTGAAAAACACCAGCTGGAAATATAATGTGGTCTTGAAATTCAATA

AGCTTCAGAAAGAAACTTTAGATAGGGAAGAAGACACCCCCTAGAGAAACTGCTTGTGGT

TAGCAAGAATTCAGAAAGCTGAATTTACAAGTCC

>875 len=161 tot-seq=39

TGGGGAATATTGGACAATGGGCGAAAGTCTGACGGAGCAACGCCGCGTGAGTGAAGAAGG

TCTTCGGATTGTAAAGCTCTGTTATACGGGACGAAAAAGACGGATGCCAACAGTATCCGT

CCGTGACGGTACCGTAAGAGAAAGCCACGGCTAACTACGTG

>876 len=160 tot-seq=39

TAGGGAATCTTCCGCAATGGACGAAAGTCTGACGGAGCAACGCCGCGTGAGTGAGGAAGG
TCTTCGGATTGTAAAACTCTGTTCTTCGGGAAGAACGAATTCGAGAGTAACTGCTCGAAT
TGTGACGGTACCGAAGGAGAAAGCCCCGGCTAACTACGTG
>877 len=173 tot-seq=39
TCATGATGAATGGCGAAGGAGCACAAAGAAACTTATGGGGTGCAGGGAGGTGAGCCGCAG
GGCCCAGGAGAGGGCAGTGGCATTACTGGTGGGGGACAGCCAGCCAGCCCTCCAAATACA
CCACACGTACCATAGGCTCTCAAGGGCTCTTTCCAGAGGAGGGGGTGGCATCC
>878 len=154 tot-seq=38
TGAGGAATATTGGTCAATGGACGCAAGTCTGAACCAGCCATGCCGCGTGCAGGAAGACGG
CTCTATGAGTTGTAAACTGCTTTTGTATTAGGGTAAACTCAGGTACGTGTACCTGACTGA
AAGTATAATACGAATAAGGATCGGCTAACTCCGT
>879 len=136 tot-seq=38
TGGGGAATATTGCACAATGGGGGAAACCCTGATGCAGCGACGCCGCGTGGGCGACGAAGC
GCTCCGGCGCGTAAAGCCCTGTCAGCAGGGAAGAAAAAAGACAGTACCTGACCAAGAAGC
CCCGGCTAACTACGTG
>880 len=138 tot-seq=38
TAGGGAATATTGGGCAATGGGCGCAAGCCTGACCCAGCAACGCCGCGTGAGGGAAGAAGG
TTTTCGGATTGTAAACCTCTGTCCTATGTGACGAAGGAAGTGACGGTAGCATAGGAGGAA
GCCCCGGCTAACTACGTG
>881 len=211 tot-seq=38
TATGCGACGGCCATCTATTGAACAAGGAATCGTGGAAGCAGAACACAGCCCATCAGCAGA
TCCTCCTGTGTCCAAGACCTACCTGCCCAGCCACCACCTACCACACATTTCTTCAAGCCA
AGCCTGGGGACAAGGGCAGACCCAGGAGCCACCAGCATCAACCACCTTCCTTCCCTAGAA
AACAGGACACCTCCACATACACACCCCTCAA
>882 len=135 tot-seq=38
TGGGGAATATTGCACAATGGGCGCAAGCCTGATGCAGCGACGCCGCGTGAGTGAAGAAGT
ATTTCGGTATGTAAAGCTCTATCAGCAGGGAAGATAATGACAGTACCTGACTAAGAAGCT
CCGGCTAAATACGTG
>883 len=155 tot-seq=38

TGAGGAATATTGGTCAATGGGAGAGATCCTGAACCAGCCAAGCCGCGTGAGGGAAGACGG

TCCTATGGGTTGTAAACCTCTTTTGTCAGGGAGCAAAAACTGCCACGCGTGGCAGGATGA

GAGTACCTGAAGAAAAAGCATCGGCTAACTCCGTG

>884 len=138 tot-seq=38

TGGGGAATATTGGGCAATGGGCGCAAGCCTGACCCAGCAACGCCGCGTGAAGGAAGAAGG

CTTTCGGGTTGTAAACTTCTTTTCTGGGGGACGAAGAAAGTGACGGTACCCCAGGAATAA

GCCACGGCTAACTACGTG

>885 len=138 tot-seq=38

TGGGGAATATTGGGCAATGGGCGCAAGCCTGACCCAGCAACGCCGCGTGAAGGAAGAAGG

CTTTCGGGTTGTAAACTTCTTTTATGAGGGACGAAGGAAGTGACGGTACCTCATGAATAA

GCCACGGCTAACTACGTG

>886 len=136 tot-seq=38

TGGGGAATATTGCACAATGGGGGAAACCCTGATGCAGCGACGCCGCGTGAGCGAGGAAGT

ATTTCGGTATGTAAAGCTCTATCAGCAGGGACGAAAAAAGACGGTACCTGACTAAGAAGC

CCCGGCTAACTACGTG

>887 len=154 tot-seq=37

TAGGGAATATTGGTCAATGGGCGGAAGCCTGAACCAGCCATGCCGCGTGATGGATGACGG

CCTTAGGGTTGTAAACATCTTTTCTGCTAGAGGAATATAGTTCACGTGTGGACAGACGAC

TGTATAGCAGGAATAAGGATCGGCTAACTCCGTG

>888 len=178 tot-seq=37

GGAGTTATGGATAAGAAATCTGGAGGCACCCCACCCCACCCTGGCTCCCAGTCAATCACT

TGACAGCTGTGTTGCCTTCGGTGTTACCTAGCCCCTGGGTGCTTCAGTGTCTGCAGGTGT

TAAATAAAAAAGGGATAACAATAGTAATAACCAAAAAGGGATAACAATAGTAATAACC

>889 len=202 tot-seq=37

AGTTGCAGGACGCAGAGGTGGAGTCTTCTGCCAAGTCTGGGAAGCCTTAAGGAAAGGAGT

GCCCGTCGGCGTCTTGGTCCTCCTGTCCCTGCTGCAGGGGCTGGGGCCTCCGGAGCTGCT

GCGGGCTCCCCTCAGGCTCTGCTTCGTGACCCGTGACCCATGACCCACAGTGCTGGCCTC

CTGTGGGGCCACTATAGCAGCC

>890 len=135 tot-seq=37

TGGGGGATATTGCACAATGGGGGAAACCCTGATGCAGCAACGCCGCGTGAGGGAAGAAGG

TTTTCGGATTGTAAACCTCTGTCCTAGGTGAAGATAATGACGGTAACCTGGGAGGAAGCT

CCGGCTAACTACGTG

>891 len=134 tot-seq=37

GCGCGCAAATTACCCAATCCCAACACGGGGAGGTAGTGACAATATGTAACAATATACGGC

CTTATACGTCGTATAATTGGAATGAGTACAATTTAAATCCCTTAACGAGGAACAATTGGA

GGGCAAGTCTGGTG

>892 len=155 tot-seq=37

TGAGGAATATTGGTCAATGGACGGGAGTCTGAACCAGCCAAGTAGCGTGAAGGATGACTG

CCCTATGGGTTGTAAACTTCTTTTATACGGGAATAAAGTGATCCACGTGTGGATTTTTGT

ATGTACCGTATGAATAAGGATCGGCTAACTCCGTG

>893 len=160 tot-seq=37

TGGGGAATATTGCACAATGGGGGAAACCCTGATGCAGCGACGCCGCGTGAGTGATGAAGG

TCTTCGGATCGTAAAACTCTGTTATTAGGGAAGAACAAATGTGTAAGTAACTATGCACGT

CTTGACGGTACCTAATCAGAAAGCCACGGCTAACTACGTG

>894 len=161 tot-seq=37

TAGGGAATCTTCGGCAATGGACGAAAGTCTGACCGAGCAACGCCGCGTGAGTGAAGAAGG

TTTTCGGATCGTAAAGCTCTGTTGTAAGTCAAGAACGTGTGTGAGAGTGGAAAGTTCACA

CAGTGACGGTAGCTTACCAGAAAGGGACGGCTAACTACGTG

>895 len=155 tot-seq=36

TGAGGAATATTGGTCAATGGACGAGAGTCTGAACCAGCCAAGTCGCGTGAAGGAAGACGG

TTCTATGAATTGTAAACTTCTTTTGTGCAGGAATAAAGTGGGTTACGTGTAACCTTTTGT

ATGTACTGTACGAATAAGGATCGGCTAACTCCGTG

>896 len=163 tot-seq=36

AAAAACAGCATGTCAGATTTCTTGGACAGGAAGAAAACCTGGAAGAATGGCAGTATTACC

CCAGGTGACAGAGCTCTGTAAATCTCAGAGTTCAGGAGCTGTGGTTGAGCTCTCTTAACA

CCTGCTTCCTTTGGCCAAGAAAGATGAAAACAACAACAAAAAC

>897 len=143 tot-seq=36

TAGGGAATCTTCGGCAATGGACGGAAGTCTGACCGAGCAACGCCGCGTGCGGGATGAAGG

CCTTCGGGTTGTAAACCGCTTTTGATTGGGAGCAAGCCTTTTGGGTGAGTGTACCTTTCG

AATAAGCGCCGGCTAACTACGTG

>898 len=161 tot-seq=36

TGGGGAATATTGGACAATGGGCGAAAGCCTGATCCAGCCATGCCGCGTGTGTGAAGAAGG

CCTTTTGGTTGTAAAGCACTTTAAGCGAGGAGGAGGCTACTGGTATTAATACTACCGGAT

AGTGGACGTTACTCGCAGAATAAGCACCGGCTAACTCTGTG

>899 len=133 tot-seq=36

TGGGGAATATTGCACAATGGGGGAAACCCTGATGCAGCGACGCCGCGTGATTTAGAAGGC

CTTCGGGTTGTAAAAATCTTTTGTATGGGAAGAAAATGACAGTACCATACGAATAAGGAC

CGGCTAATTACGT

>900 len=172 tot-seq=36

TAGGGAATCTTCCACAATGGGCGCAAGCCTGATGGAGCAACACCGCGTGAGTGAAGAAGG

GTTTCGGCTCGTAAAGCTCTGTTGTTGGTGAAGAAGGATAGAGGTAGTAACTGGCCTTTA

TTTGACGGTAATCAACCAGAAAGTCACGGCTAACTACGTGGGGGCAGCCGCG

>901 len=161 tot-seq=36

TGGGGAATATTGGACAATGGGGGGAACCCTGATCCAGCCATGCCGCGTGTGTGAAGAAGG

CCTTTTGGTTGTAAAGCACTTTAAGCGAGGAGGAGGAGCTCTTGGTTAATACCCAAGATG

CTTGGACGTTACTCGCAGAATAAGCACCGGCTAACTCTGTG

>902 len=135 tot-seq=35

TGGGGAATATTGGACAATGGGCGCAAGCCTGATCCAGCCATGCCGCGTGAGTGATGAAGG

CCTTAGGGTTGTAAAGCTCTTTTGCCGGGGACGATAATGACGGTACCCGGAGAATAAGTC

CCGGCTAACTTCGTG

>903 len=123 tot-seq=35

GAACAGAAAGGATCATGGGAGGGAAGCAGGACAGCCACTGCATCAGCATGGCATACCTTC

AGCCCCGCTCCAGTCAATCTGATGACATCAGAACTGTCCGTCTCCCTTCCTACCCCTGAC

CCT

>904 len=230 tot-seq=35

TTCTTACTGTGCCTAATTTATACATTAAATATTATCATGGTTATGTATGTGTAGGAAAAA

CCAGTGGGTGATTCGGTACTATTTGTGGTCTCAGGCATTCACTGGGGGTGTCTTAGAATG

TTATCTCCCATAGTTAATGGGGAACTACTGCACTTATTTTGTTGTAACACAACACAGCCT

CTCTAGCTCTTCAGTTAAAACTTTTCAGTTTAGAATAAAACACCCTATCA

>905 len=138 tot-seq=35

TGGGGAATATTGGGCAATGGGGGAAACCCTTACCCAGCAACGCCGCGTGAAGGAAGAAGG

TTTTCGGATTGTAAACTTCTGTCCTAAGAGAAGAAGAAAGTGACGGTATCTTAGGAGGAA

GCCCCGGCTAACTACGTG

>906 len=161 tot-seq=35

TAGGGAATCTTCCACAATGGACGCAAGTCTGATGGAGCAACGCCGCGTGAGTGAAGAAGG

TCTTCGGATTGTAAAACTCTGTTATACGGGACGAAAAAGACGGATGCCAACAGTATCCGT

CCGTGACGGTACCGTAAGAGAAAGCCACGGCTAACTACGTG

>907 len=121 tot-seq=34

GTAGGGTTTGACCTGGCAGGTTCATCTGAAACCAGTTTGTTTTCTTTACAGAGCACATGC

CTGGCTGCCTGAAGCAGTTGACCAGAACTTGCAGCCAAACCACGTGGTTTTGGTCAAATT

C

>908 len=136 tot-seq=34

TGGGGAATCTTGCGCAATGGGGGGAACCCTGACGCAGCGACGCCGCGTGCGGGACGAAGG

CCCTCGGGTCGTAAACCGCTTTCAGCAGGGATGAGACAAGACGGTACCTGCAGAAGAAGC

CCCGGCTAACTACGTG

>909 len=138 tot-seq=34

TGGGGAATATTGGGCAATGGGCGAAAGCCTGACCCAGCAATGCCGCGTGAAGGACGAAGG

TCTTCGGATTGTAAACTTCTTTGATCAGGGACGAATAAATGACGGTACCTGAAGAACAAG

CCACGGCTAACTACGTGG

>910 len=161 tot-seq=34

TAGGGAATCTTCCGCAATGGGCGAAAGCCTGACGGAGCGACGCCGCGTGAGCGAAGAAGG

CCTTCGGGTCGTAAAGCTCTGTTGTGAGGGACGAAGGAGCGCCGTTCGAAGAGGGCGGCG

CGGTGACGGTACCTCACGAGAAAGCCCCGGCTAACTACGTG

>911 len=160 tot-seq=34

TAGGGAATCTTCCGCAATGGACGAAAGTCTGACGGAGCAACGCCGCGTGAGTGATGAAGG

TTTTCGGATCGTAAAGCTCTGTTGTTAGGGAAGAACAAGTGCAAGAGTAACTGCTTGCAC

CTTGACGGTACCTAACCAGAAAGCCACGGCTAACTACGTG

>912 len=161 tot-seq=34

TGGGGAATCTTCCACAATGGGCGAAAGCCTGATGGAGCAACGCCGCGTGAGTGAAGAAGG

TCTTCGGATCGTAAAGCTCTGTTGCACGGGACGAAAACCGAGCTTGAGAATATTGAGTTT

GGGTGACGGTACCGAGCGAGGAAGCCACGGCTAACTACGTG

>913 len=225 tot-seq=34

GTGGCTCTTCTTCAGGGCTTCATTTAGGCCTGTGCTTTGGGCCCCAAGTCTGGGTACAAC

CTTCGCAGAGACAGAATCATGACTTCCCATCTATTCTCCTAGGGATGTGCCAGGGTCCAG

GGCCCTGGCTGGCTAATAGTAGAGACAACCTGATGGTGGGTTTGCCTCCTGACTGTGGGT

AGAAGGAGGTCAGGAGCAACTTTTCAGGGCCTCTTACATTCTTGT

>914 len=137 tot-seq=34

TGAGGGATATTGGGCAATGGGGGAAACCCTGACCCAGCAACGCCGCGTGAGGGAAGAAGG

TCTTCGGATTGTAAACCTAAGTAGTCAGGGACGAAGAAATGACGGTACCTGAAGAGTAAG

CTCCGGCTAACTACGTG

>915 len=196 tot-seq=34

TCCGAGGAATTTTGATGGTTTTTGGACAGCAAGTCAGAATATTTGGAATTGGGACTATTT

GGGGGTTTAAATTGTAAAGAAGAGCTTGAGAGGGAATGTTGAGGCACAAGGACAGATGAA

CTATAGGTAGCTGTAGACAAAATTCAAGTCAGTTTTAAAAATGATGAGTTGAAAATAAAC

CATGAAACTGAATTAG

>916 len=155 tot-seq=34

TGAGGAATATTGGTCAATGGGCGAGAGCCTGAACCAGCCAAGTAGCGTGCAGGAAGACGG

CCCTATGGGTTGTAAACTGCTTTTATAAGGGAATAAAGTGAGCCTCGTGAGGCTTTTTGC

ATGTACCTTATGAATAAGGACCGGCTAATTCCGTG

>917 len=116 tot-seq=34

TAGGGAATCTTCCACAATGGACGCAAGTCTGATGGAGCAACGCCGCGTGAGTGAAGAAGG

GTTTCGGCTCGTAAAGCTCTGTTGTTGGGGAAGAAGGACAGGGGGAGTAACTTAAA

>918 len=160 tot-seq=34

TGGGGAATATTGCGCAATGGGGGAAACCCTGACGCAGCGACGCCGCGTGCGGGATGAAGG

TCTTCGGATTGTAAAGTGCTGTTATAAGGGAAGAACATTTGCAATAGGAAATGATTGCAG

ACTGACGGTACCTTGTCAGAAAGCGATGGCTAACTATGTG

>919 len=134 tot-seq=33

GACCCAGCTCTCCCAGGTAGCAGGAAAGAGGCCACTGGTAATTAGGGACCAGATTTGAAT

GTGATGTGGAGAAGCCATGTGAAAGTACCCCCAGAGGAGGGCAGGCATGGCCCCTGGTCC

CCAGTTCCATTTGC

>920 len=135 tot-seq=33

TGGGGAATATTGCACAATGGGGGAAACCCTGATGCAGCAACGCCGCGTGAGTGATGACGG

TCTTCGGATTGTAAAGCTCTGTCTTTAGGGACGATAATGACGGTACCTAAGGAGGAAGCC

ACGGCTAACTACGTG

>921 len=142 tot-seq=33

TGAGGAATATTGGTCAATGGACGAGAGTCTGAACCAGCCAAGTAGCGTGCGGGATGAAGG

CCTTCGGGTTGTAAACCGCTTTCAGCAGGGACGAGGCCGCAAGGTGACGGTACCTGCAGA

AGAAGCCCCGGCTAACTACGTG

>922 len=143 tot-seq=33

TGGGGAATATTGCACAATGGGCGCAAGCCTGATGCAGCCATGCCGCGTGTATGAAGAAGG

CCTTCGGGTTGTAAACCGCTTTTGATTGGGAGCAAGCTTTCGGGTGAGTGTACCTTTCGA

ATAAGCGCCGGCTAACTACGTGG

>923 len=157 tot-seq=33

AAACCACACTGCTCAGCTTCCCGGGAGGGAAAGGTAAGGAGAAGGCCCAGGTATGGACGC

TGCATCGCCTGTCATCCTGGAAGCCCTTTCCCAAAGCTCATGATGGGCTGGCCAGAGGAA

GCTACCATAGCCCCGCCAGAGTCTGCTCACTCTACCA

>924 len=161 tot-seq=33

TAGGGAATCTTCCGCAATGGACGAAAGTCTGACGGAGCAATACCGCGTGAGTGATGAAGG

TCTTCGGATCGTAAAACTCTGTTGTTAGGGAAGAACAAGTGCTAATCTAATACATTAGTG

CCCTGACGGTACCTAACCAGAAAGCCCCGGCTAACTACGTG

>925 len=197 tot-seq=33

GATATTAAGATATAATAAGGGAGGAAGAAGGGGGGAATCTTCCACAATGGACGCAAGTCT

GATGGAGCAACGCCGCGTGAGTGAAGAAGGGTTTCGGATAGTAAAGATATGTTGTTGGTG

AAGAAGGAAAGGGGTAGTAAATGAAATTTGTTTGACGGTAATCAATTAGAAAGTCACGGC

TAACTACGTGCTACGTG

>926 len=176 tot-seq=33

GCAGGGTACATGTGCATTGGCTGAGGTGGGGTCCTGGTGGGAGCAAGGCTGCTATGTTCC

TGTGCATGCTCATGTTGGTGGTGATGATGGCAGTGGCAGAATGCTGGATGGATCAGGGCT

TCTAGTCTCTGTGTGTGAGTTTGCAGCCATGATGGTGGCTGCATGGGAGGAGGTTG

>927 len=205 tot-seq=33

TGGGGAATATTGCACAATGGGCGCAAGCCTGATGCAGCCATGCCGCGTGTATGAAGAAGG

CCTTCGGGTTGTAAAGTACTTTCAGCGAGGAGGAAGGCATTAAGGTTAATAACCTTGGTG

ATTGACGTTACTCGCAGAAGAAGCACCGGCTAACTCCGTGGGGGGAGCCGCGGTAATGCA

GAGATCGGAAGAGCGTCGTGGGGGG

>928 len=138 tot-seq=33

TGGGGAATATTGGGCAATGGGCGAAAGCTTGACCCAGCGACGCCGCGTGAGGGAAGAAGG

TCTTCGGATTGTAAACCTCTTTCAGCAGGGAAGAAGAAAGTGACGGTACCTGCAGAAGAA

GTCACGGCTAACTACGTG

>929 len=160 tot-seq=33

TGGGGAATATTGGACAATGGGCGAAAGCCTGATCCAGCCATGCCGCGTGTGTGAAGAAGG

CCTTCGGGTCGTAAAGCACTTTAAGTTGGGAGGAAGGGCTCATAGCGAATACCTGTGAGT

TTTGACGTTACCAACAGAATAAGCACCGGCTAACTTCGTG

>930 len=218 tot-seq=33

CAACTTAACACAACTTAAAACTAGACCCGTGTCTGACTGTCAGCGAGAGCACTGCCAGAG

AAGCTAGAGAGAGAGGAAACCCACCACGAATGTGGCAGAACTATCCTGTGAAGTGGGGTT

CGAGACCGAAATAAAGAGGGGCAATGAGAAAGCCAGGTGAGTACTCTTTCTCTGCTTCCT

GATCTGCAAAGATGCGAGCAGGCAGCCTCATGGAAACT

>931 len=199 tot-seq=33

TGAGGAATATTGGTCAATGGACGAGAGTCTGAACCAGCCAAGTAGCGTGAAGGATGACTG

CCCTATGGGTTGTAAACTTCTTTTATATGGGAATAAAACGTTCCACGTGTGGGATTTTGT

ATGTACCATATGAATAAGGATCGGCTAACTCCGTGCCAGCAGCCGCGGTAATAAGAAGAT

CGGAAGAGCGTCGTGTAGG

>932 len=155 tot-seq=33

TGAGGAATATTGGTCAATGGGCGAGAGCCTGAACCAGCCAAGTCGCGTGAGGGACGACGG

CCCTACGGGTTGTAAACCTCTTTTGCCGGGGGACAAACGGCGGCACGCGTGCCGTCCTGA

GGGTACCCGGAGAAAAAGCATCGGCTAACTCCGTG

>933 len=140 tot-seq=33

TGGGGAATATTGCACAATGGGCGGAAGCCTGATGCAGCGACGCCGCGTGGGGGATGACGG

CCTTCGGGTTGTAAACCTCTTTCGACAGGGACGAAGTGAAAATGACGGTACCTGTATAAG

AAGCACCGGCTAACTACGTG

>934 len=144 tot-seq=33

GGGGAGAGGCAGCAGCAAGGTGAGAGGCAGCAGCAAGGTGAGAGGCAGCAGCAAGGTGAG

AGGCAGCAGCAAGGAGAGAGGCAGCAGCAAGGAGAGAGGCAGCAGCAAGGAACAGAGCCG

GCTCCTCAGGGCTAGGGACATGGA

>935 len=154 tot-seq=33

TGGGGAATATTGCACAATGGGCGCAAGCCTGATGCAGCGACGCCGCGTGAGGGATGAAGG

CCTTCGGGTTGTAAACCTCTTTCGTCAGGGAACAAGGCACTGCTTTTTGGTGGTGTTGAG

GGTACCTGGATAAGAAGCGCCGGCTAACTACGTG

>936 len=161 tot-seq=33

TGGGGAATATTGGACAATGGGGGGAACCCTGATCCAGCCATGCCGCGTGTGTGAAGAAGG

CCTTTTGGTTGTAAAGCACTTTAAGCGAGGAGGAGGCTACCTAGATTAATACTTTAGGAT

AGTGGACGTTACTCGCAGAATAAGCACCGGCTAACTCTGTG

>937 len=161 tot-seq=33

TAGGGAATCTTCGGCAATGGGGGCAACCCTGACCGAGCAACGCCGCGTGAGTGAAGAAGG

TTTTCGGATCGTAAAGCTCTGTTGTTAGAGAAGAATGATGGTGGGAGTGGAAAATCCACC

AAGTGACGGTAACTAACCAGAAAGGGACGGCTAACTACGTG

>938 len=136 tot-seq=32

TGAGGAATCTTGGACAATGGGCGAAAGCCTGATCCAGCCATGCCACGTGAGGGATGAAGG

CTAAATAGGTTGTAAACCTCTAACTCTAGCTTAGATTATGACATGGGGTAGGGGAAAAGT

CCCGGCTAACTCCGTG

>939 len=166 tot-seq=32

TCTGGTGTTGGAATGGCCAACTAGCCAAAAACACCTGTAGAGGCAAACCTGCGGGTTGCA

GGTTGAGGGCAGGCCGTCAGGACACTCTCCTCACCACTACCACCTCCCCAGCCCCAGCCC

ACATGCTATCCTCCCCTTCACTGTGTGCAAGGTGCCCAAGGGTCTG

>940 len=179 tot-seq=32

TGGGGAATATTGGACAATGGGCGCAAGCCTGATCCAGCAATGCCGCGTGAGTGATGAAGG

CCTTCGGGTCGTAAAGCTCTTTTACCAGGGATGATAATGACAGTACCTGGAGAATAAGCT

CCGGCTAACTCCGTGCCAGCAGCGCGGTAATCATCAGATCGGAAGAGCGTCGTGTAGTG

>941 len=183 tot-seq=32

CTCCCCGACGCCTGTGCTTCCACAGCGCCAGTGCTCTGCCAGCTCCTGCACTCACAATCT

CGTCCTAGGTGACCCAGTCCCCACCCAGGCTCCTGCCATCACCCAGCGCCAGCAACTCTC

ACAGCTCCTCTCTAGCCTGGGCCTTCCCTCTACGCTCCAGGCCAGCTCCCCCAGCACGTG

GTG

>942 len=192 tot-seq=32

GGGTTGTGGGCCTGGAGCCCACTGCTGGACCCCAACACATACCACCCCATTTTAAACCAG

CCCTTGTCAAGGTCCCGTAGCTTAAGTCATTGACTTATGGCACCAAAGTGTATTTTTAAA

TTCTTACTGACCCAAAACCAGTTCTGAATAAACAGGCAGTTCTCAGGGTGGTTAGCAGAC

ACCCGCTCCACC

>943 len=140 tot-seq=32

TGGGGAATATTGCACAATGGGGGGAACCCTGATGCAGCGACGCCGCGTGCGGGATGACGG

CCTTCGGGTTGTAAACCGCTTTCAGTAGGGAAGAAGCGAAAGTGACGGTACCTGCAGAAG

AAGTACCGGCTAACTACGTG

>944 len=160 tot-seq=32

TGGGGAATCTTGGACAATGGGGGCAACCCTGATCCAGCAATGCCGCGTGGGTGAAGAAGG

CCTTCGGGTTGTAAAGCCCTTTCGGCAGGGAAGAAATCGGCGGGGCGAATAACCCTGCTG

GATGACGGTACCTGCAGAAGAAGCACCGGCTAACTACGTG

>945 len=135 tot-seq=32

TAGGGAATTTTGCGCAATGGGGGAAACCCTGACGCAGCAACACCGCGTGAATGTTAAGCC

CTTCGGGGTGTAAAGTTCTGTCAATAGGGACGAATTTTGACTGTACCTATAAAGGAAGCA

CCGGCTAACTCCGTG

>946 len=135 tot-seq=32

TGGGGAATATTGCACAATGGGCGAAAGCCTGATGCAGCAACGCCGCGTGAGTGATGACGG

CCTTCGGGTTGTAAAGCTCTGTCTTTGGGGACGATAATGACGGTACCCAAGGAGGAAGCC

ACGGCTAACTACGTG

>947 len=155 tot-seq=32

TAGGGAATTTTCGGCAATGGGGGCAACCCTGACCGAGCAACGCCGCGTGAGTGATGAAGG

TCTTCGGATTGTAAAGCTCTGTTGTAAGGGAAGAACGGCATCTAGAGAATAGATGAGTGA

CGGTACCTTACCAGAAAGCTACGGCTAACTACGTG

>948 len=226 tot-seq=32

GAGGCTGTGGGGCAGGTGACCGACCACACAGGGTCTAGTTACATAGCCTAGGGTGAACAG

GCTTGGGGACTGTTCACCCCCTCACTTCACTAAACAAATTTGTGCCCTACCAAGAAACTC

ACACCTACCTAAACCTCTAAAGGTAGCTGCATTAAAAACTGGGGTCTCTGCCGAGTCAGC

TAAAGGTCTGAAACATGAGGGCTGGACCTAAGGAGAGGGATGTGGG

>949 len=137 tot-seq=32

CGAGGAATCTTGGGCAATGGGCGAAAGCCTGACCCAGCGACGCCGCGTGAGGGATGAAGG

CCTTCGGGTCGTAAACCTCTTTTGTGAGGGAAGAAGTTCTGACGGTACCTCACGAATAAG

CCACGGCTAACTACGTG

>950 len=194 tot-seq=32

CACATGGTAGGCAGGACATTTCAATTGGTCCGATCTTTCTCTGTAGGGGAATGTCCAGTG

TATTAGAGAAGGAGGCGGGCAGGAGGCTCTGCGGAAGAAAATGAGAGTGGGGATTGGGGA

AGGCAGGCTAGGGCAGCCCTGTGGCTTGGGAAGCAGGGAACTGGGATGATCAGAACCATT

TTAAGAAGATTAAG

>951 len=160 tot-seq=32

AAGACAAGAAGGAGAAAGAAAAAAAGGGGATGTGCAGAGAGGGAAGAGTGACCAGAGACG

GCTTGAAGGACTAAGTTGAACAGAATAATACCCTTAAAACACCCTGATTCTGAGACAGAT

GAAAGGAGTTCAAAAACAAAAACAAAAAAGTCAAAATGTA

>952 len=159 tot-seq=32

TGGGGAATATTGGACAATGGGGGCAACCCTGATCCAGCAATGCCGCGTGTGTGAAGAAGG

CCTGCGGGTTGTAAAGCACTTTCAGAGGGGAAGAAAAAATCGGAATTAACACTTCCGAGC

TTGACGTTACCCTTAGAAGAAGCACCGGCTAACTCCGTG

>953 len=156 tot-seq=32

TGAGGAATATTGGTCAATGGACGTAAGTCTGAACCAGCCAAGTAGCGTGCAGGATTGACG

GCCCTATGGGTTGTAAACTGCTTTTGTTGGGGAGTAAAGTGAGGCACGCGTGCCTTTTTG

CATTTACCCTTCGAATAAGGACCGGCTAATTCCGTG

>954 len=159 tot-seq=32

TAGGGAATCTTCGGCAATGGACGGAAGTCTGACCGAGCAACGCCGCGTGAGTGAAGAAGG

TTTTCGGATTGTAAAGCTCTGTTAATCGGGACGAATGGTCGGTGTGCAAATAGTGCATGG

ACATGACGGTACCGGAATAGAAAGCCACGGCTAACTACG

>955 len=160 tot-seq=32

TGGGGAATATTGCACAATGGGGGAAACCCTGATGCAGCCATGCCGCGTGTGTGAAGAAGG

CCTTCGGGTTGTAAAGCACTTTCAGCGAGGAGGAAAGGTGGTTGGCTAATACCCAATCGC

TGTGACGTTACTCGCAGAAGAAGCACCGGCTAACTCCGTG

>956 len=162 tot-seq=32

GGCTTCTCGGTACAGAAAATGGTGAACTCTCAGTCTTCCTGCCCTGGCCATGCTCAGCTG

AACAAAACCACACCAGCTGGTCTCACTTAAATTCAGGATTACCATCGGAGGCGGGGGTTG

TGGGGAGGCTCTTAACGCTGCCAGAACCCCAAATGACACTTC

>957 len=196 tot-seq=32

TCAGACCTGGGGTGACAGGGGACAAATGCAGGGAGGGGATCTCGTCAGTGGATTCTGAGA

GCCCCAGGAGTCTCAGATGGAGGGCATGTCTTCAGAGCCCTTTGTCGTGAAACACAGGAA

TGGTTGTAATGCCAGAGTCAGCTATGCCCAACTTGGTGAAGCAAGATGCAGTGAGGGCCT

CTGAGGGAACTTAACC

>958 len=142 tot-seq=32

TGAGGAATATTGGTCAATGGGGGAAACCCTGACGCAGCGACGCCGCGTGCGGGATGAAGG

CCTTCGGGTTGTAAACCGCTTTTGATTGGGAGCAAGCCTTCGGGTGAGTGTACCTTTCGA

ATAAGCGCCGGCTAACTACGTG

>959 len=154 tot-seq=31

CCCTCCTCTTTTCTTTCTTTCCATTTTTTAAAAATAACCCATTGAGTCCAGTTGGTGCTG

CCCATATGTACATGGGTGTCGGGTCACCCGCTGGGATGAACAGCACACTGAAGACCACAC

CCCTAAAGCAAAACAGCCTCCCCTCCCCCAATAG

>960 len=160 tot-seq=31

TGGGGAATTTTGGACAATGGGCGCAAGCCTGATCCAGCCATGCCGCGTGAGTGAAGAAGG

CCTTCGGGTTGTAAAGCTCTTTCGGCCGGAACGAAATCGCATCCTCTAATACGGGGTGTG

GATGACGGTACCGGAAGAAGAAGCACCGGCTAACTACGTG

>961 len=135 tot-seq=31

TGGGGAATATTGCACAATGGAGGAAACTCTGATGCAGCGACGCCGCGTGAGTGAAGAAGT

ATCTCGGTATGTAAAGCTCTATCAGCAGGGAAGAAGATGACGGTACCTGCAGAAGCAGCC

CCGGCTAACTACGTG

>962 len=160 tot-seq=31

TGGGGAATTTTGGACAATGGGGGAAACCCTGATCCAGCAATGCCGCGTGAGTGAAGAAGG

CCTTCGGGTTGTAAAGCTCTTTTGTCAGGGAAGAAATTGATTGGGCTAATATCCCGATTA

GATGACGGTACCTGAAGAATAAGCACCGGCTAACTACGTG

>963 len=182 tot-seq=31

GAAGTGCGGAGGGGTGGGACAATTTCTTCTCTTAGGGAACTTTATTCCTTGAGGGTGTCT

TGTGCAGTTGAGGCTGTTTCCCTTTAGAGGGGATCAGCATGGAACAAATGAGCTGGTGTG

GACACAGCCGCGAGGAATGTGGTTGGAACAGGGAAGCCCAGCCCTCAAGTACCAAGGAGA

CC

>964 len=144 tot-seq=30

TGAGGAATATTGGTCAATGGACGCAAGTCTGAACCAGCCAAGTAGCGTGCAGGATGACGG

CCTTCGGGTTGTAAACCGCTTTTGATTGGGAGCAAGCTTTCGGGTGAGTGTACCTTTCGA

ATAAGCGCCGGCTAACTACGTGTG

>965 len=154 tot-seq=30

TAGGGAATATTGGGCAATGGTCGGAAGACTGACCCAGCCATGCCGCGTGCAGGAAGAAGG

CGTTATGCGTTGTAAACTGCTTTTATACGGGAAGAAAAAGCAACTGCGGTTGAAATTGCC

GGTACCGTATGAATAAGCACCGGCTAACTCCGTG

>966 len=156 tot-seq=30

TGAGGAATATTGGTCAATGGACGCGAGTCTGAACCAGCCAAGTAGCGTGCAGGATGACGG

CCCTATGGGTTGTAAACTGCTTTTGCGCGGGGATAACACCCTCCACGTGCTGGAGGTCTG

CAGGTACCGCGCGAATAAGGACCGGCTAATTCCGTG

>967 len=181 tot-seq=30

GACCAACCTGAGCCACCAACCTCTCACCCTTCCCAGCAGGCAGGCCTGGCCTTCACATTT

GGCCCCTCTCCGGAGGGACAGATGACAGGTTCCACGTGCCTGTCATTTTCCCAGCTGCAG

CTGAGGGTGAGACATTGCCCCCCCAGGCCATACACTCACTCCCCAGCACCCCCTTCCCCA

A

>968 len=192 tot-seq=30

GTGGAGAGCTCTCTGGCCCTGGCCCCAATCCCACCACCCTGAGCTCACATCCTCAGGAAG

GAGGGCATCACAGCGTGAAGCCGTGTGTGCGCAAGGTGCCTTCCACGGTGCAGGATGGGA

GGAGCCGTATGCTCGTTGCTCCAGAGCCGCGGTAATAAGTAGATCGGAAGAGCGTCGTGT

AGGGAAAGAGTG

>969 len=207 tot-seq=30

AATATCTCTATGTCCATCAGTCTATACACACAAAAGCAACCGTGTGGTACATCTATTCAA

TGGGATACTACTAAGATTTGTAAAGACATGTCTCTGGACACATGCCACAGTGGAACCCAA

GAACATCATATGCTATGTGAAAGAAGTCAGCCCCAAAGGCCGTGTGTATTGTATGATCTG

CTCCTACAGGAGTGGGAGAGTTAGAGA

>970 len=160 tot-seq=30

TAGGGAATCTTCCGCAATGGACGCAAGTCTGACGGAGCAACGCCGCGTGAGTGAGGAAGG

CCTTCGGGTCGTAAAGCTCTGTTGCCAGGGAAGAACGGGTGGAAGAGTAACTGCTTCCGC

CATGACGGTACCTGAGAAGAAAGCCCCGGCTAACTACGTG

>971 len=154 tot-seq=30

TGAGGAATATTGGTCAATGGGCGGGAGCCTGAACCAGCCATGCCGCGTGAAGGCGAGTGC

CCTACGGGCGTTAAACTTCTTTTGTGCGGGAGCAATAAGGGCCACGCGTGGCCCGATGAG

AGTACCGCACGAATAAGCATCGGCTAACTCCGTG

>972 len=176 tot-seq=30

GAAGAGGGGGCATTCCCATCCAGTGGCCTCTCTTTTCTCAGTAAAGGAGAGGCAAGTTGG

CCTACTGCCTGTGTCCCAGGGTCAGAAGGCTTGCCAAACCTATTCAGGGGCCTGTTGCAG

TGAGAGGCCTTGCATTTACAGTAGCACCACTCTTTGGGGAAGTCCACCAGCAGCTC

>973 len=138 tot-seq=30

TGGGGGATATTGCACAATGGGCGCAAGCCTGATGCAGCAACGCCGCGTGAGGGAGGACGG

TCTTCGGATTGTAAACCTCTGTCCTTGGGGAAGAAACAAATGACGGTACCCTTGGAGGAA

GCCACGGCTAACTACGTG

>974 len=154 tot-seq=30

TAGGGAATATTGGTCAATGGGTGAGAGCCTGAACCAGCCATGCCGCGTGCAGGAAGAAGG

CCTTCTGGGTTGTAAACTGCTTTTGCCAGGGGATAAAAATACCTTGCGAGGTAAATTGAA

GGTACCTGGTGAATAAGCCACGGCTAACTACGTG

>975 len=159 tot-seq=30

TGGGGAATCTTGCGCAATGGGCGAAAGCCTGACGCAGCGACGCCGCGTGGGGGAAGAAGG

CCTTCGGGTTGTAAACCTCTTTCAGGAGGGACGAAGCCACTCGGGTGAATAGCCCAGAGG

GTGACGGTACCTCCAGAAGAAGCCCCGGCTAACTACGTG

>976 len=138 tot-seq=30

TGGGGAATATTGGGCAATGGAGGCAACTCTGACCCAGCAACGCCGCGTGAAGGAAGAAGG

CCTTCGGGTTGTAAACTTTTGTTGTAGAGGACGAAGAAAGTGACTGTACTCTACGAGGAA

GCCCCGGCTAACTATGTG

>977 len=156 tot-seq=30

TGGGGAATATTGCACAATGGGCGCAAGCCTGATGCAGCGACGCCGCGTGAGGGATGGAGG

CCTTCGGGTTGTAAACCTCTTTCGCCAGTGAAGCAGGCCATCTTCTTTGTGGGGTGGTTG

ACGGTAGCTGGATAAGAAGCGCCGGCTAACTACGTG

>978 len=229 tot-seq=30

GGGGGGGGGGAAGCAGGTGCTGAACAGACAGACCCAGGGCTTGGTGGGAGGTGCAAAATC

CAACCCCAAACAGCAGACGGTGGCTTCTGGGGGTCCCGGGATGGTAAATTCTGGATCAGG

CTTTACCCTGGGCCTCAGGCAGAGAGCAGCAGGGAGGGGGGATCTGGAGCAGGTAAGGCA

CACCCAGGCCCTGGGCCAAAGGATTCTTGTGCATCAGCCTCTAACCTGT

>979 len=142 tot-seq=30

TAGGGAATCTTCGGCAATGGGGGCAACCCTGACGCAGCGACGCCGCGTGCGGGATGAAGG

CCTTCGGGTTGTAAACCGCTTTTGATTGGGAGCAAGCTTTCGGGTGAGTGTACCTTTCGA

ATAAGCGCCGGCTAACTACGTG

>980 len=160 tot-seq=30

TAGGGAATCTTCCACAATGGGCGAAAGCCTGATGGAGCAACGCCGCGTGGGTGAAGAAGG

TCTTCGGATCGTAAAACCCTGTTGTTAGAGAAGAACACGAGTGAGAGTAACTGTTCATTC

GATGACGGTATCTAACCAGCAAGTCACGGCTAACTACGTG

>981 len=160 tot-seq=30

TGGGGAATATTGCACAATGGGCGCAAGCCTGATGCAGCCATGCCGCGTGAATGAAGAAGG

TTTTCGGATCGTAAAGCTCTGTTGTTGGTGAAGAAGGATAGAGGTAGTAACTGGCCTTTA

TTTGACGGTAATCAACCAGAAAGTCACGGCTAACTACGTG

>982 len=160 tot-seq=30

TAGGGAATCTTCCGCAATGGACGAAAGTCTGACGGAGCAACGCCGCGTGAGCGAAGAAGG

TCTTCGGATCGTAAAGCTCTGTTGTTAGGGAAGAACAAGTATCGGAGGAAATGCCGGTAC

CTTGACGGTACCTGACGAGAAAGCCACGGCTAACTACGTG

>983 len=116 tot-seq=29

TTTGAAGAAAGTTTGCCATTTGCGTTGACGGAAGGCCAAAAAAACGTTATTAGCGAGATT

ACGGAAGATATGCAAGGCGCGCAGGGCGAATCGCAAGCAACCGACGAATCGGAAGA

>984 len=116 tot-seq=29

AGGCTCAAGTGATGAGCTAACCTTGATAAGGTACACAATGCCGACTAGAAAATGCTATTT

ATTCAGAAATGGTTTGAATCACTGTAGTTCTACTATCACACAACTTTAAATAGTAG

>985 len=135 tot-seq=29

TAGGGAATATTGGGCAATGGAGGGAACTCTGACCCAGCAACGCCGCGTGAAGGAAGAAGG

TTTTCGGATTGTAAACTTCTATCCTTGGTGAAGATAAGGACGGTAGCCAAGAAGGAAGCC

CCGGATAACTACGTG

>986 len=139 tot-seq=29

TTCATGAGCCCCTCCACCCAGGTTCCATCCCTAGAGCCACTAAAAAGTTAAGACAAAGGA

CGTGGAGAGAATCTTGATTGGGATAATCAAGCAAGCATACAGCGTGCTTGGGATAATCAA

GCAAGCATACAGCGTGCTT

>987 len=153 tot-seq=29

TAGGGAATATTGCTCAATGGGGGAAACCCTGAAGCAGCAACGCCGCGTGGAGGATGACAC

TTTTCGGAGCGTAAACTCCTTTTGTTAGGGAAGAACAATGACGGTACCTAACGAATAAGC

ACCGGCTAACTCCGTGGACCGGCTAACTCCGTG

>988 len=187 tot-seq=29

AATCAGCTCTGGGGGGGAAACACCTGTGCTTGTAACTGATCTTGAAGTTTATGCAACCAG

GAGAACTGTCCAACTCAAGGCTTCAGGAAATATGCTGATATCTGGGCCTGCTTCCATCAG

TTTCATCTAATTGCTGGAAAAATCATGTCACCTAAGAAGGAGGTCCACTCCCCTTCCTGC

CCCACCC

>989 len=189 tot-seq=29

GGGCGAGACCGTGAAAGAACCATCCTGTGTGCTCAGCTGTGCACTTCCCTCACGTTCTTA

CTCTGAGACCAAGCTTCCTCCACTGTCCCTGGCCAGGCAAGAGCCCCACAATGGTCACCT

GTCACCATCCAGGAAACATCTCATGAAAACTCTGCTCAATTGTACCAAGAAAATACTCTG

AGCCAACAT

>990 len=198 tot-seq=29

TAAGGAATCTTCCACAATGGGCGAAAGCCTGATGGAGCAACGCCGCGTGCAGGATGAAGG

CCTTCGGGTTGTAAACTGCTTTTATAAGTGAGGAATATGACGGTAACTTATGAATAAGGA

TCGGCTAACTACGTGCCAGCACCGCGGTAATGCCAAGATCGGAAGAGCGTCGTGTAGGGA

AAAGTGTGCCAATGTGTA

>991 len=161 tot-seq=29

TGGGGAATATTGGACAATGGGGGGAACCCTGATCCAGCCATGCCGCGTGTGTGAAGAAGG

CCTTTTGGTTGTAAAGCACTTTAAGCGAGGAGGAGGCTTACCTGGTTAATACCTGGGATA

AGTGGACGTTACTCGCAGAATAAGCACCGGCTAACTCTGTG

>992 len=160 tot-seq=29

TGGGGAATTTTGGACAATGGGCGAAAGCCTGATCCAGCAATGCCGCGTGCAGGATGAAGG

CCTTCGGGTTGTAAACTGCTTTTGTACGGAACGAAAAGCTTCGGGTTAATACCCTGGAGT

CATGACGGTACCGTAAGAATAAGCACCGGCTAACTACGTG

>993 len=158 tot-seq=29

TGGGGAATTTTGGACAATGGGGGCAACCCTGATCCAGCCATTCCGCGTGAGTGAAGAAGG

CCTTCGGGTTGTAAAGCTCTTTCGGCAGGAACGAAACGGCGAGCTCTAACATAGCTCGCT

AATGACGGTACCTGAAGAAGAAGCACCGGCTAACTACG

>994 len=160 tot-seq=29

TGGGGAATATTGCACAATGGGGGAAACCCTGATGCAGCCATGCCGCGTGTGTGAAGAAGG

CCTTAGGGTTGTAAAGCACTTTCAGTCGGGAGGAAGGGGTCAGGTTTAATACACTTGGTC

ATTGACGTTACTGACAGAAGAAGCACCGGCTAACTCCGTG

>995 len=208 tot-seq=29

AAGCTGTTAGCTGACATCCTCTGACGCACCGCATACTCAGGCACAACATGAGATGTGTTA

CAGGAGCCCCACGGCCATCTGTACTTCAGGGAGATGAGGACAAGGAGGCATCTCCCGTGT

CAAAACAACCCCTGATAAATATCTCTTAGGGGAAAATGGCTTGACGGGGCTGTACGGTGT

CTCCCCTGACATGTAACCATCACTTCTC

>996 len=156 tot-seq=29

TGAGGAATATTGGTCAATGGGCGCAAGCCTGAACCAGCCAAGTCGCGTGAGGGAAGACGG

CCCTACGGGTTGTAAACCTCTTTTGCCGGGGAGCAAGTGAGAGCACGCGTGCCCTTACGG

AGAGTACCCGGAGAAAAAGCATCGGCTAACTCCGTG

>997 len=155 tot-seq=29

TGAGGAATATTGGTCAATGGACGGAAGTCTGAACCAGCCAAGTAGCGTGCAGGATGACGG

CCCTATGGGTTGTAAACTGCTTTTATGCGGGGATAAAGTTAGTCACGTGTGACTATTTGC

AGGTACCGCATGAATAAGGACCGGCTAATTCCGTG

>998 len=155 tot-seq=28

TGAGGAATATTGGTCAATGGGCGGTAGCCTGAACCAGCCAAGTAGCGTGAAGGATGAAGG

TTCTATGGATTGTAAACTTCTTTTATAAAGGAATAAAGTGAGGCACGTGTGCCTTTTTGT

ATGTACTTTATGAATAAGGATCGGCTAACTCCGTG

>999 len=161 tot-seq=28

TAGGGAATCTTCCACAATGGACGCAAGTCTGACGGAGCAACGCCGCGTGAGTGAAGACGG

CCTTCGGGTTGTAAAGCTCTGTGATCCGGGACGAAAGAGCCTGAGGTTAATAGCCTGAGG

AAGTGACGGTACCGGAAAAGCAAGCCACGGCTAACTACGTG

>1000 len=211 tot-seq=28

CACAAGGTTGGCCATGATCTGGTGAATACAGATTAGGCAGGAGAGCGGCCATGGAGAAAC

AGACTGGTGAGGCTGCAGACGTTTGCTCATCTTTGTTTTGACGCCTCTTGTCCCAAGCCT

CAGCCTTCTCCTGCTTTCCTTGACCTTCCTGCTGTTCCCTCATTGTCTTTCTCCTGCTTT

CCTTGACCTTCCTGCTGTTCCCTCATTGTCT

>1001 len=216 tot-seq=28

AAGAAAGAATTCACAGTTACTTTAGTGGCTGTAGCAAGGAGAATCTTGAGCTGAAGGCCA

GCCTGAGCTATAGAGCAAGCATAAAGTCAATCTGGGTAATTTATGAGACCCTGTCTCAAA
ATGAAAAGTATAGAAGGGTTGGAGATAAAGCTCAGTAGTAGACTGCTTGCCTAGCATGGC
ATTTGCAAGGATCCATGGCATATCCAGGTTCAATCC
>1002 len=223 tot-seq=28
TTCCGTAAGGCAGTGAGATTAGCACAGCTGGGCAGGAGAGGGATGGTTTTAGTCAGAACA
GTGGCCCAGCAGCCCCAGCAGCCCCAGCAGCCCCAGCAGCCCCCCCAGCCCCAGCAGTCA
TCTTGGCACCATTCCGTAAGGCAGTGAGATTAGCACAGCTGGGCAGGAGAGGGATGGTTT
TAGTCAGAACAGTGGCCCAGCAGCCCCAGCAGCCCCAGCAGCC
>1003 len=166 tot-seq=28
TGGGAGAGGTGATATGGAAATTAGAATGAACCCCCCTCATCTGGTGATTGACGTTTTGGC
CGAGCCTTGCTGTTGGAGGATGCACTAAGTAAATGACTTCTTGTGGTCACACTGAAATTC
AAGATTCAGGATCGCAGAAAATGGAAGCTTTGCCTGTGGTAACCAC
>1004 len=161 tot-seq=28
TAGGGAATCTTCCGCAATGGACGAAAGTCTGACGGAGCAACGCCGCGTGAGTGATGAAGG
CCTTCGGGTCGTAAAGCTCTGTTGTTAGGGAAGAACAAGTATTGTTCGAATAGGGCAGTA
CCTTGACGGTACCTAACCAGAAAGCCACGGCTAACTACGTG
>1005 len=137 tot-seq=28
TGGGGAATATTGGGCAATGGGCGCAAGCCTGACCCAGCAATGCCGCGTGAAGGAAGAAGG
CTTTCGGGTTGTAAACTTCTTTGACAGGGGAAGAGCAGAAGACGGTACCCTGAAAACAAG
CCACGGCTAACTACGTG
>1006 len=199 tot-seq=28
GACTCGGAAAGCGCTGAACACGAGAGGTAGAGGCAAGGAAGGGCTCCAAGGGCAGTGCCA
GGGAGCGCTGATGCTCTGGAAGGAAAGAGAAGATGGGCTGGATGTTGGTCTTGTTGCCAG
AGACACTGAATGGAAAGACTGTGTAGGAGTCAGTATGTTTGGAGCTCAGATATCTGGGTT
TCGGATGTACCTTAGGGAG
>1007 len=160 tot-seq=28
TAGGGAATATTGCACAATGGAGGAAACCCTGATGCAGCCACGCCGCGTGGGTGAAGAAGG
TCTTCGGATCGTAAAACCCTGTTGTTAGAGAAGAAAGTGCGTGAGAGTAACTGTTCACGT
TTCGACGGTATCTAACCAGAAAGCCACGGCTAACTACGTG

>1008 len=140 tot-seq=28

TGGGGAATATTGCACAATGGACGCAAGTCTGATGCAGCAACGCCGCGTGCGGGATGACGG

CCTTCGGGTTGTAAACCGCTTTCAGCAGGGACGAAGCGAAAGTGACGGTACCTGCAGAAG

AAGCACCGGCTAACTACGTG

>1009 len=160 tot-seq=28

TAGGGAATCTTCGGCAATGGGGGGAACCCTGACCGAGCAACGCCGCGTGAGTGATGAAGG

TCTTCGGATCGTAAAACTCTGTTATTAGGGAAGAACATATGTGTAAGTAACTGTGCACAT

CTTGACGGTACCTAATCAGAAAGCCACGGCTAACTACGTG

>1010 len=155 tot-seq=28

TGAGGAATATTGGTCAATGGGCGAGAGCCTGAACCAGCCAAGTAGCGTGCAGGAAGACGG

CCCTATGGGTTGTAAACTGCTTTTATGCGGGGATAAAGCGGGCGACGTGTCGCCCTTTGC

AGGTACCGCATGAATAAGGACCGGCTAATTCCGTG

>1011 len=154 tot-seq=28

TGGGGGATATTGCACAATGGGCGCAAGCCTGATGCAGCGACGCCGCGTGGGGGATGACGG

CTTTCGGGTTGTAAACCTCTTTCGTCGGGGGACAAGGCTCTGCTTGGTGGTGGGGTTGAG

GGTACCCGGTGAAGAAGCGCCGGCTAACTACGTG

>1012 len=157 tot-seq=28

TGAGGAATATTGGTCAATGGCCGAGAGGCTGAACCAGCCAAGTAGCGTGCAGGATGACGG

CCCTCGTGGTTGTAAACTGCTTTTAGTCGGGAATAAAAGGGGGTTCGTGAACCCCGTATT

GTATGTACCGTCAGAAAAAGGACCGGCTAATTCCGTG

>1013 len=211 tot-seq=28

TATGACCTTGCCCTAGCTCTTTGAGGTCCCAATTCCCCTGGGTCAGGAAAAGATCATCCC

TGAGAAAACAGAGGTCCTCATGGTTGACCATGCTGCAACTTGGAATGAGGCTCTCCTTGC

AGGACAGAGGGGGCAGCACATTAGGCCTGGATCCCAAAGTGCAGAGATTCCTTTTGGGAA

AGTTTAGGGCCAGATCCAGATGTGTGGTTCT

>1014 len=151 tot-seq=28

TGGGGAATATTGCACAATGGGGGAAACCCTGATGCAGCGACGCCGCGTGAGTGAAGAAGT

ATTTCGGTATGTAAAGCTCTATCAGCAGGGAAGAAAGGTGCCCCTTGGGCGCCTGGACGG

TACCTGACTAAGAAGCCCCGGCTAACTACGT

>1015 len=142 tot-seq=28

TGGGGAATCTTGCGCAATGGGCGCAACCCTGACGCAGCGACGCCGCGTGCGGGATGAAGG

CCTTCGGGTTGTAAACCGCTTTTGATTGGGAGCAAGCTTTCGGGTGGGTGTACCTTTCGA

ATAAGCGCCGGCTAACTACGTG

>1016 len=135 tot-seq=28

CTAGGCAGAATGGAGGGGGCGGGGCTTCTCTCTCATACCTCAGGGCTACTGAGGTCTCCT

GAGGGTGATTCATTTCTAGCTGCCACTGGAAGCAGAGGTCGCAGCCACAAAGCACTCAAT

TCTACTAAGAAATAT

>1017 len=160 tot-seq=28

TGGGGAATTTTGGACAATGGGGGCAACCCTGATCCAGCCATGCCGCGTGCGGGAAGAAGG

CCTTCGGGTTGTAAACCGCTTTTGTTCGGGAAGAAATCCTCTGGGCTAATACCCCGGGGG

GATGACGGTACCGGAAGAATAAGCACCGGCTAACTACGTG

>1018 len=155 tot-seq=28

TGAGGAATATTGGTCAATGGGCGAGAGCCTGAACCAGCCAAGTAGCGTGCAGGACGACGG

CCCTATGGGTTGTAAACTGCTTTTATGCGGGGATAAAGTGAGTCACGTGTGACTTATTGC

AGGTACCGCATGAATAAGGACCGGCTAATTCCGTG

>1019 len=129 tot-seq=27

TGGACAATGGGGGCAACCCTGATCCAGCCATGCCGCGTGAGTGACTTGAAGGCCTTAGGG

TCGTAAAGCTCTTTCGGCGGGGAAGATAATGACGGTACCCGCAGAAGAAGCCCCGGCTAA

CTTCGTGTG

>1020 len=133 tot-seq=27

GGGCAAGTCAGGATCTTCAACTTTGCACGTGGTTTGCAGACCCTTCCTCCTTCCTGACAA

AGACCCCCTCCTACCCCAGGCCAGGATATTCCACATCAGGGCAGAGGGAGGAGCATCCAC

GCCCAGGTCAGAC

>1021 len=135 tot-seq=27

TGGGGAATATTGGACAATGGGGGAAACCCTGATCCAGCCATGCCGCGTGAGTGATGAAGG

CCTTAGGGTTGTAAAGCTCTTTCGTCAGGGAAGATAATGACGGTACCTGAAGAAGAAGCC

CCGGCTAACTTCGTG

>1022 len=138 tot-seq=27

TGGGGAATATTGCACAATGGGGGGAACCCTGATGCAGCGACGCCGCGTGAGTGAAGAAGT

ATCTCGGTATGTAAAGCTCTGTCAGCAGGGAAGAAGAAAGACGGTACCTGACCAAGAAGC

CCCGGCTAACTACGTGTG

>1023 len=155 tot-seq=27

TGAGGAATATTGGTCAATGGATGGAAATCTGAACCAGCCAAGTAGCGTGCAGGATGACGG

CCCTATGGGTTGTAAACTGCTTTTATGTGAGAATAAAGTTAGGTATGTATACTTATTTGC

ATGTATCACATGAATAAGGACCGGCTAATTCCGTG

>1024 len=156 tot-seq=27

TGAGGAATATTGGTCAATGGGCGCGAGCCTGAACCAGCCAAGTAGCGTGGAGGACGACGG

CCCTACGGGTTGTAAACTCCTTTTGCGCGGGGATAAAGTGCGCGACGTGCTCGCGCATTG

CAGGTACCGCGCGAACAAGGACCGGCCAATTCCGTG

>1025 len=160 tot-seq=27

TAGGGAATCTTCCACAATGGACGAAAGTCTGATGGAGCAATGCCGCGTGAGTGAAGAAGG

TTTTCGGATCGTAAAACTCTGTTGTGAGGGAAGAACAAGTATGAGAGTAACTGCTCGTAC

CTTGACGGTACCTCATTAGAAAGCCACGGCTAACTACGTG

>1026 len=161 tot-seq=27

TGGGGAATATTGCGCAATGGGGGAAACCCTGACGCAGCGACGCCGCGTGAGTGATGACGG

CCTTCGGGTTGTAAAGCTCTGTTAATCGGGACGAATGGTCTTTGTGTGAATAATGCAAAG

ATTTGACGGTACCGGAATAGAAAGCCACGGCTAACTACGTG

>1027 len=224 tot-seq=27

CCAAGCCACATCCTCCCCGACAATCAGCGAGGCACACAGGCTTTCTGCATCCTGGCCTCC

TTGTCCAGAGTGGTATCGATCATGTCAGACACTGAAAATACCACCCTTAGGTCATGCATC

CGTCCCTCCCCATGCCACCTCCACAGAGGGACTGCATCCCAGAGACCAGCCACGTCCTCT

GCAATGTGCACCTAAACCTTCACCTGTGCACCTAAACCTTCACC

>1028 len=135 tot-seq=27

TGGGGAATATTGCACAATGGAGGAAACTCTGATGCAGCAACGCCGCGTGAATGATGAAGG

CCTTCGGGTCGTAAAGTTCTTTGATTGGGGACGATAATGACGGTACCCAAAAAACAAGCC

ACGGCTAACTACGTG

>1029 len=135 tot-seq=27

TGAGGGATATTCGGCAATGGGGGAAACCCTGACCGAGCAACGCCGCGTGAGGGAAGACGG

TTTTCGGATTGTAAACCTCTGTCCTCTGTGAAGATAATGACGGTAACAGAGGAGGAAGCT

CCGGCTAACTACGTG

>1030 len=120 tot-seq=27

TAGGTTCCCCAGGGAGAAGGGAAAGTTCGGCAAAGCCACCCTCAGCAACTCTGGAATGAG

ATATTCCCCCTCCAGATTGCCACAGCTGTCCGCAGCAATTGCCTTAGTCGGGGGCATCAG

>1031 len=168 tot-seq=27

GGGACCACAGATGCTAAACAGCCGGCCATTCAGGGTTACCCTGGCCAGAATTCTCCCTTA

AGCCTCAGCTTCCAGAGCTACAAATCAGAGCTAAATAAACCCTCCTTCCACTGGGGGTGG

CGGAGGTCACGTGTGTGTGTGCACGCATGTGTGAACGTGTGTCAGAGA

>1032 len=155 tot-seq=27

GGCAGGGAAGGTCACCCCCATTTTGCAGGTGGTAAAACCGAGGCTCAGGGGGTGACATTT

CTCAGCTATAGGAGCCAGGGATGAGCTTCCGGCTCAGGGCTTGAGATAGAGGAGAAGCCC

CCAGGTGAGGGCTGTCCTGAACCTACCAAGGGCCA

>1033 len=155 tot-seq=27

TGAGGAATATTGGTCAATGGGCGGGAGCCTGAACCAGCCAAGTCGCGTGAGGGATGACGG

TCCTACGGATTGTAAACCTCTTTAGCCGGGGAGTAACGTGTCCCACGCGTGGGGCAGTGA

GAGTACCCGGAGAATAAGCATCGGCTAACTCCGTG

>1034 len=135 tot-seq=27

TGGGGGATATTGCACAATGGGGGAAACCCTGATGCAGCGACGCCGCGTGAGTGAAGAAGT

TATTCGTAATGTAAAGCTCTATCAGCAGGGAAGAAAGTGACAGTACCTGACTAAGAAGCC

CCGGCTAACTACGTG

>1035 len=150 tot-seq=27

TGGGGGATATTGCACAATGGGCGAAAGCCTGATGCAGCGACGCCGCGTGGGGGATGAAGG

CCTTCGGGTTGTAAACTTCTTTTGCTCTGAACAAGGCGCACCGTGGGGTGTGTTGAGTGT

AGGGGTTGATTAGCGCCGGCTAACTACGTG

>1036 len=160 tot-seq=27

TGGGGAATCTTCCGCAATGGACGAAAGTCTGACGGAGCAACGCCGCGTGAGTGAAGACGG

CCTTCGGGTTGTAAAGTACTTTCAGCGGGGAGGAAGGGAGTAAAGTTAATACCTTTGCTC

ATTGACGTTACCCGCAGAAGAAGCACCGGCTAACTCCGTG

>1037 len=103 tot-seq=27

CAGGGGGAGAAGGTGAAATAAAGTGCCAGCTCATGACTGCAGAGTCCCTTGCTTATTCCT

GCATTCACCTCCTCTATTCTCACCATGTCATACCGGGACCATA

>1038 len=134 tot-seq=26

GCAGTAGCAGGATACTCAGTCACTCAGCAAAGACAAAGACCCACTGGCCTGCCCACGCTC

TCCTTGTTCCTTCCCACTTCTTCCCATCTGTTTGACAGTCGAGGATCCCTCCCCTAGTCA

AGCCTGTTCTCATC

>1039 len=135 tot-seq=26

TGGGGAATATTGCACAATGGGGGAAACCCTGATGCAGCAACGCCGCGTGAAGGAAGACGG

TCTTCGGATTGTAAACTTTTGTTCTTAGTGAAGATAGTGACGGTAGCTAAGGAGAAAGCC

ACGGCTAACTACGTG

>1040 len=155 tot-seq=26

TGAGGAATATTGGTCAATGGGCGCAAGCCTGAACCAGCCAAGTAGCGTGAAGGAAGACTG

CCCTATGGGTTGTAAACTTCTTTTATACGGGAACAAACGATAGTACGCGTACTATTTTGC

GTGTACCGTATGAATAAGCATCGGCTAACTCCGTG

>1041 len=156 tot-seq=26

TGGGGGATATTGCACAATGGGGGAAACCCTGATGCAGCGACGCCGCGTGAGTGAAGGAGC

GTTTCGGCGCGTAAAGCTCTATCAGCAGGGAAGACAAACTGCCCCGGAAGGGGCAGCCGG

ACGGTACCTGACCAAGAAGCCCCGGCTAACTACGTG

>1042 len=160 tot-seq=26

TAGGGAATCTTCCGCAATGGGCGAAAGCCTGACGGAGCAACGCCGCGTGTGCGAAGAAGG

TCTTCGGATCGTAAAGCACTGTTGTTAAGGAAGAACGACAGTAAGAGTAACTGCTTACTG

TGTGACGGTACTTAACCAGAAAGCCACGGCTAACTACGTG

>1043 len=160 tot-seq=26

TGGGGAATTTTGGACAATGGGCGCAAGCCTGATCCAGCCATGCCGCGTGAGTGAAGAAGG

CCTTCGGGTTGTAAAGCTCTTTCAGCCGGAAAGAAATTGCACTTATTAATACTAAGTGTA

GATGACGGTACCGGAAGAAGAAGCACCGGCTAACTACGTG

>1044 len=225 tot-seq=26

CTCTAGCTAGCTATTATATTTGTGATCTTCTTGACTGGCCCCCACACCAGTACTGGAACC

TTGCATACGTGCATGGGCCAGAATAACCCAGACAAGGAAGTGGGTTCCTGGAAGCATTTC

TGGGAGGCCACCTGCTATGTGGTGAAGGGAAAGTTGTCCTTGGGCACAAAGGCCCAATTT

AAACAAACAGAAGTTCCGAGATGAAGGGGCCCTGCCCTGAACTTC

>1045 len=232 tot-seq=26

GTGTGAGGGGACTCCATGCATTCCACGTGGCTCTGCACCTGACACTTAATGTCCCTTAGA

GAACTAAGGCCAGGCGGGCGGGCGGCGGACAGAGGGCACGCACCATCCCATAGAGCAGGT

CCAGGGCCCGCAGCCGGATGGACTCGTCCTTGTCGTCCAGGCACTGCAGGATGAGGTCCT

TGTGGGACTGCACGGACTTGGGGTGGGTCTTCAGGATCTTGGACATTGATTG

>1046 len=159 tot-seq=26

TGGGGAATTTTGGACAATGGGCGAAAGCCTGATCCAGCCATTCCGCGTGAGTGAAGAAGG

CCTTCGGGTTGTAAAGCTCTTTCGCAAGGGAAGAAACGATGCAGTCGAATAAACTGTGTT

AATGACGGTACCTTGATAAGAAGCACCGGCTAACTACGT

>1047 len=182 tot-seq=26

AGCATTTGCTATTACTACATCTTCCTACAATACCTAAACGTCTGGCTCCCTCTTTTATTT

TGTACATGAGGACATGGAGAAGGAGAGTTTCAAGGTCTGTCTGCTGCCTGGCCTGGCGGG

GATCCTTCCTTGCCAGCACCCCCTGTTCTCCCAGGGCAGCTCTGTGCTGAGTCAGCACCC

AC

>1048 len=135 tot-seq=26

TGGGGGATATTGGACAATGGGGGAAACCCTTATCCAGCGACGCCGCGTGAGGGAAGAAGG

TTTTCGGATTGTAAACCTCTGTCAGCGGGGACGATAATGACGGTACCCGCGGAGGAAGCC

ACGGCTAACTACGTG

>1049 len=160 tot-seq=26

TGGGGAATTTTGGACAATGGGGGCAACCCTGATCCAGCCATGCCGCGTGCAGGATGAAGG

TCTTCGGATTGTAAACTGCTTTTGTCAGGGAAGAAAAGTTTAGAGCGAATAACTCTGAAT

GCTGACGGTACCTGAAGAATAAGCACCGGCTAACTACGTG

>1050 len=155 tot-seq=26

TGGGGAATATTGCACAATGGGCGAAAGCCTGAACCAGCCAAGTAGCGTGCAGGAAGACGG

CCCTATGGGTTGTAAACTGCTTTTATGCGGGGATAAAGGAGTCCACGTGTGGGCTTTTGC

AGGTATCGCATGAATAAGGACCGGCTAATTCCGTG

>1051 len=160 tot-seq=26

TGGGGAATATTGCGCAATGGGCGCAAGCCTGATGGAGCAACACCGCGTGAGTGAAGAAGG

GTTTCGGCTCGTAAAGCTCTGTTGTTGGAGAAGAACGTGCGTGAGAGTAACTGTTCACGC

AGTGACGGTATCCAACCAGAAAGTCACGGCTAACTACGTG

>1052 len=135 tot-seq=26

TGGGGAATATTGGACAATGGGGGGAACCCTGATCCAGCGACGCCGCGTGAGTGAAGAAGT

ATTTCGGTATGTAAAGCTCTATCAGCAGGGAAGAAAATGACGGTACCTGACTAAGAAGCC

CCGGCTAACTACGTG

>1053 len=136 tot-seq=26

TGGGGAATATTGCACAATGGGGGGGACCCTGATGCAGCGACGCCGCGTGGGTGAAGGAGT

ATTTCGGTATGTAAAGCCCTATCAGCAGGGAAGAACAATGACGGTACCTGACTAAGAAGC

ACCGGCTAAATACGTG

>1054 len=160 tot-seq=26

TGGGGAATTTTGGACAATGGGCGCAAGCCTGATCCAGCCATGCCGCGTGTCTGAAGAAGG

CCTTCGGGTTGTAAAGGACTTTTGTCAGGGAAGAAAAGGCCGTTGCCAATATCGGCGGCC

GATGACGGTACCTGAAGAATAAGCACCGGCTAACTACGTG

>1055 len=161 tot-seq=26

TGGGGAATATTGCGCAATGGGGGAAACCCTGACGCAGCGACGCCGCGTGCGGGATGAAGG

CCTTCGGGTTGTAAAGCTCTGTTGTAAGAGAAGAACGTGTGTGAGAGTGGAAAGTTCACA

CAGTGACGGTAACTTACCAGAAAGGGACGGCTAACTACGTG

>1056 len=135 tot-seq=26

TGGGGAATATTGGACAATGGGCGCAAGCCTGATCCAGCCATGCCGCGTGAGTGATGAAGG

CCTTAGGGTTGTAAAGCTCTTTCGTCGGGGAAGATAATGACGGTACCCGAAGAAGAAGCC

TCGGCTAACTCCGTG

>1057 len=145 tot-seq=26

TGGGGGATATTGGACAATGGGGGAAACCCTGATCCAGCGACGCCGCGTGAGTGAAGAAGT

ATTTCGGTATGTAAAGCTCTATCAGCAGGGAAGAAAAATAACCAAATTGACTGTACCTGA

CTAAGAAGCCCCGGCTAACTACGTG

>1058 len=172 tot-seq=26

GGAGCTCAACCACCAAGCGGGGAGCGAGAGAGGAGAGAGCACATGTGGTCACGAGGCTTT

CATTAAGGCCCATTAGCCTTCCTGAGGGGGTTGTGAATTGTCTGGTTTAAAGAAAACACA

GATGAAGGGGGAACTTATTAACTCTGGTGTGGGCCATTAGGTCTTATCATGG

>1059 len=101 tot-seq=25

CTTAGGGTCCCCTCCCTCTCTGTACCTGGAGAAGTGGTAGTCTTCACAGACCCTCTCGGG

AGCCCCCACCCCAAATCTGCCTAGAGAATCGCATGGAGAGC

>1060 len=137 tot-seq=25

TGGGGAATATTGCGCAATGGGGGAAACCCTGACGCAGCAACGCCGCGTGATTGAAGAAGG

TCTTCGGATTGTAAAAATCTTTTATCAAGGACGAAGAAGTGACGGTACTTGATGAATAAG

CTCCGGCTAACTACGTG

>1061 len=156 tot-seq=25

TGAGGAATATTGGACAATGGGTTAGCGCCTGATCCAGCCATCCCGCGTGCAGGATGACGG

CCTTATGGGTTGTAAACTGCTTTTATATGTGAATAAACCTATCCACGAGTTGGATAGTTG

AAGGTAGCATATGAATAAGCACCGGCTAACTCCGTG

>1062 len=160 tot-seq=25

TAGGGAATCTTCCACAATGGACGAAAGTCTGATGGAGCAACGCCGCGTGAGTGAAGAAGG

TTTTCGGATCGTAAAACTCTGTTGTCAGAGAAGAACGTGTGCGAGAGTAACTGTTCGTGC

AGTGACGGTATCTGGCCAGAAAGCCACGGCTAACTACGTG

>1063 len=161 tot-seq=25

TGGGGAATATTGCACAATGGGGGAAACCCTGATGCAGCAACGCCGCGTGAGTGATGAAGG

CCTTCGGGTTGTAAAGTACTTTCAGCGGGGAGGAAGGGAGTAAAGTTAATACCTTTGCTC

ATTGACGTTACCCGCAGAAGAAGCACCGGCTAACTCCGTGG

>1064 len=165 tot-seq=25

TAGGGAATCTTCCACAATGGACGCAAGTCTGATGGAGCAACGCCGCGTGAACGATGAAGG

TCTTATAGATTGTAAAGTTCTTTTATATGGGAAGAAACGCTAAGATAGGAAATGATTTTA

GTTTGACTGTACCATTTGAATAAGTATCGGCTAACTATGTGTGTG

>1065 len=171 tot-seq=25

GAGTACCGGAAGAGGCCACCTTACCTTTCTTGCTGCCAATAACTTGGTGAAAGGAAGAGA

GCTGATAGAGCTACCTGCCATAGACTTTGAATACATCTTCCTTGCCAATAACTTGGTGAA

AGGAAGAGAGCTGATAGAGCTACCTGCCATAGACTTTGAATACATCTTCCC

>1066 len=191 tot-seq=25

GGAGAGGGAGCCCAGAGAGAGTACACAGAGAGAGGAGAGACATAAACAGCTCGCAGGACC

AAGAAAAAGCAGCCTGCACTCTACAGGGACCAAAGGCAGCAAAACCCTCTAACTTAATTT

TTTATAGTCCCCTGCTAACTACAGCAGAGGTGACACTTAAGAAAATGAGCCCTCTCCGTT

CTTTCCAGGAG

>1067 len=216 tot-seq=25

TGAGGAATATTGGTCAATGGGCGCGAGCCTGAACCAGCCAAGTCGCGTGAGGGATGAAGG

ATCTATGGTTCGTAAACCTCTTTTATAAGGGAATAAAGTGCGGGACGCGTCCCGCTTCGC

ATGTACCTTATGAATAAGGATCGGCTAACTCCGTGCCAGCAGCGCGGTAATCTATAGATC

GGAAGAGCGTCGTGTAGGGAAAGTGAGGAATATTGG

>1068 len=218 tot-seq=25

CCCTACAGTAGGACACAGAAGCAGCAGACAGCTCCCCACCTTTGGACCCCTGATCTTCTT

TGTCTCATTCTCAAGGACTTTTGTCCTAAGGAGGACCCACAATGTGTATGCTTGCATACC

TGAGTGTGTGTATGTGTATGTGCACGCATACGCAAGTGTGCATATGTGTACTTACCATGT

CCACGTGCCTCTCTGATGTTCTTACATTTGGTTAAATT

>1069 len=225 tot-seq=25

TGGAAGTTTGTAAAGAATAGCTGTGGCGTTGCCCCTCTATTCCACGACTGTTCTCAGGCC

TACTTGTGTATTTTTACCCAGTATCACTTATTTTTAAATGACAGAAGTTTGGAGCTTCTC

AGGCAATGTGATTTTCCTGTGGCCTAAACTGCCTTAGGTAAAGCATCGAGACTAACCCAA

AAGGCTATTGCTGGGTCTCTCGGCTCCCTTCCTTATTATCTATCA

>1070 len=190 tot-seq=25

ACAGCGTGAAGAGCCTCAGGTGATCGGTGACCAAGCGCAGGTAGAGCGGCCGGCCTGATT

CCCGCTTCACCAGCAGCAGTCGCATCTGGCAAGACTCAGGACTCAGGGTGGGGTCCAGGA

GGGCTGCCCGCAGCCAGCCCTCTGCCCCAGCACCAGCCCCTCTGCCAGGCAGCTCCTCCT

CTGAGAGTCC

>1071 len=154 tot-seq=25

TGAGGAATATTGGTCAATGGGCGAGAGCCTGAACCAGCCAAGTCGCGTGAAGGAAGAAGG

TTCTATGGATTGTAAACTTCTTTTGTAAGGGAATAAAAAGAGTTACGTGTAACTTATTGT

ATGTACTTTACGAATAAGGATCGGCTAACTCCGT

>1072 len=161 tot-seq=25

TAGGGAATCTTCCACAATGGACGCAAGTCTGATGGAGCAACGCCGCGTGAGTGATGACGG

TCTTCGGATTGTAAAGCTCTGTTAATCGGGACGAATGGTCGGTGTGCAAATAGTGCATGG

ACATGACGGTACCGGAATAGAAAGCCACGGCTAACTACGTG

>1073 len=144 tot-seq=25

GATACCCCAACACCTCAGTTCCATCCTCAGGGCCACAGAAGCCCTCAGGGGTGTAGTCAA

TGGTGACTTTATTCTTATTTTTCTGGGAGGAGTAACGCCTGTGTGGGGCAGGCCTGCTCC

TTCAGCCCGCGTGGCTTACTCCTT

>1074 len=155 tot-seq=25

TGAGGAATATTGGTCAATGGACGAGAGTCTGAACCAGCCAAATCGCGTGAAGGAAGACAG

CCCTACGGGTTTTAAACTTCTTTTATAGGGGAATAAAAAAGAGAACGAGTTCTCTATTGC

ATGTACCCTATGAATAAGCATCGGCTAACTCCGTG

>1075 len=184 tot-seq=25

GGTAAACCATGAGAACTGGGGGTGGAGGACTACTCTATGAGGAGTCAGAAAGGAAATGGA

GTGATGGGAGGAGAGGAGGCAGAGGGTGGCGTCGTTGCTTCTTGCTTACCCTCCCCTGCA

ACATATCCTATGTAAATGTTCAGTGCCCCAAAAATGTACATACAGCTTATGAAAAGCAGG

ACAG

>1076 len=142 tot-seq=25

TGGGGAATATTGCACAATGGACGGAAGTTTGATGCAGCGACGCCGCGTGCGGGATGAAGG

CCTTCGGGTTGTAAACCGCTTTTGATTGGGAGCAAGCCTTCGGGTGAGTGTACCTTTCGA

ATAAGCGCCGGCTAACTACGTG

>1077 len=114 tot-seq=25

TAGGGAATCTTCCACAATGGACGCAAGCCTGATGGAGCAACGCCGCGTGAGTGAAGAAGG

GTTTCGGCTCGTAAAGCTCTGTTGTTGGTGAAGAAGGAGAGGGGGAGTAACTGA

>1078 len=232 tot-seq=25

TAGGGAATTTTTCACAATGGGCGCAAGCCTTATGAAGCAATGCCGCGTGAACGATGAAGG

TCTTATAGATTGTAAAGTTCTTTTATATGGGAAGAAACGCTAAAATAGGAAATGATTTTA

GTTTGACTGTACCATTTGAATAAGTATCGGCTAACTATGTGCCAGCAGCCGCGGTAATTT

CAGATCGGAAGAGCGTCGTGTAGGGAAAGAGGGGGGGGGTGTGTAGATCTAG

>1079 len=135 tot-seq=25

TGGGGAATATTGCACAATGGGCGAAAGCCTGATGCAGCGACGCCGCGTGAGCGAAGAAGG

CTTTCGAGTCGTAAAGCTCTGTCCTATGAGAAGATAATGACGGTATCATAGGAGGAAGCC

CTGGCTAAATACGTG

>1080 len=160 tot-seq=25

TGAGGAATATTGGTCAATGGACGCAAGTCTGACGGAGCAACGCCGCGTGAGTGAAGAAGG

ATTTCGGTTCGTAAAACTCTGTTGTTAGAGAAGAACAGCGCATAGAGTAACTGTTATGCG

TGTGACGGTATCTAACCAGAAAGCCACGGCTAACTACGTG

>1081 len=160 tot-seq=25

TGGGGAATATTGGACAATGGGCGCAAGCCTGATCCAGCCATGCCGCGTGTGTGAAGAAGG

CCTTCGGGTTGTAAAGTACTTTCAGCGGGGAGGAAGGGAGTAAAGTTAATACCTTTGCTC

ATTGACGTTACCCGCAGAAGAAGCACCGGCTAACTCCGTG

>1082 len=105 tot-seq=24

AATACATGAAAGAGGGCTCACAGAGCACTGGCCGGGCCCGGCACCCCATGGCGTCTGCAG

ATGGCCGCTGCCCTCACCCTCCTTCCCTTCATCTCCAGGGTGGGC

>1083 len=113 tot-seq=24

CTGGTCCTGGGAGACCCGAAGCTTCTCCCTGGATGTTTAGGGAGTGAGCTGCCTGTAGGG

TCCCATGTACTGGTGGCCACATGTCCATATCCCTCTTCTGTCCCTGTCCAGAC

>1084 len=135 tot-seq=24

TGGGGAATTTTGCACAATGGAGGGAACTCTGATGCAGCGACGCCGCGTGAACGAAGAAGG

TTTTCGAATCGTAAAGTTCTGTCCTGGGTGAAGATAATGACGGTAATCCAGGAGGAAGCC

CCGGCTAACTACGTG

>1085 len=138 tot-seq=24

TGGGGAATATTGGGCAATGGGCGCAAGCCTGACCCAGCAATGCCGCGTGAAGGAAGAAGG

CCCTCGGGTTGTAAACTTCTTTTATCAGGGACGAAGGATGTGACGGTACCTGATGAATAA

GCTCCGGCTAACTACGTG

>1086 len=160 tot-seq=24

TAAGGAATTTTCGGCAATGGAGGAAACTCTGACCGAGCAACGCCGCGTGAGTGATGAAGG
CCTTAGGGTTGTAAAGCTCTGTTACGAGGGAAGAAACTACTCACTAGGAAATGAGTGAGT
ACCGACGGTACCTCGCCAGAAAGTCACGGCTTACTACGTG
>1087 len=181 tot-seq=24
TGGGGAATTTTACGCAATGGGCGAAAGCCTGACGTAGCGACACCGCGTGAGCGAAGAAGC
CCTTTGGGGTGTAAAGCTCTGTCAGCAGGGACGAAATAAATGACGGTACCTGCAGAGGAA
GCATCGGCTAACTACGTGCCAGCAGCGCGGTAATAGCAGATCGGAAGAGCGTCGTGTATG
G
>1088 len=230 tot-seq=24
GCTAAGCAGTTCATTGACGTCATGGTGAATCCGAAATTCACTCATAGTTTTAGCTCTGAG
GCACGAACATCACAATATGTTCTCCTATAGGTAAGAAGACAGCTATTCATAAAATTCAAA
CTGTAGAAATAAAAAACATTTTTTTAAACACTCAAGACTGGCCAATGTCAGGCTTTGACA
AACCAAGTTATCACCTGGCCTGCCTGGCCTAGGGCCCCTCCCCCAACAGC
>1089 len=237 tot-seq=24
GAGAGCTGTTGGACAGTTTTAATGGGAGAGTTGCATGATCATGTCTGATTTTTAAGGAAG
ACTGTAACAGCAGACATGGATTGGAGGGAGAAGAGGGGCAATCATCATTTCATCTAAAAG
CCCCTCCAGGAAAGTCTAGAATCTTTGCACAGAAGCTTTTTGAGATAATTAAGCCCCACT
GGTGAATTGGAGCAAACAGGTTCAGCTTTGCAGTGAGCCAGCAGTAGTAGCTTTTGG
>1090 len=160 tot-seq=24
TGGGGAATTTTGGACAATGGGGGCAACCCTGATCCAGCAATGCCGCGTGAGTGAAGAAGG
CCCTCGGGTTGTAAAGCTCTTTTGTCAGGGAAGAAACGGTGAATCCTAATACGGTTTGCT
AATGACGGTACCTGAAGAATAAGCACCGGCTAACTACGTG
>1091 len=135 tot-seq=24
TGGGGGATATTGCGCAATGGGGGAAACCCTGACGCAGCAACGCCGCGTGAAGGATGAAGG
TTTTCGGATTGTAAACTTCTTTTATTTAGGACGAATTATGACGGTACTAAATGAATAAGC
TCCGGCTAACTACGT
>1092 len=159 tot-seq=24
TGGGGAATTGTTCGCAATGGGCGCAAGCCTGACGACGCAACGCCGCGTGGAGGATGAAGG
TCTTCGGATTGTAAACTCCTGTTGCTCGGGAAGAATGGCTCCGACCTAACACGTCGGAGT

GTGACGGTACCGGGTGAGGAAGCCCCGGCTAACTCTGTG

>1093 len=149 tot-seq=24

TGGGGAATATTGCACAATGGGCGCAAGCCTGATGCAGCGACGCCGCGTGGGGGATGGAAG

GCCTTCGGGTTGTAAACTCCTTTCGCCCGGGACGAAGCCCACCTGGTGGGTGACGGTACC

GTGGAGAAGAAGCACCGGCTAACTACGTG

>1094 len=154 tot-seq=24

TGAGGAATATTGGTCAATGGGCGGAAGCCTGAACCAGCCAAGTCGCGTGAGGGAAGACGG

CCCTAAGGGTTGTAAACCTCTTTTGCCGGGGAGCAAAAGCGCCGTGCGCGGTGCTATGAG

AGTACCCGGAGAAAAAGCATCGGCTAACTCCGTG

>1095 len=135 tot-seq=24

TGGGGAATCTTGGACAATGGGCGAAAGCCTGATCCAGCCATGCCGCGTGGATGATGAAGG

CCCTAGGGTTGTAAAATCCTTTCGTTGGGGACGATAATGACGGTACCCAAAGAAGAAGCT

CCGGCTAACTTCGTG

>1096 len=161 tot-seq=24

TGAGGAATATTGGTCAATGGACGAAAGTCTGACGGAGCAACGCCGCGTGAGTGATGACGG

TCTTCGGATTGTAAAGCTCTGTTAATCGGGACGAATGGTCGGTGTGCAAATAGTGCATGG

ACATGACGGTACCGGAATAGAAAGCCACGGCTAACTACGTG

>1097 len=142 tot-seq=24

TGGGGAATATTGCACAATGGACGGAAGTTTGATGCAGCGACGCCGCGTGCGGGATGAAGG

CCTTCGGGTTGTAAACCGCTTTCAGCAGGGACGAGGCCGCAAGGTGACGGTACCTGCAGA

AGAAGCCCCGGCTAACTACGTG

>1098 len=136 tot-seq=23

TGGGGAATATTGCACAATGGGGGGAACCCTGATGCAGCGACGCCGCGTGAGTGACGAAGT

ATCTCGGTATGTAAAGCTCTGTCAGCAGGGAAGAAGAATGACGGTACCTGAAGAAGAAGC

ACCGGCTAAATACGTG

>1099 len=137 tot-seq=23

TGGGGAATATTGGGCAATGGGGGAAACCCTGACCCAGCAACGCCGCGTGAAGGAAGAAGG

TTTTCGGATTGTAAACTTCTGTCCTTGGTGAAGAGAAGAAGACGGTAGCCAAGGAGGAAG

CCCCGGCTAACTACGTG

>1100 len=137 tot-seq=23

TGGGGGATATTGCACAATGGGGGAAACCCTGATGCAGCGACGCCGCGTGAGGGAAGACGG

TCCTCTGGATTGTAAACCTCTGTCTTCGGGGACGAAACGAGACGGTACCCGAGGAGGAAG

CCACGGCTAACTACGTG

>1101 len=138 tot-seq=23

TGGGGAATATTGGGCAATGGGCGCAAGCCTGACCCAGCAACGCCGCGTGAGTGATGAAGG

TCTTCGGATTGTAAAACTCTTTAAGCAGGGAAGAAGAAAGTGACGGTACCTGCAGAATAA

GCATCGGCTAACTACGTG

>1102 len=144 tot-seq=23

ATGATGGCCCTGTTTCCACGGGTCATAGGAAGAAACTTTGAATACGTGCTCTGATCCCTG

AAAGTCAAGAGGACCAGCAAAAGAGATTCTGAGGAGAGGGCTATGTGACACAGGAGGACA

GAGGAGGGAGACAAACAGATATGG

>1103 len=155 tot-seq=23

TGAGGAATATTGGTCAATGGTCGGCAGACTGAACCAGCCAAGTCGCGTGAGGGAAGACGG

CCCTACGGGTTGTAAACCTCTTTTGTCGGAGAGTAAAGTACGCTACGTGTAGCGTAGTGC

AAGTATCCGAAGAAAAAGCATCGGCTAACTCCGTG

>1104 len=155 tot-seq=23

TGAGGAATATTGGTCAATGGGCGGAAGCCTGAACCAGCCAAGTAGCGTGCAGGATGACGG

CCCTACGGGTTGTAAACTGCTTTTATACGGGAATAACGTGCACTACGTGTAGTGCCTTGC

ATGTACCGTATGAATAAGGACCGGCTAATTCCGTG

>1105 len=156 tot-seq=23

TGAGGAATATTGGTCAATGGGCTTACGCCTGAACCAGCCAAGTCGCGTGAGGGAAGACTG

CCCTATGGGTTGTAAACCTCTTTTGTCAGGGAGCAATTGGCGCTACGTGTAGCGTTATCG

AGAGTACCTGAAGAAAAAGCATCGGCTAACTCCGTG

>1106 len=160 tot-seq=23

TGGGGAATTTTGGACAATGGGGGAAACCCTGATCCAGCCATGCCGCGTGTATGAAGAAGG

CCTTAGGGTTGTAAAGTACTTTTGTTAGGGAAGAAAAGCTAGTTTTTAATAAAAATTAGT

GATGACGGTACCTAAAGAATAAGCACCGGCTAACTACGTG

>1107 len=160 tot-seq=23

TAGGGAATCTTCCACAATGGGCGAAAGCCTGATGGAGCAACGCCGCGTGAGTGAAGAAGG

TTTTCGGATCGTAAAACTCTGTTGTAAGGGAAGAACAAGTACAGTAGTAACTGGCTGTAC

CTTGACGGTACCTTATTAGAAAGCCACGGCTAACTACGTG

>1108 len=185 tot-seq=23

TGGGGAATATTGCACAATGGGCGCAAGCCTGATGCAGCGACGCCGCGTGAGGGATGGAGG

CCTTCGGGTTGTAAACCTCTTTTGTTTGGGAGCAAGCCTTCGGGTGAGTGTACCTTTCGA

ATAAGCGCCGGCTAACTACGTGCCAGCAGCCCGGTAATGATAAGATCGGAAGAGCGTCGT

GTTGG

>1109 len=191 tot-seq=23

TGGGGAATATTGGACAATGGGCGAAAGCCTGATCCAGCAATGCCGCGTGAACGATGAAGG

CCTTAGGGTTGTAAAGTTCTTTCGGTGGGGAAGATAATGACGGTACCCACATAAGAAGCT

CCGGCTAACTCCGTGTTTCGGTGGGGAAGATAATGACGGTACCCACATAAGAAGCTCCAG

CTAACTCCGTG

>1110 len=200 tot-seq=23

TGGGGAATCTTCCGCAATGGGCGAAAGCCTGACGGAGCAATGCCGCGTGAGTGAAGAAGG

CCTTCGGGTTGTAAAGCTCTATCCTTGTCCAAGAGAGCGAGTGGTAGTAACTGACCAATC

GAGGACGGTAGACAAGAAGGAAGCCCCGGCTAACTATGTGGAGGACGGTAGACAAGAAGG

AAGCCCCGGCTAACTATGTG

>1111 len=209 tot-seq=23

GCATGCAAATTACCCACTCCCGACCCGAAGAGGTAGTGACGAAAAATAACAATACAGGAC

TCTTTCGAGGCCCTGTAATTGGAATGAGTCCACTTTAAATCCTTTAACGAGGATCCATTG

GAGGGCAAGTCTGGTGTTCGAGGCCCTGTAATTGGAATGAGTCCACTTTAAATCCTTTAA

CGAGGATCCATTGGAGGGCAAGTCTGGTG

>1112 len=221 tot-seq=23

GAAAAAAAACTCTTTAGATTTACTCAAATTTCTGAGTTGCCAAATTCAATGATTTGATTT

AGGTAAAGCGCTGAGACTGATGCTAGATACTAGTATCACATACTCGCACAAAGGATTCGC

TGCCAGGATGACCAACTGCTCTGGACAAAGGGAAGACTACTTACAAGGTTCCTGACCTCA

TGGTGCTTAGGAGATGAGAGAGACAATGATAAGCACTAAGA

>1113 len=224 tot-seq=23

CACGGTGGAGCGAAATGAGTTCCAGTGACGTCGTTTGAGCCCCAAATCCAGCCGAGATGA

AGCCAGCCCTAGTCCCTGGCCTTTTTGAGTTTATGATCCAATAAATTCCTTTTTGTTTAA

GCCAGTTTAGGTTGGGTTTTCTGTTACTTGGAACAGAAAGCGTTCTAGCTGATACAGGTT

TCAAACAGGAATGCAACGCCGTCATGTGTTTTAGTAATGTCACT

>1114 len=155 tot-seq=23

TGAGGAATATTGGTCAATGGCCGAGAGGCTGAACCAGCCAAGTCGCGTGAGGGAAGAATG

TCCTATGGATTGTAAACCTCTTTTGTCAGGGAGCAAAGTCACCCACGTGTGGGTGTTGGA

GAGTACCTGAAGAAAAAGCATCGGCTAACTCCGTG

>1115 len=135 tot-seq=23

TGGGGAATATTGCGCAATGGGGGAAACCCTGACGCAGCAACGCCGCGTGAATGAAGAAGG

CCTTAGGGTTGTAAAGTTCTGTTTACGGGGACGATAATGACGGTACCCGTGGAGGAAGCC

ACGGCTAACTACGTG

>1116 len=137 tot-seq=23

TGGGGAATATTGGGCAATGGGGGAAACCCTGACCCAGCAACGCCGCGTGAAGGAAGAAGG

CCTTCGGGTTGTAAACTTCTTTTGGCAGGGACGAAGGACGTGACGGTACCTGCAGAAAAA

GCAACGGCTAACTACGT

>1117 len=217 tot-seq=23

ATACGAGATGCGATAGTGACTGGAGTTCAGACGTGTGCTCTTCTCCTACGGGAGGCAGCA

GTGAGGAATATTGGTCAATGGGCGAGAGCCTGAACCAGCCAAGTCGCGTGAAGGAAGACT

GCCCGCAAGGGTTGTAAACTTCTTTTGTATGGGATTAAAGTCGTCTACGTGTAGGCGTTT

GCAGTTACCATACGAATAAGCATCGGCTAACTCCGTG

>1118 len=186 tot-seq=23

GACGGGGGGGGGACAGGAACTCAGTCCAGCACAGTGAGTAGGCAGGGGACTAAGACCAGA

GCTAGCTAAGGTCATCAGGGGTCTCAAGGTTGAGTCCCGATGGCTTCATAAGAACAGAAT

GAACCTGGAAGAGCACACATTTGTACTCTCTCTGTCTTTCCCTGTCCCACTCTGGGAATC

TGCCAG

>1119 len=130 tot-seq=23

GCGCGCAAATTACCCAATCCTGACACAGGGAGGTAGTGACAATAAATAACGATACAGGGC

CTTTGGTCTTGTAATTGGAATGAGTACAACGTAAATACCTTAACGAGGAACAATTGGAGG

GCAAGTCTGG

>1120 len=182 tot-seq=23

GACCACAAGAACTGGTAGAACCCAAGGAAGGTTGGGTGGCTGTGCTGAATGGCTTGATAA

AGGTGGGCCTGGGGTGTGTCACAGGGTCAAGCCACTTAAAAGGAGACCTGGGCTGATCTG

GCTTTGGGGGTGGCAGGGCCGGCCCAAGCCTGGGAGATACAGGGTCATACCTAATCCATT

CA

>1121 len=185 tot-seq=23

TGGGGAATATTGCACAATGGGCGCAAGCCTGATGCAGCAACGCCGCGTGAACGATGAAGG

TCTTCGGATCGTAAAGTTCTGTTGCAGGGGAAGATAATGACGGTACCCTGTGAGGAAGCC

CCGGCTAACTACGTGCCAGCAGCCGGGTAATGTCTAGATCGGAAGAGCGTCGTGTAGGGA

AAGAG

>1122 len=141 tot-seq=23

TGGGGAATATTGCACAATGGGCGCAAGCCTGATGCAGCGACGCCGCGTGGGGGATGACGG

CCTTCGGGTTGTAAACTCCTTTCGACAGGGACGAAGCTATAAGTGACGGTACCTGTAGAA

GAAGCACCGGCTAACTACGTG

>1123 len=199 tot-seq=23

TGGGGAATATTGCACAATGGGCGCAAGCCTGATGCAGCCATGCCGCGTGTATGAAGAAGG

CCTTCGGGTTGTAAAGTACTTTCAGCGGGGAGGAAGGCGATAAGGTTAATAACCTTGTCG

ATTGACGTTACCCGCAGAAGAAGCACCGGCTAACTCCGTGCCAGCAGCCGCGGTAGATCG

GAAGAGCGTCGTGTAGGGG

>1124 len=140 tot-seq=23

TAGGGAATCTTCGGCAATGGGGGGAACCCTGACCGAGCAACGCCGCGTGAGGGATGGAGG

CCTTCGGGTTGTAAACCTCTTTTATCGGGGAGCAAGCGAGAGTGAGTTTACCCGTTGAAT

AAGCACCGGCTAACTACGTG

>1125 len=161 tot-seq=23

TGGGGAATCTTGCACAATGGGCGAAAGCCTGATGCAGCGACGCCGCGTGAGTGAAGAAGG

TTTTCGGATCGTAAAGCTCTGTTGTAAGAGAAGAACGTGTGTGAGAGTGGAAAGTTCACA

CAGTGACGGTAACTTACCAGAAAGGGACGGCTAACTACGTG

>1126 len=144 tot-seq=22

GGGACAATGGATAGCAGAGACCTTACAGGTCTCATGAGCTGGGACAGAGGCCTAGCTCTC

TTACTCATCAAATGCATGAACTGCATAAGGAGCTAACTTCTGTAAGCCTCAGGCTCTCTA

TCTAGTACTGAATTATTAGAAACC

>1127 len=159 tot-seq=22

TAGGGAATCTTCCACAATGGACGCAAGTCTGATGGAGCAATACCACGTGAACGATGAAGG

TCTTCTGATTGTAAAGTTCTTTTATTTAGGAAAAAAAGCGTGCTAGGAAATGAGCGCGCC

TTGATGGTACTAATTGAATAAGTGACAGCTAACTATGTG

>1128 len=160 tot-seq=22

TAGGGAATCTTCCACAATGGACGCAAGTCTGATGGAGCAACGCCGCGTGAGTGAAGAAGG

TTTTCGGATCGTAAAGCTCTGTTGTTGGAGAAGAACGTATCTGAGAGTAACTGTTCAGGT

AGTGACGGTATCCAACCAGAAAGTCACGGCTAACTACGTG

>1129 len=160 tot-seq=22

TGAGGAATTTTGGACAATGGGGGCAACCCTGATCCAGCCATTCCGCGTGAATGAAGAAGG

CCTTCGGGTTGTAAAGCTCTTTCAGCAGGAACGAAACGGCGGGCTCTAACAGGGCCTGCT

AATGACGGTACCTGCAGAAGAAGCACCGGCTAACTACGTG

>1130 len=160 tot-seq=22

TGGGGAATTTTGGACAATGGGCGCAAGCCTGATCCAGCCATGCCGCGTGCAGGATGAAGG

CCTTCGGGTTGTAAACTGCTTTTGTACGGAACGAAAAGGTTTGGCCTAATAAGCTGAGCT

CATGACGGTACCGTAAGAATAAGCACCGGCTAACTACGTG

>1131 len=161 tot-seq=22

TAGGGAATCTTCCGCAATGGACGAAAGTCTGACGGAGCAACGCCGCGTGAGTGATGAAGG

TTTTCGGATCGTAAAACTCTGTTGTTAGGGAAGAACAAGTACCGTTCGAATAGGGCGGCA

CCTTGACGGTACCTAACCAGAAAGCCACGGCTAACTACGTG

>1132 len=170 tot-seq=22

AAGACAAGCCTTGGTTGGAGAGGGAAGAATACTGACTGGGTTCAGTGTAGCCAAAACTTT

GATATCTGGAGGAAGAAGCCACGGCAGCTGCTCTGCCATGTTCCATGAGCACCTTGCTTC

TCTGAAAAGCAGTGGAGAAGCATTAGATGTTTCAACACCAGGGGTGAGTG

>1133 len=190 tot-seq=22

AATAACACTAACCACTGGAGAAATGTGAATCAGAACCCTACACCCATTATGATGGCAAGA

TCCCCTCCTCCCAAAAAAAAGTGTTGAAGAAAATGTGAGCAATTTGAGCCCTTGTCTGCT

GCTAAGGGAAATATAAGCATTGTAACCACTAAGGAAAACATTGTTGAGATACGAGTGACA

TACGAGTGAC

>1134 len=214 tot-seq=22

TGGGGAATATTGCGCAATGGGCGAAAGCCTGACGCAGCGACGCCGCGTGAGGGATGAAGG

TCTTCGGATCGTAAACCTCTGTCAGAAGGGAAAAATGTACAGTGCCCCAATCAACACTGT

ATTGATGGTACCTTCAGAGGAAGCACCGGCTAACTCCGTGCCAATCAACACTGTATTGAT

GGTACCTTCAGAGGAAGCACCGGCTAACTCCGTG

>1135 len=160 tot-seq=22

TAGGGAATCTTCGGCAATGGACGGAAGTCTGACCGAGCAACGCCGCGTGAGTGAAGAAGG

TTTTCGGCTCGTAAAGCTCTGTTGGTAGTGAAGAAAGATAGAGGTAGTAACTGGCCTTTA

TTTGACGGTAATTACTTAGAAAGTCACGGCTAACTACGTG

>1136 len=161 tot-seq=22

TGGGGAATCTTCCGCAATGGGCGAAAGCCTGACGGAGCAACGCCGCGTGAGTGAAGAAGG

CCTTCGGGTCGTAAAGCTCTGTCTTTAGGGAAGAACATCTTATGTGTGAACAATGCATAA

GACTGACGGTACCTGAGGAGGAAGCTCCGGCTAACTACGTG

>1137 len=137 tot-seq=22

TGGGGAATATTGCACAATGGGGGAAACCCTGATGCAGCGACGCCGCGTGGGCGAGGAAGT

ATTTCGGTATGTAAAGCCCTGTCAGCGGGGAAGAAGAATGACGGTACCCGACCAAGAAGC

ACCGGCTAAATACGTGG

>1138 len=135 tot-seq=22

TGGGGAATATTGCGCAATGGGGGAAACCCTGACGCAGCAACGCCGCGTGCGGGATGAAGG

CTTTCGGGTTGTAAACCGCTTTCAGCAGGGAAGATAATGACGGTACCTGCAGAAGAAGCC

CCGGCTAACTACGTG

>1139 len=209 tot-seq=22

TGGCTCTGGGGTTACCTTCGGCCTGTGCTGAAGATTCTTCTCAATCTCTGGCCCTGACCT

CTTCCTTGAAGGTCAGACTCAACGTTTGCTTTCTCTCAAGTTTGACATCAAGAGCCTGGT

TCAAAACACTCTTGACACTTCACCCCAAACCAGTCCCCACCCCCAGTTCCTTCTTCCAGT

CAAAGAACTCTGTTTCCCAAGTTATTCAA

>1140 len=160 tot-seq=22

TGGGGAATATTGGACAATGGGCGCAAGCCTGATCCAGCAATGCCGCGTGTGTGAAGAAGG

CCTGCGGGTTGTAAAGCACTTTCAGTGGGGAGAAAGAAACTATCACTAATATTGATAGTG

GATGATGTTACCCACAGAAGAAGCACCGGCTAATTCTGTG

>1141 len=160 tot-seq=22

TAGGGAATCTTCGGCAATGGACGGAAGTCTGACCGAGCAACGCCGCGTGAGTGAAGAAGG

TTTTCGGATCGTAAAACTCTGTTGTTAGAGAAGAACAGCGCATAGAGTAACTGTTATGCG

TGTGACGGTATCTAACCAGAAAGCCACGGCTAACTACGTG

>1142 len=217 tot-seq=22

GTCTGGGGAAATGGGGCTTTTTGAAATGGGAACATGAAGTTAGAGAGGTCCTGGCAAGAT

GGTAGTCTTATTTAATGATTCCGTTTGCAAGCATTGGAGACACTCACATCCTTGGGCAAT

AGGAGTGTCTTACAAAGATAGGGAAGGGGCCCTGGGGCCCAGGACAGCTGCCAGGAGTGG

CCTCTCAAGGAGCTGCTGTCCCAGATGCAGGTGAGAC

>1143 len=140 tot-seq=22

TAGGGAATCTTCGGCAATGGGCGCAAGCCTGATGCAGCGACGCCGCGTGAGGGATGGAGG

CCTTCGGGTTGTAAACCTCTTTTATCGGGGAGCAAGCGAGAGTGAGTTTACCCGTTGAAT

AAGCACCGGCTAACTACGTG

>1144 len=133 tot-seq=22

CAGGAGCAGAATCAGGAGTGGGAGAGGGAAAGGGAATGGCAGGAGAAGGGAGAGGGGGAG

AGGTAGGGTAAGGGGCCAGAGCAGAAACAGGAGGAAGAGGAGGAGGAGGAAGAGGAGGAG

GAGGAGGAGGAGG

>1145 len=161 tot-seq=22

TGGGGAATATTGCGCAATGGGGGAAACCCTGACGCAGCGACGCCGCGTGCGGGATGAAGG

CCTTCGGGTTGTAAAACTCTGTGATTCGGGACGAAAGATAAGTAGACGAATAATCTGCAT

AAGTGACGGTACCGAAAAAGCAAGCCACGGCTAACTACGTG

>1146 len=135 tot-seq=21

TCGGGAATATTGCGCAATGGAGGAAACTCTGACGCAGTGACGCCGCGTATAGGAAGAAGG

TTTTCGGATTGTAAACTATTGTCGTGAGGGAAGAAGATGACAGTACCTCAGGAGGAAGCT

CCGGCTAACTATGTG

>1147 len=135 tot-seq=21

TGGGGAATATTGGACAATGGGCGCAAGCCTGATCCAGCCATGCCGCGTGAGTGATGAAGG

CCTTAGGGTTGTAAAGCTCTTTTGTCCGGGAAGATAATGACTGTACCGGAAGAATAAGCC

CCGGCTAACTTCGTG

>1148 len=135 tot-seq=21

TGGGGAATATTGGACAATGGGCGCAAGCCTGATCCAGCCATGCCGCGTGAGTGATGAAGG

TCTTAGGATTGTAAAGCTCTTTCACCGGAGAAGATAATGACGGTATCCGGAGAAGAAGCC

CCGGCTAACTTCGTG

>1149 len=138 tot-seq=21

TGGGGAATATTGGGCAATGGAGGCAACTCTGACCCAGCAATGCCGCGTGAATGAAGAAGG

TCTTCGGATTGTAAAGTTCTTTAATTAGGGACGAAAAAAATGACGGTACCTAAGGAATAA

GCCACGGCAAACTATGTG

>1150 len=140 tot-seq=21

TGGGGAATATTGCACAATGGGCGGAAGCCTGATGCAGCGACGCCGCGTGAGGGATGAAGG

CCTTCGGGTTGTAAACCTCTTTCAGCAGGGACGAAGCGTGAGTGACGGTACCTGCAGAAG

AAGCACCGGCTAACTACGTG

>1151 len=149 tot-seq=21

TATTGGTCAATGGGCGGAAGCCTGAACCAGCCATGCCGCGTGAAGGAATGAGGCCCTACG

GGTCGTGAACTTCTTTTGTGGCGGAGCAATAAGGGCCACGTGTGGTCCGACGAGAGTACG

TCACGAATAAGCACCGGCTAACTCCGTGG

>1152 len=149 tot-seq=21

TAGGGAATCTTCCACAATGGGCGAAAGCCTGATGGAGCAATACCATGTGAACGATGAAGG

CCTTTTTGGTTGTAAAGTTCTTTTATGAGGGATAACAATGATAGTACCTCATGAATAAGT

GACAGCAAACTATGTGGCCAGCAGTAGGG

>1153 len=155 tot-seq=21

TGAGGAATATTGGTCAATGGGCGCGAGCCTGAACCAGCCAAGTAGCGTGAAGGATGACGG

CCCTATGGGTTGTAAACTTCTTTTATATGGGAATAAAGTTTGGTATGCATACCATTTTGT

ATGTACCATATGAATAAGGATCGGCTAACTCCGTG

>1154 len=160 tot-seq=21

TAGGGAATCTTCCGCAATGGACGAAAGTCTGACGGAGCGACGCCGCGTGAATGATGAAGG

TCTTAGGATCGTAAAATTCTGTTGTTAGGGATGAACAAGTGTGAGAGTAACTGCTCACAC

CTTGACAGTACTTAACCAGAAAGCCCCGGCTAACTACGTG

>1155 len=160 tot-seq=21

TGGGGAATATTGCACAATGGGCGAAAGTCTGACGGAGCAACGCCGCGTGAGTGAAGACGG

CCTTCGGGTTGTAAAGCTCTGTGATTCGGGACGAAAGGCCATATGTGAATAATATATGGA

AATGACGGTACCGAAAAAGCAAGCCACGGCTAACTACGTG

>1156 len=160 tot-seq=21

TAGGGAATCTTCCGCAATGGACGAAAGTCTGACGGAGCAACGCCGCGTGTGTGAAGAAGG

TCTTCGGATCGTAAAGCACTGTTATTAGAGAAGAACAACCGGTAGAGTAACTGTTACCGG

CTTGACGGTATCTAACCAGAAAGCCACGGCTAACTACGTG

>1157 len=207 tot-seq=21

CCAGGGTGACTGGAGTTCAGACGTGTGCTCTTCCGTCTCCTACGGAAGGCAGCTTTGGGG

AATATTGCACAATGGGGGAAACCCTGATGCAGCGACGCCGCGTGAGTGAAGAAGTATTTC

GGTATGTAAAGCTCTATCAGCAGGGAAGAAATAAAGTCCTTCGGACTTTGGACGGTACCT

GACTAAGAAGCCCCGGCTAACTACGTG

>1158 len=236 tot-seq=21

ATCACCATGCCCCCCCTAGAAGGGTTCTGATCACCGGAGGGCAGCCCCAAAGGCCACAGT

CCCCTCCTGTGCTGGCAGCTTTGCCCACACATACCCAGCAGCTCCCCAGGCTGAAAGCAG

CCCTGGCCCAGGGTCTCCATGGTTCTAGGCAGACCCTCTTTCTCCTTCGGGACAGAAAGA

CAATGTGAGTTCATTTTCCTCCATCCTCAGACCGTGACATCTCCCCTAGGCTCCCC

>1159 len=135 tot-seq=21

TGGGGAATATTGCACAATGGGCGCAAGCCTGATGCAGCAACGCCGCGTGAACGAAGAAGG

TATTCGTATCGTAAAGTTCTGTCCTATGGGAAGATAATGACAGTACCATAGAAGAAAGCT

CCGGCTAAATACGTG

>1160 len=135 tot-seq=21

TGGGGAATATTGCACAATGGGCGAAAGCCTGATGCAGCAACGCCGCGTGAAGGAAGAAGT

ATTTCGGTATGTAAACTTCTATCAGCAGGGAAGACAGTGACGGTACCTGACTAAGAAGCC

CCGGCTAACTACGTG

>1161 len=160 tot-seq=21

TGGGGAATATTGGACAATGGGGGCAACCCTGATCCAGCAATGCCGCGTGTGTGAAGAAGG

CCTGCGGGTTGTAAAGCACTTTCAGTAGAGAAGAAAAGCCTAAGGTTAATAACCTTGAGT

CTTGACGTAACCTACAGAAGAAGCACCGGCTAACTCCGTG

>1162 len=135 tot-seq=21

TGGGGAATATTGGACAATGGGCGAAAGCCTGATCCAGCGACGCCGCGTGAGTGAAGAAGT

ATTTCGGTATGTAAAGCTCTATCAGCAGGGAAGAAAATGACGGTACCTGACTAAGAAGCC

CCGGCTAACTACGTG

>1163 len=161 tot-seq=21

TGGGGAATTGTTCGCAATGGGCGCAAGCCTGACGACGCAACGCCGCGTGGAGGACGAAGA

TCTTCGGGTCGTAAACTCCTGTCGAGCGGGAAGAATGGGTGCGGGGTGAATACCCTCGCA

TCGTGACGGTACCGCTAAAGGAAGCCCCGGCTAACTCCGTG

>1164 len=156 tot-seq=21

TGGGGAATATTGGACAATGGGGGCAACCCTGATCCAGCCATGCCGCGTGTGTGAAGAAGG

TCTTCGGATTGTAAAGCACTTTAAGTTGTGAGGAAGGGGTAGAGTAATGACCTACTTTTG

ACGTTAGCAACAGAATAAGCACCGGCTAACTTCGTG

>1165 len=113 tot-seq=21

GGGCAGAGGCACGGATAACCACATTTGGTTCATTGCAGGCGCCGGGGACATCACTCAGGC

CCGGCAAGGAGCCAGTCATCCAGACTGCATGTGCCCTGCAGGGAAACCGTCTC

>1166 len=138 tot-seq=21

TGGGGAATATTGGGCAATGGGCGAAAGCCTGACCCAGCAACGCCGCGTGAGGGAGGAAGG

TTTTCGGATTGTAAACCTCTGTCCTTGGGGAAGAAGAAAGTGACGGTACCCAAGGAGGAA

GCCCCGGCTAACTACGTG

>1167 len=160 tot-seq=21

TGGGGAATATTGGACAATGGAGGCAACTCTGATCCAGCAATTCTGTGTGTGTGAAGAAGG

TCTTCGGATTGTAAAGCTCTGTTATACGGGACGAAAAAGACGGATGCCAACAGTATCCGT

CCGTGACGGTACCGTAAGAGAAAGCCACGGCTAACTACGT

>1168 len=159 tot-seq=21

TCGAGAATTTTTCACAATGGGGGAAACCCTGATGGAGCGACGCCGCGTGGGGGATGAATG

GCTTCGGCCCGTAAACCCCTGTCATTTGCGAACAATGCTTTGGTATGAACAATATCAAAG
TTGATAGTAGCAGAAGAGGAAGAGACGGCTAACTCTGTG
>1169 len=213 tot-seq=21
CCTTTGGGGGGTTGGTAGGGGAACCCAGGGGACCCGGGGAAGGGCTGCTGACTATGGGCC
TCGGGCAGGGTGCTGATCCTCTCTGAGCCTGCAAAATCCACCTCTCGTCTCAGATTGTGG
GAGGTGAGAACATCAACATCAGGAAGAAAGGAAGCCAGAAGCTGCTGGTTGTAGAAAAAT
AACTAAAACAAATGGCAGGACTCACAAGCACAT
>1170 len=155 tot-seq=21
TGAGGAATATTGGTCAATGGACGGAAGTCTGAACCAGCCAAGTAGCGTGCAGGATGACGG
CCCTATGGGTTGTAAACTGCTTTTATGTGGGAATAAAGTGGCGCACGCGTGCGCCATTGC
ATGTACCTCATGAATAAGGACCGGCTAATTCCGTG
>1171 len=138 tot-seq=21
TGAGGAATATTGGTCAATGGGCGAGAGCCTGATCCAGCAATTCTGTGTGTGTGAAGAAGG
TTTTAGGACTGTAAAACACTTTTAGTAGGGAAGAAAAAAATGACGGTACCTACAGAAGAA
GCAACGGCTAAATACGTG
>1172 len=153 tot-seq=21
TGGGGAATATTGCACAATGGGGGAAACCCTGATGCAGCGACGCCGCGTGAGTGAAGAAGT
AATTCGTTACGTAAAGCTCTATCAGCAGGGAAGAAATACTGACCTTACGGTCAGCAGACG
GTACCTGAGTAAGAAGCCCCGGCTAACTACGTG
>1173 len=160 tot-seq=21
TGGGGAATCTTCCGCAATGGACGAAAGTCTGACGGAGCAACGCCGCGTGAGTGAAGAAGG
TCTTCGGATTGTAAAGCTCTGTTGTTGGTGAAGAAGGACAGGGGTAGTAACTGACCTTTG
TTTGACGGTAATCAATTAGAAAGTCACGGCTAACTACGTG
>1174 len=161 tot-seq=21
TGGGGAATATTGCACAATGGGCGCAAGCCTGATGCAGCAACGCCGCGTGAGCGAAGAAGG
TCTTCGGATTGTAAAGCTCTGTTATACGGGACGAAAAAGACGGATGCCAACAGTATCCGT
CCGTGACGGTACCGTAAGAGAAAGCCACGGCTAACTACGTG
>1175 len=101 tot-seq=20
GGAAGCCCGGTCGATTGAGGAGAGGAAGGTGCCCGCTTCCACTTCCACCGTGGTATCGGA

CGTTGTCGGAATCAACTGGCTGGTTTCTGGGTAATTCCCCT

>1176 len=115 tot-seq=20

GAAGAGAAGCTGCAGTGCTGCAAGACTCCTGGTAGCTCCAGGCCACAGGGACTGGTCCTG

TCCTGAAGCCCCAGTCTCAGCCTGAGAGACCTTGCTCTGCCAAGTGACTGGCTGG

>1177 len=135 tot-seq=20

TGGGGAATCTTGCACAATGGGGGAAACCCTGATGCAGCGACGCCGCGTGAGCGATGAAGC

CCTTCGGGGTGTAAAGCTCTTTCGACAGGAACGATAATGACGGTACCTGGAGAAGAAGCT

GCGGCTAACTACGTG

>1178 len=135 tot-seq=20

TGGGGGATATTGCACAATGGAGGGAACTCTGATGCAGCAACGCCGCGTGAGGGAAGAAGG

TCTTCGGATTGTAAACCTTTGTCCTCAGGGAAGATAATGACGGTACCTGAGGAGGAAGCT

CCGGCTAACTACGTG

>1179 len=135 tot-seq=20

TGGGGAATATTGGACAATGGGCGAAAGCCTGATCCAGCCATGCCGCGTGAGTGATGAAGG

CCCTAGGGTTGTAAAGCTCTTTTACCTGGGAAGATAATGACGGTACCAGGAGAATAAGCC

CCGGCTAACTTCGTG

>1180 len=138 tot-seq=20

TGGGGAATATTGGGCAATGGGCGCAAGCCTGACCCAGCAACGCCGCGTGAATGAAGAAGG

TCTTCGGATTGTAAAGTTCTGTGACTGGGGACGAAAAAAATGACGGTACCCAGAGAGGAA

GCTCCGGCAAACTACGTG

>1181 len=139 tot-seq=20

TGGGGAATATTGGACAATGGACCAAAAGTCTGATCCAGCAATTCTGTGTGCACGATGACG

TTTTTCGGAATGTAAAGTGCTTTCAGTCGGGAAGAAGCAAGTGACGGTACCGACAGAAGA

AGCGACGGCTAAATACGTG

>1182 len=145 tot-seq=20

GAAGACTCGGTGTGTTTTTGAGCCTGGAGAAGCCACATGCCTCCTCTCGTGTGCTGGAGA

CACAGGGACAGCTAGGCATGTGCCTGGCCCTGGAGTTCCCGAGCAGCACAGTCTAGAAGG

CTCACAGCCGCCGTCCCCACTACAG

>1183 len=160 tot-seq=20

TAGGGAATCTTCCACAATGGGTGCAAACCTGATGGAGCAATGCCGCGTGAATGAAGAAGG

TCTTCGGATCGTAAAGTTCTGTTATTAGTGAAGAACAAATCGAGGAGTAACTGTCTCGAT

TTTGACGGTAACTAACCAGAAAGTCACGGCTAACTACGTG

>1184 len=160 tot-seq=20

TGGGGAATATTGCACAATGGGCGCAAGCCTGATGCAGCCATGCCGCGTGTGTGAAGAAGG

CTTTCGGGTTGTAAAGCACTTTCAGCGAGGAGGAAAGGGTATTGCTTAATAGGCAATATC

TGTGACGTTACTCGCAGAAGAAGCACCGGCTAACTCCGTG

>1185 len=196 tot-seq=20

ACTCTAATGCCTCACATCTCCAGGAGGTCTGAACCCCTCTGTGGGGCAGTGCAAGTTCCC

TGGAGGCTGCGATCTGATTTCAGCCCCTAGTCCTTCAACCGAGCAACACAGGGGACACCA

CCATCCCCCATAGGAAGCCTCCAGGACCGAGCTTTCCTGACTTTCCGTGCGCCTCCATGA

GAATCTGTTACGTTAC

>1186 len=201 tot-seq=20

TAGGGAATATTGGGCAATGGGCGCGAGCCTGACCCAGCCATGCCGCGTGCCGGATGAAGG

CCTTCTGGGTTGTAAACGGCTTTTCTCAGGGAAGAAAAAGGGTCTGCGGACCAAGCTGAC

GGTACCTGAGGAATAAGCACCGGCTAACTCCGTGCCAGCAGCCGCGGTATATTCAGATCG

GAAGAGCGTCGTGTAGGGAAA

>1187 len=203 tot-seq=20

GCTAGGGAGGAATGGAGGCTATGGAGTGTGTGTGTGTGGCATGGACTCACCTAGGGGT

GGGCCACGGAGGCTGCTGCTCCTATCCTATTGACGTTTCTGCGTGGGTGCCACCGGCTTG

AAGTGGCAGGCCCCGCCCCCTCCGCAATCTGCAGAGCCTGTGGCCCCTTACCTGGTATTT

CTCCATCTGAGAGAGGGTGTTGT

>1188 len=204 tot-seq=20

TTTCCTCCTGTCGGGGGGTGGTATTTTTCAGTTGGGTGGGTTAGATGTCACACACACACC

TGTCTGACCCTTCATACCCCTTAGGTCTGGGTCCCTCTACCCTTTCTGTTGCCCCACCTT

CCTGCTCACTCCCCCCAGACATGACTTATTGCTGGGAATGTGATGGAGGAAACAGAGTAA

CCTGTTCTCCCACCATTCTGGAAG

>1189 len=209 tot-seq=20

GTAGAAGAGAATTGGTCATGGGAAGGCTTTATGTTTTGTAACTTCCTGGCAAGGAAAGGA

GACTACACAATTCCTAATGCCTTTTCTACTTCCAATCTTCTTTGCCTCCTTCTGTGGGTC

TCAGATTGTTCAGGCAGGTACTGAAGACCAGATTCCAGAGATTAGATACCAGGCAGGTAC

TGAAGACCAGATTCCAGAGATTAGATACC

>1190 len=141 tot-seq=20

TGGGGAATATTGCACAATGGGCGCAAGCCTGATGCAGCGACGCCGCGTGGGGGATGACGG

CCTTCGGGTTGTAAACTCCTTTCGCTAGGGACGAAGCTTTTTGTGACGGTACCTAGATAA

GAAGCACCGGCTAACTACGTG

>1191 len=132 tot-seq=20

GGAATATTGGACAATGGGGGCAACCCTGATCCAGCAATGCCGCGTGAGTGATGAAGGCCT

TCGGGTTGTAAAGCTCTTTTCTTGGGGACGATAATGACGGTACCCGAGGAATAAGCCCCG

GCTAACTTCGTG

>1192 len=161 tot-seq=20

TAGGGAATCTTCCGCAATGGACGAAAGTCTGACGGAGCAACGCCGCGTGAGTGAAGAAGG

TTTTCGGATCGTAAAACTCTGTTGTCAGAGAAGAACAAGTTGGAGAGTAACTGCTCCAGC

CTTGACGGTATCTGACCAGAAAGCCACGGCTAACTACGTGG

>1193 len=180 tot-seq=20

GAAAGGGCCGGGAGGAGCGAGCGGAAAAGGCTGGAAGGGATCGAGAGTCAGTTACCTTGG

TCCACAGACCCCATGATGCGGAGGGGATGGCAGTGCCCCAGTCTGAACGCAGCCTCTCTC

TGCATCTGGAGGGCACGGGGACTTGGCGCGTGTTTGGAAAGGGGGATCTGTTTGTATCGA

>1194 len=154 tot-seq=20

TGGGGAATATTGCACAATGGGCGGAAGCCTGATGCAGCGACGCCGCGTGGGGGATGACGG

CCTTCGGGTTGTAAACTCCTTTCGACCGCGAGGAAGCCGCCTGGTTGGAAGGGTGGTGAC

GGTAGTGGTAGAAGAAGCACCGGCTAACTACGTG

>1195 len=160 tot-seq=20

TAGGGAATCTTCCACAATGGACGCAAGCCTGATGCAGCCATGCCGCGTGTGTGAAGAAGG

CCTTCGGGTTGTAAAGCACTTTCAGCGGGGAGGAAGGCGATAAGGTTAATAACCTTGTCG

ATTGACGTTACCCGCAGAAGAAGCACCGGCTAACTCCGTG

>1196 len=138 tot-seq=20

TAGGGAATCTTCCACAATGGGCGCAAGCCTGATGCAGCCATGCCGCGTGTGTGAAGAAGG

CCTTCGGGTTGTAAAGCACTTTCGGAGGGGAGGAAAAAAATGACGTTACCCTCAGAAGAA

GCACCGGCTAACTCCGTG

>1197 len=216 tot-seq=20

GGACGGCATACGAGATTCACGAGTGACTGGAGTTCAGACGTGTGCTACGGGAGGCAGCAG

TGAGGAATATTGGTCAATGGGCGAGAGCCTGAACCAGCCAAGTCGCGTGAAGGAAGACTG

CCCGCAAGGGTTGTAAACTTCTTTTGTATGGGATTAAAGTCACCTACGTGTAGGTGTTTG

CAGTTACCATACGAATAAGCATCGGCTAACTCCGTG

>1198 len=160 tot-seq=20

TAGGGAATCTTCCACAATGGACGCAAGTCTGATGGAGCAACGCCGCGTGAGTGAAGACGG

CCTTCGGGTTGTAAAGCTCTGTGATTCGGGACGAAAGGCCATATGTGAATAATATATGGA

AATGACGGTACCGAAAAAGCAAGCCACGGCTAACTACGTG

>1199 len=114 tot-seq=19

CTCAGACCCCACAGCTTTGGGTGTGACAAGCAATAACCTAGTTACTGACTCAGCAAGGGA

CCCAGGCAAGGCCCTGGGCCTCTGCACTTCTGGATTCTCATGTGTAAACCGCCA

>1200 len=116 tot-seq=19

AACCACAAGGTAGCAGTCCCTGAATCGCCATGTGGCACATCTGCCTTAGACTGTTATCTG

AGCAGGTAATAAACTTCTGTGATGTTAAGCCACAGAGATTTCGGGGCTTATCTGTT

>1201 len=135 tot-seq=19

TGGGGGATATTGCACAATGGGGGAAACCCTGATGCAGCAACGCCGCGTGAAGGATGAAGG

TTTTCGGATTGTAAACTTCTGTTCTTAGTGACGATAATGACGGTAGCTAAGAAGAAAGCT

CCGGCTAACTACGTG

>1202 len=135 tot-seq=19

TGGGGAATTTTGCGCAATGGGGGAAACCCTGACGCAGCAACGCCGCGTGCGGGATGACGG

CCTTCGGGTTGTAAACCGCTTTCAGTGGGAAAGATTATGACGGTACCCACAGAAGAAGCT

CCGGCTAACTACGTG

>1203 len=135 tot-seq=19

TAGGGAATCTTCGGCAATGGGGGGAACCCTGACCGAGCAACGCCGCGTGAACGATGAAGG

TCTTCGGATCGTAAAGTTCTGTTGCAGGGGAAGATAATGACGGTACCCTGTGAGGAAGCC

CCGGCTAACTACGTG

>1204 len=135 tot-seq=19

TCGGGAATATTGCACAATGGAGGAAACTCTGATGCGGTGACGCCGCGTATAGGAAGAAGG

TCTTAGGATTGTAAGCTATTGTTATGTGGGATGAAGAAGACAGTACCACATGAGAAAGCC

CTGGCTAAATATGTG

>1205 len=135 tot-seq=19

TGGGGAATTTTGCACAATGGAGGAAACTCTGATGCAGCGACGCCGCGTGAGTGATGAAGA

TCTTCGGATTGTAAAACTCTGTCCTTAGTGAAGATAATGACAGTAACTAAGAAGCAAGCC

CTGGCTAAATACGTG

>1206 len=135 tot-seq=19

TGGGGAATATTGGACAATGGGCGCAAGCCTGATCCAGCCATGCCGCGTGGGTGAAGAAGG

CCTTAGGGTTGTAAACCCCTTTCAGCGGGGAAGATAATGACGGTACCCGCAGAAGAAGTC

CCGGCTAACTCCGTG

>1207 len=136 tot-seq=19

TTTGGAATCTTGCCCAATGGGGGAAACCCTGAGGCAGCGACGCCGCGTGGAGGATGAAGG

ATTTAGGTTCGTAAACTCCTTTTGCCAGGAAAGACTTAGGACGGTACCTGGCGAATAAGC

CCCGGCTAACTCCGTG

>1208 len=136 tot-seq=19

TGGGGAATATTGGGCAATGGGCGAAAGCCTGACCCAGCGACGCCGCGTGCGGGAAGACGG

TCTTCGGATTGTAAACCGCTTTTATAAGGGACGAAAGATGACGGTACCTTATGAATAAGC

CACGGCTAACTACGTG

>1209 len=137 tot-seq=19

TGGGGAATATTGGGCAATGGGCGCAAGCCTGACCCAGCAACGCCGCGTGAAGGAAGAAGG

CTTTCGGGTTGTAAACTTCTTTTGACAGGGAAGAGCAGAAGACGGTACCTGTCGAATAAG

CCACGGCTAACTACGTG

>1210 len=138 tot-seq=19

TGGGGAATATTGGGCAATGGGCGCAAGCCTGACCCAGCAACGCCGCGTGAAGGAAGAAGG

CTTTCGGGTTGTAAACTTCTTTTATTAAGGACGAAACAAATGACGGTACTTAATGAATAA

GCTCCGGCTAACTACGTG

>1211 len=142 tot-seq=19

TGGGGAATCTTCCGCAATGGACGAAAGTCTGACGGAGCAACGCCGCGTGCGGGATGAAGG

CCTTCGGGTTGTAAACCGCTTTCAGCAGGGACGAGGCCGCAAGGTGACGGTACCTGCAGA

AGAAGCCCCGGCTAACTACGTG

>1212 len=151 tot-seq=19

TGGGGGATATTGCACAATGGAGGAAACTCTGATGCAGCGACGCCGCGTGAGTGAAGAAGT

ATTTCGGTATGTAAAGCTCTATCAGCAGGGAAGAAAGACCTCGAAAGAGGGGATGACGGT

ACCTGAGTAAGAAGCCCCGGCTAACTACGTG

>1213 len=154 tot-seq=19

TGAGGAATATTGGTCAATGGCCGGAAGGCTGAACCAGCCATGCCGCGTGAAGGTCAGTGC

CCTATGGGCGTTAAACTTCTTTTGTGCGGGAGCAATAAGGTCCACGTGTGGACCGACGAG

AGTACCGTACGAATAAGCATCGGCTAACTCCGTG

>1214 len=155 tot-seq=19

TAAGGAATATTGGTCAATGGACGCAAGTCTGAACCAGCCATGCCGCGTGGAGGATGAAGG

CTCTCTGGGTTGTAAACTTCTTTTATCTGGGAAGAAACCCATCTTTTCTAAGGTGGTTGA

CGGTACCAGATGAATAAGCACCGGCTAACTCCGTG

>1215 len=160 tot-seq=19

TGGGGAATCTTGCACAATGCGCGAAAGCGTGATGCAGCAACGCCGCGTGCGGGAAGAAGG

CCTTAGGGTTGTAAACCGCTTTCAGTTGGGACGAAGCTTCTTGGGTTAATAGCCTGTTGA

AGTGACGGTACCTTCAGAAGAAGCACCGGCTAACTACGTG

>1216 len=160 tot-seq=19

TAGGGAATCTTCCACAATGGACGCAAGTCTGATGGAGCAACGCCGCGTGAGTGAAGAAGG

CCTTCGGGTTGTAAAACTCTGTCCTCATCCAAGAGAAGGGAAGGTAGTAACTGACCTTTC

TAGGACGGTAGATGAGGAGGAAGCCCCGGCTAACTATGTG

>1217 len=160 tot-seq=19

TGGGGAATTTTGGACAATGGGGGGAACCCTGATCCAGCCATGCCGCGTGTATGAAGAAGG

CCTTAGGGTTGTAAAGTACTTTTGTTAGGGAAGAAAAGGCATTTGCTAATATCAGATGCT

GATGACGGTACCTAAAGAATAAGCACCGGCTAACTACGTG

>1218 len=160 tot-seq=19

TAGGGAATCTTCCGCAATGGACGCAAGTCTGACGGAGCAACGCCGCGTGTGTGAAGAAGG

TTTTCGGATCGTAAAGCACTGTTATTAGAGAAGAACAGCCGATAGAGTAACTGTTATCGG

TTTGACGGTATCTAACCAGAAAGTGACGGCTAACTACGTG

>1219 len=160 tot-seq=19

TAGGGAATCTTCCGCAATGGACGCAAGTCTGACGGAGCAACGCCGCGTGTGTGAAGAAGG

TTTTCGGATCGTAAAGCACTGTTGTTAGAGAAGAACGGCTACTAGAGGAAATGTTAGTAG

ATTGACGGTATCTAACCAGAAAGCCACGGCTAACTACGTG

>1220 len=161 tot-seq=19

TAGGGAATCTTCCACAATGGACGCAAGTCTGATGGAGCAACGCCGCGTGAGTGAAGACGG

CCTTCGGGTTGTAAAACTCTGTGATTCGGGACGAAAGATAAGTAGACGAATAATCTGCAT

AAGTGACGGTACCGAAAAAGCAAGCCACGGCTAACTACGTG

>1221 len=177 tot-seq=19

GCAGGATTGGAGCCTTATCCAGGGGCTCCAAAGCCAACATGGGACTGGGCAAGAAAGAAA

CAGAGCAGAGTGGAGGACTGGTGTCTGCCTCAATGGACACAGCTGCTGGACGCGAGGACG

TGAGGCCCTCCATGAGCTTCTTGCTCAGGTAGCCTTATCTTCCTTTCCACCTCCCTC

>1222 len=203 tot-seq=19

TCACAGCTATACTGTGACCAGAATCTGGGAGCACTTAGATGCTGGGTGGTCCTGGCTCGG

GCCTTTGTCTAAAGACTTGACAAGCACTGACTGTTGGACTTCCTCTCTGTGACTAGCCAA

CTGCCTGAAGACTCTCCTGTGCTCTCTTCCTGGGCTGGTTGAACTTCCTCTAAGGAGTGT

GGGTGGCTCCCCTCAGTCTGACC

>1223 len=212 tot-seq=19

GATGTGGCGTCTTCCCAGGTTCATGGCGCTCTGCTGTTTTACCAGAAGCAGACACAGAGA

TGGGAGTGGCACCTTCCCCCGAGACCGTGGGTGGGAGCTGTGTCCACCACCCAGCAGTGC

GCAAGGGCTTTGGGCCCAGGGCCCTCCTGAGAATCCCCTCTGCCTCACTGAGAAAATTGA

CTCCTGTTCGGTCCCTCTCCTGTTCGGTCCCT

>1224 len=230 tot-seq=19

CCCAGGCCTCAGGCGGGCTCCCTGCCCACACTGTGTCCAGCTCGGAGGACTCGAAGTCTC

AGCTGGGCGTGCCCCTGTGGGATAGGTGCTGCCCCCAAAGACTGCAGGAGATAAGAGGAG

ACCCTCAATTCTCCAGATGCCTTCCCTTCCGAACCCTGCCATGTGTCCTATCCTTGTGGG

TCTGTCCATCTCATCTCCCAGGCCCAGACCATCTCTCCCACCGCAGCAGC

>1225 len=136 tot-seq=19

TGGGGAATATTGCGCAATGGGGGAAACCCTGACGCAGCAACGCCGCGTGATTGAAGAAGG

CCTTCGGGTTGTAAAGATCTTTAATTGGGGACGAATTTTGACGGTACCCAAAGAATAAGC

TCCGGCTAACTACGTG

>1226 len=198 tot-seq=19

TGAGGAATATTGGTCAATGGACGAGAGTCTGAACCAGCCAAGTAGCGTGAAGGATGACTG

CCCTATGGGTTGTAAACTTCTTTTATATGGGAATAAAGTGGTCCACGTGTGGATTTTTGT

ATGTACCATATGAATAAGGATCGGCTAACTCCGTGGGGGGGCCGCGGTAATTAGAAGATC

GGAAGAGCGTCGTGTAGG

>1227 len=156 tot-seq=19

TGAGGAATATTGGTCAATGGGCGAGAGCCTGAACCAGCCAAGTCGCGTGAAGGAAGACTG

CCCGCAAGGGTTGTAAACTGCTTTTGTTTGGGAATAATCGACATTACGTGTAATGTTTTG

CATGTACCATTCGAATAAGGACCGGCTAATTCCGTG

>1228 len=140 tot-seq=19

TGGGGAATATTGCACAATGGGCGAAAGCCTGATGCAGCGACGCCGCGTGCGGGATGACGG

CCTTCGGGTTGTAAACCGCTTTCAGTAGGGACGAAGCTTTTGTGACGGTACCTACAGAAG

AAGCACCGGCTAACTACGTG

>1229 len=140 tot-seq=19

TGGGGAATATTGCGCAATGGGGGAAACCCTGACGCAGCGACGCCGCGTGCGGGATGAAGG

CCTTCGGGTTGTAAACCTCTTTCAGCAGGGACGAAGCGCAAGTGACGGTACCTGCAGAAG

AAGCACCGGCTAACTACGTG

>1230 len=161 tot-seq=19

TGGGGAATATTGCACAATGGGCGGAAGTCTGACCGAGCAACGCCGCGTGAGTGAAGAAGG

TTTTCGGATCGTAAAGCTCTGTTGTTAGAGAAGAACGTTGGTAGGAGTGGAAAATCTACC

AAGTGACGGTAACTAACCAGAAAGGGACGGCTAACTACGTG

>1231 len=160 tot-seq=19

TGGGGAATATTGCGCAATGGGGGCAACCCTGACGCAGCCATGCCGCGTGAATGAAGAAGG

CCTTCGGGTTGTAAAGTTCTTTCGGTGACGAGGAAGGTTGTTGTGTTAATAGCGCAACAA

ATTGACGTTAATCACAGAAGAAGCACCGGCTAACTCCGTG

>1232 len=160 tot-seq=19

TAGGGAATCTTCCACAATGGACGCAAGTCTGATGGAGCAACGCCGCGTGAGTGAAGAAGG

GTTTCGGCTCGTAAAGCTCTGTTGTTGGAGAAGAACGTGCGTGAGAGTAACTGTTCACGC

AGTGACGGTATCCAACCAGAAAGTCACGGCTAACTACGTG

>1233 len=122 tot-seq=19

GGCAGAGATTGGACTGAGAGAGATAGAGAGAGGGAGGGAGAAGGGGAGGGATAAAGAGGG

ATGGAGAGAAAGAGGGAGAGACAGCAAGTGAGCCAGACCATGAGGGGCACCCACCGACCT

CA

>1234 len=160 tot-seq=19

TGGGGAATATTGCGCAATGGGGGAAACCCTGACGCAGCGACGCCGCGTGCGGGATGAAGG

CCTTCGGGTTGTAAAGCTCTGTGATTCGGGACGAAAGGCCATATGTGAATAATATATGGA

AATGACGGTACCGAAAAAGCAAGCCACGGCTAACTACGTG

>1235 len=160 tot-seq=19

TAGGGAATCTTCCGCAATGGACGAAAGTCTGACGGAGCAACGCCGCGTGAGTGAAGAAGG

TTTTCGGATCGTAAAACTCTGTTGTTAGAGAAGAACAAGGATGAGAGTAACTGCTCGTCC

CTTGACGGTATCTAACCAGAAAGCCATGGCTAACTACGTG

>1236 len=115 tot-seq=19

TGATATGAGACCCTGTCCTGTGAGTCAACCCTAATGCCAGCTGGGTGATGTAAGCATCTG

TCGGGGGCACCATTCACCTGCTGGCTCCTGGCCTTTCTTTGGCCATGATCCACAC

>1237 len=155 tot-seq=19

TGAGGAATATTGGTCAATGGGCGAGAGCCTGAACCAGCCAAGTAGCGTGCAGGATGACGG

CCCTATGGGTTGTAAACTGCTTTTGTATGGGGATAAAGTTAGGGACGTGTCCCTATTTGC

AGGTACCATACGAATAAGGACCGGCTAATTCCGTG

>1238 len=107 tot-seq=18

AAGGGCTTTAAACTCAATTCTCTGAGCTGTAGCCAAACCCCAAGGCCTCCTGGACACCGA

GAGCTGGACAGTGCCTGGCTGACCACCCAGGCTGCATGCAGCGTTAC

>1239 len=110 tot-seq=18

TGAGTTTTTGACTGTAGAAAAATTTAAAGCTGTATAGATTACATAACCAAATATAGCTGT

TATGATTCCTTTTGAAATCAATCCAAGATATCACCCTTCTCCAAAAAATG

>1240 len=135 tot-seq=18

TGGGGAATATTGCACAATGGGCGAAAGCCTGATGCAGCGACGCCGCGTGAAGGATGAAGT

ATTTCGGTATGTAAACTTCTATCAGCAGGGAAGAAAATGACGGTACCTGACTAAGAAGCC

CCGGCTAACTACGTG

>1241 len=135 tot-seq=18

TGGGGAATATTGCACAATGGGGGGAACCCTGATGCAGCGATGCCGCGTGGAGGAAGAAGG

TTTTCGGATTGTAAACTCCTGTCGTAAGGGACGATAATGACGGTACCTTACAAGAAAGCT

CCGGCTAACTACGTG

>1242 len=140 tot-seq=18

TGGGGAATATTGCACAATGGGCGAAAGCCTGATGCAGCGACGCCGCGTGCGGGATGACGG

CCTTCGGGTTGTAAACCGCTTTCAGCAGGGAAGAAGCGCAAGTGACGGTACCTGCAGAAG

AAGCGCCGGCTAACTACGTG

>1243 len=147 tot-seq=18

ACCCCTATGCCACGCGTCCACCTGGGGCTGTCCATCACTTCCTGCAAGATCTTGCTGGTA

GCCAGATTCTCAGAGACCCCCAGTGAGCCCCGCCTCCTGGTTTTCACACCCTGTGTAGTT

TCCTTTCCCCAGGGATGGTCCAAGTGA

>1244 len=155 tot-seq=18

TGAGGAATATTGGTCAATGGACGAAAGTCTGAACCAGCCAAGTAGCGTGCAGGACGACGG

CCCTCTGGGTTGTAAACTGCTTTTAGTTGGGAATAAAACGCGGTACGTGTACCGCCTTGT

ATGTACCATCAGAAAAAGGACCGGCTAATTCCGTG

>1245 len=155 tot-seq=18

TGAGGAATATTGGTCAATGGACGCAAGTCTGAACCAGCCAAGTAGCGTGCAGGACGACGG

CCCTCCGGGTTGTAAACTGCTTTTATAAGGGAATAAAGTGAGCCTCGTGAGGCTTTTTGC

ATGTACCTTATGAATAAGGACCGGCTAATTCCGTG

>1246 len=159 tot-seq=18

TCGAGAATCTTTCACAATGGGGGCAACCCTGATGGAGCGACGCCGCGTGGAGGATAAGGT

CTTCGGATTGTAAACTCCTGTCATGTGTGAACAAGGTTGTCGTGTTAATAGCATGGCAAA

TTGATGGTAACACAAGAGGAAGAGACGGCTAACTCTGTG

>1247 len=160 tot-seq=18

TGGGGAATATTGCGCAATGGGGGAAACCCTGACGCAGCGACGCCGCGTGCGGGATGAAGG

CCTTCGGGTTGTAAAGCACTTTCAGCGGGGAGGAAGGCGATAAGGTTAATAACCTTGTCG

ATTGACGTTACCCGCAGAAGAAGCACCGGCTAACTCCGTG

>1248 len=161 tot-seq=18

TAGGGAATTTTTCACAATGGGCGAAAGCCTGATGGAGCAATGCCGCGTGAATGATGAAGG

TCTTATAGATTGTAAAATTCTTTTATTTGGGAAGAAAAACTAAAGTAGGAAATGACTTTA

GTCTGACTGTACCATTTGAATAAGTATCGGCTAACTATGTG

>1249 len=179 tot-seq=18

AGACGGTGCTAACAGGAAAGTTCATGGCCCTAAACACCTACATCAAAAAGACTGAAAGAG

CACAAACTGACATGCTAAGGTCACACTTCAAGGAACTAGAGAAATAAGAACAAACCAAAC

CTAAATAAACCCCCCAGCCCGGTAATCACAAGATCGGAAGAGCGTCGTGTAGGGAAAGA

>1250 len=184 tot-seq=18

GAGAAGGGAAAAGAAGCAGAGGCTTATAGCAACCAACACATGATTCTTCAGAGCTAGATG

TGGAGTCTCCTAACTGGCTTGCAGACTCCACCATAGGCTCACCCATGAGCCCTACAGGAC

ACTTCACCTGCAGAACCCCTCAACTGACAAGCACACCCAGCACTCCATGCACCCACCCAC

TAGG

>1251 len=196 tot-seq=18

AAGAGGGGAGAAGGAGAAAAATAGAGGTGAGAGTGGTAATGAGAGGTGAGAGTGGGATGA

GATTCTTTAACATCTGCTGCCTGTTCATAGTTCTCAGTCCCGAGAGACGGAATATATTAG

CATGAAAAGTACTCTCTTCATTTTATCTGGGCACTAGGTCATCTTTTAGCTCTCACTCTG

TGTACTGAACTGTATG

>1252 len=198 tot-seq=18

CAAGAGCAATAAGAGTTCTTAACCGCTGAGCCATCTCACTAGGCCATTTTGGTTTTTTTA

GGCAGCAGGATTGGGACTGGCCTGGCTCTCTCAGACTGGATGCATCCAGCAGATACTGAC

AGGCCTTCAAGTATGATTGAGAGGAGTCACCCTGGAGATGCAGGAAAACCCACAATGGGG

GATGGAAGAAGTGGGTGG

>1253 len=203 tot-seq=18

TGAGGAATATTGGTCAATGGCCGGAAGGCTGAACCAGCCAAGCCGCGTGAAGGACGAAGG

TGCCAAGCATTGTAAACTTCTTTTGTCAAGGGACAAAATCAGGCTCGAGAGCCTGACCGA

GGGTACTTGAAGAAAAAGCATCGGCTAACTCCGTGAGAGCCTGACCGAGGGTACTTGAAG

AAAAAGCATCGGCTAACTCCGCG

>1254 len=209 tot-seq=18

TGGGGAATATTGCACAATGGGCGAAAGCCTGATGGAGCAACGCCGCGTGGGTGAAGAAGG

TCTTCGGATCGTAAAACCCTGTTGTTAGAGAAGAAAGTGCGTGAGAGTAACTGTTCACGT

TTCGACGGTATCTAACCAGAAAGCCACGGCTAACTACGTGCCAGCAGTCGCGGAATGACA

AGATCGGAAGAGCGTCGTGTTGGGGAATA

>1255 len=230 tot-seq=18

GTTTGGGCAAAGGAAGGAGTAAAGCTTGAGTGCAGGCTTGATAAAGCCTTTGCCCAGCCC

CGGGAGCTCTGGAGCATGCATGGCCCAAGTTGTCCCCTGTTGGACAGAAATAGCCAGGCA

TCTGTGATGAGTCACGGGATGTAGGCTTCCCTGGGAAGGGCATGCTCATGACTGAGCAGC

TTTCAGGAGCTGAAAGATCTGACAGCTGGAGCTATCAGCCCACGGCACCC

>1256 len=160 tot-seq=18

TGGGGAATATTGCGCAATGGGGGCAACCCTGACGCAGCCATGCCGCGTGAATGAAGAAGG

CCTTCGGGTTGTAAAGTTCTTTCGGTAGCGAGGAAGGTGGTTAGTTTAATAGGCTAGCCA

ATTGACGTTAACTACAGAAGAAGCACCGGCTAACTCCGTG

>1257 len=135 tot-seq=18

TGGGGAATCTTGGACAATGGGCGAAAGCCTGATCCAGCGACGCCGCGTGGGTGATGAAGG

CCTTCGGGTTGTAAAGCCCTTTCGCCGGGGACGATGATGACGGTACCCGGAGAAGAAGCA

CCGGCTAACTCCGTG

>1258 len=148 tot-seq=18

TGGGGAATATTGCACAATGGACGGAAGTCTGATGCAGCGACGCCGCGTGGGGGATGAAGG

CTTTCGGGTTGTAAACTCCTTTTGATGCTGAACAAGGCTCTTTTTAGGGTTGAGGGTAGG

TGTTGAATAAGCGCCGGCTAACTACGTG

>1259 len=130 tot-seq=18

AACCAACCCTTCCCCTTAGGTAGCCATTGTGTCCAGAGGGTGGGCAACACATATCCAAAA

GCAGTCTCCTGGAGGGGAGAGAGGGCGTCTCACGCATCCCTTTCTGGGTCTCTGAAGGCA

GAGTGCTGAG

>1260 len=169 tot-seq=18

AGGTGGGGAGGCGATAACTATCCTAGCTCTGCCACTCACTAGCCTTGAATAAACTGGGGT

TAACATTTCCCAGCCTCAGGTTTTTTTAAATCTTAAAGTGGGGACAATAATAATAGACCT

TGATGCATGCATTGTTTTGAGGATTAAGTGAGATAAACTTGTAGACCAC

>1261 len=160 tot-seq=18

TAGGGAATCTTCCGCAATGGACGAAAGTCTGACGGAGCAACGCCGCGTGAGTGAAGAAGG

TTTTCGGATCGTAAAACTCTGTTGTAAGGGAAGAACAAGTACGTTAGTAACTGAACGTAC

CTTGACGGTACCTTACCAGAAAGCCACGGCTAACTACGTG

>1262 len=155 tot-seq=18

TGAGGAATATTGGTCAATGGGCGAGAGCCTGAACCAGCCAAGTCGCGTGAAGGAAGACGG

TTCTATGGATTGTAAACTTCTTTTGCAGGGGAGTAAAACAAGGGATGTATCCCTTCTTGC

AAGTACCCTGCGAATAAGCATCGGCTAACTCCGTG

>1263 len=135 tot-seq=18

TGGGGGATATTGCACAATGGAGGAAACTCTGATGCAGCGACGCCGCGTGAGTGAAGAAGT

ATTTCGGTATGTAAAGCTCTATCAGCAGGGAAGAAAATGACGGTACCTGACTAAGAAGCC

CCGGCTAACTACGTG

>1264 len=236 tot-seq=18

AGGGGAGTAGGCCCTAGAGTTCAGGCTTCACACGCAGGCACTCAAGCTCATCAGGCCCAG

TGGGAGCAGTGGCCTGAGGAAGTAGGCAGAAGGCTCGGCAGAGTCAGCTTGAGGAGCAGA

GGGCTAGAAGCCCAGGCCAGGGCCCAGAGTAGCACAACAGCAGAAACCCACGCCAGCGAG

AGTAAGCAGCGAGAGCCCAGGAGCCGGGCAGCAATCTGCACACTGGGCAAGGACCA

>1265 len=136 tot-seq=18

TGGGGAATATTGCACAATGGGGGAAACCCTGATGCAGCAACGCCGCGTGAGTGAAGAAGT

ATTTCGGTATGTAAAGCTCTATCAGCAGGGAAGAAGAGAGACGGTACCTGACTAAGAAGC

CCCGGCTAACTACGTG

>1266 len=138 tot-seq=18

TGGGGAATATTGCACAATGGGCGAAAGCCTGATGCAGCAATTCTGTGTGTGTGAAGAAGG

TTTTAGGACTGTAAAACACTTTTAGTAGGGAAGAAAAAAATGACGGTACCTACAGAAGAA

GCAACGGCTAAATACGTG

>1267 len=135 tot-seq=18

TGGGGAATATTGGACAATGGAGGAAACTCTGATCCAGCAATTCTGTGTGTGTGAAGAAGG

TTTTAGGACTGTAAAACACTTTTAGTAGGGAAGAAAATGACGGTACCTTACTAAGAAGCT

CCGGCTAAATACGTG

>1268 len=160 tot-seq=18

TAGGGAATCTTCGGCAATGGGGGCAACCCTGACCGAGCAACGCCGCGTGAGTGAAGAAGG

TTTTCGGATCGTAAAACTCTGTTGTTAGAGAAGAACACGAGTGAGAGTAACTGTTCATTC

GATGACGGTATCTAACCAGCAAGTCACGGCTAACTACGTG

>1269 len=136 tot-seq=18

TGGGGGATATTGCACAATGGGGGAAACCCTGATGCAGCGACGCCGCGTGAGGGAAGACGG

TTTTCGGATTGTAAACCTCTGTCTTTGGGGACGAATTGAGACGGTACCCAAGGAGGAAGC

TCCGGCTAACTACGTG

>1270 len=104 tot-seq=17

TGAAGAGAACCCAGAGAGTCAGCGCCCAAGCACACGGCACTCAGGAGACTGATGGGCGGG

AACACATAGGAAAAAGCCTCTATCAGCCACTGGGCATCTCGAGG

>1271 len=136 tot-seq=17

TCGGGAATATTGCACAATGGAGGAAACTCTGATGCAGTGACGCCGCGTGCAGGAAGAAGG

TTTTCGGATTGTAAACTGCTTTAGATAGGGAAGATAAAAGACTGTACCTATAGAATAAGC

TCCGGCTAACTACGTG

>1272 len=138 tot-seq=17

TGGGGGATATTGCACAATGGGGGGAACCCTGATGCAGCAACGCCGCGTGAGTGAAGAAGG

CCCTAGGGTCGTAAAGCTCTGTCGTATGGGAAGAAACAAATGACTGTACCATACAAGAAA

GCCCCGGCTAACTACGTG

>1273 len=142 tot-seq=17

TGGGGAATATTGCACAATGGGCGCAAGCCTGATGCAGCGACGCCGCGTGAGGGATGGAGG

CCTTCGGGTTGTAAACCTCTTTTAGCAGGGAGCAAGGCTTCGGTTGAGTGTACCTGTTGA

ATAAGCACCGGCTAACTACGTG

>1274 len=148 tot-seq=17

TGGGGAATCTTGCACAATGGGCGGAAGCCTGATGCAGCGACGCCGCGTGAGGGATGACGG

CCTTCGGGTTGTAAACCTCTTTCGCTAGGGAAGAAGCCGCTTTGTGGTGGTGACGGTACC

TGGATAAGAAGCACCGGCTAACTACGTG

>1275 len=155 tot-seq=17

TGAGGAATATTGGTCAATGGGCGAGAGCCTGAACCAGCCAAGTCGCGTGAAGGAAGACTG

TCCTAAGGATTGTAAACTTCTTTTATACGGGAATAACGGGCGATACGAGTATTGCATTGA

ATGTACCGTAAGAATAAGCATCGGCTAACTCCGTG

>1276 len=155 tot-seq=17

TGAGGAATATTGGGCAATGGGCGCAAGCCTGACCCAGCCATGCCGCGTGCAGGATGACGG

CGCTATGCGTTGTAAACTGCTTTTGTTTAGGAAGAAACCCATCTACGTGTAGGTGGTTGA

CGGTACTGAATGAATAAGCATCGGCTAACTCCGTG

>1277 len=160 tot-seq=17

TGGGGAATATTGCACAATGGGCGCAAGCCTGATGCAGCAACGCCGCGTGAACGATGAAGG

TCTTCGGGTTGTAAAGTACTTTCAGCGGGGAGGAAGGGAGTAAAGTTAATACCTTTGCTC

ATTGACGTTACCCGCAGAAGAAGCACCGGCTAACTCCGTG

>1278 len=160 tot-seq=17

TGGGGAATATTGCGCAATGGGCGAAAGCCTGACGCAGCGACGCCGCGTGAGGGATGAAGG

TTCTCGGATCGTAAACCTCTGTCAGAAGGGAAGAAATTATATCATGCTAATCAGTGGTGT

ATTGACGGTACCTTCAAAGGAAGCACCGGCTAACTCCGTG

>1279 len=161 tot-seq=17

TGGGGAATATTGCGCAATGGGGGAAACCCTGACGCAGCGACGCCGCGTGAACGATGACGG

CCTTCGGGTTGTAAAGTTCTGTTATACGGGACGAATGGTACGACGGTCAATACCCGTCGT

AAGTGACGGTACCGTAAGAGAAAGCCACGGCTAACTACGTG

>1280 len=161 tot-seq=17

TGGGGAATATTGCGCAATGGGGGAAACCCTGACGCAGCGACGCCGCGTGAACGATGAAGG

TCTTATAGATTGTAAAGTTCTTTTATATGGGAAGAAACGCTAAGATAGGAAATGATTTTA

GTTTGACTGTACCATTTGAATAAGTATCGGCTAACTATGTG

>1281 len=176 tot-seq=17

TGGGGAATATTGCACAATGGGGGAAACCCTGATGCAGCGACGCCGCGTGAGCGAAGAAGG

CTTTCGAGTCGTAAAGCTCTGTCCTATGAGAAGATAATGACGGTACCCTGTGAGGAAGCC

CCGGCTAACTACGTGCCAGCAGCCGCGGTATATAGAGATCGGAAGAGCGTCGTGTA

>1282 len=179 tot-seq=17

TGGGGAATTTTGGACAATGGGGGCAACCCTGATCCAGCAATGCCGCGTGCAGGAAGAAGG

CCTTCGGGTTGTAAACTGCTTTTGTACGGAACGAAAAGGCTCTGGTTAATACCTGGGGCA

CATGACGGTACCGTAAGAATAAGCACCGGCTAACTACGTGCCAGAGCCGCGGTAATATA

>1283 len=183 tot-seq=17

TGGGGAATTTTGGACAATGGGGGAAACCCTGATCCAGCAATGCCGCGTGCAGGATGAAGG

CCTTCGGGTTGTAAACTGCTTTTGTACGGAACGAAAAGTCTTGGGTTAATACCCTGGGAT

CATGACGGTACCGTAAGAATAAGCACCGGCTAACTACGTGCCAGCAGCCGCGGTATGCCC

AGA

>1284 len=185 tot-seq=17

CAACCTTCCATCTACCCCCCTTTTCTACTCCAGCTCCACCTGCCAGCTGCTTCTGCCACC

TTCCCCACAACTCCCAAGGCTGGATAGGTAAGGAGGATCCGGGCCAAAGGGGCCTCCTTT

CAGTAAGAGGCGGAATCACTACCTCAGCTTTCTCTCAATCCCCTTTACTACGCAAAGATC

AGCCC

>1285 len=199 tot-seq=17

GCAGGGGGGCACAGAGGGAGGCCCTGCCTGAGCCAAAGCCACTGAGAGTTTCCCAGAGCC

ACATGGGAGGGACACTCCAGGGACGTGCCTAGGGCTCAGGACAGAAGGTGTCTCCAGCGC

GGGCAGGATTTCCCGAGTGTGAGCTTCCACCTAAGAAAGCCGCTCCCTGTGGCCAGGAGA

CAATGCCCCAAACCCCCGC

>1286 len=204 tot-seq=17

TGAGGAATATTGGTCAATGGGCGAGAGCCTGAACCAGCCAAGTAGCGTGCGGGAAGACGG

CCCTATGGGTTGTAAACCGCTTTTGATTGGGAACAAAGAGCGCCACGTGTGGTGCGTTGC

GTGTACCTTTCGAATAAGCATCGGCTAATTCCGTGGTGTGGTGCGTTGCGTGTACCTTTC

GAATAAGCATCGGCTAATTCCGTG

>1287 len=206 tot-seq=17

TGTAAATGGAGCAATCTGGGAAGACCTCACTGAGGAGGTGACTTTTGAGCAAAGACCTGG

AGGAGGTAGCAAGGGAGATAGCCATGTGAGGATGCAAGGGAAAGGCATCCAGGAAGAGGG

AGCAGCATGTGCAAAGATCCTGCGGCACTGAGGCCAGGGGATGGAGCAGAAGAGGAACGG

GGTTTGATGGTTGATAAAGCATCCCT

>1288 len=220 tot-seq=17

GTCATTGGGGTTGCTCCCAGCTGATGGCTCTTTCCTCAGTGAAGTGGAAGGTGAGGACCT

CTGCTGGGCATGAAGGTGGCAGGGAATGTCTGAGCAGAGTGAAGGAAGTCTGAAATAGGT

CCCATAAAGCAAGGCAGATTTCCTGAAGGGGCCGAGGAGCATCATGGGCAGGCTGAGGCC

TCTGAAGGCTGCGGCCCCTTCTCGAGAGCCAGCACCTTGC

>1289 len=161 tot-seq=17

TAGGGAATTTTCGTCAATGGGGGGAACCCTGAACGAGCAATGCCGCGTGAGCGAGGAAGG

TCTTCGGATCGTAAAGCTCTGTTGCCGGGGACAAAAGGCAGAAAGAGTGGAAAGCTTTCT

GAGTGATGGTACCCGGCGAGGAAGTCACGGCTAACTACGTG

>1290 len=160 tot-seq=17

TGGGGAATATTGGACAATGGGGGGAACCCTGATCCAGCCATGCCGCGTGTGTGAAGAAGG

CCTTTTGGTTGTAAAGCACTTTAAGTAGGGAGGAAGACCTAATAATTAACACCTATTAGG

AATGACGTTACCTACAGAATAAGCACCGGCTAACTCTGTG

>1291 len=107 tot-seq=17

GACAGTATTTCACCATCTTGCTGTTTCCTAGCCTTGAGTTGGGCACATGTGTTGAGAACT

GGGGAAAGTTGGGGAGTCACCATCATAGACACATGAACACCTGTACT

>1292 len=160 tot-seq=17

TGGGGAATCTTCCGCAATGGACGAAAGTCTGACGGAGCAACGCCGCGTGAGTGATGAAGG

TCTTCGGATTGTAAAACTCTGTTGTTAGAGAAGAACACGAGTGAGAGTAACTGTTCATTC

GATGACGGTATCTAACCAGCAAGTCACGGCTAACTACGTG

>1293 len=138 tot-seq=17

TGGGGAATATTGCACAATGGGCGCAAGCCTGATGCAGCAACGCCGCGTGAACGAAGAAGG

TCTTCGGATTGTAAAGTTCTGTCCTTGGGGAAGAAGGAAGTGACGGTACCCAAGGAGGAA

GCCCCGGCTAACTACGTG

>1294 len=210 tot-seq=17

TAGGGAATTTTTCACAATGGGCGCAAGCCTTATGAAGCAATGCCGCGTGAACGATGAAGG

TCTTATAGATTGTAAAGTTCTTTTATATGGGAAGAAACGCTAAGATAGGAAATGATTTTA

GTTTGACTGTACCATTTGAATAAGTATCGGCTAACTATGTGCCAGCAGCCGCGGATCGGA

AGAGCGTCGTGTAGGGAAAGAGTGTGGGGG

>1295 len=160 tot-seq=17

TAGGGAATCTTCCACAATGGACGCAAGCCTGATGGAGCAACGCCGCGTGAGTGAAGAAGG

GTTTCGGCTCGTAAAGCTCTGTTGTTGGAGAAGAACGTGAGTGAGAGTAACTGTTCACGG

AGTGACGGTAACCAACCAGAAAGTCACGGCTAACTACGTG

>1296 len=109 tot-seq=16

ATTGATAAGCAAAAGGTAGCAGCTATGACAGGAAGTTTCTTTACCACTTGGTGAGTGGTT

TCTATAAACCAGCACTGACCCTTTCCCTTTGGGGCATTTCTTTGTCACC

>1297 len=135 tot-seq=16

TAGGGAATCTTCCACAATGGGGGAAACCCTGATGCAGCAACGCCGCGTGAGTGATGAAGG

TTTTCGGATCGTAAAGCTCTGTCTTTGGGGAAGATAATGACGGTACCCAAGGAGGAAGCC

ACGGCTAACTACGTG

>1298 len=135 tot-seq=16

TGGGGAATATTGCACAATGGGCGAAAGCCTGATGCAGCAACGCCGCGTGAGCGATGAAGG

TCTTCGGATCGTAAAGCTCTGTCCTTGGGGAAGATAATGACGGTACCCTTGGAGGAAGCC

CCGGCTAACTACGTG

>1299 len=135 tot-seq=16

TGGGGAATATTGCACAATGGGGGAAACCCTGATGCAGCGACGCCGCGTGAGTGAAGAAGT

ATCTCGGTACGTAAAGCTCTATCAGCAGGGAAGAAAATGACGGTACCTGATTAAGAAGCC

CCGGCTAACTACGTG

>1300 len=135 tot-seq=16

TGGGGAATATTGCACAATGGGCGAAAGCCTGATGCAGCGACGCCGCGTGAACGAAGAAGG

CTTTCGAGTCGTAAAGTTCTTTTATATGGGAAGATAATGACGGTACCATAAGAAAAAGCC

CCGGCTAACTACGTG

>1301 len=135 tot-seq=16

TGGGGAATATTGCACAATGGAGGGAACTCTGATGCAGCGACGCCGCGTGAGTGAAGAAGT

AATTCGTTATGTAAAGCTCTATCAGCAGGGAAGAAAGTGACGGTACCTGAAAAAGAAGCT

CCGGCTAAATACGTG

>1302 len=135 tot-seq=16

TCGGGAATTTTGCGCAATGGACGAAAGTCTGACGCAGCAACGCCGCGTGAGGGATGAAGG

CCTTCGGGTTGTAAACCTCTTTTCTCAGGGACGATAATGACGGTACCTGAGGAAGAAGCC

ACGGCTAACTACGTG

>1303 len=136 tot-seq=16

TGGGGAATTTTGCGCAATGGGGGCAACCCTGACGCAGCGACGCCGCGTGCGGGACGAAGT

CATTCGTGACGTAAACCGCTTTCAGCGAGGAAGAACCATGACGGTACTCGCAGAAGAAGC

CCCGGCTAACTACGTG

>1304 len=136 tot-seq=16

TGGGGAATATTGCACAATGGGGGAAACCCTGATGCAGCGACGCCGCGTGAGTGAAGAAGT

ATTTCGGTATGTAAAGCTCTATCAGCAAGGAAGAAGAATGACGGTACTTGACTAAGAAGC

CCCGGCTAACTACGTG

>1305 len=137 tot-seq=16

TGGGGAATATTGCGCAATGGGGGGAACCCTGACGCAGCAACGCCGCGTGAAGGAAGAAGG

CTTTCGGGTCGTAAACTTCAGACGCTAGTGAAGAAATAAATGACGGTAACTAGGCCGAAG

CTCCGGCTAACTACGTG

>1306 len=138 tot-seq=16

TGGGGAATTTTGGACAATGGGCGAAAGCCTGATCCAGCAACGATGCGTGGAGGATGAAGG

TTCTCGGATTGTAAACTCCTTTTGAGAGGGAAGAAAAAAATGACGGTACCTCTCGAATAA

GCCACGGCTAACTACGTG

>1307 len=138 tot-seq=16

TAAGGAATCTTGCACAATGGGGGAAACCCTGATGCAGCAACGCCGCGTGAACGATGAAGG

CCTTCGGGTTGTAAAGTTCTGATCTTGGGGAAGAAGAAGTGACGGTACCCAAGGAGAAAG

CCCCGGCTAACTACGTGG

>1308 len=138 tot-seq=16

TGGGGGATATTGCACAATGGAGGAAACTCTGATGCAGCAATGCCGCGTGAGGGAAGAAGA

TTTTCGGATTGTAAACCTCTGTCCCAGCCGAAGAAACGAATGACGGTAGGCTGGGAGGAA

GCTCCGGCTAACTACGTG

>1309 len=155 tot-seq=16

TGAGGAATATTGGTCAATGGCCGGAAGGCTGAACCAGCCAAGTAGCGTGAAGGACGACGG

CCCTACGGGTTGTAAACTTCTTTTATAAGGGAATAAAGTTGCGTACGCGTACGCATTTGC

ATGTACCTTATGAATAAGCATCGGCTAACTCCGTG

>1310 len=155 tot-seq=16

TGAGGAATATTGGTCAATGGGCGGAAGCCTGAACCAGCCAAGTAGCGTGAAGGATGACTG

CCCTATGGGTTGTAAACTTCTTTTATAAGGGAATAATTGTGGGTACGCGTACCCATTTGC

ATGTACCTTATGAATAAGGATCGGCTAACTCCGTG

>1311 len=155 tot-seq=16

TGAGGAATATTGGACAATGGGTGGGAGCCTGATCCAGCCATTCCGCGTGCAGGAAGAAGG

TCTTATGGATTGTAAACTGCTTTTATATAGGGATAAACCTACTCTCGAGAGGGTAGCTGA

AGGTACTATATGAATAAGCACCGGCTAACTCCGTG

>1312 len=160 tot-seq=16

TGGGGAATATTGCACAATGGGGGAAACCCTGATGCAGCCATGCCGCGTGTGTGAAGAAGG

CCTTCGGGTTGTAAAGCACTTTCAGTAGGGAGGAAAGGTAGCAGCTTAATACGCTGTTGC

TGTGACGTTACCTACAGAAGAAGGACCGGCTAACTCCGTG

>1313 len=160 tot-seq=16

TGGGGAATATTGCACAATGGGCGCAAGCCTGATGCAGCCATGCCGCGTGTGTGAAGAAGG

CCTTAGGGTTGTAAAGCACTTTCAGCGAGGAGGAAGGCGTTGTAGTTAATAGCTGCAACG

ATTGACGTTACTCGCAGAAGAAGCACCGGCTAACTCCGTG

>1314 len=160 tot-seq=16

TGGGGAATTTTGGACAATGGGCGCAAGCCTGATCCAGCCATGCCGCGTGTATGAAGAAGG

CCTTCGGGTTGTAAAGTACTTTTGTCAGGGAAGAAAAGCCTGAGGATAACACCCTCAGGT

CATGACGGTACCTGAAGAATAAGCACCGGCTAACTACGTG

>1315 len=160 tot-seq=16

TGGGGAATTTTGGACAATGGGGGAAACCCTGATCCAGCCATTCCGCGTGAGTGAAGAAGG

CCTTCGGGTTGTAAAGCTCTTTTGTCCGGAACGAAACGGTGGACGTGAATATCGTCCATT

ACTGACGGTACCGGGAGAATAAGCACCGGCTAACTACGTG

>1316 len=160 tot-seq=16

TGGGGAATATTGCACAATGGGCGCAAGCCTGATGCAGCCATGCCGCGTGTATGAAGAAGG

CCTTCGGGTTGTAAAGTACTTTCAGTGGTGAGGAAGGGAGTGTGGTTAATAGCCATATTC

TTTGACGTTAGCCACAGAAGAAGCACCGGCTAACTCCGTG

>1317 len=161 tot-seq=16

TGGGGAATCTTCCGCAATGGGCGCAAGCCTGACGGAGCAACGCCGCGTGAGTGAAGAAGG

CCTTCGGGTCGTAAAGCTCTGTTGTCGGGGACGAATGTTGTTGCTTAGAATATAGGTGAC

AAATGACGGTACCTGACGAGGAAGCCACGGCTAACTACGTG

>1318 len=161 tot-seq=16

TGGGGAATATTGCACAATGGGCGCAAGCCTGATGCAGCAACGCCGCGTGAACGATGAAGG

TCTTCGGATCGTAAAGCTCTGTTGTTAGAGAAGAACGTTGGTAGGAGTGGAAAATCTACC

AAGTGACGGTAACTAACCAGAAAGGGACGGCTAACTACGTG

>1319 len=214 tot-seq=16

AGAAGGCAGGGTGAAGAGGGAGGCAGGGATAGGACTGGCTGCAAGCCCAGGATCACGGGG

GCCACCAGAGGCTGGAAGAGGCAAGAAAGGGCCCTTTCTGGAGCCTTTGGAAGGAGGCCC

AACCCAGTGGACACCTTGGCTCTGGACTTTCAGCCTGCAGAAGTGCGAGGGGATGCACTT

CTGTTGTTTTTGTCCGCCCAGAGCGTGGTTAGTT

>1320 len=217 tot-seq=16

TAGGGAATCTTCCACAATGGACGCAAGTCTGATGGAGCAACGCCGCGTGAGTGAAGAAGG

TTTTCGGATCGTAAAGCTCTGTTGTAAGTCAAGAACGAGTGTGAGAGTGGAAAGTTCACA

CTGTGACGGTAGCTTACCAGAAAGGGACGGCTAACTACGTGAGTGGAAAGTTCACACTGT

GACGGTAGCTTACCAGAAAGGGACGGCTAACTACGTG

>1321 len=160 tot-seq=16

TAGGGAATCTTCGGCAATGGGCGAAAGCCTGACCGAGCAACGCCGCGTGAGTGAAGAAGG

TTTTCGGATTGTAAAACTCTGTTGTTAGGGAAGAACACGTACCGTTTGAATAAGGCGGTA

CCTTGACGGTACCTAACGAGAAAGCCCCGGCTAACTACGT

>1322 len=161 tot-seq=16

TAGGGAATCTTCGGCAATGGACGCAAGTCTGACCGAGCAACGCCGCGTGAGTGAAGAAGG

TTTTCGGATCGTAAAACTCTGTTGTTAGAGAAGAACAAGGATGAGAGTGGAAAGTTCATC

CCTTGACGGTATCTAACCAGAAAGCCACGGCTAACTACGTG

>1323 len=150 tot-seq=16

TGGGGGATATTGCACAATGGGCGAAAGCCTGATGCAGCGACGCCGCGTGGGGGATGAAGG

CCTTCGGGTTGTAAACTTCTTTTGCTCTGAACAAGGCATGCCAATGGGTGTGTTGAGTGT

AGGGGTTGATTAGCGCCGGCTAACTACGTG

>1324 len=155 tot-seq=16

TGAGGAATATTGGTCAATGGGCGAGAGCCTGAACCAGCCAAGTAGCGTGCAGGATGACGG

CCCTATGGGTTGTAAACTGCTTTTATAAGGGAATAAAGTGGGGCTCGTGAGCTCCTTTGC

ATGTACCTTATGAATAAGGACCGGCTAATTCCGTG

>1325 len=155 tot-seq=16

TGAGGAATATTGGTCAATGGTCGGGAGACTGAACCAGCCAAGCCGCGTGAGGGATGAAGG

CGCTAAGCGTCGTAAACCTCTTTTGCCGGGGAACAAGAGCGGGGACGTGTCCCCGTGTGA

GTGTACCCGGAGAAAAAGCATCGGCTAACTCCGTG

>1326 len=186 tot-seq=16

AAGGCTGGGCCAGGGGAGGAAGGGAGCTGGGGAAAGGTGTCTCTGGAGACTGAAGCAGAG

GAAGGAGAAGGGGGAACATTGGAGAGTTTCTGTAAGGGCATATCCTGTGTCTAAAGGGGG

TTGGAGAAAAGTCTGTGAGACCCAGGAGCCTGAATGTTCATGTCAGACCTCATCATATGC

TGAATC

>1327 len=155 tot-seq=16

TGAGGAATATTGGTCAATGGGCGAGAGCCTGAACCAGCCAAGTAGCGTGCAGGAAGACGG

CCCTATGGGTTGTAAACTGCTTTTATGTGGGGATAAAGTGCGTGACGTGTCCCTTATTGC

ATGTACCATACGAATAAGGACCGGCTAATTCCGTG

>1328 len=134 tot-seq=16

CTTGTGTAACAGCTTTTTTGGGGGTATCCCTCTGCCTCATCCAGTTCCTCAGGTCCCACA

CACCTACATCACTCTCCCCCAGAAGAGGATGCTCCTGCCAGCTTCTGCAGTATTTTCCAA

TCCTCCCAGTGATC

>1329 len=155 tot-seq=16

TGGGGAATATTGCACAATGGGGGAGACCCTGAACCAGCCAAGCCGCGTGAGGGAAGACGG

CCCTATGGGTTGTAAACCTCTTTTGTCGGAGAACAAAACCCGGGACGTGTCCCGGACTGC

GTGTATCCGAAGAAAAAGCATCGGCTAACTCCGTG

>1330 len=135 tot-seq=16

TGGGGAATATTGCACAATGGACGGAAGTCTGATGCAGCAACGCCGCGTGAACGATGAAGG

TCTTCGGATCGTAAAGTTCTGTTGCAGGGGAAGATAATGACGGTACCCTGTGAGGAAGCC

CCGGCTAACTACGTG

>1331 len=136 tot-seq=16

TGGGGAATATTGGGCAATGGGCGCAAGCCTGACCCAGCAACGCCGCGTGAGCGATGAAGG

TCTTCGGATTGTAAAGCTCTGTCGCAGGGGACGAAGTATGACGGTACCCTGTAAGAAAGC

CCCGGCTAACTACGTG

>1332 len=148 tot-seq=16

GAACACCGGTGGGCTGGGGGAAGGGGAAGGGACTTAAGTGTGGCAGTGGGAAATGAGTTT

TGGACAAGAACATGTGTTTCATTGAATGGCAAAAAAAATTAATCATCTTCCCTCTGCCCA

GTAACATGAGCTGGACGTCTTCTGGGAC

>1333 len=155 tot-seq=16

TGAGGAATATTGGTCAATGGGCGAGAGCCTGAACCAGCCAAGTCGCGTGAGGGATGACTG

CCCTATGGGTTGTAAACCTCTTTTGTATGGGGGGAATAAGCTTTACGAGTAGAGTGATGA

CAGTACCATACGAATAAGCATCGGCTAACTCCGTG

>1334 len=161 tot-seq=16

TAGGGAATCTTCGGCAATGGACGGAAGTCTGACCGAGCAACGCCGCGTGAGTGAAGAAGG

TTTTCGGATCGTAAAGCTCTGTTGTTAGAGAAGAATGATGGTGGGAGTGGAAAATCCACC

ATGTGACGGTAACTAACCAGAAAGGGACGGCTAACTACGTG

>1335 len=135 tot-seq=15

TGGGGAATATTGCACAATGGGGGAAACCCTGATGCAGCGACGCCGCGTGAGCGAAGAAGT

ATTTCGGTATGTAAAGCTCTATCAGCAGGGAAGAAATTGACGGTACCTGAGTAAGAAGCT

CCGGCTAAATACGTG

>1336 len=135 tot-seq=15

TGGGGAATTTTGCACAATGGGGGAAACCCTGATGCAGCGACGCCGCGTGTACGATGAAGA

TCTTCGGATTGTAAAGTACTGTCCTTGGTGAAGATAATGACTGTAACCAAGAAGCAAGCC

CTGGCTAAATACGTG

>1337 len=135 tot-seq=15

TGGGGAATTTTGGGCAATGGGGGAAACCCTGACCCAGCGACGCCGCGTGGAGGATGAAGG

CCTTCGGATTGTAAACTTCTTTGGCCAGGGACGATAATGACTGTACCTGGAGAACAAGCC

ACGGCTAACTACGTG

>1338 len=136 tot-seq=15

TCGGGAATTTTGCCCAATGGACGAAAGTCTGAGGCAGCAACTCCGCGTGAGGGATGAAGG

CTTTCGGGTCGTAAACCTCTTTTCCCAGGGAAGATCCAAGACGGTACCTGGGGAATAAGC

CACGGCTAACTACGTG

>1339 len=136 tot-seq=15

TGGGGAATATTGCACAATGGGGGAAACCCTGATGCAGCAACGCCGCGTGAGTGAGGAAGT

ATTTCGGTATGTAAAGCTCTATCAGCAGGGAATAGAAAAGAAGGTACCTGAAAAAGAAGC

CCCGGCTAACTACGTG

>1340 len=137 tot-seq=15

TGGGGAATATTGGGCAATGGAGGAAACTCTTACCCAGCAATGCCGCGTGAGTGAAGAAGG

TCTTCGGATTGTAAAGCTCTTTGATTGGGGATGAAAAAATGACGGTACCCAAAGAACAAG

CCCCGGCTAACTACGTG

>1341 len=155 tot-seq=15

TGAGGAATATTGGTCAATGGGCGAGAGCCTGAACCAGCCAAGTAGCGTGAAGGAAGACTG

CCCTATGGGTTGTAAACTTCTTTTATAAGGGAATAAAGAGCGCCACGTGTGGTGTGTTGT

ATGTACCTTATGAATAAGCATCGGCTAATTCCGTG

>1342 len=155 tot-seq=15

TAGGGAATATTGGGCAATGGATGCAAGTCTGACCCAGCCATGCCGCGTGCCGGATGAAGG

CCCTCAGGGTTGTAAACGGCTTTTATTCGGGAAGAAGAGCAGGGATGCGTCCTTGTGTGA

CGGTACCGAATGAATAAGCACCGGCTAACTCCGTG

>1343 len=160 tot-seq=15

TAGGGAATCTTCGGCAATGGGGGGAACCCTGACCGAGCAACGCCGCGTGGGTGAAGAAGG

TCTTCGGATCGTAAAACCCTGTTGTTAGAGAAGAAAGTGCGTGAGAGTAACTGTTCACGT

TTCGACGGTATCTAACCAGAAAGCCACGGCTAACTACGTG

>1344 len=160 tot-seq=15

TGGGGAATTTTGGACAATGGGCGCAAGCCTGATCCAGCCATGCCGCGTGCAGGATGAAGG

CCTTCGGGTTGTAAACTGCTTTTGTACGGAACGAAACGGCCTTTTCTAATAAAGAGGGCT

AATGACGGTACCGTAAGAATAAGCACCGGTTAACTACGTG

>1345 len=161 tot-seq=15

TAGGGAATCTTCCACAATGGGCGCAAGCCTGATGGAGCAACACCGCGTGAGTGAAGAAGG

CCTTCGGGTCGTAAAATTCTGTTGTCAGAGAAGAACGTGCGTGAGAGTAACTGTTCACGT

ATTGACGGTATCTGACCAGAAAGCCACGGCTAACTACGTGG

>1346 len=161 tot-seq=15

TAGGGAATTTTCGGCAATGGGGGGAACCCTGACCGAGCAATGCCGCGTGAAGGATGAAGG

TTTTCGGATTGTAAACTTCTGTTGTATAGGAAGAAAGGTAAGTGGAGTGGAAAGCCACTT

AATTGACGGTACTATACTAGAAAGCCACGGCTAACTACGTG

>1347 len=175 tot-seq=15

TGGGCGAAAACAGAGCGTGGTCCGAGCAATCCGTATCTGCGGTAGATGTCGGTATAGTTC

GCCGCTTCGGGATATAGCGATAATGCCCATTCGATTTCATCGTCCGCTTCGTCGAGATGT

GTATGCATATACACACCCCTGTCGATGTTTCTTTGATAAAGGTCTCCTGCGAGTT

>1348 len=180 tot-seq=15

CCCCTAGACATCCACAAGCACAATTTTTCAGTTAAAAGCAAAAGATATGGGGCTGAGAGA

GGGCTCAGTGGCTAAGAGCACTGTCTGCTATTCCAGAGGTCTTGAGTTCAATTCGGGGCT

GAGAGAGGGCTCAGTGGCTAAGAGCACTGTCTGCTATTCCAGAGGTCTTGAGTTCAATTC

>1349 len=183 tot-seq=15

TGGGGAATTTTGCACAATGGGGGGAACCTTGATGCAGCGACGCCGCGTGAGGGATGGAGG

CCTTCGGGTTGTGAACCTCTTTCGCTCGTGTGCAAGCCGCTATTTGGGTAGTGGTGAGTG

TAGCGGGTAAAGAAGCGCCGGCTAACTACGTGAGCGGGTAAAGAAGCGCCGGCTAACTAC

GTG

>1350 len=199 tot-seq=15

GGCCTGAAATTACTGGTTTTCCCTCCTGCCTGGGAAAGCAGCCTATAGCCTTGTACATGG

TGTGTGATCAACAACTGGGGTAGCACTGCTGGTGTGCAGAGGCGCTCCCCCCAAGGGCGG

GGCACGTCATCACTTACCATGATGCAGTTAGGCTTGTTTATGGACCACAGGTTGACTCGG

CAGTCATCCCCACCAGTGG

>1351 len=201 tot-seq=15

TAGGGAATATTGCACAATGGAGGAAACTCTGATGCAGCCATGCCGCGTGTGTGAAGAAGG

TTTTCGGATCGTAAAGCTCTGTTGTTGGTGAAGAAGGATAGAGGCAGTAACTGGCCTTTA

TTTGACGGTAATCAACCAGAAAGTCACGGCTAACTACGTGCCAGCAGCCGCTGACAGATC

GGAAGAGCGTCGTGTTAGGGA

>1352 len=203 tot-seq=15

CCCGGCTCCCCAGAGCTGAAAGGAAAGGCGGGAGGAGGAGAGGAGGCGCCTCTCAGCTGA

GGGGATCCCAAGAGCACCGGTTGGCTGATTGCACGTAATTAGGAAGAAGAAAAAAAAAGC

TCTTGAAAATTAGCTTCCCAAATGCAGATATAATTGCAAGTGTCCTTGCAGAGTGATTAC

AGCGTCGGCCCAATAAATGTTTC

>1353 len=203 tot-seq=15

TGGGGAATTTTGGACAATGGGCGCAAGCCTGATCCAGCCATGCCGCGTGTCTGAAGAAGG

CCTTCGGGTTGTAAAGGACTTTTGTTAGGGAAGAAAAGGATAGTGTTAATACCATTATCT

GCTGACGGTACCTAAAGAATAAGCACCGGCTAACTACGTGCCAGCAGCCGCGGTAAATAG

ATCGGAAGAGCGTCGTGTAGGGA

>1354 len=205 tot-seq=15

CAGGAGGAACAGAAAGGTTATTTTCTTTACTGACTCTTTCAAGGACATCAGCAGAATGGT

TGGGAATGTCATCATGAGCAGCCTCAGCCTCAGAATTACCAGGAGAAGCTGGTCTTCCAG

GATCAGGTTCTAATGTATATGAGTCACAGGATTTAGGGAAGCAGGCACTTTCCCTTCAGT

CGAAGCAGGCACTTTCCCTTCAGTC

>1355 len=212 tot-seq=15

GAGGCATTGACCCCTCCAGTTTATTATGGGGATTGAATGAGCTGACACATCTGGGGTACT

TAAAACTGTGCCTGGCGTGTGAGTTGTGATTATTGTTAGTCTGTCGCCATAATGGTATCA

TAAATTATCAACCACCCTTTGCCTGGATCCTTGCTTCTGAGAGCTTATCTTTCGAAGTCT

TTGTCTTTTGGATCCAGTGTGTCCCAACTACA

>1356 len=217 tot-seq=15

CATTGTTCCTGAATCCTTTTTGATGGACAAACATTCAGATCCCCCTGCAGTTTTCTGGAA

TCCTATGTAAGGGACAAACACTCAGAACCCAGCAGCAGTGTTCTGGAATCGTATGTGAGG

GACAAACTCTCAGAACCTGCAGTTTTCTGGAATCCTATGTGAGGGACAAACACTCAGAAC

CCAGCAGCAGTGTTCTGGAATCGTATGTGAGGGACAA

>1357 len=234 tot-seq=15

CCTTCAGGGGGGGAAATCCTGGGTCTCCAGGTGGCCCAACCTTGCCTGGAGTCCCCATGA

AGCCAGGCTCACCTCTCATGCCTGACAAAGCCCAAAGGAAGCAAGTCAGGTCCCGGCATG

GTAGGGGTGGTAGGGGTGGAGGATAGGCAGGGGTGAGGTAGGGAAGAACATCAGCCACCG

GCCTCTCTTCACAGGACTGCAGGAAGGAGTCCTGGCCGTGGAGTGGGGAAGGGC

>1358 len=235 tot-seq=15

CTAGGGGGGTAAAGGGCACGCAGGCGGTTTTTAAGTGAGGTGTGAAAGCCCCGGGCTTA

ACCTGGGAATTGCATTTCAGACTGGGTAACTAGAGTACTTTAGGGAGGGGTAGAATTCCA

CGTGTAGCGGTGAAATGCGTAGAGATGTGGAGGAATACCGAAGGCGAAGGCAGCCCCTTG

GGAATGTACTGACGCTCATGTGCGAAAGCGTGGGGAGCAAACAGGATTAGAAACC

>1359 len=210 tot-seq=15

AGGGAATAGGTTAAAATGAGCAGCGGCAGGGAGGTCAGAAGGGGCTCGTGGGGTGGACCA

CATGAGCTTGATTAGGATGAAGTAAGGGAGACATGAAGAAAAGGAGGTGAGGTTGAGAGA

GCTGAATGTGGAGAGGTAATGGAGGACGATGGGGACTAGGCTGAGGGATTTAGTTCCCCA

GTCAGGGGAGCTATACCTGAATCAAACTGA

>1360 len=142 tot-seq=15

TGGGGAATATTGCACAATGGGCGCAAGCCTGATGCAGCGACGCCGCGTGGGGGATGAAGG

CCTTCGGGTTGTAAACTCCTTTCGCTATCGACGAAGCCTTCGGGTGACGGTAGGTAGATA

AGAAGCACCGGCTAACTACGTG

>1361 len=156 tot-seq=15

TGAGGAATATTGGTCAATGGACGAGAGTCTGAACCAGCCAAGTCGCGTGAAGGATGACTG

CCCTATGGGTTGTAAACTTCTTTTATTGGGGAATAAAAAAGAGGACGTGTCCTTTATTGT

ATGTACCTAAATGAATAAGCATCGGCTAACTCCGTG

>1362 len=154 tot-seq=15

TGAGGAATATTGGTCAATGGGCGAGAGCCTGAACCAGCCAAGTAGCGTGCAGGAAGACGG

CCCTATGGGTTGTAAACTGCTTTTATAGGGGAATAAAGTGGACTACGTGTAGTTTATTGT

ATGTACCCTATGAATAAGGACCGGCTAATTCCGT

>1363 len=160 tot-seq=15

CTAAGAATATTCCGCAATGGGGGGAACCCTGACGGAGCGACGCCGCGTGGGCGAGGAAGG

CCGGAAGGTTGTAAAGCCCTTTTGCGCGCGAGGAATAAGGGGAGGAGGGAATGCCTTCCC

GGTGACTGTAGCGCGCGAATAAGCGCCGGCTAATTACGTG

>1364 len=179 tot-seq=15

GGGACACAGAGAGCAGAGAGGAGTAAAACTGGGCACCAGCCCATCTGGGCTCAGCATGAA

GCCAGGAGACTCTCTCCAATGTGGGAAAGGGTGAGTAAGTGAAAGCCACCAGGGGGATTT

ACGGTCTCTACCTGTGCAAAACTGGGAATGGGAGAATCCCCCTGGCTAGAGCTTCTGGC

>1365 len=157 tot-seq=15

TGAGGAATATTGGTCAATGGACGCAAGTCTGAACCAGCCAAGTAGCGTGCAGGATGACGG

CCCTCCGGGTTGTAAACTGCTTTTAGTTGGGAATAAAAGGGGGCTCGTGAGCCCCGTATT

GTATGTACCATCAGAAAAAGGACCGGCTAATTCCGTG

>1366 len=215 tot-seq=15

TAGGGAATTTTCGTCAATGGGCGCAAGCCTGAACGAGCGATGCCGCGTGAGTGAAGAAGG

CCTTCGGGTCGTAAAGCTCTGTTGCGGGGGAAAAAAGGCAGCATCAGGAAATGGGTGCTG

ACTGATGGTGCCCCGCCAGAAAGTCACGGCTAACTACGTGCCAGCAGCCGCGGTAATTGG

AGATCGGAAGAGCGTCGTGTAGGGGAAGAGAGTGC

>1367 len=136 tot-seq=15

TAGGGAATTTTGCGCAATGGGGGAAACCCTGACGCAGCGACGCCGCGTGAGCGAAGAAGT

ATTTCGGTATGTAAAGCTCTATCAGCAGGGAAGAAGAATGACGGTACCTGACTAAGAAGC

ACCGGCTAAATACGTG

>1368 len=160 tot-seq=15

TAGGGAATCTTCGGCAATGGGGGGAACCCTGACCGAGCAACGCCGCGTGAGTGAAGAAGG

CCTTCGGGTTGTAAAGTACTTTCAGCGGGGAGGAAGGGAGTAAAGTTAATACCTTTGCTC

ATTGACGTTACCCGCAGAAGAAGCACCGGCTAACTCCGTG

>1369 len=229 tot-seq=15

AGAGCTGAGTGCAACGTCCCAAGCCCCTGGGGGTAGTGGCTCCCACTTCACCTGCACCAC

AGCCTCCAAGGTCCTCTGATGCTTATCCTGCGTGCAGGGCAGCAGACAGGGCATGGGGAA

CACAGCCACCAGGTCACACACCAGCAAGGGGACAGCCAAGGCTCAACCCTGGGGGGCTGC

AGGCTCTGCCAGCCTCACTCCAGGGCCACTGTACAGTGTTGGGGACAGA

>1370 len=161 tot-seq=15

TGGGGAATATTGCGCAATGGGGGAAACCCTGACGCAGCGACGCCGCGTGCGGGATGAAGG

CCTTCGGGTTGTAAAACTCTGTTATACGGGACGAAAAAGACGGATGCCAACAGTATCCGT

CCGTGACGGTACCGTAAGAGAAAGCCACGGCTAACTACGTG

>1371 len=155 tot-seq=15

TGAGGAATATTGGTCAATGGGCGAGAGCCTGAACCAGCCAAGTAGCGTGCAGGATGACGG

CCCTATGGGTTGTAAACTGCTTTTATATGGGAATAAAGTGAGGGACGTGTCCCTTATTGC

ATGTACCATATGAATAAGGACCGGCTAATTCCGTG

>1372 len=102 tot-seq=14

CATTATTTCCCCCTTGTTTTTGCCTCTCCTTATCCTCAGTAACGTCATCCTGTTCCCCTG

GGAACAACAGCAGGCCTTCATCTCCTGGCTCAGGACTCCACG

>1373 len=132 tot-seq=14

TCAAGAAAAACTTTCTGAGGATAAACAGCCTCTTTTCTGTATTTAACAAACAGAAAATAC

GAACCAAAATAAAGCAAAAGGAAACATCCTCCAACTATAAGCAACCCTACATTTGAAATC

AATTCACCACCA

>1374 len=135 tot-seq=14

TGGGGAATATTGCACAATGGGGGAAACCCTGATGCAGCGACGCCGCGTGAGTGAAGAAGC

GCCTCGGCGCGTAAAGCTCTGTCAGCAGGAACGACGATGACGGTACCTGAAGAAGAAGCC

CCGGCTAACTACGTG

>1375 len=135 tot-seq=14

TGGGGAATCTTCCGCAATGGACGAAAGTCTGACGGAGCAACGCCGCGTGAGCGAAGAAGG

CTTTCGAGTCGTAAAGCTCTGTCCTATGAGAAGATAATGACGGTATCATAGGAGGAAGCC

CTGGCTAAATACGTG

>1376 len=136 tot-seq=14

TGGGGAATATTGGGCAATGGAGGAAACTCTGACCCAGCAATGCCGCGTGAACGATGAAGG

TCTTTTGATTGTAAAGTTCTTTAGTTTGCTAGGAAATAAATGACCAGCAAAAGAATAAGC

CCCGGCTAACTACGTG

>1377 len=136 tot-seq=14

TGGGGAATATTGCACAATGGGCGCAAGCCTGATGCAGCAACGCCGCGTGGAGGAAGACGG

TTTTCGGATTGTAAACTCCTGTTCTTAGTGAAGAAAAATGACGGTAGCTAAGGAGCAAGC

CACGGCTAACTACGTG

>1378 len=136 tot-seq=14

TGGGGGATATTGCACAATGGGGGAAACCCTGATGCAGCGATGCCGCGTGGAGGAAGAAGG

TTTTCGGATTGTAAACTCCTGTCGTAAGGGAAGAGGAAGGACTGTACCTTACAAGAAAGC

TCCGGCTAACTACGTG

>1379 len=138 tot-seq=14

TGGGGAATATTGGGCAATGGGCGCAAGCCTGACCCAGCAACGCCGCGTGAAGGAAGAAGG

CTTTCGGGTTGTAAACTTCTTTTCTCAGGGACGAAGGAAGTGACGGTACCTGAGGAACAA

GCCACGGCTAACTACGTG

>1380 len=138 tot-seq=14

TGGGGAATATTGGGCAATGGGCGCAAGCCTGACCCAGCAACGCCGCGTGAAGGAAGACGG

TCTTCGGATTGTAAACTTCTTTAGACAGGGACGAAACAAATGACGGTACCTGTAGAATAA

GCCACGGCTAACTACGTG

>1381 len=138 tot-seq=14

TGGGGGATATTGCACAATGGGGGGAACCCTGATGCAGCGACGCCGCGTGAGTGAAGAAGT

ATCTCGGTATGTAAAGCTCTATCGGCAGGGAAGAAAAAAATGACAGTACCTGACTAAGAA

GCCCCGGCTAACTACGTG

>1382 len=138 tot-seq=14

TGGGGAATATTGCACAATGGGGGAAACCCTGATGCAGCAACGCCGCGTGAAGGAAGAAGG

CCTTTGGGTCGTAAACTTCTGTTCTGAGGGAAGAAGAAAGTGACGGTACCTCAGGAGAAA

GCCCCGGCTAACTACGTG

>1383 len=140 tot-seq=14

TGGGGAATATTGCACAATGGGCGAAAGCCTGATGCAGCGACGCCGCGTGAGGGATGACGG

CCTTCGGGTTGTAAACCTCTTTCAGCTCCGAAGAAGCGAAAGTGACGGTAGGAGCAGAAG

AAGCACCGGCTAACTACGTG

>1384 len=143 tot-seq=14

TGGGGAATATTGCACAATGGGCGCAAGCCTGATGCAGCGACGCCGCGTGGGGGATGACGG

CCTTCGGGTTGTAAACTCCTTTCAGCCATGACGAAGCCTTTGTGGTGACGGTAGTGGTAG

AAGAAGCACCGGCTAACTACGTG

>1385 len=154 tot-seq=14

TAGGGAATATTGGGCAATGGCCGAGAGGCTGACCCAGCCATGCCGCGTGCAGGAAGAAGG

CCTTCTGGGTTGTAAACTGCTTTTACCAGGGAAGAAAACGCGCCTGCGGGCGTAACTGAC

GGTACCTGGTGAATAAGCACCGGCTAACTCCGTG

>1386 len=155 tot-seq=14

TGAGGAATATTGGTCAATGGACGCAAGTCTGAACCAGCCATGCCGCGTGCAGGAAGACGG

CTCTATGAGTTGTAAACTGCTTTTGTACAAGGGTAAACCCTGGGACGCGTCCCAGGCTGA

AAGTACTGTACGAATAAGGATCGGCTAACTCCGTG

>1387 len=155 tot-seq=14

TAAGGAATATTGGTCAATGGAGGCAACTCTGAACCAGCCATGCCGCGTGCAGGAAGACAG

CCCTCTGGGTCGTAAACTGCTTTTATTCGGGAATAAACCTCATTACGTGTAATGAGCTGA

ATGTACCGAAGGAATAAGGATCGGCTAACTCCGTG

>1388 len=155 tot-seq=14

TGAGGAATATTGGTCAATGGGCGCAAGCCTGAACCAGCCATGCCGCGTGCAGGAAGACGG

CTCTATGAGTTGTAAACTGCTTTTGTACTAGGGTAATTTGCGGTACGTGTACCGTATTGA

AAGTATAGTACGAATAAGGATCGGCTAACTCCGTG

>1389 len=160 tot-seq=14

TGGGGAATATTGCACAATGGGGGAAACCCTGATGCAGCGACGCCGCGTGAGTGAAGAAGG

TTTTCGGATCGTAAAACTCTGTTGTAAGAGAAGAACAAATTGTAGAGTAACTGCTACAGT

CTTGACGGTATCTTACCAGAAAGCCACGGCTAACTACGTG

>1390 len=160 tot-seq=14

TAGGGAATCTTCCGCAATGGACGCAAGTCTGACGGAGCAACGCCGCGTGTGTGAAGAAGG

TTTTCGGATCGTAAAGCACTGTTATTAGCCAAGAACACCCCTAGTAGTAACTGGCTAGGG

ATTGACGGTAACTAATCAGAAAGCCACGGCTAACTACGTG

>1391 len=160 tot-seq=14

TTAGGAATATTCGTCAATGGGGGAAACCCTGAACGAGCAATGCCGCGTGAGTGATGAAGG

TCTTTGGATTGTAAAACTCTGTTGTATGGAAAAAACGGCTAGGTTAGGAAATGAACTTAG

TGTGATGGTACCATACCAGAAAGCCCCGGCTAACTACGTG

>1392 len=160 tot-seq=14

TGGGGAATTTTGGACAATGGGGGCAACCCTGATCCAGCCATTCCGCGTGAGTGAAGAAGG

CCTTCGGGTTGTAAAGCTCTTTCAGCTGGAACGAAACGGTCCGCTCTAACATAGCGGGCT

AATGACGGTACCAGCAGAAGAAGCACCGGCTAACTACGTG

>1393 len=160 tot-seq=14

TGGGGAATATTGGACAATGGGCGCAAGCCTGATCCAGCCATACCGCGTGGGTGAAGAAGG

TCTTCGGATTGTAAAGCACTTTAAGTTGGGAGGAAGGGCATTAACCTAATACGTTGGTGT

CTTGACGTTACCGACAGAATAAGCACCGGCTAACTCTGTG

>1394 len=173 tot-seq=14

GAGCAGAGATTGAGGAAATGGCTGACCAATGACTGGCCCAACTTGGGACTTATCCCAATT

AGAGAGAGACATCTCCTGACATTATTAATAAGACTCTGCTATGCTTGCAGACAGGAGCCT

AGCATAACTGTCTTCTACGAGGCTTCACATAACTGTCTTCTACGAGGCTTCAC

>1395 len=176 tot-seq=14

GAGGACCCCAGGCTTCTGGACCAAGATGACTTAGCTTTAAATCCTAGCAAGAAATCTTAC

TAGGTCCATGACTACGGTGGTGTTAAAATGTGTCCATGCATTCTTTAACACTTCCTTCAA

AAGTTAAAACTTAGTTCTCCTTCCTTTGGGTGTCTGCTGGATTTAGTGATCCACTT

>1396 len=183 tot-seq=14

GAAAATGAAGTGGACAATGTGGGGGTCCTTAGTTGTATTTTTGTTTAGTGTGCTAGTTTT

GTGCTGGTTTGCCTCCAGCCTGGAGGTGGTACTTTCAATAGAGGGGTCCTTAGTTGTATT

TTTGTTTAGTGTGCTCGTTTTGTGCTGGTTTGCCTCCAGCCTGGAGGTGGTACTTTCAAT

AGA

>1397 len=198 tot-seq=14

TGAGGAATATTGGTCAATGGGCGGAAGCCTGAACCAGCCATGCCGCGTGAAGGACTAAGG

CCCTACGGGTCGTAAACTTCTTTAGACGCAGAGCAATAAGGTCCACGTGTGGACCGATGA

GAGTATGCGTAGAATAAGCATCGGCTAACTCCGTGCCAGCAGCCGGGTAATACCAAGATC

GGAAGAGCGTCGTGTATG

>1398 len=199 tot-seq=14

TGAGGAATATTGGTCAATGGTCGAGAGACTGAACCAGCCAAGTCGCGTGAAGGAAGACGG

CTCTATGAGTTGTAAACTTCTTTTGTACAGGAGTAAAGTGAGACACGCGTGTTTTATTGC

AAGTACTGTACGAATAAGCATCGGCTAACTCCGTGGGGGGAGCCGGGTAATACCGAGATC

GGAAGAGCGTCGTGTAGGG

>1399 len=206 tot-seq=14

CACAAGCACAGAGTTGTGGGAAAGATGGAAGCAAGGTCAGGAGAGGAACCAAGAGAAACA

GCAACATTTCAGGTGAGGGTGGAAAAGGATCATCAAAAAGACAGAGTGAGAGAGCGTGGT

CAGAGAGGTGGCAGATGACCCCGGAGAGAGTGACATCATGAAGCCAAGGGAAGTAAGGAC

TGTGGTTGTCGCAGGAGAGATGGTGC

>1400 len=208 tot-seq=14

TGGGGGATATTGCACAATGGGCGAAAGCCTGATGCAGCGACGCCGCGTGAGGGATGAAGG

CTTTCGGGTTGTAAACCTCTTTCAGTACGGAAGAAGGCCATTGTTGAGATGGTGGACGGT

ACGTGCAGAAGAAGCGCCGGCTAACTACGTGCCAGCACCGCGGTAATAGTGAGATCGGAA

GAGCGTCGTGTAGGGACAGAGTGTGCCA

>1401 len=214 tot-seq=14

ATGCTGAAGAGAACTGAATCTGATGATCAGGGTCTCAGAATGGAGGGCAGACAAGCTCGA

GAGCCATGGCTAGAGGAGAGAGTATCTAGAGAGAAATAGAAACTGACAAGAGAATCAAAG

AGCCTGCTTCTCCTGCTAATGCATGGCTGCCCTTGGGTTTCTCCCTCTAGCCGGTGGTGT

TGGGAATGAGCCCTACCTAAGCCTTCATGCACAG

>1402 len=217 tot-seq=14

TGGGGAATATTGCGCAATGGGGGAAACCCTGACGCAGCGACGCCGCGTGAGTGAAGAAGG

ATTTCGGTTCGTAAAACTCTGTTGTTAGAGAAGAACAGCGCATAGAGTAACTGTTATGCG

TGTGACGGTATCTAACCAGAAAGCCACGGCTAACTACGTGGGGGGAGCCGCGGTATACGT

AGATCGGAAGAGCGTCGTGTAGGGAAAGAGTGGGGAA

>1403 len=228 tot-seq=14

GCATACGAGATTTGACGGTGACTGGAGTTAAGACGTGTGATATTAAGATATAATAAGGGA

GGAAGAAGTAGGGAATATTAAAAAATGGAAGAAAGTATGATGGAGAAAAGAAGAGTGAGT

GAAGAAGGGTTTAGGATAGTAAAGATATGTTGTTGGTGAAGAAGGACAGGGGTAGTAACT

GACCTTTGTTTGACGGTAATCAATTAGAAAGTCACGGCTAACTACGTG

>1404 len=230 tot-seq=14

TAAAGGGGACGCAGCCTATCTGTCAGGAGCGGCGCGCGAGTCGGCTCAGCCACTGCGCAT

GCGCGAGGTGAGAGCGGTTTCTCCCGTCACAGTGGTTCCCACGGTTGTCTTAGAAACCAG

TCCCCGAGGCTTGGCAGAGCAGGAGCCCTCTGTGGCAGTGCTTGGGTGTCGACGCTCTGC

GGCTCCGGCCTCACCTCTCCATGGGGTCGAAGGGAACGTCTCCGGATGTG

>1405 len=236 tot-seq=14

ACCATTTGGAGTTCAGACGTGTGCTCTTCCGATCTCTACGGGAGGCGGGGGGGATTAAAA

AATGAGGATCCAAATGGGAAAGGTGCCCACCCATTGATCGCAGCATAAGAGATTAAGGCT

TCAATGAGCAGCAAGAAGATTCTGTAACAGAAAATGGTTGCAGAGCATGAGCTTAGGCCC

TGAAGATATCTATAATCAGTGACAGCCAGGCATAGACAGACAGGGCACCATTTGCC

>1406 len=161 tot-seq=14

TGGGGAATCTTCCGCAATGGACGCAAGTCTGACGGAGCAACGCCGCGTGAGGGAGGAAGT

TTTTCGGAACGTAAACCTCTGTTGTTCATGACGAACGAGCTTTTTATCAACAACGAAGAG

CAGTGACGGTAGTGAACGAGGAAGCCACGGCTAACTACGTG

>1407 len=159 tot-seq=14

TGGGGAATATTGGACAATGGGGGGAACCCTGATCCAGCAATGCCGCGTGTGTGAAGAAGG

CCTTCGGGTTGTAAAGCACTTTCAGTAGGGAGGAAAGGTGCGTAGTTAATACCTGCGCAA

TTGACGTTACCTACAGAAGAAGCACCGGCTAACTCCGTG

>1408 len=155 tot-seq=14

TGAGGAATATTGGTCAATGGCCGAGAGGCTGAACCAGCCAAGTCGCGTGAGGGAAGAATG

TCCTAAGGATTGTAAACCTCTTTTGTCGGGGAGCAAAAAGGCTCTGCGGAGTCGTATTGA

GAGTACCCGAAGAAAAAGCATCGGCTAACTCCGTG

>1409 len=155 tot-seq=14

TGGGGAATATTGCGCAATGGGGGAAACCCTGACGCAGCGACGCCGCGTGCGGGATGACGG

CCCTATGGGTTGTAAACTGCTTTTATATGGGGATAAAGTGGGGAACGTGTTCCCTTTTGC

AGGTACCATATGAATAAGGACCGGCTAATTCCGTG

>1410 len=137 tot-seq=14

TGGGGGATATTGGACAATGGGGGAAACCCTGATCCAGCGACGCCGCGTGAGTGAAGAAGT

ATCTCGGTATGTAAAGCTCTGTCAGCAGGGAAGAATTTATGACGGTACCTGACCAAGAAG

CCCCGGCTAACTACGTG

>1411 len=160 tot-seq=14

TGGGGAATTTTTCGCAATGGGCGCAAGCCTGACGAAGCAACGCCGCGTGGAGGATGAAGG

TCTTCGGATTGTAAACTCCTGTCGACCGGGACGAATGCACTCGGCCTAATACGTCGGAAT

GTTGACGGTACCGGTGGAGGAAGCCACGGCTAACTCTGTG

>1412 len=198 tot-seq=14

TCTTTCTCTTTCTCTCTCTCTTTTTTCTTTTTTTTTAAGGTGGGGAAAGAGACAACCAAG

AGACAGGAAGCTGATCTGCAAGGATTCGGAGTTGTGCTAAAAGGACTTTGATTTTTTTTC

CCCCTTTACGAACGCTGGCAATTGACATCACTACAGACAGCCTGGTTAGAGAACAAACTG

CCTCATCCCAAGTGGACC

>1413 len=135 tot-seq=14

TGGGGGATATTGCACAATGGAGGAAACTCTGATGCAGCGACGCCGCGTGAGGGAAGACGG

TCTTCGGATTGTAAACCTCTGTCTTTGGGGAAGAAAATGACGGTACCCAAAGAGGAAGCT

CCGGCTAACTACGTG

>1414 len=155 tot-seq=14

TGAGGAATATTGGTCAATGGACGGAAGTCTGAACCAGCCAAGTAGCGTGCAGGAAGACGG

CCCTATGGGTTGTAAACTGCTTTTATGTGGGGATAAAGTGAGGGACGTGTCCCTTATTGC

AGGTACCACATGAATAAGGACCGGCTAATTCCGTG

>1415 len=128 tot-seq=14

CAGTTCAGCAGTAGCGGGGAGCACAGTGGTGGCAGTGATCATGAGGCTGGGGAACCAAGT

CAGGGGGAACCAACCAGTACCTTTTGTACTGATAGCAAGGAGGGAGGGGACCCCAGAAGA

CCCCATGT

>1416 len=217 tot-seq=14

GTTTGTGGGGGGTGGAACAATTGGAGATCACTTTTGAACAGGCTGATTTTTAGATCATTA

GACATCAAGGAGAAAGTTAGACATATGAGCCTAGAGCTCAAGAAAGAGACCTGGGCTGGA

AATGTAAATTTGGAAGTTGTCAGAGTAGAGATTATATTTAAAACCATGACGCTGAATGCA

ATCACGAAATGAGCTAAATAGAGACAGAAGAGAGGAA

>1417 len=155 tot-seq=14

TGGGGAATCTTGCACAATGGGCGAAAGCCTGATGCAGCGACGCCGCGTGCGGGATGACGG

CCCTATGGGTTGTAAACTGCTTTTGTTTGGGAATAATCGACATTACGTGTAATGTTTTGC

ATGTACCATTCGAATAAGGACCGGCTAATTCCGTG

>1418 len=136 tot-seq=14

TGGGGGATATTGCACAATGGGGGGAACCCTGATGCAGCGACGCCGCGTGGGCGAGGGAGC

GCCTCGGCGCGTAAAGCCCTTTCGGCGGGGGAGAACGATGACGGTACCCGACGAAGAAGC

ACCGGCTAAATACGTG

>1419 len=225 tot-seq=14

TGGTCACGGTGTAGTGGACAGAGGCTGGGAGTAAAGAGACCAGTCAGGGGGCTGCTGTAA

TATTTCCGGTGAAAGCTGCTGAGGGGTGAATGAATAGTGGCAGAGGTAGGAAAAAATAAT

GAAAACATTGGCATGGAGGTGACATTGGCAAGATGAGGGTGGGAACTAGCAAAGAGGTGA

GAAGGAAGGCCAGTGGGACCCCCGGGGAGACCAATGTTTCTAAAG

>1420 len=155 tot-seq=14

TGAGGAATATTGGTCAATGGGCGAGAGCCTGAACCAGCCAAGTAGCGTGCAGGAAGACGG

CCCTATGGGTTGTAAACTGCTTTTGTTTGGGAATAATCGACATTACGTGTAATGTTTTGC

ATGTACCATTCGAATAAGGACCGGCTAATTCCGTG

>1421 len=161 tot-seq=14

TGGGGAATATTGCGCAATGGGGGAAACCCTGACGCAGCAACGCCGCGTGAGTGAAGAAGG

TTTTCGGATCGTAAAACTCTGTTGGTAGAGAAGAACGTTGGTGAGAGTGGAAAGCTCATC

AAGTGACGGTAACTACCCAGAAAGGGACGGCTAACTACGTG

>1422 len=156 tot-seq=14

CAGTCTCTTCCCTGAAGCCTGGGTCAGGGAGAGGAAGCAGGGAGGAGGCTGTGAGCAAGG

GACGCCCAAGGTGTGGGAGGCAGGGGGAGGCTCTTTGTGTAAGGGTGCCCAAGTTCAAGG

ACAACTGTCAGCATGCAGGAGAGGGGGACAGGAGTG

>1423 len=153 tot-seq=14

TGGGGGATTTTGCACAATGGGCGCAAGCCTGATGCAGCGACGCCGCGTGAGGGATGAAGG

CCTTCGGGTTGTAAACCTCTTTCGCTGGGTTGAAAGGCCATGCTTTGGGTGTGGTTGATT

TGAACTGGTAAAGAAGTACCGGCTAACTACGTG

>1424 len=138 tot-seq=14

TGGGGAATATTGGGCAATGGGCGCAAGCCTGACCCAGCAACGCCGCGTGAAGGAAGAAGG

TTTTCGGATTGTAAACTTCTGTCCTCAGGGAAGAAACAAATGACGGTACCTGAGGAGGAA

GCCCCGGCTAACTACGTG

>1425 len=205 tot-seq=14

TGGGGAATATTGCGCAATGGGGGCAACCCTGACCGAGCAACGCCGCGTGAGTGAAGAAGG

TTTTCGGATCGTAAAGCTCTGTTGTAAGAGAAGAACGTGTGTGAGAGTGGAAAGTTCACA

CAGTGACGGTAACTTACCAGAAAGGGACGGCTAACTACGTGGGGGGAGCCGGGTAATGCG

GAGATCGGAAGAGCGTCGTGTTGGG

>1426 len=104 tot-seq=13

CTCTTGTGGGAGCTCTCTTGCACGCAGGAGTGGGCAGCCAGTGGGTCTGAGGGTGCCACA

GGTCAGGCTCTCAGGTCTGTGCCACTGGAGGGTGGGACTTCCTA

>1427 len=107 tot-seq=13

ATATCTTGGCTTGGCTGTCATACTGGAGATGGGTCCTGAGTGGGCTCAGGGGTCCGCCTC

TACCTCAGAGCTTGGCAGGCACTGAGGAAGAAGGGTCTGGGGGAAAT

>1428 len=110 tot-seq=13

CATCTGAGGGTTTTGACTATGAATTTGAAGTAGGAGAGCCACCTGGCAGGTCTGGAGCAT

CTGTGATGCCCATTTTCCTGCCCCAGCTTTGTAGGAGTGAGGGGAGGAGC

>1429 len=133 tot-seq=13

ACAGAGCAGATATACAATGTATATTTACTTGTGTGTGAGTTGGCAGTAAAGTACAACAAA

GGCTGCCCAGTGGCTAACAGTGCAAAACTAGAACCACTAAACCTGCATTTTACTGTAGTG

GGCCATGTCCCTT

>1430 len=135 tot-seq=13

TGGGGAATATTGGACAATGGGCGAAAGCCTGATCCAGCAATGCCGCGTGAGTGATGAAGG

CCTTAGGGTTGTAAAGCTCTTTTACCAGGGAAGATGATGACTGTACCTGGAGAATAAGCC

CCGGCTAACTCCGTG

>1431 len=135 tot-seq=13

CGACGAATATTCCGCAATGGGCGCAAGCCTGACGGAGCGACGCCGCGTGCAGGATGAAGC

GGCTCTGCCGTGTAAACTGCTGTCAGGGTACAGAAACATTGATCATACCCAAAGGAAGGG

CCTGCTAATCTCGTG

>1432 len=135 tot-seq=13

TGGGGAATATTGCACAATGGGGGAAACCCTGATGCAGCGACGCCGCGTGAACGAAGAAGT

ATTTCGGTATGTAAAGTTCTATCAGCAGGGAAGATAATGACGGTACCTGACTAAGAAGCA

CCGGCTAAATACGTG

>1433 len=135 tot-seq=13

TGGGGAATATTGCACAATGGGCGAAAGCCTGATGCAGCAACGCCGCGTGAAGGAAGAAGG

CCTTCGGGTCGTAAACTTCTGTCCTTGGGGAAGATAATGACGGTACCCTTGGAGGAAGCC

CCGGCTAACTACGTG

>1434 len=135 tot-seq=13

CGGGGAATATTGCACAATGTGGGAAACCCTGATGCAGCGACGCCGCGTGAGTGAAGAAGT

ATCTCGGTACGTAAAGCTCTATCAGCAGGGAAGAAAATGACGGTACCTGACTAAGAAGCC

CCGGCTAACTACGTG

>1435 len=137 tot-seq=13

TGGGGGATATTGCACAATGGGGGGAACCCTGATGCAGCGACGCCGCGTGGGTGAAGGAGT

ACTCCGGTATGTAAAGCCCTATCGGCAGGGAAGAAAAAAGGACGGTACCTGACTAAGAAG

CCCCGGCTAACTACGTG

>1436 len=138 tot-seq=13

TGGGGAATATTGGGCAATGGAGGCAACTCTGACCCAGCAACGCCGCGTGAATGAAGAAGG

TCTTCGGATTGTAAAGTTCTTTAGCGAGGGACGAAAAGAATGACGGTACCTCGAGAATAA

GCCACGACTAACTACGTG

>1437 len=138 tot-seq=13

TGGGGAATATTGGGCAATGGGGGAAACCCTGACCCAGCAACGCCGCGTGAAGGAAGAAGG

CCTTCGGGTTGTAAACTTCTTTTAGTAGGGACGAAGAATGTGACGGTACCTACAGAAAAA

GCAACGGCTAACTACGTG

>1438 len=140 tot-seq=13

TGGGGAATATTGCACAATGGGGGAAACCCTGATGCAGCAACGCCGCGTGCGGGATGACGG

CCTTCGGGTTGTAAACCGCTTTCAGCCATGACGAAGCTTTTGTGACGGTAGTGGCAGAAG

AAGCACCGGCTAACTACGTG

>1439 len=140 tot-seq=13

TGGGGAATATTGGACAATGGGCGAAAGCCTGATCCAGCAACGCCGCGTGAGGGATGACGG

CCTTCGGGTTGTAAACCTCTTTCAGCAGGGACGAAGCGCAAGTGACGGTACCTGCAGAAG

AAGCACCGGCCAACTACGTG

>1440 len=140 tot-seq=13

TGGGGAATATTGGACAATGGGCGAAAGCCTGATCCAGCAACGCCGCGTGAGGGATGACGG

CCTTCGGGTTGTAAACCTCTTTCACCTGTGACGAAGCGAAAGTGACGGTAGCAGGAGAAG

AAGCACCGGCCAACTACGTG

>1441 len=143 tot-seq=13

TAGGGAATCTTCGGCAATGGACGGAAGTCTGACCGAGCAACGCCGCGTGAGTGAAGAAGG

TTTTCGGGTTGTAAACCGCTTTTGATTGGGAGCAAGCCTTCGGGTGAGTGTACCTTTCGA

ATAAGCGCCGGCTAACTACGTGG

>1442 len=150 tot-seq=13

CTCTGCCAGAGCCAAGGGTGGAACAGCACAAGACGACATCAGCAAACCCTGGTGCACCTA

ACTGTGGGCTGCTATGGAGTCTGGGATGGACTGGAGTTCCTCCTGCTCCAGGCCTCGGGA

GACAAAATCCACAAAGAGAGACCCAGTGGC

>1443 len=155 tot-seq=13

TGAGGAATATTGGTCAATGGGCGAAAGCCTGAACCAGCCAAGTAGCGTGAAGGATGACTG

CCCTATGGGTTGTAAACTTCTTTTATATGGGAATAAAACCACTTACGTGTAAGTGTCTGT

ATGTACCATATGAATAAGCATCGGCTAACTCCGTG

>1444 len=155 tot-seq=13

TGAGGAATATTGGACAATGGGTGCAAGCCTGATCCAGCCATCCCGCGTGAAGGATTAAGG

TCCTATGGATTGTAAACTTCTTTTATACAGGGATAAACCTCTCCACGTGTGGAGAGCTGA

AGGTACTGTATGAATAAGCACCGGCTAACTCCGTG

>1445 len=155 tot-seq=13

TGAGGAATATTGGTCAATGGGCGAGAGCCTGAACCAGCCAAGTAGCGTGCAGGATGACGG

CCCTCCGGGTTGTAAACTGCTTTTAGTTGGGAATAAAAAAAGGGACTTGTCCCTTCTTGT

ATGTACCTTCAGAAAAAGGACCGGCTAATTCCGTG

>1446 len=156 tot-seq=13

TGGGGGATTTTGCACAATGGGCGCAAGCCTGATGCAGCGACGCCGCGTGAGGGATGGAGG

CCTTCGGGTTGTGAACCTCTGTCGCCGGTGATGTAGGCTCTGCTTTGTGGGTGGGGTTGA

CGGTAGCCGGGGTATGAAGTGCCGGCTAACTACGTG

>1447 len=159 tot-seq=13

TGGGGAATATTGGACAATGGGCGAAAGCCTGATCCAGCCATGCCGCGTGAGTGATGAAGG

CCCTCGGGTCGTAAAGCTCTGTGGAGAGGGAAGAACCCGTTCAGCATCAGATCCTGAACG

TTGACGGTACCTCTTTAGCAAGCACCGGCTAACTCCGTG

>1448 len=160 tot-seq=13

TAGGGAATCTTCGGCAATGGACGGAAGTCTGACCGAGCAACGCCGCGTGAGTGAAGAAGG

ATTTCGGTTCGTAAAGCTCTGTTGTTAGGGAAGAATGATTATGTAGTAACTATACATAGT

AGAGACGGTACCTAACCAGAAAGCCACGGCTAACTACGTG

>1449 len=160 tot-seq=13

CTAAGAATATTCCGCAATGGAGGAAACTCTGACGGAGCGACGCCGCGTGGATGATGAAGG

TCGGAAGATTGTAAAATCCTTTTAAGTATGATGAATAACCTGATCAGGAAATGGGACAGG

GATGACCGTAATACTTGAATAAGCAACGGCTAATTACGTG

>1450 len=160 tot-seq=13

TGGGGAATTTTGGACAATGGGCGCAAGCCTGATCCAGCAATGCCGCGTGAGTGAAGAAGG

CCTTCGGGTTGTAAAGCTCTTTTGTCAGGGAAGAAACGGCCTGGGTTAATACCCCGGGCT

AATGACGGTACCTGAAGAATAAGCACCGGCTAACTACGTG

>1451 len=160 tot-seq=13

TAGGGAATCTTCCGCAATGGGCGAAAGCCTGACGGAGCAACGCCGCGTGAGTGATGAAGG

TTTTCGGATCGTAAAGCTCTGTTGCCAAGGAAGAACGTCTTCTAGAGTAACTGCTAGGAG

AGTGACGGTACTTGAGAAGAAAGCCCCGGCTAACTACGTG

>1452 len=161 tot-seq=13

TGGGGAATATTGGACAATGGGCGAAAGCCTGATCCAGCCATGCCGCGTGTGTGAAGAAGG

CCTTTTGGTTGTAAAGCACTTTAAGCGAGGAGGAGGCTACCGAGATTAATACTCTTGGAT

AGTGGACGTTACTCGCAGAATAAGCACCGGCTAACTCTGTG

>1453 len=161 tot-seq=13

TGGGGAATATTGGACAATGGGGGGAACCCTGATCCAGCCATGCCGCGTGTGTGAAGAAGG

CCTTATGGTTGTAAAGCACTTTAAGCGAGGAGGAGGGTACTGGTATTAATACTACCAGCT

ACTGGACGTTACTCGCAGAATAAGCACCGGCTAACTCTGTG

>1454 len=164 tot-seq=13

AACTAAAAAGAAGAGAAGAAAGGATTTGAACAGGGGTCAGATCATTGGTGAAGGGCATTA

TGGTTTCTGTGGCCTGGTCTGAATGTCTCTCTTATTAAAAATGGAGCAGTACAGATCATT

GCCCAAAAAAGCAAGGAAGAACAGAAGGAGGAGGACAACATGAC

>1455 len=170 tot-seq=13

GGCTGTGAGAGTCTGCTGGCGGCCCAGGGAGTCAGGTAAGGTCAGGAAGCGGTAGATGAT

GTTTTTGAGGTACTCCAGATTGGCTCCCTCCCTGCTCTGGTCCCTGATGTTCTTTTCGAT

GTGGCTCTGCAGGGCTGCAACCTCCTCACGATGCCGTTCGCCCTCCTCGT

>1456 len=172 tot-seq=13

GGGGTGCCCCCCAGGAGTGTGGTGGGCCTGGTGTTGGCCCTGTCTGCAGGAAGTGCAGCA

TGGCCCAGAGGCTCCCAAGCATGGCCTTTCATGGGGACCCTTCATGGGGATCGTGGTTCT

GCGGTTCGAGAGGAAGGGGTGAGGAAGGGAGTAAGGATACATCGTGGTGTGG

>1457 len=183 tot-seq=13

TGGGGAATATTGGACAATGGGCGCAAGCCTGATCCAGCCATACCGCGTGGGTGAAGAAGG

CCTTCGGGTTGTAAAGCCCTTTTGTTGGGAAAGAAATCCTATCGGTTAATAACCGGTGGG

GATGACGGTACCCAAAGAATAAGCACCGGCTAACTTCGTGAATAAGCACCGGCTAACTTC

GTG

>1458 len=186 tot-seq=13

TGGGGAATATTGCGCAATGGGGGGAACCCTGACGCAGCAACGCCGCGTGATTGAAGAAGG

CCTTCGGATTGTAAAGATCTTTAATCGGGGACGAAACATGACGGTACCCGAGGAATAAGC

TCCGGCTAACTACGTGCCAGCACCGCGGTAATGAGTAGATCGGAAGAGCGTCGTGTAGGG

AAAGAG

>1459 len=191 tot-seq=13

CAGGGTTTGAGAGAAATGACTAATTCTGAAAGATGCTCTGCTAAGGGTAAAGTGGTACAA

AATAGAATTACATGGTACAGAAAAGCCTTTCATGAAAGAAAGAGTCTATTGATTTGACAA

GCTTCACTGTTGTTTTATTTTACGAAATTGCCACAGCCACTCCAACTGTCAACAACTACC

ACCCTGAGCAG

>1460 len=193 tot-seq=13

TGAGGAATATTGGTCAATGGGCGAGAGCCTGAACCAGCCAAGTCGCGTGAAGGATGACTG

CCCTATGGGTTGTAAACTTCTTTTATAGGGGAATAAAAAGGAGAACGTGTTCTCTATTGC

ATGTACCCTATGAATAAGCATCGGCTAACTCCGTGTGCATGTACCCTATGAATAAGCATC

GGCTAACTCCGTG

>1461 len=196 tot-seq=13

TGGGGAATATTGGGCAATGGGCGAAAGCCTGACCCAGCAACGCCGCGTGAAGGAAGAAGG

CCTTCGGGTTGTAAACTTCTTTTACCAGGGACGAAGGACGTGACGGTACCTGGAGAAAAA

GCAACGGCTAACTACGTGTTTACCAGGGACGAAGGACGTGACGGTACCTGGAGAAAAAGC

AACGGCTAACTACGTG

>1462 len=197 tot-seq=13

GGCTTGGACAGAGCCCACGGTCCTCTCCACACCCACGAGTGCTTTCAGTCCATCTCCCCA

AACACACGACCCCTTTAGCAGCACTTTTTCCCTCTGTGGGCAGCCGGGATGGAGCCAATG

GCTCCGAACTTCCACTTGACACTCAACTGGGTGGCAACAGGCAAGAGGCTGTGTGCTTTG

GGAGGAAGAGTTTAAAC

>1463 len=199 tot-seq=13

TGAGGAATATTGGACAATGGGTGGAAGCCTGATCCAGCCATCCCGCGTGCAGGACGACTG

CCCTATGGGTTGTAAACTGCTTTTATATAGGGATAAACCTACTCTCGTGAGAGTAGCTGA

AGGTACTATATGAATAAGCACCGGCTAACTCCGTGAGTAGCTGAAGGTACTATATGAATA

AGCACCGGCTAACTCCGTG

>1464 len=204 tot-seq=13

AAAGGAAGAAGCCGGGATGACAGCGGCGGGGAGCGGGCGACACAGCAATGCCGAGGATTT

CTAGCCACCGCCAGAAGTGAGGAAGATTGAGGAAGAACGAGGAAGGCTTCCCCCGGAGCT

CTCAGAGGGCTCTCCCCGAGGTCCACGGAGGAACGGGCAAGTCCCTGCCCAGCCCGGCCT

GGCCAAGGATACGTCACGATGCCC

>1465 len=222 tot-seq=13

ATGGGCCCAAGTCACAGACAGAAGCCACAGCAGAAAATGGGAAGCATTCCTTTATTTTTC

CAGGCTTTTTTTCTTATTTTATTTTAAAGAGAAAAAATGTGACAAAATGGTGAGGATGCT

GGCAGCGCCAGCGTTCCCGGCCTGCACTCTCTCCTGGCCTCCCCTCGGACGTCTTCTGCA

GCCCAAATCTGAACAGCAGTGAAACCGTAAACATGCCCCCCA

>1466 len=209 tot-seq=13

TGCAATCCGGCTTTGTTGGGTCCGCATTTTTGCTTTAGTCTGGGGATAGGAATCAAATGT

TACCCAGAGATGTTTGTGTTTTGTTTGGGAGTTTTATTCCCTAACTCATTCCCCAAAGCA

CGTGTAACTGCTTATACATATAATCGTGGTACAACAAGGTATATACAGAGAACCCACTTG

GAAATTCAGGCAAAGCTGCATGCACGCTA

>1467 len=143 tot-seq=13

TGGGGAATATTGCACAATGGGCGAAAGCCTGATGCAGCGACGCCGCGTGCGGGATGAAGG

CCTTCGGGTTGTAAACCGCTTTTGATTGGGAGCAAGCCTTTTGGGTGAGTGTACCTTTCG

AATAAGCGCCGGCTAACTACGTG

>1468 len=157 tot-seq=13

TGGGGAATATTGCACAATGGGCGCAAGCCTGATGCAGCGACGCCGCGTGGGGGATGACGG

CCTTCGGGTTGTAAACTCCTTTCGTTAGGGACGAAGCCACACCTTTTTTTGGGTGTGGTG

ACGGTACCTTTGTTAAGAAGCACCGGCTAACTACGTG

>1469 len=156 tot-seq=13

TGAGGAATATTGGTCAATGGGAGAGATCCTGAACCAGCCAAGCCGCGTGAGGGAAGACGG

CCCTATGGGTTGTAAACCTCTTTTGCCGGGGAGCAAGCTGTGTCACGTGTGACACAGCTG

AGAGTACCCGGAGAAAAAGCATCGGCTAACTCCGTG

>1470 len=160 tot-seq=13

TAGGGAATCTTCCGCAATGGGCGAAAGCCTGACGGAGCAACGCCGCGTGAGTGATGAAGG

TCTTCGGATCGTAAAGCTCTGTTGTTGGTGAAGAAGGATAGAGGTAGTAACTGGCCTTTA

TTTGACGGTAATCAACCAGAAAGTCACGGCTAACTACGTG

>1471 len=136 tot-seq=13

TGAGGAATATTGGGCAATGGAGGAAACTCTGACCCAGCAACGCCGCGTGAGCGATGAAGG

TCTTCGGATTGTAAAGCTCTGTCGCAGGGGACGAAGTATGACGGTACCCTGTAAGAAAGC

CCCGGCAAACTACGTG

>1472 len=145 tot-seq=13

TGGGGAATATTGCACAATGGACGCAAGTCTGATGCAGCGACGCCGCGTGGGGGATGAAGG

CTTTCGGGTTGTAAACTCCTTTCAGTACAGAACAAGGCCTTTTTGGTTGAGGGTATGTGC

AGAAGAAGCGCCGGCTAACTACGTG

>1473 len=229 tot-seq=13

AGGTGGGGGGCCCCAGTCAGGGCTTGGGCAACGAGGAGCCCCGACCCGCATCACAGATGC

ACCAAGCCTGCTCGAGGGCAGTGGGAGAAGTGCGGCCCTCAGGATGCCACGTGCTCCAGG

CCCGAAACCCTCTGGCTGGAGGGGCCCTCTGGAACTGCAGGGAGGACTCTGTAGCCAGGG

TGGGGCTGGGGGCCGTGGGTGACCCTGGCTCCTTCCTTATTCTCTGAGC

>1474 len=160 tot-seq=13

TGGGGAATATTGCACAATGGGGGAAAGCCTGATGGAGCGACACAGCGTGCACGATGAAGG

TCTTCGGATTGTAAAGTGCTGTTATAAGGGAAGAACATTTGCAATAGGAAATGATTGCAG

ACTGACGGTACCTTGTCAGAAAGCGATGGCTAACTATGTG

>1475 len=161 tot-seq=13

TGGGGAATCTTCCGCAATGGACGAAAGTCTGACGGAGCAACGCCGCGTGAGTGATGACGG

TCTTCGGATCGTAAAGCTCTGTTGTTAAGGAAGAACGAGTGTGAGAATGGAAAGTTCATG

CTGTGACGGTACTTAACCAGAAAGGGACGGCTAACTACGTG

>1476 len=161 tot-seq=13

TGGGGAATATTGCGCAATGGGGGAAACCCTGACGCAGCGACGCCGCGTGCGGGATGAAGG

CCTTCGGGTTGTAAAGCTCTGTTAATCGGGACGAATGGTTTGTGTGCAAATAGTGCATAG

ACATGACGGTACCGGAATAGAAAGCCACGGCTAACTACGTG

>1477 len=110 tot-seq=12

CATCCTGATACTAAAATCCAGCAGAGAACAATGGTAAAAGAAAACATCAAGCCTGTATCC

TTGATGAACCTTGATGCAAAAATCTTCAATAAAATACTAGCAAACTGAAT

>1478 len=125 tot-seq=12

GCAGGACAGTGCCTCACCTTTATCCTGTCAAGCCGTCGCTTTATGGCTCGACTGTTTTTA

TCTAGCTCCTCTAACATCTTATAGGGGTTGAAGGAAAGCGGTTTCTCGTGGTCCACAAAG

TTTTC

>1479 len=131 tot-seq=12

TTGGAAACAGTATCACCTTTGGTTCATCACACACTGAACTTAACTTACTCATTGGTCCAT

GACGGGCCCTTTAAAAGTCTTTATTATTCCTGTTGAACATTTTAAAATAATATCATGGAC

ACCCATGAACC

>1480 len=135 tot-seq=12

TGGGGAATATTGCACAATGGGGGAAACCCTGATGCAGCGACGCCGCGTGAAGGAAGAAGT

ATTTCGGTATGTAAACTTCTATCAGCAGGGAAGAAGATGACGGTACCTGAGTAAGAAGCA

CCGGCTAAATACGTG

>1481 len=135 tot-seq=12

TGGGGGATATTGCACAATGGAGGAAACTCTGATGCAGCAATGCCGCGTGAGGGAAGACGG

TCTTCGGATTGTAAACCTCTGTCCTTGGTGAAGATGATGACGGTAGCCAAGGAGGAAGCT

CCGGCTAACTACGTG

>1482 len=135 tot-seq=12

GGGAATATTGGACAATGGGCGCAAGCCTGATCCAGCCATGCCGCGTGAGTGATGAAGGCC

TTAGGGTTGTAAAGCTCTTTCGCCCGCGAGGATAATGACGGTAGCGGGATAAGAAGCCCC

GGCTAACTTCGTGTG

>1483 len=135 tot-seq=12

TAGGGAATATTGGACAATGGGGGCAACCCTGATCCAGCAATGCCGCGTGAGTGATGAAGG

CCTTAGGGTTGTAAAGCTCTTTTACCCGAGATGATAATGACAGTATCGGGAGAATAAGCT

CCGGCTAACTCCGTG

>1484 len=135 tot-seq=12

TGGGGAATATTGCACAATGGGGGAAACCCTGATGCAGCAACGCCGCGTGAAGGATGAAGG

CCTTTGGGTCGTAAACTTCTGTCCTTGAGGAAGATAATGACGGTACTCTTGGAGGAAGCC

CCGGCTAACTACGTG

>1485 len=135 tot-seq=12

TGGGGAATATTGCACAATGGAGGAAACTCTGATGCAGCGACGCCGCGTGAACGATGAAGG

CCTTCGGGTCGTAAAGTTCTGTCCTAGGGGACGATAATGACGGTACCCTAGGAGGAAGCC

CCGGCTAACTACGTG

>1486 len=135 tot-seq=12

TCTCCTGGTACTCACTCCCTCATGCTACCTTAGCCTAGATCAACCCCCTCACCTTCCTCT

GTCAGGAGTCAGAGGGAGAGGCCCAACCTTCCCATGGCAGCTGCCATACTGGCCCCCTTG

ACCCTGCCTCCTTGA

>1487 len=135 tot-seq=12

TGGGGAATTTTGCGCAATGGGGGAAACCCTGACGCAGCAACGCCGCGTGCGGGACGAAGG

CCCTCGGGTCGTAAACCGCTTTCAGCAGGGAAGACCACGACGGTACCTGCAGAAGAAGCT

CCGGCTAACTACGTG

>1488 len=135 tot-seq=12

TGGGGAATATTGCACAATGGGGGAAACCCTGATGCAGCGACGCCGCGTGGACGATGAAGC

ATTTCGGTGCGTAAAGTCCTGTCAGCAGGGAAGAAAATGACAGTACCTGACCAAGAAGCC

CCGGCTAACTACGTG

>1489 len=137 tot-seq=12

GCAGGTCACTGAGTTCAAGGCCAGCCTGGTGTACAGATGGGGTTCCAGGACAGCCAAGCT

TAGGCAGTGAAGGAAACCACTGAAAACAGAAAGCTGGTGAAGATGGATTTGAATGAAGAG

GCACATTCCAGGCAGGC

>1490 len=137 tot-seq=12

TGGGGAATATTGGGCAATGGGCGCAAGCCTGACCCAGCGACGCCGCGTGAGGGAAGACGG

TCTTCGGATTGTAAACCTCTTTCAGCAGGGAAGACAAAAGGACGGTACCTGCAGAAGAAG

CCACGGCTAACTACGTG

>1491 len=140 tot-seq=12

TGGGGAATATTGCGCAATGGGGGAAACCCTGACGCAGCGACGCCGCGTGCGGGATGACGG

CCTTCGGGTTGTAAACCGCTTTCGCCTGTGACGAAGCGTGAGTGACGGTAATGGGTAAAG

AAGCACCGGCTAACTACGTG

>1492 len=140 tot-seq=12

TAGGGAATCTTCCACAATGGACGCAAGTCTGATGGAGCAACGCCGCGTGAGGGATGGAGG

CCTTCGGGTTGTAAACCTCTTTTATCGGGGAGCAAGCGAGAGTGAGTTTACCCGTTGAAT

AAGCACCGGCTAACTACGTG

>1493 len=140 tot-seq=12

TGGGGAATATTGCACAATGGGCGGAAGCCTGATGCAGCAACGCCGCGTGAGGGATGACGG

CCTTCGGGTTGTAAACCTCTTTTAGCAGGGAAGAAGCGAGAGTGACGGTACCTGCAGAAA

AAGCGCCGGCTAACTACGTG

>1494 len=141 tot-seq=12

GTTATGGAAAGAGCTGGTTCTTGTTCTCAGCCTGATGGGTCCCTCAGGGGCGGCGCATGT

GTAGCAGCTTTTCAGGAGTATGGGGGGTGACGGGAGCCAGGGGCCACGTGGTGCAGATGG

GAGAGAGGCCAGCAGATGGGT

>1495 len=143 tot-seq=12

TGAGGAATATTGGTCAATGGACGAGAGTCTGAACCAGCCAAGTAGCGTGCGGGATGAAGG

CCTTCGGGTTGTAAACCGCTTTTGATTGGGAGCAAGCCTTCGGGTGAGTGTACCTTTCGA

ATAAGCGCCGGCTAACTACGTGG

>1496 len=143 tot-seq=12

TAGGGAATCTTCCACAATGGGCGCAAGCCTGATGGAGCAACACCGCGTGCGGGATGAAGG

CCTTCGGGTTGTAAACCGCTTTTGATTGGGAGCAAGCCTTTTGGGTGAGTGTACCTTTCG

AATAAGCGCCGGCTAACTACGTG

>1497 len=145 tot-seq=12

TTAAATACATGGAGCAGGAACCAGCCTGCGGTGTCACAGCAGCCGCCTCAGCACCGGCCA

ACGAGCTCAACCAGGTGCACGTGGAACCAAAGACAAGCCCACTGACCACCAGAGCATGCC

GGCCAGGACAAGGGAAGTGGTGTGA

>1498 len=148 tot-seq=12

GAAAGTTGGCCTATTGTGATTGTCACCATGCCCTCAGGGCCCAAACAGCTGGGAAAAAGG

AGGCAGCAGGCAAGAGACCTGGAGGAGGCCCGCCAGGTGTCAGCCAGCAAACCTCAAACA

GAGCAGCAGCCTAAGCACTCAGAAGCAG

>1499 len=151 tot-seq=12

CCAGCACAGGGTAGAAGGGCAGTGGCAGTCCAGCTCCCTGTCAGGTGTGTCTGCCGTGTA

AAATAATAATATGCCCCCACCCATGGCTGTGTGGCCTGCACACTCCCACATGCATGCCCA

CACACAGACTGAAGGAATTGTACCCCCCTTCC

>1500 len=153 tot-seq=12

AAGCTGGGAGAGGCCACAGAGCAGACTCATCGTCGTCTTCAGCGGCCACAGGAGGGGCCC

ACACAGCTCATACCTTGACCCTGGATTTCCAGCCTCCCTGGTGTCACAGACTACACTCCT

GGTGCTCACACTATTCAGTCTATGCTACCTGTT

>1501 len=154 tot-seq=12

TGAGGAATATTGGTCAATGGACGAGAGTCTGAACCAGCCAAGTCGCGTGAAGGAAGACGG

ATCTATGGTTTGTAAACTTCTTTAGTGCGGGAACAAAGCGGCGTCGTGACGCCTGATGAG

TGTACCGCAAGAATAAGCATCGGCTAACTCCGTG

>1502 len=155 tot-seq=12

TGAGGAATATTGGTCAATGGTCGCAAGACTGAACCAGCCATGCCGCGTGCAGGAAGACGG

TCCTATGGATTGTAAACTGCTTTTATACAGGAAGAAACCCTCCCACGTGTGGGAGCTTGA

CGGTACTGTAGGAATAAGGATCGGCTAACTCCGTG

>1503 len=155 tot-seq=12

TGGGGAATCTTGCACAATGGGCGAAAGCCTGATGCAGCGACGCCGCGTGCGGGATGACGG

CCCTATGGGTTGTAAACTGCTTTTATATGGGAATAAAGTGAGGGACGTGTCCCTTATTGC

ATGTACCATACGAATAAGGACCGGCTAATTCCGTG

>1504 len=155 tot-seq=12

TAAGGAATATTGGTCAATGGACGCAAGTCTGAACCAGCCATGCCGCGTGAAGGATTAAGG

TCCTCTGGATTGTAAACTTCTTTTATTTGGGACGAAAAAGGGACTTTTTAGTTCAACTGA

CGGTACCAGATGAATAAGCACCGGCTAACTCCGTG

>1505 len=155 tot-seq=12

TGAGGAATATTGGTCAATGGGCGGGAGCCTGAACCAGCCAAGTAGCGTGAAGGATGACTG

CCCTACGGGTTGTAAACTTCTTTTGTTTGGGAATAAAAGACGCTACGTGTAGCGTTCTGC

ATGTACCTTACGAATAAGCATCGGCTAATTCCGTG

>1506 len=155 tot-seq=12

TAAGGAATATTGGTCAATGGGCGGAAGCCTGAACCAGCCATGCCGCGTGCAGGATGACTG

CCCTATGGGTTGTAAACTGCTTTTGTCCAGGAATAAACCTAGATACGTGTATCTAGCTGA

ATGTACTGGAAGAATAAGGATCGGCTAACTCCGTG

>1507 len=159 tot-seq=12

TAGGGAATCTTCCGCAATGGACGCAAGTCTGACGGAGCAACGCCGCGTGTGTGAAGAAGG

TTTTCGGACCGTAAAGCACTGTTGTTAGAGAAGAACACCCTGTAAGTAACTACTACAGGA

TTGACGGTATCTAACCAGAAAGCCACGGCTAACTACGTG

>1508 len=160 tot-seq=12

TGGGGAATATTGCGCAATGGGGGCAACCCTGACGCAGCCATGCCGCGTGAATGAAGAAGG

CCTTCGGGTTGTAAAGTTCTTTCGGTATTGAGGAAGGTTGATGTGTTAATAGTACATCAA

ATTGACGTTAAATACAGAAGAAGCACCGGCTAACTCCGTG

>1509 len=160 tot-seq=12

TGGGGAATATTGCGCAATGGGGGAAACCCTGACGCAGCGACGCCGCGTGCGGGATGAAGG

CCTTCGGATCGTAAAACTCTGTTGTTAGAGAAGAACACGAGTGAGAGTAACTGTTCATTC

GATGACGGTATCTAACCAGCAAGTCACGGCTAACTACGTG

>1510 len=160 tot-seq=12

TAGGGAATCTTCCACAATGGGCGCAAGCCTGATGGAGCAACACCGCGTGAGTGAAGAAGG

GTTTCGGCTCGTAAAGCTCTGTTGTTGGAGAAGAACGTGCGTGAGAGTAACTGGCCTTTA

TTTGACGGTAATCAACCAGAAAGTCACGGCTAACTACGTG

>1511 len=160 tot-seq=12

TGGGGAATTTTGGACAATGGGGGCAACCCTGATCCAGCCATGCCGCGTGAGTGAAGAAGG

CCTTCGGGTTGTAAAGCTCTTTCGGACGGAACGAAATGGGCGGGACTAATACTCCTGTCC

GATGACGGTACCGTAAGAAGAAGCACCGGCTAACTACGTG

>1512 len=160 tot-seq=12

TGGGGAATATTGCACAATGGGCGAAAGTCTGACGGAGCAACGCCGCGTGAGTGAAGAAGG

ATTTCGGTTCGTAAAACTCTGTTGTTAGGGAAGAAAAAATGTATAGTAACTATATACAAA

AGAGACGGTACCTAACCAGAAAGCCACGGCTAACTACGTG

>1513 len=160 tot-seq=12

TGGGGAATATTGGACAATGGGCGAAAGCCTGATCCAGCCATGCCGCGTGGGTGAAGAAGG

CCTTCGGGTTGTAAAGCCCTTTTGTTGGGAAAGAAAACTTTCCGGCTAATACCCGGAGAG

AATGACGGTACCCAAAGAATAAGCACCGGCTAACTTCGTG

>1514 len=160 tot-seq=12

TAGGGAATTTTCGGCAATGGGCGAAAGCCTGACCGAGCAACGCCGCGTGAGTGAAGAAGG

CCTTCGGGTTGTAAAGCTCTGTTGTGAAGGAAGAACGGCTCATACAGGGAATGGTATGGG

AGTGACGGTACTTTACCAGAAAGCCACGGCTAACTACGTG

>1515 len=161 tot-seq=12

TGGGGAATATTGGACAATGGAGGAAACTCTGATCCAGCAATTCTGTGTGTGTGAAGAAGG

TTTTCGGATCGTAAAGCTCTGTTGTAAGAGAAGAACGAGTGTGAGAGTGGAAAGTTCACA

CTGTGACGGTATCTTACCAGAAAGGGACGGCTAACTACGTG

>1516 len=161 tot-seq=12

TGGGGAATCTTCCGCAATGGGCGAAAGCCTGACGGAGCAACGCCGCGTGAACGATGAAGG

TCTTAGGATCGTAAAGTTCTGTTGTTAGGGACGAAGGGTAAGAATCATAATAAGGTTTTT

ATTTGACGGTACCTAACGAGGAAGCCACGGCTAACTACGTG

>1517 len=175 tot-seq=12

TGGGGAATATTGCGCAATGGGGGGAACCCTGACGCAGCGACGCCGTGTAAAGGAAGAAGG

TCTTCGGATCGTAAACTTATATCGATTGTGAAAAGTAAGGATGGTAGCAAAGTAAGAAGC

CCCGGCTAACTACGTGCCAGCAGCCGCGTAATCAGGAGATCGGAAGAGCGTCGTG

>1518 len=178 tot-seq=12

TGAGGAATATTGGTCAATGGACGCAAGTCTGAACCAGCCATGCCGCGTGCAGGAAGACGG

CTCTACGAGTTGTAAACTGCTTTTGTACTAGGGTAAACCTATCTACGTGTAGATAGCTGA

AAGTATAGTACGAATAAGGATCGGCTAACTCCGTGAATAAGGATCGGCTAACTCCGTG

>1519 len=179 tot-seq=12

TGTCTACCTGGAGGGGGCTGTGCGTATGATCGATGAGAACCAGGCCTCCTACACGACTGT

GATCCCCGAATCCCTGATCTCTGAATACGGGTGCGCGGACTTTACCGGCAGTGAGGACCG

GGCCGTGCTGATTCGGGATCTGGAGATGGTGAATCATGACGTCTATTATACGGTCGAAA

>1520 len=203 tot-seq=12

TGGGGAATATTGGACAATGGGCGCAAGCCTGATCCAGCCATGCCGCGTGTGTGAAGAAGG

CCTTCGGGTTGTAAAGCACTTTCAGCGAGGAGGAAGGGAGTAGTGTTAATAGTACTATTT

CTTGACGTTACTCGCAGAAGAAGCACCGGCTAACTCCGTGCCAGCAGCGCGGTAATGTTC

AGATCGGAAGAGCGTCGTGTTGG

>1521 len=203 tot-seq=12

TGAGGAATATTGGTCAATGGACGCAAGTCTGACGGAGCAACGCCGCGTGAGTGAAGAAGG

TTTTCGGATCGTAAAACTCTGTTGTAAGAGAAGAACAAATTGTAGAGTAACTGCTACAGT

CTTGACGGTATCTTACCAGAAAGCCACGGCTAACTACGTGCCAGCAGCCGCGGTATCCGA

AGATCGGAAGAGCGTCGTGTATG

>1522 len=204 tot-seq=12

TGGGGAATATTGGACAATGGGGGGAACCCTGATCCAGCCATGCCGCGTGTGTGAAGAAGG

CCTTTTGGTTGTAAAGCACTTTAAGCAGTGAAGAAGACCATATGGTTAATACCCATATGC

GATGACATTAGCTGCAGAATAAGCACCGGCTAACTCTGTGCCAGCAGCGCGGTAATGCTC

AGATCGGAAGAGCGTCGTGTATGG

>1523 len=208 tot-seq=12

AGGTTTTCGCTCTCACTGCCCAAGATGATCGTCTATACAGGAGCAACGTTTGCGTCTCAG

ATGGGGCCTGAGGAAGAGCTGACTCTGGACTTAAGGGCAAGGGTTAAATTTAAGGCAGGG

AGATTCTGTACCCGGGCAGCATCTCCACCTGCTAATGCCTTTCTCCTGGGCATGGAGAAT

GTAGCATTCAAAAAGTTAGCCAATCCAA

>1524 len=208 tot-seq=12

ACGATTGCCCCATCGAATCGGCCTAGGACCTTGAGTGTGTTGCCCAGGTTCCCACTAGCC

TTGGCCTCCCCCATGCGGTCACCAATGGTCCTGGAGGGCAGGAAGGAGTGAGGAGTCAGT

TGGGTCTAGTTGGGCCCTGAGATGAGCAAGAAGCCTCAGAGGGGAAAGACAGACAGTGAA

AAGTTAGCTCTACACATGCATTGGTGAT

>1525 len=210 tot-seq=12

CCTGTCAGGAGTGGAGGGTGAGTCGGCTCAGCCAATGCGCATGCGCGAGGCCCCAGCGGT

TTCTCGGGTCACAGTGGTTCCCACGGTTGTCTTAGAAACCAGTCCCCGAGGCTTTGCAGA

GCAGGAGCCCTCCGTGGCAGTGCTTGGGTATCGAGGCTCTGAGGCTCCGGTCTCACCTCT

CCACGGGCTCGAAGGGAAACTCTCCTGATG

>1526 len=212 tot-seq=12

ATGACCATTTACACTGCCAGGGTGTGTGAACCAGGGAACATCCCCCACATTACTCACTAA

GTAATGAGGAGGGGACTTAGATATGTTCCACAATTCAGATGGGCCTGCCAGTTGGCCCCT

GAGCTTCATTCTGTGTGATCCATACACAGCAGAGACACTCAGGAGGCAATGCAAATGAAT

CAACAGACAGCACTGAGGTCAGCACTGAGGTC

>1527 len=215 tot-seq=12

TAGTCCGGAAGGTAGAGGATGTTAATGGACCCATTGCGAGGACAGTGTTTTAGTAACAGA

AGAGGAGTATTTTGTTGTTCTTTAAATGACAAATCGTCTGCAATTTCTTTCTTCCTTTAG

TAGGTCAATGACGGGAAAAGATAGCATACATGTTTCCATGATGAATTTTTTTGAACTTGG

GCTCCTGCCTTTGTTTTTTGTGCAGGAAGCCACCT

>1528 len=216 tot-seq=12

TCTTGATAGAAAAGTTCTCTTCTTCCTTGACAAGTCCTGTCTCCAGCTCCTTGGGTCTCA

GGCATGGCTTTTCTTTGCAGGATAACCATCCTTAATCTCAGCTGCTCCTGATTGGCCAAG

GAATCCTGGGCTGATAGTGTTACCCAGCAGCCCCCATCCAGCTGCTCCTGATTGGCCAAG

GAATCCTGAGCTGATAGTGTTACCCCCCAGCCCCCA

>1529 len=223 tot-seq=12

TAGGGAATCTTCGGCAATGGACGAAAGTCTGACCGAGCAACGCCGCGTGAGTGAAGAAGG

TTTTCGGATCGTAAAACTCTGTTGTTAGAGAAGAATAAGTAGGAGAGTAACTGCTCTTAC

CTTGACGGTATCTAACCAGAAAGCCACGGCTAACTACGTGAGTAGGAGAGTAACTGCTCT

TACCTTGACGGTATCTAACCAGAAAGCCACGGCTAACTACGTG

>1530 len=230 tot-seq=12

CAATGACCCTCTTCACCCTCCAGCCCCGGAGCATGATGATGAGGCTGATGGCGTCCCAGG
GGCTCCTGGGTCCATTGGCCACAGTGGATGCCACCATCGGAGCCAAAGATAGGATGATCA
CAGCCCCGTCAAACACCTGTGAGAGGAGGCCCATTTCAGTGCCCTGGGCCCTGCCGTGTG
TCCGGGACCAGGGGTCAGTCAGGCAAAGCAGCTGGTGCTGCCCAACTTTT
>1531 len=138 tot-seq=12
TGGGGAATTTTGGACAATGGGCGCAAGCCTGATCCAGCAACGATGCGTGGAGGATGAAGG
TTTTCGGATTGTAAACTCCTTTTAAGGGGGACGAAACAAATGACGGTACCCCTTGAATAA
GCCACGGCTAACTACGTG
>1532 len=157 tot-seq=12
TAGGGAATCTTCCACAATGGACGAAAGTCTGATGGAGCAACGCCGCGTGAGTGAAGAAGG
TTTTCGGATCGTAAAGCTCTGTTGGTGAAGAAGGATAGAGGTAGTAACTGGCCTTTATTT
GACGGTAATCAACCAGAAAGTCACGGCTAACTACGTG
>1533 len=138 tot-seq=12
TAGGGAATCTTGCACAATGGGCGAAAGCCTGATGCAGCAACGCCGCGTGAAGGAAGAAGG
CCTTCGGGCTGTAAACTTCTGTTCTTGGGGAAGAAGTAAGTGACGGTACCCAAGGAGGAA
GCCCCGGCTAACTACGTG
>1534 len=137 tot-seq=12
TGGGGAATATTGCACAATGGGCGGAAGCCTGATGCAGCGACGCCGCGTGGGGGATGACGG
CCTTCGGGTTGTAAACTCCTTTCGCCAAAGACGAATTTTTGACGGTATTTGGAGAAGAAG
CACCGGCTAACTACGTG
>1535 len=161 tot-seq=12
TGGGGAATCTTCCGCAATGGACGAAAGTCTGACGGAGCAACGCCGCGTGAGTGAAGAAGG
TCTTCGGATTGTAAAGCTCTGTTGTAAGAGAAGAACGAGTGTGAGAGTGGAAAGTTCACA
CTGTGACGGTATCTTACCAGAAAGGGACGGCTAACTACGTG
>1536 len=161 tot-seq=12
TGGGGAATTGTTCGCAATGGGCGCAAGCCTGACGACGCAACGCCGCGTGGAGGACGAAGA
TCTTCGGGTCGTAAACTCCTTTCGATCGAGAAGAATGCCTATAGGGTGAATAGCCCTGTA
GGGTGACGGTATCGAGAGAAGAAGCCCCGGCTAACTCTGTG
>1537 len=160 tot-seq=12

TAGGGAATCTTCGGCAATGGGGGGAACCCTGACCGAGCAACGCCGCGTGAGTGAAGAAGG

TCTTCGGATTGTAAAGCACTTTAAGTTGGGAGGAAGGGCAGTTACCTAATACGTGATTGT

TTTGACGTTACCGACAGAATAAGCACCGGCTAACTCTGTG

>1538 len=135 tot-seq=12

TCGAGAATCATTCACAATGGGGGAAACCCTGATGCAGCGACGCCGCGTGAGTGAAGAAGT

ATTTCGGTATGTAAAGCTCTATCAGCAGGGAAGAAAATGACGGTACCTGACTAAGAAGCC

CCGGCTAACTACGTG

>1539 len=138 tot-seq=12

TGGGGAATCTTCCGCAATGGACGAAAGTCTGACGGAGCAACGCCGCGTGAGTGAAGAAGG

TTTTAGGACTGTAAAACACTTTTAGTAGGGAAGAAAAAAATGACGGTACCTACAGAAGAA

GCAACGGCTAAATACGTG

>1540 len=155 tot-seq=12

TGGGGAATATTGCGCAATGGGGGAAACCCTGAACCAGCCAAGTAGCGTGCAGGATGACGG

CCCTATGGGTTGTAAACTGCTTTTATATGGGGATAAAGTGGGGAACGTGTTCCCTTTTGC

AGGTACCATATGAATAAGGACCGGCTAATTCCGTG

>1541 len=161 tot-seq=12

TGGGGAATATTGCGCAATGGGGGAAACCCTGACGGAGCAACGCCGCGTGAGTGAAGACGG

TCTTCGGATTGTAAAGCTCTGTTATACGGGACGAACGGCAAGGTGGTAAATAGCCATCAT

GAGTGACGGTACCGTAAGAGAAAGCCACGGCTAACTACGTG

>1542 len=192 tot-seq=12

GGGGCGGGAGAGATGGCTCGGTGGCTGAGAGCACTGACTGCTCTTCTGAAGGTTCTGAGT

TCAATTTCGGGGGCCACATGGTGGCTCACAAACATCCGTAACAGGATCTGGCACCCTCTT

CTGGAGTGTCTGGGGACAGCTGCAGTGTACTTACGTATAGTAAATAAATAAATCTTTAAA

AAAAGAGTGGAG

>1543 len=102 tot-seq=11

AGACAGAAGGGGCCCCTTCAAGAGCAGCGTGGGGGACAGAGTCACCAGGGCAGTGAAGGA

GAGCGACACCCAGTGACACCCAGAGGCCTGCACGCTTGTCAT

>1544 len=105 tot-seq=11

GACCCAGCACTGACCTTAGAGAACTCACAGCCCACAGGGGACAGACCACCCCACTGAAGG

ATAAAACCTCAGTGGAGATGAGGCTGTGGATCTGCAGTCGGGGGG

>1545 len=123 tot-seq=11

AAACTCAGCCAGGTCCATGGTGAAGGTAAAAGGAGAAGTTAGGCTTGGCTGAAGCATAGG

CATCAAGCAACCAGCCTGGATGAAGCTGAGACAGCAGTGGGTCAGTTCCTAGAGCTGTGA

TGG

>1546 len=129 tot-seq=11

GCCCAGTGGTTCACCCCAGGCAGCTCACACTAGGAATGGGCAAGATGGGCAGGGCTTGGG

AGGAGCCATAAGGTGAGGGGAGCGTGGCACCTCACGGAGACAAGAGCAAGACCAGCAGAC

ACAGGCATG

>1547 len=135 tot-seq=11

TGGGGAATCTTAGACAATGGGCGCAAGCCTGATCTAGCCATGCCGCGTGAGTGATGAAGG

CCTTAGGGTCGTAAAGCTCTTTCGCTGGGGAAGATAATGACGGTACCCAGTAAAGAAACC

CCGGCTAACTCCGTG

>1548 len=135 tot-seq=11

TAGGGAATCTTCCACAATGGACGCAAGTCTGATGGAGCAACGCCGCGTGAACGAAGAAGG

TATTCGTATCGTAAAGTTCTGTCCTATGGGAAGATAATGACAGTACCATAGAAGAAAGCT

CCGGCTAAATACGTG

>1549 len=135 tot-seq=11

AGGGAATCTTCCACAATGGACGCAAGTCTGATGGAGCGACGCCGCGTGAGTGAAGAAGTA

TCTCGGTATGTAAAGCTCTATCAGCAGGGAAGAAAATGACGGTACCTGACTAAGAAGCCC

CGGCTAACTACGTGG

>1550 len=136 tot-seq=11

TGGGGAATATTGGGCAATGGAGGAAACTCTGACCCAGCAACGCCGCGTGAAGGATGAAGG

TTTTCGGATTGTAAACTTCTGTCGCGAGGGACGAAGAATGACGGTACCTCGTAAGAAAGC

CCCGGCAAACTACGTG

>1551 len=138 tot-seq=11

TGGGGAATATTGCACAATGGAGGAAACTCTGATGCAGCAACGCCGCGTGAAGGAAGAAGG

TTTTCGGATCGTAAACTTCTATCAATAGGGACGAAACAAATGACGGTACCTAAATAAGAA

GCCCCGGCTAACTACGTG

>1552 len=138 tot-seq=11

TGGGGAATATTGCACAATGGGCGAAAGCCTGATGCAGCGACGCCGCGTGAAGTATGAAGG

CCTTCGGGTTGTAAACTTCTTTGATCAGGGAAGAAACAAATGACGGTACCTGAAAAACAA

GCCACGGCTAACTACGTG

>1553 len=138 tot-seq=11

TGGGGAATATTGCGCAATGGGGGCAACCCTGACGCAGCAACGCCGCGTGAAGGATGAAGG

TTTTCGGATTGTAAACTTCTTTTGTCGAGGACGAAGCAAGTGACGGTACTCGACGAATAA

GCCACGGCTAACTACGTG

>1554 len=140 tot-seq=11

TGGAGAATCTTGCGCAATGGGCGAAAGCCTGACGCAGCCACGCCGCGTGCGGGAAGAAGG

CCTTCGGGTCGTAAACCGCTTTCAGGAGGGAAGAAGCGAAAGTGACGGTACCTCCAGAAG

AAGCCCCGGCCAACTATGTG

>1555 len=141 tot-seq=11

TGAAGATGATCAGCATATCCCAACTCCCCAAGAACAACCATAACAACAACCATACATTTT

CTTAAGTCTGCACCTTATTGAAAGTAATTATTCCTTCTGCTTACCTTTACCATTGACTTC

TTGAAAGAGAAATCCATGCAC

>1556 len=144 tot-seq=11

TGGGGAATTTTCCACAATGGGTGAAAGCCTGACGGAGCAATGCCGCGTGGAGGTGGAAGG

CCTACGGGTCGTCAACTTCTTTTCTCGAAGAAGAAACAATGACGGTATCTGAGGAATAAG

CATCGGCTAACTCTGTGCCAGCAG

>1557 len=145 tot-seq=11

AATGGAACAAGAAAACTAAAACCCATTCTTTGCAGAAAACACCCAAGCATATTCAGACTG

ACTGGAGTAGAGAAGTTTGCCAAGAATGAGGGTCATTCCGGGCATCCATCAGCAACAGCC

CTGGCTTTCTAGGCTAGCATCAGAG

>1558 len=150 tot-seq=11

CAAGGCAAGGTGAGGGGGATCTGTTTAGCAAATCTCGTGGCAGTGTCTGGGCATTGGGAA

TGAACATCTGGCAGATCTGAGAATTGGGGCTACAAAAGATTGAGCCACTCCCTGAAACAG

TCATACAAGGGTTCCCTGAAGGCAGCCCCC

>1559 len=154 tot-seq=11

TAGGGAATATTGGACAATGGGCGGGAGCCTGATCCAGCCATGCCGCGTGCAGGAAGAAGG

CCTTCTGGGTTGTAAACTGCTTTTGCCGGGGGATAAAATGCCCCTGCGGGGGAAATTGAA

GGTACCCGGTGAATAAGCCACGGCTAACTACGTG

>1560 len=154 tot-seq=11

TAGGGAATATTGGGCAATGGACGGAAGTCTGACCCAGCCATGCCGCGTGCAGGAAGAAGG

CCTTCTGGGTTGTAAACTGCTTTTATATAGGAAGAAAACACCCATGCGTGGGAACCTGAC

GGTACTATATGAATAAGCACCGGCTAACTCCGTG

>1561 len=155 tot-seq=11

TGAGGAATATTGGTCAATGGACGCAAGTCTGAACCAGCCATGCCGCGTGCAGGATGACGG

TCCTATGGATTGTAAACTGCTTTTGTACGAGAAGAAACACTAGTTCGTGAACTAGCTTGA

CGGTATCGTAAGAATAAGGATCGGCTAACTCCGTG

>1562 len=155 tot-seq=11

TGAGGAATATTGGTCAATGGACGCAAGTCTGAACCAGCCAAGTAGCGTGCAGGAAGACGG

CCCTCTGGGTTGTAAACTGCTTTTAGTTGGGAATAAAGTGCACCACGAGTGGTGTTTTGT

ATGTACCATCAGAAAAAGGACCGGCTAATTCCGTG

>1563 len=155 tot-seq=11

TGAGGAATATTGGTCAATGGGCGGGAGCCTGAACCAGCCAAGTCGCGTGAAGGAAGAAGG

ATCTATGGTTCGTAAACTTCTTTTGCAGGGGAATAAAGTGCAGGACGTGTCCTGTTTTGT

ATGTACCCTGAGAATAAGGATCGGCTAACTCCGTG

>1564 len=155 tot-seq=11

TGAGGAATATTGGTCAATGGACGGAAGTCTGAACCAGCCAAGTAGCGTGCAGGATGACGG

CCTTATGGGTTGTAAACTGCTTTTGTATGGGGATAAAGTCAATCACGTGTGATTGTTTGC

AGGTACCATACGAATAAGGACCGGCTAATTCCGTG

>1565 len=155 tot-seq=11

TAAGGAATATTGGTCAATGGAGGGAACTCTGAACCAGCCATGCCGCGTGCAGGATGACTG

CCCTATGGGTTGTAAACTGCTTTTGTCAGGGAATAAACCTTTCCACGTGTGGGAAGCTGA

ATGTACCTGAAGAATAAGGATCGGCTAACTCCGTG

>1566 len=155 tot-seq=11

AGGGCTAGTTGAGACACTGTGTAACATGAAGACACAGGATGACAACATAGACAATGGAGA

GAGGCACCCTGAAGATGCCACCCGATCTCCCTAGTCAAGATCAGCTTAGAACCTGAAGGG

ACCTTCCTGTGCAAGGAGAAAGTAGCAGGAGGCCC

>1567 len=155 tot-seq=11

TAAGGAATATTGGTCAATGGACGCAAGTCTGAACCAGCCATGCCGCGTGGAGGATGACGG

CCCTCTGGGTTGTAAACTCCTTTTATCTGGGAAGAAATGCACTTTTTCTAAAGTGTTTGA

CGGTACCAGAGGAATAAGCACCGGCTAACTCCGTG

>1568 len=155 tot-seq=11

TGAGGAATATTGGTCAATGGACGTAAGTCTGAACCAGCCAAGTCGCGTGAGGGAAGACTG

CCCTATGGGTTGTAAACCTCTTTTATAAGGGAAGAATAAGTTCTACGTGTAGAATGATGC

CTGTACCTTATGAATAAGCATCGGCTAACTCCGTG

>1569 len=155 tot-seq=11

TGAGGAATATTGGTCAATGGCCGAAGGGCTGAACCAGCCAAGCCGCGTGAAGGAATAAGG

CCCTACGGGTCGTAAACTTCTTTAGTCAGGGAACAAAGGGTGCCACGTGTGGCGCTGTGA

GTGTACCTGAAGAATAAGCATCGGCTAACTCCGTG

>1570 len=157 tot-seq=11

CTCAAGGGACATGGGGTCTCAGCCCGGCAAGAAACTTCAAAACATCCTCGGAGGGCCTCC

CTGGTGGCGCAGTGGATCAGAGTCCACCTGTCAACGCAGGGGACACAGGTTCAATTCCCG

GTCTGGGAAGAGCCCACATGCTGCGGAACCACTAAGC

>1571 len=159 tot-seq=11

AACATCCTTTGAGAAACCAGCAGAAAGAGCCAGGAAAGTAGACAGCTACTTGGCATCTGA

GGAGGCATCGATGAGTGGAACGAAGGGCAAGCAACAAATGTAGCCAAGAGGCTAGCTGTG

TAGGTTGCTGCAATACAAGTGACACAGCAAGACCAGTGG

>1572 len=160 tot-seq=11

TAGGGAATCTTCCACAATGGGCGAAAGCCTGATGGAGCAACGCCGCGTGGGTGAAGAAGG

TCTTCGGATCGTAAAACCCTGTTGTTAGAGAAGAACGTATCTGAGAGTAACTGTTCAGGT

AGTGACGGTATCCAACCAGAAAGTCACGGCTAACTACGTG

>1573 len=160 tot-seq=11

TGGGGAATATTGGACAATGGGCGAAAGCCTGATCCAGCCATGCCGCGTGTGTGAAGAAGG

TCTTCGGATTGTAAAGCACTTTAAGTTGGGAGGAAGGGTAGTAACTTAATACGTTGCTAC

TTTGACGTTACCGACAGAATAAGCACCGGCTAACTTCGTG

>1574 len=160 tot-seq=11

TCGAGGATCTTTCGCAATGGGCGAAAGCCTGACGGAGCGACGCTGTGTGAGTGATGAAGG

CCTTCGGGTTGTAAAACTCTTTTGCCTGTGAACAAGAACGATTCGTGAATAACGAGTCAA

TTTGAGGGTAGCAGGAGAAAAAGCACCGGCTAACTACGTG

>1575 len=160 tot-seq=11

TGGGGAATTTTGGACAATGGGCGCAAGTCTGATCCAGCAATGCCGCGTGAGTGAAGAAGG

CCTTTGGGTTGTAAAGCTCTTTTGTCAGGGAAGAAACGGTTGGGGCTAATATCCCCGGCT

AATGACGGTACTTGAAGAATAAGCACCGGCTAACTACGTG

>1576 len=160 tot-seq=11

TAGGGAATCTTCCGCAATGGACGCAAGTCTGACGGAGCAACGCCGCGTGTGTGATGAAGG

ATTTCGGTTCGTAAAGCACTGTTGTTAAGGAAGAATAGTAGTAAGAGTAACTGTTTACTA

ATTGACGGTACTTAACCAGAAAGCCACGGCTAACTACGTG

>1577 len=160 tot-seq=11

TAGGGAATTTTCGGCAATGGGGGAAACCCTGACCGAGCAACGCCGCGTGAAGGAAGAAGT

AATTCGTTATGTAAACTTCTGTCATAGAGGAAGAACGGTGCGTGTAGGGAATGACATGCA

AGTGACGGCACTCTATAAGAAAGCCACGGCTAACTACGTG

>1578 len=160 tot-seq=11

TGGGGAATTTTGGACAATGGGCGCAAGCCTGATCCAGCCATGCCGCGTGAGTGAAGAAGG

CCTTCGGGTTGTAAAGCTCTTTCAGTCGGGAAGAAACGGGGCTCTCTAACATAGGGTCCT

AATGACGGTACCGACAGAAGAAGCACCGGCTAACTACGTG

>1579 len=160 tot-seq=11

TGGGGAATATTGGACAATGGGGGCAACCCTGATCCAGCCATGCCGCGTGTGTGAAGAAGG

CCTTTTGGTTGTAAAGCACTTTAAGCAGTGAAGAAGACTCCATGGTTAATACCCATGGAC

GATGACATTAGCTGCAGAATAAGCACCGGCTAACTCTGTG

>1580 len=160 tot-seq=11

TGGGGAATTTTGGACAATGGGCGCAAGCCTGATCCAGCCATGCCGCGTGTGGGAAGAAGG

CCTTCGGGTTGTAAACCACTTTTGTCAGGGAAGAAACGGCCTTTTCTAATACAGGGGGCT

AATGACGGTACCTGAAGAATAAGCACCGGCTAACTACGTG

>1581 len=161 tot-seq=11

TTAGGAATATTCGTCAATGGGGGCAACCCTGAACGAGCAATGCCGCGTGAACGATGACGG

TCCTCTGGATTGTAAAGTTCTGTTGTTTGGAAAGAATTGTACTGATAGGAAATGATCAGT

GCTTGACGGTACCATTCAAGAAAGCCACGGCTAACTACGTG

>1582 len=161 tot-seq=11

TTAGGAATATTCGTCAATGGGGGAAACCCTGAACGAGCAATGCCGCGTGAACGATGACGG

TCCTCTGGATTGTAAAGTTCTGTTGTTTGGGAAGAACGGCTCTAATAGGAAATGATTAGA

GATTGACTGTACCATTCAAGAAAGCCACGGCTAACTACGTG

>1583 len=161 tot-seq=11

TGGGGAATATTGCACAATGGGCGAAAGCCTGATGCAGCGACGCCGCGTGAGCGAAGAAGT

ATTTCGGTATGTAAAGCTCTGTTATACGGGACGAAAAAGACGGATGCCAACAGTATCCGT

CCGTGACGGTACCGTAAGAGAAAGCCACGGCTAACTACGTG

>1584 len=161 tot-seq=11

TTAGGGATATTCGTCAATGGGGGGAACCCTGAACGAGCAATGCCGCGTGAGTGAAGACGA

TTCTATGGATTGTAAAACTCTGTTGACAGGGACGAACGACCATTATAGGAAATGATAGTG

GAGTGACGGTACCTGTCGAGGAAGCTCCGGCTAACTACGTG

>1585 len=161 tot-seq=11

TGGGGAATATTGCGCAATGGGCGAAAGCCTGACGCAGCGACGCCGCGTGAGGGATGAAGG

TCTTCGGATCGTAAACCTCTGTCAGGAGGGAAGAAGTGGTTATATGCGAATAGTGTATGA

CTTTGACGGTACCTCCAAAGGAAGCACCGGCTAACTCCGTG

>1586 len=174 tot-seq=11

GGACTGTGGGCAAGGGAGTCCCCCTTGGAAGCAAAGGTATGCTTGCACTCACCATCTCAA

GTACACCTGTACAGGGAATAGAATTAAGTTACATTTAAGCCAATTGACCAAGAAATTTGA

GTTGTATTCAGTAGGCACATTCCTTCATGACCTCAGATTCTACCAGGCCACTGG

>1587 len=178 tot-seq=11

AAGTTACAAAACCAGCAAAGTGATTTTTTAAAAATGAAATCCTTATTTTCCTTCAAAAAG

TGCTATCATTTCTGAAAATTTTTAAATTTCTTATTTTTTACAGTAGCTGTATTCTATGCT

TTTGTTGTGAAGGCTTCACTCCCAGCTCGCTATGCGTCTATGCTGAGTGTGAATATAA

>1588 len=184 tot-seq=11

TGATAGTGTCAAGTGTTTTAACTCATTTGATCCCATAGTAACCTATAGGGAAGGTGACAT
TCTCATCCCCATTTTACAGATGAGGAAACAGAGACCCAGATAGTTTAAGTACCTTGCCCA
AAGTCCCAGAGCGAGGAAGAAGTGGACTGTTCAGCACAGAGACTCACACATAGTGAGCAC
TTCA
>1589 len=200 tot-seq=11
GGCGGGGGGGTGTCCTCTGGATCGAGCCTTGAGTGGAGGGGGCTGCTCGGCATCTGTGCT
GTGTGCCCAGACTCCCCTCAGTGCACCCCTCCTGTGCTCCCCATGGGAAGACACACCCCT
CCCTCCTTCCTTTATCAGGCCTCCTGAGGGTCAGGCCCCTGCAGGGTATCAGGCCTCCTG
AGGGTCAGGCCCCTGCAGGG
>1590 len=202 tot-seq=11
GCAGAATGTGTGGGCAGTGAGACTTCCCGTGGGGAGATAGAGGCCCTGGTATCCGAGACG
ATTCTGTCACTGATGTCAACCAGAAAGTGACATCACAGTGGTCACAACTTCTCTGTGCTC
TGTGGAAACTTATGAAACCTGGTGGTGTACTCAGGAGCCAGGATGAAAAAAAAAAGCGTG
GACCCAAGAAGTGTGGCCAGAG
>1591 len=204 tot-seq=11
TAGGGAATCTTCGGCAATGGGGGGAACCCTGACCGAGCAACGCCGCGTGAGTGAAGAAGG
TTTTCGGATCGTAAAGCTCTGTTGTTGGTGAAGAAGGATAGAGGTAGTAACTGGCCTTTA
TTTGACGGTAATCAACCAGAAAGTCACGGCTAACTACGTGCCAGCAGCCGCGTAATCCTC
AGATCGGAAGAGCGTCGTGTATAG
>1592 len=208 tot-seq=11
TGGGGAATATTGCACAATGGGCGCAAGCCTGATGCAGCAACGCCGCGTGAACGATGAAGG
TCTTCGGATCGTAAAGCTCTGTTGTTGGTGAAGAAGGACAGGGGTAGTAACTGACCTTTG
TTTGACGGTAATCAATTAGAAAGTCACGGCTAACTACGTGGACCTTTGTTTGACGGTAAT
CAATTAGAAAGTCACGGCTAACTACGTG
>1593 len=214 tot-seq=11
GGAGCTGATTGAGCTCATCAGCTGTGCAACAGCGAAGCCCAGAACGGAAGGGCGGGGAAG
CTTCCGGGGCTGTGCCTGTATTAAGGTCACAGGGTGTGAAGTGATCCCACAGGCTTTCCT
GCGGGGGTTGTGGATTGGCTGGTTTAAAGAAAACATGCACGAGGAGGAAACTGATTTACA
TGACTCTGGTGTTGAGCATTAGGTCTTATCGTGG

>1594 len=215 tot-seq=11

CCCCATGACCAAGGGAAGGGAAGAGGCATGAGCACCTGGGAAGGCCGGAGGGATGGAGGT

CCCTGACTGTTGCCCCTTGCTCAAATGCCACCTCTTTGTTCCTGCCATGGGAGCTGTGAT

GAGAGGAAGGTGCAGAGAGTGCTGGGCAGAGAGTCAGAGGAAGGCCTGACTTTGGGGTGC

ACGATACAATCACCCAACCTCTCAGAGGTCGCACA

>1595 len=216 tot-seq=11

TAGGGAATCTTCCACAATGGACGAAAGTCTGATGGAGCAACGCCGCGTGAGTGAAGAAGG

GTTTCGGCTCGTAAAACTCTGTTGTTAAAGAAGAACATATCTGAGAGTAACTGTTCAGGT

ATTGACGGTATTTAACCAGAAAGCCACGGCTAACTACGTGGAGTAACTGTTCAGGTATTG

ACGGTATTTAACCAGAAAGCCACGGCTAACTACGTG

>1596 len=217 tot-seq=11

ACTATAAGCCAGTCATAAAGTACTTGTCTGGTATGAAAACGACCCCGAGTTTTATCTCCC

CTGTGAAAATAAAAAGAAAAACTGCACAAGCTGATTACTTCTGGACAAGGAGCAAAAGAT

GTCTTTAGGAAAATGTTTTCTCTGCAGGCAAAACTGTCAGAGGTGTCTTACCTGGCACAG

CGTGTAAGCCGGAAGATCGGTCCTCTGCCTCAATAAG

>1597 len=161 tot-seq=11

TGGGGAATTTTTCGCAATGGGGGAAACCCTGACGAAGCAACGCCGCGTGGAGGATGAAGG

CCTTCGGGTCGTAAACTCCTGTCGACCGGGACGAATGTGGTCTGGGTCAATACCCCGGGC

CATTGACTGTACCGGTGGAGGAAGCCACGGCTAACTCTGTG

>1598 len=150 tot-seq=11

TGAGGAATATTGCGCAATGGGCGACAGCCTGACGCAGCGACGCCGCGTGGGGGATGAAGG

TCTTCGGATTGTAAACCCCTTTCGGGAGGGAAGATGGGACGGGCAACCGTTCGGACGGTA

CCTCCAGAAGCAGCCACGGCTAACTTCGTG

>1599 len=140 tot-seq=11

TGGGGAATATTGCACAATGGGCGCAAGCCTGATGCAGCGACGCCGCGTGAGGGATGACGG

CCTTCGGGTTGTAAACCTCTTTCAGCAGGGGAGAAGCGAAAGTGACGGTACCTGCAGAAG

AAGCGCCGGCTAACTACGTG

>1600 len=143 tot-seq=11

TAGGGAATCTTCCACAATGGACGCAAGTCTGACGCAGCGACGCCGCGTGCGGGATGAAGG

CCTTCGGGTTGTAAACCGCTTTTGATTGGGAGCAAGCCTTTTGGGTGAGTGTACCTTTCG

AATAAGCGCCGGCTAACTACGTG

>1601 len=160 tot-seq=11

TAGGGAATTTTCGTCAATGGGGGGAACCCTGAACGAGCAATGCCGCGTGTGTGAAGACGG

TCTTCGGATTGTAAAGCACTGTTGTAAGAGAAAAACGGCCATGATAGGAAATGATCATGG

AGTGATGGTATCTTACCAGAAAGTCACGGCTAACTACGTG

>1602 len=160 tot-seq=11

TAGGGAATCTTCGGCAATGGGGGCAACCCTGACCGAGCAACGCCGCGTGAGTGAAGAAGG

TTTTCGGATCGTAAAGCTCTGTTGTTAAAGAAGAACGTGGGTAAGAGTAACTGTTTACCC

AGTGACGGTATTTAACCAGAAAGCCACGGCTAACTACGTG

>1603 len=135 tot-seq=11

TGGGGAATTTTCCGCAATGGGCGAAAGCCTGACGGAGCAATACCGCGTGAGGGACGAAGG

CTCTTGGGTCGTAAACCTCTTTTCTCAAGGAAGATGATGACGGTACTTGAGGAATAAGCA

TCGGCTAACTCTGTG

>1604 len=160 tot-seq=11

TGGGGAATTTTGGACAATGGGCGAAAGCCTGATCCAGCAATGCCGCGTGTGTGAAGAAGG

CCTTCGGGTTGTAAAGCACTTTTGTCCGGAAAGAAATCCTTTGGGTTAATACCTCGGGGG

GATGACGGTACCGGAAGAATAAGCACCGGCTAACTACGTG

>1605 len=155 tot-seq=11

TGGGGAATATTGCGCAATGGGGGAAACCCTGAAGCAGCCAAGTAGCGTGCAGGATGACGG

CCCTATGGGTTGTAAACTGCTTTTGTTTGGGAATAATCGACATTACGTGTAATGTTTTGC

ATGTACCATTCGAATAAGGACCGGCTAATTCCGTG

>1606 len=105 tot-seq=10

TGATCTATATTCACACCACTGCACTTCAGCCTGGGTAACAGAATGAGACCTTGTGTCTTA

AAAAATAAAAATAAAATTTTAAAAAAGAAAAGATTTCTTACTTCA

>1607 len=119 tot-seq=10

GACACGGAAAAAAAACTTAAGAGATTTTAATACATTTCCTGTAAGTCAACCTTGTGATAA

CAAGAAGATGGCCTTCAAGAATTGGCCTCAGCCAAAGGAGTATTGGTTGGCAATGACAG

>1608 len=125 tot-seq=10

TTGGAAGTGGAGCTGGGGATTGTGGTGGCCCAGCAAATGGTCTGAGGACAGCTCTGAAGG

GGCACCTGCAGCTGAGATAGGAGGTGGCAGCAGCCATCATCAGGCACTCATGGGGAAGGG

AGATG

>1609 len=129 tot-seq=10

ACAGCTTACCGAAGCCCAGATCGATATTTTCCAGACCGGCACCTGGGCCAGAGATATCCC

AGTAGTAGAAGTCAATCATGTGAACATCATGACGCTGATAGAAGCGCTTACCGGCCCACA

TGGTGGAAC

>1610 len=134 tot-seq=10

GCAGCAGGTCTGAGACCCTGCAGTGTCTGAGTCTGCCCTCAGAAGGTTCTTTCCCCACAG

ACTCTGGTCAAGGTCCGTGACACCCAGGTGCTGTAGGGTGGAGCAGAGGTCGAGCTGTTC

TTCCACGTCCTTCT

>1611 len=135 tot-seq=10

TGGGGAATATTGGACAATGGGGGCAACCCTGATCCAGCCATGCCGCGTGAGTGATGACGG

CCTTAGGGTTGTAAAGCTCTTTTCTCCGGGACGATAATGACGGTACCGGAGGAATAAGCC

CCGGCTAACTTCGTG

>1612 len=135 tot-seq=10

CTGGAGCAGAAGTGAGCGGAGAGCAGCCTTGGCTTCCTGGGCCCTGGCTGGGAGAGCGGG

GCTGCCTGGGAAAAGGAGCCCCCTCCTCCAACTCAGAGGCCCCTGGACCCTGACCTTGGC

CGGCCAGCGGCCTGA

>1613 len=135 tot-seq=10

TGAGGAATATTGGTCAATGGGCGAGAGCCTGATGCAGCAACGCCGCGTGAACGATGAAGG

TCTTCGGATCGTAAAGTTCTGTTGCAGGGGAAGATAATGACGGTACCCTGTGAGGAAGCC

CCGGCTAACTACGTG

>1614 len=141 tot-seq=10

TGGGGAATATTGCACAATGGGCGCAAGCCTGATGCAGCGACGCCGCGTGTGGGATGACGG

CCTTCGGGTTGTAAACCACTTTCAGCAGGGAAGAAGCTTTTTGTGACGGTACCTGCAGAA

GAAGCGCCGGCTAACTACGTG

>1615 len=142 tot-seq=10

TGGGGAATCTTCCGCAATGGGCGAAAGCCTGACGGAGCAACGCCGCGTGAGTGAAGACGG

CCTTCGGGTTGTAAACCGCTTTCAGCAGGGACGAGGCCGCAAGGTGACGGTACCTGCAGA
AGAAGCCCCGGCTAACTACGTG
>1616 len=143 tot-seq=10
CCATGTGAGGTTGCAGGAGGAGCACAGCTGAGCGTCAGGTATGAACTCCCTCCTGTAGAA
CAATCAGAAACTATCATATCCAATCAGAAACTATCATGCTACCAGAGGATGCATCTTGTT
AAGTCCATTGGTGGTTTTCTCTC
>1617 len=144 tot-seq=10
TGGGGAATATTGCACAATGGGCGCAAGCCTGATGCAGCGACGCCGCGTGGGGGATGACGG
CCTTCGGGTTGTAAACTCCTTTCGCAATCGACGAAGCCTTTTTTGGTGACGGTAGGTTGA
GAAGAAGCACCGGCTAACTACGTG
>1618 len=153 tot-seq=10
TGGGGAATATTGCACAATGGGGGAAACCCTGATGCAGCGACGCCGCGTGAGTGAAGAAGC
GTTTCGGCGCGTAAAGCTCTGTCAGCGGGGAAGAAACACGGTTCGCGAGAGCCGCGGACG
GTACCCGACCAAGAAGCCCCGGCTAACTACGTG
>1619 len=155 tot-seq=10
TGAGGAATATTGGTCAATGGGCGGAAGCCTGAACCAGCCATGCCGCGTGAAGGAATAAGG
CCCTATGGGTCGTAAACTTCTTTAGACGCAGAGCAATAAGGGTCTCGTGAGGCCCGATGA
GAGTATGCGTAGAATAAGCATCGGCTAACTCCGTG
>1620 len=155 tot-seq=10
TGAGGAATATTGGTCAATGGTCGGAAGACTGAACCAGCCAAGCCGCGTGGAGGAAGAAGG
CGCTAAGCGTCGTAAACTTCTTTTGTCGGGGAACAAAATCCGGTACGCGTACCGGACCGA
GCGTACCCGAAGAAAAAGCATCGGCTAACTCCGTG
>1621 len=155 tot-seq=10
TGAGGAATATTGGTCAATGGACGTAAGTCTGAACCAGCCAAGTAGCGTGCAGGATGACGG
CCCTATGGGTTGTAAACTGCTTTTATGTGAGAATAAAGTTAACTACGTGTAGTTATTTGC
ATGTATCACATGAATAAGGACCGGCTAATTCCGTG
>1622 len=155 tot-seq=10
TGAGGAATATTGGTCAATGGGCGGAAGCCTGAACCAGCCAAGTAGCGTGCAGGACGACGG
CCCTACGGGTTGTAAACTGCTTTTATGCGGGGATAAAGTGAGGGACGTGTCCCTTTTTGC

AGGTACCGCATGAATAAGGACCGGCTAATTCCGTG

>1623 len=155 tot-seq=10

TGAGGAATATTGGTCAATGGAGGCAACTCTGAACCAGCCAAGTCGCGTGAAGGATGACTG

CCCTACGGGTTGTAAACTTCTTTTTAAGGGAATAAAGTGAGCCACGTGTGGCTTTTTGC

ATGTACCTTACGAATAAGGATCGGCTAACTCCGTG

>1624 len=155 tot-seq=10

TGAGGAATATTGGACAATGGGTGCAAGCCTGATCCAGCCATCCCGCGTGAAGGATTAAGG

TCCTATGGATTGTAAACTTCTTTTGTACAGGGATAAACCATTCTACGTGTAGGATGCTGA

AGGTACTGTACGAATAAGCACCGGCTAACTCCGTG

>1625 len=156 tot-seq=10

TGAGGAATATTGGTCAATGGGCGAGAGCCTGAACCAGCCAAGTCGCGTGAGGGAAGGCGG

TCCTATGGATTGTAAACCTCTTTTGTCGGGGAGCAAAAAGGCCTCGTGAGGCCACAAGCG

AGAGTACCCGAAGAAAAAGCATCGGCTAACTCCGTG

>1626 len=157 tot-seq=10

CTTCCGATCTCTACGGGAGGCAGCGGGAAGAGAGGGAGACAGAGAAGAGCATTGTGTTGA

GAGACACCTACTGTTAAAAGTAGTAAGAAAGAACAACCAGTAAAATACAAATTTTAAGAA

GGAAGACAGTCAGACATGGTGTCTCATGCCTGTAATC

>1627 len=159 tot-seq=10

TAGGGAATCTTCCGCAATGGACGAAAGTCTGACGGAGCAATGCCGCGTGAATGAAGAAGG

TCTTCGGATCGTAAAATTCTGTTGTTAGAGAAGAACAAGGAGTAGGTAACTACTACTCCC

TTGACGGTATCTAACCAGAAAGCCACGGCTAACTACGTG

>1628 len=160 tot-seq=10

TGGGGAATATTGCGCAATGGGGGAAACCCTGACGCAGCGACGCCGCGTGAGTGAAGAAGG

ATTTCGGTTCGTAAAACTCTGTTGTTAGGGAAGAAAAAATGTATAGTAACTATATACAAA

AGAGACGGTACCTAACCAGAAAGCCACGGCTAACTACGTG

>1629 len=160 tot-seq=10

TGGGGAATATTGCACAATGGGCGCAAGCCTGATGCAGCAACGCCGCGTGAACGATGAAGG

TCTTCGGATCGTAAAGCTCTGTTGTTGGTGAAGAAGGATAGAGGCAGTAACTGGCCTTTA

TTTGACGGTAATCAACCAGAAAGTCACGGCTAACTACGTG

>1630 len=160 tot-seq=10

TAGGGAATCTTCCGCAATGGACGAAAGTCTGACGGAGCAACGCCGCGTGAGTGAGGAAGG

CCTTCGGGTCGTAAAGCTCTGTTGTGAGGGAAGAACAAGTGCCGGCGTAACTACCGGCAC

CTTGACGGTACCTCACCAGAAAGCCACGGCTAACTACGTG

>1631 len=160 tot-seq=10

TGGGGAATATTGGACAATGGGCGCAAGCCTGATCCAGCCATGCCGCGTGTGTGAAGAAGG

CCTTCGGGTTGTAAAGCACTTTTGTCCGGAAAGAAAAGTTCCCGGTTAATACCCGGGGAT

CATGACGGTACCGGAAGAATAAGCACCGGCTAACTTCGTG

>1632 len=160 tot-seq=10

TGGGGAATATTGGACAATGGGCGAAAGCCTGATCCAGCCATGCCGCGTGTGTGAAGAAGG

CCCTCGGGTTGTAAAGCACTTTCAGCGAGGAAGAACGCCTAGTGGTTAATACCCATTAGG

AAAGACATCACTCGCAGAAGAAGCACCGGCTAACTCCGTG

>1633 len=160 tot-seq=10

TGGGGAATTTTGGACAATGGGGGCAACCCTGATCCAGCCATGCCGCGTGTATGAAGAAGG

CCTTCGGGTTGTAAAGTACTTTTGTTAGGGAAGAAAAGGGAAGTGCTAATACCACTTTTT

GCTGACGGTACCTAAAGAATAAGCACCGGCTAACTACGTG

>1634 len=160 tot-seq=10

TGGGGAATCTTCCGCAATGGACGAAAGTCTGACGGAGCAACGCCGCGTGAGTGATGAAGG

TCTTCGGATTGTAAAGTGCTGTTATAAGGGAAGAACATTTGCAATAGGAAATGATTGCAG

ACTGACGGTACCTTGTCAGAAAGCGATGGCTAACTATGTG

>1635 len=160 tot-seq=10

TGGGGAATATTGGACAATGGGGGAAACCCTGATCCAGCGACGCCGCGTGTGTGAAGAAGG

CCTGCGGGTTGTAAAGCACTTTTAGTGGGGATGAAATGTGCAGGGCTAACACCTCTGCAT

TTTGACCTAACCCACAGAAAAAGCACCGGCTAACTCTGTG

>1636 len=160 tot-seq=10

TGGGGAATTTTGGACAATGGGGGCAACCCTGATCCAGCAATGCCGCGTGTGTGAAGAAGG

CCTTCGGGTTGTAAAGCACTTTTGTCCGGAAAGAAATCCCTTGCCCTAATACGGCGGGGG

GATGACGGTACTGGAAGAATAAGCACCGGCTAACTACGTG

>1637 len=161 tot-seq=10

TAGGGAATTTTGCGCAATGGACGAAAGTCTGACGCAGCAATACCGTGTGAGGGATGAAGC

ATTTCGGTGTGTAAACCTCTGTCGATGATGAATAAAGCATTCGAGAGTGGAAAGCTCGGG

TGTTGAAGGTAATCATGAAGGAAGCACCGGCTAACTCTGTG

>1638 len=161 tot-seq=10

TAGGGAATCTTCGGCAATGGACGCAAGTCTGACCGAGCAACGCCGCGTGAGTGAAGACGG

TTTTCGGATCGTAAAGCTCTGTTGTAGGGGAAGAACGTGTGTAAGAGTGGAAAGCTTACA

CAGTGACGGTACCCTACCAGAAAGGGACGGCTAACTACGTG

>1639 len=161 tot-seq=10

TGGGGAATATTGCACAATGGGCGCAAGCCTGATGCAGCGACGCCGCGTGAGGGATGGAGG

CCTTCGGGTTGTAAAGCTCTGTTGTTAAGGAAGAACGAGTGTGAGAATGGAAAGTTCATG

CTGTGACGGTACTTAACCAGAAAGGGACGGCTAACTACGTG

>1640 len=161 tot-seq=10

TAGGGAATCTTCCACAATGGACGCAAGTCTGATGGAGCAACGCCGCGTGAGTGAAGAAGG

GTTTCGGATTGTAAAGCTCTGTTAATCGGGACGAATGGTTTGTGTGCAAATAGTGCATAG

ACATGACGGTACCGGAATAGAAAGCCACGGCTAACTACGTG

>1641 len=161 tot-seq=10

TTAGGAATATTCGTCAATGGGGGAAACCCTGAACGAGCAATGCCGCGTGAACGATGACGG

CCCTATGGGTTGTAAAGTTCTGTTGTTTGGAAAGAACGATAGATAGAGGAAATGCTATAT

ATGTGACGGTACCATTCAAGAAAGCCACGGCTAACTACGTG

>1642 len=162 tot-seq=10

TGAGGAATATTGGTCAATGGACGAGAGTCTGAACCAGCCAAGTAGCGTGCAGGATGACGG

CCCTATGGGTTGTAAAGCTCTGTTATACGGGACGAACGGCAAGGTGGTAAATAGCCATCA

TGAGTGACGGTACCGTAAGAGAAAGCCACGGCTAACTACGTG

>1643 len=168 tot-seq=10

GTATTTATTCTGAAAGGCTGTGTGTTCCAACAAGCATATATTAGCAGCTTCCAACTCGGC

CCCTAGGAATGATTCTCAGTTCTGAGCCGTGCTTCAGGAAAACATTCCCTACACAAACAA

ACAAAAACATGAAGTCAGGTGTCTAAAGAGGAAAAATAACACTGTCAC

>1644 len=173 tot-seq=10

TGGGCGTTACAATGCCTTTACCTATCTGAAGGACGTCTAGTGTAACGAATGAGCCTCAGA

TATCAGAATGGCTGCAGCACTGACCCTGGGATCCGGAGAGAGGTTATAGAGGGACAGTCA
GCAAAGCTGAGAAGGGGAGAGGGATCCTGCCTGAAGGGCAGATTCCAACCAGC
>1645 len=184 tot-seq=10
TGGGGAATATTGGGCAATGGAGGGAACTCTGACCCAGCAACGCCGCGTGAGTGAAGAAGG
TTTTCGGATTGTAAAACTCTTTAAGCGGGGACGAAGAAAGTGACTGTACCCGCAGAATAA
GCATCGGCTAACTACGTGCCAGCAGCCGCGTAATAAACAGATCGGAAGAGCGTCGTGTAG
GGAA
>1646 len=188 tot-seq=10
TCCATGGGTTCGCACAGAGTCGGACATGACTAAGCAACTTCATTTTCACTTTTCACTTTG
ATGCATTGGAGAAGGAAATGGCAACCCATTCCAGTGTTCTTGCCTGGAGAATCCCAGAGA
TGGGGAACCCTGGTGGGCTGCTGTCTATCGGGTCGCACAGAGTCGGAAACGACTAAAGTG
ACTTAGCA
>1647 len=192 tot-seq=10
CCAGAGAGGCAGGAAGGGAATCAGTAGAGAAAGGAGACATGAGGCCACACAGGACAGGGG
ACCCGGAAGTAAGACAAGCTCAGCAGGGTCAGGTCCCCCAAGGTCGCTGCCTTGGATAGG
GGAAGAAAAGTGTCTGCTGGTCTTAGCAAGGATAATGTCCTGCACAGTGAGGGTTATTCC
AGCAATGCAGTG
>1648 len=213 tot-seq=10
GAGGAAAGCTGTCAGAGGGTGAGCCTGGCCCCGTGGCCGCTGGGGAGCAGCTATCTGAGC
ACCCTGTGGAAGACCCTGAGAGCCTGGGTGTGGAGGCTGAAACCTGGATGAACAAGTCCC
CAGACGGCATGTCACCTAAGAAGGAGACACCCAGCCCAGAGATGGAGACCGCAGCCCAAA
AGACAGTGCCAGCTGAAAGTCATGAGAAAGTCT
>1649 len=213 tot-seq=10
GCTAAGGGTAGGAACAGAGAACCGGGTATCTGGGAGCCTAGAACACCTGGGCATAAGGAG
AGCCACCAATCCATGTCTCAGTCAGGGACCAGGCTAGGGGCAGATATAGAGAACCGGGTA
CCTAGGAACCTGAAACACCTGAACCCAGGAGGCTAAAACCTGAGCAGACTCTGCAGAGTC
ACCAGACAGAAGCTTAAGCATCCTTCAGAGACA
>1650 len=215 tot-seq=10
GAGAGTCAGCAGGAGGGACAGGGCACTGGGGACCAGCAGAAGACTCTTCGACAGAGGTGC

CTTATCTAGGAAGAAAACCACATTCAGAATGACTGGGATTTTTCAAAACAAAACAAACCA

CTCACAAGATGAAAAGTGGGAGAGCTAATTTGTTTAGAATGAGGAGGAAGCTTAAGTCCT

GCCACTCAGGCATTTAGCTCTTCCTGAAACATCTA

>1651 len=215 tot-seq=10

GTTCCGGCCACCCCTGCCCGAGCCCTGCAGCTGTACTGTGGGCTCGCCACCAGCCAGCAG

GCAGACTGGGCCCCTTCCCCATGCCATGGTCTCCAGAGCCTCCTCCAGCTGCAGGTCTGG

GATCTGAAGCTCAGCTGCCAGCTCATGGAGCTGTGCATCTTCCTCCACAGAAGCCCCAGC

CATGGATGGGGTGAGGCGGGTTCTAGCCACATGGG

>1652 len=217 tot-seq=10

TGGGGGTGAGCGCCAGCTGGTTTGGGTCTGTCCAAGAAGGTGAGACTAGCAATGCAGAGG

GACCAAGATCAGGGTGCTTCTAAGGCCTTCACCCTGGCTTGCTGCTCTGATGGACATTGT

CTTAGGCATCATTTACGTCTAGCAAAGAGATATGCTGGTGATTTGAAATGTCTGGACGTC

TAGCAAAGAGATATGCTGGTGATTTGAAATGTCTGGT

>1653 len=225 tot-seq=10

TGAGGAATATTGGTCAATGGGCGAGAGCCTGAACCAGCCAAGTAGCGTGAGGGATGACTG

CCCTATGGGTTGTAAACCTCTTTTATTTGGGAATAACAGAAAGTATGTATACTTTTTTGC

ATGTACCATAATGAATAAGCATCGGCTAACTCCGTGTGGGAATAACAGAAAGTATGTATA

CTTTTTTGCATGTACCATAATGAATAAGCATCGGCTAACTCCGTG

>1654 len=227 tot-seq=10

GATGCGAACAGATGTCAGTGGTGGCAGATCATTGCAATTCCTGTCTGCGTTCTGCATGCA

TCCCAAAAAGAGAACATGAACGTCTTCGAACTGAAATGAAGAAAATAATTTTGGAAATAC

TCTTGGATGCCTGAGCTGGATGAGCACTCCTCAGAAACAATGAGGATCCAGTGAGGTGCA

TGTGAGGACAGGAGGCTTGTGTTGGGGGTAGCATCCCCTTTCTCCTC

>1655 len=229 tot-seq=10

AGCTGGGACAGTTATGGATCACGACCAAGTCTAGGGCGCGACGCTGGGAGTGTGTAAAAG

CTGAGGGAAGAGCCCAGCAGAGCAAGGGAGCAGGGAGCTGAGGGAAGAGCCCAGCAGAGC

GAGGGGGCAGGGAACTGGTGCCCTGCCCTGAGGCGAGCAAGGGAGCAGGGAGCTGAGGGA

AGAGCCCAGCAGAGCGAGGGGGCAGGGAACTGGTGCCCTGCCCTGAGGC

>1656 len=230 tot-seq=10

GTGCTTGTTGATGCTGTGGAAATAAAAAGGTTTCCCGTTGATGAAGAACTGGCTCTCAGT

GACAGCCACAGTGCGGATCCCCACAGGGAGTGTGTAGAAGTCAGACAAAGGCCCCAGTGA

CGTCTGTGCAGTCAGCCGCACCTAAGATAGCCAAAGCGCCAGGTGTGAGCACCCCGACAG

CCTGAGCCCCATCCGGCCCTCCCTCTGGCAGGAAGGCCCCTCGTGCACCC

>1657 len=230 tot-seq=10

TATGTGGCATCATACGAGGAACGTATCATACGTGGTACATATGATACCTAAGGTGGAGTG

GTCGGCGTTCCTGGAGGCGGCCGATAGCTTGCGCCTGATCCAGATGCCTAAAGGGCCAGT

TGAGGGATATGAGGAGAATGAAGAGTTTCTGAGGACCATGCACCACCTGCTGCTGGAGGT

GGAAGTGATAGAGGGCACCCTGCAGTGCCCGGAATCTGGACGTATGTTCC

>1658 len=181 tot-seq=10

GCAGGTATATATTGTTTTTACTACAACATAAAAATTAGATAAAAGGTATATGGGGGAAAC

AGATTCAGCAATGCTGTTTTCTTCTTCCTTTTAAGTGTATAACATTTTTACCCTCTTTCC

AGATGCAGCAGCCCCCCCAGCCCCAGCCCCCCCAGCCTCCCCAGCCCCAGCAGTCCTCGC

A

>1659 len=104 tot-seq=9

CATTAGTAAATGTAACAACTAGTAACCTATCTATATCAACAGGATTTTCACCCTCTGTTA

TCATTTTTATAATTCTTTCAACTAATACAGCAGTTTTTCCTGAG

>1660 len=106 tot-seq=9

TAAATGACTCAGACAAGGGAGGGAAGGACCTCCCAGCAGGGGACATAGGCAACAAAGAAA

ATTAAAAATAGGTAAATTCTATAAAAATACAGCCAAGGAAGGGGGA

>1661 len=106 tot-seq=9

TGGGGAATATTGCGCAATGGGGGAAACCCTGACGCAGCGACGCCGCGTGCGGGATGAAGG

CCTTCAGGTGAGTGTACCTTTCGAATAAGCGCCGGCTAACTACGTG

>1662 len=108 tot-seq=9

CACAGTCGGGATGAACTGGCAGGTGGGCTTCTGCTCCAGAAGAGATGCGGGAGCCCTGCA

GGTTTGAGTGAAGAGTGCATGGTCCAACACGGTCTTCAGAGCATCGTT

>1663 len=110 tot-seq=9

GAGGAGGAGGATCAGGAGGACCAGCAGGAGCAGGAGGAGGAGAAGGAGGAGCAGGAGCAG

GAGGACCAGCAGGAGCAGGAGGAGGAGCAGGAGCAGGAGGATCAGGAGGA

>1664 len=111 tot-seq=9

CAGAGCAGGTCAGGCAAGCCCACCTAGGAAGTCGGGGAGACGGGGCTTCCCACAGGTCCT

GGGCCTAGCGCCTGTGTCCACTCATGAGCCCCACTGAGGAAAGGGGTTTGC

>1665 len=111 tot-seq=9

CAGGTTATTTTATTTTTTGCTTTAGACTTCTCATTAAAATATATGATATATACACAAGAG

TGCACTTATCAGCACATTTGACTTATCAATAACTGTACCCACCCACTGGAA

>1666 len=123 tot-seq=9

TAGGGAATCTTCCACAATGGGCGCAAGCCTGATGGAGCAACACCGCGTGAGTGAAGAAGG

ACAGGGGTAGTAACTGACCTTTGTTTGACGGTAATCAATTAGAAAGTCACGGCTAACTAC

GTG

>1667 len=124 tot-seq=9

GGGGACTCTGGGGCCTACTTGGAGGGGGCATGGGTGCTTCACACCACAGCTTCTCAGGGA

GCAATCACTATGAGGGTCTGGTAGAATCTAACTGAAGAGTGGGCAGCTGCAGGTATGGCT

GAAG

>1668 len=129 tot-seq=9

CAGGAACTCAAAACTGCAGCAGCAGCAGCAGCAGCAGCAGCAGCAGGAGCCAGAGGAGCA

GAAGCACCAGAAGCAGCAACAGCAGAAGCAGCAGCAACAGCAGCAGAAGCAGGGACAGAA

TCACCAGCA

>1669 len=134 tot-seq=9

TGGGGAATATTGCGCAATGGGGGAAACCCTGACGCAGCGACGCCGCGTGATTTAGAAGGC

CTTCGGGTTGTAAAAATCTTTTTTATGGGATGAAAATGACAGTACCATAAGAATAAGGTC

CGGCTAAATACGTG

>1670 len=135 tot-seq=9

TGGGGAATTTTGCGCAATGGGGGAGACCCTGACGCAGCAACGCCGCGTGCGGGACGAAGG

CCTTCGGGTTGTAAACCGCTTTCAGCAGGGAATATAGTGAAGGTACCTGCAGAAGAAGCT

CCGGCTAACTACGTG

>1671 len=135 tot-seq=9

TGGGGGATATTGCACAATGGGGGAAACCCTGATGCAGCGACGCCGCGTGAGTGAAGAAGT

ATTTCGGTATGTAAACTTCTATCAGCAGGGAAGAAAATGACGGTACCTGAGTAAGAAGCA

CCGGCTAAATACGTG

>1672 len=135 tot-seq=9

TGGGGAATATTGCACAATGGGCGCAAGCCTGATGCAGCAACGCCGCGTGAACGATGAAGG

TCTTCGGATCGTAAAGTTCTGTTGCAGGGGAAGATAATGACGGTACCTTACTAAGAAGCT

CCGGCTAAATACGTG

>1673 len=135 tot-seq=9

TGGGGGATATTGCACAATGGGGGGAACCCTGATGCAGCGATGCCGCGTGAGTGAAGACGG

CCTTCGGGTTGTAAAGCTCTGTCGCAGGGGACGAAAATGACGGTACCCTGCAAGAAAGCT

CCGGCTAACTACGTG

>1674 len=135 tot-seq=9

TAGGGAATCTTCCACAATGGGCGAAAGCCTGATGGAGCAACGCCGCGTGGGTGAAGAAGG

TCTTCGGATCGTAAAGTTCTGTTGCAGGGGAAGATAATGACGGTACCCTGTGAGGAAGCC

CCGGCTAACTACGTG

>1675 len=136 tot-seq=9

TGGGGAATATTGCACAATGGGGGGAACCCTGATGCAGCGACGCCGCGTGAAGGAAGAAGT

ATCTCGGTATGTAAACTTCTATCAGCAGGGAAGAAAGAGGACGGTACCTGACTAGGAAGC

CCCGGCTAACTATGTG

>1676 len=136 tot-seq=9

TCGGGAATATTGCGCAATGGAGGAAACTCTGACGCAGTGACGCCGCGTATAGGAAGAAGG

TCTTCGGATTGTAAACTATTGTCGGTAGGGAAGAGAAAGGACAGTACCTAAGGAGGAAGC

TCCGGCTAACTACGTG

>1677 len=136 tot-seq=9

TCGAGAATCTTGGGCAATGCGCGAAAGCGTGACCCAGCAATGCCGCGTGTGTGATGAAGG

CCTTCGGGTCGTAAAGCACTGTCTCCCGTGACGAAATTTGACGGTAGCGGGGAAGGAAGC

CACGGCTAACTACGTG

>1678 len=137 tot-seq=9

CAAGGAATTTTCCGCAATGGGCGAAAGCCTGACGGAGCAACGCCGCGTGTGGGATGACGC

TTTTCGGAGTGTAAACCACTGTTCATGGGGACGAAGCATTGACGGTACCCATGGAGGAAG

GCCCGGCTAACTACGTG

>1679 len=137 tot-seq=9

ACAGATAGAGGCAGCCGATGCCAGTGCAAGGGTTGCTGCCTGACAGGCATTGAACTGGAT

ACTTCTCCATGCAGGGGCAGAAGGGCTGCTGTTGAGGTCTTGGGAAGACAACTCGGTCAA

GTACATGTGCTTCCGGC

>1680 len=137 tot-seq=9

TGGGGAATATTGCGCAATGGGGGCAACCCTGACGCAGCAATACCGCGTGAGTGAAGAAGG

TTTTCGGATCGTAAAGCTCTGTTATTGGGGAAGAAAACATGACGGTACCCAATGAGGAAG

TCCCGGCTAACTACGTG

>1681 len=137 tot-seq=9

TGGGGAATATTGGGCAATGGGCCCAAGCCTGACCCAGCAACGCCGCGTGAAGGAAGAAGG

CTTTCGGGTTGTAAACTTCTTTTGTCAGGGAAGATGAACAGACTGTACCTGACGAATAAG

CCACGGCTAACTACGTG

>1682 len=137 tot-seq=9

TGTCAGTGGACTTTCCGGGTTCCAGGCTGGGAGAGGAGGGAGAGAAGTGGGAGCTGGAAG

GCTCCAAAGGTAACAGGAAGTATTTGCATTTTTAAGAGAGAGAGACCAAAGTGTGTTTCT

AGGTTCGGGAGAGAGAT

>1683 len=138 tot-seq=9

TGGGGAACATTGGGCAATGGGCGAAAGCCTGACCCAGCAACGCCGCGTGAAGGAAGAAGG

CCCTCGGGTTGTAAACTTCTTTTGTCAGGGACGAAGCAAGTGACGGTACCTGGCGAATAA

GCCACGGCTAACTACGTG

>1684 len=138 tot-seq=9

TAGGGAATCTTCCACAATGGACGCAAGTCTGATCCAGCAATTCTGTGTGTGTGAAGAAGG

TTTTAGGACTGTAAAACACTTTTAGTAGGGAAGAAAAAAATGACGGTACCTACAGAAGAA

GCAACGGCTAAATACGTG

>1685 len=138 tot-seq=9

TGCGGAATATTGGGCAATGGGCGAAAGCCTGACCCAGCAACGCCGCGTGAAGGAAGAAGG

TCTTCGGATTGTAAACTTCTTTTATGAGGGACGAAGGACGTGACGGTACCTCATGAATAA

GCCACGGCTAACTACGTG

>1686 len=138 tot-seq=9

TGGGGAATTTTGTGCAATGGGGGAAACCCTGACACAGCGACACCGCGTGAACGAAGAAGC

CCTTTGGGGTGTAAAGTTCTGTCGGCTGGAACGAAAACAATGACGGTACCAGCAAAGGAA

GCATCGGCTAACTACGTG

>1687 len=138 tot-seq=9

TAGGGAATCTTGCACAATGGGCGAAAGCCTGATGCAGCAACGCCGCGTGAAGGATGAAGG

CCTTCGGGTTGTAAACTTCTGTTCTAAGGGAAGAAACAAATGACGGTACCTTAGGAGCAA

GCCCCGGCTAACTACGTG

>1688 len=140 tot-seq=9

TGGGGAATATTGCACAATGGGCGCAAGCCTGATGCAGCGACGCCGCGTGAGGGATGACGG

CCTTCGGGTTGTAAACCTCTTTCACCAGGGACGAAGCGTGAGTGACGGTACCTGGAGAAG

AAGCACCGGCCAACTACGTG

>1689 len=143 tot-seq=9

TGGGGAATCTTCCGCAATGGACGAAAGTCTGACGGAGCAACGCCGCGTGAGTGAAGAAGG

TTTTCGGATCGTAAACCGCTTTTGATTGGGAGCAAGCCTTTTGGGTGAGTGTACCTTTCG

AATAAGCGCCGGCTAACTACGTG

>1690 len=143 tot-seq=9

TGGGGAATATTGGGCAATGGGCGAAAGCCTGACCCAGCAACGCCGCGTGAAGTATGAAGG

CCTTCGGGTTGTAAACCGCTTTTGATTGGGAGCAAGCCTTTTGGGTGAGTGTACCTTTCG

AATAAGCGCCGGCTAACTACGTG

>1691 len=143 tot-seq=9

TGGGGAATATTGCACAATGGGCGCAAGCCTGATGCAGCGACGCCGCGTGGGGGATGACGG

CCTTCGGGTTGTAAACCTCTTTCGACTATGACGAAGCCTTTTTGGTGACGGTAGTGGTAG

AAGAAGCACCGGCTAACTACGTG

>1692 len=143 tot-seq=9

TGGGGAATCTTCCGCAATGGACGAAAGTCCGACGGAGCAACGCCGCGTGAGTGATGAAGG

CCTTCGGGTTGTAAACCGCTTTTGATTGGGAGCAAGCCTTTTGGGTGAGTGTACCTTTCG

AATAAGCGCCGGCTAACTACGTG

>1693 len=144 tot-seq=9

AGAACCTAAGTAAGGGAGGGCGAGCAAGGAAGCCGCTGCTCAGGAAGGAGGGGAGGAAAT

TAGAATGTCCAGAACATCTGGATGTATATGTCGGTCCAGAGGTTGCAGATCAAGTCACAG

TTCCCACTTCAGCCAAGAGGAGTA

>1694 len=144 tot-seq=9

ACACCATAAACTTGGCCGAGGTAGCCTAGCCAAAGAGCTGACTGAAGACTATGAGCACAA

CCAGACGTTTTTGAGGAAGATTTACCATGCATTGCTTGAGGTTGATGTATTGGATGGCAC

CCTGCAATGCCCAGAATCTTTTTC

>1695 len=145 tot-seq=9

TAGGGAATCTTCCACAATGGACGCAAGTCTGATGCAGCGACGCCGCGTGAGGGATGGAGG

CCTTCGGGTTGTAAACCTCTTTTGTTAGGGAGCAAGGCATTTTGTGTTGAGTGTACCTTT

CGAATAAGCACCGGCTAACTACGTG

>1696 len=145 tot-seq=9

GGAGAAATCGGAATGCAAATACGTGTTCACATATATTAAGTTCTTTTTATGTGCTTGCCA

CTATATCAAACACCTTACCTATGTAATTATTTCATCCTCATGAGTTAGATATTTTTATTC

CTGATGAGAAAACTGGGTCATTTGC

>1697 len=151 tot-seq=9

TAGGGAAATGGACGCAAGTCTGATGGAGCAACGCCGCGTGAGTGAAGAAGGGTTTCGGCT

CGTAAAGCTCTGTTGTTGGTGAAGAAGGACAGGGGTAGTAACTGACCTTTGTTTGACGGT

AATCAATTAGAAAGGCACGGCTAACTACGTG

>1698 len=152 tot-seq=9

CAGGGAGCTCAGGTCCTTTGGAGAGCTGGCTCTACACAGCACCCTGGAACTTCCTTTGGT

CTGAGTCTAAGTGTGTTTCATGTTTGCCTCCTCTAGCGAGAAGCTTGCACTCCGTGGGAC

AGGGGCCTCATCCCTTGCTCAGCACTGTACCT

>1699 len=155 tot-seq=9

TGAGGAATATTGGTCAATGGACGGAAGTCTGAACCAGCCATGCCGCGTGCAGGATGAATG

TGCTATGCATTGTAAACTGCTTTTGTACGAGGGTAAACACAGATACGCGTATCTGCTTGA

AAGTATCGTACGAATAAGGATCGGCTAACTCCGTG

>1700 len=155 tot-seq=9

TAAGGAATATTGGTCAATGGAGGCAACTCTGAACCAGCCATGCCGCGTGCAGGAAGACGG

CCCTACGGGTTGTAAACTGCTTTTGTACCGGAATAAACCCCTTTACGTGTAAAGGGTTGA

ATGTACGGTAAGAATAAGGATCGGCTAACTCCGTG

>1701 len=155 tot-seq=9

TGAGGAATATTGGTCAATGGGCGTAAGCCTGAACCAGCCAAGTAGCGTGAGGGATGACTG

CCCTACGGGTTGTAAACCTCTTTTGTTTGGGAATAAAGTGCGGGACGCGTCCCGCATTGC

ATGTACCATTTGAATAAGGACCGGCTAATTCCGTG

>1702 len=155 tot-seq=9

TGAGGAATATTGGACAATGGGCGCAAGCCTGATCCAGCCATGCCGCGTGAAGGATGAATG

TCCTCTGGATTGTAAACTTCTTTTATTTGGGACGAAAAAAGAGCATTCTTGCTCACTTGA

CGGTACCAAGTGAATAAGCACCGGCTAACTCCGTG

>1703 len=155 tot-seq=9

TGAGGAATATTGGACAATGGATGGAAATCTGATCCAGCCATGCCGCGTGGAGGAAGAATG

TCCTATGGATTGTAAACTTCTTTTGTACGGGAGCAATAAGGGTCATGAATGGCCTGATGA

GAGTACCGTACGAATAAGCACCGGCTAACTCCGTG

>1704 len=155 tot-seq=9

TGAGGAATATTGGTCAATGGACGCAAGTCTGAACCAGCCAAGTAGCGTGCAGGATGACGG

CCCTATGGGTTGTAAACTGCTTTTATATGGGGATAAAGTGGGGAACGTGTCATGCATTGC

AGGTACCACATGAATAAGGACCGGCTAATTCCGTG

>1705 len=155 tot-seq=9

TGAGGAATATTGGTCAATGGACGAGAGTCTGAACCGGCCAAGTAGCGTGAAGGAAGACTG

CCCTATGGGTTGTAAACTTCTTTTATAAGGGAATAAAGTCAGGTACGTGTACCTGTTTGT

ATGTACCTTATGAATAAGCATCGGCTAACTCCGTG

>1706 len=156 tot-seq=9

TGAGGAATATTGGTCAATGGGCGAGAGCCTGAACCAGCCAAGTCGCGTGAGGGAAGACGG

TCCTATGGATTGTAAAGCTCTTTTGTCGGGGAGCAAAGGGGCCACGTGTGGTCAAAGGTG

AGAGTACCCGAAGAAAAAGCATCGGCTAACTCCGTG

>1707 len=160 tot-seq=9

TAGGGAATCTTCCGCAATGGGCGAAAGCCTGACGGAGCAACGCCGCGTGAGTGATGAAGG

TTTTCGGATCGTAAAGCTCTGTTGCCAGGGAAGAACGCTTGGGAGAGTAACTGCTCTTGA

GGTGACGGTACCTGAGAAGAAAGCCCCGGCTAACTACGTG

>1708 len=160 tot-seq=9

TGGGGAATATTGGACAATGGGCGCAAGCCTGATCCAGCCATGCCGCGTGTGTGAAGAAGG

CCCTCGGGTTGTAAATCACTTTCAGTGGGGAAGAAAGCCTGGTGGTTAATACCCGTCAGG

AAGGACATCACCCACAGAAGAAGCACCGGCTAACTCCGTG

>1709 len=160 tot-seq=9

TGGGGAATATTGCACAATGGGGGAAACCCTGATGCAGCCATGCCGCGTGTGTGAAGAAGG

CCTTCGGGTTGTAAAGCACTTTCAGCGAGGAGGAAAGGTTGGTAGCTAATAACTGCCAAC

TGTGACGTTACTCGCAGAAGAAGCACCGGCTAACTCCGTG

>1710 len=160 tot-seq=9

CTAAGAATATTCCGCAATGGGGGAAACCCTGACGGAGCGACGCCGCGTGGACGATGAAGG

CCGGAAGGTTGTAAAGTCCTTTTGCGGGTGAGGAATAAGGACCGGAGGGAATGCCGGTAC

GATGACTGTAACCTGCGAATAAGCAACGGCTAATTACGTG

>1711 len=160 tot-seq=9

TAGGGAATCTTCGGCAATGGACGAAAGTCTGACCGAGCAACGCCGCGTGAGTGAAGAAGG

TCTTCGGATCGTAAAACTCTGTTGTTAGAGAAGAACACGAGTGAGAGTAACTGTCCATTC

GATGACGGTATCTAACCAGCAAGTCACGGCTAACTACGTG

>1712 len=160 tot-seq=9

TAGGGAATATTGGACAATGGAGGGAACTCTGATCCAGCAATTCTGTGTGTGTGAAGAAGG

GTTTCGGCTCGTAAAGCTCTGTTGTTGGTGAAGAAGGACAGGGGTAGTAACTGACCTTTG

TTTGACGGTAATCAATTAGAAAGTCACGGCTAACTACGTG

>1713 len=160 tot-seq=9

TAGGGAATCTTCCACAATGGACGCAAGTCTGATGGAGCAACGCCGCGTGAGTGAAGAAGG

CCTTCGGGTTGTAAAGCACTTTCAGCGGGGAGGAAGGCGTTAAGGTTAATAACCTTAGCG

ATTGACGTTACCCGCAGAAGAAGCACCGGCTAACTCCGTG

>1714 len=160 tot-seq=9

TAGGGAATCTTCGGCAATGGACGAAAGTCTGACCGAGCAACGCCGCGTGAGTGAAGAAGG

TTTTCGGATCGTAAAACTCTGTTATTAGGGAAGAACAAATGTGTAAGTAACTATGCACGT

CTTGACGGTACCTAATCAGAAAGCCACGGCTAACTACGTG

>1715 len=160 tot-seq=9

TAGGGAATCTTCCGCAATGGGCGAAAGCCTGACGGAGCAACGCCGCGTGAGTGATGAAGG

TCTTCGGATCGTAAAACTCTGTTATTAGGGAAGAACAAGGATGTAAGTAACTGTGCATCC

CTTGACGGTACCTAATCAGAAAGCCACGGCTAACTACGTG

>1716 len=161 tot-seq=9

TAGGGAATATTGCACAATGGAGGAAACTCTGATGCAGCCATGCCGCGTGTGTGAAGAAGG

TTTTCGGATCGTAAAGCTCTGTTGTTAGAGAAGAACGTTGGTAGGAGTGGAAAATCTACC

AAGTGACGGTAACTAACCAGAAAGGGACGGCTAACTACGTG

>1717 len=161 tot-seq=9

TGGGGAATATTGCACAATGGGCGCAAGCCTGATGCAGCAACGCCGCGTGAACGATGACGG

CCTTCGGGTTGTAAAGTTCTGTTATACGGGACGAATGGTACGACGGTCAATACCCGTCGT

AAGTGACGGTACCGTAAGAGAAAGCCACGGCTAACTACGTG

>1718 len=161 tot-seq=9

TAGGGAATCTTCCGCAATGGACGAAAGTCTGACGGAGCAACGCCGCGTGAGTGAGGAAGG

TTTTCGGATTGTAAAACTCTGTTGTTAGGGAAGAACAAGTACCGTTTGAATAAGGCAGTA

CCTTGACGGTACCTAACGAGAAAGCCCCGGCTAACTACGTG

>1719 len=161 tot-seq=9

TGGGGAATCTTGCGCAATGGGCGAAAGCCTGACGCAGCGACGCCGCGTGGGTGATGAAGG

CCTTCGGGTTGTAAAGCCCTGTGGGGAGGAACGAACCGTGTCCGGGTGAATATCCCGGGC

ACCTGACGGTACCTCTTTAGCAAGCATCGGCTAACTCTGTG

>1720 len=161 tot-seq=9

TGGGGAATCTTCCGCAATGGACGAAAGTCTGACGGAGCAACGCCGCGTGAGTGAAGAAGG

TTTTAGGATCGTAAAGCTCTGTTGTTTAGGACGAATGTGCCTTATGCGAATAGTATGAGG

TAATGACGGTACTAAACGAGAAAGCCACGGCTAACTACGTG

>1721 len=161 tot-seq=9

TGGGGAATCTTGCGCAATGGACGAAAGTCTGACGCAGCCACGCCGCGTGAGTGAAGAAGG

CCCTCGGGTTGTAAAGCTCTGTCGCCCGGGACGAAAATTTATTGGGTTAATAGCCCAGTG

AACTGACGGTACCGGGAGAGGAAGCACCGGCTAACTCCGTG

>1722 len=165 tot-seq=9

CTGGAGTTCAGACGTGTGCTCTTCGATCTCCTACGGAGGCAGCAGGTGCTGGGGACAAGC

TCACTGCTGAGAGTCGGTCTGGAGGATCTCATGGTGGTGCAGACCCTGGACCACATTGTC

ACAGATGGTGTTCACGCTGGTGCAAAGGTGAGAGGTGACAATGTG

>1723 len=167 tot-seq=9

ATCATAGGAGGATTTGGTGGGAGGTAAGCTTGGGGCTCAATGGGGGGTCAGTATAGGATT

CAAAGGGGGATCCATGGCAGAGTGGTTGGATGGCCAGAGTTGATGAGAGATCAGTGGAAG

TTCAATGAGGGGCAATAAGAGAATGGATAAGAGTCAGTGAAGAGGGG

>1724 len=173 tot-seq=9

GATGAGCTATAAACAAGAAAACAGGTGAACGTCTCTTTAAGAAGCATTCTTAACAAATTC

TATTCTTACTTGCCCACTAGAGAAATTGATCCTCCTGACTCTGTAGTTAATTCTCTGGAG

AGGGCAAAAGAGCCTACAGGTGAGAGAACGAATCTTAACTCTGAAGATTGGAC

>1725 len=175 tot-seq=9

CCCCTTTCTCTTCTGACCAGGCGCCCATGCTTGCAAAAGCCCATCTAAAACACTGGACAA

AGTCAGCTCCCAGTTCCAGATTCCAAAGTAACCGCTGGACCTAAAGACCCACCTACAGGG

CCCTAAACTTACCAAATGAATAACCATCACTTCACGACTCCCCTCCCCCACTCCC

>1726 len=178 tot-seq=9

ACGGGAGGCAGCAGGCGCATAATCAGGAGGGGCCCCTGAGTACCAGGGTCAGGACTTGGA

GCTCATCTGCAAGGTAGTGGGGAGTTATAGAAGGAGATAGAGCTGGAGGGTGGGGCGCAG

TAGCTTATGCCTGTACTCCCAGCACTTTGGGAAGGTGAGGCAGGAGAATCTCTGAAGC

>1727 len=179 tot-seq=9

TAGGGAATCTTCCACAATGGACGCAAGTCTGACGGAGCAACGCCGCGTGAACGATGACGG

CCTTCGGGTTGTAAAGTTCTGTTATATGGGACGAACAGGACATCGGCTAATACCCGGTGT

CTTTGACGGTACCGTAAGAGAAAGCCACGGCTAACTACGTGACAGCAGCCGCGGTAATT

>1728 len=185 tot-seq=9

CATCACAAAGACGGAGAATCCCCTTATGGCTGTTTTTTGTTTTTTCCTTTTTTTACCATT

TGTAGGCAATAGCTGGCAACAGTGAAAAGGGAAAGGACTGCATAAAGAAGTTGAAGAATG

GGGCTGGAGAGCCATCTCAGAGGTTAAGGGCACAGGCTTCTCTTGCAGAGGAGCTGGTTC

TGTTC

>1729 len=192 tot-seq=9

GAGGAGGGGGCTCTGCAGCCTGTGGAATGGCAATGTTTGTGCGTGCGTGCGTGCGTGCAT

GTGTGTGTGTATCCAACTGAGATTAAGCCCTGAAAGCAGGACTCAACCTTGCCTCCCCCT

CCCCCATGCAATCACATCCTGCCTGTGACTGTGTCACCTCCATCCCTCCAGCAGCCGCGA

TAATCTCCAGAT

>1730 len=199 tot-seq=9

CGTGTGCTCTTCCGATCTCCACGGGAGGCAGCAGCAGGCTAGATGACCTTTCCAGTGAGT

GTGAGGGCAAGCAGGCAAAAACCCAAAACTTCCTTCTTCCCTTTCCTGTTATCTGGGCAC

CAGAAGGTGCCACTCACATTTAGGGTGATTCTTCTTGCTTCAGATAATCCGAGGTCAAGA

AAACCCCACACAGAAGTGC

>1731 len=203 tot-seq=9

TGTCTTGACTCTGCCTTATCGGTCCTATCTACCTGTTATGTCAGGACTGTTGGGATTCTG

ACGCCTGAGGCTCTAGTTCAGTCAGTCAGGCTCTTAGGGTCATCAGCTCTGTAAAGTCCT

CCCTAGAGGGAGGTTGTTGGAGGACGCACGGTTTACACTGCTCTTTTGTCCTGTCTTGTT

TCTTTTGCCAGTACCATTTAGGT

>1732 len=208 tot-seq=9

GGGGTCCTTCTTTTATGTCCCCTCACTTGCACAGGGAGCTAGCTCAGTATTCTTTTTCTG

ACCTGACATCTTTAGGGACAGGAACAGGAACTTCTGCTGCCTGAACCACTGAGGCCTTTG

GGAAGGTCTGGGACGCTGACCTCTCCTTGTAGAAATGTGTGGGTCTTGCCCTTCCCTGCC

CCTGGTGGAGACCCTTCCACCCCCTTGG

>1733 len=209 tot-seq=9

CTGAGGTAACGTGCAGCCCTGAGGCTGGTCCGAACGGGAGGAGACTTCTCCAGCAGCCCA

GGTGCCAGTCCACACAGACAGGACTGGAAGCCCCTGGGCAGCAGGTCAGGTGACCCGGGG

AGTGCAGCCTGAGCCCCCAACGGCAGCAAACGTGAAGGTCTCAGGTGGTTACAGAATCAC

TCAGCCCTCAGGCCCCCACCACTCTCCTC

>1734 len=210 tot-seq=9

TGGGGAATATTGCACAATGGGGGAAACCCTGATGCAGCGACGCCGCGTGAGCGAAGAAGG

TATTCGTATCGTAAAGCTCTGTTGTAAGAGAAGAACGTGTGTGAGAGTGGAAAGTTCACA

CAGTGACGGTAACTTACCAGAAAGGGACGGCTAACTACGTGCCAGCAGCCCGCGGTAATG

GCAAGATCGGAAGAGCGTCGTGTTGGGGAA

>1735 len=211 tot-seq=9

ATGGAGGACAGAGGGAATTGGGAGGAGATATCAACTGCAGTCCCCCACAGACAGGCAGCC

AACTTCACCACCCTCTAAGGCAGAACTCAACACTCTGACCAAGTCTCTAAAACAAAACAT

TTACAAAGCCCATAGTAAAATAAAATATTGGTAGACATGTGAAGAAATAGAAAAATGGGA

CTCAAAAAAAGAAAAGCAGTCATTAGAAACC

>1736 len=212 tot-seq=9

GAGGATGTGGGTCCCCAATGGAGTGGAGGCCTGGAAAGAGGGGACATGGGTGACGAGTCT

TCGATACTGGAAGGGCAGCCTACCTGAGAGGAGCTGTTGAGGCCCCAGCCATGCCGCCCA

CAGTCGAGGGTGCCCTGCAGGCCTCTGGCCCCCAGGAAGGCAGGGCAGGAGTCCTGCATC

CTTGCCCAGCATGGCGGCCAGAAAGGTGAGAG

>1737 len=213 tot-seq=9

TGACTGGAGTTCAGACGTGTGCTCTTCCGATCTCCTATGGGAGGCAGCAGCTTGTCTCTG

ACTACCCGGAGGACATGGAGCACCCCAAATAGGAACTTTAGAGGGATTGAAATCTGTTGC

CTGTTCCACTAGGAATATTGTTTGCAAGGCACAAGGTGTCTTTTGGTAGTGAGCGCGCTC

TGCGCATGCGCAGGTCCATTCGGGAATTACTGC

>1738 len=223 tot-seq=9

GCTTTGTATGACCTGTTAAATTACAACTGACACCCAAATAAGTCCAAGTGCATTCGTCTT

TCTAGGATTATTTATCAAATTCCTACATTTTTTCAAGCTGTTTAAGATACAAAGATTAAA

AAAGAACGATTTTTTAACCTATCGCATCTCCAGTTTGGTGTAATAGAAAGTGAAATAAAA

GGATGACAGTAATACAGAGTAAAAAGTGATATGAATGTAAAAG

>1739 len=223 tot-seq=9

GATAGATGGACACACAGATGGTTGGACAGAGAGATGCATGGATATAGGCCTAGTGCATAA

CAGGCAGTCAATAATGATGGGCAGATGGAAGGGTGGATGGATATCTAGGGAGCAAAGCAC

CTGGGATGAAATGTGCACAAAGCACAACTCAAAAGTGCAAAAGATACGAGAGCCTCCTTA

AGTGGCACCTCTAAAACCTCCACTTCATTTCATCTCAGACCAA

>1740 len=230 tot-seq=9

TGGGTTTTTTTTTTCAAGCAGAAGACGGCAGACGAGAGCGACAAGGGACGGGAGGGCAGA

CGGGGGCGCGGCCGAGCGCCGACGGGAGGCAGCAGGGGGGAAGAGGGCACAAGGGGCGAA

AGCCTGATGCAGCGACGCCGCGTGAGCGAAGAAGTATTTCGGTAGGTAAAGCTCTATCAG

AAGGGAAGAAAATGACGGTACCTTACTAAGAAGCTCCGGCTAAATACGTG

>1741 len=239 tot-seq=9

ACTGGTCGAAAGGAGAGGAACATGGTAGGCAGGAGGAAGATGCATAGTGCCAGGGCCAGA

AATATGAAGCCCTTGCCCATGTGAATGTAAACTGGGGAAGAAATTTAGAAAGATAACTAC

AGGACTTAGTCATTCTAACCTCGCTCTCTCCCTCTCTCCCTCTCTCTCTCTCTCCCTAGA

TCTCTCCCTAATTCTCTCTCTCTTTCCTCTCACAGCATGAGGAGTTCAACCTCAGTACT

>1742 len=240 tot-seq=9

CTGGGGCCCTCTGCTTGTCCTAGCACCTACAGCGTTGAGACTTTTGCACCCACCCAAGTG

TGGACGTGGAGGCTGGGGGCCAAGGTCTCTGGTGACTGGACATCAGGGGGCAGGGGACAG

ACCTCCCCCTCACTCCAGGGAGTTCCTTTCTCCACTTCTTTGCCCTCGGCACACCTCCCC

GGGCAGCCAGGAACCTCCCCCGGGCCAATGCCGGGCAAGGCCTCCCCTCCACAGAGAAAC

>1743 len=161 tot-seq=9

TGGGGAATATTGCGCAATGGGGGAAACCCTGACGCAGCGACGCCGCGTGCGGGATGAAGG

CCTTCGGGTTGTAAAGCTCTGTTATACGGGACGAACGGCAAGGTGGTAAATAGCCATCAT

GAGTGACGGTACCGTAAGAGAAAGCCACGGCTAACTACGTG

>1744 len=135 tot-seq=9

TGGGGAATATTGCACAATGGGGGAAACCCTGATGCAGCGACGCCGCGTGAGTGAAGAAGT

ATTTCGGTATGTAAAGCTCTATCAGCAGGGAAGAAAAAGACGGTACCTGACTAAGAAGCC

CCGGCTAACTACGTG

>1745 len=155 tot-seq=9

TGAGGAATATTGGTCAATGGACGGAAGTCTGAACCAGCCAAGTAGCGTGCAGGATGACGG

CCCTATGGGTTGTAAACTGCTTTTATATGGGAATAAAGTGAGGCACGTGTGGCTTTTTGC

AGGTACCGTATGAATAAGGACCGGCTAATTCCGTG

>1746 len=160 tot-seq=9

TGGGGAATATTGGACAATGGGCGAAAGCCTGATCCAGCCATGCCGCGTGTGTGAAGAAGG

TCTTCGGATTGTAAAGCACTTTAAGTTGGGAGGAAGGGCAGTAACCTAATACGTTGCTGT

TTTGACGTTACCGACAGAATAAGCACCGGCTAACTCTGTG

>1747 len=106 tot-seq=8

ATCACTGGAGGTCAGGAGTTTGATACCAGCCTCGCCAATGTGGTGAAACCCCATCTATAC

TATAAATACAAAAATTAGCCGGGCATGGTGGCGCACGCCTATAATC

>1748 len=107 tot-seq=8

AGGCAGCAGCAGCAGAGGCAGCAGAGGCAGCACCAGCAGCAGCAGAGGCAGCAGAGGCAG

CAGCAGCAGAGGCAGCAGAGGCAGCAGCAGCAGAGGCAGCACCAGCA

>1749 len=109 tot-seq=8

ACACGGGCAGAAATGGGTTAGGGCTATGCATCTCATGGTCCACCCTCAATGACTAGCTTC

CTACCAACAGGCTCTACTTCCTAAAGGTTTCATATGTCCCAGTACATTG

>1750 len=110 tot-seq=8

TGGTGTCGGGAGAACATCTTGGCTCCAGAAGGCTGCGTGCTGGAAACGTACCTCTCTACA

CCCATGCTTGATCTCTGCTGCTGCCTGGGCCAGCAGCTAATCCCGCCTGG

>1751 len=112 tot-seq=8

CAAAGTCTGTGGCAAGGCTGCATCCTCAAGGGGGAGGGCAGGTAGTGGGCAGGTAGAAGA

AGCACATCATCAGATATGCTCCTGAGCACAGGTGAGATACTTTTTGGGACTC

>1752 len=117 tot-seq=8

AGTGAGTGAGAGGGGAGGTCAGGGGAGTCACAGCTTTACCAGATGGACTGGAAATTCCTT

ACTCTCTCCCTTCACTGCGATCGAAGGCGCCCTGGCAAATCCAGGAGACAGCTCCGC

>1753 len=119 tot-seq=8

TACCAAGGACAAACATCTCAGCATCCAATAAACTTGAGAATGTTTCTCTTTTTGTTTGCA

AGGCTGGTTGCTATTTCCTGCGCGCCCCATGTTTATGGACGCGCCGGCTCCTCCGGTGT

>1754 len=135 tot-seq=8

TGGGGAATATTGGACAATGGGGGAAACCCTGATCCAGCAATGCCGCGTGAATGAAGAAGG

CCTTAGGGTTGTAAAGTTCTTTCACCTGTGAAGATAATGACGGTAGCAGGAGAAGAAGCC

CCGGCTAACTCCGTG

>1755 len=135 tot-seq=8

TGGGGAATATTGGGCAATGGGGGAAACCCTGACCCAGCGACGCCGCGTGAGGGAAGAAGG

TTTTCGGATTGTAAACCTCTGTCCTTGGTGACGATAATGACGGTAGCCAAGGAGGAAGCC

ACGGCTAACTACGTG

>1756 len=135 tot-seq=8

TGGGGAATATTGCACAATGGGGGAAACCCTGATGCAGCGACGCCGCGTGAGTGAAGACGG

CCCTCGGGTTGTAAAGCTCTGTGATAGGGGACGATAATGACGGTACCCTGTCAGAAAGCC

ACGGCTAACTACGTG

>1757 len=135 tot-seq=8

TGGGGAATTTTGCACAATGGAGGCAACTCTGATGCAGCGACGCCGCGTGAAGGATGAAGG

TTTTCGGATTGTAAACTTCTGTCCTTGGAGATGATAATGACAGTATCCGAGGAGGAAGCC

CCGGCTAACTACGTG

>1758 len=135 tot-seq=8

TAGGGAATCTTCGGCAATGGACGGAAGTCTGACCGAGCAACGCCGCGTGAGTGAAGAAGG

TCTTCGGATCGTAAAGTTCTGTTGCAGGGGAAGATAATGACGGTACCCTGTGAGGAAGCC

CCGGCTAACTACGTG

>1759 len=135 tot-seq=8

TGAGGAATATTGGTCAATGGGCGAGAGCCTGAACCAGCCAAGTCGCGTGAACGATGAAGG

TCTTCGGATCGTAAAGTTCTGTTGCAGGGGAAGATAATGACGGTACCCTGTGAGGAAGCC

CCGGCTAACTACGTG

>1760 len=135 tot-seq=8

TAGGGAATATTGCACAATGGGGGGAACCCTGATGCAGCGACGCCGCGTGAGTGAAGAAGT

ATATCGGTATGTAAAGCTCTATCAGCAGGGAAGAAGATGACGGTACCTGAGTAAGAAGCC

CCGGCTAACTACGTG

>1761 len=135 tot-seq=8

TGGGGGATATTGCACAATGGGCGAAAGCCTGATGCAGCGACGCCGCGTGAGGGAAGACGG

TCTTCGGATTGTAAACCACTGTCTTCGGGGACGATAATGACGGTACCCGAGGAGGAAGCT

CCGGCTAACTACGTG

>1762 len=135 tot-seq=8

TGGGGAATATTGCACAATGGGGGGAACCCTGATGCAGCGACGCCGCGTGAATGAAGAAGG

TCTTCGGATCGTAAAGTTCTGTCGCGAGGGACGATAGTGACGGTACCTCGTGAGGAAGCC

ACGGCTAACTACGTG

>1763 len=135 tot-seq=8

TGGGGAATATTGGACAATGGGCGCAAGCCTGATCCAGCCATGCCGCGTGGATGATGAAGG

CCTTAGGGTTGTAAAGTCCTTTTAACGGGGAAGATAATGACGGTACCCGTAGAATAAGCC

CCGGCTAACTTCGTG

>1764 len=135 tot-seq=8

TGGGGAATCTTCCGCAATGGACGAAAGTCTGACGGAGCAACGCCGCGTGAGTGAAGACGG

TCTTCGGATTGTAAAGCTCTGTTGCAGGGGAAGATAATGACGGTACCCTGTGAGGAAGCC

CCGGCTAACTACGTG

>1765 len=136 tot-seq=8

TGGGGAATATTGGGCAATGGAGGAAACTCTGACCCAGCAACGCCGCGTGAAGGATGAAGG

TCTTCGGATTGTAAACTTCTGTTGTTAGGGACGAAGAATGACGGTACCTAACGAGAAAGC

CCCGGCTAACTACGTG

>1766 len=136 tot-seq=8

TCGGGAATTTTGCGCAATGGAGGAAACTCTGACGCAGTGACGCCGCGTATAGGAAGAAGG

TTTTCGGATTGTAAACTATTTTAGTCAGGGAAGAAAAATGACAGTACCTGAAGAATAAGC

TCCGGCTAACTACGTG

>1767 len=136 tot-seq=8

TGGGGAATCTTGCGCAATGGGCGAAAGCCTGACGCAGCAACGCCGCGTGGGGGATGAAGG

CTTTCGGGTTGTAAACCCCTTTCAGCAGGGACGAATCAAGACGGTACCTGCAGAAGAAGC

CCCGGCTAACTACGTG

>1768 len=136 tot-seq=8

TGGGGAATATTGGGCAATGGGCGCAAGCCTGACCCAGCAACGCCGCGTGAAGGATGAAGG

TTTTCGGATTGTAAACTTCTGTCGCGAGGGACGAAGAATGACGGTACCTCGTAAGAAAGC

CCCGGCTAACTACGTG

>1769 len=138 tot-seq=8

TAGGGAATCTTCCACAATGGACGCAAGTCTGATGGAGCAACGCCGCGTGAGTGAAGAAGG

GTTTCGGGTTGTAAAGCACTTTCGGAGGGGAGGAAAAAAATGACGTTACCCTCAGAAGAA

GCACCGGCTAACTCCGTG

>1770 len=138 tot-seq=8

TAGGGAATCTTGCACAATGGGCGAAAGCCTGATGCAGCAACGCCGCGTGAAGGATGAAGG

CCTTCGGGTCGTAAACTTCCGTTCTTAGGGAAGAAAAAAATGACGGTACCTAAGGAGGAA

GCCCCGGCTAACTACGTG

>1771 len=138 tot-seq=8

TGGGGAATATTGGGCAATGGGCGAAAGCCTTACCCAGCAATGCCGCGTGAGTGAAGAAGG
TCTTCGGATTGTAAAGCTCTTTGATCAGGGACGAACACAATGACGGTACCTGAAGAACAA
GCCCCGGCTAACTACGTG
>1772 len=139 tot-seq=8
TGGGGAATATTGGGCAATGGAGGCAACTCTTACCCAGCAATGCCGCGTGAATGAAGAAGG
TCTTCGGATTGTAAAGTTCTTTAATTGGGGACGAAAAAAAATGACGGTACCCAAGGAATA
AGCCCCGGCTAACTACGTG
>1773 len=139 tot-seq=8
TAGAAATGTATGCAAATGGGTGGATTGGGAAGCTGGGAGAATGGTACCTATATAATGAAT
TAATATAAGGGGAACAAAAATGAGAAACTGTGAAGTTACTAATTTATATACAGCCAAATA
ATCTGTATTATGCAATAAC
>1774 len=142 tot-seq=8
TGAGGAATATTGCACAATGGGCGCAAGCCTGATGCAGCGACGCCGCGTGAGGGATGGAGG
CCTTCGGGTTGTAAACCGCTTTCAGCAGGGACGAGGCCGCAAGGTGACGGTACCTGCAGA
AGAAGCCCCGGCTAACTACGTG
>1775 len=142 tot-seq=8
GGATCCACGGCCAAATCCTGGGAAAAGAGCCCATGAATGGGCATCTCAGAGTGGCCACCT
TCTGACTGCCCTCCCTCTTCCCTTTGCCTACATTCTGCCTGAAGGGAAGGAGGGGCAGCT
CCAGGGGAGGTAAACTGCAGTC
>1776 len=143 tot-seq=8
TGGGGAATATTGCACAATGGGCGCAAGCCTGATGCAGCAACGCCGCGTGAACGATGAAGG
TCTTCGGGTTGTAAACCGCTTTTGATTGGGAGCAAGCCTTTTGGGTGAGTGTACCTTTCG
AATAAGCGTCGGCTAACTACGTG
>1777 len=143 tot-seq=8
TGACGGAGCAGGAAGGAGAGGACAGAAGCAAGGGCCCCTTGGAGGGGAGAACTGGAGGGG
CTGGGAAGCTCCTGGAATATTGGTGGCTGGGAGGTGGGAGAGATCAGACGGCTTGAATTT
CATAGCTGAGATATGATGGTGCT
>1778 len=143 tot-seq=8
CAGTGGGGAATATTGGACAATGGGCGGAAGCCTGATCCAGCAACGCCGCGTGAGGGATGA

CTGCCTTCGGGTTGTAAACCTCTTTCAGTACCGACGAAGCGAGAGTGACGGTAGGTACAG
AAGAAGCACCGGCCAACTACGTG
>1779 len=144 tot-seq=8
TGGGGAATATTGCACAATGGGCGCAAGCCTGATGCAGCGACGCCGCGTGAGGGATGACGG
CCTTCGGGTTGTAAACCTCTTTCAGTAGGGAAGAAGGCCTTCGGGTTGACGGTACCTGCA
GAAGAAGCGCCGGCTAACTACGTG
>1780 len=144 tot-seq=8
CAACAGAGGCCCTGAGAAGGAACCCAGACCCAGCACCAGCGGAGATGCACATATTCCGCC
TCACATGGAATCAGAAGCAACGCCCCTACTTTACAGCTGAGAACACTGAGGCTCCAAGAG
GTTCTGGGATGTGCTCTACGTCAC
>1781 len=146 tot-seq=8
TGGGGAATATTGCACAATGGGCGCAAGCCTGATGCAGCGACGCCGCGTGGGGGATGAAGG
CCTTCGGGTTGTAAACTCCTTTCGCTAGGGACGAAGCCTTTTTTAAGGTGACGGTACCTG
GATAAGAAGCACCGGCTAACTACGTG
>1782 len=150 tot-seq=8
GGGAGGCAGCGGTGGGGAATATTGCACAATGGGCGCAAGCCTGATGCAGCAACGCCGCGT
GAGCGAGGAAGGCCTTTGGGTCGTAAAGCTCTGTCCTTGGGGAAGAAACAAATGACGGTA
CCCTTGGAGGAAGCCCCGGCTAACTACGTG
>1783 len=151 tot-seq=8
TTTATCCGTGACTGGAGTTCAGACGTGTGCTCTCCCGACCCGGGGAGGTAGTGACGAAAA
ATAACAATACAGGACTCTTTCGAGGCCCTGTAATTGGAATGAGTCCACTTTAAATCCTTT
AACGAGGATCCATTGGAGGGCAAGTCTGGTG
>1784 len=153 tot-seq=8
GCCCAGTGCTTCAGGGCAGCTCTCCTTGTGGCCGCCTGACGCCAGGACGGACTTGGGCCA
CCAGTGCCAGAGGAGAGAGAAGCGGCACCAGAGACAAGGCGATGCTTCAAAATCATCTGT
TCTCAGTTGCTGACTCAGACGCCTCCTTCTGTG
>1785 len=153 tot-seq=8
TGGGGAATATTGCGCAATGGGGGAAACCCTGATGCAGCGACGCCGCGTGAGTGAAGAAGT
ATTTCGGTATGTAAAGCTCTATCAGCAGGGAAGAAATACTGACCATAGGGTCAGCAGACG

GTACTTGACTAAGAAGCCCCGGCTAACTACGTG

>1786 len=153 tot-seq=8

GGGAGGGCAGCAGTGGGGAATATTGCACAATGGGCGCAAGCCTGATGCAGCGACGCCGCG

TGGGGGATGACGGCCTTCGGGTTGTAAACCTCTTTCGACAGGGACGAAGCTTTGGTGACG

GTACCTGTATAAGAAGCACCGGCTAACTACGTG

>1787 len=153 tot-seq=8

GGTCAGGACCACCACTCAGTGGTATAGGACACTGTGTGAATTTCAAAAAGATTCCCCTTC

CTGAGACCCTCTGACCCCAGCTGGATGTTTTGTGCAGTGAACAGCCTGCCCCGCCATCT

AAGGCAGCCAAAGCAAGCTCTGTGTTGGTATCT

>1788 len=155 tot-seq=8

TGAGGAATATTGGTCAATGGGCGGAAGCCTGAACCAGCCAAGTAGCGTGAGGGATGACTG

CCCTATGGGTTGTAAACCTCTTTTATAAGGGAATAAAATACGGGACGTGTCCTGTTTTGC

ATGTACCTTATGAATAAGGACCGGCTAATTCCGTG

>1789 len=155 tot-seq=8

TGAGGAATATTGGTCAATGGGCGAGAGCCTGAACCAGCCAAGTAGCGTGAGGGACGACTG

CCCTATGGGTTGTAAACCTCTTTTGTTTGGGAATAAAGTGCGGGACGTGTCCCGCATTGC

ATGTACCATTTGAATAAGGACCGGCTAATTCCATG

>1790 len=155 tot-seq=8

TGGGGAATATTGCGCAATGGGGGAAACCCTGACGCAGCGACGCCGCGTGCAGGATGACGG

CCCTATGGGTTGTAAACTGCTTTTGTATGGGGATAAAGTTAGGGACGTGTCCCTATTTGC

AGGTACCATACGAATAAGGACCGGCTAATTCCGTG

>1791 len=155 tot-seq=8

TGAGGAATATTGGTCAATGGGCGAGAGCCTGAACCAGCCAAGTCGCGTGAAGGAAGACGG

TCCTACGGATTGTAAACTTCTTTTGTACAGGAATAACAGGTTTTACGTGTAGAATTATGC

ATGTACTGTACGAATAAGCATCGGCTAACTCCGTG

>1792 len=155 tot-seq=8

TGAGGAATATTGGTCAATGGGCGAGAGCCTGAACCAGCCAAGTCGCGTGAAGGAAGACGG

CCCTATGGGTTGTAAACTTCTTTTGCAGGGGAATAAAGATATCCACGAGTGGATAGTTGT

ATGTACCCTGCGAATAAGCATCGGCTAACTCCGTG

>1793 len=155 tot-seq=8

TGAGGAATATTGGTCAATGGAGGCAACTCTGAACCAGCCAAGTCGCGTGAAGGACGACGG

TCCTAAGGATTGTAAACTTCTTTAGTAGGTGAGTAGAGACAGGTACGTGTACCTGTAGGC

AAGTAACCTAAGAATAAGTATCGGCTAACTCCGTG

>1794 len=155 tot-seq=8

TGAGGAATATTGGTCAATGGGCGGAAGCCTGAACCAGCCAAGTAGCGTGAAGGAAGACTG

CCCTATGGGTTGTAAACTTCTTTTATGCGGGGATAAAGTTCGCCACGTGTGGCGTTTTGC

AGGTACCGCATGAATAAGGACCGGCTAATTCCGTG

>1795 len=155 tot-seq=8

TAAGGAATATTGGACAATGGAGGCAACTCTGATCCAGCCATGCCGCGTGAAGGATGAAGG

TCTTCTGGATTGTAAACTTCTTTTATGGGGGGCGAAAACTGTTTTTTTAAGACAATTGA

CGGTACCTTATGAATAAACACCGGCTAACTCCGTG

>1796 len=155 tot-seq=8

TGGGGAATATTGGTCAATGGACGCAAGTCTGAACCAGCCAAGTAGCGTGCAGGATGACGG

CCCTATGGGTTGTAAACTGCTTTTATGTGGGGATAAAGTGCATGACGTGTCATGCATTGC

AGGTACCACATGAATAAGGACCGGCTAATTCCGTG

>1797 len=155 tot-seq=8

TGAGGAATATTGGTCAATGGACGAGAGTCTGAACCAGCCAAGTAGCGTGAAGGATGACTG

CCCTATGGGTTGTAAACTTCTTTTATATGGGAATAAAGTGCAGTATGTATACTGTTTTGT

ATGTACCATACGAATAAGGATCGGCTAACTCCGTG

>1798 len=155 tot-seq=8

TAAGGAATATTGGTCAATGGAGGGAACTCTGAACCAGCCATGCCGCGTGCAGGAAGACAG

CCCTCTGGGTCGTAAACTGCTTTTATTCGGGAATAAACCTTTCTACGTGTAGGAAGCTGA

ATGTACCGAAGGAATAAGGATCGGCTAACTCCGTG

>1799 len=155 tot-seq=8

TGAGGAGTATTGGTCAATGGACGCAAGTCTGAACCAGCCATGCCGCGTGCAGGAAGACGG

CTCTATGAGTTGTAAACTGCTTTTGTACGAGGGTAAGCTCACCTACGTGTAGGTGACTGA

AAGTATCGTACGAATAAGGATCGGCTAACTCCGTG

>1800 len=156 tot-seq=8

TGAGGAATATTGGTCAATGGGCGGGAGCCTGAACCAGCCAAGTCGCGTGAGGGAAGACGG

TCCTATGGATTGTAAACCTCTTTTGTCAGGGAGCAATTCCCGCCACGCGTGGCGGGAGGG

AGAGTACCTGAAGAAAAAGCATCGGCTAACTCCGTG

>1801 len=156 tot-seq=8

TGAGGAATATTGGTCAATGGGCGGAAGCCTGAACCAGCCAAGTCGCGTGAGGGAAGACGG

CCCTACGGGTTGTAAACCTCTTTTGCCGGGGAGCAATGCCCAGCTCGCGAGCTGGGAAGG

AGAGTACCCGGAGAAAAAGCATCGGCTAACTCCGTG

>1802 len=156 tot-seq=8

TGAGGAATATTGGTCAATGGGCGCAAGCCTGAACCAGCCAAGTCGCGTGAGGGAAGACGG

TCCTATGGATTGTAAACCTCTTTTGCCGGGGAGCAAGGCATCTCTCGTGAGGGATGTCGG

AGAGTACCCGGAGAAAAAGCATCGGCTAACTCCGTG

>1803 len=158 tot-seq=8

TTCTCAAAAGCCTCAGTCATGTGGCCCCGTAGATTATTGGCTATCCTAGCTCGGCTAGAT

TGGCCAAATATGGTGATGTTTGCCTGGGAAAAGCACAGGTAGATATTGCCCTTACGCAGC

AGCTAAGCCTAATCAACAGTGTAGGTGAGCAGGTAGTA

>1804 len=159 tot-seq=8

TGGGGAATATTGGACAATGGGCGAAAGCCTGATCCAGCAATACCGCGTGTGTGAAGAAGG

CCTTCGGGTTGTAAAGCACTTTAAGTTAGGAAGAAAAAGCTTCACCTAATACGTGAAGCC

TTGACGGTACCAACAGAATAAGCACCGGCTAACTCTGTG

>1805 len=159 tot-seq=8

TAGGGAATCTTCCGCAATGGACGCAAGTCTGACGGAGCAACGCCGCGTGAGTGACGAAGG

TTTTCGGATCGTAAAACTCTGTTGTGAGGGAAGAACAAGTACCAAGTAACTACTGGTACC

TTGACGGTACCTCACCAGAAAGCCACGGCTAACTACGTG

>1806 len=159 tot-seq=8

CGGGGAATTGTTCACAATGGGCGCAAGCCTGATGACGCAACGCCGCGTGGAGGATGAAGG

TCTTCGGATTGTAAACTCCTGTTGCTCGGGACGAAAAGCCTCGACCCAATACGTCGAGGT

CTGACGGTACCGAGTGAGGAAGCCCCGGCTAACTCTGTG

>1807 len=159 tot-seq=8

TGGGGAATTGTTCGCAATGGGCGCAAGCCTGACGACGCAACGCCGCGTGGAGGATGAAGG

TCTTCGGATTGTAAACTCCTGTTGCTCGGGACGAAAAGCTTCGACCTGACACGTCGAAGT

CTGACGGTACCGAGTGAGGAAGCCCCGGCTAACTCTGTG

>1808 len=160 tot-seq=8

TAGGGAATCTTCGGCAATGGACGGAAGTCTGACCGAGCAACGCCGCGTGAGTGAAGAAGG

GTTTCGGCTCGTAAAACTCTGTTGTTGAAGAAGAACGTGCGTGAGAGTAACTGTTCACGT

TTCGACGGTATCTAACCAGAAAGCCACGGCTAACTACGTG

>1809 len=160 tot-seq=8

TGGGGAATATTGCACAATGGGGGAAACCCTGATGCAGCGACGCCGCGTGAACGAAGAAGG

TATTCGTATCGTAAAGTACTTTCAGCGGGGAGGAAGGGAGTAAAGTTAATACCTTTGCTC

ATTGACGTTACCCGCAGAAGAAGCACCGGCTAACTCCGTG

>1810 len=160 tot-seq=8

CGAGGAATATTGGTCAATGGGCGCAAGCCTGATGCAGCCATGCCGCGTGTATGAAGAAGG

CCTTCGGGTTGTAAAGTACTTTCAGCGGGGAGGAAGGGAGTAAAGTTAATACCTTTACTC

ATTGACGTTACCCGCAGAAGAAGCACCGGCTAACTCCGTG

>1811 len=160 tot-seq=8

TGGGGAATTTTGCACAATGGGGGAAACCCTGATGCAGCGACGCCGCGTGATTTAGAAGGC

CTTCGGGTTGTAAAGCTCTGTTGTTAAGGAAGAACGAGTGTGAGAATGGAAAGTTCATGC

TGTGACGGTACTTAACCAGAAAGGGACGGCTAACTACGTG

>1812 len=160 tot-seq=8

TGGGGAATCTTCCGCAATGGGCGAAAGCCTGACGGAGCAATGCCGCGTGAACGAAGAAGG

CCTTCGGGTTGTAAAGTTCTGTCCTAGCCGAAGAGAGAGGAAGGGAGTAACTGCCCTGCC

TAGGACGGTAGGCTAGGAGGAAGCCCCGGCTAACTACGTG

>1813 len=160 tot-seq=8

TGGGGAATTTTGGACAATGGGCGCAAGCCCGATCCAGCAATGTCGCGTGAGTGAAGAAGG

CCTTCGGGTTGTAAAGCTCTTTTGTCAGGGAAGAAACGGTTGGGGCTAATATCCTCGGCT

AATGACGGTACCTGAAGAATAAGCACCGGCTAACTACGTG

>1814 len=160 tot-seq=8

TGGGGAATTTTGGACAATGGGGGCAACCCTGATCCAGCCATGCCGCGTGCACGATGAAGG

TCTTCGGATTGTAAAGTGCTGTTATAAGGGAAGAACATTTGCAATAGGAAATGATTGCAG

ACTGACGGTACCTTGTCAGAAAGCGATGGCTAACTATGTG

>1815 len=160 tot-seq=8

TGGGGAATATTGCGCAATGGGCGAAAGCCTGACGCAGCGACGCCGCGTGAGGGATGAAGG

TCTTCGGATCGTAAACCTCTGTCAGCAGGGAAGAACGGTCACTGTGCTAATCAGCAGTGA

ATTGACGGTACCTGCAAAGGAAGCACCGGCTAACTCCGTG

>1816 len=160 tot-seq=8

TGGGGAATTTTGGACAATGGGCGAAAGCCTGATCCAGCAATGCCGCGTGCAGGATGAAGG

CCCTCGGGTTGTAAACTGCTTTTGTACGGAACGAAAAGCCTGGGGCTAATATCCCCGGGT

CATGACGGTACCGTAAGAATAAGCACCGGCTAACTACGTG

>1817 len=160 tot-seq=8

TGGGGAATCTTGGACAATGGGGGCAACCCTGATCCAGCGATGCCGCGTGTGTGAAGAAGG

CCTTCGGGTTGTAAAGCACTTTAGGTGGGGAAGAAAGGCAGTTGTTGAATACGCGACTGT

TATGACGGTACCCGCAGAATAAGCACCGGCTAACTCTGTG

>1818 len=161 tot-seq=8

TGGGGAATATTGCACAATGGGCGCAAGCCTGATGCAGCAACGCCGCGTGAACGATGAAGG

TCTTCGGATCGTAAAGTTCTGTTATACGGGACGAATGGCGTAGCGGTCAATACCCGTTAC

GAGTGACGGTACCGTAAGAGAAAGCCACGGCTAACTACGTG

>1819 len=161 tot-seq=8

TGAGGAATATTGGTCAATGGACGAAAGTCTGACCGAGCAACGCCGCGTGAGTGAAGAAGG

TTTTCGGATCGTAAAACTCTGTTGGTAGAGAAGAACGTTGGTGAGAGTGGAAAGCTCATC

AAGTGACGGTAACTACCCAGAAAGGGACGGCTAACTACGTG

>1820 len=161 tot-seq=8

TAGGGAATCTTCCGCAATGGGCGAAAGCCTGACGGAGCAACGCCGCGTGAGTGATGAAGG

TCTTCGGATCGTAAAACTCTGTTATTAGGGAGGAACGAGTGTGAGAATGGAAAGTTCATA

CTGTGACGGTACTTAACCAGAAAGGGACGGCTAACTACGTG

>1821 len=161 tot-seq=8

TAGGGAATCTTCCACAATGGACGCAAGTCTGATGGAGCAACGCCGCGTGAATGAAGAAGG

GTTTCGGCTCGTAAAGCTCTGTTGTAAGAGAAGAACGAGTGTGAGAGTGGAAAGTTCACA

CTGTGACGGTATCTTACCAGAAAGGGACGGCTAACTACGTG

>1822 len=161 tot-seq=8

TGGGGAATATTGCACAATGGGCGCAAGCCTGATGCAGCAACGCCGCGTGAACGATGAAGG

TCTTCGGGTCGTAAAGTTCTGTTGTAAGAGAAGAACGAGTGTGAGAGTGGAAAGTTCACA

CTGTGACGGTATCTTACCAGAAAGGGACGGCTAACTACGTG

>1823 len=161 tot-seq=8

TGGGGAATCTTCCGCAATAGACGAAAGTCTGACGGAGCAACGCCGCGTGAGTGATGAAGG

TCTTCGGATTGTAAAACTCTGTCGTCAGGGACGAATGTACTGATTTACAATACACTTCGG

TATTGACGGTACCTGACGAGGAAGCCACGGCTAACTACGTG

>1824 len=161 tot-seq=8

TGGGGAATATTGCGCAATGGGGGAAACCCTGACGCAGCGACGCCGCGTGCGGGATGAAGG

TTTTCGGATCGTAAAGCTCTGTTGTTAGAGAAGAACGTTGGTAGGAGTGGAAAATCTACC

AAGTGACGGTAACTAACCAGAAAGGGACGGCTAACTACGTG

>1825 len=163 tot-seq=8

GGGTGGACTAGCAAATCTGTGGGCACCAGCAGGAAGAACGCCAATTTGCTGGTCCCTGGA

CCTTCCACTGCCTATCTCTACTTAGAGCTTTAGAAACCTCTGTTTAGCAGGCCTCAGCAC

CACAGCAGACTACTCAAGAGACTCAAGGCCTCAACCTCAGACT

>1826 len=164 tot-seq=8

TAGTTCAGAATCAAAAACCTCCCTGGCTCTCCAATCCTTCCAGTATGACACTCTGGTCTC

CTTCTCTATCCAGTGCACCAGCAGCAGTGGTGGAGAGCCAGAAACCTTCCTGGCTCACCA

AGCCTCCCAGCGTGACCCTGTGGCCTCCCGCTCCACCCAGAGCA

>1827 len=173 tot-seq=8

AGGTAGAGGTAGCAGGTGTGTTTCAGGTACGGACGCTCGTTGAAGTGACGCTCGAAGCTC

CGTGAGAGGAAACTCATGTCGTCCTTCTGAAGCTCCGGCTTGTAGCGTTCCTTGATAAAC

CAGTCCTGCTTGTGAACGACGGAGTAGTCCGGCAGCACCTTGATTGCCTTGCA

>1828 len=179 tot-seq=8

TCTTCTTGGGTCAACTGTACCTTGTTAGAGGTGTGGATAAAGCACTTATGGTCTTTACTC

TTTCATTAGTTTGAGGGTGGCAAGGGCAGTTCTACTACAGAGGCAGTGGCAGAGAGGCTT

TCAGTTGCCCATAGGGTCTCTGTCCAGGAAGTTGCAAAGCTTCCACTGGTTTGATAGCC

>1829 len=183 tot-seq=8

ATGGAGAACTCTGTGGTCAGGGGCCAAGGCCTCCTGGACAACGTCAAAGACGGCAGCTAC

CGGCTGGCCAATGCAAGATCTACCCTTTGCCGGAAAAACGTCCCGTGAAGAAGCTCACGG

GGACGTCGGTGCCTCAGCCCTCCTGCATGATCAACAGCATGCTGGCTGAGATCTGCCGCC

AGA

>1830 len=185 tot-seq=8

TAAGATATAATAAGGGAGGAAGCAGTAGGGAATATTACACAATGGACGCAAGTCTGATGG

AGCAACGCCGCGTGAGTGAAGAAGGTTTTCGGATAGTAAAGATATGTTGTTGGTGAAGAA

GGATAGAGGTAGTAAATGGAATTTATTTGAAGGTAATCAACCAGAAAGTCACGGCTAACT

ACGTG

>1831 len=196 tot-seq=8

TCCCAGTGCCATTTGGAGTTCAGTCACAGGAAGAACTTGCAAATGACAGCACAGCACTGG

GCACTTGACCAGGTGGGGGACCCTGTATCCCTGAGGTGGCTCAGGGGACAGGACAGAGGC

ATGACCTTGGATCCGGGAATGGGGAGCAGAGCACAGCAGGACAAACCCAAACCCATCTCC

ATCGTTAAGAAAATCC

>1832 len=203 tot-seq=8

CGAAAGGCGAAGAACATGGTGGGCAGGAGGATGAGGTATAGTTCCAGGGCCAGAAATATA

AAGCACTTGTTTATGTGAAGGTAAACTGGGGAGGAGAATCAGAAAGATTACTGCAGTTAC

CCACCACCCCACCCTTCACCCCTCTCGCTCTCTAGCTCTCTCTCTCCTTTCACTCATACC

AAGAGGAGCCCAACCTAGGTACT

>1833 len=204 tot-seq=8

GAGAAGACGCCAGAGGATAACAAGAGAAGATAGGCTGAGTGCTGCTCACTCACACACTCA

CTCAGGTCGGCCGCCCAGGCCTCTCAACAAGCCACATGGTCAGTTCGAGCATCTTCCTCC

CAATTGATAGATGACGCAGAGGAGAGGGTTGCTAGGACCTGCCCACGTCACACAGCCAGT

CATTGGCAAAGCTGGAGTTTGAAG

>1834 len=209 tot-seq=8

TCCGATCTCCTACGGGAAGCAGCAGGCATAAAATAAACACCCGAACCAATGGCCTTCTGT

CCTCTAATGGCTTTCTGTCCTCTCTGCCCAAGAGAGGTAACATTAGGCACTGGCCTAATC

ACGACTTCCTAATGTGCACAACCTTAGAAGCAGTGGGACCCCATTTCTGAGGACTCTCGT

CTTTCTCTTTCAATCCTTCCTTGATCACC

>1835 len=215 tot-seq=8

ATGTGAACAGCTTTTCCCAGCAGGAAAATTGCCTTCTGAAGGAAATGCACAGATGCAAAC

TCACAAGGCTCCAAACCCAAAGGCTCTGCAAGGAGAACCCTTGGTCCCCAAGCAGTCCAC

AATAGACACGCGAGGGTGAGCACACTGTCCCTTTTTATGCCAGCCTTCTCCTTAGGAAGT

GGCAGCAAGGGCGCCCGCAGGAGCTAACCCAGAGA

>1836 len=218 tot-seq=8

GAGTGTAAACTAATCTGGTTGTTACTTTGGTCCTGTGATGAGCAGATGCCCAGGGGAAAA

AGTCAGAATCTCAGAGCTTATGTGTCTCTTCTGTGGTCTTCTCTGCCTCTCTAGGTTTTG

GGTCGTTACCCATCTGGGCCTTCTCACTAGATCTTTCTAGCAGAAGATCTCCTGGTGCAT

AGATTTAAATGTTCTCTCTCTACACACAGCAACCACCA

>1837 len=221 tot-seq=8

AGATTACAAGGTGAATGGAGTTAAGAAGTGTGATATTAAGAGAGAAGAAGGGAGGAAGAA

GTAGGGAATATTAAAAAAGGGAAGAAAGGAGGATGGAGAAAAGAAGAGTGAGTGAAGAAG

GTTTTAGGATAGTAAAGATATGTTGTTGGTGAAGAAGGATAGAGGTAGTAACTGGCCTTT

ATTTGACGGTAATCAACCAGAAAGTCACGGCTAACTACGTG

>1838 len=223 tot-seq=8

TAGAGCATGAGTGGAAGTGCCTGTGAAACCCAACGCCTGGAATTCCGCTCCTGTGATGCC

CACATCCCTGTTCTGGTTTGCCGACTGTCCAGCGGTGAAAGGTGAGACGTGAGCATCGGG

GAAGCTGCCTGAGGGCTGCGGGGACTCTGCAAACTTTCTGTAAATCTAAAATTATTCCAA

GAGAAAAAGCCTATTTAAAAAAAAAAAAATCACCCACACTGAC

>1839 len=224 tot-seq=8

GGTGCGGGCCAGGGCCAGGGCTGGTGCCCGGGGCCGCGCTGTGAGGTGGGCAGGCGAGGA

GCGGGAAGACCATCTCTGCAAGTGCAGCATAGCCTCGGCCTAGGACAGCGGGAGTGCGTG

GCCAAAGCTGTGAGCAGAGGCACAGGTGGTGGCAGACAGTAGAGGCGCCCCATGGGGAAC

ATACTGACCTGTCGTGTGCACCCTAGCGTCAGCCTCGAGTTTGA

>1840 len=230 tot-seq=8

GCTTCCATTTCTGGTGTTTGTGCTGCATCAGCTTTGCTCCTTTGGGACTGGGTTTCCCAA

ACGCTCTTCCCGCCTTGTTCTGAGTGGTGGGAGCCACACAAGACGTCTGACGTTTGAGGA

GATTGGGAAGCAGAAGTGTGGCAGCCCCCACACACCCAAAGCAAGCAGCTACCACAAACG

CACTCGCCCCTCTGGGGTGGGCAGCAGGACGGTCCCCACAGGACCCTCAT

>1841 len=201 tot-seq=8

TGAGGAATATTGGTCAATGGACGAGAGTCTGAACCAGCCAAGTAGCGTGAAGGATGACTG

CCCTATGGGTTGTAAACTTCTTTTATATGGGAATAAAGTGTTCCACGTGTGGGATTTTGT

ATGTACCATATGAATAAGGATCGGCTAACTCCGTGCCAGCAGCCGCGGTAATTGGAAGAT

CGGAAGAGCGTCGTGTAGGGG

>1842 len=135 tot-seq=8

TAGGGAATCTTCCGCAATGGGGGAAACCCTGATGCAGCGACGCCGCGTGAGTGAAGAAGT

ATTTCGGTATGTAAAGCTCTATCAGCAGGGAAGAAAATGACGGTACCTGACTAAGAAGCC

CCGGCTAACTACGTG

>1843 len=103 tot-seq=7

GGGTGGCAGAGGCAGATGAGGCTGGGTGTCTGGGAGAAAGCTGAGCATGCAGGAGACATC

CTTCAGCATCAGGATAAAGACTGAATTAGATTCATTTTAGATC

>1844 len=104 tot-seq=7

CCCCCCCGCCTGTCAGCAGCGAGTCCAGAGGGTGTCCGAGGCCAGAATCCACATGGCCAA

GGCCCCCAGCAGCTCCAGCCGCCCACTGCCCCTCCCCATTTGTG

>1845 len=107 tot-seq=7

CGGGGTGCGGCATCAATTTCCAGTGCTTTGCCTGCCATGTGGGCCCCATCCGTGCTCATG

GTACCGCCCTCCAGCGGAGCGATGGCAGCCAGCTGCCCGTGGAGAGC

>1846 len=110 tot-seq=7

CGTTCCAGGAAGCGAGACGGAGCACAGTACGTTTTAGAAACGATGAGGCTCATCGTGGCT

GGCACAGAGAGACACAGGCAGGGCCTGGCCATGCCTAGTCTGTAGCTTTT

>1847 len=112 tot-seq=7

GAGTGAAGAAGGTTTTCGGATCGTAAAGCTCTGTTGTTGGGGAAGAAGGATAGAGGTAGG

AACTGGCCGTTATGTGACGGTAATCAACCAGAAAGTCACGGCTAACTACGTG

>1848 len=117 tot-seq=7

CACATACAGGCAGGTGTCACACGTAAGGCTGCACAGGAGGCACGGGGTCTGGAGTAACTG

CTTCTGAGACAGAGCATTCTCTGCACACAGAAAGTCAGCTCTGATGGAGAAAGGTAG

>1849 len=122 tot-seq=7

GTGAGAAGACAAAGGGATGTTTTCCTTCATGAGCTAAGCCAGCAGCAACTGTAACTGCAT

TTTGTTCAGCAATTCCCATTTCTACAGTTCTATCAGGATATTTATTTGTGAAAGGAACCA

AG

>1850 len=124 tot-seq=7

GTGGGAGGAGGGGCTGTCCAGTGGGGAGAGGGGATAAAGGGAGGATGGTGCCAAGATGCT

GTGCTCTTTGATGCTGGGGACCAGGCTGGGTCTCAGCATTCGCCAACAACTCCCAGCAAC

ATTC

>1851 len=126 tot-seq=7

CGACTGCAGGAAGACAGTAGGGAGAAGCCTTGAGCAGGGCCACAGGCGGAAGGTCACAGC

TGCCTTCCAGGTGACCAGCAGTTTGGCCGCAGGATAAAAAGTGACTGTTAAGGGGACTGT

GGAGTC

>1852 len=128 tot-seq=7

TCAGGTGGTCCAGGGGCTGGAGGTGAGGGCTTTGCCGCAGGCGCATGCTGCTCTGTGAGA

ACTCCGAGGAATAACTCACTGGAGGGCTATAAATGCTGAAGGTAACCAGGCCAAGGACTC

CAGTGCCC

>1853 len=128 tot-seq=7

TCACAGAGATGGGAGGCAAAGCAGAACTTTCAACTGGGAGAGGGTGGCGAGTGGGCTAAG

TGCTGCAGAGTGGTCTAGGAGGAGCCAGAGGAGTAGCTGCGGAGTCTGGGCGCCTGTGGA

GGGCAGCT

>1854 len=129 tot-seq=7

ACTCTGTAGGAGGGGCTACAGGGGAAGACCAGCGGCCAGCAGAGCTTATGGACAGGGAAC

TCCCAAGGAGGTAAAGACTGGATATCCAGGAGACATCATCTACGAATGCCAACTGTCCCT

CCCGTGGGG

>1855 len=129 tot-seq=7

GCCACGTTTTGGAGCTAGTAAGGGTTAGATGCGTCTGAGCCTCAGGATAAATGGAGAGAT

TAGTAGGAGACAAAGTTGGAATGGCCGCAAGTCCTCAGAATCTTTAATAAAACAGTAGTG

TTCTCGTCC

>1856 len=131 tot-seq=7

GATCCATCCTTGTTCACATGTGCTAACAGCAACAGTAGCAGCAGTGCAGGGGGCTGGAGG

GGTGAAAGGTTGCATGCTCACTGGCTGCAGTGCTAGCAGGTACTGGGGTGCCTGCCTCTG

CACAGGCATTC

>1857 len=134 tot-seq=7

TGGGGAATTTTGCACAATGGGGGCAACCCTGATGCAGCGACGCCGCGTGATTTAGAAGGC

CTTCGAGTTGTAAAAATCTTTTGTATAGGAAGAAAATGACAGTACTATACGAATAAGGTC

CGGCTAATTACGTG

>1858 len=135 tot-seq=7

TGGGGAATTTTGCACAATGGGGGAAACCCTGATGCAGCGACGCCGCGTGAGTGATGAAGG

AATTCGTTTCGTAAAACTCTGTTCTACGGGAAGAAAAGGACTGTACCATAGGAGAAAGCT

CCGGCTAAATACGTG

>1859 len=135 tot-seq=7

TGGGGGATATTGCACAATGGAGGGAACTCTGATGCAGCAATGCCGCGTGAAGGAAGAAGG

TTTTCGGATTGTAAACTTCTATCAGCAGGGAAGAAAATGACGGTACCTGACTAAGAAGCC

CCGGCTAACTATGTG

>1860 len=135 tot-seq=7

TGGGGAATTTTGCACAATGGGCGCAAGCCTGATGCAGCGACGCCGCGTGAGGGATGAAGT

ATTTCGGTATGTAAAGCTCTATCAGAAGGGAAGAAAATGACGGTACCTTACTAAGAAGCT

CCGGCTAAATACGTG

>1861 len=135 tot-seq=7

TGGGGAATCTTAGACAATGGGGGCAACCCTGATCTAGCCATGCCGCGTGATCGATGAAGG

CCTTAGGGTTGTAAAGATCTTTCGCTGGGGAAGATAATGACGGTACCCAGTAAAGAAGCC

CCGGCTAACTCCGTG

>1862 len=135 tot-seq=7

TAGGGAATCTTCGGCAATGGGGGAAACCCTGATGCAGCGACGCCGCGTGAGCGAAGAAGT

ATTTCGGTATGTAAAGCTCTATCAGCAGGGAAGAAAATGACGGTACCTGACTAGGAAGCA

CCGGCTAAATACGTG

>1863 len=135 tot-seq=7

CAGGGAATTTTGCGCAATGGGCGAAAGCCTGACGCAGCAACGCCGCGTGGGGGATGAAGG

CTCTTGGGTCGTAAACCCCTTTTGCCGGGGAAGATCATGACGGTACCCGGCGAATAAGCC

ACGGCTAACTACGTG

>1864 len=135 tot-seq=7

TGGGGAATACTGCACAATGGACGAAAGTCTGATGCAGCAACGCCGCGTGGAGGATGACAC

ATTTCGGTGCGTAAACTCCTTTTATATGAGAAGATAATGACGGTATTATATGAATAAGCA

CCGGCTAACTCCGTG

>1865 len=136 tot-seq=7

TGGGGAATATTGCACAATGGGGGGAAACCCTGATGCAGCGACGCCGCGTGAGCGATGAAG

TATTTCGGTATGTAAAGCTCTAACAGCAGGGAAGAAGATGACGGTACCTGACTAAGAAGC

CCCGGCTAACTACGTG

>1866 len=136 tot-seq=7

GCGCGCAAATTACCCACTCCCGGCACGGGGAGGTAGTGACGAAAAATAACGATACGGGAC

TCATCCGAGGCCCCGTAATCGGAATGAGAACATTTTAAATCTCTTAACGATTACCCATTA

GAGGGCCAGTCTGGTG

>1867 len=136 tot-seq=7

TGGGGAATATTGCACAATGGAGGAAACTCTGACGCAGTGACGCCGCGTATAGGAAGAAGG

TTTTCGGATTGTAAACTATTGTCATTAGGGAAGATAAAAGACTGTACCTAAGGAGGAAGC

CCCGGCTAACTATGTG

>1868 len=136 tot-seq=7

TGGGGAATATTGCACAATGGGGAAAACCCTGATGCAGCGACGCCGCGTGAAGGAAGAAGT

ATCTCGGTATGTAAACTTCTATCAGCAGGGAAGAATTAGGACGGTACCTGACTAAGAAGC

CCCGGCTAACTACGTG

>1869 len=136 tot-seq=7

GCGCGCAAATTACCCACTGTCAAATCTGTGAGGTAGTAACGAAAAATAACAATACAGAAC

TCTTTCGAGGCTCTGTAATTGGAATGAGTACACTTTAAAACGGTCAATAAGGACCGATTG

GAGGGCAAGTCTGGTG

>1870 len=136 tot-seq=7

TGGGGAATCTTGCGCAATGGGCGAAAGCCTGACGCAGCAACGCCGCGTGCGGGATGAAGG

CCTTCGGGTTGTAAACCGCTTTCAGCAGGGACGAAAACAGACTGTACCTGCAGAAGAAGC

CCCGGCTAACTACGTG

>1871 len=136 tot-seq=7

TGAGGGATCTTGCACAATGGGCGCAAGCCTGATGCAGCGACGCCGCGTGAGCGAAGAAGG

CCTTCGGGTCGTAAAGCTCTTTCTGCGTGACGAGAACGGACGGTAGCGCAGGAAGAAGT

CTCGGCTAACTACGTG

>1872 len=137 tot-seq=7

TCACACTACTATCACGCGAAGCAGTATCATCGGCAGAAGTATGCTCAACGGAAGCACCTT

CTGCAGAAACATGCTCACCTACGCGCAAACCAGTCAAATCTGAACCGCTAAAACCAGCTC

CAAGCAAAGCAGACATG

>1873 len=137 tot-seq=7

TGGGGAATATTGCACAATGGGGGGAACCCTGATGCAGCAACGCCGCGTGAAGGAAGAAGG

GTTTCGGCTCGTAAACTTCTATCGACAGGGAAGAAAGAAATGACGGTACCTGAATAAGAA

GCCCCGGCTAACTACGT

>1874 len=137 tot-seq=7

TGGGGAATATTGCACAATGGGGGGAACCCTGATGCAGCGACGCCGCGTGGGCGAGGAAGT

ATTTCGGTATGTAAAGCCCTATCGGCAGGGAAGAAGAAATGACGGTACCTGAATAAGAAG

CACCGGCTAAATACGTG

>1875 len=138 tot-seq=7

TGGGGAATATTGGGCAATGGAGGCAACTCTGACCCAGCAACGCCGCGTGAATGATGAAGG

TCTTCGGATTGTAAAGTTCTGTGATGGGGGACGAAGAAAGTGACGGTACCCCAATAGCAA

GCTACGGCTAACTACGTG

>1876 len=138 tot-seq=7

TGGGGAATATTGGGCAATGGGCGCAAGCCTGACCCAGCAACGCCGCGTGAAGGAAGAAGG

CTTTCGGGTTGTAAACTTCTTTTATTCGGGACGAAGAAAGTGACGGTATCGAATGAATAA

GCCACGGCTAACTACGTG

>1877 len=138 tot-seq=7

TTAGGAATATTGGGCAATGGAGGAAACTCTGACCCAGCAACGCCGCGTGAAGGAAGAAGG

TTTTCGGATTGTAAACTTCTGATCTTGGGGAAAAAGAAAGTGATGGTACCCAAGGAGAAA

GCCACGGCTAACTACGTG

>1878 len=138 tot-seq=7

TGGGGAATATTGGGCAATGGGCGCAAGCCTGACCCAGCAACGCCGCGTGAGGGAAGAAGG

TTTTCGGATTGTAAACCTCTGTCGCAGGGGACGAAGGAAGTGACGGTACCCTGTAAGAAA

GCCCCGGCTAACTACGTG

>1879 len=138 tot-seq=7

TGGGGAATATTGCACAATGGGGGAAACCCTGATGCAGCAACGCCGCGTGAAGGAAGAAGT

ATTTCGGTATGTAAACTTCTATCGACAGGGGAGAAACAAATGACGGTACCTGAATAAGAA

GCACCGGCTAAATACGTG

>1880 len=138 tot-seq=7

TGGGGAATATTGGGCAATGGGGGAAACCCTGACCCAGCAACGCCGCGTGAAGGAAGAAGG

TTTTCGGATTGTAAACTTCTGTCCTAAGGGAAGAAAGAAATGACGGTACCTTAGGAGGAA

GCCCCGGCTAACTACGTG

>1881 len=138 tot-seq=7

TGAGGAATATTGGTCAATGGGGGGAACCCTGATCCAGCGACGCCGCGTAAGTGAAGAAGT

ATTTCGGTATGTAAAGCTCTATCAGCAGGGAAGAAAGAAATGACGGTACCTGACTAAGAA

GCCCCGGCTAACTACGTG

>1882 len=138 tot-seq=7

TGGGGAATATTGGGCAATGGAGGCAACTCTGACCCAGCAACGCCGCGTGAATGAAGAAGG

CCTTCGGGTTGTAAAGTTCTTTAATTGGGGACGAAGGAAATGACGGTACCCAAAGAATAA

GCCACGGCTAACTACGTG

>1883 len=138 tot-seq=7

TGGGGAATATTGCACAATGGGCGAAAGCCTGATGCAGCAACGCCGCGTGAGCGATGAAGG

CCTTTGGGTCGTAAAGCTCTGTCGGAAGGGAAGAAACAAATGACGGTACCTTCCGAGGAA

GCCCCGGCTAACTACGTG

>1884 len=139 tot-seq=7

TGTATGTAACACAGTTTGAAAACATTCCATTACTATCCATTATTAATTGTGACATCACCA

GTAGTCCCTTTCGGGGACAATCTCTTGTATTCGGGAGAATCCTAGAATCACCTGTGATGT

CACCAGTGTTGTGGTGACA

>1885 len=140 tot-seq=7

TGGGGAATATTGCACAATGGGCGCAGGCCTGATGCAGCGACGCCGCGTGAGGGATGACGG

CCTTCGGGTTGTAAACCTCTTTTGTTAGGGAGCAAGCGTAAGTGAGTGTACCTTTCGAAT

AAGCACCGGCTAACTACGTG

>1886 len=141 tot-seq=7

TCCTACGGAGGCAGCAGGTTCCACTACGAGGGTCATTTTAATTTCAAATATATTGTACTT

ACGTCCTTTTTTCTTCTATTAAGAATAAAAGGTGTTATAACAAACAGTACCATTGCTAAG

GCAGGGTGAGTAAGCCCGATT

>1887 len=142 tot-seq=7

TGGGGAATATTGCACAATGGGCGCAAGCCTGATGCAGCAACGCCGCGTGAACGATGAAGG

TCTTCGGATTGTAAACCGCTTTCAGCAGGGACGAGGCCGCAAGGTGACGGTACCTGCAGA

AGAAGCCCCGGCTAACTACGTG

>1888 len=142 tot-seq=7

TGGGGAATATTGCACAATGGGCGAAAGCCTGATGCAGCGACGCCGCGTGAGGGATGACGG

CCTTCGGGTTGTAAACCTCTTTCAGCTCTGACGAAGCCTTCGGGTGACGGTAGGAGCAGA

AGAAGCACCGGCTAACTACGTG

>1889 len=143 tot-seq=7

TGGGGAATTTTTGGCAATGGGCGAAAGCCTGACCAAGCAACGCCGCGTGGATGATGAAGT

ATCTCGGTACGTAAAATCCTTTCGACGGGGACGAAGCCGCAAGGTTGACGGTACCCGTAG

AAGAAGCCCCGGCTAACTCCGTG

>1890 len=147 tot-seq=7

GTGGGACCTGCCGGAGACCAGGATGGGAGATACCTGGGAGACGCCCAGGAAATGCCCGGG

GCAGTCAGGAGGGCTCCTGCGCACCCTGGTGGCCCCAGGTCACACATGGAACTCATGAGG

AAGTCCCCGCCCAGTGTACCCGACCTC

>1891 len=152 tot-seq=7

GGAAGGGAGGGTCCAGGGAGGCTGCTGGAAAGACTACGATTCTGGGGGCTGGAACTGAGC

TCTGAGGAGCAACAGGAGGGTCCCCAAAGATTCCACTGGGAATTGTTCAGATCTCCACCT

TCCTGGGATGGAGAACATCCACTCACCCAGAA

>1892 len=154 tot-seq=7

TATTAATTTTTCATGACAGGCCTCTGCTACCATGCACTGTAATAGACAAAGGAATTTTGG

ACCCAGAGCCTCTGGAGAACAGGAAATAGATGAGACATTTTGATTGAAGAGTAAATGGCC

CACTAAAATAGCAGTTAATCCTTTTATTATCTTT

>1893 len=154 tot-seq=7

TGGGGAATATTGGACAATGGAGGGAACTCTGATCCAGCAATTCTGTGTGTGTGAAGAAGG

TTTTAGGACTGTAAACTGCTTTTATATGGGAATAAAGTGAGGGACGTGTCCCTTATTGCA

TGTACCATACGAATAAGGACCGGCTAATTCCGTG

>1894 len=155 tot-seq=7

TGAGGAATATTGGTCAATGGGCGGAAGCCTGAACCAGCCAAGTAGCGTGCAGGATGACGG

CCCTATGGGTTGTAAACTGCTTTTGCAGGGGGATAAAGTGAGTCACGTGTGACTTATTGC

AGGTACCCTGCGAATAAGGACCGGCTAATTCCGTG

>1895 len=155 tot-seq=7

TAAGGAATATTGGTCAATGGGGGCAACCCTGAACCAGCCATGCCGCGTGCAGGATGACTG

CCCTATGGGTTGTAAACTGCTTTTGTCGGGGAATAAACCCCACTACGTGTAGTGGGCTGA

ATGTACCCGAAGAATAAGGATCGGCTAACTCCGTG

>1896 len=155 tot-seq=7

TGAGGAATATTGGTCAATGGGCGCGAGCCTGAACCAGCCAAGTCGCGTGAGGGAAGACGG

TCCTATGGATTGTAAACCTCTTTTGTCGGAGAGCAAAAGATCCCACGTGTGGGATTTCGA

GAGTATCCGAAGAAAAAGCATCGGCTAACTCCGTG

>1897 len=155 tot-seq=7

TGAGGAATATTGGTCAATGGACGAGAGTCTGAACCAGCCAAGTAGCGTGCAGGATGACGG

CCCTATGGGTTGTAAACTTCTTTTATAAAGGAATAAAGTCGGGTATGCATACCCGTTTGC

ATGTACTTTATGAATAAGGATCGGCTAACTCCGTG

>1898 len=155 tot-seq=7

TGAGGAATATTGGTCAATGGGCGAGAGCCTGAACCAGCCAAGTCGCGTGAGGGAAGACGG

CCCTACGGGTTGTAAACCTCTTTTGTCAGGGAGCAAGAACGGATACGTGTATCCGAGCGA

GAGTACCTGAAGAAAAAGCATCGGCTAACTCCGTG

>1899 len=155 tot-seq=7

TGAGGAATATTGGTCAATGGGCGGAAGCCTGAACCAGCCAAGTAGCGTGCAGGATGACGG

CCCTATGGGTTGTAAACTGCTTTTATACAGGAATAAAGTTAGCCACGTGTGGTTATTTGT

AGGTACTGTATGAATAAGGACCGGCTAATTCCGTG

>1900 len=155 tot-seq=7

TGAGGAATATTGGACAATGGGTGAGAGCCTGATCCAGCCATCCCGCGTGCAGGAAGACGG

CCCTATGGGTTGTAAACTGCTTTTGTACGAGAATAAACCACCTTTCGTGAAGGGTGTTGA

AGGTACCGTACGAATAAGCACCGGCTAACTCCGTG

>1901 len=156 tot-seq=7

TGAGGAATATTGGTCAATGGGCGCGAGCCTGAACCAGCCAAGTCGCGTGAGGGAAGACGG

CCCTACGGGTTGTAAACCTCTTTTGTCAGGGAGCAACGGCCGCCACGTGTGGCGGAAAAG

AGAGTACCTGAAGAAAAAGCATCGGCTAACTCCGTG

>1902 len=156 tot-seq=7

TGAGGAATATTGGTCAATGGGCGAGAGCCTGAACCAGCCAAGTAGCGTGAAGGATGACGG

CCCTACGGGTTGTAAACTTCTTTTGTGCGGGAATAAAGGAGCCTACGTGTAGGCTTTTGC

ATGTACCGTAACGAATAAGCATCGGCTAACTCCGTA

>1903 len=158 tot-seq=7

TGGGGAATATTGCACAATGGGCGCAAGCCTGATGCAGCGACGCCGCGTGAGGGATGGAGG

CCTTCGGGTTGTGAACCTCTTTCGCCAGTGAAGAAGGTCCTGCTCTTTGTGGTGGGGTTG

ACGGTAGCTGGGTTAATGAAGCGCCGGCTAACTACGTG

>1904 len=159 tot-seq=7

GAACAGATCGGAAGAGCGTCGTGTTGGGGGATATTGCACAATGGGGGGAACCCTGATGCA

GCGACGCCGCGTGAGGGAAGAAGGTTTTCGGATTGTAAACCTCTGTCTTTGGTGACGAGA

GTGACGGTAGCCAAGGAGGAAGCCACGGCTAACTACGTG

>1905 len=160 tot-seq=7

TGGGGAATATTGCGCAATGGGCGAAAGCCTGACGCAGCGACGCCGCGTGAGGGATGAAGG

TCTTCGGATCGTAAACCTCTGTCAGAAGGGAAGAAACTAGGGTGCTCTAATCATCATCCT

ACTGACGGTACCTTCAAAGGAAGCACCGGCTAACTCCGTG

>1906 len=160 tot-seq=7

TAGGGAATCTTCGGCAATGGACGAAAGTCTGACCGAGCAACGCCGCGTGAGCGAAGAAGG

CCTTCGGGTCGTAAAGCTCTGTTGTTAGAGAAGAACATGGGTGAGAGTAACTGTTCACCC

CTTGACGGTATCTAACCAGAAAGCCACGGCTAACTACGTG

>1907 len=160 tot-seq=7

TAGGGAATCTTCCGCAATGGACGAAAGTCTGACGGAGCAACGCCGCGTGAGTGAAGAAGG
TTTTCGGATCGTAAAGCTCTGTTGTTAAGGAAGAACACCATGGATAGGAAATGATCGATG
GATGACGGTACTTAACCAGAAAGCCACGGCTAACTACGTG
>1908 len=160 tot-seq=7
TGGGGAATATTGGACAATGGAGGGAACTCTGATCCAGCAATTCTGTGTGTGTGAAGAAGG
ATTTCGGTTCGTAAAACTCTGTTGTTAGGGAAGAAAAAATGTGTAGTAACTATATACAAA
AGAGACGGTACCTAACCAGAAAGCCACGGCTAACTACGTG
>1909 len=160 tot-seq=7
TGAGGAATATTGGTCAATGGGCGCTAGCCTGAACCAGCCAAGTAGCGTGAAGGATGAAGG
TCTTCGGATTGTAAAGTACTTTCAGCGGGGAGGAAGGGAGTAAAGTTAATACCTTTGCTC
ATTGACGTTACCCGCAGAAGAAGCACCGGCTAACTCCGTG
>1910 len=160 tot-seq=7
TAGGGAATTTTCGGCAATGGGGGGAACCCTGACCGAGCAACGCCGCGTGAGGGAGGAAGT
ATTTCGGTATGTAAACCTCTGTTATAAAGGAAGAACGATGCATGTAGGGAATGACATGCA
AGTGACGGTACTTTATGAGGAAGCCACGGCTAACTACGTG
>1911 len=160 tot-seq=7
TGGGGAATATTGGGCAATGGGCGCAAGCCTGACCCAGCAACGCCGCGTGAGTGATGAAGG
TCTTCGGATCGTAAAACTCTGTTATTAGGGAAGAACAAATGTGTAAGTAACTATGCACGT
CTTGACGGTACCTAATCAGAAAGCCACGGCTAACTACGTG
>1912 len=160 tot-seq=7
TGGGGAATCTTGGACAATGGGCGCAAGCCTGATCCAGCAATGCCGCGTGGGTGAAGAAGG
CCTTCGGGTTGTAAAGCCCTTTCGGCGGGGAAGAAATCGGTCAGGCGAATAGTCTGGCTG
GATGACGGTACCCGCAGAAGAAGCACCGGCTAACTACGTG
>1913 len=160 tot-seq=7
TGGGGAATATTGCACAATGGGGGGAGCCCTGATGCAGCCATGCCGCGTGAATGAAGAAGG
CCTTCGGGTTGTAAAGTTCTTTCGGTATTGAGGAAGGTTGATGTGTTAGTAGCACATCAA
ATTGACGTTAAATACAGAAGAAGCACCGGCTAACTCCGTG
>1914 len=160 tot-seq=7
TGGGGAATATTGCACAATGGGCGCAAGCCTGATGCAGCCATGCCGCGTGTATGAAGAAGG

CCTTAGGGTTGTAAAGTACTTCCAGCGGGGAGGAAGGTGATAAGGTTAATACCCTTATCA

ATTGACGTTACCCGCAGAAGAAGCACCGGCTAACTCCGTG

>1915 len=160 tot-seq=7

TGGGGAATATTGCACAATGGGCGCAAGCCTGACGGAGCAACGCCGCGTGAGTGATGAAGG

TCTTCGGATCGTAAAACTCTGTTATTAGGGAAGAACATATGTGTAAGTAACTGTGCACAT

CTTGACGGTACCTAATCAGAAAGCCACGGCTAACTACGTG

>1916 len=160 tot-seq=7

TAGGGAATCTTCCACAATGGGCGAAAGCCTGATGGAGCAACGCCGCGTGTGTGATGAAGG

CTTTCGGGTCGTAAAGCACTGTTGTATGGGAAGAAATGCTAGGTTAGGGAATGAGCTTAG

TTTGACGGTACCATACCAGAAAGGGACGGCTAAATACGTG

>1917 len=160 tot-seq=7

TAGGGAATCTTCCACAATGGACGAAAGTCTGATGGAGCAACGCCGCGTGAGTGAAGAAGG

ATTTCGGTTCGTAAAACTCTGTTGCAAGGGAAGAACAAGTAGCGTAGTAACTGGCGCTAC

CTTGACGGTACCTTGTTAGAAAGCCACGGCTAACTACGTG

>1918 len=160 tot-seq=7

AATCGCTTGAACCCAGGAGGCGGAGGTTGCAGTGAGCTGAGATTGTACCACTGCATTCCA

GCCTGGGCGACAGAGTGAGACTGTCTCACAAAAAGACAAAAAGAAAGTCACCCTCTTAGG

TTGGTGGACATTAAACAAGACCTCCTTTCTCCATGGGTGG

>1919 len=160 tot-seq=7

TGGGGAATATTGGACAATGGGCGCAGGCCTGATCCAGCCATACCGCGTGGGTGAAGAAGG

CCTTCGGGTTGTAAAGCCCTTTTGTTGGGAAAGAAATCCAGCTGGTTAATACCCGGTTGG

GATGACGGTACCCAAGGAATAAGCACCGGCTAACTTCGTG

>1920 len=160 tot-seq=7

TAGGGAATTTTCGGCAATGGGCGAAAGCCTGACCGAGCAATGCCGCGTGAGTGAAGAAGG

CCTTCGGGTTGTAAAGCTCTGTTGTAAAGGAAGAACGGATGGCATAGGGAATGATGCCAT

TGTGACGGTACTTTACGAGGAAGTCACGGCTAACTACGTG

>1921 len=161 tot-seq=7

TAGGGAATTTTCGTCAATGGGGGGAACCCTGAACGAGCAATGCCGCGTGAGTGAAGAAGG

TTTTCGGATCGTAAAGCTCTGTTGTTAGAGAAGAACGTTGGTAGGAGTGGAAAATCTACC

AAGTGACGGTAACTAACCAGAAAGGGACGGCTAACTACGTG

>1922 len=161 tot-seq=7

TGGGGAATATTGGACAATGGGCGAAAGCCTGATCCAGCCATGCCGCGTGTGTGAAGAAGG

TTTTCGGATCGTAAAGCTCTGTTGTTAAGGAAGAACGAGTGTGAGAATGGAAAGTTCATG

CTGTGACGGTACTTAACCAGAAAGGGACGGCTAACTACGTG

>1923 len=161 tot-seq=7

TAGGGAATATTGCGCAATGGAGGAAACTCTGACGCAGCGACGCCGCGTGGGTGACGAAGG

TCTTCGGATCGTAAAGCCCTGTCGGAAGGGAAGAAAAACGTTATGGCTAACATCCATGAC

GCTTGACGGTACCTTTAAAGGAAGCACCGGCTAACTTCGTG

>1924 len=161 tot-seq=7

TTAGGAATATTCGTCAATGGGGGAAACCCTGAACGAGCAATGCCGCGTGAAGGAAGAAGG

TCCTCTGGATTGTAAACTTCTGTTGTAAGGGAAAAACGACTAAATTAGGAAATGAGTTTA

GAGTGATGGTACCTTACGAGAAAGCTCCGGCTAACTACGTG

>1925 len=161 tot-seq=7

CTAAGAATCTTCCGCAATGGACGAAAGTCTGACGGAGCGACGCCGTGTGAATGAAGAAGG

CCGAAAGGTTGTAAAATTCTTTTGCAGATGAGGAATAAGGGGAAGAGGGAATGCTTCTCT

GATGACGGTAGTCATGCGAATAAGCCCCGGCTAATTACGTG

>1926 len=161 tot-seq=7

TGGGGAATATTGCACAATGGGCGAAAGTCTGACGGAGCAACGCCGCGTGAGTGATGACGG

CCTTCGGGTTGTAAAGCTCTGTTAATCGGGACGAATGGTCTTTGTGTGAATAATGCAAAG

ATTTGACGGTACCGGAATAGAAAGCCACGGCTAACTACGTG

>1927 len=161 tot-seq=7

TGGGGAATATTGCACAATGGGCGCAAGCCTGATGCAGCGACGCCGCGTGAGTGATGACGG

CCTTCGGGTTGTAAAGCTCTGTTAATCGGGACGAATGGTCTTTGTGTAAATAATGCAAAG

ATTTGACGGTACCGGAATAGAAAGCCACGGCTAACTACGTG

>1928 len=161 tot-seq=7

TAGGGAATCTTCGGCAATGGGGGGAACCCTGACCGAGCAACGCCGCGTGAGTGATGACGG

TCTTCGGATTGTAAAGCTCTGTTAATCGGGACGAATGGTTTGTGTGCAAATAGTGCATAG

ACATGACGGTACCGGAATAGAAAGCCACGGCTAACTACGTG

>1929 len=161 tot-seq=7

CATGGGGAAAGGACGCGCGGGCCGAGTTGGCACCACAGGTAAACAGGCTGGCGAGGCGCA

GGACGCTGTCGCCGCCGCGGCCACCCAGCGATTTCCGGGCACGCAACTCCGCCTCCAGGG

CTCTCTCCCTCGCGCTCTCTCCCAGCCGCTTGCTCGCAGCC

>1930 len=161 tot-seq=7

TGGGGAATTGTTCGCAATGGGCGCAAGCCTGACGACGCAACGCCGCGTGGAGGATGAAGA

CCTTCGGGTCGTAAACTCCTTTCGACCGAGATGAATTCTCGCCGACCTAACACGTCGGCG

GGGTGACAGTATCGAGGGAAGCAGCCCCGGCTAACTCCGTG

>1931 len=161 tot-seq=7

TGGGGAATATTGCACAATGGGCGAAAGCCTGATGCAGCAACGCCGCGTGAGCGATGAAGG

CCTTCGGGTCGTAAAGCTCTGTCTTCAGGGAAGAACCGGCATAATGTAAACAATGTTATG

TCCTGACGGTACCTGAGGAGGAAGCTCCGGCTAACTACGTG

>1932 len=161 tot-seq=7

TGGGGAATATTGGACAATGGGCGAAAGCCTGATCCAGCCATGCCGCGTGTGTGAAGAAGG

CCTTCGGATCGTAAAGCTCTGTTGTAAGAGAAGAACGTGTGTGAGAGTGGAAAGTTCACA

CAGTGACGGTAACTTACCAGAAAGGGACGGCTAACTACGTG

>1933 len=162 tot-seq=7

TGAGGAATATTGGTCAATGGACGGAAGTCTGAACCAGCCAAGTAGCGTGCAGGATGACGG

CCCTATGGGTTGTAAAGCTCTGTTAATCGGGACGAATGGTCGGTGTGCAAATAGTGCGTG

GACATGACGGTACCGGGATAGAAAGCCACGGCTAACTACGTG

>1934 len=164 tot-seq=7

AGCCAGCGTTCTGCATGGGGGTTTTAATGTAGTGCCAGGCAATAATGTGAGATCCATTCT

CGTTCTGGATTAGAGGTTGGCCTTGGAAAGCCTCCAAGGGTTCAGGACTCCCTGTTGACT

CTTCTTTACGTTTCCTAGCTGTCTGCCCAGTCAGCCCAGTATTC

>1935 len=165 tot-seq=7

AACTAAGGTACACCCCTAGGATTGCACCATAAAATAAGGAGACAACTGAGAGGTGAGATG

CACAGGTGGAAAATGCCTTGTACTTCCCCTGAGCTGATGAGATTGCATGTATGGAAGAAA

TTATCTTAGAGTAAGAGTAAAGCATCCCAGTCAGGGGACCTCCAC

>1936 len=166 tot-seq=7

TGAGCGTTGATCACACAACTGTATTCCAGCCTGGGTAGCAAAGTGAGATCCCTGTCTCAC

AAAAAAGAAGAAATTCGGACACAGACACACACACAGGGAGTCTGTCACATGAAGACGAAG

GCAGAGATCAGAGTGATGCTTCTACAAGCCAAGGACTACCAAGATG

>1937 len=166 tot-seq=7

TGGGGAAGTGCTCAAGGCCAAAGGCTCTGCGGCCAACTGGGCCCCAACGGCCAAGAGCCA

TGGGGGATGGGGAAGTGCTCAAGGCCAAAGGCTCAAAGGCCAACACGCCCAGGCCACAAT

GCAGTGCCTGGGGGCTACAGTGCCCACGGCCACCTCACAAGGCCCT

>1938 len=169 tot-seq=7

TTTGAGTAAAACTTTTGTACCATGACTGTTATGTAAAGAGATTCAATTATGGTGCGCAGT

GTTTTGTGAGTAACTACCGCCCATAAAAAGTTAATTAATCAGGGCTGTGTCAATTTCCTG

GTGCCGTGTCAATTTGCTGCAAGGTAATTAATCACTAATCACCCACCAG

>1939 len=169 tot-seq=7

CTCTGTGGAATCAGGGAACAGTAGCAACAGCTTCAGTTCCCCAACAGGTGCACAACATTT

CACAGGCCACACAGCTCTGTGGACAGGAGCGTTCTAGGAAGGACAGGCAGAGCATCCCAG

CCTGCCCCAGAAGAACACAGGCCAGATGCCTCAGGATATTCCTTTGGGC

>1940 len=170 tot-seq=7

GTTCATGGTGCTCTCTGGAATCTTCCTCAAGGGAGGAGCCTGAAAGCTCTTTACAGATCA

TTGCCTGGCTTTCCCACTTCTCAGCTCTGTTGTCTGATGACCAACAGCTATGGCCATCAA

GATGGCTCAGGAGAAAGGCACTTGCTGCCAAGCATCTGTCCTCCAACCCT

>1941 len=170 tot-seq=7

TCCATGGGCCAGATATAGTCCTGGAGCGGCCAGTTTGGAACCAGAGAGTGTGTGTATGTT

AGAGGGGTGGAGTCAGGGAAGGGAGAGAATGCCCAAGGTCACTGAGATTTCAAGCCCAGG

CCACCAGGAAAGTGATATGTCCTTGCACTGGAATAGGGAAGTCGGGAGGA

>1942 len=171 tot-seq=7

CTTGCAAGCTGCCATAGTACTGAACAGATAAACCTCAAACACCTGCCATACCCCACAACT

CCCTCCTAAGTGGTCTTCCTGGATAATTGTCATGCTGGTCCCATGTGACCTCCACCTAAG

CCTCAGCTGCGTGGACTGGAGCTCATCCCATGACCCAAGTGCAGCCAACTT

>1943 len=173 tot-seq=7

AACTTAGGAAATTAGTCTGAACAGGTTAGAGGGTACACCAGAGAACCGGACAGCTTCTGG

GACGGGCAGAAGCACAGACCCGCTGAGGCAGCACCCTTGGTGGGCCGCAGACAGCCGGCC

ACCGTCCGGACCAGAGGACAGGTGTCCGCCTGGCTTGGGAGGCGGCCTCAGTC

>1944 len=173 tot-seq=7

GAAGACTTTTGAGCTGAGCCTTAAGGGGTGGTCAAGAGTTTGTTGACGGAGATTCAGGGG

TTGGTGTCAGAGAGAGCATCCTCCTCTTCTCATGGGCAGGCAGGAAAGAGAGGCCTTTGT

CCCTTTCCGTGAGGGTTGCCTCCAGTCCCCGAGTCGGCCCCGCACAGAGCCCA

>1945 len=173 tot-seq=7

CACTGAGAAGACAAAGCTAGCAGGATGGTTGGGAGATAGATTATGACCACACAGTCCCAC

AGACACCAAGCCTGAGGCTGCCCAGGGCTGTATGTGGAGGTGGAGGTGGCTTCCAGCCAG

GACACCTGAGAGGAGTCCTGAAGAGCACCAGAACATGTGGTGGTAATGGCAGC

>1946 len=175 tot-seq=7

GGTTGGATCTTACAGAGTCTTGGGTTTCACTTGGAGGGAGATGAGAAACCATTATAGAAA

TATGAGTAATGGGATGAGTGACACAAGTGGACTTGTGTTCTGAACAGCTGTCCTAGCTGA

CATGTGACAACTAGACCATAAGGAAGGCGAGGCTTTAGAAGACTGTTGCAATAAT

>1947 len=176 tot-seq=7

CACCATGTCCTCGTTTGAGGGGTAGTCTGGCAACCTGACCACCCTTACTCTATCTACAGA

AATTCCAAGAGCAAGTCAAACATGTGCTCTCTGCCTGCGATGGGCACTATGCTACTTCTC

TTTCCTCTTGCTGGAAGAAGTCAAACCCTACTCTCCAACCCATGCAGCCCTCCACC

>1948 len=176 tot-seq=7

TGGGGAATTTTGCGCAATGGGGGAAACCCTGACGCAGCAACGCCTCGTGAGTGATGGAGG

CTTTCGGGTCGTAAAGCTCTGTCAGAGGGGAAGAAATGAGACTGGCTAATATCCGGTTTT

CTTGACGGTACCCTCAAAGGAAGCACCGGCTAACTCCGTCCCAGCAGCTGGGGAAT

>1949 len=177 tot-seq=7

TAGAAAAGCTCTGTTATGTGGGAGCAAACGGCAGCAGAAATGGGCATTTTCCCAAGTCCC

TGAGGGCAGGGCCTAACGTCCAAGCCTGCTTTGTTTGGTGATAAGATTTGGCTTTGTTCT

TTTCCCGAGCCCCAAGGATACATGCTGCGGGGAGTCCTGATCTGAGTTCTCCGTTGA

>1950 len=178 tot-seq=7

GTAGCTGAGCAGGGGCCGGAGCCAGCGGAGGATGGCGGGCACCTGGCATCGGAGCCTGAG

GTGCAGCCAAGTGACAGGTCCCCCCTAGAGCACTCCTCACCTGAGAAGGAGGCCCCAAGC

CCAGAGAAGACTCTCCCACCCCAGAAAACCGTGGCGGCTGAAAGTCACGAGAAAGTCC

>1951 len=179 tot-seq=7

TGAGGTGCTGGCTAAGAGGGCGTGTGGTGGGAGTTGGACCCTCTCGCATGGCTGCAGTGT

GCAGAACGTGCTGGGAGACCCCAGGACTACAGACGGTGGCTCCTGTGCCCTGAGAAGGAC

CCGGCTTCTTCACGAGGAGATGCCCAAGGCTCTGCAGGGTAACCCAGAGCCCTTGGTCT

>1952 len=181 tot-seq=7

CACTATCAGGGGACGAGAAAAGCAGACTGACAGACAGGCCTCTTCCCGGCTCACATGCAG

GCAGCACAGAACATCGTGGGGCTCCGCTAGGGAGAGAAACAGCCTCGGTTTACCTTAAAT

GTCATCTTTGTTTCTTTCTAAGTCTCAAACATCCTGCCCTTCCCCCACTGCTTACCCCGC

C

>1953 len=183 tot-seq=7

TGGGGAATATTGCACAATGGGGGAAACCCTGATGCAGCGACGCCGCGTGGAGGAAGAAGG

TCCTCGGATTGTAAACTCCTGTTGTTGAGGAAGATAATGACGGTACTCAACAAGGAAGTG

ACGGCTAACTACGTGCCAGCAGGCGGTAATAGGCAGATCGGAAGAGCGTCGTGTTGGGGA

ATA

>1954 len=184 tot-seq=7

TGGGGAATATTGCACAATGGGGGAAACCCTGATGCAGCGACGCCGCGTGGAGGAAGAAGG

TCTTCGGATTGTAAACTCCTGTTGTTGAGGAAGATAATGACGGTACGCAACAAGGAAGTG

ACGGCTAACTACGTGCCAGCAGCGCGGTAATCATCAGATCGGAAGAGCGTCGTGTAGGGA

AAGA

>1955 len=190 tot-seq=7

TAGGGAATCTTCCACAATGGGCGAAAGCCTGATGGAGCAACGCCGCGTGGGTGAAGAAGG

TCTTCGGATCGTAAAACCCTGTTGTTAGAGAAGAAAGTGCGTGAGAGTGGAAAGTTCACA

CAGTGACGGTAACTTACCAGAAAGGGACGGCTAACTACGTGCCAGCAGCCGCGGTATGAG

CAGATAGGGA

>1956 len=192 tot-seq=7

TCCAATTTCCCCTTGGTAACCAGGACTGATTACCCCATCCTACACAGTAGCTCCCTTCCT

TGCCTGTTAATTAAGAGGCAGGAGGAGCCCAAGGTGACTAGTTAAGATGATCGTCTTAAC

TTCTGTTTCAGTGAAATTATTGTTGTGTTTCCTAGTAAAAATATTTCTCCCATTGGAGCT

AATACATCTATA

>1957 len=193 tot-seq=7

AGGGGTGGAGGCACAGGGTAGGGGGCAGGGCCAGGCTGGGACCGCCCTTGGTGGCCTCGG

GTGGAAGCCTGGGCTCCCCACCACCTAGATCCTTGCAGGCAACGGCTGCCACCAGCTGGG

TCCGAAAGGCACCTCAGCAGCTTGGGATCACCTAGAATGGCCCCATTTCAAATATTCTGT

TCCATGCCCAGCC

>1958 len=198 tot-seq=7

CTCAAACTCTCTTGACGGATGGGAAACTGATGCTCCGACGACAGGGTAGAGACTCCTCAG

CGTCAACCCGCTTTCGGCGGCAGGACAGAAACTGGGCCTGGGCCTGAGATGGTTCCTTCC

TACACCCTTCTCCAGGGTACCGTGTCCACCTCTAAGCCTCTGGACCCTCTGACATGTAAC

CTGTGTCCCCTCCCCAAG

>1959 len=204 tot-seq=7

GGCAGCTCCTAAATGGCTGAGTGACCCTCTGCTCACACTGTCATCCGATCCCTCCGGGTC

AGAGTCAGACTGTGGGATGAGGGACAAGGGAGCTGACCTCCTGCCCATTTTGCTTCTGCT

GTTCTTGTAAATAATAAACTGTCTAAATCCACGTGGGCTCATTGTCTCATCCCTGGCTGG

ATGCATGGAAGTGCGCCAAATCCA

>1960 len=206 tot-seq=7

GACACAGAGCCCTATCGGGTCAGCTTGTGAGGAACCTTCCCCCAGGGTGTTCCAGTCACG

GCTATGCCAGCACCACCTCCCAAAATCAGAAAGAAAAGCCTCTGCTGAAGCAGAGAGTGT

ATCTCAGTTTCCAGTGCAGCTGTACTGTTCGGCAGCCTCAGGCAAGCCACTGCAGCTGGC

TGCTCCAGGCCATGTTACTCTCCCCA

>1961 len=208 tot-seq=7

TTCCCTTCTATGGCAGGGCTGCTCCCACCAGCCAGTGAAGGTCACTGTGTCCAGAGTCAC

TGTCCTGGGCAGGGCTCTGGGACCTCCTGCCGCATATTCTTGGCTGATGGTGCTGATTTG

GCCCCAAATCTCTTTAGAGACCACCCAAGGCCTGCCACAGCACAGTCTATTTTTTCATAA

GGGAGCTTGAGCACGGGGGCAGTTGGGG

>1962 len=210 tot-seq=7

TAGGGAATCTTCCACAATGGGCGCAAGCCTGACCGAGCAACGCCGCGTGAGTGAAGAAGG

TTTTCGGATCGTAAAACTCTGTTGTTAGAGAAGAACAAGGATGAGAGTAACTGTTCATCC

CTTGACGGTATCTAACCAGAAAGCCACGGCTAACTACGTGCCAGCAGCGCGGTAATTACA

AGATCGGAAGAGCGTCGTGTAGGGAAAAAG

>1963 len=210 tot-seq=7

GGCCACTGGAGTGGAATGGAGACCTGGGGGCCTGTGGGAGCTGTCCAGGCGTGGACGGTG

GTGTGGGGCTGGGTGTGGGGTGCTGCTTCTGTTGACAGAATCCCCACGCTCTGCTCTTCC

CCCAGTTCTGGCTGACCATGTTCTACTTGTCGAAGATGACAGAGGAGCAGACTTTGGTCA

TGTACAGTGGGCACCCACTTGGCCTCTTTC

>1964 len=217 tot-seq=7

TCTCTGTGACTGGAGTTCAGACGTGTGCCCTTCCGATCTCCTATGGGAGGCAGCAGTGGG

GAATTTTCCGCAATGGGCGAAAGCCTGACGGAGCAATACCGCGTGAGTGAAGAAGGTTTT

CGGATCGTAAAGCTCTTTTGTTAGGGACGAATGTAGCTTTTGTAAATAATGAGAGCTAAT

GACGGTACCTAAAGAAAAAGCCACGGCTAACTACGTG

>1965 len=221 tot-seq=7

CAAGCAGAAGACGGCATACGAGATCTAAGAGTGAATGGATGAAGAAGTGTGAGATTAAGA

TATAATAAGGGAGGAAGAAGTGGGGAATATTGAGAAATGGGGAAAAAAATGAAGAAGAGAA

GAAGAGTGAGGGATGAAGGCCTTCGGGTTGTAAACCGCTTTTGATTGGGAGCAAGCCTTC

GGGTGAGTGTACCTTTCGAATAAGCGCCGGCTAACTACGTG

>1966 len=229 tot-seq=7

GGCCCTGATGGAGACTGTGCACAATAAATGTTCAATAAGTACTTGTGAATATTGAAAAAA

TGCACAGGTGCTCCCAGCAGCTCACCCCTCCAGTGGCTGTTCTTTCTACCTGTCAAAGCT

TGTGCTGACACATATACTGGGAGGTGACCCCCAGCTGCGGCTGCCCCACCAGGCACATCA

AGCCAGGCCCCGGCCCTGCTTTCACCTGGCTCGCCTATGGCTAGTCTGT

>1967 len=230 tot-seq=7

CACACATCCCAGCAACCATGGACCCCCCCCACACTCACCCTGAAAAGTACACTGTCACAT

GGGCAGAGCTGAGAGCACCCTGCCACCCCCACACCCAGAAGCCCAGGGGCACAGCACAGA

GGACTGCGCAGAGCTGAGGGTGCCAGGCAGCTGGGGTTCCCTGCAGCCACCCTCCCCACC

AGGCCCCTCCGGAGGCGTCCACAGAGGGCACATGCACCTGGCTGTGAGGA

>1968 len=234 tot-seq=7

GTGTGGAGAGAGTGAGACTGGAGGCCACCAGGCCAGGCACAGTGGCTCATGTCTGTAATC

CCAGCACTTTGGGAGGCCAGGGTGGGTGGATCACCTGAGGTTGGGAGTTGGAGACCAGCC

TGACCAACATGGAGAAACCCCGTCTCTACTAAAAATACAAACTTAGCTGGGCATGGTGGT

GAGCAGCTATAATCCCAGCTACTCGGGAGGCTGAGGCAGGAGAATTGCTTGAAC

>1969 len=234 tot-seq=7

CAGCTGGCCCAGGAGGAGGAGAGCGAGGCCAGGCGCCTGGCCACTATGATGATGAAGAAG

CCGGAGAAGTACCTGTACCAGAAGATCATGTTTGACAAAAGGCGAAAAAGCCAAGAGGCC

AAAAAGCTACCAGAGTAGCAGAAAGCCCATGATGAGGCAGTGAGATCTGAGAAGGCCGAA

AAATCAAGGCCGATTTGAGTGCCAGTGGCCCCTCAGAGGGCCGAGGCCAGCTCC

>1970 len=161 tot-seq=7

TGGGGAATATTGCGCAATGGGGGAAACCCTGACGCAGCGACGCCGCGTGCGGGATGAAGG

CCTTCGGGTTGTAAAGCTCTTTTATTAGGGAAGAACGTTGGTAGGAGTGGAAAATCTACC

AAGTGACGGTAACTAACCAGAAAGGGACGGCTAACTACGTG

>1971 len=161 tot-seq=7

TGGGGAATATTGCACAATGGGCGCAAGCCTGATGCAGCAACGCCGCGTGAAGGATGAAGG

TCTTCGGATTGTAAAGCTCTGTTATACGGGACGAACGGCAAGGTGGTAAATAGCCATCAT

GAGTGACGGTACCGTAAGAGAAAGCCACGGCTAACTACGTG

>1972 len=102 tot-seq=6

AAGAGATTGCAAAAAAAGAATTAAAACACAGGCTGTTACAGTAACCCAGGTGGAAACTGA

TGAGAATAGAATGTTTGCTTTTAATTCATAGGTGTCATTTGG

>1973 len=104 tot-seq=6

CAGGCACAACAGCAGGCAGGCGCAGACGGTAAAGATGAATTTCGCTTTCTTCGATACGGT

GGGGTAGTCAATTTCAACTTCCATGCCACGGTATTTTTTCGGCA

>1974 len=105 tot-seq=6

CCCAGTGCCTACAGTCTCCATCAGAGAAGCCACATCTGAGGATGCTGTAGGAGTCACTGG

GATGATACAGAACCGCATCACCACTGAACCGGAAACCTCACAACA

>1975 len=106 tot-seq=6

CCAATCACATCCAGGACAGCCCAAGGCATCTGTACCTGGTGTAACCAATCCCCTCTGAAT

AAACAGCTCACTCAGCTTAGGTTCCCTCCTCCTCTCCCTGCTCTGC

>1976 len=107 tot-seq=6

AAGTCACGGTCACTGGATAGCTTCACTAAGTCATAAACTACACGGGCCCAGCCGAGAGGC

TGCCTGTGGCCACTGGTGGGCTATGCGTGAAGGCTGAGGCGTGCGTT

>1977 len=110 tot-seq=6

TGGGGAATATTGCGCAATGGGCGCAACTCTGATGCAGCGACGCCGCGTGCGGGATGAAGG

CCTTCGGGTCGTAAACCGCTTTTGTTTGGGAGCAAGCCTTTTGGGTGAGT

>1978 len=110 tot-seq=6

CCTGAAGCCAGGAGGAGGGTCAAAGCAAAGCCAGAGAGGCAGCTCTTCTGGAACACATGG

GGCATGTCGAGTGGACAGGAAGTTGGTGTAGCACCAGGCAGTGTCTAGAC

>1979 len=112 tot-seq=6

GACAGGGAGCACCACAGGCTGGGAGTGCAGGCCTTTTGTTAAGTGTGGAAAGGGTGATTC

TAGTGGCCCTGAGAGCTGCAGCCCTCTTGTCTGGCTGATCAGGAAGCTACTG

>1980 len=114 tot-seq=6

TGGGGAATCTTGCGCAATGGGCGCAACCCTGATGCAGCGACGCCGCGTGCGGGATGAAGG

CCTTCGGGTCGTAAACCGCTTTTGATTGGGAGCAAGCATTCGGGTGAGTGTACC

>1981 len=117 tot-seq=6

CCCTGACGCAGCGACGCCGCGTGCGGGATGAAGGCCTTCGGGTTGTAAACCGCTTTTGAT

TGGGAGCAAGCCTTTTTGGTGAGTGTACCATACGAATAAGGACCGGCTAATTACGTG

>1982 len=117 tot-seq=6

AAAGCAAAACAGAAAGTCTCTGTTCTTGTGGAGTTTCTAGTGGGCATGTGTGGTGGTCAG

AGACTCCTGAGATAAGCAGTCCTACTATTTTACTATCTCAAAATCCTCCAAAAGAGG

>1983 len=118 tot-seq=6

GAAGGGCTTGAGAGAGCACCCACAGTGGGAGGGAAGCCTGACGGAGTCTTCCATCTTATA

TATAAAAATATAAGTAAACTTACATTTTATATGACCTTGCCCTAGCTCTTTGAGGTCC

>1984 len=122 tot-seq=6

TAAGGAAGCCAGCAGTGGGTAAGGAAGCCAGCAGTGGGTAAGGAAGCCAGCAGTGGGTAA

GGAAGCCAGCTATGGGTAAGGAAGCCAGCTGTGGGTAAGGAAGCCAGCTATGGGTAAAGA

AG

>1985 len=123 tot-seq=6

TAGGGAATCTTCCACAATGGACGCAAGTCTGATGGAGCAACGCCGCGTGAGTGAAGAAGG

GTTTCGGCTCGTAAAGCTCTGTTGTTGGTGAAGAAGGATTAGAAAGTCACGGCTAACTAC

GTG

>1986 len=124 tot-seq=6

AAGCATCGGCAGTCAGGGGTGACTTTTTTTTTGGAGCAAAGGCATTAGACAAGATGACAC

GCTGACAGGTTCAGAAAACAGTGAATGCCGTGCCAATCAGCAGATAATTTGTGTGTTTGC

AGCG

>1987 len=124 tot-seq=6

GGAACATGTCCAGGCAACTCTTGGGCTGACGGGGCCAAGAGGTGGCTGATGTGGCCCTTT

TGCCCACCCTTGATCCTCCCCTAAATCTTCATCCCCAGCCCTGTAGCCATCTCCCCTGGG

CCCC

>1988 len=125 tot-seq=6

TATGCTTTAAAGATATAATTCATCATGCCAAAGAAGTCAAGACAGGAGTTTAAAGCAATT

GAGCATATTACATCCACAACTGAGAAGCAGAGAACAGCTGATCATGGTGGCGCATGCTTC

TAATC

>1989 len=126 tot-seq=6

GCCGACTAAGTGTGAGGATTTGGGGGCCTACCCTATAAGTAGCCCACAGTAGGAACAACT

CCAAAGACTCGGAAAAACTCAAGTAGATTACTTTTAATAGGGCAGCTTCTTGTTCTCGGG

ATCACC

>1990 len=129 tot-seq=6

GCTGCTCCTTCAGCTCTCGGTTCTGTGACAGTGCTCTGCTGATGGTGGTGCGGTCACTCT

GCATGCTCTCCAGGATCTGCTTGCGCTCCTCGGCCTGCTCGCTCCAGCGCTGGGCCTCCC

GCTCCAGCT

>1991 len=129 tot-seq=6

CAAGGTGAGAGGCAGCAGCAAGGTGAGAGGCAGCAGCAAGGTGAGAGGCAGCAGCAAGGT

GAGAGGCAGCAGCAAGGTGAGAGGCAGCAGCAAGGAACAGAGCCGGTTCCTCAGGGCTAG

GGACATGGA

>1992 len=134 tot-seq=6

TAGGGAATCTTCCACAATGGACGCAAGTCTGATGGAGCAACGCCGCGTGATTTAGAAGGC

CTTCGGGTTGTAAAAATCTTTTGTATAGGAAGAAAATGACAGTACTATACGAATAAGGTC

CGGCTAAATACGTG

>1993 len=135 tot-seq=6

TGGGGAATATTGCACAATGGGGGGAACCCTGATGCAGCAACGCCGCGTGAGTGAAAAAGT

ATTTCGGTATGTAAAGCTCTATCAGCAGGGAAGAAGATGACGGTACCTGATTAAGAAGCC

CCGGCTAACTACGTG

>1994 len=135 tot-seq=6

TGGGGAATATTGGACAATGGGCGCAAGCCTGATCCAGCAATGCCGCGTGAGTGATGAAGG

CCTTCGGGTTGTAAAGCTCTTTCGCACGCGACGATGATGACGGTAGCGTGAGGAGAAGCC

CCGGCTAACTTCGTG

>1995 len=135 tot-seq=6

TCGGGAATTTTGGGCAATGGGCGAAAGCCTGACCCAGCAACGCCGCGTGAGGGATGAAGT

ATTTCGGTATGTAAACCTCGAAAGAATGGGAAGAATAAATGACGGTACCATTTATAAGCT

CCGGCTAACTACGTG

>1996 len=135 tot-seq=6

TGGGGAATATTGGACAATGGGGGAAACCCTGATCCAGCAATGCCGCGTGAGTGACGAAGG

CCTTAGGGTTGTAAAACTCTTTTCTAAGTGAAGATGATGACGGTAACTTAGGAATAAGCT

CCGGCTAACTTCGTG

>1997 len=135 tot-seq=6

TAGGGAATCTTCGGCAATGGGGGAAACCCTGACGCAGCAACGCCGCGTGCGGGATGAAGG

CCTTCGGGTTGTAAACCGCTTTCAGCAGGGAAGACATCGACGGTACCTGCAGAAGAAGCC

CCGGCTAACTACGTG

>1998 len=135 tot-seq=6

TGGGGAATATTGCACAATGGAGGGAACTCTGATGCAGCGATGCCGCGTGAGGGAAGAAGG

TTTTCGGATTGTAAACCTCTGTCAACGGGGACGAAAATGACGGTACCCGAGAAGGAAGCT

CCGGCTAACTACGTG

>1999 len=135 tot-seq=6

TAGGGAATCTTCGGCAATGGACGGAAGTCTGACCGAGCAACGCCGCGTGAACGATGAAGG

TCTTCGGGTCGTAAAGTTCTGTTGCAGGGGAAGATAATGACGGTACCTTGTGAGGAAGCC

CCGGCTAACTACGTG

>2000 len=135 tot-seq=6

TGCTCTAAGATGAAGACGCAAAAGGAGAAAAAGAACAAAACTTAAAGAGCTTCCTCCCAA

CCTACTTGCTGCTTGCCAAAGCCCACCAATCCACTGCTCCAAGGCACAGCCAACCACTCT

CCCCGAGAACCACAC

>2001 len=135 tot-seq=6

TAGGGAATCTTCCACAATGGACGCAAGTCTGATGGAGCAACGCCGCGTGAGTGAAGAAGG

CCTTCGGGTCGTAAAGCTCTTTTATATGGGAAGATAATGACGGTACCATAAGAAAAAGCC

CCGGCTAACTACGTG

>2002 len=135 tot-seq=6

TGGGGAATATTGGGCAATGGGCGAAAGCCTGACCCAGCGACGCCGCGTGAGCGAAGAAGG

TCTTCGGATTGTAAAGCTCTGTTGATAGGGACGATAATGACGGTACCTATCGAGGAAGCC

ACGGCTAACTACGTG

>2003 len=135 tot-seq=6

ATTTGCTATGGATCACTAGTGGGTAAACAAGAAGGCATGTGTGCACATGATAGTCATTAA

ATGACAGGCAAGAGTTACATGTACAAGCGAAATGATAAAGTGGCCCCCTCTAGTAGATGA

TGTCTGTGTCCATTC

>2004 len=135 tot-seq=6

TGGGGAATATTGCACAATGGGGGAAACCCTGATGCAGCGACGCCGCGTGGAGGAAGAAGG

TCTTCGGATTGTAAACTCCTGTTGTTGGGGAAAGGAAGGATGGTACCCAACAAGGAAGTG

ACGGCTAACTACGTG

>2005 len=135 tot-seq=6

TGGGGAATATTGCACAATGGGGGCAACCCTGATGCAGCGACGCCGCGTGAGCGATGAAGG

AATTCGTTTCGTAAAGCTCTGTCCTTGGAGAAGATAATGACGGTAACCAAGGAGGAAGCA

CCGGCTAAATACGTG

>2006 len=135 tot-seq=6

TGAGGAATATTGGTCAATGGGGGAAACCCTGATGCAGCGACGCCGCGTGAGCGAAGAAGG

CTTTCGAGTCGTAAAGCTCTGTCCTATGAGAGGATAATGACGGTATCATAGGAGGAAGCC

CTGGCTAAATACGTG

>2007 len=135 tot-seq=6

TGGGGAATCTTGCACAATGGGCGAAAGCCTGATGCAGCGACGCCGCGTGCGGGATGAAGG

TCTTCGGATCGTAAAGTTCTGTTGCAGGGGAAGATAATGACGGTACCCTGTGAGGAAGCC

CCGGCTAACTACGTG

>2008 len=135 tot-seq=6

TGGGGAATATTGCACAATGGGCGCAAGCCTGATGCAGCAACGCCGCGTGAACGATGAAGG

TCTTCGGATCGTAAAGTTCTGTTGCAGGGGAAGATAATGACGGTACCATAAGAAAAAGCC

CCGGCTAACTACGTG

>2009 len=135 tot-seq=6

TGAGGAATATTGGACAATGGGCGGAAGCCTGATCCAGCCATGCCGCGTGAGCGAAGAAGT

ATTTCGGTATGTAAAGCTCTATCAGAAGGGAAGAAAATGACGGTACCTTACTAAGAAGCT

CCGGCTAAATACGTG

>2010 len=135 tot-seq=6

TGGGGAATATTGGACAATGGGCGAAAGCCTGATCCAGCAATGCCGCGTGTGTGAAGAAGG

TCTTCGGATTGTAAAGCACTTTCGACGGGGACGATGATGACGGTACCCGTAGAAGAAGCC

CCGGCTAACTTCGTG

>2011 len=135 tot-seq=6

TGAGGAATATTGCGCAATGGGGGAAACCCTGACGCAGCAACGCCGCGTGCGGGATGAAGG

CCTTCGGGTTGTAAACCGCTTTCAGCAGGGAAGACATCGACGGTACCTTACTAAGAAGCT

CCGGCTAAATACGTG

>2012 len=135 tot-seq=6

TGGGGGATATTGCGCAATGGGGGCAACCCTGACGCAGCAACGCCGCGTGAAGGAAGAAGG

TTTTCGGATCGTAAACTTCTTTAAGTGGGGAAGATAGTGACGGTACCCACAGAATAAGCC

ACGGCTAACTACGTG

>2013 len=135 tot-seq=6

TGGGGAATATTGCACAATGGGCGAAAGCCTGATGCAGCAACGCCGCGTGAGTGAAGAAGA

CCTTCGGGTTGTAAAGCTCTGTCTCCAGGGACGATAATGACGGTACCTGAGGAGGAAGCC

ACGGCTAACTACGTG

>2014 len=135 tot-seq=6

TGGGGAATCTTCCGCAATGGACGAAACCCTGATGCAGCGACGCCGCGTGAGCGAAGAAGG

CTTTCGAGTCGTAAAGCTCTGTCCTATGAGAAGATAATGACGGTATCATAGGAGGAAGCC

CTGGCTAAATACGTG

>2015 len=136 tot-seq=6

CACAATTGACCCCATTGTTTTGATTATATAAACTAAATACTGTGGACTTACCACGTAAGA

ATGATTTAACTACAACTTATGAAACGTTCAAGGTATCAATTCAGAAACCATTGTATTCTA

TTGGACATCAGTAGGA

>2016 len=136 tot-seq=6

TGGGGAATATTGCACAATGGGGGAAACCCTGATGCAGCAACGCCGCGTGAAGGAAGACGG

TCTTCGGATTGTAAACTTTTGTTCTTGGTGAAGAAGAATGACGGTAGCCAAGGAGGAAGC

CACGGCTAACTACGTG

>2017 len=136 tot-seq=6

TCGGGAATATTGCGCAATGGAGGAAACTCTGACGCAGTGACGCCGCGTATAGGAAGAAGG

TTTTAGGATTGTAAACTATTTTAGACAGGGAAGAAAAAAGACAGTACCTGTAGAATAAGC

TCCGGCTAACTACGTG

>2018 len=136 tot-seq=6

TGGGGGATATTGCGCAATGGGGGAAACCCTGACGCAGCAATGCCGCGTGAAGGAAGAAGG

TTTTCGGATTGTAAACTTCTTTTGTTAAGGACGAAGAATGACGGTACTTAACGAATAAGC

TCCGGCTAACTATGTG

>2019 len=136 tot-seq=6

TCGGGAATATTGCGCAATGGAGGAAACTCTGACGCAGTGACGCCGCGTATAGGAAGAAGG

TTTTCGGATTGTAAACTATTGTCGATAGGGAAGAAAAAAGACTGTACCTATCAAGAAAGC

TCCGGCTAACTACGTG

>2020 len=136 tot-seq=6

TGGGGAATATTGCGCAATGGGGGAAACCCTGACGCAGCAACGCCGCGTGATTGAAGAAGG

CCCTCGGGTTGTAAAGATCTTTAATCGGGGACGAAGAATGACGGTACCCGAAGAATAAGC

TCCGGCTAACTACGTG

>2021 len=136 tot-seq=6

ATGAGTGTCCCAGGTACCATGGGCCTTCCCTATGTTCCTGAGCCTCTGAGAGGGTGGAGA

CCACAGCAGGCATGTGGTACCAGATGAAGGAGTGAGGGAGTAAACCAACGACCGCCATGG

CCTCCAATGGATGTCT

>2022 len=136 tot-seq=6

AGAGGGGCTGTGGTGTGGCCTTCACACACTGCAACTCCTGGAGGTCGGGCAGGGGCAGGA

TGTCCAGACAGTGGACGCATGAGTGGGGAAGCCTTCAGCCACCCTGACTCCACCTAGGAG

GTGCCCCAAGGCAGAA

>2023 len=136 tot-seq=6

TTGGGAATCTTGCACAATGGGGGAAACCCTGATGCAGCGACGCCGCGTGGAGGATGAAGG

ATCTAGGTCTGTAAACTCCTTTTTCAGGGAAAGACTTAGGACGGTACCCTGAGAATAAGC

TCCGGCTAACTACGTG

>2024 len=136 tot-seq=6

TGGGGAATATTGCACAATGGGGGGAACCCTGATGCAGCGACGCCGCGTGAGGGAAGAAGG

TTTTTGGATTGTAAACCTCTGTCCTTGGTAAAGAAGCAAGACGGTAGCCAAGGAGGAAGC

CACGGCTAACTACGTG

>2025 len=136 tot-seq=6

TCGGGAATATTGCGCAATGGAGGAAACTCTGACGCAGTGACGCCGCGTATAGGAAGAAGG

TTTTCGGATTGTAAACTATTGTCCACAGGGAAGAAACAAGACTGTACCTGTGAAGAAAGC

TCCGGCTAACTACGTG

>2026 len=136 tot-seq=6

TAGGGAATTTTGCGCAATGGGGGAAACCCTGACGCAGCAACGCCGCGTGAACGAGACGCC

CTTCGGGGTGTAAAGTTTTGTCAGTGGGGACGAACAAATGACGGTACCCACAGAGGAAGC

ACCGGCTAACTCCGTG

>2027 len=137 tot-seq=6

TGGGGAATATTGGGCAATGGGGGAAACCCTGACCCAGCAACGCCGCGTGGAGGGAGAAGG

TTTTCGGATCGTAAACTCCTGTCCTCAGGGAAGAGCAGAAGACGGTACCTGAGGAGGAAG

CCCCGGCTAACTACGTG

>2028 len=137 tot-seq=6

TGGGGGATATTGCACAATGGGGGAAACCCTGATGCAGCGACGCCGCGTGGACGAAGAAGT

ATTTCGGTATGTAAAGTCCTATCAGCAGGGAAGAAGAAATGACGGTACCTGACTAAGAAG

CCCCGGCTAACTACGTG

>2029 len=137 tot-seq=6

TGGGGGATATTGCACAATGGGGGAAACCCTGATGCAGCGACGCCGCGTGAGGGAAGACGG

CCTTCGGGTTGTAAACCTCTGTCTTCGGGGACGAATAAATGACGGTACCCGAGGAGGAAG

CCACGGCTAACTACGTG

>2030 len=137 tot-seq=6

TGGGGAATATTGGGCAATGGGGGAAACCCTGACCCAGCAACGCCGCGTGAAGGAAGAAGG

TCTTCGGATTGTAAACTTCTTTGATCAGGGACGAAGAAATGACTGTACCTGAAGAACAAG

TCACGGCTAACTACGTG

>2031 len=137 tot-seq=6

CCCGCCAGCCCACAGCAGCTTCTGTCTTCACCTCCCTCAGCCCCTGTGTTGCTGGGAGTT

ACAGATCCTAAAATGTGTCAGTGTGTCAAGCACAATGCCACCTCCCCTCTGGCCCCTCCA

GGTCACCTCACGGTCCC

>2032 len=138 tot-seq=6

TAGGGAATATTGCACAATGGACGAAAGTCTGATGCAGCGACGCCGCGTGAGTGATGAAGG

CCTTCGGGTTGTAAAGCTCTGTTCTTAGGGAATAAAAAAATGAAGGTACCTAAGAAGAAA

GGACCGGCTAACTTCGTG

>2033 len=138 tot-seq=6

TGGGGAATATTGCACAATGGGCGAAAGCCTGATGCAGCAACGCCGCGTGAAGGATGAAGG

TCTTCGGATCGTAAACTTCTGTCCAAAGGGAAGAAACAAATGACGGTACCTTTGGAGGAA

GCCCCGGCTAACTACGTG

>2034 len=138 tot-seq=6

TGGGGAATATTGGGCAATGGGCGAAAGCCTGACCCAGCGACGCCGCGTGAAGGAAGACGG

TCTTCGGATTGTAAACTTCTTTTATATGGGACGAAACAAATGACGGTACCATATGAATAA

GCCACGGCTAACTACGTG

>2035 len=138 tot-seq=6

TGGGGGATATTGCACAATGGGCGAAAGCCTGATGCAGCGATGCCGCGTGAGGGAAGACGG

TCTTCGGATTGTAAACCTCTGTCTTTGGGGACGAAACAAATGACGGTACCCAAGGAGGAA

GCACCGGCTAACTACGTG

>2036 len=138 tot-seq=6

CAGCAGGAAAGAAGTTATACCAAAAACGACTTGTACACCACAGACATTATAACCCTTTCC

TCAAAGAAACAGTCATGTTCTGTTGGGTATTATGGACAGGTCTCTGGAAATTTATCTAAT

AAAGACCAACAAACTTCC

>2037 len=138 tot-seq=6

TGGGGGATATTGCACAATGGGGGAAACCCTGATGCAGCAACGCCGCGTGAAGGAAGAAGG

TCTTCGGATTGTAAACTTCTGTTTTTAGTGAAGAAACAAATGACGGTAGCTAAAGAGGAA

GCCACGGCTAACTACGTG

>2038 len=138 tot-seq=6

CCCGCACAGCCTGTATCCCCCACTGACTCAAACCCTAACACAGAATGCATCCCCAGTAAA

TAAACTGTAGGGGCTACCCAGGGATAAGGCAGGGATTTCTCTAGGACATCACCCACCCAG

CTTCCTTAAGCTAGAACA

>2039 len=138 tot-seq=6

TGGGGAATATTGCACAATGGGCGAAAGCCTGATGCAGCGACGCCGCGTGAGCGAAGAAGT

ATTTCGGTATGTAAAGCTCTATCAGTAGGGAAGAAAGAAATGACGGTACCTACAGAAGAA

GCGACGGCTAAATACGTG

>2040 len=138 tot-seq=6

TGAGGAATATTGGTCAATGGCCGGGAGCCTGACCCAGCAACGCCGCGTGAAGGAAGAAGG

CTTTCGGGTTGTAAACTTCTTTTCTGAGGGACGAAGCAAGTGACGGTACCTTAGGAATAA

GCCACGGCTAACTACGTG

>2041 len=139 tot-seq=6

TGAGGAATATTGGTCAATGGGCGAGAGCCTGAACCAGCCAAGTAGCGTGCAGGATGACGG

CCCTATGGGTTGTAAACTGCTTTTATATGGGAATAAAGAAATGACGGTACCTACAGAAGA

AGCGACGGCTAAATACGTG

>2042 len=140 tot-seq=6

TGGGGAATATTGGACAATGGGCGGAAGCCTGATCCAGCAACGCCGCGTGAGGGATGACGG

CCTTCGGGTTGTAAACCTCTTTCGGTAGGGACGAAGCGCAAGTGACGGTACCTACTAAAG

AAGCACCGGCCAACTACGTG

>2043 len=140 tot-seq=6

TGGGGAATATTGCACAATGGGCGCAAGCCTGATGCAGCAACGCCGCGTGCGGGATGACGG

CCTTCGGGTTGTAAACCGCTTTCAGTTCCGACGAAGCGCAAGTGACGGTAGGAACAGAAG

AAGGACCGGCCAACTACGTG

>2044 len=140 tot-seq=6

TAGGGAATCTTCCACAATGGACGCAAGTCTGATGGAGCAACGCCGCGTGCGGGATGACGG

CCTTCGGGTTGTAAACCGCTTTCGCCTGTGACGAAGCGTGAGTGACGGTAATGGGTAAAG

AAGCACCGGCTAACTACGTG

>2045 len=140 tot-seq=6

AGATTGTTCTTGACTAGCTGCCCTGCCTATCAACGGCAGGTGGCCAGGAGAAATCTGGTC

GTGTGAGGACCAGGACCAAGCCCAGAGGGGCTCCTGTCTCCTGTCGTGTGCCTAGGGCAT

AGCAGACAGCCCACACCCCT

>2046 len=140 tot-seq=6

TGGGGAATCTTGCACAATGGGCGGAAGCCTGATGCAGCGACACCGCGTGCGGGACGAAGG

CCTTCGGGCTGCAGACCGCTTTCAGCAGGGACGAGGCGCAAGTGACGGTACCTGCAGAAG

AAGCCCCGGCTAACTACGTG

>2047 len=140 tot-seq=6

CGGTACTTGATTTCGTACTTGAGGATGAGTCCGTTGGGGTCTGGTGGCTCGAGCCAGCGC

AGAAGGACACTGTTCTTGCTGGAGGCCTCCCAGGCCACCTTTCCTGGAATACCATCAGCC

TCTCCTGCGGGAAGGGGCAT

>2048 len=141 tot-seq=6

GAAGTGGGTGGGTGCCCATGTGGAGAGTTGGCATCCATGGGCTCCTCCCTGGTAGGAGGA

GGGAGAGCAGACTAGGGGAGATGGGATAGTGGGAGCATGTGGACGTTGGCTTTTTTTTTT

CTTGGTGAAATGGAAGAGTTC

>2049 len=142 tot-seq=6

TGGGGAATCTTCCGCAATGGGCGAAAGCCTGACGGAGCAACGCCGCGTGAGTGAAGACGG

CCTTCGGGTTGTAAACCGCTTTTGATTGGGAGCAAGCCTTCGGGTGAGTGTACCTTTCGA

ATAAGCGCCGGCTAACTACGTG

>2050 len=143 tot-seq=6

TAGGGAATTTTCGGCAATGGGGGCAACCCTGACCGAGCAACGCCGCGTGCGGGATGAAGG

CCTTCGGGTTGTAAACCGCTTTTGATTGGGAGCAAGCCTTTTGGGTGAGTGTACCTTTCG

AATAAGCGCCGGCTAACTACGTG

>2051 len=143 tot-seq=6

GGGAAGGAGTGGATAGGGCACTCTCACGGCTTGAGGATGCAGAGCGGAGCTTGCCCTTGG

ATGGCACAGCCGTCCCTTAGGAGGAGTTGTGGCCCTCAGTCCACACCCGGCCTCGGCCTC

CAGGTCAGAGTTTGGGATGTTGA

>2052 len=143 tot-seq=6

TGAGGAGTATTCCACAATGGGCGAAAGCCTGATGGAGCAATGCCGCGTGCAGGATGAAGG

CCTTCGGGTTGTAAACCGCTTTTGATTGGGAGCAAGCCTTTTGGGTGAGTGTACCTTTCG

AATAAGCGCCGGCTAACTACGTG

>2053 len=147 tot-seq=6

GGATTTTGTTTAGTTTTCTGAAGGGATGGGATTTACCCAAACCCCCCGGTTTGACTCCGG

GATAGGTTACCTCTTGATGGTAAGGCTTCGGAAAAGTGGGTACCACGCGGAGAACTGACA

GTGACTAACATGCTCCTTCTTCATTCT

>2054 len=147 tot-seq=6

TGGGGAATATTGCACAATGGGCGCAAGCCTGATGCAGCGACGCCGCGTGAGGGATGACGG

CCTTCGGGTTGTAAACCTCTTTCAGTAGGGAAGCAGCCTCTTTTTGGGGTGACGGTACCT

GCAGAAGAAGCGCCGGCTAACTACGTG

>2055 len=147 tot-seq=6

TTAGGAATCTTCCACAATGGGCGCAAGCCTGATGGAGCGACGCCGCGTGAGGGATGAAGG

TTCTCGGATCGTAAACCTCTGAACTAGGGACGAAAGAGCCGTATGGCAGATGACGGTACC

TAGGTAATAGCACCGGCTAACTCCGTG

>2056 len=148 tot-seq=6

TTAGGAATCTTCCGCAATGGACGGAAGTCTGACGGAGCGACGCCGCTTGGAGGAGGAAGC

CCTTCGGGGTGTAAACTCCTGAACTGGGGACGAAAGCCCTGATGAGGGGGATGACGGTAC

CCAGGTAATAGCGCCGGCCAACTCCGTG

>2057 len=148 tot-seq=6

GGAGGCCGGGGAGGCACGCTGCTCCCCCTCTCAGCCTTGCCCAGTGTCCAGACCTTTCAA

AAGAAGCCAGAAAATTCACGTTTGCTTGGGAATCCCACAATTCAATAGCTTTTCAAAACA

GTGCGGGAAGAAAAGTGGGTTTGGCCCA

>2058 len=149 tot-seq=6

CCTCGAGGCAGCAGTGGGGGATATTGCACAATGGGGGAAACCCTGATGCAGCGACGCCGC

GTGGGTGAAGACGGCCTTCGGGTTGTAAAGCCCTGTTGAGGGGGACGATAATGACGGTAC

CCCTTTAGAAAGCCACGGCTAACTACGTG

>2059 len=149 tot-seq=6

CCCATTGGCCATAGCACACAATCCGAAGCCCTCGGGACCAGGAGCTGCCTCCAGCAGGCA

GTAGGACTGGAAATGTTTATCCTCTAGCAGCTGCTCCCAGCACTTGACCTCGACGTCATA

GAGATACAGGGCCCCTGTATCAGTCACTG

>2060 len=149 tot-seq=6

TGGGGGATATTGCACAATGGAGGAAACTCTGATGCAGCGACGCCGCGTGAGTGAAGAAGT

ATTTCGGTATGTAAAGCTCTATCAGCAGGGGAGAAAGGCTCGCAAGAGAGATGACGGTAC

CTGAGTAAGAAGCACCGGCTAACTCCGTG

>2061 len=154 tot-seq=6

TGAGGAATATTGGTCAATGGTCTGACGACTGAACCAGCCATGCCGCGTGAAGGTCACGGC

CCTATGGGTCTTAAACTTCTTTTGTAAGGGAGCAATAAGTCTCACGTGTGGGATGATGAG

AGTACCTTACGAATAAGCATCGGCTAACTCCGTG

>2062 len=154 tot-seq=6

CAAAAGTACCTTGGAAAAAATTCTAAACCAAATGAGCTAGAATCTGTATACTCAGGAGAT

CGAGCATTCATTGAAAAAGAACCGTTAGCTCACTTAATGACGTATCTGGAAGATACCTCA

CTTTGTCAGTTCCACAAAGCTGGAGGTAAACCTG

>2063 len=154 tot-seq=6

TGAGGAATATTGGTCAATGGGCGAGAGCCTGAACCAGCCAAGTCGCGTGAGGGAAGACGG

TACTAAGTATTGTAAACCTCTTTTGTCAGGGAACAAAGAGACCACGTGTGGTAAGATGAG

TGTACCTGAAGAAAAAGCATCGGCTAACTCCGTG

>2064 len=155 tot-seq=6

TGAGGAATATTGGTCAATGGGCGTTAGCCTGAACCAGCCAAGTCGCGTGAGGGAAGTAGG

TCCTCAGGATCGTGAACCTCTTTAGCCGGGGAGCAACGCGGCCCACGCGTGGGCCGGTGA

GCGTACCCGGAGAATAAGCATCGGCTAACTCCATG

>2065 len=155 tot-seq=6

TGAGGAATATTGGTCAATGGGCGTAAGCCTGAACCAGCCAAGTAGCGTGCAGGATGACGG

CCCTATGGGTTGTAAACTGCTTTTATGCGGGGATAAAGTGGCCCACGTGTGGGTTTTTGC

AGGTACCGCATGAATAAGGACCGGCTAATTCCGTG

>2066 len=155 tot-seq=6

TAGGGAATCTTCGGCAATGGACGGAAGTCTGAACCAGCCAAGTAGCGTGCAGGATGACGG

CCCTATGGGTTGTAAACTGCTTTTGTATGGGGATAAAGTTAGGGACGTGTCCCTATTTGC

AGGTACCATACGAATAAGGACCGGCTAATTCCGTG

>2067 len=155 tot-seq=6

TAAGGAATATTGGTCAATGGACGCAAGTCTGAACCAGCCATGCCGCGTGGAGGATGAAGG

CCCTCTGGGTTGTAAACTTCTTTTATGGGGGACGAGACCCCGGATTTTTATCCGGCCTGA

CGGTACCCCAGGAATAAGCACCGGCTAACTCCGTG

>2068 len=155 tot-seq=6

TGAGGAATATTGGTCAATGGGCGTAAGCCTGAACCAGCCAAGTAGCGTGAAGGATGACTG

CCCTACGGGTTGTAAACTTCTTTTGTATGGGAATAAAGTTCAGTACGTGTACTGTTTTGT

ATGTACCATACGAATAAGCATCGGCTAATTCCGTG

>2069 len=155 tot-seq=6

TGAGGAATATTGGTCAATGGGCGAGAGCCTGAACCAGCCAAGTCGCGTGAAGGATGAAGG

ATCTATGGTTTGTAAACTTCTTTTATAAGGGAATAAAGTGTAGGACGTGTCCTATTTTGT

ATGTACCTTATGAATAAGCATCGGCTAACTCCGTG

>2070 len=155 tot-seq=6

TGAGGAATATTGGTCAATGGGCGAGAGCCTGAACCAGCCAAGTCGCGTGAGGGAAGACAG

TCCTATGGATTGTAAACCTCTTTTGCCGGGGAGCAAAGAGCGGCACGTGTGCCGCGCCGA

GAGTACCCGGAGAAAAAGCATCGGCTAACTCCGTG

>2071 len=155 tot-seq=6

TGAGGAATATTGGACAATGGGCGCAAGCCTGACCCAGCCATGTCGCGTGCAGGATGACGG

TCCTATGGATTGTAAACTGCTTTTATACGAGAAGAAACACTCCGACGTGTCGGAGCTTGA

CGGTATCGTAAGAATAAGGATCGGCTAACTCCGTG

>2072 len=155 tot-seq=6

TGAGGAATATTGGTCAATGGGCGAGAGCCTGAACCAGCCAAGTAGCGTGCAGGATGACGG

CCCTATGGGTTGTAAACTGCTTTTATATGGGAATAAAGTGAGGGACGTGTCTCGCATTGC

AGGTACCGCGTGAATAAGGACCGGCTAATTCCGTG

>2073 len=155 tot-seq=6

TGAGGAATATTGGTCAATGGACGCAAGTCTGAACCAGCCAAGTAGCGTGCAGGATGACGG

CCCTATGGGTTGTAAACTGCTTTTATATGGGGATAAAGGAGTCCACGTGTGGATTTTTGC

AGGTACCGCATGAATAAGGACCGGCTAATTCCGTG

>2074 len=155 tot-seq=6

GAGGATAGCCCCGCCAGGAACCAGTGCCCAAGTCTATAAGGTTAAAACAGTCCCAAAGGG

CTGGGACTGGGGGCTTCCTGCTGCAGGGGCTGGGGTACAGGCTGGGGCAAGCCTCTATCT

CTCTGACTTGACCCGGTAACGCAGCACAAGGTAGG

>2075 len=155 tot-seq=6

TGTGGAATATTGGTCAATGGACGAGAGTCTGAACCAGCCAAGTAGCGTGAAGGATGACTG

CCCTATGGGTTGTAAACTTCTTTTATACGGGAATAAAGTTAGCCACGTGTGGTTTTTTGC

ATGTACCGTATAAATAAGGATCGGCTAACTCCGTG

>2076 len=155 tot-seq=6

TGAGGAATATTGGACAATGGGTGAGAGCCTGATCCAGCCATCCCGCGTGAAGGACTAAGG

CCCTATGGGTTGTAAACTTCTTTTGTATAGGGATAAACCTACTCACGTGTGAGTAGCTGA

AGGTACTATACGAATAAGCACCGGCTAACTCCGTG

>2077 len=155 tot-seq=6

TGAGGAATATTGGACAATGGGCGAGAGCCTGATCCAGCCATGCCGCGTGCAGGAAGACGG

CCCTATGGGTTGTAAACTGCTTTTATACGGGAAGAAACACTCTGACGTGTCAGAGCTTGA

CGGTACCGTAAGAATAAGGATCGGCTAACTCCGTG

>2078 len=155 tot-seq=6

TGAGGAATATTGGTCAATGGACGAGAGTCTGAACCAGCCAAGTAGCGTGCAGGATGACGG

CCCTCTGGGTTGTAAACTGCTTTTAGTTGGGAACAAAAAAGGCGACGTGTCGCCTCTGGA

GTGTACCATCAGAAAAAGGACCGGCTAATTCCGTG

>2079 len=155 tot-seq=6

TGAAGAATATTGGTCAATGGACGCAAGTCTGAACCAGCCATGCCGCGTGCAGGAAGACGG

CTCTATGAGTTGTAAACTGCTTTTGTACGAGGGTAAGTGCAGGTACGTGTATCTGTTTGA

AAGTATCGTACGAATAAGGATCGGCTAACTCCGTG

>2080 len=155 tot-seq=6

TAGGGAATCTTCCACAATGGACGAAAGTCTGATGGAGCAACGCCGCGTGAGTGATGAAGG

TTTTCGGATCGTAAAACTCTGTTGTCAGGGAAGAACACCCGGGAGAGGATCCCGGATTGA

CGGTACCTGACGAGAAAGCCCCGGCTAACTACGTG

>2081 len=155 tot-seq=6

TGAGGAATATTGGTCAATGGCCGAGAGGCTGAACCAGCCAAGTCGCGTGAAGGATGAAGG

ATCTATGGTTTGTAAACTTCTTTTATAGGGGAATAAAGTGTGGAACGTGTTCCATTTTGT

ATGTACCCTATGAATAAGCATCGGCTAACTCCGTG

>2082 len=155 tot-seq=6

TGAGGAATATTGGTCAATGGACGAGAGTCTGAACCAGCCAAGTAGCGTGCAGGATGACGG

CCCTATGGGTTGTAAACTGCTTTTGTTTGGGAATAATCGACATTACGTGTCATGCATTGC

AGGTACCACATGAATAAGGACCGGCTAATTCCGTG

>2083 len=156 tot-seq=6

TGAGGAATATTGGTCAATGGACGAGAGTCTGAACCAGCCAAGTCGCGTGAAGGAAGACGG

CCCTACGGGTTGTAAACTTCTTTTGTAAGGGAATAAATAGTCTTACGCGCTAAGACGTTG

CAGGTACCTTACGAATAAGGATCGGCTAACTCCGTG

>2084 len=156 tot-seq=6

TGAGGAATATTGGTCAATGGGCGGGAGCCTGAACCAGCCAAGTCGCGTGAGGGATGACGG

CCCTATGGGTTGTAAACCTCTTTTGCCGGGGAGCAACGGGGTCCTTGCGAGGGCCTTATG

AGAGTACCCGGAGAAAAAGCATCGGCTAACTCCGTG

>2085 len=159 tot-seq=6

TCACTCTGGATTTTTCCTTTGATGGTGTTGTACTTATAACCTCCCTGAAGGAGCTCCTCC

AAAAGCTTGCCATCCACAGTGGTAAAATGGTCCTTAACGGGAGTGTCTCCATTTCTTCCA

TTAGGAAGAGAAAGAAATCAAACTCACCCCCTTCCCTCA

>2086 len=159 tot-seq=6

TGGACCTGCAAGCTATCCAGAACATTGTCCTTAGCAGTGGTGAGATGGTCAGGTCAGCGC

CTGGGTCCTTCCCAGCTCAGGCATGCTTTTTGCCAGGATAGGAGCTTCTCAGGCCAGGGC

CTCTCTTGCCAGTGCAGGCAGTCAGGCCTGGCACCCAGT

>2087 len=159 tot-seq=6

TAGGGAATCTTCCGCAATGGGCGAAAGCCTGACGGAGCAATACCACGTGAACGATGAAGG

TCTTCTGATTGTAAAGTTCTTTTATTTAGGAAAAAAAGCGTGCTAGGAAATGAGTGCGCC

TTGATGGTACTAATTGAATAAGTGACAGCTAACTATGTG

>2088 len=160 tot-seq=6

TGGGGAATATTGCACAATGGGGGAAACCCTGATGCAGCGACGCCGCGTGTGTGAAGAAGG

TCTTCGGATTGTAAAGCACTTTAAGTTGGGAGGAAGGGCAGTTGCCTAATACGTAACTGT

TTTGACGTTACCGACAGAATAAGCACCGGCTAACTCTGTG

>2089 len=160 tot-seq=6

TAGGGAATCTTCCACAATGGGTGCAAGCCTGATGGAGCAATGCCGCGTGAGTGAAGAAGG

ACTTCGGTTCGTAAAGCTCTGTTGTTGGGGAAGAACACGGATAGGAGTCACTGCCTATCC

CTTGACGGTACCCAACCAGAAAGTCACGGCTAACTACGTG

>2090 len=160 tot-seq=6

TAGGGAATCTTCCACAATGGACGCAAGTCTGATGGAGCAACGCCGCGTGAGTGAAGAAGG

TCTTCGGATCGTAAAACTCTGTTGTTAGAGAAGAGAGATAGAGGTAGTAACTGGCCTTTA

TTTGACGGTAATTACTTAGAAAGTCACGGCTAACTACGTG

>2091 len=160 tot-seq=6

TGGGGAATCTTCCGCAATGGACGAAAGTCTGACGGAGCAACGCCGCGTGAGTGATGACGG

TCTTCGGATCGTAAAACCCTGTTGTTAGAGAAGAAAGTGCGTGAGAGTAACTGTTCACGT

TTCGACGGTATCTAACCAGAAAGCCACGGCTAACTACGTG

>2092 len=160 tot-seq=6

TAGGGAATCTTCCACAATGGACGCAAGTCTGATGGAGCAACGCCGCGTGAGTGAAGAAGG

TCTTCGGATTGTAAAGCACTTTAAGTTGGGAGGAAGGGCAGTAAGTTAATACCTTGCTGT

TTTGACGTTACCAACAGAATAAGCACCGGCTAACTTCGTG

>2093 len=160 tot-seq=6

TGGGGAATATTGCGCAATGGGGGAAACCCTGACGCAGCCATGCCGCGTGTGTGAAGAAGG

CCTTCGGGTTGTAAAGCACTTTCAGCGGGGAGGAAGGCGGTGAGGTTAATAACCTCATCG

ATTGACGTTACCCGCAGAAGAAGCACCGGCTAACTCCGTG

>2094 len=160 tot-seq=6

TGGGGAATATTGCGCAATGGGGGAAACCCTGACGCAGCGACGCCGCGTGCGGGATGAAGG

CCTTCGGGTTGTAAAGCACTTTAAGTTGGGAGGAAGGGCAGTTACCTAATACGTGATTGT

TTTGACGTTACCGACAGAATAAGCACCGGCTAACTCTGTG

>2095 len=160 tot-seq=6

TGGGGAATTTTGGACAATGGGGGCAACCCTGATCCAGCCATTCCGCGTGAGTGAAGAAGG

CCTTCGGGTTGTAAAGCTCTTTCGCAAGGGAAGAAACGATACGAGCGAATAACTCGTGTT

AATGACGGTACCTTGATAAGAAGCACCGGCTAACTACGTG

>2096 len=160 tot-seq=6

TAGGGAATCTTCGGCAATGGGGGGAACCCTGACCGAGCAACGCCGCGTGAGTGAAGACGG

CCTTCGGGTTGTAAAGCTCTGTGATTCGGGACGAAAGGCCATATGTGAATAATATATGGA

AATGACGGTACCGAAAAAGCAAGCCACGGCTAACTACGTG

>2097 len=160 tot-seq=6

TAGGGAGTTTTCGGCAATGGGGGAAACCCTGACCGAGCAACGCCGCGTGAATGATGAAGG

CCTTCGGGTCGTAAAATTCTGTTGTAAGAGACGAACTGTGAGGGTAGGAAATGCCCCTCA

CTTGACTGTATCTTACGAGAAAGGGACGGCTAACTACGTG

>2098 len=160 tot-seq=6

TGGGGAATATTGGACAATGGGGGCAGCCCTGATCCAGCCATGCCGCGTGTGTGAAGAAGG

CCTTCGGGTTGTAAAGCACTTTCAGCGAGGAGGAAAGCATCAAGGTTAATACCCTTGGTG

TGTGACGTTACTCGCAGAAGAAGCACCGGCAAACTCCGTG

>2099 len=160 tot-seq=6

TGGGGAATCTTTCGCAATGGACGAAAGTCTGACGGAGCAACGCCGCGTGTGTGAAGAAGG

TCTTCGGATTGTAAAGCACTTTAAGTTGGGAGGAAGGGCAGTAAATTAATACTTTGCTGT

TTTGACGTTACCGACAGAATAAGCACCGGCTAACTCTGTG

>2100 len=160 tot-seq=6

TGGGGAATATTGCACAATGGGCGCAAGCCTGATGCAGCCATGCCGCGTGTATGAAGAAGG

CCTTCGGGTTGTAAAGCTCTGTTGTTGGTGAAGAAGGACAGGGGTAGTAACTGACCTTTG

TTTGACGGTAATCAATTAGAAAGTCACGGCTAACTACGTG

>2101 len=160 tot-seq=6

TGGGGAATATTGGACAATGGGCGCAAGCCTGATCCAGCAACGCCGCGTGGAGGACGAAGG

TCTTCGGATCGTAAACTCCTGTCAGACGGGACGAAGGTTTCCGGGCGAGCAGTCCGGAAG

CTTGACGGTACTGTCAGAGGAAGCCCCGGCTAACTCCGTG

>2102 len=160 tot-seq=6

TAGGGAATTTTCGGCAATGGGGGGAACCCTGACCGAGCAACGCCGCGTGAACGATGAAGG

CCTTCGGGTCGTAAAGTTTTGTTGTTAGGGAAGAATGGCGCCTACAGGGAATGGTAGGCG

AGTGACGGTACCTTTCGAGGAAGTCACGGCTAACTACGTG

>2103 len=160 tot-seq=6

TAGGGAATCTTCCACAATGGGCGCAAGCCTGATGGAGCAACACCGCGTGAGTGAAGAAGG

GTTTCGGGTTGTAAAGTACTTTCAGCGGGGAGGAAGGGAGTAAAGTTAATACCTTTACTC

ATTGACGTTACCCGCAGAAGAAGCACCGGCTAACTCCGTG

>2104 len=160 tot-seq=6

TGGGGAATATTGCGCAATGGGGGAAACCCTGACGCAGCGACGCCGCGTGAGTGAAGAAGG

GTTTCGGCTCGTAAAGCTCTGTTGTTGGAGAAGAACGTGCGTGAGAGTAACTGTTCACGC

AGTGACGGTATCCAACCAGAAAGTCACGGCTAACTACGTG

>2105 len=160 tot-seq=6

TGGGGAATATTGCACAATGGGGGAAACCCTGATGCAGCGACGCCGCGTGAACGAAGAAGG

TATTCGTATCGTAAAGCTCTGTTGTTGGTGAAGAAGGACAGGGGTAGTAACTGACCTTTG

TTTGACGGTAATCAATTAGAAAGTCACGGCTAACTACGTG

>2106 len=160 tot-seq=6

CTAAGAATCTTCCACAATGGACGAAAGTCTGATGGAGCGACGCCGCGTGAATGATGAAGG

CCGTGAGGTTGTAAAGTTCTTTTCGGGAGGAGGAATGAAGTTATCAGGGAATGGATGACA

GATGACGAGAATCCCGGAATAAGCCCCGGCTAACTACGTG

>2107 len=160 tot-seq=6

TCGAGAATCTTTCGCAATGGACGGAAGTCTGACGAAGCGACGCCGCGTGTGTGATGAAGG

CTCTAGGGTTGTAAAGCACTTTCGCTTGGGAATAAGAGAAGACGGTTAATACCCGCTGGA

TTTGAGCGTACCAGGTAAAGAAGCACCGGCTAACTCCGTG

>2108 len=160 tot-seq=6

TGGGGAATCTTGCACAATGGGCGAAAGCCTGATGCAGCGACGCCGCGTGCGGGATGAAGG

CCTTCGGGTTGTAAACTGCTTTTGTCAGGGAAGAAAAGGTCTGTGTTAATACCATAGACT

GCTGACGGTACCTGAAGAATAAGCACCGGCTAACTACGTG

>2109 len=160 tot-seq=6

TAGGGAATCTTCGGCAATGGGGGCAACCCTGATGCAGCCATGCCGCGTGAATGAAGAAGG

CCTTCGGGTTGTAAAGTTCTTTCGGTGACGAGGAAGGTTGATAGGTTAATAGCCTATCAA

ATTGACGTTAATCACAGAAGAAGCACCGGCTAACTCCGTG

>2110 len=160 tot-seq=6

TGGGGAATATTGGACAATGGGCGCAAGCCTGATCCAGCCATGCCGCGTGTGTGAAGAAGG

CCTTCGGGTTGTAAAGCACTTTCAGCGAGGAGGAAGGGTGGTGTGTTAATAGCACATCAT

TTTGACGTTACTCGCAGAAGAAGCACCGGCTAACTCTGTG

>2111 len=160 tot-seq=6

TGGGGAATATTGCACAATGGGGGAAACCCTGACCGAGCAACGCCGCGTGAATGAAGAAGG

CCTTCGGGTTGTAAAATTCTGTTATAAGGGAAGAAAGGTGATAGGAGGAAATGACTATCA

ATTGACGGTACCTTATGAGAAAGCCACGGCTAACTACGTG

>2112 len=160 tot-seq=6

TGGGGAATATTGCGCAATGGGGGAAACCCTGACGCAGCGACGCCGCGTGCGGGATGAAGG

GTTTCGGCTCGTAAAACTCTGTTGTTGAAGAAGAACGTGCGTGAGAGTAACTATTCACGC

AGTGACGGTATTCAACCAGAAAGTCACGGCTAACTACGTG

>2113 len=161 tot-seq=6

TGGGGAATCTTCCGCAATGGGCGCAAGCCTGACGGAGCAACGCCGCGTGAGTGAAGAAGG

TCTTCGGATCGTAAAGCTCTGTTGACGGGGACGAACGTGCGTGATGCGAACAGTGTCATG

CAATGACGGTACCTGTCGAGGAAGCCACGGCTAACTACGTG

>2114 len=161 tot-seq=6

TAGGGAATCTTCCGCAATGGACGCAAGTCTGACGGAGCAACGCCGCGTGAGTGAAGAAGG

ATTTCGGTTCGTAAAACTCTGTTGTTAGAGAAGAACGTTTGTAGGAGTGGAAAATCTACC

AAGTGACGGTAACTAACCAGAAAGGGACGGCTAACTACGTG

>2115 len=161 tot-seq=6

TGGGGAATCTTCCGCAATGGACGAAAGTCTGACGGAGCAACGCCGCGTGAACGATGACGG

CCTTCGGGTTGTAAAGTTCTGTTATATGGGACGAACAGGATGACGGTTAATACCCGTTGT

CCCTGACGGTACCGTAAGAGAAAGCCACGGCTAACTACGTG

>2116 len=161 tot-seq=6

TGGGGAATATTGCACAATGGGCGCAAGCCTGATGCAGCCATGCCGCGTGTATGAAGAAGG

TTTTCGGATCGTAAAGCTCTGTTGTTAGAGAAGAACGTTGGTAGGAGTGGAAAATCTACC

AAGTGACGGTAACTAACCAGAAAGGGACAGCTAACTACGTG

>2117 len=161 tot-seq=6

TAGGGAATTTTCGTCAATGGGGGGAACCCTGAACGAGCAATGCCGCGTGAGTGAGGAAGG

TTTTCGGATCGTAAAGCTCTGTTGTAAGAGAAGAACGAGTGTGAGAGTGGAAAGTTCACA

CTGTGACGGTATCTTACCAGAAAGGGACGGCTAACTACGTG

>2118 len=161 tot-seq=6

TGGGGAATATTGCGCAATGGGGGTAACCCTGACCGAGCAACGCCGCGTGAGTGAAGAAGG

TTTTCGGATCGTAAAGCTCTGTTGTAAGTCAAGAACGAGTGTGAGAGTGGAAAGTTCACA

CTGTGACGGTAGCTTACCAGAAAGGGACGGCTAACTACGTG

>2119 len=161 tot-seq=6

TGGGGAATATTGGACAATGGGGGGAACCCTGATCCAGCCATGCCGCGTGTGTGAAGAAGG

CCTTTTGGTTGTAAAGCACTTTAAGCGAGGAGGAGGCTACTCTAGTTAATACCTAGGGAT

AGTGGACGTTACTCGCAGAATAAGCGCCGGCTAACTCTGTG

>2120 len=161 tot-seq=6

TAGGGATGAGGAATATTGGTCAATGGTCGGGAGACTGAACCAGCCAAGCCGCGTGAGGGA

GGAAGGTACAGAGAGTCGTAAACCTCTTTTGTCAGGGAACAAAGACTGGGACGCGTCCCC

GGATGAGTGTACCTGAAGAAAAAGCATCGGCTAACTCCGTG

>2121 len=161 tot-seq=6

TAGGGAATCTTCCGCAATGGACGAAAGTCTGACGGAGCAACGCCGCGTGAGTGATGAAGG

TTTTCGGATCGTAAAACTCTGTTGTCAGGGAAGAACAAGTGCCATTCAAACAGGGTGGCG

CCTTGACGGTACCTGACGAGAAAGCCACGGCTAACTACGTG

>2122 len=161 tot-seq=6

TGGGGAATTGTTCGCAATGGGCGCAAGCCTGACGACGCAACGCCGCGTGGAGGACGAAGA

TCTTCGGGTCGTAAACTCCTGTCGAGCGGGAAGAACACCTTCCTGGTGAACAATCGGGAG

GGCTGACGGTACCGCTAAAGGAAGCCCCGGCTAACTCCGTG

>2123 len=161 tot-seq=6

TGGGGAATTGTTCGCAATGGGCGAAAGCCTGACGACGCAACGCCGCGTGGAGGACGAAGA
TTTTCGGATCGTAAACTCCTTTCGATCGGGACGAACGACTGGTGACCTAACACGTCATCA
GAGTGACGGTACCGAGAGAAGAAGCCCCGGCTAACTCCGTG
>2124 len=162 tot-seq=6
TAGGGAATTTTTCACAATGAGCGAAAGCTTGATGGAGCAATGCCGCGTGAACGATGAAGG
TCTTTTTGATTGTAAAGTTCTTTTATTTGGGAAGAATGACTCTAGCAGGCAATGGCTGGA
GTTTGACTGTACCACTTTGAATAAGTGACGACTAACTATGTG
>2125 len=162 tot-seq=6
AGCTGGAAGTGGGTTCGGCCCACTCAAAGGGTAGACTGTTGCCCCTGGGTCTTACTCACT
GAGCTTGTGATGCAGAGTGAAATTCCTATCACGGTGAGCACCATGCCAGCAACTGCTCCT
GGGATAATAGGCAGAAATCAGGAGGGTCTGAGCTCAGCCATG
>2126 len=162 tot-seq=6
AGCTTGTGGTCCCAGCTTCTTCCCTGCACCCCGCATCAAGAGCCCTTATCCCAGGCAGCA
CAGCTCTTGGCAGTGCTTTCTATTTCCTTAAACATCCAGGACCTCTTCCCTCTCTCTCTC
TCCCTCCTTCCTGCTTGAGCAAGGCCACCCTCGCAAATTTAG
>2127 len=164 tot-seq=6
CCTGGTGTAGCAGTATCTCCACCATGGCTGGGCCACCTTTTCATGGCCGCTCATTCAAGT
TCTGGAGCACCAGGTCCAAACTTGAACTCCAGCCCTGCAAAGACGGGGGAGTAACATGCA
AGTGTAACCCACTTTTCAAGAGTCCATTATATTGTTCAATCATG
>2128 len=165 tot-seq=6
AAAAAGGTACATGGGCTCAGGGGCATCCTTCATTAGGTGGGACGTTGGGTCTGGCCCGGG
CCAGTTCTGCCTGCCTGACATCAGCCTGTCTGTCTGTCTGTCTCTCTGTCTGCAGCACCA
TCATCACCACCATCAGCAGATGCAGCAGGCCCCGGCTCCTGTGCC
>2129 len=165 tot-seq=6
GTAAACCATTATGCATCGGTAGGGGCTGTTCTGCTGGAGCCCTCTGATGGCCAGGCATGG
TCTGTCAGTGCAGGAGCTCTGATGAGGTGCCCTAGGAAGCACTCTGGTTGGGCATCTGAA
GCTGCTCTGCAAGAGGCCATGGTCAGGCTGGAGCTCCAGGAAAGG
>2130 len=167 tot-seq=6
CACAGCCCTTATTTTGGTAGTGTGTGTGTGATTAGTCAGGATGCATTGACTGTTGTTGTA

AACAATGTTTGGCTCTTTGTGAAAGGCCAGTGTTCATGTGCAGTTCAGCGTGGCTCTGCT

CCATGCAGTCTCTCAGGATCCAGGCCTCCCCACCTTGTGGCTGTGCC

>2131 len=168 tot-seq=6

AGGGATGTATGCTGTGTGCCCAGCAGCCATGGTCAGGGACAGAGGCAGAGGAGGGGTGTA

TGCTGGTGCCCAGCAACATGGTCAGGGACAGAGGCAGAGGAGGGTGTATGCTGGTGCCCA

GCATCCATGGTCAGGGACAGAGGCAGAGGAGGGAGGCATGCTGTGTGC

>2132 len=168 tot-seq=6

TCTGATCTCTGAGTAGCCATTTTCGACCCTTTCTACCCACAGAATGCATCTGGCCTGTCT

GGGACATCACAATCCACACACACAGCCTGCAGACTCCTCTCCATCCCCAGTGTTGTCTGC

AGTTTTCTGACATGACAAGGTTTCCTGCTTACAACTGCACTCACCGTT

>2133 len=175 tot-seq=6

CCCCTCCACCCTGAGCACCAGCAGCAGCAGTGGTGTTCCGTAACAAGGCTCCAGCATGAC

CCTGTGGCTTCACCCTCTACCCAGAGCACCAGCAGCAGTTGTGCAGACCCAGAAACATCT

CTGGCTAAACAAGCCCCCCAGCATGACCCTGTGGCCTCCCCCTTCACCCAGAGCA

>2134 len=180 tot-seq=6

CATGCAAGTAACATGCCCGCCACCCCCCTGCCTAACCTGCCGGTGGGGCCTGGAGACACC

ATTTCCATTGCCAGTATCCAGAACTGGATTGCCAATGTCGTCAATGAAACCCTCGCCCAG

AAGCAAAACGAAATGCTCTTGTTGTCCCGCCCACCCTCTGTTGCAAGCATGAAGGCAGCT

>2135 len=182 tot-seq=6

ATTGTGAAATGGAGAAATTACTCACATCTACTCGGAACCTTGACGAAGAGGGGGACCAGA

GTCCTTCAAGTCAGGGCTTTGTCTCCACACTTAATGAGAGGAGACTTGTATTTTTAGAAC

TCTGCTCTTTAATAATTGTGTAAGCAAAGTGTGGAAATGTAATCTATATCCAATCCATCA

AC

>2136 len=182 tot-seq=6

GGGAACCCCAGTAGGGTTCTACTTTAATCTATATTTCTGAAATCCCTTGGAAATGTTTGA

GAAGACCACATAATCTCTGAGTCTCACTTTTCTCCTCTGTGAAGTGCAGGTTTTGGACAA

GGGTGGAGATAGAGGCTCTTTTTAGAAGGAACTGCGTGGTATCAGCAGCAGTGGAATGAG

TG

>2137 len=185 tot-seq=6

TAGGGAATCTTCCACAATGGGCGCAAGCCTGATGGAGCAACACCGCGTGAGTGAAGAAGG

GTTTCGGCTCGTAAAACTCTGTTGTTGAAGAAGGACAGGGGTAGTAACTGACCTTTGTTT

GACGGTAATCAATTAGAAAGTCACGGCTAACTACGTGCCAGCAGCCGCGGTATGTCCAGA

TCGGA

>2138 len=186 tot-seq=6

CGAGGATGCTTCTTGGAGCTGCTCTCGTTCCCTCCATCCTGAAATTGATTTCTCCTGCTC

TTGTCTCTGTGTCCCCTCAGGGTCGTGAGGGGAAGCCGGGCAAGCAGGGTGAGAAAGGCC

AGAGGGGAGCTAAGGTAGGTCCCCTGCCTTCAAGGGTCACATCCCTCCCTAGCTCATCTC

CCTGGG

>2139 len=186 tot-seq=6

TGGGGAATATTGCGCAATGGGGGAAACCCTGACGCAGCCAAGTAGCGTGCAGGATGACGG

CCCTATGGGTTGTAAACTGCTTTTATGTGGGGATAAAGTGCGTGACGTGTCATGCATTGC

AGGTACCACATGAATAAGGACCGGCTAATTCCGTGCCAGCAGCCGCGGAATCAGAAGATC

GGAAGA

>2140 len=187 tot-seq=6

CAGTAGTCACAAGTCCATGAGGTGGAGCTTGAGATTAATACAATGCCTGGCTTCTTCTCT

TCTCCTTTAAGATACTAAGGAGTAGAAGGAAAACTAGAAATGTAACCATAATACAGGTAA

GAGCAGTATGTGTGCAGAAATGCTTTTATGTTATATGCAATAAAAGTAAACAGGTGACTC

TGGGGAA

>2141 len=188 tot-seq=6

CATCTGGGGTGTCCAAAGCCTGAGAATCTGTCATCCTCGGCCTTCGTGCCCTGAGCCAAC

TCCAGCAGCTCCTTGGGGACAACAGACTGTGCTTCCCCACCCCTGGAGTGGCGTCCTGCG

GCTTCACATAGCCTGATGCCTGCCTGACATGAGGAAGGAGCACTCAAAACTTCTCGAATC

CCACCTAC

>2142 len=189 tot-seq=6

CCTTACCTGTCACAGATACTTGACAGTGACCCACATTTTCAGAAACAGTAATTCTGCAGC

TGGTGGAAAACAGTATGCTTTGTGGTCCCCATAAGACCTAAGTATAATACCACTGATCTC

TTTGAAGTGCCTGAGGAACCTAAAGAGGTCATCCCTGAAAAGAAAGTGTCCCTGGTGCCT

CCTAAAAAG

>2143 len=189 tot-seq=6

CAGAGACGTGGGTTCCCACAGTGGGGTCCTGTGGCTGCAGCGGAACACCCCAGACCAGGA

GATGGAGAACACAGCTGGAGCGGCAGCGGGAGGTGCTAATCGGGGAAGGAAAATCGGTGC

TAATTGGGAAGGAAAAGAGAAAACAAGGGGAAAGAGAGCGGGTGCAAGACAGCTGGGAGG

AGTGGAATT

>2144 len=190 tot-seq=6

CCCCCCGCCCCCACCTACACTTCCCTGCCTGGAGTCAGAACAGGGCCCGGGACCTCCAGG

GGCCTCTGCACAAGACCCCACAGGCCTCGCTGGCCTCCCTGGGCATCGGTTCTGACTTCC

AGACCTCAGGCTCTGTCCGTGGATGCCCTTGGAGGCTGAGGGACCCTCCGTGCATGGGGT

CTCCATCTTC

>2145 len=191 tot-seq=6

TGGGGAATTTTCCGCAATGGGCGAAAGCCTGACGGAGCAATGCCGCGTGAAGGTAGAAGG

CCTACGGGTCATGAACTTCTTTTCCCGGAGAAGAAGCAATGACGGTATCCGGGGAATAAG

CATCGGCTAACTCTGTGCCAGCAGCCGCGTAATCCTTAGATCGGAAGAGCGTCGTGTAGG

GAAAGAGTTTG

>2146 len=199 tot-seq=6

TGATCTGGTGACTGGAGTTCAGACGTGTGCTTTCCGATCTCCTACGGGGCAGCAGTGGGG

GATATTGCACAATGGGGGGAACCCTGATGCAGCGACGCCACGTGAGGGATGACGGCCTTC

GGGTTGTAAACCTCTTTCAGTTGGGAAGAAGCCCTTTGGGGTGACGGTACCTTCAGAAGA

AGCACCGGCTAACTACGTG

>2147 len=199 tot-seq=6

CAAGAGATGTGAGGCATGTAGCTTTTAAGTCATGATGCCCTCACACCAGCTGGATGTGAG

CCTTGTAGCTCCTGACAGTGGGTGAACTACTGCAAAACTCCCCCAGAAAGCAGAGCACTT

AATGCCTTTCCTGCTGGCAAGCTGCCTCTCTTGTCCTATAGCTTCTTAGCCAATGGTTCT

CAAAGTGTGGTTCCCCAGT

>2148 len=200 tot-seq=6

GCATAAATAATGCAGGTGCCCAGAATAAAATGTAGTTATGTTGGTTTTTGCTTCTTTAGC

TAAGCAGCTACCAAAAAAAAGGAAAGAGACTGAATTGATAGATACATTTTACTGTCCAGC

TGCCAAGAAGGCATTGCCGACAATGAGGATTTCAGAATAATAACCCTAAAGAGAGGAGTT

CAGAGCATCCCATTCACTGC

>2149 len=201 tot-seq=6

CTGGAGTTCAGACGTTGCTCTTCCGATCTTCTACGGGAGGCAGCAGTAGGGAATATTGGT

CAATGGGCGAGAGCCTGAACCAGCCAAGTCGCGTGAAGGATGACTGTCTTATGGATTGTA

AACTTCTTTTGTAGGGGAATAAAGAACAGCACGTGTGCTGTAGTGAATGTACCCTACGAA

TAAGCATCGGCTAACTCCGTG

>2150 len=201 tot-seq=6

TGAGGAATATTGGTCAATGGGCGAGAGCCTGAACCAGCCAAGTCGCGTGAAGGATGACTG

TCCTATGGATTGTAAACTTCTTTTATGTGGGAATAAAAAACACTACGTGTAGTGTATTGT

ATGTACCATATGAATAAGCATCGGCTAACTCCGTGCCAGCAGCCGCGGTATGTCAAGATC

GGAAGAGCGTCGTGTTGAGGA

>2151 len=202 tot-seq=6

CAGCTCCAAAACATTGCCCCAAATCTGCCTGCTTCTCTCCCACTGCATTGCCACCACTGA

CTCTCGCCTGGAAGACCATGAGGCCTCCCGGTGGGTCGCGCTGACTCCACTCTACACACC

AGAGCATGGGCCACCAGCCAGCATCTCATCTCTCTTCCTCCTGGCCTCCGAGGTGGCCTG

ATGTGCCACGGCCCTGCCTACC

>2152 len=202 tot-seq=6

CAGGATTTGAGAGAACTGGCCCCAATTATGAAAGAAGTTCTACTTGTGGGTAAATGCTAT

CAAACAGCATTGCCTACTACAGAGAAATCTTTCATGAAAGGAAGAGTCAATTGATGCAGC

AAACTCTATTGTTGTCTTTTTTTATTTTTTTTTAGAAATTGACACGACCGTCCCAGTCTT

CAGCAACCGCCACCCTGATTAG

>2153 len=203 tot-seq=6

TCTCCAGCTGGAAATGGTCTGGGGGCTATTGATGGGGCACAATGGGAGTGAGACCGGCCC

TTCAGATTGCGTGGGAACTGGGTGAGGCCTGTGACTGCCGACTTTCCTCAACTTTCCTGA

CAACCTGCATGACTCAGCAGAGGCAGCAATCATCCTCCTAGATACACAACTCCAATGACC

TGGGAATCTCACCCCCATCCCTC

>2154 len=203 tot-seq=6

TTGGAGTGTAAGTTCCACTTTAGGATCTTCCCAAAACGAGGGAGCTGGGCCTATCAGCTC

AGAGCTCACCTGGAGGAAGGTACACTTCTAGCCCCTTTGGCCCTTCCTTGGGTGTGCACA

CTCTCACTCTAGTAGCCCAAGGCAGTCCTGGGAAGAAGAGTTAAAGGTACCAATTACAGG

TAGCAAAAGCACCCTGAAACACA

>2155 len=205 tot-seq=6

TCTGATAAGGACACCAGTCATTGGACTTAAGGCCTCTGCTAATCCAGTCTGGCCTCGTCT

CAAATTGAAGAACATCTGCACACACCCCATTTCCAAGTAAGATCCTATTCACAGGCACCA

GGGGTGCCTTCAATGCATCTTTTGGGGGGAACACAATTCAACCCACAGCATCTTCTGTGT

TGTTCTGCCCAAGGACAGCCACCCC

>2156 len=207 tot-seq=6

GAAGATATCAGGGGAAACGAAGAGAAAATGTCACATGGTACTGAAGCTGACTCTGCTGCC

TAATAGCTAAAAGGCCCCCAAAAGATGAGTCAAAGCTACTAGGCTCTCAGGCTAACCACA

TATAACAATGTGACCTTGGCTACATCCCTTCATCTGCAAAACTGAGCAGGCTGGATAATG

GGTCTTCATCATCTGTTATAACAAGAC

>2157 len=207 tot-seq=6

TGGGGAATATTGCACAATGGGCGCAAGCCTGATGCAGCAACGCCGCGTGAACGATGAAGG

TCTTCGGATCGTAAAACTCTGTTGTTAGAGAAGAACACGAGTGAGAGTAACTGTTCATTC

GATGACGGTATCTAACCAGCAAGTCACGGCTAACTACGTGCCAGCAGCCCGGTAATTATG

AGATCGGAAGAGCGTCGTGTTGGGGAA

>2158 len=207 tot-seq=6

ATTCTTACATATTCTGGTTGGACTCATGGTTAAAGAAGACAAATAGAAAAACTCACACAT

CAGCCTGGTGTGGGGATAAAACCAAAAACAGTGATGCTCACAGGCATGCCTGAGAGTGGA

ATGGTGGTGTTGGTGGGGGAGTGCCTGTTTACTGGTAGTAGGAGTAACTCTCGTCATCAT

ATTCCAGCTCATCTATGTCATCGTCAT

>2159 len=209 tot-seq=6

TAGGGAATATTGCGCAATGGGGGAAACCCTGACGCAGCGACGCCGCGTGTGTGAAGAAGG

TCTTCGGATTGTAAAGCACTTTAAGTTGGGAGGAAGGGCAGCTACCTAATACGTGATTGT

TTTGACGTTACCGACAGAATAAGCACCGGCTAACTCTGTGCCAGCAGCCGCCGGTAATAT

CTAGATCGGAAGAGCGTCGTGTTAGGGAA

>2160 len=212 tot-seq=6

TGGGGAATATTGGACAATGGGGGGAACCCTGATCCAGCCATGCCGCGTGTGTGAAGAAGG

CCTTATGGTTGTAAAGCACTTTAAGCGAGGAGGAGGCTCTTTTGGTTAACACCCAAGATG

AGTGGACGTTACTCGCAGAATAAGCACCGGCTAACTCTGTGCCAGCAGCCGGTAATGGGA

AGATCGGAAGAGCGTCGTGTAGGGAAAGAGTG

>2161 len=213 tot-seq=6

CAGACCCAGCCATGGCCAACTCCTCTGGAACTGCAATGCTAGCCTAGCCCACTGTCCCCA

GAGAGCTTTGCCTCCTGACCTCTCTCCACCACTTTCTGAGGCCAGGGTCCCCTAAGTTTG

ACTTGGTGAGCGTGGTGCACACATTGGTGCTGATCTGTATCATGGCCATGAGTATAATAC

AGGGACTACAATGCAGAGAGCAGACATAGGCAC

>2162 len=214 tot-seq=6

TAGGGAATTTTTCACAATGGGCGCAAGCCTTATGAAGCAATGCCGCGTGAACGATGAAGG

TCTTATAGATTGTAAAGTTCTTTTATATGGGAAGAAACGCTAAGATAGGAAATGATTTTA

GTTTGACTGTACCATTTGAATAAGTATCGGCTAACTATGTGCCAGCAGCCGCGGTAAGAT

CGGAAGAGCGTCGTGTAGGGAAATAGGGAATTTT

>2163 len=224 tot-seq=6

CACATCACCAACTCTATCATGGAAGCTCTCCTTATCATTCCGCTCCAGTCCTGTGATTAT

GAGGAGGGAGTCAAAACGTCCACTAATCAGGGCAGTGTGGTGTGCTATTCACAAGCTCAT

AACTGGCAGTAAAACAGAAAGGCCCATGTCTGCTCTGCTGAGTAATTTCCACACTGAGGC

CCTGAGCAGAGATGTCGTGTCATTTGTAACATCTGCCTTGGCAT

>2164 len=226 tot-seq=6

CCAGGGCGCTTTCCTGCCGCTCTCCTTCCCATCCTTGCTTGAAGGCAGGTTTAGTGAGGA

GCTGAGGAGCGGGGAGGTGCTGGGGGAGGGGGACCGGGGATGCCACAGAGGAAGCCGGCT

TTCCCGCCCGGACAAGATAGGAGGAAAGGGAGATGTCACACCCCAAAGTGTCTTGTGATA

CTCCCCACAACCACCCCTAGGTAGATACAGATCAATACCCTCATTT

>2165 len=226 tot-seq=6

GACCCAAGGCAGCTGTGGGATTCCTGGTGTTCTGACTCTTGGAACAGGCTACTCCTAGGG

GAAGGAAGACTGCAGCCACCAAAGCAGCACCCTTGGGACAAAGAAAATGGAAGTGTGTGC

TCTCCTGTGCCTCAGAGGCCCCTGCCTGTGAAAAGAAGGTGACTGTGACCCTTTCAGTGG

AGACACAGATGCTGTACTGGGCTCTGTGGAGAAGTATGGTTTGTCC

>2166 len=226 tot-seq=6

TGTGAGCATCCCCTCTGAGCATCTCTCACCACCTGCCCTGCATCTCTGCTTGGACATCTC

AAAGGCACTCAGCCCCAGCTGGCACCTTCGGGTGCCACTTCCGTGAGCTGGAAACCTGTG

GGGTCCCTACCGACTCCTTCCTAGTCACCTTTCCTTTCCCCTGCCTGGTCCATGGCCCCA

TTGTGCAGATTCACCACCTAAGCATACCCAAGCCCTCTTCTGTCTG

>2167 len=227 tot-seq=6

GCTGAACTGTTTGTGGTCGATCAACTGGTTGCTTAGGTTCTTCAGCCTTAGGAGCTTCTT

CTTTTTCTTTAACTGGTTGAGCCTTAATTTGTTGGCTACGACCTGGTAATTGCTTAGAAG

CAGCTTCTTCAGCCTTAGAATCAATTCCAGTAGCGATTACAGTTACTACCACCTCATCGC

CCAAGTTAGGATTAATTGAAGTACCGAAAATAATGTTTACATCGTCA

>2168 len=229 tot-seq=6

GATTTGTGTGACAACTGGATGAGTGTAGATGACTCAGGGAAGTCAGTGGAGTTATAGTTT

GCATGCATCAGTTAAAAAGGAAAGCCAGGTGTGGTGGTGTGAAGGGGGTGGTTCCAATGC

TAAGGTCCTGTTCTCCAGTTGATTCTTGATCTATCAGTAAAGAAAGTCAGGAACTAATTG

CTGGGTGGAGAGGAGGAGTTTATTTGTCATGCTTTGGAGGGAGAAGAAG

>2169 len=241 tot-seq=6

GTCAGAGTCAGAGGACGAGATGTGGCCATGGAGGTCGGGATGGATGGGAGTGAGGCCGCT

GCAGGAAAGGGCCGTGAGCAAAGGAGTTCTGGCACCTCTGGAGGCTGGCACAGGCAGGAA

GTGGGTTCTGGAAATGAAACACAGCCTCTGCCAACTCCTCGATCTTAGGATCTGTGCCCT

CCAGAACCGTTAGACAACCAGTGTGTGTTGTTTTACACCACCACGTTTGTGGTGATTAGT

T

>2170 len=160 tot-seq=6

TAGGGAATTTTTCACAATGGGCGAAAGCCTGATGGAGCAATGCCGCGTGAGTGAAGAAGG

TTTTCGGATCGTAAAGCTCTGTTGTTGGTGAAGAAGGATAGAGGTAGTAACTGGCCTTTA

TTTGACGGTAATCAACCAGAAAGTCACGGCTAACTACGTG

>2171 len=160 tot-seq=6

TAGGGAATCTTCCACAATGGACGCAAGTCTGATGGAGCAACGCCGCGTGAGTGAAGAAGG

GTTTCGGCTCGTAAAGCTCTGTTGTTGGTGAAGAAGGACAGGGGTAGTAACTGACCTTTG

TTTGACGGTAACTAATTAGAAAGGGACGGCTAACTACGTG

>2172 len=141 tot-seq=6

TGGGGGATATTGCACAATGGGGGAAACCCTGATGGTGCGACGCCGCGTGGGGGAATGAAG

GTCTTCGGATTGTAAACCCCTGTCATGTGGGAGCAAATTAAAAAGATAGTACCACAAGAG

GAAGAGACGGCTAACTCTGTG

>2173 len=101 tot-seq=5

TATCTGTGGACAGAAGAGTAGGCAGGGGGATGTGGCAGTGGGCGCTTTTGAGAAGTTCTC

TCCTGGCGGCTTGAATTTTCTCAGGGAAATGGGAAGTAAAG

>2174 len=104 tot-seq=5

TGTTCATACTCCTGGGACCTCCAGAGTGACTTGAGCCAAGGACATGCTGGCGGTTCCCCA

CACGTAGAAGAGGGTCCCACTATTTGAAAGGTGAGGCAGCCTGC

>2175 len=106 tot-seq=5

GCAAGGAGGGGGAAGACTAGCATAGCCAAGGCTCACCTGATCCCACGGGTCTCTGGAACA

AGTAGTGGGTGTAGGTCCAACATGTAACAGAGTCCTGGATGAGCCC

>2176 len=110 tot-seq=5

CAGGCGTTGCAGGCCGATGGGCTCGCCGGTTTCGCGACACCAGCCGTATTCGCCGCGGGC

CAGCAGTTCGAGGGCATCGTCGATCTTGTCCAAGAGCTTTTTTTCACGCT

>2177 len=110 tot-seq=5

GTGACAGATGTACTCCTCTGACAGCTCTCAGTATCAGCCCAGTGGCTCCCAGCCATTGGC

TCAGTGCAATGGACCGGCAACGCCGCTCACTATAATAACACTCATGCCTG

>2178 len=111 tot-seq=5

CCTCCTTCATCTGGAGACCCTGCAGGTGCCTGAAAAGATAATGCTTGTTCTCTTTAAGAT

CAGTTCCTGCCTTCTCTCACTACCTCCAAACCAATAATGAGGATCATGTTC

>2179 len=112 tot-seq=5

GCCAAAGCGAGTGCGCAGGTCATCTTTTTGCTGAGCTCCCCCTCTTAGGGGGAGCTGGCC

GCTTCGCGGGCTGAGGGGGGCAGCCTCGCCGACCGCCAGGAATCCATCCTCC

>2180 len=112 tot-seq=5

AGAAAAAGAGAAAAGGGAGTACTTATGTGGATGGGAATATAAACCTAGTTCAGCCACTGT

GGAACGCCATTGGAGGTTTCTCAAGGAGCTTAAAGCAGAACTACCATTCGAT

>2181 len=114 tot-seq=5

TGGGGAATCTTGCGCAATGGGGGCAACCCTGACGCAGCGACGCCGCGTGCGGGATGAAGG

CCTTCGGGTCGTAAACCGCTTTTGATTGGGAGCAAGCTTTCGGGTGAGTGCACC

>2182 len=114 tot-seq=5

AAGAGGCGAGAAGTCTCCAGGCCAGGCAGGAGAGGGAGACAGGAAAATTACTTCCCATGG

GGCAGTTCCTTCTGCTCCCCAACCCCACTCCCAGGCAGGAGAACAGGCAGGTTA

>2183 len=115 tot-seq=5

GATCTGGAGGAAGAGAGGATCTCCCAGATTGAGCAGCTGTGCCCACCCCCTTCACTTCTG

AGTTAATGAGGCCAGAGCATCCAAGCCAGGGCCCGGTCTCCGTGAGCCCATCGCT

>2184 len=115 tot-seq=5

AGGCCTAAGGACTGTGTCCTGTGATATTAAGAGTTTAGCGAAAACGGGATCAACCAGCAA

ACAGTATTAAGAAGGATGAACTTGTGAGTTAGGACAATCCGAAAAGGGCAGTGTC

>2185 len=115 tot-seq=5

AAGAGGAGCATCCATCCGCAGAAGAAGAGCCAGTTTACATCGTAGAAGAGATACTGGAAG

AGGTTTGATGTGTTGCCGCCGGGAATTACGTCAGCATTGCTGTAGTAGATCTTGG

>2186 len=116 tot-seq=5

GCCTTAAGTCCCCCTCAGAGTGTAGCCCCAGGAGATCAACACCTTCTTCGGGCTGTACAG

GCTCCTGTGGGGCAGCCAGCATGCCCACATCAAATATTAAGTCTTGCTGTTTGGTC

>2187 len=116 tot-seq=5

GCCCAGCACTCCACAGCCTTCTAGGCTAAGCTCAGCCACTGCCTTTTTTGTGATTCTGCA

GCCACCAAGCTTAGGCCTCAGGCGCTGATGGAGAGATAAAGCATCGTGTGACAGGG

>2188 len=117 tot-seq=5

GAGGAGCCGGCAGGAGGAGAGAAGGGCTGGGGTCTGAGCGTGATCCCAGAAGTGGCCACA

GAGAAGGGACCTCGGGTGGAGGCCACCAGGGCCTGGACTCCCTCCTCCTCCCTGGAG

>2189 len=117 tot-seq=5

TCTAAAATATCAGGTTTCAGGGGTGTTTTGATGCCCACGATGCGTGGCTCTGTGGCCCCT

CCTGCCCTGGTCCTTCAGAAAACTCTGAGTGGTGTCTGGGCTGTGGCCCCTGTCATG

>2190 len=118 tot-seq=5

TTCTGTTTTTGGAAGGCTCAAGTTACCTGGAAGCCCAGAAATTATCTTCCTGAACCTTTG

GAGTGGAGCCCAGCAGGTGTTTAATGGCTTTCCCTGGAGTCCTCTGTTGCCTCCCTGC

>2191 len=119 tot-seq=5

CTGGACCACATAGAGCCTCACAAGCCATAAGTCTTTCGGGTTTTCTGTAATGCACAATGG

GCAGGCTTTGGAAGGGTCAATCCAAGTAGCATCCTGATTTAAGTTTCAAACAGATATCC

>2192 len=119 tot-seq=5

GTAATGCGCGTACGCTTGGTGGTCGTGCACGAGCGCGTAGAGCAGCGCCTCAGAGGGCGA

GTAGGCGGCGGCGCGCCCGCGCTCGTCCCAGTGGAAGGCCTCGCTGGCGCGCATATCCT

>2193 len=122 tot-seq=5

GATAACCCCAACCTCCCTCTGTCTAAACAGGAAGCTCCCACGACTCTCTCCTACTCACCA

CCTCGCAAAAGGCACTTAACCCTTGGCCCGCACAGGTGCAGATGTATCTGCCCATCTACT

CT

>2194 len=122 tot-seq=5

GACAGACAGTGCTGTAGGCCCCCTCCCTGCATCTAATCGATCAGCTTCCATGTAGGATTC

CATTTCATAAAGCTCTCTGCTGCTAAAATAAACTCACAGCGCCAGCCCATCCCCTCCTAG

AA

>2195 len=124 tot-seq=5

CTGTTGGGGAGCAGTCATGGGCAGCAGAGCACCCTAATCCCCTGCAGTGATTCTCTTCAA

ACTCCAGCTACAAAACCCCCAAGTCAGCAGCCCAGTTCCTGGGGAAAGAAAAGAATTTCA

CCTG

>2196 len=124 tot-seq=5

CCTGTGCCTTCCTGTTCAGGGCACTCTGCCCCCTTTCAACCTCCGGTAAAGTTCTTCACA

GGGTAGCCCATGCATTCCAAATGCCTCATACTTGTGGGTCTCATCGTCTACCTTCTGGGT

TTGT

>2197 len=128 tot-seq=5

AGACTGAATGTTCATGAGCCACCCCTTGTGCCTCAGCCTTTTCTGAGCCCTTTAGGGATT

CTTGTTGGATCCATGGAAGGTCTAACTCCTGTCTAAGTCATGCTCCTGTCTAACAATGAG

GACCAGCA

>2198 len=130 tot-seq=5

CAGTGCTAGTGGCAAAGGGACAGACAGCCAAGAAGAAAGGGTACCTCCACTCCCGCTGGT

CCCTCCCCTAGAGGAAGAGGAAGGAGAGAGGAGAACAGCAGCATAAGCAGCTGGCAAAGG

CAAGGAAAGA

>2199 len=132 tot-seq=5

TCCACATCATCTGGGAAGTGGCTTCAAGGACCGGGGGTGTGGTTCGAGGCCATCTGCCAG

GATCCTGGCCCTCCCTGTCTGCCAGCTCCAGACCCTGTGGAGGCACGCGGGTCCGGCCAG

CCCACAGCTCGC

>2200 len=132 tot-seq=5

GCTGGAGAGTGCCTAAGACGAACTGAGGGAAAGGGACAGCCTCCATCACTGCAGCATCAG

TCGGCCGCTTTCCCCTACCAGTGCTAGGGATACTGAGTAGTTTGGACCTGGAGGAATTCC

CTACAGTGCAGT

>2201 len=133 tot-seq=5

GAGAAGGTGTGCCTGGTGTCTGTCTTTCTTATTATAAGAGGAAATTTCCTCGTCCCTCCA

TCACATTGGCCAAACTGGGTCACATGACAACCCGTAGTGGCAACAGAGTCTAGGAAAACT

GTTCTCTAAATTT

>2202 len=134 tot-seq=5

TAGGGAATCTTCGGCAATGGACGGAAGTCTGACCGAGCAACGCCGCGTGATTTAGAAGGC

CTTCGGGTTGTAAAAATCTTTTGTATGGGAAGAAAATGACAGTACCATACGAATAAGGAC

CGGCTAATTACGTG

>2203 len=134 tot-seq=5

TGGGGAATTTTGCACAATGGGGGAAACCCTGATGCAGCGACGCCGCGTGATTTAGAAGGC

CTTCGGGTCGTAAAGCTCTTTTATATGGGAAGATAATGACGGTACCATAAGAAAAAGCCC

CGGCTAACTACGTG

>2204 len=135 tot-seq=5

TGAGGAATATTGGTCAATGGGGGAAACCCTGATGCAGCGACGCCGCGTGAGCGAAGAAGT

ATTTCGGTATGTAAAGCTCTATCAGCAGGGAAGAAAATGACGGTACCTGACTAAGAAGCA

CCGGCTAAATACGTG

>2205 len=135 tot-seq=5

TAGGGAATCTTCCGCAATGGACGCAAGTCTGACGGAGCAACGCCGCGTGAGTGAAGAAGG

ATTTCGGTTCGTAAAGTTCTGTTGCAGGGGAAGATAATGACGGTACCCTGTGAGGAAGCC

CCGGCTAACTACGTG

>2206 len=135 tot-seq=5

TGGGGAATCTTCCGCAATGGACGAAAGTCTGACGGAGCAACGCCGCGTGGAGGAAGAAGG

TCTTCGGATTGTAAACTCCTGTTGTTGAGGAAGATAATGACGGTACTCAACAAGGAAGTG

ACGGCTAACTACGTG

>2207 len=135 tot-seq=5

TGGGGAATATTGGGCAATGGACGCAAGTCTGACCCAGCGACGCCGCGTGAAGGACGAAGG

CATTCGTGTTGTAAACTTCTTTGGAGAGGGACGAAATTGACGGTACCTCTCGAACAAGCC

ACGGCTAACTACGTG

>2208 len=135 tot-seq=5

CTAAGAATATTGGGCAATGGAGGAAACTCTGACCCAGCCATGCCGCGTGAATGAAGAAGG

CCTTCGGGTTGTAAAGTTCTTTTAGTCGTGAAGATGATGACAGTAGCGACAGAAAAAGCA

CCGGCTAACTTCGTG

>2209 len=135 tot-seq=5

TGGGGGATATTGGACAATGGGGGAAACCCTGATGCAGCAACGCCGCGTGAGTGAAGAAGT

ATTTCGGTATGTAAAGCTCTATCAGCAGGGAAGAAGATGACGGTACCTGAGTAAGAAGCC

CCGGCTAACTACGTG

>2210 len=135 tot-seq=5

TGGGGAATATTGCACAATGGGGGAAACCCTGATGCAGCGACGCCGCGTGAGTGAAGACGG

TCTTCGGATTGTAAAGCTCTGTTGCAGAGGACGATAATGACGGTACTCTGTGAGGAAGCC

ACGGCTAACTACGTG

>2211 len=135 tot-seq=5

TGAGGAATATTGGTCAATGGGCGAGAGCCTGATCCAGCCATGCCGCGTGAGTGATGAAGG

CCTTAGGGTTGTAAAGCTCTTTCACCGATGAAGATAATGACGGTAGTCGGAGAAGAAGCC

CCGGCTAACTTCGTG

>2212 len=135 tot-seq=5

TGGGGAATATTGGGCAATGGGGGCAACCCTGACCCAGCGACGCCGCGTGAAGGACGAAGG

CCTTCGGGTTGTAAACTTCTTTGATCGAGGACGATAATGACGGTACTCGAGGAACAAGCC

ACGGCTAACTACGTG

>2213 len=135 tot-seq=5

TGGGGAATATTGGACAATGGGCGCAAGCCTGATCCAGCCATGCCGCGTGAGTGACGAAGG

CCTTAGGGTTGTAAAGCTCTTTTGGCGGGGAAGATAATGACGGTACCCGCAGAATAAGCT

CCGGTTAACTTCGTG

>2214 len=135 tot-seq=5

TGGGGAATATTGCACAATGGGGGAAGCCCTGATGCAGCAACGCCGCGTGAGTGAAGAAGT

ATTTCGGTATGTAAACTTCTATCAGCAGGGAAGATAGTGACGGTACCTGACTAAGAAGCC

CCGGCTAACTACGTG

>2215 len=135 tot-seq=5

ATAACTGGGAAATCCAAATCTTCTCCCAAAGTACCTGTGGTGATTCAGGACAACAGTCTT

CCCACGGGGCCCACTCCACAGATCCACATACTCCAGAGGCCCGCCAGAGTGGTGGGGTCA

ATAGCCTCTGCTCCA

>2216 len=135 tot-seq=5

TGGGGAATATTGCGCAATGGGGGAAACCCTGACGCAGCGACGCCGCGTGCGGGATGAAGG

CCTTCGGGTTGTAAAGCTCTTTTATATGGGAAGATAATGACGGTACCATAAGAAAAAGCC

CCGGCTAACTACGTG

>2217 len=135 tot-seq=5

TGGGGAATATTGCACAATGGGGGAAACCCTGATGCAGCGACGCCGCGTGAGCGAAGAAGG

CCTTCGGGTCGTAAAGCTCTTTTATATGGGAAGATAATGACGGTACCCTGTGAGGAAGCC

CCGGCTAACTACGTG

>2218 len=135 tot-seq=5

TGGGGAATCTTCCGCAATGGGCGCAAGCCTGATGCAGCGACGCCGCGTGAGCGAAGAAGT

ATTTCGGTATGTAAAGCTCTATCAGAAGGGAAGAAAATGACGGTACCTTACTAAGAAGCT

CCGGCTAAATACGTG

>2219 len=135 tot-seq=5

TGGGGAATTTTGCGCAATGGGGGAAACCCTGACGCAGCAACGCCGCGTGCGGGATGAAGG

CCTTCGGGTTGTAAACCGCTTTCAGTAGGGAAGAAAATGACGGTACCTACAGAAGAAGCT

CCGGCTAACTACGTG

>2220 len=135 tot-seq=5

TGGGGGATTTTGCACAATGGAGGAAACTCTGATGCAGCGACGTCGCGTGAACGATGAAGG

CCTTCGGGTTGTAAAGTTCTGTCCTAGAGGAAGATAATGACAGTACTCTAGGAGGAAGCC

CCGGCTAAATACGTG

>2221 len=135 tot-seq=5

TGGGGGATATTGCACAATGGGGGGAACCCTGATGCAGCAACGCCGCGTGAGTGAAGAAGT

ATTTCGGTATGTAAAGCTCTATCAGCAGGGAAGATGATGACGGTACCTGCATAAGAAGCC

CCGGCTAACTACGTG

>2222 len=135 tot-seq=5

TGGGGAATCTTGCACAATGGGCGAAAGCCTGATGCAGCAACGCCGCGTGCGGGAAGAAGG

CCCTAGGGTTGTAAACCGCTTTCAGTAGGGAAGAAAATGACGGTACCTACAGAAGAAGGT

GCGGCCAACTACGTG

>2223 len=135 tot-seq=5

GCGCGCAAATTACCCAATCTTGACACAAGGAGGTAGTGACAATAAATAACAATACCGGGG

TTTTTCAACTCTGGTAATTGGAATGAGTACAATCTAAACCCCTTAACGAGGATCAATTGG

AGGGCAAATCTGGTG

>2224 len=136 tot-seq=5

TCGGGAATATTGCACAATGGAGGAAACTCTGATGCAGTGACGCCGCGTACAGGAAGAAGG

TCTTTGGATTGTAAACTGTTGTCGAAAGGGAAGAAAAAGACAGTACCTTTGGAGGAAGC

TCCGGCAAACTACGTG

>2225 len=136 tot-seq=5

TAGGGAATTTTGCGCAATGGGGGAAACCCTGACGCAGCAACGCCGCGTGATTGATGAAGC

CCTTCGGGGTGTAAAGATCTGTCAGTAGGGACGAATAATGACTGTACCTACAGAGGAAGC

ACCGGCTAACTCCGTG

>2226 len=136 tot-seq=5

TGGGGAATATTGCACAATGGGGGAAACCCTGATGCAGCAACGCCGCGTGAGTGAAGGAGT

GCTTCGGCATGTAAAGCTCTATCAGCAGGGAAGAAGAAGGACGGTACCTGACTAAGAAGC

CCCGGCTAACTACGTG

>2227 len=136 tot-seq=5

AAGTCTGACGGAGCAACGCCGCGTGAGTGAAGAAGGGTTTCGGCTCGTAAAGCTCTGTTG

TTAGTGAAGAAGGACAGAGGTAGTAACTGGCCTTTGTTTGACGGTAATTAATTAGAAAGT

CACGGCTAACTACGTG

>2228 len=136 tot-seq=5

TGGGGAATATTGCACAATGGGCGAAAGCCTGATGCAGCGACGCCGCGTGAGGGAAGACGG

TCTTCGGATTGTAAACCTCTGTCTTTGGGGAAGAAATCAGACGGTACCCAAAGAGGAAGC

TCCGGCTAACTACGTG

>2229 len=136 tot-seq=5

TGGGGAATCTTGCACAATGGGGGAAACCCTGATGCAGCAACGCCGCGTGAGCAAAGAAGT

ATTTCGGTATGTAAAGCTCTATCAGCAGGGAATAGAAAAGAAGGTACCTGACTAAGAAGC

CCCGGCTAACTACGTG

>2230 len=136 tot-seq=5

TGGGGGATATTGCACAATGGGGGAAACCCTGATGCAGCGACACCGCGTGGGTGAAGAAGC

GCTTCGGCGTGTAAAGCCCTATCAGTGGGGAAGAAAAAGGACGGTACCCAAAGAAGAAGC

ACCGGCTAAATACGTG

>2231 len=136 tot-seq=5

CAGGAGGATGCAGGGAGAGACCACGTGATTATGCAGGGGAGAGGCCATGTGAGCACACAG

GGAGAGGCTACAGGAGCGTGCAGGGAAAGGGTGCCATCTGCAAGCCACAGAGAGGCATGA

GGAGGAACACACCCTG

>2232 len=136 tot-seq=5

TCGGGAATATTGCGCAATGGAGGAAACTCTGACGCAGTGACGCCGCGTATAGGAAGAAGT

TTTTCGGAATGTAAACTATTGTCGTTAGGGAAGAGAAAGGACGGTACCTAAGGAGGAAGC

CCCGGCTAACTATGTG

>2233 len=136 tot-seq=5

TGGGGAATATTGCGCAATGGGGGAAACCCTGACGCAGCAACGCCGCGTGAGTGAAGAAGG

CCTTCGGGTTGTAAAGCTCTTTAATCAGGGACGAAGAATGACGGTACCTGAAGAATAAGC

TCCGGCTAACTACGTG

>2234 len=137 tot-seq=5

TGGGGAATTTTCCACAATGGGCGAAAGCCTGATGGAGCAATACCGCGTGAGGGATGAAGC

ATCGTGGTGTGTAAACCTCTTTTCTTAAGGAAGAATCAATGACGGTACTTAAGGAATAAG

CACCGGCTAACTCTGTG

>2235 len=137 tot-seq=5

TGGGGGATATTGCACAATGGAGGAAACTCTGATGCAGCAACGCCGCGTGAGGGAAGAAGG

TCTTCGGATTGTAAACCTTTGTTTTTGGTGACGAACAAATGACGGTAGCCAAAGAGGAAG

CCACGGCTAACTACGTG

>2236 len=137 tot-seq=5

TGGGGAATATTGCACAATGGGCGAAAGCCTGATGCAGCGACGCCGCGTGAACGAAGAAGT

ATTTCGGTATGTAAAGTTCTATCAGCAGGGAAGAAAAAATGACGGTACCTGACTAAGAAG

CTCCGGCTAAATACGTG

>2237 len=137 tot-seq=5

TGGGGGATATTGCACAATGGGCGAAAGCCTGATGCAGCAACGCCGCGTGAGGGAAGACGG

TTTTCGGATTGTAAACCTCTGTTCTAAGTGACGAACAAATGACGGTAGCTTAGGAGAAAG

CCCCGGCTAACTACGTG

>2238 len=138 tot-seq=5

TGGGGAATATTGGGCAATGGGCGAAAGCCTGACCCAGCGACGCCGCGTGAAGGAAGAAGG

CCTTCGGGTTGTAAACTTTAGTAAGCAGGGAAGAAGAAGGTGACGGTACCTGCAGAGTAA

GCCACGGCTAACTACGTG

>2239 len=138 tot-seq=5

TGGGGGATATTGCACAATGGGCGAAAGCCTGATGCAGCAACGCCGCGTGAGGGAAGACGG

TTTTCGGATTGTAAACCTCTTTTCTTAGTGAAGAAGAAAATGACGGTAGCTAAGGAATAA

GCATCGGCTAACTACGTG

>2240 len=138 tot-seq=5

TGGGGAATATTGCACAATGGGGGAAACCCTGATGCAGCAACGCCGCGTGAAGGAAGACGG

TTTTCGGATTGTAAACTTCTTTTCTTTGTGAAGAAACAAATGACGGTAGCAAAGGAATAA

GCATCGGCTAACTACGTG

>2241 len=138 tot-seq=5

TGGGGAATATTGGGCAATGGGCGCAAGCCTGACCCAGCGACGCCGCGTGAGGGAAGAAGG

TCTTCGGATTGTAAACCTTTGTAGACAGGGAAGAAGCAAGTGACGGTACCTGTAAAGTAA

GCCACGGCTAACTACGTG

>2242 len=138 tot-seq=5

TGGGGAATATTGCACAATGGGCGAAAGCCTGATGCAGCAACGCCGCGTGAGTGAAGAAGG

CTTTCGAGTCGTAAAACTCTGTCGTATGGGAAGAAAAAAATGACGGTACCATACAAGAAA
GCCCCGGCTAACTACGTG
>2243 len=138 tot-seq=5
TGGGGAATATTGGGCAATGGGCGAAAGCCTGACCCAGCAACGCCGCGTGAAGGAAGAAGG
CCTTCGGGTTGTAAACTTCTTTTGTGGGGAACGAAGGATGTGACGGTACCTCACGAATAA
GCCACGGCTAACTACGTG
>2244 len=138 tot-seq=5
TGGGGAATATTGCGCAATGGGGGCAACCCTGACGCAGCAACTCCGCGTGCAGGAAGAAGG
TCTTCGGATTGTAGACTGTTGTCGCAAGGGAAGAAGACAGTGACGGTACCTTGTGAGAAA
GTCACGGCTAACTACGTG
>2245 len=138 tot-seq=5
TGGGGAATATTGCACAATGGGCGAAAGCCTGATGCAGCAACGCCGCGTGAAGGAAGAAGG
CCTTCGGGTCGTAAACTTCTGTCCTATGGGAAGAAAAAAATGACGGTACCATAGGAGGAA
GCCCCGGCTAACTACGTG
>2246 len=138 tot-seq=5
TGGGGAATATTGCACAATGGGGGAAACCCTGATGCAGCAACGCCGCGTGAAAGAAGAAGG
CTTTCGGGTCGTAAACTTCTATCAATAGGGACGAAGAAAGTGACGGTACCTAAATAAGAA
GCCCCGGCTAACTACGTG
>2247 len=138 tot-seq=5
TGGGGAATATTGGGCAATGGGCGCAAGCCTGACCCAGCGACGCCGCGTGAAGGATGAAGG
CTTTCGGGTTGTAAACTTCTTTTCTCAGGGACGAAAGAAATGACGGTACCTGAGGAATAA
GCCACGGCTAACTACGTG
>2248 len=138 tot-seq=5
TGGGGAATTTTCCGCAATGGGCGAAAGCCTGACGGAGCAATCTCGCGTGAGGGATGACTG
CCTATGGGTTGTAAACCTCTTTTTTCAGGGAGGAATCAATGACGTGTACCTGAAGAATAA
GCATCGGCTAACTCCGTG
>2249 len=140 tot-seq=5
TGGGGAATATTGCACAATGGACGCAAGTCTGATGCAGCGACGCCGCGTGGGGGATGAAGG
CTTTCGGGTTGTAAACTCCTTTCAGTACAGAAGAAGCGAAAGTGACGGTATGTGCAGAAG

AAGCACCGGCTAACTACGTG

>2250 len=140 tot-seq=5

TGGGGAATATTGCACAATGGGCGCAAGCCTGATGCAGCAACGCCGCGTGCGGGAAGACGG

CCTTCGGGTTGTAAACCGCTTTCATCTATGACGAAGCTAACGTGACGGTAATAGAAGAAG

AAGCACCGGCTAACTACGTG

>2251 len=141 tot-seq=5

GAAATATTCTCAGAAGAAGTGATATCTAAGCTGGTGAAGAGTACGTAGGAGTTAGCCAGA

AAAAGAGTAGGGGGAAGAATATTCCAAGCAGAGAAAACAGCATATACAAAAGCTCAAAGG

AGAAAGAAAAGATGGTATTCT

>2252 len=142 tot-seq=5

TAGGGAATCTTCGGCAATGGGGGGAACCCTGACCGAGCGACGCCGCGTGCGGGATGACGG

CCTTCGGGTTGTAAACCGCTTTTGATCGGGAGCAAGCCTTCGGGTGAGTGTACCTTTCGA

ATAAGCACCGGCTAACTACGTG

>2253 len=142 tot-seq=5

TGGGGAATATTGGACAATGGAGGGAACTCTGATCCAGCAATTCTGTGTGTGTGAAGAAGG

CCTTCGGGTTGTAAACCGCTTTCAGCAGGGACGAGGCCGCAAGGTGACGGTACCTGCAGA

AGAAGCCCCGGCTAATTACGTG

>2254 len=142 tot-seq=5

TGGGGAATATTGCACAATGGGGGAAACCCTGATGCAGCGACGCAGCGTGCGGGATGACGG

CCTTCGGGTTGTAAACCGCTTTCAGCAGGGAAGAAGCCTCACGGTGACGGTACCTGCAGA

AGAAGGACCGGCTAACTACGTG

>2255 len=143 tot-seq=5

TGGGGAATATTGGACAATGGGCGCAAGCCTGATTCAGCCATGCCGCGTGAGTGATGAAGG

CCTTAGGGTTGTAAACCGCTTTTGATTGGGAGCAAGCCTTTTGGGTGAGTGTACCTTTCG

AATAAGCGCCGGCTAACTACGTG

>2256 len=144 tot-seq=5

TGGGGAATATTGCACAATGGGCGCAAGCCTGATGCAGCGACGCCGCGTGGGGGATGAAGG

CCTTCGGGTTGTAAACTCCTTTCGCTACCGACGAAGCCTCTTGGGGTGACGGTAGGTGGA

GAAGAAGCACCGGCTAACTACGTG

>2257 len=146 tot-seq=5

AATGGACGAAAGTCTGACGGAGCAACGCCGCGTGAGTGATGAAGGGTTTCGGCTCGTAAA

GCTCTGTTGTTAGGGAAGAACAAGTATCGTTCGAATAGGGCGGTACCTTGACGGTACCTA

ACCAGAAAGCCACGGCTAACTACGTG

>2258 len=147 tot-seq=5

GGGAAGAGGGAGAGGCACCAGCAGGAACTCAAAACTGCAGCAGCAGCAGCAGCAGCAGCA

GCAGCAGCAAGAAGAGCAGAAGCACCAGAAGCAGCAACGGCAGAAGCAGCAGCAACAGCA

GCAGAAGCAGGGACAGAATCACCAGCA

>2259 len=147 tot-seq=5

CAGTGTGGGAAATGAGTCGGCCATCTCACAAGTCATAGAAGGAGAGATTTGGTGGCTGAA

CCCTCCATGTGATCGGAGCCCCAGGGGCTAAGTTACTGTAGAGAGCAGACTTCATAGATC

CCATTGCTGGAACCAAGCATATGATAA

>2260 len=148 tot-seq=5

TCCCCAGCCGGCACTCACTCGGTCTGCAGCAGCTCAGGGTTGGGCACGCACTGGAGCCGG

TGCGAGAGCAGCTGGAAGGCGCTGCTCTGAGGCAGGAGCATGAGCAGGCCGTAGAGGGCC

TTGATCAGGTAGGGGTTGTTCTTCACGT

>2261 len=152 tot-seq=5

TGGGGAATCTTGCGCAATGGGCGGAAGCCTGACGCAGCAACGCCGCGTGGGGGATGACGG

CCTTCGGGTTGTAAACCTCTTTCGCGATCGACGAAGCCTACGTTTCTGCGTGGGTGACGG

TAGGTCGAGAAGAAGCACCGGCCAACTACGTG

>2262 len=152 tot-seq=5

TGGGGAATATTGCACAATGGGGGAAACCCTGATGCAGCGACGCCGCGTGGAGGGTGTAGG

CCTTCGGGTTGTGAACTCCTTTTTCTCGCGAAAAAGGCACAGTTTTGGCTGTGTTGATGG

TAGTGGGGGAAGAAGCGCCGGCTAACTACGTG

>2263 len=154 tot-seq=5

TGGGGAATCTTGCACAATGGGCGAAAGCCTGATGCAGCGACGCCGCGTGCGGGATGAAGG

CCTTCGGGTTGTAAACTGCTTTTATATGGGGATAAAGTGGGGAACGTGTTCCCTTTTGCA

GGTACCATATGAATAAGGACCGGCTAATTACGTG

>2264 len=154 tot-seq=5

TGAGGAATATTGGTCAATGGTCGAGAGACTGAACCAGCCATGCCGCGTGAAGGTGACGGC

CCTATGGGTCTTAAACTTCTTTTGTAAGGGAGCAATAAGGTTCACGTGTGGACCGATGAG

AGTACCTTACGAATAAGCATCGGCTAACTCCGTG

>2265 len=155 tot-seq=5

TGAGGAATATTGGTCAATGGACGCAAGTCTGAACCAGCCACGTCGCGTGAAGGAAGACGG

CCCTACGGGTTGTAAACTTCTTTTGTAAGGGAATAAAGTGAGCCACGTGTGGCTTTTTGC

ATGTACCTTACGAATAAGGATCGGCTAACTCCGTG

>2266 len=155 tot-seq=5

TGGGGAATCTTGCACAATGGGCGAAAGCCTGATGCAGCGACGCCGCGTGCAGGATGACGG

CCCTATGGGTTGTAAACTGCTTTTATATGGGGATAAAGTGGGGAACGTGTTCCCTTTTGC

AGGTACCATATGAATAAGGACCGGCTAATTCCGTG

>2267 len=155 tot-seq=5

TAAGGAATATTGGTCAATGGACGCAAGTCTGAACCAGCCATGCCGCGTGGAGGATGAAGG

TCCTCTGGATTGTAAACTTCTTTTATCTGGGAAGAAACCCATGGATTCTTCTGTGGTTGA

CGGTACCAGATGAATAAGCACCGGCTAACTCCGTG

>2268 len=155 tot-seq=5

TGGGGAATATTGCACAATGGGCGCAAGCCTGATGCAGCCATGCCGCGTGTATGAAGAAGG

CTCTATGGGTCGTAAACTTCTTTTATATAAGAATAAAGTGCAGTATGTATACTGTTTTGT

ATGTATTATATGAATAAGGATCGGCTAACTCCGTG

>2269 len=155 tot-seq=5

TGAGGAATATTGGTCAATGGGCGAGAGCCTGAACCAGCCAAGTCGCGTGAAGGATGAAGG

TTCTATGGATTGTAAACTTCTTTTGTCCGAGGGTAAAGTGTGCCACGTGTGGTATATTGC

AAGTATCGGACGAATAAGCATCGGCTAACTCCGTG

>2270 len=155 tot-seq=5

TGAGGAATATTGGTCAATGGGCGAGAGCCTGAACCAGCCAAGTCGCGTGAGGGATGACGG

CCCTACGGGTTGTAAACCTCTTTTATAAGGGAATAAAGGACATCACGTGTGGTGTTTTGC

ATGTACCTTATGAATAAGCATCGGCTAACTCCGTG

>2271 len=155 tot-seq=5

TGAGGAATATTGGTCAATGGTCGAAGGACTGAACCAGCCAAGTCGCGTGAGGGAAGACAG

CCCTATGGGTTTTAAACCTCTTTTGTAATGGAAGAATAAGTCTTACGTGTAAGATGATGC

CAGTACATTACGAATAAGCATCGGCTAACTCCGTG

>2272 len=155 tot-seq=5

TGAGGAATATTGGACAATGGGCGGAAGCCTGATCCAGCCATGCCGCGTGAAGGATTACGG

TCCTATGGATTTTAAACTTCTTTTGTACGTGAGCAATAAGGGTCACGCGTGGCCCGATGA

GAGTAGCGTACGAATAAGCATCGGCTAACTCCGTG

>2273 len=155 tot-seq=5

TGAGGAATATTGGTCAATGGGCGCAAGCCTGAACCAGCCAAGTAGCGTGCAGGACGACGG

CCCTATGGGTTGTAAACTGCTTTTGTACGGGGATAAAACGGCGGACGCGTCCGTCCTTGC

AGGTACCGTACGAATAAGGACCGGCTAATTCCGTG

>2274 len=155 tot-seq=5

TGAGGAATATTGGTCAATGGTCGGGAGACTGAACCAGCCAAGCCGCGTGAGGGATGAAGG

TGCCAAGCATTGTAAACCTCTTTTGTCAGGGAACAAAGGCATCCACGAGTGGGTGTGTGA

GTGTACCTGAAGAAAAAGCATCGGCTAACTCCGTG

>2275 len=155 tot-seq=5

TGGGGAATCTTCCGCAATGGACGAAAGTCTGACGGAGCAACGCCGCGTGAGTGAAGACGG

CCTTCGGGTTGTAAAGCTCTGTTGTAAGGGAAGAACGGTAGATAGAGAATATCTAAGTGA

CGGTACCTTACCAGAAAGCCACGGCTAACTACGTG

>2276 len=155 tot-seq=5

TGAGGAATATTGGTCAATGGGCGAAAGCCTGAACCAGCCAAGTAGCGTGCAGGATGACGG

CCCTCTGGGTTGTAAACTGCTTTTAGTCGGGAATAAAATGCGGCACGTGTGCCGCTTTGC

ATGTACCGTCAGAAAAAGGACCGGCTAATTCCGTG

>2277 len=155 tot-seq=5

TGGGGAATATTGGACAATGGAGGGAACTCTGATCCAGCCAAGTAGCGTGCAGGATGACGG

CCCTATGGGTTGTAAACTGCTTTTATATGGGAATAAAGTGAGGGACGTGTCCCTTATTGC

ATGTACCATACGAATAAGGACCGGCTAATTCCGTG

>2278 len=155 tot-seq=5

CAGGTGGATCACCTGAGATCAGGAGTTCGAGACCAGCCTGACCAACATGGAGAAACCCTG

TCTCTACTAAAAATACAAAATTAGTACCAGGTGCAGTGGGGGGGTGACTGTAGTCCCAGC

TACTCGGGAGGCTGAGGCAGGAGAATCGCTTGAAC

>2279 len=155 tot-seq=5

TGAGGAATATTGGACAATGGGTGAGAGCCTGATCCAGCCATCCCGCGTGAAGGACGACGG

CCCTATGGGTTGTAAACTTCTTTTGTATAGGGATAAACCTAGATACGTGTATCTAGCTGA

AGGTACTATACGAATAAGCACCGGCTAACTCCGTG

>2280 len=155 tot-seq=5

TGAGGAATATTGGTCAATGGGCGAGAGCCTGAACCAGCCAAGTAGCGTGAAGGATGACTG

CCCTATGGGTTGTAAACTTCTTTTACAAAGGAATAAAGTCGGGTATGGATACCCGTTTGC

ATGTACTTTATGGATAAGGATCGGCTAACTCCGTG

>2281 len=155 tot-seq=5

TGGGGAATATTGCACAATGGGCGAAAGCCTGATGCAGCGACGCCGCGTGAAGGAAGGAGG

TCCTATGGATCGTAAACTTCTTTTGACGCAGAGCAATAAAGTGTACGAGTACACTGATGA

GAGTATGCGTCGAATAAGCATCGGCTAACTCCGTG

>2282 len=155 tot-seq=5

TGAGGAATATTGGACAATGGGTGCAAGCCTGATCCAGCCATCCCGCGTGAAGGATTAAGG

TCCTATGGATTGTAAACTTCTTTTATACAGGGATAAACCTACTTACGTGTAAGTAGCTGA

AGGTACTGTATGAATAAGCACCGGCTAACTCCGTG

>2283 len=155 tot-seq=5

TAAGGAATATTGGTCAATGGACGGAAGTCTGAACCAGCCATGCCGCGTGAAGGATGAAGG

TCCTCTGGATTGTAAACTTCTTTTATGTGGGAAGAAACCACTCCTTTCTAGGGGTGTTGA

CGGTACCATAGGAATAAGCACCGGCTAACTCCGTG

>2284 len=155 tot-seq=5

TGAGGAATATTGGACAATGGGCGCAAGCCTGATCCAGCCATGCCGCGTGCAGGATGACGG

TCCTATGGATTGTAAACTGCTTTTGTACGGGAAGAAGCACCTCTTCGTGAAGAGGCTTGA

CGGTACCGTAAGAATAAGGATCGGCTAACTCCGTG

>2285 len=155 tot-seq=5

TGAGGAATATTGGTCAATGGGCGAGAGCCTGAACCAGCCAAGTCGCGTGAGGGATGACGG

TCCTACGGATTGTAAACCTCTTTTGTCGGGGAACAACAGGGGGCACGCGTGCCTTTGTGA

GTGTACCCGAAGAAAAAGCATCGGCTAACTCCGTG

>2286 len=155 tot-seq=5

TGAGGAATATTGGTCAATGGGCGCGAGCCTGAACCAGCCAAGTAGCGTGAAGGATGAAGG

TCCTACGGATTGTAAACTTCTTTTACACGGGAATAAAGTGGCCTACGTGTAGGCTTTTGC

ATGTACCGTATGAATAAGCATCGGCTAACTCCGTG

>2287 len=156 tot-seq=5

TGAGGAATATTGGTCAATGGCCGGGAGGCTGAACCAGCCAAGTCGCGTGAGGGATGACGG

CCCTACGGGTTGTAAACCTCTTTTGCCGGGGAGCAAGAACCGCCTCGCGCAGGCGGACTG

AGAGTACCCGGAGAAAAAGCATCGGCTAACTCCGTG

>2288 len=156 tot-seq=5

TGAGGAATATTGGTCAATGGACGAGAGCCTGAACCAGCCAAGTCGCGTGAAGGAAGACTG

CCCGCAAGGGTTGTAAACTTCTTTTGTATGGGATTAAAGTCACCTACGTGTAGGTGTTTG

CAGTTACCACATGAATAAGGACCGGCTAATTCCGTG

>2289 len=156 tot-seq=5

TGAGGAATATTGGTCAATGGGCGCAAGCCTGAACCAGCCAAGTCGCGTGAGGGATGACGG

TCCTATGGATTGTAAACCTCTTTTGTCGGGGAGCAAGGCCCGCCACGTGTGGCGGGAAGG

AGAGTACCCGAAGAAAAAGCATCGGCTAACTCCGTG

>2290 len=158 tot-seq=5

TGAGGAATTTTCCGCAATGGGCGCAAGCCTGACGGAGCGACGCCGCGTGAGGGATGAAGG

TTTTCGGATCGTAAACCTCTTTTATTAAGGAAAAAGGTCCGTATGCCAACCATGCGGGCT

TGATGGTACTTATTGAATAAGCCCCGGCTAACTACGTG

>2291 len=159 tot-seq=5

TGGGGAATCTTGCGCAATGGACGAAAGTCTGACGCAGCGACGCCGCGTGGGTGATGAAGG

CCTTCGGGTTGTAAAGCCCTGTGGGGAGAGACGAATAAGTGCGGGCTAATACTCCGCATG

ATGACGGTATCTCCTTAGCAAGCACCGGCTAACTCTGTG

>2292 len=159 tot-seq=5

TGGGGAATATTGCGCAATGGGGGAAAGCCTGATCCAGCAATTCCGCATGTGTGAAGAAGG

CCTTCGGGTTGTAAAGCACTTTAGGTTGGGAAGAAAAAGCTTCGGCGAATATCCGGAGTG

TTGACGGTACCAACAGAATAAGCACCGGCTAACTCTGTG

>2293 len=159 tot-seq=5

TGGGGAATATTGGACAATGGGCGAAAGTCTGATGGAGCAATACCACGTGAACGATGAAGG

TCTTCTGATTGTAAAGTTCTTTTATTTAGGAAAAAAAGCGCGCTAGGAAATGAGCGCGCC

TTGATGGTACTAATTGAATAAGTGACAGCTAACTATGTG

>2294 len=159 tot-seq=5

TAGGGAATCTTGCGCAATGGGCGAAAGCCTGACGCAGCGACGCCGCGTGAGTGATGAAGG

CCCTCGGGTTGTAAAGCTCTGTGGGGAGAGAAGAATAAGCACGAGTTAATACCTCGCGTG

ATGACGGTATCTCCTTAGCAAGCACCGGCTAACTCTGTG

>2295 len=160 tot-seq=5

ATGGGGACAGTCACAATTGTCCTTTCAAGCTGAGACTCTCAGATTCCCACACAGAAGGAG

AGGAAGAAAAGTCTTTCTCAGGATGAACAGATGTTCTGTCCACATGAGGAGGAGGTGGTA

GACGGTGCCAGAACCCTTCCCAGGCTCTTTCCCAGGTCAC

>2296 len=160 tot-seq=5

TGGGGAATTTTGGACAATGGGGGGAACCCTGGTCCAGCCATCCCGCGTGTGCGATGAAGG

CCTTCGGGTCGTAAAGCACTTTTGGCAGGAAAGAAACGTCATGGGCTAATACCCCGTGAA

ACTGACGGTACCTGCAGAATAAGCACCGGCTAACTACGTG

>2297 len=160 tot-seq=5

TAGGGAATCTTCCACAATGGGCGAAAGCCTGATGGAGCAACGCCGCGTGTGTGATGAAGG

CTTTCGGGTCGTAAAGCACTGTTGTATGGGAAGAAAAGTTAAGGTAGGGAATGACCTTAG

CCTGACGGTACCATACCAGAAAGGGACGGCTAAATACGTG

>2298 len=160 tot-seq=5

TAGGGAGTTTTCGGCAATGGGCGAAAGCCTGACCGAGCAACGCCGCGTGAGTGATGAAGG

TCTTCGGATTGTAAAGCTCTGTTACGAGGGACGAATTACTGGGTCAGGAAATGGGCCCGG

CTTGACGGTACCTCGCCAGAAAGTGACGGCTAACTACGTG

>2299 len=160 tot-seq=5

TGGGGAATATTGGACAATGGGCGAAAGCCTGATCCAGCCATGTCGCGTGTGTGAAGAAGG

TCTTCGGATTGTAAAGCACTTTAAGTTGGGAGGAAGGGTTGTACGCTAATACCGTGCAAT

TTTGACGTTACCAACAGAATAAGCACCGGCTAACTCTGTG

>2300 len=160 tot-seq=5

TCGGGAATTTTGCACAATGGCCGAAAGGCTGATGCAGCAACGCCGCGTGGGGGAAGAAGG

CCTTCGGGTTGTAAACCCCTGTTGACCGGGAAGAACGGCCTTATCAGGAAATGGGTAGGG

TTTGACGGTACCGGTAGAGGAAGCCCCGGCTAACTACGTG

>2301 len=160 tot-seq=5

TGGGGAATATTGCACAATGGGGGAAACCCTGATGCAGCCATGCCGCGTGTATGAAGAAGG

CCTTCGGGTTGTAAAGTACTTTCAGTTGTGAGGAAGGCGTTGAAGTTAATAGCTTCAGCG

TTTGACGTTAGCAACAGAAGAAGCACCGGCTAACTCCGTG

>2302 len=160 tot-seq=5

TAGGGAATCTTCCACAATGGATGAAAGTCTGATGGAGCAACGCCGCGTGAGTGAGGAAGG

TTTTCGGATCGTAAAGCTCTGTTGTTGGTGAAGAAGGATAGAGGTAGTAACTGACCTTTG

TTTGACGGTAATCAATTAGAAAGTCACGGCTAACTACGTG

>2303 len=160 tot-seq=5

TGGGGAATATTGCACAATGGGGGAAACCCTGATGCAGCCATGCCGCGTGTGTGAAGAAGG

CCTTCGGGTTGTAAAGCACTTTCAGTAGGGAGGAAAGGTTGTAAGTTAATACCTTGCAGC

TGTGACGTTACCTACAGAAGAAGGACCGGCTAACTCCGTG

>2304 len=160 tot-seq=5

TGGGGAATTTTGGACAATGGGCGAAAGCCTGATCCAGCCATTCCGCGTGAGTGAAGAAGG

CCTTCGGGTTGTAAAGCTCTTTCGCAAGGGAAGAAAACTTATATTCTAATAAAGTATGAG

GATGACGGTACCTTGATAAGAAGCACCGGCTAACTACGTG

>2305 len=160 tot-seq=5

TAGGGAATCTTCGGCAATGGACGGAAGTCTGACCGAGCAACGCCGCGTGAGTGAAGAAGG

TTTTCGGGTTGTAAAGTACTTTCAGCGGGGAGGAAGGGAGTAAAGTTAATACCTTTGCTC

ATTGACGTTACCCGCAGAAGAAGCACCGGCTAACTCCGTG

>2306 len=160 tot-seq=5

TAGGGAATATTCCACAATGAGCGAAAGCTTGATGGAGCGACACAGCGTGCACGATGAAGG

TCTTCGGGTTGTAAAGTACTTTCAGCGGGGAGGAAGGGAGTAAAGTTAATACCTTTACTC

ATTGACGTTACCCGCAGAAGAAGCACCGGCTAACTCCGTG

>2307 len=160 tot-seq=5

TAGGGAATCTTCCACAATGGGCGCGAGCCTGATGGAGCAACACCACGTGAGTGAAGAAGG

GTTTCGGCTCGTAAAGCTCTGTTGGTAGTGAAGAAAGATAGAGGTAGTAACTGGCCTTTA

TTTGACGGTAATTACTTAGAAAGTCACGGCTAACTACGTG

>2308 len=160 tot-seq=5

TAGGGAATCTTCCACAATGGACGCAAGTCTGATGGAGCAACGCCGCGTGTGTGAAGAAGG

TCTTCGGATTGTAAAGCACTTTAAGTTGGGAGGAAGGGCAGTTACCTAATACGTGATTGT

TTTGACGTTACCGACAGAATAAGCACCGGCTAACTCTGTG

>2309 len=160 tot-seq=5

TGGGGAATATTGCACAATGGGCGCAACCCTGATCCAGCCATGCCGCGTGCGGGATGAAGG

CCTTCGGGTTGTAAACCGCTTTTGTCAGAGACGAAAAGGGATGTACGAATAATACGTTCC

GCTGACGGTATCTGAAGAACAAGCACCGGCTAACTACGTG

>2310 len=160 tot-seq=5

TGGGGAATATTGCACAATGGGCGCAAGCCTGATGCAGCGACGCCGCGTGTGTGAAGAAGG

TCTTCGGATTGTAAAGCACTTTAAGTTGGGAGGAAGGGCAGTTACCTAATACGTGATTGT

TTTGACGTTACCGACAGAATAAGCACCGGCTAACTCTGTG

>2311 len=160 tot-seq=5

TAGGGAATTTTCGGCAATGGGGGGAACCCTGACCGAGCAACGCCGCGTGAAGGAAGAAGG

TATTCGTATCGTAAACTTCTGTCATAGAGGAAGAACGGCAATAGTAGGGAATGACTATTG

AGTGACGGTACTCTAAGAGGAAGCCACGGCTAACTACGTG

>2312 len=160 tot-seq=5

TAGGGAATTTTCGGCAATGGGGGAAACCCTGACCGAGCAACGCCGCGTGAGTGATGAAGG

CCCTAGGGTCGTAAAGCTCTGTTGTAAGGGAAGAATACTTAGGAGAGGGAATGCTCTTAA

GCTGACGGTACCTTACCAGAAAGTGACGGCTAACTACGTG

>2313 len=160 tot-seq=5

TGGGGAATATTGCACAATAGGCGCAAGCCTGATCCAGCCATGCCGCGTGTGTGAAGAAGG

TCTTCGGATTGTAAAGCACTTTAAGTTGGGAGGAAGGGCTCTTGGCTAATACCCGAGGGT

TTTGACGTTACCAACAGAATAAGCACCGGCTAACTACGTG

>2314 len=160 tot-seq=5

TGGGGAATATTGCACAATGGGCGCAAGCCTGATGCAGCGACGCCGCGTGAATGAAGAAGG

CCTTCGGGTCGTAAAATTCTGTTGTCAGAGAAGAACGTGCGTGAGAGTAACTGTTCACGT

ATTGACGGTATCTGACCAGAAAGCCACGGCTAACTACGTG

>2315 len=160 tot-seq=5

TGGGGAATATTGCGCAATGGGGGAAACCCTGACGCAGCGACGCCGCGTGCGGGATGAAGG

CCTTCGGGTTGTAAAATTCTGTTGTCAGAGAAGAACGTGCGTGAGAGTAACTGTTCACGT

ATTGACGGTATCTGACCAGAAAGCCACGGCTAACTACGTG

>2316 len=160 tot-seq=5

TGGGGAATTTTGGACAATGGGCGAAAGCCTGATCCAGCAATGCCGCGTGTGTGAAGAAGG

TTTTCGGATCGTAAAGCTCTGTTGTTGGTGAAGAAGGATAGAGGTAGTAACTGGCCTTTA

TTTGACGGTAATCAACCAGAAAGTCACGGCTAACTACGTG

>2317 len=160 tot-seq=5

CTAAGAATATTCCGCAATGGACGGAAGTCTGACGGAGCGACGCCGCGTGGATGAAGAAGG

CTGAAAAGTTGTAAAATCCTTTTGTTGATGAAGAATAAGGGTGAGAGGGAATGCTCATCT

GATGACGGTAATCGACGAATAAGCCCCGGCTAATTACGTG

>2318 len=160 tot-seq=5

TAGGGAATCTTCCACAATGGGCGAAAGCCTGATGGAGCAATACCGCGTGAGTGAAGAAGG

TCTTCGGATCGTAAAGCTCTGTTGTTAGAGAAGAACAAGAGGTGTAGTAACTGGCACCTC

CTTGACGGTATCTAACCAGAAAGTCACGGCTAACTACGTG

>2319 len=160 tot-seq=5

TAGGGAATCTTCGGGAATGGGGGCAACCCTGACGCAGCCATGCCGCGTGAATGAAGAAGG

CCTTCGGGTTGTAAAGTTCTTTCGGTAGCGAGGAAGGCATCTAGTTTAATAGACTAGGTG

ATTGACGTTAACTACAGAAGAAGCACCGGCTAACTCCGTG

>2320 len=160 tot-seq=5

TAGGGAATTTTCGGCAATGGGGGAAACCCTGACCGAGCAATGCCGCGTGAATGATGAAAT

TCTTCGGAATGTAAAATTCTGTTGTGAGGGAAGAAAGGTAGTATCAGGAAATGGGTATTA

CTTGACGGTACCTTACTAGAAAGCCCCGGCTAACTATGTG

>2321 len=160 tot-seq=5

TGGGGAATCTTCCGCAATGGGCGCAAGCCTGACGGAGCAACGCCGCGTGAGTGAAGAAGG

TTTTCGGATCGTAAAGCTCTGTTGAGAGGGACGAGAGATATAATCAGGAAATGGGTTATA

GAGGACGGTACCTTTCGAGGAAGCCACGGCTAACTACGTG

>2322 len=160 tot-seq=5

TCGAGGATCTTTCGCAATGGGCGCAAGCCTGACGGAGCGACGCTGTGTGAGTGATGAAGG

CCTTCGGGTTGTAAAGCTCTTTTGTTTGGGAACAAGAAAGATTCGTGAATAACGAGTCAA

TTTGAGGGTACCAGAAGAAAAAGTACCGGCTAACTACGTG

>2323 len=160 tot-seq=5

TGGGGAATATTGCGCAATGGGGGAAACCCTGACCGAGCAACGCCGCGTGAGTGAAGAAGG

TTTTCGGATCGTAAAACTCTGTTGTTAGAGAAGAACAAGGACGTTAGTAACTGAACGTCC

CCTGACGGTATCTAACCAGAAAGCCACGGCTAACTACGTG

>2324 len=160 tot-seq=5

TCGAGAATCTTTTGCAATGGGCGCAAGCCTGACGAAGCGACTCTGTGTGAGCGATGAAGG

CCTTTGGGTTGTAAAGCTCTTTCGGTTAGGAACAAGAGAGGTGAGTGAATAACTTGCCAA

TTTGAGCGTACTAGCTAAAGAAGCACCGGCTAACTCCGTG

>2325 len=160 tot-seq=5

TGGGGAATTTTGGACAATGGGCGCAAGCCTGATCCAGCCATGCCGCGTGCGGGAAGAAGG

CCTTCGGGTTGTAAACCGCTTTTGTCAGGGAAGAAACGCTCTGGGCTAATACCCTGGGGT

AATGACGGTACCTGAAGAATAAGCACCGGCTAACTACGTG

>2326 len=160 tot-seq=5

TAGGGAATCTTCCGCAATGGACGCAAGTCTGACGGAGCAACGCCGCGTGAGTGATGAAGG

TTCTCGGATCGTAAAGCTCTGTTGCCCTAGACGAAAAGCAAGGAGAGTAACTGCTCTTTG

TGTGACGGTATAGGAGAAGAAAGCCCCGGCTAACTACGTG

>2327 len=161 tot-seq=5

TGGGGAATCCTCCGCAATGGGCGCAAGCCTGACGGAGCAACGCCGCGTGAGTGAAGAAGG

GTTTCGACTCGTAAAGCTCTGTTGTCGGGGACGAATGTGGAGGTTGTGAATAACAGCTTC

CAATGACGGTACCTGACGAGGAAGCCACGGCTAACTACGTG

>2328 len=161 tot-seq=5

TTAGGAATATTCGTCAATGGGGGAAACCCTGAACGAGCAATGCCGCGTGAGTGATGAAGG

TCTTATAGATCGTAAAACTCTGTTGTAGAGAGAGAACACTTATTATAGGAAATGATAATA

AGCTGACGGTACTCTACCAGAAAGCCCCGGCTAACTACGTG

>2329 len=161 tot-seq=5

TGGGGAATATTGCACAATGGGCGCAAGCCTGATGCAGCAACGCCGCGTGAACGATGAAGG

TCTTCGGATCGTAAAGTTCTGTTGTTAAGGAAGAACGAGTGTGAGAATGGAAAGTTCATG

CTGTGACGGTACTTAACCAGAAAGGGACGGCTAACTACGTG

>2330 len=161 tot-seq=5

TGGGGAATATTGCACAATGGGCGAAAGCCTGATGCAGCGACGCCGCGTGAGCGAAGAAGT

ATTTCGGTATGTAAAGCTCTGTTATACGGGACGAACGGCAAGGTGGTAAATAGCCATCAT

GAGTGACGGTACCGTAAGAGAAAGCCACGGCTAACTACGTG

>2331 len=161 tot-seq=5

TGGGGAATTTTGCACAATGGGGGAAACCCTGATGCAGCGACGCCGCGTGAGTGAAGACGG

CCTTCGGGTTGTAAAACTCTGTTATACGGGACGAATGTAGGCATGGTTAATACCCATGCG

TAGTGACGGTACCGTAAGAGAAAGCCACGGCTAACTACGTG

>2332 len=161 tot-seq=5

TGGGGAATATTGCGCAATGGGGGAAACCCTGACGCAGCGACGCCGCGTGCGGGATGAAGG

CCTTCGGGTTGTAAAACTCTGTTGGTAGAGAAGAACGTTGGTGAGAGTGGAAAGCTCATC

AAGTGACGGTAACTACCCAGAAAGGGACGGCTAACTACGTG

>2333 len=161 tot-seq=5

TGGGGGATATTGCGCAATGGGGGAAACCCTGACGGAGCAACGCCGCGTGAACGATGACGG

CCTTCGGGTTGTAAAGTTCTGTTATACGGGACGAATGGCGTAGCGGTCAATACCCGTTAC

GAGTGACGGTACCGTAAGAGAAAGCCACGGCTAACTACGTG

>2334 len=161 tot-seq=5

TGGGGAATCTTCCGCAATGGACGAAAGTCTGACGGAGCAACGCCGCGTGAGTGAAGAAGG

GTTTCGACTCGTAAAGCTCTGTTGTCGGGGACGAACGTGGAGACGGCAAATAGTCGTTTC

CAATGACGGTACCTGACGAGGAAGCCACGGCTAACTACGTG

>2335 len=161 tot-seq=5

TGGGGAATCTTCCGCAATGGGCGAAAGCCTGACGGAGCAATGCCGCGTGAATGAAGAAGG

CCTTCGGGTCGTAAAATTCTGTCCTTATTGAAGAGAGGTTCATGTGTAAACAATGCATGA

ATAGGACGGTAGATAAGGAGGAAGCCCCGGCTAACTACGTG

>2336 len=161 tot-seq=5

CAAGTGATGCAGAGACAGACAGAAAAGTGAGGTTGAGGTATTCTTTGCCCTGGTTCCTCC

TTTTTCTCTGGCTGAAGGTCACAGTTCCTGTCAGGTCTCACAGGTGTCCTCTCCATACAA

CATGACTCTCTGTGGCCACTCCCTCCATTGCCCCTACAGGC

>2337 len=161 tot-seq=5

TGGGGAATATTGCGCAATGGGGGAAACCCTGACGCAGCGACGCCGCGTGCGGGATGAAGG

TTTTCGGATCGTAAAGCTCTGTTGTTAAGGAAGAACGAGTGTGAGAGTGGAAAGTTCATG

CTGTGACGGTACTTAACCAGAAAGGGGCGGCTAACTACGTG

>2338 len=161 tot-seq=5

TAGGGAATTTTCCACAATGGGCGAAAGCCTGATGGAGCAACGCCGCGTGAACGATGAAGG

CCTTCGGGTTGTAAAGTTCTGTTGTTAGGGACGAACAAGTGCCGTTCGAATAGGGCGGCA

CCCTGACGGTACCTAACGAGGAAGCCACGGCTAACTACGTG

>2339 len=161 tot-seq=5

TAGGGAATCTTCCGCAATGGGCGAAAGCCTGACGGAGCAACGCCGCGTGAGTGATGAAGG

TCTTCGGATCGTAAAACTCTGTTGTAAGAGAAGAACGTGTGTGAGAGTGGAAAGTTCACA

CAGTGACGGTAACTTACCAGAAAGGGACGGCTAACTACGTG

>2340 len=161 tot-seq=5

TAGGGAATCTTCCGCAATGGACGCAAGTCTGACGGAGCAACGCCGCGTGAACGAAGAAGG

TCTTCGGATCGTAAAGTTCTGTTGTTAGGGAAGAACACGTGCCATTCGAATAGGATGGCA

CCTTGACGGTACCTAACTAGAAAGCCCCGGCTAACTACGTG

>2341 len=161 tot-seq=5

TGGGGAATTGTTCGCAATGGGCGCAGGCCTGACGACGCAACGCCGCGTGGAGGATGAAGG

TCTTCGGATTGTAAACTCCTGTTGACCGGGAAGAATGGACCTCGCATTAACACCGTGAGG

TAATGACGGTACCGGTTGAGGAAGCCACGGCTAACTCTGTG

>2342 len=166 tot-seq=5

GGGCTAGCACAGGCCAGCCTGCTCAGCTGTGGGTGTTCTGCAGCGAACCTGCTGTGCCTG

TGTCACATACCAAGGGCATGACTAAGGGCTACTGATGCAGAGTCAGAAGTGGCCCAGCCC

TGGCCCCTATCTCCTCCCAGGCTCCTCTGAGCCTCAGCAGAGACTG

>2343 len=166 tot-seq=5

CTAAGAATATTCCGCAATGGGGGAAACCCTGACGGAGCGACGCCGCGTGGATGATGAAGG

CCGGAAGGTTGTAAAGTCCTTTTGGGCGCAAAGAATAAGTACGCGAGGAAATGCGCGTGC

GGTGACGGTAGCGCCTGAATAAGCAACGGCTAATTACGTGACAGCA

>2344 len=167 tot-seq=5

GCAGGCTGACAGGAAGTAAATTTTATTATCAAGACATGCATTCTACCCTCTGGCCTCACC

TGTCTCCTCTGTCTCCACCTTGCCCCCATCCTCACCCCCAGGGGAAAGAAAAGAAGTCAA

CAAAGCAGGCTTTTCCTGCTAGCTCTCCATGGCATGTGGCCAAGACA

>2345 len=169 tot-seq=5

AGAGAAGGGTTTGGGAGCCAGTGCTGTGTATGCGCCCCTAGTCCTGCCACCTCCCTAAAG

CTTGTGCACTCAGGTGTGAAGGCAGGTGCTGTAGGGTGGGGAGGAGCCCACCTGTCTACA

CAGGCTCACCTGTGCTTCTCCAACCGGTGGAGGCATTTATCCCTGTTTC

>2346 len=169 tot-seq=5

AAAGAAAGGGCAGGGCTTCCCTGGGCAGCACTTGGTGGGGGCCAGGGCACCAGGGCCCCC

TCTGACCAGCGAAGCCCCGAGGACCCCAGCTTGGTGCAGGAAGAGGACCCAGTGCTTCAG

TTTGCCGGTATAAAAAACTCACACCCATGCTTCCTCACCTGAATACATA

>2347 len=171 tot-seq=5

ACAATGTCAAATACAGACTCAGGAGAGACTCTCTGAGAAGGGGACACAGGAGCAAAGGTC

TAAGCCAGGGAATGGAGGGGCCCTGAGGGTGTCTGGGGCAGAGACAAGCTTAGGGGTTGA

GATTTAGGTTTAGCCCAGGAGTGGGAGCCTTGGGGGAGGACCAGGTAGTGC

>2348 len=171 tot-seq=5

AGGGGCTCAGCAGGGAGCTCTGGCAGGGTAGTTGGGAGCCCTGAGTCTCATCCAGGCTCT

GTTGCTTGGCTGTGTGATCTGGGCTAATTGCTTGCCCTTTCTGGGTCTGACCTTCTCCAT

CTACAAAATGAGGTGTATTCATTGGATCATTTCTGGGGTTCTTTCCCAGCT

>2349 len=173 tot-seq=5

AACTTAGGAAATTAGTCTGAACAGGTGAGAGGGTGCGCCAGAGAACCGGACAGCTTCTGG

GACGGGCGGAAGCACAGAGCCGCTGAGGCAGCAGCCTGGGCGGGCCGCAGATAACCGGCC

ACCATCCGGACCAGAGGACAGGTGTCCGCCTGGCTTGGGAGGCGACCTCAGCC

>2350 len=173 tot-seq=5

ACAACCCTCTCCATAAGATGGGGTGTTTCTGGAGTAAATGATAGTTCATCCATGTTGTTT

ATGTTTCTAACAATTAGAAATTTTCAACTTAGAACTCAGCATACAAAGCCAACTTTAATC

AGATCAGTGACAGCCAGTGAAGACAGCAAGCTGTATGTTTTCAGACAAGTAGG

>2351 len=176 tot-seq=5

CGACTTGGCTGCAGACTCAGAGAATGGCTTTGCTATTCGGCAGCCATAATCTGCCTCACA

CTGCAAAGCAGCCATTGCCCTCATTCCTCTGAATTCAGGGGTAGCCTAAAGTCTTCTTAA

ATTGTAAACACATCCTGGTTGGCAACCTTCGGAAACTCTGAGCCTTCTGCCCCTTC

>2352 len=179 tot-seq=5

TCTCTACGGGAGGCAGCAGTGGGGAATCTTCCGCAATGGGCGAAAGCCTGACGGAGCAAT

GCCGCGTGAGTGAAGAAGGGTTTCGGCTCGTAAAACTCTGTTGTTGAAGAAGAACGTGCG

TGAGAGTAACTGTTCACGCAGTGACGGTATTCAACCAGAAAGTCACGGCTAACTACGTG

>2353 len=179 tot-seq=5

GAACTAGTTACTCTAGATTAACCTGAATGGCTTCCTCAATACAAGAGCAACCAATGTCCT

GGGCATCCTCCCAAGTTTTTGCTGAGTCTTCCCACTGAATAGAACTGGTCACAAGGTGAG

CATTTGCTGTCTCTCCTTGTACATTCTAGTCTGATTCCTTGGAATGTGCTACCTCAGTC

>2354 len=179 tot-seq=5

TGGGGAATATTGCGCAATGGGGGAAACCCTGACGCAGCGACGCCGCGTGCAGGATGAAGG

CCCTCGGGTCGTAAACTGCTTTTATTAGAGAAGAATATGACGGTAACTAATGAATAAGGG

ACGGCTAACTACGTGCCAGCAGCGCGGTAATGCACAGATCGGAAGAGCGTCGTGTAGGG

>2355 len=180 tot-seq=5

TGAGGAATATTGGTCAATGGGCGGAAGCCTGAACCAGCCAAGTAGCGTGCAGGAAGACGG

CCCTCCGGGTTGTAAACTGCTTTTAGTTGGGAATAAAACGGGGCTCGTGAGCCCCCTTGC

ATGTACCATCAGAAAAAGGACCGGCTAATTCCGTGCCAGAGCCGCGGTAATCTACAGATC

>2356 len=182 tot-seq=5

GAGGCCAGGGAAGGAGGCAGGGAGACTTCAGGAAAGGTCTGCGGTGCTGTGTGAGGATTT

TCTCTGGACCACCAACTTTTCAAGGTGTTGATAGTGATGGCGGGTAGTAGCTGCTGGCTT

GGCAGGGCAATGCTGTAACTCCAGGCACTGTGGGGAGGGGGCCTCCATGCAGGAGGATGA

CA

>2357 len=182 tot-seq=5

AGGCAGTGATTTAAAAATGGCTTTCCAGAGACCCAGCCGCGCTCCCTGCCCTTGAGGCGG

AGGCTGGAGGTCGCGAGTCGGTGGAGCCCCGCTCGGTGCTGCGGAGACTGCAGTGGCGGC

TGGGACCCGGAGCCGTGGGCGAGGCCGGCGCGAAGAACTAAGGGAGGACTCAGGCGACAA

CT

>2358 len=183 tot-seq=5

TCGGGAATTTTGCTCAATGGGCGCAAGCCTGAAGCAGCAACGCCGCGTGAGGGATGAAGG

CCTTCGGGTTGTAAACCTCTTTTATCAGGGACGATTATGACGGTACCTGATGAATAAGCC

ACGGCTAACTACGTGCCAGCCAGCCGCGGTAATTACAAGATCGGAAGAGCGTCGTGTCGG

GAA

>2359 len=183 tot-seq=5

CAAGCAGAAGACGGCATACGAGATAAGCTAGTGCGGGAGGCAGCAGCAGTGTTCTAGAAT

ACTATGTGTGTTATAAACACTCAGAACCCAGCAGCAGTGTTCTGGAATCCTATATGAGGG

ACACACCCTCAGAACCCAGCAGCAGTATTCTAGAATCCTTTGTGAGGGACAAGCACTCAG

AAC

>2360 len=183 tot-seq=5

GCAGAGACAAGATGGAGTAGAAAAGAGCAGGAGAGAGAGGGGGCAGGGAGAGAGACAGAG

AGAAGCCTAGGAGTCAGATACAGTCAGGGACAAAAGCGGAGAGAACAGAATCAACTGCAC

ACAGGTAGACACGGGGGTGGGGATACATAGATTGGAATGTGGGGGTGGGGAGCACCAGGG

AGC

>2361 len=184 tot-seq=5

ACTGATTTTGTCTTGATAGTCTGGGTCAATCACCCTAGCCAACACTGTAACTCCCTTCTT

AGCCCACTGACTTAAACTTAGGAGGAGCCCGAGGTGTCCAGGTGGCAATCTTAACTTCTA

GTTTAATGGAATCATTGTGTCCCCTGGTGGCAGCGTTTCTCCCTCTGGAACTAAGACTTC

TAGA

>2362 len=189 tot-seq=5

CTTGGTCATTGGGCAAGGCTTGTCAACTGTGTGGGTGGTGGCCTCACCGCCCCACTGCCC

TGGAAAGGAGAGCAGACTCCTTGCCGTCCCCCTCAGGGAACCTGGAGAGATCAGCTTGGC

TCTTGGCTTTCAGCCATGAGAGAGAAACAGGGTATGGGGTTCACACGATTCCTGAGCATC

TCTGGTGGG

>2363 len=190 tot-seq=5

TGGCAGCAGAGGACTGGCTGGGCAGGGAGTTCTCAGGCCCCTAAGCAGCATATGTGAGGT

CCACAATGGTAGAGTAGCAGTGGCAGGCCAGTATTTAGGCTGTCAAAAGTCAAGTGAGGG

TGCCAATGGTGGTGGCAGCGGGCTGAGAAGGTTAGTCCCCATGTCTCCATGTGGCATATG

TGAGTATATG

>2364 len=190 tot-seq=5

CCAACCGTGACTGGAGTTCAGACGTGTGCTCTCCGATCTCCTACGGGAGGAGCAGTGGGG

AATATTGCACAATGGGCGCAAGCCGGATGCAGCGACGCCGCGTGAGCGAAGAAGTATTTC

GGTAGGTAAAGCTCTATCAGCAGGGAAGAAAATGACGGTACCTGACTAAGAAGCTCCGGC

TAAATACGTG

>2365 len=190 tot-seq=5

CAAGCAGAAGACGGCATACGAGATCCGTAGGTGACTGGAGTTCAGACGTCTGCTCTTCCG

ATCTCCTACGGGAGGCAACAGCAGCGCCAGGCCTGGGGGGAAGAGAGAACAGCTGTTGAG

TTTGGCTGCCTCTCCTGCTGTCAGGGGTCACTGTCAAAATATTTAACAACCAGTGAAACT

GGGAACTGGC

>2366 len=191 tot-seq=5

AGAAGTGGAAGGGGACAGTGACCATATCCCAATGTCACAGAGTACATGCAAGAAACAATG

TACAGCCATTTTTTATGCCCATTCCTTATTTCTCCTGATAATACAGCCTGCTCCAGGCAC

AGCAGGGATGCTCAACCCAAGTCCCTCCCAGAATTTTCAAGTGAGAGACAGGGAGGACGC

TTCCTCTTTGG

>2367 len=191 tot-seq=5

AGCGGGAGATATGAAGCTGGAGCGAAAGAATCAGCTGGAATCAGATGAACGCATGGGAAG

CAGTCGCCATGCACAGCAATCCCGTGACGCTCCCGTCAGTGGAAGCAGAAAGAAAAAGCA

GACTGCAAAGTTCCGTACACAGGAACAAAGCGCCGGAGAGGGAAAGCTTCTTGAGAAGGA

ACGGCACCTCT

>2368 len=191 tot-seq=5

GTGGATAGAAGCGTAAACCTTGAGGTGAGGTAGAGCTGGCTTTGAGCCCCAGATCTGGCA

CATTCTAGCTGTGACTCCCTGCTGTGCTTGGGGCCTCAGTGTTCCCATCTGTAAGATGGG

CTGTTACTGGTACCTGTTTCCTGAGTTATGTGAGCAGGAGCCTACATGTAAAGAGCCTGC

CACATGATCAG

>2369 len=192 tot-seq=5

GTGGGAGGTCCCCTCCAGAACAGTATGTGGTTCCAAACTCCTGAGGGAGGCGGGACAGAC

CCCAAGGAAGTAGATGAGAACCTGAGTGAGGCTCTGTGGGGAGAGAGGATGTTCCTCAGC

CCTCATGAGGACGTGTCAGTTTTACTTGAGATTAGGCCCTGGCCTTGGCTGGGCAGCCTG

CCAGGTAGGTTC

>2370 len=193 tot-seq=5

GAAACACTGGGGGCGCGCACAAAACAACTCCCTAGTCTGTATTTCCTCTCCTCCTTTGGA

CCCATGCCTTCTTATTCTCCTGAGACAAGCGGATCTTTCTGTGTTCAGAGTTTTATTTAC

AAGGGAACGTTGATGTAATTAGCAGTGTGTTTAGGGTGATCTTTACTGATCTGTATCACA

AAGCCTTCTTACC

>2371 len=194 tot-seq=5

GTTCAGACGTGTGCTCTTCCGTCTCTTACGGGAGGCAGCAGGGCAAGCTGCAGCAGAGAG

CACGCAGACACAGCTAGGTGAACAGTGGGCTCATCTCTGGAGTGCCTGCCTGCCACAAGG

CAGAGGGCTGGCTGGGAATGGCGGTTGTACTCACCACAACAGGGGAACACAGAACAGGCC

AGGCAGTGTCAGGG

>2372 len=195 tot-seq=5

TAGGGAATCTTCCACAATGGACGAAAGTCTGATGGAGCAATACCATGTGAACGATGAAGG

TCTTTTTGATTGTAAAGTTCTTTTATGAGGGATAACAACTGATAGTACCTCATGAATAAG

TGACAGCAAACTATGTGCCAGCAGCCCGCGGTAATAAGCAGATCGGAAGAGCGTCGTGTA

GGGAAAGAGGGGAGA

>2373 len=195 tot-seq=5

GTAAAGAGGCCCCTGCCCTGAGCACTTTCCCTCCCCCGTGTCGCCCTGCCCAGCCCCTTG

ATCAGACGATGGCAATGGAGGGAGGCCTCTGCATCTGGAATCTTCCAGCCCTCCTGCTCC

CTTGGCTGGGTATATTTGTCTTGGCTGCCTTCACCTGGGCTTTTTTAAAACAGATTTTCT

TTCCTTTGCTTTCTC

>2374 len=196 tot-seq=5

GACGTTGCTCTTCCGATCTTCTACGGGAGGCAGCAGTAGGGAATCTTCCACAATGGACGC

AAGTCTGATGGAGCAACGCCGCGTGAGTGAAGAAGGGTTTCGGCTCGTAAAGCTCTGTTA

TAAGGGAAGAACATTTGCAATAGGAAATGATTGCAGACTGACGGTACCTTGTCAGAAAGC

GATGGCTAACTATGTG

>2375 len=197 tot-seq=5

TAGGGAATCTTCCACAATGGACGAAAGTCTGATGGAGCAACGCCGCGTGAGTGAAGAAGT

ATTTCGGTATGTAAAGCTCTATCAGCAGGGAAGAAATAGCGACCTTTGGGTCGCCAGACG
GTACCTGATTAAGAAGCCCCGGCTAACTACGTGCCAGCAGCCGCGTAATGAACAGATCGG
AAGAGCGTCGTGTATAG
>2376 len=198 tot-seq=5
AGAAGTGTGATATTAAGATATAATAAGGGAGGAAGAAGTAGGGAATATTAAAAAATGGAA
GCAAGTCTGATGGAGCAAAGCCGAGTGAGTGAAGAAGGTTTTAGGATAGTAAAGATATGT
TGTTGGTGAAGAAGGATAGAGGTAGTAAATGGAATTTATTTGACGGTAATCAACCAGAAA
GTCACGGCTAACTACGTG
>2377 len=199 tot-seq=5
TGGGGAATATTGCACAATGGGCGCAAGCCTGATGCAGCAACGCCGCGTGAACGATGAAGG
TCTTCGGATCGTAAAACTCTGTTGTTAGAGAAGAACAGCGCATAGAGTAACTGTTATGCG
TGTGACGGTATCTAACCAGAAAGCCACGGCTAACTACGTGCCAGCAGCCGCGGTAAGATC
GGAAGAGCGTCGTGTATGG
>2378 len=200 tot-seq=5
AATTCTTGTTCAGACAAGGAGATCATCATGTGGCCCTTTAAGAGCTTCACCAGATGGTGG
GAGCCAGAGAATGAGCAGCATACAGGAAGGCTCAGCCAGCCCTATGGGGATGGAGCAGAG
TGAACCAGAGTCACAGAGCAGAAAGCAGTCACCAAGGAGAGAAGGGCCACAAGCCCACAG
CTGCAATAAATTAGCTTCTT
>2379 len=200 tot-seq=5
AATTTTCCATCAAATTCTTCTTTATTAAAAAAAAATAGATTCCTTTTCAAAATAGACAAG
CTTGGAAATTTTAAAAATAACAGTATTCATTAACAACTCCCACACTAACTGTGGTTACCA
AACCTTGAGAATCTAAGTGCCAAATTCCATGTGCTAGCATTCACATGCTTTCTACTTTGA
TTCCCACAGTGGCTCCCACT
>2380 len=201 tot-seq=5
TGGTCTAATGGCATTTACTCTATAGCCCAGACAGGCCTAAACTTATCTAATCCTCCTGCC
TCATCATTCTAAGTTGCTAGGATTATAGGAAGACGCCACCATGACTAATTTAGCCTTCCT
GCTTTCTTAGTTCATCTGACAGAAAATCCAGTGACTTCTAAATGTGTTTACCCTCTGAGA
AGGGTAGTCAGGTCTCCAATC
>2381 len=203 tot-seq=5

CTGGAGAAGGGAGAGCACACATGGGAAGACTCGGCTACGCTGTTTTGTTTTTGCTAAAAG

TGCAAAATGACTACTGAACAGCCATTGCACTAAGCAAAGTTTATCTGAGAACCATGTACC

TCTCGTGTTTCATTACAAGCGCTATACAGTTGATTGCATATCTCTAAGTAGCAGCATAGC

ACATGGTGATACATGTGTCTTGT

>2382 len=204 tot-seq=5

AGAGGAGCCAAGCAGGAAGATGGGGCAGAAAAGGCTGCGCTTGCTCCGGGCCGGGGCAAG

CATGCCAGAGAGCAGGCCTGATCCAGAATGGTCCCAGGATGGCCGCAAGTGGCCTCAGAC

CCAGGGACACCACCGAGGTGGCAGTGCGTGAGGGCACCGGCTGGGGAGGGGGCTTGTCCT

TCCGCTCCAGCATCCGCTGAAGGC

>2383 len=207 tot-seq=5

CCTTGCGCTTCAACTGCGACCAACAGGCAGAACAGAAGAAGAAGAAGAGGCACAGCAGCA

CCGGAGACAGCGCGGATGCAGGGCCGCCTGCAGCGGGGAGCGCTCGTGGAGAGAAAGAGA

TGGAGGGTGTGGCCCTCAAGCATGGTCCATCCCTCCCCCAAGAGCGGAAGCAAGCCCCTT

CCACCCGGAGGGACTCCGCTGAACCTT

>2384 len=207 tot-seq=5

TGAAGAGAATATTATAAAAATATTAAATAACATAAAGTTCTATGTTTCTTATGGAGGAAC

CCACAAAAACTGTATTTTGTTTTGTTTTGTTTTTTTTCTTGAGATGGAGTTTCACTCTTG

TTGCCCAGGCTGGAGTGCAATGGTGTGATCTTGGCTCATTGCAAGCTCTGCCTCCTGGGT

TCACGCCCTTCTCCTGCCTCAGCCTCC

>2385 len=208 tot-seq=5

CCACATGCTTCGGAAACGGGGCTGCCCCCCAATGTCCCAGAGCTGAGAGAGAAGAGGATA

GGGAGAAGGTGGCCTCAGGAGCAGGCAGACAACTGAAAGCCATTGGGTTCTTAGCTTCCC

TTCTGCAGTCTCCCCCCACCCTAAGGTCACAGCTCCTTCCCCCTCCTACGTCTACAGTGC

CTATCTTCAGCTCCCTCTCCCCAGTCCT

>2386 len=208 tot-seq=5

TGGGGCATATTGGACAATGGGCGCAAGCCTGATCCAGCCATGCCGCGTGTGTGAAGAAGG

CCTTATGGTTGTAAAGCACTTTAAGCGAGGAGGAGGCTACTGAAGTTAATACCTTCAGAT

AGTGGACGTTACTCGCAGAATAAGCACCGGCTAACTCTGTGCCAGCAGCCGCGGTATCCT

CAGATCGGAAGAGCGTCGTGTTGGGGAA

>2387 len=208 tot-seq=5

TGAATATTAGGGAAGGAACCACGAGTTCAGAGAAAAACAAATCAGTAAATCCACTTCCAC

TTTTTTTCCCCCAAACTGTCGGTCACTCCAAGAGGTGAGGGGAGGCTTGCTTTTCCCCAC

TGGTAGCATAATGAGTTTCCTAAGCCATACTCCACTCAGCAAGGCTGCCGGGCAACCGGC

TCCCCCAGCACACTGGAAGCAGCCCTTG

>2388 len=209 tot-seq=5

GGCACCTGAAGCGGCTGAACAGACCTTCTTCCACTGTCCCCAGGAACCTCATGGCACTGA

AGTGGACCCAGCCAGTTGGCACTGGGTTTGTTGTGCAAGTGAGATGGAAGGTTACCAAGG

AAAAAAAAAATGAAGTCAAGGGAATAAGAAAATCCCCAGGACTTCTACCCGACCCAATGC

AATAAACGCGATAGAAACAAACCCTGGAG

>2389 len=209 tot-seq=5

ATGATGTAGAGGAAAAGTACCGCACAGGAAATGGGGCTCTGACTCCACGACTAACCAGCT

ATGTGACCTGGGGCAAGGCACTTAAGCCTCAATCTCCTCATCCCTAAAATGAGGGGACTG

AATTGGACCCTTTTCTAATTCCCTTCCAGTTCCTACAATAATTTGTCATCATCCTTCCAC

ACTTGGGCTCTTGGCCTCTAAGTCCATGT

>2390 len=210 tot-seq=5

TGACTGGAGTTCAGACGTGTGCTCTTCCGAGCGCCGACGGGAGGCAGCAGTAGGGAAGCG

GCCACAAGGGACGCAAGGAGGATGGAGCAAAGAAGATGGAGTGAAGAAGGGTTTCGGAGA

GGAAAGATCTGGGGGGGGTGAAGAAGGACAGGGGTAGTAAAGGACATTGGTTTGAAGGGA

ATCAATTAGAAAGGCACGGCTAACTACGTG

>2391 len=212 tot-seq=5

TGGTGACTGGAGTTCAGACGTGTGTCTTCCGATCTCCTACGGGAGGCAGCAATAGGGAAT

CTTCCGCAATGGACGAAAGTCTGACGGAGCAACGCCGCGTGAGTGAAGAAGGGTTTCGGC

TCGTAAAACTCTGTTGTCAGGGAAGAACGCCGACGGGAGTAACTGCCCGTCGGGTGACGG

TACCTGACCAGAAAGCCACGGCTAACTACGTG

>2392 len=214 tot-seq=5

AGAGTCTGAGAAACAGCAGAACTTTTGATAGCTCCGTGGACCAAAAGGACAAAACTGTCA

TCAAAGTAAGGGTGAATAAGAAATAGTCTGAAAATGTATTTATATGTAGGATATATAAAG

AGCTACAAATCAATAAAGACAGCGAAATAGAAAGCAAAAGACTTGAACAGTTACTTCACC

AAAGAGAATGTTCAAAATGAGTAATATTACCCAA

>2393 len=217 tot-seq=5

CATCTGTATTTGTTACTCTTCAGTAAAATATTGACAAATAAACATAGCATCACATTTTAC

AAATCAGAGCATCACTAGCATTCATTCTTGGGGTTTCCCCCATCTTGTTCTTCAGCTGGA

CACTGGACATCTCGAGTCTCCCTAGACAGCACAAGGTGTCCACCTTTTGCCCATCCTTGA

TGTTCAGAATGGGCAAATGTGATGGTTTGCCAAGTTC

>2394 len=219 tot-seq=5

GGAGCAGGTGTGAGGTGAGGTGCCACGCCTTGAGAAACCCCAGGGATCGGCACACTGTTC

TCACGTAGCAGCCAGAGTATCTGCCACCATCCCCACACTACAGGAATCCAATGAGATCCC

AGAGGGAGGGTGAGGGACCTTCCAGAGCCCAGCAGGTGGCAGCAGAGCCAGGACTCACCC

AGACCCACCCTCAGGTTCAGTAGGTGGCCCTCTCCCCAC

>2395 len=223 tot-seq=5

GAAGGACGAGCTGTGCATCTCTGTGGGGCAGCCACATCTTTGCTCTGCTAGGGGGTACAG

GTGCACGGGACCACATCCCTGTCCTTAGGAGAGGTGCACCCCCAGCGAGGAGGAGATGAG

TCTTCAGGCTGCACAGCTCCTGGCCAGGCAGGGCAGCACATTCCTGCCACCTGCATTTGT

TACGGGCCCTCTGTGTGCCCGGCTCTGCTCTGAGCCCTGCGGA

>2396 len=225 tot-seq=5

GAACACAAGTTCAAGGTCACCCTCCGTGACACAGCAAGTTCCAAGACAGCTCGGTATATA

TGAGAACCTGTTTTAAAAAATTGAAAATCCAGGAGAAGTCCTAGCATCCAGTTGTGATGC

TAACTGGCGTTTCTAAAGATGCCCCCTCTAAGTTCTGGGTAAAGCAAGGAGAGCTCTGTT

ATTCCCAACTGGCTACTGAAAGATCAAAAAAAAGCCAGGAACTTT

>2397 len=225 tot-seq=5

CAGTCCACAGAAAAGGCTCCCGCTTGGGAGTAAGACACACAGGATGCTAAGTTCTGCCTC

TGCCACCCTGGCAGCTCCCTGGCAGCGGACAAGTCTCGGGCCTCTCTGGGTTTCAGTTTC

TTTGTGGGGAAATGAAGATGGCCACGCCCACCTCCTCAGGTGCACTGGGTGCAGGGCCAA

CACCTAGCAAGGGCTCAGGAAAGAGTGTCCCCATCAGCCACAGCA

>2398 len=228 tot-seq=5

TTTTTAAAGAAGAAGAAGGAATAAGAGATGAATGAGTGAATGGAGGGAAGAAGGGGGAGA

GGAAGAGAGAAGAAGGGAGGAAGAAGGGGGGAAGAGGGAGAAATGGGGGAAAAAATGAAG

AAGAGAAGAAGAGTGAGGGATGAAGGAATTAGGGTTGTAAACCGCTTTTGATTGGGAGCA

AGCCTTCGGGTGAGTGTACCTTTCGAATAAGCGCCGGCTAACTACGTG

>2399 len=228 tot-seq=5

AGTGGCAAGAAGGATCCAGGCAGTGGCACTTACCCCGTGAGAGTCACCTCTCCTCCCACC

TCTCCCTCTCTTCAGGCAGGACTTTGGGGAATTTGGTTCAACGGCCTCAGAGTTTGGGGA

CTCCTAGATCTCCCTGTGCATGCTCACACCAGACTGAATCGAGGCTTTGTGTCACAGGTG

AAGTAGTGTCTAAGACTAATCCATTCTTCCTCTTCCCCAACAAGTTCA

>2400 len=228 tot-seq=5

CATCTCCAGATCTAGCTGTCTGGGGAGGCGCAGGCTCATTTCCACTAGAAAAGCGGGATT

TGCCTTCCCTTAAATGCACATTCCACTCTGCGTTTTTAAAAGGCAAAATAAAGACACTTC

CCAGAAGCATTAAAAACAAGGAACAGAAATGTCCAAGGATCACAGTGTCCAGCCCCTGGC

TTAGAAGATGAAGCAGTGTCTGAAGGCACCTGTTCTTCCCAAATTAAC

>2401 len=233 tot-seq=5

GGAGCAGGTGGCTCGTCCAAGGGGGAAGCCAAGAAAGCTTTATGAACAGGTTCTACAGAA

AAGGGCAGGATTAAATGACCCAACAAGCTGCACCCTGGGGCCAGATGCAGGAGGCCAGCA

TCCCTGAAGGGGCCAGTAGAGGGAAGGTTACCAGAACAGGTGAGAACCAGGGCTGCCAAA

GAGGCCCAGACAGCAGCTGCAGCCTTGGGTGGAGGAACCTGCCTAACTGTGGC

>2402 len=237 tot-seq=5

GAATAGTGGAGTAAGGAATTTCAAAAATTCACTTCTGCATTAAGGAATAAAAACATTGGC

AAAAATGGTCTAAATATCTTTTTTAGAACTCTGGAAATTACCTAAAGACTTGCAACAATC

CAAGACACATCTGTTAAAGAAAAATGGCTGAATCTGGGTAAGAACAGCAAACTTTGTGGT

ATTTTAATTTGTCCTAATTCTACCCCTCTTTCTCCAGCTCTGCACTAGCCTTGCGAA

>2403 len=238 tot-seq=5

AGGAGCGCAGAGTGTTGCTTTGTGAGAAGGACCCGCCCAGCGTTGCTGGCCTTTAAGATG

GAGGAAGGGGGCCGTGTGGTAGGAATGTGGGGGCCTCAGGAGGGAAGGAAATTGGCTCTC

CCAGGAGCTTCCAGAAAGGAATGCACCCCTGCTGACACTTTGATTTTGGCCCCAGTGAGC

CTCGTGTTGGACTCCTGGACTACGGAGCTGTAAGATAATAAATGTGTGCTGTTTTAAG

>2404 len=239 tot-seq=5

TGTAGGCAGGATCTTGCCTGCGTCTTGCAGCTGCTGCAAACTCACTTTCAGTCCCGCGGT

GACAGCTGGAGCTTCCTCCAGGCCGGCCTTGGGGGCTCCTGCTCCTGCTGGCAGCTTCTC

CGCGTGGTGGCCCTGCAGCTCTGCCCGGAGGCTGGCCAGGTCCCCTTGCAGGGCGGCCAC

CTGGTAGAAAGACACCACCGTGAGGCAGCAAGACAGCAGTGCCAGCAGCAAGGTTGCAG

>2405 len=134 tot-seq=5

GCGCGCAAATTACCCACTCCCGACCCGGGGAGGTAGTGACGAAAAATAACAATACAGGAC

TCTTTCTAGGCCCTGTAATTGGAATGAGTCCACTTTAAATCTTTTAATGAGGATCAATTG

GAGGGCAAGTCTGG

>2406 len=155 tot-seq=5

TGAGGAATATTGGTCAATGGACGGAAGTCTGAACCAGCCAAGTAGCGTGCAGGATGACGG

CCCTATGGGTTGTAAACTGCTTTTATATGGGAATAAAGTGAGGCACGTGTGCCTTTTTGC

AGGTACCGTATGAATAAGGACCGGCTAATTCCGTG

>2407 len=100 tot-seq=4

TGATGGGTCAGGCACAAATCTTGCCCTCAGGAATGTGAATTCTAGTGGGGACACAGAAAC

AAACCACACGACCACAGAAGAGATGGGAAAAAGAACACAG

>2408 len=103 tot-seq=4

GTTGTGGCCAAATGGGTGTTTCTCATGGAAGATCTTAGAGGTGAAAGAGCAAGTACAGTG

TGTGCCTTCTGGACTGGTGGCTGAAACAGCAGAGAAAGGAAGG

>2409 len=106 tot-seq=4

CCTGGGTGGGGCCCAGATTCCAGTTGACCCACTCAGCCACTAAAGGCTCTAGGGAAAGAG

CGGTGCTCTGTGCTGGTTGTGGAGTGTTACTTGAGGACTCTGAAAC

>2410 len=106 tot-seq=4

TAGTGGTGGAAGTGACTCTCATCAGGCTGTCCAGGCACAGCAGTGTCCCTAGAGAGCAGC

TCTCAGTGTTCCAGGGCCTAGAGAAGCTTCCTGATCCCCCACCATC

>2411 len=106 tot-seq=4

GCACTTAACAAATGCTTGCCAAGTAAATCGTTGGAATATGTTGGAGATCATCTAATCTCT

GGGGGTTTGCAGTGTTCCTGGCAGCCTCAGAGGCTGTGAACATCTC

>2412 len=108 tot-seq=4

CCACAGTCTGTAAAAATTATGACATGGAATTACACAATGTGGCAGCCCTGGAAACTACCC

AAACCACTTCTATTTCTAGTCCTGTATTCCCTTCAATACCTACTACAT

>2413 len=109 tot-seq=4

GGCACTAAGCATGTGGACAAGTAGTGTGTGTAGATAATGCACACACGCGCAGACACATTT

AAAGCAACAACTGCCACCTTGTCCCTCCACATCCGGGGTCCATCACCTC

>2414 len=109 tot-seq=4

CAAACTCGAAGGTAGAGAGCCTCGCCTACCAAACTCCGGAGAAAGCAATGACAGTGTGTG

CTCTACGCACTCCTCACGCGCCTGAGGACACAGCACAGAAAGGAGGATT

>2415 len=112 tot-seq=4

TAGGGAATCTTCCACAATGGACGCAAGCCTGATGGAGCAACGCCGCGTGAGTGAAGAAGG

GTTTCGGCTCGTAAAGCTCTGTTGTTGGGGAAGAAGGTCAGGGGACGTAACT

>2416 len=113 tot-seq=4

ATTCCAGCCTGCAGCTGAAGAATTAACACTACGCTGTGAATAACTTTGTTTACTCTCTTC

CACATTTTAAAAGAAGATACCAGACTACCACTTCAGAGTTGGAATTTCAAACA

>2417 len=114 tot-seq=4

GTGAGTGAAGAAGGTTTTCGGCTCGTAAAGCTCTGTTGTTGGTGAAGAAGGACAGAGGTA

GTAACTGGCCTTTATTTGACGGTAATCAATTAGAAAGTCACGGCTAACTACGTG

>2418 len=115 tot-seq=4

TGGGTGTGGTGAAAGGTGTTCAGGGTCTGGTTGCATCTTGTAGCTGGAGCTTAGGGAAAC

TGCAGAGAGGGTGTAGGCTCTGAGAGGGAGCAGCCAGGCTCCAAGGCTTTTTGTC

>2419 len=116 tot-seq=4

GCCAGGCTGGGTGTGGAGGGTTCAGGCCTCTCGGATGCATCTGGAGTGTGCTGGGGCTGG

GCACCAGGCCTCCTGGGAGGTCTGTCTTGGTTGCTGGAGGGTGGGGTCCTGATGTC

>2420 len=116 tot-seq=4

TGACCGCACTTGTGAGGAGTGCCATGCCTGGCTCCGAGGCTCCTAGTTTCAAGTGGTGTA

ACAGTTCCAGGCTATTGAGTAAGAGTTCTTATTAGGCCATGTGATCCAGCAGCCGC

>2421 len=119 tot-seq=4

GTTGTTTTGCTTTCGTAAGCCATATTATGTAAGTAACATCTTGAACATGGAGCTGAGCCA

ACCTAGAGGTCACATCAGGAGGGAAAAGGGGAAAGAGGCTATTCAAGGAAGTGGAAAGA

>2422 len=121 tot-seq=4

GACGGTAAGGAGGGCGTCACAGGCCCTGGGCATGTTGGGTGGGGGGCAGCAGGACGGTAA

GGAGGGCGTCACAGGCCCTAGGCATGACGGGCGGGGGAAAGTGAGGACACAGAGCCTGGG

C

>2423 len=124 tot-seq=4

GGGCAGGAGAAGGCTTCATTCATTTGCTTACTCCTTTTAATTAGTAATGCTTTGTATACC

TACCTGTGATGTGCTATCCTCGTAGGCACTTCAGGGCAGCTCTTCTGCAGCATGGAGAGG

AGAG

>2424 len=126 tot-seq=4

CAGCTCCGGGTACGGGGCCCCGGGTCCTGGGCTGGTGCACGGGAAGGAGATGCCGAAGGG

GTATCGGCGGCCCCTGGCACACGCCAAGCCACCGTATTCCTATATCTCACTCATCACCAT

GGCCAT

>2425 len=126 tot-seq=4

GCATTATTCCTTAGGCTTTCCTGTGGGGGTTGCATATTGATTTGTTTTTCTTTTTTAAAA

GCACATGCATACGAGAGAACTTATTATTTACATGACTCTGGTGTTGATCACTAGGTTGTA

TCTTGG

>2426 len=126 tot-seq=4

GGGGCAGCGAGCCCCGCCCTCCCCAGCTGAGGGGGCGGGAGGCCACGCCCCGGGGGGGGC

TCCAGCGACCGACGGTTGAGCCCCTTGCGTTGTTCTCCTCCTCCCGGCCGTGTCGCACCG

CCGGGA

>2427 len=127 tot-seq=4

ACTCGTTTTTTGATGAGGAATAGCAGTTGTAGAGAAGTGTGACTGCAGATCACATGACTA

GAAGTGACAGGTCAAGACTAGATTCCACTATCAGTCAGGGTTCTCTGGAGAAACAGCTCC

CCCCGGG

>2428 len=128 tot-seq=4

CATTTACCCCGGAATTGGAGTTCATCAGGTGGAAGCACATTCCAGGGAAAGGGGCCGGCG

TGTGCACAGTCCAGAGGTAGGAAGGAGCTGCAGAGCTGGGCTGTGGTTTCTGCCCTGCCT

GTTACCAC

>2429 len=130 tot-seq=4

GTGATTCTCCTGCCGCAGCCTCCTGATTAGCTGGGATTACAGGCACACACCATGCCTGGC

TAATTTTTGTACTTTTAGTAGAGACGAGGTTTCACCATGTTGGCCAGGTTGGTATTGAAG

CACCAGTGTA

>2430 len=131 tot-seq=4

TTGCAGTGAGCCAAGATCTAAGCCTGGGCAACAGAGCAGAAACTCCATCTCAAAAAAATA

CAAAATAAAATTTAAAAATAAATTTTTACTACCCAGCCTTGGGCTGGGTGTAGTGGCTCA

CGCCTGTAATC

>2431 len=131 tot-seq=4

CCCGGTGGGGGGACTTGTGCTGAGCTCTGGTTCTGCAGAGGGCAAGTCTCACCTCTCTGT

GCCTCAGTTTCCTTATCTGCAGAATGGGAGTATGTGAATCACCATCCTCTTAACTGCTCC

ACCCAGTTCCT

>2432 len=133 tot-seq=4

AAATGAGAACAGGAAGGCATGGCAAGATACTAACATCATGGAAGACATTTTTCATGAATT

ATGTCCCCCATAGTTGTCCTGGGAGTTGGGTCCCCACTTACTGAAAGAGAAAGCCCAGAG

GTATGCAAGAGTG

>2433 len=133 tot-seq=4

TGAGCATCCTGAAAAAGAAAATTAAAGGATTTCATAAAGGAAATGGCTGAGCCTCACAGC

TGGCAAGCAATGCCTGCTTCATCCCTAACAGCAGCAATCTTACGCCATTATCAGCGACAT

GGCATCCTCTCCC

>2434 len=134 tot-seq=4

CTCTAACTTTTAGCCTGCTAGTCTTGAAGTCACAGGAAAAAAGAGAAATGTTGAAAAGTG

AATTTAGCATTTAGTTCTACATTGTTAAAAGTTTATAATGAAAGCTCTCTAAGTGAAAGG

GGATAGCAGAATAA

>2435 len=134 tot-seq=4

GTTTCTTAGACTGTAAGTCAAAATGGAAAATTAAATTCACGAGGGAAACACAAAGCGTCT

CCATTCATCTCTTTAATACCCAGGTGCATTATGGGCTTCATCAGCAAGGAGCGAGAACGC

GCTCTGCTCAAGGA

>2436 len=134 tot-seq=4

TCACAACAGTGGCAATTGTGGTGTGCAGAGGGTGGCAGAGTCACCAGCGTCTATGCATGT

GTTCATACCAGCAATGACAGTGCGACTGAGTGCCCACACATTAGTGGTGTGGGGGGGCAG

CATGTTGCTTCACA

>2437 len=134 tot-seq=4

TGGGGAATATTGGACAATGGAGGAAACTCTGATCCAGCAACTCTGTGTGTGAAGAAGGTT

TTAGGACTGTAAAACACTTTTAGTAGGGAAGAAAAAAATGACGGTACCTACAGAAGAAGC

GACGGCTAAATACG

>2438 len=134 tot-seq=4

TGGGGAATTTTGCACAATGGGGGAAACCCTGATGCAACGACGCCGCGTGATTTAGAAGGC

CCTCGGGTTGTAAAAATCTTTTGTATAGGAAGAAAATGACAGTACTATACGAATAAGGAC

CGGCTAATTACGTG

>2439 len=135 tot-seq=4

TGGGGAATATTGGGCAATGGGGGAAACCCTGACCCAGCAACGCCGCGTGAAGGAAGAAGG

TCTTCGGATCGTAAACTTCTATCCTTGGTGAAAATAGTGATGGTAGCCAAGAAGGAAGCC

CCGGCTAACTACGTG

>2440 len=135 tot-seq=4

TGGGGAATATTGCGCAATGGGGGAAACCCTGACGCAGCGACGCCGCGTGCGGGATGAAGG

CCTTCGGGTTGTAAAGCTCTTTCACCGGAGAAGATAATGACGGTATCCGGAGAAGAAGCC

CCGGCTAACTTCGTG

>2441 len=135 tot-seq=4

TGGGGAATCTTCCGCAATGGACGAAAGTCTGACGGAGCAACGCCGCGTGAGTGATGAAGG

CCTTAGGGTTGTAAAGCTCTTTCACCGGAGAAGATAATGACGGTATCCGGAGAAGAAGCC

CCGGCTAACTTCGTG

>2442 len=135 tot-seq=4

TGGGGAATCTTGCGCAATGGGCGAAAGCCTGACGCAGCCATGCCGCGTGGATGATGAAGG

TCTTAGGATTGTAAAATCCTTTCACCGGTGAAGATAATGACTGTAGCCGGAGAAGAAGCC

CCGGCTAACTTCGTG

>2443 len=135 tot-seq=4

TGGGGAATATTGCACAATGGAGGAAACTCTGATGCAGCGACGCCGCGTGAGCGAAGAAGT

ATTTCGGTATGTAAAGCTCTGTCAGCAGGGAAGATAATGACGGTACCTGACCAAGAAGCC

CCGGCTAACTACGTG

>2444 len=135 tot-seq=4

TGAGGAATATTGGTCAATGGGGGAAACCCTGATGCAGCGACGCCGCGTGAGTGAAGAAGT

ATCTCGGTATGTAAAGCTCTATCAGCAGGAAAGATAATGACGGTACCTGACTAAGAAGCC

CCGGCTAACTACGTG

>2445 len=135 tot-seq=4

TGGGGAATATTGGGCAATGGGGGAAACCCTGACCCAGCGACGCCGCGTGAGTGAAGACGG

CCTTCGGGTTGTAAAGCTCTGTTGCAGAGGACGATAATGACGGTACTCTGTGAGGAAGCC

ACGGCTAACTACGTG

>2446 len=135 tot-seq=4

TGGGGAATATTGGGCAATGGGCGAAAGCCTGACCCAGCGACGCCGCGTGGAGGAAGAAGG

TTTTCGGATTGTAAACTCCTGTCTTCGGGGAAGATAATGACGGTACCCGAGGAGGAAGCC

ACGGCTAACTACGTG

>2447 len=135 tot-seq=4

TAGGGAATTTTGCGCAATGGGCGAAAGCCTGACGCAGCAACGCCGCGTGATCAAGACGCC

CTTCGGGGTGTAAAGATCTGTCAGTGGGGACGAAACTTGACGGTACCCACAGAGGAAGCA

CCGGCTAACTCCGTG

>2448 len=135 tot-seq=4

TGGGGAATATTGCACAATGGGCGAAAGCCTGATGCAGCGATGCCGCGTGAGGGAAGAAGG

TCTTCGGATTGTAAACCTCTGTTTTCAGGGACGATAATGACGGTACCTGAGGAGGAAGCT

CCGGCTAACTACGTG

>2449 len=135 tot-seq=4

TGGGGAATATTGCTCAATGGGGGAAACCCTGAAGCAGCAACGCCGCGTGGGTGAAGACGG

CCTTCGGGTTGTAAAGCCCTGTCTTCGGGGACGATAATGACGGTACCCGAGGAGGAAGCC

ACGGCTAATTACGTG

>2450 len=135 tot-seq=4

TGGGGAATATTGCACAATGGGCGCAAGCCTGATGCAGCAACGCCGCGTGAACGATGAAGG

TCTTCGGATCGTAAAGTTCTGTTGCAGGGGAAGATAATGACAGTACCATACGAATAAGGA

CCGGCTAATTACGTG

>2451 len=135 tot-seq=4

TGGGGAATATTGCACAATGGGCGCAAGCCTGATGCAGCGACGCCGCGTGAGTGAAGAAGT

ATTTCGGTATGTAAAGCTCTGTCAGCAGGGAAGAAGATGACGGTACCTGAATAAGAAGCC

CCGGCTAACTACGTG

>2452 len=135 tot-seq=4

TGGGGAATATTGCACAATGGGCGCAAGCCTGATGCAGCAACGCCGCGTGAGTGATGAAGG

TTTTCGGATCGTAAAGCTCTGTCTTTGGGGAAGATAATGTCGGTACCCAAGGAGGAAGCC

ACGGCTAACTACGTG

>2453 len=135 tot-seq=4

TGGGGAATATTGGACAATGGGCGCAAGCCTGATCCAGCCATGCCGCGTGGATGATGAAGG

CCCTAGGGTTGTAAAGTCCTTTCGGCGGGGAAGATAATGACGGTACCCGCAGAAGAAGCC

CCGGCTAACTTCGTG

>2454 len=135 tot-seq=4

TGGGGAATATTGGACAATGGGCGCAAGCCTGATCCAGCAATGCCGCGTGAGTGATGAAGG

CCCTAGGGTTGTAAAGCTCTTCTACCCGGGAAGATAATGACTGTACCGGGAGAATAAGCC

CCGGCTAACTCCGTG

>2455 len=135 tot-seq=4

TGGGGAATATTGCACAATGGGCGCAAGCCTGATGCAGCGACGCCGCGTGAAGGAAGAAGT

ATTTCGGTATGTAAACTTCTATCAGCAGGGAAGAAGATGACGGTACCTGAGTAAGAAGCA

CCGGCTAAACACGTG

>2456 len=135 tot-seq=4

TGGGGAATATTGCACAATGGAGGGAACCCTGATGCAGCGATGCCGCGTGAGGGAAGAAGG

TTTTAGGATTGTAAACCTCTGTCTTAGGGGACGATAATGACGGTACCCTAGGAGGAAGCT

CCGGCTAACTACGTG

>2457 len=135 tot-seq=4

TGAGGGATATTGGGCAATGGGGGAAACCCTGACCCAGCAACGCCGCGTGAGGGATAACGG

TTTTCGGATCGTAAACCTCTGTCCTCTGTGAAGATAGTGACGGTAGCAGAGGAGGAAGCT

CCGGCTAACTACGTG

>2458 len=135 tot-seq=4

TGGGGAATATTGCACAATGGGCGAAAGCCTGATGCAGCAACGCCGCGTGAGTGATGAAGG

CCTTCGGGTCGTAAAGCTCTTTGATCAGGGATGATAATGACAGTACCTGAAAAACAAGCC

ACGGCTAACTACGTG

>2459 len=135 tot-seq=4

TGGGGGATATTGCACAATGGGCGAAAGCCTGATGCAGCGATGCCGCGTGGAGGAAGAAGG

TCTTCGGATTGTAAACTCCTGTCTTTAGGGACGATAATGACGGTACCTAAGGAGGAAGCT

CCGGCTAACTACGTG

>2460 len=135 tot-seq=4

TGGGGAATCTTCCGCAATGGACGAAAGTCTGACGGAGCAACGCCGCGTGAGTGAAGAAGT

ATTTCGGTATGTAAAGCTCTATCAGCAGGGAAGAAAATGACGGTACCTGACTAAGAAGCC

CCGGCTAACTACGTG

>2461 len=135 tot-seq=4

TGGGGGATATTGGACAATGGGGGAAACCCTGATCCAGCGATGCCGCGTGAGGGAAGAAGG

TTTTCGGATTGTAAACCTCTGTGGAGGGGGACGATAATGACGGTACCCCTTTAGGAAGCC

ACGGCTAACTACGTG

>2462 len=135 tot-seq=4

TGGGGAATATTGGACAATGGGGGCAACCCTGATCCAGCGACGCCGCGTGAGCGAAGAAGT

ATTTCGGTATGTAAAGCTCTATCAGAAGGGAAGAAAATGACGGTACCTTACTAAGAAGCT

CCGGCTAAATACGTG

>2463 len=135 tot-seq=4

TGGGGAATATTGCACAATGGGGGGAACCCTGATGCAGCGACGCCGCGTGAGTGAAGAAGC

GCCTCGGCGCGTAAAGCTCTGTCAGCAGGGAAGAAGATGACGGTACCTGACCAAGAAGCC

CCGGCTAACTACGTG

>2464 len=135 tot-seq=4

TGGGGAATATTGCACAATGGGCGAAAGCCTGATGCAGCGACGCCGCGTGAACGATGAAGT

ATTTCGGTATGTAAAGTTCTATCAGCAGGGAAGATAATGACGGTACCTGACTAAGAAGCC

CCGGCTAACTACGTG

>2465 len=135 tot-seq=4

TGGGGAACATTGGACAATGGGCGAAAGCCTGATCCAGCCATGCCGCGTGAGTGATGAAGG

CCTTAGGGTTGTAAAGCTCTTTCGGCGGGGACGATAATGACGGTACCCGCAGAAGAAGCC

CCGGCTAACTTCGTG

>2466 len=135 tot-seq=4

TGGGGGATATTGCACAATGGGGGAAACCCTGATGCAGCAACGCCGCGTGAAGGAAGAAGG

TCTTCGGATTGTAAACTTTTGTCGCAAAGGAAGATAATGACGGTACTTTGTGAGAAAGCC

ACGGCTAACTACGTG

>2467 len=135 tot-seq=4

TGGGGAATATTGGACAATGGGCGCAAGCCTGATCCAGCCATGCCGCGTGAGTGATGAAGG

CCCTAGGGTTGTAAAGCTCTTTCAGCGAGGAGGATAATGACGTTACTCGCAGAAGAAGCC

CCGGCTAACTTCGTG

>2468 len=136 tot-seq=4

GAACAAGCTCAGAGATGACAGTGAACGAAGGTCCTCAATGACCCTATCCAAATCTAGAAG

GAGAATAGAACGAGACAGGAAGGAGACTTAGGAGCCTGGAGAACCAGAGTCCAACAAGAG

TAGCCAGACAGCCTGG

>2469 len=136 tot-seq=4

TAGGGAATTTTGCGCAATGGGCGAAAGCCTGACGCAGCAATGCCGCGTGAATGTTAAACC

CTTCGGGGTGTAAAGTTCTGTCAGTGGGGACGAACAAATGACGGTACCCACAGAGGAAGC

ACCGGCTAACTCCGTG

>2470 len=136 tot-seq=4

TGGGGGATATTGCACAATGGGGGGAACCCTGATGCAGCGACGCCGCGTGGGTGAAGAAGC

GCTCCGGCGCGTAAAGCCCTGTCAGCAGGGAAGAAAAAGGACGGTACCTGACTAAGAAGC

CCCGGCTAACTACGTG

>2471 len=136 tot-seq=4

GCGCGCAAATTACCCACTCCCGGCTCGGGGAGGTAGTGACGAAAGATAACGATACGGGAC

TCATCCGAGGCCCCGTAATCGGAATGACTACACTTTAAATCCTTTAAGGAGTATCAATTG

GAGGGCAAGTCTGGTG

>2472 len=136 tot-seq=4

TGGGGAATATTGCACAATGGGGGAAACCCTGATGCAGCGACGCCGCGTGAGCGAGGAAGT

ATTTCGGTATGTAAAGCTCTGTCAGCAGGGGAGAAGAATGACGGTACCTGAAGAAGAAGC

ACCGGCTAAATACGTG

>2473 len=136 tot-seq=4

TAAGGGATATTGCGCAATGGGCGAAAGCCTGACGCAGCAACGCCGCGTGCCCGAGGAAGG

CCTTCGGGTCGTAAAGGGCTTTTAGGGCTGAAGAGAACGGACGGTAAGCCCGGAAGAAGT

GTCGGCTAACTACGTG

>2474 len=137 tot-seq=4

TGGGGAATATTGCACAATGGGGGAAACCCTGATGCAGCGACGCCGCGTGAGTGAGGAAGT

ATTTCGGTATGTAAAGCTCTATCAGCAGGGAAGAAGAAATGACGGTACCTGACTAAGAAG

TCCCGGCTAACTACGCG

>2475 len=137 tot-seq=4

TGGGGAATTTTCCGCAATGGGCAAAAGCCTGACGGAGCAATGCCGCGTGGAGGTAAAAGG

CCTACGGGTCATGAACTTCTTTTCCTGGAGAAGAAACAATGACGGTATCTGGGGAATAAG

CATCGGCTAACTCTGTG

>2476 len=137 tot-seq=4

TGGGGAATATTGGGCAATGGGCGAAAGCCTGACCCAGCGACGCCGCGTGAGGGAAGAAGT

CCTTCGGGATGTAAACCTTAGTCGACAGGGACGAAGAAATGACGGTACCTGTAGAGGAAG

CCACGGCTAACTACGTG

>2477 len=137 tot-seq=4

TGGGGAATATTGCACAATGGGCGCAAGCCTGATGCAGCGACGCCGCGTGAAGGATGAAGG

TCTTCGGATCGTAAACTTCTATCAGCAGGGAAGAAACCATGACGGTACCTGACTAAGAAG

CCCCGGCTAACTACGTG

>2478 len=137 tot-seq=4

CAAGCCTGATGGAGCAGCACCGCGTGAGTGAAGAAGGGTTTCGGCTCGTAAAGCTCTGTT

GTTGGAGAAGAACGTGCGAGAGAGTAACTGGTCACGAAGTGACGGTAACCAACCAGAAAG

TCACGGCTAACTACGTG

>2479 len=137 tot-seq=4

TGGGGAATTTTCCGCAATGGGCGAAAGCCTGACGGAGCAATGCCGCGTGAAGGCAGAAGG

CCCACGGGTCATGAACTTCTTTTCTCGGAGAAGAAAAAATGACGGTATCTGAGGAATAAG

CATCGGCTAACTCTGTG

>2480 len=137 tot-seq=4

CCGAGGTTGGATGGGCCACGCCCAGCTCAGCAGAGCTCCCTGCAACCGGCCAAGCCCTCA

GTCTGCAAACTTTCTGGTACTGGGTTTGGGAGGGCCATGGGATTGGACAAGACTCCAGTG

CCACCCAGAGGCTGAAC

>2481 len=138 tot-seq=4

TGGGGAATATTGCACAATGGAGGGAACTCTGATGCAGCGATGCCGCGTGAGGGAAGAAGG

TTTTAGGATTGTAAACCTCTGTCTTCGGGGACGAAAAAAAAGACGGTACCTGAGGAGGAA

GCTCCGGCTAACTACGTG

>2482 len=138 tot-seq=4

TGGGGAATCTTGCGCAATGAGGGAAACCCTGACGCAGCGACGCCGCGTGCGGGATGGAGG

CCTTCGGGCCGTGAACCGCTTTCAGCAGGGAAGACATTAAGGACGGTACCTGCAGAAGAA

GCCCCGGCTAACTACGTG

>2483 len=138 tot-seq=4

TGGGGAATATTGGGCAATGGGCGAAAGCCTGACCCAGCAACGCCGCGTGAAGGAAGAAGG

TTTTCGGACTGTAAACTTCTTTAGACAGGGACGAAGGAAGTGACGGTACCTGTAGAATAA

GCCACGGCTAACTACGTG

>2484 len=138 tot-seq=4

TGGGGAATATTGCACAATGGGCGCAAGCCTGATGCAGCAACGCCGCGTGAACGATGAAGG

TCTTCGGATCGTAAAGTTCTGTTGCAGGGGAAGAAAAAAATGACGGTACCTACAGAAGAA

GCAACGGCTAAATACGTG

>2485 len=138 tot-seq=4

TGAGGAATATTGGTCAATGGACGCAAGTCTGAACCAGCAATTCTGTGTGTGTGAAGAAGG

TTTTAGGACTGTAAAACACTTTTAGTAGGGAAGAAAAAAATGACGGTACCTACAGAAGAA

GCGACGGCTAAATACGTG

>2486 len=138 tot-seq=4

TGGGGGATATTGCACAATGGGGGGAACCCTGATGCAGCAACGCCGCGTGTGGGAAGAAGG

TTTTCGGATTGTAAACCACTGTTCTTAGTGAAGAAGAAAGTGACGGTAACTAAGAAGAAA

GCTCCGGCTAACTACGTG

>2487 len=139 tot-seq=4

GGCTCCAGGGGGCTGGCTGCTCTTAATTCCTAAATGGAAGCAGATCCCCTTTCCCCCAAA

CCATCCTTGATGATGATGGTCAGAGATGGATAGGCTGTCATCTGAAGAAACTGAGAGGGT

TCCAGGGGGACAAGGTAGA

>2488 len=139 tot-seq=4

TTGGGAATATTGGACAATGGAGGAAACTCTGATCCAGTGACGCCGCGTGAAGGAAGAAGG

TCTTCGGATTGTAAACTTATTTTGTCAGGGAAGAAAAAAAATGACTGTACCTGAAGAAAA

AGCACCGGCTAACTACGTG

>2489 len=139 tot-seq=4

TGTGGGGAATATTGGGCAATGGGGGAAACCCTGACCCAGCGACGCCGCGTGAGGGATGAA

GGTTTTCGGATTGTAAACCTCTGTCTTCGGGGACGAAAAAATGACGGTACCCGAGGAGGA

AGCCACGGCTAACTACGTG

>2490 len=139 tot-seq=4

CTTTAGCTTGTCTTTGCTCCTGCATCTGGGTACCTCTGACCCTGACACCCCTAGCCCACT

TTCCCTGGTGCCCAACTGACATAGATGCCCCTCAGCGAGGCAGAGGAGGCCACTGTGGGT

GGTGTGGCCAGGGGCTCCT

>2491 len=140 tot-seq=4

TGGGGAATATTGCACAATGGGCGCAAGCCTGATGCAGCGACGCCGCGTGAGGGATGACGG

CCTTCGGGTTGTAAACCTCTTTTGCCAGGGACGAAGCGCAAGTGACGGTACCTGGAGAAG

AAGGACCGGCCAACTACGTG

>2492 len=140 tot-seq=4

TAGGGAATCTTCCACAATGGACGCAAGTCTGATGGAGCAACGCCGCGTGAGGGATGACGG

CCTTCGGGTTGTAAACCTCTTTCAGCAGGGACGAAGCGCAAGTGACGGTACCTGCAGAAG

AAGCACCGGCTAACTACGTG

>2493 len=140 tot-seq=4

TGGGGAATATTGCACAATGGGCGAAAGCCTGATGCAGCAACGCCGCGTGCGGGATGACGG

CCTTCGGGTTGTAAACCGCTTTCAGTAGGGACGAAGCTAACGTGACGGTACCTACAGAAG

AAGGACCGGCCAACTACGTG

>2494 len=140 tot-seq=4

TGGGGAATATTGCGCAATGGGCGAAAGCCTGACGCAGCGACGCCGCGTGGGGGATGACGG

CCTTCGGGTTGTAAACCTCTTTCAGCAGGGACGAAGCGAAAGTGACGGTACCTGCAGAAG

AAGCACCGGCCAACTACGTG

>2495 len=140 tot-seq=4

TGGGGAATATTGCACAATGGAGGGAACTCTGATGCAGCGACGCCGCGTGAGGGATGGAGG

CCTTCGGGTTGTAAACCTCTTTTATCGGGGAGCAAGCGAGAGTGAGTTTACCCGTTGAAT

AAGCACCGGCTAACTACGTG

>2496 len=140 tot-seq=4

ATGTGTCTCAAGCTGTAACTTCTGATTTGAGCAGCAGGGAAGGAGCAGTTCCTCTCAGCA

TAAGATGAGGGATTTTGTCATCAAATATCTTCCCATCCCACTTTGTACTGTCACACGCCA

AAGCCAGCTCCACACAGGGC

>2497 len=140 tot-seq=4

TGGGGAATATTGCACAATGGGCGGAAGCCTGATGCAGCGACGCCGCGTGAGGGATGACGG

CCTTCGGGTTGTAAACCTCTTTCAGCAGGGACGAAGCGTGAGTGACGGTACCTGCAGAAG

AAGCGCCGGCCAACTACGTA

>2498 len=141 tot-seq=4

CCACGGGTCCCCTCACTGGGAGGGACATGGTCACTAGCCTTGGCCCTTTTTGGCTTAATC

CCTTCTTTCTTGACTGGGATATCCTCTTCTATTCAACCAGTAACTGAGAGAATATTCAAC

TGGTTCTTCCGCAGTCTAAGT

>2499 len=142 tot-seq=4

TGGGGAATCTTGCGCAATGGGGGCAACCCTGACGCAGCGACGCCGCGTGCGGGACGAAGG

CCTTCGGGTCGTAAACCGCTTTCAGCAGGGACGAGGCCGAAAGGTGACGGTACCTGCAGA

AGAAGCCCCGGCTAACTACGTG

>2500 len=142 tot-seq=4

TGGGGAATATTGCACAATGGGCGCAAGCCTGATGCAGCGACGCCGCGTGAGGGATGAAGG

CCTTCGGGTTGTAAACCTCTTTCAGCGGGGACGAAGCCGTGAGGTGACGGTACCCGCAGA

AGAAGCGCCGGCCAACTACGTG

>2501 len=142 tot-seq=4

GGACGAAAGTCTGATGGAGCAACGCCGCGTGAGTGAAGAAGGGTTTCGGCTCGTAAAACT

CTGTTGGTAGTGAAGAAGGATAGGGGTAGTAACTGGCCTTTATTTGACGGTAATTAATTA

GAAAGTCACGGCTAACTACGTG

>2502 len=142 tot-seq=4

TGGGGAATTTTGGACAATGGGGGAAACCCTGATCCAGCCATGCCGCGTGCGGGATGACGG

CCTTCGGGTTGTAAACCGCTTTTGATCGGGAGCAAGCCTTCGGGTGAGTGTACCTTTCGA

ATAAGCACCGGCTAACTACGTG

>2503 len=142 tot-seq=4

TGAGGAATATTCCACAATGGGCGAAAGCCTGATGGAGCAATGCCGCGTGCGGGATGAAGG

CCTTCGGGTTGTAAACCGCTTTCAGCAGGGACGAGGCCGCAAGGTGACGGTACCTGCAGA

AGAAGCCCCGGCTAACTACGTG

>2504 len=142 tot-seq=4

TGGGGGATATTGCACAATGGAGGAAACTCTGATGCAGCGACGCCGCGTGCGGGATGACGG

CCTTCGGGTTGTAAACCGCTTTTGATCGGGAGCAAGCCTTCGGGTGAGTGTACCTTTCGA

ATAAGCACCGGCTAACTACGTG

>2505 len=142 tot-seq=4

TAGGGAATATTCCACAATGAGCGAAAGCTTGATGGAGCGACACAGCGTGCACGATGAAGG

TCTTCGGATTGTAAACCGCTTTTGATTGGGAGCAAGCTTTCGGGTGAGTGTACCTTTCGA

ATAAGCGCCGGCTAACTACGTG

>2506 len=142 tot-seq=4

TGGGGAATATTGGACAATGGAGGGAACCCTGATGCAGCGACGCCGCGTGCGGGATGAAGG

CCTTCGGGTTGTAAACCGCTTTCAGCAGGGACGAGGCCGCAAGGTGACGGTACCTGCAGA

AGAAGCCCCGGCTAACTACGTG

>2507 len=142 tot-seq=4

TGGGGAATATTGGACAATGGGCGCAAGCCTGATCCAGCCATGCCGCGTGCGGGATGAAGG

CCTTCGGGTTGTAAACCGCTTTCAGCAGGGACGAGGCCGCAAGGTGACGGTACCTGCAGA

AGAAGCCCCGGCTAACTACGTG

>2508 len=142 tot-seq=4

TAGGGAATATTCCACAATGAGCGAAAGCTTGATGGAGCGACGCCGCGTGCGGGATGAAGG

CCTTCGGGTTGTAAACCGCTTTCAGCAGGGACGAGGCCGCAAGGTGACGGTACCTGCAGA

AGAAGCCCCGGCTAACTACGTG

>2509 len=142 tot-seq=4

TGGGGAATATTGGGCAATGGGCGAAAGCCTGACCCAGCAACGCCGCGTGCGGGATGAAGG

CCTTCGGGTTGTAAACCGCTTTCAGCAGGGACGAGGCCGCAAGGTGACGGTACCTGCAGA

AGAAGCCCCGGCTAACTACGTG

>2510 len=144 tot-seq=4

TGGGGAATATTGCACAATGGGCGCAAGCCTGATGCAGCGACGCCGCGTGAGGGATGACGG

CCTTCGGGTTGTAAACCTCTTTCAGTACAGAACAAGGCCTTCGGGTTGAGGGTATGTGCA

GAAGAAGCGCCGGCTAACTACGTG

>2511 len=144 tot-seq=4

TGAGGAATATTGGTCAATGGGCGAGAGCCTGAACCAGCCAAGTAGCGTGAAGGAAGACTG

CCCTTCGGGTTGTAAACCGCTTTTGATTGGGAGCAAGCCTTTTGGGTGAGTGTACCTTTC

GAATAAGCGCCGGCTAACTACGTG

>2512 len=144 tot-seq=4

TTATTCTGTCTGGAAGAGAAACATTTATTCTGTGTTTGACATTGTGCATGGTATCTAATT

GACCAGTAATAGCATTTTCTGTGTAATTCTGGAGTGATTTCTTAAAGGTAAAAGCAGTAG

AAAAAGTACATTTTCTGATACTGT

>2513 len=144 tot-seq=4

TGGGGAATATTGCACAATGGGCGCAAGCCTGATGCAGCAACGCCGCGTGCGGGATGACGG

CCTTCGGGTTGTAAACCGCTTTTAGTAGGGAAGAAGGGCTTCGGCTTGACGGTACCTGCA

GAAAAAGGACCGGCTAACTACGTG

>2514 len=145 tot-seq=4

TAGGGAATCTTCGGCAATGGACGAAAGTCTGACCGAGCAACGCCGCGTGAGGGATGGAGG

CCTTCGGGTTGTAAACCTCTTTTGTTAGGGAGCAAGGCATTTTGTGTTGAGTGTACCTTT

CGAATAAGCACCGGCTAACTACGTG

>2515 len=146 tot-seq=4

TGGGGAATATTGCACAATGGGCGCAAGCCTGATGCAGCGACGCCGCGTGAGGGATGACGG

CCTTCGGGTTGTAAACCTCTTTCAGTTGGGACGAAGCCAGCTTGCTGGTGACGGTACCTT

CAGAAGAAGCACCGGCTAACTACGTG

>2516 len=146 tot-seq=4

CACAGACCTGGATGTCATTGTGCGGGTTCCAGTCCGAGTCGTAGATGATGACAGTGTCCG

CCGTGGCCAGGTTGATGCCCAGACCACCTGCCCGGGTTGAGAGGAGGAAGCAGAACTGCT

GGGCCCCGGGGGCTGAAAAAGAGAGG

>2517 len=146 tot-seq=4

TGCAGGCCACTCTGGTGATTGGGTGGCTCTGCTCCATGGAGTGACCCAGGGATCCACGTT

GCTTCCATCTTGATGTTCAGCCATCCCCCACAGTGTTGCCTGCCTGAGTTGCAGTCTAGA

CTGGCTCACCCCACTGAGTTGTCCTT

>2518 len=147 tot-seq=4

TAGGGAATCTTCCACAATGGACGCAAGTCTGATGGAGCAACGCCGCGTGAGTGAAGAAGG

GTTTCGGCTCGTAAAGCTCTGTTGTTGGGGAAGAAGGATAGAGGGAGTAACTGGGCTTTA

TTTGACGGTAATCAACCAGAAAGTCAC

>2519 len=147 tot-seq=4

TGAGGAATATTGGTCAATGGACGCAAGTCTGAACCAGCCAAGTAGCGTGCAGGATGACGG

CCCTATGGGTTGTAAACTGCTTTTATATGGGGATAAAGTAGGGAACGTTTGCAGGTACCA

TATGAATAAGGACCGGCTAATTCCGTG

>2520 len=148 tot-seq=4

TGGGGAATATTGCACAATGGGGGAAACCCTGATGCAGCGACGCCGCGTGAGTGAAGAAGT

ATTTCGGTATGTAAAGCTCTATCAGCAGGGAAGAAAAAACCGCAAGGTTATGACGGTACC

TGACTAAGAAGCCCCGGCTAACTACGTG

>2521 len=149 tot-seq=4

ACAAAATGGACGCAAGTCTGATGGAGAAACGAAGCGTGAGTGAAGAAGGGTTTCGGCTCG

TAAAGCTCTGTTGTTGGTGAAGAAGGACAGGGGTAGTAAATGACCTTTGTTTGACGGTAA

TCAATTAGAAAGTCACGGCTAACTACGTG

>2522 len=149 tot-seq=4

CCGTTTTGGGAGGGGGGGATTCTGTACTTTACAGATAATTGAAAGATCCGCCATGATCGT

TTAAGCATTGCGTTCTTGGTGTTCTATAAACTCCCATGTAGAATCTGTTTTTTTCCCATA

GGTTGTGCATTTCTGGCTCTCAAATAGTC

>2523 len=149 tot-seq=4

AGCAGCCGGAGGGTGGAGATGGGACACGTATGGGTCACCTCCACCGTGCAGAGGACAGCA

GAGAAGGACAGCAGAGAAGCCCAGCAGCTGGAGGGTGGAGATGGGCCACGTATGGGTCAC

CTCCACCAGGCAAAGGACAGCAGAGAAGC

>2524 len=150 tot-seq=4

TGGGGGATATTGCACAATGGGCGCAAGCCTGATGCAGCGACGCCGCGTGAGTGAAGAAGT

ATTTCGGTATGTAAAGCTCTATCAGCAGGGAAGAAAGGCCCTATTAGGGAGATGACGGTA

CCTGACTAAGAAGCCCCGGCTAACTACGTG

>2525 len=150 tot-seq=4

AAATGTTACAGTTTTCCTCTAGCCCAGGACAGCTCTGGTGAGAAATTCTCCCTGAAGAGC

CAGCCCACCTTGCTGTACCCCATAACCCCTCTGCAGCCACTGGCTGCAATAGGTGCAGCT

CTGCTGTGCACCTCAATCTCCTCTGCAATC

>2526 len=151 tot-seq=4

TGACAACTGGCTCCTGCTTACTTTGTCATAGTCGTACTGTGACGAACATCTGGTGTCAAA

TCGTAGGTACAGGCAGCGAGCTCCTGGGATATGAACAGTCTCCTTAAACTTATAGTTGTC

TCTGACAGGGTGTACTGTTTCCACGGTCTTC

>2527 len=151 tot-seq=4

ACAATCTTAACTTCAGGAAATGCAATCACATTTTACTAGCTTTGCAAAAAATGAATAAAA

TGACATAGCTGAACAGAACTCAAGTAGGATCTGTGAACGGCAGCATGAAGTGGATAATGG

GATAACGTACCGATGAGGGAAATTTATATAC

>2528 len=152 tot-seq=4

CCAGCCTGGGCCGAGCCAGCCCCTCTCCCTTTGTACCCCAGGGACATGCACTGTAACGAG

ATCACAGTGCCTACTGAATTATTCAGAGCTGCCCTGAGTGTCAGTTTTATTATAAACATG

CAGATTGCTCCAGGCTCCAAACTTCCAAACCT

>2529 len=153 tot-seq=4

GGGTTGCTTTCTCCCAACCCCCACATTTGCCTCCTGATTCCAACTCACGAAGCACTGTCT

CCAAAAGCCCCCAACCACATTCTACACCTTGAGCACTCCACTTTGGTGGAGTATGCTTTG

TAACTGAAGTAGGTGGTAGGTATCATACACAAG

>2530 len=155 tot-seq=4

TGAGGAATATTGGTCAATGGACGGAAGTCTGAACCAGCCAAGTAGCGTGCAGGACGACGG

CCCTCCGGGTTGTAAACTGCTTTTAGTTGGGAATAAAGTGCGGGACGCGTCCCGTTTTGT

ATGTACCATCAGAAAAAGGACCGGCTAATTCCGTG

>2531 len=155 tot-seq=4

TGAGGAATATTGGACAATGGGTGAGAGCCTGATCCAGCCATCCCGCGTGAAGGATTAAGG

ACCTATGGTTTGTAAACTTCTTTTTACAGGGATAAACCTAGATACGTGTATCTAGTTGA

AGGTACTGTAAGAATAAGCACCGGCTAACTCCGTG

>2532 len=155 tot-seq=4

TGAGGAATATTGGTCAATGGTCGGAAGACTGAACCAGCCATGCCGCGTGCAGGAAGACGG

CCTTATGGGTTGTAAACTGCTTTTGTAAGGGAAGAATAAGTCGTATGTATACGGCGATGA

CGGTACTTTACGAATAAGCATCGGCTAACTCCGTG

>2533 len=155 tot-seq=4

TGAGGAATATTGGTCAATGGACGAGAGTCTGAACCAGCCAAGTAGCGTGAAGGATGACTG

CCCTATGGGTTGTAAACTTCTTTTATAAGGGAATAAAGTGCGCCACGTGTGGTGTTTTGT

ATGTACCTTATGAATAAGGATCGGCTAACTCCGTG

>2534 len=155 tot-seq=4

TAAGGAATATTGGTCAATGGACGAAAGTCTGAACCAGCCATGCCGCGTGAAGGATTAAGG

CCCTCTGGGTTGTAAACTCCTTTTATGTGGGACGAAAAAAGACCTTTTCAGGTCACTTGA

CGGTACCATAGGAATAAGCACCGGCTAACTCCATG

>2535 len=155 tot-seq=4

TGAGGAATATTGGACAATGGTCGGAAGACTGATCCAGCCATGCCGCGTGCAGGATGACGG

CCTTATGGGTTGTAAACTGCTTTTGTAAGGGAAGAATAAGGAGTACGAGTACTTTGATGA

CGGTACCTTACGAATAAGCATCGGCTAACTCCATG

>2536 len=155 tot-seq=4

TGAGGAATATTGGTCAATGGGCGAAAGCCTGAACCAGCCATGCCGCGTGAAGGAATAAGG

CCCTATGGGTCGTAAACTTCTTTTGTATCGGAGCAATAAGGGCCACGTGTGGTCCGATGA

GATTACGATACGAATAAGCATCGGCTAACTCCGTG

>2537 len=155 tot-seq=4

TGAGGAATATTGGTCAATGGCCGGGAGGCTGAACCAGCCAAGTCGCGTGGAGGACGAAGG

TCCTATGGATTGTAAACTTCTTTTGTGCGGGAATAAAGTCGGGGACGCGTCCCCGTTTGC

ATGTACCGCACGAATAAGCATCGGCTAACTCCGTG

>2538 len=155 tot-seq=4

TGAGGAATATTGGTCAATGGGCGCGAGCCTGAACCAGCCAAGTAGCGTGCAGGATGACGG

CCCTATGGGTTGTAAACTGCTTTTATGCGGGGATAAAGTCGGCTACGTGTAGCCGTTTGT
AGGTACCGCATGAATAAGGACCGGCTAATTCCGTG
>2539 len=155 tot-seq=4
TGGGGAATATTGCGCAATGGGGGAAACCCTGACGCAGCCAAGTAGCGTGAAGGATGAAGG
CTCTATGGGTCGTAAACTTCTTTTATATAAGAATAAAGTGCAGTATGTATACTGTTTTGT
ATGTATTATATGAATAAGGATCGGCTAACTCCGTG
>2540 len=155 tot-seq=4
TGAGGAATATTGGTCAATGGGCGGGAGCCTGAACCAGCCAAGTCGCGTGAGGGAAGAAGG
TACTGAGTATTGTAAACCTCTTTTGCCAGGGGACAAAGGCGGCCACGGGTGGCCGTAAGA
GGGTACCTGGAGAAAAAGCATCGGCTAACTCCGTG
>2541 len=155 tot-seq=4
TGAGGAATATTGGTCAATGGGCGAGAGCCTGAACCAGCCAAGTAGCGTGAAGGAAGACTG
CCCTATGGGTTGTAAACTTCTTTTATGAGGGAATAAAAAGGGGAACGTGTTCCCTATTGC
ATGTACCTTATGAATAAGCATCGGCTAACTCCGTG
>2542 len=155 tot-seq=4
TGAGGAATATTGGTCAATGGAGGCAACTCTGAACCAGCCATGCCGCGTGCAGGATGACGG
TCCTATGGATTGTAAACTGCTTTTGTACAGGAAGAAACCTCTCTACGAGTAGAGACTTGA
CGGTACTGTAAGAATAAGGATCGGCTAACTCCGTG
>2543 len=155 tot-seq=4
TGAGGAATATTGGTCAATGGGCGAGAGCCTGAACCAGCCAAGTCGCGTGAAGGATGAAGG
TTCTATGGATTGTAAACTTCTTTTATTCGAGGGTAAAGTGTGGTACGTGTACCATATTGC
AAGTATCGAATGAATAAGCATCGGCTAACTCCGTG
>2544 len=155 tot-seq=4
TGAGGAATATTGGTCAATGGGCGCAGGCCTGGACCAGCCAAGTCGCGTGAGGGAAGACGG
TCCTACGGATTGTAAACCTCTTTTGTCGGGGAGTAACGTGCGGGACGGGTCCCGTATTGA
GAGTACCCGAAGAAAAAGCATCGGCTAACTCCGTG
>2545 len=155 tot-seq=4
TGGGGAATATTGCGCAACGGGGGAAACCCTGAACCAGCCAAGTAGCGTGCAGGATGACGG
CCCTATGGGTTGTAAACTGCTTTTACGCGGGGATAAAGTGCGTGACGTGTCTCGCATTGC

AGGTACCGCGTGAATAAGGACCGGCTAATTCCGTG

>2546 len=155 tot-seq=4

TGGGGAATCTTCCGCAATGGGCGAAAGCCTGAACCAGCCAAGTAGCGTGCAGGATGACGG

CCCTATGGGTTGTAAACTGCTTTTATATGGGAATAAAGTGAGGGACGTGTCCCTTATTGC

ATGTACCATATGAATAAGGACCGGCTAATTCCGTG

>2547 len=155 tot-seq=4

TGAGGAATATTGGTCAATGGACGCAAGTCTGAACCAGCCAAGTAGCGCGCAGGATGACGG

CCCTATGGGTTGTAAACTGCTTTTACGCGGGGATAAAGTGCGTGACGTGTCTCGCATTGC

AGGTACCGCGTGAATAAGGACCGGCTAATTCCGTG

>2548 len=155 tot-seq=4

TGGGGAATATTGCGCAATGGGGGAAACCCTGACGCAGCGACGCCGCGTGCGGGATGAAGG

CTCTATGGGTCGTAAACTTCTTTTATATAAGAATAAAGTGCAGTATGTATACTGTTTTGT

ATGTATTATATGAATAAGGATCGGCTAACTCCGTG

>2549 len=155 tot-seq=4

TGAGGAATATTGGTCAATGGGCGGGAGCCTGAACCAGCCAAGTCGCGTGAAGGAAGAAGG

TACTTAGTATTGTAAACTTCTTTTGTCGGGGAACAAAGGCGGAGACGCGTCTCCGGATGA

GTGTACCCGAAGAAAAAGCATCGGCTAACTCCGTG

>2550 len=155 tot-seq=4

TGAGGAATATTGGTCAATGGACGGAAGTCTGAACCAGCCATGCCGCGTGCAGGATGACGG

CTCTATGAGTTGTAAACTGCTTTTGTACGAGGGTAACCCCGGGTACGTGTACCCGGCTGA

AAGTATCGTACGAATAAGGATCGGCTAACTCCGTG

>2551 len=155 tot-seq=4

TGAGGAATATTGGTCAATGGAGGCAACTCTGAACCAGCCATGCCGCGTGCAGGATGACGG

TCCTATGGATTGTAAACTGCTTTTATACGGGAAGAAAACAAGCTACGAGTAGCTAATTGA

CGGTACCGTAAGAATAAGGATCGGCTAACTCCGTG

>2552 len=156 tot-seq=4

TGAGGAATATTGGTCAATGGACGCAAGTCTGAACCAGCCAAGTAGCGTGCAGGAAGACTG

CCCGCAAGGGTTGTAAACTTCTTTTGTATGGGATTAAAGTCACCTACGTGTAGGTGTTTG

CAGTTACCATACGAATAAGCATCGGCTAACTCCGTG

>2553 len=156 tot-seq=4

TGAGGAATATTGGTCAATGGCCGGGAGGCTGAACCAGCCAAGTCGCGTGAGGGAAGACGG

TCCTATGGATTGTAAACCTCTTTTGCCGGGGAGCAAGCTGTGCCACGTGTGGCGCAGCTG

AGAGTACCCGGAGAAAAAGCATCGACTAACTCCGTG

>2554 len=156 tot-seq=4

TGAGGAATATTGGTCAATGGCCGGGAGGCTGAACCAGCCAAGTCGCGTGAGGGAAGACGG

TCCTATGGATTGTAAACCTCTTTTGTCAGGGAGCAAAAACTGCCACGCGTGGCGCAGCTG

AGAGTACCCGGAGAAAAAGCATCGGCTAACTCCGTG

>2555 len=156 tot-seq=4

GCACATCATGTGGAGAAAGCAGGACAGAGGAAGGGAGGTGTCATGCACTTTAAACAGCCA

GATCTTGTCCAAAGTCACTCACTATCAAGAGGACAGAGCCATAGGGATGGCACTAAACCA

CTCAAGAGAAATCCATCCCCATGATCCAATCACCTC

>2556 len=156 tot-seq=4

ATGGCACACGGGGGCGGGGTGTCGGAGGCGGCCTGTGCAGATCCCCTGACCCCCTGCCTT

CTCAGGGCGACAGAAACAAGGCCGTCATACTTAGAGGTGCTGGGCGGCTGAGGAGTGGAG

CGGGTGGGAGGTGCGGGGCTGCAGGGGGATTAGCTC

>2557 len=156 tot-seq=4

TGAGGAATATTGGTCAATGGGCGCAAGCCTGAACCAGCCAAGTCGCGTGAGGGAAGACGG

TCCTATGGATTGTAAACCTCTTTTGCCGGGGAGCAATGCAGGCCTTGCGAGGCCTGAGGG

AGAGTACCCGGAGAAAAAGCATCGGCTAACTCCGTG

>2558 len=156 tot-seq=4

TGAGGAATATTGGTCAATGGGCGAGAGCCTGAACCAGCCAAGTCGCGTGAAGGAAGACTG

CCCGCAAGGGTTGTAAACTTCTTTTGTATGGGATTAAAGTCACCTACGTGTAGGTGTTTG

CATGTACCATTCGAATAAGGACCGGCTAATTCCGTG

>2559 len=157 tot-seq=4

ACGGAGACTGAGCCTTTGGATGCAGGAAGACCTGGCCGACTGGAGCAAGACAGATGCACA

CCTACAGCAGGCAAGAAATCCCAGTCCGCCTTAGCTCAGTCAGGCCAGCCTGGGGGGCCT

GCTTCCACGCACTGCCTGCTGTCCGCCTTCTCCTATT

>2560 len=159 tot-seq=4

TGGGGAATATTGGACAATGGGTGCAAGCCTGATCCAGCAATTCCGCGTGGGTGAAGAAGG
CCTTCGGGTTGTAAAGCCCTTTAAGTTGGGACAAAAAAATCGGCGATAACATCGCCGGTC
TTGATGGTACCGACAGAATAAGCACCGGCTAACTCTGTG
>2561 len=159 tot-seq=4
TGGGGAATTTTGCGCAATGGGCGAAAGCCTGACGCAGCAACGCCGCGTGTGTGATGAAGG
TCTTTGGATTGTAAAGCACTTTCGACCGGGACGAAACCGCATTGGCTAACATCCAATGCT
CTGACTGTACCGGGAGAAGAAGCACCGGCTAACTCTGTG
>2562 len=159 tot-seq=4
TAGGGAATCTTCCACAATGGACGAAAGTCTGATGGAGCAACGCCGCGTGAGTGAAGAAGG
GTTTCGGCTCGTAAAGTTCTTTTATTTAGGAAAAAAAGCGTGCTAGGAAATGAGCGCGCC
TTGATGGTACTAATTGAATAAGTGACAGCTAACTATGTG
>2563 len=159 tot-seq=4
ATGGTTGGTGAGTTCTTTCTTATCCATTCTGTAGTTCTGTATCTTTTAAGTGGAGAGTTT
AGGCCATTTGCATTCAATGTTCTATTTTTGTGCTGGTTGGTCTCCGGCTGGAAGGTGGTG
CTTTCCAGAGAGCATCAGCTGTGGTAATATGAAGAGGAA
>2564 len=160 tot-seq=4
TGGGGAATATTGCACAATGGGGGAAACCCTGATGCAGCAACGCCGCGTGAGTGAAGAAGG
CCCTCGGGTTGTAAAGCACTTTCAGTGAGGAAGAACGCCTGGTGGTTAATACCCATCAGG
AAAGACATCACTCACAGAAGAAGCACCGGCTAACTCCGTG
>2565 len=160 tot-seq=4
TGGGGAATATTGCACAATGGGGGAAACCCTGATGCAGCGACGCCGCGTGAGTGAAGAGGG
CCTTCGGGTTGTAAAGCACTTTTGTCCGGAAAGAAAACGCCGTGGTTAATACCCGTGGCG
GATGACGGTACCGGAAGAATAAGCACCGGCTAACTACGTG
>2566 len=160 tot-seq=4
TGGGGAATATTGCACAATGGGCGCAAGCCTGATGCAGCCATGCCGCGTGTCTGAAGAAGG
CCTTCGGGTTGTAAAGGACTTTTGTCAGGGAAGAAAAGGCTGTTGCTAATATCGACAGCT
GATGACGGTACCTGAAGAATAAGCACCGGCTAACTACGTG
>2567 len=160 tot-seq=4
TGGGGAATATTGCACAATGGGCGCAAGCCTGATGCAGCCATGCCGCGTGTATGAAGAAGG

CCCTCGGGTTGTAAAGCACTTTCAGTGAGGAAGAACGCCTGGTGGTTAATACCCATCAGG

AAAGACATCACTCACAGAAGAAGCACCGGCTAACTCCGTG

>2568 len=160 tot-seq=4

TAGGGAATTTTCGGCAATGGGGGGAACCCTGACCGAGCAATGCCGCGTGAGTGAAGACGG

CCTTCGGGTTGTAAAGCTCTGTTGTAAGGGAAGAACGGTAATGATAGGGAATGATCATTA

AGTGACGGTACCTTACCAGAAAGCCACGGCTAACTACGTG

>2569 len=160 tot-seq=4

TGGGGAATCTTGCGCAATGCGCGAAAGCGTGACGCAGCAACGCCGCGTGGGGGAAGAAGG

CTCTCGGGTTGTAAACCCCTTTCAGGGAGGACGAAGGCATGGCCGTTAATAGCGGATCAT

GCTGACGGTACTCCCACAAGAAGCCCCGGCTAACTACGTG

>2570 len=160 tot-seq=4

TGGGGAATATTGCACAATGGGCGCAAGCCTGATGCAGCAACGCCGCGTGAGTGAAGAAGG

GTTTCGGCTCGTAAAGCTCTGTTGTTGGAGAAGAACGTATCTGAGAGTAACTGTTCAGGT

AGTGACGGTATCCAACCAGAAAGTCACGGCTAACTACGTG

>2571 len=160 tot-seq=4

TGGGGAATATTGCGCAATGGGGGAAACCCTGACGCAGCGACGCCGCGTGCGGGATGAAGG

CCTTCGGGTTGTAAAACCCTGTTGTTAGAGAAGAAAGTGCGTGAGAGTAACTGTTCACGT

TTCGACGGTATCTAACCAGAAAGCCACGGCTAACTACGTG

>2572 len=160 tot-seq=4

TAGGGAATCTTCCACAATGGGCGAAAGCCTGATGGAGCAACGCCGCGTGGGTGAAGAAGG

TCTTCGGATCGTAAAACCCTGTTGTTAGTGAAGAAGGATAGAGGCAGTAACTGGCCTTTA

TTTGACGGTAATCAACCAGAAAGTCACGGCTAACTACATG

>2573 len=160 tot-seq=4

TAGGGAATCTTCGGCAATGGGGGGAACCCTGACCGAGCAACGCCGCGTGAGTGAAGAAGG

ATTTCGGTTCGTAAAACTCTGTTGTTAGAGAAGAACAGCGCATAGAGTAACTGTTATGCG

TGTGACGGTATCTAACCAGAAAGCCACGGCTAACTACGTG

>2574 len=160 tot-seq=4

TGGGGAATATTGCACAATGGGGGAAACCCTGATGCAGCGACGCCGCGTGAGCGAAGAAGT

ATTTCGGGTTGTAAAGTACTTTCAGCGGGGAGGAAGGGAGTAAAGTTAATACCTTTGCTC

ATTGACGTTACCCGCAGAAGAAGCACCGGCTAACTCCGTG

>2575 len=160 tot-seq=4

TAGGGAATCTTCCACAATGGACGCAAGTCTGATGGAGCAACGCCGCGTGAGTGAAGAAGG

GTTTCGGCTCGTAAAACTCTGTTATTAGGGAAGAACATACGTGTAAGTAACTATGCACGT

CTTGACGGTACTTAATCAGAAAGCCACGGCTAACTACGTG

>2576 len=160 tot-seq=4

TGGGGAATATTGGACAATGGGGGGAACTCTGATCCAGCAATTCTGTGTGTGTGAAGAAGG

TTTTCGGATCGTAAAACTCTGTTGTAAGAGAAGAACAAATTGTAGAGTAACTGCTACAGT

CTTGACGGTATCTTACCAGAAAGCCACGGCTAACTACGTG

>2577 len=160 tot-seq=4

TAGGGAATCTTCCACAATGGGCGCAAGCCTGATGGAGCAACACCGCGTGAGTGAAGAAGG

GTTTCGGCTCGTAAAACTCTGTTGTTGAAGAAGAAAGATAGAGGTAGTAACTGGCCTTTA

TTTGACGGTAATTACTTAGAAAGTCACGGCTAACTACGTG

>2578 len=160 tot-seq=4

TGGGGAATATTGGACAATGGGCGCAAGCCTGATCCAGCCATGCCGCGTGTGTGAAGAAGG

CCTTCGGGTTGTAAAGCACTTTCAGCGAGGAGGAAGGGATGTATGTTAATAGCATGCATT

TTTGACGTTACTCGCAGAAGAAGCACCGGCTAACTCTGTG

>2579 len=160 tot-seq=4

TGGGGAATATTGGACAATGGGCGCAAGCCTGATCCAGCCATGCCGCGTGTGTGAAGAAGG

CCCTAGGGTTGTAAAGCACTTTCAGTGGGGAGAAAGGCCTTTTACCTAATACGTAAGAGG

ATTGATGTTACCCACAGAAGAAGCACCGGCTAACTCCGTG

>2580 len=160 tot-seq=4

TGGGGAATTTTGGACAATGGGCGCAAGCCTGATCCAGCAATGCCGCGTGCGGGAAGAAGG

CCTTCGGGTTGTAAACCGCTTTTGGTAGGAACGAAAAGCTCTGGGCTAATACCCTGGGGT

GATGACGGTACCTGCAGAATAAGCACCGGCTAACTACGTG

>2581 len=160 tot-seq=4

TGGGGAATCTTGCGCAATGCGCGAAAGCGTGACGCAGCAACGCCGCGTGGGGGAAGAAGG

CCTTCGGGTTGTAAACCCCTTTCAGTTGGGACGAAGCTTCGTCGGTGAATAGCCGGCTGG

AGTGACGGTACCTTCACAAGAAGCCCCGGCTAACTACGTG

>2582 len=160 tot-seq=4

TAGGGAATCTTCCACAATGGGCGCAAGCCTGATGGAGCAACACCGCGTGAGTGAAGAAGG

GTTTCGGCTCGTAAAGCTCTGTTGTTGGAGAAGAACGTATCTGAGAGTAACTGACCTTTG

TTTGACGGTAATCAATTAGAAAGTCACGGCTAACTACGTG

>2583 len=160 tot-seq=4

TAGGGAATCTTCCACAATGGACGAAAGTCTGATGGAGCAACGCCGCGTGAGTGAAGAAGG

TTTTCGGATCGTAAAGCTCTGTTGTTAAAGAAGAACACGTATGAGAGTAACTGTTCATAC

GTTGACGGTATTTAACCAGAAAGTCACGGCTAACTACGTG

>2584 len=160 tot-seq=4

TAGGGAATTTTCGTCAATGGGGGGAACCCTGAACGAGCAATGCCGCGTGAGTGAAGAAGG

TCTTCGGATCGTAAAGCTCTGTTGTAAGAGAAAAACGGATCATGTAGGGAATGACATGAT

AGTGATGGTATCTTACCAGAAAGCCACGGCTAACTACGTG

>2585 len=160 tot-seq=4

CTAAGAATCTTCCACAATGGACGAAAGTCTGATGGAGCGACGCCGCGTGGACGACGAAGG

CCGTGAGGTTGTAAAGTCCTTTTCCGGAGGGGGAACAACCCGTGCAGGGAATGGCATGGG

GATGACGTGAATCCGGGAATAAGCCCCGGCTAACTACGTG

>2586 len=160 tot-seq=4

TAGGGAATCTTCCACAATGGACGCAAGTCTGATGGAGCAACGCCGCGTGAGTGAAGAAGG

GTTTCGGCTCGTAAAGCTCTGTTGGTAGTGAAGAAAGATAGAGGTAGTAACTGTTCACGC

AGTGACGGTATCCAACCAGAAAGTCACGGCTAACTACGTG

>2587 len=160 tot-seq=4

TAGGGAATATTCCACAATGGGCGCAAGCCTGATGGAGAAAAAAAGAGTGAGTGAAGAAGG

GTTTAGGATAGTAAAGCTCTGTTGTTGGAGAAGAACGTGCGTGAGAGTAACTGTTCACGC

AGTGACGGTACCCAACCAGAAAGTCACGGCTAACTACGTG

>2588 len=160 tot-seq=4

TAGGGAATCTTCCACAATGGACGAAAGTCTGATGGAGCAACGCCGCGTGAATGAAGAAGG

CCTTCGGGTCGTAAAATTCTGTTGTTGGAGAAGAACGTGCGTGAGAGTAACTGTTCACGC

AGTGACGGTATCCAACCAGAAAGTCACGGCTAACTACGTG

>2589 len=160 tot-seq=4

TAGGGAATTTTCGGCAATGGGGGGAACCCTGACCGAGCAACGCCGCGTGAAGGAAGAAGG

AATTCGTTCTGTAAACTTCTGTTATAAAGGAAGAAAGAGGCAGTCAGGAAATGGACTGCA

GGTGACGGTACTTTATGAGAAAGCCACGGCTAACTACGTG

>2590 len=160 tot-seq=4

TGGGGAATATTGCGCAATGGGGGAAACCCTGACGCAGCGACGCCGCGTGTGTGAAGAAGG

TCTTCGGATTGTAAAGCACTTTAAGTTGGGAGGAAGGGTTGTAGATTAATACCCTGCAAT

TTTGACGTTACCGACAGAATAAGCACCGGCTAACTCTGTG

>2591 len=160 tot-seq=4

TGGGGAATTTTGGACAATGGGGGCAACCCTGATCCAGCCATACCGCGTGTGCGATGAAGG

CCTTCGGGTTGTAAAGCACTTTTGGCAGGAAAGAAACGTCGCGGGCTAATACCCCGTGGA

ACTGACGGTACCTGCAGAATAAGCACCGGCTAACTACGTG

>2592 len=160 tot-seq=4

TGGGGAATATTGGACAATGGGGGCAACCCTGATCCAGCAATGCCGCGTGTGTAAAGAAGG

CCTGCGGGTTGTAAAACACTATCGTTGGGGAGGAAGGTGTTGAGTCGAATAGGCTTAACA

ATTGACGTTACCCAAAGAAGAAGCACCGGCTAACTCTGTG

>2593 len=160 tot-seq=4

TAGGGAATATTCCACAATGAGCGAAAGCTTGATGGAGCGACACAGCGTGCACGATGAAGG

TCTTCGGATTGTAAACTGCTTTTGTCAGGGAAGAAAAGGTCTGTGTTAATACCATAGACT

GCTGACGGTACCTGAAGAATAAGCACCGGCTAACTACGTG

>2594 len=160 tot-seq=4

TGGGGAATATTGCGCAATGGGGGAAACCCTGACGCAGCGACGCCGCGTGCGGGATGAAGG

CCTTCGGGTTGTAAACCGCTTTTGGCAGGAACGAAACGGTGTCCGCTAATACCGGGCGCT

AATGACGGTACCTGCAGAATAAGCACCGGCTAACTACGTG

>2595 len=160 tot-seq=4

TAGGGAATCTTCCGCAATGGACGCAAGTCTGACGGAGCAACGCCGCGTGTGTGAAGAAGG

TCTTCGGATCGTAAAGCACTGTTATCAGAGAAGAACACCCCATAGAGTAACTGTTATGGG

ATTGACGGTAACTGATCAGAAAGCCACGGCTAACTACGTG

>2596 len=160 tot-seq=4

TGGGGAATTTTGGACAATGGGCGCAAGCCTGATCCAGCAATGCCGCGTGCGGGAAGAAGG

CCTTCGGGTTGTAAACCGCTTTTGTACGGAACGAAAAGGCTCTGGCTAATACCTGGGGCT

GATGACGGTACCGTAAGAATAAGCACCGGCTAACTACGTG

>2597 len=160 tot-seq=4

TGGGGAATATTGCGCAATGAGGGAAACCCTGACGCAGCAACGCCGCGTGAGTGAAGACGG

CCTTCGGGTTGTAAAGCTCTGTGATTCGGGACGAAAGGCCATATGTGAATAATATATGGA

AATGACGGTACCGAAAAAGCAAGCCACGGCTAACTACGTG

>2598 len=160 tot-seq=4

TGGGGAATATTGGACAATGGGCGAAAGCCTGATCCAGCCATGCCGCGTGGGTGAAGAAGG

CCTTCGGGCTGTAAAGCCCTTTTGTTGGGGAAGAAATCCTGCTGGCTAATATCCGGCGGG

GATGACGGTACCCAAAGAATAAGCACCGGCTAACTTCGTG

>2599 len=160 tot-seq=4

TGGGGAATATTGCACAATGGGCGAAAGCCTGATGCAGCGACGCCGCGTGAGTGAAGAAGG

CCTTCGGGTTGTAAAACTCTGTCCTCATCCAAGAGAAGGGAAGGTAGTAACTGACCTTTC

CAGGACGGTAGATGAGGAGGAAGCCCCGGCTAACTATGTG

>2600 len=160 tot-seq=4

TGGGGAATATTGCGCAATGGGGGAAACCCTGACGCAGCCATGCCGCGTGTATGAAGAAGG

CCTTCGGGTTGTAAAGTACTTTCAGCGGGGAGGAAGGCGACAGGGTTAATAACCCTGTCG

ATTGACGTTACCCGCAGAAGAAGCACCGGCTAACTCCGTG

>2601 len=160 tot-seq=4

TGGGGAATATTGCACAATGGGCGCAAGCCTGATGCAGCCATGCCGCGTGTATGAAGAAGG

CCTTCGGGTTGTAAAGTACTTTCAGCGGGGAGGAAGGGGTTAAGGTTAATAACCTTAGCC

ATTGACGCTACCCGCAGAAGAAGCACCGGCTAACTCCGTG

>2602 len=160 tot-seq=4

TGGGGAATATTGCGCAATGGGGGAAACCCTGACGCAGCCATGCCGCGTGTGTGAAGAAGG

TCTTCGGATTGTAAAGCACTTTAAGTTGGGAGGAAGGGCAGTAAATTAATACTTTGCTGT

TTTGACGTTACCGACAGAATAAGCACCGGCTAACTCTGTG

>2603 len=160 tot-seq=4

TGGGGAATATTGGACAATGGGGGCAACCCTGATCCAGCGATGCCGCGTGAGTGAAGAAGG

CCCTCGGGTCGTAAAGCTCTTTAGGTTGGGAAGAAGTATGGTGGAGGATAATGCCATCAT

GTTGACGGTACCAACAGAATAAGCACCGGCAAACTCTGTG

>2604 len=161 tot-seq=4

TGGGGAATATTGCGCAATGGGGGAAACCCTGACGCAGCGACGCCGCGTGAACGATGACGG

CCTTCGGGTTGTAAAGTTCTGTTATATGGGACGAACAGGGCATCGGTTAATACCCGGTGT

CTTTGACGGTACCGTAAGAGAAAGCCACGGCTAACTACGTG

>2605 len=161 tot-seq=4

TGGGGAATCTTCCGCAATGGGCGCAAGCCTGACGGAGCAACGCCGCGTGAGTGAAGAAGG

GTTTCGGATCGTAAAGCTCTGTTGTTAGAGAAGAACGTTGGTAGGAGTGGAAAATCTACC

AAGTGACGGTAACTAACCAGAAAGGGACGGCTAACTACGTG

>2606 len=161 tot-seq=4

TGGGGAATATTGCACAATGGGCGCAAGCCTGATGCAGCGACGCCGCGTGAGTGAAGAAGG

TTTTCGGATCGTAAAACTCTGTTGGTAGAGAAGAACGTTGGTGAGAGTGGAAAGCTCATC

AAGTGACGGTAACTACCCAGAAAGGGACGGCTAACTACGTG

>2607 len=161 tot-seq=4

TGGGGAATATTGCACAATGGGCGCAAGCCTGATGCAGCCATGCCGCGTGTATGAAGAAGG

CCTTCGGGTTGTAAAGCTCTGTTGTAAGAGAAGAACGAGTGTGAGAGTGGAAAGTTCACA

CTGTGACGGTATCTTACCAGAAAGGGACGGCTAACTACGTG

>2608 len=161 tot-seq=4

TGGGGAATCTTCCGCAATGGGCGAAAGCCTGACGGAGCAATGCCGCGTGAGTGAAGAAGG

CCTTCGGGTTGTAAAACTCTGTCCTTATCGAAGAGAGGGGAGCATGTGAATAATGTGTTC

TTAGGACGGTAGATAAGGAGGAAGCCCCGGCTAACTACGTG

>2609 len=161 tot-seq=4

TAGGGAATCTTTCGCAATGGACGAAAGTCTGACGGAGCAACGCCGCGTGAGCGAAGAAGG

TCTTCGGATTGTAAAGCTCTGTTGTTAGGGAAGAACAAGTACCGTTCGAAGAGGGCGGTA

CCGTGACGGTACCTAACGAGAAAGCCACGGCTAACTACGTG

>2610 len=161 tot-seq=4

TGGGGAATATTGCGCAATGGGGGAAACCCTGACGCAGAGACGCCGCGTGAGTGATGAAGG

TCTTCGGATTGTAAAACTCTGTTGTCAGGGACGAATGTACTGATCTATAATACATTTCGG

TATTGACGGTACCTGACGAGGAAGCCACGGCTAACTACGTG

>2611 len=161 tot-seq=4

TGGGGAATCTTCCGCAATGGACGAAAGTCTGACGGAGCAACGCCGCGTGAACGATGACGG

CCTTCGGGTTGTAAAGTTCTGTTAATCGGGACGAATGGTTTGTGTGCAAATAGTGCATAG

ACATGACGGTACCGGAATAGAAAGCCACGGCTAACTACGTG

>2612 len=161 tot-seq=4

TGGGGAATATTGCGCAATGGGGGAAACCCTGACGCAGCGACGCCGCGTGCGGGATGAAGG

CCTTCGGATTGTAAAACTCTGTTGTCAGGGACGAATGTACTAATCTATAATACATTTCGG

TATCGACGGTACCTGACGAGGAAGCCACGGCTAACTACGTG

>2613 len=161 tot-seq=4

CAGTGCCTCTCCTGAGAGGAGCTGGTGGGACTGAGATGGATGGGACTGAGATGGATGGGG

CTGGGGCAGGGGAGGAGGAGAAGTCAGTCCTGGGTCACAGATACCACACTGGCCCCTAAA

TGGAGCAAAGAAACAGCCAGAAGCATCCAGACCTCTGCAAA

>2614 len=161 tot-seq=4

TGGGGAATCTTGCACAATGGGCGAAAGCCTGATGCAGCGACGCCGCGTGCGGGATGAAGG

CCTTCGGGTTGTAAAGCTCTGTTAATCGGGACGAATGGTCGGTGTGCAAATAGTGCATGG

ACATGACGGTACCGGAATAGAAAGCCACGGCTAACTACGTG

>2615 len=161 tot-seq=4

TAGGGAATCTTCCACAATGGACGCAAGTCTGATGGAGCAACGCCGCGTGAGTGAAGAAGG

TTTTCGGATCGTAAAACTCTGTTGTTAGAGAAGAACAAGGATGAGAGTAGAATGTTCATC

CCTTGACGGTATCTAACCAGAAAGCCACGGCTAACTACGTG

>2616 len=161 tot-seq=4

TGGGGAATCTTCCGCAATGGACGAAAGTCTGACGGAGCAACGCCGCGTGAGTGAAGACGG

CCTTCGGGTTGTAAAGCTCTGTGATCCGGGACGAAAGAGCCTGAGGTAAATAGCCTTGGG

AAGTGACGGTACCGGAAAAGCAAGCGCCGGCTAACTACGTG

>2617 len=161 tot-seq=4

TGGGGAATATTGCGCAATGGGGGAAACCCTGACGGAGCAACGCCGCGTGAGTGATGACGG

TCTTCGGATTGTAAAGCCCTGTTAATCGGGACGAATGGTTTGTGTGCAAATAGTGCATAG

ACATGACGGTACCGGAATAGAAAGCCACGGCTAACTACGTG

>2618 len=161 tot-seq=4

TAGGGAATATTCCACAATGAGCGAAAGCTTGATGGAGCGACACAGCGTGCACGATGAAGG

TCTTCGGATTGTAAAGCTCTGTTAATCGGGACGAATGGTTTGTGTGCAAATAGTGCATAG

ACATGACGGTACCGGAATAGAAAGCCACGGCTAACTACGTG

>2619 len=161 tot-seq=4

TGGGGAATATTGGGCAATGGGCGAAAGCCTGACCCAGCAACGCCGCGTGAGTGAAGAAGG

TCTTCGGATTGTAAAACTCTGTTATACGGGACGAAAAAGGCGGATGCCAACAGTATCCGT

CCGTGACGGTACCGTAAGAGAAAGCCACGGCTAACTACGTG

>2620 len=161 tot-seq=4

GAGGGGGTGTGAGGCAGTCAGTGGAGAGAACAGGAGAGATCATGAAGGTGCTAGAAGGGG

AGAAGGCCTGAGGACAGCGCGCGCTGGCTTTGCCCTTGGAGCTGGAGCCCTTCCGGAATC

TCCTGGCTTCTGCCCTTCCCAGCAGTCCTGTGGATCAATAT

>2621 len=161 tot-seq=4

TGGGGAATATTGCGCAATGGGCGAAAGCCTGACGCAGCAACGCCGCGTGAGTGATGAAGG

CCTTCGGGTCGTAAAGCTCTGTCGGAGGGAACGAATGCTCTTGAGGTTAACAGCCTCGGG

AGGTGACGGTACCCTCAAAGGAAGCACCGGCTAACTCTGTG

>2622 len=161 tot-seq=4

TTAGGAATATTCGTCAATGGGGGAAACCCTGAACGAGCAATGCCGCGTGAAGGATGACGG

TCCTATGGATTGTAAACTTCTGTTGTTAGGGAAGAACGACTTAAGTAGGAAATGACTTAA

GAGTGACGGTACCTTTCAAGAAAGCTCCGGCTAACTACGTG

>2623 len=161 tot-seq=4

TGGGGAATATTGGACAATGGGCGCAAGCCTGATCCAGCCATGCCGCGTGTGTGAAGAAGG

CCTTTTGGTTGTAAAGCACTTTAAGCGAGGAGGAGGCTCCTTTAGTTAATACCTAAAGTG

AGTGGACGTTACTCGCAGAATAAGCACCGGCTAACTCTGTG

>2624 len=161 tot-seq=4

TGGGGAATTGTTCGCAATGGGCGCAAGCCTGACGACGCAACGCCGCGTGGAGGATGAAGA

TCTTCGGGTCGTAAACTCCTGTCGAGCGGGAAGAAAACCCCTGGCGTTAACAGCGCCGGA

GGTTGACGGTACCGCGAGAGGAAGCCCCGGCTAACTCCGTG

>2625 len=162 tot-seq=4

AGTCGGAAAGCCTGGGAAAAAAAGACTTCTACTTGGAAAGGAGCAACTTTTTAAGTTAGA

AAACACATTTTTCTTTTCTGCTTTCGGTCACTCATCTTTGCAAAAATAGAAGTAACTATC

AATTTATCCAAAATATAAAAGAACATAGGAATGAGAGCACTG

>2626 len=162 tot-seq=4

TGAGGAATATTGGTCAATGGGCGGGAGCCTGAACCAGCCAAGTAGCGTGCAGGAAGACGG

CCCTATGGGTTGTAAAACTCTGTTATACGGGACGAAAAAGACGGATGCCAACAGTATCCG

TCCGTGACGGTACCGTAAGAGAAAGCCACGGCTAACTACGTG

>2627 len=166 tot-seq=4

TGGAGGCTCTGAGAGGTGTCCTGGCTTGCTTACAGCAGTTATGGGAGGAGGCCTCAATTT

CTCTTTCTGTCTCTGGCATTGCTACTGGAGCAAAGTGGCTGGAAATGGGGAATAATCCCA

CTGGGGCCTGCGAAGATCTGTGGATCTGAGAATCCGCTGACCCGGA

>2628 len=169 tot-seq=4

GGCGGGGAGGGTGCCAGTGTACAGGTTACCCCGGTAGAGCTCTGTGTTCCTGGAATATTT

CCACTGTTAATTATTCATCGGGTTTTAATTAGAAGCAGGCACTTCCCCTGAAAAAGTGCC

ACCAACAGCTAACTTCAAAAACAGCCGACTCCCTAGCTGTGCCACCTAA

>2629 len=170 tot-seq=4

AAGAGGTAAGGGAATGGAAGACATCACATGACTAAGAGGATGGGAAGGGGTGCTATAAAA

CTGTTTTTTAGATATGCCATGACCATTGCAATCATGAGCATGGCACAGCTGTGCTTACCT

GCAGATGACCTGCATGAAAAGCTAAGACCTGAATAGGAAGGAGAGGAGAA

>2630 len=171 tot-seq=4

CAGCAGCTTTTCCTTTCAAGCCCTGTTTTTGGATGGTGCATTTTTACTGTTGGAGGATTC

TTGTACCTTCCCATCGTCATCCTAAGTTGAGCGTATACTGTTTTCTACTATCCTGTGAAA

ACAGAGCGGTTTGCGTCTCAGGCTTTGGCTTGCTTCTTCACCCCCAGCTCT

>2631 len=171 tot-seq=4

GGAGCCCCATCCTCGATGTGAAGGGCGCTTGCTCAGGTTGCTGCAACAAGCCCTGGCGTT

TGAGCAGGGCCAAAGTTTCCTCATCACCTGAACTTTCCCTTTCATGCTGTTCTTCTAAGG

CCAGCTGGCGCTGGGCATTCCACAGTCCTCGCAAGGCATTCAGGCGGTATT

>2632 len=172 tot-seq=4

TGGAGCCAGGACAGCAGCCCCACCGAGCGGAAGGCATCACACACAAAATGGGAGGAGCCC

ATGACAGTCTGCCCACAGAGCAAGGGGCCCCGCCTTCCGTGCCCTGACCGGAGATCAGGC

AACAGATGGAGGACGCGTCAGTGGACAAAACAGCTAGGTTCCTGCCCAGTCC

>2633 len=172 tot-seq=4

GGAGGAGGCTGAAGAGGGACATGGACTGGTAGCATGGAGGCTGCTGATAACCACCCCAGG

GCCTGACCACAGTGGTGGGACATGGGGGAGGTCAAGGAATGGATGCCACCCTTCCAAGAA

AAGGGTGCACAGAGCAGGCTGCAGGAAAGGCTCTGACGCCCTCACCCTGCAC

>2634 len=173 tot-seq=4

CGGGAGGCAGCAGGAGGGAATCTGCCACAAGGGACGAAAGGCGGAGGGAGCAACGCCGCG

TGAGTGAAGAAGGGTTTCGGCTCGTAAAGCTCTGTTGGTAGTGAAGAAAGATAGAGGTAG

TAACTGGCCTTTATTTGACGGTAATTACTTAGAAAGTCACGGCTAACTACGTG

>2635 len=174 tot-seq=4

TCTCATCCAGCTGACGGTTGATGTCGGTAACATGTTTATCGGAGATCGGATAAACCTGCA

TGATCACCATCGAAATAGCGGCCAGCGCGATTGGGATCCACACCGCGGTCATAATGATCC

CATCGATGGCGCTGGCGCTCTGCACGGCGCCGCTTTCATTAAATCCATACACCG

>2636 len=176 tot-seq=4

AGACAGGCAGCCCACAGGAGTTGTCCTCAGGTCGCTGCCCCTCAGCGGGCCTGTGAGCAG

TTGCGTCACCCTCATCTCTGGTTGAGGCCAAGGTGGCCCGTAGCACCCTGAGAGCACATT

CCTACCCCAGCTGATGACCCGGCCCATGAGAATTCCTGCCACAGGAGAAGCTCTGA

>2637 len=179 tot-seq=4

CCGACGAGCTAGAAACAGAAGCAGCGGAAGCAGCATCATTATTCGCCTTTTCGACTCCCC

TCTTGTTTAACTTATTGTTATGCACGCCGTCAGTGTAAACGAAATCGTTTAGCAACGCAT

AGCCGGTTCCAATTCCAGCAGAATTATTTCCATCGGAATTATTTCCAGCAGAATTATTT

>2638 len=180 tot-seq=4

TGGAGTCAGACGTGTGCTCTTCCGATCTCCTATGGGAGGCAGCAGTGGGGAATATTGCAC

AAGGGGCGAAAGCCGGATGCAGCGACGCCGCGTGAGCGAAGAAGTATTTCGGTATGTAAA

GCTCTATAAGAAGGGAAGAGAATGACGGTACCTTACTAAGAAGCTCCGGCTAAATACGTG

>2639 len=181 tot-seq=4

TGGGGGATATTGCACAATGGAGGAAACTCTGATGCAGCGACGCCGCGTGAGGGAAGAAGG

CCTTCGGGTTGTAAACCTCTGTCTTTGGGGACGAAGAAAATGACGGTACCCAAGGAGGAA

GCCACGGCTAACTACGTGCCAGAGCCGCGGTAATTTTTAGATCGGAAGAGCGTCGTGTAG

G

>2640 len=182 tot-seq=4

AAGCTAGCCTGGGCTAAATGAGACCCTGTCTCAAACAAACAAAATTTAAAAAAAAATCTA

TTTTCAATTTAGGTGAAAAACATCTCTGCATACTAGAAAAAGATGACCCAAAGTGGCAGA

AATCTGAAGATCAAATCTTAAGATTGGGAAAACAGGGACACCTGCCCTCTTCATCGCCCT

TC

>2641 len=182 tot-seq=4

TGGGGAATATTGCACAATGGGGGAAACCCTGATGCAGCGACGCCGCGTGGAGGAAGAAGG

TCCTCGGATTGTAAACTCCTGTTGTTGAGGAAGATAATGACGGTACTCAACAAGGAAGTG

ACGGCTAACTACGTGCCAGCAGCCGCGTAATCAACAGATCGGAAGAGCGTCGTGTTGGGA

AA

>2642 len=182 tot-seq=4

AGAAAGAGGGTCTGGCAGGAGGGGCCAGGACGGGGCTCCACTGGAAAGAAGAGGCACAGA

TCAAAGGGGACAGGTTCAGGAAGGGGTGCTGTCCACAGCGTCAAGTGCTGCCAAGAGGTC

AAGATGGCGGATGAGGAAGGAGGATGCTGGACAGGGCATTGGTAACTGTGGCTGGGTCAG

CT

>2643 len=184 tot-seq=4

TTTGAATCACTGAGCCATCTCTCCAGCTCTGCCCAGTCTTTCTTTACCCTTGCAGGTTCA

TGGAGTTTGAGGCCGAGGAAGAAACACAGATACAGAATGCACAGTTGATGAATGGCTCCC

CGGGTCTGTCCCCTATAGCCCCTTTGAAACTTGATCCTCCAGGGTCCATGGTACCTGAGG

CTTG

>2644 len=184 tot-seq=4

CCTCCTCTGTCCCCCAGAGGTGAACTGTCGGAGATGGAAAAGCACGAGTGCAGTGAGTGC

CCAGAGCCCAGTGCAAGGTAGGCAGCAGCTGGCACCCAGCACCCGAGAGCCCAGTGCCAG

GACAGGAGACGTCCCAACAGCCAGCACCCGAGACAGCCCAGCAGCAGCCAGCGCCTGAGA

TAGC

>2645 len=184 tot-seq=4

ACAGGAGGGGAAACTGAGGCTTGGAAAAGGCACATGGTCCCATGTAGCGTCACTGTGGCT

CTTCGGACTCTTCCAGGTCCCTCTGCCTGTCCTGGGCCTGCCATTGTTCCACCTCCAGGT

CTGCCCACTGCCTGCACCTCTCCTCCCCGTCTCACCTTGCCCTGCACTGCAGCTGCCACG

ACCT

>2646 len=185 tot-seq=4

TGGGGAATATTGCACAATGGGGGAAACCCTGATGCAGCGACGCCGCGTGGAGGAAGAAGG

TCTTCGGATTGTAAACTCCTGTTGTTGGGGAAGATAATGACGGTACCCAACAAGGAAGTG

ACGGCTAACTACGTGCCAGCAGCCGGGTAATTTCTAGATCGGAAGAGCGTCGTGTAGGGA

AGGAG

>2647 len=186 tot-seq=4

TTTTTTTTTTCAAGCAGAAGACGGCATACGAGATCAAGTGGTGACTGGAGTTCAGACGTG

TGCTTTTCCGATCTCCTACGGGACGCAGCAGGATTGATTGGGGGTCAGCAGCTAGTTTGA

GCCAGAGCGGGACTGAGAGGCCGAGCCTCCTGCCCCTGCGGCTGCCTGGCCTGGCTCACC

ACTTCC

>2648 len=187 tot-seq=4

GTAAAGATTCCAGCCATCTCTCAGTTGGACACTCCTGGGAAGCATCCTGTCTCCTTCTCA

CAAGTTCTAGAAGAACCAACAGCACACCTATTGCTAGAACCCAAGAACACACAGCCCTTC

CTGCAGCCTTATCTCGCTCTTCCTACAACATGTTTCCACCTCCTTGGCCGTTCATCTGAG

CCGACAG

>2649 len=187 tot-seq=4

GACCTGGGCTTATGCCCTTGTCACATTCTGTATGAAGCCAGGAGGATGGAGCTTGATGAC

ATCTCCTAACGATACCCACATGCACATCTCTGTCATCCTTAAAGCACTGAGTCTTGGACT

TCAGGTTCTCCTTGTGCCGGTCCACAAGGGAACCAACTCACTCCACGGTTTTCTGCTTGG

CTTCCTC

>2650 len=187 tot-seq=4

CAGGGAGAGGCCCACGATGGGGCCTGCCACCAGCACTCAGACTACCCATATTTGGGCTTA

GGCCGAGTTCCAGCCAAGGAAAGGGCAAAAAGCAAATTAAAATCCGACAATGAGAATGAC

GGGTATGTCCCCGATGTGGAAATGAGTGACTCAGAGAGTGAGGCATCAGAAAAGAAATGT

ATACACA

>2651 len=188 tot-seq=4

GGCGAGGGGAGCCCTGGATGGATGGGGGAGGCGCAGATGTGCGGCCACTGTTCCAGGACA

TTGGCCACTGGAGTGCCAGGGCAGAGGAAGAGGAGACACCCGGCAGGACAGTGAAGGGGC

AGTGAGGAGGGAGGAGCAGTCCTGAGGAAGCAAGGGGGCAAGAGTGCAGTGAGACGACGA

GACAGTGT

>2652 len=189 tot-seq=4

ATTGTTAATTTCTTTGGCTTACACATGCCTTGGATTTATCCTTGGAGAGAGTCTGTTATG

GGAAGAGCTGATGTTAATGGTGGATTGACTTTCTGTAACGTAGATAATTCAAAGTAGGTC

TATGAACGGAAAGCTCATTAAAATTGGAAATGGGGAGGTGTCCGCCTGGCTTGGGAGGCG

GACTCAGCC

>2653 len=190 tot-seq=4

GATTAACATACATCATATACATGCATGAAATTGCTAAAGAATGACATGTTAACTCCGCTC

TGGGATCCAGAATCTTCTGGGGCGCTCTGGAACCTCAAGCCCATGTCTGCTCCTCTCTGA

GCCTTAATTTCTCCAAGAGTGAAGTGAGAAGGCTGGGGCCTCCTGGGTCCCCAGGACAGG

AAAATGCCTG

>2654 len=191 tot-seq=4

AGAAATCTCAACCCTAAGAGAGACTTTGCTTTTCTCAAGTTTCTTGTCATTTGCACCTCA

AAGGCCACAAAAACTAGCCAAATCCTCCCTGCCAGTCCAGATTCCCTTATCATTTACAAA

CAAATTTGCATGTGGTGGCTCAGCCAGCAGCCGCGGGTAATTTCCAGATCGGAAGAGCGT

CGGGTAGGGAA

>2655 len=192 tot-seq=4

GGAGTTGTTGTGAGGCAGAGTGAAGGGGATACGATATTTCGGTTCTCCTGTGGATCTGTC

TTTGAGTGCAGTGTGCTCTCACTAACCCTCTCCACCCCCCACCCCAGGTAGGTCCTATTG

CCCAGGCAAAGCAGTTTCGTGAAGAGTAGTGCCAGAGAGGGACTGACACCTATGGACTGT

GGGCAAACTTGT

>2656 len=192 tot-seq=4

GTCAGTGATGGTGGAAAAGAGACCCATGTGCTCTGGGAAATGCTAAAAACACCTACAGTA

ACCAGGGATCCCAAGCTTTGCCCAGCTACCTTTCAAGGAAGAGCTGGGAGAAGGCCTCCT

GTGGTCCCATGAAGCTCAGCAGAGAGTCGTGGGCATTGACAGGAACAGGGCAAAGCAGAG

ATGAGCCTAGGG

>2657 len=194 tot-seq=4

AACTGCTCTTCCGATCTCCTACGGGAGGCAGCGGGGCTAACGGATCAGTGAATTTTCCGC
TGACAGCAGGCCTTGAGGGCCATGGGATCATTCTGTGCTTCAGCGAGGCCCTGCCTGCCT
CGGCAGATGTGGTGAGCCCATCCTTTCTCGCCCAGAGTGGTCCAAAATCGCATCTGAAGA
GGAGTCCTGAGAAC
>2658 len=194 tot-seq=4
AAGCAGTATGGTGTGGTGCTTCAAACGGTGGTATGTCACTAATTCCATAGTAAGGAAGTG
TGCCACAAAGTGAACGGATTGATGCAACTTCAAGCTGCTCTTGTTCATTTAGGGGACACA
TAATACTTGGTAAGCGAGAAGCAAGCATAGTTTTTCCAGAGCCAGGAGGGCCAATCATCA
TCACATGATGTCCT
>2659 len=194 tot-seq=4
CAGAGAGTAGGCGGCTGGGCCACGTCCTTCACAGGGCGTCATGTGCCTTTCTATTTTCAT
CTTAGAAAATTTCCTCAGCAAACCGATGAGAGATTGTGGTTGCAAGCTTCAGTATTTGCC
TCGCTTCCCTTTTCATATTTACAGAATTAAAACAACCTCAAGTACCTCAGACTCTGCATT
CCAAACCAAGGCAC
>2660 len=196 tot-seq=4
ACAGTTGAAAATAATCTAGTGCAGACATCCATAAGCTACTGAGGTATTTCAGAAAGATAA
CAAAAAGCTAACAAATTGCATGTCTTTTCCACTTACCATTACTTAGTGTTTCTAATAAGT
AAATGTGGTTTCTCAGCTGAACTGCTCTTTCAAATTATTCTTAGTTAAGTAATATTCCCT
GTGAAATCTGTCATTA
>2661 len=196 tot-seq=4
TGAAGAGAGCAGTGGTTCTCCCAGCAAGGTGTTTGAGCTCTGAGAACAGACAGAGTGCCT
CCTCAAGTGGGTCCCTGACTCCCACGTAGCCTAACTGGGAGACATCTCCCAGTAGGGGCC
AACTGACACCTCACACAGGTGGGTGCCCCTCTGGGACAAAGCTTCCAGATATTTGTTGTT
CTGCAATATTTGCTGT
>2662 len=199 tot-seq=4
TTGTTTGCGAGGATTGAGGCTGTGCTGGCAAAGTGTGTACAATAGCGATGTCCTAAGAAA
GCATCTACAGAAGACCAAAGCACTATGGAGTGATCCAGGCAAACGGGAGAAGTTCGGTGT
GGAAGGAAGAAAAAGTACACGGGCAACCCCACATCTTCAGCTTAGATGCCTGACTTTTCA
GCTTCCACCCTCTCAGTCA

>2663 len=199 tot-seq=4

CAGACAATGTTTCTTTAAAAGTCTCCATCTGCAAATCCCATTAGATGTGCATATAAGGTT

TCTGATGATTAATAGTTGCCTGCTAGGGTTTTGGTTGGAGGGGATCTTGTGTGTGTGGGT

TAACCCCTCCCTCTCCTCTCTCACATTTCACTTCAGAAATTTGTCACTCTCTCCCCACCG

CAGGATTTAAAAGTGTGTC

>2664 len=199 tot-seq=4

TTCTCTAATTCCCAGCTGTCTACCCCCCTCTGGTGGCCACTTCTCAGCTAAGAGAGAAGC

CCCAGGCAGCAGGCACCATGGGAGGAAGTGGCCTGGGAAGTTGAAACAATTTAGGTGAGC

CAGGAGGGCGGAATCCTTGAAGCGGGCTGGCCCCAATAACAGGAGTTCGTTTTCGCCCCT

CGCCCAAACTTCCCTACCT

>2665 len=199 tot-seq=4

GGAGGACCCAGTTTGAATGTCATCATTAAAAGGCTCTGTAGATGAGCAGCAGGATGTGCG

GTCTGGAGGACCAGAGTGAAGAAGCGTGGTCAGAACTGATGGCAGATGATCACGGCAAGG

AAGGGGCAGAGGGCAACAGAGCCAAGTGGCATTTGCTGCACCCTAACTGGATATGTCGAG

AAAGGTGAAAAAGAAATGT

>2666 len=200 tot-seq=4

AGACCTGCAGCTCTCTCTGATGGAGCAGGGCAGGGGCAGGAGAGGGAGAGAAGAGCAGAA

CACATGTAATGACAACACTCATAAGTGTAAACCCACACTAGCGGCGCACAGTGCTCCTCA

AACAGTGCTACTACACCAGCAGACGACCACACACATCGTGGAACACTAGCAGCACACAGC

GCTCCTTAAACAGCGCTACA

>2667 len=200 tot-seq=4

CACTTACCGAGTGCCCCGTGAGGACTTGAGTGAGGCACTTCCCTTCTCCAGCTTATTTCA

CCATCTGTCGAATGAAGATGGGGAGAAGGGGGATGCCTACCTTGAAGGATAGTAGTGAGG

CTTAAATGAGGTAATAAGCAAAGTATCAGTGAGGTGCTGAGCGTTTTATATGGTCTCTGC

TGTTAGAGTGGCCCCATCTG

>2668 len=202 tot-seq=4

CGAGCTGGGTGTGCAGGTCTTCGGGCCCCTGAGTAGTGTATGTGAGTGCCAGCAATGGTG

GGCCTAGGCAGACCAGTCCTTGGGCCTCCAAGCAGCTTGCTTGGGTGCTGGCAGTAGTTG

GCCAGGTAGGCCTTGTGTTCCTGGATGGCATGCATGGCATTGGTGGTGGTACATGAGCTC

TGCTGAGTGGCATGCACAGGTG

>2669 len=203 tot-seq=4

TAGGGAATCTTCCACAATGGACGCAAGTCTGATGGAGCAACGCCGCGTGAGTGAAGAAGG

CCTTCGGGTTGTAAAGCACTTTCAGATGGGAAAAAGTAGTAATTCGAATAGGGTTGCTA

TGTGATATTACTATCAGAAGAAGCACCGGCTAACTCCGTGCCAGCAGCGCGGTAATCTAC

AGATCGGAAGAGCGTCGTGTAGG

>2670 len=203 tot-seq=4

GTGAATCACAGCCAGCAGGGACATACAAATGCCAATGCAGTGCTGTTTAGCCAAGTGAAA

GTGACTTCAGAGACACACATGCTACAGCAGCAGCAGCAGGCCCAGCAGCAGCAGCAGCAG

CACCCGGTTTTACACCTTCAGCCCCAGCAGATAATGCAGCTCCAGCAGCAGCAGCAGCAG

CAGATCTCTCAGCAACCTTACCC

>2671 len=203 tot-seq=4

GCTGGTGCATTTGGATGGGCAGGACCAAAGGGGCAAGCAAAGAGAGCCACCCTGGCACCA

CTTAACACTGCGGCCATTTGCCCACAGAGCTTCCCAGATATTGCTAACCCCTGGAGGAGG

CAGGAATCCTCCAGTGTCCCCCCGTGCAGCGTGCACACCCCAAAACGCTCAGGCTTGAAG

CTGCCGTCTAGCTCCTTGATAGC

>2672 len=204 tot-seq=4

TCAGGAGGTGGCTCAGCCCAATGTGCTTTCAGGAAGAAGAGTCCCCATATGGGGAGGTGG

CACTGAAGCGGGACCGCTCTTTTTCGGAACACGACCTGGCCCAGCTTCGGAGCGAGATGG

CATCTGGCCTTCAGTCAGCCGCTCAGCCCCCAGGAGGGACGGAGCCACCTCGGGCCAGAG

CAGGTAGCATGCACTCCTGGAGGC

>2673 len=205 tot-seq=4

GGCAATGGTGATCTAAGAGTCTTATTTACCATTTTAGGACCTAGCAGTATTGTATGGGCA

CCCAGTATTTGTTGAATAAATGAAACCCAACTCTCTGAGACTCTTCAGGCAGATTCTTAC

ACTCTCCTTGCTTCTAGCAAATGGAAAATTACCTTACTGACATCTGCAGCATACACACCA

GAGGAGCCCAGGAGTGGTGGCCAGA

>2674 len=205 tot-seq=4

TGGGGAATATTGGACAATGGAGGGAACTCTGATCCAGCAATTCTGTGTGTGTGAAGAAGG

TTTTAGGACTGTAAAGCTCTGTTGTTAAGGAAGAACGAGTGTGAGAATGGAAAGTTCATG

CTGTGACGGTACTTAACCAGAAAGGGACGGCTAACTACGTGCCAGCAGCCGCGGTATCCA

AAGATCGGAAGAGCGTCGTGTATGG

>2675 len=206 tot-seq=4

GGAACAATACTAACACCTCCCTCGTAGGTAAAAGTCTAATGAGATGAAGCAGGTGAAAGC

ACTTTATCAACTGGGGTTATAAACTAGGTACTCACAGCCTCCAGCAGCAAGCCACTCAGG

GAATCCAGGGAGATAAGGGGTAGGAGGCAGAAGGTGTCCAGGAGTCATGGGGTTTGGGAT

CAGGAACAAGCAGCTGACTCTCAGCT

>2676 len=206 tot-seq=4

TGCGCTCTCCCAACGGCGCGGATCCTCTTTGGAAATTAATATTAAAAAAAAAAAAGCCGA

GGACGCAGAGGGGAAGGTGGGGGGTAAGAGGGAAGGCGAGACACACACACACACACACAC

GCACACGCACACACGGACACACACACACGGAGAGAGAGAGAGAGAGAGACAGAGCCCCAC

AGTGAGAGGAAGAAAGGCAACAGTCG

>2677 len=206 tot-seq=4

CCTGGTCATTTTGTGGAGGCAACTTTTGAGTCCAAGAGGGCCTCTGCAAGCCTTTGGCCC

CAAAGCAGACCAGCACTGTCACCATAGCTCCAATAGAGGCCACATTTGCAGTGTCTGGGA

GAAATAAGATTGATCTATCCTCATCTCATTGGACAGGGCTCACTACCAGCTTCCAGCCCA

GTGGTGCTGCTTCTGCCTGAACTTGG

>2678 len=208 tot-seq=4

GCAGGTCAGATCTCTTTAACCAGAGTTTTGGAGACTGGAAGGAAACCGGAGAAGGAAGCG

TAGGGAAGACTTGGGGAACACAGGAAGGAGGCTAGTTTTAGACCTGGGTCAGAGGGAAGG

AACCCCTTCCCCCAGAACCCCAGATTCCGTCCACACCAGCAAATGAGAACCTTCCAGTCC

ATGGGACCCACCTTGTCATACATCCGAT

>2679 len=208 tot-seq=4

TCTACAATCATACTTCACAATTCTTTATGATAAAGAATAATTTTAATGGTAATTTTTTAT

CTAGCTTTGTCTATGTTTCTAACATGTTTATTTACATGGTTTACAATTCAAAAGATACAA

AAGGGTATATGGTGAAAAGTTCCCCCTGTTGTTTCTACTTGGGGAGTGAAAGGGAACGGG

GGAATTGTGACGAAACAACACTAGTCCC

>2680 len=209 tot-seq=4

CCACCGAGGTGGCCAGAGACGAGACGCTGCCAGCCCATTCTCCCGCAAAACGAAGACCCT

TCTTCCCCAAGGTCATTTGATCTGTTATTGCTCAGCTGCCATATCTGCCCCAGGGTCGCG

TCCTCCAAGGCGTGCCCTGCAGAGGCGGGCTGTTCTGAGGGGTGTGCCGGCAGCAGAGCC

TGGCCACAGCCAGGGCCCTGTCTCGCTCC

>2681 len=210 tot-seq=4

GAGGAGTGACTGGAGTTCAGAGTGTGCTCTTCCGATCTCCTACGGGAGGCAGTAGTAGGG

AATCTTCCACAATGGACGCAAGTCTGATGGAGCAACGCCGCGTGAGTGAAGAAGGTTTTC

GGGTTGTAAAGCTCTGTTGTAAGGGAAGAACGGTAGATAGAGAATATCTAAGTGACGGTA

CCTTACCAGAAAGCCACGGCTAACTACGTG

>2682 len=210 tot-seq=4

TAGGGAATCTTCCACAATGGGCGAAAGCCTGATGGAGCAACGCCGCGTGGGTGAAGAAGG

TTTTCGGATCGTAAAACCCTGTTGTTAGAGAAGAAAGTGCGTGAGAGTAACTGTTCACGT

TTCGACGGTATCTAACCAGAAAGGGACGGCTAACTACGTGCCAGCAGCCGCGTAATGTTT

AGATCGGAAGAGCGTCGTGTAGGGAAAGAG

>2683 len=211 tot-seq=4

TAGTCACACCAGACTCCCAGTGGAGGACTCACCTGCAAAGCTTTTGAACCCAATTTGCTC

CTGTCTGATAAAATGCAGGGACAAAAATGGAGCAGAGACTGAAGAAATGACCAGTGACTG

GCTCAACAGGCATCAATCTCTGACAGTATTACTGATTCTATGTTGTGCTTGCAGAAAGGA

GGCTTGCTTGGCTGTCCTCTGAAAGGCTCTA

>2684 len=211 tot-seq=4

GTGACTGGAGTTCAGACGGGGGCGCGGCAGAGCGCCGACGGGAGGCAGCAGGAGGGAAGC

GGCCACAAGGGACGCAAGGCGGAGGGAGCAACGCCGCGGGAGGGAAGAAGGGGGGCGGCG

CGGAAAGCTCTGTTGTTGGTGAAGAAGGACAGGGGTAGTAACTGACCTTTGTTTGACGGT

AATCAATTAGAAAGTCACGGCTAACTACGTG

>2685 len=213 tot-seq=4

CAGTGTGTCAAACCACGAGTGGGGAAAGGTGATGGGTAAGGCCACCATTTCTTGTAAACA

TTGCAGGCAATAGGCTGATGTCATAACAACCTTCAGTGTAGAACTGTGATTTCAAGAGGC

CTTGGTGGTTGTTTTGTTTTGTTAACAACCTTCTCCTCCCTCCCCCTTTCTCTCCCCACT

CCTCCTCTTTTTCTTCCTCTTCTCCCCACCTCC

>2686 len=213 tot-seq=4

GGCATGGGACTGTGCTGGGCTCATGAGCTGTGGGCCCCAAACCAACTGCAGTGGACTGAG

AGCCAAGGACGGGCCCGAGTCAGCCAGGATCAGGCACAGACCCCGCCACAGATTGGGGAG

GCTGGGTCTTTCCCAGTTGGTTCGGGGCCCATGGCTCATGAGCCCCACACAGCATTTGAA

AGCAGGCATCCGGTACTGAGGAAGGACATCGGG

>2687 len=214 tot-seq=4

CCCAGTGGTGTATCTGAGGATACATTTGAAGACACGTTGTTGCCCATATTCTGGAGGTTC

CTGGTGTTCGTACCAGCATTCAGAGAAGAGCTCAGAAACCAAAAAGGATATCAGCCAGGC

TAGCGTGCACAAGTCCTCACAAGGACCTCTAGTGGCTACCCAGTAAGCCCTGGACTTACT

GTGGAAAGTGTTCCACCCTGGCAAGGGATTTCAG

>2688 len=214 tot-seq=4

ATACGAGATACGGTAGTGACTGGAGTTCAGACGTGTGCTTTCCGATCTCCTATGGGAGGC

AGCAGTGGGGAATATTGGGCAATGGGCGAAACCCTGATGCAGCGACGCCGCGTGAAGGAA

GAAGTATCTCGGTATGTAAACTTCTATCAGCAGGGAAGAAAGTGGCTTCGGCCACGTGAC

GGTACCTGACTAAGAAGCCCCGGCTAATTACGTG

>2689 len=214 tot-seq=4

CGAGATCTCCAGGTGACTGGAGTTCAGACGTGTGCTCTTCCGACTACGGGAGGCAGCAGT

AGGGAATCTTCGGCAATGGGCGAAAGCCTGACCGAGCAACGCCGCGTGAGTGAGGAAGGC

CTTCGGGTTGTAAAACTCTGTTGTTTGGGAAGAAGGGAAAGGGTAGGACCCTTTGGTGAC

GGTACCAAACGAGAAAGCCCCGGCTAACTACGTG

>2690 len=216 tot-seq=4

TGGGGAATTTTGGACAATGGGGGAAACCCTGATCCAGCCATCCCGCGTGTATGATGAAGG

CCTTCGGGTTGTAAAGTACTTTTGGCAGAGAAGAAAAGGTACCTCCTAATACGAGGTACT

GCTGACGGTATCTGCAGAATAAGCACCGGCTAACTACGTGCCAGCAGCCCGCGGTAATGC

CGAGATCGGAAGAGCGTCGTGTAGGGAAAGAGTGGG

>2691 len=218 tot-seq=4

CTCTCTCCATGTGGAAGAAAACATCAGTAATGGGCAGAAAGCATTTTTCTCCAAAACATA

GTTGAAAGAGGTTGGAGAAGGGTGTGTGGAAATGGTCGTTATCTGAAAGGTGGGGATATG

AAAAGGGGGAGGGCAGGACTTGACTCCTTTGGCAGGCAAATGGTGAGAGGTAGTGCCAGC

TTTGAGACCACTGAGAGATTATGGTGTGTTGTCTGTGG

>2692 len=224 tot-seq=4

ACGCGTAGGTGCCCTCACCGGCCTTTGCCTCTGCTTGGGCCTGACCCTCGAGCTAGTAGG

CAAAGGGAAGGTCAGGAGGCAGAGGACAGCCACAGCCCCTCGCCTCTGTGTGGACAGAGA

AGGGCTGTGGCCCCTGCACGGCGAGCAGAAGGCACTGGGCTGGGGTCCTACCTTCGGGGG

ATAGTGGAGGTTGAGCAACTGGTAAAGGCGGAAGTTGACAAAGC

>2693 len=225 tot-seq=4

CCTGAGTCAGGGACTTATAGATAAAACTCCCATCTCCCTCGGACAGAGCACCTGGGGGAA

AGGGAGGCTATGAGCGCAGCTTCAGCAGACTTAAACATTCCTGCCTTCCAGCTCTGAAGA

GAGCAGCAGATCCCCCAGCACAACATTCAAGCTCTGCTAAGTGACAGACTACCTCCTCAA

GCAGGTCCCTGAACCCCATGCCTCCTGACTGGGAGACACCTCCCA

>2694 len=227 tot-seq=4

AGCAGAAGACGGCATACGAGATTCAGCGGTGACTGGAGTTCAGACGTGTGCTCTTCCGAC

GGGAGGCAGCAGTGAGGAATATTGGTCAATGGACGAGAGTCTGAACCAGCCAAGTAGCGT

GAAGGATGAAGGTTCTATGGATTGTAAACTTCTTTTATACGGGAATAAACGAATCCACGT

GTGGATTTTTGCATGTACCGTATGAATAAGGATCGGCTAACTCCGTG

>2695 len=229 tot-seq=4

AAGATAGAGCTCTCTGAGTTTGATGTCAACCTGGCCTACATACTTAGCTAGGGCTACACA

GTGAGACCCTGGTTCAAATCAAATGAACAAAATAACAACAATAACAACAGCACACACACA

TTCACATGTACATCTTGACACTATCACAACTTATGAATCACTCTAACTACTATCATAAAT

CCAATTTTTAATTTTGGTAAGCTAAAATTAGTGGTTAAAGATATTCAAA

>2696 len=229 tot-seq=4

CAAGCAGAAGACGGCATACGAGATCTGATCGTGACTGGAGTTCAGACGTGTGCTCTTCTA

CGGGAGGCAGCAGTGAGGAATATTGGTCAATGGGCGAGAGCCTGAACCAGCCAAGTCGCG

TGAAGGAAGACTGCCCGCAAGGGTTGTAAACTTCTTTTGTATGGGATTAAAGTCACCTAC

GTGTAGGTGTTTGCAGTTACCATACGAATAAGCATCGGCTAACTCCGTG

>2697 len=230 tot-seq=4

TAGTGGCAGGGCCAAGGGCCAAGAAGCAGCCCTGCTGGGATCTGAGGAGATGGTGGTGCT

GGGAAGCTGCAGGGCCAAGGGCTGCTGAGGTGTCCAGCGTGGTCCAGAGATTGGTTACTG

ACAGGAGGATTAAGCACTTACTGACAGAGAAGGGATTTATACACATAGAAAAAAAGAAGA

CAAGAAAGAACCCTCAGGTAGTAAGTTGGAATTGGTAGTATGTATTTTAT

>2698 len=230 tot-seq=4

AAAAGTCAGAAGACTTCAAGTCACTTTTCTCAGCATCTTTTGCTACACAAGTAAAGATAA

AATTCAAAGAATGTAGCTTTTTGTCTTATAAATTCTTTACGTGCAGATATTTTATTGAGA

ACATAGGAAAATGAGTGAATGTGATGTGCCAATATGGTGCTAGAAGCCAGCAGTGAACTT

TGGCAGTTCAGGAAAAAAGCTCCATCCTTCCAGGCAAGCTCTGGTATCTG

>2699 len=230 tot-seq=4

AATGCCTCCTGGAGTGGGTCCCTGACCCCCAAGTAGCCTAACTGGAAGGCACCCCCCAGT

AAGGGGAGATGGACACCTCACACAGCCGGGCACCCCTCTGAGACAAAACTTCGAGAGGAA

AAATCAGGCAGCAACATTTGCTGTTCACCAATATTCACTGTTCTACAGCATCTGCTGCTG

ATACCCAGGCAAAGAGGGTCTGGAGTGGACCTCCAGCAAACTCCAACAGA

>2700 len=231 tot-seq=4

ATCCTTGGGCATGTGAAATAGTCCCTTAATGCAGAAAGAGATCATATTACTCCTTTCTCA

AATTCCAGAAATGATTTAGGTCTCTGAATCAAAATATCTTACTTCATAGACTGGATAAGG

CCAAACCGAGCCAGCAGCAGGTCACAGTACAGCTCCAGGATCTCCATGGCCTCCACGAGG

TAGTCTTCCCGGATAATGTGCTCCACACGGATCCGAGCTCGTTCATCTTTC

>2701 len=239 tot-seq=4

TGCCACGTTTTTGGGAGAAGGTATATATTGCAGTAAAGGATCGTATGGATAGTTCTAACA

TCTTACAACGTAGGTTGTTTTATAAGGCTTTGCATATAGGTCGTAGTCGTAATGTCTTTT

ATAAGTCTAAGGGAAAGAGAGTTCCTTTAGGCTTGGAGCTTAAATATAGGTTCTATAATT

CCACTGTATTAAGTTTAGTTCGAAAGCAATTAGGACTTCTTAATCCAAATATTTTTCCA

>2702 len=242 tot-seq=4

TGAAGATGCTGAGAAGAGCTGCAGGATTTGAGGTATCTTACGAATGTGAGCCAAAAGCAT

TGCCTGAGGATTTACAAAAGATAAGAAGTGTCAAGGGAGAAGATACCATGTCAAGAATGA

CTCTGGGGTTCAGTCCTGTGGGGCTGCCTGTAATTCTCAATGGCAAGGTGGCCAATGCAG

TGCAATGAAAGATGCCCAACAGAGAATCACAGCCCATCGTAACCCGGCGCCAGCTCGCCT

CT

>2703 len=100 tot-seq=3

GGACTGCAATTGAGCATTCTCTCACCTTTCCCTTCTCAGAGCTCCCCAAGCCTATGCCCA

GGTGGGAAGAGGATTACCTGAGAGCCAGAAGCCCCGGGTC

>2704 len=100 tot-seq=3

CTAAAGTGGCAATGCTTAGACTCTTTCGTTTTGTTACAAAGGGGCGACCGCGTGGAGTTC

ATCAGTGTAGCTGCGGCAACCAAGATCTGCTTTGCATGAC

>2705 len=100 tot-seq=3

TGTCCTAGCAAAGGCATGAAAGACAAGAAATTAATCCAGGTGAAAAATTAGGAGAGTCAA

AAGCGTTTCATGCCAGGCATGGTGGCGCACACCTGTAATC

>2706 len=101 tot-seq=3

CCTCAGAAATGAAGCATGGTCAGAGCAAGGCCAGCCTCCTGCATCATGGTGGCTTCAAAG

TGCTTCAGTCTCTGAAGGGATCAGTGGGGAGGAGTTCAGCC

>2707 len=101 tot-seq=3

CGTGGATGGAGAGAGAGGAGGGATGCAGAGATGTTTAGGGCATGAGCTGGCCGGTGATGG

ATAGCATGGGGTAGGCTGGTGGTGGTGAGGGGGTCCAGGGT

>2708 len=102 tot-seq=3

CATTCTGACACTGTTCAACCAGCCCAGATTACCCTCTGATAGGCAGGCCACTGGTCAGGG

ATGAGGAGTGCCCCGCCCCTATCCTCACAGAGTGAGCTTGTC

>2709 len=102 tot-seq=3

TGAGGGGCACAAGCTGCCTCGACCACCAGAACAACGCCGCCAGCGTCCAGCAGCAACGAG

GGGCACAAGCTGCCTCGACCACCAGAACAACACCGCAAGCGT

>2710 len=102 tot-seq=3

GTGTGGCCCGGCTGGATGGGTCTATTTCTCCCCTGGCCGCCTATACAGGCTTGATGTGCC

TGGGTGCAGAGCCTCAGGAGTGCAGGCCAAGCCACTCCAGTC

>2711 len=104 tot-seq=3

ACTGGGGTTGGGGGTGGAGGAGAAAGACTCTTCGCGCTTTCCAAGCTGGAAGTTGTCAGG

AATCTTATTTTTAAGTCTTTGCCAAAGCAGCTCAAACCTAGTGT

>2712 len=104 tot-seq=3

GGAGAAACTCAGCCATAATATCTGCACAACAGCCCCTCCATGCAAACACCATTCCAGTAA

TGGGAAATTTTAATGATGTAAAGCAGCAAGGTCACACGATATGT

>2713 len=104 tot-seq=3

CAATGTTTCAAACGGCCAGGCGGCCCAGTACGGCACCACAGCCAGCCAATGTTCGGTTTC

CACTACGGTACGGCTGCCGTCAGCCAGTTCGCGTTGAACGTAGT

>2714 len=104 tot-seq=3

GGCTAGGGAAGGTAACAGGCAGGCATGTTTTAAGGCCATTAGCAAAGAACAATGGGAAGC

TGAAAGGTAATCCCAGTGAATAGAAGACACAAAGTGGGCTTGTG

>2715 len=105 tot-seq=3

ATTCAGTGTGGAGGGCAGTCCCCCCTGGTGGCTGGGTGAGCCTCTGGGCGAGCGGCCCTC

TCGGGAGGTGGTGCGGCCCCTGGTGGCCTTGAGCTGTGTTTCTCC

>2716 len=105 tot-seq=3

TGGGGAATATTGCGCAATGGGGGCAACCCTGATGCAGCGACGCCGCGTGAGGGATGAAGG

CCTTCGGGTCGTAAACCGCTTTTGATTGGGAGCAAGCATTTTGGG

>2717 len=105 tot-seq=3

AGGCCCGCCACACATATTTACTGAGTGAACAATTGTTCCTAAGTGCGTTAAAAAAGTACA

GTCACGATATCTTGTAGTTGTACATTGTTAACAGCAATAGGAAAG

>2718 len=105 tot-seq=3

ATCACCTGAGGTCAGGAGTTCGAGACTAGCCTGACCAACACGGAGAAACCCCATCTCTAC

TAAAAATACAAAATTAGTCTGGCATGGTGGCCCATTCCTCTAATC

>2719 len=106 tot-seq=3

GCACTGTTTGGGGCCACTGCCTTGGTTCCTGCTCAGCACCAGACTTTGAGGGCCATGGTT

TTATACTGCTAGTGTAGGCCTCAGCATGGTAAGAAACAGCAAGCAG

>2720 len=107 tot-seq=3

TAGGGAATCTTCCACAATGGACGCAAGCCTGATGCAGCAACGCCGCGTGAGGGAAGAAGG

GTTTCGGCTCGTAAAGCTCTGTTGGTGGGGAAGAAGGACAGGGGGAG

>2721 len=108 tot-seq=3

GGGTGGTAAAGAAACTTGCAACTCCTCAGGGTCAGCCATGGGGAAGGGTTGGGATGGCAG

AGCCAACCAGAGCAGCCAAACCAACACTAACTCTCTTTCAACTACTTG

>2722 len=108 tot-seq=3

CATGACAGTTTATAAGTGCCATAGCAACATTCAGGTCTAAAAAGGGGAGGCATGAATAAT

CCACCCCTTGCTTAGCACATAATCAAGAAAAAAACATACAAATGGCAA

>2723 len=109 tot-seq=3

GATGTCATAGTCTAGGTCTATACCAGGCATTTTAAGCTGGTGACCTGGGTACAGATGTGT

AAGAAATGTGCAAGAAGTGCTTTCACCAGTTTTCTGAGAATCAAACCTG

>2724 len=109 tot-seq=3

GAGCGGAGACAGCAGGCACTTAGCATGGCCCCAGGGGCAGCCGACGCCCAAATCGGAACT

GCAGACCCCGGGGACTTCGATCAGTTGACTCAGTGCCTCATCCAGGCCC

>2725 len=110 tot-seq=3

TCCTCGGAGGCCTCTCTCATGGACCCTCTCCTGTATTCTCTCCAGGCTTGGGTTCAAGAG

CGGCTGCCACTGGCCATGCAGACAGAGCGAGGCACTGGCTTGCAGGCTGT

>2726 len=110 tot-seq=3

TAGGGAATCTTCCACAATGGACGCAAGCCTGATGGAGCAACGTCGCGTGAGTGAAGAAGG

GCTTCGGCTCGTAAAGCTCTGTTGTTGGGGAAGAAGGACAGGGGTAGGAA

>2727 len=110 tot-seq=3

CAGTCATCGCCCACTCTGTCCCCGCTGTCATACATCACTCAGGCCGTGGCCTTGAATGCC

CTGTCTCTGGAGCAGCAGCTACCCTACGCCTTCTTCACCCAGACGGACTC

>2728 len=110 tot-seq=3

CAATGCAAAGCCCCAGGCACTGCCCAGAGGAGGCAGCATTAGGTGGGGCAGCAGGGTAGG

GAGCAGAGGAGCATGAAGAACTTTAGCTGATGTGGGTTAAGAGAGCTAGG

>2729 len=110 tot-seq=3

GTGAAGAAGGTCTTCGGATCGTAAAGCTCTGTTGTTGGTGAAGAAGGATAGAGGGAGTAA

CTGTCCTTTATTTGACGGTAATCAACCAGAAAGGCACGGCTAGCTACGTG

>2730 len=111 tot-seq=3

ATCAGGATCAAGTTTTAGACATCTTTATACAGCACTGCTGAAGAAAGCCATGCTTGATTC

GTGATTTTAACCGTCCTCTCCAATACTTAAAGGAAGGAGATCACCCCCCCC

>2731 len=111 tot-seq=3

AGCAAGTGCAGGAGGAACAACAAAGGCCTAAGTGAAAATCTGGCTGTGCATGACAGCTCC

GCATTAACCTTCAGAGAAGCTCTCCTGGGAGGTAACCTTGGCAGACTCCAG

>2732 len=113 tot-seq=3

TGGGGAATATTCCGCAATGGGCGAAACCCTGATGCAGCGACGCCGCGTGCGGGATGAAGG

CCTTCGGGTCGTAAACCGCTTTTGATTGGGAGCAAGCTTTCGGGTGAGTGTAC

>2733 len=113 tot-seq=3

ACACAAGAACCACTTTTCCTATGTTTCCTCATGCCTGCAACCTCATCTGTTCCTACATCT

ATCTTTATGGCCCCCAAGTCTAGCTTAGACATAGTCTTTTCCACAATAGGGTC

>2734 len=113 tot-seq=3

ACAAGACATGTCTGAGGGTACTGGGGTGGGGGGATACACAATGATGTCACACACAGGAAA

TATCAGAGGGGGACTTGGCACTTCACAAGGCTGGCTCCTCTTCCATAGGCTCA

>2735 len=114 tot-seq=3

CCAGCTGTGGGTAAGGAATCCAGCAGTGGGTAAGGAAGCCAGCTGTGGCTAAGGAAGCCA

GCTATGGGTAAGGAAGCCAGCTGTGGGTAAGGAAGTCAGCAGTGGATAAGGAAG

>2736 len=114 tot-seq=3

AGGGCCAGGGGCAGCCTTCATTTTACATCCTTGATCTTGGATAATTTAAATAATAGAGCT

GCTTTTCTGTATACACTAGTGGTGTGAAAAGGAAGTTATCCACCTAGGACATAT

>2737 len=115 tot-seq=3

TTTCACCAGCACAAAGAGACTGAAAGTCCGGCACATTTTCAATATGTGACTAGAATAATC

CACAGCGGCACCCCGGCACAAAAGCAGGGTGACCGTGTCACAGCACCCTGGGCCA

>2738 len=115 tot-seq=3

CGTGAGTGAAGAAGGGTTTCGGCTCGTAAAGCTCTGTTGTTGGTGAAGAAGGTCAGGGGT

AGTAACTGATCTTTGTGTGACGGTAATCAATTAGAAAGTCGCGGCTAACTACGTG

>2739 len=115 tot-seq=3

TAGGGAATCTTCCACAATGGGCGCAACCCTGATGGAGCAACGCCGCGTGCGGGAAGAAGG

TTTTCGGATCGTAAAGCTCTGTTGTTGGGGAAGAAGGATAGAGGTAGTAACTGGC

>2740 len=115 tot-seq=3

CGTGAGTGAAGAAGGGTTTCGGATCGTAAAGCTCTGTTGTTGGTGAAGAAGGATAGAGGG

AGTAACTGGCCTCTAATTGACGGTAACCAACCAGAAAGTCACGGCTAACTACGTG

>2741 len=116 tot-seq=3

GAAGAAGGACAGGGGTAGTAACTGACCTTTGTTTGACGGTAATCCATTAGAAAGTCACGG

CTAACTACGTGCCAGCAGCCGCGGTATCCATAGATCGGAAGAGCGTCGTGTAGGGA

>2742 len=116 tot-seq=3

CCCTGACGCAGCGACGCCGCGTGCGGGATGAAGGACTTAGGGTTGTAAGAAGATTTTGAT

TGGGAGAAAGATTTCGGGTGAGTGTACCTTTCGAATAAGCGCCGGCTAACTACGTG

>2743 len=117 tot-seq=3

GAGAGAAGTCAGCTCCCTTTTCCCTGCCTGCTCTTTTCCCCCACTTGCCAGGGTCCCTGA

TGGGGGGAAGGGAAGAGGGAGGAAGGAAAGGGAGAGGAGCGAAGGGCAGGCAGCCAC

>2744 len=118 tot-seq=3

AAGGAGGTGAAAGAAAAGCCCAGGATTGCAGGGAGACAATACCCATATTGAGGGTGAAGG

GCAAGTCCTCTGGGATTATCAAAGCAGAAGGTGTTGTTGGCATCCACACAGTGCTGGG

>2745 len=119 tot-seq=3

GAGCCTGTTCAGGGAGGTTAATTCTAATCTCCCACAACCTGGTGAGGAATGGATGGACGT

GTTCCCACTTATAGATGAGGATTCAGGGGCAAATGAAACCCCCCTGCAATGAGACCCTG

>2746 len=119 tot-seq=3

CGACCACCAGACCCTCAGCCCAGTGATCCCCCACATCCCTTTAATACATCAGATCCTAAA

CATGGCAGTGTTGGGTCCTCCATCAAGGATGAGGCCTCACTCCCTGACTGCTCCAGCCC

>2747 len=119 tot-seq=3

AGCAGGTGCTCGCGCCTGCCCGGAAACCGACAGCGCCGTCCAGTACAGGAAACCGCACCG

CACCGCCAGCAGCCGCAAGGACGGCAGTGCCGTCCAGCGCAGGAAATCGCACCATGCAG

>2748 len=119 tot-seq=3

GAAGGACAGGGAGCAAAGGCAAAGCCTGTACAACATCTGGCACGCTGTGCCTCCTCAGCC

AGCAAGGCCGCCACGGTCACGCGGCAGATGCCTGTGGGCTGTCTTCCCATGGACGACCA

>2749 len=120 tot-seq=3

ACCTGTGGGTAAAGAAGCCGTCTCAGACATTCCACTGCAGCTGAGCATCCAGATGACCAG

TCCCTGACACTGTTTAACCACACAGTGAGAGCTGCCAAATGAGACCAGCAGAAAAACTGT

>2750 len=120 tot-seq=3

GGAACGAGCAATATCAACACATATTTGGAAGATAAAAAGCTGACCGAGGAGTTAGACTCT

TGAGAAAACTGAGTGTCAAGTGCCTAGAGACACAAGTCAATTCAAACTCCAGAATTACAG

>2751 len=120 tot-seq=3

AATCACTTGAACCCAGAAGGCGGAGGTTGCAGTAAGTCAAGATCGCGCCATTGCACTCCA

GCCTGGGTGACAGAGTGAGACTGTTTCAAAAAAAAAAAGGAACACCAAGAAAATTCCTCA

>2752 len=122 tot-seq=3

GACTCCGCGGAAAGGAACACGACCTCGGAGGAGGACCCGGGTGCAGAGCAAGTGCTCCTG

CACCTGCCCTTCACCATGGTGACCGAGCAGCGGCGCCTGGACGACATCCTGGGGAACCTC

TC

>2753 len=122 tot-seq=3

CTAGGCAGAGAGGGTGAGGGCCGATGGTGACCTGGAAGGGGCAGCGAGGGAGCGGGACTG

CATGAGGATTCTGCGCGTGGGTGAGCCCAACTGTCATTTTCACATTTTGTAGAAAGAGCT

CT

>2754 len=122 tot-seq=3

GCAGAGGCTGGGTCAACACCAGCTTCTATTTGCAGAGTGGACTTCTAGGCACCCCCATTG

GAAGGTGGATGTTCTGGGCCTGGCAGTCTTGCAGTCCAGGATGCAGGAAGCAGGGCCGAC

AG

>2755 len=124 tot-seq=3

TGGGGAATATTGCACAATGGGCGAAAGCCTGATGCAGCGACGCCGCGTGAGTTCGGTATG

TAAAGCTCTATCAGAAGGGAAGAAAATGACGGTACCTTACTAAGAAGCTCCGGCTAAATA

CGTG

>2756 len=124 tot-seq=3

AGAACAGAATCCAGGTGCCCAGATTCTCATCTGCACATCTGCCAATGGGTGAGGGAGGGC

CACAGACTGGAGGGAAGGCAGAAGAGAACGTCTCCATGATTTCAACCAGCCGGTCCCCAT

CTCT

>2757 len=125 tot-seq=3

TCCCTCTTATCGGGTTTGTTCAGCTTTTGTTTCAAACTTAAAACCATTTTTCAGGAACAA

TTATCTCCCTAAATTCTCTATCTGCCTCTCAGATCAGCTGCAGCCAGGCAGCTGCAACCA

GATAG

>2758 len=126 tot-seq=3

AGGCAGTGCTAAAAGTAAAGTTTATAGCCCTAAACGCTTGCATCAAAAAGTCTGAAAGAG

CACAAACTGACAATCTAAGGTCATACCTTAAGGAACTAGAGAAACAAAAATAAAACAAAC

CAAAAC

>2759 len=126 tot-seq=3

GCGCGGAACTTACCCACTCGAGACTTTCGGAGGTGGTAATGACCAGTTACATGTCAACCT

TACGGTTGTTTGTATAGGATCGCACTTTTCCAGTGCGGTGCAATCTAGCAGAGGGCCAGT

CTGGTG

>2760 len=130 tot-seq=3

CTCTGAAGAGGCCATCACTGGCCTGGCCTCAGGGGATGTAGCCTTTCCCCCCTGAACCCC

TGCTGGGCAGGCAGCATTCTGCAGCAGCCTCCAAGATGGGCCTCCTGCATTCTCCTAGGC

CAGACTCCTC

>2761 len=130 tot-seq=3

AAAACAGGGCAGAGTCGTGGACCAGATCATAGTCCAAAACTCTGGGCCTCAGTTTCTCTG

ACTTAAAGATGAGGAGGGATTAGGATTCCTGTGGCTTGCCCTCTGGAAATCCCAAGGACC

CACCCCGGCC

>2762 len=130 tot-seq=3

GAGAAGGAGGGCAAACCCAGCTACAGAGCTACTCCCTCGACCGTGGAAAGGGAGGGAGAA

GGAGGACCTCCCTTTGGAAGGAAATCCATTGCAATAGCCCCAGTCGCCGGCTTTGGTCCT

GGCTCCACAT

>2763 len=131 tot-seq=3

GAAGGAACAGCCAACTAATGACTGGCCCAACTTGAAAGAGCCATGTGAGAGAGCCCACCC

AGCACTATTAATGATACTGTGCTATGCGTATAGACAGGGACCTAGCATAAGTGTCTCTTG

AGAAGCTTCAG

>2764 len=131 tot-seq=3

TGAGGAATATTTCACAATGAGCGAAAGCTTGATGGAGCAATGCCGCGTGCAGGATGAAGG

CCCTCGGGTTGTAAACTGCTTTTATAAGAGAAGATTATGACGGTAACTTATGAATAAGGG

ACGGCTAACTA

>2765 len=131 tot-seq=3

CTTTCATCAGAGGACTGAGTGGTTCCCATTTAGCCTGCTATTCCTGCTGCCCTCAGACTG

CCCAGGGAGATCCTAACACACCTTCCAGCACATTCCTGTCCTGCCTTCTATTGGAACTCT

CCCTTATTTCT

>2766 len=131 tot-seq=3

TCTTCCATTAGCACCACTGAGATTCTAGGGTAGACTGGGGCAAAAGTTGAGCAAGTGCCA

GCTGCCCAGCTGTGGCTGAATATAGGACTCAGGGCAGCCTCCACAAAACAAATAATGAAC
AAGCAAGCTAG
>2767 len=132 tot-seq=3
CAATGGAAGTGGTGAGATGAGATCAAATTCTGTATTTTCTGGGAAAGTGGAGTCATGGTT
TCCTGCCAGATTCAATGGGGAGTAGAGAGAGAAAGAGAGGCACAAAGATGACACCAGGCA
GGAATGGAGTTG
>2768 len=132 tot-seq=3
ACCCAAAGGTAGGGGCAGGGAAAGGAGGGGCCAAGACTTTTTCAGATACTCGGTTTTTTC
TAAGGCCTTGACCTCAGCAGCTGAACTCATGGTCATGGCTGCCTTGGCCTTATTGTCCAC
TGTAGCCTCCTC
>2769 len=132 tot-seq=3
AATCGCTTGAACCTAGGAGGCAGAGGCTGCAGTGAGCCGAGATTGCACCACTACACTCCA
GCCTGGATAACAGAGTGAGACTCCACCTAAAACAAAAAAGAAAAAAAGAAAAAAAAGCCT
TCTCCTGTACCC
>2770 len=133 tot-seq=3
GACAGAGATGATCAGATTTACATTTTAGACAGCACTCTTCATCTGCAGCATGCAAATATA
GGAAAGGCCAATTTCAGAGGCAGGAGGACAGTGGGAAGCACGTCATCATGGGCCAGGTGA
GGGGTGTTGATGC
>2771 len=133 tot-seq=3
GGGAAGAATTCACAAAGGTACAATATCTGAGTTGGTACACAATCAGTTCATGGTTACTGA
TGGATAAATATCTTGCATGGCCTTCTGCAAAAACCTGGGCTGACATCTTTTCCTCCAGTG
ACTGCCTTCCTCC
>2772 len=133 tot-seq=3
AGTGCCCTGAGTCGGAGGAGCCTGGGGAGGGGGAGAGGGAGCTTTGGCTCCGGATTGGAG
AGAGTCATGGATGTCCTCCCTTCCTGAGGGGACATGGAGAACGGGCAGCTACATGGAGAA
TGGGCAGCTGAGC
>2773 len=133 tot-seq=3
GTGGACCCCAGCTTTGGTAGACTTGACTAATGGACTACCTGTCTACTCTACCCTTGGGGC
TCTGCCCTGAGAAATGACTTTGGCAGCTGAGCTCAGAGAGGGAGGCCAGACGCAATGGTT

CATGCATGTAATC

>2774 len=133 tot-seq=3

ATTAAGTCATAGTAAGGGACAATATTCAGGTTTAGGTGTCAACGTTATTTGTCTCAACAT

GTAGATGTCCTAACTGAAGCATGGGAAGGCATAGGCTATGCTTACCCCCAAGCAGACCAT

GCCCAGGGCCAAG

>2775 len=134 tot-seq=3

GTGTACAGATGAAAATAGTCTGTAAGAATATCGTAGGCCAGTTAGCGCCCCATAAGTGTT

CATTTGTTTGGGGTTATAAAAAGCATCAGCAAGGATTTTCCAGGAAAAAGAAATGCAGAT

CTTTCTGCTTCCTG

>2776 len=134 tot-seq=3

TAGGGAATCTTCGGCAATGGGGGGAACCCTGACCGAGCAACGCCGCGTGATTTAGAAGGC

CTTCGAGTTGTAAAAATCTTTTGTATGGGAAGAAAATGACAGTACCATACGAATAAGGAC

CGGCTAATTACGTG

>2777 len=134 tot-seq=3

TAGGGAATCTTCCACAATGGGGGAAACCCTGATGCAGCGACGCCGCGTGATTTAGAAGGC

CTTCGGGTTGTAAAAATCTTTTGTATAGGAAGAAAATGACAGTACTATACGAATAAGGAC

CGGCTAATTACGTG

>2778 len=134 tot-seq=3

TGGGGAAATTTGCACAATGGGGGAAACCCTGATGCAGCGACGCCGCGTGATTTAGAAGGC

CTTCGGGTTGTAAAAATCTTTTGTATAGGAAGAGTTTGACAGTACTATACGAATAAGGTC

CGGCTAAATACGTG

>2779 len=134 tot-seq=3

TAGGGAATATTGCTCAATGGGGGAAACCCTGAAGCAGCAACGCGCGTGGAGGATGAAGGT

TTTCGGATTGTAAACTCCTTTTATTAGAGAAGATAATGACGGTATCTAACGAATAAGCAC

CGGCTAACTCCGTG

>2780 len=135 tot-seq=3

TAGGGAATCTTCCGCAATGGGCGAAAGCCTGACGGAGCAACGCCGCGTGAGTGAAGAAGT

ATTTCGGTATGTAAAGCTCTATCAGCAGGGAAGATAATGACGGTACCTGACTAAGAAGCT

CCGGCTAAATACGTG

>2781 len=135 tot-seq=3

TGGGGAATTTTGGACAATGGGCGCAAGCCTGATCCAGCAATGCCGCGTGAGTGAAGAAGT

ATCTCGGTATGTAAAGCTCTATCAGCAGGGAAGAAAATGACGGTACCTGACTAAGAAGCC

CCGGCTAACTACGTG

>2782 len=135 tot-seq=3

TAGGAAATCTTCCACAATGGACGCAAGTCTGATGGAGCAACGCCGCGTGAGTGAAGAAGG

CTTTCGAGTCGTAAAGCCCTGTCCTATGAGAAGATAATGACGGTATCATAGGAGGAAGCC

CTGGCTAAATACGTG

>2783 len=135 tot-seq=3

GCGCGCAAATTACCCAATTCCGATACGGAGAGGTAGTGACAATAAATACTGATACAGGGC

TCTTTTGGGTCTTGTAATTGGAATGAGTACAATTTAAACCTCTTAACGAGGAACAATTGG

AGGGCAAGTCTGGTG

>2784 len=135 tot-seq=3

TGAGGAATATTGGGCAATGGGCGCAAGCCTGACCCAGCAACGCCGCGTGAAGGAAGACGG

TTTTCGGATTGTAAACTTCTTTTAAGAGGGACGATAATGACGGTACCTCTTGAATAAGCC

ACGGCTAACTACGTG

>2785 len=135 tot-seq=3

TAGGGAATTTTCCACAATGGGCGAAAGCCTGATGGAGCAACGCCGCGTGCAGGATGAAGG

CCTTAGGGTTGTAAACTGCTTTTATCTGTGACGAATATGACGGTAGCAGATGAATAAGGA

TCGGCTAACTCCGTG

>2786 len=135 tot-seq=3

TAGGGAATCTTCCACAATGGGCGCAAGCCTGATGGAGCAACACCGCGTGAGTGAAGAAGG

ATTTCGGTATGTAAAGCTCTATCAGCAGGGAAGAAAATGACGGTACCTGACTAAGAAGCC

CCGGCTAACTACGTG

>2787 len=135 tot-seq=3

TAGGGAATCTTCGGCAATGGGGGCAACCCTGATGCAGCGACGCCGCGTGGAGGAAGAAGG

TCTTCGGATTGTAAACTCCTGTTGTTGAGGAAGATAATGACGGTACTCAACAAGGAAGTG

ACGGCTAACTACGTG

>2788 len=135 tot-seq=3

TGGGGGATATTGCACAATGGGGGGAACCCTGATGCAGCGACGCCGCGTGGGTGAAGAAGC

GCTCCGGCGCGTAAAGCCCTGTCAGCAGGGAAGAAGGTGACGGTACCTGACCAAGAAGCC

CCGGCTAACTACGTG

>2789 len=135 tot-seq=3

TGGGGAATATTGCACAATGGGGGAAACCCTGATGCAGCGACGCCGCGTGAGCGAAGAAGG

CTTTCGAGTCGTAAAGCTCTGTCCTATGAGAAGATAATGACGGTACCTACAGAAGAAGCA

ACGGCTAAATACGTG

>2790 len=135 tot-seq=3

TGGGGAATATTGCACAATGGGGGAAACCCTGATGCAGCGACGCCGCGTGAGTGAAGAAGT

ATCTCGGTATGTAAAGCTCTATCAGCAGGAAAGATAACGACGGTACCTGACCAAGAAGCC

CCGGCTAACTACGTG

>2791 len=135 tot-seq=3

TGGGGGATATTGCACAATGGGGGAAACCCTGATGCAGCGACGCCGCGTGAGGGAAGAAGG

TTTTCGGATTGTAAACCTCTGTCCTCAGTGACGATAATGACGGTAGCTGAGGAGGAAGCC

ACGGCTAACTACGTG

>2792 len=135 tot-seq=3

TGGAGAATATTGCACAATGGGGGAAACCCTGATGCAGCAACGCCGCGTGGGTGAAGAAGT

ATTTCGGTATGTAAAGCCCTATCAGCAGGGAAGAGAATGACGGTACCTGACTAAGAAGCC

CCGGCTAACTACGTG

>2793 len=135 tot-seq=3

TTAGGAATTTTCCACAATGGGCGCAAGCCTGATGGAGCAACGCCGCGTGCAGGATGAAGG

CCTTAGGGTTGTAAACTGCTTTTATACGTGAAGAATATGACGGTAGCGTATGAATAAGGA

TCGGCTAACTCCGTG

>2794 len=135 tot-seq=3

TGGGGAATATTGCACAATGGGGGGAACCCTGATGCAGCGACGCCGCGTGGGCGATGGAGT

ATTTCGGTATGTAAAGCCCTATCGGCAGGGAAGAAAATGACAGTACCTGACTAAGAAGCC

CCGGCTAACTACGTG

>2795 len=135 tot-seq=3

TGGGGAATATTGCACAATGGGGGAAACCCTGATGCAGCGACGCCGCGTGAACGAAGAAGG

TATTCGTATCGTAAAGTTCTGTCCTACGGGAAGATAATGACAGTACCATAGAAGAAAGCC

ACGGCTAACTACGTG

>2796 len=135 tot-seq=3

TAGGGAATCTTCGGCAATGGGCGCAAGCCTGATCCAGCCATGCCGCGTGAGTGATGAAGG

CCTTAGGGTTGTAAAGCTCTTTCACCGGAGAAGATAATGACGGTATCCGGAGAAGAAGCC

CCGGCTAACTTCGTG

>2797 len=135 tot-seq=3

TGGGGAATATTGCACAGTGGGGGAAACCCTGATGCAGCGACGCCGCGTGGGTGAAGAAAT

GTTTCGGCATGTAAAGCCCTATCAGCAGGGAAGAAAATGACGGTACCTGACTAAGAAGCC

CCGGCTAACTACGTG

>2798 len=135 tot-seq=3

TGGGGAATATTGCACAATGGGGGGAACCCTGATGCAGCGACGCCGTGTGGGTGAAGGAGT

ACTTCGGTACGTAAAGCCCTATCAGCAGGAAAGAAAATGACGGTACCTGACTAAGAAGCC

CCGGCTAACTACGTG

>2799 len=135 tot-seq=3

TAGGGAATCTTCCACAATGGACGCAACTCTGATGCAGCGACGCCGCGTGAACGAAGAAGG

CTTTCGAGTCGTAAAGTTCTTTTATATGGGAAGATAATGACGGTACCATAAGAAAAAGCC

CCGGCTAACTACGTG

>2800 len=135 tot-seq=3

TGGGGAATATTGCACAATGGGCGCAAGCCTGATGCAGCAACGCCGCGTGAACGATGAAGG

TCTTCGGATCGTAAAGTTCTGTTGCAGGGGAAGATAATGACAGTACCATAGAAGAAAGCT

CCGGCTAAATACGTG

>2801 len=135 tot-seq=3

TGGGGGATATTGCACAATGGGGGGAACCCTGATGCAGCAACGCCGCGTGAGGGAAGAAGG

TTTTCGGATTGTAAACCTCTGTCCTTGGTGAAGATAATGACGGTAGCCGAGGAGGAAGCT

CCGGCTAACTACGTG

>2802 len=135 tot-seq=3

TGGGGAATATTGGACAATGGGGGCAACCCTGATCCAGCAATGCCGCGTGAGTGATGAAGG

CCTTAGGGTTGTAAAGCTCTTTTACCAGGGACGATGATGACGGTACCTGGAGAATAAGCC

CCGGCTAACTTCGTG

>2803 len=135 tot-seq=3

TGGGGAATATTGCACAATGGGGGAAACCCTGATGCAGCGACGCCGCGTGAGCGAAGAAGG

CTTTCGAGTCGTAAAGCTCTGTCCTATGAGAAGATAATGACGGTATCATACGAATAAGGA

CCGGCTAATTCCGTG

>2804 len=135 tot-seq=3

TGGGGAATATTGCACAATGGGCGCAAGCCTGATGCAGCAACGCCGCGTGAACGATGAAGG

TCTTCGGATCGTAAAGTTCTGTTGCAGGGGAAGATAATGACGGTATCATAGGAGGAAGCC

CTGGCTAAATACGTG

>2805 len=135 tot-seq=3

TGGGGAATATTGCGCAATGGGGGAAACCCTGACGCAGCGACGCCGCGTGAGTGAAGAAGT

ATTTCGGTATGTAAAGCTCTATCAGTAGGGAAGATAATGACGGTACCTACATAAGAAGCC

CCGGCTAAATACGTG

>2806 len=135 tot-seq=3

CTGGAGCAGAAGTGAGCAGAGAGCAGCTTTGGCTTCCTGGGCCCTGGCTGGGAGAGCGGG

GCTGCCTGGGTAAAGGAGCCCCCTCCTCCAACTCAGAGGCCCCTGGATCCTGACCCTGGC

CGGCCAGCGGCCTGA

>2807 len=135 tot-seq=3

TGGGGAATCTTGCGCAATGGGGGGAACCCTGATGCAGCAACGCCGCGTGAGCGATGAAGG

CCTTCGGGTCGTAAAGCTCTGTCCTCAAGGAAGATAATGACGGTACTTGAGGAGGAAGCC

CCGGCTAACTACGTG

>2808 len=135 tot-seq=3

TGGGGAATTTCGCACAATGGGCGCAAGCCTGATGCAGCGACGCCGCGTGAGCGATGAAGG

TCTTCGGATTGTAAAGCTCTGTCCTGGGTGAAGATAATGACGGTAATCCAGGAGGAAGCC

CCGGCTAACTACGTG

>2809 len=135 tot-seq=3

TGGGGAATTTTGCGCAATGGGGGAAACCCTGACGCAGCAACGCCGCGTGCGGGACGAAGG

CCTTCGGGCTGTAAACCGCTTTCAGCAGGGAAGACTTTGACGGTACCTGCAGAAGAAGCT

CCGGCTAACTACGTG

>2810 len=135 tot-seq=3

TGGGGAATATTGGACAATGGGCGAAAGCCTGATCCAGCGACGCCGCGTGAAGGATGAAGT

ATTTCGGTATGTAAACTTCTATCAGCAGGGAAGAAGATGACGGTACCTGACTAAGAAGCC

CCGGCTAACTACGTG

>2811 len=135 tot-seq=3

AGGCGGAGGGAGCAACGCCGCGGGAGGGAAGAAGGGGGGCGGAGCGGAAAGCTCTGGGGT

TGGTGAAGAAGGATAGAGGTAGTAACTGGCCTTTATTTGACGGTAATCAACCAGAAAGTC

ACGGCTAACTACGTG

>2812 len=135 tot-seq=3

TGGGGAATATTGGACAATGGGCGAAAGCCTGATCCAGCAATACCGCGTGAATGATGAAGG

CCTTAGGGTTGTAAAGTTCTTTTAGTTGGAAAGATAATGACGGTACCAACAGAAAAAGCC

CCGGCTAACTCCGTG

>2813 len=135 tot-seq=3

TGGGGAATTTTGCGCAATGGGGGGAACCCTGACGCAGCAACGCCGCGTGCGGGATGAAGG

CCTTCAGGTTGTAAACCGCTTTCAGCAGGGAAGATAGTGACGGTACCTGCAGAAGAAGCC

CCGGCTAACTACGTG

>2814 len=135 tot-seq=3

TGGGGAATTTTGCACAATGGGGGAAACCCTGATGCAGCGACGCCGCGTGAATGATGAAGG

TCTTCGGATTGTAAAATTCTGTCCTTAGCGAAGGTAATGACAGTAGCTAAGAAGCAAGCC

CTGGCTAAATACGTG

>2815 len=135 tot-seq=3

TGAGGAATATTGGTCAATGGGGGAAACCCTGATGCAGCGACGCCGCGTGAGCGAAGAAGG

TTTTCGAATCGTAAAGCTCTGTCCTATGGGAAGATAATGACGGTACCATAGGAGGAAGCC

CCGGCTAACTACGTG

>2816 len=135 tot-seq=3

TGGGGAATATTGCACAATGGGCGAAAGCCTGATGCAGCGACGCCGCGTGAAGGATGAGGT

ATTTCGGTATGTAAACTTCTATCAGCAGGGAAGAAAATGACGGTACCTTACTAAGAAGCT

CCGGCTAAATACGTG

>2817 len=135 tot-seq=3

TGGGGAATTTTGCACAATGGAGGAAACTCTGATGCAGCGACGCCGCGTGAGCGAAGAAGT

ATTTCGGTATGTAAAGCTCTATCAGAAGGGAAGAAAATGACGGTACCTTACTAAGAAGCT

CCGGCTAAATACGTG

>2818 len=135 tot-seq=3

TGGGGAATACTGCACAATGGGGGAAACCCTGATGCAGCGACGCCGCGTGAGCGAAGAAGT

ACCTCGGTATGTAAAGCTCTATCAGCAGGGAAGAAAGTGACAGTACCTGACTAAGAAGCC

CCGGCTAACTACGTG

>2819 len=135 tot-seq=3

TGGGGAATATTGCGCAATGGGGGAAACCCTGACGCAGCCATGCCGCGTGAGTGATGAAGG

CCTTAGGGTTGTAAAGCTCTTTCACCGGAGAAGATAATGACGGTATCCGGAGAAGAAGCC

CCGGCTAACTTCGTG

>2820 len=135 tot-seq=3

TGGGGAATATTGCACAATGGGGGAAACCCTGATGCAGCGACGCCGCGTGGGTGAAGAAGT

ACTTCGGTACGTAAAGCCCTATCAGCAGGGAAGAAGATGACGGTACCTGATTAAGAAGCC

CCGGCTAACTACGTG

>2821 len=135 tot-seq=3

TGAGGAATATTGGGCAATGGAGGCAACTCTGACCCAGCCATGCCGCGTGAGTGAAGAAGG

TTTTCGGATTGTAAAGCTCTTTCGCACGGGACGATGATGACGGTACCGTGATAAGAAGCC

CCGGCTAACTTCGTG

>2822 len=135 tot-seq=3

TGAGGAATATTGGGCAATGGAGGAAACTCTGACCCAGCCATGCCGCGTGAGTGAAGAAGG

TTTTCGAATTGTAAAGCTCTTTCGGATGTGAAGATGATGACGGTAACATCTAAAGAAGCC

CCGGCAAACTTCGTG

>2823 len=136 tot-seq=3

CCTTCGTATCCCCAGGTCCTTTCACATTCCCTCAGAGAGATGACTAATGCCAAGAGTGTG

AGACCCTCAGTGGCAAAGGTGAGTTGGTGGCGGCTCCTTTCACATGGATCGGAGCCTGGA

GGAAACCACTTCCTTC

>2824 len=136 tot-seq=3

TGGGGAATCTTGCGCAATGGGGGCAACCCTGACGCAGCGACGCCGCGTGCGGGATGAAGG

CTCTAGGGTCGTAAACCGCTTTCAGCAGGGAAGAGACAAGACTGTACCTGCAGAAGAAGC

CCCGGCTAACTACGTG

>2825 len=136 tot-seq=3

TGGGGAATATTGCGCAATGGGCGAAAGCCTGACGCAGCGACGCCGCGTGAGGGATGAAGG

CCTTCGGGTTGTAAACCTCTTTCAGCAGGAACGAATTCAGACGGTACCTGCAGAAGAAGC

ACCGGCTAACTACGTG

>2826 len=136 tot-seq=3

GCGCGCAAATTACCCACTCCCGACCCGGGGAGGTAGTGACGAAAAATAACAATACAGGAC

TCTTTCAAAACCCTATAATTAAAATAAATCCACTTTAAATCCTTTAACGAGGATCCATTG

GAGGGCAAGTCTGGTG

>2827 len=136 tot-seq=3

TGGGGAATATTACACAATGGGGGAAACCCTGATGCAGCGACGCCGCGTGAGCGATGAAGT

ATTTCGGTATGTAAAGCTCTATCAGCAGGGAAGAATTAGGACGGTACCTGACTAAGAAGC

ACCGGCTAAATACGTG

>2828 len=136 tot-seq=3

GCGCGCAAATTACCCACTCCCGACCCGGGGAGGTAGTGACGAAAAATAACAATACAGGAC

TCTTTTGAGGTCCTGTAATTGGAATGAGTCCACTTTAAGTCCTTTAATGAGGATCCATTG

GAGGGCAAATCTAGTG

>2829 len=136 tot-seq=3

TGGGGAATATTGCACAATGGGGGAAACCCTGATGCAGCGACGCCGCGTGAGGGAAGAAGG

TTTTCGGATTGTAAACCTCTGTCTTTGGTGACGAAAGATGACGGTAGCCAAAGAGGAAGC

TACGGCTAACTACGTG

>2830 len=136 tot-seq=3

TGGGGAATCTTCCGCAATGGACGAAAGTCTGACGGAGCAACGCCGCGTGAACGATGACGG

CCTTCGGGTTGTAAAGTTCTGTTATACGGGGAAGATAATGACGGTACCCTGTGAGGAAGC

CCCGGCTAACTACGTG

>2831 len=136 tot-seq=3

TGGGGGATATTGCACAATGGGGGAAACCCTGATGCAGCAACGCCGCGTGAGGGAAGACGG

TCTTCGGATTGTAAACCTAAGTTGTCAGGGACGAATTATGACGGTACCTGAAGAGAAAGC

TCCGGCTAACTACGTG

>2832 len=136 tot-seq=3

TCGGGAATATTGCGCAATGGAGGAAACTCTGACGCAGTGACGCCGCGTATAGGAAGAAGG

TTTTCGGATTGTAAACTATTGTCTGCAGGGGAGAAAAAAGACTGTACCTGCAAAGAAAGC

TCCGGCTAACTACGTG

>2833 len=136 tot-seq=3

TAAAGAATCTTCCACAATGGACGAAAGTCTGATGGAGCAATACCGCGTGGGTGATGAATG

TCGAAAGATTGTAAAACCCTTTTCTGAGGGAAGAATTATGACGGTACCTCAGGAATAAGC

ACCGGCTAACTCCGTG

>2834 len=136 tot-seq=3

TAAGGGATATTGCGCAATGGGCGAAAGCCTGACGCAGCAACGCCGCGTGAAGGATGAAGG

CCTTCGGGTCGTAAACTTTTTTTGACGCTGACGAGGAAGGACGGTAGGCGTCGAATAAGC

CACGGCTAACTACGTG

>2835 len=137 tot-seq=3

TGGGGAATATTGGACAATGGGGGGAACCCTGATCCAGCGACGCCGCATGAGTGAAGAAGT

ATTTCGGTATGTAAAGCTCTATCAGCATGGAAGAAGAAATGACGGTACCTGAGTAAGAAG

CCTCGGCTAACTACGTG

>2836 len=137 tot-seq=3

TGGGGAATATTGGGCAATGGGGGAAACCCTGACCCAGCAACGCCGCGTGAAGGAAGAAGG

TCTTCGGATCGTAAACTTCTATCCTCGGTGAAGAGGAGAAGACGGTAGCCGAGAAGGAAG

CCCCGGCTAACTACGTG

>2837 len=137 tot-seq=3

TGGGGGATATTGCACAATGGGGGAAACCCTGATGCAGCGACGCCGCGTGGGCGAAGAAGT

ATTTCGGTATGTAGAGCCCTATCAGCAGGGAAGAAGAAATGACGGTACCTGAGTAAGAAG

CACCGGCTAAATACGTG

>2838 len=137 tot-seq=3

TGGGGAATATTGGGCAATGGGCGCAAGCCTGACCCAGCAACGCCGCGTGAAGGAAGGAGG

CCTTCGGGTTGTAAACTTCTTTTGTCGGGGACGAGTAGAAGACGGTACCTGGCGAATAAG

CCACGGCTAACTACGTG

>2839 len=137 tot-seq=3

TGGGGAATATTGCACAATGGGCGAAAGCCTGATGCAGCAACGCCGCGTGAGTGAAGAAGG

TCTTCGGATTGTAAAACTCTGTCCTTGGGGAAGAACAAATGACGGTACCCTTGGAGGAAG

CCCCGGCTAACTACGTG

>2840 len=137 tot-seq=3

TGACATTGTTCATGAGATGAAAAATGAAAAAAAAACAAGACTCAGAAGGCTCAATAACTT

CCCAAATTCACAGAGCCAGTGTGTGGCAGGGCTGTGAGCAAGACCACATCTGCTGGATCT

CAAAGCCCAGGTGTCTT

>2841 len=137 tot-seq=3

TGGGGAATATTGGGCAATGGACGCAAGTCTGACCCAGCAACGCCGCGTGAAGGAGGAAGG

CTTTCGGGTTGTAAACTTCTTTTGTCAGGGAACAGTAGAAGAGGGTACCTGACGAATAAG

CCACGGCTAACTACGTG

>2842 len=137 tot-seq=3

TGGGGAATATTGGGCAATGGGCGAAAGCCTGACCCAGCGACGCCGCGTGAAGGAAGAAGG

TCTTCGGATTGTAAACTTCTTTTATTAGGGACGAACAGATGACGGTACCTAATGAATAAG

CCACGGCTAACTACGTG

>2843 len=138 tot-seq=3

TAGGGAATATTGCACAATGGAGGAAACTCTGATGCAGCGACGCCGCGTGAGTGATGAAGG

CCTTCGGGTCGTAAAGCTCTATCGCATGGGAATAACACAATGAATGTACCATGCAAGAAA

GGATCGGCTAACTTCGTG

>2844 len=138 tot-seq=3

TAGGGAATATTGCGCAATGGGCGAAAGCCTGACGCAGCGACGCCGCGTGAGTGATGAAGG

CCTTCGGGTCGTAAAGCTCTGTTGTACGGGAAAAAGAAAGTGATGGTACCGTATAAGAAA

GGAACGGCTAACTTCGTG

>2845 len=138 tot-seq=3

TGGGGAATTTTCCGCAATGGGCGAAAGCCTGACGGAGCAATACCGCGTGAGGGAGGAAGG

CTCTTGGGTTGTAAACCTCTTTTCTCAAGGAATAAGAAAGTGAGGGTACTTGAGGAATAA

GCATCGGCTAACTCCGTG

>2846 len=138 tot-seq=3

TAGGGAATCTTCGGCAATGGACGGAAGTCTGACCGAGCAACGCCGCGTGAGTGAAGAAGG

TTTTCGGGTTGTAAAGCACTTTCGGAGGGGAGGAAAAAAATGACGTTACCCTCAGAAGAA

GCACCGGCTAACTCCGTG

>2847 len=138 tot-seq=3

TGGGGAATATTGCACAATGGGGGAAACCCTGATGCAGCAACGCCGCGTGAAGGAAGAAGA

TCTTCGGATCGTAAACTTCTGTCCTTAGGGAAGAAAAAAATGACGGTACCTAAGGAGGAA

GCCCCGGCTAACTACATG

>2848 len=138 tot-seq=3

TGGCTTTGCTTATGGAGGTGGCATTGGAGGGGGCTTTGGTGGCCCTGGCTTCTCTGTCTG

TCCCTCTGGAGGCATTCAGGAAGTTACTGTCAACCAGAGCCTCCTGACTCCCCTCAACCT

GCAAATCGACCCCACTAT

>2849 len=138 tot-seq=3

TGGGGAATATTGCGCAATGGGGGAAACCCTGACGCAGCGACGCCGCGTGTGTGAAGAAGG

TTTTAGGACTGTAAAACACTTTTAGTAGGGAAGAAAAAAATGACGGTACCTACAGAAGAA

GCAACGGCTAAATACATG

>2850 len=138 tot-seq=3

TGGGGAATATTGCACAATGGGGGAAACCCTGATGCAGCAACGCCGCGTGAAGGAAGAAGG

GTTTCGGCTCGTAAACTTCTATCAACGGGGAAGAAAAAAATGACGGTACCCGAATAAGAA

GCACCGGCTAACTACGTG

>2851 len=138 tot-seq=3

TGGGGGATATTGCACAATGGGGGAAACCCTGATGCAGCGACGCCGCGTGAGTGAAGAAGT

ATTTCGGTATGTAAAGCTCTATCAGCAGGGAAGAAAGAAATGACGGTACCTGAATAAGAA

GCCCCGGCTAACTACGTG

>2852 len=138 tot-seq=3

TAGGGAATCTTGCACAATGGGCGAAAGCCTGATGCAGCAACGCCGCGTGAAGGATGAAGG

CCTTCGGGTTGTAAACTTCTGTTCTTAGGGAAGAAGAAAGTGACGGTACCTAAGGAGAAA

GCCCCGGCTAACTACGTG

>2853 len=138 tot-seq=3

TCGGGAATATTGGGCAATGGAGGAAACTCTGACCCAGCAACGCCGCGTGAAGGATGAAGG

TCCTCGGATTGTAAACTTCTGTCCTATGGGAAGAATAAAATGACGGTACCATAGAAGAAA

TCTCCGGCAAACTACGTG

>2854 len=138 tot-seq=3

TGGGGAATATTGCACAATGGGGGAAACCCTGATGCAGCGACGCCGCGTGAGTGAAGACGG

CCTTCGGGTTGTAAAGCTCTGTTTCGAGGGACGAAAACAATGACGGTACCTCGGGAGGAA

GCCACGGCTAACTACGTG

>2855 len=138 tot-seq=3

TGGGGAATATTGCACAATGGGGGAAACCCTGATGCAGCAACGCCGCGTGGAGGATGAAGG

ATTTCGGTTCGTAAACTCCTATCAACGGGGAAGAAAAAAATGACGGTACCCGAATAAGAA

GCACCGGCTAACTACGTG

>2856 len=138 tot-seq=3

TGGGGGATATTGCACAATGGAGGAAACTCTGATGCAGCAACGCCGCGTGAGGGAAGAAGG

TTTTCGGATTGTAAACCTCTGTTTTTAGTGAAGAAACAAATGACGGTAGCTAAAGAGGAA

GCCACGGCTAACTACGTG

>2857 len=138 tot-seq=3

TGGGGAATATTGGGCAATGGGCGAAAGCCTGACCCAGCAATGCCGCGTGAACGAAGAAGG

TCTTCGGATTGTAAAGTTCTGTCCTATGTGAAGAAAAAAATGACGGTAACATAGGAGGAA

GCCCCGGCTAACTACGTG

>2858 len=138 tot-seq=3

TGGGGAATCTTGCACAATGGGCGAAAGCCTGATGCAGCAACGCCGCGTGAGCGAAGAAGG

CCTTTGGGTCGTAAAGCTCTGTCCTAAGGGAAGAAGGAAGTGACGGTACCTTAGGAGGAA

GCCCCGGCTAACTACGTG

>2859 len=138 tot-seq=3

TGGGGAATATTGCACAATGGGGGAAACCCTGATGCAGCGACGCCGCGTGAGTGAAGAAGT

AATTCGTTATGTAAAGCTCTATCAGCAGGGAAGAAAGAAATGACGGTACCTGACCAAGAA

GCCCCGGCTAACTACGTG

>2860 len=138 tot-seq=3

TAGGGAATCTTCGGCAATGGACGCAAGCCTGACCCAGCAACGCCGCGTGAAGGAAGAAGG

CTTTCGGGTTGTAAACTTCTTTTCTCAGGGACGAAGCAAGTGACGGTACCTGAGGAATAA

GCCACGGCTAACTACGTG

>2861 len=138 tot-seq=3

TGGGGAATATTGGGCAATGGACGGAAGTCTGACCCAGCGACGCCGCGTGAGGGAAGAAGG

TCTTCGGATTGTAAACCTTAGTAAGCAGGGAAGAAGGAAGTGACGGTACCTGCAGAGTAA

GCCACGGCTAACTACGTG

>2862 len=138 tot-seq=3

TGGGGAATATTGGGCAATGGGCGCAAGCCTGACCCAGCAATGCCGCGTGATGGATGAAGG

TTTTCGGACTGTAAACATCTTTTATCAGGGACGAAACAAATGACGGTACCTGATGAATAA

GCCACGGCTAACTACGTG

>2863 len=138 tot-seq=3

TGGGGAATATTGGGCAATGGGCGAAAGCCTGACCCAGCAATTCTGTGTGTGTGAAGAAGG

TTTTAGGACTGTAAAACACTTTTAGTAGGGAAGAAAGAAATGACGGTACCTACAGAAGAA

GCGACGGCTAAATACGTG

>2864 len=138 tot-seq=3

TGGGGAATATTGCGCAATGGGGGCAACCCTGACGCAGCAACGCCGCGTGATTGATGAAGG

TCTTCGGATTGTAAAAATCTTTAATCAGGGACGAAGAAAATGACGGTACCTGAAGAATAA

GCTCCGGCTAACTACGTG

>2865 len=138 tot-seq=3

TGGGGGATATTGCACAATGGGGGAAACCCTGATGCAGCAACGCCGCGTGAGGTATGAAGG

CCTTTGGGTTGTAAGCCTCTGTCCTAGGGGAAGAAAAAAATGACGGTACCCGAGGAGGAA

GCCCCGGCTAACTACGTG

>2866 len=138 tot-seq=3

TGGGGAATATTGCACAATGGGGGAAACCCTGATGCAGCAACGCCGCGTGAAGGAAGAAGG

CCTTCGGGTTGTAAACTTCTGTCCTAGGGGAAGAAACAAATGACATTACCCTTGGAGGAA

GCCCCGGCTAACTACGTG

>2867 len=138 tot-seq=3

TGGGGGATATTGCACAATGGGCGAAAGCCTGATGCAGCGACGCCGCGTGAGGGAAGACGG

CCTTCGGGTTGTAAACCTCTGTCATTCGGGACGAAGAAAGTGACGGTACCGAAGAAGGAA

GCTCCGGTTAACTACGTG

>2868 len=138 tot-seq=3

TGGGGAATATTGGGCAATGGGCGAAAGCCTGACCCAGCAACGCCGCGTGAAGTATGAAGG

CCTTCGGGTTGTAAACTTCTTTGATCAGGGAAGAAACAAATGACGGTACCTTACTAAGAA

GCTCCGGCTAAATACGTG

>2869 len=138 tot-seq=3

TGGGGAATTTTGCACAATGGGGGAAACCCTGACCCAGCAACGCCGCGTGAAGTATGAAGG

CCTTCGGGTTGTAAACTTCTTTGATCAGGGAAGAAACAAATGACGGTACCTGAAAAACAA

GCCACGGCTAACTACGTG

>2870 len=139 tot-seq=3

CGCAAGCCTGATGGAGCAACGCCGCGTGAGTGAAGAAGGGTTTCGGCTCGTAAAGCTCTG

TTGTTGGAGAAGAACGTGCGAGGTAGTAACTGTTCACGAAGTGACGGTAACCAACCAGAA

AGTCACGGCTAACTACGTG

>2871 len=139 tot-seq=3

TGGGGAATATTGGACAATGGACCGAGAATCTGATCCAGCAATTCTGTGTGCACGATGAAG

TTCTTCGGAATGTAAAGTGCTTCCAGTTGGGAAGAAAGAAATGACGGTACCAACAGAAGA

AGTGACGGCTAAATACGTG

>2872 len=139 tot-seq=3

CTATATGCAGGTGGGGAGTGTGTTCTTTGGCCTCAAGTGTTCGCTGTACGTGAGGTAGCC

TATCCTCGGGGTGCTTATGAATGTGTGGTGGCTTTGCTGCTAGGGGAAGCAGCGTGGCTG

CCAGTGGCTTGCCCTTTGG

>2873 len=140 tot-seq=3

TGGGGAATATTGTGCAATGGGGGAAACCCTGACGCAGCGACGCCGCGTGCGGGATGAAGG

CCTTCGGGTTGTAAACTCCTTTCGTCAGGGACGAAGTTTGTTTGACGGTACCTGGATAAG

AAGCACCGGCTAACTACGTG

>2874 len=140 tot-seq=3

TGGGGAATATTGCACAATGGGCGCAAGCCTGATGCAGCGACGCCGCGTGAGGGATGACGG

CCTTCGGGTTGTAAACCTCTTTCACCAGGGACGAAGAGAAATTGACGGTACCTGGGGAAG

AAGCACCGGCCAACTACGTG

>2875 len=140 tot-seq=3

TGGGGAATATTGCACAATGGGCGGAAGCCTGATGCAGCGACGCCGCGTGGGGGATGACAG

CCTTCGGGTTGTAAACCTCTTTCAGCAGGGAAGAAGCGAGAGTGACGGTACCTGCAGAAG

AAGCGCCGGCTAACTACGTG

>2876 len=140 tot-seq=3

ACGCAAGTCTGATGGAGCAACGCCGCGTGAGTGAAGAAGGGTTTCGGCTCGTAAAGCTCT

GTTGTTGGTGAAGAAGGACAGGGGTAGTGGCGGGGCTTTAAATGACGGTAATCAATTAGA

AAGTCACGGCTAACTACGTG

>2877 len=140 tot-seq=3

TGGGGAATATTGCACAATGGGCGCAAGCCTGATGCAGCGACGCCGCGTGCGGGATGACGG

CCTTCGGGTTGTAAACCGCTTTTGATCGGGAGCAAGCGAGAGTGAGTTTACCCGTTGAAT

AAGCACCGGCTAACTACGTG

>2878 len=140 tot-seq=3

TGGGGAATATTGCGCAATGGGCGGAAGCCTGACGCAGCGACGCCGCGTGGGGGATGACGG

CCTTCGGGTTGTAAACCTCTTTCGACCGGGACGAAGCGAGAGTGACGGTACCGGTATAAG

AAGCACCGGCCAACTACGTG

>2879 len=140 tot-seq=3

TGGGGAATATTGCGCAATGGGGGAAACCCTGACGCAGCGACGCCGCGTGGGGGATGACGG

CCTTCGGGTTGTAAACTCCTTTCACCATCGACGAAGGTTTTCTGACGGTAGATGGAGAAG

AAGCACCGGCTAACTACGTG

>2880 len=140 tot-seq=3

TGGGGAATATTGCACAATGGGCAGAAGCCTGATGCAGCAACGCCGCGTGCGGGATGACGG

CCTTCGGGTTGTAAACCGCTTTCAGCAGGGACGAAGCTAACGTGACGGTACCTGCAGAAG

AAGCACCGGCTAACTACGTG

>2881 len=140 tot-seq=3

TGGGGAATATTGCACAATGGGCGAAAGCCTGATGCAGCAACGCCGCGTGCGGGATGACGG

CCTTCGGGTTGTAAACCGCTTTCACCCATGACGAAGCTAACGTGACGGTAGTGGGAGAAG

AAGCACCGGCTAACTACGTG

>2882 len=140 tot-seq=3

TGGGGAATATTGCGCAATGGGCGAAAGCCTGACGCAGCGACGCCGCGTGAGGGATGACGG

CCTTCGGGTTGTAAACCTCTTTCAGCTCTGACGAAGCGAGAGTGACGGTAGGAGCAGAAG

AAGCACCGGCCAACTACGTG

>2883 len=140 tot-seq=3

TGGGGAATATTGGACAATGGGCGGAAGCCTGATCCAGCAACGCCGCGTGAGGGATGACGG

CCTTCGGGTTGTAAACCTCTTTCGCCTGTGACGAAGCGCAAGTGACGGTAACAGGTAAAG

AAGCACCGGCCAACTACGTG

>2884 len=140 tot-seq=3

TGGGGAATATTGCGCAATGGGCGGAAGCCTGACGCAGCGACGCCGCGTGGGGGATGACGG

CCTTCGGGTTGTAAACCTCTTTCGCCAGGGACGAAGAGCAATTGACGGTACCTGGAGAAG

AAGCACCGGCCAACTACGTG

>2885 len=141 tot-seq=3

TGGGGAATATTGCACAATGGGCGCAAGCCTGATGCAGCGACGCCGCGTGGGGGATGACGG

CCTTCGGGTTGTAAACTCCTTTCGCCAAGGACGAAGAGAGTATTGACGGTACTTGGAGAA

GAAGCACCGGCTAACTACGTG

>2886 len=141 tot-seq=3

AGAAAGGCAAAGCCCAAAGCAAACAAATCCAAAAAAAGATGCAAGAAGTGAAGAAAGAAA

TATTCAAGGAAAAAGCATAAAGAAAAAATCCTCAGGAAACATTGGACACACTTATAGAAA

TGCAAAATGCTCTGGAAAGTC

>2887 len=141 tot-seq=3

TGGGGAATATTGCGCAATGGGGGAAACCCTGACGCAGCGACGCCGCGTGGGGGAATGAAG

GTCTTCGGATTGTAAACCCCTGTCATGTGGGAGCAAATTAAAAAGATAGTACCACAAGAG

GAAGAGACGGCTAACTCTGTG

>2888 len=142 tot-seq=3

CAGCCCGAGGAGTTGAATGAAAAGGCACAGTGGAGACTTCCAACCATGCAGCTAGCAGCT

CATCCCCAGCTCTGAGCAGCTGCCATCCATGGCTTCTAACCATGATTTCCACCTCTCTCT

GCATCCCATCCACACAGCAGGC

>2889 len=142 tot-seq=3

TAGGGAATGGACCACAATGGAAGAAAGTCTGATGGAGAAACGCCGCGAGAGTGAAGAAGG

TTTTCGGATGGTAAAGCTCTGTTGTTGGTGAAGAAGGATAGAGGTAGTAACTGGCCTTTA

TTTGACGGTAATCAACCAGAAA

>2890 len=142 tot-seq=3

TAGGGAATATTGCACAATGGAGGAAACTCTGATGCAGCCATGCCGCGTGTGTGAAGAAGG

CCTTCGGGTTGTAAACCGCTTTTGATTGGGAGCAAGCTTTCGGGTGAGTGTACCTTTCGA

ATAAGCGCCGGCTAACTACGTG

>2891 len=142 tot-seq=3

TGGGGAATATTGCGCAATGGGGGAAACCCTGACGCAGCGACGCCGCGTGATTTAGAAGGC

CTTCGGGTTGTAAACCGCTTTTGATTGGGAGCAAGCCTTTTGGGTGAGTGTACCTTTCGA

ATAAGCGCCGGCTAACTACGTG

>2892 len=142 tot-seq=3

TGGGGAATATTGCACAATGGAGGGAACTCTGATGCAGCGACGCCGCGTGAGTGATGAAGG

CCTTCGGGTTGTAAACCGCTTTTGATTGGGAGCAAGCTTTCGGGTGAGTGTACCTTTCGA

ATAAGCGCCGGCTAACTACGTG

>2893 len=142 tot-seq=3

TGGGGAATATTGCACAATGGGCGGAAGCCTGATGCAGCAACGCCGCGTGCGGGATGACGG

CGTTCGGGTTGTAAACCGCTTTTGATTGGGAGCAAGCCTTCGGGTGAGTGTACCTTTCGA

ATAAGCGCCGGCTAACTACGTG

>2894 len=142 tot-seq=3

TAGGGAATTTTTCACAATGGGCGCAAGCCTTATGAAGCAATGCCGCGTGAACGATGAAGG

TCTTCGGGTTGTAAACCGCTTTTGATTGGGAGCAAGCTTTCGGGTGAGTGTACCTTTCGA

ATAAGCGCCGGCTAACTACGTG

>2895 len=142 tot-seq=3

TAGGGAATCTTCGGCAATGGGGGGAACCCTGACCGAGCAACGCCGCGTGCGGGATGAAGG

CCTTCGGGTTGTAAACCGCTTTCAGCAGGGACGAGGCCGCAAGGTGACGGTACCTGCAGA

AGAAGCCCCGGCTAACTACGTG

>2896 len=142 tot-seq=3

TGGGGAATATTGGACAATGGGCGAAAGCCTGATCCAGCCATGCCGCGTGTGTGAAGAAGG

TCTTCGGGTTGTAAACCGCTTTTGATTGGGAGCAAGCTTTCGGGTGAGTGTACCTTTCGA

ATAAGCGCCGGCTAACTACGTG

>2897 len=142 tot-seq=3

TGGGGAATATTGCGCAATGGGGGAAACCCTGACGCAGCGACGCCGCGTGAGGGATGGAGG

CCTTCGGGTTGTAAGCCTCTTTTGTTTGGGAGCAAGCCTTCGGGTGAGTGTACCTTTCGA

ATAAGCGCCGGCTAACTACGTG

>2898 len=142 tot-seq=3

TAGGGAATCTTCCACAATGGACGCAAGCCTGATGCAGCGACGCCGCGTGCGGGATGACGG

CCTCCGGGTTGTAAACCGCTTTTGATCGGGAGCAAGCCTTCGGGTGAGTGTACCTTTCGA

ATAAGCACCGGCTAACTACGTG

>2899 len=142 tot-seq=3

TAGGGAATCTTCCGCAATGGGCGAAAGCCTGACGGAGCAACGCCGCGTGCGGGATGAAGG

CCTTCGGGTTGTAAACCGCTTTTGATTGGGAGCAAGCCTTCGGGTGAGTGTACCTTTCGA

ATAAGCGCCGGCTAACTACGTG

>2900 len=142 tot-seq=3

CCACGTGGAACTCGTGTCTCGAACACAGTGCGGTCTTCACACCTCAGCGCTGGGCATCGG

GAGGCAGCCTTCATGTGATGCTACCCTATGAGGCCAGCAAGGGCTCCCGGGAAATGAAAA

CGGCAGCAGCCGGCACCCAGTC

>2901 len=143 tot-seq=3

AGGTAACAGCAGAGAATAACCCCTATGCACAGATGCCGACCGGTGCCATGTTACCCTCTT

GTTATTAAACGCGTTCTCACGCCCGGCCAGGAAGAACACAACAGACCAGAATCTTCTGCG

GCAAAACTTTATTGCTTACATCT

>2902 len=143 tot-seq=3

TGGGGAATATTGCGCAATGGGGGAAACCCTGATGCAGCGACGCCGCGTGGGGGATGACGG

CCTTCGGGTTGTAAACTCCTTTCGCAACTGACGAAGCTTTTTTTGTGACGGTAGGTTGAG

AAGAAGCACCGGCTAACTACGTG

>2903 len=143 tot-seq=3

TGAGGAATATTGGTCAATGGACGCAAGTCTGACGCAGCGACGCCGCGTGCGGGATGAAGG

CCTTCGGGTTGTAAACCGCTTTTGATTGGGAGCAAGCCTTTTGGGTGAGTGTACCTTTCG

AATAAGCGCCGGCTAACTACGTG

>2904 len=143 tot-seq=3

TGGGGAATATTGCACAATGGGCGCAAGCCTGATGCAGCGACGCCGCGTGGGGGATGACGG

CCTTCGGGTTATAAACTCCTTTCGCTAGGGAAGAAGCCTTTTGGGTGACGGTACCTGGAT

AAGAAGCACCGGCTAACTACGTG

>2905 len=143 tot-seq=3

TAGGGAATCTTCCACAATGGACGAAAGTCTGATGGAGCAACGCCGCGTGAGTGAAGAAGG

CCTTCGGGTTGTAAACCGCTTTTGATTGGGAGCAAGCCTTTTGGGTGAGTGTACCTTTCG

AATAAGCGCCGGCTAACTACGTG

>2906 len=143 tot-seq=3

TGGGGGATATTGCACAATGGAGGCAACTCTGATGCAACAACGCCGCGTGCGGGATGAAGG

CCTTCGGGTTGTAAACCGCTTTTGATTGGGAGCAAGCCTTTTGGGTGAGTGTACCTTTCG

AATAAGCGCCGGCTAACTACGTG

>2907 len=143 tot-seq=3

TGGGGAATTTTGGACAATGGGGGCAACCCTGATCCAGCCATGCCGCGTGCAGGATGAAGG

TCTTCGGATTGTAAACTGCTTTTGATTGGGAGCAAGCCTTTTGGGTGAGTGTACCTTTCG

AATAAGCGCCGGCTAACTACGTG

>2908 len=143 tot-seq=3

GACGAGAATACTAGGCGCCATCATAAATTAAACCTCTGCTCAGTGGCTAATCTCACGGGC

GCCTCACAAAGACACAGACGGACTGCCTACAGCTCACTCGCGGCCTTGAGAGCTTACAAG

CAGAGGCGCCTCCCTCCCAGCTG

>2909 len=143 tot-seq=3

GATGTTCTTCACGTCCTCCGCCCGGAACGGCAGACTGTCTGTCCGGATGCCGCTCTCTTC

CAGCACTTCCAGGGTGTGGGACATATGGTCCAATATATGCAGGTACCGTATTCCTCCCCC

CTGCATCCCCATGCTAATCTGTC

>2910 len=144 tot-seq=3

TGGGGAATATTGCGCAATGGGCGGAAGCCTGACGCAGCGACGCCGCGTGGGGGATGACGG

CCTTCGGGTTGTAAACCTCTTTCGACCTTGACGAAGCCTTTTGGGGTGACGGTAGGGGTA

GAAGAAGCACCGGCCAACTACGTG

>2911 len=145 tot-seq=3

ATCTTAAGTGTGTCTTACCGAGGTTGGCTTTTGATCTGAGTGCAAAGTCCATATTAGTAG

GTAGGTGGTGCCCTTCTGAGAAGCATGGCACCTGGGACATCATGTTCTGTCAGAAAGAAG
CTGAAAGGAAGCAGAGTGACTGTGT
>2912 len=145 tot-seq=3
TGGGGAATATTGCACAATGGGCGCAAGCCTGATGCAGCGACGCCGCGTGCGGGATGACGG
CCTTCGGGTTGTAAACCGCTTTCAGTACAGAAGAAGCCCTTTTTGGGTGACGGTATGTGC
AGAAGAAGCGCCGGCTAACTACGTG
>2913 len=145 tot-seq=3
GGTTGTTCCTCTTGAGAAGGGAGCCTGGAGCTGGGGGCTTAATGAGTAATTCAAAGAGGA
AGAGGCTTGGAAGCTACCCTAGGAGGAGGCATGGGAAGCCTCAAGGTCTCTTGTGAGGAT
GGCAGAGTGAGCAGAATTACCAAGA
>2914 len=145 tot-seq=3
TGAGGAATATTGGTCAATGGGCGCGAGCCTGAACCAGCCAAGTCGCGTGAGGGATGACGG
CCCTACGGGTTGTAAACCTCTTTTGTCGGGGAGCAAATTCCGTTACGTGTAACGGAGTCA
AGAGTACCCGAAGAAAAAGCATCGG
>2915 len=146 tot-seq=3
TGGGGAATATTGCACAATGGGCGCAAGCCTGATGCAGCGACGCCGCGTGAGGGATGACGG
CCTTCGGGTTGTAAACCTCTTTCGCTAGGGAAGAAGCCACTTTTGTGGTGACGGTACCTG
GATAAGAAGCACCGGCTAACTACGTG
>2916 len=146 tot-seq=3
CTGTGGTCCCAAAGGCTTGGTCAGCCTGCCTTAACCCCCTCTCATGCCAGCCCACTCACC
CCAGCCGATGCCCAGGTAGAGGCTGAAGAAGCTCCTCTCGGGGAGGGTGGCCAGGCCCAG
CAGGTTGTGCAGGTACAGGCCCTCCA
>2917 len=146 tot-seq=3
CAATGGGCGCAAGCCTGATGCAGCCATGCCGCGTGTATGAAGAAGGCCTTCGGGTTGTAA
AGTACTTTCAGCGGGGAGGAAGGGGATGTGGTTAATAACCGCATTCATTGACGTTACCCG
CAGAAGAAGCACCGGCTAACTCCGTG
>2918 len=146 tot-seq=3
AGAGGGCTATGAAAGTGGGTCCCAGAAAGGACCCCCCTCCACATACACACCGCAAGCATC
TTTGGGGTTGGCCGGAGGGGCGGGCCCTGGCTGGGCTCAGGCGAGAATCCGCTGGAATGG

GAGGTGAGGATGATGGATGGCGCCTT

>2919 len=147 tot-seq=3

TGGGGAATAGGGCGCAATGGGGGAAACCCTGACGCAGAGATGCCGCGAGCGGGATGAAGG

CCTTCGGGTGGTAAACCGCTTTTGATTGGGAGCAAGCCTTTTGGGTGAGTGTACCTTTCG

AATAAGCGCAGGCTAACTACGTGCCAG

>2920 len=147 tot-seq=3

ACAATGGACGAAAGTCTGATGGAGCAACGCCGCGTGAGTGAAGAAGGGTTTCGGCTCGTA

AAGCTCTGTTGTTAGAGAAGAAAGATAGAGGGAGTAACTGGCCTTTATTTGACGGTAACT

ACTTAGAAAGTCACGGCTAACTACGTG

>2921 len=149 tot-seq=3

AAGCTCTCAAGGTGACTCTGTGTGTGTTGAACTTTGGTGAGCACTGACGTAGGGGAAGAA

CAGTGTTCAGAAGAAGGCTGAAGACTCTACCCCAAGGGCATTGAGGAGGCAGAAAGGAGC

ACTAGATCCAGAAACTGAGGTGGAGCAGT

>2922 len=149 tot-seq=3

GAAGGGAGGACAACTCTCTGGGGAGCAGAGAAGTGGTCAACAGGAGGAGGTCAAAGGCCC

CAGGGCGGGTTTTGCTTACAGCGTGAGAGTTGTCCCATGTTTGGGGCCGATAGAAGTGAT

CCAGTCCCCAGCAGCCGCGGTATAAGAAG

>2923 len=150 tot-seq=3

ACCCTCCCCACCCCCCACACACCCCCCTGCAATGATGGCATGCTTAAAGGCATGGAGGCT

GCCTCCAAGAAGACGTGTGAGATTGTAAATGTGCATCACAAGCGCCTCCGGCATCACAGA

GCTCCCTTCCACTGGGGGTGAAGCACTCTC

>2924 len=150 tot-seq=3

TGGGGAATATTGGACAATGGGCGGAAGCCTGATCCAGCAACGCCGCGTGAGGGATGACTG

CCTTCGGGTTGTAAACCTCTTTCAGTATCGATGAAGCGCCCGGATAGGGTGGTGACGGTA

GGTACAGAAGAAGCACCGGCCAACTACGTG

>2925 len=151 tot-seq=3

AGCGCTTGGGAGTGCAAGGCTTGACCATCAGTGGGTACCTCCGGCCTGCAAGAGCAGAAG

CAGAACAGAGCGTCACCTGTGCACACTGCAGAAAGGACACAGAGGAGAGTGTCTGTGGGG

CCACGTTGCTCCTCAGGACCCTTCAGTTTAT

>2926 len=152 tot-seq=3

TGGGGAATATTGCACAATGGGCGAAAGCCTGATGCAGCGACGCCGCGTGAGGGACGACGG

CCTTCGGGTTGTAAACCTCTTTCAGTAGGGAACAAGGCATCATTTTTGTGGTGTTGAGGG

TACTTGCAGAAGAAGCACCGACTAACTACGTG

>2927 len=152 tot-seq=3

CACTTAGGATGGGGTACAAGTGGATGGAAGGACAACACTGCCTGAACCGAGGGAGAGTGG

AGGTGCTGCATCCACCCACCTTGCCTGCTGAGGGTGCTTCTGAGATCTGTGCTGAGGTTT

GAAGTCCGCAGACGTCTAGAAGGTGCCTGAAG

>2928 len=152 tot-seq=3

CGGGGAGCTGCCAGCACCCTAGTGCCTGGGGTCTCTGAGACTAGTGCCAGCCCGGGAAGC

CCGTCTGTCCGCAGCATGTCAGGGCCAGAATCCTCCCCTCCCATTGGTGGGCCCTGTGAA

GCTGCTCCTTCATCCTCACTGCCCACTCCACC

>2929 len=152 tot-seq=3

TGGGGAATATTGCGCAATGGGGGAAACCCTGACGCAGCGACGCCGCGTGCGGGATGAAGG

CCTTCGGGTTGTGAACTCCTTTTTCTCGTGAAAAAGGCATGCTTTTGGGTGTGTTGATGG

TAGCGGGGGAAGAAGCGCCGGCTAACTACGTG

>2930 len=153 tot-seq=3

ATACCATTAGTTCCCTTTGTTGGGTCCTGCTGTTGTCCTGGACAGGAAGAAGTGTGTGAA

GTCCCTTTCTTTGTATTCTTTTCCAGGAAAAGTGATCCAAGTTGGAGGCCCCACTCCTGG

CACGCTACCAAGTACTTTGATGTCCACCCTGAG

>2931 len=153 tot-seq=3

TAGGGAATCTTCCACAATGGACGCAAGTCTGATGGAGCAACGCCGCGTGAGGGATGAAGG

CCTTCGGGTTGTAAACCTTTTTCGCTCATGGTCAAGCCGCAGCTTTGGGTTGTGGTGAGG

GTAGTGGGTAAAGAAGCGCCGGCTAACTACGTG

>2932 len=154 tot-seq=3

TAGGGAATATTCCACAATGAGCGAAAGCTTGATGGAGCGACACAGCGTGCACGATGAAGG

TCTTCGGATTGTAAACTGCTTTTATAAGGGAATAAAGTGAGTCTCGTGAGACTTTTTGCA

TGTACCTTATGAATAAGGACCGGCTAATTCCGTG

>2933 len=154 tot-seq=3

TGGGGAATATTGGACAATGGGCGGAAGCCTGATCCAGCAACGCCGCGTGAGGGATGACGG

CCTTCGGGTTGTAAACCTCTTTCAGTACCGACGAAGTTGTGCTTTTTTGGGTGTGGTGAC

GGTAGGTACAGAAGAAGCACCGGCCAACTACATG

>2934 len=154 tot-seq=3

TGGGGAATCTTCCGCAATGGACGAAAGTCTGACGGAGCAACGCCGCGTGAGTGAAGAAGG

TCTTCGGATTGTAAACTGCTTTTATATGGGAATAAAGTGAGGGACGTGTCCCTTATTGCA

TGTACCATACGAATAAGGACCGGCTAATTCCGTG

>2935 len=154 tot-seq=3

TAGGGAATATTGGGCAATGGAGAAAACTCTGACCCAGCCATGCCGCGTGCAGGAAGAAGG

CCCTCTGGGTTGTAAACTGCTTTTGATAGGGAAGAAAGAGGTTATGCGTAACCAGTTGAC

GGTACCTATCGAATAAGCACCGGCTAACTACGTG

>2936 len=154 tot-seq=3

TGGGGAATATTGCACAATGGGGGAAACCCTGATGCAGCAACGCCGCGTGAGTGATGAAGG

TTTTCGGATCGTAAACCTCTTTTATAAGGGAATAAAGTGCGGGACGTGTCCCGTTTTGTA

TGTACCTTATGAATAAGGATCGGCTAACTCCGTG

>2937 len=154 tot-seq=3

ATATCATTTGGTGTGGTGTCTAGGAAAACTGGAATGAACTCCAGAATGTTCAGTTCAGTT

CCTGACTGAGAGGCCTGGGGAACAGTCAACCAACGCTGACTGAGTCCGTGGATGGAGTCC

TGTGCTCTGGTGTGAGAGAAAACCAGACTCTTAG

>2938 len=154 tot-seq=3

CCACCACATGTGATTAGTTTCTTGTTGTTCTCTAACTGGGAGCCAGATGGATCAAGTAGC

TCAGGACCTAAAACTGACCTACTTAGAGCCATCAGGTCTGATGAGGTGAGCATAGCTTGG

GAGTCCAAAGACCTGAATGCCAGTCCTAGTCCTT

>2939 len=154 tot-seq=3

TAGGGAATATTGGGCAATGGGCGCAAGCCTGACCCAGCCATGCCGCGTGCCGGAAGAAGG

CCCTCTGGGTTGTAAACGGCTTTTGACGGGGAAGAAACGACCCTTGCGAGGGGCATTGAC

GGTACCCGCAGAATAAGCACCGGCTAACTCCGTG

>2940 len=154 tot-seq=3

TGAGGAATATTGGTCAATGGTCTGACGACTGAACCAGCCATGCCGCGTGAAGGTCACGGC

CCTATGGGTCTTAAACTTCTTTTGCGAGGGAGCAATAAGGTCTACGTGTAGTCCGATGAG

AGTACCTCGCGAATAAGCATCGGCTAACTCCGTG

>2941 len=154 tot-seq=3

TGCTGACCACTGTGACACCGTGCCACCCAAACAAAAACAGAAAATGCTGGAGAAACTCAA

CAGATCTGGCAGCATCTGTGGAGAGGGAATGTTGAAAATGATGCTGTAAGAATGTGTTGA

GATTTTTCAGCATCTTCCGTTTTTGTTTCAGATT

>2942 len=155 tot-seq=3

TGAGGAATATTGGTCAATGGACGAGAGTCTGAACCAGCCAAGTAGCGTGAAGGATGACTG

CCCTATGGGTTGTAAACTTCTTTTATATGGGAATAAAGTGAGGGACGTGTCCCTTATTGC

ATGTACCATACGAATAAGGGCCGGCTAATTCCGTG

>2943 len=155 tot-seq=3

TGAGGAATATTGGTCAATGGGCGAGAGCCTGAACCAGCCAAGTAGCGTGAAGGATGAAGG

TTCTATGGATTGTAAACTTCTTTTATAAGGGAATAAAGTGCTTTACGTGTAGAGTTTTGT

ATGTACCTTATGAATAAGCATCGGCTAACTCCGTG

>2944 len=155 tot-seq=3

TGAGGAATATTGGTCAATGGGCGCGAGCCTGAACCAGCCAAGTCGCGTGAAGGAAGACGG

CCCTACGGGTTGTAAACTTCTTTAGTCGGAGAGTAAAGGTCGGTACGTGTACCGATTTGC

AAGTATCCGAAGAATAAGCATCGGCTAATTCCGTG

>2945 len=155 tot-seq=3

TGGGGAATATTGCGCAATGGGGGGAACCCTGACGCAGCGACGCCGCGTGCAGGATGACGG

CCCTATGGGTTGTAAACTGCTTTTATAAGGGAATAAAGTGAGTCTCGTGAGGCTTTTTGC

ATGTACCTTATGAATAAGGACCGGCTAATTCCGTG

>2946 len=155 tot-seq=3

TGAGGAATATTGGTCAATGGCCGGAAGGCTGAACCAGCCAAGTCGCGTGAGGGACGACTG

CCCTATGGGTTGTAAACCTCTTTTATAAGGGAAGAATAAATGCTACGTGTAGCATGATGC

CTGTACCTTATGAATAAGCATCGGCTAACTCCGTG

>2947 len=155 tot-seq=3

TGGGGAATATTGCACAATGGGCGCAAGTCTGAACCAGCCAAGTAGCGTGCAGGATGACGG

CCCTATGGGTTGTAAACTGCTTTTGTATGGGGATAAAGTTAGGGACGTGTCCCTATTTGC

AGGTACCATACGAATAAGGACCGGCTAATTCCGTG

>2948 len=155 tot-seq=3

TGAGGAATATTGGTCAATGGCCGAGAGGCTGAACCAGCCAAGTAGCGTGAAGGATGAAGG

TTCTATGGATTGTAAACTTCTTTTATACGAGAATAACACCATCTACGTGTAGATGCCTGC

ATGTACCGTATGAATAAGCATCGGCTAACTCCGTG

>2949 len=155 tot-seq=3

TGAGGAATATTGGTCAATGGACGAGAGTCTGAACCAGCCAGGTAGCGTGCAGGATGACGG

CCCTATGGGTTGTAAACTGCTTTTACGCGGGGATAAAGTGCGTGACGTGTCGCGCATCGC

AGGTACCGCGTGAATAAGGACCGGCTAATTCCGTG

>2950 len=155 tot-seq=3

TGAGGAATATTGGTCAATGGGCGCGAGCCTGAACCAGCCAAGTCGCGTGAGGGAAGACGG

TCCTATGGATTGTAAACCTCTTTTGCCGGGGAGCAAAAACTGCCACGCGTGACAGGATGA

GAGTACCTGAAGAAAAAGCATCGGCTAACTCCGTG

>2951 len=155 tot-seq=3

TGGGGAATATTGCACAATGGGGGGAACCCTGATGCAGCGACGCCGCGTGAGGGAAGACGG

CCCTATGGGTTGTAAACCTCTTTTGTCGGAGAACAAAACCCGGGACGTGTCCCGGACTGC

GTGTATCCGAAGAAAAAGCATCGGCTAACTCCGTG

>2952 len=155 tot-seq=3

TGGGGAATATTGCGCAATGGGGGAAACCCTGACGCAGCGACGCCGCGTGCGGGATGACGG

CCCTATGGGTTGTAAACTGCTTTTATATGGGAATAAAGTGAGGGACGCGTCCCTTATTGC

ATGTACCATATGAATAAGGACCGGCTAATTCCGTG

>2953 len=155 tot-seq=3

TGAGGAATATTGGTCAATGGTCGGAAGACTGAACCAGCCAAGCCGCGTGAAGGAAGAAGG

TGCTCGGCATCGTAAACTTCTTTTGTCAGGGAACAAAGGGCGGTACGTGTACCGCTGTGA

GTGTACCTGAAGAAAAAGCATCGGCTAACTCCGTG

>2954 len=155 tot-seq=3

TGAGGAATATTGGTCAATGGACGGAAGTCTGAACCAGCCAAGTAGCGTGCAGGGTGACGG

CCCTATGGGTTATAAACTGCTTTTGTATGGGGATAAAGTTAGGGACGTGTCCCTATTTGC

AGGTACCATACGAAGAAGCACCGGCTAACTCCGTG

>2955 len=155 tot-seq=3

TGAGGAATATTGGTCAATGGGCGGGAGCCTGAACCAGCCAAGCCGCGTGAGGGAAGAAGG

CCCTACGGGTCGTAAACCTCTTTTGCCGGGGAACAAAAGCGGGTATGTATACCCGTATGA

GTGTACCCGGAGAAAAAGCATCGGCTAACTCCGTG

>2956 len=155 tot-seq=3

TGAGGAATATTCCACAATGGGCGAAAGCCTGATGGAGCAATGCCGCGTGCAGGATGACGG

CCCTATGGGTTGTAAACTGCTTTTATATGGGAATAAAGTGAGGGACGTGTCCCTTATTGC

ATGTACCATATGAATAAGGACCGGCTAATTCCGTG

>2957 len=155 tot-seq=3

TAGGGAATTTTCGGCAATGGGGGGAACCCTGACCGAGCAACGCCGCGTGAGTGAAGACGG

CCTTCGGGTTGTAAAGCTCTGTTGTAAGAAAAGATAAACTAGGAGAGGATCTTAGTCAGA

CATTATCTTACCAGAAAGCCACGGCTAACTACGTG

>2958 len=155 tot-seq=3

TGAGGAATATTGGTCAATGGGCGAGAGCCTGAACCAGCCAAGTAGCGTGCAGGAAGACGG

CCCTATGGGTTGTAAACTGCTTTTATGTGGGGATAAAGTGCGTGACGTGTCATGCATTGC

AGTTACCATACGAATAAGCATCGGCTAACTCCGTG

>2959 len=155 tot-seq=3

TGAGGAATATTGGTCAATGGGCGCAAGCCTGAACCAGCCAAGTAGCGTGAAGGATGACTG

CCCTATGGGTTGTAAACTTCTTTTATATGAGAACAAATGTAGTTACGTGTAACTATTTGC

GTGTACCATATGAATAAGCATCGGCTAACTCCGTG

>2960 len=155 tot-seq=3

GGAGGAATATGGGTCAAGGGGCGAGAGCAGGAACCAGCCAAGGAGCGGGCAGGAGGACGG

CCCGAGGGGTTGTAAACGGCTTTTATATGGGAATAAAGTGAGGGACGTGTCCCTTATTGC

ATGTACCATATGAATAAGGACCGGCTAATTCCGTG

>2961 len=155 tot-seq=3

TGAGGAATATTGGTCAATGGGCGGGAGCCTGAACCAGCCAAGTAGCGTGCAGGAAGACGG

CCCTATGGGTTGTAAACTGCTTTTATGCGGGGATAAAGGAGTCTACGTGTAGGTGTTTGC

AGTTACCATACGAATAAGCATCGGCTAACTCCGTG

>2962 len=155 tot-seq=3

TGGGGAATATCGCACAATGGGCGAAAGCCTGAACCAGCCAAGTAGCGTGCAGGATGACGG

CCCTATGGGTTGTAAACTGCTTTTATGCGGGGATAAAGTGCGCGACGTGTCGTGCATTGC

AGGTACCGCATGAATAAGGACCGGCTAATCCCGTG

>2963 len=155 tot-seq=3

TGAGGAATATTGGTCAATGGTCGAGAGACTGAACCAGCCAAGTAGCGTGCAGGATGACGG

CCCTCCGGGTTGTAAACTGCTTTTAGTTGGGAATAAAGTTCGGGACGTGTCCCGTTTTGT

ATGTACCATCAGAAAAAGGACCGGCTAATTCCGTG

>2964 len=155 tot-seq=3

TGAGGAATATTGGTCAATGGGCGGAAGCCTGAACCAGCCAAGTCGCGTGAAGGATGACTG

CCCTATGGGTTGTAAACTTCTTTTGTAATGGGAGAATAAGGATTACGTGTAATCCGATGA

CTGTACTTTACGAATAAGCATCGGCTAACTCCGTG

>2965 len=155 tot-seq=3

TGAGGAATATTGGTCAATGGACGAGAGTCTGAACCAGCCAAGTAGCGTGCAGGACGACGG

CCCTATGGGTTGTAAACTGCTTTTATGCGGGGATAAAGTCACCCACGTGTGGGTGTTTGC

AGGTACCGCATGAATAAGGACCGGCTAATTCCGTG

>2966 len=155 tot-seq=3

TAAGGAATATTGGTCAATGGACGCAAGTCTGAACCAGCCATGCCGCGTGAAGGATTAAGG

TCCTCTGGATTGTAAACTTCTTTTATCTGGGACGAAAAAAGGCGATTCTTCGTCACTTGA

CGGTACCAGATGAATAAGCACCGGCTAACTCCGTG

>2967 len=155 tot-seq=3

TGAGGAATATTGGTCAATGGGCGAGAGCCTGAACCAGCCAAGTAGCGTGAGGGATGACCG

CCCTACGGGTTGTAAACCTCTTTTATAAGGGAATAAAATGAAGTACGCGTACTTCTTTGC

ATGTACCTTATGAATAAGCATCGGCTAACTCCGTG

>2968 len=155 tot-seq=3

TGGGGAATCTTGCACAATGGGCGAAAGCCTGATGCAGCGACGCCGCGTGCAGGATGACGG

CCCTATGGGTTGTAAACTGCTTTTATGTGGGGATAAAGTGCGTGACGTGTCATGCATTGC

AGGTACCACATGAATAAGGACCGGCTAATTCCGTG

>2969 len=155 tot-seq=3

TGGGGAATATTGCACAATGGACGGAAGTTTGATGCAGCCAAGTAGCGTGCAGGATGACGG

CCCTATGGGTTGTAAACTGCTTTTGTTTGGGAATAATCGACATTACGTGTAATGTTTTGC

ATGTACCATTCGAATAAGGACCGGCTAATTCCGTG

>2970 len=155 tot-seq=3

TGAGGAATATTGGTCAATGGGCGAGAGCCTGAACCAGCCAAGTAGCGTGCAGGATGACGG

CCCTATGGGTTGTAAACTGCTTTTATATGGGAATAAAGGAGTCCACGTGTGGGCTTTTGC

AGGTATCGCATGAATAAGGACCGGCTAATTCCGTG

>2971 len=155 tot-seq=3

TGAGGAATATTGGACAATGGGTTAGCGCCTGATCCAGCCATCCCGCGTGAAGGATTAAGG

ACCTATGGTTTGTAAACTTCTTTTGTACAGGGATAAACCTACTCTCGTGAGAGTAGCTGA

AGGTACTGTACGAATAAGCACCGGCTAACTCCGTG

>2972 len=155 tot-seq=3

TGAGGAATATTGGTCAATGGACGCAAGTCTGAACCAGCCATGCCGCGTGCAGGATGACGG

TCCTATGGATTGTAAACTGCTTTTGTACGAGAAGAAACCTTTCTACGTGTAGAAAGCTGA

CGGTATCGTAAGAATAAGGATCGGCTAACTCCGTG

>2973 len=155 tot-seq=3

TGAGGAATATTGGTCAATGGGCGGAAGCCTGAACCAGCCAAGTCGCGTGGAGGAAGACGG

CCCTACGGGTTGTAAACTCCTTTTACCTGGGAATAACGGGCGCTACGCGTAGCGCTGTGC

AAGTACCGGGAGAATAAGCATCGGCTAATTCCGTG

>2974 len=155 tot-seq=3

TGAGGAATATTGGTCAATGGCCGGAAGGCTGAACCAGCCAAGTCGCGTGAGGGAATGAGG

CCCTACGGGTCGTGAACCTCTTTTGCCGGGGAGCAAAGCCGCCCACGTGTGGGCGGAAGG

AGAGTACCCGGAGAAAAAGCATCGGCTAACTCCGT

>2975 len=155 tot-seq=3

TGAGGAATATTGGTCAATGGGGGGAACCCTGAACCAGCCAAGTAGCGTGCAGGATGACGG

CCCTATGGGTTGTAAACTGCTTTTGTGCGTGGATAAAGTAGGCCACTTGTGGCCTTTTGC

AGGTACCGCACGAATAAGGACCGGCTAATTCCGTG

>2976 len=155 tot-seq=3

TGAGGAATATTGGTCAATGGGCGAGAGCCTGAACCAGCCATCCCGCGTGCAGGATGACGG

CCCTATGGGTTGTAAACTGCTTTTCTGCTCCAAGAAACCTGATTACGAGTAATCAGTTGA

CGGTAGAGTAGGAATAAGCATCGGCTAACTCCGTG

>2977 len=155 tot-seq=3

TGGGGAATCTTCCGCAATGGACGAAAGTCTGACGGAGCAACGCCGCGTGAGTGAAGACGG

CCCTATGGGTTGTAAACTGCTTTTGTTTGGGAATAATCGACATTACGTGTAATGTTTTGC

ATGTACCATTCGAATAAGGACCGGCTAATTCCGTG

>2978 len=156 tot-seq=3

TGAGGAATATTGGTCAATGGGCGCAGGCCTGAACCAGCCAAGTAGCGTGAAGGATGACTG

TCCTATGGGTTGTAAACTTCTTTTATAAAGGAATAAAGTCGGGTATGGATACCCCGTTTG

CATGTACTTTATGAATAAGGATCGGCTAACTCCGTG

>2979 len=156 tot-seq=3

TGAGGAATATTGGTCAATGGGCGGAAGCCTGAACCAGCCAAGTCGCGTGAGGGATGACGG

CCCTACGGGTTGTAAACCTCTTTTGCCGGGGAACAAATGGCGGGACGCGTCCCGCAGTGG

AGTGTACCCGGAGAAAAAGCATCGGCTAACTCCGTG

>2980 len=156 tot-seq=3

TGGGGAATCTTGCACAATGGGCGAAAGCCTGATGCAGCGACGCCGCGTGAAGGAAGACTG

CCCGCAAGGGTTGTAAACTTCTTTTGTATGGGATTAAAGTCACCTACGTGTAGGTGTTTG

CAGTTACCATACGAATAAGCATCGGCTAACTCCGTG

>2981 len=157 tot-seq=3

CCCGCCAACAGACCAAAGGGATTAACAGCCTCCGCCGTCCACACCCACACATGCACCCTC

CGGTTGCCCCCTGTAAGAAACTGAGGTGCCCTCCCACCCGCTCGTTCACACCTACACGAG

GCCGGAGCAAAGGTCAGTATCCACCGCACCCCTCTTG

>2982 len=157 tot-seq=3

GAAGGCAGCCTACGGGGAGAAGATCATGCTTCCCCAGGACCTTAGGAAAAACAAGGGAAA

AGCTGGGAGGATGAGTCTACAGAGAGAGGGAGGAAGGGAGCCCCACTTTGGCCACCCAGC

TGCACCCTAGTGTTTGGGTCCTTGGCCTCTATAGTGT

>2983 len=158 tot-seq=3

GCTCAGGTTCTGTACTTGTCCCTACATGTCCACTCAGTCTCAGCCTGGGTCTACTCTCTA

ACCTTAATTGGAGGTCTTCCCTGCCTACCTCTGGGATGTCATTAGAAAAATGATACAAGT

TGGAAACTCACAGCCCTGGCTGTACAGACAGCGACCGC

>2984 len=158 tot-seq=3

TGGGGAATTTTGCACAATGGGGGAAACCCTGATGCAGCGACGCGTGAGTGAAGAGGGTTT

TCGGATCGTAAAGCTCTGTTGTTAAGGAAGAACGAGTGTGAGAATGGAAAGTTCATGCTG

TGACGGTACTTAACCAGAAAGGGACGGCTAACTACGTG

>2985 len=159 tot-seq=3

TTTTTTTTTTCAAGCAGAAGACGGCATACGAGATAACACTGTGACTGGAGTTCAGACGTG

TGCTCTCCGATCTCCTACGGGAGGCAGCGGTGATTAACCTTTAGCAATAAACGAAAGTTT

AACTAAGCTATACTAACCCCAGGGTTGGTCAATTTCGTG

>2986 len=159 tot-seq=3

TGGGGAATATTGCGCAATGGGGGAAACCCTGACGCAGCGACGCCGCGTGCGGGATGAAGG

CCTTCGGGTTGTAAAGTTCTTTTATTTAGGAAAAAAAGCGTGCTAGGAAATGAGCGCGCC

TTGATGGTACTAATTGAATAAGTGACAGCTAGCTATGTG

>2987 len=159 tot-seq=3

TGGGGAATATTGGACAATGGGCGCAAGCCTGATCCAGCAATGCCGCGTGTGTGAAGAAGG

CCTTTGGGTTGTAAAGCACTTTAGGCAGGGAAGAAAAAATCGGCGATAACATCGCCGGTC

TTGACGGTACCTGAGGAATAAGCACCGGCTAACTCTGTG

>2988 len=159 tot-seq=3

TGGGGAATTGTTCGCAATGGGCGCAAGCCTGACGACGCAACGCCGCGTGGAGGATGAAGG

TCTTCGGATTGTAAACTCCTGTTGCCCGGGACGAAACGCTCCGACCTAACACGTCGGAGC

CTGACGGTACCGGGTGAGGAAGCCCCGGCTAACTCTGTG

>2989 len=160 tot-seq=3

TAGGGAATCTTCCACAATGGACGAAAGTCTGATGGAGCAACGCCGCGTGAGTGAAGAAGG

CCTTCGGGTCGTAAAGCTCTGTTGCGGGGGAAAAAAGGCAGCATCAGGAAATGGGTGCTG

ACTGATGGTGCCCCGCCAGAAAGTCACGGCTAACTACGTG

>2990 len=160 tot-seq=3

TGGGGAATATTGCACAATGGGGGAAACCCTGATGCAGCGACGCCGCGTGGAGGAAGAAGG

TCTTCGGATTGTAAAGCACTTTAAGTTGGGAGGAAGGGTTGTAGATTAATACTCTGCAAT

TTTGACGTTACCGACAGAATAAGCACCGGCTAACTCTGTG

>2991 len=160 tot-seq=3

TGAGGAATATTGGTCAATGGGCGCAGGCCTGAACCAGCCAAGTAGCGTGAGTGATGAAGG

TCTTCGGATCGTAAAACTCTGTTATTAGGGAAGAACAAATGTGTAAGTAACTATGCACGT

CTTGACGGTACCTAATCAGAAAGCCACGGCTAACTACGTG

>2992 len=160 tot-seq=3

TGGGGAATATTGCACAATGGGGGAAACCCTGATGCAGCGACGCCGCGTGAGTGAAGAAGG

CCCTCGGGTTGTAAAGCTCTTTTGTCAGGGAAGAAAAGGTGATCTCTAATACAGGTCACT

CATGACGGTACCTGAAGAATAAGCACCGGCTAACTACGTG

>2993 len=160 tot-seq=3

TGGGGAATTTTGGACAATGGGCGAAAGCCTGATCCAGCAATGCCGCGTGTGTGAAGAAGG

CCTTCGGGTTGTAAAGCACTTTCAGTGAGGAAGAACGCCTGGTGGTTAATACCCATCAGG

AAAGACATCACTCACAGAAGAAGCACCGGCTAACTCCGTG

>2994 len=160 tot-seq=3

CTGCAGAGGGACTCTGTATCTCAGGAACGATGGCTCTGGCTCATTACTTAGCTTCTCCTA

AGAAATGTCCTTCCCTTGCAGGAGCTGAGGTTCCACTGAGCCAGGCACAGCTCAGAGATT

GACAGTCACCTCAGCTCCCAGCAGCCTCTGGTCAATCCAG

>2995 len=160 tot-seq=3

TGGGGAATTTTGGACAATGGACGCAAGTCTGATCCAGCCATTCCGCGTGCAGGATGAAGG

CCCTCGGGTTGTAAACTGCTTTTGTACGGAACGAAAAGCCTTCTTCTAATACAGGAGGGT

CATGACGGTACCGTAAGAATAAGCACCGGCTAACTACGTG

>2996 len=160 tot-seq=3

TGGGGAATCTTGCACAATGGGCGAAAGCCTGATGCAGCGACGCCGCGTGCGGGATGAAGG

CCTTCGGACCGTAAAACTCTGTTATTAGGGAAGAACATATGTGTAAGTAACTGTGCACAT

CTTGACGGTACCTAATCAGAAAGCCACGGCTAACTACGTG

>2997 len=160 tot-seq=3

TGGGGAATATTGCGCAATGGGGGAAACCCTGACGCAGCGACGCCGCGTGCGGGATGAAGG

TCTTCGGATCGTAAAACTCTGTTATTAGGGAAGAACATATGTGTAAGTAACTGTGCGCAT

CTTGACGGTACCTAATCAGAAAGCCACGGCTAACTACGTG

>2998 len=160 tot-seq=3

TGGGGAATACTGCACAATGGGCGCAAGCCTGATGCAGCCATGCCGCGTGTATGAAGAAGG

CCTTCGGGTTGTAAAGTACTTTCAGCGGGGAGGAAGGTGTTGCGGTTAATAACCGCAGCA

ATTGACGCTACCCGCAGAAGAAGCACCGGCTAACTCCGTG

>2999 len=160 tot-seq=3

TGGGGAATATTGGACAATGGGCGCAAGCCTGATCCAGCCATGCCGCGTGAGTGAAGAAGG

CCTTCGGGTTGTAAAGCTCTTTTGTCCGGAAAGAAAAGCCCTGGATTAATACTTTGGGGT

TATGACGGTACCGGAAGAATAAGCACCGGCTAACTTCGTG

>3000 len=160 tot-seq=3

TGGGGAATATTGGACAATGGGCGCAAGCCTGATCCAGCCATGCCGCGTGGGTGAAGAAGG

CCTTCGGGTTGTAAAGCCCTTTTGTCCGGGAAGAAATCGTCTGGGCTAATACCCCGGGCG

GATGACGGTACCGGAAGAATAAGCACCGGCTAACTTCGTG

>3001 len=160 tot-seq=3

TGGGGAATATTGCGCAATGGGGGAAGTCTGACCGAGCAACGCCGCGTGAGTGAAGAAGGT

TTTCGGATCGTAAAGCTCTGTTGTAAGAGAAGAACGAGTGTGAGAGTGGAAAGTTCACAC

TGTGACGGTATCTTATCAGAAAGGGACGGCTAACTACGTG

>3002 len=160 tot-seq=3

TGGGGAATATTGCACAATGGGCGCAAGCCTGATGCAGCGACGCCGCGTGAGTGAAGAAGG

GTTTCGGCTCGTAAAGCTCTGTTGTTAAAGAAGAACGTGGGTAAGAGTAACTGTTTACCC

AGTGACGGTATTTAACCAGAAAGCCACGGCTAACTACGTG

>3003 len=160 tot-seq=3

TGGGGAATATTGCACAATGGGGGAAACCCTGATGCAGCGACGCCGCGTGAGCGATGAAGT

ATTTCGGTATGTAAAGCTCTATCAGCGGGGAGGAAGGGAGTAAAGTTAATACCTTTGCTC

ATTGACGTTACCCGCAGAAGAAGCACCGGCTAACTCCATG

>3004 len=160 tot-seq=3

TGGGGAATATTGCGCAATGGGCGAAAGCCTGACGCAGCGACGCCGCGTGAGGGATGAAGG

TTCTCGGATCGTAAACCTCTGTTATTAGGGAAGAACAAATGTGTAAGTAACTATGCACGT

CTTGACGGTACCTAATCAGAAAGCCACGGCTAACTACGTG

>3005 len=160 tot-seq=3

TGGGGAATTTTGGACAATGGGCGCAAGCCTGATCCAGCAATGCCGCGTGCAGGAAGAAGG

CCTTCGGGTTGTAAACTGCTTTTGTACGGAACGAAACGGTCCTGGTTAATACCCGGGGCT

AATGACGGTACCGTAAGAATAAGCACCGGCTAACTACGTG

>3006 len=160 tot-seq=3

TGGGGAATATTGCACAATGGGGGAAACCCTGATGCAGCCATGCCGCGTGTGTGAAGAAGG

CCTTCGGGTTGTAAAGCACTTTCAGTCAGGAGGAAAGGTGGCTAGCTAATATCTAGTTGC

TGTGACGTTACTGACAGAAGAAGCACCGGCTAACTCCGTG

>3007 len=160 tot-seq=3

TGGGGAATTTTGGACAATGGGGGAAACCCTGATCCAGCCATCCCGCGTGTGCGATGAAGG

CCTTCGGGTTGTAAAGCACTTTTGGCAGGAAAGAAACGGCGCGTGCTAATACCATGCGCA

ACTGACGGTACCTGCAGAATAAGCACCGGCTAACTACGTG

>3008 len=160 tot-seq=3

TAGGGAATCTTCCACAATGGACGCAAGTCTGATGGAGCAACGCCGCGTGAGTGAAGAAGG

GTTTCGGCTCGTAAAGCTCTGTTGTTGGTGAAGAAGGACAGGGGTAGTAACTATTCACGC

AGTGACGGTATTCAACCAGAAAGTCACGGCTAACTACGTG

>3009 len=160 tot-seq=3

TGGGGAATTTTGGACAATGGGGGAAACCCTGATCCAGCCATCCCGCGTGTGTGATGAAGG

CCTTCGGGTTGTAAAGCACTTTTGACAGGGAAGAAATAGCAGAGGCTAATATCCTTTGTG

AATGACGGTACCTGTAGAATAAGCACCGGCTAACTACGTG

>3010 len=160 tot-seq=3

TAGGGAATCTTCCACAATGGACGCAAGTCTGATGGGGCAACGCCGCGTGAGTGAAGAAGG

GTTTCGGCTCGTAAAACTCTGTTGTAAGAGAAGAACAAATTGTAGAGTAACTGCTACAGT

CTTGACGGTATCTTACCAGAAAGCCACGGCTAACTACGTG

>3011 len=160 tot-seq=3

TAGGGAATCTTCCACAATGGACGCAAGCCTGATCCAGCCATGCCGCGTGTCTGAAGAAGG

CCTTCGGGTTGTAAAGGACTTTTGTCAGGGAAGAAAAGGCTGTTGCTAATACCGACAGCT

GATGACGGTACCTGAAGAATAAGCACCGGCTAACTACGTG

>3012 len=160 tot-seq=3

TGGGGAATCTTGCACAATGGGCGAAAGCCTGATGCAGCGACGCCGCGTGAGTGAAGAAGG

ATCTCGGTTCGTAAAACTCTGTTGTTAGGGAAGAAAAAATGTATAGTAACTATATACAAA

AGAGACGGTACCTAACCAGAAAGCCACGGCTAACTACGTG

>3013 len=160 tot-seq=3

TGGGGAATCTTGCACAATGGGCGAAAGCCTGATGCAGCGACGCCGCGTGCGGGATGAAGG

CCTTCGGATTGTAAAGTGCTGTTATAAGGGAAGAACATTTGCAATAGGAAATGATTGCAG

ACTGACGGTACCTTGTCAGAAAGCGATGGCTAACTATGTG

>3014 len=160 tot-seq=3

TGGGGAATATTGCGCAATGGGGGAAACCCTGATGGAGCAACACCGCGTGAGTGAAGAAGG

GTTTCGGCTCGTAAAACTCTGTTGTTGAAGAAGAACGTGCGTGAGAGTAACTGTTCACGC

AGTGACGGTATTCAACCAGAAAGTCACGGCTAACTACGTG

>3015 len=160 tot-seq=3

TAGGGAATCTTCCGCAATGGACGCAAGTCTGACGGAGCAACGCCGCGTGAGTGAAGAAGG

TTTTCGGATCGTAAAACTCTGTTGTAAGAGAAGAACAAATTGTAGAGTAACTGCTACAGT

CTTGACGGTAATCAATTAGAAAGTCACGGCTAACTACGTG

>3016 len=160 tot-seq=3

TGGGGAATCTTGCACAATGGGCGAAAGCCTGATGCAGCGACGCCGCGTGCGGGATGAAGG

TTTTCGGATCGTAAAGCTCTGTTGTTGGTGAAGAAGGATAGAGGTAGTAACTGGCCTTTA

TTTGACGGTAATCAACCAGAAAGTCACGGCTAACTACGTG

>3017 len=160 tot-seq=3

TGGGGAATATTGCGCAATGGGGGAAACCCTGACGCAGCGACGCCGCGTGAGTGAAGAAGG

GTTTCGGCTCGTAAAGCTCTGTTGGTAGTGAAGAAAGATAGAGGTAGTAACTGGCCTTTA

TTTGACGGTAATCAACCAGAAAGTCACGGCTAACTACGTG

>3018 len=160 tot-seq=3

TAGGGAATCTTCCACGATGGACGCAAGTCTGATGGAGCAACGCCGCGTGAGTGAAGAAGG

TTTTCGGATCGTAAAGCTCTGGTGGTAGTGAAGAAAGATAGAGGTAGTAACCGGCCTTTA

TTTGACGGTAATTACTTAGAAAGTCACGGCTAACTACGTG

>3019 len=160 tot-seq=3

TAGGGAATCTTCCACAATGGACGAAAGTCTGATGGAGCAACGCCGCGTGAGTGAAGAAGG

TTCTCGGATCGTAAAACTCTGTTGTGAGGGAAGAACAAGTACCGGAGTAACTGCCGGTAC

CTTGACGGTACCTCATTAGAAAGCCACGGCTAACTACGTG

>3020 len=160 tot-seq=3

TGAGGAATATTGGTCAATGGGCGAGAGCCTGAACCAGCCAAGTCGCGTGAATGAAGAAGG

CCTTCGGGTTGTAAAGTTCTTTCGGTATTGAGGAAGGTTGATGTGTTAATAGTACATCAA

ATTGACGTTAAATACAGAAGAAGCACCGGCTAACTCCGTG

>3021 len=160 tot-seq=3

TGGGGAATTTTGGACAATGGGGGAAACCCTGATCCAGCCATGCCGCGTGCGGGAAGAAGG

CCTTCGGGTTGTAAACCGCTTTTGTCGGGGACGAAAAGTCGTATTTAAATAAAGTACGAT

GCTGACGGTACCTGAAGAATAAGCACCGGCTAACTACGTG

>3022 len=160 tot-seq=3

TGGGGAATTTTGGACAATGGGCGCAAGCCTGATCCAGCAATGCCGCGTGAGTGAAGAAGG

CCTTCGGGTTGTAAAGCTCTTTTGTCAGGGAAGAAACGGGGTTGGCTAATATCCGATCCT

AATGACGGTACCTGAAGAATAATCACCGGCTAACTACGTG

>3023 len=160 tot-seq=3

TGGGGAATATTGCGCAATGGGGGAAACCCTGACGCAGCGACGCCGCGTGCGGGATGAAGG

CCTTCGGGTTGTAAAACTCTGTTATTAGGGAAGAACAAATGTGTAAGTAACTATGCACGT

CTTGACGGTACCTAATCAGAAAGCCACGGCTAACTACGTG

>3024 len=160 tot-seq=3

TAGGGAGTTTTCGGCAATGGGGGAAACCCTGACCGAGCAACGCCGCGTGAATGATGAAGG

CCTTCGGGTCGTAAAATTCTGTTGTAAGAGACGAACGGCAAGGGTAGGAAATGCCCCTTG

TGTGACTGTATCTTACGAGAAAGGGACGGCTAACCACGTG

>3025 len=160 tot-seq=3

TGAGGAATATTGCGTAATGGGGGAAACCCTGACGCAGCGACGCCGCGTGAAGGATGAAGG

TCTTCGGATTGTAAACTTCTGTTAAGTGGGAGGAAAGAATTGCTTTTAATAGAAGCAAGA

GATGACAGTACCACTAGAGAAAGCACCGGCTAAATTTGTG

>3026 len=160 tot-seq=3

TGGGGAATATTGCACAATGGACGCAAGTCTGATGGAGCAACGCCGCGTGAGTGAAAAAGG

TCTTCGGATTGTAAAACTCTGTTGTTAGAGAAGAACACGAGTGAGAGTAACTGTTCATTC

GATGACGGTATCTAACCAGCAAGTCACGGCTAACTACGTG

>3027 len=160 tot-seq=3

TGGGGAATATTGCACAATGGGGGGAACCCTGATGCAGCAACGCCGCGTGAGTGATGAAGG

TTTTCGGATCGTAAAGCTCTGTCTTTGGGGAAGAACGTGCGTGAGAGTAACTGTTCACGC

AGTGACGGTATCCAACCAGAAAGTCACGGCTAACTACGTG

>3028 len=160 tot-seq=3

TAGGGAATCTTCCGCAATGGACGAAAGTCTGACGGAGCAACGCCGCGTGAGTGATGAAGG

TTTTCGGATCGTAAAACTCTGTTGTTAGGGAAGAACAAGTACGAGGGTAACTACTCGTAC

CTTGACGGTACCTAACCAGAAAGCCACGGCTAACTACGTG

>3029 len=160 tot-seq=3

TAGGGAATCTTCCGCAATGGACGAAAGTCTGACGGAGCAACGCCGCGTGAACGATGAAGG

TTTTCGGATCGTAAAGTTCTGTTATCAGGGAAGAACAAGTATCGGAGTAACTGCCGGCAC

CTTGACGGTACCTGACCAGAAAGCCACGGCTAACTACGTG

>3030 len=160 tot-seq=3

TGGGGAATATTGCGCAATGGGGGAAACCCTGACGCAGCGACGCCGCGTGCGGGATGAAGG

CCTTCGGGTTGTAAAGCACTTTCAGTAAGGAGGAAAGGTTAAGTTTTAATAGAGCTTAGC

TGTGACGTTACTTACAGAAGAAGCACCGGCTAACTCCGTG

>3031 len=160 tot-seq=3

TGGGGAACTTTGGACAATGGGCGAAAGCCTGATCCAGCCATGCCGCGTGAGTGAAGAAGG

CCTTCGGGTTGTAAAGCTCTTTCGCAAGGAAAGAAAACTTATCTATTAATACTAGGTGAG

GTTGACGGTACCTTGATAAGAAGCACCGGCTAACTACGTG

>3032 len=160 tot-seq=3

TAGGGAATCTTCCACAATGGGCGCAAGCCTGATGGAGCAACACCGCGTGAGTGAAGAAGG

GTTTCGGCTCGTAAAGCTCTGTTGTTGGAGAAGAACGTGCGTGAGAGTAACTGTTCATAC

GTTGACGGTATTTAACCAGAAAGTCACGGCTAACTACGTG

>3033 len=160 tot-seq=3

TAGGGAATCTTCCACAATGGGCGCAAGCCTGATGGAGCAATGCCGCGTGAATGAAGAAGG

TCTTCGGATCGTAAAGTTCTGTTATTAGAGAAGAACAAGTTGAGGGATAACTATCTCGAC

TTTGACGGTACCTAACCAGAAAGTCACGGCTAACTACGTG

>3034 len=160 tot-seq=3

TGGGGAATATTGGACAATGGGCGCAAGCCTGATCCAGCCATGCCGCGTGTGTGAAGAAGG

CCTTCGGGTTGTAAAGCACTTTTGTTGGGGAAGAAAAGCCATGGGTTAATACCCTGTGGT

TCTGACGGTACCCAAAGAATAAGCACCGGCTAACTTCGTG

>3035 len=160 tot-seq=3

TGGGGAATATTGCACAATGGGCGCAAGCCTGATGCAGCAACGCCGCGTGAACGATGAAGG

TCTTCGGATTGTAAACTGCTTTTGTCAGGGAAGAAAAGGTCTGTGTTAATACCATAGACT

GCTGACGGTACCTGAAGAATAAGCACCGGCTAACTACGTG

>3036 len=160 tot-seq=3

TAGGGAATCTTCCACAATGGACGCAAGTCTGATGGAGCAACGCCGCGTGAGTGAAGAAGG

TCTTCGGATCGTAAAACTCTGTTGTTAGAGAAGAACACGAGTGAGAGTAACTGTTCATTC

GATGACGGTAATTACTTAGAAAGTCACGGCTAACTACGTG

>3037 len=160 tot-seq=3

TGGGGAATATTGGACAATGGGCGAAAGCCTGATCCAGCCATACCGCGTGTGTGAAGAAGG

TCTTAGGATTGTAAAGCACTTTAAGTTGAGAGGAAGGGTTGTAGCCTAATACGCTGTAAC

TTTGACGTTACCGACAGAATAAGCACCGGCTAACTCTGTG

>3038 len=160 tot-seq=3

TAGGGAATCTTCGGCAATGGACGAAAGTCTGACCGAGCAACGCCGCGTGAGTGATGAAGG

TTTTCGGATCGTAAAACTCTGTTGCCAGGGAAGAAGGATGAGGAGAGTAACTGCTCTTCA

TTTGACGGTACCTGAGAAGAAAGCCCCGGCTAACTACGTG

>3039 len=160 tot-seq=3

TGGGGAATATTGGACAATGGAGGAAACTCTGATCCAGCAATTCTGTGTGTGTGAAGAAGG

CCTTCGGGTTGTAAAGTACTTTCAGCGGGGAGGAAGGGAGTAAAGTTAATACCTTTGCTC

ATTGACGTTACCCGCAGAAGAAGCACCGGCTAACTCCGTG

>3040 len=160 tot-seq=3

TAGGGAATCTTCCGCAATGGACGAAAGTCTGACGGAGCAACGCCGCGTGAGTGATGAAGG

TTTTCGGATCGTAAAGCTCTGTTGCCAGGGAAGAACGTCCGTTAGAGTAACTGCTAACGG

AGTGACGGTACCTGAGAAGAAAGCCCCGGCTAACTACGTG

>3041 len=160 tot-seq=3

TGGGGAATATTGCGCAATGGGGGAAACCCTGACGCAGCAACGCCGCGTGTGTGAAGAAGG

TCTTCGGATTGTAAAGCACTTTAAGTTGGGAGGAAGGGCAGTAAGTTAATACCTTGCTGT

TTTGACGTTACCAACAGAATAAGCACCGGCTAACTTCGTG

>3042 len=160 tot-seq=3

TGGGGAATCTTCCGCAATGGACGAAAGTCTGACGGAGCAACGCCGCGTGAGTGAAGAAGG

TCTTCGGATTGTAAAACTCTGTCCTCATCCAAGAGAAGGGAAGGTAGTAACTGACCTTTC

CAGGACGGTAGATGAGGAGGAAGCCCCGGCTAACTATGTG

>3043 len=160 tot-seq=3

TGGGGAATATTGGACAATGGGCGCAAGCCTGATCCAGCCATGCCGCGTGTGTGAAGAAGG

CCTTCGGGTTGTAAAGCACTTTTGTTGGGGAAGAAAAGCAGTCGGTTAATACCCGGTTGT

CATGACGGTACCCAAAGAATAAGCACCGGCTAACTTCGTG

>3044 len=160 tot-seq=3

TAGGGAATCTTCCACAATGGGCGAAAGCCTGATGGAGCAATACCGCGTGAGTGAAGAAGG

TCTTCGGATCGTAAAGCTCTGTTGTTAGAGAAGAACACGCAGAACAGTAACTGGTTCTGC

GCTGACGGTATCTAACCAGAAAGTCACGGCTAACTACGTG

>3045 len=160 tot-seq=3

TAGGGAATCTTCGGCAATGGGGGCAACCCTGACCGAGCAACGCCGCGTGAGTGAAGAAGG

TTTTCGGATCGTAAAGCTCTGTTGTTAGAGAAGAACAAGGACGTTAGTAACTGAACGTCC

CCTGACGGTATCTAACCAGAAAGCCACGGCTAACTACGTG

>3046 len=160 tot-seq=3

TAGGGAATCTTCGGCAATGGGGGCAACCCTGACCCAGCCATGCCGCGTGTGTGAAGAAGG

TCTTCGGATTGTAAAGCACTTTAAGTTGGGAGGAAGGGCAGTAAATTAATACTTTGCTGT

TTTGACGTTACCGACAGAATAAGCACCGGCTAACTCTGTG

>3047 len=161 tot-seq=3

TAGGGAATTTTCGTCAATGGGCGAAAGCCTGACGGAGCAACGCCGCGTGAGTGAAGACGG

CCTTCGGGTTGTAAAGCTCTGTTATACGGGACGAATAGTCTTGTGGTTAATACCCATAAG

AAGTGACGGTACCGTAAGAGAAAGCCACGGCTAACTACGTG

>3048 len=161 tot-seq=3

TAGGGAATCTTCGGCAATGGGGGGAACCCTGACCGAGCAACGCCGCGTGAGTGAAGAAGG

TTTTCGGATCGTAAAGCTCTGTTGTTAAGGAAGAACGTTGGTAGGAGTGGAAAATCTACC

AAGTGACGGTAACTAACCAGAAAGGGACGGCTAACTACGTG

>3049 len=161 tot-seq=3

TAGGGAATCTTCCACAATGGACGAAAGTCTGATGGAGCGACGCCGCGTGAGTGAAGAAGG

TTTTCGGATCGTAAAGCTCTGTTGTAAGAGAAGAACGAGTATGAGAGTGGAAAATTCATA

CTGTGACGGTATCTTACCAGAAAGGGACGGCTAACTACGTG

>3050 len=161 tot-seq=3

TTAGGAATATTCGTCAATGGGGGAAACCCTGAACGAGCAATGTCGCGTGAGTGAAGAAGG

TCCTTTGGATCGTAAAACTCTGTTGTATGGGAAGAACAGCCAAATTAGGAAATGAATTTG

GTCTGACGGTACCATGCCAGAAAGCTCCGGCAAACTATGTG

>3051 len=161 tot-seq=3

TAGGGAATCTTCCACAATGGGCGCGAGCCTGATGGAGCAACGCCGCGTGAGTGATGACGG

TCTTCGGATTGTAAAGCTCTGTTAATCGGGACGAATGGTTTGTGTGCAAATAGTGCATAG

ACATGACGGTACCGGAATAGAAAGCCACGGCTAACTACGTG

>3052 len=161 tot-seq=3

TAGGGAATTTTCGGCAATGGGGGAAACCCTGACCGAGCAACGCCGCGTGAGTGATGACGG

CCTTCGGGTTGTAAAGCTCTGTTGTTAGAGAAGAACGTTGGTAGGAGTGGAAAATCTACC

AAGTGACGGTAACTAACCAGAAAGGGACGGCTAACTACGTG

>3053 len=161 tot-seq=3

TAGGGAATTTTCCACAATGGGCGAAAGCCTGATGGAGCAACGCCGCGTGAACGATGAAGG

TCTTCGGATTGTAAAGTTCTGTTGTCAGGGACGAACAAGTGCCGTTTGAACAAGGCGGCA

CCTTGACGGTACCTGACGAGAAAGCCACGGCTAACTACGTG

>3054 len=161 tot-seq=3

TGAGGAATATTGGTCAATGGACGCAGGTCTGAACCAGCCAAGTAGCGTGAGTGAAGAAGG

TTTTCGGATCGTAAAGCTCTGTTGTAAGAGAAGAACGAGTGTGAGAGTGGAAAGTTCACA

CTGTGACGGTATCTTATCAGAAAGGGACGGCTAACTACGTG

>3055 len=161 tot-seq=3

TAGGGAATCTTCCGCAATGGACGCAAGTCTGACGGAGCAACGCCGCGTGAGTGAAGAAGG

ATTTCGGTTCGTAAAACTCTGTTGTTAGAGAAGAACGAGTGTGAGAATGGAAAGTTCACG

CTGTGACGGTACTTAACCAGAAAGGGACGGCTAACTACGTG

>3056 len=161 tot-seq=3

TGGGGAATATTGGACAATGGGGGGAACCCTGATCCAGCCATGCCGCGTGTGTGAAGAAGG

CCTTTTGGTTGTAAAGCACTTTAAGCGAGGAGGAGGCTCCTGTAGATAATACCTACAGTG

AGTGGACGTTACTCGCAGAATAAGCACCGGCTAACTCTGTG

>3057 len=161 tot-seq=3

TAGGGAATCTTCCGCAATGGGCGCAAGCCTGACGGAGCAACGCCGCGTGAGCGAGGACGG

CCTTCGGGTTGTAAAGCTCTGTTCTTCGGGAAGAAGGGCGGGAGGGTGAACAGCTCTTCC

GCTTGACGGTACCGAAGGAGAAAGCCCCGGCTAACTACGTG

>3058 len=161 tot-seq=3

TAGGGAATCTTCCGCAATGGACGAAAGTCTGACGGAGCGACGCCGCGTGAGCGAAGAAGG

TCTTCAGATCGTAAAGCTCTGTTGTTAGGGAAGAAGAAGCGCCGTTCGAAGAGGGCGGCG

CGGTGACGGTACCTAACGAGAAAGCCCCGGCTAACTACATG

>3059 len=161 tot-seq=3

TGGGGAATATTGCACAATGGGGGAAACCCTGATGCAGCGACGCCGCGTGAGCGAAGAAGG

CTTTCGAGTCGTAAAGCTCTGTCCTATGAGAAGAACGAGTGTGAGAGTGGAAAGTTCACA

CTGTGACGGTATCTTACCAGAAAGGGACGGCTAACTACGTG

>3060 len=161 tot-seq=3

TGGGGAATATTGCACAATGGAGGGGACTCTGATGCAGCGACGCCGCGTGAGTGAAGAAGG

TTTTCGGATCGTAAAGCTCTGTTGTAAGAGAAGAACGAGTGTGAGAGTGGAAAGTTCACA

CTGTGACGGTATCTTACCAGAAAGGGACGGCTAACTACGTG

>3061 len=161 tot-seq=3

TGGGGAATATTGCACAATGGAGGGAACTCTGATGCAGCGACGCCGCGTGAACGAAGAAGG

CTTTCGAGTCGTAAAGCTCTGTTGTTAGAGAAGAACGTTGGTAGGAGTGGAAAATCTACC

AAGTGACGGTAACTAACCAGAAAGGGACGGCTAACTACGTG

>3062 len=161 tot-seq=3

TGGGGAATCTTGCACAATGGGCGAAAGCCTGATGCAGCGACGCCGCGTGCGGGATGAAGG

CCTTCGGGTTGTAAAACTCTGTGATTCGGGACGAAAGATAAGCAGACGAATAATCTGCAT

AAGTGACGGTACCGAAAAAGCATGCCACGGCTAACTACGTG

>3063 len=161 tot-seq=3

TGGGGAATCTTCCGCAATGGACGAAAGTCTGACGGAGCAACGCCGCGTGAACGATGACGG

CCTTCGGGTTGTAAAACTCTGTTGTCAGGGACGAATGTACTAATCTATAATACATTTCGG

CATCGACGGTACCTGACGAGGAAGCCACGGCTAACTACGTG

>3064 len=161 tot-seq=3

TGAGGAATATTGCGCAATGGGGGAAACCCTGACGGAGCAACGCCGCGTGAGTGAAGACGG

CCTTCGGGTTGTAAAACTCTGTGATTCGGGACGAAAGATAAGCAGACGAATAATCTGCAT

AAGTGACGGTACCGAAAAAGCAAGCCACGGCTAACTACGTG

>3065 len=161 tot-seq=3

TGGGGAATATTGCACAATGGGCGCAAGCCTGATGCAGCGACGCCGCGTGAGGGATGGAGG

CCTTCGGGTTGTAAACCTCTGTTGGTAGAGAAGAACGTTGGTGAGAGTGGAAAGCTCATC

AAGTGACGGTAACTACCCAGAAAGGGACGGCTAACTACGTG

>3066 len=161 tot-seq=3

TAGGGAATCTTCCGCAATGGACGAAAGTCTGACGGAGCAACGCCGCGTGAGTGATGAAGG

CCTTCGGGTCGTAAAGCTCTGTTGTTAGGGAAGAACAAGTGCCGTTTGAATAAGGCGGCA

CCTTGACGGTACCTAACCAGGAAGCCACGGCTAACTACGTG

>3067 len=161 tot-seq=3

TGGGGAATATTGCGCAATGGGGGAAACCCTGACGCAGCGACGCCGCGTGCGGGATGAAGG

CCTTCGGATCGTAAAGCTCTGTTGTAAGTCAAGAACGAGTGTGAGAGTGGAAAGTTCACA

CTGTGACGGTAGCTTACCAGAAAGGGACGGCTAACTACGTG

>3068 len=161 tot-seq=3

TAGGGAATCTTCCACAATGGACGAAAGTCTGATGGAGCAACGCCGCGTGAGTGAAGAAGG

GTTTCGGCTCGTAAAGCTCTGTTGGTAGAGAAGAACGTTGGTGAGAGTGGAAAGCTCATC

AAGTGACGGTAACTACCCAGAAAGGGACGGCTAACTACGTG

>3069 len=161 tot-seq=3

TAAAAAATCTTCCGCAATGGACAAAAGTCTAACGGAGCAACGCCGCGTGAACGATGACGG

CCTTCGGGTTGTAAAGTTCTGTTATACGGGACGAATGGCGTAGCGGTCAATACCCGTTAC

GAGTGACGGTACCGTAAGAGAAAGCCACGGCTAACTACGTG

>3070 len=161 tot-seq=3

TGAGGAATATTGGTCAATGGACGCAAGTCTGACGGAGCAACGCCGCGTGAGTGATGACGG

CCTTCGGGTTGTAAAGCTCTGTTAATCGGGACGAATGGTCTTTATGTGAATAATGTAAAG

ATTTGACGGTACCGGAATAGAAAGCCACGGCTAACTACGTG

>3071 len=161 tot-seq=3

TGAGGAATATTGGTCAATGGGCGAGAGCCTGAACCAGCCAAGTAGCGTGCAGGATGACGG

CCCTATGGGTTGTAAACTGCTTTTGTCAGGGAAGAAAAGGTCTGTGTTAATACCATAGAC

TGCTGACGGTACCTGAAGAATAAGCACCGGCTAACTACGTG

>3072 len=161 tot-seq=3

TGGGGAATATTGGACAATGGGCGGAAGCCTGATCCAGCCATGCCGCGTGTGTGAAGAAGG

CCTTTTGGTTGTAAAGCACTTTAAGTGGGGAGGAGGCTTACCTGGTTAATACCTGGGATA

AGTGGACGTTACCCACAGAATAAGCACCGGCTAACTCTGTG

>3073 len=161 tot-seq=3

TGGGGAATATTGCACAATGGGCGAAAGCCTGATGCAGCGACGCCGCGTGAGCGAAGAAGG

TTTTCGGATCGTAAAGCTCTGTTGTAAGTCAAGAACGAGTGTGAGAGTGGAAAGTTCACA

CTGTGACGGTAGCTTACCAGAAAGGGACGGCTAACTACGTG

>3074 len=161 tot-seq=3

AGGGAAAACCTCTCAGGACCCAGAAGTAGTGTTATGAAATCTCTTGTGAGGGACAAACAT

TCAGAACCTTGTAGCAGTGTTCTGGAATCCTTTCTGAAGAGAAACATTCAGACCCTCATA

GCAGTGTTCTGGAATTCTATGTTAGGGACAAACACTCACCC

>3075 len=161 tot-seq=3

TGGGGAATATTGGGCAATGGGCGCAAGCCTGACCCAGCAACGCCGCGTGAGTGAAGAAGG

TTTTCGGATCGTAAAGCTCTGTTGTAAGTCAAGAACGGGTGTGAGAGTGGAAAGTTCACA

CTGTGACGGTAGCTTACCAGAAAGGGACGGCTAACTACGTG

>3076 len=161 tot-seq=3

TGAGGAATATTGGTCAATGGACGCAAGTCTGACGGAGCAACGCCGCGTGAGTGAAGACGG

CCTTCGGGTTGTAAAACTCTGTGATTCGGGACGAAAGATAAGTAGACGAATAATCTGCAT

AAGTGACGGTACCGAAAAAGCAAGCCACGGCTAACTACGTG

>3077 len=161 tot-seq=3

TGGGGAATATTGCACAATGGGCGCAAGCCTGATGCAGCCATGCCGCGTGAACGATGAAGG

TCTTATAGATTGTAAAGTTCTTTTATATGGGAAGAAACGCTAAAATAGGAAATGATTTTA

GTTTGACTGTACCATTTGAATAAGTATCGGCTAACTATGTG

>3078 len=161 tot-seq=3

TGGGGAATATTGGACAATGGGCGAAAGCCTGATCCAGCCATGCCGCGTGTGTGAAGAAGG

TCTTCGGATTGTAAAACTCTGTTATACGGGACGAAAAAGACGGATGCCAACAGTATCCGT

CCGTGACGGTACCGTAAGAGAAAGCCACGGCTAACTACGTG

>3079 len=161 tot-seq=3

TCGAGGATCTTCCGCAATGGGCGCAAGCCTGACGGAGCGACGCCGCGTGTGGGATGAAGG

CCTTCGGGTTGTAAACCACTGTCACTCGTTAAGAAAGGGTCCGGCGTTAACAGCGTCGGG

CACTGACGAAGGCGAGAGAGGAAGCCACGGCTAACTTCGTG

>3080 len=161 tot-seq=3

TAGGGAATCTTCGGCAATGGGGGCAACCCTGACCGAGCAACGCCGCGTGAGTGAAGAAGG

TTTTCGGATCGTAAAACTCTGTTGGTAGAGAAGAACGTTGGTGAGAGTGGAAAGCTCATC

AAGTGACGGTAACTACCCAGAAAGGGACGGCTAACTACGTG

>3081 len=161 tot-seq=3

TGAGGAATATTGGTCAATGGACGAGAGTCTGACGGAGCAACGCCGCGTGAGTGAAGAAGG

TCTTCGGATTGTAAAACTCTGTTATACGGGACGAAAAAGGCGGATGCCAACAGTATCCGT

CCGTGACGGTACCGTAAGAGAAAGCCACGGCTAACTACGTG

>3082 len=163 tot-seq=3

CCCAGTGAGCACACACGTGTAGCTCAGAAGATAGCAGTGCTTCCAGGCTTCCAAGTCTGC

ACTCAATCACTAACAAAAAGGCAGAGGGTCTAGAGAACAGGGCCACAATTCCTAATTAAG

TCGTTTTCATTACAACAATACTTAAAGTATTGTTACTACCCTC

>3083 len=163 tot-seq=3

TCGCTTGCCTGGTTCTGGGATATGAGTGTGTCCAGGATGTGTGGTCTCATCTTCTCCCTG

CCTCTCCGCAGTTGTGCTGGTACGGGAGTGTCTCTGCAGTGGGCCTGTTAATTGGTGCTT

CAGCTGGCTGATTGACTCTAATGCGGGTGAACATTTATTTACC

>3084 len=163 tot-seq=3

CACATCAAAAAGTTAATACACCGTGATAAAGCAGGCTTTATTCCTGGGATTCAAGGCTGG

TTCAACATATGCAAGTCAATAAATGTATTCACCATATAAACGGAATTAAATGCAAAAAGC

TTATGATCATCTCAATAGACATAGAAAAAACTTTGAATAAAAT

>3085 len=164 tot-seq=3

CCACCTGCCATTTGTCCACTGGGTTCATGCCTACCCCATGACTGCAGGCTTCTTCATCCT

GTCTTATCCGGCACAAGTGAGCAGGAAGCAGACCCAACCGAGAGGATTCCAAGTTACATC

ACCCTGTGCTCTGCCTATATTGCTGGTCTTTGCTCTCAGAAGTT

>3086 len=164 tot-seq=3

AGGTTAGGAATATTCGTCAATGGGGGAAACCCTGAACGAGCAATGCCGCGTGAATGAAGA

AGGCCCTATGGGTTGTAAAATTCTGTTGTATGGAAAGAAATGTAAAGTTAGGAAATGAAC

TTTACTTGACGGTACCATTCAAGAAAGCCACGGCTAACTACGTG

>3087 len=164 tot-seq=3

CGCTGCAATGATGCCAACGAAGAAGGTCATGGACGCAAAGCGCAAGCCACAAGAGGTGCC

GGAAGGGCTGCAGGCAGGCGTGCGGATGGCGGACGAAAGAATGGAAAAAGCGTGAAACGC

CCCTCTTATCAGCAGCAAGATACCGGCGACTGGCGACAGTTCTT

>3088 len=165 tot-seq=3

AAAAAAAGTCTACTCAGGAGCTGAATTTAATAAAATGAGCACTTTTGACTGCTTTGTGAA

GCGTGGCATTTTTTTTTTTGTTTTTTTGCTGTGAGTTCATGGTAGTAAAGAACATGAGTG

ACATGACTGCGCAGTCTGGCCCGAGTTCATGCCATTGTGGTTGCC

>3089 len=166 tot-seq=3

CTATTATAACTGTTCCATTCTTTCAACCTTATCTGTGTGGTTTGAAGTCTGTTCCAAGTA

AGTGTAGTTGAGGCACCAGCCAAGGATTAGGGAAGAGTTTGTGTGCAGAATTTAGGACTC

TCCCTTTCTGAAAATCTCCTTCATGTAATTTCTCCTCTCTCACTTC

>3090 len=166 tot-seq=3

AAGAAGTAGGGAATATTAAAAAATGGAAGAAAGTATGATGGAGAAACGAAGAGTGAGTGA

AGAAGGGTTTCGGCTCGTAAAGCTCTGTTGTTGGTGAAGAAGGACAGGGGTAGTAACTGA

CCTTTGTTTGACGGTAATCAATTAGAAAGTCACGGCTAACTACGTG

>3091 len=167 tot-seq=3

TTTCCCGCCAGTCCTGTGTGTTTCCAGACTAGACTTTCCCTTCTCACCTCTCTACATAGT

CATTCATTGATTAGAGGCAAAGTGTTGACTTCCAAGAAAACTGAAAGTGTACCCAGGATA

AAGGTGATGAAGTTGATGCAGGGTTCAGAGAGGCTCCTGTGACTGAA

>3092 len=167 tot-seq=3

AAGCCGATAACGTAGATGTCGCTTTCGCCCGTATCTAAATTACGGATGACGTTCAAGCCA

TTCAGCATGATCGTCACGGTAATGATGGCGAATACTCCCCCAATAACCGGTGCGGGAATC

GCTGCCAGAAACGCTGACAACTTAGTAAAGAAGCCGAGGAGAATAAA

>3093 len=167 tot-seq=3

TGGGGAATTTTGCACAATGGGCGGAAGCCTGATGCAGCGACGCCGCGTGAGTGAAGAAGG

GTTTCGGCTCGTAAAGCACTGTTGGTAGTGAAGAAAGATAGAGGTAGTAACTGGCCTTTA

TTTGACGGTAATTACTTAGAAAGTCACGGCTAACTACGTGCCAGCAG

>3094 len=168 tot-seq=3

GTGGAGGACGACTCGGTGCAGCAGCCACTGTGGAGACGACCTGGTGGCTCCTCAAAGACA

CGCACAGATCTCCCATCCGACCCAGCACGACCACTCCCAGCTATGTATCCAGGTGGAGTG

GAAGCCCGCAGCCACGCAGAAGCCCGTGCGCAGACGCCGCCGTGGACT

>3095 len=168 tot-seq=3

GTGTGCTCTTCCGATCTCCTCCGGGAGGCAGCAGTGGGGAATATTGGACAATGGGCGCAA

GCCTGATCCAGCCATGCCGCGTGAGTGATGGAGGCCTTAGGGTTGTAAAGCTCTTTCAGT

AGGGAAGATAATGACGGTACCTACAGAAGAAGCCCCGGCTAACTTCGT

>3096 len=168 tot-seq=3

GGATGCCTCTCAGGTGCAGCGTAGCCTTGACCTACTATGCAGGGCGAAGCATCCTGGTGG

AGCCACACAGCTCCGGGTTCTGGTGGCCCTGCTCGGGCGAGGCAGCTCCGTCAGCTGAGG

GCCATTCTTCTGAGAAGGGGCAGCTGTGTGCTTTAGCAGCTGACATTC

>3097 len=168 tot-seq=3

TATAGCCCTGTACACTATGGTGACTAACCAGAAATGGGCATTCTGTAAATGCTAGATACA

AAATAAATAAATAAGGAGGCATTTTTTTTCTAGCTTCAGATAGATTGATAGCCTGTAACT

GGGAGAATATGGGCTTGGCAGAATCCTACCTGCACCAGAAGTGAACAC

>3098 len=168 tot-seq=3

TTGGCTGGACTCACGGGACAGTGACAGGCAGTTGACATGAGGCCCCTGACAGTCTGTCCT

GAGCCGCAGCCAGATTAATGTTGCTGAAAACGTACCTTCCCATAGCACAACCCATCACCC

TCACAACCGCCCTGCAAGCTCCCCTGAACAGTGTGACCTCCTGTCCAT

>3099 len=168 tot-seq=3

TCGTGTATGGGGAATTTTGCGCAATGGGCGAAAGCCTGACGCAGCAACGCCGCGTGGAGG

ATGAAGGTTCTCTGAATCGTAAACTCCTGTCGGCCGGGAAGAATAGACGGAGTGCTAATC

AACTCCGGCTTGACTGTACCGGCAAAGGAAGCCCCGGCTAACTCCGTG

>3100 len=169 tot-seq=3

AAGGCGGGCACCAAAGGATAGGCACCCGGAAGGTGGACTCCGAGGAGGAGAGAGGACAGG

GGTCTCTCACCCCAGCTCCTGGTCACCATGCTGCTGGCTGTGCTGCTGCTGCTACCCCTC

CCAAGCTCATGGTTTGCCCACGGGCACCCACTGTACACGCGCCTGCCCC

>3101 len=170 tot-seq=3

GTGGAGAGTTGGGTAGGGAGATCTGTGAGGGAGGGGCTTGTGCAGGAAGGAGTTTAGGTT

TTACTGATTGAAGTGGGAAGCCATTAGAAAGATCGCCCTGGTGCTTTGTGGAGAACAGTT

CACAGCAAGAAAGCAGGGAGACAGGTTAGGGGCTCTCGCAGCGATTTAGG

>3102 len=171 tot-seq=3

TTTTTTTTTTCAAGCAGAAGACGGCATACGAGATAACACTGTGACTGGAGTTCAGACGTG

TGTCTTCCGATCTCCTACGGGAGGCAGCGGCCAACGGCTTGACTTCTCTGGCCACTGAGG

CCACAACTGGGCACTCCCTGCTTTCTCTCCATCTCTCACATCCACGGTCCC

>3103 len=171 tot-seq=3

TTTCCTTGACCCAGAGAGAGTTGTCTGGAGAGGGCAGCCTGCCAGCAGCAGTGGCCAGGG

GATTCAGTCACCGCTGGCCCACAGGGGAGGGGAATGGGGTGATAAATAGCCCATCACTCT

CTCCCTTCGCCCTCTGATCTCTTGCTAGTGCATCTAATTTGCCACCTCTAA

>3104 len=172 tot-seq=3

GCTGGAAGAAATCTGCTGTTTGAGAAGTGGAGGAAACGGGCTGGAGAGGAGAGATAACAG

CCCGTTCTCTGTGTATCTGCATGCGTCTGCCGGCGAGGGTGGCTGGAGCCGGGCGGTCTG

AGGCCATATCTAGCGAGATTTTTGTTCTGGGTTGTGAAAGTGACCTATGGAG

>3105 len=172 tot-seq=3

GATGAGTGTGAGGCAGTGGAGGCACTGGAATCCAGGCAGGAGAAGGGAGAGGCAGAGGAA

GGCTCAGGTCCAAGTTGGTCTATTTTCTCAATCTACCACCAACCAGCTCTTTCTGCAACT

ATGGTGAGGTTATTCTAATCCATTTTCTTCCATGACCCATTAAAGGTAGCCA

>3106 len=172 tot-seq=3

GGCAGCCGGCTTTGGACAACTGACTCTCTGGGAGGACTTGGAGTCCCTTCCTATGAGTGG

GTCCTTCCCTCTCAGGCTGGCAGGGAGTGCCCCAACTTCTCCCCTTTGTCACTCTTCCCA

CAGGGTGTGTCTGGGCAGGCAGAGGGTGTTGTGGCTCAAGCACTTGGTTGGA

>3107 len=173 tot-seq=3

ATGATTATAAAAGTGTGGTTGTTGTCCAGCTCAAGAAGAGGGAGGATCTAATTCTACACA

ATAAAATAAAACTGGGGAGGAACAGCGAGGAAAGCCCCAGGAAGTAATGGAATTGGAAAA

AGAGCTGATATACGCACTCGGATTTGCTGAGTAAGAAAAACATCACGAGGTAG

>3108 len=174 tot-seq=3

CTCTCAGTGACCTGTTTGTCCACCACCAGTGTCAGCCACCTGGCAGTCACCTGAAGCCTC

CCTCACCCTCCAGTCCTAGTGAAGCTCCCCAGGTTCCTCAATCACCTGTTGCCTGGGCTT

TGTAAGTTTCTTAATCTCCCTCCTTCTAGTTTTGTCCCTACAATCCTGTTCCAC

>3109 len=174 tot-seq=3

CCCCAGGGGGCCGTGCAGAGCCGGGGGTTGCCTCAAGCCTGACTCAGCAGGGGAGCGGAT

GGGAGACTGGGCAGGGGCCCGTGGGGGGCTCGGTTTGGGGCCCCTGACCCATTCCCTCAG

GCTAAGCGAGCTACACTCCACCCCGGTCACTGACCACATGGCTCTCCTACCAGC

>3110 len=175 tot-seq=3

GGAGGGAGACATGTGTCGGGGCCTGGAGGGCGACAGTTCCTCTGCATCCCATGCCAAAGG

AAGAGCAGGAAGACCCAGGAGATGATAAGCTTGGAGCCAACTGTGGTCATCACTGGGAGG

GGCAAAGCGAGCAACTGAGAGCCCCAAAGAGACAGGGGTCTTATTGTCACTACCT

>3111 len=175 tot-seq=3

TGAGGGTGGAGGGGCATGGACTTGGTGTCCAGCCAGGATCCACTGGCCTCAGCCTCTGGA

AAACGGGGTATCATGAGCTTTAGCACAGGTCATAAATAGGGTGGGGGTCCCTGGAAGAGG

ATCTGAGGACCATCCCTGGGGATTTCGTCCAGCCACCATCCAGCTGCCATGACTC

>3112 len=175 tot-seq=3

ACGGACTGAGGGTGGGGCCACCAGGGGACTTGCTCACTTCCAGGGGGGCCAGGCGGTCAG

TGGGCCACTGCAGCATGGTCAGCACTGACGGAGTCCTTGATAGTGGGGAGGGGTCTCCAA

TCGGCAGGGAGGGCTCTCCAATCAGCAGAGAGGGGCTCCCATGACCTCTGTCCAA

>3113 len=175 tot-seq=3

ACACATTAACAGATCAACAATCGTGATCCTTCATCTGGACGCTTTCCTTCTGCACTCTTC

CCCAGTCACCCGTCCTGGTTTTCCTATCAGCTGCCAAAGGCACAGCTTTGATGTTAAGAT

TAAGATTGCAGCGAAGGAAGATGGGAAGGAATCTTTGCTGGGAGCTCGCAGTCAG

>3114 len=176 tot-seq=3

AATGGGGGAGGATGGGAAGGGATTCTAACATGTCAACAAATGGCAGTTTTCTCAGAATAC

TGGATGACTTAGCATAAAACTGGAGTGAAGACTGTGCTTCTGAAATGTTGAACATTGTTT

AATAATTTGGCACTGTACGTTGAGAGGTTTCTGTCAACACTGTCAACATCCAGACT

>3115 len=176 tot-seq=3

AGATCTGGGAGAGGTGGCAGGGAGGGGATGTCACCCTGTGTCTTAGTCCTCAAAGAAGGC

TTCGCAGAGGTGGTGTGGAAAGAGGCCGTCAGCTCTGGCCCAGTGGCCGTGGGCATGCGC

CTTTCCTCCTCTGAGCTCAGTGTCCCTTTTATGATGGGCCCACCAGCACCTGCCGT

>3116 len=176 tot-seq=3

GCAACTGGGAAGTGTCTGGTGCTGTCACCTCCCTGGTTGTGAAAACAGCAGCATACTCAG

GTTGTGCATCATCGTGTACACTGCACGGGATAAAAGGGCTCCTACCCTGGGCTTCTCATC

CACAGCATCCTTAGGAGCAGCCTTCTTCTCCTACCTCCTCTGCATCTGGTGAGTGG

>3117 len=176 tot-seq=3

GTAGTTTGTTTGTTTGTTTGTTTTAGTTTGTTTCTGTTACACTGTTTTTTGGTTTCTAGC

ATTAGATTACCAGAATTTCCATCTCTGTGCTTACATTATCCATCTGTTTCTTGCATGTTG

CCTACTTTTCCCATTTGAGCCCTTAACAATGATTTTTGAATTCCCTGTCTGGTAAA

>3118 len=177 tot-seq=3

AGCCAGCTATAGACACCATCCCCCCAGCAGGGCCCCAATGTCTCCACTAGGTGGTGTCTC

CTCACTTCCTCCATGGCCTACAAACCTCCCTGCTAATGCACACAGTGGCTGGGCCATGGG

TTGGCAGTGGCTGACTGGTCAGCAGGTACGTGCCTGAGTGAGGCTGGCCTGTCAGCC

>3119 len=177 tot-seq=3

TGAAAGGGCGGCCAGGGAGGGCAGGTGCCACATGTACCTGAACCCATGCTCCTCCCTGAA

GCGCTGTTCATCTGTGCAATGGTAGGTATAAGGGGGCAGGGACCAGGGACCTACCTGCTA

CACTGTCCTTGGTCACCAAGGCAGGGCCCAGAGGGCAGAAGGTGTCGAAGGTTTTTC

>3120 len=177 tot-seq=3

CGGGGGCGCGGCCGAGCGCCGACGGGAGGCAGCAGGGGGGAAGAGGGCGCAAGGGGGGAA

ACCCGGACGCAGCGACGCCGCGTGCGGGATGAAGGCCTTCGGGTTGTAAACCGCGTTTGA

TTGGGAGCAATCCTTCGGGTGAGTGTACCTTGCGAATAAGCGCCGGCTAACTACGTG

>3121 len=177 tot-seq=3

TCAAAGGGCAGCCAGGGAGGGCAGGTGCCACATGTACCTGAACCCATGCTCCTCCCTGGA

GGGCTGTTCATCTGTGCAATGGTAGGTACCAGGGGGCAGGGACCAGGGACCTACCTGCTA

CACTGTCCTTGGTCACCAAGGCAGGGCCCAGAGGGCAGAAGGTGTCGAAGGTTTTTC

>3122 len=179 tot-seq=3

GGAAGAGATAGTGACATTGAGACTTAGTATAGCAGTTGTTAATACATTATCATTGCAGTG

CAGGTCATCATTACAGCCTGACTTTCAACTGCTAGAGGGCTTTCCTTGGGAGCTACAGCC

TGTTCTGCTTCTGTGCCAGGCAGGCCAGCAGTGCTGGAGAATTAGTGCCACTCACATCC

>3123 len=179 tot-seq=3

AGTCCCACTCTTATGTATTTACCTAGAGAAATGAAAGCACATGTTTACACAAAGAGCTGC

CATAAATGTTCACAGCAGCTTTATTTGTAATAGCTCCAAATTGGAAACAATGCTGATGTC

CAGCAGCAGGTGAATGGGTAAACTAACTTGGTAATCCTACAATGGGATACTACACCCAG

>3124 len=180 tot-seq=3

TGGGGAATATTGCACAATGGGCGCAAGCCTGATGCAGCCATGCCGCGTGAGCGATGAAGT

ATTTCGGTATGTAAAGCTCTATCAGCAGGGAAGAAAATGACGGTACCTGACTAAGAAGCA

CCGGCTAAATACGTGCCAGCAGCCGCGGATTGACAGATCGGAAGAGCGTCGTGTAGGAAA

>3125 len=180 tot-seq=3

GGGACTGTGCAACTGTGCAGCAGGTCTGTGTCCTATCCCCGTCCACATTGGAAGCTCTCA

TAGGGCAAGGCCTGGGAACTGAGCAGTTCTCTGCATGCTCCTTTTGAGAGCATCCCAACT

TCTGTAGGGTCTTGATCTTCATCCATAATGATGACTGGGTTCATTTACCTCACCTTACTC

>3126 len=181 tot-seq=3

TGAGGAATATTGGTCAATGGACGAGAGTCTGACCGAGCAACGCCGCGTGAGTGAAGAAGG

TTTTCGGATCGTAAAACTCTGTTGTTAGAGAAGAACAAGGATGAGAGTAACTGTTCATCC

CTTGACGGTATCTAACCAGAAAGCCACGGCTAACTACGTGCCAGCAGCCGCGGAATACGA

A

>3127 len=181 tot-seq=3

GCAGATAACAATTACCTTGTCTCCTTGGAAGCCAGGATCTCCTGACACACCTGGCACCCC

TTGTTCACCCTGAGTACACACCAGCAAAGAGATCATAAGGAGAGAGCCAGCAACTTGCAA

GCTTCCCCCCTCACCCCCGAACAGAGGACCCCATGGAGCCTCATGGAGGCTGCTCTTGTG

G

>3128 len=181 tot-seq=3

AGCTAACTGCTTTAATTGGGCCTTCGGGGTGTGGAAAGTCAACTTTCTTGCGCTGTTTAA

ATAGGATGAATGATGATATTCCAAACATTCATATTAGCGGTGATATTCAGTTTGAAGGAC
AAAACATTTATGGTTCTAAGGTGGATTTGGTAGAATTACGTAAAGAAGTTGGGATGGTAT
T
>3129 len=182 tot-seq=3
TGGGGAATATTGCACAATGGGGGAAACCCTGATGCAGCGACGCCGCGTGAGCGAAGAAGG
CCTTCGGGTCGTAAAGCTCTTTTATATGGGAAGATAATGACGGTACCATAAGAAAAAGCC
CCGGCTAACTACGCGCCAGCAGCCGGGTAATGCCAAGATCGGAAGAGCGTCGTGTTGGGG
AA
>3130 len=183 tot-seq=3
TGGGAATATTGCACAATGGGGGAAACCCTGATGCAGCGACGCCGCGTGGAGGAAGAAGGT
CTTCGGATTGTAAACTCCTGTTGTTGAGGAAGATAATGACGGTACTCAACAAGGAAGTGA
CGGCTAACTACGTGCCAGCAGCCGCGGTATATCGAGATCGGAAGAGCGTCGTGTATGGGA
ATA
>3131 len=183 tot-seq=3
GAAACAGCCCTTGGACTCTGACCATGTGTTCTTTGAACAAAAAAGTCGGGATCATAACTC
TCTGAGTATAACTCGAACAGCTTGTGAAAGGATGGGAAATCCAACATAAGTGGGCTGGTA
GAACCAGAAGGTGGAACCACAGTCGGGTGGTTCAGGCACAGAGGCTTCCCAGGGTGTCAC
AAG
>3132 len=184 tot-seq=3
CCCCAGCTCCTGTGCCCTTCCCCCTCCAGGCTGGCTTCTTCAAGCGGAATCGACCGCCTC
TGGAGGAAGATGAAGAGGAAGAGTGACAGCAGAGGGGGCGGGGTTCCTGGTGGGAAAGCT
GGGTACCTGCTTCCTGTTTCCAGCCAGTTGCCTTTGCTCAGAACTCCATGGGGGTCTTCC
CTTT
>3133 len=184 tot-seq=3
GGGGAGGCAACGAGTGTGCTTTTCAGTACATTAGGACGTCTATTAGTCATCCAAGTGGAG
ACGCAGATGCAATGCTTAAGAAATGAGTCAAAGAACTTTAGAAACAGAAGCGGCTTTAGA
GAGCGCCAACCCAGGAGTCCCTGCCCGGCAGAAAACGAGGGGATGGTGGACCTCGAAGCC
TCTG
>3134 len=184 tot-seq=3

AGTGAGGCGAGATCATGCCACTGCACTCCAGCCTGGGCAACAAGAGCAAAACTCCTTCTC

AAAAAAAAAAAAAAAATCAGATAATGATCATAGTATCTGGTTTTAACATCATATCAAGGA

AAGTGGCACTGAAGAGGGTAGGAAGGACAGTCTTGCACTGCCCATACCATCCCTCACCCA

GCCC

>3135 len=185 tot-seq=3

CTGGAGTTCAGACGCGTGCTCTTCCGATCTCCTACGGGAGGCCGCAGTGGGGGATATTGC

ACAATGGGGGAAACCCTGATGCAGCAACGCCGCGTGAGGGATGAAGGTTTTCGGATTGTA

AACCTCTGTCCTTAGGGAAGAAAAAAATGACGGTACCTAAGGAGGAAGCTCCGGCTAACT

ACATG

>3136 len=185 tot-seq=3

CAGTATTCTGGAATCCTATGTGAGGGAGAAACATTCAGAACCCAGTCACTGTGTGCTGGA

ATCCTATGTGAGGGAGAAACACTCACAACTCAGTCACTGTGTGCTGGAATCCTATCTGAG

GGACAAACATTCAGACATGTGTAGAAGTGTCCTGGAATCCTATGTGAGGGACAAACACTG

AGAAC

>3137 len=185 tot-seq=3

GATGCCGATGGTAGCCCCAATTGTGTAGCCGATGATCAACTGCTGAATCGTGACCACCAG

GTGCGGCAAGATCGTGGGGAAGTTCGTTACAAGCGCGCTCGCGATGGCGCTGGGCGCGGG

AAAGACGTACGAAGGCACCTCGAAGAAACGCACCAGAAACTCGAGCCCCAGGATGATGAC

GACGG

>3138 len=186 tot-seq=3

TCTGTTTTGAGGTAGAGGTGATGTTATGTGGACATGAGAGGAGGAGAGGAGTCAGGGATG

ACACTGGGGCATTTGTCCTGAGCAGCTGTGTCCTGGAGGAGCCCAACCGGGGGATGCAGG

AGCAAGAGGTGGGTTTTGGCCAGTGTGTCGGAGAGGCCCGTGAGCCTCCCAGGGGGAGAT

GCTGAG

>3139 len=188 tot-seq=3

CACAGGATGGGCCACTTCAATGATCTCAAATGACAAAAGGAACTCACATGCCCTGGGTTC

AGACTCCGCCCCCATCGCTTACGTCATTTCCGCCCATCTGACAGCAGTCTGTGAGGTCGG

GGCTGTTGAGTGACTTTTTCAGGGTGGTGTAAAGTAACATCAACCACCCCCCCCAACTGG

GATTCACA

>3140 len=188 tot-seq=3

AAGCCACTGTGTCTAAGTGAGGACACAGGAAGCATTCACGCGTTCACTGAGAACTTGCAT

AAAGCGAATGTGTGGCCCAGAAGTTGACTCCCAGAGAAGACCCTCCTAGTTCCACTCTAA

GCCAGAAATAGCTGCACACATTTGAGAAGACACCACAGCCCCAACACTCTGACCCCCGTG

TACTTTCC

>3141 len=188 tot-seq=3

GGTGAGAAGGATGGAGAGGAGGTCTGGATTGGCAAATGAAAGATGTACAGTGTAGCTTCT

CCTGATCTTTGTTACCAGTTCTGCTTCTAACAGCCTTTTCTGTCACTATTTTTAACTCCA

ACAGCCTCATGAATGGTCTGCTTGCTTGCATGCACTGTTGCCCCTTATAGCACAACCCTT

TTTTTCCC

>3142 len=189 tot-seq=3

GCCAAAGGGGCCTTGATCAAGCTGGTTGCAACCATGGGAATGGAGCTTCACTCCACAGAG

ACTTCAGTGATCCACAGGGTAGACCATGGGCCTCAGAGCTATCCTACCCAAGTACTCCCA

AGTACTATCCTACCCTGGTACTCCCAGCAGCCATTGGTTGAGAGCAGTAGATATCCTACC

CAAGTACTC

>3143 len=190 tot-seq=3

CCCCCAACAGCCCAGCAGCCTCTCAGAGGGTCCTCTGCATGGGCCTGGGATTAGGGCCCC

TGCCCAACACCCCAGCAGCCTCTCAGAGGGTCCTCTGCATGGGCCTGGGATTAGGGCCCC

TGCCCAGCACCCCAGCAGCCTCTCAGAGGGTCCTCTGGACGGGACTGGGATTAGGGCCCC

TCCCTGAACT

>3144 len=192 tot-seq=3

CACAGCAGGCATGTTCTGGAATCCTCTGTGAGGAAGAACCATTCAGACCCTCGTAGCAGT

GTTCTGGAATCCTATATGACAGACGAACATTTAGACCCCAGCAGCAGTGTTCTGGAATCC

TGTGTGAGGGACAAACATTCACACCCTCGGAGCAGTGTTCTGGAATCCTATAAAGGGACA

AACACTCAGAAC

>3145 len=193 tot-seq=3

CAGTGACCGTGGGGCTGGTGGCCTGGGCTGGGGTGTTTGACTCCAGTCAGCCCATCTGCC

AGGCGGGCCCAGTCTCACCACGCCTATTCGCTCTGATTCCTGCCAAGGACCCTGCCTCCC

TCCTTGCAGGCTCAGGAGCCTCCATGGCCCCTGGATGAATCTCCCACTTGCCTAGCAATC

AGAGTCCTCTTCC

>3146 len=194 tot-seq=3

GGTTGGGGGCCGCATCGTTGTCTCCAGGCAAACAAGTCAAAGAATCCCTTTCTGCCTCCT

CATCCCCAGACAGGCTTCCTGAGTACCTTTCATCACACACTAATCCTGGGGATGTGTCCC

GGGAAGATGTGTCCATCTTGTCATCCTCTGATGACTTGGGGCAGGCCTCTGTAGTGTCCG

CTCCAGAGAAGCAG

>3147 len=194 tot-seq=3

CATCCAGAGACATATAGCAAATATTATTAAATCTACATGGAGAGATAGAGTCCAATAAAA

TGATAGTTGAGAACTTCAATATCCTACTCTCAGCATTGGACAGTTCATCTAGACATAAAA

TCAACAAAGAAACATTAGATTTAAGCTGCACTTTGGACCAAATGGACCTAACAGATATTT

TCAGAATATTTCAT

>3148 len=195 tot-seq=3

TGGTGGGTAACTGTTTGCTGGTGTTATCCTGCTGGGTTCCCTCTACAGCCCAAAATATTG

CTTGAAGTGGGTCAGCTGGAAAACAGTGTTCAGAGCCAATTCTTAGAGTCTGGCAGAGAT

TCACAGTAAAGTCAAGCACACCCTCAACACCCCTGTTTTTCTGTCCACCCCTCTTCAGGT

GTTCCCAACACCAGC

>3149 len=195 tot-seq=3

CCTACGGGAGGCAGCAAGAAAGGTTTCCCTATACGCCCCTGTCCGTCCACCGAGGAGATA

TGAAGGCGGGTGTACAACCCGTCACCTCTCCGGCTGCTGAACACAAACCAATGTGAATTG

CTGCTCCAGTTGTGATAGCTTTCCGTATCGTCACTGTTCACCTCCTCCAGGTTACGATAG

GCTCCGGTCTTCAAG

>3150 len=196 tot-seq=3

AAGTGTGATATTAAGATATAATAAGGGAGGAAGAAGTAGGGAATATTAAAAAATGGACGC

AAGTCTGATGGAGCAACGCCGCGTGAGTGAAGAAGGGTTTAGGATAGTAAAGATATGTTG

TGGGGGAAGAAGGAAAGGGGGAGGAAAGGAAATTTGTTTGACGGTAATCAATTAGAAAGT

CACGGCTAACTACATG

>3151 len=196 tot-seq=3

ATGGCTAAGTTACGGATGGCTATGACTTCCATCCTGCTGTCACTCTCCGCCTTACTGGTC

CCCTCTCTTTTGGCCCTCCTGATTGCGTGCTCTGACAACACAAGCTGCCGTGTTGTGTGA

TGCTCTACGGGGCAGCCTCCATGGCAAGGAACCCAGGGAGGCTTCGGTCCAGCGGCCTGC

AAGGAACTGAAACCCA

>3152 len=196 tot-seq=3

GGGTCTTGAGGCTGAGTCAGGCTGATGATCTTGATGATGACTCCAAATCAGGATCAGGGT

CATGATCTTTCTAGGGTTAAGGTCAGTGTCCAAGCTAAGGGGGGAAGACAGTACCAACCA

AAGCGGCTGACATTCAGGAAAAGGCTCCAGGCTCAAAAAGAGCTCAAGAACAGGTTCTCA

AAAGGTAGCCCTTGGA

>3153 len=197 tot-seq=3

TAGGGAATCTTCCACAGTGGACGCAAGTCTGATGGAGCAACGCCGCGTGAGTGAAGAAGG

TTTTCGGATCGTAAAGCTCTGTTGTTGGTGAAGAAGGTTTTCGGATCGTAAAGCTCTGTT

GTTGGTGAAGAAGGATAGAGGTAGTAACTGGCCTTTATTTGACGGTAATCAACCAGAAAG

TCACGGCTAACTACGTG

>3154 len=198 tot-seq=3

CCCCCTATGAGATGCTTCCCTGAAGTTTCTCATCTAATTCTTACAATAACCTTTACGAGG

TAGATGCTGTGTGACCTTGTCCCATATTAAGGGGAACCTGAGCTTGCAGAAGTCAGTGGA

CTCTCCCCAGGGGCCAACTAGTATGAAGCGGAGCCAGGTGTGAAGGACTCACCTACACAT

GCTGTCTGCACAACTGTG

>3155 len=199 tot-seq=3

TCAGAGGTGAAAGCGCTTAGCTCAACTGAGCAACTGCCTTTGAAACTGTCAGTCTTGAAT

GATTGTGAAGTAGTTGGAATGTGTAGTGTAGCGGTGAAATGCTTAGATATTACACAGAAC

ACCGATAGCGAAGGCATATTACTAACAATTTATTGACGCTGATGGACGAAAGCGTGGGGA

GCGAACAGGATTAGAAACC

>3156 len=200 tot-seq=3

TGGGGAATATTGCACAATGGGGGAAACCCTGATGCAGCGACGCCGCGTGGAGGAAGAAGG

TCTTCGGATTGTAAACTCCTGTTGTTGGGGAAGATAATGACGGTACCCAACAAGGAAGTG

ACGGCTAACTACGTGCCAGCAGCCGCGGTATAGAAAGATCGGAAGAGCGTCGTGTAGGGA

AAGAGTGGGCCAATGTGTAG

>3157 len=200 tot-seq=3

CAGCTAGTGGGTGGCCCATCTTTGCAACGAAAACCTGGCATAGCACTGGCCTTTTGAGGC

GTGTTCCCTCTAAGGCAGTGACCTCCAAGGGCGGGGTGAGGACACTTACACACCAGCAGG

AGCCAAGCGAAATGCTTGCTAAGGGAGAGAATGTGCCCATGGCTTCTCCCCTTCCACCAG

CACATGTGGCCAAGAATCTG

>3158 len=200 tot-seq=3

GTTGACTGGGAAGGGCAAATGGAGCAGCTCTGCCAAGGCACTCATTTCCTGTGGAGGTAG

TGTGTAGCTTCAGTTCAGTCCCTAGAGGACATGGTACAGTGGGAATAAGAGGGGTAAATG

GAATAGCTGCATAGCAACTTGGCTCTCAAGGTAAGATGTAGCGGCAGATCAGGGATGGCA

GGGCACCAGGCAGGAGTAGT

>3159 len=200 tot-seq=3

CCCTTAGTTCATAATAAATGCTCAATTCAGTTTTCATAAATGGATGAAAAAGAGGATGGA

TGGAAGAAAGGAAGGATGGATAAAAAAGAAGGAAGGAAGGAAGGAAGGAGGCAAGGAAGG

AAGCAAGGAAGGAGTGACAGTGATGGACCACATTGCTTCTGACTTTCTGATTTCTAAGAA

GGAAGAAAAACAAGGTAATT

>3160 len=201 tot-seq=3

GGGGAGAGAAGGAGGGGAGGGAGGAGAGAGGAAGGACCTGATGTAGGCTTTGAGGTTTCA

AAAACCCTAGTGACCTCTTCCAAAAAGGCCTCATCTATTTCCACAAGGCCCCATGCTGTG

AAACAGTGGACCTTGCCAGGAAACTTGGTAAGGTAGAGTGTGTCTGAGTCCCCAATCCCC

AGGCATCCCCAATGACTATGC

>3161 len=202 tot-seq=3

ACCAGCTAGCACGGTAAGTACTGGAAAAGGGGTTTTTCTCCGCCCCTGCCCTGATCCTGA

TCCCCCAGGAAATAGTAGTAATTCGGGACCAAATTGCCATTCTCACCTTTGCCACGAACT

TGGACTTTCTCTAGGGACCAGTCTAACAAGCAAGGTGACGAAACGAACAGTGTTGGAGAG

GAAGGGACCGCTACACAAGACA

>3162 len=202 tot-seq=3

TGAGGGATATTGCACAATGGGGGAAACCCTGATGCAGCGACGCCGCGTGGGTGATGAAGG

CCTTCGGGTCGTAAAACCCTTTTCCAGGTGACGAGGAAGGACGGTAGCCTGGGAAGAAGT

CTCGGCTAACTACGTGCCAGCAGCCGCAGTAATCTGTAGATCGGAAGAGCGTCGTGTAGG

GAAGAGTGTGCCAATGTGTAGA

>3163 len=202 tot-seq=3

TGGAGTTCAGACGGGGGCGAGGCCGAGAGCCGAAGGGAGGCAGCAGGGAGGAAGAGGGGG
CAAGGGGCGAGAGCCGGAACCAGCCAAGGCGCGGGAAGGAAGACGGCCCGCAAGGGTTGT
AAACTTCTTTTGTATGGGATTAAAGTCACCTACGTGTAGGTGTTTGCAGTTACCATACGA
ATAAGCATCGGCTAACTCCGTG
>3164 len=203 tot-seq=3
TCCCAGAGCACTGCTGAGCCCATGAGCACTTTCTAGGCAGAAAGCTGCATGGAACCTGGG
GACACCAGCTGCTTAAAATCTCCAAATGGAGTGACCATTGCAGTCATCTCCCATCTGCCC
TCTGCTGCTCTCCTCCACTCCGGTCGGCTCTCTAGCATGCAGAAGACAGTGGGCGTCATG
CCGGAGGCTTCCCCTTTCTTGTT
>3165 len=203 tot-seq=3
GTCCCACAGCTGTGTCTCATGTCTTGGGTGTGAGTATGGGAAGCAAGGCTGCTCCAGGGG
CTGTGGGCCCCACTCTCCTCCCCTTCTGCCCGGATGGTGGGGATGGGGGTGGGGACAGAC
CCAGCAGAGAGGGCCCCTCCCGATGGGACATGAAGCCAGATGGCAGTCAGGCTCTGTGGG
CAGCTGCATGTGCCCCCGACGTC
>3166 len=203 tot-seq=3
GAAACCAAGGCACACACTGGTAAAGTGATGCACTCAGGGTAACTTCATAGTTTCTCACGT
GTTAGAAATACTCTGTCCCTTCACTGCCCTGGGTGCCAGGCTTATAAATTTAGCTCCATT
CTACGGGAGCTGGAACTCAGATTGAATGTGTCTATCAATCATCTTTGGGATTCTGAGCCT
CTGGTAGAACTCTAGAGCCTCTC
>3167 len=203 tot-seq=3
CCCGAGCCTCAAGGCAAGTCCAGGACTCAGACCGGGGAGGCCCGGGCCAGCAGCTCCCAG
TGTGACCTGACAAAAACACGTAGGGGCAGGGACGGTCCCCACCCCCAGGGACACAACCCC
TGGTCTTGGACCAGTAGAGGACACGGAGGGTTCAGACCCCTCCTCAGACCCTCCCCACAT
CTGAAACTGCCTCCCCCCAACCA
>3168 len=204 tot-seq=3
TGGGGAATATTGCGCAATGGGGGAAACCCTGACGCAGCAACGCCGCGTGAGTGATGAAGG
CCTTCGGGTCGTAAAGCTCTGTCGGCAGGAACGAGGGTGTTTGTCTCTAATAAAGGCAAG
CATTGACGGTACCTGCAAAGGAAGCACCGGCTAACTCTGTGCCAGCAGCCGCGGTCCAGA
TCGGAAGAGCGTCGTGTTGGGGAA

>3169 len=204 tot-seq=3

GTAGTGGGTCCCAATCCAGGTCCTGGAGATGGGCCACCAGGTGGAGGTAGGGAATGAAGT

AGCCAGTGTTGATCAGGGTGAGGGCAACAGTGTAACGGAGGAAGGGGCCATGATGGAGGA

GAGAGGTGAGTTGGGCCCTGGGACCACCCACAGCAGGGTCCTCAGCCAGGGAGGGTGGGC

GGAGGAGAGCACCACAGGCCACTA

>3170 len=204 tot-seq=3

GGGTTGTTGAAGCCACTGTCTCTCTCAAATGTGCTGGTTAGGGCGCAATAGAAAACCACT

TCCTCTGCTGTGGGAGGAGAAGAAACTCATCCCGAAGCCAGCTGCAGGCTGAAGAGCGGG

TTCAGGTCACCCCTGTACAGCAGGACAGGGCCACACACACATGTCGCGGCCCGGGTCCCT

CACTGCGTTGGGAGTCCGGTGTCC

>3171 len=205 tot-seq=3

CAGAAATGGGGGGGTGTCAGTTGTAGGATTATCAGAAAGCCCTCAGTGGTGGGAGGCAGG

CGTAGATGCCAAAGGTAATATAGATAAACCTGTAACAATACCATGTGCACCTTAAAAGCC

TTAGTCTAGGGATCGTTTACGGAGCCTCCCTGGAGACCAGACCTAGGGTCAATGGTTCAA

AATAATTACATTCTTTTCGTTTCTG

>3172 len=205 tot-seq=3

CTTGCAATGCGGTTTGTTTTTCCACATGTGGGTATGTGAGGAAGCCAGATATAGGATAGG

CTCAAGCCCCTTCTTTCCTCCGAGGTTCTCACGTCTCCAGCTAAACTCCACCTCCTAAAA

GTTTCAAAGCCTTCCCAAAACAGCATCACCAGATGGAGACCATGTGTTCAAACACATCCT

CCTGTGGAGCATGCCTCCCGTCCCA

>3173 len=205 tot-seq=3

CAGGTAGATGTCACCCATGACAACACGCACAATGGGATGAAGGCTGTCTCCAAAGCGGTG

TACAACTGAGAGGCCAATAAGTGGCACATAGAAGGCGAGTACCACACCAATGTGTGACAC

ACAGGTTCCAAAGGCCTTGGCCCGCTCTGACTTGGAAGGCAGTTGCAGAACCGTTCGTAT

TATCAGAAAATAGGACAAGGAGATG

>3174 len=205 tot-seq=3

AGAGAGCAATGCCCCACTCCAGAGCCTCCCCTCCCGTGCTGGGGATGTGGGTCAAGGGCT

TCCCAGGAATGCAGCTGCCCCTCCAGGGCTGGGGGCTGGCCAGCGCCTCCTGTGTTGGGC

AGCAGCTCTCTGAAGCCAGGGCCCCCACGCCACTTCCAGGTGTGGACAACCCCGCTGATC

AATGTCTCTCTGTGTTCTGCCTTCC

>3175 len=206 tot-seq=3

TAGGGAATTTTCGTCAATGGGGGAAACCCTGAACGAGCAATGCCGCGTGAGTGAAGAAGG

CCTTCGGGTCGTAAAGCTCTGTTGTAAGAGAAAAACGGTAATGATAGGAAATGATCATTA

AGTGATGGTATCTTACGAGGAAGCCACGGCTAACTACGTGCCAGCAGCCGGGTAATATGA

AGATCGGAAGAGCGTCGTGTAGGGAA

>3176 len=206 tot-seq=3

CTTTCCAGACACCGCAGAGAAAGGCTACCATGGGCCTTAATGAGGACTGTACCACACAGA

ACTTCTCCACAAGGGAAGGGGAGGAGAGTGGTGGGGTGCCTTTTTACCCGGGGGTCCTGG

TGCACTCTGGCTATGTAGATCATCCTGCCACAGATCATTGACACTTCATAGGGATTCCAA

GTTCCACCAGCGGCCGCGGTAATAAC

>3177 len=207 tot-seq=3

AAAAGAGTGCTTGGGAATCAGAATCTGCAGGACTTGGTGGATGAGATGAGGGCAGAGGAG

TCAGGGATGATCCTGGTTCTCTGACTTGACCTGCTGGGTTGTTGGAGGAATGCTTGCCTG

AGTCAGGGAGGGGTCCCACAGGAGGAGCATGCTGGGTTATCAAGCATCCAGAGACTTCCC

TGACGGCTCCCCCTTCCCATTCTCCAG

>3178 len=208 tot-seq=3

AGACAAAAGTGGGAAAACCAAGAAAAGATAAACGGCAGAGATGACAGAGATAAGCTAAAA

AGTGGAAGGGGAAAACCCAGGCAGAAACAGCAGCTACAAAGGCCAAGCCAGGGAGTGACT

CAGACAGAGACAGGTAGGCAGAGAAGAAGGCAAAGTAGACCCTGAGGCAGTCGCCCACTC

ATTCAAGCCAATCCATGCCCCCAGCCCA

>3179 len=208 tot-seq=3

TGGGGAATATTGCGCAATGGGCGCAAGCCTGATGCAGCCATGCCGCGTGTATGAAGAAGG

CCTTCGGGTTGTAAAGTACTTTCAGCGGGGAGGAAGGCGATAAGGTTAATAACCTTGTCG

ATTGACGTTACCCGCAGAAGAAGCACCGGCTAACTCCGTGCCAGCACCGCGGTAATGAGC

AGATCGGAAGAGCGTCGTGTGGGGAATA

>3180 len=208 tot-seq=3

TGACCACATTTTCCGTGCCATTGACATCCAGAACGGAAAAGTAGTGTGGACCTATAACAA

CGTAAAAGGATATGTAGAAACAAAGCCCTTGGTTACAAACAATCAAGTTATCTTCGGTGC

ATGGGACAACACACTTTACTCGCTGAATCAAAAGAACGGTCATGAGAATTGGCGTTGGAC

GGGCAATCTCACTCGAATGCACTTCTCA

>3181 len=208 tot-seq=3

GCTCCGCCCAGTCCCTGCGGTGTTCCAGCAGCATCTTCAGTGTGTAAGCGTTTCTACCTT

CATCTTTCTCTGAGGGGGACTCGGGTGATGCATCATTGTCCCTGGTTACAGGTGACAAAA

TGGGCTCAACTGTGGGAGGGGCCTCTGTTTGTAGGACTCCCTGCACCAGGTTTTCCTTCT

TTACACACACACAGCTCTCTGATTAGTG

>3182 len=209 tot-seq=3

TGTAGTTTTGAATCTCCTCAAATCCTCTCATGGAGATAGATCAAACCAAACAAAAAACCC

ATAAGTAGCCTTTATCACAAAACTAGAGGACGGTTTTCCCTATGAACCCCAAAACAAGAG

GGTGGGGCAAACCATCACCAATGGGCACCAGACTTGCCTGGTATGGCACCCACACAAGAG

GGAGTGGTAGACAGTATGCCTTCAAACTG

>3183 len=209 tot-seq=3

ATCTTAAAGACAGATCCAATCATTTCCGCTGTCTCCTCATGGGCATGGAGATGAGTCAAG

GGAAAGAATAAACATGTTTGTTTGGAGAGACATCGGTCAGGAATAGGGTCTCTGGTGGTG

CCAAGGGTGACTCAGGGGCCACTTTGAGGATCAACTACAATAGATTACTGAGGTGAAAGC

AGAATCTCACCAATGAGGCCAAGAAGAGC

>3184 len=209 tot-seq=3

CGGTGACTGGGAGTGCAGCTGTGAGGCTGGGACGCCCGGGTTTCCTGAGCCCCGGGAGAG

CAGCGCGGGACTCCGTGGCGAAGGGACCGAGAACTTTCGGGACCTTTACAGAAACAGCCG

CACACCTTCGAGCAGCCTATATATATTGTATTTCGATTTTAGCCCAAGCCAGGTCTCCCT

GAAGTCCCATCTCCTGGAACCTCCGCCCG

>3185 len=210 tot-seq=3

CGGGCTTTTCTTTCTTTTTTTTTTCTTTTGTTTTTGTTTTGTTTTGGTATTTTTGTTGTT

GTTTTGTTTTGTTTTGTTTTGAGACAGGGTTTCTCTGTGTAGCACTGCCTGTCCTGAAAC

TCACTTTGTAGACCAGGCTGGCCTTGAACTCAAAAATCTGCCTGCCTCTGCCTCCCAAGT

GCTGGGATTAAAGGTGTGCACCATCACTGC

>3186 len=210 tot-seq=3

TGGGGAATATTGCGCAATGGGGGAAACCCTGACCGAGCAACGCCGCGTGAGTGAAGAAGG

TTTTCGGATCGTAAAGCTCTGTTGTAAGAGAAGAACGTGTGTGAGAGTGGAAAGTTCACA

CAGTGACGGTAACTTACCAGAAAGGGACGGCTAACTACGTGCCAGCAGCCGGGTAATGCA

TAGATCGGAAGAGCGTCGTGTTGGGGAATA

>3187 len=210 tot-seq=3

TAGGGAATTTTTCACAATGGGCGAAAGCCTTATGAAGCAATGCCGCGTGAACGATGAAGG

TCTTATAGATTGTAAAGTTCTTTTATATGGGACGAAACGCTAAGATAGGAAATGATTTTA

GTTTGACGGTAATCAATTAGAAAGTCACGGCTAACTACGTGCCAGCAGCCGCGGTATACA

AGATCGGAAGAGCGTCGTGTAGGGAAAGAG

>3188 len=210 tot-seq=3

GGGGGTGGGTGGGGGAATGGCATGACCCAAGGCCTGGCACGAAGTTGGCATGGAGCACCA

CATGGTGTTGCTGAAGAGGGCCGTGGGGCTGCAGCATGAAGGGCCTGGCATGCTAGATGA

TGGACTTGACTTTCTATAGGTGATGGGGAGCCCTGGAAGACTTCAGAGCAGGGGAGGGAC

AAGGAGCAAGGTGGCCGTGGGAAGATGGCT

>3189 len=210 tot-seq=3

CAGCAGGGGCCTGGCCTCCCTCAGCCCTGGAACAGCAGGTCCCTGCTAAACCAGAACAGG

GGCCTGTGAGGTCTCATTGTGAGTGTCCAGCCTTAGTGAGCTCCCAGTGGGTCTATATGT

TGGGGTTCTGGGGAGGGGGTAGTGGCATGCAAGGACAGCCTGGGTGTGATATCCTTGGCA

CCAGTGATAAGCCACAGGGCTTCCTGTCAC

>3190 len=210 tot-seq=3

TGCACTAATTATTGTCAAGGAAAATTTCTCCTTATGCCCTGAGTTGTGGGAATGTGGAAT

TACCATGAGTAAACTGGATACCCTACAACTTTGCATTAGTAAAGCCAACACCACTATTGA

AAAGGATTTCTACAAAGACAGATACATCAGAAAATGATAGCTCATTTGGTGGAGCAGAGA

AACAAGGATTGCATGTTTCTGCAAATAATG

>3191 len=211 tot-seq=3

GAATGAGTACTTATGGCCATGCTCCACTGCAGCTGTTCCCAGGCCAAGTCCCATGGCCTC

CATGAAGGCTGGGGTTCTGTCTCTGCCAACTCTCTAGGCAGTTTCCCCTGTCGGCTGAAA

TTTCCATGGAGGTCATAGGGTCTCTCTCCTCTACCTAGGTTACCAGAGGTCTGTGGCAGA

GTGAACCATATCTATTCCACTCACCCTTTCC

>3192 len=212 tot-seq=3

AGGATGGCAGGATGTGTCAGGCATGGTAGAGAGGGTCAGAGGCCCTCAAGTAATGGGGAC

AGCTGGAGTCTCCTCTGGAAATCAGCCTGCACTTTGACCTGGGGAGAAGTTCTCAGACAT

TATTTCCCAAGGGGACAAAGGGCCTGCTGAGACGCAGTCCCTCTGATACTGCTTTATGGT

GACTTGGCCAGGGGTTGGCCAGCATAGCACAG

>3193 len=213 tot-seq=3

AGGCCAGGTGTGACAGGCCAGAACGGTTTCTACCAATGCCACATTTACAGTTTTATATCT

TCACGGGTATGCTTTCAGGAGAGGGCACAGGGCATGTCGGAGACCAGTGGGACACAGCTG

GCTTGGAACACAGATGACTCTGAAGCTGGAATCCTTAGGAATCTCTAGGCAACTCCTTCC

CTGAGGAAGGAAGCACTGGGTGTGGTACCTCTC

>3194 len=213 tot-seq=3

AGTGGTGCCACCAGCTGTTGATCCAAATCTTCTCAATCCCAAGCCCACATTTATGTAGAT

GCACTTGCCCATGTCACACATGAACATTGGGCAATGCGATCCTAGATATGCACGAGTTTC

CCACCTTACTTAGAGAAAAGGAAAGGAACATTTGCTGGGAGCCTGCAGTGTCCAATACTG

TGGTTTGCATGTCCGTATTCTCAGTGAATTCTC

>3195 len=213 tot-seq=3

AGATGGTGACTGGAGTTCAGACGTTGCCTCCGATCTCCTACGGGGGCAGCAGTGGGGAAT

CTTCCGCAATGGGCGAAAGCCTGACGGAGCAACGCCGCGTGAGTGAAGAAGGTCTTCGGA

TCGTAAAGCTCTGTTGTCGGGGACGAATGTTGAGGCCTAGAACATAGGTTTCAAATGACA

GTACCTGACGAGGAAGCCACGGCTAACTACGTG

>3196 len=213 tot-seq=3

GGGGACATGTGAAGACATGGAGAAGGGGCTGGTGCTGGACCCACAGGGGCTTCTGGAAAC

TAGGGAGTGAGATGCTTCCGGGCTGGCCTGGGCTCTTCCTGTGCAGCAGAGCTCAGCAGA

GCTGCAGGGGAGGCACCTGCAGCCCAGAGCTGGGTACAACCCCCGTGGGCCTGCCAGAAT

GGGACATCTGAGCAAGGAGAGAACCAGCAAGAA

>3197 len=214 tot-seq=3

ACTAGTTGGGTCTGGGTGGGTGATAGCCACCAGTAATGGGGACTTATTATTAAGCTGCCC

AGAGTTATCACTCATCACCCCAGTCACAGAGCAGCTGGAGACGAGGCCATTCTTCTCTTT

TCCTGGAGTTGCAGCCTGAGGGCTCTGAGTTACTGAGTAAAAGTTGGCGTTCCAAGCACT

GGTTTCTTGTTCCTCACCATGTTGAGTGAGAGTG

>3198 len=214 tot-seq=3

TGAGGAATATTGGTCAATGGGCGTGAGCCTGAACCAGCCAAGTAGCGTGCAGGAAGACGG

CCCTATGGGTTGTAAACTGCTTTTATGCGGGGATAAAGGAGTCCACGTGTGGATTTTTGC

AGATACCGCATGAATAAGGACCGGCTAATTCCGTGCCAGCAGCGCGGTAATCTTAAGATC

GGAAGAGCGGTCGTGTAGGGAAAGAGTGTGCTGA

>3199 len=214 tot-seq=3

TCTAATGGTAATATTCCCAGTTAATCACACCCCCAAAGGCACAGTCCCAGCTGGGATGAT

ACCTCCCGTGCATGGAACATACAAGACGCTCTGAGTGACCAATAACAAGGGAACTGGAAA

AAGAACAAGGGTAGTACAGGTTGAAGATTGGCCTTGTTCTTTCTCTGGACTGCAAAACGT

TCCATGGATGGATTCTAGAACTTCGCGAACCTGT

>3200 len=214 tot-seq=3

GCATCAGGGTCCCAGGTGCACCGGAGGGGAGCAGACAGATGCAAGCCAGGCTGCTCTACT

TGGCAGGGAGAAGGAGGAGGAGGAGCAGGGGCCCCTCAGTTGCTGCAGGGCCTCCCTAGG

AACTTACCGCCTACCTCTACACCGTAGTTCCTGCTTCTCAGAGTCCCTTTGTTAGAACAC

CTGTGCCTTCCTCCGCACCCAACTCCATCTCTTC

>3201 len=215 tot-seq=3

TGAGGGATATTGGACAATGGGCGGAAGCCTGATCCAGCCATGCCGCGTGAAGGACTAAGG

CCCTATGGGTCGTAAACTTCTTTTGACGCAGAGCAATAAGGACCACGCGTGGTCCGATGA

GAGTATGCGTCGAATAAGCATCGGCTAACTCCGTGCCAGCAGCCGCGTAATTCAAAGATC

GGAAGAGCGTCGTGTAGGGGAAGAGTGTGCCAATG

>3202 len=215 tot-seq=3

AGAATGTAGCAGTGGAGATTCAAGCATTCAGCCAGTGGAGGGACTAGATGAGTTAAGGGC

CTTTTCACCCATTCCTCTTGAGAAATACTGTGAGAACCCAGAGTAGGTGCCAAACTCCCG

CAAGGAACAGGCACTAGGACAGAGGCCACGGAGACAGGGCCACTCAAGCACACCCACAGC

AAAGAGCTGGCTCTGCTTGACAAGTGGCAGGTCAG

>3203 len=216 tot-seq=3

AGCTGGACCTTTAGGATGACCTTGAGAAGAGCCACAGGTTCTCAGAACTAACAATTCTAT

TATTCTTCATTTCTCTCTCGTGATGACAGTTTCTGCTTTGTAAGCTCAGAGTGTTCTACT

AAGAATGTGACAGAAAACCCATTTGGACCAGCTGTGGATCATCTGTAGATGTGTCTCCCA

CTCAGGCCAACACTGTGAGCTAATGAGCTGACCCTC

>3204 len=217 tot-seq=3

CGAATCGAGCTTGTCGCGGAATTCCGGCACACCCTTTCGAAGAATTCGAGAGTGGAACGG

CACGTCAGTTCCTGGAACAAACATGAATGGAGGCTTGCCGCCATACTCCTTAGCGCGCTT

AGAAGCGTCTGCCGCCAATGCCTTCAAGCCTTTAATAGTTCCAGCAATTGCGTACTGCTG

ACCTGCCAAGTTGTAGTTTACAATTTGCAAGAATTCG

>3205 len=217 tot-seq=3

TCAAGCAGAAGACGGCATACGAGATAGTCATGTGACTGGAGTTCAGACGTGTGCTCTTCA

GATATAATAAGGGAGGAAGAAGTGGGGAATATTGAAAAATGGGAGAAAGAATGATGAAGA

GAAGAAGAGTGAGAGAAGAAGTATTTAGGTATGTAAAGCTCTATCAGAAGGGAAGAAAAT

GACGGTACCTTACTAAGAAGCTCCGGCTAAATACGTG

>3206 len=217 tot-seq=3

GAAACCAGAGCAAATTTTGATGCATTGCTTAAAACAGATGAAGAGGACGAGCAAAGGGGA

GACATGTCTCTTAATTCTATGTGAAAACAAGGATATTTATGTATAGACTTCGCTCTTCAG

GCATTGTAGAACTGACAGCTAATTATGCAGCCTGGATAGCATCTTGCCGAAGATGAACAA

CTCTGGTGGACCAAGTGAAGGCCAAACTAGTGATGTC

>3207 len=218 tot-seq=3

ACAGTGGAAGACTAATGGGATTAATACTTGGGATTCATTAGCCAAGGATTCTGGAACTTT

TCCAGTCCTCTCCACCAAATGGCCCAAACTGCCCCCTTAATAGCATTAAGTGAAATTACA

AATCAGAGACGGTGGCTGATCAATCTCCTTGGGGCTTCTCTACAGCTTTTGTGCAGTAGG

GGTGGTGACAGTCCTTCAGTGGGGCGACTGGTCCTCTG

>3208 len=218 tot-seq=3

TCCGTAGGCATTGTTGATGGCACCGCTACCATGTTTGAACGCATGGGCTACGGAATTTAT

GAACTTGTCACCTTCTGGGCTCCTACTTACAAATGCACGTACAAGCCCCCTTATCAGGGG

CGCTGCGGCATGAGCGGCCTCAAGGAATACAATCCCAATGGCGGCCTGAGCGAATTCCCT

GCGGAACTCAGCTTCCAGAGTTACTACAAGTATTCACG

>3209 len=219 tot-seq=3

GCGTCTAGGTGGTTATATAAGTCTGATGTGAAAATGCAGGGCTCAACTCTGTATTGCGTT

GGAAACTGTATAACTAGAGTACTGGAGAGGTAAGCGGAACTACAAGTGTAGAGGTGAAAT

TCGTAGATATTTGTAGGAATGCCGATGGGGAAGCCAGCTTACTGGACAGATACTGACGCT

GAAGCGCGAAAGCGTGGGAAGCAAACAGGATTAGATACC

>3210 len=220 tot-seq=3

GATTACAAGGTGACTGGAGTTCAGACGTGTGATCTTAAGATATAATAAGGGAGGAAGAAG

TAGGGAATATTAAAAAATGGAAGAAAGTATGATGGAGAAAAGAAGAGTGAGTGAAGAAGG

TTTTAGGATAGTAAAGCTCTGTTGTTGGTGAAGAAGGATAGAGGTAGTAACTGGCCTTTA

TTTGACGGTAATCAACCAGAAAGTCACGGCTAACTACGTG

>3211 len=220 tot-seq=3

GGCTTCCATCCTGCCCGCACCAGACACCGCTGTTGAGTGCGTGGAAGCGTCACGCCGCCC

CACTACGCCGCTCCTCTCCGCCAGGTCTCTTCTGGGGACTTGCCCAAGGCTCTGGGCAGA

CAGAGCTGACACGCCGCCATCTAGCCGCCGGAAGCAGACTCTTGACCTGTGAAGCTGGCG

GCGGCACTGCTCTGAGGCCAGTCACACGAGCGACAGGCAC

>3212 len=221 tot-seq=3

CTCTAACATCTCACATTCATGCCAGGCTTTTGGCCCAAGCCAGGCCGGTCGAGCCACATC

TGAGACTGGCTTGCTGTGGACTGTGCTGGGAGAGGCTGCTAGTCCAGGCCAGAGTGCCAG

TCTTTCCAGCTCTGATGCTTTCCCTGTTGCTTGCAGCAGAGCCAAAGCGCCCAATGCACC

CACCAAGGGTACTTGGGTAAGGCCCAGTGCAGTCACGGTGC

>3213 len=222 tot-seq=3

ATGGGACAGGAGGCAGCGGAGGGGGAGTTGAGAGCGGGGAGTCAAGAGGTGGGAGCTGCA

GAATTTGGCATCTCTTCCCATGACTGTGAAGTGTTTAGAGACTGGCTTGGGCTAACGTGG

GAGCTCACTAGGACCCACCATCTCTTGCTGCATGTAGATGCTAATTGAGAGTGGGAAGGC

AGGCAATTGAAACACTTATTTTTTATCATATAAGAAAAGAAA

>3214 len=222 tot-seq=3

AATCGCTTGAACCCAAGAGGCAGAGGTTGCAGTGAGCCAAGATCGTGCCACTGCACTCTA

GCCTGGGTGACAGAGCCAGACTCCGTCTAAAAAAAAAAAAAAGGGCAGAGAGAAAGATAC

ACACAGACACAGATGGAGAGGAGGGGGCTGAGAATTGTGTGGCCAGTATGTGGGCAGAAA

GAGAAACTGAGGCTGGGGCTTAGAGGAAATCACAGGGGGAGG

>3215 len=223 tot-seq=3

GCTGAGATTGTCGCTCAGTGGTGACGCACTTTCCTGACATAAGCAATACTTTGTGTTGAA

GCCCCAAACCTCAAGGGGAAATAGACACTGGTGAGTGAGCATGCTGATGAGATGCCACGA

GAGAAACAGCTTCAGCATACTGTCTTCTGGTTTCATCCAGTGCATTCAGGTCCTCACAGA

AGACCTCTGTGAGCAACTCTCGTTCTCACCAAAATCAGCAGCA

>3216 len=223 tot-seq=3

TAGGGAATTTTTCACAATGGGCGCAAGCCTTATGAAGCAATGCCGCGTGAACGATGAAGG

TCTTATAGATTGTAAAGTTCTTTTATATGGGAAGAAACGCTAAAATAGGAAATGATTTTA

GTTTGACTGTACCATTTGAATAAGTATCGGCTAACTATGTGCCAGCAGCCGCCGGTAATG

TATAGATCGGAAGAGCGTCGGTAGGGAAAGAGTGTGCCAATGT

>3217 len=223 tot-seq=3

TTTGCATGAGCTGGTGGCCCTTCAACGGATCAGGAGGCAGGCACAGGCCTACCGTTCGGT

GCCCCTTGTTCCAAAGGTGCTGCGCCGCATGATGCGTGGTGGCCGCCCAGTGGCGGTCCA

GATAGGGAAGGTTGCTGCTCTGGTAGGAGGTGCCGCGCACAATGCAGGGGATGCGGTGCG

TCTTGAACCAGAGCTGGGTTTGCTCCGAGGAACGGTGAAGTAT

>3218 len=224 tot-seq=3

TTTGACCCCCACACTGTGTTTTTCCCACTGACATCAAATCCTGAGGTTTTAAAATTAATC

CCATACAAAGACAGTTCTTCATTGTGGTGCCTGTTTGAAATTAGACCAGCAATAAGGACC

TTTTTAAAATGTCTCGTTGTTAAGATAGGCCCCTGTTGGAGCTGAACAGCTGCCTGCCAG

GGAGGGGGTGGGAACAGGGAGGGACAGTGCGCGGGTGAGACGCC

>3219 len=224 tot-seq=3

CCTCACCGTGTGACCCGGGCAGTGAGTTCACTTCTCTGTGCCTGTTTCCTCCTCTATAAA

ATTGGGTTTTTAGCTGAATTTTCCTTACAAGGTGTTAAGAGGATTAAGTGGGCCAATTCT

TAGTTGCAACTCAGCCCGGAGTGAATGTTTCTGTCTTGTCTCCTCTGTGAGTAGAAAAGG

GTGAGAAGGAGGCCCTGGTCTGCTGGATAGGGTAAGGGTTAAGG

>3220 len=224 tot-seq=3

CAGGGTGGAGATTCTAGGAAAGAGGAAAGGCCTTCCAGGAATTTGGGCGGGAAGGGCTGA

CGAAAACGCAGGGCCCAAGGAAGGGGAGGCCACTCACTCATCCAAGGTCACGCGGCTGAG

CCAGGACCCCCCAGGGGCTCCTATACACGTGGGCACTGGCAGAGGAGCACTGCCATGGAG

GACCCCGCTCCCCACCTGCCACCACCATCCTCGGTGCAGGTCAG

>3221 len=225 tot-seq=3

CAGAGAGCCCCAGAGACAAGGGGCCACTGAGGCTAAAGCAAGCACGACTGCTCACACAGG
AGTGCAGCGGCACAAAGGAGAGCTCTCGCCGGTCAACACTGCAGAAGCAGTGCTCGGCGT
GCGCTCCCTGGTGGGCATTGGTCGGTCAGCGCTGTCCCCAGTGCTGTAGGGTGGCTACCG
ACTGGGATTCCTAGTTTTCCTACTGTGCTTGAGGTCATTCCCACA
>3222 len=227 tot-seq=3
AGGCCCGCCTGGGCCAGGGCGTGCACCAGACCAGAATCCTGCGGATTTAGTATTGTGAGG
GTAGAAGACCAGCAAGCAGCATGCCCTCGTGGTTACTGATCTTATGAAGAACACTTGTCT
TTCATATTTGGGACTGTGTTAAGGTTTTCTGGAATATTTTTGGTCGTAGTAACGATTCTG
CTTTAGGAGCTTTCTTCTCTGGTGTTGCACAGACCTGAGTGATTTGA
>3223 len=228 tot-seq=3
GTTGTGTGCAGAGAGACTTGGGAAGGGAAAATGTTGATGGAGAGGTCCACATGAAGAGGC
TGGGGCTTCATCCTGGGGTATCCCTTCTCTTGAGATTGACAGTAGGGTTGAGGAGGATTT
GAAGAGTTTAATCAGGGGGCTGAATGTGGTTGGATCTCTGTCAGATATCCAGAGGCTGGA
GGCAGGGAGGCCGGGGAGGATGCCACCTTATAATCCCCGAGAAAGATG
>3224 len=228 tot-seq=3
CAGGCCTTTGCCTGACAGACAAACAAGGCACAAGGTTACCATGCTCTTCTTCAGTGGGCC
AAACTGGACACTTCTGCACTCTCCTTCCAACAGAGCTCCTGCCCACCTGTCCTCACCTCA
TCTATGGGGATCAGAGTCTGCATGGCCATGATCAGCTTGGGCACCAACATCCGAGCGAGA
GGCTGAGGGACACATCACAGAGAGCATGATGCAGCCCAATCTCACACC
>3225 len=229 tot-seq=3
CTCCATAAAGTCCTCCAGGCATCCTGAAAAATGCTGAATCCTATGATGCACACAACTTAC
TCTCAGTCTGGTATCCCTACCTCATATTCAATCCTTCTTCCATATGACACGTTTTCAAAT
ACTTGATAGCTATCATGCCCCCTTTGTCTTCTGTCCCCTAGGCTAAGTTTCCCTTGATCC
TTCAATCAGAAAATAAGTATGCCTGAGTGAAGCCATTTGAACAAAATTC
>3226 len=230 tot-seq=3
CGGGCAGAGGAGCAGCTATATCTTCATAGCTGAGGGGATGGGACCAGGGAGAGGTAACTG
AGATGCCTGAAGCTGACATTTAAAGAGGCACTCACTCTCAGGGTCAGCACCCAAATTTTG
TTTCTCACCTACTTTTCTGGTCACATCCTAGTCTCAGCCTTGTCACTAGGTACTTTTCCT
GTGCCAGACTTTGGGCTAAGGGCTTGTGTGCACCATCACATTCCATCCTC

>3227 len=230 tot-seq=3

GTTAGAGAACCTTACACACATATTTATGCTATGCACTGTCCCTGGATAATCTAATTTACT

CTCTTGACTTTAGCTAACACATGTGAGAAGAAAACTCTTGAACCTACCTCATCAGCCATG

ACTTTGCTCCCAGGCACAAACTCCATGTGGCTTTCAGTTTAAAGTCTCACCTCTGCACCT

GAAAGTCCTAACCGCACCTGACACTCATTGTGAAAACACTACCTGCACTA

>3228 len=230 tot-seq=3

GGGCGTAAAGCGAGCGCAGGCGGTTTGATAAGTCTGAAGTTAAAGGCTGTGGCTCAACCA

TAGTTCGCTTTGGAAACTGTTTAACTTGAGTGCAAGAGGGGAGAGTGGAATTCCATGTGT

AGCGGTGAAATGCGTAGATATATGGAGGAACACCGGTGGCGAAAGCGGCTCTCTGGCTTG

TAACTGACGCTGAGGCTCGAAAGCGTGGGGAGCAAACAGGATTAGATACC

>3229 len=230 tot-seq=3

ATTTGTTACAGCAGCAAGAGGAAACATGCAAGAAGCAAGGCCAACTCAGAGCAAAAATTC

AGAAGGCAAAGCTGGTCTCTGGTGTGCCCACATGCCCAGTACCATGACTGACAAGTTGGC

TGTCTTAGTCTGTTTGGGCTGCACTAACAATACCATAAACTGAGTGGCTTATAATAAACA

GAAATGTATTTCTCATAATACTAGAGGCTGGGAAGTCCAAGATCAAGGTG

>3230 len=232 tot-seq=3

TAAAATGTCCAAAATGGCAGCGCCTATCAAAATAGAGGCACTATGACATCTCCTCAGCCC

AAGGCCATCACAGGAGCTGTCCATGGTGGCAGCACCTTTCCTGCCTCAGCCTACTTCTGT

CCTGAGGAGCTGTCCACAGTGGTGAGACCTTCTTTCAATGCCCCAGCCTACAGCCTTGAT

GAAGCACCACTACCGGAGTTGTTCACAGAGGCAGCACCTCTCTCCAGCCTCC

>3231 len=232 tot-seq=3

TAGGGAATTTTTCACAATGGGCGAAAGCCTTATGAAGCAATGCCGCGTGAACGATGAAGG

TCTTATAGATTGTAAAGTTCTTTTATATGGGAAGAAACGCTAAGATAGGAAATGATTTTA

GTTTGACTGTACCATTTGAATAAGTATCGGCTAACTATGTGCCAGCACCGCGGTAATTGT

TAGATCGGAGGAGCGTCGTGTAGGGAAAGAGTGTGCCAATGTGTAGATCTAG

>3232 len=233 tot-seq=3

CATTGGGCAGACAGAGGAGCTGAGCTGTAGTGCAGTCTCAAGGGGAAGCCCCAGTTGACC

CCATGGTATGCGTTGGACCTGGAATGTCCCTTTAAAAGTGTCCCGAGTTAGGATGCAGGG

GCTGAACCCCATGTCCATAAGCCCTTGGATGGAGCTGAGCCTGCAAAACATCTGACCCTG

AGCACAGCTCTTTAGCAGGAGCACAGGGGAGGAGGGTGTCTGCAGCAGCACTT

>3233 len=235 tot-seq=3

TGGAGCCAAGCTGTTCCCACGTGCATCAACTGTCCCGTTGGAGCCAGGGTGGCCCCACG

GGGGTGTGTACAGGGCCGCGAGTGGGCTCCTACCTGAGAAAGATCCCCTTGAGGTTGGGC

GTGGAGTCGAAGGCATTGGCGGGCACCGCACTAATCTTGTTGTTCTGCAGGTACAGGTAC

TCAAGTGACTCGGGGAGCCCCTCGGGGATCTCTGTGAGCTGATTCCCGGCGATGT

>3234 len=240 tot-seq=3

AGAGGGAGGCGTGGCAGCAGGCAGTGGGCACCTGTGGGCTGGGCCTCCTGTTCCAGGCAG

ACAGCCACCTCCCCTGGAGGCCCACAACACCACAGATGAGGAAGAGAAGCACCACAGATG

TCTCAAGGATGTTCAGTGCCAGGACGCCCCCAAGGCACCTGCACTAAGGTCTAGGCGTCC

TTCATCTCAGCGAGGGCACAAGTGAGATACAGCACAGTAACAACATTCATGGGAAGGGAC

>3235 len=241 tot-seq=3

GAACAGAACAATCCTGTGTGTGTCGCCAAGCTATGGTGTGACGGAGGTCACATAACCTCT

TACAGCCTCCATTTCTTTGCCTGTGAACTGGAGATATATTGCCCCTCATCGAGTTGTGAA

GATTAAATGAAAGAGTGTATATTGAAAATACTTAGCATAGTGCTTGGCTCAGAGTAAGTG

TCCAAAAAGTGTTTGCTCCCATCTCTCCTCCCCTTAATTGCATTTTGTTCCCCCACCCAA

C

>3236 len=159 tot-seq=3

TGGGGAATATTGCGCAATGGGGGAAACCCTGACGCAGCGACGCCGCGTGCGGGATGAAGG

CCTTCGGGTTGTAAAGCTCTTTTATTAGGGAAGAAAGGTTAAGATAGGAAATGATTTTAG

TTTGACTGTACCATTTGAATAAGTATCGGCTAACTATGT

>3237 len=100 tot-seq=2

TGAACCCTGAGACTGATTTAGGCATGCAGGAGTTTGGCTGAATAAAGGTAGCTTAAGGTC

TGTTTTGTTTTGGAGATTGGAGGTGGGGGGATTAGATACC

>3238 len=100 tot-seq=2

GAGCCGCAGGTCGGGCCTGAACTGCCGCTGGGAGAGGAAGCAGCGCCCTTGCCAGGAACG

TGGGGCTGTGGTAGCAGCAACACCTGCTCCAGCTTCACCT

>3239 len=101 tot-seq=2

GAGAGGGAAGTCCCCTTTGTCACCAAGTCCCTGGGGAGCCTGAGAAAGGAAGGATACAAG

TGCAGTGGTCCCTCTAGGGCAGCTTTCAGCGAGGCTGGACA

>3240 len=101 tot-seq=2

GAGGGTAACTTGAGCAAGTGGAAGAGCCAGGGCAGGAAACAAACAGGACTTAAGCAAAGA

CAGGTGAAAAGTGCTGAGAGAAGACAGAGAGCTCAGATAGC

>3241 len=101 tot-seq=2

GTAGGGTGGACCTCTCATTAGGCCTTCCTATGGTGCACTTGGCTGTAGGCAGCAGGGAGC

AGGGTGAATTAATCCCCGGGCCCCTAAAGAATGCACGTGTG

>3242 len=101 tot-seq=2

CGAGGAAGAGAGCCTGGCAGGGCAGACGGGGGATGAGCAAGGGCTGTGGCCGCAGCCAGG

GGAGCCCTGTGCCCAGGGAGAGCTGGCACTGTCCTCTTCAC

>3243 len=102 tot-seq=2

GAAGGGCTGCAGGTAGACGTGCGGAGAGCGGACGCAAGAATGGAAAAAGCGTGAAACGCC

CCTCTTATCAGCAGGAAGACACGGGCGACTGGCGACAGTTCT

>3244 len=103 tot-seq=2

TGGGGAATATTGCACAATGGGGGAAACCCTGATGCAGCGACGCCGCGTGAGCGAAGAAGG

CTTTCGGGTCGTAAAGCTCTGTTGTTTGAGAAGATAATCACGG

>3245 len=103 tot-seq=2

GCAGGAAAGGGTGAGCACATGCCAAGCAGCAACAAGAGAAGACAAGAAGCCTCCAAGAGA

GTATGTATTTCTCTGCAGAAGAGGAATGGTGAGGGGGCCATTA

>3246 len=103 tot-seq=2

CATCTTCTCTCTTAAATATTCTCTGCCAGAGCAGAGCATGGCCTCCATCAGTAGGGTGAC

CATAGGATTGGCTGTTCAAAGAGACACTTTACAGTTGCACTTC

>3247 len=103 tot-seq=2

CCCCCTCATCCCTGTGCCCACCCTGGCTTTACCCAGCTGTGTGCAAAGTTGTGTTTCAAA

GCTGGAGCTCGGAGCATGCTCCCTCCAGGGTCCCACCCAAACC

>3248 len=104 tot-seq=2

GGATCACTGACATCATGGCTATTAGAGACCTAAAATCTTATGGACATACTATGGTAACGG

TTGCCATCATACATGTGATATCAATATAACATAGACCTCCATGA

>3249 len=104 tot-seq=2

TAGGGAATCTTCCACAATGGACGCAAGTCTGATGGAGCAACGCCGCGTGAGTGAAGAAGG

GCTTCGGCTCGTAAAGTCCTGTTGTTGGGGAAGAAGGACACGGG

>3250 len=104 tot-seq=2

TAGGGAATCTTCCACAATGGACGCAAGCCTGATGCAGCAACGCCGCGTGAGTGAAGAAGG

CTTTCGGATCGTAAAGCTCTGTTGTCGGGGAAGAAGGATAGAGG

>3251 len=104 tot-seq=2

TATTCAAAGCAATGGCCTGGAGTGAATCTAGACAGAGCCCTGAGCCCTCCACTATTTACA

GGCAAGGTGAGGAGGAACAAGCAAAGGAGACTAAGAGGGAATCG

>3252 len=104 tot-seq=2

GGACTCCAGCCAGGGAGACAGCAAGGCGGCCAGCCAGTGGCAGCTGCTGACCTTCTGGCC

ACAGAGAAAGGACAGACACATGGGGAATAATTTACAAACACAGG

>3253 len=104 tot-seq=2

GCAGGCAGGCAGGCAGGCAGGAAGGAAGGAGTTGTAGATCTTTGGCAGCCCAAAAGGGTA

AGAGTCTCTCCCAGATTCCCTGGGGATTCAAGCACCTGTCGGTC

>3254 len=104 tot-seq=2

ATGATTTGAAATGAATTTCTAGGAAGGGTATATAAAAGAATTGTTTGTTCAGAGTTGATC

CAATCCCTGAAAAAATAAGGATGAAGTCAAGTTATATCCAATAT

>3255 len=104 tot-seq=2

AGCCACAAGAAGACAATTCTCCTTTGAGCCAGCGAATGCCTGTCCCTGAAAAGTCTGGAA

GCCACCCCTCTGCTGCCACCAAGTGGCCGTGACGAGCCTGAAAA

>3256 len=105 tot-seq=2

AGACCAAATAGGACTAATTAAGCAGTGTTGGTTTTTCAACCAACACGATATTTCCACTGG

GTATCAAATTCACGTTCAGGTTGAGAAGAGGGTGTGAGTAAGCCA

>3257 len=105 tot-seq=2

TGGGGAATATTGCGCAATGGGGGAAACCCTGATGCAGCGACGCCGCGTGAGGGATGAAGG

CTTTCGGGTCGTAAACCTCTTTTGTTTGGGAGCAAGCTTTCGGGT

>3258 len=105 tot-seq=2

TAGGGAATCTTCCACAATGGACGCAAGCCTGATGGAGCAACGCCGCGTGAGCGATGAAGG

TTTTCGGATCGTAAAGCTCTGTTGGTGGGGAAGAAGGATAGAGGT

>3259 len=105 tot-seq=2

GGGAGTGAGGTGAACTCTGTGAGGGTCCTTAGTTGTATTTTTGTTAAGTGCACTGGTTTT

GTGTTGGCTGGCCTCCAGCCAGTAGGTGGCATTTTCAAGAGCATA

>3260 len=105 tot-seq=2

GAGAGGCAGCAGCAAGGTGAGAGGCAGCAGCAAGGTGAGAGGCAGCAGCAAGGTGAGAGG

CAGCAGCAAGGAACAGAGCCGGTTCTTCAGGGCTAGGGACATGGA

>3261 len=105 tot-seq=2

GAAGGGTTTCGGCTCGTAAAGCTCTGTTGTTGGAGAAGAACGTGTGTGAGAGTAACTGTT

CCCGAAGTGACGGTAGCCAACCAGAAAGTCACGGCTAACTACGTG

>3262 len=105 tot-seq=2

GAGGCTCAGAGCCAGGGCCAGGGAGGCAGGTGGAGAGGGTGTCCGTGGAGGGGCTGCACC

GTCAGCCTGGGCCCCCAGTGGAAAGCAAAATAGAAAGGCCCTGTG

>3263 len=105 tot-seq=2

CACTGGAGGCAGCATAGTGGTCGTGCCAGAGTTATGGGAGAAAACACGTGTCAGTTGGAC

CTAACTGCATCATAGAGACTTCATCGTTAGGGTCCACAAACCAGG

>3264 len=106 tot-seq=2

AGAAGGGTTGCGGCTCGTAAAGCTCTGTTGGTAGTGAAGAAAGATAGAGGGAGTAACTGG

CCTTTATTTGACGGGAATTACTTAGAAAGGCACGGCTAACTACGTG

>3265 len=106 tot-seq=2

TAGGGAATCTTCCACAATGGACGCAAGTCTGATGGAGCAACGCCGCGTGAGTGAAGAAGG

GTTTCGGCTCGTAAAGTTCTGTTGTTGGGGAAGAAAGAAAGGGGAA

>3266 len=106 tot-seq=2

CTACCTGTGATAACTGTGCTTTTCAACTAAAGAAAACCTGGGACACCTGACCTCCTCCTT

GGTCACAAGCCCTGAGTCAGGCAATTATAGTGCAATGACTTCACAC

>3267 len=106 tot-seq=2

CATGGGACTCTTGTTCACATGGTCCATGCACACTGAACTTAAATATTGATTTTCAAGAAT

AACTTCCAGGTTGCTCATCACTTGTCTGCCTGGGTGTTTACTTCCA

>3268 len=107 tot-seq=2

TGGGTAAGGAGGCCAGCTGTGGGTAAGGAAGCCAGCTGTAGGTAAGGAAGCCAGCTATGG

GTAAGGAAGCCAGCAGTGGGTAAGGAAGTCAGCAGTGGATAACGAAG

>3269 len=107 tot-seq=2

TGGGTAAGGAAGCCAGCTGTGGGTAAGGAAGCTAGCTGTGGGTAAGGAAGCCAGCTGTGG

GTAAAGAAGCCAGCTGTGGGTAAGGAAGCCAGCTATGGGTAAAGAAG

>3270 len=107 tot-seq=2

TAGGGAATCTTCCACAATGGACGCAAGTCTGATGCAGCAACGCCGCGTGAGGGAAGAAGG

TTTTCGGATCGTAAAGCTCTGTTGTTGGGGAAGAAGGTTAGAGGCAG

>3271 len=107 tot-seq=2

CTTAACTCTTTTTCTCTCTGAGCACGAGCAAGCCGAGCTTGTCCTGGCTCTCCATTGTCT

GTCTGCAAAGATGGACATCTCTGGCTTTCTCTCTCTTTCTCTGGGTG

>3272 len=107 tot-seq=2

TAGGGAATCTTCCACAATGGACGCAAGTCTGATGGAGCAACGCCGCGTGAGTGAAGAAGG

GTTTCGGATCGTAAAGCTCTGTTGTTGGAGAAGAAGGTTAGGGGGAG

>3273 len=107 tot-seq=2

TGGGGAATATTGCGCAATGGGCGCAACCCTGACGCAGCCACGCCGCGTGCGGGATGACGG

CCTTCGGGTTGTAAACCTCTTTTGATTGGGAGCAAGCCTTTTGGGTG

>3274 len=107 tot-seq=2

TGGGGAATCTTGCGCAATGGGGGCAACCCTGATGCAGCGACGCCGCGTGCGGGATGAAGG

CCTTCGGGTTGTAAACCGCTTTTGTTTGGGAGCAAGCCTTTTGGGTG

>3275 len=107 tot-seq=2

TGGGGAATCTTCCGCAATGGGCGCAACCCTGATGCAGCGACGCCGCGTGCGGGATGAAGG

CCTTCGGGTCGTAAACCGCTTTTGATTGGGAGCAAGCCTTTTGGGTG

>3276 len=107 tot-seq=2

TGGGGAATCTTCCGCAATGGGGGCAACCCTGATGCAGCGACGCCGCGTGCGGGATGAAGG

CCTTCGGGTTGTAAACCGCTTTTGATTGGGAGCAAGCTCTCGGGTGA

>3277 len=107 tot-seq=2

TAGGGAATCTTCGGCAATGGACGGAAGCCTGACCGAGCAACGCCGCGTGAGCGAAGAAGG

TTTTCGGATCGTAAGGCTCTGTTGTTAGAGAAGAACGTTGGAAGGAG

>3278 len=107 tot-seq=2

GACAAAGAAACGCTCAACGAACTGAGCGTCAATGAGGTCTTCGAGCGACGGCTGGCACAA

GAGCCTGAAATGGCCGAAGAACGTCGGCAACGCATGAACCAGATGTT

>3279 len=107 tot-seq=2

CAGTGATCTGGGCTTCGGAGGCTTGTCAGAGCCTCCCCTGGCCTCCTCTTGCCTGCGGTG

GGGTGGGCGTGCTCCCTCTCCTCCATGATGCTGGCACAGTGGGTAAC

>3280 len=107 tot-seq=2

CCAACACCACCCCATGTGTCACTTAGTATCATCTCAGCAAGTTTCTTTCAATCTCATGCC

CCCTTATTTTCTTTCTCTGCTTTACGAAGCCGGAGGAACTTTCTACT

>3281 len=108 tot-seq=2

AGCGACGCCGCGTGCGGGATGAAGGCATTCGGGTTGTAAAAAGATTTTGATTGGGAGAAA

GAATTTTGGGTGAGTGTACCTTTCGAATAAGCGCCGGCTAACTACGTG

>3282 len=108 tot-seq=2

TGCAGGGCCCGGCCGGAGTGGGCATTTGTCACAGTGATTCTGACTACATCTGTACACACG

GTGCCGGGAGTGATGCTGCTATGCTGGCTGCCTTCCTTAGCCCAGAGT

>3283 len=108 tot-seq=2

TCGGGAATCTTCCACAATGGACGAAACCCTGATGGAGCAACGCCGCGTGAGGGAAGAAGG

TTTTCGGATCGTAAAGCTCTGTTGTTGGTGAAGAAGGATAGAGGTAGT

>3284 len=108 tot-seq=2

TAGGGAATCTTCCACAATGGACGCAAGTCTGATGGAGCAACGCCGCGTGAGTGAAGAAGG

GCTTCGGCTCGTAAAGCTCTTTTGGTGGGGAGGAAGGACAGGGGTAGT

>3285 len=108 tot-seq=2

TAGGGAATATTGCACAATGGACGCAAGCCTGATGCAGCAACGCCGCGTGAGTGAAGAAGG

TTTTCGGATTGTAAAGCTCTGTTGTTGGTGAAGAAGGATAGAGGTAGT

>3286 len=108 tot-seq=2

CTGGCGGTTGTCCAGCCCAGATTTCCGTTGGGGAGATTGGCTGGTCCAAGCTGCAGAAAG

TGGTAGGACAGCCCAGAGCTTCTTGGAAATACGACCTCCATGTCCAGG

>3287 len=108 tot-seq=2

GCGCGCAAATTACCCACTCCCGACCCGGGGAGGTAGTGACGAAAAATAGAGGTAGTAACT

GGCCTTTATTTGACGGTAATTACTTAGAAAGTCACGGCTAACTACGTG

>3288 len=108 tot-seq=2

GAAGAAGGGTTTCGGCTCGTAAATCTCTGTTGTTGGTGAAGAAGGACAGGGGTAGTAACT

GACCTTTGTTTGCCTGTAATCAATTAGAAAGTCGCGGCTAACTACGTG

>3289 len=108 tot-seq=2

AGACCCTCAAGCAGGGTTGCTCAGGGTGACCCTGTGCTTTCCAGAGCCCGTTTGCTGTCC

AGGGGATGAGGGCTTAGCTTTGGCGATTATGTGGTCCCTAATAAGTTC

>3290 len=109 tot-seq=2

TAGGGAATCTTCGACAATGGACGCAAGTCTGACCGAGCAACGCCGCGTGAGTGAAGAAGG

TTTTCGGATCGTAAAGCTCTGTTGTTGGAGAAGAAGGAGTGGGGGAGTG

>3291 len=109 tot-seq=2

TAGGGAATCTTCCACAATGGGCGCAACCCTGATGCAGCAACGCCGCGTGAGTGAAGAAGG

TTTTCGGATTGTAAAGCTCTGTTGTTGGTGAAGAAGGTTAGGGGTAGTA

>3292 len=109 tot-seq=2

CCCTGATGCAGCGACGCCGCGTGGGTGAAGGAGCGTTTCGGAGCGTAAAGCCCTATCGGC

AGGGAAGAAGATGACGGTACCTGACCAAGAAGCCCCGGCTAACTACGTG

>3293 len=110 tot-seq=2

TTTACTTGGTTGATGGGTGGTGTGGTGGCTAACCATGTGGTGTACGATCCTTGCATGGTG

CTTTTGTGGTATAGAAGGGAACTTTAGCTCCAGCCACGCCGGAGAATTGG

>3294 len=110 tot-seq=2

ATACCAGCCTCTGGCCTCCACAGGCACCCAACACACATAAACAAACCAACAAATGAACAA

ATAAATACCTGGGCCTCAATCTAGCAGGTGCCCAAGCATGAGGAGAAAGG

>3295 len=110 tot-seq=2

GAGCAGCAGGAGAGGCAGCAGGAACAGCAACAGCAACAGCAGCAGCAGCAGCAGGTGCAG

CAGCAGAAGTAGCAGCAGCAGCAGGACCAGGAGCAGCAGGAGCAGCAGGT

>3296 len=110 tot-seq=2

TAGGGAATCTTCCACAATGGACGCAAGCCTGATGCAGCAACGCCGCGTGAGGGAAGAAGG

CCTTCGGATCGTAAAGCTCTGTTGATGGTGAAGAAGGATAGAGGTAGTAA

>3297 len=110 tot-seq=2

CGGGAAGAACAGCAGCTGGTTGTGAGACAGGTCCAGCGTCTCCAGCTCGAAGGCAGCCTC

TCCCCCGGTCGCGAGGAACCACTCCAGGACGTTGTAGCTGACGTTGAGGA

>3298 len=110 tot-seq=2

GATCACTTGAGGTCAGGAGTTCAAGACCAGTCTGGCCAACATTTAGGTGAGATCCTGTCT

CTGCTAAAAGTACAAAAATTGGCTAGGCATGGTAGCAGGCACCTGTAACC

>3299 len=110 tot-seq=2

GTGTAGTAAGAAAAGGATCTAGGCAGGAGTCCTCTTAGGACAAGTCCCTGGGCTTCTACA

TCTTTCATTTTTATCTACAGTAGTGGTTTTCATTCAAGTGTGGTCTGGGA

>3300 len=110 tot-seq=2

TGGGGAATCTTGCGCAATGGGGGAAACTCTGACGCAGCGACGCCGCGTGCGGGATGAAGG

CCTTCGGCTCGTAAACCGCTTTTGATTGGGAGCAAGCCTTCGGGTGAGTG

>3301 len=110 tot-seq=2

CAGGCCACTCCCTGCCTTGGATCAGACCACAGCCTCGCCTGCCGGCCCACCCCTCTTCAG

AGCAGGTTGTAACGTTACCTCCTGCCATCATGAAGCCACCTCCAGCCCTC

>3302 len=110 tot-seq=2

CTTCCAGGTCATAGGTGGATTGAAAGATTTTCTGACTGGAAATTGGTTGAAAGAGTTATG

TTATCTTTTTTATTTATTTATTTTTTTGAGACAGAGCCTCACTTTGTCAC

>3303 len=111 tot-seq=2

GTTCTAGTCCTCCAGCTCTTTAGAGCCAGAAAACTCCCTGCTGGAAGGAAGGACACCTAA

GTCTCCTAGCTCTGCTGGCTACTGAGGTACCAGTCCAATCTCAGAGTCAGC

>3304 len=111 tot-seq=2

GGGTTTGTATCGATGTCCTTGGCCCATGTTACCACCAAAGGCCCTGAGGCCGGCCCTGGT

CTGGGCGTCTTGCCAGTGCCATGGTAGATGTCTAGGGGCTGTGCTTAGCCA

>3305 len=111 tot-seq=2

TAGGGAATCTTCCACAATGGACGAAAGCCTGACGGAGCAACGCCGCGTGAGTGATGAAGG

TTTTCGGATCGTAAAGCTCTGTTGATGGTGAAGAAGGATAGAGGTAGTAAC

>3306 len=111 tot-seq=2

TGTAGGCCACTGCCCCACCTTCAGGGCTATAGTTTCTTTCTTCTGGAAACCACACAGGAC

ATGAGAATCCCAGACTGGGCTGCCCTTCCTGGAAGAGGATGTGTGTGTGAG

>3307 len=111 tot-seq=2

TAGGGAATCTTCCACAATGGACGAAAGTCTGATGGAGCAACGCCGCGTGAGGGAAGAAGG

CTTTCGGATTGTAAAGCTCTGTTGTTGGTGAAGAAGGATAGAGGTAGTAAC

>3308 len=111 tot-seq=2

ACTGGCCAGGAGCCTCTCCAGGGGGTTGACCACGTCCTGGGCAAGATGGCTCAGGTTGGT

AGAAAGCCGTTCTCCCATGACAGGGGATGCAAGCCATTTGCACCCTTCACG

>3309 len=111 tot-seq=2

GGTCACGAAAGGAAGGGGCACCCAGGAGCTGGGGGATGCTTGGAGGGAGGGAATGAGTGG

GCAGACAGAGGGAACAGCTGGGGGTTGAAGGGACCTGGGGAGAGAAGAGAA

>3310 len=111 tot-seq=2

TGAATGAATGAAGTGAAATGGTATTTATCCATTTCCTGGAAAGGTCCAGGGTTTGGCTCT

GCAGGGCCAAGAGAACAGCTTTAGCTGTGCCTTAACCCAGTCCTGGAGAAG

>3311 len=112 tot-seq=2

TAGGGAATCTTCCACAATGGACGCAAGTCTGATGGAGCAACGCCGCGTGAGTGAAGAAGG

GTTTCGGCTCGTAAAGCTCTGTTGTTAGAGAAGAACAGCGCATAGAGTAACT

>3312 len=112 tot-seq=2

TGGGGAATATTGCGCAATGGGGGCAACCCTGACGCAGCGACGCCGCGTGAGGGATGAAGG

CCTTCGGGTTGTAAACCGCTCTTGATTGGGAGCAAGCCTTTTGGGTGTGTGT

>3313 len=112 tot-seq=2

TGAGGAATATTGGTCAATGGGCGCGAGCCTGAACCAGCCCAGTAGCGTGAAGGAAGACGG

CCCTATGGGTTGTAAACTGCTTTTATGTGGGGATAAAGTGCGCGACGTGTCA

>3314 len=112 tot-seq=2

TAGGGAATCTTCCACAATGGACGCAAGCCTGATGGAGCAACGCCGCGTGAGGGAAGAAGG

TCTTCGGATTGTAAAGCTCTGTTGTTGGGGAAGAAGGATAGAGGCAGTAACT

>3315 len=112 tot-seq=2

CAGTGAGAGTGAGGAGGAAGACGTGGGAGAGTACCGTCCCCACCACCGAGTGACCGAGGG

CACCATCAGGGAGGAACAGGAGTATGAAGAAGAGGTGGAGGAAGAACCCCGC

>3316 len=113 tot-seq=2

CTCCAGGCCCCTTCCCCGGGTGGGTCCAGAGGACACTCCCCTCCTACCCCGTAGCCTCCA

CTAGTGGAAGGTGGTGAAGACAGAGGTGTCCTTGAGTCCACAGGGCCTCTGGT

>3317 len=113 tot-seq=2

GGAAGGACTTCTCCTGGGATAATTTCAACGTCCCTTTGATTGATTCATTCGATAAACCTC

CATCCAGCTTCCTGACTCTCCATACACCTGGGACGCTCTGCTACCTTTTGAAC

>3318 len=113 tot-seq=2

TAGGGAATCTTCGGCAATGGACGCAAGTCTGATCGAGCAACGCCGCGTGAGTGAAGAAGG

TTTTCGGATCGTAAAGCTCTGTTGTTAGAGAAGAAGGTTGGTAGGAGTGGATA

>3319 len=113 tot-seq=2

TAGGGAATCTTCCACAATGGACGCAAGTCTGATGGAGCAACGCCGCGTGAGTGAAGAAGG

TAACTGACCTTTGTTTGACGGTAATCAATTAGAAAGTCACGGCTAACTACGTG

>3320 len=113 tot-seq=2

GGAGAGGGGAGCCAGGGAGAACACAGCCTCAGTCCTGGAAGGAGAGGGCCTGCTGGGAAG

AACAGAACCCAGCTGTCTTGGCCCCAGGTGAGCCCTCACCTGTGCCTCAGGCC

>3321 len=114 tot-seq=2

GTGAGTGAAGAAGGGTTTCGGCTCGTAAAGCTCTGTTGGTAGTGAAGAAGGACAGGGGTA

GTAACTGACCTTTATTTGACGGTAATCACTTAGAAAGTCACGGCTAACTACGTG

>3322 len=114 tot-seq=2

GTGAGTGAAGAAGGGTTTCGGATCGTAAAGCTCTGTTGTTGGTGAAGAAGGACAGAGGTA

GTAACTGGCCTTTGTTTGACGGTAATCAATTAGAAAGTAACGGCTAACTACGTG

>3323 len=114 tot-seq=2

TGAGTGAAGAAGGTTTTCGGATCGTAAAGCTCTGTTGTAAGAGAAGAATGGGAGTGAGAG

TGGAAAGTTCACTCCGTGACGGTATCTTACCAGAAAGGGACGGCTAACTACGTG

>3324 len=114 tot-seq=2

GTGAGTGAAGAAGGGTTTCGGATCGTAAAGCTCTGTTGTTGGTGAAGAAGGAAAGGGGGA

GGAAATGAACTTTGTTTGACGGTAATCAATTAGAAAGTCACGGCTAACTACGTG

>3325 len=114 tot-seq=2

GATCACTGTGGTCACTTGCAAACATGTGAGATGCTGGGAGCCCTTTGTATAGAAGCAAGG

GACTGCGTGGAAGGAGTGGGAGGTAGTGGGGATTCAGAAATGGAACTTGTGTGC

>3326 len=114 tot-seq=2

ATGGCCTTTCTCCAAAGGATGAGCAGGCCTGCTGACAGCTTCCCTTTGAGCTTCATGTCT

CCAGAACTGCCAGGGAGTACATTTCCATTGACACACTGTTGGTGGTGATTTGCT

>3327 len=114 tot-seq=2

ACAACTGTGTTCATCTGAAAGGAGGAGGCCCCACTCCCGTGCAGGCGCTGCCCCTGGGGG

TGAAATTCCCAAGCTTGTTGGGGATTCTCCCGCCTGGCGTTGCCGCTCTGAATG

>3328 len=114 tot-seq=2

TGAGGCCATTGTGCTGAATGAAATCACCTCTACCCAAAAGGGCTCATTCTGCAGGACTCC

ACTCTAGACACCCTGAGCAGTCAAATTCATAGACAGAAAGCAGGATGGTGGTGG

>3329 len=114 tot-seq=2

CAGTTGGAGCCTGTCAAGTGGGCGAGGTGGGGTTTTTCCCTTTGTCCTTCATTCTAACTG

CTCTTTCTTCCTTTTCTCAACATGTGTAGGTGGCAGCTGGTCTAAGCACCAAAC

>3330 len=115 tot-seq=2

GAGCTACAAGAAGCAGTCATCTGTGGGAAAGGCCCAAGTGGGGAAAAGTAAGAAATCTGG

AGATGAGAGAAGGGGACAGTAGGTAGGGCCATTAATGATTCTGGATTCAAACCTG

>3331 len=115 tot-seq=2

TGGCCTTTTAAGAGATTTTTTTTTTTTAAAGAAAAGTGGTTTTTCTGCTTAAACATTAG

TAACTGGAGCCAACAATCACTGTCCCCACCATGCCTGTCACTCTTCACTCCCCAC

>3332 len=115 tot-seq=2

GGAGAAGGTCGAGAATCAAGACCAGCGTCTGACGAGGAGAAAGCTTTGTTTGGAAATGCT

AAGGCTACTTCTACAATGCGTGTAACTTACGTAAATGTTGTAGCAATGGTCGTTC

>3333 len=115 tot-seq=2

CGTGAGTGAAGAAGGGTTTCGGATCGTAAAGCTCTGTTGTTGGTGAAGAAGGATAGAGGT

AGCAACTGGCCTTTATTTGACGGTAATCAATTAGAAAGTCACGGCTAACTACGTG

>3334 len=115 tot-seq=2

GCTGGGTGGCAAAACGGGCGGGGGCGGAGGGGCCGGTGTTGGGCTTACATCTCCTGCTTC

CCTGAGCGCCTGCACGGCAGCTTGCCCTTCTTATAGAGGAAATAGAGGACAGCGC

>3335 len=116 tot-seq=2

AGGGATGCATGCTGTGTGCCCAGCAGCCATGGTCAGGGACAGAGGCAGCAGAGGGATGCA

TGCTGTGTGCCCAGCAGCCATGGTCAGGGACAGAGGCAGCAGAGGGATGCATGCTG

>3336 len=116 tot-seq=2

TAGGGAATCTTCCACAATGGACGCAAGCCTGATGGAGCAACGCCGCGTGAGTGAAGAAGG

TTTTCGGCTCGTAAAGCTCTGTTGTTGGTGAAGAACGTGAGGGGGAGTAACTGGCC

>3337 len=116 tot-seq=2

TGGGGAATATTGCACAATGGGCGCAAGCCTGATGCAGCCACGCCGCGTGAGTGATGAAGG

CCTTCGGGTTGTAAAGCTCTTTCACCGGAGAAGATAATGACGGTATCCGGAGAAGA

>3338 len=116 tot-seq=2

TAGGGAATCTTCCACAATGGGCGCAAGCCTGATGGAGCAACACCGCGTGAGTGAAGAAGG

GTTTCGGCTCGTAAAGCTCTGTTGTTGGGGAAGAAAGTGAGTGGGAGTAACTGATC

>3339 len=116 tot-seq=2

GCGTGAGTGAAGAAGGGTTTAGGCTCGTAAAACTCTGTTGGTGGTGAAGAAGGACAGGGG

GAGTAACGGACCTTTGTTTGACGGTAATCAATTAGAAAGTCACGGCTAACTACGTG

>3340 len=116 tot-seq=2

TGATTGCAGATACAACCTTCTTTCCATCTCCCTACTCCACACACCCTACTGAGCCACCCC

AACCCCACTGCCCTAACCTATGCTTCACTCTTGTGATTTCTAAGGGGTTTGTGTTC

>3341 len=116 tot-seq=2

GCGTGAGTGAAGAAGGGTTTCGGCTCGTAAAGCCCTGTTGTTGGTGAAGAAGGACAGGGG

TATTAACTGGCCTTTGTTTGACGGTAATCAATTAAAAAGTCACGGCTAACTACGTG

>3342 len=116 tot-seq=2

GTAGTGCTGAGAGGAGCTGCAGGCACTGCCAGCGCTTGGCTCTGCCTCTCAGATTCCCCT

ACTCTCTGGAAAGCTTGCCGCGGTGAGGAGGGGGCTGATTCATAGCAATTTTCTAC

>3343 len=116 tot-seq=2

ATCCAACAAAGACAAAGAAAAAAGAATTTAAAAAATGAACAAAACCGCACTCAGTGCTCA

ATGGTGCCCAGGCTGGAGTGCAGTGGCGTGATCTCAGCTCGCTACAACCTCCACCT

>3344 len=116 tot-seq=2

TAGGGAATCTTCGGCAATGGGCGCAACCCTGACCGAGCAACGCCGCGTGAGTGAAGAAGG

CTTTCGGATTGTAAAGCTCTGTTGTAAGAGAAGAACGTGTGTGAGTGTGGAAAGTT

>3345 len=117 tot-seq=2

TTTTTTTTTCAAGCAGAAGACGGCATACGAGATGTAAACTGCTTTTGTACTAGGGTAAAC

CTATCTACGTGTAGATAGCTGAAAGTATAGTACGAATAAGGATCGGCTAACTCCGTG

>3346 len=117 tot-seq=2

CACTGCTATGTGTTATTTTCACAGAAGTTAATAACCAGCAGCCGCAGTAATCTACAGATC

GGAAGAGCGTCGTGTAGGGAAGAGTGTGCCAATGTGTAGATCTCGGTGGTCGCCGTA

>3347 len=117 tot-seq=2

GAAGCCGGGAGGGGAGGCCGGCCGGGGAGACCGCAGGAAGCCGGGAGGGGAGGCCGGCCC

GGGAGACCGCAGGAAGCCGGAAGCCGGGTCTCTAGGAAGCCGAGGGAGTGTGTGAGC

>3348 len=117 tot-seq=2

CGCTGAATCCAGAGCTGCTCCAGCCTCAGCAGCCCTATCTGTGATGGCATGCTGATGTCA

TCAGCACCAAAAGCCCTTGCAGGTTTCAACTCGGCAATTAAACGGCTCCTCTGAAGC

>3349 len=117 tot-seq=2

TGAGCCAAGATCACGCCATTACACTCCAGCCTGGGCAACAGAGCAAGACTCTGTGTCAAA

AATAAATAAATAAATAAATAAGCAATGATCTCCCCCACCCAGATGGGTAAACTGAGG

>3350 len=117 tot-seq=2

TGGGTGGGGGGTGGCAGGCAGGTTCACCCAGCAACTCCGGCTTCTTGTTGGACAGAATCT

GGTAGAAGATGTGGTAGTTTCTCTCAGCTTTCAGCTGGAAGATCACCCGGGACTTCT

>3351 len=117 tot-seq=2

GAAGTTTTTGGGAGGAGACAGCAGACTGGGGAGCAAGTCAGTTCTCACGGCCGCTGAATA

ATTCACAGGGCAGCCGGGATGGCATATGCTTCCTCTAAGCCCTTAGCAGCAGGAATG

>3352 len=118 tot-seq=2

TGGGGAATCTTCCGCAATGGGGGCAACCCTGACGCAGCGACGCCGCGTGAGGGATGAAGG

CCTTCGGGTCGTAAACCGCTTTTGATTGGGAGCAAGCCTTTTGGGAGAGTGTACCTTT

>3353 len=118 tot-seq=2

CCGCGTGAGTGAAGAAGGGTTTCGGATCGTAAAGCTCTGTTGTTGGAGAAGAAGGAGTGA

GAGAGTAACTGGTCACGAAGTGACGGTAGCCAACCAGAAAGTCACGGCTAACTACGTG

>3354 len=118 tot-seq=2

TGGGGAATATTGCGCAATGGGCGCAACCCTGATGCAGCGACGCCGCGTGAGGGATGAAGG

CCTTCGGGTTGTAAACCGCTTTTGATTGGGAGCAAGCTCTCGGGTGAGAGCACCTTTC

>3355 len=119 tot-seq=2

GCTAAAAGGGAGGCTAAAAGGAAAGAGAAAGTAACCACTGAGATTGAGCAAGTGAGATCT

GCTGCGTGTAATTTTTTTTAAATCACACTTCTGCCTTCCCAAGAGCTCTACCCACACTC

>3356 len=119 tot-seq=2

CTTTGGACTCTGCAAAGGAGACACGAGACAGAGATCAGACGTGGTGTGACCACACAGCCA

CATGAACAACTCGCCAGAATAACTGGCCGGGCAAGAGAGAAACGTGCCCGTCCCGCATC

>3357 len=119 tot-seq=2

GGGGGAAAAGAACACACACACGGACCCCCAAACACTGTAGTCTCCTTCTCCCTCTGCTGC

CCCCTGGACCAGCAAGAGGAAAATACACCTTGTTGTCCACCAGAGATGAAAAAGGATCC

>3358 len=120 tot-seq=2

GGAGGAAACTCTGGACCAGCAACGCCGCGTGAATGATGAAGGTCTTCGGATGGTAAAGTT

CGTTTCTAAGGGAAGAAGATAGTGGCGGTACCTTAGGAATAAGCCGCGGCTAACTACGTG

>3359 len=120 tot-seq=2

GAAAGACGGAGGCAGAGAAGCCGCGTGAGGGAGGAAGGCCTGCGGGGGGTAAACCGCGGG

AAGCAGGGAAGAGGCAGCAAGGTGAAGGGACCTGCAGAAGAAGACCCGGCTAACTACGTG

>3360 len=120 tot-seq=2

TGGGGAATCTTGCACAATGGGCGCAAGCCTGACGCAGCGACGCCGCGTGCGGGATGAAGG

CCTTCGGGTTGTAAACCGCTTTTGATTGGGAGCAAGCCTTTTTGGTGAGTGTAACTTTCG

>3361 len=120 tot-seq=2

AATTCAACATGAGATTTGGTGGGGGACACAGGTCGAAACTATATAAATAGTCCCACAAAC

CCAATCCACAATCCCCGACCTGGAAGAGCTCCAAAAACCCAGAGGCTTTTTGTAACCCAC

>3362 len=120 tot-seq=2

CAAGAGAGTATTGAAAGTCTCCTAGGTGCAGACCTTTCCCTTCCCCAAGGCTTTTCCTTC

CTAGGCAATCCCCTGAGCTGGATATCACATGGGAGGGAATGCTTTCTTATGGTCTCTGTA

>3363 len=120 tot-seq=2

CTTCTTTTCCTTTGGGTAGATACTCAGTAGTGGGATTGCTGGATTGAATCATAGATCTAT

TTTTAGTTCTTTGGGAAATTTCCATACTGTTTTCCACAGAGGTTGCACTAATTTACATTC

>3364 len=120 tot-seq=2

TGAAAACCCCAAGGCAAGCGTGGAAGAGTAGAACAGCTCCAGGGGCAGTGGGAAGATGAG

GTCACCCCCGCATGTTGACGGACACCAAGGGTGGGGGTGGAGGATGTGAAGGGGATCAGC

>3365 len=120 tot-seq=2

CAGAAAAGAACCACAGTGAGTTAATCCCGAGCATCGAGGACCAGGAGGGGCACCACTTTA

AATGGCTTATCGCTATTGATGTGCCAGTCCTGCTCCTTAGTTTTCTAATAAAAGCCCTCA

>3366 len=120 tot-seq=2

TGAGCTGTGATCATGCCTCTGACTCAAGCATGGGCAACAGAGCAAGACCATGTCTCTAAA

ACAATGAAATATTATAAAAACAAAACATACGTCATCATATCATTCAGGGCTCAACATTCT

>3367 len=121 tot-seq=2

GAGAAAGAGTGACAATCCAAGTTGAGGAAATAGAGAAGATTGGGAGGAGTAGAATAAGGT

GCACCTACTGTGTGCTAAATACTGTACTATGTGCTTTACATATGATAGACCAATTGACCT

T

>3368 len=121 tot-seq=2

GACCCTTACTGGGCCTCTGTGTATATCTCCTGGTCTTTATACCCATTCCCTGGGTCCCTC

ACTTGCTCCCAGACCCTGTAATTGCCACTGGGAAGTCCTTAGCTATTCATCATCCCCACA

G

>3369 len=121 tot-seq=2

AAACCCATGATGCTCTCCAAGGCTGGTTCCTTCTCTGTGCAGAGGGCCTCAGGTAATCAT

CTGCCTCCTACACTGGGGCTCTGAGTGAGAGCTCAGCATTCTGCTTCCCAAAAATACCTA

C

>3370 len=121 tot-seq=2

CTTTAATCTTCTCCTTCTTTGAGCAGTGGACTTGAGCAGCTGGCGGGAGAAGATTGCTCG

GGATGTGGCTGGGGCCACCTGGGGTAATGGCTCTGGGGAGGAGGAGGAAGAGGAGGATGG

C

>3371 len=122 tot-seq=2

TCAGAACGGCAGCTCCATATATTATTCATTCCAATCAAATAAGACACAGTGGTGCCTCAG

TAAACTTGCTTTTCATATCAGAATTGGTCTTATTGAGTAATACACACGGCAGACCCCAGC

TT

>3372 len=122 tot-seq=2

GCTTAGGAGTGTTAAAGGCTTAGGGAGGTTGGAGGGGAAGGTTCCTGGCCGGGCTGGCTC

GGACTGTTTATCTCTGAGAAGGGCTGCCTGACTGGGTGGTTTTGGCTGAATTTCACCTCT

CC

>3373 len=122 tot-seq=2

GGGCAGCTCTCTGGTGAGGAAGGAAACTCACACCTTGGCCAACCTCTTCGAAACCTGCCC

TTCTGCCCCAGCTTCCATCCAGTGTGATCTACCCCACGGACTATTACAGCCAGTCCTCAC

TC

>3374 len=122 tot-seq=2

CAAAGGCAAAACCCAGTCCATCCTCGCTTTCCCCGTGCCACAGCCAGCTTGTGCCGGCCC

CCACCAGGCACAGATGCAACACCCTGCACTCGATGCCCACAGAAGCCCCTCTGCAGAGCC

GG

>3375 len=122 tot-seq=2

GCATCAGGGCATGTGGACGTGGTGAGGACAGAGGCAGCCACGGGCGTGTAGGAAGAAGCT

TGGCAGAAAAAAAGGGGTGGAAAGAGGGAGAACAAAGGCAGGCGGGAGAAGAGTATGGGA

GC

>3376 len=123 tot-seq=2

CAATCTCGTGCGTCGGGATGCGCAGGATGAGCTCCTCGTTGTCGCGCCAGGTGAGCAGCA

GGCAGCGTGCGGACAGGCTCAGGATGCTGTCCTGCTCTGGTGTGGTCTTCAGAGGCAACT

CCT

>3377 len=123 tot-seq=2

GGAGCTGAGGCAGCGGGATGCTCTCATCGACGAGCTGGAGCTGGAGTTGGATCAGAAGGA

CGAACTGATCCAGAAGCTGCAGAACGAGCTGGACAAGTACCGCTCGGTGATCCGACCAGC

CAC

>3378 len=123 tot-seq=2

TCAGCCAAGCAGAGCTGTAACATTGAGCTCTAACACTAACCTAAGGCTCTTTTAAGAGCC

ATCATTTTTCCTAGTAGGCAGTAGAGCTGATAAGACAGGCAAGCCTGATACCCCCCACTC

CGA

>3379 len=123 tot-seq=2

TCAGCTCGCACATTTGTTCCTTTATTCCCAAGGAGAACATGAGGAAAGGACGTAGATATC

GCCCAAATGTATAATTTACCAGGTAAGAAAAGCCCAATGGCTTTCTAATTATTGCTGGAT

GTC

>3380 len=123 tot-seq=2

TGGGGAATATTGCGCAATGGGGGAAACCCTGACGCAGCGACGCCGCGTGCGGGATGAAGG

ACAGGGGTAGTAACTGACCTTTGTTTGACGGTAATCAATTAGAAAGTCACGGCTAACTAC

GTG

>3381 len=123 tot-seq=2

GGTGGGGGCCTTTCATCCTCACATTAGCACCGAGAGCTCGGGAGCTGCTTCTGTGCACAC

CCAGACTGTCCCCCAGCACCTCTAGCCTGCTCACAGCTGGGCACACGGCCACCTCTCTCC

CCT

>3382 len=124 tot-seq=2

TGGGGAATATTGGACAATGGGCGAAAGCCTGATCCAGCAATGCCGCGTGGGTGAAGAAGG

TCTTCGGATCGTAAAGCCCTTTCGGCGGGGACGATGATGACGGTACCCGCAGAAGAAGCC

CCGG

>3383 len=124 tot-seq=2

GGGCCAGCAGAGAGGAGAGGTGACTAGAGGAGGGGTCACAAGACCATATACGCAGGCAGC

CTCTAGAAGCTGGGAAGGCCAGGAAACCAGGAAAGCTGGGAAGGCCAGGAGCCTGCAGGA

GGGA

>3384 len=124 tot-seq=2

GCAGCAGGCAGCAGGCAGCAGGCAGCAGGTTCACTCTTGGAACCCAGGTGACCTACGATT

CTCATTTGTCAGCCACACATGTTCTCACTCTCTCTTTCCCCCCAAAGACTTCTCTTTTCT

CCAG

>3385 len=124 tot-seq=2

CCGTGAAAGGGCCCGCAGGGCCGCTCCTACGCCTCCTGCAGGTCGGAAGCAGTCAGTGCT

GCCCGGACCCCCTCGTGAACCGGACGGGCCTCAAAGCACGACCCCAGAGGGCCCCTGGCA

GCCC

>3386 len=124 tot-seq=2

GCAACGCCGCGTGAGTGAAGAAGGGTTTCGGCGCGGAAAGCTCTGTTGGTAGTGAGGAAA

GATAGAGGTAGGAACTGGCCTTTATTTGACGGTAATTACTGAGAAAGTCACGGCTAACTA

CGTG

>3387 len=125 tot-seq=2

CAGCCAGGAATGTTCCCAAGTTCAGGCTACATGGCAAGACCCTCTTTCCTGTGCCAACAG

TTGGTGGCTATGATAAACATCAACTCCATCCCATGCCACTTCACAGTGACCCATTTTGCA

GTTGC

>3388 len=125 tot-seq=2

GCACAGGAACAGACCCAGCAAGGCCAGAGATAAGAGATGGAGAAGGCAGCTTAGCCTGGA

AGAAAAGAACCTGACGAGGTTGAAGGGAGGGAGCACAGTTTGAGTTTGAGCATCGTTGTG

AGAAC

>3389 len=125 tot-seq=2

ATGAAAGCCATGTGGTTTAAAGGGCCAGATAGTAAATATTTCAGGCTTCATAGGCCATTT

GTGGTCTCCATCCCATACATTTTATCTTTTAAAATATTTCTTCCAACGCTTTAAATGCGT

AAAAT

>3390 len=125 tot-seq=2

GCAGGCCTAGACTCTTGGGTGAAGAAATACCTAACTCTTCTTTTCGGTGAGAAGGGAGTT

GACAGCTCTACCACACAGAGGCCTGCCAGGAAACAGCAGGGACACTCATGGTCTCTTAGA

GACTT

>3391 len=125 tot-seq=2

TAGGGAATCTTCCACAATGGACGCAAGTCTGATGGAGCAACGCCGCGTGAGTGAAGAAGG

TTTTCGGATCGTAAAGCTCTGTTGTTGGTGAAGAAGGATAGAGGTAAGTCACGGCTAACT

ACGTG

>3392 len=125 tot-seq=2

AATGCTATGTCACTATATACAGAAACAAGACAACCTGAAGCTAAATGGATGCCCCCTGCA

GAGTCAACAGGTCCAGCCTCACAGTGCACGCCCTGAGCTACAGCCTCTCCCAAAAGGCAT

CTTCC

>3393 len=125 tot-seq=2

TCACGAGGGCAGCTCAATTCGAACCTTGCGCAAGCCACGGCATGTCGTATCGGATATAAA

AATGCCACAGTCGTCCTCCCTACCCCTCTAGTCTTACGCATGGGACCTGTGGATTCTCTC

ACGCT

>3394 len=125 tot-seq=2

AGCAACGCCGCGTGAGTGAAGAAGGGTTTCGGCTCGTAAAGCTCTGTTGTTGGTGAAGAA

GGACAGGGGAAGTAACTGGGCTGAGTTTGACGGTAATCAATTAGAAAGTCACGGCTAACT

ACGTG

>3395 len=125 tot-seq=2

AGCAACGCCGCGTGAGTGAAGAAGGGTTTCGGCTCGTAAAGCTCTGTTGTTGGTGAAGAA

GGATAGAGGTAGTAACTGGTCTTTGTTTGACGGTAATCAATGAGAAAGGCACGGCTAACT

ACGTG

>3396 len=126 tot-seq=2

AGACTGAGGGAAGGGCCAACCAATAGCAGACCTAACGACCAATTTCATGGGAAAGCACCA

ATCTCTAACTCTATTAATGATGCTATGCTTGTAGACAGGAGCTTCTTATCCTCTGAGAGA

CTTCAC

>3397 len=126 tot-seq=2

TGGGTAAGGAAGCCAGCTGTGGGTAAGGAAGCTAGCTGTGGGTAAGGAAGCTTGCTGCGG

GTAAGGAAGCCAGCAGTGGGTAAGGAAGCCAGCTGTGGGTAAGGAAGCCAGCTATGGGTA

AAGAAG

>3398 len=126 tot-seq=2

GCCCTGGGGTGGGAAGTAAAAGGTGAGAGTGAGAACACCCCTCAAGAGAACTTGAAGAAG

GAGCAGAATGGGACCAGTGAGTTCATTTTAGTGAAGGCTGATGCAGCATAACCCCCGGGC

CTTCCT

>3399 len=126 tot-seq=2

AGGACCACCCCCACAAGGAGGCAGCAAGTCCCGAAGTGCCCGACCTCCTCCAGGAAAGCC

ACAAGGACCACCCCAACAAGAAGGCAACAATCCTCAAGGTCCCCCACCTCCAGCAGGAGG

CAATCC

>3400 len=126 tot-seq=2

GGGGCGCTGCAATCACAACAGTGAGAGGCTGGGTGGCTCCGGGAACAGAGTTCACACACA

CTCAATCCACCCTCTCTCACAGCTCAAACTCCTCTACCTTAAACTTCTGGGTGAAGCCCG

TTCTCT

>3401 len=126 tot-seq=2

ATCTGGTGTTCTGTTTAGGTATAGAATTAAAACCCACAAGGTCAAACAAGGAAAGTTCCC

AAGAAAAGAAACTTTATAATCCAAAAGATTATTACAGATAGCATTATATTGTATATCATT

AGGTGT

>3402 len=127 tot-seq=2

GGCTAGTGACAAGCTTTCTTCTGCCCTATAAAGCAAAAGTTTCTGTACCTTTATCTGTCT

GCCACTGACTCTTCCTTTAAGCAAAAACCCTTTTGTTTTTAGAAACTGAAATCCCAGAAC

TACAGTC

>3403 len=127 tot-seq=2

GTCCCCACAGCCAAGCCCGTGCCCTGGCTGCCCCCTCCCACTGGCCCCAGCCTCCGCTTG

CTCCAATCTTCTCTCCTCAGATTCCCAGTTTGCAAGTCCCTCTTCCTCCATCCATCCTCT

ACCACAT

>3404 len=127 tot-seq=2

ACACAGGGGTGAAGCCCAAGGGCTCTAGAGCCAGACAGGCCAAGTTCAAATTCCAACTGT

ACCACTGAACTAGCTGAGCAACATTGGGCGATGCTTTACTCCAACCACCACTCCTAGCCT

TTCCTCC

>3405 len=127 tot-seq=2

AGTCTAGGTTGCAGCTCAAAAGACAGAGTTGATAGTTTTGTCAGGCAGAGGTGTTGTGGA

GGAGTGTATCATTGCTGAGGAGGTCTATGATGGATGGTAGAGCAGCAACTAAGCTGGCCT

CAGAAGT

>3406 len=127 tot-seq=2

CTGAACCAGCCAAGTAGCGTGCAGGATGACGGCCCTATGGGTTGTAAACTGCTTTTATAT

GGGAATAAACTGCATTACGTGTAATGTTTTGCATGTACCATTCGAATAAGGACCGGCTAA

TTCCGTG

>3407 len=128 tot-seq=2

CTGTGGGCACGTTGGGCAGGTCTGCCAGACAACCTAGAGTTATCTGTGTCCTTGATCTTG

GTCTTTTGGTGGTATAGGTGGAGATGATAGCGTGTGAACAAGCATGTGAGTGTGCTTTAC

TCCAGGGG

>3408 len=128 tot-seq=2

CCAGAGACAGAGGTTCTGACCCTCAGCTTCAAGGAACCAGGGCCACCAACAGGCAGGCCT

TGAGATCCTGTGACCACTCATGCCCCCAGTTCTCCCACATGACCTCCTGCAGCCACAGAG

CAGTGTGC

>3409 len=128 tot-seq=2

GAGCGAAAATTGCCCAATCCCGACACGGGGAGGCAGTGACGAGAAATAACAACTCTTGGT

TATTCTAAGAAGTGTAATGAGGATAATTTAAAGCCCTTACCGAAAGCAATTGGAGGGCAA

GTCTGGTG

>3410 len=129 tot-seq=2

GGAGCAGGTGAGAGAGCCCAGGGCAGTGAGCGAAGAGAGGCAGGAGATGGTGGCACAGGG

GATCTATTTTGAAGGTAGGACCCACAGGATTTGCTGATGGATAGGATGCAGCGGACAGAA

GACAGAAGT

>3411 len=129 tot-seq=2

GACAGGAAGAAAGAGGTGGATGGAGAGCTCCAGTGGGCCCTGGCCCCAAGACCTGATCCA

AGTACAACCACCCAGCCAAACCACGGGCGTCCGGGCAATCCAAGAGTCTGCCTCCCAATT

GGGTAAAGC

>3412 len=129 tot-seq=2

GGAAGAAGGCCATGCAGAAGGCAAGGACTGACTAGGCAATTATGTCAGCAGATCCTGGCC

TAATGGGGCATTTTTAACAGACACTTTTGCCCTCAGTGGCCAGAAACTCATAATCCAGAG

GAACTGTGC

>3413 len=130 tot-seq=2

GATAAATTTACTTGAGCTTTTCATGTAGCTGGGGTTTTATGTTTGAAATGGGAAACGTTG

GGGTTGGTTGCCAATCATATTCCTGAGGAGAGGTGTCAATCAGCTGCTGCTGAGACACAA

GCTGCACACC

>3414 len=130 tot-seq=2

GATGGAGCAACGCCGCGTGAGTGAAGAAGGGCTTCGGCTCGTAAAGCTCTGTTGTTGGTG

AAGAAGGAAAGGGGTAGTAAATGAAATTTGTTTGACGGTAATCAATTAGAAAGTCACGGC

TAACTACGTG

>3415 len=130 tot-seq=2

GCAGCGTTAGCAGGGGACAGAGCTCCCTCTAGTGGGCCTGGATGGCAAAGATGGTTCAGA

GTGGATGGTGCTGCGGGCACTGGGAGCACAGGTATGCCTAGCAATTGGGGAACAGGCAGA

GGAAGAGAAG

>3416 len=130 tot-seq=2

CATTCACCCATTTCACACAAGGGAAAATGAAAGCTCAGAGAGGGACAGTGGTGTGCCTCA

GGCCACACAGCCAGTGTGTGGCAGAGCTGGGACTTGAACCAGATCTGTGGCTCAGAGTCC

ACGAATTCCA

>3417 len=130 tot-seq=2

GAGAGAGAGCAAAGGGGGGAGCTGCCAAACACTTTTATACAATCAGATTTTGTGAAAACT

CTCCCTCATATCATGAGAACAGTATGGGAGAGCCCACCCCCATAATTCAATCACCTCCCA

CCAGGTCCCT

>3418 len=131 tot-seq=2

TGATGGAGCAACGCCGCGTGAGTGAAGAAGGGTTTCGGCGCGTAAAGCTCTGTTGTTGGT

GAAGAAGGACAGGGGGAGTAACTGACCGTGGTTTGACGGTAAGCAAGTAGAAAGTCACGG

CTAACTACGTG

>3419 len=131 tot-seq=2

CCGCCAGTGTGGAACAGCTTGCTTCTTGGAGGAATCAGTATTTTGACCGCTCTTTAGACA

TATCCCGCAGCCTGGCTCCGAGGCAGAACTACGCCCGGCAGCCTGGCCTGTGGACCCCTC

CTCCGGCACCC

>3420 len=131 tot-seq=2

TAGTACTAGTGGCAAAGGGACAGACAGCCAAGGAAGAAAGAGTAGCTCCACTCCCGTCGG

TCCTTTCCCTTGGGGAAGAGGAAGGAGAGAGGAGAACAGCAGAATACGCGGCTCACAGAG

GCAAGGAAAGA

>3421 len=132 tot-seq=2

AGAAATGTATTAGGGGCTTGATCATTCTCTGTCTGAGTGGGCCACAGCCAGAGACTTTGT

GACCTCTGACTATTAGGAAGTTTACAAGCCAAGGTACACACGCAAGTGTCTACTCCCCCT

GGTAAAGAAGCC

>3422 len=132 tot-seq=2

CCTTACTTTTCTCATGAGAAAATGTGATTTTTGACCTTAAAAATATTGGCATGCTGAAAT

GTTGGAGATTAATTTTAACAAGTGTATGACCGTTTGTATGATTGTTTATTTTGGTGTCAT

GTTTTGCATTCC

>3423 len=132 tot-seq=2

CTGATGGAGCAACGCCGCGTGAGTGAAGAAGGGTTTCGGCTCGTAAAGCTCTGTTGTTGG

AGAAGAACGTGAGTGGGAGTAACTGTTCACAGAGTGACGGTATCCAACCAGAAAGGGACG

GCTAACTACGTG

>3424 len=132 tot-seq=2

CTGATGGAGCAACGCCGCGTGAGTGAAGAAGGTTTTCGGGTCGTAAAGCTCTGTTGTTGA

AGAAGAACGTGAGTGAGAGTAACTGTTCACGAAGTGACGGTATTCAACCAGAAAGTCACG

GCTAACTACGTG

>3425 len=132 tot-seq=2

CTGATGGAGCAACGCCGCGTGAGTGAAGAAGGGTTTCGGATCGTAAAGCTCTGTTGTTAG

AGAAGAACGCGAGTGAGAGTAACTGTTCATTCAATGACGGTATCCAACCAGAAAGTCACG

GCTAACTACGTG

>3426 len=132 tot-seq=2

AAAGGGGACTTCACAGAGCGAGAGGAGGAGCCGGCAGGCTGTGCGCCCCTATCCAGGGCT

GTCAATCTTCCTCTTTCACAAGCACTAATTTTCTTGATTTTTCTGCAAGCCCAGATCTAC

TGCTGAACTACC

>3427 len=132 tot-seq=2

CTGAGGGAGCAACGCCGCGTGAGTGAAGAAGGGTGGCGTCGCGGAAAGCGCGGTTGTTGG

TGAAGAAGGACAGGGGTAGTAACTGACCTTTGTTTGACGGTAATCAATTAGAAAGTCACG

GCTAACTACGTG

>3428 len=132 tot-seq=2

TAGGGAATCTTCCACAATGGACGCAAGTCTGATGGAGCAACGCCGCGTGAGTGAAGAAGG

GTTTCGGCTCGTAAAACTCTGTTGTTGGGGAAGAAGGACAGGGGTAGTGACTGACCTTTG

TTTGGCGGGAAT

>3429 len=132 tot-seq=2

GCTCTAATGAATGTGCTCCTGACACCCCTGGTTGGGACTCTCAGGCAAGCCGTACCCCTG

AGTAAGGGCGGGTGCCAGAGTCGGCCTGCAGCGTCGGGAGGGCACAACCCCAACTGGCCT

ATTCCAGACCTC

>3430 len=132 tot-seq=2

CGAAGTGGAGGGACTCTCCACTCGGTAAGTAGGTTCATTTAGAGTGCCTTGTCTCGTCGA

TCGCTGGGAGCCTCCAGTGGCAAGCCGAGGACTCCAAAGCTGCCTGTGTACTTGTGAGAG

AGAGTGCTGTGC

>3431 len=132 tot-seq=2

CCCCCCTTTGCCTCCAAACCCCAGTGCATTCCCAGCTTTTTCTTGGTCTCCACACTCCAG

TCCAAGCCTCTGTCCTGTCCCCAGAATTCCCTGAAGAAGGTCCAGGGAGAGGGCTGTGAG

TTCTCTATAAGA

>3432 len=132 tot-seq=2

AGGAAAGAGGAAGAAGAAGGAGAGAGAGAGTGATCCAGAGTAGGAAGCCCCAGAGAATTA

GCCAAGGTGCCACCCGGGCTATGAGGGAAGTGGGGAGCTGGTGGAGAAGAAAGGGGGCGG

AAAGCCACAGGG

>3433 len=133 tot-seq=2

TGGCTTCTAGAAACTTCCGGAGAGGAGTCAGAGGCCCAGGTAGAGGGCATCTAAGGAGAA

TCCTTCCTGCCCAGCACCCCATAAAACGAGGCAGAGCATGACCTGTGTGATGGTCTGTTA

ACAGCCTGGAGGC

>3434 len=133 tot-seq=2

AAAGGATGAGTCAGCTGGTTCCCTCCCAGGTGACTGTGAAAAGTCACACAAGGTAGTGAA

TGGTAGATGCGCTCCTTACAAGCTGTGATATCCTATGAAGCTATTAATTTTTATCCCGTG

GTTTCTCACATTA

>3435 len=133 tot-seq=2

GATGGAGCAACGCCGCGTGAGTGAAGAAGGGTTTCGGCTCGTAAAGCTCTGTTGTTGGTG

AAGAAGAACGTGCGTGAGAGTAACTATTCACGCAGTGACGGTATTCAACCAGAAAGTCAC

GGCTAACTACGTG

>3436 len=133 tot-seq=2

CCTGGACAATTGGAGCATGTAATGACTATGGATGCAATGGTTCAGATGCGCAGTGGACGA

CGAGCATTAGCTCCAGCCTCATGGAGGAGACCCACAAGCGCCCCCGCTGCAGAACCTTCT

GCACGGCCTCTGT

>3437 len=133 tot-seq=2

TGACCCCTGTCACAACCGTGCCGGGGTCTCAGGCCCACTCTCCACCGCGGGGTCCCCGTG

TCGCCGCGGCCCGTTTGTATTCAGATCAGTCTGTGAGGAAGGGTCGAGGCGTGTGGTCCA

CCCGTGGCCCGCA

>3438 len=133 tot-seq=2

AGAGAGAGTTGTATTTAGCTAGTGTTATGGTTTCACTAGGGCTGCTGAAACAAAGTGCCA

TAAAGTGGGTGGCTTTCACAATAGACGTCTACTGTCTCACAGTTCTGGAGGCTAGAAGTC

TGAGAACAAGGTG

>3439 len=133 tot-seq=2

TTAGGAATCTTCCGCAATGGGCGAAAGCCTGACGGAGCGATACCGCTTGAAGGACGAAGC

CCTTCGGGGTGTAAACTTCTGAACTCGGGACGATAATGACGGTACCGAGGTAATAGCACC

GGCTAACTCCGTG

>3440 len=134 tot-seq=2

TAGGGAATCTTCCGCAATGGGGAAACCCTGATGCAGCGACGCCGCGTGAAGGAAGAAGTA

TCTCGGTATGTAAACTTCTATCAGCAGGGAAGAAAATGACGGTACCTGACTAAGAAGCCC

CGGCTAACTACGTG

>3441 len=134 tot-seq=2

AACGGCTGAGGCCACCTTGCTGGTTCTGTCAGCTCCGACCCCAAAGCTTTTGGGGCCTCA

TTCTGTACCATACCCCAGTGCCCACGAATGCCAGCAGCCATGGCAGCTCTTCTTCACAGT

CATTCTTTCAGCCA

>3442 len=134 tot-seq=2

TAGGGAATCTTCCACAATGGATGCAAGTCTGATGGAGCAACGCCGCGTGATTTAGAAGGC

CTTCGGGTTGTAAAAATCTTTTGTATGGGAAGAAAATGACAGTACCATACGAATAAGGAC

CGGCTAATTACGTG

>3443 len=134 tot-seq=2

TGGGGAATATTGGACAATGGGGGGAACCCTGATCCAGCAATGCCGCGTGAGTGAGAAGGC

CTTCGGGTTGTAAAGCTCTTTTAGATGGGAAGATAATGACGGTACCATCAGAATAAGCAC

CGGCTAACTTCGTG

>3444 len=134 tot-seq=2

TGGGGAATATTGCACAATGGGGGAAACCCTGATGCAGCGACGCCGCGTGAACGAAGAAGG

TATTCGTATCGTAAAGTTCTGTCCTATGGGAAGATAATGACAGTACCATAGAAGAAGCAC

CGGCTAACTCCGTG

>3445 len=134 tot-seq=2

TGTTCTGGAATCCTTTGTGAGGGACAAATATTCAGACCCTCGTAGCAATGTTATGGAATC

CCATGTGCTATATACACCTTCAGACCCCCGTAGCAGTGTTCTCGAATGCTATGTGAGGGA

CAAATAATCAGAAC

>3446 len=134 tot-seq=2

TGGGGAATCTTCCGCAATGGACGAAAGTCTGACGGAGCAACGCCGCGTGAGTGATGACGG

TCTTCGGATTGTAAAGCTCTGTTAATCGGGACGAATGGTCGGTGTGCAAATAGAAAGTCA

CGGCTAACTACGTG

>3447 len=134 tot-seq=2

CAGGCTGGAGGTGACAGGGCGTGCCTGTATGCCAGGGTGTGGACAGAGACCTCGGGAGGC

TCCTCCACAGCTGACAGAGGTGCAAGTGTGTTACACAGGGTCCAGGGCTTATCCAGCAGG

AGGCAGAACCCATT

>3448 len=134 tot-seq=2

TAGGGAATCTTCCACAATGGGGGAAACCCTGATGCAGCGACGCCGCGTGATTTAGAAGGC

CTTCGGGTTGTAAAAATCTTTTTATGGGATGAAAATGACAGTACCATAAGAATAAGGTC

CGGCTAAATACGTG

>3449 len=134 tot-seq=2

TAGGGAATCTTCCACAATGGACGCAAGTCTGATGCAGCGACGCCGCGTGATTTAGAAGGC

CTTCGGGTTGTAAAAATCTTTTTATGGGATGAAAATGACAGTACCATAAGAATAAGGTC

CGGCTAAATACGTG

>3450 len=134 tot-seq=2

TTTTTTCAAGCAGAAGACGGCATACGAGATAAGAGAGTGACTGGAGTTCAGACGTGTGTC

TTCCGATCTCTTACGGGAGGCAGCAGCCGCGGTAATAAAGAGATCGGAAGAACGTCGTGT

AGGGAAGAGTGTGC

>3451 len=134 tot-seq=2

GGCCACTGGTCCTCAGTCACACAGCGGCAGGGTGAGCTCCTGATCTCCAGCAGGCCCCCT

CCATCTGCACGTCCATCGCTGGAATGTAGCAGGTGCTGGGCTAGGCCCTAATAACATAGG

TTCATCCCCCCACC

>3452 len=134 tot-seq=2

CCCACAGACTCGGGCTCTGGAGGTCTACAGAGCAGCTGGGAGGGTGATGCCTAGGCGGGG

GCGGAGATGCCAGGCCCCTGGGCTGCAGGTGGAGGTAAATGGGGAAGGGAGGCCGGGGGT

CTCTGGGCCTTTCG

>3453 len=134 tot-seq=2

GCGCGCAAATTACCCAATCCCGACACGGGGAGGTAGTGACAATAAATAACAATATAGGGC

CTTACAGGTCTTATAATTGGAATGAGTACAATTTAAATCCCTTAACGAGGAACAATTGGA

GGGCAAGTCTGGTG

>3454 len=134 tot-seq=2

CCGGACCGAGCAACGCCGCGGGAGGGAAGAAGGGTTGCGGATCGTAAAGCTCTGTTGTAA

GAGAAGAACGTGTGTGAGAGTGGAAAGTTCACACAGTGACGGTAACTTACCAGAAAGGGA

CGGCTAACTACGGG

>3455 len=135 tot-seq=2

TAGGGAATTTTCGTCAATGGGCGCAAGCCTGAACGAGCGATGCCGCGTGAGTGAAGAAGT

ATTTCGGTATGTAAAGCTCTATCAGCAGGGAAGAAAATGACGGTACCTGACTAAGAAGCC

CCGGCTAACTACGTG

>3456 len=135 tot-seq=2

TGGGGAATATTGCACAATGGGGGAAACCCTGATGCAGCGACGCCGCGTGAGCGATGAAGT

ATTTCGGTATGTAAAGCTCTATCAGCAGGGAAGAAAATGACGGTGCCTGACTAAGAAGCA

CCGGCTAACTCCGTG

>3457 len=135 tot-seq=2

TAGGGAATCTTCCGCAATGGGCGAAAGCCTGACGCAGCGACGCCGCGTGAAGGATGAAGT

ATTTCGGTATGTAAACTTCTATCAGCAGGGAAGAAAATGACGGTACCTGACTAAGAAGCC

CCGGCTAACTACGTG

>3458 len=135 tot-seq=2

TGGGGAATATTGCACAATGGGCGCAAGCCTGATGCAGCCATGCCGCGTGAGTGAAGAAGT

ATTTCGGTATGTAAAGCTCTATCAGCAGGGAAGAAAATGACGGTACCTGATTAAGAAGCC

CCGGCTAACTACGTG

>3459 len=135 tot-seq=2

TGGGGAATATTGCACAATGGGCGCAAGCCTGATGCAGCCATGCCGCGTGTAGGAAGAAGG

TTTTCGGATTGTAAACTCCTGTCGTTAGGGACGATAATGACGGTACCTAACAAGAAAGCA

CCGGCTAACTACGTG

>3460 len=135 tot-seq=2

TGGGGAATATTGCACAATGGGCGCAAGCCTGATGCAGCAACGCCGCGTGAACGATGAAGG

TCTTCGGATCGTAAAGTTCTGTTGCAGGGGAAGATAATGACGGTAATCAATTAGAAAGTC

ACGGCTAACTACGTG

>3461 len=135 tot-seq=2

TGGGGGATATTGCACAATGGGCGAAAGCCTGATGCAGCGACGCCGCGTGAGGGAAGACGG

TTTTCGGATTGTAAACCTCTGTCTTCGGGGACGAAAATGACGGTACCCGAGGAGGAAGCC

ACGGCTAACTACGTG

>3462 len=135 tot-seq=2

TGGGGAATATTGCACAATGGGGGAAACCCTGATGCAGCGACGCCGCGTGAGCGAAGAAGT

ACCTCGGTATGTAAAGCTCTATCAGCAGGGAAGAAGATGACGGTACCTGACCAAGAAGCT

CCGGCTAAATACGTG

>3463 len=135 tot-seq=2

TGGGGAATATTGCAGAATGGAGGGAACTCTGATGCAGCGACGCCGCGTGAGCGAAGAAGT

ATTTCGGTATGTAAAGCTCTATCAGCAGGGAAGATAATGACGGTACCTGACTAAGAAGCT

CCGGCTAAATACGTG

>3464 len=135 tot-seq=2

TAGGGAATCTTCGGCAATGGACGGAAGTCTGACCGAGCAACGCCGCGTGAGTGAAGAAGG

TTTTCGTATCGTAAAGCTCTGTCCTATGGGAAGATAATGACGGTACCATAGGAGGAAGCT

CCGGCTAACTACGTG

>3465 len=135 tot-seq=2

TGGGGAATATTGCACAATGGGGGAAACCCTGATGCAGCGACGCCGCGTGAGCGATGAAGG

AATTCGTTTCGTAAAGCTCTGTCCTAGGGGAAGATAATGACAGTACCCTTGGAGGAAGCC

CCGGCTAAATACGTG

>3466 len=135 tot-seq=2

TGGGGAATATTGCACAATGGAGGGAACTCTGATGCAGCGACGCCGCGTGAACGAAGAAGG

CTTTCGAGTCGTAAAGTTCTTTTATATGGGAAGATAATGACAGTACCATACGAATAAGGA

CCGGCTAATTACGTG

>3467 len=135 tot-seq=2

TGGGGAATATTGGGCAATGGGCGCAAGCTTGACCCAGCAACGCCGCGTGAAGGAAGAAGG

CTTTCGGGTTGTAAACTTCTTTGATCAGGGACGAAAATGACGGTACCTGAAGAACAAGCC

ACGGCTAACTACGTG

>3468 len=135 tot-seq=2

TGGGGGATATTGCACAATGGGGGAAACCCTGATGCAGCGACGCCGCGTGAAGGAAGAAGT

ATCTCGGTATGTAAAGCTCTATCAGCAGGGAAGATAATGACGGTACCTGACTAAGAAGCT

CCGGCTAAATACGTG

>3469 len=135 tot-seq=2

TGGGGAATATTGCGCAATGGGGGAAACCCTGACGCAGCGACGCCGCGTGAACGAAGAAGG

TCTTCGGATTGTAAAGTTCCGTCCTGGGTGAAGATAATGACGGTAACTCAGGAGGAAGCC

CCGGCTAACTACGTG

>3470 len=135 tot-seq=2

TGGGGAATATTGGACAATGGGCGCAAGCCTGATCCAGCCATGCCGCGTGAGTGATGAAGG

CCTTCGGGTTGTAAAGCTCTTTCAGATGGGACGATGATGACGGTACCATCAGAAGAAGCC

CCGGCTAACTTCGTG

>3471 len=135 tot-seq=2

TGGGGAATCTTGCACAATGGGCGAAAGCCTGATGCAGCGACGCCGCGTGAGTGAAGAAGT

ACTTCGGTATGTAAAGCTCTATCAGCAGGGACGATAATGACGGTACCTGACTAAGAAGCC

CCGGCTAACTACGTG

>3472 len=135 tot-seq=2

TGGGGAATATTGCACAATGGGGGAAACCCTGATGCAGCGACGCCGCGTGGAGGAAGAAGG

CCCTCGGGTTGTAAACTCCTGTCTTTGGGGACGATAATGACGGTACCCAAGGAGGAAGCC

ACGGCTAACTACGTG

>3473 len=135 tot-seq=2

TGGGGAATATTGGACAATGGGGGAGACCCTGATCCAGCCATGCCGCGTGAGTGACGAAGG

CCTTCGGGTTGTAAAGCTCTTTTACATGGGAAGATAATGACGGTACCATGAGAATAAGCA

CCGGCTAACTTCGTG

>3474 len=135 tot-seq=2

TGGCAGGAGAGGGGGGTTGAGGGGAATGAGGGATTGATGGGAACTCAACTACAGAGCTCA

GTTGGGAAACTCACACCCCATTGGAGCTGGGATTGATTGCTGGAACTTTCTCACCCTTCT

CAGTTTGTCTCAGAG

>3475 len=135 tot-seq=2

TAGGGAATCTTGCGCAATGAGGGAAACCTTGACGCAGCGACGCCGCGTGCGGGATGAAGG

CCTTCGGGTTGTAAACCGCTTTCAGCAGGGACGAAATTGACGGTACCTGCAGAAGAAGGC

CCGGCCAACTACGTG

>3476 len=135 tot-seq=2

TGGGGAATCTTGGACAATGGGGGCAACCCTGATCCAGCGACGCCGCGTGGGTGATGAAGG

CCTTCGGGTTGTAAAGCCCTTTTGTCGGGGACGATGATGACGGTACCCGGCGAATAAGCA

CCGGCTAACTCCGTG

>3477 len=135 tot-seq=2

TGGGGAATATTGCACAATGGGCGCAAGCCTGATGCAGCAACGCCGCGTGAACGATGAAGG

TCTTCGGATCGTAAAGTTCTGTTGCAGGGGAAGATAATGACGGTATCTGAATAAGAAGCC

CCGGCTAACTACGTG

>3478 len=135 tot-seq=2

TAGGGAATCTTCGGCAATGGACGGAAGTCTGACCGAGCAACGCCGCGTGAGCGATGAAGG

CTTTCGAGTCGTAAAGCTCTGTCCTATGGGAAGATAATGACGGTACCATAGGAGGAAGCC

CCGGCTAACTACGTG

>3479 len=135 tot-seq=2

TGGGGGATATTGGACAATGGAGGTAACTCTGATGCAGCGACGCCGCGTGAGCGAAGAAGG

CTTTCGAGTCGTAAAGCTCTGTCCTATGAGAAGATAATGACGGTATCATAGGAGGAAGCC

CTGGCTAAATACGTG

>3480 len=135 tot-seq=2

TGGGGAATATTGCACAATGGGCGCAAGCCTGATGCAGCCATGCCGCGTGTATGAAGAAGG

CTTTCGAGTCGTAAAGTTCTTTTATATGGGAAGATAATGACGGTACCATAAGAAAAAGCC

CCGGCTAACTACGTG

>3481 len=135 tot-seq=2

TGGGGGATATTGCACAATGGGGGAAACCCTGATGCAGCGACGCCGCGTGAGGGAAGAAGG

TTTTCGGATTGTAAACCTCTGTCCCCGGTGAAGATAATGACGGTAACCGGGGAGGAAGCC

ACGGCTAACTACGTG

>3482 len=135 tot-seq=2

TGGGGAATATTGCACAATGGGGGAAACCCTGATGCAGCGACGCCGCGTGAGTGAAGACGG

CCTTCGGGTTGTAAAGCTCTGTTGTGGGGGACGATAATGACGGTACCCCATCAGAAAGCC

ACGGCTAACTACGTG

>3483 len=135 tot-seq=2

GCGCGCAAATTACCCAATCCCGACACGGGGAGGTAGTGACAATAAATAACGATACAGGGC

CCTTATGGGTCTTGTAATTGGAATGAGTACAATGTAAATACCTTAACGAGGAACAATTGG

AGGGCAAGTCTGGTG

>3484 len=135 tot-seq=2

TGAGGAATATTCCACAATGGGCGAACGCCTGATGGAGCAATGCCGCGTGAGCGAAGAAGT

ATTTCGGTATGTAGAGCTCTATCAGAAGGGAAGAAAATGACGGTACCTTACTAAGAAGCT

CCGGCTAAATACGTG

>3485 len=135 tot-seq=2

TGAGGAATATTCCACAATGGGCGAAAGCCTGATGGAGCAATGCCGCGTGCAGGATGAAGG

CCCTCGGGTCGTAAACTGCTTTTATTAGAGAAGAATATGACGGTACCTTACTAAGAAGCT

CCGGCTAAATACGTG

>3486 len=135 tot-seq=2

TGGGGAATATTGCACAATGGGGGAAACCCTGATGCAGCGACGCCGCGTGAGTGAAGACGG

CCTTCGGGTTGTAAAGCTCTGTTGCAGAGGACGATAATGACGGTACTCTGCTAGAAAGCC

ACGGCTAACTACGTG

>3487 len=135 tot-seq=2

TGAGGAATATTGGTCAATGGGGGAAACCCTGACGCAGCAACGCCGCGTGCGGGATGAAGG

CCTTCGGGTTGTAAACCGCTTTCAGCAGGAAAGACATCGACGGTACCTGCAGAAGAAGCC

CCGGCTAACTACGTG

>3488 len=135 tot-seq=2

TGGGGAATTTTGCACAATGGAGGAAACTCTGATGCAGCGACGCCGCGTGAGCGATGAAGG

TCTTCGGATTGTAAAGCTCTGTCCTTGGTGAAGATCGTGACGGTAATCAAGGAGGAAGCC

CCGGCTAACTACGTG

>3489 len=135 tot-seq=2

TGGGGAATCTTGCACAATGGGCGAAAGCCTGATGCAGCGACGCCGCGTGCGGGATGAAGG

CCTTCGGGTTGTAAAGTTCTATCAGCAGGGAAGAAAATGACGGTACCTGACTAAGAAGCC

CCGGCTAACTACGTG

>3490 len=135 tot-seq=2

TAGGGAATCTTCGGCAATGGGGGGAACCCTGACCGAGCAACGCCGCGTGAACGAAGAAGG

TATTCGTATCGTAAAGTTCTGTCCTATGGGGAGATAATGACAGTACCATAGAAGAAAGCT

CCGGCTAAATACGTG

>3491 len=135 tot-seq=2

TGGGGGATATTGCACAATGGGGGGAACCCTGATGCAGCGACGCTGCGTGAGTGAAGAAGT

GCCCCGGCATGTAAAGCTCTGTCAGCAGGGAAGAAGATGACGGTACCTGACCAAGAAGCA

CCGGCTAACTCCGTG

>3492 len=135 tot-seq=2

TGAGGAATTTTTCACAATGGACGAAAGTCTGATGGAGCAATACCACGTGAACGATGAAGG

TCTTCGGATCGTAAAGTTCTGTTGCAGGGGAAGATAATGACGGTACCCTGTGAGGAAGCC

CCGGCTAACTACGTG

>3493 len=135 tot-seq=2

TGAGGAATATTGGTCAATGGGAGAGATCCTGAACCAGCAACGCCGCGTGAGGGAAGACGG

TTTTCGGATTGTAAACCTCTGTCCTCTGTGAAGATGATGACGGTAGCAGGGGAGGAAGCT

CCGGCTAACTACGTG

>3494 len=135 tot-seq=2

TGGGGAATATTGCACAATGGGCGCAAGCCTGATGCAGCAACGCCGCGTGAACGATGAAGG

TCTTCGGATCGTAAAGTTCTGTTGCAGGGGAAGATAATGACGGTACCGGAATAGAAAGCC

ACGGCTAACTACGTG

>3495 len=135 tot-seq=2

TAAGGGATATTGGTCAATGGGGGAAACCCTGATGCAGCGACGCCGCGTGAGTGAAGAAGT

ATTTCGGTACGTAAAGCTCTATCAGCAGGGAAGAAAATGACGGTACCTGAGTAAGAAGCC

CCGGCTAACTACGTG

>3496 len=135 tot-seq=2

TGGGGAATCTTGCGCAATGGGCGAAAGCCTGACGCAGCAACGCCGCGTGCGGGATGAAGG
CCTTCGGGTTGTAAACCGCTTTCAGCAGGGAAGAAATTGACGGTACCTGCAGAAGAAGCC
CCGGCCAACTACGTG
>3497 len=135 tot-seq=2
TGGGGAATATTGCACAATGGGGGAAACCCTGATGCAGCGACGCCGCGTGAACGAAGAAGG
TATTCGTATCGTAAAGTTCTGTCCTATGGGAAGATAATGACGGTACCTGAATAAGAAGCC
CCGGCTAACTACGTG
>3498 len=135 tot-seq=2
TGGGGAATATTGCACAATGGGCGCAAGCCTGATGCAGCGACGCCGCGTGAGGGATGGAGG
CCTTCGGGTTGTAAAGCTCTATCAGCAGGGAAGAAGATGACGGTACCTGAATAAGAAGCC
CCGGCTAACTACGTG
>3499 len=135 tot-seq=2
TGGGGAATATTGCACAATGGGGGAAACCCTGATGCAGCAACGCCGCGTGAGTGAAGAAGG
TCTTCGGATTGTAAAGCTCTGTTCTTGGTGAAGATAATGACGGTAGCCAAGGAGAAAGCT
CCGGCTAACTACGTG
>3500 len=135 tot-seq=2
TGGGGAATATTGCACAATGGGGGAAACCCTGATCCAGCCATGCCGCGTGAGTGATGAAGG
CCTTAGGGTTGTAAAGCTCTTTCACCGATGAAGATAATGACGGTAGTCGGAGAAGAAGCC
CCGGCTAACTTCGTG
>3501 len=135 tot-seq=2
TGGGGAATATTGCACAATGGGGGAAACCCTGATGCAGCAACGCCGCGTGAGTGAAGAAGG
TTTTCGGTTCGTAAAGCTCTATCTGTGGGGAAGAAAATGACGGTACCCAAGGAGGAAGCC
ACGGCTAACTACGTG
>3502 len=135 tot-seq=2
TGGGGAATATTGCGCAATGGGGGAAACCCTGACGCAGCGACGCCGCGTGCGGGATGAAGG
CCTTCGGGTTGTAAAGCTCTGTCTTTGGGGAAGATAATGACGGTACCCAAGGAGGAAGCC
ACGGCTAACTACGTG
>3503 len=135 tot-seq=2
TGGGGAATATCGGACAATGGGCGCAAGCCTGATCCAGCCATGCCGCGTGAGTGATGAAGG

CCTTAGGGTTGTAAAGCTCTTTCACCGATGAAGATAATGACGGTAGTCGGAGAAGAAGCC

ACGGCTAACTACGTG

>3504 len=135 tot-seq=2

TGGGGAATCTTGCACAATGGGGGCAACCCTGATGCAGCGACGCCGCGTGAGCGATGAAGG

CTTTCGAGTCGTAAAGCTCTGTCCTATAGGAAGATAATGACGGTACTATAGGAGGAAGCC

CCGGCTAACTACGTG

>3505 len=135 tot-seq=2

TGAGGAATATTGGTCAATGGGCGGGAGCCTGAACCAGCCAAGTCGCGTGAGTGAAGAAGT

ATTTCGGTATGTAAAGCTCTATCAGCAGGGAAGAAAATGACGGTACCTGACTAAGAAGCC

CCGGCTAACTACGTG

>3506 len=135 tot-seq=2

TGGGGAATCTTCGGCAATGGACGCAAGCCTGATGCAGCAACGCCGCGTGAACGATGAAGG

TCTTCGGGTCGTAAAGTTCTGTTGCAGGGGAAGATAATGACGGTACCCTGTGAGGAAGCC

CCGGCTAACTACGTG

>3507 len=135 tot-seq=2

TGGGGAATATTGCGCAATGGGGGAAACCCTGACGCAGCAACGCCGCGTGAACGAAGAAGG

TATTCGTATCGTAAAGTTCTGTCCTATGGGAAGATAATGACAGTACCACAGAAGAAAGCT

CCGGCTAAATACGTG

>3508 len=135 tot-seq=2

TGGGGAATATTGGACAATGGGCGCAAGCCTGATCCAGCCATGCCGCGTGAGTGATGAAGG

CCCTAGGGTTGTAAAGCTCTTTCACCGGAGAAGATAATGACGGTATCCGGAGAAGAAGCC

ACGGCTAACTACGTG

>3509 len=135 tot-seq=2

TGGGGGATATTGCACAATGGGGGAAACCCTGATGCAGCAACGCCGCGTGAATGATGAAGG

TCTTCGGATTGTAAAATTCTGTTCTTGGTGAAGATAATGACGGTAGCCAAGGAGAAAGCT

CCGGCTAACTACGTG

>3510 len=135 tot-seq=2

TGGGGAATATTGCACAATGGGCGCAAGCCTGATGCAGCAACGCCGCGTGAACGATGAAGG

TCTTCGGATCGTAAAGTTCTGTTGCAGGGGAAGATAATGACGGTATCTAACCAGCAAGTC

ACGGCTAACTACGTG

>3511 len=135 tot-seq=2

TGGGGAATCTTCCGCAATGGACGAAAGTCTGATGCAGCGACGCCGCGTGAGTGAAGAAGT

ATTTCGGTATGTAAAGCTCTATCAGTAGGGAAGATAATGACGGTACCTACATAAGAAGCC

CCGGCTAAATACGTG

>3512 len=135 tot-seq=2

TGGGGAATCTTCCGCAATGGACGAAAGTCTGACGGAGCAACGCCGCGTGGAGGAAGAAGT

ATCTCGGTATGTAAACTTCTATCAGCAGGGAAGAAAATGACGGTACCTGACTAAGAAGCC

CCGGCTAACTACGTG

>3513 len=135 tot-seq=2

TGGGGAATATTGCACAATGGAGGAAACTCTGATGCAGCGACGCCGCGTGAGTGAAGAAGT

ATTTCGGTATGTAAAGCTCTATCAGCAGGGAAGACAGTGACGGTACCTGACTAAGGAGCT

CCGGCTAGATACGTG

>3514 len=135 tot-seq=2

TAGGGAATCTTCCACAATGGGGGAAACCCTGATGCAGCGACGCCGCGTGAACGAAGAAGG

TGTTCGTATCGTAAAGTTCTGTCCTATGGGAAGATAATGACAGTACCATAGAAGAAAGCT

CCGGCTAAATACGTG

>3515 len=135 tot-seq=2

TGGGGAATTTTGCACAATGGGGGAAACCCTGATGCAGCGACGTCGCGTGAACGATGAAGG

CCTTCGGGTTGTAAAGTTCTGTCCTTGGCGAAGATAATGACAGTAGCCAAGAAGCAAGCT

CCGGCTAAATACGTG

>3516 len=135 tot-seq=2

TAGGGAGTCTTCGGCAATGGGGGCAACCCTGACCGAGCAACGCCGCGTGAGTGAAGAAGG

TTTTCGGATCGTAAAGCTCTGTTGTAAGAGAAGAACGAGCGTGAGAGTGGAAAGTTCACA

CTGTGGCGGTAACTT

>3517 len=135 tot-seq=2

TGGGGAATATTGCACAATGGGCGCAAGCCTGATGCAGCAACGCCGCGTGAACGATGAAGG

TCTTCGGATCGTAAAGTTCTGTTGCAGGGGAAGATAATGACGGTACCGCGTGAATAAGGA

CCGGCTAATTCCGTG

>3518 len=135 tot-seq=2

TGGGGAATTTTGGACAATGGGGGCAACCCTGATCCAGCAACGCCGCGTGAACGATGAAGG

TCTTCGGATCGTAAAGTTCTGTTGCAGGGGAAGATAATGACGGTACCCTGTGAGGAAGCC

CCGGCTAACTACGTG

>3519 len=135 tot-seq=2

GGGGAATATTGCACAATGGGGGAAACCCTGATGCAGCGACGCCGCGTGCGGGATGAAGGC

CCTCGGGTCGTAAACCGCTTTCAGCAGGGATGAGACAAGACGGTACCTGCAGAAGAAGCC

CCGGCTAACTACGTG

>3520 len=135 tot-seq=2

TGGGGAATATTGCACAATGGGGGAAACCCTGATGCAGCGACGCCGCGTGAAGGAAGAAGT

ATCTCGGTATGTAAACTTCTATCAGCAGGGAAGAAAATGACGGTACACAACAAGGAAGTG

ACGGCTAACTACGTG

>3521 len=135 tot-seq=2

TGAGGAATATTCCACAATGGGCGAAAGCCTGATGGAGCAATGCCGCGTGCAGGATGAAGG

TCTTCGGATCGTAAAGTTCTGTTGCAGGGGAAGATAATGACGGTACCCTGTGAGGAAGCC

CCGGCTAACTACGTG

>3522 len=135 tot-seq=2

TGGGGAATATTGCACAATGGGGGGAACCCTGATGCAGCAACGCCGCGTGAGTGAAGAAGT

ATTTCGGTATGTAAAGCTCTATCAGCAGGGAAGATAATGACGGTACCTGACGAATAAGCC

ACGGCTAACTACGTG

>3523 len=135 tot-seq=2

TAGGGAATCTTCCACAATGGGCGCAAGCCTGATGGAGCGACGCCGCGTGAGTGATGACGG

CCTTCGGGTTGTAAAGCTCTGTCTTCAGGGACGATAATGACGGTACCTGAGGAGGAAGCC

ACGGCTAACTACGTG

>3524 len=135 tot-seq=2

TGGGGAATATTGGACAATGGGCGAAAGCCTGATCCAGCGACGCCGCGTGAAGGAAGAAGT

ATCTCGGTATGTAAACTTCTATCAGCAGGGAAGAAAATGACGGTACCTGACTAAGAAGCT

CCGGCTAAATACGTG

>3525 len=135 tot-seq=2

TGAGGAATCTTCCACAATGGGCGAAAGCCTGATGGAGCAACGCCGCGTGCAGGATGAAGG

CCTTCGGGTTGTAAACTGCTTTTATTAGTGAAGAATATGACGGTAACTAATGAATAAGGG

TCGGCTAACTACGTG

>3526 len=135 tot-seq=2

TGAGGAATCTTCCACAATGGGGGCAACCCTGATGGAGCAACGCCGCGTGCAGGATGAAGG

CCTTCGGGTTGTAAACTGCTTTTATAAGTGAAGAATATGACGGTAACTTATGAATAAGGA

TCGGCTAACTACGTG

>3527 len=135 tot-seq=2

TGGGGAATATTGCACAATGGGGGAAACCCTGATGCAGCAACGCCGCGTGAACGAAGACGG

CCTTCGGGTTGTAAAGTTCTTTGATTGGGGAAGAAAATGACGGTACCCAAAAAACAACCC

ACGGCTAACTACGTG

>3528 len=135 tot-seq=2

TGGGGAATATTGCGCAATGGGGGAAACCCTGACACAGCGACGCCGCGTGAGTGAAGAAGT

ATTTCGGTATGTAAAGCTCTATCAGCAGGGAAGAAGATGACGGTACCTGAGTAAGAAGCC

CCGGCTAACTACGTG

>3529 len=135 tot-seq=2

TGGGGAATCTTGCGCAATGGGCGAAAGCCTGACGCAGCCATGCCGCGTGAATGATGAAGG

TCTTAGGATTGTAAAATTCTTTCAGTAGGGACGATAATGACGGTACCTACAGAAGAAGCC

CCGGCTAACTTCGTG

>3530 len=135 tot-seq=2

TGGGGAATCTTCCGCAATGGACGAAAGTCTGACGGAGCAACGCCGCGTGAGCGAAGAAGG

CCTTCGGGTCGTAAAGCTCTTTTATATGGGAAGATAATGACGGTACCATAAGAAAAAGCC

CCGGCTAACTACGTG

>3531 len=135 tot-seq=2

TGGGGAATATTGCACAATGGGGGAAACCCTGATGCAGCGACGACGCGTGAGCGAAGAAGG

CTTTCGAGTCGTAAAGCTCTGTCCTATGAGAAGATAATGACGGTACCATAAGAAAAAGCC

CCGGCTAACTACGTG

>3532 len=135 tot-seq=2

TGGGGAATCTTCCGCAATGGGCGCAAGCCTGACGGAGCAATGCCGCGTGAGTGAAGAAGT

ATTTCGGTATGTAAAGCTCTATCAGAAGGGAAGAAAATGACGGTACCTTACTAAGAAGCT

CCGGCTAAATACGTG

>3533 len=135 tot-seq=2

TGGGGAATATTGCACAATGGGGGAAACCCTGATGCAGCGACGCCGCGTGAGCGAAGAAGG

TATTCGTATCGTAAAGCTCTGTCCTATGGGAAGATAATGACGGTACCTTACTAAGAAGCT

CCGGCTAAATACGTG

>3534 len=135 tot-seq=2

TGGGGAATATTGCGCAATGGGGGAAACCCTGACGCAGCGACGCCGCGTGAGTGAAGAAGT

ATCTCGGTATGTAAAGCTCTATCAGCAGGGAAGAAAGTGGCGGTACCTGAGTAAGAAGCC

CCGGCTAACTACGTG

>3535 len=135 tot-seq=2

TGGGGAATATTGCGCAATGGGGGAAACCCTGACGCAGCGACGCCGCGTGCGGGATGAAGT

ATCTCGGTATGTAAACTTCTATCAGCAGGGAAGATAGTGACGGTACCTGACTAAGAAGCC

CCGGCTAACTACGTG

>3536 len=135 tot-seq=2

TGGGGGATATTGCACAATGGGGGGAACCCTGATGCAGCAACGCCGCGTGGGTGAAGGAGT

ACCCCGGTACGTAAAGCCCTATCGGCAGGGAAGAAGATGACGGTACCTGACTAAGAAGCC

CCGGCTAACTACGTG

>3537 len=135 tot-seq=2

TGGGGAATATTGCACAATGGGGGGAACCCTGATGCAGCAACGCCGCGTGGGTGAAGAAGT

ATTTCGGTATGTAAAGCCCTATCAGCAGGGAAGAAAATGACGGTACCTGACTAAAAAGCC

CCGGCTAAATACGTG

>3538 len=135 tot-seq=2

TGGGGAATATTGCACAATGGAGGAAACTCTGACGCAGCAACGCCGCGTGAGTGATGAAGG

TCTTCGGATCGTAAAGCTCTTTGATTGGGGACGATAATGACGGTACCCAAAGAACAAGCC

ACGGCTAACTACGTG

>3539 len=135 tot-seq=2

CAGGGAATCTTGCGCAATGGGCGAAAGCCTGACGCAGCAACGCCGCGTGGGGGATGACGG

CCTTCGGGTTGTAAACCCCTTTCAGGAGGGAAGAAATTGACGGTACCTCCAGAAGAAGCC

CCGGCCAACTACGTG

>3540 len=135 tot-seq=2

TGGGGAATCTTGCACAATGGGGGAAACCCTGATGCAGCGACGCCGCGTGAGCGATGAAGC

CCTTCGGGGTGTAAAGCTCTTTCGGCAGGGAAGATGATGACGGTACCTGCAGAAGCAGCT

GCGGCTAACTACGTG

>3541 len=135 tot-seq=2

TGGGGGATATTGCACAATGGGCGCAAGCCTGATGCAGCGACGCCGCGTGTGGGAAGACGG

CCCTCGGGTTGTAAACCACTGGCTTTGGGGACGATAATGACGGTACCCAAGGAGGAAGCT

CCGGCTAACTACGTG

>3542 len=135 tot-seq=2

TGGGGGATATTGCACAATGGAGGAAACTCTGATGCAGCAACGCCGCGTGAGGGAAGAAGG

TTTTCGGATTGTAAACCTCTGTCCTCGGTGAAGATAATGACGGTAGCCGAGGAGGAAGCC

ACGGCTAACTACGTG

>3543 len=135 tot-seq=2

TGGGGAATATTGCACAATGGGGGAAACCCTGATGCAGCGACGCCGCGTGAGCGAAGAAGG

CTTTCGAGTCGTAAAGCTCTGTCCTATGAGAAAAAAATGACGGTACCAACAGAAGAAGTG

ACGGCTAAATACGTG

>3544 len=136 tot-seq=2

GCACCAAACAAGTGAAGACCGGATAGGCTGACAGACCTCATCAAGTTTCAGTTTCCCCTT

CCCAAACCCATATCTCATTGGGGGAACTGAGGCACAGTTGTGACCCACATGAGTAGGTCT

GGATCTATTCCCCAAT

>3545 len=136 tot-seq=2

TGGGGAATTTTGGACAATGGGCGCAAGCCTGATCCAGCGACGCCGCGTGAAGGAAGAAGT

ATCTCGGTATGTAAACTTCTATCAGCAGGGAAGAAAGAGGACGGTACCTGACTAAGAAGC

CCCGGCTAACTACGTG

>3546 len=136 tot-seq=2

TGGGGAATATTGCGCAATGGGCACAAGCCTGATGCAGCAACGCCGCGTGAAGGAAGACGG

TTTTCGGATTGTAAACTTCTGTTCTTAGTGAAGAATAATGACGGTAGCTAAGGAGCAAGC

CACGGCTAACTACGTG

>3547 len=136 tot-seq=2

ACTGATAAGCAACTTGCATCATACCAACTGGCCATTGAAGATTATTTTTCGAGTAAAATA

CAGCCTTCCTTTAGAACCTCTACCCAGAAAGCAGTAACATCAATAATCTATAAAGTAGCA

CTCATTCTTGCCACCA

>3548 len=136 tot-seq=2

TGGGGAATATTGCGCAATGGGGGAAACCCTGACGCAGCGACGCCGCGTGCGGGATGAAGG

CCTTCGGGTCGTAAACCGCTTTCAGCAGGGAAGAGAGAAGACTGTACCTGCAGAAGAAGC

CCCGGCTAACTACGTG

>3549 len=136 tot-seq=2

TGGGGAATATTGCACAATGGAGGGAACTCTGATGCAGCGACGCCGCGTGCGGGACGGAGG

CCTTCGGGTCGTAAACCGCTTTCAGCAGGGAAGAGAGAAGACTGTACCTGCAGAAGAAGC

CCCGGCTAACTACGTG

>3550 len=136 tot-seq=2

GCACGCAAGTTATCCACTCAGGACCTGGGGATGTAGTGATGAAAAATAACAATACAGGAC

TCTTTCGAGGCCCTGTAATTGGAATGAGTCCACTTTAAATCCTTTAACGAGGATCCATTG

GAGGGCAAGTCTAGTG

>3551 len=136 tot-seq=2

TGGGGAATATTGCACAATGGGGGAAACCCTGATGCAGCGACGCCGCGTGGGTGAAGAAGT

ATTTCGGTATGTAAAGCCCTATCGGTAGGGAAGAAGCAAGACGGTACCTGACTAAGAAGC

CCCGGCTAACTACGTG

>3552 len=136 tot-seq=2

TGGGGAATATTGCACAATGGGGGAAACCCTGATGCAGCGACGCCGCGTGAGTGAAGAAGT

AATTCGTTATGTAAAGCTCTATCAGCAAGGAAGAAAAAAGACGGTACTTGACTAAGAAGC

CCCGGCTAAATACGTG

>3553 len=136 tot-seq=2

TGGGGAATATTACCCACTCCCGACCCGGGGAGGTAGTGACGAAAAATAACAATACAGGAC

TCTTTCGAGGCCCTGTAATTGGAATGAGTCCACTTTAAATCCTTTGACGAGGATCCATTG

GAGGGCAAGTCTGGTG

>3554 len=136 tot-seq=2

TGGGGGATATTGCACAATGGGGGGAACCCTGATGCAGCGACGCCGCGTGCAGGAAGAAGG

TTTTCGGATTGTAAACTGCTTTAGACAGGGAAGAAACAAGACAGTACCTGTAGAATAAGC

TCCGGCTAACTACGTG

>3555 len=136 tot-seq=2

TGGGGAATATTGCGCAATGGGGGCAACCCTGACGCAGCAACGCCGCGTGATTGAAGAAGG

CCTTCGGGTTGTAAAGATCTTTAATCAGGGACGAATTTTGACGGTACCTGAAGAATAAGC

TCCGGCTAACTACGTG

>3556 len=136 tot-seq=2

TGGGGAATTTTGCACAATGGGGGAAACCCTGACGCAGCAACGCCGCGTGGAGGATGACAC

TTTTCGGAGCGTAAACTCCTTTTATCAGGAAAGAATAATGACGGTACCTGATGAATAAGC

ACCGGCTAACTCCGTG

>3557 len=136 tot-seq=2

TGGGGGATATTGGACAATGGGGGAAACCCTGATGCAGCGACGCCGCGTGAAGGAAGAAGT

ATCTCGGTATGTAAACTTCTATCAGCAGGGAAGAAAGCAGACGGTACCTGACTAAGAAGC

CCCGGCTAACTACGTG

>3558 len=136 tot-seq=2

TGGGGGATATTGCACAATGGAGGGAACTCTGATGCAGCGACGCCGCGTGAGGGAAGAAGG

TTTTCGGATTGTAAACCTCTGTCTTCGGTGACGAAACGAGACGGTAGCCGAGGAGGAAGC

CACGGCTAACTACGTG

>3559 len=136 tot-seq=2

TGGGGAATATTGCACAATGGGCGGAAGCCTGATGCAGCAACGCCGCGTGAGCGATGAAGG

TCTTCGGATCGTAAAGCTCTGTCCTCAAGGAAGAATTTTGACGGTACTTGAGGAGGAAGC

CCCGGCTAACTACGTG

>3560 len=136 tot-seq=2

CTGGGGAATATTGCACAATGGGGGGAACCCTGATGCAGCGACGCCGCGTGGGTGAAGGAG

CACCTCGGTGCGTAAAGCCCTATCGGCAGGGAAGGAACAGACGGTACCTGACTAAGAAGC

CCCGGCTAACTACGTG

>3561 len=136 tot-seq=2

TGGGGAATCTTGCACAATGGGCGAAAGCCTGATGCAGCGACGCCGCGTGCGGGACGGAGG

CCTTCGGGTCGTAAACCGCTTTCAGCAGGGAAGAGTCAAGACTGTACCTGCAGAAGAAGC

CCCGGCTAACTACGTG

>3562 len=136 tot-seq=2

TGGGGAATATTGCACAATGGGCGAAAGCCTGATGCAGCAACGCCGCGTGAAGGAAGAAGG

CTTTCGAGTCGTAAACTTCTGTCCAAAGGGAAGAATAATGACGGTACCTTTGAAGAAAGC

CCCGGCTAACTACGTG

>3563 len=136 tot-seq=2

TAGGGAATATTGCTCAATGGGGGAAACCCTGAAGCAGCAACGCCGCGTGGAGGATGACGC

TTTTCGGAGCGTAAACTCCTTTTCTTGGGAAAGAATTATGACGGTACCCAAGGAATAAGC

ACCGGCTAACTCCGTG

>3564 len=136 tot-seq=2

AAGTCTGATGGAGCAACGCCGCGTGAGTGAAGAAGGGTTTCGGCTCGTAAAGCTCTGTTG

GTGGTGAAGAAGGATACGGGTAGTAACTGGCCTTTGTTTGACGGTAATCACTTAGAAAGT

CACGGCTAACTACGTG

>3565 len=136 tot-seq=2

GAACTGAGGCCCTTGCCAAAAGTCATTGGATTGAGCCATCTTGGAAGCAGATCTTCTATC

CCTAATCAACACTTCAGATGACTGCAGCCCTGGAGGCCAGATTAACTGCAACCTCAAGAA

AGTCCCTAAGCCAGGA

>3566 len=136 tot-seq=2

TGGGGAATCTTGCACAATGGACGAAAGTCTGATGCAGCAATTTCGCGTGAAGGATGAAGC

ATTACGGTGTGTAAACTTCTTTTTTGGAGGAAGACGAATGACGGTACTTCAAGAATAAGA

GACGGCTAACTACGTG

>3567 len=136 tot-seq=2

GGGATGGAGAACTCTGGGAAAGGTGGGATTACATTGCAGCATCGGCAGAGTACTAAGGAT

TGCCGTGGTCCTCCAGAAGGCCAAGGTCGTGCGTCTGCCATCAGTTCTTCACGCAGTCCG

ACAGTGCTGTGTGGAG

>3568 len=136 tot-seq=2

ACTGGATTGGGATCTACGCTGGGCCACCCACTAGCAGGCGTTTGGCCAAGGGGCCTCAGT

GGCCTCCTCTGTGCAGAAGGGGAGGAGACTGGGACCCCTCCCCTGTGACCTCGCTGGGGA

GCAGATTGCGGAGCAG

>3569 len=136 tot-seq=2

TGGGGAATTTTGCGCAATGGGGGAAACCCTGACGCAGCAACGCCGCGTGCGGGATGAAGG

CCTTCGGGTCGTAAACCGCTTTCAGCAGGGAAGAACATCGACGGTACCTGCAGAAGAAGC

CCCGGCTGACTACGTG

>3570 len=136 tot-seq=2

AAGTCTGATGGAGCAACGCCGCGTGAGTGAAGAAGGTTTTCGGATCGTAAAGCTCTGTTG

TTGGTGAAGAAGGAGAAAGGGAGTAACTGGCCATTATTTGACGGTAACCAACCAGAAAGT

CACGGCTAACTACGTG

>3571 len=136 tot-seq=2

TGGGGAATATTGTGCAATGGGGGAAACCCTGACACAGCGACGCCGCGTGAGTGAAGAAGG

CCTTCGGGTCGTAAAGCTCAATAGTATGGGAAGAAAGAAATGACGGTACCATACGAAAGC

CCCGGCTAACTACGTG

>3572 len=136 tot-seq=2

TTGGGGGAGAGGAGGCATAAGACCTGGAAATCCAGGAAACATGGAGGGCACTTGCAGATG

GTCGATCTGCCAGGGGACCTTCCCGCAAGCATGGAAGTCTGTGTCCCCACGCCCAGGTTG

AGTCACCGCCGGCTCC

>3573 len=136 tot-seq=2

CGAGGAATCTTCTACAATGGGCGCAAGCCCGATAGAGCAACGCCGCGTGGAGGACGACGG

CCTTCGGGTTGTAAACTCCTTTCGGGGGGGATGAGAACGGACAGTACCCCCAGAAGCAGC

CCCGGCTAACTCTGTG

>3574 len=136 tot-seq=2

TCGGGAATATTGCGCAATGGAGGGAACTCTGACGCAGTGACGCCGCGTATAGGAAGAAGG

TTTTCGGATTGTAAACTATTGTCATTAGGGAAGAAAAAAGACAGTACCTAAGAAGGAAGC

TCCGGCTAACTACGTG

>3575 len=137 tot-seq=2

GTTAATCTGAGTTCAAGGACAGCATGATCTACAGTGTGACTTCCAGGACAGCCAAGGATA

CAAGAGAAACTATCTAGAAACACCAAAATAATAATAATAATAAATAGTAATTTAAATGCT

ATACAAAGTAGCACTTC

>3576 len=137 tot-seq=2

TGATGACTGTTGTTATTTGAACCCAGACCCACTGCCCTTCTCTTTATCCCAGACAGCCTT

TCCTGTTTGTGTATAAAGAGTAACATTTTGATAAATATATTGAGAAAGTACAGGATGGTC

CCCATGAAAGTATCTAC

>3577 len=137 tot-seq=2

TGGGGAATTTTCCGCGGTGGGCGAAAGCCTGACGGGGCAATGCCACGTGGAGGTGGAAGG

CCTACAGGTCGTCAACTTCTTTTCTCGGAGAAGAAACAATGACGGTATCTGAGGAATAAG

CATCAGCTATCTCTGTG

>3578 len=137 tot-seq=2

TAGGGAATCTTCCACGATGGGCGAAAGCCTGATGGAGCAATACCATGTGAACGAAGAAGG

TCTTTTTGATTGTAAAGTTCTTTTATGAGGGATAACGACTGATAGTACCTCATGAATAAG

TGACAGCAAACTATGTG

>3579 len=137 tot-seq=2

TGAGGAATTTTCCGCAATGGGCGAAAGCCTGACGGAGCAATGCCGCGTGAAGGATGAAGG

CCTATGGGTTGTAAACTTCTTTTCTTAGAGAAGAAATTTTGACGGTATCTAAGGAATAAG

CATCGGCTAACCCTGTG

>3580 len=137 tot-seq=2

TGGGGAATATTGGGCGATGGGGGAAACCCTGACCCAGCAACGCCGCGTGGAGGAAGAAGG

TTTTCGGATTGTAAACTCCTGTCCTTGGTGAAGAGAAAGAGACGGTAGCCAAGGAGGAAG

CCCCGGCTAACTACGTG

>3581 len=137 tot-seq=2

TGGGGAATTTTCCGCAATGGGCGAAAGCCTGACGGAGCAATGCCGCGTGGAGGTAGAAGG

CCCACGGGTTGTGAACTTCTTTTCTCGGAGAAGAAACAATGACGGTATCTGAGGAATAAG

CATCGGCTAATTCTGTG

>3582 len=137 tot-seq=2

TGGGGAATATTGCACAATGGGCGAAAGCCTGATGCAGCGACGCCGCGTGAGTGAGGAAGT

ATTTCGGTATGTAAAGCTCTATCAGCAGGGAAGAAAGAAATGACGGTACCTGAGTAAGAA

GCTCCGGCTAAATACGT

>3583 len=137 tot-seq=2

TGGGGAATATTGCGCAATGGGGGAAACCCTGACGCAGCAACGCCGCGTGAAGGATGAAGG

TCTTCGGATTGTAAACTTTTGTCTTATGGGAAGAAGAAAGTGACGGTACCATAGGAGGAA

GCCACGGCTAACTACGT

>3584 len=137 tot-seq=2

TGGGGAATATTGGGCGATGGGCGAAAGCCTGACCCAGCGACGCCGCGTGAGGGAAGAAGG

TCTTCGGATTGTAAACCTTTGTAGACAGGGACGAAGAAATGACGGTACCTGTAAAGTAAG

CCACGGCTAACTACGTG

>3585 len=137 tot-seq=2

TGGGGAATATTGGGCAATGGGCGCAAGCCTGACCCAGCAATGCCGCGTGAGCGATGAAGG

TAGTTTTATTGTAAAGCTCTTTTCTCAGGAACGAACAAATGACGGTACCTGAGGAATAAG

CCCCGGCTAACTACGTG

>3586 len=137 tot-seq=2

TGGGGAATATTGCACAATGGGGGAAACCCTGATGCAGCGACGCCGCGTGAAGGAAGAAGG

TATTCGTATTGTAAACTTCTGTCGCAGATGACGAAAGAATGACGGTAATCTGCAAGAAAG

CACCGGCTAACTACGTG

>3587 len=137 tot-seq=2

ACCCAGTAGCTCAGAGATAGAAAAGCCACTGGCATTTGCAGGAGTGCAGAAGGCCAGGCC

TGCTCAGAGCAGAAGGTTCACTGGGAAGGTTAGTGTGTGACATGGTCAGAGAAGTCACAG

AGGATCTAGGAATAATC

>3588 len=137 tot-seq=2

TGGGTGAGATGAGCTGAACAGCTCATCTTTGAGAGCATGGGATAAAGTTTTTTTCCTTCC

TTGGAAGATTTGAAGAAGTGGAACAACAGGAAGGCCATGGTCTCTGTTCTCTGCCCATAG

TTACACAAGCTATGCTG

>3589 len=137 tot-seq=2

TGGGGGATATTGCGCAATGGGGGAAACCCTGACGCAGCAACGCCGCGTGAAGGAAGAAGG

TCTTCGGATTGTAAACTTTTGTCTTGTGGGACGAATCAATGACGGTACCACAGGAGGAAG

CCACGGCTAACTACGTG

>3590 len=138 tot-seq=2

TGGGGGATATTGGTCAATGGAGGAAACTCTGAACCAGCAACGCCGCGTGAATGATGAAGG

TCTTCGGATTGTAAAGTTCTTTTCTAAGGGAAGAAGATAGTGACGGTACTTTAGGAATAA

GCCTCGGCTAACTACGTG

>3591 len=138 tot-seq=2

TGGGGAATATTGGGCAATGGGCGCAAGCCTGACCCAGCAGCGCCGCGTGAGGGAAGAAGG

CTTTCGGGTTGTAAACCTCTGTCCTTGGTGGCGAAGGAAGTGACGGTAGCCAAGGAGGAA

GCCCCGGCTAACTACGTG

>3592 len=138 tot-seq=2

TAGGGAATCTTCGGCAATGGACGGAAGTCTGACCCAGCAATTCTGTGTGCACGATGAAGT

TTTTCGGAATGTAAAGTGCTTTCAGTTGGGAAGAAGAAAGTGACGGTACCAACAGAAGAA

GTGACGGCTAAATACGTG

>3593 len=138 tot-seq=2

TGGGGAATATTGCACAATGGGCGAAAGCCTGATGCAGCAACGCCGCGTGAGCGAAGAAGG

CTTTCGGGTCGTAAAGCTCTGTCCTTGGGGAAGAAAAAAATGACGGTACCCTTGGAGGAA

GCCCCGGCTAACTACGTG

>3594 len=138 tot-seq=2

TAGGGAATCTTCCACAATGGACGCAAGTCTGACGCAGCAACGCCGCGTGATTGAAGAAGG

CCTTCGGGTTGTAAAGATCTTTAATCAGGGACGAAACAAATGACGGTACCTGAAGAATAA

GCTCCGGCTAACTACGTG

>3595 len=138 tot-seq=2

TGGGGAATATTGCGCAATGGGGGCAACCCTGACGCAGCAACGCCGCGTGATTGAAGAAGG

TCTTCGGATTGTAAAAATCTTCAATTAGGGACGAAACAAATGACGGTACCTAAAGAATAA

GCTCCGGCTAACTACGTG

>3596 len=138 tot-seq=2

ACAATAGCAGAGAGTGTACAGATTATAGCGGCACAAATGGTGAGCCCACAGACGCTGAAG

AGCAGGTTCTGGGGAAGGGAAAGGAGGAGAGGAGTCAGGAGACGGCTAAGTTCCACACAT

TATGGGCCTGTGAGTACC

>3597 len=138 tot-seq=2

TGGGGAATATTGGGCAATGGGCGCAAGCCTGACCCAGCAACGCCGCGTGAAGGAAGAAGG

CTCTCGGGTTGTAAACTTCTTTTGAGAGGGACGAAGTAAGTGACGGTACCTCTTGAATAA

GCCACGGCTAACTACGTG

>3598 len=138 tot-seq=2

TGGGGGATATTGCGCAATGGGGGCAACCCTGACGCAGCAACGCCGCGTGAAGGAAGAAGG

CCTTCGGGTTGTAAACTTCTGTCTTTAGGGAAGAAGAAAGTGACGGTACCTAAGGAGGAA

GCCACGGCTAACTACGTG

>3599 len=138 tot-seq=2

TGGGGAATATTGGGCAATGGGCGAAAGCCTGACCCAGCGACGCCGCGTGAGGGAAGACGG

CCTTCGGGTTGTAAACCTCTGTTGCAGGGGAAGAAGGAAGTGACGGTACCCTGCGAGGAA

GCTCCGGCAAACTACGTG

>3600 len=138 tot-seq=2

TCGGGAATATTGCGCAATGGAGGAAACTCTGACGCAGTGACGCCGCGTGCAGGAAGAAGG

TTTTCGGATTGTAAACTGCTTTTAACAGGAAAGAAAAAAATGACGGTACCTGTTGAATAA

GCTCCGGCTAACTACGTG

>3601 len=138 tot-seq=2

TGGGGAATATTGCACAATGGGGGGGACCCTGATGCAGCGACGCCGCGTGGGTGAAGGAGT

ATTTCGGTATGTAAAGCTCTATCAGCAGGGAAGAAAGAAATGACGGTACCTGACTAAGAA

GCCCCGGCTAACTACGTG

>3602 len=138 tot-seq=2

TGGGGAATATTGGACAATGGAGGAAACTCTGATCCAGCAATTCTGTGTGTGTGAAGAAGG

TCTTCGGATTGTAAAACACTTTTAGTTGGGAAGAAACAAATGACGGTACCAACAGAAGAA

GCGACGGCTAAATACGTG

>3603 len=138 tot-seq=2

TGGGGAATATTGCGCAATGGGGGAAACCCTGACGCAGCGACGCCGCGTGAGTGAAGAAGG

CTTTCGAGTCGTAAAACTCTGTCGTATGGGAAGAAAAAAATGACGGTACCATACAAGAAA

GCCCCGGCTAACTACGTG

>3604 len=138 tot-seq=2

TGGGGAATATTGGGCAATGGGGGAAACCCTGACCCAGCAACGCCGCGTGAAGGAAGAAGG

CTTTCGGGTTGTAAACTTCTTTTACCGAGGACGAAGAAAGTGACGGTACTCGGAGAAAAA

GCCACGGCTAACTACGTG

>3605 len=138 tot-seq=2

TGGGGAATATTGCACAATGGGGGGAACCCTGATGCAGCAATGCCGCGTGAAGGATGAAGG

TTTTCGGATTGTAAACTTCTTTTGTACGGGACGAAGAAAGTGACGGTACCGTAAGAATAA

GCCACGGCTAACTACGTG

>3606 len=138 tot-seq=2

TAGGGAATCTTCGGCAATGGGGGGAACCCTGATCCAGCGACGCCGCGTGAGTGAAGAAGT

ATTTCGGTATGTAAAGCTCTATCAGCAGGGAAGAAAGAAATGACGGTACCTGACCAAGAA

GCCCCGGCTAACTACGTG

>3607 len=138 tot-seq=2

TAGGGAATCTTCGGCAATGGGGGGAACTCTGATCCAGCAATTCTGTGTGTGTGAAGAAGG

TTTTAGGACTGTAAAACACTTTTAGTAGGGAAGAAAAAAATGACGGTACCTACAGAAGAA

GCAACGGCTAAATACGTG

>3608 len=138 tot-seq=2

TGGGGAATATTGCACAATGGGCGAAAGCCTGATGCAGCAACGCCGCGTGAACGAAGAAGG

CTCTTGGGTCGTAAAGTTCTGTTCTGAGGGAAGAAGAAAGTGACGGTACCTCAGGAGAAA

GCCCCGGCTAACTACGTG

>3609 len=138 tot-seq=2

TGGGGAATCTTCCGCAATGGGCGAAAGCCTGACGGAGCAACGCCGCGTGAGTGAAGAAGG

CTTTCGAGTCGTAAAACTCTGTCGTATGGGAAGAAAAAAATGACGGTACCATACAAGAAA

GCCCCGGCTAACTACGTG

>3610 len=138 tot-seq=2

TGGGGAATATTGCACAATGGGGGAAACCCTGATGCAGCGACGCCGCGTGAGTGAGGAAGT

ATTTCGGTATGTAAAGCTCTGTCAGCGGGGAAGAAAGAAATGACGGTACCCGACCAAGAA

GCACCGGCTAAATACGTG

>3611 len=138 tot-seq=2

TGGGGAATATTGGGCAATGGGAGGAATCCTGACCCAGCGACGCCGCGTGAACGAAGACGG

CCTTCGGGTTGTAAAGTTCTTTTATGTGGGAAGAATGAAGTGACGGTACCACATGAATAA

GCCCCGGCTAACTACGTG

>3612 len=138 tot-seq=2

TCGGGAATATTGGACAATGGAGGAAACTCTGATCCAGTGACGCCGCGTGAAGGAAGAAGG

TCTTCGGATTGTAAACTTATTTTGTCAGGGAAGAATAAAGTGACTGTACCTGAAGAAAAA

GCACCGGCAAACTACGTG

>3613 len=138 tot-seq=2

TGGGGAATATTGCACAATGGGGGAAACCCTGATGCAGCAACGCCGCGTGAAGGAAGAAGG

CCTTCGGGTTGTAAACTTCTTTTACCAGGGACGAAGGACGTGACGGTACCTGGAGAAAAA

GCAACGGCTAACTACGTG

>3614 len=138 tot-seq=2

TGGGGAATATTGGGCAATGGGCGGAAGCCTGACCCAGCAACGCCGCGTGAAGGAAGAAGG

CTTTTGGGTTGTAAACTTCTTTTCTAAGGGACGAAGAAAGTGACGGTACCTTAGGAATAA

GCCACGGCTAACTACGTG

>3615 len=138 tot-seq=2

GGTGGGGGATATTGCACAATGGGGGGAACCCTGATGCAGCAACGCCGCGTGAGGGAAGAA

GGTTTTCGGATTGTAAACCTCTGTCCTTAGGGAAGAGAGAGGACGGTACCTAAGGAGGAA

GCTCCGGCTAACTACGTG

>3616 len=138 tot-seq=2

TGGGGAATATTGGGCAATGGGCGAAAGCCTGACCCAGCAACGCCGCGTGAAGGAAGAAGG

CCTTCGGGTTGTAAACTTCTTTTACCAGGGACGAACACAAAGACGGTACCTGGAGAAAAA

GCAACGGCTAACTACGTG

>3617 len=138 tot-seq=2

TGGGGAATATTGGGCAATGGGCGAAAGCCTGACCCAGCGACGCCGCGTGAGGGAAGAAGG

TCTTCGGATTGTAAACCTTTGTAAGCAGGAAAGAAGAAAGTAACGGTACCTGCAAAGTAA

GCCACGGCTAACTACGTG

>3618 len=138 tot-seq=2

TGGGGAATATTGGGCAATGGGCGAAAGCCTGACCCAGCAACGCCGCGTGAGTGATGAAGG

CCTTCGGGTTGTAAAACTCTTTGGACAGGGACGAAAAAAATGACGGTACCTACAGAAGAA

GCAACGGCTAAATACGTG

>3619 len=138 tot-seq=2

TGGGGAATCTTGCACAATGGGCGAAAGCCTGATGCAGCGACGCCGCGTGAGTGATGAAGG

CCTTCGGGTTGTAAAACTCTTTGGACAGGGACGAAGAAAGTGACGGTACCTGTAGAACAA

GCCACGGCTAACTACGTG

>3620 len=138 tot-seq=2

TGGGGAATATTGCACAATGGGCGAAAGCCTGATGCAGCAACGCCGCGTGAAGGATGAAGG

TCCTTGGATTGTAAACTTCTGTCCATAGGGAAGAAACAAATGACGGTACCTATGGAGGAA

GCCCCGGCTAACTACGTG

>3621 len=138 tot-seq=2

CTCCAGGCACTGGGACCTTGCCATGCAGGTGAGCTTTCCTGTTGCTGTTGCAAGAAAATG

TTCCAAAGAGCTAAGAGAGACGTGTAGAGGACATCTAAGCTGCCTGGTAAACAGTCAGTG

GCAGATTTCTAACTGAAC

>3622 len=138 tot-seq=2

TGGGGAATATTGCACAATGGGGGGAACCCTGATGCAGCGACGCCGCGTGAAGGAAGAAGT

ATCTCGGTATGTAAAGCCCTATCAGCAGGGAAGAAAGAAATGACGGTACCTGACTAAGAA

GCCCCGGCTAACTACGTG

>3623 len=138 tot-seq=2

TGGGGAATATTGGGCAATGGGCGCAAGCCTGACCCAGCAACGCCGCGTGAAGGAAGGAGG

CTTTCGGGTTGTAAACTTCTTTTAAGAGGGACGAAAGAAATGACGGTACCTCTTGAATAA

GCCACGGCTAACTACGTG

>3624 len=138 tot-seq=2

TGGGGAATATCGGGCAATGGGCGAAAGCCTGACCCAGCAACGCCGCGTGAGGGAAGAAGG

TTTTCGGATTGTAAACCTCTGTCCTAAGTGACGAAGGAAGTGACGGTAGCTTAGGAGGAA

GCCCCGGCTAACTACGTG

>3625 len=138 tot-seq=2

TGGGGAATATTGCACAATGGGGGAAACCCTGATGCAGCAACGCCGCGTGAAGGAAGAAGG

GTTTCGGCTCGTAAACTTCTATCAATAGGGATGAAAAAAATGACAGTACCTAAATAAGAA

GCCCCGGCTAACTACGTG

>3626 len=138 tot-seq=2

TGGGGAATATTGGGCAATGGGCGAAAGCCTGACCCAGCGACGCCGCGTGCAGGAAGAAGG

TCTTCGGATTGTAAACTGCTGTTGTAAGGGAAGAAGAAAGTGACGGTACCTTACGAGGAA

GCCACGGCTAACTACGTG

>3627 len=138 tot-seq=2

TGGGGAATATTGCACAATGGGGGGAACCCTGATGCAGCAACGCCGCGTGAAGGAAGAAGT

AATTCGTTATGTAAACTTCTATCAACGGGGAAGAAAGAGATGACGGTACCCGAGTAAGAA

GCCCCGGCTAATTACGTG

>3628 len=139 tot-seq=2

GGGCCTGTGCCAAGACAGAGAGGGGCAGGAGAGCCCCAGCCAGCTTGACTGGGGTGGCTC

CACAGGCCAGCCCTGTGCTCAGCAGGTGGGCGCCACAAGGCCACTTCCTGCCACCCACAT

CACTTTAGAGTGTGAGCAC

>3629 len=139 tot-seq=2

CGCAAGCCTGATGGAGCAACGCCGCGTGAGTGAAGAAGGGTTTCGGCTCGTAAAGCTCTG

TTGTTGGAAAAGAACGAGAGTGGGAGTAACTGTTCACGCAGTGACGGTATCCAACCAGAA

AGTCACGGCTAACTACGTG

>3630 len=139 tot-seq=2

AATCACTTGAACCCGGGAGGCAGAGGTTGCAGTGAGCTGATATCGCACCACTGCTCTCCA

GCCTGGGCAACAGAGTGAGACATAGTCTCAAAAAAAAAAGAACATGAATTGGATCACATC

ATTCGCCCACTGAACATCT

>3631 len=139 tot-seq=2

TAGGGAATCTTCCACAATGGACGCAAGTCTGATGGAGCAACGCCGCGTGAGTGAACTCTA

TTGTTGGTGAAGAAGGATAGAGGTAGTAACTGGCCTTTATTTGACGGTAATCAACCAGAA

AGTCACGGCTAACTACGTG

>3632 len=139 tot-seq=2

TGGGGAATATTGCGCAATGGAGAAGAACTCTGACGCAGCAATACCGCGTGAGCGAAGAAG

GTCTTCGGATCGTAAAGCTCTGTTATCAGGGAAGAAGAAAATGACGGTACCTGATGAGGA

AGTCCCGGCTAACTACGTG

>3633 len=139 tot-seq=2

GCCTGGGCAGCACACAGGTAGGCTCAGGTGCCCTTGGCTACTCAGAATTGTGGGCAGGGT

CCTGCTGGCATCTGGAGCCCCCAGCATCAAGAGGGTCTTACTCCCACCTTCAGGCCTCCT

TCCCATCCCCCACCCTCAG

>3634 len=140 tot-seq=2

GTCAGGGTGAATGTGTAGTGTCCACCAGGGGCTAAATTCCTTATTTGATTGCAGCAGTTC

TAGTCTGAGCCATTCATGGATACTGAGGCCCTTCCAAGCCTTTAGTTAAAAAAGGTTCTG

AATACTCAGAGAGTAAAGCA

>3635 len=140 tot-seq=2

TGGGGAATATTGCACAATGGGCGCAAGCCTGATGCAGCGACGCCGCGCGGGGGATGAAGG

CCTTCGGGTTGTAAACCCCTTTCAGCAGGGAAGAAGCGAAAGTGACGGTACCTGCAGAAG

AAGCGCCGGCTAACTACATG

>3636 len=140 tot-seq=2

TGGGGAATATTGCGCAATGGGCGAAAGCCTGACGCAGCAATGCCGCGTGCGGGATGACGG

CCTTCGGGTTGTAAACCGCTTTCAGCCATGACGAAGCGAAAGTGACGGTAATGGAAGAAG

AAGCACCGGCCAACTACGTG

>3637 len=140 tot-seq=2

TGGGGAATATTGCACAATGGGCGAAAGCCTGATGCAGCGACGCCGCGTGCGGGATGAAGG

CCTTCGGGTTGTAAACCGCTTTTGACGGGGAGCAAGCGAGAGTGAGTGTACCCGTCGAAT

AAGCACCGGCTAACTACGTG

>3638 len=140 tot-seq=2

TGGGGAATATTGCACAATGGGCGAAAGCCTGATGCAGCGACGCCGCGTGAGGGATGACGG

CCTTCGGGTTGTAAACCTCTTTCACCAGGGACGAAGCTAACGTGACGGTACCTGGAGAAG

AAGCACCGGCTAACTACGTG

>3639 len=140 tot-seq=2

TGGGGAATATTGCACAATGGGCGCAAGCCTGATGCAGCGACGCCGCGTGAGGGATGAAGG

CCTTCGGGTTGTAAGCCTCTTTCGTCAGGGAAGAAGCGAAAGTGACGGTACCTGAAGAAG

AAGCACCGGCTAACTACGTG

>3640 len=140 tot-seq=2

TAGGGAATCTTCGGCAATGGACGAAAGTCTGACCGAGCAACGCCGCGTGAGGGATGGAGG

CCTTCGGGTTGTAAACCTCTTTTATCGGGGAGCAAGCGAGAGTGAGTTTACCCGTTGAAT

AAGCACCGGCTAACTACGTG

>3641 len=140 tot-seq=2

CTGGGCTGAGTGGTGGTGGGCCCCTACATGTACACCCGTGGCAGAGAGCTGGAGCACTGG

GACGAGATGCTCAGCAAGCCCAAGGAGCTGGTGAAGCGCTGGCATGCGCTCTGCCGCACC

ACGGAGCCCTGACCCTTCGC

>3642 len=140 tot-seq=2

GTTGAGCCCAAGACAGGCTGTAGTCCCCAAGAGCAGAAATTGTCCTGTGAACAGTCACTG

TCATTCACTACAGAAGCATTTTACAAATGCTTTTTTTTTTTCATACAAGCCATAGTCCCC

ACATAGCTATATTAGAAACC

>3643 len=140 tot-seq=2

CCTGCGGTACATCTGCTCGCCCTGCGGGTGCCGTCCATCCTTGAGGGTCTGCACGTCGTT

GAAGAGCAGCCGGATCATGCTATCCGCCTTGTCCAAGTCCCGTTCCACCTCCCCCGCCCG

CTGTGGCACTTTGCCTGCAG

>3644 len=140 tot-seq=2

GAGAGCGTTGCAGAAATTCAGGATACCGATCATCAGCATCACGCTCCCCACAAAAAACGG

GATCATATACTGGTGAAAGACAGCATTATGAAAATTCCACTGATAGGTGGTAAAGGATGT

CACCGTGACTCCCGTCCCCT

>3645 len=140 tot-seq=2

GTGCTCTGCTGGAGCTCTCCAGTGGTCAGGCATGGTCTGCTGGAGAAGGAACTATGATGA

AGGTCCCCAGAAAGTGCCCCAGTTGGGCAAGTGAGGCTACGTTGCAAGTAGGCATGGCCA

GGCTGGGGCACCAAAAGAGG

>3646 len=141 tot-seq=2

TGGGGAATATTGGACAATGGGGGCAACCCTGATCCAGCCATGCCGCGTGGGGGAATGAAG

GTCTTCGGATTGTAAACCCCTGTCATGTGGGAGCAAATTAAAAAGATAGTACCACAAGAG

GAAGAGACGGCTAACTCTGTG

>3647 len=141 tot-seq=2

GGGAATATTGCGCAATGGGGGAAACCCTGACGCAGCGACGCCGCGTGCGGGATGAAGGAA

TTAGGGTTGTAAAAAGAGGGGGATTGGGAGAAAGAATTTTGGGTGAGTGTACCTTTCGAA

TAAGCGCCGGCTAACTACGTG

>3648 len=141 tot-seq=2

TGGGGAATATTGCGCAATGGGGGAAACCCTGACGCAGCGACGCCGCGTGCGGGATGAAGG

CCTTCGGGTTGTAAACTCCTTTCGCTAGGGACGAAGCTTTTTGTGACGGTACCTAGATAA

GAAGCACCGGCTAACTACGTG

>3649 len=141 tot-seq=2

TGGGGAATATTGCACAATGGGCGGAAGCCTGATGCAGCGACGCCGCGTGAGGGATGACGG

CCTTCGGGTTGTAAACCTCTTTCAGTAGGGAAGAAGCTTTTTGTGACGGTACCTGCAGAA

GAAGCGCCGGCTAACTATGTG

>3650 len=141 tot-seq=2

GGGAATATTGCGCAATGGGGGAACCCCTGACGCAGCGACGCCGAGTGAGGGATGAAGGAA

TTAGGGTTGTAAAAAGATTTTGATTGGGAGAAAGAATTTTGGGTGAGTGTACCTTTCGAA

TAAGCGCCGGCTAACTACGTG

>3651 len=141 tot-seq=2

CGAAAGTCTGACGGAGCAACGCCGCGTGAGTGATGAAGGGTTTCGGCTCGTAAAGCTCTG

TTGTTAGGGAAGAAACAGTACCGAACGAACTAGGTCGGTACCTTGACGGTACCTAACCAG

AAAGCCACGGCTAACTACGTG

>3652 len=141 tot-seq=2

TGAGGAATATTGGTCAATGGGCGAGAGCCTGAACCAGCCAAGTAGCGTGCAGGATGACGG

CCCTATGGGTTGTAAACTGCTTTTATATGGGAATAAAGTGAGGGACGTGTCCCTTTCGAA

TAAGCGCCGGCTAACTACGTG

>3653 len=141 tot-seq=2

CTGAAAATCTCAATTCAGTTAGCTGGAGTCTATGCTGCACATAGTTCAGGGCTTAGCCAG

AGAGATGGATAGAATTTATGCACAGAATTTGTGGTGCTTTTTCTTTGGCTCTCTTTCTTC

TGAGATTTCCCCCCTCATTTT

>3654 len=141 tot-seq=2

GGATAACCCATTCTTGAGAGGAGATTTGCTATAAAATGGAGCAGATAAGTGGTGTAGTAG

CTGGAGGGGTCACAGAGTCAAGAAAGAATTTTTCAAAATAGAAGAGGTATTACAACTTGT

TGATATGTTGATCTAAACGAT

>3655 len=141 tot-seq=2

TAGGGAATCTTCCACAATGGACGAAAGTCTGATGGTGCGACGCCGCGTGGGGGAATGAAG

GTCTTCGGATTGTAAACCCCTGTCATGTGGGAGCAAATTAAAAAGATAGTACCACAAGAG

GAAGAGACGGCTAACTCTGTG

>3656 len=141 tot-seq=2

TGAGGAATATTGGTCAATGGGAGAGATCCTGATGGTGCGACGCCGCGTGGGGGAATGAAG

GTCTTCGGATTGTAAACCCCTGTCATGTGGGAGCAAATTAAAAAGATAGTACCACAAGAG

GAAGAGACGGCTAACTCTGTG

>3657 len=141 tot-seq=2

GGGGGTGGAGTGAAGCCCAGGAAACAGCCCAGGTGAAGGGAGCTGGCGCTCCAGACTGAA

TGCAGTTTCCTAAGATCCACTGGCGTCGTGTATGCAAATGAGAAGGGCTTCCCGGAGCTG

CCCTGAGCCAGGCGCCTTCTG

>3658 len=141 tot-seq=2

TCGAGGATCTTCGGCAATGGGGGCAACCCTGACCGAGCGATGCCGCGTGAGCGATGAAGG

CCTTCGGGTTGTAAAGCTCGAAAGTGGGGAGAAACGCCGAAAGGCTTGATCTATCCACAG

TAAGCTCGGGCTAAGTTCGTG

>3659 len=142 tot-seq=2

GCAAGCACCCGCCCCCACAAGGTCAGAGATGGTGCGCAAAGGGACCGAGGTAGAAGAGCC

TCTGGGGTCTGTTTTCAGTCTAGGGGACTTCTGGGGCGTGATGGGTGGGGTTAGCAACAC

ACCCTTCCTTAGGACCTGGGCC

>3660 len=142 tot-seq=2

TAGGGAATCTTCGGCAATGGGGGCAACCCTGACCGAGCAACGCCGCGTGAGTGAAGAAGG

TTTTCGGGTTGTAAACCGCTTTCAGCAGGGACGAGGCCGCAAGGTGACGGTACCTGCAGA

AGAAGCCCCGGCTAACTACGTG

>3661 len=142 tot-seq=2

TGGGGAATTTTGGACAATGGGGGCAACCCTGATCCAGCCATGCCGCGTGCAGGATGAAGG

TCTTCGGATTGTAAACTGCTTTCAGCAGGGACGAGGCCGCAAGGTGACGGTACCTGCAGA

AGAAGCCCCGGCTAACTACGTG

>3662 len=142 tot-seq=2

ATCGGAGGTTATCAGGCATGGTGGCCCCGCAGAGACCTGCAGCTCAGCCCCTCAGGAACA

AAGGGAGGTGGTGCAAAACACGGACTGTACAGTTATCCTACGTCAGGGATGAAGGATGGA

TCTGGGCCTTCTACGGCAACTC

>3663 len=142 tot-seq=2

GGGGAAGGGTCTGTCAAAAGGATTGCAGAAAATACGTGATGACCCGAAAAGAGGGAGTGT

TGATCTTTTATAGGAACCAAACAATACATATCCAGAAGGCCAGCGAACTCTGAAGCTTGC

GAGCTGAGTTCCTTTGTGCTGA

>3664 len=142 tot-seq=2

TGAGGAATATTGGTCAATGGGGGGAACCCTGACCGAGCAACGCCGCGTGAACGAAGAAGT

TATTCGTAATGTAAAGTTCTTTTATCAGGGAAGAAAGAGGAATTTGACGGTACCTGAGGA

ATAAGCTCCGGCTAACTACGTG

>3665 len=142 tot-seq=2

TAGGGAATCTTCGGCAATGGGGGGAACCCTGACCGAGCAACGCCGCGTGAGTGAAGAAGG

TTTTCGGGTTGTAAACCGCTTTTGATCGGGAGCAAGCCTTCGGGTGAGTGTACCTTTCGA

ATAAGCACCGGCTAACTACGTG

>3666 len=142 tot-seq=2

TGAGGAATATTGGTCAATGGGCGAGAGCCTGAACCAGCCAAGTAGCGTGCGGGATGACGG

CCTTCGGGTTGTAAACCGCTTTTGATCGGGAGCAAGCCTTCGGGTGAGTGTACCTTTCGA

ATAAGCACCGGCTAACTACGTG

>3667 len=142 tot-seq=2

TGGGGAATATTGCACAATGGGCGCAAGCCTGATGCAGCGACGCCGCGTGAGGGATGACGG

CCTTCGGGTTGTAAACCTCTTTCGCCAGGGACGAAGGGTGATACTGACGGTACCTGGAGA

AGAAGCACCGGCTAACTACGTG

>3668 len=142 tot-seq=2

TGGGGAATATTGCACAATGGGCGCAAGCCTGATGCAGCAACGCCGCGTGAGCGATGAAGG

TCTTCGGGTCGTAAACCGCTTTTGATTGGGAGCAAGCCTTCGGGTGAGTGTACCTTTCGA

ATAAGCGCCGGCTAACTACGTG

>3669 len=142 tot-seq=2

TGGGGAATATTGCGCAATGGGCGAAAGCCTGATGCAGCGGCGCCGCGTGCGGGATGAGGG

CCTTCGGGTTGTAAACCGCTTTCAGCAGGGACGAGGCCGCAAGGTGACGGTACCTGCAGA

AGAAGCCCCGGCTAACTACGTG

>3670 len=142 tot-seq=2

TGAGGAATATTCCACAATGGGCGAAAGCCTGATGGAGCAATGCCGCGTGCAGGATGAGGG

CCCTCGGGTTGTAAACCGCTTTTGATTGGGAGCAAGCCTTCGGGTGAGTGTACCTTTCGA

ATAAGCGCCGGCTAACTACGTG

>3671 len=142 tot-seq=2

TGAGAACTCCGGGAGTCACAGAGGAGAAGGGGAGAGACAGGGAGGCAGCACAGAGGAGAA

GGGGAGAGACAGGGAGGCAGTGGTGAGAACTCCAGGAGCCACAGCCCACCCCAGAAATGT

GGGGTGTCAGATGAGCCTGGGG

>3672 len=142 tot-seq=2

TGAGGAATATTCCACAATGGGCGTAAGCCTGATGGAGCAATGCCGCGTGCGGGATGAAGG

CCTTCGGGTTGTAAACCGCTTTTGATTGGGAGCAAGCTTTCGGGTGAGTGTACCTTTCGA

ATAAGCGCCGGCTAACTACGTG

>3673 len=142 tot-seq=2

TAGGGAATATTCCACAATGAGCGAAAGCTTGATGGAGCGACGCCGCGTGCGGGATGAAGG

CCTTCGGGTTGTAAACCGCTTTTGATTGGGAGCAAGCTTTCGGGTGAGTGTACCTTTCGA

ATAAGCGCCGGCTAACTACGTG

>3674 len=142 tot-seq=2

TGGGGAATATTGGACAATGGAGGCAACTCTGATCCAGCAATTCTGCGTGCGGGATGAAGG

CCTTCGGGTTGTAAACCGCTTTTGATTGGGAGCAAGCTTTCGGGTGAGTGTACCTTTCGA

ATAAGCGCCGGCTAACTACGTG

>3675 len=142 tot-seq=2

TGGGGAATATTGGGCAATGGGCGAAAGCCTGACCCAGCAACGCCGCGTGAGTGATGAAGG

CCTTTGGGTTGTAAACCGCTTTTGATTGGGAGCAAGCTTTCGGGTGAGTGTACCTTTCGA

ATAAGCGCCGGCTAACTACGTG

>3676 len=142 tot-seq=2

TGGGGAATATTGGGCAATGGACGCAAGTCTGACCCAGCAACGCCGCGTGAAGGAAGAAGG

CTTTCGGGTTGTAAACCGCTTTTGATTGGGAGCAAGCCTTCGGGTGAGTGTACCTTTCGA

ATAAGCGCCGGCTAACTACGTG

>3677 len=142 tot-seq=2

TGGGGAATTTTGGACAATGGGCGCAAGCCTGATCCAGCAATGCCGCGTGCGGGAAGAAGG

CCTTCGGGTTGTAAACCGCTTTTGATTGGGAGCAAGCCTTCGGGTGAGTGTACCTTTCGA

ATAAGCGCCGGCTAACTACGTG

>3678 len=142 tot-seq=2

TGGGGAATATTGCACAATGGACGGAAGTTCGATGCAGCGACGCCGCGTGGAGGGTGTAGG

CCTTCGGGTTGTAAACCGCTTTCAGCAGGGACGAGGCCGCAAGGTGACGGTACCTGCAGA

AGAAGCCCCGGCTAACTACGTG

>3679 len=142 tot-seq=2

TGGGGAATCTTCCGCAATGGGCGCAAGCCTGACGGAGCAATGCCGCGTGAGGGATGAAGG

CCTTCGGGTTGTAAACCGCTTTCAGCAGGGACGAGGCCGCAAGGTGACGGTACCTGCAGA

AGAAGCCCCGGCTAACTACGTG

>3680 len=142 tot-seq=2

GGACGAAAGTCTGATGGAGCAACGCCGCGTGAGTGAAGAAGGGTTTCGGCTCGGAAAGCT

CTGTTGTTAGTGAAGAAAGATAGAGGTAGTAACTGGCCTTTAGGTGACGGTAATTACTTA

GAAAGTCACGGCTAACTACGTG

>3681 len=142 tot-seq=2

TAGGGAATTTTCGGCAATGGGGGGAACCCTGACCGAGCAACGCCGCGTGAACGAAGAAGT

ATTTCGGTATGTAAAGTTCTTTTATTGGTGAAGAAAGAGGAATTTGACGGTAGCCAATGA

ATAAGCTCCGGCTAACTACGTG

>3682 len=143 tot-seq=2

CAGTTTCCAAGAAGTTGATGGTCACCCCCAAAGTAGCAGAAGCTGGAGTTCAAGGGAAGG

TCCTCGTGACCTCTTATTGGGAGAGATGCCATAGATACTGACGATGTTGTAAACAGACAA

GACAAACTACAACATTAAAATAC

>3683 len=143 tot-seq=2

TCCATGTATGATGAGATAAGTATTTTCTTCTGTAAGGTGTTTAGTAGCTCAGGTGGAAGA

GTGTTTGCCTAGCTTGGCAGACCCTAGGACTGATCCTCCGCACAGCATAAACCAGATGTG

CTGATGCACATTTGTAATGAATA

>3684 len=143 tot-seq=2

CCCATCCGTACTCCAGCATGGTCGGGAGCATAAGGCTGCACCCCTAAGCGGAGGAGCTCC

TGGTGAGTGAGGGCTGTCGGGAGAGGAAGTTTATTTTTCTCTAGGGGTACAGGTATGGAA

AGTCACCCACACTCATGCACATC

>3685 len=143 tot-seq=2

TGGGGAATATTGCACAATGGAGGAAACTCTGATGCAGCAACGCCGCGTGAATGATGAAGG

CCTTCGGGTTGTAAACCGCTTTTGATTGGGAGCAAGCCTTTTGGGTGAGTGTACCTTTCG

AATAAGCGCCGGCTAACTACGTG

>3686 len=143 tot-seq=2

TGGGGAATATTGCGCAATGGACGAAAGTCTGACGGAGCAACGCCGCGTGAGTGATGACGG

TCTTCGGATTGTAAACCGCTTTTGATTGGGAGCAAGCCTTTTGGGTGAGTGTACCTTTCG

AATAAGCGCCGGCTAACTACGTG

>3687 len=143 tot-seq=2

TGAGGAATATTGGTCAATGGGCGCTAGCCTGAACCAGCCAAGTAGCGTGCAGGATGACGG

CCCTATGGGTTGTAAACCGCTTTTGATTGGGAGCAAGCTTTCGGGTGAGTGTACCTTTCG

AATAAGCGCCGGCTAACTACGTG

>3688 len=143 tot-seq=2

TGAGGAATATTGGTCAATGGACGCAAGTCTGAACCAGCCAAGTAGCGTGCAGGATGACGG

CCCTATGGGTTGTAAACTGCTTTTATATGGGAGCAAGCTTTCGGGTGAGTGTACCTTTCG

AATAAGCGCCGGCTAACTACGTG

>3689 len=143 tot-seq=2

GGGGGAAACCCTGACGCAGCGACGCCGCGTGCGGGATGAAGGCCTTCGGGTTGTAAAGTT

CTGTTATGCGGGGCGAATGGTACGACGGTCAATACCCGTCGTAAGTGACGGTACCGTAAG

AGAAAGCCACGGCTAACTACGTG

>3690 len=143 tot-seq=2

CCCCAGCAGCAGCTCCAGCAGCAGCAGCCCCAGCAGCAGCCCCAGCAGCAGCAGCCCCAG

CAGCAGCCACAGCAGCAGCAGCCCCAGCAGCAGCAGCTCCACCAGCAGCCCCAGTAGCAG

CTCTAGCAGCAGCAGCAGCAGCC

>3691 len=143 tot-seq=2

TGGGGAATATTGGACAATGGAGGGAACTCTGATCCAGCAATTCTGTGTGTGGGATGAAGG

CCTTCGGGTTGTAAACCGCTTTTGATTGGGAGCAAGCCTTTTGGGTGAGTGTACCTTTCG

AATAAGCGCCGGCTAACTACGTG

>3692 len=144 tot-seq=2

ACACCTGGGAAGAGGCCGGAGAAGGTCTGGTTGCAAGATACCACCACCACCTCAGAGGAT

GTTATGTTGTGTGCATCAAGTGCATATCTGAAGCTCACGTCTGTCTTGTGTGCACACTGT

CCTCCATGCAAAGCAGCCGCAGAC

>3693 len=144 tot-seq=2

CCTGGGTGTTTCCCAAAGACCTGACTTTCTTATCTAACAGAAGAGCGATTGACAAAAGGC

AAAGAAGGCAGGGTGAGCTCTTGGTACACAGGGGACTTGGGGGAGAAGCTAGGCCTTGTG

CTGTGTGGGCAGAAGCTGTGAGGG

>3694 len=144 tot-seq=2

TAGGGAATTTTTCACAATGGGCGCAAGCCTTATGAAGCAATGCCGCGTGAACGATGAAGG

TCTTATAGATTGTAAAGTTCTTTTATATGGGAGCAAGCCTTTTGGGTGAGTGTACCTTTC

GAATAAGCGCCGGCTAACTACGTG

>3695 len=144 tot-seq=2

TGGACGAAAGGCGGACGGAGCAACGCCGCGTGAGGGATGACGGGCGTCGGATTGGAAAGC

TCTGTTAATCGGGACGAATGGTCGGTGTGCAAATAGTGCATGGACATGACGGTACCGGAA

TAGAAAGCCACGGCTAACTACGTG

>3696 len=144 tot-seq=2

CCGCATATACCTATGAAGCCATTGAAGGAGAGGGGCCCGATTTCCTGGCCCAGAAAACCC

CCATTGTCATGGCATACTGGGGAGCCGCCAATGCAGACACCGCTTACGGAAGTCCGGATT

TTAATCTTCAGGTTATCGGATTCC

>3697 len=145 tot-seq=2

AGGTCTTTTAGGGCAGGACGATAACAATCAAAACAGTATGTAAGGGCCTGCCACGCAAGC

ACACACATGCTCATTGCCCCTGGGAGGCTCCCAAAGCTGGGGTGAAGTGGGGCTTTGTCA

CAAATACGCCTTTCTCCTTTCCTCA

>3698 len=145 tot-seq=2

GCGGTTTGATGTGGTTTCAGTGAGGGGTGGTTATAGGAGCTATGGCAGGAGATGGGGTCA

GAAAGGTGGGCAGGGACCTGGTATTCCGTGCCAAGAAACAGGGACAGGGAGACATGGCCC

AAGCCTCGGGAGTGAGGGTATAGAA

>3699 len=145 tot-seq=2

GCAAGAGGCAGGGAGAAGGAAAATCAGAGGGAGAGGAGGGGAAGGAGGAGCCTGGAAAGA

GAGGGAAAGGGAGTGGGAGGGGAGCTGAGAAAGGGGAGGTGGGGAGAGGCCAGGGAGCAG

AGGGAGAGAGGAGAAGAGGGGGCAG

>3700 len=145 tot-seq=2

CTGTTAAAGACATCAACACTCTTTCAAACGATGGAGCACAAAGCCTGAGAACAGCTGATT

CACCAGATTGACACACCTTGCTTTCAAGTTCTGTGGCTCATTCCTTTGTAACACTTCACA

TCAAATTTTCTCTCTCCAACCTATC

>3701 len=146 tot-seq=2

CAATGGACAAAAGTCTGATGTAGCAACGCCGCGTGAGTGAAGAAGGTTTTCGGATAGTAA

AGCTCTGTTGTTGGTGAAGAAGGATAGAGGTAGTAAATGGAATTTATTTGACGGTAATCA

ACCAGAAAGTCACGGCTAACTACGTG

>3702 len=146 tot-seq=2

CAATGGACGCAAGTCTGATGGAGCAACGCCGCGTGAGTGAAGAAGGGTTTCGGCTCGTAA

AGCTCTGTTGGTAGTGAAGAAAGATAGAGGTAGTAACTGTTCATTCGATGACGGTATCTA

ACCAGCAAGTCACGGCTAACTACGTG

>3703 len=146 tot-seq=2

CAATGGACGCAAGTCTGATGGAGCAACGCCGCGTGAGTGAAGAAGGTTTTCGGCTCGTAA

AGCTCTGTTGTTGGTGAAGAAGGACAGAGGTAGTAACTGACCCTTGTTTGACGGTAATCA

ACCAGAAAGTCACGGCTAACTACGTG

>3704 len=146 tot-seq=2

CAATGGACGCAAGTCTGATGGAGCAACGTCGCGTGAGTGAAGAAGGGTTTCGGCTCGTAA

AGCTCTGTTGTTGGTGAAGAAAGACAGAGGTAGTAACCGACCTTTGTTTGACGGTAATTA

ATTAGAAAGTCACGGCTAACTACGTG

>3705 len=146 tot-seq=2

CTCCCTGCTGAGCCTGTGAACAGTAGGAGGCCCAGGGGCCTGGGTGTGGCAGCTTCTGTG

TCTGGGCTTTCCAAGCTCCAGCTTGGCTGGCCCTCTTTCCTGATGGAGGACCTGTTGAGG

GAGCACTGCTCCCCTGACCGGAATGA

>3706 len=146 tot-seq=2

GGGGATGGAGTGGGAAGGTGGTCTTCCCCTGGAGTTGGGCCGCTCAGCTGCCGGGCTCTC

AGCTGAATTCCCCTTGGCGTCCATGTCATCGACAGCCTACTGACATCTGTCAGTGTGTTC

TTCTCCCCGTGTGTTCCTCTCGATGT

>3707 len=146 tot-seq=2

TGAGGAATTTTTCACAATGGACGAAAGCCTGACGGAGCAACGCCGCGTGAGTGATGAAGG

TCTTCGGATCGTAAAACTCTGTTATTAGGGAAGAACATATGTGTAAGTAACTGTGCACAT

CTTGACGGTACCTAATCAGAAAGCCA

>3708 len=146 tot-seq=2

CTTGCCTTGCAGGAGTAAAGCCATCTTGAAGTAAAACAATCATAATGACTAATATTTGAC

TCCTGCATACCAAGGTATTCTGCAGCAAGGTCAAAAACAATGTATGTAGCATGAATAACC

CCTCATAAAATTGCTTATCTAACTTC

>3709 len=146 tot-seq=2

GAGGGGAGACATCACAGAGAGCGTGCCTAGAGGTGGGGAAATGAGAAGGGAATTGGAGGC

AGAGTGCAGAAGGAGGAAGGAACAGAAGGCTAAGTGGCTTTTGGGTTCCCCTCATCCCAA

AATGTCCTCTCAGGGTCAGCCAGGAG

>3710 len=146 tot-seq=2

CCAGAAACAGCCTGAGAAGGAAGCTCCTCTGTCTCCACCGCATCTCTGGTCGGTCCCTGT

GGCCTCTGCTCCTGCCTCCTCACTTCCCCACCATCCTTCCTTCACTTTCTCGGATTCAGA

ATTTGTGGGAGAAGTTTGACGGGTCA

>3711 len=146 tot-seq=2

ACACGGTGGGAGTGGAGTGGAGCAGGCTGTGCAGAGAATGTCTCTTTGGAGAGGAGCTTG

GAGCTTGGATCTGGCCTGCCAAGTATCTTGAGGTGGAACATGTCAGCAGAGGGAACAGCA

GTGCCGGTCTCTAAGGTGGTAATGAT

>3712 len=147 tot-seq=2

TAGTCTTTGTCTTAATATCTCCCAGTGAGGATTGTAGCTGGGGACGGCACAGACACATTC

CAGCTCCCACACGCAGGAGCTACCCGAGAAACGGCCAGATTAGACGCTGAAGGGCTTGAT

CATCAGTCTACATGATTGCTATGTTGG

>3713 len=147 tot-seq=2

CCTATTGAGTTGGGGAAGAGACAGGAGCCATGGGGAATGCTGTAAGGGCTGTGGGGACCC

AGCAGACTAGAAGAGGGAGTTGAATGTCAGCTACTTGAAGAAAGGTCCACAGTGCTGGCG

CTGAGGCCCCGGGAGACCTGAAAGAGA

>3714 len=147 tot-seq=2

GACCTTAAGTTCTATGTGGACCACATTCTGGATACTCAAAGATGTGGTGACAGACAAGAA

AATGAAGGCATTTAGACACTTGAGCCTGGTGACTAAATTAGAAGTTAACACTGTGGCATG

GACAAGCACTGGTTTTTCTAAGATCCT

>3715 len=147 tot-seq=2

TAGGGAATAGGCCACAATGGAAGAAAGTCTGATGGAGAAACGCCGCGGGAGTGAAGAAGG

TTTTCGGATAGTAAAGCTCTGTTGTTGGTGAAGAAGGATAGAGGTAGTAACTGGCCTTTA

TTTGACGGCAATCAACCAGAAAGTCAC

>3716 len=147 tot-seq=2

TCAGGTAAATTGCCATTGGCCACTTCCCCAGTTGGACTTGGAGTAGCAGGGGAAGGTTTA

GGAGTGCACAGCCTTTGAGGCAGGCAAGACATTTGTTTTTCATTCGAGGATCCAAAGATA

GCTCCAGGGTGGGTAAGCATGTGATCC

>3717 len=147 tot-seq=2

TGGGGAATTTTGGACAATGGGGGAAACCCTGATCCAGCAACTCTGCGTGAGGGACGAAAA

TCTTCGGATTGTAAACCTCTTTTGTATGGGAAGAAATCCCGCAAGGGTGTGACGGTACCA

TGCGAATAAGCCACGGCTAACTATGTG

>3718 len=147 tot-seq=2

TGGGAGACCATGAAAGCCAGGCTGGCCACCTTCACTTACCTTGGTTTGAACTCATGTTGG

AAGTGACAGTGGTGGCCGTGTGAGTGGTGCCCGTCTCGTGGGTCTCACAGGGGGGGTTGG

AGCACACCCTCTGTGTTGGGAAAGGAG

>3719 len=147 tot-seq=2

ACAATGGACGAAAGTCTGATGGAGCAACGCCGCGTGAGTGAAGAAGGTTTTCGGCTCGTA

AAGCTCTGTTGGTAGTGAAGAAAGATAGAGGGAGTAACTGGCCTTTATTCGACGGTAATT

ACTTAGAAAGGCACGGCTAACTACGTG

>3720 len=148 tot-seq=2

GGGAGTTAGTGAAGTCCTGTGGATACTCACACCCAGAGCACTGAATTCAAATGTTTTTAT

ACCTGAGATGTAGCAATGAAGGTAATATCTGGTATGATAGATGTTTGTATACCCCTCTTT

TAGGTGTAATGGATCCTTTCTTGGTTCT

>3721 len=148 tot-seq=2

GGGAGAGCCTGTCAGGAGAGGGGGGACATACCACGGCTGGAGGAGGCACTCTTCAGACCT

AGATCCAAAGCAAACCATGCTTCCTGCTGCTCGGAGCACCCAACCAGCACCGAATCTTCA

ATTCTTTGCGAATCCTGTCCGAACTTAC

>3722 len=148 tot-seq=2

TTTTTTTTTTGCTAAAAAACTTTTTTGTGGTTCCTTATCTTATTCTTCCATTTTTGTGAA

TCAGAAAACCAAAGACAATCTTTGCTAGTGTCTACACAGGCAAGGAAGCCCAGAATCTCG

AGGCTCTTCTGTGCGACCCACTTCAGTG

>3723 len=148 tot-seq=2

CTGAAGTCAGCCCCTCCACCCCGAGAGTGAGTCAGAGCAGGCTCACCAGCTAGGAGATGT

GCTCTGCTCATTCACCAGTGAAAGCCTCCTTTGTCCCTCCTCCATAGGGAAGGACAATGT

CGTGCCCTGGTTTCTTTGTGTTCAGGGG

>3724 len=148 tot-seq=2

CACAATGGACGCAAGTCTGATGGAGCAACGCCGCGTGAGTGAAGAAGGGTTTCGGCTCGT

AAAGCTCTGTTGGTGGCGAAGAAAGACAGAGGTAGTAACTGACCTTTATTTGACGGTAAT

CAATTAGAAAGTCACGGCTAACTACGTG

>3725 len=148 tot-seq=2

CAGCAAGAAGAGGAGGACTTGCAGAAGCTGTCCCTGCAGCCCCCCTACTATGATCTGGGG

GTCTCCCCCTCCTACCACCCCTCGGTAAGGACAACCGAACCTCGAGGAGAGTGCCCCTAG

CCCAGGACCACCCAGCCTGGCCATGAGC

>3726 len=148 tot-seq=2

AAAAATGAGAGAAAGATTGATGGAGAGAAAAAGAGTGAAAGATGAAGGTATTAGGATTGT

AAAGTGATGTTATAAGGGAAGAAGATTTGGAATAGGAAATGATTGAAGACTGACGGTACC

TTGTCAGAAAGCGATGGCTAACTATGTG

>3727 len=148 tot-seq=2

GAAGGACCCGCCAGGAAAGGGGGCGGCTGGAAGCAGCAGTGGGGAGAAGGCTGAGCGCAG

CTTTGTGTGGATTATATTTTGTCTCTCAGATTCCCTTCCAGAGCGTCACTCCTGTTCCGA

ACCTCAGTGCAAACCAGAAAGGGGCCCC

>3728 len=149 tot-seq=2

GGGCCAGCCCTTGGCAAATTGTTTCAGGTAATGAAGATGTCTCTGACATCTTTTAGTTCT

TCTCCCTCATCCCCCTTTCCAGGTCTGAGTTCTGAAGTTAGGAGGCTTCAGCGTCACACA

ATGGGATGGTGGGACGTCCAGTTCCACAC

>3729 len=149 tot-seq=2

ACGGGAGGCAGCAGGGGGGAATATGGCAAAAGGGGGGAAACCAGGAGGCAGCGACGACGA

GGGAAGGAAGAAGGAGCTCGGTATGGAAAAGTCTAGCAGAAGGGAAGAGAGTGAAGGGAC

CTGACTAAGAAGACCCGGCTAACTACGTG

>3730 len=149 tot-seq=2

AGCTGCAGCAGAGAGCAGGCAGAAGCAGCTAGGTGAACAGTGGGCTTAACTCTGGGGTGC

TTGCCTGGCACCAGGCAGAGGGCTGGCTGGGAATGGCGGTTGTACTCACCACCACAGGGG

GACACAAAATAGCCCAGGCAGTGTCAGGG

>3731 len=149 tot-seq=2

TAGGGAATCTTCCACTATGGGCGAAAGCCTGATGCAGCGACGCCGCGTGAGTGAAGAAGT

ATTTCGGTATGTAAAGCTCTATCAGCAGGGAAGAAAGGCTCGTAAGAGAGATGACGGTAC

CTGACTAAGAAGCCCCGGCTAACTACGTG

>3732 len=149 tot-seq=2

CCGCAGCTGGACATGCCGCTGGTCCTGGACCAGGGCCTGACCAGGCAGATGCGGCTACGC

GTGGAGAGCCTGAAGCAGCGCGGGGAGAAGCGCCAGGATGGGGAGAAGCTGCTGCAGCCA

GCGGAGTCTGCGTACCGCCTCAACTTCAC

>3733 len=149 tot-seq=2

GGAGGCTTGGTGGGTGGGGCAGCTAGCTTGAGTCTGCCCTGGCAGCGTCCCCTGCTGATC

CTTGCTCCCCGTGTCCTGGCTAGGGAGGATCACCCCGACCTAATCCAGAATGCCAAGAAA

TCGGACATCCCAGAGAAGCCCAAAACCCC

>3734 len=149 tot-seq=2

GATATTCAGAGGCTGGGCTGGCAGTTCCCATCCCGTGGTCGCAGAGTGCTATGGAGGGGA

GATAATAGGTGTGATTTCTGTGCAGAGGATGGACAGGGCTCCAGGGACAGAGGATGCAGG

AGTGACAGGCATCATCAGATACCATTTGT

>3735 len=150 tot-seq=2

TCCACAATGGACGAAAGTCTGATGGAGCAACGCCGCGTGAGTGAAGAAGGTTTTCGGATC

GTAAAGCTCTGTTGTTGGTGAAGAAGGACGGGGGTAGTAACTGACCTTTGTTTGACGGTA

ATCAATTAGAAGGTCACGGCTAACTACGTG

>3736 len=150 tot-seq=2

GGCAAGCCGCTCATAGCGCACAGAGGTCCAGCGGCCAGGGAGAAGCCAGTACTTGTAACT

ATGCTGGGCCCGGGGTGGCGCTTCTTCCATCAGTGTCTGCTCCTGGGATTCGTGCAGGGA

GTCCTGGTCCAGCAGGTCAAACTGCAGTCC

>3737 len=151 tot-seq=2

ATATTGCACAATGGGGGAAACCCTGATGCAGCGACGCGTGAAGGAAGAAGGTTTTCGGAT

TGTAAACTTCTGTTATAAAGGAAGAACGGCGGCTACAGGAAATGGTAGCCGAGTGACGGT

ACTTTATTAGAAAGCCACGGCTAACTACGTG

>3738 len=151 tot-seq=2

CAGGCAGGTGGTGAGTAGTCCGTGCCTCATGCTCTGAACTGGCACAAGGAAATGTGTTCA

CAAAGTTTCTCAGCTCACAGCCCAGAAAGTACAGCCCCCTTTCTCCTAAATTGCATCAGT

AGAAGAGGATCTCTTGGGACGACTTGTGTGA

>3739 len=151 tot-seq=2

TTAAAAAATGGACGCAAGTCTGATGGAGCAACGCCGCGTGAGTGAAGAAGGTTTTAGGAT

AGTAAAGATATGTTGTTGGGGAAGAAGGAGAGAGGGAGGAAATGGAATTTATTTGAAGGT

AATCAACCAGAAAGTCACGGCTAACTACGTG

>3740 len=151 tot-seq=2

TGGGGAATATTGGACAATGGGCGGAAGCCTGATCCAGCAACGCCGCGTGAGGGATGACTG

CCTTCGGGTTGTAAACCTCTTTCATCATTGACGAAGTCCAGCTTTTTGTTGGGTGACGGT

AGGTGGAGAAGAAGCACCGGCCAACTACGTG

>3741 len=151 tot-seq=2

CACAGCAGAAACCAAAAGGTGAAATGCATATGAGAGAATCAAAGCCACGCACAGTTTTCT

AAGTTATAGCTATCTCTCAGGTCAATCCCCAGTTTGAAGTCTCCCTTGCTTCTGAAGGGT

TCAAAGTAGCTGAACACAACTTTTCCATTAC

>3742 len=152 tot-seq=2

TAGGGAATCTTCCACAATGGACGAAAGTCTGATGGAGCGACGCCGCGTGAGTGAAGAAGT

ATTTCGGTACGTAAAGCTCTATCAGCAGGGAAGAAAGAAAGTCCTTTGGACTTTGGACGG

TACCTGACCAAGAAGCCCCGGCTAACTACGTG

>3743 len=152 tot-seq=2

TAGGGAATATTGCACAATGGGGGAAACCCTGATGCAGCAACGCCACGTGTGGGAAGAAGC

ATTTCGGTGTGTAAACCACTGTCGTGAGGGAATAAGGCTGAGTTTCGGCTCAGTTGAATG

TACCTCGAGAGGAAGCACCGGCAAACTTCGTG

>3744 len=152 tot-seq=2

GAGGACGGCACAGGAGAGGAAGGCCTGGGGAGATGAGGACCCGGCCTGGCTGGGAAGAGG

CCCCTGGAGGCTGAGGGAGACCAGGCCCAGGCCATTGGGCCACGTCATGCGCAGACACCA

CCCCTGGCCTGGTAGGAGCCAGCATCTGGTTC

>3745 len=152 tot-seq=2

TGGGGAATTTTGCACAATGGACGCAAGCCTGATGCAGCGACGCCGCGTGAGGGATGAAGG

CCTTCGGGTTGTAAACCTTTTTCGCTCATGGTCAAGCCATACTGTTGGGTGTGGTGAGGG

TAGTGGGTAAAGAAGCGCCGGCTAACTACGTG

>3746 len=152 tot-seq=2

TGGGGAATATTGCACAATGAGCGCAAGCTTGATGCAGCGACGCCGCGTGGAGGGTGTAGG

CCTTCGGGTTGTTAACTCCTTTTGCCCGCGAAAAAGGCACGATTTCGGTCGTGTTGATGG

TAGCGGGAGAAAAAGCACCGGCTAACTACGTG

>3747 len=152 tot-seq=2

TGGGGAATATTGCGCAATGGGGGAAACCCTGACGCAGCGACGCCGCGTGCGGGATGAAGG

CCTTCGGGTTGTGAACTCCTTTTTCTCGCGAAAAAGGCACAGTTTTGGCTGTGTTGATGG

TAGTGGGGGAAGAAGCGCCGGCTAACTACGTG

>3748 len=152 tot-seq=2

TAGGGAATCTTCCACAATGGACGCAAGTCTGATGGAGCAACGCCGCGTGGAGGGTGTAGG

CCTTCGGGTTGTGAACTCCTTTTTCTCGCGAAAAAGGCACAGTTTTGGCTGTGTTGATGG

TAGTGGGGGAAGAAGCGCCGGCTAACTACGTG

>3749 len=152 tot-seq=2

TGGGGAATATTGCACAATGGGGGAAACCCTGATGCAGCGACGCCGCGTGAGTGAAGAAGT

AATTCGTTATGTAAAGCTCTATCAGCAGGGAAGAAAAAGGCATACATGCCTAAATGACGG

TACCTGAATAAGAAGCCCCGGCTAACTACGTG

>3750 len=152 tot-seq=2

TGGGGAATATTGCACAATGGGCGCAAGCCTGATGCAGCGACGCCGCGTGAGGGATGGAGG

CCTTCGGGTTGTGAACTCCTTTTTCTCGCGAAAAAGGCACAGTTTTGGCTGTGTTGATGG

TAGTGGGGGAAGAAGCGCCGGCTAACTACGTG

>3751 len=152 tot-seq=2

TGGGGAATATTGCACAATGGACGGAAGTTTGATGCAGCGACGCCGCGTGGAGGGTGTAGG

CCTTCGGGTTGTGAACTCCTTTTTCTCGTGAAAAAGGCATGCTTTTGGGTGTGTTGACGG

TACCTTACTAAGAAGCTCCGGCTAAATACGTG

>3752 len=152 tot-seq=2

TAGGGAATCTTCGGCAATGGACGGAAGTCTGACCGAGCAACGCCGCGTGAGTGAAGAAGG

TTTTCGGATCGTAAAGCTCTGAAGAACGAGTGTGAGAGTGGAAAGTTCACACTGTGACGG

TATCTTACCGGAAAGGGACGGCTAACTACGTG

>3753 len=152 tot-seq=2

CTACGTTAACCTTGGCCAAGTCCTGGCAGCACGAAATTTGCAAGGACCTGTGAGATATGG

AGCCAGTAATGCTTTGCAGCTGACAGATGAAAGACAAACAATAAAAACAGAGGTGAGAGG

GGAAGCCTGTAACTGTCTTACCCCCACCAGTG

>3754 len=153 tot-seq=2

TTTTCCCTAAATGGCAGATTGGCATTGGAAGTTGATTAATTCGGTGGATAAGAAGCTTAA

CGTGGTTTTAGAGCCAAAAGCAGTAAAAGTTTATTCGAACAAAAAAATTTTTTTAATGTG

GTTCTGTTTTCCAAGCCTGTTTGTGCATCTTTG

>3755 len=153 tot-seq=2

ATTGGTCAGTGACTGGACTTCAGACGTGTGCTCTTCCGATCTCCTACTGGAGGCAGCAGG

GATGGCAGGGCAGAGGGAGGCAGGAGACTCTTTAGCTTTAGGAGGCAGTGGTAGGATCCT

GGAGTGGAGGACGGTGCCTTGCCACAGTGGCAG

>3756 len=153 tot-seq=2

AGGAATATTGGTCAATGGACGCAAGTCTGAACCAGCCAAGTAGCGTGCAGGATGACGGCC

CTATGGGTTGTAAAATGATTTTATATGGGGATAAAGTGGGGAAAGTGTTAACTTTTGCAG

GTACCATATGAATAAGGACCGGCTAATTCCGTG

>3757 len=153 tot-seq=2

ATTCAAACAACAGAGGGAAAGCCCTGGAGTCCTAAGTAACAGAAAAACTTTCAGACCCTC
ATATCAGTGTTCTGAAATTCCAAGTGAGGGGGAAACACTCAGAACCCAGCAGCTGTGTTC
TGGAATCCATTGTGACGGACTTACAAGTGGACC
>3758 len=153 tot-seq=2
TGGGGAATATTGCGCAATGGGGGAAACCCTGACGCAGCAACGCCGCGTGAGGGATGAAGG
CCTTCGGGTTGTAAACCTTTTTCGCTCATGGTCAAGCCGCAGCTTTGGGTTGTGGTGAGG
GTAGTGGGTAAAGAAGCGCCGGCTAACTACGTG
>3759 len=153 tot-seq=2
CAGGTGGATCACCTGAGGTCAGGAGTTTGAGACCAGCCTGGCCAATAAGGTGAAAACTCG
TCTCTACTACTAAAAAAAAAATTAGCCAGGCATGGTGGCGGGTGCCTGTAATCCCAGCTA
CTCAGGAGGCTGAGGCAGGATAATCCCTTGAAC
>3760 len=153 tot-seq=2
ATATATAGATAGAAATCCAAAAGTAGAGTGAAAGAATGGGAATATGGTAGAATCTGTTTT
ATAGCTAAAAGTGCTATGTTTGTGACACATTAATTAGACACTCAATGGGGACTCCCAAGA
TTGGAACAGTGGAGTTGAGCTTCCACAATCAAC
>3761 len=153 tot-seq=2
TTTATCTAGAACCTGCTTTAGCAACGTTTCCAAAGTCAGGAAGCAGGGATCTGCTGTGAG
TTTATCTGGAATGTGAAGGAAGCAGGTGAAGACCAGCTTAATCTCTTGAAAATGGGGAGA
GGGGGCCGGGCACGGTGGCTCACGCCTGAAATC
>3762 len=154 tot-seq=2
ATGCAAAGGTCTAAAGGGGGCTTCTCTAGCAGAGAGTTGGGAGAAAGTCCATTGTGGCTA
GTACAATTAGGTGAAAGAGGCAGGTGGCAGAAAAGTCTGGAGAGAGAATCATCCCATCGG
TGACCACAGAAGCAGGACACTGACCTACCTACTG
>3763 len=154 tot-seq=2
AATCATTAGCAGTCAGGCTGTGTGAATTTTTTTGTGGTAAGAGAGATAAATGTCCCTCTT
GTTCTTTGTGGTTTCCTTTGCCATAAGAGCCACTGCTTTGTGAAAGTTCTTCTTTCCTTT
CTCTGGCTTGCCCATCCCTGGATTGATGTAGTAG
>3764 len=154 tot-seq=2
ATATTAAAAAATGGAAGAAAGTATGATGGAGAAAAGAAGAGTGAATGAAGAAGGAATTAG

GGTAGTAAAATTATGTTGTAAGAGAAGAAAGTGAGTGAGAGTAACTGTTCACGTATTGAC

GGTATCTGACCAGAAAGCCACGGCTAACTACGTG

>3765 len=154 tot-seq=2

TGAGGAATATTGGTCAATGGACGAGAGTCTGAACCAGCCAAGTCGCGTGAAGGAAGACGG

ATCTATGGTTTGTAAACTTCCTTAGTGTGGGAACAAAGCGTGGTTGCGACCGCAGATGAG

TGTACCACAAGAATAAGCATCGGCTAACTCCGTG

>3766 len=154 tot-seq=2

TGGGGAATATTGCACAATGGGGGGAACCCTGATGCAGCGACGCCGCGTGGGTGAGGAAGG

CCTTCGGGTTGTAAAGTACTTTCAGTCGGGAGGAAGGTGTCAAGGTTAATAACCTTGGCA

ATTGACGTTACCGACAGAAGAAGCACCGGCTAAC

>3767 len=154 tot-seq=2

TGTAGTGCTATTTACTAAAAATAACAGGTTTTATTCAGAATGACACGGTCCTTGCTCAAG

AACAACAGGAGTAACCAGAGAATGAGGGGAGATAAGTTAGCAATCAGTATTGATAAAGAA

ACATTTGAGAAACACCCCAAAACTGAAGTACTGA

>3768 len=155 tot-seq=2

TGAGGAATATTGGTCAATGGGCTAAGGCCTGAACCAGCCAAGTCGCGTGAGGGAAGACGG

TCCTATGGATTGTAAACCTCTTTTGTCGGGGAGCAACGAGGCCACGTGTGGCCGAAGTGA

GAGTACCCGAAGAAAAAGCATCGGCTAACTCCGTG

>3769 len=155 tot-seq=2

GCTAAGATGTTTGGGAACCAGCTGCAAGCCACATAGATCATTTTCTTTGAATAGAGAGCA

ATTGCACAGCGCCTGGAGTTGCTATCTTCTCCTCCCATGTCAGCCAGGCTGGCTTGTCTG

TCACAGAGCTGTTTGAGCTCAGAGTTCTCTCTTCA

>3770 len=155 tot-seq=2

TGAGGAATATTGGTCAATGGGCGCGAGCCTGAACCAGCCAAGTAGCGTGAAGGATGAAGG

CTCTATGGGTCGTAAACTTCTTTTATACGGGAATAAACGAATCCACGTGTGGATTTTTGC

ATGTACCGTATGAATAAGGATCGGCTAACTCCGTG

>3771 len=155 tot-seq=2

TAGGGAATCTTCCGCAATGGGCGTAAGCCTGAACCAGCCAAGTCGCGTGAGGGATGAAGG

TTCTATGGATCGTAAACCTCTTTTATAAGGGAATAAAGTGCGGGACGTGTCCTGTTTTGT

ATGTACCTTATGAATAAGGATCGGCTAACTCCGTG

>3772 len=155 tot-seq=2

TGAGGAATATTGGTCAATGGACGAGAGTCTGAACCAGCCAAGTAGCGTGAAGGATGAAGG

TTCTATGGATTGTAAACTTCTTTTATACGGGAATAAAGTTTTCCACGTGTGGAATTTTGT

ATGTACCATATGAATAAGGATCGGCTAACTCCGTG

>3773 len=155 tot-seq=2

TGAGGAATATTGGTCAATGGACGAGAGTCTGAACCAGTCAAGTAGCGTGAAGGATGAAGG

TTCTATGGATCGTAAACCTCTTTTATAAGGGAATAAAGTGCGGGACGTGTCCTGTTTTGT

ATGTACCTTATGAATAAGGATCGGCTAACTCCGTG

>3774 len=155 tot-seq=2

TAAGGAATATTGGTCAATGGAGGCAACTCTGAACCAGCCATGCCGCGTGAAGGATGAAGG

CCCTCTGGGTTGTAAACTTCTTTTGTAAGGGAAGAAATACAGACATTCTTGTTTGTTTGA

CGGTACCTTAAGAATAAGCACCGGCTAACTCCGTG

>3775 len=155 tot-seq=2

TGGGGAATTTTCCGCAATGGGCGATGGCCTGAACCAGCCAAGTAGCGTGAAGGATGACTG

CCCTATGGGTTGTAAACTTCTTTTATAAAGGAATAAAGTCGGGTATGCATACCCGTTTGC

ATGTACTTTATGAATAAGGATCGGCTAACTCCGTG

>3776 len=155 tot-seq=2

TGAGGAATATTGGTCAATGGACGCAAGTCTGAACCAGCCATGCCGCGTGCAGGAAGAATG

TGCTATGCATTGTAAACTGCTTTTGTACGAGGGTAATAACAGGTACGTGTACCTGGATGA

AAGTATCGTACGAATAAGGATCGGCTAACTCCGTG

>3777 len=155 tot-seq=2

TGGAGAATATTGCACAATGGGGGAGAGCCTGAACCAGCCAAGTAGCGTGAAGGATGACTG

CCCTATGGGTTGTAAACTTCTTTTATAAAGGAATAAAGTCGGGTATGTATACCCGTTTGC

ATGTACTTTATGAATAAGGATCGGCTAACTCCGTG

>3778 len=155 tot-seq=2

TGAGGAATATTGGTCAATGGACGCAAGTCTGAACCAGCCATGCCGCGTGCAGGAAGACGG

CTCTATGAGTTGTAAACTGCTTTTGTATGAGGGTAAAATTAGATACGTGTATCTAACTGC

AAGTATCATACGAATAAGGATCGGCTAACTCCGTG

>3779 len=155 tot-seq=2

TAAGGAATATTGGTCAATGGAGGCAACTCTGAACCAGCCATGCCGCGTGCAGGAAGACTG

CCCTATGGGTTGTAAACTGCTTTTATCCGGGAATAAACCTCTCTACGTGTAGGGAGCTGA

ATGTACCGGAGGAATAAGGATCGGCTAACTCCGTG

>3780 len=155 tot-seq=2

TGAGGAATATTGGTCAATGGGCGAGAGCCTGAACCAGCCAAGTAGCGTGCAGGATGACGG

CCCTATGGGTTGTAAACTGCTTTTATGCGGGGATAAAGTGTGCCATGTATGGTGTTTTGC

AGGTACCGCATGAATAAGGACCGGCTAATTCCGTG

>3781 len=155 tot-seq=2

TAAGGAATATTGGTCAATGGGCGGAAGCCTGAACCAGCCATGCCGCGTGCAGGATGACTG

CCCTACGGGTTGTAAACTGCTTTTGTCCAGGAATAAACCTTTCTACGTGTAGGAAGCTGA

ATGAACTGGAAGAATAAGGATCGGCTAACTCCGTG

>3782 len=155 tot-seq=2

TGAGGAATATTGGACAATGGGTTAGCGCCTGATCCAGCCATCCCGCGTGAAGGACGACGG

CCCTATGGGTTGTAAACTTCTTTTGTACAGGGATAAACCTATTTACGTGTAAATAGCTGA

AGGTACTGTACGAATAAGCACCGGCTAACTCCGTG

>3783 len=155 tot-seq=2

TGAGGAATATTGGTCAATGGGCGTAAGCCTGAACCAGCCAAGTAGCGTGAAGGATGACGG

CCCTACGGGTTGTAAACTTCTTTTGTCGGGGAATAAACCGTCCCACGTGTGGGGCGCTGC

ATGTACTCGGTGAATAAGGACCGGCTAATTCCGTG

>3784 len=155 tot-seq=2

TGAGGAATATTGGTCAATGGGCGAGAGCCTGAACCAGCCAAGTAGCGTGCAGGATGACGG

CCCTATGGGTTGTAAACTGCTTTTATATGGGAATAAAGTGAGGCACGTGTGCCTTTTTGT

ATGTACCGTATGAATAAGGATCGGCTAACTCCGTG

>3785 len=155 tot-seq=2

TGGGGAATATTGCACAATGGGCGAGAGTCTGAACCAGCCAAGTAGCGTGCAGGATGACGG

CCCTATGGGTTGTAAACTGCTTTTATAAGGGAATAAAGTGAGTCTCGTGAGACTTTTTGC

ATGTACCTTACGAATAAGGACCGGCTAATTCCGTG

>3786 len=155 tot-seq=2

TGAGGAATATTGGTCAATGGACGCAAGTCTGAACCAGCCACGTCGCGTGAAGGAAGACGG

CCCTACGGGTTGTAAACTTCTTTTGTAAGGGAATAAAGTGCAGTACGTGTACTGTTTTGC

ATGTACCTTACGAATAAGGATCGGCTAACTCCGTG

>3787 len=155 tot-seq=2

TAGGGAATTTTCGGCAATGGGGGAAACCCTGACCGAGCAACGCCGCGTGAGTGATGAAAG

TCTTCGGATTGTAAAGCTCTGTTGTAAGGGAGGAACGGCTTGAGGAGAACTCAAGAGTGA

CGGTACCTTACCAGAAAGCCACGGCTAACTACGTG

>3788 len=155 tot-seq=2

TGAGGAATATTGGACAATGGGTGGAAGCCTGATCCAGCCATCCCGCGTGAAGGATGAAGG

TTCTATGGATTGTAAACTTCTTTTATATAGGGATAAACCTATCCTCGTGAGGGTAGCTGA

AGGTACTATATGAATAAGCACCGGCTAACTCCGTG

>3789 len=155 tot-seq=2

TGAGGAATATTGGTCAATGGACGCAAGTCTGAACCAGCCATGTCGCGTGAAGGATGACTG

TCCTATGGATTGTAAACTTCTTTTATATGGGGATAAAGTGTGAGACTTGTCTCATTTTGC

ATGTACCATATGAATAAGCATCGGCTAACTCCGTG

>3790 len=155 tot-seq=2

TGAGGAATATTGGTCAATGGGCGCAAGCCTGAACCAGCCATGCCGCGTGCAGGAAGACGG

TCCTATGGATTGTAAACTGCTTTTATACAGGAAGAAACACCTCCACGTGTGGAGGCTTGA

CGGTACTGTAGGAATAAGGATCGGCTAACTCCGTG

>3791 len=155 tot-seq=2

TGAGGAATATTGGTCAATGGGCGATAGCCTGAACCAGCCAAGTCGCGTGAGGGAGGAAGG

CTCTATGGGTCGTAAACCTCTTTTATAAGGGATTAAAGTTGGGGTCGTGACCCCGTTGGA

AGGTACCTTATGAATAAGCATCGGCTAACTCCGTG

>3792 len=155 tot-seq=2

TGAGGAATATTGGTCAATGGAGGCAACTCTGAACCAGCCACGTCGCGTGAAGGATGACGG

CCCTACGGGTTGTAAACTTCTTTTGTAAGGGAATAAAGTTAGTTACGTGTAACTATTTGC

ATGTACCTTACGAATAAGGATCGGCTAACTCCGTG

>3793 len=155 tot-seq=2

TAAGGAATATTGGTCAATGGACGGAAGTCTGAACCAGCCATGCCGCGTGCAGGATGACTG

CCCTATGGGTTGTAAACTGCTTTTGTCGAGGAATAAACCTATCTACGTGTAGATAGCTGA

ATGTACTCGAAGAATAAGGATCGGCTAACTCCGTG

>3794 len=155 tot-seq=2

TGAGGAATATTGGTCAATGGACGAGAGTCTGAACCAGCCAAGTCGCGTGGGGGATGACGG

TCCTACGGATTGTAAACCTCTTTAGGCGGGGAGTAATGTGCTCTACGAGTAGAGTAGTGA

GAGTACCCGCAGAATAAGCATCGGCTAACTCCGTG

>3795 len=155 tot-seq=2

CGAGGAATATTGGTCAATGGGCGAGAGCCTGAACCGGCCAAGTAGCGTGCAGGATGACGG

CCCTATGGGTTGTAAACTGCTTTTACGCGGGGATAAAGCGCGTGACGTGTCGCGCATCGC

AGGTACCGCGTGAATAAGGACCGGCTAATTCCGTG

>3796 len=155 tot-seq=2

TGAGGAATATTGGGCAATGCCCGCAAGGGTGACCCAGCAACGCCGCGTGGAGGATGAATG

CCGTAAGGTTGTAAACTCCTGTTAGAGGGGAAGAATAATTCCGATCAAATCGGAACTTGA

CTGTACCCTCAGAGAAAGCCCCGGCTAACTACGTG

>3797 len=155 tot-seq=2

TGGGGAATCTTCCGCAATGGACGAAAGTCTGACGGAGCAACGCCGCGTGAGTGATGACGG

CCCTATGGGTTGTAAACTGCTTTTATATGGGGATAAAGTGGGGAACGTGTTCCCTTTTGC

AGGTACCATATGAATAAGGACCGGCTAATTCCGTG

>3798 len=155 tot-seq=2

TGAGGAATATTGGTCAATGGGCGAGAGCCTGAACCAGCCAAGTAGCGTGAAGGATGACTG

CCCTATGGGTTGTAAACTTCTTTTATATGGGAATAAAGTAGGCTATGTATAGCCTTTTGT

ATGTACCATATGAATAAGGATCGGCTAACTCCGTG

>3799 len=155 tot-seq=2

TGAGGAATATTGGTCAATGGCCGAGAGGCTGAACCAGCCAAGTCGCGTGAGGGATGAAGG

TTCTATGGATCGTAAACTTCTTTTATATAAGAATAAAGTGCAGTATGTATACTGTTTTGT

ATGTATTATATGAATAAGGATCGGCTAACTCCGTG

>3800 len=155 tot-seq=2

TGGGGAATATTGCACAATGGGCGCAAGCCTGAACCAGCCAAGTAGCGTGAAGGATGAAGG

CTCTATGGGTCGTAAACTTCTTTTATATAAGAATAAAGTGCAGTATGTATACTGTTTTGA

ATGTATTATATGAATAAGGATCGGCTAACTCCGTG

>3801 len=155 tot-seq=2

TGGGGAATATTGCGCAATGGGGGAAACCCTGACGCAGCGACGCCGCGTGCGGGATGAAGG

CCCTATGGGTTGTAAACTGCTTTTATGCGGGGATAAAGGAGTCCACGTGTGGGCTTTTGC

AGGTACCGCATGAATAAGGACCGGCTAATTCCGTG

>3802 len=155 tot-seq=2

TGAGGAATATTGGTCAATGGGCGAGAGCCTGAACCAGCCAAGTCGCGTGAAGGAAGAATG

TCCTATGGATTGTAAACTTCTTTTGCAGGAGAATAAAGTGCACCACGTGTGGTGTTTTGT

ATGTACCCTGCGAATAAGGATCGGCTAACTCCGTG

>3803 len=155 tot-seq=2

TGAGGAATATTGGTCAATGGGCGAGAGCCTGAACCAGCCAAGTCGCGTGAGGGAAGACGG

TCCTACGGATTGTAAACCTCTTTTGTCGGGGAGCAATAAGGCGTACGTGTACGCAGATGC

GAGTACCCGAAGAAAAAGCATCGGCTAACTCCGTG

>3804 len=155 tot-seq=2

TGAGGAATATTGGTCAATGGGCGAGAGCCTGAACCAGCCAAGTCGCGTGAGGGAAGAATG

GTCTATGGCCTGTAAACCTCTTTTGTCAAGGAAGAATAAGCGGCACGAGTGCCACCTTGC

CAGTACTTGACGAATAAGCATCGGCTAACTCCGTG

>3805 len=155 tot-seq=2

TGGGGAATTTTGGACAATGGGGGCAACCCTGATCCAGCCATGCCGCGTGCAGGATGACGG

CCCTATGGGTTGTAAACTGCTTTTATAAGGGAATAAAGTGAGAGTCGTGACTCTTTTTGC

ATGTACCTTATGAATAAGGACCGGCTAATTCCGTG

>3806 len=155 tot-seq=2

TGGGGAATATTGCACAATGGGCGAAAGCCTGAACCAGCCAAGTAGCGTGCAGGATGACGG

CCCTATGGGTTGTAAACTGCTTTTATGCGGGGATAAAGTCACCCACGTGTGGGTGTTTGC

AGGTACCGCATGAATAAGGACCGGCTAATTCCGTG

>3807 len=155 tot-seq=2

TGGGGAATATTGCACAATGGGGGAAACCCTGATGCAGCGACGCCGCGTGAGCGAGGAAGT

ATTTCGGTATGTAAAGCTCTGTCAGCAGGGAAGAAGAGAGTTGAAATACACTCTCCAATG

ACGGTACCTGAAGAAGAAGCACCGGCTAAATACGT

>3808 len=155 tot-seq=2

TGAGGAATATTGGTCAATGGACGGAAGTCTGAACCAGCCAAGTCGCGTGAAGGATGAAGG

TTCTATGGATTGTAAACTTCTTTTGTCTGAGGGTAAAAAAGCGGACGTGTCCGTTCTTGC

AAGTATCAGACGAATAAGCATCGGCTAACTCCGTG

>3809 len=155 tot-seq=2

TGAGGAATATTGGTCAATGGGTGAGAACCTGAACCAGCCAAGTAGCGTGCAGGATGACGG

CCCTATGGGTTGTAAACTGCTTTTGTACGGGGATAAAGGAGCTTACGTGTAAGTTTTTGC

AGGTACCGTACGAATAAGGACCGGCTAATTCCGTG

>3810 len=155 tot-seq=2

TGAGGAATATTGGTCAATGGTCGGAAGACTGAACCAGCCAAGTAGCGTGAGGGACGACTG

CCCTATGGGTTGTAAACCTCTTTTATATGGGAATAAAAAAGTCTACGTGTAGACAATTGT

ATGTACCTTATGAATAAGCATCGGCTAATTCCGTG

>3811 len=155 tot-seq=2

TGAGGAATATTGGTCAATGGGCGAGACCTTGAACCAGCCAAGTAGCGTGCAGGATGACGG

CCCTATGGGTTGTAAACTGCTTTTACGCGGGGATAAAGTGCGTGACGTGTCGCGCATCGC

AGGTGCCGCGTGAATAAGGACCGGCTAATTCCGTG

>3812 len=155 tot-seq=2

TGAGGAATATTGGTCAATGGGCGCAAGCCTGAACCAGCCATGTAGCGTGAAGGATGACGG

TTTTTTTGATTGTAAACTTCTTTTCTGTGCGATTAAAGTGTGGCATTTATGCCATATTGC

AGTTATCTCAGGAATAAGCATCGGCTAACTCCGTG

>3813 len=155 tot-seq=2

TGAGGAATATTGGTCAATGGGCGGAAGCCTGAACCAGCCATGCCGCGTGAAGGAATAAGG

TCCTATGGATCGTAAACTTCTTTTGAGCGGGAGCAATAAGGTCTACGAGTAGACCGATGA

GAGTACCGTTCGAATAAGCATCGGCTAACTCCGTG

>3814 len=155 tot-seq=2

TGGGGAATATTGCGCAATGGGGGAAACCCTGACGCAGCGACGCCGCGTGCGGGATGACGG

CCCTATGGGTTGTAAACTGCTTTTACGCGGGGATAAAGTGCGTGACGTGTCTCGCATTGC

AGGTACCGCGTGAATAAGGACCGGCTAATTCCGTG

>3815 len=155 tot-seq=2

TGAGGAATATTGGTCAATGGGCGAGAGCCTGAACCAGCCAAGTCGCGTGAGGGATGAAGG

TTCTATGGATCGTAAACCTCTTTTATAAGGGAATAAAGTTTTCCACGTGTGGAATTTTGT

ATGTACCATATGAATAAGGATCGGCTAACTCCGTG

>3816 len=155 tot-seq=2

TGAGGAATATTCCACAATGGGCGAAAGCCTGATGGAGCAATGCCGCGTGCAGGATGACGG

CCCTATGGGTTGTAAACTGCTTTTATATGGGGATAAAGTGGGGAACGTGTTCCCTTTTGC

AGGTACCATATGAATAAGGACCGGCTAATTCCGTG

>3817 len=155 tot-seq=2

TGAGGAATATTGGTCAATGGACGAGAGTCTGAACCAGCCAAGTAGCGTGCAGGATGACGG

CCCTATGGGTTGTAAACTGCTTTTATGCGGGGATAAAGGAGTCCACGTGTGGGCTTTTGC

AGGTACCGCATGAATAAGGACCGGCTAATTCCGTG

>3818 len=155 tot-seq=2

TGAGGAGTATTGGTCAATGGACGCAAGTCTGAACCAGCCATGCCGCGTGCAGGAAGACGG

CTCTATGAGTTGTAAACTGCTTTTGTACGAGGGTAAACGCTCTTACGCGTAAGAGCCTGA

AAGTATCGTACGAATAAGGATCGGCTAACTCCGTG

>3819 len=155 tot-seq=2

TGAGGAATATTGGTCAATGGGCGAGAGCCTGAACCAGCCAAGTAGCGTGCAGGAAGACGG

CCCTACGGGTTGTAAACTGCTTTTATGAAGGAATAAAGTCACCCACGTGTGGGTGTTTGC

ATGTACTTTATGAATAAGGACCGGCTAATTCCGTG

>3820 len=155 tot-seq=2

TGGGGAATATTGCACAATGGGGGAAAGCCCGACCGAGCAACGCCGCGTGAGTGATGACGG

CCTTCGGGTTGTAAAGCTCTGTTGTAAGAAAAGATAAACTAGTAGAGAATATTAGTTAGA

CATTATCTTACCAGAAAGCCACGGCTAACTACGTG

>3821 len=155 tot-seq=2

TGGGGAATATTGGACAATGGAGGAAACTCTGATCCAGCAATTCTGTGTGTGTGATGACGG

CCCTATGGGTTGTAAACTGCTTTTATATGGGGATAAAGTGGGGAACGTGTTCCCTTTTGC

AGGTACCATATGAATAAGGACCGGCTAATTCCGTG

>3822 len=155 tot-seq=2

TGAGGAATCTTGCACAATGGGCGAAAGCCTGATGCAGCGACGCCGCGTGCGGGATGAAGG

CCTTCGGGTTGTAAAGCTCTGTTGTAAGAAAAGATAAACTAGTAGAGAATATTAGTTAGA

CATTATCTTACCAGAAAGCCACGGCTAACTACGTG

>3823 len=155 tot-seq=2

TGGGGAATATTGCGCAATGGGGGAAACCCTGACGCAGCGACGCCGCGTGCAGGATGACGG

CCCTATGGGTTGTAAACTGCTTTTGTTTGGGAATAATCGACATTACGTGTAATGTTTTGC

ATGTACCATTCGGATAAGGACCGGCTAATTCCGTG

>3824 len=155 tot-seq=2

TGGGGAATCTTCCGCAATGGACGAAAGTCTGACGGAGCAGCGCCGCGTGAGTGATGACGG

CCTTCGGGTTGTAAAGCTCTGTTGTAAGAAAAGATAAACTAGTAGAGAATATTAGTTAGA

CATTATCTTACCAGAAAGCCACGGCTAACTACGTG

>3825 len=155 tot-seq=2

TGGGGAATCTTGCACAATGGGCGAAAGCCTGAACCAGCCAAGTAGCGTGCAGGATGACGG

CCCTATGGGTTGTAAACTGCTTTTGTTTGGGAATAATCGACATTACGTGTAATGTTTTGC

ATGTACCATTCGAATAAGGGCCGGCTAATTCCGTG

>3826 len=155 tot-seq=2

TGAGGAATATTGGTCAATGGGCGAGAGCCTGAACCAGCCAAGTCGCGTGAGGGAAGACGG

TCCTACGGATTGTAAACCTCTTTTGTCGGGGAACAAAAGGCGCCACGCGTGGTGTATAGA

GTGTACCCGAAGAAAAAGCATCGGCTAACTCCGTG

>3827 len=155 tot-seq=2

TGAGGAATATTGGTCAATGGGCGCAAGCCTGAACCAGCCAAGTAGCGTGAAGGATGACTG

CCCTATGGGTTGTAAACTTCTTTTATATGGGAACAATGTGCTCTACGTGTAGAGTTTTGC

GTGTACCATATGAATAAGCATCGGCTAACTCCGTG

>3828 len=155 tot-seq=2

TGAGGAATATTGGACAATGGGTGAGAGCCTGATCCAGCCATCCCGCGTGAAGGACGACGG

CCCTATGGGTTGTAAACTTCTTTTGTACAGGGATAAACCTTTCCACGTGTGGAAAGCTGA

AGGTACTGTACGAATAAGCACCGGCTAACTCCGTG

>3829 len=155 tot-seq=2

TGAGGAATATTGGTCAATGGACGAGAGTCTGAACCAGCCAAGTAGCGTGCAGGATGACGG

CCCTATGGGTTGTAAACTGCTTTTGTACGGGGATAAAGTGAGCCACGTGTGGTTTATTGC

AGGTACCGTACGAATAAGGACCGGCTAATTCCGTG

>3830 len=155 tot-seq=2

TGAGGAATATTGGTCAATGGTCGAGAGACTGAACCAGCCAAGTAGCGTGATGGACGACGG

CCCTACGGGTCTTAAACATCTTTTATATGAGAATAACAAGGGGAACGCGTTCCCCCCTGC

ATGTACCATATGAATAAGCATCGGCTAATTCCGTG

>3831 len=155 tot-seq=2

TAAGGAATATTGGACAATGGCCGCAAGGCTGATCCAGCCATGCCGCGTGCAGGAAGAAGG

CCCTATGGGTCGTAAACTGCTTTTGAACGAGAGAAAACCCTTTTACGTGTAAGAGGCTGA

TAGTATCGTTAGAATAAGCATCGGCTAACTTCGTG

>3832 len=155 tot-seq=2

TGGGGAATATTGCGCAATGGGGGAAACCCTGACGCAGCCAAGTCGCGTGAGGGATGAAGG

TTCTATGGATCGTAAACCTCTTTTATAAGGGAATAAAGTGCGGGACGTGTCCCGTTTTGT

ATGTACCTTATGAATAAGGATCGGCTAACTCCGTG

>3833 len=155 tot-seq=2

TGGGGAATATTGGACAATGGAGGGAACTCTGAACCAGCCAAGTAGCGTGCAGGATGACGG

CCCTATGGGTTGTAAACTGCTTTTGTTTGGGAATAATCGACATTACGTGTAATGTTTTGC

ATGTACCATTCGAATAAGGACCGGCTAATTCCGTG

>3834 len=156 tot-seq=2

CGTGGTGAAGGAGGTCCCCAGGCCTGTCTCGGCCAGACGCATCTTCCAGGGAAGCAGAAG

AAATCAGCCGGAGGAATAACCAAGACAGCTGCTTTGCTATTTCTTGAAGGGGCTTAAAGA

GGAGATCGGGTTCTCTCCGTTTGTTCTGTGCACGAG

>3835 len=156 tot-seq=2

TGAGGAATATTGGTCAATGGGCGAGAGCCTGAACCAGCCAAGTAGCGTGCGGGATGAAGG

CCCTTGTGGTCGTAAACCGCTTTTATCAGTGAATAAAGTGCACCACGTGTGGTGTCATTG

CAGGTAGCTGAAGAAAAAGGACCGGCTAATTCCGTG

>3836 len=156 tot-seq=2

GAGCCATTGAAGGATTTTCAGCAGGGAAGCATCGAGATCCGAGTTTCATCTTCCTCATTC

CTCCATTTATGGTGCTCTTGTAGCATTTGGTTCTTACACAGCTGCATGAGGGTTTGCCAT

GGTGTACTGCAATTGTTTGTCTACATGTCTTCTCTC

>3837 len=156 tot-seq=2

TGGGGAATATTGCACAATGGGCGCAAGCCTGATGCAGCAACGCCGCGTGAAGGAAGACTG

CCCGCAAGGGTTGTAAACTTCTTTTGTATGGGATTAAAGTCGTCTACGTGTAGACGTTTG

CAGTTACCATACGAATAAGCATCGGCTAACTCCGTG

>3838 len=156 tot-seq=2

TGGGGAATATTGCGCAATGGGGGAAAGCCTGAACCAGCCAAGTCGCGTGAAGGAAGACTG

CCCGCAAGGGTTGTAAACTTCTTTTGTATGGGATTAAAGTCGTCTACGTGTAGACGTTTG

CAGTTACCATACGAATAAGCATCGGCTAACTCCGTG

>3839 len=156 tot-seq=2

GCACAGCGGTGATGCCATGAACCACAGGGCATGAGTTATGAGTGGCTGCGAATGAATGAG

AAACACCTATCAGCCCAGGCAATATGGAAGCACCAGGTAAGAGCCACAGGCTGTTTCCTA

GCAGGCTGCTACAAACACACTGAAAATGGCATGTGA

>3840 len=156 tot-seq=2

TGGGGAATATTGCACAATGGGGGAAACCCTGAACCAGCCAAGTCGCGTGAGGGAAGACGG

TCCTATGGATTGTAAACCTCTTTTGCCGGGGAGCAAGCTGTGTCACGTGTGACACAGCTG

AGAGTACCCGGAGAAAAAGCATCGGCTAACTCCGTG

>3841 len=156 tot-seq=2

TGAGGAATATTGGTCAATGGGCGAGAGCCTGAACCAGCCAAGTCGCGTGAAGGAAGAATG

TCCTAAGGATTGTAAACTTCTTTAGCGAGCGAGTAAGGACTTCCACGAGATGGGAGTTTG

AAAGTAGCTCGAGAATAAGTATCGGCTAACTCCGTG

>3842 len=156 tot-seq=2

TGAGGAATATTGGTCAATGGGCGAGAGCCTGAACCAGCCAAGTCGCGTGAAGGAAGACTG

CCCGCAAGGGTTGTAAACTTCTTTTGTATGGGATTAAAGTCGTCCACGTGTGGGCTTTTG

CAGGTACCGCATGAATAAGGACCGGCTAATTCCGTG

>3843 len=156 tot-seq=2

TGGGGAATATTGCACAATGGGCGCAAGCCTGAACCAGCCAAGTCGCGTGAAGGAAGACTG

CCCGCAAGGGTTGTAAACTTCTTTTGTATGGGATTAAAGTCACCTACGTGTAGGTGTTTG

CAGTTACCATACGAATAAGCATCGGCTAGCTCCGTG

>3844 len=156 tot-seq=2

TGAGGAATATTGGTCAATGGGCTTACGCCTGAACCAGCCAAGTCGCGTGAGGGAAGACTG

CCCTATGGGTTGTAAACCTCTTTTGTCAGGGAGCAACAACATCTACGAGTAGGTGTTACG

AGAGTACCTGAAGAAAAAGCATCGGCTAACTCCGTG

>3845 len=156 tot-seq=2

TAGGGAATATTGGGCAATGGGCGCAAGCCTGACCCAGCCATGCCGCGTGCAGGAAGAAGG

CCTTCTGGGTTGTAAACTGCTTTTGACAGGGAAGAAAAGGCTTTACGTGTAAGGCAATCG

ACGGTACCTGTAGAATAAGCACCGGCTAACTCCGTG

>3846 len=156 tot-seq=2

TGAGGAATATTGGTCAATGGGCGCATGCCTGAACCAGCCAAGTCGCGTGAGGGACGACGG

CCCTACGGGTTGTAAACCTCTTTTGCCGGGGAGCAAGGCTTTCCACGTGTGGGGAGCCGG

AGAGTACCCTGAGAAAAAGCATCGGCTAACTCCGTG

>3847 len=156 tot-seq=2

TGGGGAATCTTCCGCAATGGACGAAAGTCTGACGGAGCAACGCCGCGTGAGTGAAGACTG

CCCGCAAGGGTTGTAAACTTCTTTTGTATGGGATTAAAGTCACCTACGTGTAGGTGTTTG

CAGTTACCATACGAATAAGCATCGGCTAACTCCGTG

>3848 len=157 tot-seq=2

CTCCTGATGAGGAGCCTTTCAGCAGATCCTGCCCACTCCCGGCTCCATGGTCTGATTCTG

AGACAGCCCATGCCTGAGTAGAGGTTTTGGCTGGGGTGTGAGCCTGGCACTCAGCCCCTT

CCTGGGTCCTTCCACGGCCTCGGCTGGCTCCCAGCCT

>3849 len=157 tot-seq=2

TGAGGAATATTGGTCAATGGTCGCGAGACTGAACCAGCCAAGTAGCGTGCAGGATGACGG

CCCTCTGGGTTGTAAACTGCTTTTAGTTGGGAATAAAAGGCGGTACGTGTACCGCGTATT

GTATGTACCATCAGAAAAAGGACCGGCTAATTCCGTG

>3850 len=157 tot-seq=2

CTCCTACGGGAGGCAGCAATGGGGAATATTGGGCAATGGGCGAAAGCCTGACCCAGCGAC

GCCGCGTGAGGGAAGAAGATCTTCGGATTGTAAACCTTAGTCAACAGGGAAGAAGAAAGT

GACGGTACCTGTGGAGGAAGCCACGGCTAACTACGTG

>3851 len=157 tot-seq=2

AGGGAATCTTCCACAATGGACGCAAGTCTGATGGAGCAACGCCGCGTGAGTGAAGAAGGG

TTTCGGCTCGTAAAGCTCTGTTGTTGGTGAAGAAGGAAAGGTAGTAACTGACCTTTCCAG

GACGGTAGATGAGGAGGAAGCCCCGGCTAACTATGTG

>3852 len=157 tot-seq=2

AGTAGGAACCAACAGAATCTATCTTCCTACCTAGACAATAATTACACTGGCAGAATCAGT

GCAATATAACTATTTTGGAACTCTGGAGTCTATTGAAGGCTTGCAAATTCCAGGTGAAGG

TTAGAACAGTAAATTGCAGTCAATTTCAGCTCTTAGC

>3853 len=157 tot-seq=2

TTTTTTTTCAAGCAGAAGACGGCATACGAGATTACCAAGTGACTGGAGTTCAGACGTGTG

CTTTCCGATCTCCTACGGGAGCAGCAGAGACTGAAGGACAATGGGCTTTGTTGGACAACC

CAGATATCCCTGCACAGGCAGGGTGTACACAACTACA

>3854 len=158 tot-seq=2

GAAGGAGGAAGGGCCGTGGTGGCTGCCGGTGGAGAACAGGCCCATTATCGACAGTAGGAG

TGTCATGGGGCCTGGTCATTTCTGTTCCTCCTGAGAATGTTATTTTAAAGAAAAGTCTTC

CTCATGACTGAAGGAGATTTTCACACGTAAGACATAGA

>3855 len=158 tot-seq=2

GTTCCAGTCTAACTGCCTGAGGCATGCTATATATCCTGGGGTATGTTGCATGTATTCTGT

ATGTCAGGTCTAGTCAACTTTATTAAAGTTATGTAATACTTGGCACATACATAAATTATA

GTGACAGAGCCAGTGCAATGCGGTGGAGAGCCCTAAAG

>3856 len=158 tot-seq=2

TGGGGAATATTGCACAATGGGCGCAAGCCTGATGCAGCCATGCCGCGTGTGAAGAAGACC

TTCGGGTTGTAAAGCACTTTCAGCGGGGAGGAAGGCGTTAAGGTTAATAACCTCATCGAT

TGACGTTACCCGCAGAAGAAGCACCGGCTAACTCCGTG

>3857 len=158 tot-seq=2

TATCAATAATGCGGTAAGGCACTTCCACCTTCGCAAGCATTTCCTGCTCCATGCCGAGCA

ACTTCTGATGCTCAGCGCGCGAATCTTCCTGCTTGCAGTACACGAACATTTCCACCTTAT

CGAACTGGTGCACGCGAATAATGCCGGAAGTATCCTTA

>3858 len=158 tot-seq=2

CTTCCACAATGGACGCAAGTCTGATGGAGCAACGCCGCGTGAGTGAAGAAGGGTTTCGGC

TCGTAAAGCTCTGTTGTTGGTGAAGAAGGACAGGGGTAGTAACTGTAACTGACCTTTGTT

TGACGGTAATCAATTAGAAAGTCACGGCTAACTACGTG

>3859 len=158 tot-seq=2

TGGGGAATATTCCGCAATGGGCGCAAGCCTGACGGAGCAACGCCGCGTGAATGAAGAAGG

TTTTCGGATTGTAAAGTTCTGTCATTAGGGACGAAGGACCTGTGCGAATAGTGCAGGGGA

TGACGGTACCTAAGGAGGAAGCTCCGGCTAACTACGTG

>3860 len=159 tot-seq=2

CACCCCTCCACTGCAGCTTCTATCTGACTGGTAGAAGTAGTCTCCAGATTCCATCTCCCC

ACTGTTGAGCATTTTGGCTAAGGCAATCCACACTGAGTCCTTGGAGCCTCCCCTATGGCA

GGTCTCTGGGACTTCTAGAGATTCACCCCAGTCCTACCC

>3861 len=159 tot-seq=2

CCGATCTCCTACGGGAGGCTGCAGTGGGGAATATGGCACAATGGGGGAAACCCTGATGCA

GCGACGCCGCGTGAAGGAAGAAGTATCTCGGTATGTAAACGTCTATCAGCAGGGAAGAGA

TTGACGGTACCTGACTAAGAAGCCCCGGCTAACTACGTG

>3862 len=159 tot-seq=2

TGGGGAATTTTGCGCAATGGGCGAAAGCCTGACGCAGCAACGCCGCGTGTGTGATGAAGG

TCTTCGGATTGTAAAGCACTTTCGACCGGGACGAAAACCCGTTGGCTAACATCCAACGGC

TTGACGGTACCGGGAGAAGAAGCACCGGCTAACTCTGTG

>3863 len=159 tot-seq=2

TGGGGAATTTTGCACAATGGGGGAAACCCTGATGCAGCGACGCCGCGTGATTTAGAAGGC

CTTCGGGTTGTAAAACCCTGTTGTTAGAGAAGAAAGTGCGTGAGAGTAACTGTTCACGTT

TCGACGGTATCTAACCAGAAAGCCACGGCTAACTACGTG

>3864 len=159 tot-seq=2

TAGGGAATCTTCGGCAATGGACGGAAGTCTGATGGAGCAACACCGCGTGAGTGAAGAAGG

GTTTCGGCTCGTAAAACTCTGTTGTTGAAGAAGAACGTGCGTGAGAGTAACTGTTCACGC

AGTGACGGTATTCAACCAGAAAGTCACGGCTAACTACAT

>3865 len=159 tot-seq=2

TAGGGAGTATTTGGCAATGGGGGAAACCCTGACCGAGCAACGCCGCGTGTGCGATGGAGT

CCCTCAGGGACGTGAAGCACTGTGGGGGGCCGCCAAACGGGCGGGCAGGAAATGGCCCGT

CCCCGAGGAGCCCCCTAGAAAGCGACTGCTAACCACGTG

>3866 len=159 tot-seq=2

TGGGGAATCTTGCGCAATGGGCGGAAGCCTGACGCAGCAACGCCGCGTGAGGGAAGACGG

CCTTCGGGTTGTAAACCTCTTTCAGGGGGGACGAAGCCTCCGGGGTTAATAGCCCACGGG

GTGACGGTACCCCCAGAAGAAGCCCCGGCTAACTACGTG

>3867 len=159 tot-seq=2

TGGGGAATATTGCACAATGGGGGAAACCCTGATGCAGCCATGCCGCGTGTGTGAAGAAGG

CCTTCGGGTTGTAAAGCACTTTTGCATTCGAGGAAGACAGCGTATAAACAAATGCGCTAT

TTGACGTTAGAGTGTGAATAAGCACCGGCAAACTCCGTG

>3868 len=159 tot-seq=2

GTTTGGCAGGACATGGGCTCGTGCAGTCCTTTGGCTCCAATATTCTATTTGATGTAGTTG

TCATTATCCGATCCTTCCCCCAGCAGGTAGAGGAGGCACCTTGTTGCAGAATGAACATGC

CCACCTGCTAACAGCCCATCTGCTGACTATAGGAGCCCA

>3869 len=159 tot-seq=2

TGGGGAATATTGCACAATGGGCGAAAGCCTGATGAGCAACGCCGCGTGAAGGAAGAAGGT

TTTCGGATTGTAAACTTCTGTTATAAAGGAAGAACGGCGGCTACAGGAAATGGTAGCCGA

GTGACGGTACTTTATTAGAAAGCCACGGCTAACTACGTG

>3870 len=159 tot-seq=2

TAGGGAATCTTCCGCAATGGGCGAAAGCCTGACGGAGCAACGCCGCGTGAGTGATGAAGG

TCTTCTGATTGTAAAGTTCTTTTATTTAGGAAAAAAAGCGTGCTAGGAAATGAGCGCGCC

TTGATGGTACTAATTGAATAAGTGACAGCTAACTATGTG

>3871 len=159 tot-seq=2

TGGGGAATCTTGCGCAATGGGCGAAAGCCTGACGCAGCGACGCCGCGTGAGTGATGAAGG

CCTTCGGGTTGTAAAGCTCTGTGGAGGGGGACGAATAAGGCATGGCTAACATCCATGTCG

ATGACGGTACCCCTTTAGCAAGCACCGGCTAACTCTGTG

>3872 len=159 tot-seq=2

TTTTTTTTTTCAAGCAGAAGACGGCATACGAGATCAAGTGGTGACTGGAGTTTAGACGTG

TGCTCTTCCGATCTACTACGGGAGGCAGCAGCAGTTGGAACAGCAAGTCTTCCAGTCTCT

CCTGAGGCAGAGACCTAAAGCATGAACGAACTCCTTAGC

>3873 len=159 tot-seq=2

ATTATCCCAGCAGCGTGAGCTTTGCAAAATTCCCGTCTCGTGGCTGAGCAAACCTGCTCG

ATGTGCCACAACTTTCCGTCAGTAACATGCCTATATGCTCATTTTCAAACATTATCTCTG

CCAGGAGGGATAATTTGCTGTAATATGAGGATGAGACAT

>3874 len=159 tot-seq=2

GTGTCTGATAGCTGTACTGTGCTTTACGGTTCATGAGCACGCACACATAGGCCCATAATA

TCATATCTCTGGTCACCCTGGTAGGTAGATATTATTGCCCCAGGTTTCAGGTAAGAAGAC

TAAAGACCAGGGGTCAATTGCTTGCTCAGAGTCCCACAG

>3875 len=159 tot-seq=2

CCCCAGGAGGACACATCAGTAACGATAATGGTGGTGATGATAATGACATGATAATAATAG

CAGTGATGATGATGATGATCATGATTAACAGCCATTATCTACTGAGTGCCTGAATTTGTG

TCAGACACTGTCCAGCACTGTCACCCGGGTCAGGTGCCA

>3876 len=159 tot-seq=2

TGAGGAATTTTTCACAATGGACGAAAGTCTGATGGAGCAATACCACGTGAACGATGAAGG

TCTTCTGATTGTAAAGTTCTTTTATTTAGGAAAAAAAGCGTGCTAGGAAATGAGTGCGCC

TTGATGGTACTAATTGAATAAGGACCGGCTAATTCCGTG

>3877 len=160 tot-seq=2

TGGGGGATATTGGACAATGGGGGAAACCCTGATCCAGCCATGCCGCGTGTCTGAAGAAGG

CCTTCGGGTTGTAAAGGACTTTTGTCAGGGAAGAAAAGGCTGTTGCTAATACCAACAGCT

GATGACGGTACCTGAAGAATAAGCACCGGCTAACTACGTG

>3878 len=160 tot-seq=2

TGGGGGATATTGGACAATGGGGGAAACCCTGATCCAGCGACGCCGCGTGAGTGAAGAAGG

CCTTCGGGTCGTAAAGCTCTGTTGCGGGGGAAAAAAGGCAGCATCAGGAAATGGGTGCTG

ACTGATGGTGCCCCGCCAGAAAGTCACAGCTAACTACGTG

>3879 len=160 tot-seq=2

TAGGGAATTTTCGTCAATGGGCGCAAGCCTGAACGAGCGATGCCGCGTGAGTGAAGAAGG

CCTTCGGGTCGTAAAGCTCTGTTGGTAGTGAAGAAAGATAGAGGTAGTAACTGGCCTTTA

TTTGACGGTAATTACTTAGAAAGTCACGGCTAACTACGTG

>3880 len=160 tot-seq=2

TGGGGAATTTTGGACAATGGGCGCAAGCCTGATCCAGCCATGCCGCGTGTCTGAAGAAGG

CCTTCGGGTCGTAAAGCTCTGTTGCGGGGGAAAAAAGGCAGCATCAGGAAATGGGTGCTG

ACTGATGGTGCCCCGCCAGAAAGTCACGGCTAACTACGTG

>3881 len=160 tot-seq=2

TAGGGAATCTTCGGCAATGGACGGAAGTCTGACCGAGCAACGCCGCGTGAGTGAAGAAGG

TTTTCGGATCGTAAAGCTCTTTTGTCAGGGAAGAAAAGGTGATCTCTAATACAGGTCACT

CATGACGGTACCTGAAGAATAAGCACCGGCTAACTACGTG

>3882 len=160 tot-seq=2

TGGGGAATATTGGACAATGGGCGAAAGCCTGATGGAGCAACGCCGCGTGAGTGATGAAGG

TCTTCGGATCGTAAAACTCTGTTATCAGGGAAGAACAAATGTGTAAGTAACTGTGCACAT

CTTGACGGTACCTGATCAGAAAGCCACGGCTAACTACGTG

>3883 len=160 tot-seq=2

TGGGGAATATTGCACAATGGGCGCAAGCCTGATGCAGCCATGCCGCGTGTATGAAGAAGG

CCTTCGGGTTGTAAAGTACTTTCAGCGGGGAGGAAGGGAGCAAAGTTAATACCTTTGCTC

ATTGACGTTAAATACAGAAGAAGCACCGGCTAACTCCGTG

>3884 len=160 tot-seq=2

TGGGGAATATTGGACAATGGGCGCAAGCCTGATCCAGCCATGCCGCGTGAGTGATGAAGG

TCTTCGGATCGTAAAACTCTGTTATCAGGGAAGAACAAACGTGTAAGTAACTGTGCACGT

CTTGACGGTACCTGATCAGAAAGCCACGGCTAACTACGTG

>3885 len=160 tot-seq=2

TGGGGAATATTGGACAATGGGGGAAACCCTGATCCAGCCATGCCGCGTGTGTGAAGAAGG

CCCTCGGGTTGTAAAGTTCTTTCGGTATTGAGGAAGGTTGATGTGTTAATAGTACATCAA

ATTGACGTTAAATACAGAAGAAGCACCGGCTAACTCCGTG

>3886 len=160 tot-seq=2

TAGGGAATCTTCCGCAATGGGCGAAAGCCTGACGGAGCAACGCCGCGTGAGTGATGAAGG

CCCTCGGGTTGTAAAGCTCTTTTGTCAGGGAAGAAAAGGTGATCTCTAATACAGGTCACT

CATGACGGTACCTGAAGAATAAGCACCGGCTAACTACGTG

>3887 len=160 tot-seq=2

TGGGGAATATTGCGCAATGGGGGGAACCCTGACGCAGCCATGCCGCGTGAATGAAGAAGG

CCTTAGGGTTGTAAAGTTCTTTCAGCGGGGAGGAAGGGAGTAAAGTTAATACCTTTGCTC

ATTGACGTTACCCGCAGAAGAAGCACCGGCTAACTCCGTG

>3888 len=160 tot-seq=2

TGGGGAATATTGCACAATGGGCGCAAGCCTGATGCAGCCATGCCGCGTGAATGAAGAAGG

CCTTAGGGTTGTAAAGTTCTTTCGGTATTGAGGAAGGTTGATGTGTTAATAGCACATCAA

ATTGACGTTAAATACAGAAGAAGCACCGGCTAACTCCGTG

>3889 len=160 tot-seq=2

TGAGGAATATTGGTCAATGGACGAGAGTCTGACCGAGCAACGCCGCGTGAGTGAAGAAGG

TTTTCGGATCGTAAAACTCTGTTGTTAGAGAAGAACAAGGACGTTAGTAACTGAACGTCC

CCTGACGGTATCTAACCAGAAAGCCACGGCTAACTACGTG

>3890 len=160 tot-seq=2

TGGGGAATTTTGGACAATGGGCGCAAGCCTGATCCAGCAATGCCGCGTGTGTGAAGAAGG

CCTTCGGATTGTAAAGCACTTTAAGTTGGGAGGAAGGGCAGTTGCCTAATACGTAACTGT

TTTGACGTTACCGACAGAATAAGCACCGGCTAACTCTGTG

>3891 len=160 tot-seq=2

TGGGGAATATTGGACAATGGGCGCAAGCCTGATCCAGCCATGCCGCGTGTGTGAAGAAGG

CCTTAGGGTTGTAAAGCACTTTCAGTGGGGAGGAAGGGGTGAATGTTAATAGTATTCATC

TTTGACGTTACCCACAGAAGAAGCACCGGCTAACTCCGTG

>3892 len=160 tot-seq=2

TGGGGAATTTTGGACAATGGGCGAAAGCCTGATCCAGCAATGCCGCGTGTGTGAAGAAGG

CCTTCGGGTTGTAAAGCACTTTTGTCCGGAAAGAAATCCCTGATCCTAATATGGCCGGGG

GATGACGGTACCGGAAGAATAAGCACCGGCTAACTACGTG

>3893 len=160 tot-seq=2

TGGGGAATATTGGACAATGGGGGGAACCCTGATCCAGCCATGCCGCGTGTGTGAAGAAGG

TCTTCGGATTGTAAAGCACTTTAAGTTGGGAGGAAGGGTTTGCAGTTAATACCTGCGAAT

TTTGACGTTACCAACAGAATAAGCACCGGCTAACTTCGTG

>3894 len=160 tot-seq=2

TGGGGAATATTGCACAATGGGCGCAAGCCTGATGCAGCCATGCCGCGTGTATGAAGAAGG

CCTTCGGGTTGTAAAGCTCTTTTGTCAGGGAAGAAAAGGTGATCTCTAATACAGGTCACT

CATGACGGTACCTGAAGAATAAGCACCGGCTAACTACGTG

>3895 len=160 tot-seq=2

TGGGGAATATTGCACAATGGGCGCAAGCCTGATGCAGCCATGCCGCGTGTATGAAGAAGG

CCTTCGGGTTGTAAAGCCCTTTTGTTGGGAAAGAAATCCAGCCGGCTAATACCTGGTTGG

GATGACGGTACCCAAAGAATAAGCACCGGCTAACTTCGTG

>3896 len=160 tot-seq=2

TAGGGAATCTTCCACAATGGGCGCAAGCCTGATGGAGCAACGCCGCGTGAATGAAGAAGG

TCTTCGGATCGTAAAGTTCTGTTGTTAGAGAAGAACACGTGTGAGAGTAACTGTCCATGC

GTTGACGGTATCTAACCAGCAAGTCACGGCTAACTACGTG

>3897 len=160 tot-seq=2

TAGGGAATTTTCGGCAATGGGCGAAAGCCTGACCGAGCAACGCCGCGTGAGGGAGGAAGT

ATTTCGGTATGTAAACCTCTGTTATAAAGGAAGAACGGCGCATGTAGGGAATGACATGCG

AGTGACGGTACTTTATGAGAAAGCCACGGCTAACTACGTG

>3898 len=160 tot-seq=2

TGGGGAATTTTGGACAATGGGCGCAAGCCTGATCCAGCCATGCCGCGTGCGGGAAGAAGG

CCTTCGGGTTGTAAACCGCTTTTGGCAGGAACGAAACGGCGCGAGCGAATACCTTGCGCT

AATGACGGTACCTGCAGAATAAGCACCGGCTAACTACGTG

>3899 len=160 tot-seq=2

TAGGGAATCTTCCACAATGGACGCAAGTCTGATGGAGCAACGCCGCGTGAGTGAAGAAGG

TCTTCGGATCGTAAAGTACTTTCAGCAGGGAGGAAGGGAGTAAAGTTAATACCTTTGCTC

ATTGACGTTACCCGCAGAAGAAGCACCGGCTAACTCCGTG

>3900 len=160 tot-seq=2

TAGGGAATCTTCGGCAATGGGGGCAACCCTGACCGAGCAACGCCGCGTGAGTGAAGAAGG

TTTTCGGATCGTAAAGCTCTGTTGTAAGAGAAGAACGTGTGTGAGAGTAACTGTTCACGT

TTCGACGGTATCTAACCAGAAAGCCACGGCTAACTACGTG

>3901 len=160 tot-seq=2

TAGGGAATCTTCCACAATGGGCGAAAGCCTGATGGAGCAACGCCGCGTGGGTGAAGAAGG

TCTTCGGATCGTAAAACCCTGTTGTTAGAGAAGAAAGTGCGTGAGAGTAACTGTTCACGT

TTCGACGGTAATTACTTAGAAAGTCACGGCTAACTACGTG

>3902 len=160 tot-seq=2

TGGGGAATTTTCGACAATGGGGGGAACCCTGATCGAGCCATCTCGCGTGTGGGACGAAGG

CCTTCGGGTTGTAAACCACTTTTCTCAACGAAGAAAAGTCTTAGGCTAATACCTTAAGAT

GCTGACGGTAGTTGAGGAATAAGCACCGGCTAACTACGTG

>3903 len=160 tot-seq=2

TAGGGAATCTTCCGCAATGGGCGAAAGCCTGACGGAGCAACGCCGCGTGAGTGAAGAAGG

ATTTCGGTTCGTAAAGCTCTGTTGTTGGTGAAGAAGGACAGGGGTAGTAACTGACCTTTG

TTTGACGGTAATCAATTAGAAAGTCACGGCTAACTACGTG

>3904 len=160 tot-seq=2

TAGGGAATCTTCCACAATGGACGCAAGTCTGATGGAGCAACGCCGCGTGAGTGATGACGG

TCTTCGGATTGTAAAGCTCTGTTGTTGGTGAAGAAGGACAGGGGTAGTAACTGACCTTTG

TTTGACGGTAATCAATTAGAAAGTCACGGCTAACTACGTG

>3905 len=160 tot-seq=2

TAGGGAATCTTCCACAATGGACGCAAGTCTGATGGAGCAACGCCGCGTGTCTGAAGAAGG

CCTTCGGGTTGTAAAGGACTTTTGTCAGGGAAGAAAAGGGCGGGGTTAATACCCCTGTCT

GATGACGGTACCTGAAGAATAAGCACCGGCTAACTACGTG

>3906 len=160 tot-seq=2

TGGGGAATTTTGCACAATGGGGGAAACCCTGATGCAGCGACGCCGCGTGATTTAGAAGGC

CTTCGGGTTGTAAAACTCTGTGATTCGGGACGAAAGATAAGTAGACGAATAATCTGCATA

AGTGACGGTACCGAAAAAGCAAGCCACGGCTAACTACGTG

>3907 len=160 tot-seq=2

TGGGGAATCTTCCGCAATGGGCGAAAGCCTGACGGAGCAATGCCGCGTGAGTGAAGAAGG

CCTTCGGGTTGTAAAGCTCTATCCTTGTCCAAGAGAGCAGAGTATAGTAACTGATAGTCT

GAGGACGGTAGACAAGAAGGAAGCCCCGGCTAACTATGTG

>3908 len=160 tot-seq=2

TGGGGGATATTGCGCAATGGGCGCAAGCCTGATGCAGCCATGCCGCGTGTATGAAGAAGG

CCTTCGGGTTGTAAAGTACTTTCAGCGGGGAGGAAGGTGTTGTGGTTAATAGCCGCAGCA

ATTGACGTTACCCGCAGAAGAAGCACCGGCTAACTCCGTG

>3909 len=160 tot-seq=2

TGGGGAATTTTGGACAATGGGCGAAAGCCTGATCCAGCAATGCCGCGTGTGCGAAGAAGG

CCTTCGGGTTGTAAAGCACTTTTGTCCGGAAAGAAATCCGTCTGGCTAATACCCGGATGG

GATGACGGTACCGGAAGAATAAGCACCGGCTAACTACGTG

>3910 len=160 tot-seq=2

TGGGGAATTTTGGACAATGGGGGCAACCCTGATCCAGCCATGCTGCGTGAGTGAAGAAGG

CCTTCGGGTTGTAAAGCTCTTTCGGACGGAACGAAATTGCCTGGACCAATACTCTGGGTA

GATGACGGTACCGTAAGAAGAAGCACCGGCTAACTACGTG

>3911 len=160 tot-seq=2

TGGGGAATTTTGGACAATGGGCAAAAGCCTGATCCAGCAATGCCGCGTGTGTGAAAAAGG

CCTTCGGGTTGCAAAGCACTTTTGTCCGGAAAAAAATGGCTCTGGTTAATACCTGGGGTC

GATGACGGTACCGGAAAAATAAGGACCGGCTAACTACATG

>3912 len=160 tot-seq=2

TAGGGAATCTTCCACAATGGACGAAAGTCTGATGGAGCAACGCCGCGTGAGTGAAGAAGG

TTTTCGGATCGTAAAACTCTGTTGTTGGAGAAGAACACGTTTGAGAGTAACTGTTCAGAC

GTTGACGGTATCCAACCAGAAAGCCACGGCTAACTACGTG

>3913 len=160 tot-seq=2

TGGGGAATATTGCACAATGGGCGCAAGCCTGATGCAGCCATGCCGCGTGTATGAAGAAGG

GTTTCGGCTCGTAAAACTCTGTTGTTGAAGAAGAACGTGCGTGAGAGTAACTGTTCACGC

AGTGACGGTATTCAACCAGAAAGTCACGGCTAACTACGTG

>3914 len=160 tot-seq=2

TGGGGAATCTTGCGCAATGGGGGGAACCCTGACGCAGCGACGCCGCGTGCGGGACGGAGG

CCTTCGGGTTGTAAAGTACTTTCAGCGGGGAGGAAGGGAGTAAAGTTAATACCTTTACTC

ATTGACGTTACCCGCAGAAGAAGCACCGGCTAACTCCGTG

>3915 len=160 tot-seq=2

TGGGGAATTTTGCACAATGGGGGAAACCCTGATGGAGCAACACCGCGTGAGTGAAGAAGG

GTTTCGGCTCGTAAAACTCTGTTGTTGGAGAAGAACGTGCGTAAGAGTAACTGTTTACGC

AGTGACGGTATCCAACCAGAAAGTCACGGCTAACTACGTG

>3916 len=160 tot-seq=2

TAGGGAATATTGCACAATGGAGGAAACTCTGATGCAGCCATGCCGCGTGTGTGAAGAAGG

TTTTCGGATCGTAAAACTCTGTTGTTAGAGAAGAACAAGGACGTTAGTAACTGAACGTCC

CCTGACGGTATCTAACCAGAAAGCCACGGCTAACTACGTG

>3917 len=160 tot-seq=2

TGAGGAATATTGGTCAATGGACGCAAGTCTGAACCAGCCATGCCGCGTGTATGAAGAAGG

CCTTCGGGTTGTAAAGTACTTTCAGCGGGGAGGAAGGGAGTAAAGTTAATACCTTTGCTC

GTTGACGTTACCCGCAGAAGAAGCACCGGCTAACTCCGTG

>3918 len=160 tot-seq=2

TAGGGAATCTTCCACAATGGGCGCAAGCCTGATGGAGCAACACCGCGTGAGTGAAGAAGG

GCTTCGGCTCGTAAAACTCTGTTGTTGGAGAAGAACGTGCGTAAGAGTAACTGTTTACGT

TTCGACGGTATCTAACCAGAAAGCCACGGCTAACTACGTG

>3919 len=160 tot-seq=2

TAGGGAATCTTCGGCAATGGACGGAAGTCTGACCGAGCAACGCCGCGTGAGTGAAGAAGG

TTTTCGGATCGTAAAGCTCTGTTGTTGAAGAAGAACGTGCGTGAGGGCAACTGTTCACGC

AGTGACGGTATTCAACCAGAAAGTCACGGCTAACTACGTG

>3920 len=160 tot-seq=2

TAGGGAATCTTCCACAATGGACGCAAGTCTGATGGAGCAACGCCGCGTGGGTGAAGAAGG

TCTTCGGATCGTAAAACCCTGTTGTTAGAGAAGAAAGTGCGTGAGAGTAACTGTTCAGGT

AGTGACGGTATCCAACCAGAAAGTCACGGCTAACTACGTG

>3921 len=160 tot-seq=2

TAGGGAATCTTCCACAATGGGCGAAAGTCTGACGGAGCAACGCCGCGTGAGTGATGACGG

TCTTCGGATTGTAAAGTACTTTCAGCGGGGAGGAAGGGAGTAAAGTTAATACCTTTGCTC

ATTGACGTTACCCGCAGAAGAAGCACCGGCTAACTCCGTG

>3922 len=160 tot-seq=2

TGGGGAATTTTGCACAATGGGGGAAACCCTGATGCAGCCATGCCGCGTGTATGAAGAAGG

CCTTCGGGTTGTAAAGTACTTTCAGCGGGGAGGAAGGGAGTAAAGTTAATACCTTTACTC

ATTGACGTTACCCGCAGAAGAAGCACCGGCTAACTCCGTG

>3923 len=160 tot-seq=2

TAGGGAATCTTCCACAATGGGCGCAAGCCTGATGGAGCGACACCGCGTGAGTGAAGAAGG

GTTTCGGCTCGTAAAGCTCTGTTGTTAAAGAAGAACGTGCGTGAGAGTAACTGTTCAGGT

AGTGACGGTATCCAACCAGAAAGTCACGGCTAACTACGTG

>3924 len=160 tot-seq=2

TGGGGAATATTGCACAATGGGCGCAAGCCTGATGGAGCAACACCGCGTGAGTGAAGAAGG

TTTTCGGATCGTAAAGCTCTGTTGTTGGTGAAGAAGGATAGAGGCAGTAACTGGCCTTTA

TTTGACGGTAATCAACCAGAAAGTCACGGCTAACTACGTG

>3925 len=160 tot-seq=2

TAGGGAATCTTCCACAATGGGCGCAAGCCTGATGGAGCAACACCGCGTGAGTGAAGAAGG

TCTTCGGATCGTAAAGCTCTGTTGTAAGTGAAGAACGGCTCATAGAGGAAATGCTATGGG

AGTGACGGTAGCTTACCAGAAAGCCACGGCTAACTACGTG

>3926 len=160 tot-seq=2

TGGGGAATATTGCACAATGGAGGGAACTCTGATGCAGCGACGCCGCGTGAACGAAGAAGG

CTTTCGGATCGTAAAGCTCTGTTGTTGGTGAAGAAGGATAGAGGCAGTAACTGGCCTTTA

TTTGACGGTAATCAACCAGAAAGTCACGGCTAACTACGTG

>3927 len=160 tot-seq=2

TGGGGAATATTGGACAATGGGCGCAAGCCTGATCCAGCAATGCCGCGTGTGTGAAGAAGG

CCTGAGGGTTGTAAAGCACTTTCGTTGGGGAAGAAGGGGATGGCCTGAATAGGGTCATTT

TTTGACGGTACCTGAAGAAGAAGCACTGGCTAACTCTGTG

>3928 len=160 tot-seq=2

TGGGGAATCTTCCGCAATGGGCGCAAGCCTGACGGAGCAATGCCGCGTGAGTGAAGAAGG

CCTTCGGTTCGTAAAACTCTGTTGTTAGAGAAGAACAGCGCATAGAGTAACTGTTATGCG

TGTGACGGTATCTAACCAGAAAGCCACGGCTAACTACGTG

>3929 len=160 tot-seq=2

TGGGGAATATTGGACAATGGGCGCAAGCCTGATCCAGCCATGCCGCGTGTGTGAAGAAGG

CCTTCGGGTTGTAAAGCACTTTCAGCGAGGAGGAAGGGTTGTGTGTTAATAGTACACAGC

CTTGACGTTACTCGCAGAAGAAGCACCGGCTAACTCTGTG

>3930 len=160 tot-seq=2

TGGGGAATTTTGGACAATGGGCGCAAGCCTGATCCAGCCATTCCGCGTGAGTGAAGAAGG

CCTTCGGGTTGTAAAGCTCTTTCAGCAGGAACGAAACGGTGGGTCTTAATACGGCCTGCT

AATGACGGTACCTGCAGAAGAAGCACCGGCTAACTACGTG

>3931 len=160 tot-seq=2

TGGGGAATTTTGGACAATGGGGGAAACCCTGATCCAGCCATGCCGCGTGCGGGATGAAGG

CCTTCGGGTTGTAAAGCTCTGTTGTTGGTGAAGAAGGACAGGGGTAGTAACTGACCTTTG

TTTGACGGTAATCAATTAGAAAGTCACGGCTAACTACGTG

>3932 len=160 tot-seq=2

TAGGGAATCTTCCACAATGGACGCAAGCCTGACGGAGCAACGCCGCGTGAGTGATGAAGG

TCTCCGGATCGTAAAACTCTGTTATTAGGGAAGAACAAACGTGTAAGTAACTGTGCACGT

CTTGACGGTACCTAATCAGAAAGCCACGGCTAACTACGTG

>3933 len=160 tot-seq=2

TAGGGAATCTTCGGCAATGGGGGGAACCCTGACCGAGCAACGCCGCGTGAGTGAAGAAGG

TTTTCGGATCGTAAAACTCTGTTGTAAGAGAAGAACAAATTGTAGAGTAACTGCTACAGT

CTTGACGGTATCTTACCAGAAAGCCACGGCTAACTACGTG

>3934 len=160 tot-seq=2

TAGGGAATCTTCGGCAATGGACGAAAGTCTGACCGAGCAACGCCGCGTGAGTGAAGAAGG

TTTTCGGATCGTAAAACTCTGTTGTTAGAGAAGAACGTGGGTAAGAGTAACTGTTTACCC

AGTGACGGTATTTAACCAGAAAGCCACGGCTAACTACGTG

>3935 len=160 tot-seq=2

TGGGGAATATTGCACAATGGAGGGAACTCTGATGCAGCGACGCCGCGTGAACGAAGAAGG

CTTTCGGGTTGTAAAGTACTTTCAGCGGGGAGGAAGGGAGTAAAGTTAATACCTTTGCTC

ATTGACGTTACCCGCAGAAGAAGCACCGGCTAACTCCGTG

>3936 len=160 tot-seq=2

TGGGGAATATTGCACAATGGGGGAAACCCTGATGCAGCGACGCCGCGTGGAGGAAGAAGG

TCTTCGGATTGTAAAGCTCTGTTGTTAAAGAAGAACGTGGGTAAGAGTAACTGTTTACCC

AGTGACGGTATTTAACCAGAAAGCCACGGCTAACTACGTG

>3937 len=160 tot-seq=2

TAGGGAATCTTCCACAATGGACGCAAGTCTGATGGAGCAACGCCGCGTGAGTGAAGAAGG

GTTTCGGCTCGTAAAGCTCTGTTGTTAAAGAAGAACGTGGGTAAGAGTAACTGTTTACCC

AGTGACGGTAACTTACCAGAAAGGGACGGCTAACTACGTG

>3938 len=160 tot-seq=2

TGGGGAATATTGCGCAATGGGGGAAACCCTGACGCAGCGACGCCGCGTGCGGGATGAAGG

CCTTCGGGTTGTAAACCGCTTTTGTCAGGGACGAAAAGGACCGTGTTAATACCATGGTCT

GCTGACGGTACCTGAAGAATAAGCACCGGCTAACTACGTG

>3939 len=160 tot-seq=2

TGGGGAATATTGCACAATGGAGGAAACTCTGATGCAGCGACGCCGCGTGAAGGATGAAGT

ATTTCGGTATGTAAAGTACTTTCAGCGGGGAGGAAGGGAGTAAAGTTAATACCTTTGCTC

ATTGACGTTACCCGCAGAAGAAGCACCGGCTAACTCCGTG

>3940 len=160 tot-seq=2

TGGGGAATATTGCGCAATGGGGGGAACCCTGACGCAGCCATGCCGCGTGAATGAAGAAGG

CCTTCGGGTTGTAAAGTTCTTTCGGTATTGAGGAAGGAGTGTATGTTAATAGCATGCATT

ATTGACGTTAAATACAGAAGAAGCACCGGCTAACTCCATG

>3941 len=160 tot-seq=2

TGGGGAATATTGCGCAATGGGGGAAACCCTGACGCAGCGACGCCGCGTGCGGGATGAAGG

CCTTCGGGTTGTAAAGCTCTGTTGTTAAAGAAGAACGTGGGTAAGAGTAACTGTTTACCC

AGTGACGGTATTTAACCAGAAAGCCACGGCTAACTACGTG

>3942 len=160 tot-seq=2

TGGGGAATTTTGGACAATGGGCGCAAGCCTGATCCAGCCATTCCGCGTGAGTGAAGAAGG

CCTTCGGGTTGTAAAGCTCTTTCGCAAGGGAAGAAAACTTAACCTCTAACATAGGTTGAG

GCTGACGGTACCTTGATAAGAAGCACCGGCTAACTACGTG

>3943 len=160 tot-seq=2

TAGGGAATCTTCCACAATGGACGCAAGTCTGATGGAGCCATGCCGCGTGTGTGAAGAAGG

TCTTCGGATTGTAAAGCACTTTAAGTTGGGAGGAAGGGCAGTAAATTAATACTTTGCTGT

TTTGACGTTACCGACAGAATAAGCACCGGCTAACTCTGTG

>3944 len=160 tot-seq=2

TGGGGAATATTGCGCAATGGGGGAAACCCTGACGCAGCGACGCCGCGTGCGGGATGAAGG

CCTTCGGGTTGTAAAGCGCTTTTGTCCGGAAAGAAATCCTTGGCTCTAATACAGTCGGGG

GATGACGGTACCGGAAGAATAAGCACCGGCTAACTACGTG

>3945 len=160 tot-seq=2

TAGGGAATCTTCCACAATGGACGCAAGTCTGATGGAGCAACGCCGCGTGAGTGAAGAAGG

CCTTCGGGTTGTAAAGTTCTTTCGGTAATGAGGAAGGGATGTTGTTAAATAGATAGCATC

ATTGACGTTAATTACAGAAGAAGCACCGGCTAACTCCGTG

>3946 len=160 tot-seq=2

TGGGGAATTTTGGACAATGGACGCAAGTCTGATCCAGCCATTCCGCGTGCAGGATGAAGG

CCCTCGGGTTGTAAACTGCTTTTGTACGGAACGAAAAGGTTTCCATTAATACTGGGAGCT

CATGACGGTACCGTAAGAATAAGCACCGGCTAACTACGTG

>3947 len=160 tot-seq=2

TAGGGAATCTTGCACAATGGGCGCAAGCCTGATGCAGCCATGCCGCGTGTATGAAGAAGG

CCTTCGGGTTGTAAAGTACTTTCAGCGGGGAGGAAGGCGGTGCGGTTAATAACCGCGCCG

ATTGACGTTACCCGCAGAAGAAGCACCGGCTAACTCCGTG

>3948 len=160 tot-seq=2

TAGGGAATCTTCCGCAATGGACGCAAGTCTGACGGAGCAACGCCGCGTGTGTGAAGAAGG

TCTTCGGATCGTAAAGCACTGTTATTAGAGAAGAACACCCTGTAGAGTAACTGTTACAGG

ATTGACGGTATCTAACCAGAAAGTGACGGCTAACTACGTG

>3949 len=160 tot-seq=2

CTAAGAATCTTCCGCAATGGACGCAAGTCTGACGGAGCGACACCGCGTGAATGAAGAAGG

CCGAAAGGTTGTAAAATTCTTTTAAGTCTGAAGAATAAGCAGTGTAGGGAATGATACTGT

GATGACGGTAAGGCTTGAATAAGCCCCGGCTAATTACGTG

>3950 len=160 tot-seq=2

TAGGGAATCTTCCGCAATGGACGAAAGTCTGACGGAGCAACGCCGCGTGAGTGAAGAAGG

ATTTCGGTTCGTAAAACTCTGTTGTTAGGGAAGAAAAAATTGTAGAGTAACTGCTACAGT

CTTGACGGTATCTTACCAGAAAGCCACGGCTAACTACATG

>3951 len=160 tot-seq=2

TGGGGAATCTTGCACAATGGGCGAAAGTCTGATGGAGCAACGCCGCGTGAGTGAAGAAGG

GTTCCGGCTCGTAAAGCTCTGTTGGTAGTGAAGAAAGATAGAGGTAGTAACTGGCCTTTA

TTTGACGGTAATCAACCAGAAAGTCACGGCTAACTACGTG

>3952 len=160 tot-seq=2

TAGGGAATCTTCCGCAATGGGCGCAAGCCTGACGGAGCAACGCCGCGTGAGTGATGAAGG

TTTTCGGATCGTAAAGCTCTGTTGCCAAGGAAGAACGCCTGGGAGAGTAACTGCTCCTAG

GGTGACGGTACTTGAGAAGAAAGCCCCGGCTAACTACGTG

>3953 len=160 tot-seq=2

TAGGGAATCTTCCACAATGGGCGCAAGCCTGATGGAGCAACACCGCGTGAGTGAAGAAGG

GTTTCGGCTCGTAAAACTCTGTTGTTGAAGAAGAACGTGCGTGAGAGTAACTGGCCTTTA

TTTGACGGTAATTACTTAGAAAGTCACGGCTAACTACGTG

>3954 len=160 tot-seq=2

TGGGGAATTTTGGACAATGGGCGCAAGCCTGATCCAGCCATGCCGCGTGTCTGGAGAAGG

CCTTCGAGTTGTAAAGGACTTTTGTCAGGGAAGAAAAGGCTGTCGCTAATACCGACAGTT

GATGACGGTACCTGAAGAATAAGCACCGGCTAACTACGTG

>3955 len=160 tot-seq=2

TGGGGAATATTGGACAATGGGGGAAACCCTGATCCAGCCATGCCGCGTGTGTGAAGAAGG

TCTTAGGATTGTAAAGCACTTTAAGTTGGGAGGAAGGACAGTTAACTAATATTTGACTGT

TTTGACGTTACCGACAGAATAAGCACCGGCTAACTTCGTG

>3956 len=160 tot-seq=2

TGGGGAATTTTGCACAATGGGGGAAACCCTGATGCAGCGACGCCGCGTGATTTAGAAGGC

CTTCGGGTTGTAAAGCTCTGTTGTAAGAGAAGAACGAGTGTGAGAGTGGAAAGTTCACAC

TGTGACGGTATCTTACCAGAAAGGGACGGCTAACTACGTG

>3957 len=160 tot-seq=2

TAGGGAATCTTCCACAATGGACGCAAGTCTGATGGAGCAACGCCGCGTGAGTGAAGAAGG

GTTTCGGTTCTTAAAACTCTGTTGTTAGGGAAGAAAAAATGTATAGTAACTATATACAAA

AGAGACGGTACCTAACCAGAAAGCCACGGCTAACTACGTG

>3958 len=160 tot-seq=2

TGGGGAATTTTGGACAATGGGCGCAAGCCTGATCCAGCAATGCCGCGTGCGGGAAGAAGG

GTTTCGGCTCGTAAAGCTCTGTTGTTGGTGAAGAAGGACAGGGGTAGTAACTGACCTTTG

TTTGACGGTAATCAATTAGAAAGTCACGGCTAACTACGTG

>3959 len=160 tot-seq=2

TGGGGAATATTGCACAATGGGGGAAACCCTGATGCAGCAACGCCGCGTGAGTGATGAAGG

TTTTCGGGTTGTAAAGCACTTTCAGCGGGGAGGAAGGCGATAAGGTTAATAACCTTGTCG

ATTGACGTTACCCGCAGAAGAAGCACCGGCTAACTCCGTG

>3960 len=160 tot-seq=2

TAGGGAATTTTTCACAATGGGCGCAAGCCTTATGAAGCAATGCCGCGTGTATGAAGAAGG

CCTTCGGGTTGTAAAGTACTTTCAGCGGGGAGGAAGGGAGTAAAGTTAATACCTTTGCTC

ATTGACGTTACCCGCAGAAGAAGCACCGGCTAACTCCGTG

>3961 len=160 tot-seq=2

TGGGGAATATTGCACAATGGGCGCAAGCCTGATGCAGCCATGCCGCGTGTATGAAGAAGG

CCTTCGGGTTGTAAAGTGCTGTTATAAGGGAAGAACATTTGCAATAGGAAATGATTGCAG

ACTGACGGTACCTTGTCAGAAAGCGATGGCTAACTATGTG

>3962 len=160 tot-seq=2

TAGGGAATCTTCCACAATGGGCGCAAGCCTGATGGAGCAACACCGCGTGAGTGAAGAAGG

GTTTCGGCTCGTAAAGCTCTGTTGTTGGAGAAGAACGTGCGTGAGAGTAACTGTTCACGC

AGTGACGGTAATTACTTAGAAAGTCACGGCTAACTACGTG

>3963 len=160 tot-seq=2

TAGGGAATTTTCGGCAATGGGGGGAACCCTGACCGAGCAACGCCGCGTGAAGGAAGAAGG

TTTTCGGATTGTAAACTTCTGTTATAAAGGAAGAACGACTGTAGGAGGGAATGCCTATAG

AGTGACGGTACTTTATGAGAAAGCCACGGCTAACTACGTG

>3964 len=160 tot-seq=2

TAGGGAATCTTCGGCAATGGGGGAAAGCCTGATCCAGCCATGCCGCGTGTGTGAAGAAGG

TCTTCGGATTGTAAAGCACTTTAAGTTGGGAGGAAGGGCAGTTACCTAATACGTGATTGT

TTTGACGTTACCGACAGAATAAGCACCGGCTAACTCTGTG

>3965 len=160 tot-seq=2

TGGGGAATATTGCGCAATGGGGGAAACCCTGACGCAGCGACGCCGCGTGCGGGATGAAGG

GTTTCGGCTCGTAAAACTCTGTTGTTGGAGAAGAACGTGCGTAAGAGTAACTGTTTACGC

AGTGACGGTATCCAACCAGAAGGTCACGGCTAACTACGTG

>3966 len=160 tot-seq=2

TAGGGAATTTTCGTCAATGGGGGGAACCCTGAACGAGCAATGCCGCGTGAGTGAAGAAGG

TCTTCGGATCGTAAAGCTCTGTTGTAAGAGAAAAACGGGTATCATAGGGAATGATGATAT

CGTGATGGTATCTTACCAGAAAGCCACGGCTAACTACGTG

>3967 len=160 tot-seq=2

TGGGGAATATTGGACAATGGAGGGAACTCTGATCCAGCAATTCTGCGTGTGTGAAGAAGG

TCTTCGGATTGTAAAGCACTTTAAGTTGGGAGGAAGGGCAGTTACCTAATACGTGATTGT

TTTGACGTTACCGACAGAATAAGCACCGGCTAACTCTGTG

>3968 len=160 tot-seq=2

TAGGGAATCTTCGGCAATGGGGGGAACCCTGACCGAGCAACGCCGCGTGCGGGATGAAGG

CCTTCGGGTTGTAAACCGCTTTTGTCAGAGACGAAAAGGGACGTACGAATAATACGTTCC

GCTGACGGTATCTGAAGAATAAGCACCGGCTAACTACGTG

>3969 len=160 tot-seq=2

TAGGGAATCTTCGGCAATGGGGGGAACCCTGACCGAGCAACGCCGCGTGAGTGAAGAAGG

TTTTCGGATCGTAAAGCTCTGTTATTAGGGAAGAACAAATGTGTAAGTAACTATGCACGT

CTTGACGGTACCTAATCAGAAAGCCACGGCTAACTACGTG

>3970 len=160 tot-seq=2

TGGGGAATTTTGCACAATGGGGGAAACCCTGATGCAGCGACGCCGCGTGATTTAGAAGGC

CTTCGGGTTGTAAAGCTCTGTTAATCGGGACGAATGGTCGGTGTGCAAATAGTGCATGGA

CATGACGGTACCGGAATAGAAAGCCACGGCTAACTACGTG

>3971 len=160 tot-seq=2

TGGGGGATATTGGACAATGGGGGAAACCCTGATCCAGCGACGCCGCGTGAATGAAGAAGG

CCTTAGGGTTGTAAAGTTCTTTCGGTATTGAGGAAGGTTGATGTGTTAATAGCACATCAA

ATTGACGTTAAATACAGAAGAAGCACCGGCTAACTCCGTG

>3972 len=160 tot-seq=2

TGGGGAATATTGCACAATGGGCGAAAGCCTGATGCAGCGACGCCGCGTGAGCGAAGAAGT

ATTTCGGTATGTAAAACTCTGTCCTCATCCAAGAGAAGGGAAGGTAGTAACTGACCTTTC

CAGGACGGTAGATGAGGAGGAAGCCCCGGCTAACTATGTG

>3973 len=160 tot-seq=2

TGGGGAATATTGCGCAATGGGCGAAAGCCTGACGCAGCGACGCCGCGTGAGGGATGAAGG

TTTTCGGATCGTAAACCTCTGTCAGAAGGGAAGAAGTTGCATGGGGCCAATCACCTGTGC

ATTGACGGTACCTTCAAAGGAAGCACCGGCTAACTCCGTG

>3974 len=160 tot-seq=2

CAGGGAATCTTCCGCAATGGACGAAAGTCTGACGGAGCAACGCCGCGTGAGTGATGAAGG

TTTTCGGATCGTAAAACTCTGTTATTAGGGAAGAACAAATGTGTAAGTAACTGTGCACAT

CTTGACGGTACCTAATCAGAAAGCCGCGGCTAACTACGTG

>3975 len=160 tot-seq=2

TGGGGAATATTGCACAATGGGGGAAACCCTGATGCAGCAACGCCGCGTGAGTGATGAAGG

TTTTCGGATCGTAAAGCTCTGTTGTTAGGGAAGAACGTGCGTGAGAGCAACTGTTCACGC

AGTGACGGTATCTAACCAGAAAGTCACGGCTAACTACGTG

>3976 len=160 tot-seq=2

TGGGGAATTTTGGACAATGGGCGAAAGCCTGATCCAGCAATGCCGCGTGAGTGATGAAGG

TCTTCGGATCGTAAAACTCTGTTATTAGGGAGGAACATATGTGTAAGTAACTGTGCACAT

CTTGACGGTACCTAATCAGAAAGCCACGGCTAACTACGTG

>3977 len=160 tot-seq=2

TGGGGAATATTGGACAATGGGCGCAAGCCTGATCCAGCCATACCGCGTGGGTGAAGAAGG

TCTTCGGATCGTAAAACTCTGTTATTAGGGAAGAACATATGTGTAAGTAACTGTGCACAT

CTTGACGGTACCTAATCAGAAAGCCACGGCTAACTACGTG

>3978 len=160 tot-seq=2

TAGGGAATCTTCGGCAATGGACGGAAGTCTGACCGAGCAACGCCGCGTGAGTGATGAAGG

TCTTCGGATCGTAAAACTCTGTTATTAGGGAAGAACATATGTGTAAGTAACTGTGTACAT

CTTGACAGTACCTAATCAGAAAGCCACGGCTAACTACGTG

>3979 len=160 tot-seq=2

TAGGGAATCTTCCGCAATGGGCGAAAGCCTGACGGAGCAACGCCGCGTGAGTGATGAAGG

TCTTCGGATCGTAAAACTCTGTTGGTAGTGAAGAAAGATAGAGGTAGTAACTGGCCTTTA

TTTGACGGTAATTACTTAGAAAGTCACGGCTAACTACGTG

>3980 len=160 tot-seq=2

TGAGGAATTTTTCACAATGGACGAAAGTCTGATGGAGCAATACCACGTGAGTGATGAAGG

TCTTCGGATCGTAAAACTCTGTTATTAGGGAAGAACATATGTGTAAGTAACTGTGCACAT

CTTGACGGTACCTAATCAGAAAGCCACGGCTAACTACGTG

>3981 len=160 tot-seq=2

TGGGGAATCTTCCGCAATGGACGGAAGCCTGACCGAGCAACGCCGCGTCAGTGAAGAAGG

GTTTCGGCTCGTAAAGCTCTGTTGTTGGTGAAGAAGGACAGGGGTAGTAACTGACCTTTG

TTTGACGGTAATCAATTAGAAAGTCACGGCTAACTACGTG

>3982 len=160 tot-seq=2

TAGGGAATTTTCGGCAATGGGGGGAACCCTGACCGAGCAACGCCGCGTGAGGGAGGAAGT

ATTTCGGTATGTAAACCTCTGTTATAAAGGAAGAACGGCATATGCAGGGAATGGCATATG

AGTGACGGTACTTTATGAGGAAGCCACGGCTAACTACGTG

>3983 len=160 tot-seq=2

TGGGGAATATTGGACAATGGGCGAAAGCCTGATCCAGCCATGCCGCGTGTGTGAAGAAGG

TCTTCGGATTGTAAAGCACTTTAAGTTGGGAGGAAGGGGTTGAAGCTAATACCTTCAATC

TTTGACGTTACCAACAGAATAAGCACCGGCTAACTCTGTG

>3984 len=160 tot-seq=2

TAGGGAATCTTCCGCAATGGACGCAAGTCTGACGGAGCAACGCCGCGTGAGTGAAGAAGG

TCTTCGGATCGTAAAGCTCTGTTGTTAGAGAAGAACAGCGCATAGAGTAACTGTTATGCG

TGTGACGGTATCCAACCAGAAAGCCACGGCTAACTACGTG

>3985 len=160 tot-seq=2

TGGGGAATATTGCGCAATGGGGGAAACCCTGATGGAGCAACGCCGCGTGAATGAAGAAGG

CCTTCGGGTCGTAAAATTCTGTTGTCAGAGAAGAACGTGCGTGAGAGTAACTGTTCACGT

ATTGACGGTATCTGACCAGAAAGCCACGGCTAACTACGTG

>3986 len=160 tot-seq=2

TGGGGAATATTGCGCAATGGGGGAAACCCTGAACGAGCAATGCCGCGTGAGTGAAGAAGG

TCTTCGGATCGTAAAGCTCTGTTGTAAGAGAAAAACGACATTCATAGGGAATGATGAGTG

AGTGATGGTATCTTACCAGAAAGTCACGGCTAACTACGTG

>3987 len=160 tot-seq=2

TTAAGAATATTTCGCAATGGACGAAAGTCTGACGGAGCGACGCCGCGTGGATGAAGAATG

CCGAAAGGTTGTAAAATCCTTTTAAGCCTGAGGAATAAGCACAGTAGGGAATGATTGTGC

GGTGACAGTAGGGCTTGAATAAGCACCGGCTAATTACGTG

>3988 len=160 tot-seq=2

TAGGGAATCTTCCACAATGGACGCAAGTCTGATGGAGCAACGCCGCGTGAGTGAGGAAGG

CCTTCGGGTCGTAAAGCTCTGTTGCCAGGGAAGAACGCCAAGGAGAGTAACTGCTCTTTG

GGTGACGGTACCTGAGAAGAAAGCCCCGGCTAACTACGTG

>3989 len=160 tot-seq=2

TGGGGAATCTTCCGCAATGGACGAAAGTCTGACGGGGCAACGCCGCGTGAGTGAAGACGG
CCTTCGGGTTGTAAAGCTCTGTGATTCGGGACGAAAAGCCGTATGTAAATAATATATGGA
AATGACGGTACCGAAAAAGCAAGCCACGGCTAACTACGTG
>3990 len=160 tot-seq=2
TGGGGAATATTGCGCAATGGGGGAAACCCTGACGCAGCGACGCCGCGTGCGGGATGAAGG
CCTTCGGGTTGTAAAGCACTTTAAGTTGGGAGGAAGGGCAGTAAATTAATACTTTGCTGT
TTTGACGTTACCGACAGAATAAGCACCGGCTAACTCTGTG
>3991 len=160 tot-seq=2
GACCAGCGTATCCCATTGTTCTGACCTGCATTTCAAGTGCAACTTCCAACTAGCACGGTC
CAATTTTGTCAACGGTAAGACGTCCTCCTCCACACACGCAGGTGAACAGAGTATGGGCTC
CAAAAAGCAATTCCATTTACTGGAAGAACCAGATCATGAT
>3992 len=160 tot-seq=2
TGAGGAATATTGGTCAATGGGCGAGAGCCTGAACCAGCCAAGTAGCGTGCAGGATGAAGG
TCTTCGGATTGTAAACTGCTTTTGTCAGGGAAGAAAAGGTCTGTGTTAATACCATAGACT
GCTGACGGTACCTGAAGAATAAGCACCGGCTAACTACGTG
>3993 len=160 tot-seq=2
TGGGGAATCTTGCACAATGGGCGAAAGCCTGATGCAGCGACGCCGCGTGCGGGATGAAGG
GTTTCGGCTCGTAAAGCTCTGTTGGTAGTGAAGAAAGATAGAGGTAGTAACTGGCCTTTA
TTTGACGGTAATTACTTAGAAAGTCACGGCTAACTACGTG
>3994 len=160 tot-seq=2
TAGGGAATCTTCCACAATGGGCGCAAGCCTGATCCAGCCATGCCGCGTGTGTGAAGAAGG
TCTTCGGATTGTAAAGCACTTTAAGTTGGGAGGAAGGGTTGTAGATTAATACTCTGCAAT
TTTGACGTTACCGACAGAATAAGCACCGGCTAACTCTGTG
>3995 len=160 tot-seq=2
TGGGGAATATTGCACAATGGACGCAAGTCTGATGGAGCAACGCCGCGTGAGTGAAGAAGG
GTTTCGGCTCGTAAAGCTCTGTTGTTAAAGAAGAACGTGGGTGAGAGTAACTGTTCACCC
AGTGACGGTATTTAACCAGAAAGCCACGGCTAACTACGTG
>3996 len=160 tot-seq=2
TGAGGAATATTGGTCAATGGGCGAGAGCCTGAACCAGCCAAGTAGCGTGCACGATGAAGG

TCTTCGGATTGTAAAGTGCTGTTATAAGGGAAGAACATTTGCAATAGGAAATGATTGCAG

ACTGACGATACCTTGTCAGAAAGCGATGGCTAACTATGTG

>3997 len=160 tot-seq=2

TGGGGAATTTTGGACAATGGGCGCAAGCCTGATCCAGCCATGCCGCGTGCGAGAAGAAGG

CCTTCGGGTTGTAAACCGCTTTTGTCAGGGAAGAAAAGTTCCGGGCTAATACCCTGGGAC

GATAACGGTACCTGAAGAATAAGCACCGGCTAACTACGTG

>3998 len=160 tot-seq=2

TGGGGAATATTGCACAATGGGCGCAAGCCTGATGCAGCCATGCCGCGTGTATGAAGAAGG

CCCTAGGGTTGTAAAGTACTTTCAGTCGGGAGGAAGGCGCTGATGCTAATATCATCAGCG

ATTGACGTTACCGACAGAAGAAGCACCGGCTAACTCCGTG

>3999 len=160 tot-seq=2

TGGGGAATATTGGACAATGGGCGCAAGCCTGATCCAGCCATGCCGCGTGAGTGATGAAGG

CCTTAGGGTTGTAAAGCTCTGTTGTTGGTGAAGAAGGATAGAGGTAGTAACTGGCCTTTA

TTTGACGGTAATCAACCAGAAAGTCACGGCTAACTACGTG

>4000 len=160 tot-seq=2

TAGGGAATCTTCGGCAATGGGGGGAACCCTGACCGAGCAACGCCGCGTGAGTGAAGAAGG

GTTTCGGCTCGTAAAGCTCTGTTGTTGGTGAAGAAGGACAGGGGTAGTAACTGACCTTTG

TTTGACGGTAATCAATTAGAAAGTCACGGCTAACTACGTG

>4001 len=160 tot-seq=2

CTCCTTAATTGTCCAAGGGATGTGGGCTCCTGCTCCACCCTGGGGAATAAGAGGCATTCT

TCGTGAGCACTGAATCCTCAGTGATCCTCAGAGCCTGGACACTCCAGCTTAGGGCTCTCT

GCCTGAGTCCCCTCCAGGCTGGATGCAGACATGAACACGC

>4002 len=160 tot-seq=2

TGGGGAATATTGCGCAATGGGGGAAACCCTGACGCAGCGACGCCGCGTGCGGGATGAAGG

CCTTCGGGTTGTAAAGCACTTTTGTCCGGAAAGAAATCGCGCTGGTTAATACCTGGCGTG

GATGACGGTACCGGAAGAATAAGCACCGGCTAACTACGTG

>4003 len=160 tot-seq=2

TGGGGAATATTGCACAATGGACGGAAGTTTGATGCAGCGACGCCGCGTGTATGAAGAAGG

CCTTCGGGTTGTAAAGTACTTTCAGCGGGGAGGAAGGGAGTAAAGTTAATACCTTTGCTC

ATTGACGTTACCCGCAGAAGAAGCACCGGCTAACTCCGTG

>4004 len=160 tot-seq=2

TGGGGAATATTGCACAATGGGGGAAACCCTGATGCAGCGACGCCGCGTGAGCGATGAAGG

CTTTCGGATCGTAAAACTCTGTTGTTAGAGAAGAACAAGGACGTTAGTAACTGAACGTCC

CCTGACGGTATCTAACCAGAAAGCCACGGCTAACTACGTG

>4005 len=160 tot-seq=2

TAGAGAATTTTCGGCAATGGGCGAAAGCCTGACCGAGCAACGCCGCGTGAGGGAGGAAGT

CCTTCGGGATGTAAACCTCTGTTATAAAGGAAGAACGGTAACTGCAGGGAATGGCAGTTA

AGTGACGGTACTTTATGAGGAAGCCACGGCTAACTACGTG

>4006 len=160 tot-seq=2

TAGGGAATCTTCCACAATGGACGAAAGTCTGATGGAGCAACGCCGCGCGAGCGATGAAGG

CCTTCGGGTCGTAAAGCTCTGTTGTTAGGGAAGAACAAGTATCGGAGTAACTGCCGGTAC

CTTGACGGTACCTAACCAGAAAGCCACGGCTAACTACGTG

>4007 len=160 tot-seq=2

TGGGGAATATTGCACAATGGGCGCAAGCCTGATGCAGCCATGCCGCGTGTATGAAGAAGG

CCTTCGGGTTGTAAAGTACTTACAGCGGGGAGGAAGGGATGGTGCTTAATACGCGCCGTC

ATTGACGTTACCCGCAGAAGAAGCACCGGCTAACTCCGTG

>4008 len=160 tot-seq=2

TGGGGAATTTTGGACAATGGGCGCAAGCCTGATCCAGCCATTCCGCGTGAGTGAAGAAGG

CCTTCGGGTTGTAAAGCTCTTTCGCAAGGGAAGAAAACTTACTTTCTAATAAAGAGTGAG

GCTGACGGTACCTTGATAAGAAGCACCGGCTAACTACGTG

>4009 len=160 tot-seq=2

TGGGGAATATTGCGCAATGGGGGAAACCCTGACGCAGCGACGCCGCGTGCGGGATGAAGG

CCTTCGGGTTGTAAAGTTCTTTCGGTAATGAGGAAGGGATGTTGTTAAATAGATAGCATC

ATTGACGTTAATTACAGAAGAAGCACCGGCTAACTCCGTG

>4010 len=160 tot-seq=2

GGGCTGTTCCTGTGGCCCCGTATTCCCATCACTGCATGTCCTGAGGTGTCCTGAGGTCCA

GAGTGCTCCCTTCCTTCTGGAGCTCCCCCTGCCGGGCACCCTCCTTGTGGTGACTGTGGG

TCTGGATGGACCCTTGCTCCCCATCAGTCCTGTACAGCTC

>4011 len=160 tot-seq=2

TGGGGAATATTGCGCAATGGGGGAAACCCTGACGCAGCGACGCCGCGTGAGTGAAGAAGG

TTTTCGGATCGTAAAACTCTGTTGTTAGAGAAGAACAAGGATGAGAGTAACTGTTCATCC

CTTGACGGTATCTAACCAGAAAGCCACGGCTAACTACGTG

>4012 len=160 tot-seq=2

CAGCACAGCGACCACCTCCAACTCTCCCAGAGGAGGGTGTGGACGTAGACACCAGTGGGG

TCCCAGAACCTCAGGTGGGATGGTGAAGGAATGGGTGGGTCCCACTGAGGCCCAGAGAGG

GCGAGTGAGGACCCAGCATGGCCCAGCATGGCCCAGCTGG

>4013 len=160 tot-seq=2

TAGGGAATCTTCGGCAATGGGGGCAACCCTGACCGAGCAACGCCGCGTGAATGAAGAAGG

CCTTCGGGTTGTAAAGTTCTTTCGGTAGCGAGGAAGGCATTTAGTTTAATAGACTAGGTG

ATTGACGTTAACTACAGAAGAAGCACCGGCTAACTCCGTG

>4014 len=160 tot-seq=2

TAGGGAATCTTCGGCAATGGGGGCAACCCTGATCCAGCCATGCCGCGTGTCTGAAGAAGG

CCTTCGGGTTGTAAAGGACTTTTGTCAGGGAAGAAAAGGATAGGGTTAATACCCCTGTCT

GATGACGGTACCTGAAGAATAAGCACCGGCTAACTACGTG

>4015 len=160 tot-seq=2

TAGGGAATCTTCCACAATGGACGAAAGTCTGATCCAGCCATGCCGCGTGTGTGAAGAAGG

TCTTCGGATTGTAAAGCACTTTAAGTTGGGAGGAAGGGCAGTAAGTTAATACCTTGCTGT

TTTGACGTTACCAACAGAATAAGCACCGGCTAACTTCGTG

>4016 len=160 tot-seq=2

TGGGGAATTTTGGACAATGGGGGCAACCCTGATCCAGCCATGCCGCGTGCGGGAAGAAGG

CCTTCGGGTTGTAAACCGCTTTTGGTCGGGAAGAAAAGGTTATCCCTAATAAGGATGACT

CTTGACGGTACCGGCAGAATAAGCACCGGCTAACTACGTG

>4017 len=160 tot-seq=2

TGGGGAATTTTGCGCAATGGACGAAAGTCTGACGCAGCAACGCCGCGTGGGTGATGAAGG

TCTTCGGACTGTAAAACCCTGTCGTCAGGGACGAAGGTGGCGGAGTTAATAACTCCGTTG

CTTGACGGTACCTGGAGAGGAAGCCCCGGCTAACTCTGTG

>4018 len=160 tot-seq=2

TAGGGAATCTTCGGCAATGGGGGCAACCCTGACCGAGCAACGCCGCGTGAGTGAAGAAGG

TCTTCGGATTGTAAAGCACTTTAAGTTGGGAGGAAGGGTTGTAGATTAATACTCTGCAAT

TTTGACGTTACCGACAGAATAAGCACCGGCTAACTCTGTG

>4019 len=160 tot-seq=2

TAGGGAATATTGGACAATGGGCGAAAGCCTGATCCAGCAACGCCGCGTGGGTGACGAAGG

CCTTCGGGTCGTAAAGCCCTGTGGGGAGGAGCGAATGGCTCCGGCCTAACACGTCGGGGA

GTTGACGGTACCTCCTTAGCAAGCACCGGCTAACCACGTG

>4020 len=160 tot-seq=2

TAGGGAATATTGGACAATGGGCGCAAGCCTGATCCAGCCATGCCGCGTGAGTGAAGAAGG

CCTTCGGGTTGTAAAGCTCTTTTGTCCGGAAAGAAAAGCCTTGGGTTAATACCCTGAGGT

TCTGACGGTACCGGAAGAATAAGCACCGGCTAACTTCGTG

>4021 len=160 tot-seq=2

TGAGGAATATTGGTCAATGGACGAGAGTCTGAACCAGCCAAGTAGCGTGCAGGATGACGG

CCTTCGGGTTGTAAAGCTCTGTGATTCGGGACGAAAGGCCATATGTGAATAATATATGGA

AATGACGGTACCGAAAAAGCAAGCCACGGCTAACTACGTG

>4022 len=161 tot-seq=2

TAGGGAATATTGCGCAATGGACGAAAGTCTGACGCAGCCACGCCGCGTGAGTGAAGAAGG

CCTTCGGGTTGTAAAGCTCTGTCAACTGGGAAAAACGATTATCCGGGGAAAGTTCGGGTA

ATGTGATGGTACCAGTGGAGGAAGCACCGGCAAACTCTGTG

>4023 len=161 tot-seq=2

TCGAGAATCATTCACAATGGGGGCAACCCTGATGGTGCGACGCCGCGTGGGGGAATGAAG

GTCTTCGGATTGTAAAACTCTGTTATTAGGGAAGAACAAATGTGTAAGTAACTATGCACG

TCTTGACGGTACCTAATCAGAAAGCCACGGCTAACTACGTG

>4024 len=161 tot-seq=2

TGGGGAATTTTGGACAATGGGCGCAAGCCTGATCCAGCAATGCCGCGTGTGTGAAGAAGG

TTTTCGGATCGTAAAGCTCTGTTGTAAGTCAAGAACGAGTGTGAGAGTGGAAAGTTCACA

CTGTGACGGTAGCTTACCAGAAAGGGACGGCTAACTACGTG

>4025 len=161 tot-seq=2

TGGGGAATATTGGACAATGGGGGGAACCCTGATCCAGCCATGCCGCGTGTGTGAAGAAGG

CCTTATGGTTGTAAAGCACTTTAAGCGAGGAGGAGGCTCCCGTAGCTAATATCTACGGAG

AGTGGACGTTACTCGCAGAATAAGCACCGGCTAACTCTGTG

>4026 len=161 tot-seq=2

TAGGGAATCTTCGGCAATGGACGGAAGTCTGACCGAGCAACGCCGCGTGAGTGAAGAAGG

TTTTCGGATCGTAAAGCTCTGTTGTAAGAGAAGAACGAGTGTGAGAGTGGAAAGTTCACA

CTGTGACGGTAATCAATTAGAAAGTCACGGCTAATTACGTG

>4027 len=161 tot-seq=2

TGGGGAATCTTCCGCAATGGGCGAAAGCCTGACGGAGCAACGCCGCGTGAGTGAAGACGG

CCTTCGGGTTGTAAAACTCTGTTATACGGGACGAAAAAGGCGGATGCCAACAGTATCCGT

CCGTGACGGTACCGTAAGAGAAAGCCACGGCTAACTACGTG

>4028 len=161 tot-seq=2

TGGGGAATCTTCCGCAATGGGCGAAAGCCTGACGGAGCAATGCCGCGTGAGTGAGGAAGG

CCTTCGGGTTGTAAAACTCTGTTGTTAGAGAAGAACGTTGGTAGGAGTGGAAAATCTACC

AAGTGACGGTAACTAACCAGAAAGGGACGGCTAACTACGTG

>4029 len=161 tot-seq=2

TGGGGAATATTGCACAATGGGGGAAACCCTGATGCAGCGACGCCGCGTGAGCGAAGAAGT

ATTTCGGTATGTAAAGCTCTGTTGTTAGAGAAGAACGTTGGTAGGAGTGGAAAATCTACC

AAGTGACGGTAACTAACCAGAAAGGGACGGCTAACTACGTG

>4030 len=161 tot-seq=2

TGGGGAATCTTGCGCAATGGGGGGAACCCTGACGCAGCGACGCCGCGTGCGGGACGGAGG

CCTTCGGGTCGTAAAGCTCTGTTGTTAGAGAAGAACGTTGGTAGGAGTGGAAAATCTACC

AAGTGACGGTAACTAACCAGAAAGGGACGGCTAACTACGTG

>4031 len=161 tot-seq=2

TAGGGAATCTTCCACAATGGGCGAAAGCCTGATGGAGCAACGCCGCGTGGGTGAAGAAGG

TCTTCGGATCGTAAAACCCTGTTGTTAGAGAAGAACGAGTGTGAGAATGGAAAGTTCATG

CTGTGACGGTACTTAACCAGAAAGGGACGGCTAACTACGTG

>4032 len=161 tot-seq=2

TAGGGAATATTGCACAATGGAGGAAACTCTGATGCAGCCATGCCGCGTGTGTGAAGAAGG

CCTTCGGGTTGTAAAGCTCTGTGATCCGGGACGAAAGAGCCTTGGGTAAATAGCCTAAGG

AAGTGACGGTACCGGAAAAGCAAGCCACGGCTAACTACGTG

>4033 len=161 tot-seq=2

TAGGGAATCTTCCGCAATGGACGCAAGTCTGACGGAGCAACGCCGCGTGAGTGAAGACGG

CCTTCGGGTTGTAAAGCTCTGTGATTCGGGACGAATGGTCGGTGTGCAAATAGTGCATGG

ACATGACGGTACCGGAATAGAAAGCCACGGCTAACTACGTG

>4034 len=161 tot-seq=2

TGGGGAATCTTCCGCGATGGACGAAAGTCTGACGGAGCAACGCCGCGTGAGTGATGACGG

TCTTCGGATTGTAAAGCTCTGTTAATCGGGACGAATGGTCGGTGTGCAAATAGTGCATGG

ACATGACGGTATCTTACCAGAAAGCCACGGCTAACTACGTG

>4035 len=161 tot-seq=2

TGGGGAATATTGCACAATGGGCGCAAGCCTGATGCAGCCATGCTGCGTGAGTGAAGAAGG

TTTTCGGATCGTAAAGCTCTGTTGTAAGAGAAGAACGTGCGTGAGAGTGGAAAGTTCACA

CAGTGACGGTAACTTACCAGAAAGGGACGGCTAACTACGTG

>4036 len=161 tot-seq=2

TTAGGAATATTCGTCAATGGGGGAAACCCTGAACGAGCAATGCCGCGTGAACGATGACGG

TCCTCTGGATTGTAAAGTTCTGTTGTTTGGAAAGAATAGCTTCGATAGGAAATGATCGAA

GTTTGACGGTACCATTCAAGAAAGCCACGGCTAACTACGTG

>4037 len=161 tot-seq=2

TAGGGAATCTTCCACAATGGACGCAAGTCTGACCGAGCAACGCCGCGTGAGTGAAGAAGG

TTTTCGGATCGTAAAGCTCTGTTGTAAGAGAAGAACAGGTGTGAGAGTGGAAAGTTCACA

CTGTGACGGTATCTTACCAGAAAGGGACGGCTAACTACGTG

>4038 len=161 tot-seq=2

TAGGGAATTTTCCGCAATGGGCGAAAGCCTGACGGAGCAACGCCGCGTGAGTGAGGACGG

CCTTCGGGTTGTAAAACTCTGTTCTTGAGGAAGAATTCCTTCCAGGCGAACAGCCTGGAA

GGTTGACGGTACTCAAGGAGAAAGCCCCGGCTAACTACGTG

>4039 len=161 tot-seq=2

TGGGGAATTTTGCGCAATGGGCGAAAGCCTGACGGAGCAACGCCGCGTGAGTGATGACGG

CCTTCGGGTTGTAAAGCTCTGTGATCGGGGACGAACGGTCCGTAAGCTAATACCTTATGG

AAGTGACGGTACCCGAATAGCAAGCCACGGCTAACTACGTG

>4040 len=161 tot-seq=2

TTAGGGATATTCGTCAATGGGGGAAACCTTGAACGAGCAATGCCGCGTGAGTGATGACAG

TTCTTTGGATTGTAAAACTCTGTTGTTAGGGAAGAACGACTCGTGTAGGAAATGACACGG

GAGTGACTGTACCTTTCAAGAAGGCTCCGGCTAACTACGTG

>4041 len=161 tot-seq=2

TGGGGAATTGTTCGCAATGGGCGCAAGCCTGACGACGCAACGCCGCGTGGAGGATGAAGA

CCTTCGGGTCGTAAACTCCTTTCGATCGAGACGAATTCCTTCGGGGTGAACAATCCCGGA

GGTTGACGGTACCGAGAGAAGAAGCCCCGGCTAACTCCGTG

>4042 len=161 tot-seq=2

TGAGGAATATTGGTCAATGGGCGAGAGCCTGAACCAGCCAAGTAGCGTGCAGGATGACGG

CCCTATGGGTTGTAAACTGCTTTTATATGGGAAGAAACGCTAAAATAGGAAATGATTTTA

GTTTGACTGTACCATTTGAATAAGTATCGGCTAACTATGTG

>4043 len=161 tot-seq=2

TGGGGAATATTGCACAATGGGGGAAACCCTGATGCAGCGACGCCGCGTGAACGAAGAAGG

TATTCGTATCGTAAAGTTCTGTCGTAAGAGAAGAACGAGTGTGAGAGTGGAAAGTTCACA

CTGTGACGGTATCTTACCAGAAAGGGACGGCTAACTACGTG

>4044 len=161 tot-seq=2

TGGGGAATCTTGCACAATGGGCGAAAGCCTGATGCAGCGACGCCGCGTGCGGGATGAAGG

TTTTCGGATCGTAAAGCTCTGTTGTAAGAGAAGAACGAGTGTGAGAGTGGAAAGTTCACA

CTGTGACGGTATCTTACCAGAAAGGGACGGCTAACTACGTG

>4045 len=161 tot-seq=2

TGGGGAATATTGGACAATGGAGGCAACTCTGATCCAGCAATTCTGTGTGTGTGAAGAAGG

TTTTAGGACTGTAAAGCTCTGTTGTTAGAGAAGAACGTTGGTAGGAGTGGAAAATCTACC

AAGTGACGGTAACTAACCAGAAAGGGACGGCTAACTACGTG

>4046 len=161 tot-seq=2

TTAGGAATATTCGGCAATGGGGGAAACCCTGACCGAGCAATGCCGCGTGTGAGATGAAGG

TCCTCTGGATTGTAAATCACTTTTATTTGGGAAGAATTGTAAGTAGAGGAAATGTTACTT

ACTTGACGGTACCTTTTGAATAAGCCCCGGCTAACTACGTG

>4047 len=161 tot-seq=2

TCAAGAATCATTCACAATGGGGGAAACCCTGATGGTGCGACGCCGCGTGAGTGAAGAAGG

TTTTCGGATCGTAAAGCTCTGTTGTAAGTCAAGAACGAGTGTGAGAGTGGAAAGTTCACA

CTGTGACGGTAGCTTACCAGAAAGGGACGGCTAACTACGTG

>4048 len=161 tot-seq=2

TAGGGAATCTTCGGCAATGGGGGGAACCCTGACGGAGCAACGCCGCGTGAGTGAAGACGG

CCTTCGGGTTGTAAAGCTCTGTGATCCGGGACGAAAGAGCCTGAGGTAAATAGCCTAAGG

AAGTGACGGTACCGGAAAAGCAAGCCACGGCTAACTACGTG

>4049 len=161 tot-seq=2

TGGGGAATTTTGGACAATGGGGGAAACCCTGATCCAGCCATGCCGCGTGCGGGATGAAGG

CCTTCGGATCGTAAAGCTCTGTTGTTAAGGAAGAACGAGTGTGAGAATGGAAAGTTCATG

CTGTGACGGTACTTAACCAGAAAGGGACGGCTAACTACGTG

>4050 len=161 tot-seq=2

TGGGGAATATTGCACAATGGGGGAAACCCTGATGCAGCGACGCCGCGTGAACGAAGAAGG

TATTCGTATCGTAAAGCTCTGTTGTTAAGGAAGAACGAGTGTGAGAATGGAAAGTTCATG

CTGTGACGGTACTTAACCAGAAAGGGACGGCTAACTACGTG

>4051 len=161 tot-seq=2

TGGGGAATCTTCCGCAATGGACGAAAGTCTGACGGAGCAACGCCGCGTGAACGAAGAAGG

TTTTCGGATCGTAAAGCTCTGTTGTTAGAGAAGAACGTTGGTAGGAGTGGAAAATCTACC

AAGTGACGGTAACTAACCAGAAAGGGACGGCTAACTACGTG

>4052 len=161 tot-seq=2

TGGGGAATATTGCACAATGGGCGCAAGCCTGATGCAGCAACGCCGCGTGAACGATGACGG

CCTTCGGGTTGTAAAGTTCTGTTATACGGGACGAATGGCGCAACGGTCAATACCCGTTGC

GAGTGACGGTACCGTAAGAGAAAGCCACGGCTAACTACGTG

>4053 len=161 tot-seq=2

TTAGGAATATTCGTCAATGGGGGAAACCCTGAACGAGCAATGCCGCGTGAGTGATGAAGG

CCCTATGGGTTGTAAAACTCTGTTGTAAAGGAAGAATACCTAGTATAGGAAATGATGCTA

GGATGACGGTACTTTACCAGAAAGCCCCGGCTAACTACGTG

>4054 len=161 tot-seq=2

TGGGGAATCTTCCGCAATGGGCGCAAGCCTGACGGAGCAACGCCGCGTGAGTGAAGAAGG

CCTTCGGGTCGTAAAGCTCTGTTGTCGGGGACGAATGTTACTGCTTAAAATATAGGTAGT
AAATGACGGTACCTGACGAGGAAGCCACGGCTAACTACGTG
>4055 len=161 tot-seq=2
TGGGGAATATTGCGCAATGGGGGAAACCCTGACGGAGCAACGCCGCGTGAGTGAAGAAGG
GTTTCGACTCGTAAAGCTCTGTTGTCGGGGACGAACGTGGAGACGGCGAATAGCCGTTTC
CAATGACGGTACCTGACGAGGAAGCCACGGCTAACTACGTG
>4056 len=161 tot-seq=2
TTAGGAATATTCGTCAATGGGGGAAACCCTGAACGAGCAATGCCGCGTGAGTGATGAAGG
CCCTATGGGTTGTAAAACTCTGTTGTTAAGAAAGAATGGTAGAATTAGGAAATGAGTTCT
ACTTGACGGTACTTTTCAAGAAAGCCACGACTAACTACGTG
>4057 len=161 tot-seq=2
TGGGGAATATTGGACAATGGGGGGAACCCTGATCCAGCGACGCCGCGTGAGTGAAGAAGG
TTTTCGGATCGTAAAGCTCTGTTGTAAGTCAAGAACGAGTGTGAGAGTGGAAAGTTCACA
CTGTGACGGTAGCTTACCAGAAAGGGACGGCTAACTACGTG
>4058 len=161 tot-seq=2
TAGGGAATATTCCACAATGAGCGAAAGCTTGATGGAGCGACGCAGCGTGCACGATGAAGG
TCTTATGGATTGTAAAGTGCTGTTGCAAGGGAAGAAAAGGTAGTTGAGGAAATGCCTCTA
CATTGACGGTACCTTGTTAGAAAGCGATGGCTAACTATGTG
>4059 len=161 tot-seq=2
TAGGGAGTCTTCCGCAATGGGCGAAAGCCTGACGGAGCAACGCCGCGTGAGTGATGAAGG
TTTTCGGATCGTAAAACTCTGTTGTTAGAGAAGAACAAGTGCCGTTTGAATAAGGCGGTA
CCATGACGGTACCTAACGAGAAAGCCCCGGCTAACTACGTG
>4060 len=161 tot-seq=2
TGGGGAATATTGCGCAATGGGGGAAACCCTGACGGAGCAACGCCGCGTGAGTGATGACGG
CCTTCGGGTTGTAAAGCTCTGTTAATCGGGACGAAAGGCCTTCTTGCGAATAGTGAGAAG
GATTGACGGTACCGGAATAGAAAGCCACGGCTAACTACGTG
>4061 len=161 tot-seq=2
TAGGGAATTTTTCACAATGGGCGCAAGTCTTATGAAGCAATGCCGCGTGAACGATGAAGG
TCTTATAGATTGTAAAGTTCTTTTATATGGGAAGAAACGCTAAGATAGGAAATGATTTTA

GTTTGACTGTACCTTTCGAATAAGCGCCGGCTAACTACGTG

>4062 len=161 tot-seq=2

TGGGGAATATTGCGCAATGGGGGAAACCCTGACGCAGCGACGCCGCGTGCGGGATGAAGG

TCTTCGGATTGTAAAACTCTGTTGTCAGGGACGAATGTACTGATTTACAATACACTTCGG

TATTGACGGTACCTGACGAGGAAGCCACGGCTAACTACGTG

>4063 len=161 tot-seq=2

TGGGGAATCTTCCGCAATGGGCGCAAGCCTGACGGAGCAACGCCGCGTGAACGATGACGG

CCTTCGGGTTGTAAAGTTCTGTTATATGGGACGAACAGGACATCGGTCAATACCCGGTGT

CTTTGACGGTACCGTAAGAGAAAGCCACGGCTAACTACGTG

>4064 len=161 tot-seq=2

TGGGGAATATTGCACAATGGGCGCAAGCCTGATGCAGCGACGCCGCGTGAGCGATGAAGG

TCTTCGGATTGTAAAACTCTGTTGTCAGGGACGAATGTACTGATCTATAATACACTTCGG

TATTGACGGTACCTGACGAGGAAGCCACGGCTAACTACGTG

>4065 len=161 tot-seq=2

TGGGGAATCTTCCGCAATGGACGAAAGTCTGACGGAGCAGCGCCGCGTGAGTGATGAAGG

TCTTCGGGTTGTAAAACTCTGTTGTCAGGGACGAATGTACTAATCTATAATACATCTCGG

TATCGACGGTACCTGACGAGGAAGCCACGGCTAACTACGTG

>4066 len=161 tot-seq=2

TGAGGAATATTGGTCAATGGACGGAAGTCTGACGGAGCAACGCCGCGTGAACGATGACGG

CCTTCGGGTTGTAAAGTTCTGTTATACGGGACGAATGGCGTAGCGGTCAATACCCGTTAC

GAGTGACGGTACCGTAAGAGAAAGCCACGGCTAACTACGTG

>4067 len=161 tot-seq=2

TGGGGAATCTTCCGCAATGGGCGAAAGCCTGACGGAGCAACGCCGCGTGAGTGAAGACGG

CCTTCGGGTTGTAAAGCTCTGTTATACGGGACGAATGGCAAGGTGGTAAATAGCCATCAT

GAGTGACGGTACCGTAAGAGAAAGCCACGGCTAACTACGTG

>4068 len=161 tot-seq=2

TGGGGAATCTTCCGCAATGGGCGAAAGCCTGACGGAGCAACGCCGCGTGAGTGAAGACGG

CCTTCGGGTTGTAAAGCTCTGTTATAGGGGACGAACGGCCGGGTAGCGAAGAGGTAGCCG

GCATGACGGTACCGTAAGAGAAAGCCACGGCTAACTACGTG

>4069 len=161 tot-seq=2

TGGGGAATCTTCCGCAATGGGCGCAAGCCTGACGGAGCAACGCCGCGTGAGTGAAGAAGG

TCTTCGGATCGTAAAGCTCTGTTGACGGGGACGAACGTGCGGAGTGCGAATAGCGCTTTG

TAATGACGGTACCTGTCGAGGAAGCCACGGCTAACTACGTG

>4070 len=161 tot-seq=2

TAGGGAATCTTCCACAATGGACGCAAGTCTGATGGAGCAACGCCGCGTGAGTGAAGACGG

TCTTCGGATTGTAAAGCTCTGTTATACGGGACGAACGGCAAGGTGGTAAATAGCCATCAT

GAGTGACGGTACCGTAAGAGAAAGCCACGGCTAACTACGTG

>4071 len=161 tot-seq=2

TAGGGAATCTTCCACAATGGGCGCAAGCCTGATGGAGCAACACCGCGTGAGTGAAGAAGG

GTCTCGGCTCGTAAAGCTCTGTTGTTAGAGAAGAACAAGGATGAGAGTAGAATGTTCATC

CCTTGACGGTATCTAACCAGAAAGCCACGGCTAACTACGTG

>4072 len=161 tot-seq=2

GCAGAAATTGAGGTGTGGAAGGAGAGAGTATTTCCATGACACAGAATAAAACACTCCAAT

CAGCCTCCGGTCCTGAGGTTCACCTGCACCAAGAATTTCTCTGTTAGGGTTACACATAAC

CTCCTCTAAGATCACTTAAGAATATTCTTCTTAGGAGAATC

>4073 len=161 tot-seq=2

CGGTGTCAATAATGCGGTAAGGCACTTCCACCTTAGCGAGCATCTCTTGCTCCATTCCAA

GCAACTTTTGATGTTCTGCGCGAGAATCTTCCTGCTTGCAATAAACAAACATCTCTACTT

TATCGAATTGATGCACGCGAATAATGCCAGAAGTATCTTTT

>4074 len=161 tot-seq=2

TGGGGAATATTGCGCAATGGGGGAAAGCCTGACGGAGCAACGCCGCGTGAGTGAAGACGG

CCTTCGGGTTGTAAAGCTCTGTGATTCGGGACGAAAGATAAGTAGATGAATAATCTGCAT

AAGTGACGGTACTGAAAAAGCAAGCCACGGCTAACTACGTG

>4075 len=161 tot-seq=2

TGGGGAATATTGCACAATGGGGGAAACCCTGATGCAGCGACGCCGCGTGGAGGAAGAAGG

TCTTCGGATCGTAAAACTCTGTTGGTAGAGAAGAACGTTGGTGAGAGTGGAAAGCTCATC

AAGTGACGGTAACTACCCAGAAAGGGACGGCTAACTACGTG

>4076 len=161 tot-seq=2

TCACGCCCACACTAAGGTCCAGGATGGAGAGAGAGAAAGAGAGAGGCTGATTGATCGACT

GGGCTTTACTGCTCCTTCTCTGACTTCCGTGGGTTCCCATTGTGCCTCTAAGAAAGCAGG

ATTCAGCGGGCGTCAGTGCTGTGGGCTATATTTATACCCAT

>4077 len=161 tot-seq=2

TGAGGAATATTGGTCAATGGACGAGAGTCTGAACCAGCCAAGTAGCGTGAGTGAAGAAGG

TCTTCGGATTGTAAAACTCTGTTATACGGGACGAAAAAGGCGGATGCCAACAGTATCCGT

CCGTGACGGTACCGTAAGAGAAAGCCACGGCTAACTACGTG

>4078 len=161 tot-seq=2

TGAGGAATATTGGTCAATGGGCGAGAGCCTGAACCAGCCAAGTCGCGTGAGTGAAGACGG

TCTTCGGATTGTAAAGCTCTGTTATACGGGACGAACGGCAAGGTGGTAAATAGCCATCAT

GAGTGACGGTACCGTAAGAGAAAGCCACGGCTAACTACGTG

>4079 len=161 tot-seq=2

TGGGGAATCTTCCACAATGGGCGAAAGCCTGATGGAGCAACGCCGCGTGAGTGAAGAAGG

TCTTCGGATCGTAAAGCTCTGTTGCACGGGACGAAAACTGGGTTTGAAAACATTGAACCT

GGGTGACGGTACCGAGCGAGGAAGCCACGGCTAACTACGTG

>4080 len=161 tot-seq=2

AGCAGAGAACAAGAGGTTGGGGTGGGGAAGGGGAGAGATGGGACCAGACATGGGAGGGCA

AAGTGGAGAGAGAGCGCAATGAGAATGAGGCCAGAAGTGAGGAAGAGAAAACACCACAGT

CAGTGACAATCCAAACAGTCCCAGCGCCGCGTAATAGGAAG

>4081 len=161 tot-seq=2

TGAGGAATATTGGTCAATGGACGAGAGTCTGAACCAGCCAAGTAGCGTGAGTGAAGAAGG

TTTTCGGATCGTAAAGCTCTGTTGTAAGAGAAGAACGTGTGTAAGAGTGGAAAGTTCACA

CAGTGACGGTAACTTACCAGAAAGGGACGGCTAATTACGTG

>4082 len=161 tot-seq=2

TGGGGAATATTGGGCAATGGGCGCAAGCCTGACCCAGCAACGCCGCGTGAAGGAAGAAGG

CTTTCGGATCGTAAAGCTCTGTTGTAAGAGAAGAACGTGTGTGAGAGTGGAAAGTTCACA

CAGTGACGGTAACTTACCAGAAAGGGACGGCTAACTACGTG

>4083 len=161 tot-seq=2

TAGGGAATTTTCGGCAATGGGCGAAAGCCTGACCGAGCAACGCCGCGTGAGTGAAGACGG

TCTTCGGATTGTAAAGCTCTGTTATACGGGACGAACGGCAAGGTGGTAAATAGCCATCAT

GAGTGACGGTACCGTAAGAGAAAGCCACGGCTAACTACGTG

>4084 len=162 tot-seq=2

ACAGCGCTACAGACACAGCCAGGGCTGCTGAGCTATGCATGTGGCCCCCTGCCAACACAC

CCTGTTTCGCAGAGAGGATGGCGACCCGCCTAAGGCTACATGGCAGAGACCAGGGGAGCT

GAGGTTTGAACCCCAGGGTCAGCTCCAGAGGTAGACTCCTGG

>4085 len=162 tot-seq=2

TGAGGAATATTGGTCAATGGGCGCTAGCCTGAACCAGCCAAGTAGCGTGAAGGATGAAGG

CTCTATGGGTCGTAAAGCTCTGTTGTTAGAGAAGAACGTTGGTAGGAGTGGAAAATCTAC

CAAGTGACGGTAACTAACCAGAAAGGGACGGCTAACTACGTG

>4086 len=162 tot-seq=2

TGAGGAATATTGGTCAATGGGCGAGAGCCTGAACCAGCCAAGTAGCGTGCAGGATGACGG

CCCTATGGGTTGTAAAACTCTGTGATTCGGGACGAAAGATAAGTAGACGAATAATCTGCA

TAAGTGACGGTACCGAAAAAGCAAGCCACGGCTAACTACGTG

>4087 len=162 tot-seq=2

TGAGGAATATTGGTCAATGGACGAGAGTCTGAACCAGCCAAGTAGCGTGCAGGATGACGG

CCCTATGGGTTGTAAAACTCTGTTATACGGGACGAATGTAGGCATGGTTAATACCCATGC

GTAGTGACGGTACCGTAAGAGAAAGCCACGGCTAACTACGTG

>4088 len=162 tot-seq=2

GCTTAGAGGAGCGTGACCACACTTTGGGGGCTGCGTTCTTCATTAGCTGTGCTACGGTCA

CCTGGGCTTTGGGGCACCTGTCTGGATGGGATGTTGCTCAACGGGAGGAAAGGAGCTACC

TTCTTTGTCCTCTTGTTCAGGCACACCAGAGCTGAGCAACAC

>4089 len=163 tot-seq=2

GGATGGATGAGGGTGCCTGAGTTAGGCATTCTGGAGGGTGTGAGAAGCCAGGGAGACAGG

AGAGTGGCAAGGAATTCTTGAGCAACCTCGCAGGCCGGCTGCATGCAGATTATGGTTTTG

CCCCTCCCGTCTCAGCCTCTCTGGCAGCACCTCTCTGCACCCC

>4090 len=163 tot-seq=2

ACGGTGTCACACCTGTTCGCTGGCTGCTGAGCCACCCCCCCCCAAAGCCTGGCTAGCACT

AGAGGTTTGGTTATGCTGGGTTTCATCATTGTTTCTGGTCGCACTTCCTCAAGGCATTAA

TTGGTCTGTTGTGTTCCCCGCAGTGCTATAAAAGTTCCAAGGA

>4091 len=163 tot-seq=2

GGACCCTTCCACCCTGGAAGGATTCCTTGCGGTAAGAGTTGACACATGTTCAGTGTTTAG

AGTTGTCTGATGGACAGGACCAAGAGGTTGTCTGCAGATGAGCTTTAGCATCAAGGACAT

GACCAGTATTGATGCGGCTGAGAAGTTCATGGTTGCAATGTGA

>4092 len=163 tot-seq=2

TAGGGAATTTTTCACAATGGGCGCAAGCCTTATGAAGCAATGCCGCGTGAACGATGAAGG

TCTTATAGATTGTAAAGTTCTTTTATATGGGAAGAAACGCTAAGATAGGAAGAGCGTCGT

GTAGGGAAAGAGTGTGCCAATGTGTAGATCTCGGTGGTCGCCG

>4093 len=163 tot-seq=2

CTGATGAAGCCCCGTGGGGACCAGGAAGGTCGGCCTAGGAGGAGCTTCAGGAGCCCCATC

ACTCAATTCTGGCCATCCACACCTTGGAGCATCCGTAGGAATGCTGGAGCCCACCCTGGC

TTCTCTCAGACAAGCAGCCAGTCTGGAATCCACTCAGCCTCCC

>4094 len=163 tot-seq=2

ACCCATCCAAATAAATAATATTCTCTTCAAAGCCTACCCCCTTAGAAAGCTCTGTGCTGA

TTCCACTGGGGCTGTGTTTTCTCAGAACATTTTTGGAAAGTCCTATTGTGGAGCAGCCTT

TGTCTCCCAGAGAGTGTCCAGGATAAAGTTAATAAGCATCCAA

>4095 len=163 tot-seq=2

CCGCAAAACAACAACCAGCCAAATGTAATACTGCCATTGTCGGCTGATTTTGTTCTTCAA

AAACTGTTTTCCAAAAAGTAAAGCCAGAATAAGTAAACTGCCGGAAAAAGACATGGACAG

GAAAATCTTCAAAACTGCGTTCATTGCTGCCCTTTTCCTTTCT

>4096 len=163 tot-seq=2

TATGTTGCATTGTTTCATGTGTCAGAGCCCTCTGCAGACCTGTTACTGTGTCCCCTGTGA

GTCTAGTACTCAGGGAAGCCCTCCCTGGCAGTAGCACTTTGCCCTGGGGAAGGAGAAGGA

GACGCAGCAATCTGTAGGTGTGGCGCCCGCATGTGGGTAAGAG

>4097 len=163 tot-seq=2

GTGGAGGATATGGCCGGGAGTATTGCTGAGTTTATAGGATTAAAAGGCCCCACTGGAGAG

CTGCTGAGTTGATTGAATTCCCTAGACCCCAGATGCCAAGGTAAATGCCACTGGGGCAGC

TCTAGCACTTTTCAATAGTGCTGCCTGTGGCCCTGAGAGTGTG

>4098 len=164 tot-seq=2

CAACAAACTAATGCGCAATTTGAAGAGGCGCGTGCGCAGCGGATAATACTTATGCCGAAA

CTAGTAAGCAATATTGATCATAACGCCGCATTGTCTTATGAACTTACTGCTTTGTTGAAG

AAAAAAATTTATAATGAGCATACGTCTTCATCTCAACGCAAGAA

>4099 len=164 tot-seq=2

GGTGATGGGGCCTGGAAGCTGGGAGGGGTGCTGGACCTGCTGGGATGGGAGCAGGGGGAG

AGCTGATTGTTTTGTCCCCTCCTGAGTGCGAGAGTAATCTGTAACCCTTCCACAGCCTGT

GATGCGGGCCTGGAGCTCTGCACTGAGCTGCACGTTCTCTGTGC

>4100 len=164 tot-seq=2

TGGGGAATATTGCACAATGGGGGAAACCCTGATGCAGCGACGCCGCGTGAGTGATGAAGT

ATTTCGGTATGTAAAGCTCTATCAGCAGGGAAGAATAGGTCATCAGCCTTTGGCTGGTGA

CATGAATGACGGTACCTGACTAAGAAGCTCCGGCTAAATACGTG

>4101 len=165 tot-seq=2

GATCAGATGTGGAGAGAGACAAGAGAGTCCCAGAGAGGACTGGAAAGAATGGAAGGAAGG

AATGGAAATATACAACTGCAGGAAGTGAAGGGAGTAGGGGGATCTCTAGGAAGTCCCAGA

GACCTGGGATGGGGAAGGCACCCATGAGTCAATGCATGTAACTGT

>4102 len=165 tot-seq=2

TAATATTTCACTCTGCCAATCTCAACCGCCTTTTGCCCGAGCCTTTCCCTGGGACAGAGG

GCTCTGCGCAGGCGCCAGCAGCCCAGGGTTCCCGGGCCGCACGCGGCACTGGAGCAGGTA

CCATGTGATTCCAGGGAGCGCCTCGTGCCCCAGTGACACACTTTT

>4103 len=165 tot-seq=2

CCGCCATTTCCAGCTTCCTTCTTTCTGCTGTTCCAGTTGACCAGGCTCAAGCTCTGAGGT

CACTGTAGAGTGACTGAGGTTTTGCAAGGCTTTTCTGCCTGGCAAACATGGACAGCACAC

CATTCTCCTAGGAGCTAGAGAGATGAAGACAACAGTCTCCGCCCT

>4104 len=165 tot-seq=2

CCCGCCTCTTCCAGGCCCCCACCGGCTCGAAGTAGAGCGCAGCCCCGGCTGCACTTCCTC

TGCTGCAGCCATCGTCTTTGCAGCAGCCAGTCCAGATGAGTCGTCACTGCCATCACTTGG

ACATCAGACTCCAGGTTCGGTAGCCTTTGGACTCTGAGATTTGCA

>4105 len=165 tot-seq=2

GAAGGGGCAATCAAAGGAGAGAGAAGGTGGCATTTTTGCAGATTTGAAGGAGGGAAGAAG

TGAGACCTGGGGATTCACAGCCAGGGTAGGGGAGCTTTCAGGCAGAGAGAATGGCACAGG

CAAAGGCCCTGAGGCAGGAGTGGCTGGAGTGGCCAGAGCGGAGAC

>4106 len=165 tot-seq=2

AACTAAGGTACACCCCTAAGCATGTACCATAAAATAAGGAGACAACTGAGAGGTGAGATG

CACAGGTGGAAAATGCCTTATACTTCCCCTGAGCTGATGAGATTGCACGTATGGAGGAAA

CTATCTTAGAGTAAGAGTACAGGATCCCAGTGAGGGGACCACCGC

>4107 len=165 tot-seq=2

CAATGTTCAATATCTAGGACAATACACAAAACTTCTTAAGAATGAGCACTAAACCTTGTT

GCATTCAGAGATCTTTTGCCATTACCATACCAAGTGACTTCCATGGGACATTTATCCCTT

CTCACTTAGCTTGCCACTGCTGCAGGTCTTTTGTCACTCCTCATC

>4108 len=166 tot-seq=2

CCAGCCACCAGAGCTCCCAGAGGAGAAAGGGTCCTTTCTCCCCTTTCAAGAGACAGCCCC

AGAGTTCCCAGTGGCCCAGCCCTGAGGTTGTTCTGCTTAGGGCGTGGGGACACAGACTTA

CCTCCGCCTTCGCTCCGCATGCCGATGGTGCCGCCAGTGTAAATGG

>4109 len=166 tot-seq=2

TGGGGAGGGGGGGAGAGAGAGAGAGAGAGAGGCAGAGGAGAAAGGAGAGGCTGAGGGTAA

GACTCCCGTAGAGGCTGAGATCGAGGGAGGTGATGGGAGGCCAGAGCAAAGGTGGGGGCT

GCAGAGCAGAAACAAACACAGAAAGACCGAGAGAGGAGAGCAGGCA

>4110 len=166 tot-seq=2

CCGATCTCCTACGGGAGGCAGGAGTGGGGGATATTGCACAATGGGGGAAACCCTGATGCA

GCAACGCCGCGTGAGGGAAGAAGGTTTTCGGATTGTAAACCGCTTTTGATTGGGAGCAAG

CCTTCGGGTGAGTGTACCTTTCGAAGAAGCGCCGGCTAACTACGTG

>4111 len=167 tot-seq=2

AAGAAGAATCGGTGATGGTGCTACACCCAGCTTTGGCTGAGGTGGCTGATTTTGAAGCCA

GCTTGGTGGGTTTTGCAGATCTCTCTTCGGCACCAGTTGATTCGGACCCAGAACCACTTT

TAACACAAGAACTCTCTGTCCTCTTTCTTTTCTGATTCCGTGAACTA

>4112 len=167 tot-seq=2

TGACTGGGTGTCTGTGTCCACCGCAGTGACTCCTAACAGCTTTTCATCACATGCCCAACA

ACCCAGACATCTGGGTGTAAACCCGCTAGAGCATAGTCCTGCCCCCGCCCAACACCGTTT
AGCAATTTCCCTTCCTGAACACACGAGGCGAGCAGGCCATTTTCTCT
>4113 len=167 tot-seq=2
GAGCTATGGTGCAGAACTGGGCAGATAGGAGGAAGGCTTGATATTCTCTGCTTAAGGACA
AGATGAGATGGGACAGAGGATGTGGGCCCCCTTCCCTCCTCCAGAAGATCATACGTGCTA
CATCAGGGGGATATAACATTGCAGAAAGTAAAAGGCCAGCATCATCC
>4114 len=167 tot-seq=2
CAGCAGGAGGGAAGGGGGAAAAAGGGGCGCAAGACGGAGGAAGCAAGGCCGCGGGAACGA
GGAAGGTCTTATAGATTGTAAAGTTCTTTTATATGGGAAGAAACGCTAAGATAGGAAATG
ATTTTAGTTTGACTGTACCATTTGAATAAGTATCGGCTAACTAGGTG
>4115 len=167 tot-seq=2
TTGCTGACCCTGAGGTATGGCAATCACTTCTCAATACATTCAAGTATGCGATCATCGAGG
TTCCATTCTCAATCGCAATTGCACTTATACTTGCAGTATTCCTGAATCGTAAGATGCATG
GAAGAACAATATACAGAGCAATATTTTTCCTGCCAATGGTAGTTGCA
>4116 len=167 tot-seq=2
CACAGTTTGAGAGGACTGAGTCCAATTTTCAAAGAAATTCTGCTGTGGGTTAAATGCTAT
CAAACAGCATTGCATGCTACAGAGAAATCTTTTGTGAATGGAAAAATCAATTGATACAGC
ACTTCACTGTTGTCTTATTTTAAGAAATTGCCACACACACAGCCATC
>4117 len=167 tot-seq=2
CTGCGAGGGGGCAGGAGGCAGCCCCGGCCGCCCCGGGCCTTACCCTGCCAGCTCCAGAAG
TAAACTCGGAAGGCTGAGGCCCCGCGCGCTGCGCGCCGCTAGCACAGCGGAGATCTGCGG
CAGCTTCAGCGCGGCGCACACGCCCAGCGTGCGCCAGTTACACAGCC
>4118 len=168 tot-seq=2
GCCGCTGAGGGCAGAGTTGGAGGAGCTGCCAAACCGCAGTGCATCTGGGGCTGCTGAATG
TGTTGCAGCCTCCCCTGGGGTTTGCCCTGTGAGACCCCAGCCCCCTCAGGCTGAGGGTAA
GGTGTCCATGAGGCTGGAGAACAGGGTCAGGAACCCTACATTAGGAGG
>4119 len=168 tot-seq=2
TGAGGAATATTTTGCAATGGGCGAAAGCTTGACAGAGCAATATCACGTGAGGGACTGAGA
AATTCCGAGTTTATTATCTTTCTTTCGAAGGCCTACGGGTTGTAAACCTCTTTTCTCAAT

GAAGAAGATTTGACATTATTTGAGGAATAAGTATCGGCTAACTCTGTG

>4120 len=168 tot-seq=2

ATAGTGGGGAGTCATTGAAAGTTTTCTGCAGAGACAAGATGAAAGCTAGCCTTTAGGGGG

TTAATCGGGCAGCACTACGGTAGGTGGAGGTGAACTAGAAAAAGGACTTGGGTGGAGGAA

GGGGCATTCAGAAAGCAGTTTATGCCATGCAGAAGCAGTTCACGGTGT

>4121 len=168 tot-seq=2

ATCACCCCAGCCTGGGATGAAGAGAAGGTGCTGAGAGGAGACCTACCACTCAAATTGATC

ACCCAATCAGCCCCTCCCCAGAACGACAATGGGTTCTCAGGGCCTTTGGGGCCTGTGCTC

TAAGTGCTGACAAACCTTCGTGATTATCTCAAGGCCTGTTCAACTCTT

>4122 len=168 tot-seq=2

CCATGAGATTCCTGCCAAGCCACCTGAAACCCATACATGAGCATCCACCAGCAAATACCT

TATCTGGGACAATATGAACTTGGCATTTTGCTCATCCTCCCCCTCCCCCTTGACAGAGCG

AGCACAGCCCTCCTTCCTCCTGAGTCCAAATGTGGTCCAGGAATGCTC

>4123 len=169 tot-seq=2

CTATACAGGGAGACTGTTGGGAGGAGGGGGCGGGGCACACAAACTCAAACATGAAAATGT

CACAAAGATGACAAACCAGTGGCATGAAATGCCAATTTAACAAGTAAAAGTCTGGCCTGG

ATCAGGGGTGAAGTTCAGGGTGGAGCACTAGGTTAGCTGAGATGAGACC

>4124 len=169 tot-seq=2

TCAAGCAGAAGACGGCATACGAGATGGCAATGTGACTGGAGTTCAGACGTGTGCTTTCCG

ATCTCCTATGGGAGGCAGCAGTGGGTAAGGAAGCCAGCTGTGGCTAAGGAAGCCAGCTAT

GGGTAAGGAAGCCAGCTGTGGGTAAGGAAGTCAGCAGTGGATAAGGAAG

>4125 len=169 tot-seq=2

GGTCCGGCCTCAAGGGCCACCAGCAACATTCTGCTGGCCCTGGAAACAAAATAATGAGGG

CCTGGCCCTTCAACAGTATTTCTCTAACTCTGCTGCCACTCAGGGGCAGTTCCATATATT

GCTTTGTATCATGGTTACTTGTTATCGCTTTCTAAATGTTTTACTTCGT

>4126 len=169 tot-seq=2

CACCCTCTGCAGTATGGTTTTCAGGAAATCCAGAAGTGGAGGGATCCACCAGAAACTCCA

GGCACGTATGCAGCTGATGGGCCCGGCTGGCTTGGGGAGCATAGGTTGTCCCCAGGGAAA

GGGACTGTGCCAACCCTAGGAGGTGGTCTGCACATGGTGTCCGAGGGAG

>4127 len=169 tot-seq=2

CTGCTTTGTCTGGTTACCCTCAGACTGCCACGCTATGAGGAAGCCCAGCCTATTCACATG

GAGGCACTACATGGAGAGACAGAGTGATGACTGCCCAGCTGCAGCTCATGCAACCATCCC

AGCCCAGGGGCCAGACACGAGAGTGAGCAAGCGCCTGGGCGTTCCAGCT

>4128 len=170 tot-seq=2

AAGATGTGCAGGGACAAAGAAAGAGCAGAGATGGAATGAATGATCAACCAATGACGGTCC

CAAACTAAGACCCATCCCATAGGCAAGAACCAATCTCTTGACACTATTAATGACACTCTG

CTATGCTTGGAGACCGGAACCTAGCAGAATTGACCTCTGAAAGGCTCCAT

>4129 len=170 tot-seq=2

GGATCCGACCGCAAGGGCTGGTCCCTGAGGCCACTAAGGGGTGCGCTGGGCTAAGGCCGC

CTGGGGGCAGGAGCCAGCGCGATCGTGGCGGGCCAGCAGGCGGGGCTGGCTGAGGACTAG

CAGCGACCCGAGACCCCAACCCTTCCCACGCGGACCCCGGGATCCCTCTC

>4130 len=170 tot-seq=2

AGTCTAAGGAAATTTTCTTAGACAGAAGTCTGAAACTAGCATGGAGAATAAATGGTTAAG

GAAAGAGAGAGAAAGAGAGACAGCGAGAGAGAGCGAGAGTGAGAGCGAAAGCGAGAGCGA

GAGAGACAGATCAGAGATGGGACCTCCAAAGCCAGGATGTAGCAAGGTTA

>4131 len=170 tot-seq=2

GGGAGAAAGGCAGGGCTGGGCAGAGGTGGATGGGCATCTCTGGGTGGGGTGAGTCCAGTG

TGAAGTGCCCTTCCTGGGTAATAGGGTGAGGGCTCTCTCCACTTACACACCCTCCATCCC

TACCGATCACTCCAATCCAGTATCCACCTCCCCCACCAGACCGGTAGCCC

>4132 len=171 tot-seq=2

CTGGGTCTGTCTCCTCTTATGGCTGCCTTAGCAGAGACAGGGACCGACAGGGGCTGAGAA

ATGGCTCTGCACACAGGCAGGTGCCCTGGATTGCACAGGAGCAGAATGCCACAGGGCAAC

GCAGGCCAACACAACACTGTTGCAGACGTATGCCCCACACTCTCACTTCCC

>4133 len=171 tot-seq=2

CTGAGATGATCCCCACCTCCTCTGCCCACAAGTCCTCCCCCTCCTTAGCAGCTTGGGACA

AGTCTCACGTTCCCACCTCCAGAGCTGCAGTGGCCCCTTGACCATGGGTCGGGGAGGGTA

GAGTGACATGTTTGCTGACAGACTGGTGGCCAGCGTGCAGAGGGGAACAGG

>4134 len=171 tot-seq=2

ACTGGAATATCAATTACAGAAGGAGCCAAGAAAACATGGACATTTAGGGTGTGTATGCAT
TCTGTTGCTGGGATAAAATACTATGAGCAAAAGCAACTTATACAATGAATAACTGGTCTT
GTCTTATGGTTCCAGAGGGACAAAAGCCAATCATAGTAGGAGGCATGGCAG
>4135 len=171 tot-seq=2
TGGGGAATCTTGGACAATGGGCGAAAGCCCGATCCAGCAATATCGCGTGAGTGAAGAAGG
GCAATGCCGCTTGTAAAGCTCTTTCGTCGAGTGCGCGATCATGACAGGACTCGAGGAAGA
AGCCCCGGCTAACTCCGTGCCAGCAGCCGCGGGAGTAGACGGAAGAGCGTC
>4136 len=171 tot-seq=2
TTTCAAGCAGAAGACGGCATACGAGATAAGTTGGTGACTGGAGTTAAGACGTGTGCTCTC
CGATCTCCTACGGGAGGAAGCAGCTCCAGCGTCAAATTTGTCTCCACCACCTCCTCCTCT
CGGAAGAGCTTCAAGAGCTAAGAGCCTGCAGCAAGTTACTGCCTCCCAAGC
>4137 len=172 tot-seq=2
AAGAATGGGAGCCTAGGGTTGGCAAAACTGCCCTGGGCTTGAGGAAGCCAGTTTGGAAAA
GAAGCTTAGCAGGAGGAGGGGCCCCTAAGCAAGACCATGTCAGGCAGGTTTCCACCAGGG
CATGAAGGTGCATTTCAACGGCAGCCCCTAACTTAGTCTCTGGGGCAATTGG
>4138 len=172 tot-seq=2
TCCGACCTGTGTTTAACAGGACCGCTCTGGTTCCTGCTCTAAGACAGGCTGAGGGGACAC
GGGGGCGCCTGGGAGACCTGCTGAGACTGTCGAGGAAATGAAGGAAATAGAGGGGACAAA
ACATGGAAGTGGCAAGAAGCAGCTGGGTTCTGGGTGTGTTTTGAAAGTAGAG
>4139 len=172 tot-seq=2
CCAAGCCACTCACTGGTGGTCCCACAACACCCCATGGAAAGAGGACAGACCCACAGTCCC
ACCTGGACCAGGGCAGAGACTGCTGAGACCCAGCACCAGAACCAACCAAGAAACACCAGG
CAACAGCATCAGAGGGGGCTCTGGCAGAACAGAGGAGGGGAGGTCTCCTTCA
>4140 len=172 tot-seq=2
CAAAGCTCCAACCTGGACCCACGCAGCTCACAGTAGCTCCCGCTTAGGCTACACACAGGC
CTGCAATCCACAGATAAACACAGCTGGAGAGAAAAAGCAGCCGGGAATGCAGCTGGGCTG
TTTTCTCAAGAGAAGCCCAAGTGGTAAGTAGGGATGCAGCCGCCCTCCCTGC
>4141 len=172 tot-seq=2
CACCATGTCCTCGTTTGAGGGGTAGTCTGGCAACCTGACCACCCTTACTCTACAGAAATT

CCAAGAGCAAGTCAAACATGTGCTCTCTGCCTGCGATGGGCACTATGCTACTTCTCTTTC

CTCTTGCTGGAAGAAGTCACACCCTACTCTCCAACCCATGCAGCCCTCCATC

>4142 len=173 tot-seq=2

CTGAAGGGGCAGGTCGGATACACGTGGCTTTGCTTGAGATCCTGGGAAAAGCCCTGCTTT

TCCTGGTCTCTCTCCGTCTCTTAGCTCCTGTCCAGAGCACTTTCCTTGCTTGGCCCCCAG

ACTCAGGGCTCTCCTGGCTTTTCTCCCCTCTCTCTCAATTGCTCTTCCTCTGG

>4143 len=173 tot-seq=2

GTGACTGGAGTTCAGAACGTGTGCTCTTCCGATCTCCTACGGGGGGCAGCAGAGGGATGC

ATGCTGTGTGCCCAGCAGCCATGGTCAGGGACAGAGGCAGCAGAGGGATGCATGCTGTGT

GCCCAGCAGCCATGGTCAGGGACAGAGGCAGCAGAGGGATGCATGCTGTGTGC

>4144 len=173 tot-seq=2

ACTTCTCATTACAAACTATGCCAATCAGAAGACAACAGAGCAGCATCTTCAAAAAATTTA

AAACACAAACAAAATCCAAAGTCAGTTTGGAATTCTACACCCACCCAGCAGAGCTATCTT

TCAAAAAATGAAGATAAAGAGATTTTCAGATTTTAAAAAAACAGTGATAATTT

>4145 len=173 tot-seq=2

AGTCGTGATCAGGTCCAGCTAGAGTTCTGCTTTTAGACACACAAGGCCTGAACGGGTTTA

GAATGCTTATTTCATGCGTCGCCTTTCTCAGAAAGATACTGGGTGAGGCATTCTACTGAA

ACAAAGGAATTAAGCCCAGAAAAACAGCAGATCTAAATTCAGGAGAGGGGGCC

>4146 len=173 tot-seq=2

GGTTTCTTCATGCCCTTTCTTGGTTCTCTGATGATAGGCAAAGGCAGTAGAGACAGAAAG

GAAAAGAGGAACGGAGTGCAAGAGACAGAAAGGAGAAGAGGAACGGAGTGCAAGAGACAG

AAAGGAGAAGAGGAACGGAGTGCAAGGCAAGCCACCCTAAGAGGCCAGCCCTG

>4147 len=173 tot-seq=2

GCTTTCTGGCTTCTCTTCTTAGCTACACGTGCAGCAGGGTTATGGCAGTGCAGGGAATCA

GCAGATAGGGTCTCTGGCTGAGCAAGAAGTTCCAAGGGAGCACAGATATGACACTGCAGG

GGGCCTGTTCTCAGGGGTCTGCAGTCCCTGTGTGACCCTGCTAGAGCCTTGCA

>4148 len=174 tot-seq=2

GGGAGTCCCCTAACTCACACCGCTGTCAGCTCTTGAGGGAGGTGTGCCAACCACTGACTG

TCCCCACCAGCTGCGTATCACTGGAGAAATTCCTCATTGAAGTCTCAGTTCCCTCCCTGC

CAAAGAAAAATGATAATCCATGCCTCCTACTGGCCACATCTAAGGAGCCTGGAC

>4149 len=174 tot-seq=2

CGGGATAAACGCCGCCATACCGCCCATCGCAAACGCGCCGCGTTTGTGGCAGGTCTTAAT

CAACAAGCGTGAGTAGGCGCTGAGGAAGGGTTTATCCATCGTCACCACCGGCCGGTCCGG

CAGTACGCGATCCGGATGGTTTTTCAGCGTCTTGATATAACTGAAGATGTAATC

>4150 len=174 tot-seq=2

TGGGGGATATTGCACAATGGGCGCAAGCCTGATGCAGCAACGCCGCGTGAACGATGAAGG

TCTTCGGATCGTAAAGTTCTGTTGCAGGGGAAGATAATGACGGTACTCTGTGAGGAAGCC

CCGGCTAACTACGTGCCAGCAGCCGCGGAATGCACAGATCGGAAGAGCGTCGTG

>4151 len=174 tot-seq=2

CCCAAGGAGGGAAGTGTGGGAGCTGCGGGGACCATGCCCAGCCTGGGATACCACCCTTCT

AACCCACCCTCACCCTCTGCAGGTGGTCCATCCCGAATCCAGGCATCACCCACCCTCGAT

CCCGAGACAGTAGCCCCACCTGGACTCAGGGTCCCTGCCTCCCGGACGGCAAGG

>4152 len=175 tot-seq=2

AGGGGGAAGAGTGTAGGCAGAGGGTAAGACTTTTGTTTTAAGAGATATTCTGGGTTGCTG

TATAATCATAAAATCTTTACTTATCTAAGTTACTTAGTTACTGTAGCTAACCTGCCTTCT

GTGTTCCTCTGAGGCTAGAAACTGCAGTCTCTGGCATTTAGCACCTCATCCTAAA

>4153 len=175 tot-seq=2

GAGCTTGAAGCAGCTTCTCATTTTACACTCACAGGAAACGGTGAGCAAGAAAAGCCATTC

CTAGTGTCTGGCTTTTTTCCACTCTGCGCCGTCCAGGGTCTCCTGCAGGGGCTAGCCCTC

TCTCCAGTCAAGAAGAGCTTCTCAAAGGAACTGACCCAACAGAGACACTGCTTCA

>4154 len=175 tot-seq=2

GGAGCTCAACCAGCCTGTGGTTGAGCCTGCGGGACCTGCCTTTATTAAGGCCCAGGGTAC

TATTCCTTAGGCTTTCACTTGGGACTTGTGGATCTGGGTCATTTAAAGAAAACATACCTA

AAGGAGGGAACTCATTTACATGACTCCAGTGTTGATCATTAGATTTTTTCCACAG

>4155 len=175 tot-seq=2

CCTCTCTTGAAGAAGCCAGCACTGCTTTAAAATGAGTTCACCCTGCCATCCAGGCAGCTC

TACCACCAGGGTTTCCTGTTTGTGACTCAGCCCCGAGACAGCAGGACAGTTGTGCTGTGA

TCCAGCTCAGCCCCTGGCTCCTCCTCATCCTCTTTCCTTCTATTTTTCCCCTCTC

>4156 len=175 tot-seq=2

TGGGCGAAAACAGAGCGTGGTCCGAGCAATCCGTATCTGCGGTAGATGTCGGTATAGTTC

GCCGCTTCGGGATATAGCGATAATGCCCATTCGATTTCATCGTCCGCTTCGTCGAGATGT

GTATGCATATACACACCTTTGCCGACGTTTTTCTGATAAAGGTCTCCGGCGAGTT

>4157 len=176 tot-seq=2

AAGCCAGCACTGGACTGCCCACCCCACTTCACATCATCCCATCTTAGGACCTTCAGTGAG

CCAGCTCAGGGGCTCAGATGGACAAAGGGCAGAGGCTTTGTCCTCCTCAGATAATAACAG

AGATGGGTCACGCAGTGGCCACAGGAGGGTTGTGAACAAAAAATAAAGCACTGGCC

>4158 len=176 tot-seq=2

CCGACGGGAGGCAGCAGGGAGGAAGGGGGCACAAGGGACGAAAGGCGGAGGGAGCAAGAC

CACGGGAACGAGGAAGGTCTTCTGATTGTAAAGTTCTTTTATTTAGGAAAAAAAGCGCGC

TAGGAAATGAGCGCGCCTTGATGGTACTAAGTGAATAAGTGACAGCTAACTATGTG

>4159 len=176 tot-seq=2

CAGAAGTTTAGACAATTATGGTTGGATGATTCTTAACTGTCCTAATCCCAAGTCCTCAAT

TTATAAGTGAGGAACTGGAAAATCATTCACACAGCTAGCTGCAAGCAATGACAGCCTTGG

AGAGAAATCAACCTGTGTACAGCTCAGTCCCATCTGAGTTCTTTGGAGAACAAACT

>4160 len=176 tot-seq=2

AGCTTGCAGCGTGGCTCAAGGGAAGTGGGCAAGGATAGAACAAGTTGAGGCCAGGCTGGA

AGTTGCATGCAAGGACTAAATTAGGCACGGCCTTCAGACATCTTGAGGATCTGGAGGCCT

TAACCCAAATGGTAAGGAAGCCAGGAACGGTTTTAGGCAGAGGCTAAAAGATCACT

>4161 len=176 tot-seq=2

GGCATATGGACAAGGCGGAGCAGGCCAGCCGTTAGGCCGTGCACCGTATCCGCAGGGCGG

AATGAGGCAGCCTGGAAACCGCGCGCCGTATCCGCAGGGAGCGGCGAACCAGATGAGAAA

CCGTCAGGGAAGCGTTCCCGGTCATATGCGCCCGAGCCAGCCTCAGGGAACAATGG

>4162 len=177 tot-seq=2

TCAGTCTCATAGAGGCAGACTCTTGACATGGACTAGCCTTCCTTCCCACAGGTTATTTAT

TTGTCTGTTTTCATTCAGAAGGCTCCTCGGGAGTTTCCAGTGTTTTACAGCAGCCTCAGC

CCAATCCAGGCCCTGAGAAGGAGAATGTTCGGACTGTGGTGAGGATGGTGACTCTAT

>4163 len=177 tot-seq=2

AGCTGAGGGCCCAGACGCCAGCAGCTCACCCCTTAGACAGGAGCAAAGTGCAGAGGGCAG

ATGGTGGCATCCAGTGTGCGGTCTGAGGGAGGCCCTGGGTCAAGGTGGAGGTCTGCAGGA

GTGTAGAGCGGACAGCATCCAGGTGGACAGCTAGGGGAGGCTGGGGGCCACTAGGCC

>4164 len=177 tot-seq=2

GTTGTGCTCTTCCGATCTCCTACGGGAGGTAGCAGTGGGGAATATTGCGCAATGGGGGAG

ACCCTGGCGCAGCGACGCCGCGTGCGGGATGAAGGCCTTCGGGTTGTAAACCGCTTGTGA

GTGGGAGCAAGCCTTCGGGTGAGTGTACCTTTCGAATAAGCGCCGGCTAACTACGTG

>4165 len=177 tot-seq=2

AGTGGAAAACGTATCCCCTAAATGGCTGGCCAACCTTAGCATATGGACAGTGTCACCTCT

CTCACACAGAGCCACTAAAAACTAAACACCAAAACCAGTTTTCTTGGGTAAAGTTTTCTA

AGATGGAAAATTTAAGCAGCAAGATGTGTCAAGTTGTAGATGTTGGCCAGGAAAAAG

>4166 len=178 tot-seq=2

CCACAGCTCCTTCTGTGGGCAAAGCTGTCCCGGGTGTGGATCATGACAGGGGACATGAAA

GCAACAGCTAGAGCCTTTCTCAGGCTTCTGAGTCCCCGTGAGTATCAGTGAGGTCCACTG

ATCATCTTTTGCTCTCAAACCCATACCCCTACCTAAACATCTGGATCTCCATATCCCA

>4167 len=178 tot-seq=2

AGCCAGTGTGTGCTTCATGGGGTCAGGAAGGTAAGGGGCTCAGGCTCAGGAGGGAACAGA

CAAGCCCAGGGCAGGCCATACATCCTTCTCCAGCCTCTGTCCCTCTCTCCACAGCTCTCA

AAACCTGCCCGACAGGTTTGGGTTCCTGAGCTACCTGGCCTCCCGCCTCCCTGCCTGC

>4168 len=178 tot-seq=2

GGAGGGTTGTTTGGACTGTGGTGTGTAACCCAGAGCCACCCCCATTCCTGAAGCAGGAAG

CCCCTGCTCTGGGAGGCCCCTGAAGAGGGGCAGGAGGAAAAGGAAGGATGCCGCTGACTA

CTGTCATCTCTCTACTCCCAGCAGGCCCCTCTGCAAGGTCATCTCTCAGGTTACCAGA

>4169 len=178 tot-seq=2

AAATGTAGTTGTGTTGGTTTTTGCTTCTTTAGCCAAGCAGCTACCAAAAAAAAAAAAAGG

AAAGAGACTGAATTGATAGATACATTTTACTGTCCAGCTGCCAAGAAGGCATTGCCGACA

ATGAGGATTTCAGAATAATAACCCTAAAGAGAGGAGTTCAGAGCATCCCATTCACTGC

>4170 len=179 tot-seq=2

CACTCTGTAGACCAGGCTGGCCTCGATCTTAGAAATCTGCCTGCTTCTGCCTCCCAAATG

CTGGGTATTAAAGGCATGCGCCTCCACTGTCCAGCTCAGCTGGCACATCTTACCAGATAT

GTCAAAAGTGTATTAAACAGGGTCCACAGCCAAGCAGGACAGCTGGAATCAAGTCTCCT

>4171 len=179 tot-seq=2

CCCTGGCTGTCCTGGAACTCAGACTGGCCTTGAATTCATAGAGAGCAGCCTGTCTCTACC

TTCCAAGTGCTGGGACTAAAGGCTAGTATTTGTCTAATTCTAAGCAGCCTCTGACCACTT

GTTTCTCCAAAGGTGGAGCGAATCAAGGAGCGTGTGGAAGAAAAAGAAGGGATTCCCCC

>4172 len=179 tot-seq=2

TGGGGAATATTGCACAATGGGGGAAACCCTGATCCAGCGACGCCGCGTGGAGGAAGAAGG

TCTTCGGATTGTAAACTCCTGTTGTTGAGGAAGATAATGACGGTACTCAACAAGGAAGTG

ACGGCTAACTACGTGCCACAGCCGCGGTAATAGTTAGATCGGAAGAGCGTCGTGTATGG

>4173 len=179 tot-seq=2

TGGGGAATATTGCACAATGGGGGAAACCCTGATGCAGCGACGCCGCGTGGAGGAAGAAGG

TCTTCGGATTGTAAACTCCTGTTGTTGAGGAAGATAATGACGGTACCTAACAAGAAAGCA

CCGGCTAACTACGTGCCAGCAGCCGCGTAATCGCCAGATCGGAAGAGCGTCGTGTAGGG

>4174 len=179 tot-seq=2

AAGCAGGCGGCCCAGGGCAGTGGTGGCTACATGGCCCCTGCTCCTGTCAGGGGCTGCAGG

GACCACGCACCACGATGTTGTTCGGCTCCATAGACTCCACGGCCACCAGGAACTTGTGCT

GAGCCTGCTGGTACTCCTCACTGTGCTCAAACGCAAAGAAGGAGAGGCCTTTTTTGATT

>4175 len=179 tot-seq=2

CCACATAACATGAAGAGAGAATCTGTGCATTTGGGAGATGGAGAGCACAGTGATTGTGTG

ACTTTGCATTGAAACTCTCTGCTTTCCCCATCACGGTGAAAGCAACACCAGGCAGAACTC

AGCTGGTGCTCATGAAGGGAGCATTTAGACCAACCCTAGCCAGATAGGAATCACTCATC

>4176 len=179 tot-seq=2

CTTGACAGATGTATGATCTTGAGCAAGTTTCTAAACCGCTCTAAGCCTCAGTTACCTCAT

CTACAAAATGGGGATTCCAATAGTACCTGATAGTAAATATAAATGGTTTGCACCATGCCT

GGCGCAGCAAACTCCATAAATAGTGGCTATTGTTATCCTCACCTCCCCCATTAACTCCT

>4177 len=180 tot-seq=2

TGGGGAATATTGCACAATGGGGGAAACCCTGATGCAGCGACGCCGCGTGGAGGAAGAAGG

TCTTCGGATTGTAAACTCCTGTTGTTGGGGAAGATAATGACGGTACCCAACAAGGAAGTG

ACGGCTAACTACGTGCCAGCAGCCGCCGGTAATCACCAGATCGGAAGAGCGTCGTGTTGG

>4178 len=180 tot-seq=2

GCGCCGACGGGAGGCAGCAGTGGGGAAGAGGGCACAAGGGGCGCAAGAAGGATGCAGCCA

GGAAGAGGGTATGAAGAAGGCCTTCGGGGGGGAAAGGACTTGCAGCGGGGAGGAAGGGAG

GAAAGGGAAGAAAGGGGCGCATTGAAGGGACCCGCAGAAGAAGAACCGGCTAACTCCGTG

>4179 len=180 tot-seq=2

AGAAGGGGGAGAGGAAGAGAGAAGAAGGGAGGAAGAAGGGGGGAAGAGGGAGAAAGGGGG

GAAAAAAGGAAGAAGAGACGAAGAGGGCGGGAGGAAGGCCGCCGGGGTGGAAACCGCGTT

GGAGGGGGAGCAAGCGGGCGGGGGAGGGGACCGGGCGAAGAAGCGCCGGCGAACGACGTG

>4180 len=180 tot-seq=2

TGGGAGAAAGATGCCAGTGTGCTGGTGCACATCTGTGTGGGCCACTCTGCTGGAGCTCTT

TCATGGTCAGGCATGGTCTTCCAGCACAAAGCTATGATGCGGGCCCTTGGCAGGCACCCC

ATTTGGGCATCAAAGACTGTACTGCAAGCAGGCGTGGCCAAGCTGGGGCCCCATAAGAGG

>4181 len=180 tot-seq=2

TACAGGGAAGGCGCGCGCCACTTGCCAGACTGCAATCTAGCTTTTCTCAACCTCAGTTTT

ATTACACGTAAAATAGAAGCTAACATGAGCCTTACTTCTCTTCCAGAGTACAAAATGGAA

AGTCTAATGAACATTAATCATCTTTGCAATAACAGCTAACAGTTGAGTGCTAAAGATAGG

>4182 len=181 tot-seq=2

TGGGGAATATTGCACAATGGGGGAAACCCTGATGCAGCGACGCCGCGTGGAGGAAGAAGG

TCTTCGGATTGTAAACTCCTGTTGTTGAGGAAGATAATGACGTACTCAACAAGGAAGTGA

CGGCTAACTACGTGCCAGCAGCGCGGTAATGCATAGATCGGAAGAGCGTCGTGTTGGGGA

A

>4183 len=181 tot-seq=2

ACAATGATGTGAAGAGCCAAGGGACAGGGGATGGATGTACATGCGCCAAGGGCAGCCAAG

GATGGCCAGCCATCAGGAGAAACTGAGGTTCATAGCTTTGCCAACATCTTGATTTCAGAC

CCCTGGGGCTACAGGAATGGGGCATTCTGAGGTACACAGCCACCCATCTTGGAAACTGTT

C

>4184 len=181 tot-seq=2

ATCTCCTACTGGAGGCAGCAGTAGGGAATCTTCCACAATGGACGCAAGTCTGATTGAGCA

ACGCCGCGTGAGTGAAGAAGGTTTTCGGATCGTAAAGCTCTGTTGTTGGTGAAGAAGTAT

AAATTTAGTAACTGGCCTTTATTTGACGGTAATCAACCAGAAAGTCACGGCTAACTACGT

G

>4185 len=181 tot-seq=2

TAGGGAATCTTCCACAATGGACGAAAGTCTGATGGAGCAATACCATGTGAACGATGAAGG

TCTTTTTGATTGTAAAGTTCTTTTATGAGGGATAACAACTGATAGTACCTCATGAATAAG

TGACAGCAAACTATGTGCCAGCAGCCGCGGGTAATCTTTAGATCGGAAGAGCGTCGTGTA

G

>4186 len=181 tot-seq=2

TGAATGAGACACAGTTCTTGCCTTCTAGAAGCTCTGACTCTGGACAGAAATGAAGATTGA

TGGGAATTTTGTAATATAAAAATAAAAGGCTAACTGTGAGCAATGGTGATAAAGGCCACA

TAAGCTATTGGCTCAGGCACTGTGGCTGCACCTAAGGGAGGCACCCCACCCAGTTCTGGA

G

>4187 len=181 tot-seq=2

CCATTCCACAAGGGAGATGGGTCAACATGGCTGACTAGAAACAGCTCATGTGTGCCTGTC

TCATAGGGAGTGGACAGAGTGGCTAGTGAACATTGACCTTGCAAATCGGTTGTCGAAGAA

AACACATTAGGATCCAGCAACACAGTGAGGGGACAGACAGAGCAGAGAGGAGAAAAGCTG

G

>4188 len=182 tot-seq=2

ACCCCTAAACAAGGAACCGCAACCTCTCACTTTACAGAGAGGTTCACAGGAATAGTAACA

GACCTGACAAAGTAAAACTTCTTTCAGTATCATATGGACAGCAGACACGTGTCCTTCAGC

CCCTCCCCATCTGAAACTCTCATGTCTTGGAACAGTAGGGAAGGTGGCTTCATTCACCTT

TC

>4189 len=182 tot-seq=2

TGGGGAATATTGCACAATGGGGGAAACCCTGATGCAGCGACGCCGCGTGGAGGAAAAAGG

TCTTCGGATTGTAAACTCCTGTTGTTGAGGAAGATAATGACGGTACTCAACAAGGAAGTG

ACGGCTAACTACGTGCCAGCAGCCGCGTAATGAAGAGATCGGAAGAGCGTCGTGTAGGGG

AA

>4190 len=182 tot-seq=2

TGGGGAATATTGCACAATGGGGGGAACCCTGATGCAGCAACGCCGCGTGAAGGAAGAAGG

CTTTCGGGTTGTAAACTTCTATCAATAGGGACGAAAAGAATGACGGTACCTAAATAAGAA

GCCCCGGCTAACTACGTGCCAGCAGCCGCGGAATCACGAGATCGGAAGAGCGTCGTGTAT

GG

>4191 len=182 tot-seq=2

TGATATGGAATCGTATGTGAAGTACAAAGACACAGAACCCAGCAGCAGTGTTCTGGAATC

CTATCTGGGCGACAAACATTCAGAACTTCCTAGCAGTGTCCTGGAATCCTTTGTGAGGAA

AAAACATTCAGAGCCTCATAACAGTGTTCTGGAATCCTATGTGAGGCACAAACACACAGA

AC

>4192 len=182 tot-seq=2

AGCCCAGCAGCCTCTCAGAGGGTCCTCTGCATGGGCCTGGGATTAGGGCCCCTGCCCAAC

ACCCCAGCAGCCTCTCAGAGGGTCCTCTGGACGGGACTGGGATTAGGGCCCCTCCCGGAA

CTCCAGCAGCCTCTCAGAGGGTCCTCTGCATGGGCCTGGGATTAGGGCCCCTCCCCGACA

CC

>4193 len=182 tot-seq=2

GGAAAGCAGGTCACTGTCAACATCCTGAACTCAGTCCTCCCTCACTCTGGCCCCTAAGGC

CTCTATCCCCAACTACATCCTCACCAGTCCCCAGCTTAGAGGGTGGACACCCCCTTGTAG

GGGCATGGAGATGCTTGATGAGTAGAGCACAGGCCAGATTTCAGCCCCTTTCATCCCCAG

GC

>4194 len=182 tot-seq=2

AGCTGGGAACACTCCTGCATCACCCATGAGCCCTGCCGGGGGTCTCAGTGTCCCCGTCTG

CGAAATGGGATAAGGGCTCTGCACACCAGCCTTGCTCCCAGGGGCCAGAGAGGGGAGGGA

CAGTGTCCCTAGTGACAGATCCAGAAACTGAGGCCAGGAGGCGCTGGCCTGGACGCCCCT

GC

>4195 len=183 tot-seq=2

CCATGCCCTGCGTGCCCCACTCACACAGATGCACCTAAAAGCAGAGACAGGAAAATGCAC

CAGGGCAGGGTATGAGAAGGAAAAGTCAACAGACAGCTGCTGTTCCTGGGAGAAAACAAC

TCACGCCCTTCCCTGGCTACAGGGCCGCTGCCCAGAAGACACAGTGTCTCTGCTGGAAAG

AGA

>4196 len=183 tot-seq=2

CAGGGTTGGGTTGTGGTGTGTGAACCCGAAGAAACCTTCCCTGCCTGTTGACTCTTCCCT

TGGGGTCACAATAGACGTCCTGATAAGTCCCCCAAACACCAAGAGGACCAGAGACCACTC

TGCCCTTGGAAAGACCTCAGCCTGGGAGTCTGTGAGTGACGGCACAGCCCTGGATCTCCC

CTC

>4197 len=183 tot-seq=2

CCTGTCTATTGGGACAGCTTGGTCAGCAAGGGGTGACCTTTGACCTCCAGCCCATGGAAT

GGATGGCTGCAGCCACACATGCCTTGTCTGCCCCTGCAGAGGTGAGGCCCCCCAGGGAGG

GCACAGACATGTTTTCTCTGAGTCAGACACTAGGACTGCATTCAAGGGGAAAAGACTGAA

ATG

>4198 len=183 tot-seq=2

ATCACAGCCCCAGTTGCTATGGGACACCTCTGCCCGCAGAGAGGGCAAGATGCCGAACGT

CCCTTATTGATGTGATTGCCTCCTTGGAGATGGGAATGCCCCTCTAAAAGGGCCAGGCTA

GGAGCCGACAGCACCACGGGCTCTCGGGTTTGCCATGGAACCGGGCCTGAAGCAGCCTGC

ATC

>4199 len=184 tot-seq=2

AGTGAGCAATAGGTAAGCCAGGCATAAAGCCTAGGTGTATGTTGAGGGTGGGGATGCAGT

GTGCTGAGATTGTCCCTTGACATTTAACTCAATCCTGGACTCCATTCCCTCACCAGAGGT

GTAGACAAGAGCACATACTGTGACTACAAGCTCACCTTTATAGTGCACATCCATGGGCTA

CCGC

>4200 len=184 tot-seq=2

ACTAAACATCCCAAAATACACAGGATAGCCTGATACAACGCAGAATTATCTGGCCCAAAA

CATCAATGGCATGGAGGTTGAGAAACCCTGCTGAATTACAGAGGGTGCCCGTCCTTACCC

ACTGAAACCAGGTTCCTAAAGTTCTCCAGCATCACATCTCGGTACAGCTTCCTCTGGGCA

GAGT

>4201 len=184 tot-seq=2

GGAGGAAGGTGGGGATGACGTCAAGTCATCATGACCCTTATGACCTGGGCTACACACGTG

CTACAATGGGCAGTACAACGAGAAGCGAGCCTGCGAAGGCAAGCGAATCTCTGAAAGCTG

TTCTCAGTTCGGACTGCAGTCTGCAACTCGACTGCACGAAGCTGGAATCGCTAGTAATCG

CGGA

>4202 len=184 tot-seq=2

GTTCTATACTTTCATTTTTGCACTTAATACATCTTGGAGATCTTTTTGTATCTGCAGAGA

GAAGGCTGCTTCATTCTCTGGTACAGCTTGACCATCATTTCCTTAGCCAGCTCCCAGGTA

GTGGATGTTTGTGTGGCAGGTGCTGCTGCTGCCCTGTCCCATATCTCCCACCTACCAGAG

CTCA

>4203 len=185 tot-seq=2

GTTTTTCCTGCCCCTCCCGGCTGTCAGAAGCCCCACCTGCATCTTGGTCCAGCTGACTTA

GCTCAGGCCGGAGCCTTTCCTTGACGCTGCTCCCCATCTGTTTCTCATGGGTCCAGCCTG

AAGACCAGTGGTGTCAGATGGGGAATGGGCAGGTACCCATTCAATCTGCCTGAGCCCAGC

CAATG

>4204 len=185 tot-seq=2

CAATGAATCAGACAAAAAAGACAAAAAAAAAAGAAAACTTAGAGGAAGCATGAGCAGTCA

CATGCTGAAGTACAAAACCCAAACCACACAAATGCTCCAGCTGTGTGTCACAATTCTTCA

GGGAGGCTGGGCATCCGTGGGAGCCATGGCAGCTAGCCCAGGGAGGGGACTCCTTCTTCT

CGTGG

>4205 len=185 tot-seq=2

TAGGGAATTTTTCACAATGGGCGCAAGCCTTATGAAGCAATGCCGCGTGAGTGAAGAAGG

GTTTCGGCTCGTAAAGCTCTGTTGTTGGTGAAGAAGGACAGGGGTAGTAACTGACCTTTG

TTTGACGGTAATCAATTAGAAAGTCACGGCTAACTACGTGCCAGCAGCCGGCGGTAATGC

TAAGA

>4206 len=185 tot-seq=2

AAGGGATCGGTGGAAGGTTCTGGAGTTCATGTAGGTCTTGTGTTCTACAGTTGAGTCCCC

TTCTAGGCTGTGTGCCCACAGTGTAAATACCAGCATCTATAAAACACCTCCTGTTATTCT

CACTTCCTCCTCCACCAGACAGGGGTCCGGTCAAGTCCTGATGCTCTTTGGTGTAGAGCT

GCCCT

>4207 len=185 tot-seq=2

GTTCAGACGTGTGCTCTTCCGATATAATAAGGGAGGGAGAAGTGGGGAATATTGAGAAAT

GGGGGAAAAAATGAAGAAGAGAAGAAGAGTGAGGGATGAAGGAATTAGGGTTGTAAAAAG

ATTTTGATTGGGAGAAAGACTTTTGGGTGAGTGTACCTTTCGAATAAGCGCCGGCTAACT

ACGTG

>4208 len=185 tot-seq=2

GCGATCCAGGAAGCAGTGGTGGTGGTGGGCACCCAGCCAGTGCGGGCAGTGAGGCGCCCA

GCCAGGGCCTGCCCCAGGGACAAAGAGACTCCCTGGCTCCGGGGAGAGAGCGCTGCCCCC

ACACTGAGAAGGGGGCACTGACCCAGCCCAGCCAGGCACATAGGGCAGTGGTGCCAGGCA

GCTTG

>4209 len=185 tot-seq=2

CAGTGTTTTGGAATCTCGTGTGAGCTGCAAAGATTCAGAATTTCATAGCAGTGTTCACGA

ATCCAATGTGAGGGACAAACACTCAGAACCCAGCAGCAGTGTTCTGGAATCCTATGGGAG

GTACAAACACTCAGAACCCAGCGGCAATGTTCTGTAATCCTATGTGAGGGACAAACACTC

AGAAC

>4210 len=185 tot-seq=2

GGCTGGACAGATCCACCAGGCAAGCTTCTTAGGCATGTGTATGTGTGTTTCTTGCAGGGG

AAGCAGAAGTATACACTTCCGCTGTACCACGCAATGATGGGTGGCAGTGAGGTGGCCCAG

ACCCTCGCCAAGGAGACTTTTGCATCCACCGCCTCCCAGCTCCACAGCAATGTTGTCAAC

TATGT

>4211 len=186 tot-seq=2

CAAATTAGGAAAAGGGGGTCTTAGACGCGTGTCACAGTGTAGCGATTCCAGAGAGGATAA

CTCAGGTGGCTGAAAGGCGGCTTTCTTTCAAGTTCTCAATGAGATAGGCTAGGTGATGTA

ATATCCAAAATGAATTCAAGACCACTCCGTACCTTCCCCTTTTGAGAAAGGACTTTCCAA

ACTGTA

>4212 len=187 tot-seq=2

TGGGGAATCTTGGACAATGGGCGAAAGCCCGATCCAGCAATATCGCGTGAGTGAAGAAGG

GCAATGCCGCTTGTAAAGCTCTTTCGTCGAGTGCGCGATCATGACAGGACTCGAGGAAGA

AGCCCCGGCTAACTCCGTGCCAGCACCGCGGTAATATAAGATCGGAAGAGCGCGTGTAGG

GAAAGAG

>4213 len=187 tot-seq=2

CTTCATCAGGTGGGAGCCCTGCACAGCTGTGCCCTAAGGACTTCTCATACCCACAGTCCT

TTCTCCCTCAATGGCTGCTTGGCTAAGCACCACATCTGCTTCAACTCCCCTGAGCAGGGA

TGTCTCAGGACTTCCTCCCTTGGGGGAGGCTGCCATGACCCACACCCTTTCAGGACCTGT

GCCCAAG

>4214 len=187 tot-seq=2

CATTGCAGGATGGCCCCACCATCCACCCAGGAGCCTGGCCTCCCTCTCTGGGTACCCCTC

ACCCCCAAAACCTGTCCGTCATGAGCCCTGTCCCTCCCCACCTGCCTCCCCTTGACATAT

CCCCTCCTCTGTGTCCCTGTAGCCTAGAACTGCTTAGGCCTCATCCTCCCCTTCCTGGAT

CCATGCC

>4215 len=188 tot-seq=2

GAATGGCTGCTGGTCTGGGACTAAGTTACGGTTCTCACGACCTAGTGACAATGTAAACTC

GCTACTGGATGTGGCATGTGCCAGGGCTCTCTGAAATTGTAGGCTAAACACCTGGCTTTG

ATCAAAGCCAACAGGAAAACAGTAGGTCACTATTGCTGTCATCCCACAACAATAAGATAT

ACAAATCC

>4216 len=188 tot-seq=2

AAACTCTTTATGGAAGATAACTCCTAGAATCTTCCAGGCAACCCAGTGAGATAGTTATCA

TTATAAATCCTCCCGCCCCATTTTACAGATGAGGAAACTGAGGCTCAGAGAGGGGAAGAC

ACTAGCCCAAGGCTGCACAGGGATGGAGTCTGGTTTTGTCTTCAAGGCCTTGGCTCTTAG

CCATGATG

>4217 len=188 tot-seq=2

GAGGCATGAGACTGGACCCCATCAGTGGGCATCCTGACAGTGGTGGATGGCACGGCATGA

ATAACACATGAAGCCTCCATAATTGGTGTTATTACACATGCATTTCATCTCCATTCATAA

TGATTGGACACTTAAACCCAGCCGCCGTTCCTCTGCCGAGTGCTGCTGTCATCCTTCTAG

CCGAGATC

>4218 len=188 tot-seq=2

CCTACAGCGGAAAGCATGCAGACAAGTGGTTCTGTGAATTATCTGAAGCAAATGCATTGT

ACTTCACAGACTCTGTAGTAGAAAACCACCAGTGTGAATTTAGTGGGTCACGCGCTTTGA

TCAGGGAGAATGAGTTCGCAGCAGTTCATCCATCCACGCCGCCCAGCCTGACTCCCTTTC

ATGCCATC

>4219 len=188 tot-seq=2

AAATGATGTTCTGGGATTTTTGAGCCTATACCTTAAGCAGCCTTGTAGCTTCTGCTTCTG
CTTTTGGGACCCAGCAATTATGTAAAAGCTACTCTACTGGAAAGAGAAGCCAAGTGAGGA
GAGAGGCCCCTGAGGATGGGAGACCATAAAAGAGAGAGGACACACAGAGAAGAACAGAGT
ACGTACCA
>4220 len=189 tot-seq=2
TGAGATGGGTCCTATCTTTCCTGGGGGAGCTGGTTACAATATTCAGCAAGGCAACCTCTG
AAGAACAAACATGTCTTGCTTATGCAGCAAGCAGAGACCTGGCCCTGATAGAGCCAAAGA
GGATGTTTGGAGTAGGAGAGGGACAAAGAAGATAATAAAGAGACCTGTAGGTACATATGA
ACAGAAACA
>4221 len=190 tot-seq=2
CGTGGGGTGGGTCTACCTAATTTCTCAAGGGTTGAGGGCAAGTGCTGGCCGAGGCCTAGG
CTCCATAGAAGCCAGCGGTGCAGGGCCACTAGGAATCCAACTGGAGCCACCGGGGCCAAA
ACCCTCTGTAGACTCTGGCAAGAGGTAAGTTGCTGGTAAGAACTTCCCTTGAGGGGTGGC
AGACTGGAAG
>4222 len=190 tot-seq=2
GGAGAGAGAGAGAGAGAGAGAGAGAGAGAGAGAGAGAGAGAGAGCAAGTGAAGGGAGTCAAC
ACAAAACAACCCCACACAGAGCTGTAGGCCTGGCCTCTGCTCTTCCAAATGTCTCCTGAC
TCAAGTGTAGAGTGGGTGAAGTCACAGGCCCCTTACCTCCCAGCCCTCACGGAGTGTAGC
TCTCAGGGTT
>4223 len=190 tot-seq=2
TGGGGAATATTGCACAATGGGGGAAACCCTGATGCAGCAACGCCGCGTGGAGGAAGAAGG
TCTTCGGATTGTAAACTCCTGTTGTTGAGGAAGATAATGACGGTACTCAACAAGGAAGTG
ACGGCTAACTACGTGCCAGCAGCCGCGCGGTAATTTATAGATCGGAAGAGCGTCGTGTAG
GGAAAGGCCA
>4224 len=190 tot-seq=2
GGAGACCAAGTTAGATTATGCATAAAAACTTTTAAAAGCATTTGTATTTCTGATGCAGCT
TCTGGGCACTACAAACTCAACTAAGAAACGAAAAGACAAAACATAGATAGTCTCCATGCT
CAGACAGTTTAAATGCTTCATGAAAAAGAATACCCAAGTGTCAGCCACAGTACAGCGCAC
ACCCAAAGTG

>4225 len=190 tot-seq=2

CTCTTCCGATCTCCTACGGGAGGCATCAGTAGGGAATCTTCGGCAATGGACGGAATTCTT

GCCGAGCAACGCCTCTTGAGTGAAGAAGGTTTTCGGATCGTAAAGCTCTGTTGTTAGAGA

AGAACGTTGGTAGGAGTGGAAAATCTACCAAGTGACTGTAACTAACCAGAAAGTGACGGC

TAACTACGTG

>4226 len=190 tot-seq=2

TGCCCCTGAGCCACGTCCACACCCACTGCCGTGCACCTTTGCAGTAGCCCCACTCTGACC

CCGGCCTCTGCCCTGGAGCTTGCTTTGGTCGATGGGACACAGTGGGTATGATGCAGCACT

GGCATGAGAAGTGCTTGCACATCCCTGCTCGTCCCTCCTGTTTCCCTGGACCTTTGTCCT

GAAGACATGC

>4227 len=191 tot-seq=2

AGTCCTCAAAGTCTGAGGGCACAGGTCCCAGTGCTGTGACTGATCCACACATTCCTGAGG

CATTTAAAAGATACTACTGAACTAAAACAGCCATCATCCACCATCGCCTCCCACCCCCAA

AAAACCAACAGGATTTGGAAGCCTCAGGAGGCTGAGAGCCTTCCCTTTCGAAGATGCAAA

GCTAAATGTTG

>4228 len=191 tot-seq=2

GACCCTGGACAGTGGGCTTCAGGTACACGCAGCAGGGTTCTGGACAATGGGCTTTAGGGT

TCTGGAGGCCAGTGCAAGGCCCGTCCAGCTGCCATCTATTCCTTCCACGAACCTCTGTTT

ATTCAGCACAGACCATATTCTGCCTGCCAGGCACTGCGCTGGCGCAGCTGGTGTCAAAGC

ACGCTCCCAGA

>4229 len=191 tot-seq=2

GGCAGGGGGGGGTCCACAGGCCACTGGATGCCCACATTAAGCCTGTCAAACCTTTGAAGA

ACAAGTTTGGGGATTTGGGCAGCCTTTGCTATGTGACATTTAGGAAGAAAAATACCAGCT

TTTGAATTACTGCAGACGACTGGGGGCCTAGGCAGTATGCAGTCTATACATTCATAAGGA

TTCCTGTACCC

>4230 len=191 tot-seq=2

CTGGGAGGGGCACTGCTTCCCTGGCCTCCCCAGCGGGAGTCGGCTCCAGTGCCCCAGCAG

TAACTGTATCAATGACATGCCCTTTATCACTTCCTTCTCTTTCTCACCTCATTTCCTTAT

GTCCTGGGATCACCTCCCAGGAAACCACTTGTGCTGGAACTTTTGTCTCCTGGGCCTTCC

TCATAGGGTCT

>4231 len=191 tot-seq=2

TTTTTTTTTTTAACTTAAGAGACTTATACAAAGTATGAGACCTACCAAAGATCTCATCCA

TGGGCACTGGAAGCGCTAGGTGATAGAAGAAACTTAAACAGAAAACGACAGCAAATGTTA

AAATCTTTTTCCAGTCCCACCATGTTATTTCACATACCGATTATACCATTACTAAATATG

GCCTCCCCTTC

>4232 len=191 tot-seq=2

GAACACCTTTACCAAATCTCCTCCCAGCCTTCGCAATGCCGAGGAAAACAGTCTCAACTT

CTCCAATCTGTCTACGCAACTTAAGTAAATCACGAGCGACAAATTGTGTCCTTAATCTGA

TCGCTTGGGAGCAAAGCTGGGGAGAGAGGGTTTTCATAAAGAATTTGTGATTTCTGTAGC

ATCTTTCATCA

>4233 len=192 tot-seq=2

CTGGAACACAGCAGGGCAAGGGCTCGGCTGTTAACTGGTGTAAACCAGTGTGAGACCGAA

GGATCTTCTCAGTGAGTTGTCTCAGAGAGTCCCCAGACCTGTGAGGCCCATTCTGTTGCC

AGGTGAACAGGTAAGCAAATGTGCTACATCCATAAGATGGAGTAATAGTTGACCAGGAAA

ATGAACCGACTG

>4234 len=192 tot-seq=2

ACCCGCACAAGCGGTGGAGCATGTGGTTTAATTCGAAGCAACGCGAAGAACCTTACCAAG

TCTTGACATCCCTCTGACCGACTCTTAACCGAGTCTTTCCTTCGGGACAGAGGAGACAGG

TGGTGCATGGTTGTCGTCAGCTCGTGTCGTGAGATGTTGGGTTAAGTCCCGCAACGAGCG

AAACCCCTATCC

>4235 len=192 tot-seq=2

CGCCAGTGCCACAATAGCAAATGTGCGTGCACACGGCTGACCGGAGCAATCCTGGGAGGA

TCTTTCTGGCTGAGTGACAAATGACATCCCAGAGATTATAAAGCTTGACCCTGGACACAG

CAAGGTGGCTTCTGCAGGCAACAGAGCCAATGCCATCCCTGTTCTCCCCACCCCTGGCTG

CAAAGCCCTTTC

>4236 len=192 tot-seq=2

GAAAAAAAAAAAAATGCAGAGGAACAATTCCTCTAGCCCTGGAAAATTCGTTTGAATTGC

AAGCTTAAAATGAACATAATAGGAGTGAAACCCAAATCCTCTGGCTTTCACCAGAACAGC

CTCACAGGGAAACAAATTTAACTGTGTTCCAAATTGGGCCAGGCTCCCACTATTCCACTG

CAGGTGACAGTA

>4237 len=192 tot-seq=2

CAGGGTTTGAGAGGATTGACTCCAATTTTGAAAAAATTCTACCGTGAGTAAAATATAATC

AAATAGCACCATATGCTACAGAGAAATCTTTCATGAAAGGAGGAGTCAAATGATGGGGCC

AACTTTGTCTTCGTTTTATTGTATGGAACCACTATAGTCACCCCAATCTTCAGCAACCAC

TGCCCTGGTCAG

>4238 len=192 tot-seq=2

CTCTCAGCCTAGCTATCTGCTGTGTGATCCCAAGGCCTCTTCAAAACCTCTATGTGCCCA

TAACTGTCTAGCGGGCACCCAGATGTCTGGCCTGGCTGCAGAAAGGGCAGGAGAAGGGAA

GGAGATGAGACCTCCACTCCAGCCCCCCAGCCCCCACCAAAGCCCTGGCATCACTGTCAG

CCCACAGAGAGG

>4239 len=193 tot-seq=2

GCTCACCTATGGACTGGTTGACGGCCTTCATGCACAGGTTCTCCACCAGAGCCTTCCCTG

GGGGTGCCTGGCCGGGGGCTCTGGGCTGGGAGTGAAGGGGATGACCCATCAGGACTGTTC

CCTGCCTGCAGCTCCCCCGGGGAAAAGACCCTGCCAGGCTTTGCAGCCAGACCAGGAGGC

TTTGTGGCCAATG

>4240 len=193 tot-seq=2

GTTGAGAGCTGCGAGCCCAGGCCTGTTCTTCTGCACCCTGGTTCGCTCTGGTGCATGTGG

CCAGCTCCCAAGGACAAGAGGGAACCATCGGGCCTGAGGGCCCGTGACCTCTGATTTCTT

AAACACGGAGCCGAAGACCAGGTCTGGCATCAGCCAAGGATTCACGGTCCTTTCTCGCTG

TGCCTGGACCCTC

>4241 len=193 tot-seq=2

ATCTGAGTGGTCCCCTGGCTCAGGGCCAAAATGAGCAAACAGGTCCAAGGGAGTTTTGCC

TTCCATGCCTTTTTCTTTCCAGGTGCCCCCACTGGGAGGAGCAGGAGAGTGGGGAGTTCC

TGGGAGGGAAGGTTCAGGGCTCCCAAAACCATTCTGCATTCGATGAGGCCCCACAGGCCC

TTCTTTAGTTACC

>4242 len=194 tot-seq=2

AATATTGTTGCTATCATCCAAACACACAAATCTGTGAGAGCCATACCTATTCAAACCACC

ACAGGGATATTGTTCAAGACCATAGAAAACATGAAAGCCAGGACTGGAATAATACTAAGA

GCCATATTTTTGAAGCATGACCATTTGCCAGACTTTGTTCTAAGAGCATTAAGGACATCG

CATCATTAAATTTC

>4243 len=194 tot-seq=2

GTAAGGCGTTAGGTCCATTTGCAGTCTCTGAGCTTGAGGGAGGTGAGCTTGGTCCATCTT

TGTCCTCATGCAAAGTCCTGCTGCAAGTAAACCTCCCTGGGCAAGACAGAAGGGGAGAGG

TGAATGCCTCTGAAATGAAGGCTTGGCTCTGAGAAATCCCTGAACACGTCAGAGGGTAAA

GGGCTGGCGTTTAT

>4244 len=194 tot-seq=2

AACTTGCTTGAGTCTGGAAAAGGCAGGAGAGTGTCTCAGACACACGGATGTCCTTGGTTC

TCATACCTGTAACCTACTTTTGTCGAAAAGAAAACTAACAGCCCCCTATCAAGCCAGGGA

CTTGGGCTGAGTCCTGGTGTCCAGCCCTCCCTGGAGTAGGAACATGTCCAGGAAATAAGC

GCCATACCTCTGGC

>4245 len=194 tot-seq=2

GATGTGAATGATGAAATGATAAATAGGGGCTGAGTGGCTGAGTGGTCAAGGCTCAGCCTT

CTGTAACCACAACATCAAGGGATGCCTAAAAATAGTAGAAAGAGCCCAGGGAGGCGGGAT

TAATCATCTCCAGCTACTTGTGAGAGTACTTGGAACTGTACAGTGCCTCTCAGCTTCAGA

GAAGTCACAGGGAG

>4246 len=194 tot-seq=2

GGACTGGCTGGCTGACCTTGTGAAAGTAGCTTCACCTCTCTGGGCCCAAGCCTGCCCATC

CATCCAGGGGAGGTGCACATGCTTCCCTCAGGGGTTTAGGGTGGAGGCTCAGTGCGACTT

TGTGGGCAAAGTGCCTGGCACATGGGCCTGGGATTCCTGACATAAGAGCCACTTGTGGCC

TTGCCTCAGTGTCC

>4247 len=194 tot-seq=2

CCTCAGCCTTCCAAAGTGCTGGGATTACAGGCGTGAGCCACCGCGCCCGGACATAGAGGG

TATTTTTAAATGCTTTGAAATTCTCAAAGAGAAAAGGAAGAAAAAAAGTCTCAATAGGAA

AAATGTGACAAACGGAATCCCGGCGACCTGTTAATGGTTAATTCTCACAACAGTGTCTCA

GTGCATATCCTTGG

>4248 len=195 tot-seq=2

TAGATGTGCTACAAAATTCTGAGAAGAGCCCCTTAGCATAAAAAATTAAGGCCTAGGGCA

CTGCAGTCAGTGGAGATTTGCAAAAGATCTTTAAAAGGTGATGTATTCCTAGATCCTTTT

TCATTCCAGTTTTAGGGTGCCGTAACTTTACTTAAGGAAAGCAATAATTATTCATAGTGG

GGAAGTACCAGAGAA

>4249 len=195 tot-seq=2

TTTTTTCCTGGTTTTCTTTCCAAATCCTGGTTGGGTAATTTCTTTATCAAACAGATAAGG

GTTCAGACGGTCGAAGGTCCCAAACACACAATTCAATGGTAAGTAACAAAGCAGCCATGT

GGACTGAACCCACATGTGTTTTTGATGCCTGAAGTGAAGAGACTCTTAAGATAAATGAGA

GGAGACTCTAAGAAA

>4250 len=195 tot-seq=2

CAGCAGTTGCAGGGCAGGGTCAGGGGATTCCTCAGCCGGAACCTCTGGCACTTCCCCTTC

TCTGAGTTCATCCTCCCACGGTGCTCTTTAGCATTCTGTCTTGATCAGGGTACGGTTCTC

TTAGCCCTTGGCTCTGGCTTTCACCAGGACCCTGTTTGTTTTTCTTTCTCAGATGTGGGA

TGGGGAAGGACAAGA

>4251 len=195 tot-seq=2

ATTGTCTAGTGACTGGAGTTCAGACGTGTGCTCTTAAGATATAATAAGGGAGGAAGAAGG

AGAGAAAATTAAAAAATAAAGAAAAGGGGAGGTAGTGAAGAAAAATAAAAATAAAGGAAT

ATTTAGAGGAAATGTAATTGGAATGAGTCAACTTTAAATCCTTTAACGAGGATCCATTGG

AGGGCAAGTCCGGTG

>4252 len=195 tot-seq=2

GCACACAGAGCCCTGTAACCTGACGGAGCAGACTGCTCTTGCACAAAGGGCCCTGCAACC

TGAGCCTGTTGACTTTGGGGAGGAGCCCAAGGGAGCCGAGGCCTCTGGGCGCACACACTG

CCGGCAGGGGTTAAAAGCAAGCTGGCCCGGATTTGAAGCGTGTGGAAGCCTGACCTGAGA

TACGGGGAGAAGACA

>4253 len=195 tot-seq=2

GAGGGCTAGAGAGGATTGACTCCAATGTTGAAAGAAATTCTGCACTAGGTAAAATGTGAT

CAAACAGCATCTCATCCTACAGATAAATCTTTTGTGAAAGGAAGAGGGATCAATGTGACA

AATTCCATTGTTGTCTCTAAGAAACTGCCACAGGCATGCCAATCTTCAACAACTAACACC

CAGATCAGTCAGCAG

>4254 len=196 tot-seq=2

GAGGTGGGAGAGGGCAAGACCCTGGCCACAGAAAGAATTAGGGCCTTTGTATCTGGGCAA

ACACAACACAGATGATAACTCTTGAAATTCCCCCATGATGCCTCATCCCTGAGTGAGGTT

TAGGCGGAGTAAGGGGTACTGGAGCTCCTCCTCCCTACCCTACCATTTGGGCATCCCCAT

GAAGTCTGTCTCACCG

>4255 len=196 tot-seq=2

CAAAGAGGTGGGAGGGTAGCCATGGGTATGTGCCAACGAAGTAGCGCAGGGAAGACTATG

GTGGGAAGAAGGTGCAAGTGGACTAATGCTCATGGGCAGGGGCCCCTCTGCTGGGGCTCT

CAGGCAGGAGCAGTCTGCCAGCGAAGGAGCTATGATGAGGGCCCGAGGAAGCACCCTGTT

TAGGCAAGCACCAGGT

>4256 len=196 tot-seq=2

CAGGGGATAAACACACTGTCCTCCCCTTGCTGGTCTCCCCCAGGGCCTCCAGGTGGAGGG

ACGGGTGGCACAGTTCAACCGCCCCCACCCCTCCGCCATCCCCCTTCTGCCCCATGTGGT

GTATGCACAGGATGGGGCAGAGCTGAGCAAGCACAGCTCCTAAAGAGGAGGGCAGGAAAC

TTGCTGCTCTTACAGC

>4257 len=196 tot-seq=2

CTGTAATTAGCCCCACTTTGCAGATGAGTTGAGCATTTTACCCAAGTCACTCTGTTTCTA

AATGACAGAGAGATTTGAACCTGTCTGGACTTTTAAGTCCCTGCTCTCACACTGCCCTGG

GAATCCAGCATCAGTGGGAACTGGAAAATGAGTTTTCAGATCCCTGGAGCCCACAGACAA

TCCCCACTTGGCAAGC

>4258 len=197 tot-seq=2

TATGAACGGGAGAGAAGGGTGCACAGTGAAAACACATCAAAGGATGGAATAGTGGAATCA

AGATAAACACCTATGCACAAGGAAAGAATTATCTATCGACTGCCTTGCGGATGTCAAACA

CTGTCATAGCTGGCTGTGCTGTTAGAAACAGGCTGGGATGTAGCTGTCCCAACTCTTCTC

TCCTGCAAGCTTCCACA

>4259 len=197 tot-seq=2

CCAGCAGGGTGTAAGGTAAGACACTTCCAGGTACCCTGTGGGGAGCTGAACTGAACTCAG

GACACTGCCCCCCTACCCTAGTCCACCTAGGGTGTCCTCATACATGCCTTCTGCAGACCA

CCAAAGCATCTGAGCTCGTTTCCTCCAGACAGGTTTTCAAGTCCCTAACCTTCAGCACCA

GCGTCCCCTCCACCATG

>4260 len=197 tot-seq=2

CAGGAGGATTAGAAATTTAAGATTACATTCAGCTATATGAGATGCTATCTAATAAAAAAT

ATGAAACACCCAAAAGCAAGGGACTCTTCAATTAGCGAAGTCAGAGGTGCTGACAAAGGT

GAGCAAATGATTTTTTCACGTTTACTGGAAAAAAATAATTAGGGATAAACTGAAAGAGGA

TGCAGAAGCAGAGGAGA

>4261 len=197 tot-seq=2

CTGGCTGGCTCCCAGGATTGTAACTTTTAAGTTTTTCAAAGTCTAATTTTAATGATGGTT

CTTAAATGCTTTAAACCTCCCTTTTAACCCACCAGTGGTGGGAAAGAAAGGATCCAGGGG

AAGTGGACCTGCTTAGAAATGGTTCTTGGAGCAAATTCTATCTGCGTTGTCAGGAAATCG

GCAGTTGTTCACAGGAG

>4262 len=197 tot-seq=2

CTGAGTGACTGGAGTTAAGAAGTGTGATATTAAGATATAATACGGGAGGAAGAAGTGGGG

AATATTGAGAAATGGGGGAAAAAATGAAGAAGCGACGACGCGTGCGGGATGAAGGCCTTC

GGGTTGTAAACCGCTTTTGATTGGGAGCAAGCCTTCGGGTGAGTGTACCTTTCGAATAAG

CGCCGGCTAACTACGTG

>4263 len=197 tot-seq=2

CTGAGTGAATGGAGTTAAGAAGTGTGATATTAAGATATAATAAGGGAGGAAGAAGTGGGG

AATATTGAGAAATGGGGGAAAAGATGAAGAAGAGAAGAAGAGTGAGGGATGAAGGCCTTC

GGGTTGTAAACCGCTTTTGATTGGGAGCAAGCCTTCGGGTGAGTGTACCTTTCGAATAAG

CGCCGGCTAACTACGTG

>4264 len=197 tot-seq=2

GACGTGTGATATTAAGATATAATAAGGGAGGAAGAAGTAGGGAATATTAAGAAATGGGAG

AAAGAATGAAGGAGAAAAGAAGAGTGAGTGATGAAGGTCTTCGGATCGTAAAACTCTGTT

ATTAGGGAAGAACATATGTGTAAGTAACTGTGCACATCTTGACGGTACCTAATCAGAAAG

CCACGGCTAACTACGTG

>4265 len=197 tot-seq=2

ATTCATGTGTGACTGGAGTTCAGACGTGTGCTCTTCCGATATAATAAGGGAGGAAGAAGT

GGGGAATATTGGAAAATGGAGGGAAATATGATAAAGAAATTATGTGTGTGTGAAGAAGGT

TTTAGGAATGTAAAAAAATTTTAGTAGGGAAGAAAAAAATGACGGTACCTACAGAAGAAG

CAACGGCTAAATACGTG

>4266 len=197 tot-seq=2

TAGGGAATTTTTCGCAATGGGCGCAAGCCTTATGAAGCAATGCCGCGTGAACGATGAAGG

TCTTATAGATTGTAAAGTTCTTTTATATGGGAAGAAACGCTAAAATAGGAAATGATTTTA

GTTTGACTGTACCATTTGAATAAGTATCGGCTAACTATGTGCCAGCCAGCCGCGGTAATT

AGTAGATCGGAATAGGG

>4267 len=197 tot-seq=2

GTGGCCAACATGCAGGAGATAGGAGTGACTCCTTTTACAAGTGCCCAAAGTCAACAGCTG

ATGACGAAGCCAATTTTTATGGCCATGCTCTTACTAATGTCTTCTGTCAAATCACTTATC

TCCAAGAAAGTAAGACAGGAGGTAAAAGAGGACAGGTCGTAGGGTCTGTGTAACCCACTC

AATCCAAGCTTCCAGCT

>4268 len=197 tot-seq=2

TTCTTCAAAAAGAACTTGACTGGAGCACAGAACAGTTCTGATATTAGTTTGGAAAAAAAT

GTGGCTCCAACATTTACAAATGATATTTGGAAAAGGCATCTCATCTCCTAGGTCCTGTAC

AAGAAGGGGTGTGGGGAACACAGGTAGAGGCATCCAAGCTCAGAGATGCTACAGTGGTGG

CGCTGTCCACATCTACC

>4269 len=198 tot-seq=2

GAAGAGAGAGTCACGGGACCTGGAGTGGGCTTCTGAAACCTCAAAGCCCACCTTCAGTAA

CACACTTCCTCCAGTAAGACCACACCCCCTAATAGTGCCACCCCCTGGTGACTAAACATT

CAAATCTATGAGCCTACCTGGTCCATTCCTATTCAAACCACCATATATGCCTTGGGAGAT

AGAGGCAAAATGATCTGA

>4270 len=198 tot-seq=2

AAATTACCAGTGTACACGGAAGATGGTCAGACACATCAAATGACACCATAGCATGCAATC

AGCTAAACCCAGAATGCCAAAAATTCCAAGGGACAAATAATCCAGTTTCTCCGAAAAATT

AAATGACAAAAAGCAAAAAAAAAAAAAAAAAAGAGGAGAGGGAACCTATGGATAAAAGGGG

ACTTAAAAGATATATCAG

>4271 len=198 tot-seq=2

CTGTAACAGGCCGTGTGGAACTGAGACGTGACCTGTGGAGCTCTGTTCCCTACTACTGTT

TCACCTTTGTGACATGATGTGCCATGACAAGCGACCAGTCAGGGAGAGTACAGCTACCCC
AGTTCTGAAAGGAGGGTGGTGACTCTGGCCCACAATCAAGAGATGCCAAGGAGTGTGTGG
GCTCCCATGGCAAACACG
>4272 len=199 tot-seq=2
TGAAAGCATTTTCTCTACATACAAGCCACCAGGGCCCATTTAATGTAATTAATTGTCTTT
TTCAGTTCCGTGAATAGTACCCCAGAGATCTGCAGGAGTTAATGGACTTTCAGGAAGTAG
GCTCTGCTCCCTGGCTCCCACCTGGCCCAAAGTCATTCTTGCCACTCTCATTCCAGGGTG
TCTTTACTCATCTAGGACT
>4273 len=199 tot-seq=2
AGACGTGTGATATTAAGATATAATAAGGGAGGAAGAAGTAGGGAATATTAGGAAATGGGG
GAAAAAATGAAAGAGAAAAGAAGAGTGAGTGAAGAAGGTTTTAGGATAGTAAAGCTCTGT
TGTAAGAGAAGAACGTGTGTGAGAGTGGAAAGTTCACACAGTGACGGTAACTTACCAGAA
AGGGACGGCTAACTACGTG
>4274 len=199 tot-seq=2
AGGAGCAATGCGAGTGACGCCAAGAGGCTTCCGGAGCTGTTGACTCTTATGTGAATCATG
AGCCCACTGTCCATTCACACACGTATCTCTTGAAGGAAGAGTCCAGCGTCTTCCTCATGC
TACCCATGGTGGGCAAAGCAACATGGTTTCTACCATCATGGAGTTTATATTCCAGTAGGG
AAGACAGGCCAGTTCCATC
>4275 len=200 tot-seq=2
GGGAAGCAGTGTGATTTCTGGGCAAGAAACTGCCAGTGAGCGTCTTGCTCACTTGTGACT
TGCCTTCTCTAGAACTAGCACACTGTAAAGGAAGGGACCGCTACGCCACCTCCTCCAAGA
GCTGAGTGCCCCAGTGGCTCTAGCTCAGCCCGTCCTCTGCCCACGTCCAGCAGGGAGGTC
CTGGCTCTCTAGGTGCTCTG
>4276 len=201 tot-seq=2
TATCTGGGGTGTCCAAAAGCTTGAGAATCTGTCATCCTCGGCCTTTGTGCAGGCTGGGAC
CCCTGAGCCAACTCCAGCAGTTCCTTGGGGACCACAGACTGTGCTTCCCCACCCCTGGAG
TGGGGTTCTGTGGCTTCTCATAGCCTGATGCCTGCCTGACATGAGGAAGGAGCAGTCAAA
ACTTCTCAAATTCCACCTCAT
>4277 len=201 tot-seq=2

AGAAACTTGCCTCCAGATGTGTTAATATGATTATTAGGTAGAAACAGAAATGTAGAGTTA
TTTACTGTGTAAAAATGTGTGTCACTCCAAGAGTCTCCCAAGAACTAGAGACCTCAGAGG
ACTTCTTCTGGAAAAGCCCAGTGAGTCTTAAAGGCTGGGCAGAGAGATGCTCAGCAGGAC
CACCTCCCTATTACACTCTTC
>4278 len=201 tot-seq=2
TGGAAGTCCAGGGTCCAGAGGGTCAGACGAGGAGAAGGGATTCTGGAGTGGCCTCCATAG
GAAGGGCCCTGGGAGGAAGCTGGTCCGCGTGCAGGTGTGTTACTCACTGCTGCAGAAGCA
ACTTGCTGAAGGGGTTGGCCCCTGACGCGGAGGTTTCGGAGCTCTAGAAGGCCCAGCACC
TGCAGCCTGGGGGCTCACTGC
>4279 len=201 tot-seq=2
AATAGCTGCAGCACTGTCAGTGAGCTCCAGTGTCTAGCACCGGCCCTTCTGTCACATTCC
TCCTTCTGATCACCCACCAACTTTGTCATTTGAGTCTTTGGGGTCTAGGGAACCTCACTC
TGAAGGGGGGCAGCCTTGTCATCATTTATACATACCCCATTCATCCTCTCTGGGTAAAAA
TCCAAAACCAGAAAAGAAAAG
>4280 len=201 tot-seq=2
TAGGGAATTTTTCACAATGGGCGCAAGCCTTATGAAGCAATGCCGCGTGAACGATGAAGG
TCTTATAGATTGTAAAGTTCTTTTATATGGGAAGAAACGCTAAGATAGGAAATGATTTTA
GTTTGACTGTACCATTTGAATAAGTATCGGCTAACTATGTGCCAGCAGCCGCGGTATCGG
CAGATCGGAATAGGGAATTTT
>4281 len=201 tot-seq=2
GACATTCGGTTTGCCCTCACCTTTTGACGGACATTTGGTTTGCTCTCACCTTTCGGCTAC
TGGGGATTATGCTGCTATGTGTGCACTGGGGTGTGGGTCTGTGAAGTCTCTGCTTCAGTT
CTTTGGTGATGTTCCTGGGAGTGGAATGGCTGGGTCATGTGGAGATTCTGTTTAATTCTC
TGGGAAATGCCACGTTGTTTC
>4282 len=202 tot-seq=2
TGCAGTTAAATCCTGGGCAGGAGCAAAGCCTCGAGGCCCCCAGTTTTAACCCTTACTTTG
TATCCCAATGCAGAAACCACCAGCTTGCCCCCAGCTGGCTGCCTGGTTCCCACCTTTCTG
ATTGATGGTGGCTAAGCAGTCACATGATAGGGCAGTCTAAAGAGTATAAACTCTGTTGGC
ACAATGTCTCACACCTGTAATC

>4283 len=202 tot-seq=2

GGGCAGACGGGGGAGAGGAAGAGAGAAGAAGGGAGGAAGAAGGAGGGAAGAGGAAAAAAG

GGAAGAAAGGAGGAGGGAGAAAAGAAGAGGGAGGGAAGAAGGGGGGAGGCGCGTAAAGCG

CTGGTGGGGGGGAAGAAGGACAGGGGGAGGAACGGACCGGGGGGGGACGGGAAGCAAGGA

GAAAGGCACGGCTAACTACGTG

>4284 len=202 tot-seq=2

CAAATACTAAGTCATGACGGGTAGGAGCTTGGATTCCAGATTTTCCAAGGGCACTAGGGG

ATCCAGGGGCATAAGCAGCAGTGTATGTGTGCAGTCCTGGAGATTCGGGAACTGACAATG

CAGTGGCCAGCAGTGGGCAAGTAGTGGTCTGCAGTCAGTGCAATGATTAAGAGTTTAAAA

GGAGGCAGACAATGGCCCAAAC

>4285 len=203 tot-seq=2

TCTCAGCCTATCCACAGACAGTGGTCATACATGGACTTCGAAAAGAGACTGGAGAGGTCA

ACGAAGGGATGGTCACCCATAATGCCATCCTGGGTCTTGACTTCTCCTGGACCAGGTCTC

CAGGAAAGCGCTAAACTCTGCACTCCACAGAGAAGCAAGAAAAGGCTCTGAAGGAACCTT

AACTCGACACACAACTACTAATC

>4286 len=203 tot-seq=2

AAGCCCATAGACTTCGAGGTACAGGTAGGGGGCTGGGAAGATAGCCTGGGAGCTGCAGTT

GGCACCAGGGGTCCAGTGGTTCCACCCCAGAGCGGGCAGACTGGCAAAGAGCAGGGGACC

AGCCCAGGTGAGGAGCAGGGCCAGCCGAATGCTCCCAGGGGGCTGAAGTGGCCTCAGGAC

TGCCATGTAGCGCTCCCCGTGCA

>4287 len=203 tot-seq=2

TGAAAACGGCCATGTTGCTACTTAAATGCTAAACCTTTAATGGTGTATTAATAGACAGTT

ATTAAGTTGCAAATTAGGAGCTTAAATCTACATAAATCAGCTGTGTACTGACTGTATCTC

ACAGTAACAAATTCACCCAATCTGTATCTTGTCTTCTTTGTAACAGTGTTAAATATTAGC

ATATCTCGCTCTCAGGTGTGATT

>4288 len=203 tot-seq=2

CTTACTAGTGAACTCTGTCAAATGTTTAAAGAAGAACCAATTCTTCACAAATTCTTCCAA

AATGTAAAAGAGGAGAAAACACTTCCCAAATTATTCTATAAGGCCTGATGCCAAAATCAA

AGACAACACAAGAAAAAAACAAGACTATTATGTGTTTTGAATATAAATGCAACAGTTCTC

AATAAAATATTAGCAAACTGGAT

>4289 len=204 tot-seq=2

GTGGCCGTCAGGGGCCGGAGCTCCAGCAGGTGGGCAGGGCCCAGCCCGGCGGCAAGGGAG

CAGAAGCCAGAGGTGGCGAGATTTGGACAAAGTTCCTGAGGCCCTTGGTGACAGAGGATT

CAGTGACAAGAGAACGGAGCCCAGAAGAAGAGGGGAACATCCCACAGAGACCCCCTCCTG

AGTAAATGAGAGAATCAGACCAGA

>4290 len=204 tot-seq=2

CTAGAGGGAGAGTGGAATCAAGGGAGGGTGGTTTTAAGATGGAAGAAATGATACTGTTTT

TGTGTATGTGAGAGTAATCTAGTAGAGAGACGGAAATGGGTGGTGTGGGAAAGATGAGAG

AATTGCTTGAGCAGTCCTTGAGCAACGTGAGAGGATAAGGCCTCACGCCTAAGAGGGGAG

ATACTCGACTGGCCATCGAGAGGA

>4291 len=204 tot-seq=2

AGAGGCTGACCACGGAGGAGCAGGAGAAGGTGGCCGTGCCAGAGGGGGAGAAAGTAGAGA

CCCCCGGAGTGGATAAGGAGGGTGAAAAAGAAGAGCCGCAGTCGGAGAAGGTGTCAAAGC

AAGGGCTGCCAGCTGAGAAGACACCTTCACTCTGTCCAGAGCTGACAACAGATGATGCCA

GACCCCTGAGCAGGCTAAGCAAAG

>4292 len=204 tot-seq=2

CTGGGGAACCAGGAGCCCTTTGGTATTACTTGCAGGTAGAAAAGGGGAGGAAGCTGGGAT

GAGGTGGGGAGGTCAGCGGGGGCCATGCTGAGGGCATGGGGCTGGTGATGGTGGAAGAAA

TTTTAAAGCAGGTGGAATGACATGGTCAGTTGCATAGACAGATAAATCACTCCAGCCCTG

TAGCGGAGGGCAGATGGGGGAGGT

>4293 len=204 tot-seq=2

CCCAGCCGCTCTCTTGAAAATGCCATAGCAACCCCTGGCTGGTTGCCTAGCAATTCCTCC

CTTTTCCCTTTTCCTCCCCTAAAGCCCAAGCGAGTAGTCCACAGGCTTCTGGTGGAACAT

GTCTCTGTGCCATGGCAAGTGCCTGTGTGTGTGTGTGCGCACTCATGTGCGTGAGAGGAC

AGATCCGGAAGGGAACACTTCCTG

>4294 len=204 tot-seq=2

ACGGCATACGAGATGCGATAGTGACTGGAGTTCAGACGTGTGCTCTCCGATCTCCTACGG

AGGCAGCAGTGGGGAATCTTGCACAATGGGGGAAACCTTGATGCAGCGACGCCGCGTGAG

CGATGAAGGTCTTCGGATCGTAAAGTTCTGTTGCAGGGGAAGATAATGACGGTACCCTGT
GAGGAAGCCCCGGCTAACTACGTG
>4295 len=204 tot-seq=2
CGTGAGCATCACACGGGTTCAGCTTCTCCAGAGCGTTTAGGAAGGCGCCTTACTTGTCAC
CAGAGTCCCGGGAGGGCAGGCCTGGTCTATATCGACAGGGCAGCAACCCACTGCCTACGC
AGTGCCCGGCTCCAGCACACCAGGCGGCGGTCAGAGGGCAGCAAGGCGGCCAGCCCTGAC
CCTTCAGCACAAGCCAACGGCCTG
>4296 len=204 tot-seq=2
TGGGGAATATTGCACAATGGGCGCAAGCCTGATGCAGCCATGCCGCGTGTATGAAGAAGG
CCTTCGGGTTGTAAAGTACTTTCAGCGAGGAGGAAGGCGTTAAGGTTAATAACCTTGGCG
ATTGACGTTACTCGCAGAAGAAGCACCGGCTAACTCCGTGCCAGCAGCGCGGTAATGCGT
AGATCGGAAGAGCGTCGTGTTGGG
>4297 len=205 tot-seq=2
GGCAGAGAGTTGCATGTGCAACTCAAGAGTTCAAGGAGACCTCTACAAGCCTTGGGCCCT
GGAGCAGACCAGCACTGGTATCACAGCCCCAGTAGAGGCCATATATTTGTGGCACCTGGG
AGCAATAAAATTGCTCCACCTCCTTCACCAGATGGGGCTCAGTGCCAGCTTCTGACTCAG
TGGTCCTGCTATGGCCTGAACTTGG
>4298 len=205 tot-seq=2
GTGGAGCTCTGAGTGGGGGAAGGCGGTCTGTGGGAGCCTCGTGTTGTCAAGGTAAGAGAT
GTCTCCCTTACTTTGTAAACATGTTTAACCATGTTATAAAAGCTGTTCTAAACCGTCAGA
CGTGGGATCTAAACAACAACAAATGTTCTGCAGTTGGCTGATCTGGAAAGTGAGCAAGCC
ACGGTGACTCCCCCTCCTTGTGTCC
>4299 len=206 tot-seq=2
CAGGTGGATGTAGGCTCCTAGATTCCTTGCTTCACCAGCACAGTGATTGCGGCCACAGCA
GCACAGTGATTGTGGACTCTGGGGCTGGAGGAGGCAGCAACAGTGGTGTGACTTTGGGCT
TCTGTAACAACAGCAGGAATGCACTCCTTCCCTGCAAGGTGATATGTGCAGTGATTCCAA
TGAGAATCATAGCAGCAGTGGAGGCT
>4300 len=207 tot-seq=2
TCTGAGAAGGCTTTATAGAGGCAGTAGGAGGTAGAATAGAAGGTAAAAGATGTGAGCCGA

GGATTAGAGATGAGTAGAAATAGTCCTGGTGAAGAGGGAGAGGAAGGAGGTTGCTATTTC

AGAGAGGAGGCTGGAGATGTCACGCACAGGATCTGCAAGAGCCAGGAGGGCAAGATATAG

GTTGCAGAGATGAACCAGGTCAGCTCA

>4301 len=207 tot-seq=2

CAGAAACGGCACACCTGGGCACCTGCATCATCAGTGAGGTCAGAGGGAGCCACCTGGGGA

CCCAGGAGGCTACAGGAGGAACTTCCAGGAGAATTCGGGTAGGGACGTCAGCATGGGGCT

CGCACAGGGTCCCTGGCAAAGAGGAGCCATGGGGGAGGATTCCGGCCCCAAGCTGTCCTC

TCACACAAGGCTGTACATCAGCCGACA

>4302 len=208 tot-seq=2

TGGGGAATATTGCACAATGGGCGCAAGCCTGATGCAGCCATGCCGCGTGTATGAAGAGGG

CCTTCGGGTTGTAAAGTACTTTCAGCGGGGAGGAAGGCGATAAGGTTAATAACCTTGTCG

ATTGACGTTACCCGCAGAAGAAGCACCGGCTAACTCCGTGCCAGCAGCCGCTAATAGGGA

GATCGGAAGAGCGTCGTGTTGGGGAATA

>4303 len=208 tot-seq=2

AGTGGGGGCAGGCTGGGGCGGATGGAGGAGGCCTGTTCCTGGGGAAGGCCGAGAGGGGTC

CTCTGGCATAGCTCACTGCTGACCCCATTCAAAAGGCTTTTCTGAAACGATGTGGAGAAA

GTGCTGGAAAGAGCAGAGACATGATGGGAAGTGGATGCCAGCTTGGGGGCTCAATGCTCC

CCTCAGCGTTCTGGGGCCTTTGGAGTTC

>4304 len=208 tot-seq=2

AATCGCTTGAGCCCGGGAGGCGGAGGTTGCATTGAGCAGAGATCACGCCACTGCACTCCC

GCCTGGTGACAGAGGGAGACTCCGTCTCAAAATAAATAAATAAATAAATAAAAATTTAAC

AAAAAAAGGTAGAGGCAGCTCTCAGGTGAGTAAATTCGCAGATGCCCACGCATGTCTGGA

ACATACCCCAGGGGTCATTAGAGACTGC

>4305 len=208 tot-seq=2

AGATTCTGTCAGATGATGTATCGTCAACAAAACTCAACCTTCCATTGCTGGCTCTGAAGA

GGGAAGGGGTCATGAACCTAGGATTGCTGTCAACCTCTAGACACTGGAAAGGGAAAGGGA

ACAAATGGTTTTCCAAAGCCTCCAGGAGGACCCAGACCAGGGACACCTTGATACTAGCCC

AAGAACACTAATATCAATAGTCTGACTT

>4306 len=208 tot-seq=2

GCAAAGAGAGACAGCTTGGCAGGGAAACTCCCATTTTTAAAACTATCAGATCTCATGAAA

CTTACTGACTATCATGAGAACAGCACGGAAAAGACCAGCCCCCATAATTCAGTTACCTCC

TACCAGGTTCATCCCATGACACGTGGGAATTGTGGGAGCTACAGTTCAAAGATGAGACTT

GGGTGGGGACACAGCCAAACCACATCAG

>4307 len=208 tot-seq=2

GAGCTACAATGCAGAAAAGCCATGGATTAATAGGAACTGAAGCGCCGGGAGCCATGAAGC

TGCAGGACCCATGAGGCAGAAAAAGCCATGGGCTAGCATCGAGGGGGGCAGAAAGAAGTT

AGTCAGTAGCAGTGGGAGGAGTATAAATACAGCCAGAAAGGAGTTGAGTCACCAATTTGG

GAAGCACTAGAGAAGGGAGCAACAGATG

>4308 len=208 tot-seq=2

CAGTTGTTAAAAGTGCAGGTGAGATGACATTGCCACTTAGGTGACAGCCGACAGCAATGA

TTTCCCTGCCTAAAAAGAGACCACACCAGAAACAATGCTGTGCAGATGCAGGCAGAGGCT

ACGTGACACGGTGCTGGCATGTTGGGACAGGAGACAGACGTGGAGACTCTGGGCATTCTA

AGAAGGGGGTTCCTTCAGACTACACATG

>4309 len=209 tot-seq=2

TACACATGAGACCCCCAAGGACTCACCCTGTCAGCATATTAAACAGTTGTATTCTGAGGA

AATTCAATTAAAACACCAAAGAAAAATACTCGTACACTCACAGTGGAAGCTCGGCCAGCG

CTGTGCTGGCAGCAAAGATGAGGTAAGAGGAAGCCAGGTAGGTCAGAGCGAGTTCTAGAG

ACCTGACCCAGGAGTTCTAAGTCTGAGGT

>4310 len=209 tot-seq=2

GTAATTTTGATCTTGTGTTTGGATCAAAATATAGTACAAATCAAACACAATTGGTGTGAG

TGCATTTAATAAATGAAGTCTTGACCATTTGTGTGTTGCCAAGCTCCAAAAAGCAGTCTG

ATCATTCATGTACTTGCAGGGTCCATTTATTTCTAATCATCCTAGCTTAACTTCATTTAA

AGAATAAATTAATCGTTTTTAAACTTATG

>4311 len=210 tot-seq=2

ACAACCTTAAACTTCCCTGTTAGACAGGGTTCTTTTCAGCATCCTTTGAGGGTTCTCCTC

TTAGAGTGGAGAGGAAGGACTGAGGCTTACTGTACTGTGTGTCGGGAGTCCCTCTACAGA

CCCTTCCGGCTTAGCAGTTGGGGAAAGTGACGCAGGGAATTTAAGACTAGCAGGGAATCC

ACCAGACCCACTTCTGCTTGTTTGGTTCAG

>4312 len=210 tot-seq=2

TGGGGAGGTGAGGACTAGGATTTGGAGTCAAAATAGACCTGGGTTTGAATCTCAGCCCCA

CTACTTATTAACTGTGTGACCATGGACCTATATCTCTGTGAAACTGATAAAATAATAGTA

TCCACCTCATTATCATATGTCAGGCACTCAGCATAGCAGCAGTGATCAATGGCAGCTCAT

ACTCCTTATTAGACAATCCAATCATCTGCC

>4313 len=210 tot-seq=2

GATCACTTGAGCCCAGGAGTTTGACACCAGACTAACAAAGAGAGCCAACATAAGTGACTG

GCCCTTCTGAGTGGTAGCCTCTCAAAGTTTAGACGTCAATGAAAGAGAAGGAAGAAACAG

AAGTGTTGGCTGTGTCTGGAAGATGAGAGACAGGGGGTATCTAGAGAAGACCAACTCCTG

CCTCATGGATTCTGTCGGTCAACATAGCAA

>4314 len=210 tot-seq=2

GCAGCCTACTGGTCAGCCAAGGGGTCCTCCCTGAACCTGTCTCCTCCTGCAGCCCTGGGC

TCCCCACTTCCCTGTGTGTGTGCCTCTTACCTGACAGCCAGGCCTGGGGTGACACACAGT

GTGCGCTTTTGTCTTGCAGGGCCATGGGTGTCATCTCCTACCTCAGGTGAGCAGAGCCCT

GCCCCATCAGCTGCCTCCTCCAAAGCTGAC

>4315 len=211 tot-seq=2

AGCCACAAGACACTGTTATCATAGCACCCCCACAGTACGTAACAACTTCTGCATCCAATA

TCGTCTCAGCGACTTCAGTACAGAATTTCCAGGTAGCTACAGGACAGGTGGTTACCATAG

CTGGTGTCCCGAGCCCACAGCCCTCCAGGGTAGGATTCCAGAACATTGCGCCCAAGCCAC

TTCCTTCTCAGCAAGTTTCACCATCAGTGGT

>4316 len=211 tot-seq=2

TGGGGAATTTTGGACAATGGGGGAAACCCTGATCCAGCCATCCCGCGTGTATGATGAAGG

CCTTCGGGTTGTAAAGTACTTTTGGCAGAGAAGAAAAGGTACCTCCTAATACGAGGTACT

GCTGACGGTATCTGCAGAATAAGCACCGGCTAACTACGTGCCAGCACCGCGGTAATATTA

AGATCGGAGAGCGTCGTGTAGGGAAAGAGTG

>4317 len=211 tot-seq=2

CAGGAAGATCGGGAAGTTCAAGGCCAGCTTGCTCTACAAAGTCAGACCCATTTCTCAAAA

GAAGGAAAAAGAAAAATGAAAGAAATGTAATGACTAAAAGTGGACAGTCATCCTTGACTG

CATAGTGAGTTCAGGGTCAGCTGGAATATGTAGGACTCTGTCAAAATAAAGATGATGACT

ATACCTGTATGCAGGTGGCATTGGAGATCCA

>4318 len=211 tot-seq=2

TGGCGTGGCCTGCGTCGGGCTGGCCACCTGGGTGCCAGAGCATTCGCCCGTGGGAGATGG

AGATGTCTCTCCCGGGTCCAGATGTCCAGGGATCAGGTGAAAATGATGAGAGGAGCCTGG

GCGGCCGGGCTCCGCCATAAGGGCCAGGTGCGGCCCCAGGTGGTGCTTCCTCTCTGACCT

TCCCTTGCCGCCTAGCATGGCTCCCCGCATG

>4319 len=211 tot-seq=2

GCTGGAGTGCAGTGTGGCGTGATCTTGTCTTACTGCAATCTCCGCCTCCCGGGTTTAAAT

GATTCTCCTCTCTCAGCCTCCTAAGTAGCTGGGACTACAGGCATATGTACCCTCTTTTTA

TAATTAAAGATTTCTTACCAAAAATCCAGATGTCCAGCTTCTGGTGGACACAGAATATCT

GAGAACAGTAGCTGGCATTCCCATGCGGCAA

>4320 len=211 tot-seq=2

CCGACCTTTTAGTCGGAGAAACGTGCACGGGCGAATAGAGGAAAGGTGGTGCGGGCACAC

GTCCGGCGTGTCTCAGAGCTACGGCATCACTCCTCAGGGCCACACGGCCTGGCGCGTCAG

AGAGCTGCAAGTGCGCCGACGAACTTATCCAACGCGGGACGAGTGTTCGCAGCTGCATCG

TCAGGAACGTTCTCCGTGCCGATCACGTAGA

>4321 len=212 tot-seq=2

AAGAAGGTATGTGAGTCATTGTGGGCTAGGAAGGTGGCTATGTCCAGGCCCCCAGCTGCA

GCCTGGCTGGCAAGCTCAGGTGTGACCCAGGCCCCAGCAGCCTCTGTGGGCCCATCCTGC

CCATCGGTGCCACCGCTCAAAAACAGCACATCTATCGGCCCCAGCGGCCACCTTCTCAAC

TCTGCTCCAACACGCAGGGCCAGTTCCTGGTT

>4322 len=212 tot-seq=2

CTCGTGAGGCAGGAAGCCTGGAGAGGGAAGAGCTTCCGGAAAGACAAGGTTGGTCACATC

ACATGATATAGGAGATTGAGTCAAACGAGGACAGAGAAGTGAACACTCGCTGTGGCCACA

TAAGGCATCAGTTACCAGTAGAACCATCTCATGGGGTATGATGGAGACAAGAACCCAGGT

AGAGAGGCTCTAGGAGAGGGTGAGAACGTGAG

>4323 len=212 tot-seq=2

CAGGAGGAAGAAGAGAACTTTCTCCCACTTTGGAGCTAGAAGGTGGGTCTTGAGCATAAT

GCTCAGATAAGGGAAGAGTCCCAGGTAGTCTCGTACATCTATTTACCCAACTCCACTCAC

CTCCGTAGCAATGCCTCCTGCCCTCCAACTCATCAACTCAACACCAGCTGCCAGACTTTG

TGCCAGGCACCAACCAGCAGAAACCAGTGAGG

>4324 len=212 tot-seq=2

CCTGCACCCCACTAGATCAAGGAAATACACACAAATCACACAGAAAGATGATCTCACAAA

ACGACTAACAGGACTGAACTATTTAACATTAACAGCCCAGGAAAGGCATTCTAACTCACT

AGATCAACAATGAAAAACAACAGACGTCGGTGTCCCCCACAGCCAGTCACTACCTTAATA

ACAAGCTGTATGTTTCAATTCCAATCACAGCC

>4325 len=213 tot-seq=2

TAGGGAATCTTCCACAATGGGCGCAAGCCTGATGGAGCAACACCGCGTGAGTGAAGAAGG

GTTTCGGATCGTAAAGCTCTGTTGTTAAGGAAGAACGAGTGTGAGAATGGAAAGTTCATA

CTGTGACGGTACTTAACCAGAAAGGGACGGCTAACTACGTGCCAGCAGCCCGCGGTAATA

GCTAGATCGGAAGAGCGTCGTGTAGGGATAGGG

>4326 len=213 tot-seq=2

TAGGGAATTTTTCACAATGGGCGCAAGCCTTATGAAGCAATGCCGCGTGAACGATGAAGG

TCTTATAGATTGTAAAGTTCTTTTATATGGGAAGAAACGCTAAAATAGGAAATGATTTTA

GTTTGACTGTACCATTTGAATAAGTATCGGCTAACTATGTGCCAGCAGCCGCGGTAAATC

GGAAGAGCGTCGTGTAGGGAAAGAGTGTGCCAA

>4327 len=213 tot-seq=2

CAGAAAACCCAAAATAACATTGGCTTATATAAAAAAAAGAGTTTACATTTGTTTCCTATT

AAAAAAAAAAAGCAGTTCAGAGCTGATCCCGTGGCTCCATGTTCATCAAGTACCCAGGTG

GCTTCTCAGTTGCTGTGCCCTCCTCAGCATGCTGCTTCTGTCTCATGGCCCCAAAGGCTT

GCTCTAGTGCTGCCTGTTACATCCACGTTCCAA

>4328 len=213 tot-seq=2

GAGTTCAGACGTGTGCTCTTCCGATCTCCTACGGGAGCAGCAGGGAGACACAGAGAGTAG

AGAGGAATAAAGCTGGACACCAGTCTGTCTAGGTTCAGTGCAGGAGAACCTCTCGGACAT

GGAAAAGGGTGAGTGAATGAGAGTCCCCTGAGGAATTCACACTTTCCATAGGGACCTCTG

CAAGACTGGAAACGGGAGAATCCCCCTGGACTC

>4329 len=213 tot-seq=2

TGGCAGAGACAAGGACTTGTTCTCCTATATGTTTGTGGAAGGTGGGTGAAGAGAAGGGAG

AAGTGACGGCTGCTTCCCCAATGCCTTTGAGCTGTGGGAGGGGGTTTTAGAGGTATTAGT

TATCTAGGCTTCCTTGGGATGGCTCAGAAACTAAAGTGCCTTTTGCATAGCATTGCTTCT

AAAGGCAAATGAAATAGAAATGATAGATAAATT

>4330 len=213 tot-seq=2

CCGAAGTTTCCATAAAACGGTACAGAACCTTACGATGGACACTGACGATTACGAAGGGCA

GGCACCATTACCAACCCTGCAAAGAAACTGAAATCCAGCAGGAACAGCAGAAGGAAACAG

CAGTGCCTAGACGACCCAGAAAATAGAGAGAAAAGAAAACTCAGTAATCAACTTGAAAAT

GACAGCAATAAAGTCATTAGCCATGTCACGAAA

>4331 len=213 tot-seq=2

TGAGCCTAGACAGTGGCTCCAGAGGCATTCACTAAGAAGCAGAGACAGCTCAGGTTAGAA

GCACTGTAACACTCACTAAGAGCCCTGGCTAAAAGTGGTGCCTAAGTTGAGTGACTCCAT

TTTCAAAAGCAAGCAAGAAGAGTGAAGCAAAGTTTACTGATGCCAGACTGTGTGTTAGGT

ACTTACTAGCATGGGTCAGGCCATTCAATCTTT

>4332 len=214 tot-seq=2

GCTTTTCCTGTGTTTCTGGGAGAGAGAGAATAACTATTGTCCAGAGGCCACTTTTCTTCC

TGCGTTGACTGACTTGAGAAAATGGAAGAGCCCAATCTCTGGGTCTTGCCCAAAGCCTTT

CAGTTTGTAAATTGGTCCCTTCAGACAGGGCCTCTGAGTGGAAAGCCAGGGCAGTGTGAC

CTCTCTAAATCAATAGTCTTTAGAGGGGGAATTG

>4333 len=214 tot-seq=2

TGGGGGATATTGCACAATGGGCGCAAGCCTGATGCAGCGACGCCGCGTGAGGGATGACGG

CTTTCGGGTTGTAAACCTCTTTCGCTACCGAACAAGGCTCCATTTTTGTGGGGTTGAGGG

TAGGTGGATAAGAAGCGCCGGCTAACTACGTGCCAGCAGCCCGGTAATCCGTAGGTAATA

GCCAGATCGGAAGAGCGTCGTGTAGGGAAAGAGT

>4334 len=214 tot-seq=2

CAAGTGACTGGAGTTCAGACGGGGGCGCGGCAGAGCGCCGACGGGAGGCAGCAGGGGGGA

AGAGGGCACAAGGGGCGCAAGCCGGAGGCAGCCAGGCCGCGGGGAGGAAGAAGGCCGGCG

GGGTGGAAAGTACTTTCAGCGGGGAGGAAGGGAGTAAAGTTAATACCTTTGCTCATTGAC

GTTACCCGCAGAAGAAGCACCGGCTAACTCCGTG

>4335 len=214 tot-seq=2

GGCATACGAGATTAGGCAGTGACTGGAGTTCAGACGTGTGCTCCTCCTACGGCAGCAGTG
AGGAATATTGGTCAATGGGCGAGAGCCTGAACCAGCCAAGTCGCGTGAAGGAAGACTGCC
CGCAAGGGTTGTAAACTTCTTTTGTATGGGATTAAAGTCACATACGTGTAGGTGTTTGCA
GTTACCATACGAATAAGCATCGGCTAACTCCGTG
>4336 len=215 tot-seq=2
CCTGGCACAAACTGGTATGGGTGCTGGTGGTAACTGGAAGAAGCCTGATCTCCTCTGCAG
ATGTGTCTCAAAGTGGGACAAAGTGATTGCTCTGTGTAATTAACCACCAGGGACGCAGCA
GAAGCCCTGAAATGACTCAGAGGATTGTCTGCACATGGGGTTGGAGAGGCTGCAATTCTT
TAACTTTCTTTAGCTGTGTTGAGACTTCCAATAGC
>4337 len=215 tot-seq=2
TAGGGAATTTTTCACAATGGGCGCAAGCCTTATGAAGCAATGCCGCGTGAACGATGAAGG
TCTTATAGATTGTAAAGTTCTTTTATATGGGAAGAAACGCTAAGATAGGAAATGATTTTA
GTTTGACTGTACCATTTGAATAAGTATCGGCTAACTATGTGCCAGCAGCCGAATCGGGAG
ATCGGAAGAGCGTCGTGTAGGGAAAGAGTGGGCTA
>4338 len=215 tot-seq=2
TGGGGAATATTGCGCAATGGGGGAAACCCTGACGCAGCGACGCCGCGTGCGGGATGAAGG
CCTTCGGGTTGTAAACCGCTTTTGATTGGGAGCAACACCGCGTGAGTGAAGAAGGGTTTC
AGCTCGTAAAGCTCTGTTGTTGGAGAAGAACGTGCGTGAGAGTAACTGTTCACGCAGTGA
CGGTATCCAACCAGAAAGTCACGGCTAACTACGTG
>4339 len=215 tot-seq=2
AATGCCTGGCACATGGTAAACAGCCAGTACAAATTACTATTTATTGCGGTTGCTGTTATT
ACTAACCCAGTGTAGGTCCTGTAACATCTCAGCTTTAGTTTCTTCTCCTGTAAAGGGAAC
AATACTAACACCTCCCTCATAGGTAAAAGTCTAATGAGATGAAGCAGGTGAAAGCACTTT
ATCAACTGGGGTTATAAACTAGGTACTCACAGCCT
>4340 len=215 tot-seq=2
AGCCTGTCTGTGAATTTCCACATCAGAACCACCAGGGGGAGCTCACTCACACAGGAATCC
CAGCCCATCTACGAGGGGATGGTGGGAAAAGCAAACCAACTTTCTTCAGTAAGAATGACC
CTTCTGCACGTGGATGAATTATCGCACATAAGCGACAGCTTACTGACAATGGTGGTGAGA
TGAAGAGCCCTTGAGAAAATGTATTTAGAGAAAGC

>4341 len=216 tot-seq=2

AAAGAGGGCCTCAAATCCTCTGGAACTTTGGTTACAGGTATTTGTGAGCCTCCTAATATG

GACCTGGCAACTGAACCTGGGTCCTTTGATAGAGCAGTAAGTACTCTTACCCACTGAGCC

ATTTTTCCAGTCGCTGTCATTGATTGTGTCATAGATTTGCCCATGATAGCTCTTTAGTGA

GAGCACTGGCAACAAAGTAGGTGCCACTTTCCGTCC

>4342 len=216 tot-seq=2

AACAAAGACAGAAGATTACTTTAGGTTATAAGACTTCAGTGGTGAGGTGTAATAAGAAAT

CATGGTACATGAAAAGGAGCACTGGGGAGCAGAAGGTGTGAACCAGAATCAGACATAGCA

ATGGCTTTGATCCTGACAGTGTATCTACTGGCAGAGGATTTTATCAAGCAATAGAAAGCT

AAGACAGCATACTTCAGCCTTTTGACCTTCACTCTG

>4343 len=216 tot-seq=2

TAGGGAATTTTTCACAATGGGCGCAAGCCTTATGAAGCAATGCCGCGTGAACGATGAAGG

TCTTATAGATTGTAAAGTTCTTTTATATGGGAAGAAACGCTAAAATAGGAAATGATTTTA

GTTTGACTGTACCATTTGAATAAGTATCGGCTAACTATGTGCCAGCAGCCGATAATATTA

GATCGGAAGAGCGTCGTGTAGGGAAAGAGTTAGGGA

>4344 len=216 tot-seq=2

CGGTAGTGACTGGAGTTCAGACGGGGGCGCGGCAGAGCGCCGACGGGAGGCAGCAGGGGG

GAAGAGGGCACAAGGGGCGCAAGCCGGAGGCAGCCAGGCCGCGGGGGGGAAGAAGGCCGG

CGGGGTGGAAAGCACTTTCAGAGGGGAGGAAGGCGTTAAGGTTAATAACCTTGGCGATTG

ACGTTACCCGCAGAAGAAGCACCGGCTAACTCCGTG

>4345 len=218 tot-seq=2

GAAGTAGGCAAATCTCTGTCAGGCCAAGGCTAGCCTGGTCTATGCAGCTGGTTTCTGGCC

ATCCAGAATTACCTAGTAAGAACCCATCTCCAAATAAAATGCACTTCCCACATCCCAAGA

GGAGGAAGAAGAGATTCTGACTCTCACTGTCCACTTGAGAGGGAGAGAAACCCTCTGTAT

ACACACATAAATGCTCCCAGCACCACTCTGAGCCACGG

>4346 len=218 tot-seq=2

TAGGGAATTTTTCACAATGGGCGCAAGCCTTATGAAGCAATGCCGCGTGAACGATGAAGG

TCTTATAGATTGTAAAGTTCTTTTATATGGGAAGAAACGCTAAAATAGGAAATGATTTTA

GTTTGACTGTACCATTTGAATAAGTATCGGCTAACTATGTGCCAGCAGCCGCAGCCGCGG

TAATATTGAGATCGGAAGAGCGTCGTGTAGGGAAGTTT

>4347 len=218 tot-seq=2

CAGTCTGAATGACCCTTTTCCACAAGTCTTCTGTGACCACTACAAGCAATCCCTCCTCCT

TCTCATTGCTTCATGCAATTATTTTCTATAGAAATTGCTCAGCATCCTCATTTGCTCTGT

AAATTATCTTTTTATACAATTATAGATGCCCACATGTATGCTTAATTAGCTCGATGAAGA

ACAGAATAGCACATTGGTAAGAGCATGGTTTGGGGGGT

>4348 len=218 tot-seq=2

TAGGGAATTTTTCACAATGGGCGCAAGCCTTATGAAGCAATGCCGCGTGAACGATGAAGG

TCTTATAGATTGTAAAGTTCTTTTATATGGGAAGAAACGCTAAAATAGGAAATGATTTTA

GTTTGACTGTACCATTTGAATAAGTATCGGCTAACTATGTGCCAGCAGCCGCAATATCCA

GATCGGAAGAGCGTCGTGTAGGAAAGAGTGTGCTAGGG

>4349 len=218 tot-seq=2

GGGTCACCTCTACCTCCAGCCCTTGTTTCAGAGGCCCATATCCCATACCCTTGACCATTC

ACAATAGAGGGACAGAGGTGAGGCCGATGGGGATTGGGAGTGGCTTAGTGCACAGGGAAG

AAGTCAGGCTGAAGAACAAGCAGCCCTTCCCCTGCCCTCCCAGGTCTAGGTCAGGCAGGG

CACAGGCTAACCTCCAAACCCAGATGGGGGGTATGGAG

>4350 len=218 tot-seq=2

GGAGGCTCAAAGCAGGAACTCCAGCCATGTTTGCAGGAATTGTAGCAATATCGTTCATGT

ACATAGAAAGTGGATCATCCATCTTCTCGCCAAACTTAAATGCCGTCGTAGGGCTTGTTG

GAGAAATAAGAACATCTACTTTCTTAAACGCTTCTTCGAAATCGCGAATAATAAGCGTAC

GAACTTTTTGTGCAGAACCATACCATGCGTCGTAATAG

>4351 len=218 tot-seq=2

CCTGGGCTGTGTGCCACTGTTAACACCTGGCAGAGTTCTCGAGCCTCGGGTCTCAGGGTG

CCCTCACTGCCTGTTCTCCACTGGTTCTTACTCAGCACCTGATCCTTATCACAGCAACCA

CGGAAAACCCAGCCTCACAAAATCTGGCGTCCACGTCGGGAGTGTGGTTCCATCCACTGC

GAAAGCCCCACACATCTCCCCGGCCTTTCATGTGGAGC

>4352 len=219 tot-seq=2

CAAAGCATGCGAAGAAGGTGTAGGGTCCCCAGCAGACGCAGTACGCAAAGATCATCACCA

CCACCATGCGCGTCACTTCCTTCTCTGCCTTCTGGGTGGATTCAGACTCTTTCTGCTGCT

TTGCCACCTAAGGGAGACAGGGATAGTGAGTCGGGGGTTGGAGGGCAGCAAGAGGCAGGT
GACCCAGGCATAGGGAGTTGTGGGGAGGAGGAGAGGAGC
>4353 len=219 tot-seq=2
GGTCCTTGAGGGAAGTAGGCTCTCCTTGAAGGATAAGCAATGGCTGCATGAAGTAGCAGG
TATTGAAGCTAGGGCTTCGAAGGATGAATAGGAGCTTGCCAAACAAAGAAGGTGGGACAT
GCAAACCAAGTGGAGGGACTGTGGCCCAAACTCAGGGATACATGGATGGAGTAGCAGGAG
AAAAACAGACTGGGGGAGCAGGAAGAGTCAGTGTGAGCT
>4354 len=220 tot-seq=2
AAGCAATGGCTGTGGGCTAGATGAAAGGGTAGGAACTCCTGCAGCCCCAGCGTTTCATGA
GGAATATAGTCCTGGCTAGCACAGTGACTTGGCTTTTCTGTCCCAAGACTCATGCTCAGA
GCTGCATGCCCTGCTCTGGCCACTGTCTGAAGCTGCCCAGCTCCCAGGCCACAGGAGAGG
CAGGGAAGAGAGGCAGAACAGCCCTACTGTGAGCCTCCTA
>4355 len=220 tot-seq=2
GGAGTTCAGACGTGTGCTCTTCCGATCTTCTACGGGAGGCAGCAGAGGAAGTCCTGATGA
ACTAGGAAACAGAACAAGGACATCACATAATCCTCATGGACAAAGCCATTCCACATTACG
GAGGTGGTCATGAGTCACTGTAGGGCCTGGGGAGGGGGCATCAGATGGCCCAGACCCAAA
CAGAGTGAGGGGTGGAAAGGTATCCTACGACTCCCAGGAG
>4356 len=221 tot-seq=2
TTTTACTTGGAGAGTTGTACAGAGACAGACAGGTTGAAGAGAGAACAAGCCAGACACAGG
GAAGACAGAATGAACCATCGAAGGAGAAGAAACCAGAAGACTGGAACAGATTGCCATAGT
TAGTATGAGGCCAAGCAGAGCATTTCAAGAGAAGCAGTGAGAGAGAAGCCAGATTGACTC
AGGCAGCTTGGAGAGGAGTTTAATTGTGCTGTGTTGACCAG
>4357 len=221 tot-seq=2
ACAGGCATCAGGGGTTAGCTGTGACCTTTTGTTCCTATTATTAGATCCTCAGCAGCCATC
TGTCCCATTAGCCTCCAGCTTGTGGTGGTGCTTGGGCCTCACCAACTCCTGGTACCTGGC
CAGGGCTAGGGTGTGGTCAGGCTGTTGCCCAGGAGCCCAGTTACCAACCTCAGGGTAGGG
CTGCCACCTCCCAGCCTAGAAGGCCATGAGTCACCCTTCCA
>4358 len=221 tot-seq=2
CAGTCACACAGGATGGGAGCTTGAATCTCCCGATCTCAGCAAGGCAGGGATCTATGAAGG

CATCCAGGCTCTACCCTCTGGGTGGCTCCACACCGAGTGCTTTTCCCTCAGCTCTTTGAG

GCCAGCCAGCACCACCAGGAGCCCCTGGCACAGCTCACAAAAGAAAAAGCAAAGGCCAGG

CATGCACACGGGGAAACACAAAAGTAGACACCTCCCCCAGG

>4359 len=221 tot-seq=2

ATGAGAAGAGTCACAGCCAGTCAGGTCAGAGTCGGGTCCTGCTCCGAGGATCCCCAAAGG

AGGTTTGAGGGAAGACTCAGCTGGCTCGACTCAACCCCCTTAGTGAGAACAAACTCACGG

GAAGAGGAACATCAATGAGGCCAGGAATGACAGAGGGAAAGAGGCGTCTGGAGTCACGAA

GGGGCTTTGGGGTCTTCACAACACCCCAGACATGGGCGTTG

>4360 len=222 tot-seq=2

ACCCTTCTGTCTTTGTGACCACATGTGGCTGTCCTGGCACTTCTATCCCAGGGCTCCTCA

TCTAGGGGAGAGGTCACAGTCCAGACACCTCTGATTCTGACCCCATCCTTCTTCTGGCCC

CAGGAGGGAGACTGGAAAGCATCTTCTTATCTAAGCACCTTTATTCCCCAAAGCCTGCAT

GTTGATGATGCCGACTCATTGCCAGGCCCCGCGTTCACATAC

>4361 len=223 tot-seq=2

GACCAAGACAAGCTAGCAGTTCTGGAGCAGAAGCCCAGGGTGTCACAGCCAAGCTGTCGG

CTGTCGTGGACATGTAATGTCATGAGCTGGCCAGACGTCCACGTCAGAATGCTAACATTT

GCATGTTCTTAGTGCAGCTCTGATCTTGAAGTCTGCAAGCCACCGATCTATCACCAAGTA

AGGCCAAGCAGTCTCTCACACCATGATGCCACAGGCTTCAATT

>4362 len=223 tot-seq=2

CTCCCGCAGGGCTGAAGTCTGGCGTAAGATGATGGATTTGATTCGCCCTCCTCCCTGTCA

TAGAGCTGCAGGGTGGATTGTTACAGCTTCGCTGGAAACCTCTGGAGGTCATCTCGGCTG

TTCCTGAGAAATAAAAAGCCTGTCATTTCAAACACTGCTGTGGACCCTACTGGGTTTTTA

AAATATTGTCAGTTTTTCATCGTCGTCCCTAGCCTGCCAACAG

>4363 len=223 tot-seq=2

CTGGGGCAATGTCTCAGGGTGGGAGGTGGAGGCAGAGACAGAATGAGGCCCTGCTTTGTT

GGGGCTGTGTGGGGTGGCCGGAGTCTACTGGCCTGGGGTTTCTTGGATGTGCAAGATAGG

AGGCTGCTTTTGACAAATTCCTTTGTGAGCAAAGTCATCACGAGTGTGTGGGCTCAGAGA

CAGGCCCTCCATGCCAACCACCACCTGCTTCCCTCGTCCCACA

>4364 len=224 tot-seq=2

CCAGCATGACTGACAACTAGTAACCAAACAACAGGAAACCTGGAAAGATTTCAATATGGC

TGAATAGTTGTATCCAACTCTCCTCCCCTACATAGAAGTGCCGAAACAACAAATGTACAG

CAGTATTTTGAAGTGAATGCTGTGGATGGGCCATGAATATCACCCAGTGGAAGATAGGAG

TTTCCTAAGATCAGGAAACCAGGGTGCCAGCAGAGATGATCAGC

>4365 len=224 tot-seq=2

CTCCATCAGGTCAGGCCAGGCCATGCCGGAGGTGATCCCTAGGGCTTCCTTCCCCTCATG

GCCCACATCCAGCCACCAGGAGGTCCCGAGGGCTCCACCTTCAGGTTCTGGCCCACACCG

GCCCACCCCTCACCTCCTCCAAGGAGTCTGTGGGGGTCCACACCACTGTCATCCCCTCCT

CGTCCTCACTGGTGTCCCTGCTTCCTTCCTAGGCCTACTTCCAA

>4366 len=224 tot-seq=2

CACAGAAGGCGGTGGTGGGTGCGGTGGGAGCCAAGGCCGATGAGCCTCTGCAGGATGACT

CTTGAGAAATGGACCAAGTGCCAGTTCTTCCAAGTCACTTGTGGGTGTTCTTCTGTCCCA

TAGTGACAGGGGCAGGCTCAGAGGCACATTTCAGGAGCTATTTGTTCCTCTGTTTTCCAC

TGAGACACCTTCGCACAGCACCCGGTCCAGTCACCCCCCTCCAC

>4367 len=225 tot-seq=2

TTTACGTGGAAAGACAGGGTTGAGTGGGTTCACGGGGGTTCCTGGAGCACCTGGTACCCG

GGGTGCGAGGGAGTGAGTGTTTTGGGGCTCTAGGCTGAGAAATTTTGACTTTTTCCAGTA

GGCAATAGGGAGCCACAGAAGGTCTTTGAGCAGGGAGTGAGGATCAGATTTACAAGTAAG

GAAAGTTCATCTGGGATGGAAAAGGAGGGGCCAGAAAGCTACAGA

>4368 len=225 tot-seq=2

TCCGATCTCCTACGGGAGGCAGCAAGGCAGCGAGGAGAAGTGTGCCTCCCGGGGGGAAGT

CTCAGGATTGTGGCCGCGGGTGAGGTGGATGGGAGAGGGGAGAATGACTTTCACTGGGCA

AGGGAGAGAGGCTCCTGCTCTGAGACTCCCCTGAGAAGAGGCCGAAGGAGGCCCTGGGTG

TGAGAATCTACAGGATGTAGAGCTGGGAATCAGCCAGGACCCCCT

>4369 len=225 tot-seq=2

GAAGATTGCGAAGTTGTTTGAGATGACGAGGCTTGCGGGGCTTGAGCGCCGTTGTTAGCT

CCAACAGCAGGCCTTTTCTTTACAGCATACTCAGTAATCAGCTTCTTACTCAAACGCAAG

TAGCGCTTAGCATCCGCATCCGTACCGTCGTCAATAACAAACTTGCCTTCGTCGTTTTTA

ATGTAGTACTGATAATCCCCGGAATGGTATACAGATGGACGGTAT

>4370 len=226 tot-seq=2

GTCCTGGGGAGAGAGTGGACCCCGTTTTTTGCATTAGCCCATATTGGGAAGCAATGGGGA

TGGGCTAGAAAGCTGGTGCCTCAAGAGTGGTTATTGATGTCCTAATTACAAGAGGATGCT

AATTTAATTTGAACCTAATTACAGTGCACTACCCTGGGTGGCATGTGTTTTAATAATTAA

CGTCCTTCAGATGCGTAGGAGTGTAATAATATTTACACTAGGTTGG

>4371 len=226 tot-seq=2

TCTGTTGCCAAGTGAGGACATTATTGAGATGAAAGCGGTGCTGAGGGGTCAGGGGGTCTC

TACAGGTGTGCTGGGCAGGGCCTGAACCCCTGGCATGCACAGATGGTCCATGCTTACATC

ACTCCTGGCTGCCCTGTGGGCTGGGTTGGACATTTCTGGGTCATGGGAACCAGATAAGCT

CAGGTACAAGTGGGTGGGTGAGATGCTGTGTGAGCTCAGGGATTTC

>4372 len=227 tot-seq=2

CTAAAAAGAATCCTGACGTGTTGGCTTGGAATGGAAGGTATTGATGTGAATCCACAATAT

AGTGATAGTTATAGAGATATAGAGGTACACATATATTTATAGATGGATACAGATACAGAT

ATCCACACATATAGTAACAAACATACATGTGTATGTGTGGTTGTGTGTGACCCTGTATCA

TTTATTTCCTAACTGTGTCCACTGAAAGGGCCTAAAAGTCTGACACC

>4373 len=228 tot-seq=2

TGGGGAATATTGGGCAATGGGCGAAAGCCTGACCCAGCAACGCCGCGTGAGTGATGAAGG

CCTTCGGGTTGTAAAACTCTTTGGACAGGGACGAAGAAAGTGACGGTACCTGTAGAACAA

GCCACGGCTAACTACGTGCCAGCAGCGCGGTAATAAGCAGATCGGAAGAGCGTCGTGTAG

GGAAAGAGTGGCCAATGGGGAATATTGGGCAATGGGCGAAAGCCTGAC

>4374 len=229 tot-seq=2

GGCATACGAGATTCGCTTGTGACTGGAGTTCAGACGTGTGATCTTAAGATATAATAAGGG

AGGAAGAAGTAGGGAATATTAAAAAATGGAAGAAAGTATGATGGAGAAAAGAAGAGTGAG

TGAAGAAGGTATTAGGATCGTAAAACTCTGTTGTTAGAGAAGAACACGAGTGAGAGTAAC

TGTTCATTCGATGACGGTATCTAACCAGCAAGTCACGGCTAACTACGTG

>4375 len=230 tot-seq=2

CGGGGGTCTGGGAGAAAGCATAAGGAAACCATCAGAGGTGTAGTCATATGCCTGCTTAAG

ATGTAGAATGCTGCTGATGCAGTCTTTCCGGAACGTTATGAGCCTTAATAAATATCAAAT

GAGCTTCGTCTTGGCTTAATGATGCTGAGAGATGTGTGAATGTTTTGAGTAGACTGTATT

TACCAGTTCATAAAGAAGTATTTGCAGCATTACAAGGTGCCTATTGCAGG

>4376 len=230 tot-seq=2

TGTGCAGCAGCTGAAACTCCTCCTCCGTCCTGATGCCAGCCTCCAGGCAGCCACAGACAT

CCTCTTCAGAGAAGTAGGTCTTGCGGGAGGACACCCCCTCATAGGCCAACGTGCCCATGG

TTCGGGCTGCATAGGCCATCAGGGACAAATTGGAGGGGTCAGTGCTGTCCAGGGTTTCCC

CGCTGAAGTTGAGGCGCAGGAAGCTGGTATAGATGCTTGTAAGGGTCTGC

>4377 len=230 tot-seq=2

ACACCGGCCCTCTTTACTTTTGGACTATTGAAATGCTGTGAATAGCTGACCCTCGATTTC

AATTATGTCCGCAATCGGAATCGTCTGCCCATTTACCATAACAAGCTCCTGCGCGACAGC

ATCCAGTTTTTTCATCCGGACGGCGGCGATGATATATTCGCCGCCGTCCTTTTTCTTATC

CGGCAGAAAATACGTGATCTCCGCCTCCGGACGCTCCTCGATAACCTCTG

>4378 len=231 tot-seq=2

GCACTGAGTGGATCTGTGGTGTTAGTGACTTCTGAGCAACCAGGGGCTGGTTAGTGGGGA

TTTCAGAGGAGGTGTCAGCAAAGGAAACATTGCAGATGTGGCCACCTGTCCACTCTTCAA

GGCAAAGTGTCACCACATCCACATGAGGAGCCATTGTGTCATTTTAATACAAGGTCACAG

TTCACCCTGCTCTCTCACTTTGCTGATGTCCCTGATTACGCAGCAGCAGCA

>4379 len=231 tot-seq=2

CCGGCATACGAGATTAACTCGTGACTGGAGTTCAGACGTGGGCTCGGCCGAGCGCCGACG

GGAGGCAGCAGGAGGGAAGCGGCCACAAGGGACGAAAGGCGGAGGGAGCAACGCCGCGGG

AGGGAAGAAGGGGGGCGGCGCGGAAAGCTCTGTTGGTAGTGAAGAAAGATAGAGGTAGTA

ACTGGCCTTTATTTGACGGTAATTACTTAGAAAGTCACGGCTAACTACGTG

>4380 len=232 tot-seq=2

CAGAGGATGCAGGACACTGGTTCACCTGAGGATGCTGGCTTTCCTTTGTGTGTGCTTTTG

CAGCAGTAGTGGTTGTTGCTCAGGGTGGGGGACAGTGTTGTTGATGTCTGTGTGCTTGTT

GGTGCCAGCAGTGATGGCCGCACAGAGAGTGGGCAAAATCGCTGGCATCTATCTGGGCAT

GTGTTCACACCAGTGGTGCAGTGGTGGTGGAGGGGGATGGAGTACGCTCATG

>4381 len=232 tot-seq=2

ACCAACTACTAACAAAAAAGTAAATGACACTCTTTGTTCTGAGTATGTTAGAACTCAGAA

CATCAAACTCTCTATCTATATAGCTCTATCTATATCTAGAATTTTACTTAAGTAATAGGG

CTTCTATCACTAACTTTAGGAATTCTAGGCCTACATAAGATCTCTCCCCTCTACTTGGTT

CAGTAAGAAGTATATTGGTACTCACCGTGTACTGCCAATCATAAGGTTGATA

>4382 len=235 tot-seq=2

TAGTACAGCATCCTCTGTAGAGTGTCAGTGGCAGCAATAATCCAGATTCCTTTTTCCTGT

CCATCTTCTGAGCATGGTGCTCAAGATTTAATTGATTTTAGTTGACTGTAACCAAGAAAC

CAGCTAGAACTTTCTTAGTTTTCATAAGTCTGCAGACCTGACCCTGAAACCCAGCAAGCA

AGCCCAGCCTTCCTGCCAGCAGCTGCGGTAATAAGAAGATCGGAAGAGCCGTAGT

>4383 len=235 tot-seq=2

ACAGTGATTATCAGATATGAAATTGCAGATAACTATGAGAACTATGGAATTTTGCATTTA

GATAAAGTCCAGAGGTGACTGCCCAGGCCTTCGAGGTAAGTATTTTAGTGGAATCATTGT

AGTCCAGTGCAGTGTTTATCAATTCACACATTATGGACATTTTGAGCAGGACAATTTTTC

TGTTGTGTAGGACTGATCTCAACTAGCACCCCTCGTCCTCAGGCATTAAATTCCA

>4384 len=235 tot-seq=2

GGAAGTGCTGGGTAGAGAAGAGTGGGGTCCCTGGTGAGGGCTCCACCCTCAGGCCTATGC

CCATGGACCTAAGTGAGGACAGGCACTCCTGGTTTTGCACCCAAATGTTGCATTTTCTAA

GCCCACTCTGACCCACCATGCCCCACATCCTGGGCCCATAAAATCTTCAAGACCCTAGTG

GGCATGGACACAAGTGGCTGGACGTGGAGAGGAGCAGAAAAGCACACTGACTGAT

>4385 len=236 tot-seq=2

TTCTCTACAGTGGCTTTCTCTGCTTCCTGCAAACGATTCTCACTGCGAGGCTCTCAGAGT

TTCCCCTTGGGACTTTACACTTGAATAGACTTACCCATTAGTCATTTGCCTTCATATTCA

GTCAGGATTTTTATTTAGTCTAATTAGTCTTCCTTTTTCACACAACTAGAACAAACCTCA

ATCCTTATGAGGATGTGGTTATAACCTTCACACCTCTGTAGAATTGGTAGCTTCTG

>4386 len=236 tot-seq=2

GAAGGCAGTTGCTGCTGGGGAAAGCAATTCTGTCTCCTTCGGCAATCTCAGCAGTGGCCA

CTTGTCAGAAGAGCCGGCAGGGGAAAAAAATGACAAAAAAATCAGGTCCAATTTATCACC

TTCAAATTGTGTTGAATAAGCTACTGTAGCTACTTGGTTTTGAAGAACTCTCCCTTCTTC

CCCCTACACAGAACAAACACCACTAAATGCCTCTCCACCTGCGCAGCTGAGCGTTG

>4387 len=238 tot-seq=2

GGGTCTCTGGGCAGGTGAGGACATCAGTGCCACCTTTCCTTCCCCACTCTCTCAGGTCCG

GAGGTGACGCCAGGCCCTGGGGCCTACAGCCCAGAGAAGGTGCCCCCTGTGCGCCATCGG

ACACCGCCGGCTTTCACCCTGGGCTGCCGCCTCCCCCTGAAGCCCCTGGACACCTCAGCC

CCTGCCCCTAATGCCTACACCATGCCCCCTCTGTGGGGCTCACAGATCTTCACCAAGC

>4388 len=238 tot-seq=2

AGGTTGTAGAAATGCTCCTTGAGGCAGATGCCACACCCCAATTTCATGGAGTGATTTGGG

CTGAGCCGAGTCTGCAGCAGGCAGAAGGCTCTGAGATGTTGTCCTAGCCTGGGCAAAGGA

CAATTCAGAGCTCGGGGGAATAGGGGTGTGCTCAGCACGACTGGGTGGACGGGCCCTTTG

TAACAGAACACTTTGTGAGGGCCCTGCTGGAGGCCCAGGGTGGCTGTTCCTAACAATA

>4389 len=100 tot-seq=1

TAGGGAATCTTCCACAATGGACGAAAGTCTGATGGAGCAACACCGCGTGAGTGAAGAAGG

GTTTCGGCTCGTAAAGCTCTATTGGCAGGGAAGAAAGTTA

>4390 len=100 tot-seq=1

GGGTGGGAGCTCAACGGAAAGAGCAGAGCTAGAGTCAGAAAGGCAGGGGTGTTGATTTGT

CAATGGTCTTAACCGTGGCAGGCCAACAGAAGGAAAGGGG

>4391 len=100 tot-seq=1

GAGTGGTGATTAAAAAGCCAGAGAAAAAGAGTCTGCTGTAACAGTGTGCTTCTTAGAAAT

GTGAGAAGCAGCCGGGCAGTGGTGGCACACACCTTTAATC

>4392 len=100 tot-seq=1

GATCGCCATTGAGTGTAAGGTCAGGAGGAACTAAAGATCAAAACTCTGTCTCAAAAATAA

TAAGAATTGAGCTGGGCCGTGGTGACGCAGGCCTTTAATC

>4393 len=100 tot-seq=1

ATAGCAATGTACAGGCTGGCCAGTGCCTCGAATGCCACCCGTGACTGTGACAGACTCTGG

CCTGCAGAGCTGAAAGATAAGAGGGCCGTGACAATCTATC

>4394 len=100 tot-seq=1

TACTGATGACAGAGCAAATGCATATTCCTTTGGAGGGTGGTGAGGTGACTAAAATGTAAG

ATGCTGTTTGAGCTTGGACGCCATGCGGGCTCATGGAAGG

>4395 len=100 tot-seq=1

TACAGGCAGTTGTGAGCTTCCCTACGTGAGTGCTGAAAACTGAACTTGGGTCCTATGGAA

GAACAGGAAGCTCTCTTAACCACTTGTCATCTCTCCAGCC

>4396 len=100 tot-seq=1

CACCACCACCAACATACTACCACCAGCAGAAGCAGCAGCACCATCACCACCAGCAGCAGC

AGTAGCATAACCACCAGCAGCAACGGCAGCACCATCACCA

>4397 len=100 tot-seq=1

CCGCGTGAACGAAGAAGTTATTCGTAATGTAAAGTTCTTTTATCAGGGAAGAAAGAGGAA

TTTGACGGTAGCTTACCAGAAAGGGACGGCTAACTACGTG

>4398 len=100 tot-seq=1

CAGTGAATGGAAGCCCAACATCTAGATCAGTCCCACAAGGCAAGGAGGCGAAGAAGAGAG

AGAGTGAATCTTCCTTCTTTCAATGTCCTTATGTAGGTCT

>4399 len=100 tot-seq=1

TTATGTTCTCTCTGCATCGAATAGAAAAATGATGCCCTGCCTCCATTGGGAAGAAGAAAG

CAAAAAATATCAGTAATGAATTGTAAGATACAAACTGCCT

>4400 len=100 tot-seq=1

TGGGGAATATTGCACAATGGGGGCAACCCTGATGCAGCGACGCCGCGTGAGTGAAGAAGT

TATTCGTTATGTAAAGCTCTATTAGCAGGGAAGAAAATGA

>4401 len=100 tot-seq=1

TGTGAACATGATGATCAGGTACACTGTTCTTTCTCTCCATTAATGTTGTACCTCCATGTG

CTTGTCTTAGGGTGAAATACAAAGAGGACTGTGGCTCCCA

>4402 len=100 tot-seq=1

GACTCAGGTTTTATCAGCCTTGCTATCACAGCCCTGTCAAATCACACAAAGCAATCAGTA

ATGCTCGCCTTTCTCAGCCTGGAAGGAGGCAGTCACATCC

>4403 len=100 tot-seq=1

CAACCTGTTACTGTCCTTCATAGAGGTCTGGTCTCAGAGTCTTGCAGGAGGCAGAATCCA

CAGTTCCATCCAGGTTTCCATTTGGTTCTGCCTGGCTCTT

>4404 len=100 tot-seq=1

TAGGGAATCTTGCACAATGGACGCAAGCCTGATGGAGCGACGCCGCGTGAGTGAAGAAGG

GTTTCGGTTCGTAAAGCTCTATCAGTGGGGAAGAAAATGA

>4405 len=100 tot-seq=1

TAGGGGATATTGCACAATGGGCGCAAGCCTGATGGAGCGACGCCGCGTGAGTGAAGAAGG

ATTTCGGCTCGTAAAGCTCTATCAGTAGGGAAGAAAATGA

>4406 len=100 tot-seq=1

CCAGCAGCCGCGTAATTAGCAGATTGGAAGAGCGTCGTGTAGGGAAAGAGTGTGCCAATG

TGTAGATCTCGGTGGTCGCCGTATCATTAAAAAAAAAAAA

>4407 len=100 tot-seq=1

TAGGGAATCTTGCACAATGGACGAAACTCTGATGGAGCCACGCCGCGTGTGTGAAGAAGG

CCTTCGGATCGTAAAGCACTGTCGTTGGTGAGGAAGGAAA

>4408 len=100 tot-seq=1

TAGGGAATCTTCCACAATGGACGCAAGTCTGATGGAGCAACGCCGCGTGAGTGAAGAAGG

GCTTCGGCTCGTAAAGCTCTTTTGTTGGGGAAGAAAGGCA

>4409 len=100 tot-seq=1

TAGGGAATATTGCACAATGGAGGAAACTCTGATGCAGCGATGCCGCGTGTGTGAAGAAGG

ATTTCGGGTTGTAAAGCACTATCGGAGGGGAGGAAAAAAA

>4410 len=100 tot-seq=1

TAGGGAATATTGCACAATGGACGCAACTCTGATGCAGCCATGCCGCGTGTGTGAAGAAGG

CCTTCGGGTTGTAAAGCACTTTCGGAGGGGAGGAAGGAAA

>4411 len=100 tot-seq=1

TAGGGAATCTTCCACAATGGACGCAAGTCTGATGGAGCAACGCCGCGTGTGTGAAGAAGG

GTTTCGGCTCGTAAAGCACTGTTGGTGGGGAAGAAGAAAA

>4412 len=100 tot-seq=1

TGGGGAATCTTCCACAATGGGCGAAAGCCTGATGCAGCAACGCCGCGTGAGGGAAGAAGG

CTTTCGGGTCGTAAAGCTCTGTTGTTGGTGAGGAAGCATA

>4413 len=100 tot-seq=1

TGGGGAATATTGCACAATGGACGCAAGCCTGATGCAGCAACGCCGCGTGAACGAAGAAGG

TCTTCGGATCGTAAAGCTCTGTTGCTGGGGAAGAAAATGA

>4414 len=100 tot-seq=1

TGGGGAATATTGCACAATGGGCGCAAGCCTGATGCAGCAACGCCGCGTGAACGAAGAAGG

TCTTCGGATCGTAAAGCTCTGTTGCAGGGGAAGATAGAGA

>4415 len=100 tot-seq=1

TAGGGAATCTTCCACAATGGACGCAAGTCTGATGGAGCAACGCCGCGTGAGTGAAGAAGG

GTTTCGGCTTGTAAAGCTCTGTTGTCGGGGACGAAGGAGA

>4416 len=100 tot-seq=1

TAGGGAATCTTCCGCAATGGGCGCAAGCCTGATGCAGCAACGCCGCGTGAGCGAAGAAGG

GCTTCGGCTCGTAAAGCTCTGTTGCTGGGGAAGAAAGTGA

>4417 len=100 tot-seq=1

TAGGGAATCTTCCGCAATGGGCGCAAGCCTGATGGAGCAACGCCGCGTGAGTGAAGAAGG

TTTTCGGATCGTAAAGCTCTGTTGTTGGAGAAGAAGGGGT

>4418 len=100 tot-seq=1

TAGGGAATCTTCGACAATGGACGCAACTCTGATGCAGCAACTCCGCGTGTGTGAAGAAGG

TTTTCGGCCTGTAAAACACTTTTAGTAGGGAAGAAAGACA

>4419 len=100 tot-seq=1

TGGGGAATATTGCACAATGGGCGAAACCCTGATGCAGCGACGCCGCGTGAGCGAAGAAGG

GTTTCGGATCGTAAAGCTCTGTCCTTTGGGAAGAAAGTGA

>4420 len=100 tot-seq=1

TAGGGAATCTTCCACAATGGGCGAAACCCTGATGGAGCGACGCCGCGTGAGTGAAGAAGG

ATTTCGGCTCGTAAAGCTCTGTTGGTGGTGAAGATAGTGA

>4421 len=100 tot-seq=1

TAGGGAATCTTCCACAATGGACGCAACCCTGATGGAGCAACGCCGCGTGAGGGAAGAAGG

GTTTCGGCTTGTAAAGCCCTGTTGTTGGTGAAGAAGGTCA

>4422 len=100 tot-seq=1

TAGGGAATCTTCCACAATGGACGCAACTCTGATGGAGCAACGCCGCGTGAGTGAAGAAGG

GTTTCGGACTGTAAAACTCTTTTGGTGGGGAAGAAGAAAA

>4423 len=100 tot-seq=1

TTTTTTTTTTTTCAAGCAGAAGACGGCATACGAGATGTGCTCTTCCGATCTCCTACGGGA

GGCAGGAGCCGCGGTAATGACCAGATCGGAAGAGCGTCGT

>4424 len=100 tot-seq=1

TAGGGAATCTTCCACAATGGACGCAACTCTGATGGAGCAACGCCGCGTGAGTGAAGAAGG

TTTTCGGACTGTAAAGCTCTGTTGGTGGTGAAGAAGGATA

>4425 len=100 tot-seq=1

TTTTTTCAAGCAGAAGACGGCATACGAGATGCCTAAGCTGGAGTTCAGACGTGTGCTCTT

CCGATCTCTTACGGGAGGCAGCAGCCGCGGTAATCCCGAG

>4426 len=100 tot-seq=1

TAGGGAATCTTCCACAATGGGCGCAACCCTGATGCAGCGACGCCGCGTGAGGGAAGAAGG

TTTTCGGATTGTAAACCTCTGTTGTTGGGGAAGAAGGTTA

>4427 len=100 tot-seq=1

TAGGGAATATTGCACAATGGACGCAACTCTGATGCAGCAACGCCGCGTGAGTGAAGAAGG

TTTTCGGACTGTAAAGCTCTGTTGTTGGGGAAGAAGGATA

>4428 len=100 tot-seq=1

TAGGGAATCTTCCACAATGGGCGAAACCCTGATGGAGCGACGCCGCGTGAGGGAAGAAGG

ATTTCGGATCGTAAAGCTCTGTTGGTGGGGAAGAAAGTGA

>4429 len=100 tot-seq=1

TAGGGAATCTTCCACAATGGGCGCAAGGCTGATGGAGCAACGCCGCGTGAGTGAAGAAGG

GGTTCGGCTCGTAAAACTCTGTTGTTGGGGAAGAAAGACA

>4430 len=100 tot-seq=1

TGAGGAATTTTCCACAATGGACGCAAGTCTGATGGAGCAACACCACGTGAACGATGAAGG

TTTTCTGATTGTAAAGCTCTTTTATTTAGGAAAAAAAGCG

>4431 len=100 tot-seq=1

TAGGGAATATTCCACAATGGACGCAAGCCTGATGGAGCAACGCCGCGTGAGCGAAGAAGG

GCTTCGGATCGTAAAGCTCTGTTGCTGGGGAAGATGGTGA

>4432 len=100 tot-seq=1

TAGGGAATCTTCCACAATGGACGCAACTCTGATGGAGCAACGCCGCGTGAGTGAAGAAGG

GTTTCGGCTCGTAAAGCCCTGTTGTTGGGGAAGATGGAGA

>4433 len=100 tot-seq=1

TAGGGAATCTTCCACAATGGACGCAAGCCTGATGGAGCAACGCCGCGTGAGTGAAGAAGG

GTTTCGGCTCGTAAAGCTCTGTCCGCGGTGAAGAAGGACA

>4434 len=100 tot-seq=1

GAGCTCAGGAGTTCAAGGCCAGTCTGGGCAACATGGTAAAACCCTATCTCTACAAAAAAA

TTTGAAAATTAGCCAGGCGTGGTGGTGCATTCCTGTGGTC

>4435 len=100 tot-seq=1

GACGGCCCTATGGGATGTAAACTGCTTTTAGAGGGGAATAAAGTGAGGGACGTGTCCCTT

ATTGCATGTACCATAAGAATAAGGACCGGCTAAGTCCGTG

>4436 len=100 tot-seq=1

TGGGGAATCTTCCACAATGGACGCAACTCTGATCCAGCAACTCCGCGTGTGTGAAGAAGG

TTTTCGGATCGTAAAGCTCTTTTGGTGGGGAAGAAGAATA

>4437 len=100 tot-seq=1

TGGGGAATCTTCGACAATGGAGGCAACTCTGATGGAGCAATGCCGCGTGTGTGAAGAAGG

TTTTAGGACTGTAAAGCTCTTTTGGTGGGGAAGAAGGAAA

>4438 len=100 tot-seq=1

TAGGGAATCTTCCACAATGGACGCAAGCCTGATGGAGCAACGCCGCGTGAGTGAAGAAGG

GTTTCGGCTCGTAAAGTTCTGTTGGTGGGGAAGATAGTGA

>4439 len=100 tot-seq=1

TGGGGAATATTGCGCAATGGGCGAAACCCTGACCCAGCGACGCCGCGTGCGGGATGAAGG

CTTTCGGGTTGTAAACCTCTTTTGATTGGGAGCAAGCTTT

>4440 len=100 tot-seq=1

TAGGGAATCTTCCACAATGGGCGCAACCCTGATGGAGCGACGCCGCGTGAGTGATGAAGG

TCTTCGGATTGTAAACTCCTGTTGTTGGGGAGGATGGTTA

>4441 len=100 tot-seq=1

CTTATAGATTGTAAAGTTCTTTTATATGGGAAGAAGCACTAAGATAGGAAATGATTTTAG

TTTGACTGTACCAATTGAATAAGTAACGGCTAACTATGTG

>4442 len=100 tot-seq=1

TAGGGAATCTTCCACACTGGACGCAACCCTGATGCAGCAACGCCGCGTGAGTGATGAAGG

CTTTCGGATTGTAAAGCTCTGTTGTTGGTGAAGAAGGTTA

>4443 len=100 tot-seq=1

GGTCTTATAGATCGTAAAACTCTGTTGTAAAGGAAGAACACTTATTATAGGGAATGATAA

TAAGTTGACGGTACTTTACCAGAAAGCCCCGGCTAACTAC

>4444 len=100 tot-seq=1

TGGGGAATATTGCACAATGGGCGCAACCCTGATGCAGCAACGCCGCGTGAGTGAAGAAGG

CCTTCGGGTCGTAAAGCTCTTTTATTTGGGAAGAAAATGA

>4445 len=100 tot-seq=1

TAGGGAATATTGCACAATGGAGGAAACCCTGATGCAGCGACGCCGCGTGAGCGAAGAAGG

GTTTCGGGTCGTAAAGCTCTGTTCTTTGAGAAGATAATGA

>4446 len=100 tot-seq=1

TGGGGAATATTGGACAATGGACGCAACTCTGATGCAGCAACTCTGCGTGTGTGAAGAAGG

TTTTAGGACCGTAAAACTCTTTTAGTGGTGAAGAAAAATA

>4447 len=100 tot-seq=1

CACAGAGGGTCAAAGCATGAACCCCCAGATCCTAGATGTGAGGCCCGGCCTAGCTCCTTC

TTAGCTGCATGAGGTCAGGCAAGCACTAACAGTTTGAGTT

>4448 len=100 tot-seq=1

TGGGGAATCTTCGACAATGGACGAAACTCTGATGCAGCAACTCCGCGTGAGTGAAGAAGG

TTTTAGGACTGTAAAACACTTTTAGTGGGGAAGAAGAAAA

>4449 len=100 tot-seq=1

TGGGGAATATTCGACAATGGACGCAACTCTGATGCAGCAATGCTGCGTGTGTGAAGAAGG

TTTTAGGCTCGTAAAGCACTTTTAGTGGGGAAGAAAAACA

>4450 len=100 tot-seq=1

TAGGGAATATTGCACAATGGACGCAACCCTGATGGAGCAACGCCGCGTGAGTGAAGAAGG

GTTTCGGCTCGTAAACCTCTGTTGGTGGGGAAGAAAGACA

>4451 len=100 tot-seq=1

TAGGGAATCTTCCACAATGGACGCAAGCCTGATGCAGCAACGCCGCGTGAGTGAAGAAGG

TTTTCGGAATGTAAAGCTCTGTCAGTAGTGAAGAAGAAAA

>4452 len=100 tot-seq=1

CATGGGCCAGGGGAGGCACCCAGCCGGGCACACGAGCCACCACAATGCGGGACAAGGGAG

CAGGTGGTGAGGCGGGCACGGGGGAAGTGCAGTCGGTGAA

>4453 len=100 tot-seq=1

TGAGGAATATTGGTCAATGGGCGCGAGCCTGAACCAGCCAAGTAGCGTGTAGGAAGACGG

CCCTATGGGTTGTAAACCGCTTTTATGTGGGGGTAAAGTG

>4454 len=100 tot-seq=1

TCTTCGGATTGTAAAGCACTTTAAGTTGGGAGGAAGGACAGTAAGCGAATACCTTGCTGT

TTTGACGTTACCGACAGAATAAGCACCGGCTAACTCTGTG

>4455 len=100 tot-seq=1

CAGCAGCCCGGACAAGACCAGCAGCCAGGATAAGAACAGCTGGCCCAGCAGCCCATACAA

GACCAGCAGCCCAGACATAGCCCAGCAGCCCAGACATAGC

>4456 len=100 tot-seq=1

CCTCTGCCCCAGGCCCTTTTCCAGCTGTGTGCCCTCCTGACTGCCTAGGGAAGGTGACCA

TGGCACGGGCAGCCTGCTCTGAGCCAGGCACCCACTGCGC

>4457 len=100 tot-seq=1

GAACAAGATTGAATTAAGATCACATTTTTCCGGAACGATGCCGTTCTCACGCAAGAAGTT

GGCCAGAATGGTCGCCGGCACGCCGAACTCGGTGTACTGG

>4458 len=100 tot-seq=1

AGGAAGAGAAGAGGAGGGCAGGCAGGGTGGGGGGCACGGGGGCCAGGCACTTACGTTGGT

GAAGAAGAACAGGACCCCAGTGAAGAGCGTAATGACCTCG

>4459 len=100 tot-seq=1

GAGGAAAGGCAGGCCCACGCGCAGCCACTGGACTGCTTGGCAAATGCTCAGGGCAGAGGG

GGTCCCCAGGCCCACTGCAGAAAGGCTCTGTGAGGGGCCA

>4460 len=100 tot-seq=1

GTTTCGGCTCGTAAAGCTCTTCTGGTAGTGAAGAAAGATACAGTTAGTAACTGGCCGTTA

TTTGACGGTAATTACTTAGAAAGTCACGGCTAACTACGTG

>4461 len=100 tot-seq=1

AACAAAGACTATCATTTAGCCAGCAACTGTGGAGGTGGCTCTTGAGTAGGCAGTCTGACC

CGGGGCCATGCCCCTCATGGCCTCGCAGTTGCCTACTGGG

>4462 len=100 tot-seq=1

TAGGGAATATTCGACAATGGACGCAACTCTGATCCAGCAATTCCGCGTGTGTGAAGAAGG

TTTTCGGACCGTAAAACTCTTTTAGTGGGGAAGAAAGAAA

>4463 len=100 tot-seq=1

GTGACAATAAATACTGATACGAGGCTCTTTTGGGCCTTGTAATTGGAATGAGTACAATTT

AAATCTCTTAACGAGGAACAATTGGAGGGCAAGTCTGGTG

>4464 len=100 tot-seq=1

TCTTCGGATTGTAAAGTTCTGTTATTAGTGAAGAACATTTGCAATAGGAAATGATTGCAG

ACTGACGGTACCTTGTCAAAAAGCGATGGCTAACTATGTG

>4465 len=100 tot-seq=1

TGGGGAATATTGCACAATGGAGGGAACTCTGATGCAGCGACGCCGCGTGAGTGAAGAAGT

ATTTCGGCTCGTAAAGCTCTGTTGGTAGTGAAGAAAGATA

>4466 len=100 tot-seq=1

GTTTCGGTTCGTAAAGCTCTGTTGTTGGAGAAGAACGGGCGTGAGAGTAACGGTTCACGC

AGTGACGGTATCCATCCAGAAAGTCACGGCCAACTACGTG

>4467 len=100 tot-seq=1

AGCGACGCCGAGTGAGCGAAGAAGTATTTCGGTATGTAAAGCTCGATAAGAAGGGAAGAA

AATGACGGTACCTTACGAAGAAGCTCCGGCTAAATACGTG

>4468 len=100 tot-seq=1

CTACGTGCCAGCAGCCGCGGTAATACGTAGGGCGCGAGCGTTGTCCGGATTTATTGGGCG

TAAAGAGCTCGTAGGTGGTTAGTCGCGTCTGTCGTGAAAG

>4469 len=100 tot-seq=1

GTCAGGGGCTTCCCAGAGCCCTCCCTTCTAGGCAGGGGCAGGAGGTGACTCTCCCAAGGT

GCCCCAGGAGAAGCAGAAACCAGAGGAAGAGGCCAGACAT

>4470 len=100 tot-seq=1

GGAAGAAGCTGGAGCCGCCTCCCCCACCACAACCACAGCAGCCTGGATACAGGAACCCGA

ATGTCACTCAGTTCAGACTGAACTGGCTCTGCTGCCCACC

>4471 len=100 tot-seq=1

TTTCGGATCGTAAAGCTCTGTTTTAAGAGAAGAACGTGTGTGAGAGTGGAAAGTTCACTC

AGTGACGGTAACTAGCCAGAAAGGGACGGCTAACTACGTG

>4472 len=100 tot-seq=1

TTGTCAGGTCCCAGATATGAGACAAAAAAGACTCAGGGAAAGTTCCTAGATTTCTGATTT

GAGTACGTAAATGGTATTATCATTTACTGAGCTGTAGAAT

>4473 len=101 tot-seq=1

CAACGCCGCGTGAATGATGAAGGTCTTCGGATGGTAAAGTTCTTTACTAAGGGGAGAAGA

TAGTGACGGTACCTTAGGAATAAGCCTCGGCTAACTACGTG

>4474 len=101 tot-seq=1

TTTTCGGATCGTCCAGCTCTGTTGTAAGTCAAGAACGAGTGTGAGAGTGGAAACTTCACA

CTGTGCCTTTGGCTTACCTGCCTTTGACGGCTAACTACGTG

>4475 len=101 tot-seq=1

CAGCCATGCCGCGTGTCTGAAGAAGGTCTTCGGATTGTAAACTCCTGTTGTTGAGGAAGA

TAATGACGGTACTCAACAAGGAAGTGACGGCTAACTACGTG

>4476 len=101 tot-seq=1

TGGCGAGAACGAGGGCACTGCTGGGTCTGCTGGGCCCTGCAAGGGCCCCCCAGTAAGTC

CTCTGCCAGTGCCAAGACCACCGAACAGAAGCTCAATAGCC

>4477 len=101 tot-seq=1

TGGGGAATATTGCACAATGGGCGCAACCCTGACGCAGCCACGCCGCGTGCATGAAGAAGG

TCTTCGGATTGTAAACTGCTTTCGCCAGGGAGGAAGACAGT

>4478 len=101 tot-seq=1

TGGGGAATATTGCACAATGGGGGCAAGCCTGATGCAGCCATGCCGCGTGAGTGAAGAAGG

ATTTCGGGATGTAAAGCTCTTTCAGCAGGGAGGAAAATGAC

>4479 len=101 tot-seq=1

TAGGGAATATTCGGCAATGGGGGCAACCCTGATCGAGCAACGCCGCGTGTGTGAAGAAGG

TCTTCGGATTGTAAAGCTCTGTCGTAAGTCAAGAACGAGTG

>4480 len=101 tot-seq=1

TGGGGAATTTTCCGCAATGGGCGCAAGCCTGACGGAGCAATACCGCGTGAGGGATGACGG

CCTTCGGGTCGTAAACCTCTTTTATTAAGGAGGAAGGTCTG

>4481 len=101 tot-seq=1

TAGTGACAATAAATAACAATACCGGGCTCCTCGAGTCTGGTAATTGGAATGAGCACAATC

TAAATCCCTTAGCGAGGATCCATTGGAGGGCAAGTCTGGTG

>4482 len=101 tot-seq=1

TAGGGAATCTTCGGCAATGGGGGCAACCCTGACCGAGCAACGCCGCGTGAGTGAAGAAGG

GTTTCGGCTCGTAAAACTCTGTTGTTGAAGAAGAACGTGCG

>4483 len=101 tot-seq=1

CACTCCAAGAGAGTGAACTGCTTGTTATACAGCTTGGTTTAAAAACTACATAGGAAAACA

AAACCTCCTGAGGGTGGTAACCCTTTGTTCCTCGTTGGCTC

>4484 len=101 tot-seq=1

TACTTTTTGGTGTGATAGTCCTCAAAGCCAGCCAGCAACCGCGGTAATTAGCAGATCGGA

AGAGCGTCGTGTAGGGAAAGAGGGTGCCAATGTGTAGATCT

>4485 len=101 tot-seq=1

TAGGGAATCTTCCACAATGGGCGCAACCCTGATGCAGCAACACCGCGTGAGTGAAGAAGG

GTTTCGGCTCGTAAAGCTCTGTTGGTTGGGAAGAAAGTGTC

>4486 len=101 tot-seq=1

TAGGGAATCTTCGGCAATGGACGCAAGTCTGACCGAGCAACGCCGCGTGAGTGAAGAAGG

TTTTCGGATCGTAAAGCTCTGTTGGCAGAGAAGAAGGAGTG

>4487 len=101 tot-seq=1

TAGGGAATCTTCCACAATGGACGCAAGTCTGATGGAGCAACGCCGCGTGAGTGAAGAAGG

GTTTCGGATCGTAAAGTCCTGTTGTTGGGGAGGAAGGACAG

>4488 len=101 tot-seq=1

TGGGGAATCTTGCGCAATGGACGAAACCCTGACGCAGCGACGCCGCGTGAGGGATGAAGG

CCTTCGGGTTGTAAAGCGCTGTTGATCGGGACGAATGGTCC

>4489 len=101 tot-seq=1

TAGGGAATCTTCCACAATGGGCGAAACCCTGATGCAGCAACGCCGCGTGAGCGAAGAAGG

TTTTCGGATCGTAAAGCTCTGTTGATGGGGAAGAAGGATTG

>4490 len=101 tot-seq=1

TGGGGAATATTGGACAATGGAGGGAACTCTGATCCAGCAACTCCGCGTGTGTGATGAAGG

TTTTAGGACTGTAAACCGCTTTTAGTTGGGAAGAAAAAAAT

>4491 len=101 tot-seq=1

TGGAGGCCTTCGGGTTGTAAACCTCTTTCGCCAGTGAAGCAGGCCTGCTCCTTGTGGGTG

GGTTGACGGTAGCTGGATAAGAAGCGCCGGCTAACTACGTG

>4492 len=101 tot-seq=1

TAGGGAATCTTCCACAATGGACGCAAGCCTGATGCAGCAACGCCGCGTGAGTGAAGAAGG

CTTTCGGCTCGTAAAGTTCTTTTGGTGGTGAGGAAGGAATG

>4493 len=101 tot-seq=1

TTTTTTTCAAGCAGAAGACGGCATACGAGATAGGTTCGTGACTGGAGTTCAGACGTGTGC

TCTTCCAATCTCCTACGAGAGGCAGCAGATCGGAAGAGCGT

>4494 len=101 tot-seq=1

CGGTAATCAACCAGAAAGTCACGGCTAACTACGTGCCAGCAGCCCGCGGTAATGCTCAGA

TCGGAAGAGCGTCGTGTAGGAAAGAGTGTGCCAATGTGTAG

>4495 len=101 tot-seq=1

CCCAGCAGCCCAGCGCCGCGGTAATTTTTAGATCGGAAGACGTCGTGTAGGGAAAGAGTG

TGCCAATGTGTAGATCTCGGTGGTCGCCGTATCATTAAAAA

>4496 len=101 tot-seq=1

TAGGGAATATTCCACAATGGACGAAACCCTGATGGAGCAACGCCGCGTGAGTGAAGAAGG

TTTTCGGTTCGTAAAGTTCTGTCGTTGGTGAAGAAGGATAG

>4497 len=101 tot-seq=1

TAGGGAATCTTCCACAATGGACGCAAGTCTGACGGAGCAACGCCGCGTGAGTGAAGAAGG

TTTTCGGATCGTAAAGCTCTGTTGATGGGGAAGAAGGAGAG

>4498 len=101 tot-seq=1

TAGGGAATCTTCCGCAATGGGCGCAAGCCTGATGGAGCAACGCCGCGTGAGTGAAGAAGG

TTTTCGGTTCGTAAAACTCTGTTGTTAGAGAAGAACATCAC

>4499 len=101 tot-seq=1

TGCCTGGAGAATCCCAGGGATGGCAGAGCCTGGTGGGCTGCCGTCTGTGAGGTTGCTCAG

AGTCGGACACGACTGAAGCGACTTAGCAGCAGCAGCAGCCG

>4500 len=101 tot-seq=1

GATAATAAACAAGACAAGCAGGATCAGGACCAGCAACAACAGCAAAACCAGCAACAACAG

CCTGATCAACAGAATCCTCAGGATAAAAACGAAGATAAAGA

>4501 len=101 tot-seq=1

GGGACTGGCATGTCAGCATTGCACTGAGTGTGTGTATACCTGGCCAGGCCTTGACAGTGC

CTGGGCTTGGCCCCAACTTTGCTCCAAGATCAAAGTGGGTG

>4502 len=101 tot-seq=1

CAAGAGACAGTTGTCTTGGAGAGAGAAAATGGATTGTTTTCCAAGTTCTTACCCTGCTTG

GTGCCACTTATTTGCCAGGAACTACATTCCGCCAGCTCCCA

>4503 len=101 tot-seq=1

TAGGGAATCTTCGGCAATGGGGGCAACCCTGATCCAGCCATGCCGCGTGAGTGATGAAGG

CCTTCGGGTTGTAAAGCTCTTTTGGCGGGGACGATGATGAC

>4504 len=101 tot-seq=1

GTCCAGATGAAAGATTTTTATAAGTGGTCCTGGCAAGAGATGATGGGGACTTGGGTTAAG

ATGGTAGAACCAAGAGGACTTGCTGAGGAACTGGATTTTGG

>4505 len=101 tot-seq=1

GCTCACAGGCCAACCCGAGTTCTGGGTGGGGGCAGGCTCGGCAGGCCCCACACTCAGAGT

GGGCCGCCTGGCCCTGGGCAGTGAGGGGCACAGCACCTTGG

>4506 len=101 tot-seq=1

CATGTGCAGGAAAGCAGGCATTCCCAGAAACCCCAGAAAGAAGTGGCAGCCAAGAAACAG

GCCCCAGGAGCACGACGGGGACACAGCCTCCTCAACCATTC

>4507 len=101 tot-seq=1

CTTGGTAGAACTGGAAGTTGGAGAACAGTGAGGTAGACACAACCAGAGTCTGGAACAGGG

GCCCTTTCTTTCCCAGACGAGTTTCCCCTCGCTTTTCTGGA

>4508 len=101 tot-seq=1

GGTTTCGGCTCGTAAAGCTCTGTTGTTAGTGCAGAAGGACAGGGGTAGTAACTGACCTTT

GTTTGACGGTAATCAATTAGACACTCACGGCTAACTACGTG

>4509 len=101 tot-seq=1

GTAGGTTTTCTAGTCAAAAGCATCAGCATTGGTTTGGCGGACAGCCAGCATTCACCACCA

GACAAGGGAGTGGTGATTCCATGCTCAACCCTTTGTGTCTT

>4510 len=101 tot-seq=1

CATTCGCGCTCTTAAGAGGCTTGGTAGCTGGGCATGTATCTGGAACAGTAACCTGAGCCA

TTGCTGGAGCAGCAAAAGCGAAACCGGAGAGGAGGGTGGCG

>4511 len=101 tot-seq=1

TAGGGAATCTTCCACAATGGACGCAAGCCTGATACAGCAACGCCGCGTGAGTGAAGAAGG

TTTTCGGATTGTAAAGCTCTGTTGTTGGTGAAGAAGGCTAG

>4512 len=101 tot-seq=1

GGGACGAAAAATAACAAGACAGGACGCGGGCGAGGCCCGGGAAGGGGAAGGAGTCCACTG

TAAATCCTTTAACGAGGATCCATTGGAGGGCAAGTCTGGTG

>4513 len=101 tot-seq=1

TGGGGAATCTTGCACAATGGGCGCAACCCTGATACAGCCACGCCGCGTGCATGAAGAAGG

CCTTCGGGTCGTAAAGTACTTTCAGCGGGGAGGAAGGGAGT

>4514 len=101 tot-seq=1

CAAGGGAGGCTGTGGCAGGGTAGGCTTTCTTCAAGGTGCATGAAGATGTGGGGCCCTGTA

ATCAGGGGACAGGGTCACTATCAGTGGCTCATACCTTGGCC

>4515 len=101 tot-seq=1

CGGATGCCGGCCCACCTCCCCACCCCACTGCCTCCACTCAAGGTTGGGGCAGTGGGGGAC

AGGACGTGGGGCAGCGGACTCAGTGTCGGGAGGAGCAGTTG

>4516 len=101 tot-seq=1

TCCAAACAAACCTACAATCGAAGTAAAGCAGCGCGCTTTCAATAGCGAGGCAAGGTCCCT

TGCTCCAACCAGAAACGGGATGATACCTGGTGAACCCCCAT

>4517 len=101 tot-seq=1

GGTGGCCGAGAAAAACAGGGTGGATCGGCCCTGGTCCAGGCATTCGCGCAGGTTCACCGA

TGGGTTTATGCACATCAGCTTGACCAGAAAGCTGCCGTCCT

>4518 len=101 tot-seq=1

AAGACGGCATACGAGATAACCGAGTGACTGGAGTTCAGACGTGTTCTCTTCCGATCTCCT

TCGGGAGGCAGCAGCCGGGTAATAAATAGATCGGAAGAGCG

>4519 len=102 tot-seq=1

CTTAGCCTTAGATGACCCTAGGGATGGGCTATGTAAGCCCATCTCTAATCTCCAAGCCTG

GATTCTCAGGTACCCAGAATATTTGGAAGGGAGTCCACCCAT

>4520 len=102 tot-seq=1

AAGTGGAACTTGCAGAGGTGACTTAGCCAGAAACAGGTAGAGCTGGGGCTCTGAACAGAA

AGATTATGATCTACTGTCAGGGATGGTGGCCATACCATGATC

>4521 len=102 tot-seq=1

CTTGCTCCCACCAAGCCACCGGCTAACAGGATGAAGTTCCCAAGCAAGGCGTGCATCTTG

TTGGGACAGCAGTCAGAGCCCTGGCCCGGTCTCCCTTCACAG

>4522 len=102 tot-seq=1

GTTTTCGGATCGTAAAGCTCTGTTTGAAGAGAAGAACGAGTGTGAGAGTGAAAAGTTCAC

ACTGTGACGGTAACTTACCAGAAAGGGACGGCTAACTACGTG

>4523 len=102 tot-seq=1

ATGACTGCCCTATGGGTTGTAAACTTCTTTTATGCGGGAATAAAGTGGCCCACGTGTGAA

TTTTTGTATGTACCATATGAATAAGGATCGGCTAACTCCGTG

>4524 len=102 tot-seq=1

TAGGGAATCTTCGGCAATGGACGGAAGTCTGACCGAGCAACGCCGCGTGAGTGAAGAAGG

TTTTCGGATTGTAAACTTCTGTTATAAAGGAAGAACGGCGGC

>4525 len=102 tot-seq=1

AACAGCCCCAGATTGGGAGCATGCAAGTGAGCTTTAGCCACGCGAAAGGGTCTGGCCTCA

CCTTGTCTTGCACCTTCCGGGCCATCCAGCACTTGGACACAC

>4526 len=102 tot-seq=1

TAGGGAATCTTCGGCAATGGACGGAAGTCTGATCGAGCAACGCCGCGTGAGCGAAGAAGG

ATTTCGGATCGTAAAGCTCTGTTGTTAGAGAAGAAAGTTGGT

>4527 len=102 tot-seq=1

TAGGGAATCTTCCACAATGGACGCAAGCCTGATGGAGCAACGCCGCGTGAGGGAAGAAGG

GTTTCGGCTCGTAAACCTCTGTTGTTGGGGAAGAAAGAAAGG

>4528 len=102 tot-seq=1

TTTTTTCAAGCAGAAGACGGCATACGAGATGATCTGGTGACTGGAGTTCAGACGTGTGCC

TTTTTGTATGTACCGTATGAATAAGGATCGGCTAACTCCGTG

>4529 len=102 tot-seq=1

TAGGGAATCTTCCACAATGGACGAAAGTCTGATGGAGCCACGCCGCGTGGGTGAAGAAGG

CCTTCGGGTCGTAAAGCACTTTTGGTGGTGAGGAAAGAAAGG

>4530 len=102 tot-seq=1

TAGGGAATATTGCACAATGGAGGAAACTCTGATGCAGCCACGCCGCGTGTGTGAAGAAGG

CTTTCGGGTCGTAAAGCTCTTTCGGTGGGGAGGAAAAAAATG

>4531 len=102 tot-seq=1

TGGGGAATCTTGCACAATGGGCGCAAGCCTGATGCAGCAACGCCGCGTGAATGAAGAAGG

TTTTCGGATCGTAAAGCTCTGTTGCTGGGGAAGATAGTGACG

>4532 len=102 tot-seq=1

TGGGGAATATTGCACAATGGGCGAAAGCCTGATCCAGCAATGCCGCGTGAGTGATGAAGG

CCTTAGGGTTGTAAAGTTCTTTCACCGATGAAGATAATGACG

>4533 len=102 tot-seq=1

TAGGGAATCTTCCACAATGGACGCAAGCCTGATGGAGCAACGCCGCGTGAGTGAAGAAGG

TTTTCGGCTCGTAAAACTCTGTTGTTGGAGAAGAACGACTGG

>4534 len=102 tot-seq=1

CGCGTGGAGGATGGAGGCCTTCGGGTTGTAAACATCTTTTGTTAGGGAGCAAGGCACTTT

GGGTTGAGTGTACCTTTCGAATAAGCACCGGCTAACTACGTG

>4535 len=102 tot-seq=1

TGGGGAATATTGGGCAATGGGCGAAAGCCTGATCCAGCCATGCCGCGTGTGGGAAGAAGG

CCTTCGGGTTGTAAAGCGCTTTTAGTTGGGAGGAAGGGCATT

>4536 len=102 tot-seq=1

TGAGGAATATTGGTCAATGGGCGAGAGCCTGAACCAGGAATAAAGTGAGGGACGTGTCCC

TTATTGCATGTACCATATGAATAAGGACCGGCTAATTCCGTG

>4537 len=102 tot-seq=1

GTTGCTGGAGCCAGGCTTTGAATGATGAGGAAGCCCTTTGCTAGATGGTGAGCAGGGAGG

AAGGCATTCTGGGCCCTTGCTATTTAGTGTGTGGTTTCTGGA

>4538 len=102 tot-seq=1

TTTTTTTTTTCAAGCAGAAGACGGCATACGAGATTACGTTGTGACTGGAGTTCAGACGTG

TGCGAGGCAGCAGCCGCGGTAATACTTAGATCGGAAGAGCGT

>4539 len=102 tot-seq=1

TGGGGAATATTGGACAATGGAGGCAAGTCTGATCCAGCAATGCTGCGTGTGTGAAGAAGG

TTTTCGGACTGTAAAACACTTTTAGTAGGGAAGAAAAAAATG

>4540 len=102 tot-seq=1

AGCCCAGCCTCAGGATCTGCAATCTCAGACAGAATCGCTTTTGCGGCTGGCAAGGTAGGG

CTAGGCAGCAGGCACAGGTGCCATTTCACAGATTACCTCGGG

>4541 len=102 tot-seq=1

TAGGGAATCTTGGGCAATGGACGGAAGCCTGACCCAGCAACGCCGCGTGAGTGAAGAAGG

CTTTCGGATTGTAAAGCTCTGTTGGTAGGGAAGAACGTTGTT

>4542 len=102 tot-seq=1

CAACAGGCACAAGCTCTGTACAAGTGTGCTGCAGGAACAATCCAGTGAGGAATGCCAGTC

CTGACAGTGAGGGACAAGCCATGGGGGTCTCTGTCCCCTTCT

>4543 len=102 tot-seq=1

GGCTGGAGGGCTCTTACTCATGAGAACACAGAGAAGGAGCAGGTGGAAAGGCGAGAAGAA

AACCCAGGGAGCTGAGAAGAGGAAGTGGTATTTTCAACAAAC

>4544 len=102 tot-seq=1

GGTTTTCGGATCTTACAGCTCTGTTGTTGGTGAAGGAGGATAGAGGTAGTAACTGGCCTT

TATTTGACGGTAATCAACCAGGAATTCACGGCTAACTACGTG

>4545 len=102 tot-seq=1

TTCACCTCTTGTCTGTGGTACCTCTTCTAGGAGCTGCACCTTCCCATCAGTGCTTCAAGA

GGTATCTGACTTGATATTTTCTGTGGGTCCTGGACATTCACC

>4546 len=102 tot-seq=1

ACCGGAGGGTGTGGGGCTGGCCAGGCTCCCCTGGGCACCAGGCGTGTGACCCCCGCTTCT

CCCAGCTCCTCCTCTCATCCCCAGCTGGGCTCATCCCAAGTC

>4547 len=102 tot-seq=1

GACAGAAGAGGAACAGAAGCAAACACACAGAGAAGAAGGTGATGTAAGGACAGAGGCAGG

GATTGGAGAGCTGTGCTATAGGCCAAGGAGAGCAAAGGAGTG

>4548 len=102 tot-seq=1

TAGGGAATCTTCCACAACGGACGAAAGTCTGATGGAGCAACGCCGCGTGAGTGAAGACGG

TTTTCGGATCGTAAAGCTCTGTTGTTGGTGACGAAGGATAGA

>4549 len=102 tot-seq=1

CATTTACTTTCCTCTAAGTTGCCTACATCACCCCTCCCCTCTCCCCTGGGAAGAAGGTAC

ATACAAGCTCCAACCCCTTCCCTTCTGGGAATCCCCAAGTTA

>4550 len=102 tot-seq=1

CATACGAGATCCGTAGGTGACTGGAGTTCAGACGTGGGCTCTTCCGATCTCCTACGGGAA

GCAGCAGTAGGACCTTTCGAATAAGCGCCGGCTAACTACGTG

>4551 len=102 tot-seq=1

TGGGGAATCTTCGACAATGGGCGCAAGCCTGATCCAGCAATACCGCGTGGGTGAAGAAGG

CTTTCGGGTTGTAAAGCCCTTTTGTTGGGAAAGAAAGGCAGT

>4552 len=103 tot-seq=1

GGGAATTTACCAGAGCTCCTACTTAGTTGCTTTTTTTCACCTTGATCCCAAATCAGCAGC

CTGCATTTCAGCAAAGGGCATGGGGCCAGCCCCATCTCAGGTC

>4553 len=103 tot-seq=1

CGGGCCTATATGTGTGCACAGTTCCTACGGGCCCAGCCTTCATGGGAACAGGCACCCTGG

ATCCCTGATGGGCTCTTGATGGTAACCGAATAAGAGCGATGAG

>4554 len=103 tot-seq=1

CAGGTGAAGGCAGAACGCTTTGTTGCTACTGTTGTTATTTGGGGGCAGAATCTCACTGTT

GCTCAGCTGTCCCCCACCTCCTAGGCTTAAGCACTGGTGTGGA

>4555 len=103 tot-seq=1

AGAGGACACCAGACATCCCCAACAGTGGCAGCTCCCAGGCAGAGAGCCCGGCTGTGAGTG

CCATGTTGCATGCCATCGCTGCCAGCCACCTGCCTGTGTGCAG

>4556 len=103 tot-seq=1

TAGGGAATCTTCCACAATGGACGCAAGTCTGATGGAGCAACGCCGCGTGAGTGAAGAAGG

GTTTCGGCTCGTAAAGCTCTGTTATATGGGACGAACAGGACAT

>4557 len=103 tot-seq=1

TGGGGAATCTTCCACAATGGACGAAAGCCTGACGGAGCAACGCCGCGTGAGTGAAGAAGG

TCTTCGGATTGTAAAGCTCTGTTGTACGGGAAGAAAGAGACGG

>4558 len=103 tot-seq=1

TGGGGAATATTGCACAATGGGCGGAAGCCTGATGCAGCGATGCCGCGTGTGGGATGAAGG

CCTTCGGGTTGTAAACCACTTTCAGCAGGGAAGAAGCCACAAG

>4559 len=103 tot-seq=1

TAGGGAATCTTCCACAATGGGCGCAACCCTGATGGAGCAACACCGCGTGAGTGAAGAAGG

GTTTCAGCTTGTAAAGCTCTGTTGTTGGAGAACAAGGTGTGTG

>4560 len=103 tot-seq=1

TAGGGAATCTTCCACAATGGACGCAACTCTGATGGAGCAACGCCGCGTGAGTGAAGAAGG

TTTTCGGATCGTAAAGCCCTGTTGTCGGTGAAGAAGGATAGGG

>4561 len=103 tot-seq=1

TGGGGAATATTGCACAATGGGGGAAACCCTGACCCAGCAACGCCGCGTGCATGATGAAGG

CCTTAGGGTTATAAAGTTCTTTCACCTGTGAAGAAAATGACGG

>4562 len=103 tot-seq=1

TGGGGAATCTTGCACAATGGGCGCAAGCCTGATGGAGCAACGCCGCGTGAGCGATGAAGG

TTTTCGGATCGTAAAGCTCTGTTGTTGGGGAAGATGATGACGG

>4563 len=103 tot-seq=1

TGGGGAATATTGCGCAATGGGGGAAACCCTGACGCAGCGACGCCGCGTGCGGGATGAAGG

CTTTCGGGTTGTAAACCTCTTTTGTTGGGGAGCAAGGTTTCGG

>4564 len=103 tot-seq=1

TTTCATGGAGCCCAGAAATAAGTTCTGCGGCATATGCAAATGTAAGCAATGGGGGCAGCA

GAACGGGCCTCTAGGTTCAAAAATGGCAGTTGTCTCCAAGAGG

>4565 len=103 tot-seq=1

TAGGGAATCTTCCGCAATGGACGCAAGCCTGACGGAGCAACGCCGCGTGAGTGAAGAAGG

TTTTCGGATCGTAAAACTCTGTTGTCAGAGAAGAACATATCGT

>4566 len=103 tot-seq=1

TAGGGAATCTTCCGCAATGGACGCAAGTCTGACGGAGCAACGCCGCGTGAGTGATGACGG

TCTTCGGATTGTAAAGCTCTGTTAATCGGGAAGAACGGTCGGT

>4567 len=103 tot-seq=1

TACGGGAGGCAGCAGTAGGGAATATTGCACAATGGGCGCAAGCCTGATGCAGCCATGCCG

CGTGTATGAAGAAGGGCTTCGGGTTGTAAAGTACTGTCGGCAG

>4568 len=103 tot-seq=1

TAGGGAATCTTCCGCAATGGACGGAAGTCTGACGGAGCAACGCCGCGTGAGTGCAGAAGG

TTTTCGGCTCGTAAAGCTCTGTTGTTGGTGAAGAACGACAGGG

>4569 len=103 tot-seq=1

TAGGGAATCTTGCACAATGGACGAAACCCTGATGCAGCAACGCCGCGTGCGGGAAGAAGG

TCTTCGGATTGTAAAGCTCTGTTGTTGGTGAAGAAGGATAGTG

>4570 len=103 tot-seq=1

TAGGGAATCTTCGGCAATGGGCGCAACCCTGACCGAGCAACGCCGCGTGAGTGAAGAAGG

TTTTCGGATCGTAAAGCTCTGTTGTTAGAGAAGAACGTGAGGG

>4571 len=103 tot-seq=1

TAGGGAATCTTCCACAATGGGGGCAAGCCTGATGGAGCGACGCCGCGTGAGCGAAGAAGG

GTTTCGGCTCGTAAAGCTCTGTTCTATGAGAAGAAAGTGACGG

>4572 len=103 tot-seq=1

TGGGGAATATTCCACAATGGACGAAAGTCTGATGCAGCAACGCCGCGTGAGGGAAGAAGG

TCTTCGGATTGTAAAGCTCTGTTGTTGGGGAAGAAGGATAGAG

>4573 len=103 tot-seq=1

TCAAGCAGAAGACGGCATACGAGATGCCTAAGCAGACGTGTGCTCTTCCGATCTCTTACG

GGAGGCAGCAGTCAAGCCCAACAACCAGCACCCACCCCAACCT

>4574 len=103 tot-seq=1

TAGGGAATCTTCCACAATGGACGCAAGTCTGATGGAGCAACGCCGCGTGAGGGAAGAAGG

GTTTCGGCTCGTAAAGCTCTGTTGGCAGGGAAGAAAGAGAGGG

>4575 len=103 tot-seq=1

TAGGGAATCTTCCACAATGGACGCAAGCCTGATGGAGCAACGCCGCGTGAGTGAAGAAGG

GCTTCGGCTCGTAAACCCCTGTTGTTGGGGAAGAAGGGCAGGG

>4576 len=103 tot-seq=1

TAGGGAATCTTCCACAATGGATGCAAGTCTGATGGAGCAACGCCGCGTGAGTGAAGAAGG

GCTTCGGCTCGTAAAGCACTGTTGGTGGGGAAGAAGGACAGGG

>4577 len=103 tot-seq=1

TAGGGAATCTTCCACAATGGGCGCAAGCCTGATGGAGCAACGCCGCGTGAGCGAAGAAGG

TTTTCGGATCGTAAAGCTCTGTCGTTGGAGAAGATGGTGAGAG

>4578 len=103 tot-seq=1

TAGGGAATATTCCACAATGGACGAAACCCTGATGCAGCAACGCCGCGTGAGTGAAGAAGG

TTTTCGGATTGTAAAGCTCTGTTGTTGGTGAAGATGGTTAGAG

>4579 len=103 tot-seq=1

AAGGTTTCGGATCGTAAAGCTCTGTTGTTGGAGAAGAAGGAGAGAGATAGTAACTGGCCA

TTATTTGACGGTAATCAACCAGAAAGTCACGGCTAACTACGTG

>4580 len=103 tot-seq=1

TAGGGAATCTTCCACAATGGACGCAAGCCTGATGGAGCAACGCCGCGTGAGCGAAGAAGG

TTTTCGGATCGTAAAGCTCTGTTGTTGGTGAAGAAGGTTACGG

>4581 len=103 tot-seq=1

TAGGGAATATTCCACAATGGGCGCAACCCTGATGGAGCGACGCCGCGTGAGTGAAGAAGG

TTTTCGGATCGTAAAGTTCTGTTGTTGGGGAAGAAGGATAGAG

>4582 len=103 tot-seq=1

TGGGGAATCTTGCACAATGGGCGCAAGCCTGATGCAGCAACGCCGCGTGAGTGAAGAAGG

CTTTCGGCTCGTAAAGCTCTGTTGTTGGTGAGGAAGGTTAGGG

>4583 len=103 tot-seq=1

CGCAGCAACGCCGCGTGAAGGAAGAAGGTCTTCGGATCGTAAACTTTTGTCTTCAGGAAC

GAAAATGACGGTACCTGAGGAGGAAGCCACGGCTAACTACGTG

>4584 len=103 tot-seq=1

TAGGGAATCTTCCACAATGGACGCAAGTCTGATGGAGCAACGCCGCGTGAGTGAAGAAGG

GTTTCGGCTCGTAAAGCTCTATTGGTGGGGAAGAAAGTCAGGG

>4585 len=103 tot-seq=1

CATGAGCGGCAGACAGGCGGCCAAGAAGTGAGGCCCCAGACAGGGATCTGAGCGTGATCT

TTGCCCTTGGGACGCAAGGCATCCATCCAAGAGGTCACAGAAA

>4586 len=103 tot-seq=1

TAGGGAATCTTCCACAATGGACGCAAGCCTGATGGAGCAACGCCGCGTGAGTGAAGAAGG

GTTTCGGCTCGTAAAGTCCTGTTGTTGGTGAAGAAAGACACGG

>4587 len=103 tot-seq=1

TAGGGAATCTTCGGCAATGGGGGCAACCCTGATCGAGCAACGCCGCGTGAGTGGAGAAGG

TTTTCGGATCGTAAAGCTCTGTTGTTAGAGAAGAACGAGTGTG

>4588 len=103 tot-seq=1

TGGGGAATATTGCGCAATGGGCGAGACCCTGAAGCAGCGACGCCGCGTGCGGGATGAAGG

CCTTCGGGTTGTAAACCGCCTTTGATTGGGAGCAAGCCTTCGG

>4589 len=103 tot-seq=1

TGGGGAATCTTGCGCAATGGGGGAAACCCTGACGCAGCGACGCCGCGTGCGGGATGAAGG

CTTTCGGGTTGTAAACCTCTTTTGTTTGGGAGGAAGCCTTCGG

>4590 len=103 tot-seq=1

TAGGGAATCTTCCACAATGGACGCAAGTCTGATGGAGCAACGCCGCGTGAGTGATGAAGG

AATTCGTTTCGTAAAACTCTGTTCTTTGTGAAGAAGGGCACTG

>4591 len=103 tot-seq=1

TAGGGAATCTTCCACAATGGGCGCAAGCCTGATGGAGCAACGCCGCGTGAGTGAAGAAGG

GCTTCGGCTCGTAAAGCTCTGTCCTTGGGGAAGAAAGTGACGG

>4592 len=103 tot-seq=1

TAGGGAATCTTGCACAATGGACGCAACCCTGATGCAGCAACGCCGCGTGAGGGAAGAAGG

CTTTCGGGTTGTAAACCTCTGTTGTTGGTGAGGAAGGATACGG

>4593 len=103 tot-seq=1

CGGCATACGAGATGTAAAGTTCTTTCGGTAATGAGGAAGGGATGTTGTTAAATAGATAGC

ATCATTGACGTTAATTACAGAAGAAGCACCGGCTAACTCCGTG

>4594 len=103 tot-seq=1

GATGACGGTCCTATGGGTTGTAAACTGCTTTTATAGGGGAATAAAGTGAGGGACGGGTCC

CTTATTGCATGTACCATAAGAATAAGGACCGGCTAATTCCGTG

>4595 len=103 tot-seq=1

ACAGATGTGAAAAATGGGACTGTGGCTTGGTTTAGATGTAATGAGTGAAGGAAATCAAAT

CTAGAATGATGAGTTCTGACATGAAAAATGGTGGGTGATGGTG

>4596 len=103 tot-seq=1

TAGGGAATCTTCCACAATGGACGAAAGTCTGATGGAGCAACGCCGCGTGAGCGAAGAAGG

TTTTCGGATCGTAAGGCTCTGTTGTTGGTGAAGATGGTTACAG

>4597 len=103 tot-seq=1

GATGACGGCCCTATGAGTTGTAAACTGCTTTTATAGGGGGATAAAGTGGGGAACGTGTTC

CCTTTTGCGGGTACCATATGAATAAGGCCCGGCTAATTCCGTG

>4598 len=103 tot-seq=1

TAGGGAATCTTCCACAATGGACGCAAGTCTGATGGAGCAACGCCGCGTGAGTGAAGAAGG

GTTTCGGCTCGTAAAGCCCTGTTGGTGGTGAAGAAAAATAGGG

>4599 len=103 tot-seq=1

TAGGGAATCTTCGGCAATGGCCGGAAGCCTGACCGAGCAACGCCGCGTGAGTGAAGAAGG

ATTTCGGATCGTAAAGCTCTGTTGTCAGAGAAGAACGAGAGTG

>4600 len=103 tot-seq=1

TAGGGAATCTTCCACAATGGACGGAAGTCTGATCGAGCAACGCCGCGTGAGTGAAGAAGG

TTTTCGGATCGTAAAGCTCTGTTGTTAGTGAAGAAGGAGAGAG

>4601 len=103 tot-seq=1

TGGGGAATATTCCACAATGGGCGCAACCCTGATGCAGCGACGCCGCGTGAGCGAAGAAGG

GTTTCGACTCGTAAAGCTCTGTCCTTTGAGAAGATAATCACGG

>4602 len=103 tot-seq=1

TGGGGAATCTTCCACAATGGGCGCAACTCTGATGCAGCGACGCCGCGTGAGCGATGAAGG

TTTTCGACTCGTAAAGCTCTGTCCTATGGGAAGATAGTGACGG

>4603 len=103 tot-seq=1

TGGGGAATCTTCCACAATGGACGCAACCCTGATGCAGCGACGCCGCGTGCGTGATGAAGG

CCTTCGGCTCGTAAACCGCTTTTGTTTGGGAGCAAGCTCTCGG

>4604 len=103 tot-seq=1

TGGGGAATATTGCACAATGGGCGCAAGCCTGATGCAGCAACGCCGCGTGAACGATGAAGG

GCTTCGGATCGTAAAGTTCTGTTGCTGGGGAAGAAAGTCACGG

>4605 len=103 tot-seq=1

TAGGGAATCTTCCACAATGGACGCAAGTCTGATGGAGCAACGCCGCGTGAGTGAAGACGG

GCTTCGGCTCGTAAAGCTCTGTTGTTGGTGACGAAGGACCGGG

>4606 len=103 tot-seq=1

TAGGGAATCTTCCACAATGGACGCAACCCTGATGCAGCAACGCCGCGTGAGTGAAGAAGG

GTTTCGGCTCGTAAAGCTCTGTTGTTGGGGAAGATGGTCACGG

>4607 len=103 tot-seq=1

GATTTGGGGGCTGAGATGGAAGGTTGGGGCTTAGAGGGGTCTGAAAGGACTTTGACTGGA

GGGATTGGAAGGAGTGGGAGGAAAGAACATGGTGTGTGGGGGG

>4608 len=103 tot-seq=1

CAAAGGGAAAAGACAAGAGATCTATTTGGGGAGCCAAGCAGTTCCCTGCAAAGTTGCGCC

ACTGAAGCTCTGCATCCTAGCAACCTGCATTCTGCCCACTTTT

>4609 len=103 tot-seq=1

ACGGGAGGTTCCCGGTGGAGGGGCTTAAGGAGAGGGTCTGTACGTTTATCCCGGTTACCT

GTCTGAGAGGGGTGACGTGGTGCGCTATGGCATGGGTTCAGTA

>4610 len=103 tot-seq=1

TAGGGAATCTTCCGCAATGGGCGAAAGCCTGATGGAGCAACGCCGCGTGAGTGAAGAAGG

TCTTCGGATCGTAAAACTCTGTTATTAGGGAAGAAGGTTTGTG

>4611 len=103 tot-seq=1

CAGCACCTTCTAGGACAGGTGGCAATCCAGCAGCAACAGCAGCAGGGTCCTGGAGTACAG

ACAAACCAAGCTCTGGGTCCCAAGCCCCAGGGCCTTATGCCTC

>4612 len=103 tot-seq=1

GGCCTTCGGGTTGTAAAGCTCTGTTAATCGGGACGAATGGCTACCATGCGAATAGTTTGG

GAGTTTGACGGTACCGGAATAGAAAGCCACGGCTAACTACGTG

>4613 len=103 tot-seq=1

GGTGGATGGGGAAAAGCAGGGCTGTGCCCTAGGCTGACCTATGAACTCTGCTCCCACTCC

AAACAGCTGTGTCACCAGCAGGTCACCTACACTCAGCCTAGCT

>4614 len=103 tot-seq=1

TAGGGAATTTTCCACAATGGACGCAAGTCTGATGGAGCAATGCCGCGTGAGTGATGAAGG

TTTTCGGATCGTAAAGTTCTGTTGTTGGGGAAGAAGGATAGAG

>4615 len=103 tot-seq=1

TAGGGAATCTTCCACAATGGACGCAACTCTGATGGAGCGACGCCGCGTGAGTGAAGAAGG

TTTTCGGATCGTAAAGCTCTGTTGGTGGGGAAGAAGGAGAGAG

>4616 len=103 tot-seq=1

CAATTCCGAGTCTTTACGTCATTCTTTGAACATATAAACCAGAAGTTACCAGAGCCTCCT

CCTTTAACCAAAAACCTATTTGCTCAGCTGGCTGAAAACATTG

>4617 len=103 tot-seq=1

TGGGGAATATTGGACAATGGGCGCAAGCCTGATCCAGCCATGCCGCGTGTGTGAAGAAGG

CCTTCGGATTGTAAACTACTTTTGTCTGGGAGGAAGGGCAGTA

>4618 len=103 tot-seq=1

GAAAGGGAAGGTTCCCTAGCCCAAGAGCTGAAGATTACCACAGAAGGACAAATCAGTCGG
GAAAAACATATCCTGCTCCCATCCATCAGTGATCCTGGAGAGT
>4619 len=103 tot-seq=1
GAAGTGTGTGCTGAGCCAGGGCAGGGAGGAGGAATGTGAGCTATGAACGCCGCCCGAGCT
CCTCGGCTCTCTGGGCCCCCCTTCCCCACGAGCCCATGTGCTG
>4620 len=103 tot-seq=1
TAGGGAATCTTCCACAATGGACGCAAGTCTGATGGAGCAACGCCGCGTGAGCGAAGAAGG
TCTTCGGATCGTAAAGCTCTGTTGTTGGTGAAGATGAATACAG
>4621 len=103 tot-seq=1
TAGGGAATCTTTCGTAAAGCTCTGTTGTTGGTGAAGAAGGATAGAGGTAGTAACTGGCCT
TTATTTGACGGTAATCAACCAGAAAGTCACGGCTAACTACGTG
>4622 len=103 tot-seq=1
TGTCTTTGTGTTCCCCTCTCATGTTTGTTAGGGCATTGTTGATTGTTTATATCCTATGTA
TGGTGCAGTGGCGGAAGAGTGGCCTCTCAAAAGATATGTCCGC
>4623 len=103 tot-seq=1
CGGACTGAGGTGGTGAGGACTGGGGACGGGGGGTCTCAGGTGAGAGCGGAGAAGTCTCTC
CATCCTGGGATGCAGGGGTGACACACTTGAGTGTAGTGGGGTC
>4624 len=103 tot-seq=1
GGGGGCGGGGCCCAGACATCAGTATTTTTTCAGAACTCCCTATATGATTCATGGGCAGCG
GAGCTTACAAACCCACTCTGGAGCACAGCGTTATCTGGTCTTT
>4625 len=103 tot-seq=1
TGGGGAATATTGCACAATAGGCGAAAGCCTGATGGAGCGACGCCGCGTGAGCGAAGAAGG
ATTTCGGAATGTAAAGCTCTATCAGAAGGGAAGAAAATGACGG
>4626 len=103 tot-seq=1
CTGCTGTTATCCCGCCGTCACTCAGGGGCAGTGTGTCATCTGCTCACTATTCCCTGAGCT
CATTTGCCTGGCCCTCTGTGATGTGGAGAGGGAGGGGCCGCCC
>4627 len=103 tot-seq=1
CTTGGGGCACACATCCTCAGGCTTATAGGTGCCGGTGGGAGAGACAGAAGCATCCGCAGC
AAGGATTTTAATTCATCTCTGGTGCCTATATCACTAAGCAATG

>4628 len=103 tot-seq=1

TAGTGACGAAAAATAACAATACAGTACGCTTTCGAGGCCCTGTAATTGTAATGAGTCCAC

TTTAAAGCCTTTAACGAGGAACCATTGGAGGGCAAGTCTGGTG

>4629 len=103 tot-seq=1

CGTATTGAATTTACACTTGAAGGTGCAAGCTGACAGCACCTGGTGTTCTCTATCGATGGA

AGTGGGCCGGCCGAGAGGGTCCACGCGCACGTTTGACTGGTCA

>4630 len=103 tot-seq=1

CAGGGAATCTTGGGCAATGGGGGCAACCCTGATCGAGCAACGCCGCGTGAGTGAAGAAGG

TTTTCGGATTGTAAAGCTCTGTTGTAAGAGAAGAAAGTGTGGG

>4631 len=103 tot-seq=1

TAGGGAATCTTCGGCAATGGGCGCAAGCCTGACCGAGCAACGCCGCGTGAGTGAAGAAGG

ATTTCGGAACGTAAAGCTCTGTTGTAAGAGAAGAACATGTGTG

>4632 len=104 tot-seq=1

GGAAGAGGACCTATTTGAGAGCTGCGGCCACCCGGAAGAGGAAGAGGAAGAGGAGGAAGA

AGATCAGGAAGAGGAGCAGGAGGAGGAGGGAGATTTAGCTTTGG

>4633 len=104 tot-seq=1

CTGTGAATGGAAGTCCAAGATCTAGCAGTTGCTCAGACTCACCAAACAAGCAGGCATAGA

AGAGAGAGTCCATCTTCCTTCTTTCAATGTCCTTATGTAGGTCT

>4634 len=104 tot-seq=1

TAGGGAATAGTCCACAATGGAGGAAAGTCTGATGGAGAAACGCCGCGTGAGCGAAGAAGG

GTTTCGGCTCGTAAAGCTCTGTTGGTAGTGAAGAAAGGTAGAGG

>4635 len=104 tot-seq=1

TAGGGAATCTTGGACAATGGGGGCAACCCTGATCCAGCCATGCCGCGTGTCTGAAGAAGG

CCCTCGGGCTGTAAAGGACTTTTGTCCGGGAAGAAATGCGCGAT

>4636 len=104 tot-seq=1

TAGGGAATCTTGCACAATGGACGCAAGTCTGATGGAGCAACGCCGCGTGAGTGGAGAAGG

GTTTCGGCTCGTAAAGCGCTGTTGTTGGGGAAGAAAGACACGGG

>4637 len=104 tot-seq=1

TGGGGAATCTTCCGCAATGGACGCAAGCCTGACGGAGCAATGCCGCGTGAGTGATGACGG

CCTTCGGATTGTAAAGCTCTGTCAATCGGGACGAATGGTCGGTG

>4638 len=104 tot-seq=1

TGGGGAATTTTGGACAATGGGGGAAACACTGATCCAGCCATCCCGCGTGTGCGATGAAGG

ACTTCGGGTTGTAAAGCACTTTTGGCAGGAAAGAAAAGTAGTGG

>4639 len=104 tot-seq=1

TGGGGAATCTTGCACAATGGGCGAAACCCTGACGCAGCGACGCCGCGTGCGGGAGGAAGG

CCTTCGGGTTGTAAACCGCTTTTGATTAGGAGCAAGCCCTCGGG

>4640 len=104 tot-seq=1

TAGGGAATCTTCCACAATGGGCGCAAGTCTGATGGAGCGACGCCGCGTGCGGGAAGAAGG

GCTTCGGGTCGTAAACCGCTTTTGTTTGGGAGGAAGGTCTGGGG

>4641 len=104 tot-seq=1

TGGGGAATCTTGCGCAATGGGCGAAACTCTGACGCAGCGACGCCGCGTGAGGGATGAAGG

CCTTCGGGTTGTAAAGCTCTTTTGATTGGGAGCAAGGCTTTTGG

>4642 len=104 tot-seq=1

TGGGGAATATTGCGCAATGGGGGAAACTCTGATGTAGCGACGCCGCGTGCGGGATGAAGG

CCTTCGGGTTGTAAACCGCTTTTGTTTGGGAGCAAGCCCTGTGG

>4643 len=104 tot-seq=1

GGAGGCTACTTTCCGGGAGAGGGCAGCAGCACAGGAGAAGGCCCTGCCGCCTACAGGGGT

CCTACTCCACTCCGGGACCCTGCCCATCTGTGGCCATCCTCCCA

>4644 len=104 tot-seq=1

TAGGGAATCTTCGGCAATGGACGGAAGCCTGACCGAGCAACGCCGCGTGAGTGAAGAAGG

TTTTCGGATTGTAAAGTTCTGTTGTAAGTGAAGAACGAGTGTGT

>4645 len=104 tot-seq=1

TGGGGAATATTGCGCAATGGGCGAAACCCTGACGCAGCGACGCCGCGTGCGGGATGAAGG

CCTTCGGGTTGTAAACCGCTTTTGATGGGGAGGAAGGTTTCGGG

>4646 len=104 tot-seq=1

TGGGGAATATTGGACAATGGACGCAACTCTGATCCAGCAATTCCGCGTGTGTGAAGAAGG

TTTTAGGACTGTAAAGCACTTTTAGTGGGGAAGAAAGAATGAGG

>4647 len=104 tot-seq=1

TAGGGAATCTTCCACAATGGACGCAAGCCTGACGGAGCAACGCCGCGTGAGTGAAGAAGG

GCTTCGGCTCGTAAAACTCTGTTGTTGGGGAGGAAGGCCCGGGG

>4648 len=104 tot-seq=1

TGGGGAATATTGCACAATGGGCGAAACCCTGACGCAGCGACGCCGCGTGCGGGATGACGG

CCTTCGGGTTGTAAACCGCTTTTGATGGGGAGCAAGCCTTTTGG

>4649 len=104 tot-seq=1

TAGGGAATCTTCCACAATGGACGAAAGTCTGATGGAGCAACGCCGCGTGAGTGAAGAAGG

TTTTCGGATTGTAAAGCTCTGTTGTTGGGGAAGAAGAATAGGGG

>4650 len=104 tot-seq=1

TAGGGAATCTTCCACAATGGGCGCAACCCTGATGGAGCAACGCCGCGTGAGTGAAGAAGG

GCTTCGGCTCGTAAAGCTCTGTTGTTGGTGAAGAAGGTCTGGGG

>4651 len=104 tot-seq=1

CGGCATACGAGATAAGGCCGTGACTGGAGTTCAGACGTTGCTCTTCCGATCTCCTATGGG

AGGCAGCAGATCTTCATGCCTACAGTCGGCCGCCTCAGCTATGA

>4652 len=104 tot-seq=1

TGGGGAATATTGCACAATGGGCGAAACCCTGATGCAGCGACGCCGCGTGCGGGATGAAGG

CCTTCGGGTTGTAAACCGCTTTCAGCAGGGACGACGCCGCCAGG

>4653 len=104 tot-seq=1

TAGGGAATCTTCCACAATGGGCGCAAGCCTGATGGAGCAACACCGCGTGAGTGAAGAAGG

GTTTCGGCTCGTAAAGTTCTGTTGTTGGGGAAGAACATGAGGGT

>4654 len=104 tot-seq=1

TGGGGAATATTGCACAATGGGGGCAACCCTGACGCAGCGACGCCGCGTGCGGGAAGAAGG

CCTTCGGGTTGTAAACCGCTTTTGATTGGGAGGAAGCCTTTTGG

>4655 len=104 tot-seq=1

TGAGGAATATTCCACAATGGGCGCAAGCCTGATGCAGCAACGCCGCGTGAGTGATGAAGG

TTTTCGGATCGTAAAGCTCTGTTGGTTGGGAAGAAGGATAGAGG

>4656 len=104 tot-seq=1

TAGGGAATCTTCCACAATGGACGAAACCCTGATGGAGCGACGCCGCGTGAGTGATGAAGG

CTTTCGGATCGTAAAGCTCTGTTGTTGGTGAAGAAGCATAGGGG

>4657 len=104 tot-seq=1

TAGGGAATCTTGCACAATGGGCGCAAGCCTGATGGAGCAACGCCGCGTGAGTGAAGAAGG

TTTTCGGATCGTAAAGCTCTGTTGTTGGTGAAGAAGGCTGGAGG

>4658 len=104 tot-seq=1

TGGGGAATATTCCGCAATGGGGGAAACCCTGACGCAGCGACGCCGCGTGCGGGATGAAGG

CCTTCGGGTCGTAAACCGCTTTTGTTTGTGAGCAAGGCTTTTGG

>4659 len=104 tot-seq=1

TAGGGAATCTTCCACAATGGACGCAAGCCTGACGGAGCAACGCCGCGTAAGTGAAGAAGG

CTTTCGGATCGTAAAGCTCTGTTGTTGGTGAAGAAGGATAGTGG

>4660 len=104 tot-seq=1

TAGGGAATCTTCCACAATGGACGCAAGCCTGATGGAGCAACGCCGCGTGAGGGAAGAAGG

GTTTCGGCTCGTAAAGCTCTTTTGTTGGGGAGCAAGGAAAGGGG

>4661 len=104 tot-seq=1

TGGGGAATCTTCGACAATGGAGGCAACTCTGATGCAGCAACGCCGCGTGAGTGAAGAAGG

GTTTCGGCCTGTAAAACACTTTTAGTGGGGAAGAAAGAATGGGG

>4662 len=104 tot-seq=1

TGGGGAATATTCCACAATGGACGCAAGCCTGATGCAGCAACGCCGCGTGAGGGAAGAAGG

TCTTCGGATCGTAAACCTCTGTTGTTGGTGAAGAAGGTTACGGG

>4663 len=104 tot-seq=1

TAGGGAATCTTCCACAATGGACGCAAGCCTGATGGAGCAACGCCGCGTGAGTGAAGAAGG

GCTTCGGATCGTAAACCCCTGTTGTTGGGGAAGAAAGTGAGGGG

>4664 len=104 tot-seq=1

TGGGGAATCTTGCGCAATGGGGGAAACTCTGACGCAGCGACGCCGCGTGCGGGATGAAGG

CCTTCGGGTTGTAAACCGCTTTTGGTTGGGAGCAAGCCCTCGGG

>4665 len=104 tot-seq=1

TAGGGAATATTGCACAATGGACGAAACCCTGATGGAGCAACGCCGCGTGAGGGAAGAAGG

ATTTCGGATTGTAAAGCTCTGTTGGTGGTGAAGAAGGATTGAGG

>4666 len=104 tot-seq=1

AGGAAGACGGATCTATGGTTTGTAAACTTCTTTAGTGCGGGAACAAAGGGGTGTCGTGAC

ACCGGATGAGTGTACCGCAAGAATAAGCATCGGCTAACTCCGTG

>4667 len=104 tot-seq=1

CACTAGGTGCCCAAAGATTTCCCTCTCCATCATAACTGCTCCCTCCCTGAGGCACTTTTT

CTGCAGAGTGCCTCCAGCAAGACACCTCACTATGGCTTGTTCCT

>4668 len=104 tot-seq=1

TAGGGAATCTTCCACAATGGACGCAAGTCTGATGGAGCAACGCCGCGTGAGCGAAGAAGG

TTTTCGGATCGTAAAGCTCTGTTGGTAGTGAAGAAAGTTAGAGG

>4669 len=104 tot-seq=1

TAGGGAATCTTCCACAATGGGCGCAAGTCTGATGCAGCAACGCCGCGTGAGTGAAGAAGG

TTTTCGGATCGTAAAGCTCTGTTGTTGGTGAGGAAGGATAGTGG

>4670 len=104 tot-seq=1

TAGGGAATCTTCCACAATGGACGCAAGTCTGATGGAGCAACGCCGCGTGAGTGGAGAAGG

GCTTCGGCTCGTAAAGCTCTGTTGGTAGGGAGGAAAGATAGAGG

>4671 len=104 tot-seq=1

TAGGGAATATTCCACAATGGGGGAAAGCCTGACGGAGCAACGCCGCGTGAGGGAAGAAGG

TTTTCGGATTGTAAAGCTCTGTTGTTGGGGAGGAAGGATAGGGG

>4672 len=104 tot-seq=1

TAGGGAATCTTCCACAATGGACGCAAGTCTGATGGAGCAACGCCGCGTGAGGGAAGAAGG

TTTTCGGATCGTAAAGTTCTGTTGTTGGTGAAGATGGTTAGAGG

>4673 len=104 tot-seq=1

ACCGCCGCCGCCGGCCTAGTTACCATCACACCCCGGGAGGAGCCGCAGCTGCCGCAGCCG

GCCCCAGTCACCATCACCGCAACTATGAGCAGCGAGGCCGAGAC

>4674 len=104 tot-seq=1

TGGGGAATATTCCACAATGGGCGAAAGCCTGATGGAGCGACGCCGCGTGAGCGAAGAAGG

ATTTCGGTATGTAAAGCTCTATCAGAAGGGAAGAAAGTGATGGT

>4675 len=104 tot-seq=1

GTGAAGAAGGTCTTCGGATTGTAAACTGCTTTTGTATGGGGATAAAGTTAGGGACGTGTC

CCTATTTGCAGGTACCATACGAATAAGGACCGGCTAATTCCGTG

>4676 len=104 tot-seq=1

TGGAGGGACTTGGAGAAGGCAGAGCTATCATTAGTGCCTGCCCCAAGCCGGACCCAATCT

GCTCACCAAAGCAGGAGGCCACGCTTGCCCATGTGTCCTAGTAC

>4677 len=104 tot-seq=1

TCCAAAAGAAAATAAAGGGAAAGATTCCTATGGCATGCCTTGCCAAAGTGAGCATGCTCT

GGGAGTCAGGAATGCTCCGGCATTTAGGAGCGAGCAGGGGAGAG

>4678 len=104 tot-seq=1

GGAATGAGTGGGAGCCTCTGAGGTCACTGGGGCCATGGGGAACGGGGTATCTCTCTGTCA

CCGCAGGATTCCTTCCCAGCCCGGTTCAAAGCCATCCACTTCAT

>4679 len=104 tot-seq=1

TAGGGAATCTTCCGCAATGGGCGAAAGCCTGACGGAGCAACGCCGCGTGAGTGATGAAGG

TCTTCGGATTGTAAAGCTCTGTTAGTAGGGAAGAACATAAGTGT

>4680 len=104 tot-seq=1

GGATGAAGGTCTTATGGATTGTAAACTGCTTTTGTAAGGGAAGAATAAGGGCTACGCGTA

GTTTGATGACGGTACCTTACGAATAAGCATCGGCTAACTCCGTG

>4681 len=104 tot-seq=1

CCAACCTGGGCCCATGATTGACAATGGCCTGGAGTTCATCCAGAGAGTGGGGACCTGGCC

TGATATTGCTGGGGCCCCTCCTGACTCCTGCCCCACTGGGCCTC

>4682 len=104 tot-seq=1

GCGGAAGTGGCGGCGCATGCGCACTGGCACCCTCTCGGTGTCCTTTGAGCTGCGGCCGCA

GAGTGCGCGGGAGACCTACCCATTCCGCGGCCGGACCACCACTC

>4683 len=104 tot-seq=1

TGGGGAATATTGCGCAATGGGGGAAACCCTGACGCAGCGCTTTTGATTGGGAGCAAGCCT

TTTGGGTGAGTGTACCTTTCGAATAAGCGCCGGCTAACTACGTG

>4684 len=104 tot-seq=1

TAGGGAATCTTCCACAATGGACGCAAGTCTGATGGAGCAACGCCGCGTGAGTGAAGAAGG

GTTTCGGCTCGTAAAGCTATTAGAAAGTCACGGCTAACTACGTG

>4685 len=104 tot-seq=1

CATCCTGTCCAGCGCCAGCTTCATCAGCTTGGGCAGCAGTGCCCTCACCTACCAGGTGAG

CAGCGGGCTGCTGACTTCCACAGCCAGCCTTCCCTCCACCCAGG

>4686 len=104 tot-seq=1

CAATAAAAAAGCAGGCAAATGCTCTGAGCAGACAACATGAAGAAACACAAATGATCAACA

CATACACGGAAGCAAGCTCTCCCTCATTAGAAACCCCGGTAGTC

>4687 len=104 tot-seq=1

TTTTTCTTAAAAAAATTATTTTTCAGAGAGGGCGGTTCTTGCTATGTTGCCAAGGCTGTT

CCTGAACTCCTGGTCTCAAGCTATTACAGGCATGTGACCCATGA

>4688 len=104 tot-seq=1

CCAAGATTCCCCAGCGCCCAGGGCTTTCACCTGTCCCACCCTCTGCAGCTAAGGAAACCC

CCTTTCTGGACCCTTGGCGTCCCAGGTCCCTGTGCCTTGCTCAG

>4689 len=104 tot-seq=1

AGGTTTTCGGATCGTAAAGCTCTGTTGTATGAGAAGAACGTGTGTGAGATTGGAAAGTTC

TCACTGTGACGGTAACTTACCAGAAAGGGACGGCTAACTACGTG

>4690 len=104 tot-seq=1

AATCACCTAAACTCATTAGAGAATGGTCAAGCCCACAGCAATGATGAGGAAGGCACGCTT

GCGTTTTCTGGGCTGTAGTTTCTGGATGCAGTCATGTCCAAGGC

>4691 len=104 tot-seq=1

AAGGTTTTCGGATCGTAAAGCTCTGTTGTTGGTGAAGAAGAATAGAGGGAGTAACTGGTC

TTTATTTGACGGTAATCAACCAGAAAGGCACGGCTAACTACGTG

>4692 len=104 tot-seq=1

TGGGGAATCTTCCACAATGGACGCAAGCCTGATGGAGCAACGCCGCGTGAGTGAAGAAGG

CTTTCGGATCGTAAAGCTCTGTTGATGGTGAAGAAGGATATAGG

>4693 len=104 tot-seq=1

GCTCTGTGCCTGCTGAGAAGAAGTCAGCAGGAGAAGTCAGAGTCCCCCAGAAGTTGAGCC

CAAGGCAGGGATTTGAGTACAAGTAGATGATTAGGGAGGTGATC

>4694 len=105 tot-seq=1

GCCACTTCCTGCTGGGAGATGTCTCTCGCTGGAGGAGGGATGGGATGAATGGCTGCATCA

AGCCAGGGAGATACATGCTCTGGTGTCTGGACAGAATCAACACCT

>4695 len=105 tot-seq=1

TGGGGAATTTTCGACAATGGGCGAAAGCCTGATCCAGCAACGCCGCGTGCGGGAAGAAGG

CCTTCGGGTCGTAAACTTCTTTTGTACGGGACGAAAAAGCTTCTC

>4696 len=105 tot-seq=1

AAGCATGATCCTGGAGGCAGGAGCTGATGCAGAGGTCATGGAGGGGTTGATGCTTACTGG

CTTGTTCCATGGCTTGCTCATCCTGCCTTCTTATAGAACAGAGGA

>4697 len=105 tot-seq=1

GGCAACAGGACCACCCTGGGGTGGGACTAGCTATAGGCAATAAAGACCAGGCTTTCTCAA

ACAGATCAGCCCTGAGTAAGTTTAAGGTACAGCATCCAGAAGCAG

>4698 len=105 tot-seq=1

GTGAGTAGGGGAGTGCATGGGAAGTTACGATGAGTCAGGCAGGTCCTTTGGAGCCTCGTG

TTCTGGCTAAAGGGCTTTGCAAGGGGAGCAATGTGTGGTATTGGG

>4699 len=105 tot-seq=1

AATAGGCTAACCCTGGCCAGAGCCTTGAGTTTGCCATCTCTGAGGCATGGAAGAGGATGG

GTCACATGTGAGCCCTAGTGTGTCATGCTCAGGGACAGAGACAGG

>4700 len=105 tot-seq=1

GAAGGTTTTCGGATCGTAAAGCTCTGTTGGTGGTGAAGAAGTGTAGAGGTAGTAACTGGC

CTGTATTTGACGGTAATCAACCAGAAAGTCACGGCTAACTACGTG

>4701 len=105 tot-seq=1

CCAGCCAAGTAGCGTGAGGGATGACTGCCCTATGGGTTGTAAACCTCTTTTATATGGGAA

TAACAGAGATTACGTGTTCCCTATTGCATGTACCATAATGAATAA

>4702 len=105 tot-seq=1

TAGGGAATCTTCCACAATGGACGCAAGTCTGATGGAGCAACGCCGCGTGAGGGAAGACGG

TTTTCGGATCGTAAAGCTCTGTTGTTGGTGAAGAAGGATAGTGTT

>4703 len=105 tot-seq=1

GAAGGGTTTCGGCTCGTAAAGCTCTGTTGTTGGTGAAGAAGGGCAGGGGTAGTAACAGAC

CTTTATTTGACGGTAATCAATTAGGAAGTCACGGCTAACTACGTG

>4704 len=105 tot-seq=1

GACGCCGCGTGCGGGATGAAGGCCTTAGGGTTGTAAAACGCGTTTGATTGGGCGCAAGAC

TTTTGGGTGAGTGTACCTTTCGAATAAGCGCCGGCTAACTACGTG

>4705 len=105 tot-seq=1

CCCTTGGTGTCACAGACCCTGGGGCCTAGGAGAGAAGAATGGTTTCTGGGGCCAGCCCCA
TGGCCCTGCTGCTGTGTGCAGCCTCAGGACACTGCTGCCTGCATC
>4706 len=105 tot-seq=1
AGGATGACTGCCCTATGGGGTGTAAACTGCTTTTGTAGGGGGATAAAGTTAGGGACGTGT
CCCTATTTGCAGGTACCATACGAATAAGGCCCGGCTAATTCCGTG
>4707 len=105 tot-seq=1
GAAGGGTTTCGGCTCGTAAATCTCTGTTGTTTGTGAAGAAGGACAGGGTTATTAACTGAC
CTTTGTTTGACGGTAATCAATTAGAAAGTCACGGCTAACTACGTG
>4708 len=105 tot-seq=1
TTTTTTTTTTCAAGCAGAAGACGGCATACGAGATCTGATCGTGACTGGAGTTCAGACGTG
GCTCTTCCGATCTCCTACGGGAGGCAGCGGCCGCCCAGCAGCCGC
>4709 len=105 tot-seq=1
TAGGGAATCTTCCGCAATGGGCGCAAGCCTGATGGAGCAACGCCGCGTGAGGGAAGAAGG
GTTTCGGCTCGTAAAGCTCTGTTGTTGGGGAAGAAGGAGAGGGGT
>4710 len=105 tot-seq=1
AGAGGGAAAAGGTGTTGATCCAGCAGCCGCGGTAACTTAGATCGGAAGAGCGTCGTGTAG
GGAAGAGTGTGCCAATGTGTAGATCTCGGTGGTCGCCGTATCATT
>4711 len=105 tot-seq=1
TGGGGAATATTGCGCAATGGGCGAAACCCTGACGCAGCAACGCCGCGTGCGGGAAGAAGG
CCTTCGGGTTGTAAACCGCTTTTGTTGGGGAGCAAGCTTTCGGGT
>4712 len=105 tot-seq=1
GAAGGCCTTCGGGTTGTAAAGCACTTTCAGCGAGGAGGAAGGGTTAGTCGTTAATAGCGG
CTAATTTTGACGTTACTCGCAGAAGAAGCACCGGCTAACTCTGTG
>4713 len=105 tot-seq=1
TGGGGAATATTCCGCAATGGGCGAAACTCTGACGCAGCGACGCCGCGTGAGGGATGAAGG
CTTTCGGGTCGTAAACCGCTTTTGTTTGGGAGCAAGCTTTCGGGT
>4714 len=105 tot-seq=1
TGGGGAATCTTGCACAATGGGCGCAACCCTGATGGAGCAACGCCGCGTGAGTGAAGAAGG
GTTTCGGCTCGTAAAGCTCTGTTGTTGGGGAGGAAGGTCACGGGT

>4715 len=105 tot-seq=1

TAGGGAATCTTCCACAATGGACGCAAGCCTGACGGAGCAACGCCGCGTGAGTGATGAAGG

GTTTCGGCTCGTAAAGCTCTGTTGTTGGGGAAGAAGGATAGGGGT

>4716 len=105 tot-seq=1

TAGGGAATATTGCACAATGGAGGCAACTCTGATGCAGCCACGCCGCGTGTGTGAAGAAGG

CCTTCGGGTTGTAAAGCTCTTTTGTTGGGGAGGAAGGATTGAGGT

>4717 len=105 tot-seq=1

TGGGGAATCTTCCGCAATGGGCGCAACCCTGACGCAGCAACGCCGCGTGCGGGATGAAGG

CCTTCGGGTTGTAAACCGCTTTTGTTTGGGAGCAAGCCTTTTGGG

>4718 len=105 tot-seq=1

TGGGGAATATTGCGCAATGGGGGAAACCCTGATGCAGCGACGCCGCGTGTGGGATGAAGG

CCTTCGGGCTGTAAACCGCTTTTGATTGGGAGCAAGCCTTCTGGG

>4719 len=105 tot-seq=1

GAAGGGTTTCGGCTCGTAAAACTCTGTTGTTGGTGAAGAAGGGCAGGGGTAGTAACTGAC

CTTTGTTTGACGGTAATCAAGTAGAAAGTCGCGGCTAACTACGTG

>4720 len=105 tot-seq=1

TGGGGAATATTGCGCAATGGGGGAAACCCTGATGCAGCGACGCCGCGTGCGGGAAGAAGG

CCTTCGGGTTGTAAACCGCTTTTGTTTGGGAGGAAGCATTCGGGT

>4721 len=105 tot-seq=1

TGGGGAATCTTGCGCAATGGGGGCAACCCTGACGCAGCGACGCCGCGTGCGTGATGAAGG

CCTTCGGCTTGTAAACCTCTTTTGATTGGGAGCAAGCTTTCGGGT

>4722 len=105 tot-seq=1

TGGGGAATATTGCGCAATGGGGGCAACCCTGACGCAGCGACGCCGCGTGCGGGATGAAGG

CCTTCGGCTTGTAAACCGCTTTTGTTTGGGAGCAAGCACTCGGGT

>4723 len=105 tot-seq=1

GAAGGGTTTCGGCTCGTAAATCTCTGTTGTTGGTGAAGAAGGACAGAGGTAGTAACTGAC

CTTTATTTGACGGTAATCAATTAGAAAATCACGGCTAACTACGTG

>4724 len=105 tot-seq=1

TAGGGAATCTTCCACAATGGACGCAAGTCTGATGGAGCAACGCCGCGTGAGGGAAGAAGG

GCTTCGGCTCGTAAACCTCTGTTGTTGGTGAAGAAGGACAGGGGG

>4725 len=105 tot-seq=1

TGGGGAATATTGCGCAATGGGCGGAACCCTGACGCAGCGACGCCGCGTGCGGGATGAAGG

CTTTCGGGTTGTAAACCGCTTTTGATTGGGAGCAAGGCTATTGGG

>4726 len=105 tot-seq=1

TAGGGAATCTTGCACAATGGACGCAAGTCTGATGCAGCAACGCCGCGTGAGTGATGAAGG

GTTTCGGCTCGTAAACCTCTGTTGATGGGGAAGAAGGAAAGGGGT

>4727 len=105 tot-seq=1

TGGGGAATCTTGCACAATGGACGCAAGCCTGATGGAGCAACGCCGCGTGCGGGATGAAGG

CTTTCGGATCGTAAACCTCTGTTGTTGGTGAAGAAGCATAGGGGT

>4728 len=105 tot-seq=1

TAGGGAATCTTCCGCAATGGGCGCAACCCTGACGCAGCGACGCCGCGTGCGGGAAGAAGG

CCTTCGGCTCGTAAACCGCTTTTGATTGGGAGCAAGCTCTCGGGT

>4729 len=105 tot-seq=1

GAAGGGTTTCGGCTCGTAAAGCTCTGTTGTTGGAGAAGAACGTAACGGAGAGTAACTGAT

CAGGGAGTGACGGTAACCAACTAGAAAGTCACGGCTAACTACGTG

>4730 len=105 tot-seq=1

AGGGTCCTGGGGATAGTGGCTGTCCCCCCAAATCCAGCAAGGCACTCATAATTATTATTT

GTCCTATCTAATCAAGTAATACACAATAAGTTTTTTATTTTTTCC

>4731 len=105 tot-seq=1

GAAGGGTTTCGGCTCGTAAATCTCTGCTGTTGGAGAAGAACGTATCTGAGAGTAACTGGT

CAGGTAGTGACGGTAGCCAACCAGAAAGTCACGGCTAACTACGTG

>4732 len=105 tot-seq=1

TAGGGAATCTTCCACAATGGGGGCAACCCTGATGGAGCAACGCCGCGTGAGTGAAGAAGG

TTTTCGGATCGTAAAGCTCTGTTGTTGGTGAAGAAGGATAGTGGT

>4733 len=105 tot-seq=1

TGGGGAATATTGGACAATGGAGGTAACTCTGATCCAGCAACTCTGCGTGAGTGAAGAAGG

TTTTAGGACTGTAAAACACTTTTAGTAGGGAAGAAAAAAATGACG

>4734 len=105 tot-seq=1

ATTCAGAACCTCGTAGCAGTGTTCTGGAAACCTATGTGAGGGACAAACACTCTTAACATA

GCAGCGGTGTTCACGAATCTCATGTGAGGGACAAACACTGAGAAC

>4735 len=105 tot-seq=1

CCAGCAACGCCGCGTGAAGGAAGAAGGATTTCGGGTTGTAAACTTCTTTTAAGAGGGACG

AAGGAAGTGACGGTACCTCTTGAATAAGCCCCGGCTAACTACGTG

>4736 len=105 tot-seq=1

AAGGTTTTCGGATCGTAAAGATCAGTTGTAAGAGAAGAACGAGTGTGAGAGTGGAAAGAT

CACATTGTGACGGTAGCTTACCAGAAAGGGACGGCTAACTACGTG

>4737 len=105 tot-seq=1

TGTTGCTGAGGTCCTTCCTCCACTGTATCGCGTCTGAGCATGTAACCTGGTGTTGCCAGG

GGCGTAGAAGGGGAGTTCGCTGTGTGTTGGCACAGCATGAGTAAC

>4738 len=105 tot-seq=1

GCACAGTGTCAATGGGCAGGAATAATTCCTGCCAATTAAGCCCTTGCTTCAAAGAAGGAG

GTCATGCTCCATGGCATATATAAGGCCCAGGAAACTTAAAGGCTG

>4739 len=105 tot-seq=1

CCAGCAATTCGGTGTGTGTGAAGAAGGTTTTAGGACTGTAAAACACTTTTAGTAAGTAAG

AAAGAAATGACGGTACCTACAGAGGAAGCGACGGCTAAATACGTG

>4740 len=105 tot-seq=1

GAAGGCTTGTTCTTTTGGCAGTTCAGTGACGCCATTAATAACGCGGTACTTGGGCTCTTT

GCTCACTTCTGGCGCATCTTTATGATAAAGATCTGCGGTGACGCG

>4741 len=105 tot-seq=1

GGCATACGAGATAACACTGTGAATGGAGTTCAGACGTGTGCTCTTCCGATCTCCTACGGG

AGGCAGAAGGAGACAAAGAGAGGAGGGGAGAAGGGCATGAAATCT

>4742 len=105 tot-seq=1

GAGCATTGCGATCGCCGCGCGGCGGTGGCGATATTTAAAGGGGACGCAGCCTATCTGTCA

GGAGTGGAGCGTGAGTCGGCTCAGCCAATGCGCATGCGTGAGGCA

>4743 len=105 tot-seq=1

CAGCTGAGGCAGTTTCTAGTAGGTAAATTGTTCCAAGGTGAATCATTGGAATTACAGGTC

ATGATGCTATCTCACGGCCATAAATCATCTCATCACATTATCTCT

>4744 len=105 tot-seq=1

TGCTAACAGCATTTCTACTATGGAATCAGTAACATGGTGGACCCTTTCTGCTGTGCAGAA

CATGCCTCTGACAGGAATTGAGCATTTCCAAGCCTAGGTCATAGG

>4745 len=105 tot-seq=1

GACTGGCCCCTACCGTTTCAAAAGGGACAGGTCACAGGCTGAGCAGGATGGGGCGGCAGG

GGCAGGCTGCCTCCCCACACGCAGAGGTCCCACCCCAGCTCCCTA

>4746 len=105 tot-seq=1

CCCATGCCTGGTGTGGTCTCATTGCAGTGTCTCTCTCTCTCCTTGGCCACACCTGCAGTA

CATCTTCGAAGTCAAGAAGCCAGAAGATGAAGTCCTGATCTGCAT

>4747 len=105 tot-seq=1

GATGCAGCGACGCCGCGTGAGCGAAGAAGTATTTCGGTATGTAAAGCTCTATCAACAGGG

AAGATAATGACGGTACCTGACTAGGAAGCCCCGGCTAACTACGTG

>4748 len=105 tot-seq=1

CCAGCAATTCTGTGTGTGTAAAAAAAGTTTTAGAACAATAAAACACTTTTAGTAGGGGAG

AAAAAAATGACGGTACCTACAGAAGAAGCAACGGCTAAATACGTG

>4749 len=105 tot-seq=1

GGAAGAGGGATGAACAACAATTAATTTGGATGGATTTGGAGCTGAAAGGAGTTACTGTCT

GTCTATAATTCTTTCCCTTCATTCCAGATTTCAGGACTTTCAAGG

>4750 len=105 tot-seq=1

GAAGGGTTTCGGCTCGTAAAGCTCTGTTGTTGGTGAAGAAGGATAGGAGTAGTAACTGAC

CTTTGTTTGACTGTAATCATTTAGAAAGTCACGGCTAACTACGTG

>4751 len=105 tot-seq=1

GTGAGAGGAGTGGCAGCAGGTGAGACATTTGAAAGAGAGACTGAGAGTTAGAATATGTGC

GAGAGGGAAGGAAAGGGAGAGACAGAGAGAAGGGGAATCCTGTGA

>4752 len=105 tot-seq=1

GGGCAGAGAGGCAAGCCTGCCAGGGTGGCCAGAGCTGTGTGCCCTGCAGAGCTTCTGAAC

TGGACATACAGGTCCAGTCCCAAAAGTAGTGTCCAGGGAGCACCC

>4753 len=105 tot-seq=1

TCCACCCACAGGTGGGGTATGGAGACCGCCCGCTTCCTTCCTATTCCATGATGCTGTGGC

TCTGGGCTGTTGGCTGTCCTCAGTTGCGCTTGGGATTCCTGCCAG

>4754 len=105 tot-seq=1

GATGCAGCGACGCCGCGTGAAGGACGAAGTATTTCGGTACGTAAACTTATATAAGCAGGG

AAGAAAATGACGGTACCTGACCAAGAAGCCCCGGCTAACTACGTG

>4755 len=105 tot-seq=1

GGGAGGAAGGTACAGAGTATCGTAAACCTCTTTTTCAGGGAACAAAGGCTGGGACGTGT

CCCCGGATGAGTGTACCTCAAGAAAAAGCATCGGCTAACTCCGTG

>4756 len=105 tot-seq=1

GAACCAGCCAAGTAGCGTGAGCGATGAAGGCTTTCGAGTCGTAAAGCTCTGTCCTATGGG

AAGATAATGACGGTACCATAGAAGAAAGCCCCGGCTAATTACGTG

>4757 len=106 tot-seq=1

TGGGGAATATTGGACAATGGGCGCAAGCCTGATGCAGCCATGCCGCGTGAGTGATGAAGG

TCCTAGGGTCGTAAAGCTCTTTCACTGGTGAAGATAATGACGGTAA

>4758 len=106 tot-seq=1

TGGGGAATTTTGCACAATGGGCGCAAGCCTGATCCAGCAACGCCGCGTGAGTGAAGAAGG

CCTTCGGGTTGTAAAGCTCTTTTGTCGGGAAAGAAAACCTCTGTCC

>4759 len=106 tot-seq=1

AAAAAGTCAAGTAAAGTTTGTGCAGCCCATACATCCTTGAATTCATGGCCTTGTGCTGTC

ACACCTACCAGGACAACACACTTACAGAAAACTGACTCCACATTTC

>4760 len=106 tot-seq=1

TGGGGAATATTGCACAATGGGCGCAAGCCTGATGCAGCCATGCCGCGTGTATGAAGCAGG

TCTTCGGGGTGTAAAGTACTTTCAGCGGGGCGGAAGGCGATAAGGT

>4761 len=106 tot-seq=1

TAGGGAATCTTCCACAATGGACGCAAGTCTGATGGAGCAACGCCGCGTGAGTGAAGAAGG

GTTTCGGCTCGTAAAGCTCTGTTGGCAGGGAAGAAAGGTAGAGGAC

>4762 len=106 tot-seq=1

AGGGTAGAGGCCAGGCTGGAAGTGAGATGGGCAGCAGGGCCCCGTGGCAGGGGCTGGCCT

GCCAATCAAACAAAGGCTGTAGTTGCGGAATAAAGCCCACATTTCC

>4763 len=106 tot-seq=1

TGGGGAATCTTGCGCAATGGGCGAAACCCTGATGCAGCGACGCCGCGTGCGGGAAGAAGG

CCTTCGGCTCGTAAACCGCTTTTGATTGGGAGCAAGCTTTCGGGTG

>4764 len=106 tot-seq=1

TAGGGAATCTTCCACAATGGACGAAAGTCTGATGGAGCAACGCCGCGTGAGAGTAACTGT

TCACGCAGTGACGGTATCCAACCAGAAAGTCACGGCTAACTACGTG

>4765 len=106 tot-seq=1

TAGGGAATCTTCCACAATGGACGCAAGTCTGATGGAGCAACGCCGCGTGAGTGAAGAAGG

GTTTCGGCTCGTAAAGCTCTGTCGGTGGGGAAGAATGACAGGGGTA

>4766 len=106 tot-seq=1

TAGGGAATCTTCCACAATGGACGCAAGTCTGATGGAGCAACTCTGCGTGTGTGAAGAAGG

TTTTCGGATCGTAAAGCTCTGTTGTTGGTGAAGAAGGAAAGAGGTA

>4767 len=106 tot-seq=1

AGAAGGGTTTCGGCTCGTAAAGCCCTGTTGTTGGTGAAGAAGGATAGAGGTAGTAACTGG

CCTTTGTTTGACGGTAATCAATTAGAAAGTCACGGCTAACTACGTG

>4768 len=106 tot-seq=1

TGGGGAATCTTCCGCAATGGGCGCAACTCTGACGGAGCGACGCCGCGTGCGGGAAGAAGG

CCTTCGGGTCGTAAACCGCTTTTGTTTGGGAGCAAGCTTTCGGGTG

>4769 len=106 tot-seq=1

AACCAGCCTAGAGTGTCTGCAGTAAGTATTGACTCAGAGAAAGGCAGGGCAGTAAAGGCA

TATTTCCCCAAGGAAGGAGGCCTTCTGGTATCAGCATTTCAAGTTC

>4770 len=106 tot-seq=1

AGGAGTGTGCCACATTAGCATGCTGGAATAATTTTAATACATCCTTCTTTAAAAAATGGG

CTTTTTGAGTAACAAGCATCCTAATGATCAATTCTGTCATTTCATT

>4771 len=106 tot-seq=1

AGAAGGTTTTCGGCTCGTAAAGCTCTGTTGTTGGTGAAGAACGTGCGGGAGAGTAACTGT

TCACTCAGTGACGGTATCCAACCAGAAAGTCACGGCTAACTACGTG

>4772 len=106 tot-seq=1

TGGGGAATCTTGCACAATGGGCGAAAGCCTGATGCAGCGACGCCGCGTGCGGGATGAAGG

CCTTCGGGTGAGTGTACCTTTCGAATAAGCGCCGGCTAACTACGTG

>4773 len=106 tot-seq=1

TAGGGAATCTTCCACGATGGACGCAAGTCTGATGGAGCAACGCCGCGTGAGGGAAGAAGG

GCTTCGGCTCGTAAAGTTCTGTTGTTGGGGAAGAAGGACAGGGGTA

>4774 len=106 tot-seq=1

TGGGGAATCTTGCGCAATGGGGGCAACCCTGACGCAGCGACGCCGCGTGAGGGATGAAGG

CCTTCGGCTTGTAAACCGCTTTTGATTGGGAGCAAGCCTTCGGGTG

>4775 len=106 tot-seq=1

TAGGGAATCTTCCGCAATGGACGCAAGTCTGACGGAGCAACGCCGCGTGAGGGAAGAAGG

GTTTCGGCTTGTAAAGCTCTGTTGTTGGGGAAGAAGGAGTGGGGTA

>4776 len=106 tot-seq=1

AGCACTCTGTAAAATGGACCAATCAGCAGGATGTGGGCGGGGACAAATAAGGGACTAAAA

GCTGGCGACCCGCCCCACTCCCTCCAGTCCCTCCAGTCCCTCCCAG

>4777 len=106 tot-seq=1

TAGGGAATATTCCACAATGGGCGCAACCCTGACGCAGCGACGCCGCGTGCGGGATGAAGG

CCTTCGGGTTGTAAAGCGCTTTTGTTGGGGAGCAAGCTTTCGGGTG

>4778 len=106 tot-seq=1

GTTCACCCCCAAATTCCCAGGAAAGAATAAAGAGGAAATAAGTGCAGCAGGACAGCAGCA

AGGACTGACATTAGAGAGAAGGAAGAATTTCCCAATGCCAAAAGAA

>4779 len=106 tot-seq=1

TGATGCAGCGACGCCGCGTGAACGAAGAAGGCTTTCGAGTCGTAAAGTTCTTTTATATGG

GAAGATAATGACGGTACCATAGGAGGAAGCCACGGCTAACTACGTG

>4780 len=106 tot-seq=1

TGATGAAGCAATACCACGTGAGTGATGAAGGTTTTCGGATCGTAAAGCTCTGTCTTTGGG

GAAGATAATGACGGTACCCAAGGAGGAAGCCACGGCTAACTACGTG

>4781 len=106 tot-seq=1

GGGAGGCGGCAGGGATGGCAGGGCAGAGGGAGGCAGGAGACTCTTTAGCTTTAGGAGGCC

GTGGTAGGATCCTGGAGTGGAGGACGGTGCCGTGCCACAGTGGCAG

>4782 len=106 tot-seq=1

CTGATGCAGCGACGCCGCGTGAGCGAAGAAGTATTTCGGTATGTAGAGCTCTATCAGAAG

GGAAGAGAGTGATGGTACCTTACTAAGAAGCTCCGGCTAAATACGT

>4783 len=106 tot-seq=1

CAAGCAGAAGACGGCATACGAGATAACACTGTGACTGGAGTTCAGACGTGTGCTATCCGA

TCTCCTACGGGAGGCAGCGGCCGCGGTAATATTGAGATCGGAAGAG

>4784 len=106 tot-seq=1

AGGTAGTGACGAAAAATAACAATACAGGACTCTTTCGAGGCCCTGTAATTGGAATGAGGC

CACTTTAAAGCCGGTAACGAGGATCCATTGGAGGGCAAGTCTGGTG

>4785 len=106 tot-seq=1

AATCGCTTGAACCCAGCAGCCGCGGAATCCGCAGATCGGAAGAGCGTCGTGTAGGGAAAG

AGTGCGCCAATGTGTAGATCTCGGTGGTCGCCGCATCATTAAAAAA

>4786 len=106 tot-seq=1

TAGGGCAGGGTTGGGTCAGTATCTGGCATTTGCTCCTGGAGTGAGGCAGAGGTTATTGTT

CTGGAGGTTCAGTGTAAAATAGGAAGAAAGGGAACAGGCTAGTGCC

>4787 len=106 tot-seq=1

GGGTGCTTGAGGCCAGAAGTTCGAGGTAACAGTGAATCATGATTGTGCCCCTTCATCCCA

GCCTGAATGACAGAGCGAGACCCTATCTCTAACAAAACTATATGAT

>4788 len=106 tot-seq=1

TAGGGAATCTTACACAATGGACGCAAGTCTGATGGAGCAACGCCGCGTGCGTGAAGAAGG

TTTTCGGATCGTAAAGCTCTGTTGTTGGTGAACAAGGATACAGGTA

>4789 len=106 tot-seq=1

AGGAAAAGAGAGGAAGCAACAAAGAGAGGGAAACCTGCTATCGTAGCCAATGACCCCTCC

AAGAGGGAAAGGCAGTCCACACAGAGGACCCTGCCTCTGATGAAGC

>4790 len=106 tot-seq=1

TGAGCCAGCCATGCCGCGTGAGTGATGAAGGCCTTAGGGTGGTAAAGCTCTTTTGTCCGG

GACGATAATGACGGTACCGGAAGAATAAGCCCCGGCTAACTTCGTG

>4791 len=106 tot-seq=1

GAAGGTGTTCGGATCGTAAAGCGCTGTTGTAAGAGAAGAACGTGTGTGAGAGTGTAAATT

TCACACAGTGACGGTAACTTACCAGAAAGGTACGGCTAACTACGTG

>4792 len=107 tot-seq=1

TGGGGAATATTGCACAATGGGCGAAAGCCTGATCCAGCCATGCCGCGTGTGTGAAGAAGG
TCTTCGGATTGTAAAGCACTTTAAGCTGGGAGGAAAATCAGTTTCCT
>4793 len=107 tot-seq=1
CAAAAAAAAAAACAAAAAAAAAACAGCACAAATGAAGGTGAGGTTGATACTTGGACAAGA
AAATTGTTTTGACACATGTGAAAACACCACTTGAGTTTTATATGTTC
>4794 len=107 tot-seq=1
AGCTGCCTCCTACCTTCAGCACACACCTCTTGACCCAGCAAGGTTCCTTCCCTGTCGTCC
CTTCAATCTTTATTTCTGGAAACCAGGCTACTCTGGTTATGGAGATA
>4795 len=107 tot-seq=1
AAGAAGGGTTTCGGCTCGTAAAGCTCTGTTGTTGGTGAAGAAGGACAGGAAAAAAAACTG
ACCAAGATTTGACGGTAATAAAAAAGAAAGTCACGGCTAACTACGTG
>4796 len=107 tot-seq=1
TGGGGAATATTCCGCAATGGGGGAAACCCTGACGCAGCAACGCCGCGTGCGGGATGAAGG
CCTTCGGGTCGTAAACCGCTTTTGATTGGGAGCAAGACTTTTGGGTG
>4797 len=107 tot-seq=1
TGGGGAATATTGCGCAATGGGCGAAACTCTGACGCAGCGACGCCGCGTGAGGGATGAAGG
CTTTCGGGTTGTAAACCGCTTTTGATTGGGAGCAAGCCTTTTGGGTG
>4798 len=107 tot-seq=1
CTCTGTCTTGGGCACTGTATTAGATTCCTAGAGCTGAGCAACAAGAACAAAACTCAATCT
AAAAAAATAAAAATAATAAAGTAAAATCCAACATCGCCACAGTCCGC
>4799 len=107 tot-seq=1
TAGGGAATCTTCCACAATGGGCGCAAGCCTGATGGAGCAACACCGCGTGAGGGAAGAAGG
GTTTCGGCTCGTAAAGCTCTGTTGTTGGAGAAGAAAGTGGGCGAGAG
>4800 len=107 tot-seq=1
TAGGGAATCTTCCACAATGGACGCAAGTCTGATGGAGCAACGCCGCGTGAGGGAAGAAGG
TTTTCGGATCGTAAAGCTCTGTTGTTGGGGAAGAAGGACAGAGGAAG
>4801 len=107 tot-seq=1
AGCGACGCCGAGTGCGGGATGAAGGAATTAGGGTTGTAAAAAGAGGGTGATTGGGAGAAA
GATTTAGGGTGAGTGTACCTTTCGAATAAGCGCCGGCTAACTACGTG

>4802 len=107 tot-seq=1

AAAAGTAGTGGCCACTGCCATTTCCAAGGACATAATGCTTCCCACCAATTTTTACTCATC

AGCGTTGCCCCCTGGCATTCCACCCCTCGAGTAACGCCAACACCCAG

>4803 len=107 tot-seq=1

GCAGCAACGCCGCGTGCGTGATGGCGGCCTTCGGGTTGGAAACCGCTTGCGCCTGTGACG

AAGCGTGAGTGACGGTAATGGGTAAAGAAGCACCGGCTAACTACGTG

>4804 len=107 tot-seq=1

TAGGGAATATTGCACAATGAGCGAAAGCCTGATGGAGCGACACAGCGTGCAGGATGAAGG

CCTTCGGATTGTAAAGTGCTGTTATAAGGGAAGAACATTTGCAGTAG

>4805 len=107 tot-seq=1

AAGAAGGGTTTCGGCTCGTAAAGCTCTGTTGTTGGTGAAGAAGGATAGAGTTAGTAACTG

ACCTTTGTTTGACGGTACTCAATTAGAAAGTCACGGCTAACTACGTG

>4806 len=107 tot-seq=1

TAGGGAATCTTCCACAATGGACGCAAGCCTGATGGAGCAACGCCGCGTGAGGGAAGAAGG

CCTTCGGATCGTAAACCTCTGTTGTTAGAGAAGAACACGAGTGAGAG

>4807 len=107 tot-seq=1

AAGAAGGGTTTCGGCTCGTAAAGCTCTGTTGTTGGTGACGAAGGACAGGGGTAGTAACTG

ACCTTTGTTTTTCGTTCATCAATTAGAAAGTCACGGCTAACTACGTG

>4808 len=107 tot-seq=1

TGGGGAATCTTGCACAATGGGCGCAAGCCTGATGCAGCGACGCCGCGTGCGGGATGAAGG

CCTTCGGGTCGTAAACCGCTTTTGTTTGGGAGCAAGCCTTTTGGGTG

>4809 len=107 tot-seq=1

TGGGGAATATTGGACAATGGAGGCAACTCTGATCCAGCAATTCCGCGTGTGTGAAGAAGG

TTTTAGGACTGTAAACCCCTTTTAGTAGGGAAGAAAGAAATGACGGT

>4810 len=107 tot-seq=1

TAGGGAATCTTCCACAATGGACGCAAGCCTGATGGAGCAACGCCGCGTGAGTGAGGAAGG

GCTTCGGATCGTAAAGCTCTGTTGTTGGTGAAGAAGGGCAGGGGTAG

>4811 len=107 tot-seq=1

TAGGGAATATTCCACAATGGACGCAAGTCTGACGGAGCAACGCCGCGTGAGTGAAGAAGG

CTTTCGGATCGTAAACCTCTGTTGTTGGTGAAGAAGGATAGAGGTAG

>4812 len=107 tot-seq=1

TGGGGAATATTGCGCAATGGGCGAAACCCTGACGCAGCAACGCCGCGTGAGGGATGAAGG

CCTTCGGGTTGTAAACCGCTTTTGATTGGGAGCAAGGCTTTTGGGTG

>4813 len=107 tot-seq=1

AGAAGGCTCTTGGGTTGTAAAGCTCTTTCGGCCGGGAAGAAGGGTCTGCATGCGAACAGC

ATGCAGATTTGACGGTACCGGAAGAAGAAGGACCTGCTAACTTCGTG

>4814 len=107 tot-seq=1

TAGGGAATATTCCACAATGGACGCAAGCCTGACGGAGCAACGCCGCGTGCGTGAAGAAGG

CTTTCGGATCGTAAAGCTCTGTTGTTGGGGAGCAAGGATAGTGGTAG

>4815 len=107 tot-seq=1

TAGGGAATCTTCCACAATGGACGCAACCCTGATGGAGCAACGCCGCGTGAGTGAAGAAGG

GTTTCGGCTCGTAAAGTTCTGTTGTTGGTGAAGAAGGTCAGGGGAAG

>4816 len=107 tot-seq=1

CCAGGACACTCCTTCCCTCCGCCGGCCACGTTTGCAGGGAGCCCCTGAGAGGCAGCGGCA

GCCCGGATGCTGAATCCCATTCGCCCCGGTCATTTCCGGAGCGTCTT

>4817 len=107 tot-seq=1

TAGGGAATCTTCCACAATGGACGCAAGTCTGATGGAGCAACGTCGCGTGAGTGAAGAAGG

GTTTCGGCTCGTAAACTTCTGTTGTTGGTGAAGAAGGACGGGGGTAG

>4818 len=107 tot-seq=1

TAGGGAATCTTCCACAATGGGCGCAAGCCTGATGGAGCAACACCGCGTGAGTGAAGAAGG

GTTTCGGCTCGTAAAGCTCTGTTGGTGGAGAAGAAAGTATCTGTGCG

>4819 len=107 tot-seq=1

TAGGGAATATTCCACAATGGACGCAAGTCTGATGCAGCAACGCCGCGTGAGGGAAGAAGG

TTTTCGGATCGTAAAGCTCTGTTGTTGGTGAAGAAGGCTAGAGGTAG

>4820 len=107 tot-seq=1

TAGGGAATCTTCCACAATGGGCGCAAGCCTGATGGAGCAACGCCGCGTGAGTGAAGAAGG

TTTTCGGATCGTAAGTAATCAACCAGAAAGTCACGGCTAACTACGTG

>4821 len=107 tot-seq=1

TGGGGAATCTTGCACAATGGGCGAAAGCCTGACGCAGCGACGCCGCGTGCGGGATGAAGG

CCTTCGGGTTGTAAACCGCTTTTGTTTGGGAGCAAGCCCTGTGGGTG

>4822 len=107 tot-seq=1

GCAGGATGACGGACCTATGGGTTGTAAACTGCTTTTATATGGGGATAAAGTGGGGAACGG

GTTCCCGTTTCCAGGTACCATATGAATAAGGACCGGCTAATTCCGTG

>4823 len=107 tot-seq=1

TAGGGAATCTTCCACAATGGACGCAAGCCTGATGGAGCAACGCCGCGTGAGTGATGAAGG

CCTTCGGATTGTAAAGCTCTGTTGTTGGTGAAGAAGGATAGAGGTAA

>4824 len=107 tot-seq=1

CAGTAGCAGCCTCTCAGCAGGACAAGGGGCCAACCTAGAGTCCCCTTTTGCTGTGGTGAC

TCCCAAGAACTGTGATGGCTCACCTGGGGGAAGGTTCTAGAAGCTCC

>4825 len=107 tot-seq=1

TGGGGAATATTGCGCAATGGGCGAAACCCTGACGCAGCGACGCCGCGTGCGGGATGAAGG

CCTTCGGGTCGTAAACCGCTTTTGTTTGGGAGCAAGCTTTGGGGTGA

>4826 len=107 tot-seq=1

CAGGGTGATGCGGGTAAGGCCCATACAAAAGGCCGACAGCGCGATACCCGCGAGGATCAG

TTTGTTTCTGTCATGGGTATGACGAAATCCGCCTCCTGCGGTCATGA

>4827 len=107 tot-seq=1

CTGGTGCAGCGACGTCGCGTGAGCGAAGAAGTATTTCGGTGTGTAAAGCTCTATCAGAAG

GGAAGAAAATGACGGTACCTTACTAAGAAGCTTCGGCTAAATACGTG

>4828 len=107 tot-seq=1

CCTTTAAATTGTACCGTCTTGAGCATAGAAAAGCCTGAATTATGTAAAAGCTGGCCAGAT

GCCCACATCTGGCAACAGTCAGGTTGTTTGGTAAGCAGGAGGTTCCC

>4829 len=107 tot-seq=1

TTAATTCTCCGTGGCATTAGCTCCCCTGCCCCACCATGCTCCAGCACCTACTGGGGTGCC

TGGCTTGACTCCCCGGCTCTACAAAACAGCCATCTTCCTTTGTCCTT

>4830 len=107 tot-seq=1

CCCTGATGCAGCAACGCCGCGTGAGTGATGAAGGTTTTCGGATCGTAAAGCTCTTTCTAT

GGGAAAGATAATGACGGTACCCAGGGAGGAAGCCACGGCTAACTACG

>4831 len=107 tot-seq=1

CTGATGCAGCAACGCCGCGTGAGTGATGAAGGTTTGAGGGTAGTAAAGCTCTGTGGTGGG

GGAAGATAATGACGGTACCCAAGGAGGAAGCGACGGCTAACTACGTG

>4832 len=107 tot-seq=1

TAGGGAATCTTCGGCAATGGACGGAAGTCCGACCGAGCAACGCCGCGTGAGGGAAGAAGG

TTTTCGAATCGTAAAGCTCTGTTGTAAGGGAAGAACGAGTGTGAGAG

>4833 len=107 tot-seq=1

AAGACGCTACAGGAGCAGGGTGAGAGGCTGCGAAAGCAGGAGCAGAGGCTACGCAAACAG

GAGGAGAGGCTGCGAAAGGAGGAGGAGAGGCTGCAAAAGCAGGAAAA

>4834 len=107 tot-seq=1

GAACACCGGGTCCAGGCCGCAAAGCTCAATGGCGTGGTAGACAGCCTTGTGGCAGTGGCG

GGCCACCAGAACCTGGTCCCCCCGGCGGGTGGCCGCCCGGATGGCCG

>4835 len=107 tot-seq=1

GAGGTAGTGACGAAAAATAACAATACATGACTCTTTCGAGGCCCTGTAATTGGAATGAAT

CCACTTTAAATCCTTTAACGAAGATCCATTGGAGGGCAAGTTTAGTG

>4836 len=107 tot-seq=1

CTTGGGTTACCCCTAGGGTCAGGTCATCACCATTTGCCTCATCTCCTTCCACAGCCAACT

TGGAAGGATTCCCCAGCCTTGTGCCAGGAGAGGCTGGCACGGCTGCC

>4837 len=107 tot-seq=1

AAGAAGTTTTTCGGATCGTAGAGCTCTGTTGTTGTTGAATAAGGATAGAGGTAGTAACTG

GCCTTTATTTGACGGTAATCAACCAGAAAGTCACGGCTAACTACGTG

>4838 len=107 tot-seq=1

TAGGGAATCTTCCACAATGGACGCAAGTCTGATGGAGCAACGCCGCGTGAGTAGTAACTG

GCCTTTATTTGACGGTAATCAACCAGAAAGTCACGGCTAACTACGTG

>4839 len=107 tot-seq=1

TGCATTTCAATATCTGGGGAAAAGCATCTCAGTCCAGGGACTGGCAGATGTCACAGCTCA

GAGGTGGGAGCTGCCCTGAGGGAGTGTGTCCCGGAACGACCAGGAGG

>4840 len=107 tot-seq=1

AAGGAGAATGAGGAGTACATCTTGGTTTGTGTCTAGGTGAGTTTGAGGGGACATCACACT

GGAGTTGGCTCCAACTTTGCTAGCATAAAATAAATGTGTTAGATGAT

>4841 len=107 tot-seq=1

ATGAAGTTCTTCGGATCGCAAAACTCTGTTATTAGGGAATAACAAACGTGTAAGTAACTG

TGCACGTCTTGACGGTACCTAATCAGAAAGCCACGGCTAACTACGTG

>4842 len=107 tot-seq=1

CTGATACAGCGACGCCGCGTGAGGGAAGAAGGTTTTCGGATTGTAAACCTCTGTTCTTAG

TGACGATAATGACGGTAGCTAAGGAAAAAGCACCGGCTAACTACGTG

>4843 len=107 tot-seq=1

GCAGGATGACGGCCGTATGTGTTGTAAACTGCTTTTGTTTGGGAATAATCGACATGACGT

GTAATGTTGTGCATGTACCATTCGAATAAGGACCGGCTAATTCCGTG

>4844 len=107 tot-seq=1

GAATTCTTGAACCCAGGAGTTCGAGACCAACCTGGGCGACATGGTGAAACTCTGTCTCTA

TAGAAAATAAAAAAATTAGCTGGGTGTGGTGGCACATGCCTGTAGTC

>4845 len=107 tot-seq=1

CTGATGCAGCGACGCCGCGTGAGCGAAGAAGTATCTCGGTATGTAAAGCTCTATAGGCAG

GGAAGAAAATGACGGTACCTGACGAAGAAGCCCCGGCTAACTACGTG

>4846 len=107 tot-seq=1

CGGATGCAGCGACGCCGCGTGAGCGATGAAGTATTTCGGTATGTAAAACTATATCAGCAG

GGAAGAAAATGACGGTACCTGACTAAGAAGCACCGGCTAAATACGTG

>4847 len=107 tot-seq=1

GGAAGGTTACCAAGGAAAAAAAAAAAAATGAAGTCAAGGGAATAAGAAAATCCCCAGGAC

TTCTACCCGACCCAATGCAATAAACGCGATAGAGACAAACCCTGGAG

>4848 len=107 tot-seq=1

AGAAGGTTTTCGGATCGTAAAGCTCTGTTGTAAGAGAAGAACGAGTGTGAGAGAGGAAGG

TTCACACTGTGACGGTAACTTTCGAAAAAGGGACGGCTAACTACGTG

>4849 len=107 tot-seq=1

CTGATGCAGCGATGCCGCGTGAGCGAAGAAGTATTTCGGTATGTAAAGCTCTATAAGCAG

GGAAGAAAATGACGGTACCTGACGAAGAAGCTCCGGCTAAATACGTG

>4850 len=108 tot-seq=1

TTTTTTTTCAAGCAGAAGACGGCATACGAGATAAGGCCGTGATGGAGTTCAGACGTGTGC

TCTTCCGATCTCCTACGGGAGGCAGTAGTCGGGCCGCCTCAGCTATGA

>4851 len=108 tot-seq=1

TACAACACCTGGATGTCTGCTAGCTTTACTTTCTCTGGCAGCCACTAGAGCAGCATACAG

GGGAATTGCTTCAGGACCTAGGCATTGAGAGACTTTCCATGGAGGGCT

>4852 len=108 tot-seq=1

CCTATAGGAGGTGGGAACCCAGAGCATTTGTAGACCAGTCAGAGCCTACAGCCCTAAAGG

ACCTGGCCTAGCCACACCCAGTGTTTGAAGAACCCCACTACCACAAGT

>4853 len=108 tot-seq=1

TGGGGAATATTGCACAATGGGGGAAACCCCGATGCAGCAACGCCGCGTGAGTGAAGAAGG

ATTTCGGTATGTAAAGCTCTATCAGCAGGGAAGATAAAGACGGTTTCT

>4854 len=108 tot-seq=1

GAGTGGGAATGGAATGGGGGTCTTCCAAAGGGGAGACCTGGAAAGGGGAAAACATTTGAA

ATGTAAATAAAGAAAATATCCAAGAAAGAAAAAGAGGCAGAGGCCAGA

>4855 len=108 tot-seq=1

TCAAGCAGAAGACGGCATACGAGATGGCAATGTGACTGGAGTTCAGACGTGTGCTCTTCC

GTTCTCCTACGGGAGGCAACAGCATAGCCTGGGAGGGTGAGTGGTTAC

>4856 len=108 tot-seq=1

AACAGAAGAACGGCGCGGGGGTTGGGGGAAGGGAGCGGGTGCCGAGACTCACACGAGGGC

CTGGAGCCGGGGACGGGGAGAGGAGACTCGGGACACAGCCTCTGAGTA

>4857 len=108 tot-seq=1

TGGGGAATATTGCACAATGGGCGCAAGCCTGATGCAGCCATGCCGCGGTAATTCGTAGAT

CGGTAGAGCGTCGTGTAGGGAAAGAGTGTGCCAATGTGTAGATCTCGG

>4858 len=108 tot-seq=1

TGGGGAATATTCCGCAATGGGGGCAACTCTGATGCAGCGACGCCGCGTGCGGGATGAAGG

CCTTCGGCTCGTAAACCGCTTTTGTTTGGGAGCAAGCTCTCGGGTGAG

>4859 len=108 tot-seq=1

TAGGGAATCTTCCACAATGGGCGAAAGCCTGATGGAGCAACGCCGCGTGGGTGAAGAAGG

TCTTCGGATCGTAAAACTCTGTCGTTGGAGAAGAACGTGCGTGAGAGT

>4860 len=108 tot-seq=1

GAAGAAGGGTTGCGGCTCGTAAAGCTCTGTTGTTGGTGAAGAAGGACAGAGGTAGTAACT

GGCCTTGGTTTGACGGTAATCAATTAGAAAGTCACGGCTAACTACGTG

>4861 len=108 tot-seq=1

CAGCGACGCCGCGTGGGGGATGACGGCCTTCGGGTTGTAAACTCCTTTCACTAGGGACGA

AGTGTATTTGTGACGGTACCTGGAGAAGAAGCACCGGCTAACTACGTG

>4862 len=108 tot-seq=1

TCGGGAATCTTCCACAATGGACGCAACCCTGATGGAGCAACGCCGCGTGAGTGAAGAAGG

TTTTCGGCTCGTAAAGCTCTGTTGTTAGTCAAGAACGACAGGGAGAGT

>4863 len=108 tot-seq=1

TAGGGAATCTTCGGCAATGGACGCAAGTCTGATCGAGCAACGCCGCGTGAGTGAAGAAGG

TTTTCGGATCGTAAAGCTCTGTTGTTAGAGAAGAAGGAGAGGGGGAGT

>4864 len=108 tot-seq=1

GAAGAAGGGTTTCGGCTCGTAAAGCTCTGTTGTTGGTGAAGAAGGATAGAGGTAGTAACT

GACCTTTATGTGACGGTAATCAATTAGAAAGTCACGGCTAACTACGTG

>4865 len=108 tot-seq=1

TGGGGAATCTTCGACAATGGGCGCAAGCCTGATCCAACAATACCGCGTGAGTGATGAAGG

CCTTCGGGTCGTAAAGCTCTTTTACCAGGGATGATAGTGACAGTACCT

>4866 len=108 tot-seq=1

TGACCCAGCAACGCCGCGTGAAGGAAGAAGGCTTTCGGGTTGTAAACTTCTTTTGTCAGG

GACGAAACAAATGACGGTACCTGACGAATAAGCCACGGCTATCCACGT

>4867 len=108 tot-seq=1

TAGGGAATCTTCCACAATGGACGAAAGTCTGATGGAGCAACGCCGCGTGCGTGAAGAAGG

CTTTCGGATCGTAAGGCTCTGTTGTTGGTGAAGAAGGATAGAGGTAGT

>4868 len=108 tot-seq=1

GAAGAAGGGTTTCGGATCGTAAAGCTCTGTGGTTGGTGAAGAAGGACAGAGGTAGTAACT

GGCCTTTATTTGACGGTAATCAAATAGAAAGTCACGGCTAACTACGTG

>4869 len=108 tot-seq=1

TGGGGAATATTCCACAATGGACGCAAGTCTGATGGAGCAACGCCGCGTGAGTGAAGAAGG

TTTTCGGATTGTAAAGCTCTGTTGGTGGTGAAGAAGGATAGAGGGAGT

>4870 len=108 tot-seq=1

CAATGCTGTTTTCTTCTTCCTTTTAAGTGTATAACATTTTTACCCTCTTTCCAGATGCAG

CAGCCCCAGCAGCCCCAGCCCCAGCAGCCTCCCCAGCCCCAGCAGTCC

>4871 len=108 tot-seq=1

TAGGGAATCTTCCACAATGGGCGCAAGCCTGACGGAGCAACACCGCGTGAGGGAAGAAGG

GTTTCGGCTCGTAAAGCTCTGTTGTTGGGGAAGAACGTGGGTGGGAGT

>4872 len=108 tot-seq=1

TAGGGAATCTTCCACAATGGACGAAAGCCTGATGCAGCAACGCCGCGTGCGTGAAGAAGG

CTTTCGGGTCGTAAAGCTCTGTTGTTGGTGAAGAAGGTTAGAGGTAGT

>4873 len=108 tot-seq=1

TAGGGAATCTTCCACAATGGGCGCAAGTCTGATGGAGCAACGCCGCGTGAGGGAAGAAGG

ATTTCGGATCGTAAAGCTCTGTTGTTGGTGAAGAAGGATAGGGGAAGT

>4874 len=108 tot-seq=1

GAAGAAGGGTTGCGGCTCGTAAAGCTCTGTTGTTGGTGAAGAAGGACAGGGGGAGTAACG

GACCTTTGTTTGACGGTAATCAATTAGAAACTCACGGCTAACTACGTG

>4875 len=108 tot-seq=1

TAGGGAATCTTCCACAATGGACGAAAGCCTGATGCAGCAACGCCGCGTGCGGGATGAAGG

TTTTCGGATTGTAAAGCTCTGTTGTTGGTGAGGAAGGATAGAGGTAGT

>4876 len=108 tot-seq=1

TAGGGAATCTTCCACAATGGACGCAAGTCTGATGGAGCAACGCCGCGTGAGTGAAGAAGG

GTTTCGGATCGTAAAGCTCTGTTGTCAGTGAAGAAGGACTGGGGGAGT

>4877 len=108 tot-seq=1

GAAGAAGGGTTTCGGCCCGTAAAGCTCTGTTGTTGGTGAAGAAGGACAGGGGTAGTAACT

GACCATTGTTTGACTGGAATCAATTAGAAAGTCACGGCTAACTACGTG

>4878 len=108 tot-seq=1

TAGGGAGTCTTGCACAATGGACGCAAGTCTGATGGAGCCACGCCGCGTGCGTGAAGAAGG

CTTTCGGATCGTAAAGCTCTGTTGTTGGTGAGGAAGGATAGAGGTAGT

>4879 len=108 tot-seq=1

TTTCAGCCATGCTATCTCAGTTATTGCTTGCACTAGGGCAGAGTTCGCAGGGAGCCGAAG

CAAAATAAAATCCCAGTGCTTTGAGAATGGGAGGCATTCAGAGGCAAC

>4880 len=108 tot-seq=1

AAAGAAGGGTTTCGGCTCGTAAAGCTCTGTTGTTGGTGAAGAAGGACAGGGGTAGTAACT

GACCTTTGTTTAACAAAAATCAATTAGAAAGTCACGGCTAACTACGTG

>4881 len=108 tot-seq=1

TAGGGAATCTTCGGCAATGGACGCAAGTCTGATGGAGCAACGCCGCGTGAGTGAAGAAGG

ATTTCGGATCGTAAAGCTCTGTTGTAGGAGAAGAAGGAGAGTGAGAGT

>4882 len=108 tot-seq=1

TGGGGAATATTGCGCAATGGGAGCAACCCTGACGCAGCGACGCCGCGTGCGGGATGAAGG

CCTTCGGGTCGTAAACCGCTTTTGATTGGGAGCAAGGCTTCGGGTGAG

>4883 len=108 tot-seq=1

TAGGGAATCTTCCACAATGGGCGCAAGTCTGATGGAGCAACGCCGCGTGAGTGAAGAAGG

GTTTCGGCTCGTAAAGCTCTGTTGTTGGTGAAGAAGGAACGGGGTATT

>4884 len=108 tot-seq=1

TGGGGAATATTGCACAATGGACGGAAGTCTGATGCAGCGACGCCGCGTGGAGGGAGAAGG

CCTTCGGGTTGTGAACTTCTTTTTCTCGTGAAAAAGGCATGCTTTTGG

>4885 len=108 tot-seq=1

CCTGATGGAGCAATGCCGCGTGCAGAATGAAGGCCCTCGGGTCGTAAACTGCTTTTATGA

GAGAAGAATATGACAGTAACTAATGAATAAGGGACGGCTAACTACGTG

>4886 len=108 tot-seq=1

CTCCTCTCAAGCTCACTCTCTAGGTCTCCAGGGTAGGAGTGTTTTTCTAGCAAGAATCTT

AGTCAGGAGTAAGCTCTGTGCGAGAGACCTGTGAATAACCAGATAACC

>4887 len=108 tot-seq=1

GATGTGGATGGTGTGGCTGCTCTTTGTGCATTCACAGTGGCAGCCTGCCGAGCAGCCCAG

CCAACAGTGCCCTGTCTTTGCTCTTTTGTTGTTTCGTACCATGTGGTG

>4888 len=108 tot-seq=1

TGGGGAATATTGCACAATGGGCGCAAGCCTGATGCAGCCATGCCGCGTGTGTGAAGAAGG

CTTTCGGGATGTAAAGCACTTTCAGCGGGGAGGAAGGCGATAAGGTTA

>4889 len=108 tot-seq=1

TAGGGAATCTTCGGCAATGGACGGAAGCCTGACCGAGCAACGCCGCGTGAGTGAAGAAGG

ATTTCGGATCGTAAAGCTCTGTTGTAAGGGAAGAACAAGTGTGAGAGT

>4890 len=108 tot-seq=1

AAGAAGGTGTTCGGATCATAAAGCTCTGTTGTAAGAGAAGAACGAGTGTGAGAGCGGAAA

GTTCACAATGTGACGGTATCTTACCAGAAAGGGACGGCTAACTACGTG

>4891 len=108 tot-seq=1

AAGAAGGTGTTCGGATCGTAAAGCTCTGTTGTAAGAGAAGAACGAGTGTGAGAGTGGAAA

GTTCTCACTGTGACGGTACCTTACGAGAAAGGGACGGCTAACTACGTG

>4892 len=108 tot-seq=1

GAAGAAGGGTTTCGGCTCGTAAAGCTCTGTTGTAAGTGAAGAAGGGCAGGGGTAGTAACT

GGCCTTTGTTTGACGGTAATCAATTAGAAAGGCACGGCTAACTACGTG

>4893 len=108 tot-seq=1

GAAGAAGGCCTTCGGGTTGTAAAGCCCTTTTGTTGGGAAAGAAAAGCAATCGATTAATAC

TCGGTTGTTATGACGGTACCCAAAGAATAAGCACCGGCTAACTGCGTG

>4894 len=108 tot-seq=1

GATCGCTTGAGTCCAAGAGTTTGAGATCAGCCTAAGCAACACAGGGAGACACCATCTCTA

CAAAAATTTTTTTAAAATGGCTGGGCATGGTAGTGTGCATCTATAGTC

>4895 len=108 tot-seq=1

TAGGGAATCTTCCACAATGGACGCAAGTCTGATGGAGCAACGCCGCGTGAGTGAAGAAGG

GTTTCGGCTCGTAAAGCTCTGTTGTTGGTGAAGAAGGACAACTACGTG

>4896 len=108 tot-seq=1

CCTGATGGAGCAACGCCGCGTGCAGGAAGACGTCCTTCGGGTTGTAAACTTCTTTTATAA

GCGAGAAATATGATGGTAACTTATGAATAAGGCCCTGCTAACTACGTG

>4897 len=108 tot-seq=1

CCTGATGCAGCGACGCCGCGTGAGCGAAGAAGGATTTCGGTTTGTAAAGCTCTATCATTA

GGGAAGAAAATGACGGTACCTTACAAAGAAGCGCCGGCTAAATACGTG

>4898 len=108 tot-seq=1

TAGGGAATCTTCCACAATGGACGCAAGTCTGATGGAGCAACGCCGCGTGAGTGAAGAAGG

TTTTCGGATCGTAAAGTTCTGTCGTCGGGGAAGAAGGATAGAGGTAGT

>4899 len=108 tot-seq=1

AGCCCAGGACAAGGTGAAAAGCAAAGAAATGTCTGCAGGACATAGCGTCCCGGGCTTGCC

TAGAACTGCGCCCTCCGCAACGTTTCTAAACCACTCTCCGCCCGTTTC

>4900 len=108 tot-seq=1

TGCAGGATGACGGCCCGATGGGTTGTAAACTGCTTTTATATGGGGATAAAGGGGGGAACG

TATGCCCTTTTGCAGGTACCATATGAATAAGGACCGGCTAATTCCGTG

>4901 len=108 tot-seq=1

TGGAGCTTTCATTTAAATGCCTTGTCTCCTTGTGGCGCAGGAAGGTTGGATTTTCAAGAA

CTTTCCATCTCTGTCCCCCTGTCTGTTACTGGAGAGCAGTATGTAGTG

>4902 len=108 tot-seq=1

TAGGGAATATTCCGCAATGGGGGCAACCCTGACGCAGCGACGCCGCGTGAGGGATGAAGG

GCTTCGGGTTGTAATCCGCTTTTGATTGGGAGCAAGCTTTCGGGTGAG

>4903 len=108 tot-seq=1

CAGCCAAGGGTAGGCACGGATGCCTCAGTCAGCCCTTTGTGTCTTGAAGCACGGGCAACT

GGCTGGGCAACTTTCTCAGGGCTTCCTCTATCAGCAATCTGGGCTGGA

>4904 len=108 tot-seq=1

GAAGAAGGTCTTCGGATTGTAAAGCACTTTAAGTTGGGAGGAAGGGTATTAACCTAATAC

GTTAGTACTTTGACGTTACCGACAGAATAAGCACCGGCTAACTCTGTG

>4905 len=108 tot-seq=1

CCCAGAATCAGATCCCGAATCCCCTTCTCTCTGGTACAGCCACCATCATCTCTTTCCTGG

AGGACAACAGCCTCCTCCCTGGTCTCCATGCTTCCACCCAAGCCCCTG

>4906 len=108 tot-seq=1

CCTGGTAAGAAGAGGAGAGGACACAGCTGCACACTTGGGAAGGAGGCCGCGGGACGACAG

AGGCAGAAGTTGCAGCGATGTCTCTGAACCGAGGGGCACCAAGGATTG

>4907 len=108 tot-seq=1

AGTGGGGTAGGACACACGTGGCTGTGAAGTGGTCTGCTCTTCCTTAGGGTTTCATTGTTC

CCTGGAGAGCTCTGACCCTGTGCAGAAGGGAATCAGAGCTCAATAAGG

>4908 len=108 tot-seq=1

AGCCCTAGGATCCCACCCGACAGGAAGTGCAGAACACACCCAGGGAGATGTCTCAATAGC

TGAGTGCAGATTTCCAAAACTGACAGTGCAACACAGCAGAAGTCACAA

>4909 len=108 tot-seq=1

TGATGTGATTGTCCTCGTGGGCACATACATCTCTGCAAATAGCCCATTCTCTTAACCACT

GTGGTATTGTGTAATGAAGACATAGAAGAGTGAAAAGCTGTGTGCCTT

>4910 len=108 tot-seq=1

TAGGGAATCTTCGGCAATGGGGGCAACCCTGACCGAGCAATGCCGCGTGAGTGAAGAAGG

TTTTCGGATTGTAAAGCTCTGTTGTATGAGAGGAACGTGTGTGAGTGT

>4911 len=108 tot-seq=1

CCTGATGCAGCGACGCCGCGTGAGCGAAGAAGGCTTTCGAGACGTAAAGCTCTGTCCTAT

GGGAAGATAATGACGGTACCATAGGAAGAAGCCCTGGCTAAATACGTG

>4912 len=109 tot-seq=1

TGGGGGATATTGCACAATGGGGGAAACCCTGATGCAGCGACGCCGCGTGTGGGAAGAAGG

TCCTCCGGATTGTAAACCCCTGTCCCCAGGGACGAAGATGAAGGTACCT

>4913 len=109 tot-seq=1

GGCACTAAGCATGTGGACAAGTAGTGTGTGCAGATAATGCACACACTCGCAGACACATTT

AAAGCAACAACTGCCACCTTGTCCCTCCACATCCGGGGGCAATCACCTC

>4914 len=109 tot-seq=1

CCGTAAATGGAAGTCCAAGGATCTAGCAGTTGTTCAGTCCCACAAGGAAAGCAGGCAAAG

GAGAGAAAGAGTGTTAATCTTCCTTCTTCCAAAGTACTCACATAGGTCT

>4915 len=109 tot-seq=1

GCCTGATCCAGCCATGCCGCGTGAGTGATGAAGGCCCTAGGATTGTAAAGCTCTTTCGCC

GATGAAGATAATGACGGTAACCGGAGAAGAAGCCCCGGCTAACTGCGTG

>4916 len=109 tot-seq=1

GAACGCTGGGGCCCAGAGAGCCAGGGAGCCCGTGACAAAGGCCACAGAGGTGAAGCCAAG

GGAAGACAGGACGAAACTAGGACTGCAGACATTGAGAGGAAGTCAGACC

>4917 len=109 tot-seq=1

TGACGTATTTGCAAAGTGGAAATATCAGCAGAGGTGAGAAACCCTTTTAGAAAAGAAATC

TGAGAGGTAAAATATAAGAAATGGAAATGGAAAAACTCCCAAGGAGAAT

>4918 len=109 tot-seq=1

TGGGGGATCTTCCGCAATGGACGAAAGTCTGACGGAGCAACGCCGCGTGAGTGATGACGG

CCTTCGGATTGTAAAGCTCTGTTAATCGGGACGAATGGTCGGAGGGCAA

>4919 len=109 tot-seq=1

TGATCCAGCAATTCTGTGTGCACGATGAAGTTCTTCGGAAGGTAAAGTGCTTTCAGTTGG

GAAGAAGAAAGTGACGGTACCAACAGAGGAAGTGACGGCTAAATACGTG

>4920 len=109 tot-seq=1

CCCTGATCCAGCAATGCCGCGTGAGTGATGAAGGCCTTCGGGTTGTAAAGCTCTTTCGGC

GGGGAAGATGATGACGGTACCCGCAGAAGAAGCCCCGGCTAACTTCGTG

>4921 len=109 tot-seq=1

GAGTGAAGAAGGGTTTCGGCTCGTAAAACTCTGTTGTTGAAGAAGGATAGAGGTAGTAAC

TGGCCTTTATTTGACGGTAATCAACCAGAAAGTCACGGCTAACTACGTG

>4922 len=109 tot-seq=1

TGGGGAATCTTCCGCAATGGACGAAAGTCTGATGGAGCAACGCCGCGTGAGGGATGACGG

TTTTCGGATTGTAAAGCTCTGTTAATCGGGACGAATGGTTTGTGTGCAA

>4923 len=109 tot-seq=1

TAGGGAATCTTCGGCAATGGACGCAAGTCTGATGGAGCAACGCCGCGTGAGTGAAGAAGG

GTTTCGGATCGTAAAGCTCTGTTGTTGGAGAAGAACGTTGGTAGGAGTG

>4924 len=109 tot-seq=1

CCCTGACGCAGCGACGCCGCGTGAGTGGTGAAGTATTTCGGTATGTAAAGCTCTATCAGC

AGGGAAGATAATGACGGTACCTGACTAAGAAGCACCGGCTAAATACGTG

>4925 len=109 tot-seq=1

GCAGGAGGCGGGAGAGGAGATGGCATTGGCATGCAGGTGGACGGACTCCTGTCGCCTGTC

CTGCATGGCCAGCAGCGCACGAGGAAGGAGGAAACAAAAGCCTGAGGCT

>4926 len=109 tot-seq=1

TGAAGAAGGCCTTCGGGTTGTAAAGGTCTTTCAGCGGGGAGGAAGGGAGTAAAGGTAATA

TCTTTGCTCATTGACGTTACCCGCAGAAGAAGCACCGGCTAACTCCGTG

>4927 len=109 tot-seq=1

GCCATTGGGTGGGTCTGTGGGGTCGGCCATTGTGAGCCACTAGCCTGACTTGGTTGTTTC

CTGGTAACCAGGTGACATAGGAGGCTTTGTGGGTACTAGCCATTGGGCA

>4928 len=109 tot-seq=1

CTCTGATGCAGCGACGCCGCGTGAGCGAAGAAGGAATTCGTTTCGTAAAGCTCTGTCCTG

GGGGAAGGTAATGACGGTACCCCAGGAGGAAGCCCCGGCTAACTACGTG

>4929 len=109 tot-seq=1

CTCTGATGCAGCGACGCCGCGTGAACGAAGAAGGTCTTCGGATTGTAAAGTTCTGTCCTG

GGTGAAGATAATGACGGTACCTGCAGAAGAAGCCCCGGCTAACTACGTG

>4930 len=109 tot-seq=1

CTGACGGAGCAACGCCGCGTGAGTGATGAAGGTTTTCGGATCGTAAAACTCTGTTGTTAG

GGAAGAACAAGTGCCATAGTAACTGATGGCACCTTGACGGTACCTAACC

>4931 len=109 tot-seq=1

TGAAGAAGGTTTTCGGCGCGTAAAGCTCTGTTGTTGGTGAAGGAGGACAGGGGTAGTAAC

TGACCTTTGTTTGACGGTAATCAATTAGAAAGGCACGGCTAACGACGTG

>4932 len=109 tot-seq=1

CCCTGATGCAGCGACGCCGCGTGAAGGAAGAAGTATCTCGGTATGTAAACTTCTATCAGC

AGGGAAGATAATGACGGTACCTGACTAAGAAGCACCGGCTAAATACATG

>4933 len=109 tot-seq=1

TGGGGAATCTTCCGCAATGGGGGAAACCCTGATGCAGCGACGCCGCGTGCGGGATGAAGG

CTTTCGGGTCGTAAACCGCTTTTGATTGGGAGCAAGCCTTTTGGGAGAG

>4934 len=109 tot-seq=1

TAGGGAATCTTCCACAATGGACGCAACCCTGACGGAGCAACGCCGCGTGCGTGAAGAAGG

CCTTCGGATCGTAAACCTCTGTTGATGGTGAAGAAGGATAGAGGTAGTA

>4935 len=109 tot-seq=1

GTAGGAGACGCCGGGATTCATCCCAGCTCTGTCACTGCCGTGAACGCTGGCCCCATCTCC

GCGGGCTCTTTCCTCTCTGGAGCTTCTGCTCAGAATACACTGCCAAGGG

>4936 len=109 tot-seq=1

TAGGGAATCTTCGGCAATGGACGGAAGCCTGACCCAGCAACGCCGCGTGAGTGAAGAAGG

CTTTCGGATTGTAAAGCTCTGTTGTTAGAGAAGAACGTTGGTAGAAGTG

>4937 len=109 tot-seq=1

CCCTGATGCAGCGACGCCGCGTGGGGGATGACGGCCTTCGGGTTGTAAACCTCTTTCAGT

AGGGAAGAAGCCTCTTCGGGGGTGACGGTACCTGCAGAAGAAGTACCGG

>4938 len=109 tot-seq=1

CCCTGATGCAGCGACGCCGCGTGAGCGAAGAAGTATTTAGGTATGTAAAGATATATAAGA

AGGGAAGATAATGACGGTACCTGACTAAGAAGCTCCGGCTAAATACGTG

>4939 len=109 tot-seq=1

GGGCGCAAGCCTGATGCAGCAACGCCGCGTGAACGATGAAGGTCTTCGGATCGTAAAGTT

CTGTTGCAGGGGAAGATAATGACGGTACCCAAGGAGGAAGCCACGGCTA

>4940 len=109 tot-seq=1

TAGGGAGTGGGTGCATATCTGGGCATTTGGCCAGGTGCCGGGAGAGGAAAACGGAAACCT

GCTCCTATTCCTCTACAGCTGTTGACGTCAGCCGGTGACATAACCCGGC

>4941 len=109 tot-seq=1

TGAGGAATATTGCTCAATGGACGAGAGTCTGAACCAGCCAAGCCGCGTGAAGGATGACTG

CCCTATGGGTTGTAAACTTCTTTTATACGGGAATAAAGTTAGCCACGTG

>4942 len=109 tot-seq=1

CAGCAGCTTGACATGCTCATCCTTTGATCCCCCTAGTTGTTTGCATAGGTGCTCCTGGTA

GCTGGTGTGATGGGTCGATCCACAGGTTCTCAGACAATGTGCTGGGGTC

>4943 len=109 tot-seq=1

GGCCTTCTGGGCACAGAGCGTGGGCACTAGGGTCCGAACTGTCTCTGGGCCTAGGAGACG

ACATTCCACCCCATCCTGCACAAGATGACAAGCTCCCTGCTTCATGTTG

>4944 len=109 tot-seq=1

CCCTAATCCAGCAACGCCGCGTGAGTGAAGAAGGTTTTCGGATTGTAAACCTCTGTCCTT

GGTGACGATAATGACGGTAGCCAAGGAGGAAGCCACGGCTAACTACGTG

>4945 len=109 tot-seq=1

TAGGGAATCTTCCGCAATGGGCGAAAGCCTGACGCAGCAACGCCGCGTGAGTGAAGAAGG

TCTTCGGATCGTAAAACTCTGTTATTAGGGAAGAACATATGTATGAGTA

>4946 len=109 tot-seq=1

GAAGAAGGTGTTCGGATCGTAAAGTTAGGTTGTAAGAGAAGAACGAGTGTGAGAGTGGAA

AGTTCACAATTTGACGGTATCTTACCAGAAAGGGACGGCTAGCTACGTG

>4947 len=109 tot-seq=1

TGAGGAATATTTCACAATGGGCGAAAGCCTGATGCAGCAATACCACGTGAACGATGAAGG

TCTTCTGATTGTAAAGTTCTTTTATTTAGGAAGAAAAGCGTGCTAGCAA

>4948 len=109 tot-seq=1

GCAAGCCTGATGCAGCCATGCCGCGTGTATGAAGAAGGCCTTAGGGTTGTAAAGTACTTT

CAGCGGGGAGGAAGGTGATAAGGTTAATACCCTTATCAATTGATGTTTT

>4949 len=109 tot-seq=1

AGAAAAGGGAATGCTTCTGCACTGTTAGTGGGAATGTAAATTAGTTCAGTCACTGTGGAA

AGCAGCTTGGAGATTTCTCAAGGAACTAAAAATAGTACTACCATTTGAC

>4950 len=109 tot-seq=1

CCTTAGCATCCTGCTTAACGGTCTTAGCAGCATCTGGTGGAGCAACAACAGCGTTCTTAA

AAGTAACCTCTGGGTGATCCTTCTTGAAGGCTTTCTCCATAGCCTGAAT

>4951 len=109 tot-seq=1

GATCCAAGTCAAAACTTCCTGCTTCTCTGGAAATCTCTGCCCATCCACCCTCTCCCTGCT

GTCTCTTGCTGTCTCTGGTTCCCATCACCCTTTGTCTGAAGGCCTCTCC

>4952 len=109 tot-seq=1

TGAAGAAGGCCTTCGGGTTGTAAAGCTCTTTCGGCCGGAACGAAATCGCACGGGCCAATA

CCTTGTGTGGATGACGGTACCGGAAGAAGAAGCACCGGCTAACTACGTG

>4953 len=109 tot-seq=1

TCATGGAGTTGGCTAGTTACTGAATCCTGGAACTCAGTATCTACCAATAACGAGGGCCTC

ACCTGGTGTAAGAGGCTCAGCCAGTGCCCTTTGTACTTCAGCTTTTCTC

>4954 len=109 tot-seq=1

CCTTCCAGGCAGCAGAACATGCCATGAGTGCAGGGGCTGCAGGGCGGGGACCTGGCAGGT

GGTACAAGTGAGGGGATCCTGGTCTCAGCCTGTGCATAAGCAGCTCCCT

>4955 len=109 tot-seq=1

CCCTGATGCAGCTACGCCGCGTGAGCGTAGAAGGCTTTCGAGTCGTAAAGCTCTGTCCTA

TGAGAAGATAATGACGGTAGCATAGGAAGAAGCCCTGGCTAAATACGTG

>4956 len=109 tot-seq=1

CCCTGACGCAGCAGCGCCGCGTGAAGGAAGAAGGCCTTCGGGTTGTAAACTTCTTTAAGT

GTGGAAGATAATGACGGTACACACAGAATAAGCCACGGCTAACTACGTG

>4957 len=109 tot-seq=1

TGACCCAGCAATTCTGTGTGTGTGAAGAAGGTTTTAGGACTGTAAATCTCTTGTAGTAGG

GAAGAAAAAAATGACGGTACCTACAGAAGAAGCACCGGCTAAATACATG

>4958 len=109 tot-seq=1

GTCTTCACTCCCTCTCCATCTTTCTGCATTCTCTGAGGCCTTTCTGTCCCTGAACATCTA

GGGTTCAACAGCCTAGCCCTTCAGAGGAACCATCGGACAATGATGTCCT

>4959 len=109 tot-seq=1

GAGAAGGGAAAGCCCCGAAGATGCCATTATGCAGAGCCCTGGCCTCCACCTCCCAGCAAG

AGCTGGACCCAGCCTGCAAGCATGGCCGGCATGATTTCTGACGAAGCTC

>4960 len=109 tot-seq=1

TGAGGAATATTGGTCAATGGACGAGAGTCTGAACCAGCCAAGTAGCGTGCAGGATGACGG

CCTTCTGGGTTGTAAACTCCTTTTGTTTGGGGATAATCGACATTACGTG

>4961 len=109 tot-seq=1

CAAGCAGAAGACGGCATACGAGATAGTTGGGTGACTGGAGAAGAACACGTGCTAGGTAAC

TACTAGCGCCTTGACGGTATCTAACCAGAAAGTCACGGCTAACTACGTG

>4962 len=109 tot-seq=1

GAACCAGCCAAGTAGCGTGAAGGATGCAGGCTCTATAGGTCGTAAACTTCTTTTATATAA

GAATAAAGTGCAGTATGTATACTTTTTTGTATGTATTATGTGAATAAGG

>4963 len=109 tot-seq=1

CCCTGATGCAGCAACGCCGCGTGAGTGAAGAAGTATTTCGGTATGTAAAGCTCTATCAGC

AGGAAAGAAAATGACTGTACCTGACGAAGAAGCCCCGGCTAACTACGTG

>4964 len=109 tot-seq=1

CCCTGGTGCAGCGACGCCGCGTGAGCGAAGAAGTATTTCGGTATGTAAAGCTATAGCGGC

AGGGAAGAAAATGACGGTACCTGACTAAGAAGCCCCGGCTAACTACGTG

>4965 len=109 tot-seq=1

GAAGAAGGTTTTCGGATCGTAAAGCGCTGTTGTAAGAGAAGAACGTGGGTGAGAGTGGAA

AGTTCACACAGTGACGGTAACGTACCAGAAAGGGACGGCGAACTACGTG

>4966 len=109 tot-seq=1

TGGGGAATATTGCACAATGGGGGAAACCCTGATGCAGCGACGCCGCGTGAGTGAAGAAGC
ATTTCGGTATGTAAAGAACTATCAGCAGGGCAGAAGAAATGACGGTACC
>4967 len=109 tot-seq=1
CCCTGATGCAGCGACGCCGCGTGAGCGATGAAGTATTTCGGTATGTAAAGCTCTATCAGC
GGGGAAGAGAATGACGGTACCTGACTAAGAAGCCCCGGCTAACTACGTG
>4968 len=110 tot-seq=1
CAGCCCCGAGTGGATCAGAAGCTCAAGAGCTCTTCTCCGATACTTATCCTTCACCTATTG
GGATCTTCAGATCCATAAGAGTCTTTGTATATTTCCTTTTGAGCCAAATG
>4969 len=110 tot-seq=1
AATTTCTCCAAATTAAACACATTGGCTGTAAAAGTCCCATTTACAGCTATGCCTCAACCA
CTGTTTCCAAAGGAGGAACTTGGCATGCTAGGAAGAAATGCAGATCGTGC
>4970 len=110 tot-seq=1
TGGGGAATATTGCACAATGGGCGCAACCCTGATGCAGCGACGCCGCGTGTATGAAGAAGG
CTTTCGGGTTGTAAAGTACTTTCAGCGGGGAGGAAGGGAGTAAAGTTAAT
>4971 len=110 tot-seq=1
GGGTCAGCTTGTTAGGGTTTTATGCATCTTCAGTGAAAAATTACATAAGAGTCTTTATCA
GGGAAGGGCCAGGGTATAACACCATCTTACCTACAAGTTCTCAGCATTTG
>4972 len=110 tot-seq=1
CGTGTGTTTGGAAGATTTGAGCATAGAGATAGTGGAAGACAGGGCAGAGGACACAGGTGC
ATGCATGGAAGAACTGGATCAAGCACAGATACTTTGCCTTAACCGTTAGC
>4973 len=110 tot-seq=1
GTGCGCCACCGTGGGGACTGTGACGGGGCAGGCTCAGTGAGAAAAATGACAGATGGCTAG
ATGGGTTGATCTAGGGATTACTAAATCAGCAAGCTCTCCTTCTCCAGCCC
>4974 len=110 tot-seq=1
CCCTGACGCAGCAACGCCGCGTGAAGGGTGAAGGTTTTCGGATTGTAAACTTCTTTTATT
AAGGGCGAAACTTGACGGTACTTAATGAATAAGCTCCGGCTAACTACGTG
>4975 len=110 tot-seq=1
CGTGAGGGACGACTGCCCTATGGGTTGTAAACCTCTTTTGTTTGGGAATAAAGTGCGCCA
CGTGTGGCGCATTGCATGTACCATTTGAATAAGGACCGGCTAATTCCGTG

>4976 len=110 tot-seq=1

GTGAAGAAGGGTTTCGGCTCGTAAAGCTCTGTTGTTGGGGAAGAAGGGCAGGGGTAGTAA

CGGACCTTTGTTTGACGGTAATCAATTAGAAAGTAACGGCTAACTACGTG

>4977 len=110 tot-seq=1

TGGGGAATATTGCGCAATGGGCGAAACCCTGACGCAGCGACGCCGCGTGCGGGAAGAAGG

CCTTCGGGCTGTAAACCGCTTTTGATTGGGAGCAAGGCTTTTGGGTGAGT

>4978 len=110 tot-seq=1

ATGAAGAAGGCCTTCGGGTTATAAAGTACTTTCAGCGGGGAGGAAGGGAGTAAAGGTAAG

ACCTTTGCTGATTGACGTTACCCGCAGAAGAAGCACCGGCTAACTCCGTG

>4979 len=110 tot-seq=1

GTGAAGAAGGTTTTCGGATCGTAAAGCCCTGTTGTTGGAGAAGAAGGTGAGTGAGAGTAA

CTGGTCACGTTTCGACGGTAACTAACCAGAAAGCCACGGCTAACTACGTG

>4980 len=110 tot-seq=1

TAGGGAATATTGCACAATGGAGGAAACTCTGATGCAGCCACGCCGCGTGTGTGAAGAAGG

CCTTCGGGTTGTAAAGCACTTTTGGTGGGGAGGAAAAAATGAGGTTACAC

>4981 len=110 tot-seq=1

AACGATGAAGGTCTGCTGAGTGTAAAGTTCTTTTATTTAGGAAAAAAAGCGAGCTAGGAA

ATGAGTGCGACTTGAGGGTACTAATTGAATAAGTGACAGCTAACTATGTG

>4982 len=110 tot-seq=1

ATGAAGAAGGCCTTCGGGTTGTAAAGTACTTTCAGCGGGGAAGAAGGGAGTAAAGTTAAA

CCCTTTGCTCATTGACGTTACCCGAAGAAGAAGCACCGGCTAACTCCGTG

>4983 len=110 tot-seq=1

GTGAAGAAGGTTTTCGGATCGTAAAGGTCGGTTGTTGGTGAAGAAGGATAGAGGTAGTAA

CTGGTCTTTATTTGACGGTAATCAACCAGAAAGTCCCGGCTAACTACGTG

>4984 len=110 tot-seq=1

TAGGGAATCTTCCACAATGGACGCAACCCTGACGGAGCAACGCCGCGTGAGTGAAGAAGG

TTTTCGGATCGTAAAGCTCTGTTGTTGGTGAAGAAGGATTGTGGTAGTAA

>4985 len=110 tot-seq=1

ATATGACATTGAACAGATATGTCTGGGGGCTTCAAATCCTCCTTGGGCCTCCACTCTCTT

TGAAATAGATGAATTTGTAAAGATCTATGACTTGCCAGGGGAAGATGACA

>4986 len=110 tot-seq=1

GTGAAGAAGGTCTTCGGACCGTAAAACCCTGTTGTTAGAGAAGAAAGTGAGTGGGAGTAA

CTGTTCACGTTTTGACGGTATCTAACCAGAAAGCCACGGCTAACTACGTG

>4987 len=110 tot-seq=1

TAGGGAATCTTCCACAATGGACGCAAGTCTGATGGAGCAACGCCGCGTGAGTGAAGAAGG

GCTTCGGCTCGTAAAGCTCTGTTATTTGTGAAGAAAGACAGGGGTAGTAA

>4988 len=110 tot-seq=1

GTGAAGAAGGGTTTCGGCTCGTAAAGCTCTGTTGTTAGAGAAGAAGGACAGGGAGAGTAA

CTGATCTTTGTTTGACGGTAATCAATTAGAAAGTCACGGCTAACTACGTG

>4989 len=110 tot-seq=1

TGGAGAATATTGCACAATGGACGCAAGCCTGACGCAGCAACGCCGCGTGAGGGGTGAAGG

TCTTCGGATCGTAAAGCTCTGTTGGTGGTGAGGAAGGATAGAGGTAGTAA

>4990 len=110 tot-seq=1

CTCCTAGAAAGGCAGCCCCTGCTGGAAGCATGCGACACCCTGCGCCGGGGCCTGACTGCC

CACGGCATCAACATCAAGGTGAGTGCCGCACCAAGGTCAGGGACATCACC

>4991 len=110 tot-seq=1

TAGGGAATCTTCGGCAATGGACGGAAGTCTGACCGAGCAACGCCGCGTCAGGGGTAGTAA

CTGACCTTTGTTTGACGGTAATCAATTAGAAAGTCACGGCTAACTACGTG

>4992 len=110 tot-seq=1

TGAGGAATATTGGTCAATGGGCGAGAGCCTGAACCAGCCAAGTCGCGTGCAGGACGACGG

CCCTATGGGTTGTAAACTGCTTTTATGTGGGGATAAAGTCTGCGACGTGT

>4993 len=110 tot-seq=1

CCCTGACGCAGCAACGCCGCGTGAATGAAGAAGCCCTTCGGGGTGAAAAGTTCTGTCAAT

GGGGACGAAACTTGACGGTACCCATAGAGGAAGCACCGGCTAACTCCGTG

>4994 len=110 tot-seq=1

CGTTCAGGAAGACGGCTCTACGAGTCGTAAACTGCTTTTGTACTAGGGTCAGCCTATCTA

CGTGTAGATAGCTGAAAGTATAGTACGAATAAGGATCGGCTAACTCCGTG

>4995 len=110 tot-seq=1

GTGTGTGTGAAGAAGGTTTTAGGACTGTAAACTGCTTTTATGCGGGGATAAAGTCGCCCA

CGTGTGGGTGTTTGCAGGTGCCGCATGAATAAGGACCGGCTAATTCCGTG

>4996 len=110 tot-seq=1

TAGGGAATCTTCGGTAATGGACGGAAGTCTGATCGAGCAACGCCGCGTGAGTGAAGAAGG

ATTTCGGATCGTAAAGCTCTGTTGGTAGAGAAGAACGTTGGTAGGAGTGG

>4997 len=110 tot-seq=1

GGGATAAGGATGGAGAGAGGCTGAGGCCAAACGGTCCAGGTTTGTGGACTGCACATAGCC

ATGGGAGAGAGCGAACTGCGTGGGCACCATGACCCAGGCAGCAATGTAAA

>4998 len=110 tot-seq=1

GTGAAGAAGGGTTTCGGCTCGTAAAGCTCTGTTGTTTGTGAAGAAGGTCAGGGTTAGTAA

CTTACCTTTGTTTGACGGTAACCAATTAGAAAGTCACGGCTAACTACGTG

>4999 len=110 tot-seq=1

GTGAAGAAGGGTTTCGGCTCGTAAAACTCTGTTGTTTGTGAAGAAGAACAGGGGTAGTAA

CTGGTCTTTGTTTGACGGTAACCAATTAGAAAGTCACGGCTAACTACGTG

>5000 len=110 tot-seq=1

CCACGTCCGGGAAGTGAGGAGCGTCTCCGCCCGGCAGTCGCCCCGTCCGAGAAGTGAGGA

GCCCCTCCGCCCGGCAGCCGCCCCGTCCGGGAAGTGAGGAGCGTCTCCGC

>5001 len=110 tot-seq=1

GGTAAAAATTGATGACCAGCATCTAGCACAAGACAACTAGACAATTCCCAATTATCCACA

TAGATAGGAAATGTGCCTGAAGACTGAATCACTTATATCAGCAGTAAAAG

>5002 len=110 tot-seq=1

GTGATGAAGGGTTTCGGCTCGTAAAACACTGTTGTAAGAGAAGAATGACATTGATAGTAA

CTGGTCAATATATGACGGTAACTTACCAGAAAGTAACGGCTAAATACGTG

>5003 len=110 tot-seq=1

TGAAGACGGCCTTCGGGTTGTAAAGCTCTGTGGTTCGAGACGAAAGATGAGCAGACGAAT

AATCTGCATAAGTGACGGTACCGAAAAAGCAAGCCACGGCTAACTACGTG

>5004 len=110 tot-seq=1

CTGATCCAGCAATTCGGTGTGTGTGAAGGAGGTTTTAGGACTGTAAAACACTTATAGTAA

GGAAGAAAAAAATGACGGTACCTACAGAGGAAGCAACGGCTAAATACGTG

>5005 len=110 tot-seq=1

CTGACCCAGCAACGCTGTGTGAGTGATGAAGGTTTTCGGGTTGTAAAACTCTTTGAGCAG

GGAAGAAAAAAGTGACGGTACCTGTAGAACAAGCCACGGCTAAATACGTG

>5006 len=110 tot-seq=1

TGGGGAATCTTCCACAATGGGCGCAAGTCTGATGCAGCGACGCCGCGTGAGGGAAGGAGG

CCTTCGGCTCGTAAACCTCTTTTGTTAGGGAGCAAGGACTTTTGTGTTGA

>5007 len=110 tot-seq=1

GTGAAGAAGGCTTTCGGGTTGTAAAGCACTTTCAGTGGGGAAGAAGGCCTGCGGGTTAAT

AGCCGGCAGGGACGACATCACCCACAGAAGAAGCACCGGCTAACTCCGTG

>5008 len=110 tot-seq=1

TGGGGAATATTGGACAATGGAGGCAACTCTGATCCAGCAATTCTGTGTGTGTGAAGAAGG

TTTTAGGACTGTAAAAGGTACCTACAGAAGAAGCGACGGCTAAATACGTG

>5009 len=110 tot-seq=1

AGCTTGGAAAGAAGGTCTGATACCAGAGGCCTGGGGTGGGTCAGATGCCCTGTGGCCTGT

TCATTTGCTATTATTATTGACATTGCAACCTGTTCCCTTTTCTCTCCCTC

>5010 len=110 tot-seq=1

CCCTGATCCAGCGACGCCGCGTGAGTGAAGAAGTATTTCGGTATCTAAAGCTCTATCAGC

AGGGAAGAAAGCAGACGGTACCTGACTAAGAAGCCCCGGCTAACTACGTG

>5011 len=110 tot-seq=1

AGCCTGATGCAGCGACGCCGCGTGAGCGAAGAAGTATGTCGGTATGTAAAGCTATATTAG

AAGGGAAGAAAATGACGGTACCTTACGAAGAAGCTCCGGCTAAATACGTG

>5012 len=110 tot-seq=1

GTGAAGAAGGGTTTCGGCTCGTAAAGCTCTGTTGTTGGTGAAGAAGGACAGGGGTAGTAA

CTTGCCTTTGTTTGACTGTAATCAATGAGAAAGTCACGGCTAACTACGTG

>5013 len=110 tot-seq=1

CTGATGCAGCGACGCCGCGTGAGCGAAGAAGTAGTTCGGTATGTAAAGCTCTATGAGTAG

GTAAGAAAAAAATGACGGTACCTGACTAAGAAGCCCCGGCTAACTACGTG

>5014 len=110 tot-seq=1

GTGAGGGAAGACGGTCCTATGGATTGTAAACCTCTTTTGTCGGGGAGCAATGGTCGTGAC

GAGTGAAGACTGTGAGAGTACCCGAAGAAAAAGCATCGGCTAACTCCGTG

>5015 len=110 tot-seq=1

GTGAAGAAGGGTTTCGGCTCGTAAAGCTCTGTTGTTGTTGAAGAATGTAAGGGGGAGTAA

CTGACCTTTGTTTGACGGTAATCAATTAGAAAGTCACGGCTAACTACGTG

>5016 len=110 tot-seq=1

TTCTCTGCCTTGCTCCATTGTGGCTTGGGGATGTATGAATGAAGGAGGCCACGCGAAGCT

GCTGACGAAGGGATCTGGGGAGCCCGGGCATCCCACACCCCACAGTTACC

>5017 len=110 tot-seq=1

GTGAAGAAGGGTTTCGGCTCTTAAAGCTCTGTTGGTAGTGAAGAAAGATAGAGGGAGTAA

CTGGCCCTGATTTGACGGTAATTACTTAGAAAGTCACGGCTAACTACGTG

>5018 len=111 tot-seq=1

TGGGGAATATTGCACAATGGGGGAAACCCTGATGCAGCGACGCCGCGTGAGCGAAGAAGT

ATTTCGGTACGTAAAGCTCTATCAGCAGGGAAGAAGATGAAGGGACCTGAC

>5019 len=111 tot-seq=1

GCGTGAGGGATGAAGGTACAGAGTAGCGTAAACCTCTTTTGGCAGGGAACAAAGGCGGGG

ACGCGTCCCCGGATGAGTGTACCTGAAGAAAAAGCATCGGCTAACTCCGTG

>5020 len=111 tot-seq=1

TGGGGAATTTTGGACAATGGGGGCAACCCTGATCCAGCAATGCCGCGTGTGTGAAGAAGG

CTTTCGGGTTGTAAAGCACTTTTGTCCGGAAAGAACACGCCGTGGCTAATA

>5021 len=111 tot-seq=1

AACCCTGATGCAGCGACGCCGCGTGAGCGGTGAAGTATTTCGGTATGTAAAGCTCTATCA

GCAGGGAAGAAAATGACGGTACCTGACTAAGAAGCCCCGGATAACTACGTG

>5022 len=111 tot-seq=1

TGTGAAGAAGGCCTTCGAGTTGTAAAGCACTTTCAGCGGGGAGAAAGGCGTTAAGGTTAA

TAACCTTGGCGATTGACGTTACCCGCAGAAGAAGCACCGGCTAACTCCGTG

>5023 len=111 tot-seq=1

TAGGGAATCTTCGGCAATGGACGGAAGTCTGACCGAGCAACGCCGCGTGAGTGAAGAAGG

TTTTCGGTTCGTAAAGCTCTGTCGTTGGAGAAGAACGTTGGAAGGAGTGGA

>5024 len=111 tot-seq=1

CCGTCAGTATAAGTAAGATCGTTCAATAACGCATAGCCAACACCAATTCCGGCAGATCCG

TTACCAGCTCCGTTACCAGATCCGTTACCGAATCCGCCGGCACCAGCCTCG

>5025 len=111 tot-seq=1

TGGGGAATCTTCCACAATGGACGCAAGCCTGATGCAGCAACGCCGCGTGAGTGAAGAAGG

TCTTCGGATCGTAAACCTCTGTTGTTAGAGAAGAACACGAGTGAGAGTAAC

>5026 len=111 tot-seq=1

TTTTTTTTCAAGCAGAAGACGGCATACGAGATGATCTGGTGACTGAAGTTCAGACGTGTG

CTCTTCCGATCTCCTACGGGAGACAGCAGCATTCTTCCTCATTTGCATATG

>5027 len=111 tot-seq=1

TTTTTTTTTTCAAGCAGAAGACGGCATACGAGATATTGGCGTGACTGGAGTTCGACGTGT

GCTCTTCCGATGGCAGCAGTGGAAACTCTTCCAGGAGCTCCTAGAGCAAGA

>5028 len=111 tot-seq=1

TCCTTGAACAATGTTTTGAAGGCAGAGAAATGGTTCTCTGGATCTGGGCAGCACACAGCT

AACGCTGCTCTACATGTGTAGGTGAGAAAGACTCAGGCCTCGCAGCCTGAA

>5029 len=111 tot-seq=1

TGGGGAATCTTCGACAATGGACGCAACTCTGATGGAGCAACTCCGCGTGTGTGAAGAAGG

TTTTAGGACTGTAAAACACTTTTAGTAGGGAAGAAAGAAATGACGGTACCT

>5030 len=111 tot-seq=1

AGTGAAGAAGGTTTTAGGATAGTAAAGATATGTTGGGGGGGAAGAAGGAGAGAGGGAGGA

AAGGGAATTTATTTGACGGTAATCAACCAGAAAGTCACGGCTAACTACATG

>5031 len=111 tot-seq=1

AGTGAAGAAGGGTTTCGGCTAGTAAAGCTCTGTTGTGTGTGAAGAAGGATAGGGGTAGTA

ACTGACCTTTGTTTGACGGTAATAAATTAGAAAGTCACGGCTAACTACGTG

>5032 len=111 tot-seq=1

AGTGAAGAAGGTGTGCGGATCGTAAAGCTCTGTTGTTGGTGAAGAAGGATAGAGGGAGTA

ACTGGCCTTTAGTTGACGGTAATCAACCAGAAAGTCACGGCTAACTACGTG

>5033 len=111 tot-seq=1

TAGGGAATCTTCCACAATGGGCGCAAGTCTGATGGAGCAACGCCGCGTGAGTGAAGAAGG

TTTTCGGCTCGTAAAGCTCTGTTGTTGGTGAAGAAAGAGAGGGGTAGTAAC

>5034 len=111 tot-seq=1

AGTGAAGAAGGGTTTCGGCTCGTAAAGCTCTTTTGTTTGTGAAGAAGGACAGGTTTAGTA

ACTGACCTTTGTTTGACGGTAATCAATTAGAAAGTCACGGCTAACTACGTG

>5035 len=111 tot-seq=1

TAGGGAATCTTCCACAATGGACGCAAGCCTGATGGAGCAACGCCGCGTGAGTGAAGACGG

GCTTCGACTCGTAAAGCTCTGTTGTTGGTGAAGAAGGACAGGGGTAGTAAC

>5036 len=111 tot-seq=1

TAGGGAATCTTCGGCAATGGACGCAAGTCTGACCGAGCAACGCCGCGTGAGTGATGAAGG

CTTTCGGATCGTAAAGCTCTGTTGTTAGAGAAGAACGTTGGTTGGAGTGGA

>5037 len=111 tot-seq=1

TGGGGAATATTGCACAATGGGCGCAAGCCTGATGCAGCGACGCCGCGTGAGGGATGACGG

CCTTCGGGTTGTAAACCTCTGTTAGCAGGGAAGAAGAGAACTTGACGTGAC

>5038 len=111 tot-seq=1

TAGGGAATCTTCGGCAATGGACGCAAGTCTGACCGAGCAACGCCGCGTGAGTGAAGAAGG

CTTTCGGATCGTAAAGCTCTGTTGTTAGAGAAGAACGATGGGAGGAGTGGA

>5039 len=111 tot-seq=1

TAGGGAATCTTCCACAATGGGCGCAAGCCTGATGCAGCAACGCCGCGTGAGTGATGAAGG

CTTTCGGATCGTAAAGCTCTGTTGATGGTGAAGAGGGCTAGAGGTAGTAAC

>5040 len=111 tot-seq=1

GAGGATCCCTTGAGCTGAAGACTTCGACACCAGCCTGGGCAACATAGTAAGACCCTATGT

GTATAAAATATACAAACAGCTAGCCGGGTATGGTGGCATGCAACTGTAGTC

>5041 len=111 tot-seq=1

TGGGGAATTTTGCACAATGGGGGAAACCCTGACCGAGCAACGCCGCGTGAAGGAAGAAGG

TTTTCGGATTGTAAACTTCTGTTATAAAGGAAGAACGGCGGCTACAGGAAA

>5042 len=111 tot-seq=1

TTTTTTTTTCAAGCAGAAGACGGCATACGAGATCGTACTGTGACTGGAGTTCAGACGTGG

GCTCTTCCGATCTCCTACGGGAGGAAGCAGTCTGAATGGGCAGCCAGTGAG

>5043 len=111 tot-seq=1

GGGGGGGGTGGATGATGGGTGTGGCTGGGCCAGGCCTGCAGGGCCTCACAGGGCAGTTTC

CTCTTCCCTTCCCTTGGGCCCTGGCCGTCTTCTGGCCTGGCATTCTTCCAC

>5044 len=111 tot-seq=1

GAACGAGGAAGGGCGGCGGAGGGTAAAGTTCTTTTATTTAGGAAAAAAAGCGTGCTAGGA

AATGAGCGCGCCTTGATGGTACTAATTGAATAAGTGACAGCTAACTATGTG

>5045 len=111 tot-seq=1

CAGATGAAAAGATAGAAAATATGAAATAAAGGCTAAGAATGTTGTGACTTACATTTACTT

ACAATATGGTTATAAGTAAACTGAGGTGCTAATATCCAAACAATGAGATTC

>5046 len=111 tot-seq=1

GCTCTGAGGGATGTGACAGTGCTGTCATGGAGAGGGGACAGTGGACTAGGATGCCAGAGG

CCTGCAAGACCAGTGCTGGCTGACACCGGGACAGAGCTCACAGCAGCTTCC

>5047 len=111 tot-seq=1

TCTGATCCAGCAATTCTGTGTGTGTGTAGAAGGTTTTAGGACTGTAAAGCGCTTTTAGTA

GGAAAGAAAAAAATGACGGTACCTACAGAAGAAGCAACGGCTAAATACGTG

>5048 len=111 tot-seq=1

TGGGGAATATTGGACAATGGAGGAAACCCTGATGCAGCAATGCCGCGTGTGTGAAGAAGG

CTTTAGGACTGTAAAACACTTTTAGTAGGGAAGAAAAATTTGGCTTTACTT

>5049 len=111 tot-seq=1

AAGCCTGATGCAGCGACGCCTCGTGAGCGAAGAAGTATTTCGGTATGTAAAGCTCTATAA

GAAGGGAAGAAAATGACGGTACCTTTCTAATAAGCTCCGGCTAAATACGTG

>5050 len=111 tot-seq=1

TGCGCTTGTGGGCCAGCTGCAGTTCGGGGTAGGCACGGGCTTGGCAGGCCCTGCACTCGG

AGCAGCCTGCCGGCCCTGCCGCCCCAAGCTGTGAGGGGCTTAGCATCTTCG

>5051 len=111 tot-seq=1

GAGCGATGAAGGTCTTCTGATTGTAAAGTTCTTTTATTTAGGAAAAAAAGCGTGTTAGGA

AAGGAGCGCGCCTTGATGGTACTAATTGAATAAGGGACAGCTAACTATGTG

>5052 len=111 tot-seq=1

CTCTGATACAGCAATTCGGTGTGTGAGAAGAAGGTTTTAGGACGGTAAAACACTTTTAGT

AGGGAAGAAAAAAATGACGGTACCTACAGAAGAAGCAACGGCTAAATACGT

>5053 len=111 tot-seq=1

CACGATGAAGGTCTTCGGACTGTAAAGTGCTGTTATAAGGGAAGAACATTTGCAATAGGA

AATGATTGCAGAATGACAGTACCTTGTCAGAAAGCGAGGGCTAACTATGTG

>5054 len=111 tot-seq=1

GCGCGCAAATTACCCACTCCCGACCCGGGGAGGTAGTGACGAAAAATAACAATACAGGAC

TCTTTCGAGGCCCTGTAATTGGAATGGATCCATTGGAGGGCAAGTCTGGTG

>5055 len=111 tot-seq=1

GTTCCATCTTACAGGCAGACAAGGAAGTGAGGATAACAGTGACTTGTCCAGGTTACACAG

CTCTGTACCGCCCCTGCCAAGCTGATTTGGCAGTTGGAGGCTCACTAGGAT

>5056 len=111 tot-seq=1

GCGTGCAGGATGACGGCCCTATTGGTTGTAAACTGCTTTTGTTGTGGAATAATCGACATT

ACGTGTAATGTTTTTCATCTACCATTCTAATAAGAACCGGCTAATTCCGTG

>5057 len=111 tot-seq=1

CGCGTGAGGGACGACGGTGCTACGGATTGTAAACTTCTTTAGGCGGGGAGTAATGTGCTA

TACGAGTAGAGTAGTGAGAGTACCCGCAGAATAAGCATCGGCTAACTCCGT

>5058 len=111 tot-seq=1

TACTAACCACTGTGCCACCGTTCTGCCAATCCAATCTCCAACTCTGTCCCTATAGTCCTG

CAAGTTTAACTTCCCTCAATCTTTGTTGGGAAACAATGATCTTCTCCACTT

>5059 len=111 tot-seq=1

CACGATGAAGGTCTTCGGATTGGAAAGTGCTGTTATAATGGAAGAACATTTGCAAGAGGA

AAGGATTTCAGACTGACGGTACCTTGTCAGAAAGCGATGGCTAACTATGTG

>5060 len=111 tot-seq=1

ACGCAGAGACGCCGCGTGCGGGATGAAGGCCTTAGGGTTGTAAAGCGCTTTTGATTGGGA

GCAAGCATTCGGGTGAGTGTACCTTTCGAATAAGCGCCGGCTAACTACGTG

>5061 len=112 tot-seq=1

AAGTCAAAGCAGGCGGATGAGCGCCTGTCACAGCAGACTTCTATGAAGAATGCAGAAAAC

TCCATGACTCAAAAGCTAATAGTAAGAAATGGAACAAAGAATATAAAAAGAA

>5062 len=112 tot-seq=1

AGCAGGGCTGTGGCCTGCAGAGAGAGGAGCTGATAAGAAAGACAAGGCCACAGCAAGAGT

CTAAAAAGTGGGTTCTACTTAACTCAGGTACCCCAGCAAGTCTCCTCTGTCC

>5063 len=112 tot-seq=1

TCAAGCAGAAGACGGCATACGAGATCGTGATGTGACTAGAGTTCAGACGTGTGCTCTTCC

GATCTCCTACAGGAGGCAGCAGACGTGTGCTCTTCCGATCTCCTACGGGAGG

>5064 len=112 tot-seq=1

TAGGGAATATTGCACAATGGAGGAAACTCTGATGCAGCCACGCCGCGTGTGTGAAGAAGG

CCTTCGGGTTGTAAAGCTCTTTTGGTGGGGAGGAAAGAAATGGCGTTACCCT

>5065 len=112 tot-seq=1

CGCGTGCAGGATGACGGTCCTATGGATTGTAAACTGCTTTTGTACGGGAAGAAACACTCC

CTCGTGAGGGAGCTTGACGGTACCGTAAGAATAAGGATCGGCTAACTCCGTG

>5066 len=112 tot-seq=1

TAGGGAATCTTCCACAATGGACGAAAGTCTGATGGAGCAACGCCGCGTGAGTGAAGAAGG

TTTTCGGATCGTAAAGCTCTGTTGTTGGTGAAGAAGGGTTTCGGCTCGTAAA

>5067 len=112 tot-seq=1

TAGGGAATATTCCACAGTGGACGCAAGCCTGATGGAGCAACGCCGCGTGAGTGATGAAGG

TTTTCGGATCGTAAAGCTCTGTTGTTGGTGAAGAAGGTTAGAGGTCGTAACT

>5068 len=112 tot-seq=1

TAGGGAATCTTCCACAATGGACGCAAGTCTGATGCAGCAACGCCGCGTGAGTGATGAAGG

CTTTCGGATTGTAAAGCTCTGTTGTTGGTGAAGAAGGCTAGAGGTAGTAACT

>5069 len=112 tot-seq=1

TAGGGAATCTTCCGCAATGGACGAAAGCCTGATGGAGCGACGCCGCGTGAGTGAAGAAGG

TTTTCGGATCGTAAAGCTCTGTTGTTGGTGAAGAAGGATAGGGGAAGTAACT

>5070 len=112 tot-seq=1

TAAGGAATCTTCCACAATGGACGAAAGTCTGATGGAGCAACGCCGCGTGAGTGAAGAAGG

TTTTCGGATCGTAAAGCTCTGTTGTTGGTGAAGAAGGCTAGTGGTAGTATCT

>5071 len=112 tot-seq=1

TAGGGAATATTCCACAATGGACGCAAGCCTGATGGAGCAACGCCGCGTGAGCGATGAAGG

TTTTCGGAACGTAAAGCTCTGTCGTCGGTGAAGAAGGATAGAGGTAGTAACT

>5072 len=112 tot-seq=1

TAGGGAATCTTCCACAATGGACGCAAGTCTGATGGAGCAACGCCGCCTGAGCGAAGAAGG

CTTTCGGATCGTAAAGCTCCGTTGTTGGTGAAGAAGGATAGAGGTAGTAACT

>5073 len=112 tot-seq=1

GAGTGAAGAAGGGTTTCGGCTCGTAAAGCTCTGTTGGTAGTGAAGAAAGACAGAGGTAGT

AGCTGACCTTTGTTTGACGGTAATTAATTAGAAAGTCACGGCTAACTACGGG

>5074 len=112 tot-seq=1

TGAGTGAAGAAGGGTTTCGGCTCGTAAAGCTCTGTTGTTGGTGAAGAAGGACAGGGGTAC

TAACTGACCTTTGTTTGACCTTAATCAATTAGAAATTCACGGCTAACTACGT

>5075 len=112 tot-seq=1

TAGGGAATCTTCCACAATGGACGAAAGTTTGATGGAGCAACGCCGCGTGAGGGAAGAAGG

TTTTCGGATCGTAAAGCTCTGTTGTTGGTGAGGAAGGATAGAGGTAGTAACT

>5076 len=112 tot-seq=1

TGGGGAATCTTCCACAATGGACGCAAGTCTGATGCAGCAACGCCGCGTGAGGGAAGAAGG

CTTTCGGATCGTAAAGCTCTGTTGTTGGTGAGGAAGGATAGAGGTAGTAACT

>5077 len=112 tot-seq=1

TAGGGAATCTTCCACAATGGATGCGACTCTGATGGAGCAACGCCGCGTGAGTGACGAAGG

TTTTCGGATCGTAAAGCTCTGTTGTTGGTGAAGAAGGATAGTGGCAGTAACT

>5078 len=112 tot-seq=1

CTCACTGGAAAGTGGGCTTCTAGGGAGGCTGTCAGTATCTTTGCAGCCACTTAGCTCTGG

TTCTTAGAGTCCTTTAATCGCCGGCTCCTCCATGACATGAGGCCTAATGAGG

>5079 len=112 tot-seq=1

TAGGGAATCTTCCACAATGGACGAAAGCCTGATGGAGCAACGCCGCGTGAGTGAAGAAGG

GTTTCGGCTCGTAAAGCTCTGTTGGCAGTGAAGAAAGATAGAGGAAGTAACT

>5080 len=112 tot-seq=1

GAACCCTGATGCAGCGACGCCGCGTGGGTGAAGGAGCACCTCGGTGCGTAAAGCCCTGTC

GGCAGGGAAGAAAATGACGGTACCTGGAGAAGAAGCCCCGGCTAACTACGTG

>5081 len=112 tot-seq=1

AAACCCTGATGCAGCAACGCCGCGTGAATGATGAAGGTTTTCGGATCGTAAAGCTCTGTC

TGTGGGGAAGATAATGACGGTACCCGAGGAAGAAGCCACGGCTAACTACGTG

>5082 len=112 tot-seq=1

GACAAGGGAGGCAGCGGGAGGAATGCAGTGGCCAGGCAGGTGAGGGACCTCAGGACCAGC

AGCTCAGCCGCATCCTTGCATGCAGTGGCCAGGCAGGTGAGGGACCTCAGGA

>5083 len=112 tot-seq=1

CCTAGCAGGTGCCTTGGGAACTGAATACGCAGCACCCTTCCATTATTCATTCCCAAGCCC

AGGATCCACTAGCCGGGAGCTCAGCCGGGAAAGGGATCCCCAAGCGGGCCAC

>5084 len=112 tot-seq=1

ACACTGGATCTCCTGGCTGGGGAGCAGGGCCCAGAGGGGAGGGGGCCGCGCAGGCCTGCC

CTCACCACCCACACACAGCAGCCTCCCCAGGAGGTCAAGTGCCCTTGGAGAG

>5085 len=112 tot-seq=1

ATTTAAGAGTTTGGGGAGGAATGCTATTAGCCACCACCCTGAAGAATCAAGGACAAATGA

GAATGGACTTGGCAGGGGCTTCAGGGGATTTGGACGAAGCCAGGGGTGGACT

>5086 len=112 tot-seq=1

AAAGCCTGATGCAGCGACGCCGCGTGAGCTAAGAAGTATTTCTGTATGTAAAGCTCTATC

AGAATGTAAGAAAATGACGGTACCTTACTAAGAAGCTCCGGCTAAATACGTG

>5087 len=112 tot-seq=1

GTTTCACTCAAGGTGCGGTCCTGGGTGCGGATAAACAGCCGCAGTTCCACCGGCTCCACA

CTGTCGCTGGTCGGGTACCAATCGAACAGAATGCGATAGCCCTTGATGTTGT

>5088 len=112 tot-seq=1

CAAGTCTGATGGAGCAACGCCGCGTGAGTGATGACGGTCTTCGGATTGTAAAGCTCTGTC

TTTAGGGACGATAATGACGGTACCTAAGGAGGAAGCCACGGCTAACTACGTG

>5089 len=112 tot-seq=1

CTCTGATCCAGCAATTCTGTGTGTGGGAAGAAGGTTTTAGGACGGTAAAACACTTGTAGT

AGGGAAGAAAGAAATGACGGTACCTACAGAAGAAGCGACGGCTAAAGACGTG

>5090 len=112 tot-seq=1

AGCGTGAAGGATGACGGCGCTATGGGTTGTAAACTGCTTTTATATGGGAATAATCGGCAT

TACGTGTAATGTTTTGCATGTACCATACGAATAAGGACCGGCTAATTCCGTG

>5091 len=112 tot-seq=1

GAGTGAAGAAGGTTTGCGGATCGGAAAGCTCTGTTGCTGGTGAAGAAGGATAGAGGTAGT

AACTGGCCGGTATTTGACGGTAATCAACCAGAAAGTCACGGCTAACTACGTG

>5092 len=112 tot-seq=1

GCCTGACCCAGCAACGCCGCGTGAAGTATGAAGGTCTTCGGGTTGTAAACTGCTTTGAGC

AGGGAATAAACAAATGACGGTACCTGAAACACAAGCCACGGCTAACTACGTG

>5093 len=112 tot-seq=1

AAAGCCTGATGCAGCGACGCCGCGTGAGCGAAGAAGTATTTCGATATGTAAAGCTCTATC

AGAAGGGAATGAAATGACGGTACCTTACTAAGAAGCTCCGGCTAAATACGTG

>5094 len=112 tot-seq=1

GACGCAGCGACGCCGCGTGCGGGATGAAGGCCTGCGGGTTGTAGACCGCTTGTGATTGGG

AGCAAGCCGTCGGGTGAGTGTACCTTTCGAATAAGCGCCGGCTAACTACGTG

>5095 len=112 tot-seq=1

CGCGTGAGGGAGGAAGGTACAGAGTATCGTAAACCGCTTTTGTCAGGGAACAAAGACTGG

GACGCGTCCCCGGATGGGTGTACCTGAAGAAAAAGCATCGGCTAACTCCGTG

>5096 len=112 tot-seq=1

CCCTGATGCAGCGACGCCGCGTGAGTGAAGAAGTAATTCGTTACGTAAAGCTCTATCAGA

AGGAAAGAAAAAAATGACGGTACCTGATTAAGAAGCCCCGGCTAACTACGTG

>5097 len=112 tot-seq=1

CGCCAGTCCACCGGGCTCATGCCGGTGACGCGGCGGAATTCCCGGTTGAAGTTGGACTTG

GTCTGGAAACCCGAGGAAAACATGATCGCCGTTACCGATTGCTCCGTCTCTT

>5098 len=112 tot-seq=1

AGTGAAGAATGTTTTCGGATCGTAAAGCTCTGTTGTAAGAGAAGAACGTGTGTGAGAGTT

GAAAGTTCACACAGTGACGGTATCTTACCAGAATGGGACGGCTAACTACGTG

>5099 len=113 tot-seq=1

GACGGGAGGCAGCAGGAGAGAAAAAAGGGGGCCTGGGGAGCGCTGAAAGGGGAGATGCAG

AGAGAGGGGCTCGTAAAAAAAGAAAGGAGCGGGAAGGATCATATTGGTCCTGC

>5100 len=113 tot-seq=1

CAACAGGAGCAGAAGGAACAGGAGCAGCAGGAGGAACAGTGGTAACAACAGCAGCAGCAG

CAACCAGAGCAGCAGCAGGAGCAGCAGCAACAGGAGCAGCAGGTCCAGCAGGT

>5101 len=113 tot-seq=1

TAGCGTGAAGGATGACTGCCCTATGGGTTGTAAACTTCTTTTATACGGGAATAAAGTTAG

CCGCGTGTGGCTTTTTGTATGTACCGTATGAATAAGGATCGGCTAACTCCGTG

>5102 len=113 tot-seq=1

GCAAGCCTGATGCAGCCATGCCGCGTGTAGGAAGAAGTATCTCGGTATGTAAACTTCTAT

CAGCAGGGAAGATAATGACGGTACCTGACTAAGAAGCCCCGGCTAACTACGTG

>5103 len=113 tot-seq=1

GCGTGAGGGATGGAGGCCTTCGGGTTGTGAACCTCTTTCGCCAGTGAAGCAGGCCTGCCT

CGTTTGTGGGTGGGTTGACGGTAGCTGGATAAGAAGCGCCGGCTAACTACGTG

>5104 len=113 tot-seq=1

TGAGTGAAGAAGGGTTTCGGAGCGGAAAGCTCGGTGGGGGGTGAAGAAGGACAGGGGTAG

TAACGGACATTTGTTTGACGGGAATCAATTAGAAAGGCACGGCTAACTACGTG

>5105 len=113 tot-seq=1

TGAGTGAAGAAGGGTTTCGGCTCGTAAAGCTCTGTTGTTGGTGAAGAAGGACAGGGGTAC

TAACTGACCTTTGTTCGACGGTAATCGATTAAAAAGTCACGGCTAACTGCGTG

>5106 len=113 tot-seq=1

GAAGAGGGCCAGTGAGAGAATGTTCCATGAACCTCCCAAGCCTGTCAAGGCAAGGGACAC

ATTTTGGCCATTTGAGCCCCTCTCGTGGGATGGATTCAGAATGAACTGGACTG

>5107 len=113 tot-seq=1

TAGGGAATCTTCCACAATGGACGCAAGTCTGATGGAGCGATACCGCGTGAGTGAAGAAGG

GTTTCGGCTCGTAAAGCTCTGTTGTTGAAGAAGAACGTGCGTGAGAGCAACTG

>5108 len=113 tot-seq=1

TGAGTGAAGAAGGGTTTCGGCTCGTAAAGCTCTGTTGCTGGAGAAGAACGTAACTGAGAG

TAACTGTTCAGTGAGTGACGGTATCCAACCAGAAAGTCACGGCTAACTACGTG

>5109 len=113 tot-seq=1

TAGGGAATATTGCACAATGGAGGAAACTCTGATGCAGCCATGCCGCGTGTGTGAAGAAGG

CCTTCGGGTTGTAAAGCACTTTCGGCGGGGAGGAAAAAATGAGGTTAACCTCA

>5110 len=113 tot-seq=1

GACAGCACAGTGGCCCATCTGCCTCTTGCCTTGCTCTTCACCAGGATGCCTGGTGTGTCC

CTCCATGGCCAGGCTTTACAGAACGCAGTCCCACCTGGAGCAGCCACTCGGAC

>5111 len=113 tot-seq=1

GAGTGAAGAAGGTTTTCGGATCGTAAAGCTCTGTTGTAAGAGAAGAACGAGTGTGAGAGT

GGAAAGTTCACACTGTGACGGTACCGGAAAAGCAAGCCACGGCTAACTACGTG

>5112 len=113 tot-seq=1

TGAGTGAAGAAGGGTTTCGGCTCGTAAAGCTCTGTTGTTAAAGAAGAACGTGGGTAAGAG

TAACTGATTGCACAGTGACGGTAGTTAACCAGAAAGCCACGGCTAACTACGTG

>5113 len=113 tot-seq=1

ATCTCCTACGGGAGCCAGTAGCCGCGGTAATGGATAGATCGGAAGAGCGTCGTGTAGGGA

AAGAGTTGCCAATGTGTAGATCTCGGTGGTCGCCGTATCATTAAAAAAAAAAA

>5114 len=113 tot-seq=1

TGAGTGAAGAAGGGTTTCGGCTCGTAAAGCGCTGTTGTTGGTGAAGAAGGACAGGGGTAT

TAACTGACCTTTGTTTGACGGTAAGCAATTAGAAAGTCACGGCTAACTACGGG

>5115 len=113 tot-seq=1

TGAGTGAAGGAGGTTTTCGGCTCGTAAAGCTCTGTTGCTGGTGAAGAAGGATAGAGGTAG

TAACTGGCCTTTATTTGACGGTAATTATCCAGAAAGTCACGGCTAACTACGTG

>5116 len=113 tot-seq=1

TAGGGAATCTTCCACAATGGACGCAAGCCTGATGGAGCAACGCCGCGTGAGGGAAGAAGG

GCTTCGGCTCGTAAAACTCTGTTGTTGGGGAAGAAGGACAGGGGTAGGGACTG

>5117 len=113 tot-seq=1

CAGGAGGATCACTTGAGGTCAGGAGTTCGAAACCAGCCTGGGCAACATGGCAAAACTCTG

TCTCTACTAAAAATACAAAAATTAGCTGGGCACGGTGGTACATGCCTGTAGTC

>5118 len=113 tot-seq=1

TGAGTGAAGAAGGGTTTCGGCTCGTAAAGCTCTGTTGTTGGCGAAGAAGGAAAGGGGTAG

TAAATGAACTTTGTTTGAGGGTAATCAATTAGAAAGTCACGGCTAACTACGTG

>5119 len=113 tot-seq=1

TGAGTGAAGAAGGTTTTCGGAGCTTAAATCTCTGTTGTTGGTGAAGAAGGATAGAGGTAG

TAACTGGCCTTTATTTGACGGTAATCAACCAGAAAGTCGCGGCTAACTACGTG

>5120 len=113 tot-seq=1

TAGGGAATCTTCCACAATGGACGCAAGTCTGATGGAGCAACGCCGCGTGAGTGAAGAAGG

GTTTCGGCTCGTAAAGCTCTGTTGTTGGTGAAGAAGGACCGGCTAATTCCGTG

>5121 len=113 tot-seq=1

GGAGGATGAAGGCCTTCGGGTCGTAAACTCCTGTTGATCGGGAAGAATGGGTTCTGGGTG

AATAGCCCGGAGCAATGACGGTACCGATTGAGGAAGCCACGGCTAACTCTGTG

>5122 len=113 tot-seq=1

TGAGTGAAGAAGGGTTTCGGATCGTAAAGCTCTGTTGTTGGTGAAGAAGGATAGAGGTAT

TACCTGGCCTTTATTTGACGGTAATCACCCAGAAAGTCACGGCTAACTACGTG

>5123 len=113 tot-seq=1

TGAGTAAAGAAGGGTTTCGGCTCGTAAAGCTCTGTTGGTAGTGAAGAAATATAGGGGTAG

TAACTGGCATTTATTTGACGGTAATTACTTAGAAAGTCACGGCTAACTACGTG

>5124 len=113 tot-seq=1

TAGGGAATTTTCGGCAATGGGGGAAACCCTGATGCAGCGACGCCGCGTGGAGGAAGAAGG

CCTTAGGATTGTAAACTCCTATTGTTGAGGAAGATAATGACGGTACTCAACAA

>5125 len=113 tot-seq=1

CAATGATTTTCGAGCCTTCCTGCTGTGCGAAGCATCCGCCCACTGCAATCATCATTCCGG

GGCGGTTACGTTTCACATCTGCCAGCTGTCCCAAATGACCATAGAGTTTCGTA

>5126 len=113 tot-seq=1

GACAAAAGCAAGTGGGACTGCAGAGGGAAGGAGGATAAGCAGGGGAAAATCCCTGCAGGG

AAGCAGCAGCTCCAACAGCAGGGAGCTTGCAAGTCGCACCTTAGAACTGCTCT

>5127 len=113 tot-seq=1

AGTCCAGCCCCTGGGACCTCTGGAACCTGTTTCTCAGGTTCTATTCACAGTCATCTTTGG

AAGGTATGGACAGTGAGAAACTGCAGGGCAAGGGACCTTCTCATCCCTGATCC

>5128 len=113 tot-seq=1

ACAAACAGACAGGGACCCCCGCCCTTGAGGCACTGACCTTCTCACAGGACCCAAGTGAGG

CAGAAAACCATGAGCCATGACCAGGAAGACGTAAGATGGTGCCCGTACCGCCA

>5129 len=113 tot-seq=1

GGGAAACCCTGATGCAGCGACGCCGCGTGAACGAAGAAGGTATTCGTATCGTAAAGTTCT

GTCCTATGGGAAGATAATGACAGTACCATACGAATAAGGACCGGCTAATTACG

>5130 len=113 tot-seq=1

TTCAAGCAGAAGACGGCATACGAGATAACACTGTGACTGGAGTTCAGACTGTGCTCTTCC

GATCTCCTACGGGAGGCAGCGGGCAGATTGCATTTTGAGGCTTCCAGACTTGC

>5131 len=113 tot-seq=1

TGACAGTAGTGGGATGAGTGGGTCCTCAGGTCTCTGGGTGGTGTGCATGGCGTTCGTGAT

GGCAGTAGTGGTTGTGGGCCAATCCTCGGGCCCCCAAGTAGCAAACATGGGTG

>5132 len=113 tot-seq=1

AAGATGATTTGATACTCGATGACTAATTACTGGACTCCTAGGATCTCCCTGTCAAAGGCC

ATAATTGGCACAGAAATGGGAGATAAGATTGGACAGGCAGGTGTTAAGTGGTC

>5133 len=113 tot-seq=1

GGGAGGAAGAAGGCCGGCGGGGGGGAAAGGACTTGCAGCGGGGAGGAAGGGAGTAAAGTT

AATACCTTTACTCATTGACGTTACCCGCAGAAGAAGCACCGGCTAACTCCGTG

>5134 len=113 tot-seq=1

CCCGGGGAGGTAGGGACGAAAAAGAACAAGACAGGCCTCTTTCGAGGCCCTGTAAGTGGA

ATGAGTCCACTTTAAATCCTTTAACGATGATCCATTGGAGGGCAAGTCTGGTG

>5135 len=113 tot-seq=1

TGAGTGAAGAAGGTTTTCGAATCGTAAGGCTCTGTTGTTGGTGAAGAAGGATAGAGGTAG

TAACTGGCCTTTATTAGACGGTAATCAACCAGAAATTCACGGCAAACTACGTG

>5136 len=113 tot-seq=1

TGAGTGAAGAGGGTTTTCTGATCGTAAAGCGCTGTTGTTGGTGAAGAAGGATAGAGGTAG

TAACTGGCCTTTATGTGACGGTAATCAACCAGAAAGTCACGGCTAACTACGTG

>5137 len=113 tot-seq=1

CAGGCAGATTGCTTGAGCCCAGGAGTTTGAGACCAGCCTGGGCAATATAGCAAAACCCTG

TCTCTACAAAAAATACAAAAATTAGCTGGGTGTGGTGGCATGCGCCTGTGGTC

>5138 len=113 tot-seq=1

GAAACCCGGACGCAGCAACGCCGCGTGCGGGATGAAGGCCTTCGGGTTGTAAACCGCTGT

AAGCAGGGAAGACAGCGACGGTACCTGCAGAAGAAGCCCCGGCCAACTACGTG

>5139 len=113 tot-seq=1

GCAAGCCTGATGCAGCGACGCCGCGTGAGCGAAGAAGTATTTCGGTATGTAAAGCTATAT

CAGCAGGGAAGAAAATGACGGTACCTGACTAAGAGGCTCCGGCTAAATACGTG

>5140 len=113 tot-seq=1

ACCCTGACCCAGCAACGCCGCGTGAAGGAAGAAGGCTTGCGGTTTGTAAACGTCTTTTAC

CAGGGACGAAGAAAGTGACGGTACCTGGAGAAAAAGCCACGGCTAACTACGTG

>5141 len=113 tot-seq=1

GAGGGCAGGTGCAATGCTGGGGAAACTGCAGGATTCAGGAGCCAGTGGAGCTCCTGACAG

CCTCGATGAGGCCGCAGGCCAGAATCCACACTGATCCCCTTCGGCTCAGTGGT

>5142 len=113 tot-seq=1

CCGCGTGAGGGAGGAAGGTACAGAGTATCGTAATCCTCTTTTGTCAGGGAACAAAGACTG

GGACGCGTCCCCGGATGAGGGTACCTGAAGAAAAAGCATCGGCTAACTCCGTG

>5143 len=113 tot-seq=1

GAGTGAAGAAGGTTTTCGGATCGTAAAACTCTGTTGTAGGAGAAGAACGTGTGTGAGAGT

GGAAAGTTCACACAATGACGGTAACCAACCAGAAAGGGACGGCTAACTACGTG

>5144 len=113 tot-seq=1

GACGGCATACGAGATTCACGAGTAACTGGAGTTCAGACGTGTGCTCTTCCGATCTACTAC

GGGAGGCAGCAGCAGCAGAGGCAGCAGAGGCAGCAGCAGAGGCAGCACCAGCA

>5145 len=114 tot-seq=1

GTCCATCCATCCATCCATCCATCCATCCCTCCATCCATCATCATGTCCCCCCCCCCCACC

TGGCCTTAAAGATTCATAAGATCCTCCACTGTGCCACCTGTCTCTAGTTTGTAG

>5146 len=114 tot-seq=1

TGAGTGAAGAAGGGTTTCGGATCGTAAAGCTCTGTTGGAAGAGAAGAACGAGTGGGAGAG

TGGAAAGTGCACACTGTGACGGGATCTTACCAGAAAGGGACGGCTAACTACGTG

>5147 len=114 tot-seq=1

GTGAGTGAAGAAGGGTTTCGGCTCGGAAAGCTCGGTGGTGGGTGAAGAAGGACAGGGGTA

GTAAATGACATTTGTTTGACGGGAATCAATTAGAAAGTCACGGCTAACTACGTG

>5148 len=114 tot-seq=1

CGCAAGTCTGATGGAGCAACGCCGCGTGAGTGAAGAAGGGTTTCGGCTCGTAAAGTTCTT

TTATATGGGAAGATAATGACGGTACCATAAGAAAAAGCCCCGGCTAACTACGTG

>5149 len=114 tot-seq=1

GTGAGTGATGAAGGTCTGCGGATCGTAAAACTCTGTTATTAGGGAAGAACAAATGCGTAA

GTAACTGTGCGCGTCTTGACGGTACCTAATCAGAAAGCCACGGCTAACTACGTG

>5150 len=114 tot-seq=1

TGGGGAATATTGCGCAATGGGCGCAACTCTGATGCAGCGACGCCGCGTGAGGGATGAAGG

CCTTCGGGTCGTAAACCGCTTTTGATTGGGAGCAAGCTTTCGGGTGAGTGTACC

>5151 len=114 tot-seq=1

TAGGGAATATTGCACAATGGAGGCAACTCTGATGCAGCAATGCCGCGTGTGTGAAGAAGG

CCTTCGGGTTGTAAAGCACTTTCGGAGGGGAGGAAAAAAATGGGGTTACCCTCA

>5152 len=114 tot-seq=1

GTGAGTGAAGAAGGGTTTCGGCTCGTAAAGCTCTGTTGTTGGTGAAAAGGGACAGGGGGA

GTAACTGACCTTTGTTTGACGGTAACCAATTAGAAAGTCACGGCTAACTACGTG

>5153 len=114 tot-seq=1

TGAGGAATATTGGTCAATGGGCGTTAGCCTGAACCAGCCAAGTAGCGTGAAGGATGAAGG

CTCTATGGGTCGTAAACTTCTTTTATATAAGAATAAGGACCGGCTAATTCCGTG

>5154 len=114 tot-seq=1

GCCGCGCGCAGGATGACGGTCCTATGGATTGTAAACTGCTTTTGTACAGGAAGAAACACT

GGTTCGTGAACCAGCTTGACGGTACTGTAAGAATAAGGATCGGCTAACTCCGTG

>5155 len=114 tot-seq=1

GTGAGTGAAGAAGGGTTTCGGCTCGTAAAGCTCTGTTGTTGGTGAAGAAAGACAGGGGTA

GTAACGGACCTTTGTTTGACGGTAATCGATTAGAAAGGCACGGCTAACGACGTG

>5156 len=114 tot-seq=1

TAGGGAATCTTCCACAATGGACGCAAGTCTGATGGAGCAACGCCGCGTGAGTGAAGAAGG

GTTTCGGCTCGTAAAACTCTGTTGTTGGTGAAGAACGACATGGGGAGTAACTGA

>5157 len=114 tot-seq=1

GTGAGTGAAGAAGGTTTTCGGATCGTAAAGCTCTGTGGGTGGTGAAGAAGGATAGAGGTA

GTAACTGGCCTTTATTGGACGGTAATCAACCAGAAAGTCAGGGCTAACGACGTG

>5158 len=114 tot-seq=1

CCCGCGTGAAGGAATAAGGACCTATGGTTTGTAAACTTCTTTTCTACAGGGATAAACCCC

GGGACGTGTCCCGGGCTGAAGGTACTGTAGGAATAAGCACCGGCTAACTCCGTG

>5159 len=114 tot-seq=1

GTGAGTGAAGAAGGGTTTCGGAGCGTAAAGCTCTGTTGTTGGTGAAGAAGGATAGAGGCA

GTAACTGACCTTTGTTTGACGGTAATCAACCAGAAAGTCACGGCTAACTACGTG

>5160 len=114 tot-seq=1

TAGGGAATCTTCCGCAATGGACGGAAGTCTGACGGAGCAACGCCGCGTGAGTGAAGAAGG

TTTTCGGATCGTAAAGCTCTGTTGCTAGAGAAGAACGTTGGTGGGAGTGGATAA

>5161 len=114 tot-seq=1

GTGAGTGAAGAAGGGTTTCGGCTCGTAAAGCTCTGTTGTTGGTGAAGAAGGACAGGGGTG

GTGACTGACCGGTAAATGACGGTAATCAATTAGAAAGTCACGGCTAACTACGTG

>5162 len=114 tot-seq=1

TGACGCAGCGACGCCGCGTGCGGGAGGAAGGCCGTCGGGGTGTAAACCGCTTTTGATTGG

GAGCAAGCCTGGGGGGGGAGTGTACCGTTCGAATAAGCGCCGGCTAACTACGTG

>5163 len=114 tot-seq=1

GTGAGTGAAGAAGGTTTTCGGATCGGAAAGCTCTGTTGTTGGTGAAGAAGGATAGAGGTA

GGAAATGGCATTTATTTGACGGTAAGAAACAAGAAAGTCACGGCTAACTACGTG

>5164 len=114 tot-seq=1

CGCAAGCCTGATGCAGCAACGCCGCGTGAACGATGAAGGTCTTCGGATCGTAAAGTTCTG

TTGCAGGGGAAGATAATGACGGTACCCTGTGAGGAAGAAAAGGATAAATACGTG

>5165 len=114 tot-seq=1

TGGGGAATATTCGACAATGGAGGGAACTCTGATCCAGCAATTCCGCGTGTGTGAAGAAGG

TTTTAGGATTGTAAAACTCTTTTAGTAGGGAAGAAAAAAATGATGGTTCCTAAA

>5166 len=114 tot-seq=1

TGAGGTATGATCATGCTACTGCACTCCACCTTGGGTGACAGAGAAAGACCTTGTCTCTTA

AAACAAACACACAAAAATAATAAAGTTATTTCTCTGCTTGATAGACCAGACTCC

>5167 len=114 tot-seq=1

TAACAAATGTAGGGGCCTCAATTGGAAAGGAAGGACTAGGGCCTTTAGAATAATGGTCAC

TGTCAGGACAATGAGGAGTATCTAGCCCAGAGACTAGCTATAGGTACACCCCAA

>5168 len=114 tot-seq=1

CCGCCATAAAATCCACTCTTTAGCGTGTGCGACAGAGTGGTCTTGAGTAGGGTCACAAGC

GTCACCGCTAGTTCCAGAGCCTTTGCATCACCACGGAAAGAAACCCTGGACCCT

>5169 len=114 tot-seq=1

GGAAACCCTGATGCAGCAACGCCGCGTGAGTGATGAAGGATTTCGGATCGTAAAGCTCTA

TCTTTAGGGAAGATAATGACGGTACCCGAGGAAGAAGCCACGGCTAACTACGTG

>5170 len=114 tot-seq=1

TGACGCAGCGACGCCGCGTGCGGGATGAAGGACTTCGGGTTGAAAACCGCTATTGATTGG

GAGCAAGCCTTTTGGGTGAGTGTACCTTGCGAATAAGCGCCGGCTAACTACGTG

>5171 len=114 tot-seq=1

CGTGGACGATGAAGGTCTTCTGATTGTAAAGTTCTTTTATTTAGGAAAAAAAGCGTGCTA

GGAAATGAGCGCGCCTTGAGGGTACTAAGTGAATAAGGGACAGCTAACTAGGTG

>5172 len=114 tot-seq=1

GGGACAAATGAGAAGGAGAGCAAGAGGGAGCACATCAATGAGAAACAATGATGATGAGAT

AATTAGAGTGAGCAATAAAGAATTCATCACAGGGACAGAGGACCAGACCAATGA

>5173 len=114 tot-seq=1

AACTCTGATCCAGCAATTCGGTGTTTGTGAAGAAGGTTTTAGGACGGTAAAACTCTTTTA

GTAGGGAAGAAAGAAATGACGGTACCTACAGAAGAAGCGACGGCTAAATACGTG

>5174 len=114 tot-seq=1

TGGGGAATATTGCACAATGGGCGCCAGCCTGATGCAGCGACGCCGCGTGAGGGATGGAGG

CCTTCGGGTTGTACACCTCTTTTATCGGGGAGCCCGCGCGAGTGAGTTTACCCG

>5175 len=114 tot-seq=1

GGGTAGGAACATCTATATAACCCCCTTCCCTTTCAGCACCGTCCCTGGGGTTCTCAGCTG

GCGCCCCCTCCAGCCCCCTGGCAGTGTCAGTCAGGGGTGCTGGCTGTGCCCACT

>5176 len=114 tot-seq=1

CCAAGGGAGGCTCATGTGGGGCCAGCCTCTGGGATGGGGGAGGATGGGCAAGATGTCCTA

GGCTGTGCCTTTCTCAGTGGCTTTTACATCTGATTTCCCTGCTTCACAGAGAGG

>5177 len=114 tot-seq=1

GAAGGGTAAGTGCATAGAGTGAGAAGAGAGAGAGGTTGAGAAGTGGATAGTGGGAAAAGG

GCATTTGAGGTTGGACAAAGGAAGAGGATGGAGAGAGAGAAAGAGAGTGGGGAA

>5178 len=114 tot-seq=1

GCCGCGTGCAGGAAGACGGCTCTATGAGTTGTAAACTGCTTTTGTACGAGGGTAAACTCA

CATACGTGTAGGTGACTGGAAGTATCGTACGAATAAGGACCGGCTAACTCCGTG

>5179 len=114 tot-seq=1

TTGTCCAAACCCTTGTAAGTAAAAATGTCTGCAAGATATAATCCCTGTGTTTTCATCAGA

GGCTTCTAGGGCTCCTGGTGTGAATAAGAATCCTCCACGCAGTTTGTTATTCAC

>5180 len=114 tot-seq=1

GGTAGGGGTGGTAGGGGTGGAGGATAGGCAGGGGTGAGGTAGGGAAGAACATCAGCCAAC

GGCCTCTCTTCACAGGACTGCAGGAAGGAGTCCGGGACGTGGAGTGAGGAAGGG

>5181 len=114 tot-seq=1

GGTCAAAAAACTTAAGAGATTTTAATACATTTCCTGTAAGTCAACCCTGTGATAACAAGA

AGATGGCCTTCAAGAATAGGCCTCAGCCAAAGGAGTATTGGTTGGCAATGACAG

>5182 len=114 tot-seq=1

AAGTCTGAGCCAGCAATTCTGTGTGCACGATGACGTGTTTCGGAATGTAAAGTGCTTTCA

GTTGGGAAGAAAAAAATGACGGTACCAACAGAAGAAGGGACGGCTAAATACGTG

>5183 len=115 tot-seq=1

GCAGTGTGGGCAGAGGCCCCCATCCCTGGAGTCAGGCAGACACACCTGCCTTCCTTCCAG

GGCGAACCTCAGTACTCCAGCCATTCCAGCAGCAACACCCTCGCCAGCAACGCCT

>5184 len=115 tot-seq=1

AGGTTAATTCTGGGCATTTTGTATTCCTCTCTCACTTCTCGGCTCTTTGAATCAGAGAGA

CTATAATGTTAGACTACTCAGCCTTTGCTCCAAGAAAACTGCTTTTAAACTACTT

>5185 len=115 tot-seq=1

TTTTTTTTCAAGCAGAAGACGGCATACGAGATTGTGCCGTGACGGAGTTCAGACGTGTGC

TCTTCCGATCTCCTACGGGTGGCAGCAGTGGTAGATGGAAAGTAAGTGATCTGGG

>5186 len=115 tot-seq=1

AAACCCTGATCCAGCGACACCGCGTGAGTGAAGAAGTATTTCGGTATGTAAAGCCCTATC

AGCAGGGAAGAAAGAAATGACGGTACCTGAGTAAGAAGCCCCGGCTAACTACGTG

>5187 len=115 tot-seq=1

GTGAGTGAAGAAGGTTTTCGGAGCGGAAAGCTCTGTTGTTAAGGAAGAACGAGTGGGAGA

ATGGAAAGTTCATGCTGTGAAGGGACTTAACCAGAAAGGGACGGCTAACTACGTG

>5188 len=115 tot-seq=1

TAGGGAATCTTCCACAATGGACGAAAGCCTGATGGAGCAACGCCGCGTGAGTGAAGAAGG

CTTTCGGATCGTAAAGCTCTGTTGTTGGGGAAGAAGGTTACAGGTAGTAACTGGC

>5189 len=115 tot-seq=1

TGCCGCGTGAAGGACTAAGGCCCTATGGGCTGTAAACTTCTTTAGACGCAGAGCAATAAG

GGCCACGCGTGGTCCGATGAGAGTATGCGTAGAATAAGCATCGGCTAACTCCGTG

>5190 len=115 tot-seq=1

AGAAGACGGCATACGAGATGACTGAGTGACTGGAGTTAGACGTGTGCTCTTCCGATCTCC

TACGGGAGGCAGCACGGTAGTGCATTTGTGGGCGGCTGGATTGGGGCCAGGGGAG

>5191 len=115 tot-seq=1

ATGAGTTTCATTACACAACTGAGGAGAATGAGGCTCAGGAAATGGAATTAACTTGCTAGG

GGCCCTGCAGCTGGGATGAAGCCTTATTGCCATCTATTAGTCTGAGAAGGATCTC

>5192 len=115 tot-seq=1

CGTGAGTGAAGAAGGGTTTAGGATGGTAAAGATATGTTGGTAGTGAAGAAAGAGAGAGGT

AGTAAATGGAATTTATTTGACGGTAATTACTTAGAAAGTCACGGCTAACTACATG

>5193 len=115 tot-seq=1

CGAAAGAACAGTCGCAGAGGGAAAGCGGGAAAGAGATGGGGGAAAGTGTGTGTGTGTGAG

TGTGTGCTTGTGTGCATGTGTGTGCGTGTGTGTGTCAAGGAAAAAAGCTCGCAGT

>5194 len=115 tot-seq=1

CTGACGCAGCGACGCCGCGTGCGGGATGAAGGCCTTCGGGTTGTAAACCGCTATTGATTG

GGAGCAGGCCTTTTGGGTGAGTGTACCTTTCGAGCAAGCGCCGGCTAACTACGTG

>5195 len=115 tot-seq=1

GGCTCCCCGGGCAGTGCCTGGGCCTAGGAGGGCTCCCCTGGCCAGGTGCATCCAGGCAGA

GACTTGAACAGTGGAGAGGAGAGTTACGGCGTGGAGAGCAAGGAGCAGGGAGGGG

>5196 len=115 tot-seq=1

CGTGAGTGAAGAAGGTTTTCGGATCGTAAATCTCTATTGTAAGAAAAGAACAAATTGTAG

ATTAACTGCTACAGTCTTGACGGTAGCTTACCAGAAAGCCACGGCTAACTACGTG

>5197 len=115 tot-seq=1

CGGGAGCGCGTGGCCAAAGCTGTCAGCAGAGGCACAGGTGGTGGCAGGCAGTAGAGGCGC

CCCATGGGGAACATACTGACCTGTCGTGTGCGCCCTAGCGTCAGCCTCGAGTTTG

>5198 len=115 tot-seq=1

TGGGGAATTTTACGCAATGGGGGAAACCCTGACGTAGCGACACCGCGTGAGCGAAGAAGC

CCTTTGGGGTGTAAAGCTCTGTCAGCAGGAACGAACAAAATGACGGTACCTGCAG

>5199 len=115 tot-seq=1

AGTAGCGTGCAGGATGACGGCCCTATGGGTTGTAAACTGCTTTTATATGGGAATAAAGTG

AGGGGCGTGTACCTTAATGCATGTACCACATGAATAAGGACCGGCTAATTCCGTG

>5200 len=115 tot-seq=1

CCTAGACTTTTTAATAATGGCATTTGAAAAAGTACAAGTCACAAAAGCAACACTCGATGA

TAGTAAAGAACAGAAACGCCGAATATGTAGGTGGAGCAAGTGGCGTCCCATCCCG

>5201 len=115 tot-seq=1

GAGTCTGAACCAGCCAAGTAGCGTGCAGGATGACGGCACTATGGGTTGTAAACTGCTTTT

GTTTGGGAATAATCGACATTACGTGTAATGTTTAGCATGTACCGTGCGAATAAGG

>5202 len=115 tot-seq=1

CGTGAGTGAAGAAGGTTTTCGGATCGTAAAGCTCTGTTGTTGTTGAAGAAGGATAGAGTT

AGTAACTGGCCTTTATTTGACTGTAATCATCCAGAAAGTCACGGCTAACTACGTG

>5203 len=115 tot-seq=1

TAGGGAATTTTCGGCAATGGGGGCAACCCTGACCGAGCAACGCCGCGTGAAGGAGGAAGG

TCTTCGGACTGTAAACTTCTGTTATAATGGAAGAAAGACGGATAAAGGGAATGAT

>5204 len=115 tot-seq=1

CCCATTTGCCAGAACTCAGCACCAAGGTCAGGTGCTGCCAGAGGCTCAAGAATGTGGTCT

CTAGCTGAGTAGCCCTAGGCCCACCTAAAAGGTGGCTGAATGGTCTTCGGGCCAG

>5205 len=115 tot-seq=1

ATCTCTACATTGCTGATGAGGGGGAGAAAGGAACACAAAGCACAGATTATTTTTGGGAAT

GTGACTGGAGTTATAACTACACATGAAAAGGCAAATAATGAGTTAATGAGAAAAA

>5206 len=115 tot-seq=1

CGTGAGTGAAGAAGGGTTTCGGCTCGTAAAGCTCTGGTGGTAGTGAAGAAAGATAGAGGT

AGTAACGGGCCTTTATTTGACGGTAACTACTTAGAAAGGCACGGCTAACTACGTG

>5207 len=115 tot-seq=1

CGTGAGTGAAGAAGGGTTTCGGCTCGGAAAGCTCCGTTGGTAGTGAAGAAAGATAGAGGG

AGTAACGGGCCTTTATTTGACGGTAATTACTTAGAAAGGCACGGCTAACTACGTG

>5208 len=116 tot-seq=1

AAGTCGCGTGAGGGATGACGGCCCTACGGGTTGTAAACCCCGTTTGTAAGGGAGCGAGGG

GCGCCACGTGTGGCGCTGTGAGAGTACCTGAAGAAAAAGCATCGGCTAACTCCGTG

>5209 len=116 tot-seq=1

GGAGACCTGCTTGCAAGCAGATCAACCATAGCCAGACAGGTCCTGCTCCATGGCTCTGAC

AGTGCCCAGGGAGCCCATGTAATTACTGCCGGAACTGACATCTGAGGCTGTCACCC

>5210 len=116 tot-seq=1

TGTTTTTAGTTGCAAACTGTGGGTGTTACCTTTAGAATGGAGCTTGCCTTGAGAGCCACA

CAACTAGAGTAAGGGAATTTGAGTGGTAGCCCGGTGTGGTGGTTATACCTGTAATT

>5211 len=116 tot-seq=1

AAGTAGCGTGAAGGATGACTGCCCTATGGGTTGTAGACTTCTTTTATATGGGAATAAAGT

GGTGCACGTGTGGATTTTTGTATGTACCATATGAATAAGGGGCGGCTAACTCCGTG

>5212 len=116 tot-seq=1

GGGGAAACCCTGATGCAGCAACGCCGCGTGAGTGAAGAAGTATCTCGGTATGTAAAGCTC

TATCAGCAGGGAGGAAAATGACGGTCCCTGACTAAGAAGCCCCGGCTAACTACGTG

>5213 len=116 tot-seq=1

CGTGAGTGAAGAAGGGTTTCGGCTCGTAAAGCTCTGTTAATCGGGACGAATGGTCGGTGT

GCAAATAGTGCATGGACATGACGGTCCCGGAATAGAAAGCCACGGCTAACTACGTG

>5214 len=116 tot-seq=1

CTATCGCTAATGTTTACGCACAAAAACAGCCAATCGCAAATCTAACAGAACACTATGACT

TGATTATTAACTTGGTAGATGTCAATGCTGGAGGAACAACACAACGTATTATTTGG

>5215 len=116 tot-seq=1

GCGTGAGTGAAGAAGGGTTTCGGCTCTTAAAGCTCTGTTGTTGGTGAAGAAGGACAGGGA

TAGTAACTGACCTTTGTTTGACGGTAATCAATTAGAAGGTCGCGGCTAACTACGTG

>5216 len=116 tot-seq=1

GCGTGAGTGAAGAAGGTTTGCGGAGCGTAAAGCTCTGTTGGTGGTGAAGAAGGATAGAGG

TAGTAACTGGCCTTTATTTGACGGTAAGCAACCAGAAAGGCACGGCTAACTACGTG

>5217 len=116 tot-seq=1

CCCTGACGCAACGACGCCGCGTGAGGGATGAAGGCCTTCGGGTTGTAAACCGCTTTTGAT

TGGGAGCAAGCTGTCGGGAGAGTGTACCTTTCGAATAAGCGCCGGCTAACTACGTG

>5218 len=116 tot-seq=1

CCCTGACGCAGCGACGCCGCGTACGGGATGAAGGACTTCGGGTTGTAAAAAGATTTTGAT

TGGGAGAAAGAATTCGGGTGAGTGTACCTTTCGAATAAGCGCCGGCTAACTACGTG

>5219 len=116 tot-seq=1

GTGAGGGTGGGGCCTCCCCAGGAGAAAGAACTGCCGCTGTCCTGGGCTGGGCTGGGCTGG

GAGAGGCCAGACCGGCTTCACTGCTCAGATGCCACCTCCTCTCTCTCCGCTCTTCT

>5220 len=116 tot-seq=1

TGGGGAATCTTGCACAATGGGCGAAGGATGAAGGCCTTCGGGTTGTAAACCGCTTTCAGC

AGGGACGAGGCCGCAAGGTGACGGTACCTGCAGAAGAAGCCCCGGCTAACTACGTG

>5221 len=116 tot-seq=1

GCCTGATGCAGCGACGCCGCGTGCAGGATGAAGGCCTTCGGGTTGTAAACCGCTTCCAGC

AGGGACGAGGCCGCAAGGTGAGGGTACCTGCAGAAGAAGCGCCGGCTAACTACGTG

>5222 len=116 tot-seq=1

GCGTGAGTGAAGGAGGGTTTCGGATCGTAAAGCTCTGTTGTTGGTGAAGAAGGAGAGAGG

TAGTAACTGACCTTTATTTGACGGTAATCAATTAAAAAGTCACGGCTAACTACGTG

>5223 len=116 tot-seq=1

AGTCGCGTGAAGGAAGACTGCCCGCAAGGGTTGTAAACTTCTTTTGAAGGGGATTAAAGT

CGTCGACGGGTAGACGTTTGCAGTTACCATACGAATAAGCATCGGCTAACTCCGTG

>5224 len=116 tot-seq=1

GTAACTCTGATCCAGCAATTCAGTGGTTGTGAAGAAGGTTTTAGGACGGTAAAACACTTT

TAGTAGGGAAGAAAAAAATGACGGTACCTACAGAAGAAGCAACGGCTAAATACGTG

>5225 len=116 tot-seq=1

GAGAGAGAGAGAGAGATTGAGGAAGTGCCACACTTTAAACCATCAGATCTCACGAGAACT

CTCTCACTATCACAAGAACAGCATGTGGGAAACTGCCCCTGTGATCTAATCACCTC

>5226 len=116 tot-seq=1

TTTCAAGCAGAAGACGGCATACGAGATCTCTCTGTGACTGGAGTTCAGACGTGTGCTCTT

CCGAGCTCCTATGGGAGGCAGCAGTGGAAACTCTTCCAGGAGCTCCTAGAGCAAGA

>5227 len=116 tot-seq=1

CGGGAGGTGCCAGGCTCGTTTCAACATCAGCTCTGGCAAAAACTAACAGAGCAAGAACTC

ACTCCTTCCCGAGGGGACAAGAGGATGGCACCAAGAGGCACATGACCCCAAGACTC

>5228 len=116 tot-seq=1

GAGAGGCAGGGTGGCATCACTCCCGGGCCGGGCCCTGCCCCGCAGAGGGTGGGGGTGCCC

AGCAGGCTCTGCCGGGAGCCCCACTCTGATCCCTGAGGCAGACAAGACCATCCATT

>5229 len=116 tot-seq=1

GGGAAACCCTGACGCAGCAACGCCGCGTGCGGGATGAAGGCCTTCGGGTTGGAAACCGCT

TTCAGCAGGGAAGATCCAAGACGGTACCTGCAGAAGAAGCCCCGGCTAACTACGTG

>5230 len=116 tot-seq=1

GCAGGGAGTCAATTGATAGCCTCAATCCAGAAAGATGTCTGCAGTATAGAGCAAGCCTTC

CCACCCAGTAGCAAGCAGAGCTCCAGGAGGGCTGAGACCAACCCACCCCCGACCCC

>5231 len=116 tot-seq=1

GGGGAAGCCCTGATGCAGCGACGCCGCGTGAGTGAAGACGGCCTTCGGGTTGTAAAGCTC

TGTGGAGGGGGACGATAATGACGGTACCCATCTAGAAAGCCACGGCTAACTACGTG

>5232 len=116 tot-seq=1

TAGGGAATCTTCGGCAATGGACGGAAGTCTGGACGAGCAAAGCCGCGTGAGTGAAGAAGG

TTTTAGGATCGTAAAGCTCTGTTGTTAGAGAAGAACGGTGGTAGGAGTGGAAAATC

>5233 len=116 tot-seq=1

CATAGAACTAAGCCTCTCCACCTGTCCTTCCTGTGCCCAGCACCAAGGGAAGGAGCATCT

GGAAGGGGGAGAGGGAGGAGGTGCACAGAGCCTGACCACAGCACCCTCTTCCGCTA

>5234 len=116 tot-seq=1

CACGTGAACGATGAAGGTCTGCTGATTGTAAAGTTCTTTTATTTAGGAAAAAAAGCGCGC

TAGGAAATGACCGCGACTTGATGGTACTAAATGAATAAGTGACAGCTAACTAGGTG

>5235 len=116 tot-seq=1

CACGTGAACGATGAAGGTCTGCTGATTGTAAAGTTCTTTTATTTAGGAAAAAAAGCGTGC

TAGGAAATGAGCGCGACTTGAGGGTACTAAGTGAATAAGTGACAGCTAACTAGGTG

>5236 len=116 tot-seq=1

TTTTTTTTTCAAGCAGAAGACGGCATACGAGATCAAGTGGTGACTGGAGTTCAGACGTGT

GCTCTGCCGATCTCCTACGGGAGGCAGAAGCCGCGGTAATGGCAAGATCGGAAGAG

>5237 len=116 tot-seq=1

GAGGGAGGGAAGAAGGGGGGAGGAGAGGAAAGAGAGGGGGGGAAAGAAGAAAAAGGAGGA

GAGGAACGGGGCAGACGTGGACGGGAGGGAACCAGAAAGGCACGGCGAACGACGTG

>5238 len=116 tot-seq=1

ATATGCACAGGGTGACCTCAAAACACACCTAGGGGCTGCCCCTGGATCCACTGGCGGCCT

CCTCTCTGCCTCCGATATACCTGGACTCTCCTGCCTCCCTTTTGAAACCATGCCTG

>5239 len=116 tot-seq=1

CCAGCCAAGTAGCGTGCAGGATGACGGCCCTATAGGTTGTAAACTCCTTTTATATGGGGA

TAAAGTGGGGTACGTGTTCCCTTTTTCAGGTACCATATGAATAAGGACCGGCTAAT

>5240 len=116 tot-seq=1

GCGTGAGTGAAGAAGGGTTTCGGCTCGTAAAGCTCTGTTGGTAGTGAGGAAAGATAGAGG

TAGTAACTGGCCTTGATTTGACGGTAATTACTGAAAAAGTCACGGCTAACTACGTG

>5241 len=116 tot-seq=1

CAGCGTGTGAATGCACCAGTTGTATCAGTAGGTGTATCAGTATCTGCACCAGTTGTATCA

GTAGGTGTATCAGTATCTGCAGGCGTAGCCATCGTCTGGTAGATACACACTCAGTA

>5242 len=116 tot-seq=1

CTTATGAGGGAAGGCAGTCTCAGTGGCTTCTCCGCGGAGGGAACACGTGCCTGTCTCACA

ATCAGCACACCCCAACTGCACTATGCTGAGCCCAGCAGCACACAAGACCCATATCA

>5243 len=116 tot-seq=1

TGGGGAATATTGCGCAATGGGGGAAACCCTGACGCAGCGACGCCGCGTGCGGGATGAAGG

CCTTCGGGTTGTAAACCGCTTTTGATTGGGAGCAATAAGCGCCGGCTAACTACGTG

>5244 len=116 tot-seq=1

CAAACATTCAGACAGTTGTGGCAGTGTTCTGGTATCCTATGTGAGGGACAAACACTCAGA

TCCAGCAGCATTGTTCCTGAATCCTTTTTGATGGACAAACATTCAGATCCCCCTGC

>5245 len=116 tot-seq=1

GCGTGAGTGAAGAAGGTTTTCGGATCGTAAAGCGCTGTTGTTGGTGAAGAAGGATAGAGG

TAGTAACTGGCCTTTATGGGACGGTAATCAACCAGAAAGGCACGGCGAACTACGTG

>5246 len=116 tot-seq=1

AATTGAAGAATAAAGCGCGAGAGGCTGCCGGAATCAATTATTTGGCGGCGGCAGTGAGCG

TTGACGGTGTCGATGACTTGCGCGCCATGGCGGAGAGTCTGCGTTCACAGCTGGCT

>5247 len=116 tot-seq=1

CCCTGACGCAGCGACGCCGCGGGCGGGATGAAGGCCTTCGGGTTGGAAACCGCTGTTGAT

TGGGAGCAAGCCTTCGGGTGAGGGTACCTTTCGAATAAGCGCCGGCTAACTACGTG

>5248 len=116 tot-seq=1

GGCGCAGGCCTGATCCAGCCATGCCGCGTGAGTGATGAAGGCCTTAGGGTTGTAAAGCGC

TTGCACCGATGAAGATAATGACGGTAGTCGGAGAAGAAGGCCCGGCTAACTTCGTG

>5249 len=116 tot-seq=1

GGGTTAGAACCTCTTGCCTGGCACCAGTAAGAGGAGAACTGGGTCCCAGGCCTGTTTGGA

ATCCCTGCTGGAACCAGCCAGAGGGAAGGGAGGATGTGGACCCTCCTCAGGAACCC

>5250 len=116 tot-seq=1

CCCTGACGCAGAGACGCCGCGTGCGGGATGAAGGTCTTCGGGTTGTAAAACGCTTTTGAT

TGGGAGCAAGCCTTCGGGTGAGGGTACCTTTCGAATAAGCGCCGGCTAACTACGTG

>5251 len=116 tot-seq=1

GCGCGAAACCTCCGCAATGTGAGAAATCGCGACGGGGGGATCCCAAGTGCCATTCCTAAC

GGGATGGCTTTTCATTAGTGTAAAAAGCTTATGGAATAAGAGCTGGGCAAGACCGG

>5252 len=117 tot-seq=1

AAGAAGAAAGAGAAGCGGGGGCGGGGGAGCACTTTCTAAAAAAGTAATTTTATTTTATGT

GTTTATGTATTTGGCGTGCATGTCTGTGTACCTGCTACAAGTGCAGGCCAGAATAGG

>5253 len=117 tot-seq=1

AGGAGACCTGCTTGCAAGAAGATCAACCATAGCCAGACAGGTCCTGCTCCATGGCTCCGA

CAGTGCCCAGGGAGCCCATGTAATTACTGCCGGAACTGACATCTGAGGCTTTCACCT

>5254 len=117 tot-seq=1

GACCGTTCCTTAACCGGAACCTTCCTTCGGGACAGAGGAGACAGGTGGTGCATGGTTGTC

GTCAGCTCGTGTCGTGAGATGTTGGGTTAAGTCCCGCAACGAGCGCAACCCCTATCC

>5255 len=117 tot-seq=1

TGGGGAATAGGGCACAATGGGGGAAACCCTGATGCAGGGACGCCGCGAGAACGAAGAAGG

TCTTCGTATAGTAAAGTTCTGTCCTATGGGAAGATAATGACAGTACCATAGAAGAAA

>5256 len=117 tot-seq=1

GCGTGAGTGAAGAAGGGTTTCGGCTCGTAAAGCTCTGTTGTTGGTGAAGAACGAGTGTGA
GAATGGAAAGTTCATGCTGTGACGGTACTTAACCAGAAAGGGACGGCTAACTACGTG
>5257 len=117 tot-seq=1
AAACTTCGTTAGTGAATTCTTATCCATTCTGCCATTCTATATATTTTAAGTGGGGCATTT
AGGTCAGTTACATTCAACGTTAGTATTGAGATGTGAGGTACTATTCTATTCATCGTG
>5258 len=117 tot-seq=1
TGGAGCAGCGCCGCGTGAGTGAAGAAGGGTTTCGGCTCGTAAAGCTCTGTTGTTGGTGAA
GAAGGACAGGGGTAGTAACTGACCTTTGTTTGACGGTAGTCAATTCGAACGTCTCTT
>5259 len=117 tot-seq=1
CGCCGCGTTAGTGAAGAAGGGTTTCGGCTCGTAAAGCCCTGTTGGTGAAGAAGGACAGGG
GTAGTAACTGACCTTTGTTTGACGGTAATCAATTAGAAAGTCACGGCTAACTACGTG
>5260 len=117 tot-seq=1
TGGGGAATATTGGGCAATGGGCGCAAGCCTGATCCAGCAACGCCGCGTGAAGGAAGAAGG
CTTTCGGGTTGTAAACTTCTTTTAAGTGGGAAGAGTAGAAGACGGAACAACTTGAAT
>5261 len=117 tot-seq=1
GCCATGCAGTCCTGCATCCCATGGCACTGCCAATTACTGCAAGAGCTGGTCATGGTTAGC
CCTTTACAGGTGACCCCACTGAGGACCAGAGAGCTTGAGAAACTATCCCACATTTGT
>5262 len=117 tot-seq=1
TAGGGAATCTTCGGCAATGGACGCAAGTCTGATGGAGCAACGCCGCGTGAGTGAAGAAGG
TTTTCGGATCGTAAAGCTCTGTTGTTAGAGAAGAAAGTGGGAAGGAGTGGAAAATCT
>5263 len=117 tot-seq=1
TTGCAGTAAGCCGAGATCACACCATTGCACTCCAGGCTGGACGACAGAGCAAGACCTTGT
CTCAAAAAAAACAAAAAAAAAGTTATGGATAGGGCGTGGTGGCTCATGCCTATAATC
>5264 len=117 tot-seq=1
CTAGAGCTAGTATATGAGGAATTTTGTTTTTGTTTATTTTTTTAAGAAGGGTGGGCTGTG
GTTGAAGATGTCATGTCACTATTTTGAGCTCTAGATGGCCCTCCCACAGGCTAGGAT
>5265 len=117 tot-seq=1
GACCACAGGAAAGGGGAGGAATTTCAGACTGCGGTGTGATGAACAGCTGCCAGTCCCACC
CCAGTGACAACACCCAGCTCCGGCCCCGCAAAACTTCAGTTTTTGAAGATTCTATAA

>5266 len=117 tot-seq=1

TGTCCTCCCCTGGGGGCTGCAGCGTCCTCTCCTGGGCTCTCTCCTGGGGGCTGCAGTGTC

TACCTTGCTGGCTGGTCTCTTTTCTGTCTTTCCTGCTTCTCTGCTGACCATCTCCAC

>5267 len=117 tot-seq=1

TGAAGGAAGTTCTACTGTGGATAAAATGCTATCAAACAGCATCACATGCTACAGGGAAAT

GTTTTGTGAAAGGAACAAACTTCATTGTTTTAAGAAATTGACACAGGCACCCAACCT

>5268 len=117 tot-seq=1

CCAAGCATTTATTTCACCACGGTATGGTGTGGGCCTACTATGTGACAGGCTCATTGCTAC

ACCAGAGCCATAGACACAGCCAGCTCAAGGCAGCCACCTCTGTCTGTAGCCTCCTCT

>5269 len=117 tot-seq=1

TAGGGAATCTTCCACAATGGACGCAAGTCTGATGGAGCAACGCCGTGAAGAAGGATAGAG

GTAGTAACTGGCCTTTATTTGACGGTAATCAACCAGAAAGTCACGGCTAACTACGTG

>5270 len=117 tot-seq=1

GACCCAGGACAGGTGGTTGAAGGAATCAGGGAGGACTTCTCAGAGGAGGTAGCATCAAAG

CCCATAGCCTGATTTGTGGCACCCTCTCAGGGTTGGGGTCCCCAGTGCCAAACTGGG

>5271 len=117 tot-seq=1

GGCCACGTGGGGAACGGCCATGCTGGCGGTGGAGAGGAGTTTGCATTTCATGCTGAAAGC

CACAGAAGACAATAAAGATGCGCTGCAAAGGGAGAAACAGGAAGCTTGGAAATGAGT

>5272 len=117 tot-seq=1

CTATGGAGGGTGCGCCTGGTCCCCCAGCAGTGGCTGCACTCGAATTCTCACCGGGCCTCA

GCTGCCTCCCCACGGGGCAGAGCTCAGGACCTGCAGCCCACCATGCCTGAGCCTCCC

>5273 len=117 tot-seq=1

CAAGTAGCGTGCAGGATGACGGCGCTATGGGTTGTAAACTTCTTTTATATGAGAATAATC

GACAGTACGTATAATGTTTTGCATGTACCATATGAATAAGGACCGGCTAACTCCGTG

>5274 len=117 tot-seq=1

CGCGTGAGTGAAGAAGGTTTTCGGATCGTAAAGCGCTGTTGGTGGGGAAGAAGGATAGAG

GGAGTAACTGGCCTTTAGTTGACGGTAATCAACCAGAAAGTCACGGCTAACTACGTG

>5275 len=117 tot-seq=1

GGGGGAAACCCTGATGCAGCGACGCCGCGTGAGCGAAGAAGTATTTCGGTAGGTAAAGCT

CTACCAGAAGGGAAGAAAATGACGGTACCTTACGAAGGAGCTCCGGCTAAATACGTG

>5276 len=117 tot-seq=1

CGCGTGAGTGAAGAAGGGTTGCGGCTCGTAAAACTCTGTTGTTGAGGAAGAACGTGAGTG

AGAGTAACTATTCACGCAGTGACGGTATTCAACCAGGAAGCCACGGCTAACTACGTG

>5277 len=117 tot-seq=1

GGGGGAAACCCTGATGCAGCGACGCCGCGTGAGCGAAGAAGTATTTCGGTATGTAAACCT

ATATCAGCAAGGAAGATAATGACGGTACTTGACTAAGAAGCTCCGGCTAAATACGTG

>5278 len=117 tot-seq=1

CAGGTGATTAGAAGCTTCAGTGCAATGGTTTGTGTTATGGTGTCATGTGCTCTGCACAGG

GCCTGGCTCCCCGACAGCCCTGGGTCAGGGGCCAGTTAGGATGATGGATGATTACTG

>5279 len=118 tot-seq=1

TGAGCCAGATATTTTGAGATATCCTGGGAGACAGAATGCAGATTGGATACTCAGACGGGC

AGGGGGAAAAAAAGAATGAGCCATCAACCCCACCACTAGTGGTGAGTGCATCCCTGTT

>5280 len=118 tot-seq=1

CCGCGTGAGTGAAGAAGGGTTTCGGCTCGTAAAGCTCTGTTGTTAAAGAAGAACACGTAT

GAGATTAACTGTTCATTCGATGACGGTATCTAACCAGCAAGTCACGGCTAACTACGTG

>5281 len=118 tot-seq=1

AATGGGGGAAACCCTGATGCAGCGACGCCGCGTGGAGGAAGAAGGTCTTCGGATTGTAAA

CTCCTGTTGTTGAGGAAGATAATGACGGTAATCAATTAGAAAGTCACGGCTAACTACG

>5282 len=118 tot-seq=1

AAATTTTAAGTGTACAATATAGTATTGTTTAAGTATAATCTCGTAGAGCAGATCTCTTGC

TTAACTTAAACTTTACGCTTCTTGATCAGTAGGTCCCCATTCCCCTTCCACCTGTGCT

>5283 len=118 tot-seq=1

CGCGTGCGGGATGACGGCCCTCGGGTTGTAAAGCTCTGTGATCCGGGACGAAAGAGCCTG

AGGTAAATAGCCTGTGGAAGTGACGGTACCGGAAAAGCAAGCCACGGCTAACTACATG

>5284 len=118 tot-seq=1

TGGGGAATATTGCACAATGGAGGCAACTCTGATGCAGCAATTCCGCGTGTGTGAAGAAGG

GTTTAGGATTGTAAAACACTTTTAGTAGGGAAGAAAAAAATGACGGTACCTACAGAAG

>5285 len=118 tot-seq=1

CGCGTGAACGATGAAGGTCTTATAGATTGTAAAGTTCTTTTATATGGGAAGAAACGCTAA

GATAGTAACTGGCCTTTATTTGACGGTAATCAACCAGAAAGTCACGGCTAACTACGTG

>5286 len=118 tot-seq=1

CGCGTGTGAGATGAAGGTCCTCTGGATTGTAAATCACTTTTATTAGGGAAGAACGGCAAG

TATAGGAAATGCTACTTACGTGACGGTACCTTTTGAATAAGCCCCGGCTAACTACGTG

>5287 len=118 tot-seq=1

CCGCGTGAGTGAAGAAGGGCTTCGGATCGTAAAGCTCTGTTATAGGTGAAGAAGGACAGG

AGTAGTAACTGACCTTTGTTTGACGGTAATCAATTAGAAAGTCACGGCTAACTACGTG

>5288 len=118 tot-seq=1

TGGGGAATATTGCGCAATGGGGGAAACCCTGACGCAGCGACGCCTCTTTCTTTATTAATT

CCTTCTTTTTTTAAACCTCTTTTTATTTTTATCAATCCTTTTTTTTTATTTTACCTTT

>5289 len=118 tot-seq=1

TTTTTTTTCAAGCAGAAGACGGCATACGAGATTATAGCGTGACTGGTTCAGACGTGTGCT

CTTCCGATCTCCTACAGGAGGCAGCAGCTCCAGCAGCCCCAGGGGGCTCTAGCCACAG

>5290 len=118 tot-seq=1

CAAAATACAACAAAGAAACAAACAATAAATATTCTTGGAAACTAAATTTTAAAATAACTC

AATATCATTTAGTAACTCAAAGATTAAATAACACATGGCTGATGGGCAGCCCGGGATT

>5291 len=118 tot-seq=1

TGGCGTTCTGTCCGAGCCTGTTTGTGTTGCTATAAAGGAATACTCGAGGCTGGGTAACTT

ATGAAGAAAAGAGGTTTATTTGGCTCAAGGTTCTGCAGGCTGTACAAGAAACATGGCG

>5292 len=118 tot-seq=1

CCCGACCCGGGGAGGTAGTGGCGAAAAATAACAATACAGGGCTCTTTCGAGGCCCTGTAA

TTGGAATGAGTCCACCTTAATTCCTTTAACGAGGATCCATTGGAGGGCAAATCTGGTG

>5293 len=118 tot-seq=1

GGCCCTGGAGGGACTGAGATCTGGGAGGAGTTAGGAAGGGCCAGAGGCTGAGGATCCTGA

CCCCAGGGCTTGTGATAGCAGTAGAGGAGAAGCAGTACTGATTCCTGGGGCTGTGTAG

>5294 len=118 tot-seq=1

AGGAGCACAGTAGCTCCAGATACTTGCCTCACACCGAATTTTTCTGAATATAAATGGATT

TAAACAAACTATTTAAAATTGTTATAGAATATTATGAAAACATTCACAGTTTATTAGA

>5295 len=118 tot-seq=1

CCCGCCTTTGACGCAGCAGCACCCAGGGGGCCCGGGACAGGCAGGGGCCCGAGGGGAGCC

ACCCGCCTTCGACGCAGCAGGAGGTAAACGGAATGTTCCTTACCTACTTGGAGCCCGG

>5296 len=118 tot-seq=1

TGGGGAATATTGGACAATGGAGGCAAATCTGATCCAGCAATTCCGCGTGTGTGAAGAAGG

TTTTAGGACTGTAAAACACTTTTAGCAGGGAAGAAAAAAATGACGGTACCTACAGTAG

>5297 len=118 tot-seq=1

GGGCTTGAGGAAATGGGATGGATCAACACCAACACTGGGAACCTAACGTATGCCCAGGGC

TTCACAGGACGGTTTGTCTTCTCCATGGACACCTCCGTCAGCATGGCATATCTTCATA

>5298 len=118 tot-seq=1

TGAGCTTCTGTGATTGTCTCCCTGTGGACTGATTGCTGTAGGGCCTGCGGAAAGTTGCTG

GACACTGCCAGCGGAACCGAGGCTGACTCAGACTGCCCGGAGTATCCCTCCTCAACTG

>5299 len=118 tot-seq=1

CCGCGTGAGTGAAGAAGGGTTGCGGCTCGGAAAGCGCTGTTGTTGGTGAAGAAGGACAGG

GGTAGTGACTGGCCTTTGTTTGACGGTAATCAATTAGAAAGTCACGGCTAACTACGTG

>5300 len=118 tot-seq=1

CTGTATGTTGTCTGAGAGAATTAATTAGCTTAGGCCAGGGTGATAAAGTCTGGTACAGTT

TTATTGGGCATCACAGGCCATCTGAGGCCTGAGTGTGTCCTAAGCACAGAATGTTGTG

>5301 len=118 tot-seq=1

TGCCTGGTTCTGAGCAAACTCCCAGGGAAGAAAACGGCCCTGTCTCCATGGCCAGGTTCT

TGTGGTGTCCAGTGCGCGTCTCTCCTCCATCACACTCTCCCGGCTTGCGCAGGAGGGG

>5302 len=118 tot-seq=1

GGTTGGAAGAATCAGGCCAGTGTGGTGCCATGTGCTCCTGTGGATTGGTCCTGTCTGCCT

CTGCCCACCCCAGGGAGAGGGAGCACAGCCAAATGGTCAAGAACTTTGACTTTTCATT

>5303 len=118 tot-seq=1

GGACGGAAGTCTGATGCAGCAACGCCGCGTGCGGGATGAAGGCCTTCGGGTTGTAAACCG

CTTTCACCGCCGACGAATTGAAGACGGTAGGCGGAGAAGAAGCACCGGCTAACTACGT

>5304 len=119 tot-seq=1

GAGAGAGAGAGAGAGAGAGAGAGAGAGAGAGAGAGAGAGAGAGAGCACGCGCATGAGAGAGA

GAGGGATGCCTGCAAAGCTATCCTTTGAGCATGAGCGGTAGGTGCCTACGGAGCCTTCT

>5305 len=119 tot-seq=1

CCAAGTCGCGTGAGGGAAGACGGTCCTATGGATTGTAAACCTCTTTTGTCGGGGAGCAAA

GAGGCCACGTGTGGTCAAAAGTGAGGGTACCCGAAGAAAAAGCAGCGGCTAACTCCGTG

>5306 len=119 tot-seq=1

CCTCTGCTTCCCCAGGAAATGGTCCCACCAGACAACCACGGGACTTGAGGCAAGCCAACT

CACTCCCACTGGCCTCAGCTGCCCCTCTGATGAAGGGTTCTACCGCAAATAATGGCTTG

>5307 len=119 tot-seq=1

ATGGCCCACGCAGATGGGTCCATGCTGGGAAAAAAAAATGAGCTGCCCTCTGATCCTGCT

GCTGGATGTAGTTTGCTCTGCTGCCACCAGGCCAGCCACTTATAAGGCAGTGGCTCCAC

>5308 len=119 tot-seq=1

CCATTCAGAGAAGTCAAATACAAAACAGTATTGGTTTTTTTGAAAGTGATCTTAACTGTA

GGCATAGAGAAATGAGATAAAAGTTTTCTTTGGTGTCTTGTGAGACATATCCGTATAGC

>5309 len=119 tot-seq=1

TTCTGGGACGGGCAGAAGTACAGAGCCGCTGAGGCAGCACCCTTGGCGGACCGCAGACAG

CCGGCCACCGTCCGGACCAGAGGACAGGTGTCCGCCTGGCTTGGGAGGCGGCCTCAGCC

>5310 len=119 tot-seq=1

TAAACAACAGCGAGTGTCTCTTGGAGGCCGGTTAAGAACAGAGAAGTAGACTGGACAGGT

GACTGAGGTGTTAACAGCACATATGGAAGGCTCTTGCAGGAGACAGGTCTGATTCCAGA

>5311 len=119 tot-seq=1

CGCGTGAACGAAGAAGGTATTCGTATCGTAAAGTTCTGTCCTATGGGAAGATAATGACAG

GGGTAGTAACTGACCTTTGTTTGACGGTAATCAATTAGAAAGTCACGGCTAACTACGTG

>5312 len=119 tot-seq=1

ATGGGCGCAAGCCTGATGCAGCAACGCCGCGTGAACGATGAAGGTCTTCGGATCGTAAAG

TTCTGTTGCAGGGGAAGATAATGACGGTACTCAACAAGGAAGTGACGGCTAACTACGTG

>5313 len=119 tot-seq=1

TAGGGAATCTTCCACACTGGACGCAAGTCTGATGGAGCAACGCCGCGTGAGCGAAGAAGG

TTTTCGGATCGTAAAGTTCTGTTGTTGGTGAAGAAGGATAGAGTTATTAACTGGCCTTT

>5314 len=119 tot-seq=1

TGAGGAATATTGGTCAATGGGCGATGGCCTGAACCAGCCAAGTAGCGTGAAGAAGGACAG

GGGTAGTAACTGACCTTTGTTTGACGGTAATCAATTAGAAAGTCACGGCTAACTACGTG

>5315 len=119 tot-seq=1

CGCCGCGTGGGTGATGAAGGCCTTCGGGTTGTAAAGCCCTGTGGGGAGGGACGAATAACA

GTGACCCAATACGTCATTGGATGACGGTACCTCCTTAGCAAGCACCGGCTAACTCTGTG

>5316 len=119 tot-seq=1

GAGGAAACTCTGATCCAGCAATTCTGTGTGTGAGAAGAAGGTTTTAGGACTGTAAAACAG

GTTTAGTAGGGAAGAAAAAAAGGACGGTACCTACAGAAGAAGCAACGGCTAAATACGTG

>5317 len=119 tot-seq=1

CTGTGGAAACTGCACTTGTCCCCTCTGGGGAGCTGGTGTGGCAAAGGTGTGTGTCTTTCC

TTGGGAGGCAAATGGATGCCTTAGAAGGTGGCCAGACTTGAGTTGTTTAAGAATCGTTG

>5318 len=119 tot-seq=1

GGCGTGCCCAGACTCCTGGAGCCTGTCCCCTAACAACCCACTCCCCAAGGCCTCGCTGAC

CCTGAAGCTGGGCTTCGCCTGCCCCCTATTCCTCCGGGTGGGGCTGTAGGAGCCTCAGC

>5319 len=119 tot-seq=1

CCCGCCGCGCTCCTGACCTCCCGGGCTGACTCCCTCTGTCGTGGGGCCGTCCCGTGCACG

CTGGGCGCTGGGCAGCCCTGAGCTCCCGTCAGGACTGTCAATGTCTGGCCTACACTACG

>5320 len=119 tot-seq=1

GAGGATGACAGAAAGAGAAGTCTGGGGTCCAGGGAGCCAGACAGAGAGCACAGAGGGGAG

GAAACATTTTTGGAGTTGGGCCTTGGCACTGGGGCTGGAAGAGAGAAAGTGTGTACAGG

>5321 len=119 tot-seq=1

TGGGGAATCTTGCACAATGGGCACAAGCCTGATATAGCGACGCCGCGTGGGGGATGACGG

CCTTCGGGTTGTAAACCTCTTTCGACTATGACGAAGCCTTTTTGGTGTCGGTAGTGGTA

>5322 len=119 tot-seq=1

AACCAGGGATGGCTGTGAGTCGGGGAATCGGATGCTAAGATCTTGAGGATGACACAGATT

CCAGGTTGTGGAGGGCCAGCCCTGCCAAGGGATGGGAATGAATTGTGGACATGGACCTC

>5323 len=119 tot-seq=1

CAGAGCTGAAGAAATGCATGTCTAGAACTTCCCTGTTTATACTCGGGCCTCTTGAAAACA

GGGAATGTGCTTTACCCATTTGCCTGCCTTCTTCAGTCAAGCTTCATGAGTGGTAACAC

>5324 len=119 tot-seq=1

TAGGGAATCTTCCACAATGGACGCAAGTCTGATGGAGCAACGCCGCGTGAGTGAAGAAGG

TTTTCGGATCGTAAAGCTCTGTTGCGGTAATCAACCAGAAAGTCACGGCTAACTACGTG

>5325 len=119 tot-seq=1

GCCAAGTAGCGTGCAGGATGACTGCCCTATGGGTTGTAAACTTCTTTTATATGGGGATAA

AGTGTTGCACGTGTTCCCTTTTGCAGGTACCATATGAATAAGGACCGGCTAATTCCGTG

>5326 len=119 tot-seq=1

CAGAGCCAAAAGATAGCGGCTCAATTAGTCTTGGTCCTACAAAACCCTATTATCCACTCT

CAAAGTTACGAGGAGAACACACACCAGCTCACCTGTATGCAAGAGCCAAAGCCCAGGCC

>5327 len=119 tot-seq=1

TTCAAGCAGAAGACGGCATACGATATCGACAAGTGACTGGAGTGCAGACGTGTGCTCTTC

CGATCTCCTACGGGAGGCAGCAAGAGACAAAGAGAGGAGGGGAGAAGGGTATGAAATCC

>5328 len=120 tot-seq=1

GAGGCTTTTGCCTAGTGAGCAGCGCCTCCCCTAGGCTGAGAGCAGAAGAGGAGGAGAGGC

AGGGTACCAGTCCCAGGAGGGATGACAGCACACAAGAAATTGACACCCACCACCAGCTGA

>5329 len=120 tot-seq=1

GCCATAAAGAGGGCAATATGAGTGTTTGTGTCAGAGATAATCATCAGGAATGGCCTGTCA

AAATATATTTTCATAGAGTAGATTACACCTTGACAACATTGGAGAGTTTGATATCCTGTG

>5330 len=120 tot-seq=1

ACATTTAGAGGAGAGTAGCTACTCCTTGCCACACCCCATGGTAACAAGTATCATCACATG

GAGAAATTGGAATGCACACACACATACTACTCGAGTGAAATATAAATTTGTTGTGGCCGG

>5331 len=120 tot-seq=1

CTGATGCAGGGGACATGGAGGGTGCTGCTTACCGACCATTCTGCTTTCTTATAGATTCCA

GGACCAGCAGCGGGTAATTTCTAGATCGGAAGAGCGTCGTGTAGGGAAAGAGTGGGCCAA

>5332 len=120 tot-seq=1

TAGGGAATCTTCGGCAATGGACGGAAGTCTGACCGAGCAACGCCGCGTGAGTGAAGAAGG

ATTTCGGATTGTAAAGCTCTGTTGTCAGAGAAGAACGTTGGTAGGAGCGGCAAATCTACC

>5333 len=120 tot-seq=1

AAGACGGCATACGAGATCACTGTGTGACTGGAGTTCAGACGTGTGCTCTTCCAGCAGTCG

CGGTAATGCAGAGATCGGAAGAGCGTCGTGTAGGGAAAGAGTGGGCCAATGTGTAGATCT

>5334 len=120 tot-seq=1

TAGAGAATATTGCACAATGGAGGAAACTCTGATGCAGCGACGCCGCGTGATTTAGAAGGC

CTTCGGGTTGTAAAAATCTTTTCTATGGGAAGAAGATGACAGTACCATAGGAATAAGGAC

>5335 len=120 tot-seq=1

CGCCGCGTGAGTGAAGAAGGGTTGCGGCGCGTAAAGCTCTGGTGGTGGTGAAGAAGGACA

GGGGTAGTAACTGACCTTTGTTTGACGGGAATCAATTAGAAAGGCACGGCTAACTACGTG

>5336 len=120 tot-seq=1

CAGCAGGAAGTCTCAGCCAGCATGATAAGCAGGAGCAGAATGCCAGCAGCCCGCGGTAAT

CTTAAGATCGGAAGAGCGTCGTGTAGGGAAAGAGTGGGCCAATGTGTAGATCTCGGTGGT

>5337 len=120 tot-seq=1

CGCCGCGTGAGTGAAGAAGGGTTTCGGTTCGTAAAGCTCTGTTGTTGGTGAAGAAGGACA

GATATAGTAACTGTTATTGGTTTGACGGTAATCAATTAGAAAGTCACGGCTAACTACGTG

>5338 len=120 tot-seq=1

CGCCGCGTGAGTGAAGAAGGTTTTCTGATCGTAAAGCTCTGTTGTTTGTGAAGAAGGATA

TAGTTAGTAACTGGCCTTTATTTGACGGTAATCATCCAGAAAGTCACGGCTAACTACGTG

>5339 len=120 tot-seq=1

GCTTGTGGTAAATATTTGGCGTCCAGCTCCATTTGGCATGGTTATATTTGCTCCATTGGG

GGAAAAAATAACTCTCTGTTTGCCATGGTGATGGATTTGTCGTTCTCCAATTAGAAGTTG

>5340 len=120 tot-seq=1

AATGGGGGAAAAAGTGATGAAGAAAAGACGAGTGAGTGATGAAGGTTTTCGGATCGTAAA

GCTCTGTCTGTGGGGAAGATAATGACGGTACCCAAGGAGGAAGCCAAGGCTAACTACGTG

>5341 len=120 tot-seq=1

TGGAAAGCTGTGGGCTGGCACTGAAATGAGCAGCTGCTGCTATGTCACTGAGTGGATGTG

GGGTGCCGGTGAAATCAGTGTATCACTGAGCCCTGATAGAATGGTGGAAAATATATATTC

>5342 len=120 tot-seq=1

TTTTTTTTTTCAAGCAGAAGACGGCATACGAGATTTATCCGTGACTGGAGTTCAGCGTGT

GCTCTTCCGATCTCCTACGGGAGGCAGCGGCCCCGTCTGAGAAGTGAGGAGCCCCTCCGC

>5343 len=120 tot-seq=1

GAGTAGCAATTTCTTCTCTAAGTTTCTCTTCAACCCTCCTTCTCCAAAGGAAGAGATTTG

TTTCTGGCTGGCAAATGCCTGCTATTTGCCCTTGCTTTACATGCATTATCTTATTTAATG

>5344 len=120 tot-seq=1

GATTTTTAAGAAGCTCTTCAGGTGATTCTAATGTAAGGCCAAATTTAGAAACCACTGCTT

AAGCTTTGGTGCTTAAGAATTGCTGCTACTTCTTTTATTCTAGCCCTCCTGTGATGAGAT

>5345 len=120 tot-seq=1

AATTGCTTGAACCCAGGAGGTGGATATTCCAGTGAGCCGAGATCACACCATTGCACTCCA

GCCTGGGCAACAAGAGCGAAACTCTGTCTCAAAAAAAAAAACACCCTAATTCCAGGCCAA

>5346 len=120 tot-seq=1

AAGGGGCGAAAGCCTGATGCAGCGACGCCGCGTGAGAGAAGAAGTATTTCGGTATGTAAA

GCGCTATCAGAAGGGAAGAAAAGGACGGTACCGGACGAAGAAGCTCCGGCTAAATACGTG

>5347 len=120 tot-seq=1

GGAAACTGTTTTTGGGTTTCTGGACCGACGGTACCTGCAAGGTGGGGATGATGAACCCGG

CGCTTGGGCACTGAGGTTGGACCTGACGGAGGCGGAAGTGGCATCATTTCCTGGCTCTTG

>5348 len=120 tot-seq=1

TGGGGAATATTGCGGCGACGCCGCGTGCGGGATGAAGGCCTTCGGGTTGTAAACCGCTTT

TGATTGGGAGCAAGCTTTCGGGTGAGTGTACCTTTCGAATAAGCGCCGGCTAACTACGTG

>5349 len=120 tot-seq=1

TGGGGAATATTGCACAATGGGCGCAAGCCTGATGCAGCAACGCCGCGTGAACGATGAAGG

TCTTCGGATCGTAAAGTTCTGTTGCAGGGGAAGATAATGACGGTACCCTGTTCTTCCGCC

>5350 len=121 tot-seq=1

AACATGTTTTGGATTAATTCCCATTCATGTCTTGACATGTTGTTTGGAGTAGGGGAGGTA

GGTGGACCATGGCAGGAAAGCTCTTGCTTCCAAGCTCAGCAGGTCTCTCCTGTGCTTCCC

T

>5351 len=121 tot-seq=1

TCTGATGGAGCAACGCCGCGTGAGTGAAGAAGGGTTTCGGCTCGTAAAGCTCTGTTGTTG

GTGAAGAAGGACAGGGGTAGTAATGACGGTAATCAATTAGAAAGTCACGGCTAACTACAT

G

>5352 len=121 tot-seq=1

CTCGTCCATGAACGGGCACTGCGAAGAGAGGTCAGGGACACAGCGGCCATTAAGGGCTGG

CACAGCTGTCCTTCAAGGGCAGGCAGCACTCCACAAGGGTCACAGCAGAGAAAGAAACAG

C

>5353 len=121 tot-seq=1

TTGAGATTTGGGTGGGCCCACAGCCAAGCCGTATCACCTACTGGAACCAGAAGAAGAATG

AGCAACACATGAAACAGAGAGGGCCACCTCAGCTGCTCTAGCTGAGACCAGCCTAGATCA

G

>5354 len=121 tot-seq=1

CAGCCATGCCGCGTGTGTGAAGAAGGGCCTATGGGTTGTAAACCTCTTTTGTCGGAGAAC

AAAACCCGGGACGTGTCCCGGACTGCGTGTATCCGAAGAAAAAGCATCGGCTAACTCCGT

G

>5355 len=121 tot-seq=1

GCGGCCATGCCGCGTGTATGAAGAAGGCCTATGGGTTGTAAACTGCTTTTGTATGGGGAT

AAAGTTAGGGACGTGTCCCTATTTGCAGGTACCATACGAATAAGGACCGGCTAATTCCGT

G

>5356 len=121 tot-seq=1

CAATGGGGGAAACCCTGATGCAGCAACGCCGCGTGAGGGAAGAAGGTTTTCGGTTTGTAA

AGCTCTGTCCGCAGTGAAGATAATGACGGTACCTGAGGAAGAAGCCCCGGCTAACTACGT

G

>5357 len=121 tot-seq=1

TCCACAATGGACGCAAGTCTGATGGAGCAACGCCGCGTGAGTGAAGAAGGGTTTCGGCTC

GTAAAGCTCTGTTGTTGGTGAAGAAGGACAGGGGTAGTAGAAAGTCACGGCTAACTACGT

G

>5358 len=121 tot-seq=1

GGACCGAGAGTCTGATCCAGCAATTCTGTGTGCACGATGTAGTTTTTCGGAATGTAAAGT

GCTTTCAGTTGGGAAGAAAGAAATGACGGTACCAACAGAAGAAGTGGCGGCTAAATACGT

G

>5359 len=121 tot-seq=1

GCAGCGACGCCGCGTGAGGGATGGAGGCCTTCGGGTTGTAAACCTCTTTTATATAAGAAT

AAAGTGCAGTATGTATACTGTTTTGTATGTAGTATATGAATAAGGAGCGGCTAACTCCGT

G

>5360 len=121 tot-seq=1

CTGCTAAGTGTCCAAACTGTGTTCAAATAAGGCAAACACCATCCTGTAACCAATCCAGCT

GTTTCCATACCTTACTGCCAGTTTCTGTACATCATTTCCCTTTTCTGTCTATAAATCTTC

T

>5361 len=121 tot-seq=1

ATGCCGCGTGTATGAAGAAGGCCTTCGGGTTGTAAATTTCTTTCAGCAGGGAAGAAGGGA

GTAAAGTTAATACCTTTGCTCATTGACGTTACCCGCAGAAGAAGCGCCGGCTAACTCCGT

G

>5362 len=121 tot-seq=1

CTCTGCACTTCCCAGCTCTGTCAATCCCTAAGGCTATGCAGCAGCCACCCCCACCAGGCC

CTCGGGGCACTGAAGCCTCACCCACCTCCCCAGGGGCAGGACATTGATCCGCTTCCGCAC

C

>5363 len=121 tot-seq=1

CGCCGCGTGAGTGAAGAAGGTTTTCGGAGCGTAAAGCTCTGTTGTAAGAGAAGAACGTGT

GTGAGAGGGGAAAGTGCACACAGTGACGGTGACTGACCAGAAAGGGACGGCTAACTACGT

G

>5364 len=121 tot-seq=1

TGGCTCAGTCACACTTCCCGCAGGAGAGGGCCAGCCTGGCTGAGCCTGTATTTCTCCCTC

CCCCATGGAGTCCTCCTGCTCCCCTCTGGCTCCCGGAGTCCACAGACCTCTGCATCTCCC

T

>5365 len=122 tot-seq=1

TTCCCTTCTTTGTTTGTTTGTTTGTTTGTTTGTTTTCAGATCAATTACAAAGCATGGTAA

GTTTAGCAAAATGTTGAATGTTTTAATTTGAATTTCAAATGTTCTGGTCAAAACTTTTTT

AG

>5366 len=122 tot-seq=1

ATTAGTTGAGAGAGCACTGGCTAGCGTGCTCAATGCTCCAAATTCAATCCCCCAGTATGG

TGTAAACTGGCACAGTAGCACATGCTTGTACTCCTAACATTCGGGAGATATGTGTAAGAA

AC

>5367 len=122 tot-seq=1

CCAGCCAAGTAGCGTGAAGGATGAAGGGTCTATGGAGTGTAAACTTCTTCTAGACGGGAA

TAAACGAATCCACGGGTGGATTTTTGCATGTACCGTATGAATAAGGAGCGGCTAACTCCG

TG

>5368 len=122 tot-seq=1

AATTCTATGTTATCTATATTTTAGAAGATAATATTCCCAAATGCACCAACATCTGTACTC

TGATGCCCACTTGTTTTCAAGTATAATGGGCTAAAGGTTGGAAAGACAACTTGAGTTTGG

TC

>5369 len=122 tot-seq=1

AACGCCGCGTGAGTGAAGAAGGATTTAGGTTGGTAAAAATATGTTGTTAGAGAAGAAAAG

AGAATAGAGTAAATGTTATGAGTGTGAAGGTATCTAACCAGAAAGCCACGGCTAACTACG

TG

>5370 len=122 tot-seq=1

ACTCCAGCTCAGCGCACAGCATGAGGAGGACGAGGGAACAATAGGGCGTTCCTTTGTGCC

TGCCCTTGAAACGCGGTGGAGGCCACTCAGTGAGCCCGGGCAGTGCCATCGCTCACGTGT

CC

>5371 len=122 tot-seq=1

GGAGCGGGTGGGACATAGGGCTGAGCAGGCCAGGCCCCAAGGAGCACGTGCTTTTCTGGA

CAGGCAGAGGGACAGTGCCCTGTGTTCCAGGACACACAGATGCATGAGGAGGAGGAGGCC

AG

>5372 len=122 tot-seq=1

CAGCCAAGTCGCGTGAGGGATTAAGGCCCTACGGGTCGTAAACCTCTTTTGACAGGGAGC

AAATGCGCCCACGTGTGGGCGAGTGGAGAGTACCTGAAGAAAAAGCATCGGCTAACTCCG

TG

>5373 len=122 tot-seq=1

TTTCCCTCTTGAGTGTCTGCTCTGATAAAGAACCTTCCCAGAGGCTGGTCCTTGTCTCAA

GCCCACTAGAAATTCTGCCATGAGTGTGAGTTTTAGAGACACTGGGGAAAATAGAATACA

CA

>5374 len=122 tot-seq=1

GGGCAACTGTGCAAACCTGTCCACCAAGAGCTGATGGGCTCATGGGTCCAGAGTGGAGAT

GTCACTGCTGCAGGAGCCGGACGGCCAATGCTGAGCCAGGCCAGGAAGCCACAGCAAGTC

AG

>5375 len=122 tot-seq=1

TAGGGAATTTTTCACAATGGGCGCAAGCCTTATGAAGCAATGCCGCGTGAACGATGAAGG

TCTTATAGATTGTAAAGTTCTTTTATATGGGAAGACCGGAAGAGCGTCGTGTAGGGAAAG

AG

>5376 len=122 tot-seq=1

CACGGCGGTGAAAATGGCGGGCAGCCGGTGATAATCGTAGGTGCCGTCCCGTTTCACTTT

GGCGATGACTTCGGCCATGGTGTCCGGGAGTATCCCGAATTTTTGCTGGAACAGACCTAT

GG

>5377 len=122 tot-seq=1

AGGCAGTGCGAAGAGGAAAGTTTGTAGCTCTAAACACCTACGTCAAAAAGTCTGAAAGAG

CACAGACAATCTAAGTTCACATCTCAGGGAACTAGAGAAGCAGGAACAAGCCAAACCCAA

TC

>5378 len=122 tot-seq=1

GTGCATTGAGTTGAACTGAAATTCAACTGAGTTGAGTTGGATGTAAAGAGCAGGGCTGGC

TCTCAGCTGCTGCCCATCCCTGGCCAGGCCTCACCTCTCGGTGAACATCTTCACCTTGCT

GA

>5379 len=122 tot-seq=1

CCAGCCAAGGAGCGGGCAGGAGGACGGCCCGAGGGGGGGGAAACGGCGGGGGGGGGGGAA

GAACGGACATGACGTGTAATGTTTTGCATGTACCCTTCGAATAAGGACCGGCTAATTCCG

TG

>5380 len=122 tot-seq=1

AGAGCCTTGGGAGGGAGCACAGCCCTGTGGTGCCTTTTCTGGGCCCAGTGACCTGGATTT

TGAACTCTGGCCTTCAGCATACATTTCTGCTGTTTTCAGCTATCACATTTGTGTGTTTTG

TT

>5381 len=122 tot-seq=1

AACCATGAAGCAGGAGGCGGCAGTGCTAATGGCCCCTGTACCCGAAGTAGCTCTAATTGC

AGAGTGAGGCAGCCAGAGGGGCCCCTCATGGGCAGAGGAAGGCAGGGACAGTGCTCCTAC

CT

>5382 len=122 tot-seq=1

TCATGGGCTCCAGTTTATGCTAGTGAGTTCCAGCTCATTCTCCACTTTACCAACTGCCTG

CCCCGGGGAACTCAAACTCCAGCATCAGATATGAAGACAACAACCGTGTAGAAACTGCTT

AA

>5383 len=122 tot-seq=1

AAATTTGGACACAGCCACCCATGTGCACACAGGGATAACACAGGCAAAGATGTCAAGATG

AAGGCAAAGATTGGGGTGATGCTTCTATATGCCAAGGAACACCAAACATGGCCAGCAAAC

AC

>5384 len=122 tot-seq=1

AACGCCGCGTGAGTGAAGAAGGGTTTCGGCTCGTAAAGCTCTGTTGGTAGTGAAGAAAAA

TAATGGTAGTAACTGGCCATTATTTGACGGTAACTACTTAGAAAGTCACGGCTAACTACG

TG

>5385 len=123 tot-seq=1

TGGGGAATAGAGCACAATGGGGGAAACCCTGATGTAGAAACGCCGCGGGAGTGAAGAAGG

TTTTCGGATGGTAAAGCTCTGTTAGCAGGGAAGAGGAAGGACGGTACCTGCAGAGGAAGC

CAC

>5386 len=123 tot-seq=1

AGACAACGACTGCCCCAAACTGAGACCCTTCCCATGGACAAGAATCAACCCGACACTATT

AATGATGGTCTGTTATACTTGCAGACAGGAACCTAGCATAACTGTTCTCTGAGAGGTTAT

CAC

>5387 len=123 tot-seq=1

CGGCAGCCAGGTGTTTCCTTCCTTTTCAAGTCTAATCCACTAAGAGCAAACACCAGCTTG

TTGCCACACAGCCTTACTTCAGAAAAGGAAACAAAGTATACAGGCTAACAACATGTGTCA

ATG

>5388 len=123 tot-seq=1

CAGCCCTGCACAGCGTGTCCTGTCACCCCAATCATGTGCCAGAGGACTCACGAGCCTAGG

TGGGCTTCTTTCAAATGGCTCCTAGGAGTGAACAAGGTGCTGCCATGGCCAGTGGAGACT

ATC

>5389 len=123 tot-seq=1

GGGAGGGAGGGTTACAATCTAACGTTTGGCTAAGGGTATAGGTAGAGGGGTAGGGACTAT

GATTTGCACTGGGCATCACTTAAAAGTTGAGAAAGCAAGGGTACAGCCAAAAGGTCCTTA

AGA

>5390 len=123 tot-seq=1

GACAACAGAAAACAGATGCCAATTATTTGCCGATGGCTGCTGGAAAACAAAACATTTTAT

GACATTTACTCTGCCTTAGCAGAGCTGCTTCTAGTGCCAAAGAAAAATAAACTGGTCAAA

TTA

>5391 len=123 tot-seq=1

GCGACGCCGCGTGCGGGATGAAGGCCTTCGGTTCGTAAAACTCTGTTGTTAGGGAAGAAA

AAATGTATAGTAACTATATACAAAAGAGACGGTACCTAACCAGAAAGCCACGGCTAACTA

CGT

>5392 len=123 tot-seq=1

ACCAGCCAAGTAGCGTGCAGGACGACGGCCCTATGGGTTGTAAACTGCTTTTATGGGGGG

ATAAAGTGCGTGACGGGGCATGCATTGCAGGTACCACATGAATAAGGACCGGCTAAGTCC

GTG

>5393 len=123 tot-seq=1

TACCAATGAGACCTTTCAGGAGCTCAAAAGATCCCACCAGGCTCCCATCAGCCAAGATCT

GTGGCCGGGGGCAGGGTAGCAAGCCAATTTCTTGGGTGGAGCTGGGCTTTGTCCAGGTGC

CTT

>5394 len=123 tot-seq=1

ACCAGCCAAGTAGCGTGAAGGATGACTGCCCTATGGGTTGTAAACTTCTTTTATACGGGA

ATAAAGTGAGTCTCGTGAGACTTTTTGCATGTACCTTATGAATAAGGACCGGCTAATTCC

GTG

>5395 len=123 tot-seq=1

AAGACGGCATACGAGATTGGTCAGTGACTGGAGTTCAGACGTGTGCTCTTCCAACAGCCG

CGGTAATCCCAAGATCGGCAGAGCGTCGTGTAGGGAAAGAGTGTGCCAATGTGTAGATCT

CGG

>5396 len=123 tot-seq=1

TAGGGAATCTTCGGCAATGGGGGCAACCCTGACCGAGCAACGCCGCGTGAGTGAAGAAGG

ACAGGGGTAGTAACTGACCTTTGTTTGACGGTAATCAATTAGAAAGTCACGGCTAACTAC

GTG

>5397 len=123 tot-seq=1

TAGGGAATATTGCACAATGGAGGAAACTCTGATGCAGCCATGCCGCGTGTGTGAAGAAGG

ACAGGGGTAGTAACTGACCTTTGTTTGACGGTAATCAATTAGAAAGTCACGGCTAACTAC

GTG

>5398 len=123 tot-seq=1

ATAGCTTTAGGGTAGTTTCTAGGGTGCTTTGGCTTGTGGGACTGAGAGCTTTAGGCTCTA

GAGTCTCAGCTGTGGGGATTCATTGTAGATGAGAGCACCAAGCCAGAAGAGGGAAGGGGA

GCC

>5399 len=123 tot-seq=1

GGTGGGCACCAGGACAGTCAGAAGGGAGATCTCAGAACATTTAAGGAGACTAGAGGATAT

TTAAACAAGTAGCCAGTGAGGCACTGGAGATGAAGAGAAGGGAAGTACGTCTGCTCAAGG

ATG

>5400 len=123 tot-seq=1

CGCAGTGTACGGGGGACAGGAGTGGCGCCCCCCTCAGTGGCCGGGTCCTCCTCGGGGGCC

CAGCTGTGGGGACACCCAGCAGTGAGCGAGAAAGCCCATCAGGCATCCCACCCTCTCCCA

CCC

>5401 len=123 tot-seq=1

ACCAGCCAAGCCGCGTGAGGGAGGAAGGTACAGAGTATCGTAAACCTCTTTTGTCAGGGA

ACAAAGGCGGGGACGTGTCCCCGGATGAGTGTACCTTTCGAATAAGCGCCGGCTAACTAC

GTG

>5402 len=123 tot-seq=1

TGGGGAATATTGCACAATGGGCGCAAGCCTGATGCAGCCATGCCGCGTGTGTGAAGAAGG

ACAGGGGTAGTAACTGACCTTTGTTTGACGGTAATCAATTAGAAAGTCACGGCTAACTAC

GTG

>5403 len=123 tot-seq=1

GAGTAGTCAGCTGCTCCCCGGCGAATGGGAGGGAGTGAGGCCACTGGTTCAAGGAAGAGG

ACTTCCAGAGCATGGAGTCAATCAGGCAGCACCTGCCTGGAGGTTCTCTGGAGGCGCCGA

GTA

>5404 len=123 tot-seq=1

TAGGGAATCTTCCACAATGGACGCAAGTCTGATGGAGCAACGCCGCGTGAGTGAAGAAAG

ATAGAGGTAGTAACTGGCCTTTATTTGACGGTAATTACTTAGAAAGTCACGGCTAACTAC

GTG

>5405 len=123 tot-seq=1

TGGGCAGCTGGGAGCTGCGACTCATTCAAAGAGAGGGAGTTAGTGCAGGGTACCGAGATC

AGGAAGAGGAGTGGTGCAGAGGTGGGAGGTGATGAGACTCAAGACTACAGAGAGAAGAAA

GGG

>5406 len=123 tot-seq=1

CCACAATGGACGCAAGTCTGATGGAGCAACGCCGCGTGAGTGATGAAGGGTTTCGGCTCG

TAAAGCTCTGTTGTTGGTGAAGAAGGACAGGGGTAGTAACTGACCTTCACGGCTAACTAC

GTG

>5407 len=123 tot-seq=1

TGGGGAATATTGCACAATGGGCGCAAGCCTGATGCAGCCATGCCGCGTGTATGAAGAAGG

ATAGAGGTAGTAACTGGCCTTTATTTGACGGTAATCAACCAGAAAGTCACGGCTAACTAC

GTG

>5408 len=123 tot-seq=1

AAAAACAAGCAAGTGTGGTGTCATTAATGTTTAAATATCTAATTTTGAAGACCTCAGAAG

GCTACTAAAACACCTCATTCATTTTGTTTGCAATTAAGAAAGAAAATTATATTATAGCTC

TTG

>5409 len=123 tot-seq=1

TTTGAAAATTCAGTTCTGAGACCCCATTCAGGCATTCTTCTTGGAGGTATCCAGTAGGCA

GCTATTATAGGAGTCTGGAGTGTTGGAGAGGGGCTGGAGCTGAAGAGGGCTTTTTGGAAT

TCG

>5410 len=123 tot-seq=1

CTCAGGGTACGGGGGCCCGGGCCCGGGGCTGGTCCACGGGAAGGAGATGCCGAAAGGGTA

CCGGCGGCCCCTGGCCCACGCCAAGCCGCCCTACTCCTACATCTCGCTCATCACCATGGC

CAT

>5411 len=123 tot-seq=1

TTTTTTTTTTTTTTTTTGAGATAGAGTCTCGCTCTGTTGCCCAGGCTGGAGTGCAGTGGC

ACAATCTTGGCTCACTGCAACCTCTGCCTCCTGGGTTCAAGGGATTCTCCTGACTCAGCC

TCC

>5412 len=123 tot-seq=1

AGCTGGAGAAGAGCTTGAGGCCTTGGCTGGGCACCCAGGAGCCCCCTGGTCCAGGCCAGC

CCTGGGACTCCAAGAGGCCATGCTCTGCCTTGGGACCAGAGAGCCACGTGGGGCATTGAC

AGG

>5413 len=123 tot-seq=1

AACGCCGCGTGAGTGAAGAAGGTTGTCGGATCGTAAAGCTCTGTTGGAAGAGAAGAACGT

GTGTGAGAGTGGAAAGTTCAAACAGTGACGGTAACTTACCAGAAAGGGACGGCGAACGAC

GTG

>5414 len=123 tot-seq=1

ACCAGCCAAGTAGCGTGCAGGATGACGGCCCTATGGGTTGTAAACTGCTTTTGTTTGAGA

ATAAAGGGCAGTACGTGTAATGTTTTGCATGTACCATTCGAATAAGGACCGGCTAATTCC

GTG

>5415 len=124 tot-seq=1

CGCCTCCTGGCGACAAAAGTGTATGCGCCGCGTGGGAAATCTCCAGAGTTAAATCAGGAC

GGGGTGGAGCAGAGCTGAGGAGAGCAGGTTCCTGGCAGAATCCGTGGCCAATCCTGGCTG

AGCT

>5416 len=124 tot-seq=1

CCACTGTTGTGGCCCAGTAAATTCCTTTTATTTTGTATCTATTAAAATTTAATGTGAATC

ATTCGCAGCTAGTGTGGCCATAACAGCAAAGAGAAGGTAAATTGGTTCAGAGAGGCTCTT

TCTG

>5417 len=124 tot-seq=1

AGAAAGAGCTTGGTGGCGCTAGGGCGTCCTTGTGGATGTGACAGGGAAGAACAGTTTGGA

ATTTTTTCCCCCGTTTCATTGCCTGGTGATGAGAGCCAGAAGGGATGCATGCCTTCTACA
CTTG
>5418 len=124 tot-seq=1
AGGAGGAGCAGGAGGAGGAGGAGGAGGAGGAGGAGGAGCAGGAGGAGCAGGAGGAGGAGG
AGGAGGAGGAGGAGGAGCAGGAGGAGCAGCAGGAGGAGGAGCAGGAGGAGGAGGATCAGG
AGGA
>5419 len=124 tot-seq=1
CAGAGCTAACTGCCCTTTTATACCTGGGCTCTGTTCATCTTCATTGGGAAACAGGTCATC
CAGGGAGTCTTTGGGGACATCACCCTTCTCCTCCTGGAAAGGAAGACAGGAATGATTGTC
AGCC
>5420 len=124 tot-seq=1
CCACAGCCGGGCTCTTTGACCAGCTCACAGCCCAGAGGGCTGGGGGGACACATGGACAGA
GCTTTCTCGTCGCAGGGTTCACAATGCACGAAAGAACCCAGGCCTTGAGCCGGTACGGCA
TAGG
>5421 len=124 tot-seq=1
GGCAACCAATGAGCCACTACACTGGCTTGAAATTAAAAGTTCTAAGAAAGTTAAAAGGGG
GCTGAGGAATGGCTCAGTGGGTGAGAGCACTGACTGCTCTTCCAGATGGGCCTGAGTTCA
ATTC
>5422 len=124 tot-seq=1
GCAGGTGAATTTCTGAGTTTGAGGCCAGGCTGGTCTACAAAGTGAATTCCAGGACAGTCA
GGGCTACACAGAGAAACCCTGTCTCGAAAAAACCAACCCCCCCAAAAGAAAGACTCCAAA
ACAA
>5423 len=124 tot-seq=1
GATGCCCAGCGTCTGCGGGGGCCACCTGGGGGGTGAGTCCTCTGGCCCCAGTCACGCCTT
CAGATGACCGCAGTCCCGGCTGACATCCTGAGTGTAACTTTATGAGAGGCAAGAGAAGCC
AGGA
>5424 len=124 tot-seq=1
CGGCAATGGACGGAAGTCTGACCGAGCAACGCCGCGTGAACGAAGAAGTACCTCGGTATG
TAAAGTTCTATCAGCAGGGAAGAAAATGACGGTACCTGACTAAGAAGCCCCGGCTAACTA

CGTG

>5425 len=124 tot-seq=1

TGTTGTCAGTGAGCTAAACCCTCCTCTTCCAGGGCAGGAAGTCAATGGACAACATCGAAA

CCCGATACATCAAGAAACAGCAATAGAATAGCATTTCAGGACTTCATGGCAACCCCAGAA

CCAG

>5426 len=124 tot-seq=1

TAGGGAATCTTCCACAATGGACGCAAGTCTGATGGAGCAACGCCGCGTGAGTGAAGAAGG

GTTTCGGCTCGTAACTGGCCTTTATTTGACGGTAATCAACCAGAAAGTCACGGCTAACTA

CGTG

>5427 len=124 tot-seq=1

ATAGATGATTGGCTATTTCTTTACCTACTGTTTTTGCCTAATTAGCATTTTAGTGAGCTC

TCTGATTTGTTGGGTGTGAGCTAAGTTGCAAGCCCTGTGTTTAAAGGTGGATGCAGTCAC

CTTC

>5428 len=124 tot-seq=1

GCACAATGGGGGAAACCCTGATGCAGCGAAGCCGAGTGAGCGAAGAAGGCCTTCGGGTTG

TAAAGCTATGTTAGATGGGAAGATAATGACGGTACCATAAGAAAAAGCCCCGGCTAACTA

CATG

>5429 len=124 tot-seq=1

GCAACGCCGCGTGAGTGAAGAAGTGTTTCGGCTCATAAAACTCTGTTGTTGGTGAAGAAG

GGCGGGGGTAGTAACTGACCTTTGTTTGACGGTAATCAATTAGAAAGTCACGGCTAACTA

CGTG

>5430 len=124 tot-seq=1

GATGCAGCGACGCCGCGTGAGCGAAGAAGTATTTCGGTATGTAAACTGCTTTTATGTGGG

GATAAAGTGCGTGACGTGTCATGCATTGCAGGTACCACATGAATAAGGACCGGCTAATTC

CGTG

>5431 len=124 tot-seq=1

TTGTTTTGACTGTGAGATTCTGATGGGTTAAATTGAAACATGGCAGGTTAGAATGAATCA

GACAGACCTATTTGGTTTTGTTTTATGTGACCCCTGAATTGGGTTATGAGTACAATTAGA

AACC

>5432 len=124 tot-seq=1

GACAGGGTGGTCACGCTCACCTGCGTGATCGTTTCAAGGAACGGGCGCAGATAGGTGAAG

ACGGCGAACTGGCCCATGAACAACAGCATGATTGCGAGCATTCCAATTGCGACCTGAGAA

TTGC

>5433 len=124 tot-seq=1

AGGAGAGGGAAGGAGGGAGAGTGAAACAATAATTCTTGGTAGTAACACAGTCATTCGTTA

AGCATTTACTCAGTGCTGGACGTGGAGCTAAGCACATCCCAGCCCTCAGCTCTTTTAAAA

AGCT

>5434 len=124 tot-seq=1

GTAACAGGCATTGTTATCTAGCATTTGACGGTGCTCATTTCAGACAAAGAGAATGAGGCT

CAGATAAATTAAGTAACTTTCTTAAATCACAGAGCCACTAAGTAATGAAGTGTGAATTCA

AACC

>5435 len=124 tot-seq=1

CCCTTATTTATAAAAAGAGAAAATAGCGACACACACATACACACACACTCACGGAGAATG

CCACGTGACAATGGAGACAGGGATTGCAGTGATGTTGCTACAAGCCAAGAAACACCCAGG

ATCG

>5436 len=124 tot-seq=1

AACCAGCCAAGTAGCGTGCAGGACGACGGCCCTATGGGTTGTAAACTGCTTTTATGCGGT

GATAACCGGCATCACGTGTGAGGCCATGCAGGTACCGCATGAATAAGGACCGGCTAACTC

CGTG

>5437 len=124 tot-seq=1

TGGGGAATCTTACACAATGGACGCACGCCTGATGGAGCAACGCCGCATGAGTGAAGAAGG

GTTTCGGATCGTAAAGCTCTGTTGTTGGTGAAGAAGGATAGCGGTAGTAACTGGCCTTTA

TTTG

>5438 len=124 tot-seq=1

GAACCAGCCAAGCCGCGTGAGGGATGAAGGTACAGAGTATCGTAAACCTCTTTTGTCAGG

GGACAAAGGCGGGATGTGTCCCCGGATGAGTGTACCTGAAGAAAAAGCATCGGCTAACTC

CGTG

>5439 len=124 tot-seq=1

GCAACGCCGCGTGAGTGAAGAAGTGTCTCGGCTCGGAAAGCTCTGTTTGTAGTGAAGAAA

GATAGAGGGAGGAACGTGCCTTTATTTGACGGTAATTACTGAGAAAGGCACGGCTAACTA

CGTG

>5440 len=125 tot-seq=1

GAACCAGCCAAGCCGCGTGAGGAAGGAAGGTACAGAGTATCGTAAACCTCTTTTGCGAGG

GAACAAAGACTGGGACGCGTCCCCGGATGAGTGTACCTGAAGAAAAAGCATCGGCTAACT

CCGTG

>5441 len=125 tot-seq=1

CAATGGGGACTCGGTGGCCTGAGTAACCGCCTGGCTGCTGCCCAGGCAGCCGGCTGTAGA

AGAGCTTGCCCAGCGCTCCTGATCCTCAGCTTAGTGGTCCCGGATCTGAAGCAGGGGCAT

CACAG

>5442 len=125 tot-seq=1

CAGGGCATTCTGGGACTGTAGGAGGCAGTTTCTGAAGTAAAAGCTTGGCCATGGGCTTGG

TGCTGTGAGCCAGCCAAGAAGAGTCTGGTTATCAAAACCTCAGCAGGCTTCCTACGTGTG

AAGTG

>5443 len=125 tot-seq=1

TCGGTCGGAGCTGCAGGTGTCGCCCAGGCTTTGTTCGCCCAGGGCCTGGCCTGATGCTGG

AGAGGCAGCTGGCAGGAGCCCTTGCCTGCCCGGAGATGACCCCTCCCATCGAGTCCATAG

AAGTG

>5444 len=125 tot-seq=1

AGCATGAGCTCTGGGTACCTTGGGCATGAACTGGGGGGAAAGTCCCACAGTCCCCAGAGG

ACTCTCCACTCGATCTTAGGATCACTGGAGAGTGGAAAACAACATCTGTTCCAATTGTGA

CAGGC

>5445 len=125 tot-seq=1

AGCAACGCCGCGTGAGTGAAGAAGGGTTTCGGCTCTTAAAGCTCTGTTGTTGTTGAAGAA

GGACAGAGGTAGTAACTGACCTTTTCTTGACGGTAATCAATTAGAAAGTCACGGCTAACT

ACGTG

>5446 len=125 tot-seq=1

GAAGCATTTCCACTCTTGTTTTTTTTTTTGTTTGTTTGTTTTTTTCTGACACTTTCTTTT

CCCTTGGCTTCTCTGACAATTGGAGATGCAAGAGTGGATACAGCCATACCAATGAGAAGG

TGATT

>5447 len=125 tot-seq=1

CAGCGACGCCGCGTGATTTAGAAGGCCTTCGGATCGTAAAGCTCTGTTGTTGGTGAAGAA

GGATAGAGGTAGTAACTGGCCTTTATTTGACGGTAATCAACCAGAAAGTCACGGCTAACT

ACGTG

>5448 len=125 tot-seq=1

AGCACCGCCGCTTGAGTGAAGAAGGGTTTCGGCTCGTAAAACTCTGTTGTTGGTGAAGAA

GTACAGGGGTAGTAACTGCCCTTTGTTTGACGGTAATCAATTAGAAAGTCACGGCTAACT

ACGTG

>5449 len=125 tot-seq=1

AGCAACGCCGCGTGAGTGAAGAAGGTTTTCGGATCTGAAAGCTCGGTTGTTGGTGAAGAA

GGATAGAGGTATTAAATGGCCTTTATTTGACGGTAATCAACCAGAAAGTCACGGCTAACT

ACGTG

>5450 len=125 tot-seq=1

CAATGGGCGCAAGCCTGATGGAGCAACACCGCGTGAGTGAAGAAGGGTTTCGGCTCGTAA

AACTCTGTTGTTGAAGAAGAACGTGCGTGAGAGTACCTTTCGAATAAGCGCCGGCTAACT

ACGTG

>5451 len=125 tot-seq=1

GAGAATCGCTTGAACCAGGTAGGCAGAGGTTGCAGTGAGCTGAAATTGCCCCACTGCATT

CCAGCCTGGGTGACAGAGAAAGACCCTGTCTTAAAAATAACAATAATAATAATTCATAAG

AAAAC

>5452 len=125 tot-seq=1

AATGGGCGAAAGCCTGATGCAGCGACGCCGCGTGAGGGATGAAGGAATTAGGGTTGTAAA

AATATTTAAGAAGGGAAGAAGAGAAAGTGACGGTACCTGCAGAAGAAGCACCGGCTAACT

ACGTG

>5453 len=125 tot-seq=1

GGACTCGGTGGGAGGGGGTCCTAGGGAACAAAGAGAAATGGATCCGGAGCCGAGGACTCA

CCCGCAGCTCCACCCGCACCCAGCTGTTAGAACCGTTACAAGATTCCAGAAGGACCGAGT

CTTTG

>5454 len=125 tot-seq=1

AGCCATGCCGCGTGTATGAAGAAGGCCTTCGGGTTGTAAAGGACTTTCAGCGGGGAGGAA

GGGAGTAAAGTGTATAACTTGGCTCATTGACGTTACCCGCAGAAGAAGCACCGGCTAACT

CCGTG

>5455 len=125 tot-seq=1

GCAATGCCGCGTGAAGGAAGAAGGTCCTCTGGATTGTAAACTTCTGTTGTAAGGGAAAAA

CGACCTATGTAGGAAATGACAGAGGAGTGATGGTACCTTACGAGAAAGCTCCGGCTAACT

ACGTG

>5456 len=125 tot-seq=1

TGGACAATGGGGGCAACCCTGATCCAGCCATGCCGCGTGAGTGAAGAAGGCCTTCGGGTT

GTAAAGCTCTTTCGGCGGGGACGATGATGACGGTACCCGCATAAGAAGCCCCGGCAAACT

TCGTG

>5457 len=125 tot-seq=1

AGCAAGTTTAGTGCAGTGAGAGCAGCATCATTCCTGAGGAAGAGCCAGTGGCCAACAAAG

GCGAGGAAAAAAAAGACAATGAGAACATTCCAATAGAGACAGAGGAGACACGCCTTGAAG

AAACA

>5458 len=125 tot-seq=1

AAGATAAGCAGAGCCTGGGCAGACCCCTGGCCCCCAGAATGGGACTCTCCCTGGGCTGGC

CAGACTCAGCAAGGAGCAGGCAGGCCCCCTCCTCTTCTGTGGTCCTAGAAATCCCAGGCT

GAGCC

>5459 len=125 tot-seq=1

AGCAACGCCGCGTGAATGAAGAAGGGTTTCGGCTCGTAAAGCTCTGTTGGTGGTGAAGAA

GGACAGGGGTAGTAACTGACCTTTATTTGACGGTAATCAATTAAGAAGTCACGGCTAACT

ACGTG

>5460 len=125 tot-seq=1

GAACTTACAGACCTTAGAACCACCCTCCACTAGACAGTGTGAGACTTGTGCTTCAGGGGC

TCAGAACACACAACATGTTCGTCACTTCAACCCTAAGAACAAGCAAAGACATTGAAAACA

AAGTA

>5461 len=125 tot-seq=1

TGCACAATGGGGGAAACCCTGATGCAGCGACGCCGCGTGAACGAAGAAGGTATTCGTATC

GTAAAGTTCTGTTCTATGGGAAGATAATGACGGTACCATACAAGAAAGCGCCGGCTAAAT

ACGTG

>5462 len=125 tot-seq=1

AGCAGAGGGGCTCCCTCCATGGAGGGCAGAAGTGTGGTCCGGGGTGAGGGACGCAGGCAG

GGGCCCCCTGAGGCAGGCCAGGCTGTCTGCTGGAAAGGTGGGGCCCTCCACACGCTGCCC

ACCCC

>5463 len=125 tot-seq=1

GCGATTGTTTTGATTCCGGAAATAGCTCTGACGTATCAGACGGTCATGCGTTTTTACCGA

AGATTTGGGGAGAGGATTTCCATCATGAACTCCAGGCTTTCGGCAGGGGAACGTTTTGAT

CAGTT

>5464 len=125 tot-seq=1

TGCACAATGGGCGAAAGCCTGATGCAGCGATGCCGCGTGAGCGAAGAAGTATTTCGGTAT

GTAAATCTCTATCAGAAGGGAATAAAATGACTGTACCTTACTAAGAAGCTCCGGCTAAAT

ACGTG

>5465 len=125 tot-seq=1

GAACCAGCCAAGTCGCGTGAGGGAAGACGGCCCTACGGGTTGTAAACCTCTTTTGCCGGG

GAACAAAGGCGGCCACGCGTGGCCGGATGAGCGTACCCGGAGAAAAAGCATCGGCTAACT

CCGTG

>5466 len=125 tot-seq=1

GCAACGCCGCGTGAGTGAAGAAGGTTTTCGGAGCGTAAAGCTCTGTTGTAAGAGAAGAAC

TTGTGTGAGAGTGGAAAGTTCACGGGGTGACGGTAACTTACCAGAAAGGGACGGCTAACT

ACGTG

>5467 len=125 tot-seq=1

CGGGGAGTATTGCACAATGGGGGAAACCCTGATGCAGCGACGCCGCGTGAGTGAAGAAGT

TATTCGTAACGTAAAGCTCTATCAGCAGGGAAGAAAATGACGGTACCTGACTAAGAAGCC

CCGGC

>5468 len=125 tot-seq=1

TGGCTATGAGGAAGCAGAGAAAATTAATTTGGTAGAGACAGCATTAGAGAGTGCCCTCCA

AGAAGAAGGGTCTTGAGGAGGAAAAGAAATAGCCAGCAGCCGGGTAATCACAAGATCGGA

AGAGC

>5469 len=126 tot-seq=1

GTGGGAGGAAAACTTCCAAGAGACCAGCATTGCGTGGGGAGTGTCCTGGGCGGTGGGTAA

GGCAGGAGCAGGTTAGCAAAGCCTGAGAGAGACAAGCAGCGGCTTCAGCTGGGCCGGGCT

GGGGGT

>5470 len=126 tot-seq=1

TGGGGAATATTGGGCAATGGGCGCAAGCCTGACCCAGCAACGCCGCGTGGAGGAAGAAGG

TCTTCGGATTGTAAACTCCTGTTGTTGAGGAAGATAATGACGGTACTCAACAAGGAAGTG

ACGGCT

>5471 len=126 tot-seq=1

CTGACAACTGGGTAACATTTCCTCAAGCTCCAGAGCTTTCTCCAGAAAACAAGCATCAGT

AAACTCCACATGACAGAGAATGCCAGGGTTCTTCAATCCCATATCCTCCCAGGGAGGACT

GAGACC

>5472 len=126 tot-seq=1

TGAGGAATAGGGGTCAATGGAGGAAAGTCTGAACCAGACAAGTAGCGGGCAGGATGACGG

CCCTATGGGGTGTAAACTGCTTTTATATGGGGATAAAGTGGGGAACGTGTTCCCTTTTGC

AGGTAC

>5473 len=126 tot-seq=1

TGGGGAATGGGGCACAATGGGGGAAACCCTGATGAAGAGACGCTGCGGGATTTAGAAGGC

CTTCGGGTTGTAAAAATCTTTTGTATGGGAAGAAAATGACAGTACCATACGAATAAGGAC

CGGCTA

>5474 len=126 tot-seq=1

TAGGGAATCTTCCACAATGGGCGAAAGCCTGATGGAGCAACGCCGCGTGAACGATGAAGG

TCTTCGGATCGTAAAGCTCTGTTGCAGGAGAAGATAATGACGGTACCCTGCTAGGAAGCC

CCGGCT

>5475 len=126 tot-seq=1

GAGCAACGCCGCGTGAGTGATGAAGGGTTTCGGCTCGTAAAACTCTGTTGTCAGGGAAGA

ACGTCCCCGTGAGTAACTGCACGGGGAGTGACGGTACCTGACCAGAAAGCCACGGCTAAC

TACGTG

>5476 len=126 tot-seq=1

TTCCCCCTCCCAGGACTTCCTATACCTCACACTCCTGGAGTGCAATGCCTTATCTCAGAC

AATAGTGAGCTCAATATGCCTGTGCTATGTCAGGGTATGGCACCATCAGCTACCCAGTTG

TCCAAA

>5477 len=126 tot-seq=1

GAGCAACGCCGCGTGAGTGAAGAAGGATTTCGGTTCGTAAAACTATGTTGTTAGAGAAGA

AAAGAGAATAGAGTAAATGTTATGCGTGTGACGGTATCTAACCAGAAAGCCACGGCTAAC

TACGTG

>5478 len=126 tot-seq=1

GAGCAACGCCGCGTGAGTGAAGAAGGGTTTCGGGTCGTAAAACTCTGTTAGTAGTGAAGA

AAGATAGTAGTAGTAACTGGCCTTTATTTGACGGTAATTACTTAGAAAGTCACGGCTAAC

TACGTG

>5479 len=126 tot-seq=1

TGAACCAGCCAAGTAGCGTGAAGGATGACGGCCCTATGGGTTGTAAACTGCTTTTATACG

GGGATAAAGTGCGTGACGTGTCATGCATTGCAGGTACCTTATGAATAAGGACCGGCTAAC

TCCGTG

>5480 len=126 tot-seq=1

CAGCCAAGTCGCGTGAGGGAAGACGGGTTTCGGCTCGTAAAGCTCTGTTGGTAGTGAAGA

AAGATAGAGGTAGTAACTGGCCTTTATTTGACGGTAATTACTTAGAAAGTCACGGCTAAC

TACGTG

>5481 len=126 tot-seq=1

GACCTGGGCTTGTGCCCTTGTCACCTTCTGTATGAAGCCAGGAGGATGAAGCTTGATGAT

ATCTCCTGTAGCGATTGAAGGGGAGTTGACAGGTATAAATTGGCCTGAGCAGGAATCTCA

CTATTT

>5482 len=126 tot-seq=1

TGGGCAATGGGGGAAACCCTGACCCAGCGACGCCGCGTGAGTGAAGAAGTATTTCGGTAT

GTAAAGCTCTATCAGCAGGAACGAGAAAAGACGGTACCTGACTAAGAAGCCCCGGCTAAC

TACGTG

>5483 len=126 tot-seq=1

GCTGTGACCGTCTGTTCTGCCGGGAGGCAGGCCTCATTCTCGATGAGGTAAAACCCCAGG

CCATGGAGACGGCCATGGAGCCAGGGACAGCACCAACTCAAGACTCACGTAAGAGACTAG

CATCCT

>5484 len=126 tot-seq=1

GCAAAGAGAGAATGAGAGAAAAGTGAAAGCGGAAACCCCTTATAAAACCATCAGATCTCA

TGAGATTTATTCACTACCATGAGAACAGTATGGGGGAAACCACCCCCATGATTCAATTAT

CTCCCA

>5485 len=126 tot-seq=1

GGCTCACGACATGTAGGCATTCAAAGATCACCTCCAGGCACCCCACAGGCCCTTGTGAAT

TTCCACTAAAAAGCAACCTTTGTGCCATTTGCTCAGGACTCTGCAGGTCCAAGGGATTGT

TTAGAA

>5486 len=126 tot-seq=1

GTTTGGGGTGGGGCTGGGTATCTGATGTTTACTCAGACCTCCTCATCCTCATCCTGCAGG

TGGGCAGATGCTTGGTTGAGGGAAGAGTGCCCCCACCTCGCCCCTCAACTTTCTAGGCAG

AATCAT

>5487 len=126 tot-seq=1

CAGCGACGCCGCGTGCGGGATGAAGGATTTCGGTTCGTAAAACTCTGTTGTTAGAGAAGA

ACAGCGCATAGAGTAACTGTTATGCGTGTGACGGTATCTTACCAGAAAGGGACGGCTAAC

TACGTG

>5488 len=126 tot-seq=1

GGGCACAAGGGGGGAAACCCGGAGGCAGCGACGCCGCGTGAGCGAAGAAGGCTTGCGAGG

CGGAAAGCTCTGTCCTATGAGAAGATAATGACGGTATCATAGGAGGAAGCCCTGGCTAAA

TACGTG

>5489 len=126 tot-seq=1

TGACGAACAGCAGCACGGTGATCAGCGCATCGCTCGCCAGATCGTAAAAGTGTCCGATCT

TGCTCGATTTGCCGCTAATTCTGGCTAATTCGCCATCGGTATGGTCAACGAAATTCGAGA

TCACGA

>5490 len=126 tot-seq=1

CAATGGAGATGGGAGGAGAGCCAGAGGATGGCATGCAGGCTTAATCCTGCAGAGGCTCTG

CAGTGCGGGCGAGGCCTGGGGCTTGGTCTCACGTCTCCACGGGCTCGAAGGGAAACTCTC

CTGATG

>5491 len=126 tot-seq=1

TGAACCAGCCAAGTAGCGTGCAGGACGACGGCCCGATGGGTGGTAAACTGCTTTTATGTG

GGGATAAAGTGCGGGACGTGTCATGCAGTGCAGGTACCACATGAATAAGGACCGGCTAAT

TCCGTG

>5492 len=126 tot-seq=1

GAGCAACGCCGCGTGAGTGAAGAAGGGTTTCGGCTCGTAAAGCTCTGTTGTTGGAGAAGA

ACAAGCTTGATAGTAACTGTTCACCCAGTGACGGTATCTAACCAGAAAGTCACGGCTAAC

TACGTG

>5493 len=126 tot-seq=1

CCCTGACGCAGCGACGCCGCGTGCGGGATGAAGGCCTTCGGTATGTAAAGCTCTATCAGC

AGGGAAGAAAGGTGCCCCTTGGGCGCCTGGACGGTACCTGACTAAGAAGCCCCGGCTAAC

TACGTG

>5494 len=126 tot-seq=1

GAGCAACGCCGCGTGAGTGAAGAAGGGTTTCGGCTCGTAAAGCTCTGTTGTTAGTGAAGA

AAAATAGAGATAGTAACTGGTCGTAATTTGACGGTAATTACTTAGAAAGGCACGGCTAAC

TACGTG

>5495 len=126 tot-seq=1

CAGTGTTCTGGAATCCTGTGTGAGGAACAATCATTCAGACACTCGTAGCTGTGTTCTGGA

ATTCCGTGTGATGGACGAACATTCACACCCTCGGAGCAGTGTTCTGGAATCCTATAAAGG

GACAAA

>5496 len=127 tot-seq=1

AGAGTCCAGGGTGGCAGAGGAATATCTATGGCCTGGAGGCTTGGCACAAGGACTCTGGAA

TCCACTGGTGCCAGATTGGACAAGTTATGCAACACCCGGGCTGAGTTCATTTTTTTGTAA

AGTAGGG

>5497 len=127 tot-seq=1

GCAGCCATGCCGCGTGTATGAAGAAGTCCTTCGGGTTGTAAATTACTTTCAACCCGGAGG

AAGATAGTCAAGTTAATACCTTTGCTCATTGACGTTACCCGCAGAAGAAGCACCGGCTAA

CTCCGTG

>5498 len=127 tot-seq=1

GTTGATCCACGCCCAAACACTTGACAGATACACCAGCAGTCCAATTTGGTAGAGCTGGGA

TAGCAAACACGAATCAGCAGCCGAGGCACTGACTAGCAGAGCCAACTCGGACTCAGCCTC

AGCAGTG

>5499 len=127 tot-seq=1

GGAGCAACGCCGCGTGAGGGAAGAAGGGTTGCGGCTCGTAAAGCTCTGTTGTTGGGGAAG

AAGGACAGGGGGAGGAACTGACCGGGGGGTGACGGTAATCAATGAGAAAGTCACGGCTAA

CTACGTG

>5500 len=127 tot-seq=1

CCAGCAATGCCGCGTGAGTGAAGAAGGCCTTCAGGTTGTAAAGCTCTTTTGTCAGGGAAG

AAACGGCTGAGGTTAATACCTTCGGCTAATGACGGTACCTGAAGAATAAGCACCGGCTAA

CTACGTG

>5501 len=127 tot-seq=1

GATGGAGCAACGCCGCGTGAGTGAAGGGTTTCGGCTCGTAAAGCTCTGTTGTTGGTGAAG

AAGGACAGGGGTAGTAACTGGCCTTTGTTTGGCGGTAATCAATTAGAAAGTCACGGCTAA

CTACGTG

>5502 len=127 tot-seq=1

GGAGCAACGCCGCGTGAGTGAAGAAGGGTTTCGGCTCGTAAAGCTCTGTTGTTAGAGGAG

AACGTGCGGGAGAGCAACTGTTCACGGAGTGACGGTAACTAACCAGAAAGTCACGGCTAA

CTACGTG

>5503 len=127 tot-seq=1

GAGTTTTTTTTTTTTTCGTGAGGATGAAGCGTGAGTTGATGTACATAGAACCAGGGTAGG

TCTCTGATAAGGCAGGTGAGTGGCTTGTGTGGAGCATCATACTCCAGCCCACACCTGGCA

GTGGGCA

>5504 len=127 tot-seq=1

GGGCAATGGAGGCAACTCTGACCCAGCAACGCCGCGTGAAGGATGAAGGTTCTCGGATTG

TAAACTTCTGTATTAGAGGAAGAAGATAGTGACGGTACTCTAATAGAAAGCTACGGCAAA

CTACGTG

>5505 len=127 tot-seq=1

TAGGGAATCTTCCACAATGGACGCAAGTCTGATGGAGCAACGCCGCGTGAGTGAAGAAGG

GTTTCGGCTCGTAGTAACTGACCTTTGTTTGACGGTAATCAATTAGAAAGTCACGGCTAA

CTACGTG

>5506 len=127 tot-seq=1

GGAGCAACGCCGCGTGAGTGAAGAAGGGTTTCGGCTCGTAAACCTCTGTTATAAGTGAAG

AAGGGCGGGGGTAGTAACTGACCTTTGTTTGACGGTAATCAATTAGAAAGTCACGGCTAA

CTACGTG

>5507 len=127 tot-seq=1

TGGGGAATATTGCACAATGGGCGAAAGCCTGACGCCGCGTGAGCGAAGAAGTATTTCGGT

ATGTAAAGCTCTATCAGAAGGGAAGAAAATGACGGTACCTTACTAAGAAGCTCCGGCTAA

ATACGTG

>5508 len=127 tot-seq=1

CTGAACCAGCCAAGCCGCGTGAAGGATGAAGGTACAGAGTATCGTAAACCTCTTTTGTCA

GGGAACAAAGGCGGGGACGCGTCCCCGGATGAGTGTACCGTAAGAAAAAGCATCGGCTAA

CTCCGTG

>5509 len=127 tot-seq=1

TCCCAGCGGTCCTAGGCTGTCGGGAAACTGCAGCCCAGCCTCAGGGCCATGGGAGGTGGT

ATCCAGGCCTGGGTGGGGCCAGGCCTCTGCTCACAGGGATGTGGGGTCAGTGCTTGGGAG

CCTGTGG

>5510 len=127 tot-seq=1

TGGACCCGATCCCTCGTTCTTGGTTCACCTGGGTAGCAACCGCAGCTGCTTCTCAAAGGT

AAGCCCTCAATCTTGACCACCAAAAAGCATCAAGGAGCTGAAGCCCAAGGAAATGCAGTC

TGCTATG

>5511 len=127 tot-seq=1

CAGATTTCTGGAATCCTATGTGAGGGACAAAGATTGAGAACCCCGTAGAAGTGTTCTGAA

ATCATATGTGAGGGACAAACTTCCAGACCCCAGCAGCGGTAATACACAGATCGGAAGAGC

GTCGTGT

>5512 len=127 tot-seq=1

GGAGCAACGCCGCGTGAATGAAGAAGGGTTTCGGCTCGTAAAGCTCTGTTATTAGTGAGG

AAGGACAAGAGTAGTAACTGACCTTTGTTTGACGGTAATCAATTAGAAAGTCACGGCTAA

CTACGTG

>5513 len=127 tot-seq=1

GGAGAAAAGAAGAGTGAGTGAAGAAGGAATTAGGGTTGTAAAGATATGTGATTAGGGAAG

AAAGGATATATGTGAATAATATATGGAAATGACGGTACCGAAAAAGCAAGCCACGGCTAA

CTACGTG

>5514 len=127 tot-seq=1

TAACCTCGGCAGACTTAAATGTCCCTGTCTGACAGCTTTGAGGAGAGCAGTGGTTCTCTC

AGCATGCAGCTGGAGATCTGAGAATGGGCAGACTGCCTCCTCAAGTGGATCCCTGACCCC

TGATCCC

>5515 len=127 tot-seq=1

CCTGTAGGTCGTGTCTAAAAACATGAGCGTGGATGAGCTCTTCCAGGGATGAGTGAGGAT

AAGAAGAGGAGGCACATTGAGTCCTGGAGCTGTTGCTGTGTGTAGAGCTTGAAGAGATGA

GGAAGAA

>5516 len=127 tot-seq=1

ATTGCACAATGGGGGAAACCCTGATGCAGCGACGCCGCGTGAGCGAAGAAGGCTCTCGAG

TCGTAAAGCTCTATCCTATGAGAAGAAAATGACGGTATCATAGGAAGAAGCCCTGGCTAA

ATACGTG

>5517 len=127 tot-seq=1

GGAGGCCCCAGCATTGTATGCCAACCAATGGGAGCTGCAGACAACCAATCTCCTACCTAC

CTTTAAGTCTGACCTACATTTGTCCAGAGTATCTACACCTCTCCCATATCTTCTGAAACA

ATGAAGC

>5518 len=127 tot-seq=1

GGACAGGCATTATGAGGCATTGGGGGGAGCTGTGGGGCTGGACATTGTGAGTCACTAGCC

TGACCTGGCTGTTTCCTGGTAACCAGGTGACATAGGAAGCTTTGTGGGTTATAAGCATCA

GCCAGGG

>5519 len=127 tot-seq=1

CTGAACCAGCCAAGTAGCGTGCAGGATGACTGCCCTATGGGTTGTAAACTTCTTTTATAT

GGGAATAAAGTGGGAAACGTGTGGATTTTTGCATGTACCATATGAATAAGGACCGGCTAA

TTCCGTG

>5520 len=127 tot-seq=1

CTGAACAAGACAAGTAGCGTGAAGGATAACGGCCCTATGGGTTGTAAACTGCTTTTATAT

GGGGATAAAGTGGGGAACGTGTTCTCTTTTGCAGGTACCATATGAATAAGGACCGGCTAA

TTCCGTG

>5521 len=127 tot-seq=1

ATTGCACAACGGGGGAAACCCTGATGCAGCGACGCCGCGTGGACGATGAAGTATTTCGGT

ATGTAAAGCTCTATCAGCAGGGAAGAAAATGACGGTACCTGACTAAGAAGCCCCGGCTAA

ATACGTG

>5522 len=127 tot-seq=1

CTGAACCAGCCAAGTAGCGTGCAGGATGACGGCACTATGGGTGGTAAACTGCTTTTTTTT

GGGAATAAAGGGCATTACGTGTAATGTTTTGCATGTACCATTCGAATAAGGACCGGCTAA

TTCCGTG

>5523 len=127 tot-seq=1

CTGAACCAGCCAAGTAGCGTGCAGGATGGCGGCACTATGGGTTGTAAACTGCTTTTGTAT

GGGAATAATCTGCAGTACGTGTAATGTTTTGCATGTACCATTCGAATAAGGACCGGCTAA

TTCCGTG

>5524 len=127 tot-seq=1

CTGAACCAGCCAAGTAGCGTGCAGGATGAAGGCCCTATGGGTTGTAAACTTCTTTTATTT

GGGAATAAACTACAGTATGTGTAATGTTTTGCATGTATCATACGAATAAGGACCGGCTAA

TTCCGTG

>5525 len=127 tot-seq=1

GGAGCAACGCCGCGTGAGTGAAGAAGGTTCTCGGGTCGTAAAGCTCTGTTGTTGGTGAAG

AAGGATAGATGTAGTAACGGGCCTTTATTTGACGGTAATCATCGAGAAAGTCACGGCTAA

CTACGTG

>5526 len=127 tot-seq=1

GGAGCAACGCCGCGTGAGTGAAGAAGGTTTTCGGATCGTAAAACTCTGTTGTTGGTGAAG

AACGATAGAGATAGTAACTGTCCTTTATTTGACGGTAACCAACCAGAAAGTCACGGCTAA

CTACGTG

>5527 len=127 tot-seq=1

CTGAACCAGCCAAGTAGCGTGAAGGATGAAGGTTCTATGGATTGTAAACTTCTTTTATAC

GGGAATAAACGGATGCACGTGTGGATTGGTGCATGTACCTTATGAATAAGGATCGGCTAA

CTCCGTG

>5528 len=127 tot-seq=1

TCAAGCAGAAGACGGCATACGAGATTCACGAGTGACTGGAGTTCAGACGTTGCTCTTCCG

ATCTCCTTCGGGAGGCAGCAGAGCATACGAGATTCACGAGTGACTGGAGTTCAGACGTGT

GCTCTTC

>5529 len=128 tot-seq=1

GCAGAGGAGGGAGGGGAGCACGGGAAAGTGCAAGGAGCCGGGGAATCCTGTGCCGCCCTA

GACCACAGCACAAGGCCTTTTGGCCTGAGGCCCCTACAACCCACAGCTCTCACGCTGCCT

GCTGCTCA

>5530 len=128 tot-seq=1

TGCAGCGACGCCGCGTGAGTGAAGAAGTATTTCGGTATGTAAAGGACTTTTGTCCGGGAA

GAGAAGCGCGATGTTAATACCATTGCGTGCTGACGGTACCGGAAGAATAAGCACCGGCTA

ACTACGTG

>5531 len=128 tot-seq=1

TGCCATCCCGGTTTCATCTCTTTTGGAGATGTTATCTAGTAGCAGGATTGCTTGTTCATA

CCGAAGTTCTGTTTTTAAGTTCTAAAGGCATCTCAATATTTTATCCACAATTGGGATATT

CATTTGCA

>5532 len=128 tot-seq=1

CAACTTACATGGTGAGCACAAAGGGTTGGCAGAATAGAAGCTTGGGAAAAGCATCCCTTC

TGCAGCCATCCGGTCTAAATTTGGGGTCTCTCTGGAAGGTTCTCCATTCACACCCAAGTC

ACCCCACC

>5533 len=128 tot-seq=1

CCAGCCAGGCCGCGTGTGTGAAGAAGGCCTTATGGTTGTAAAGCACTTTAAGCGAAGAGG

AGGCTACTTTAGTGAATGCCTAGAGATAGTGGATGTTACTCGCAGAATAAGCACCGGCTA

ACTCTGTG

>5534 len=128 tot-seq=1

CATGACTGAGGTGGTGGTATATATGTTTATGTTGGCTGTTTCATTCCCCTAAATGCATTT

GACATCTCTAATTAATCCCTTTGGAGCAGTGGCTGCTGGAATTCTCAGCTCAGAACCTTG

GCTCATGC

>5535 len=128 tot-seq=1

TGGAGCAACGCCGCGTGAGTGAAGAAGGGTTTCGGCTCGTAAAGCTCTGTTGTTGGTGAA

GAAGGGCAGGAGGAGTAACATACCTTTATTTGACGGTAATCAATTAGAAAGTCACGGCTA

ACTACATG

>5536 len=128 tot-seq=1

TGGGGCATATTGCACAATGGGCGAAAGCCTGATGCAGCGACACCGCGTGAGTGAAGAAGT

ATTTCGGTATGTAAAGCTCTATCAGCAGGGAAGAAAATGACGGTACCTGACTAAGAAGCC

CCGGTTTA

>5537 len=128 tot-seq=1

ACTGAACCAGCCAAGTAGCGTGAGGGATGACTGCCCTATGGGTTGTAAACCTCTTTTATA

TGGGAATAAAAAAGATTACGTGTAGTCTATTGTATGTACCTTATGAATAAGCATCGGCTA

ATTCCGTG

>5538 len=128 tot-seq=1

TTTGAGACCAGCCTGGCCAATATGGTGAAACTCTGTCTCTACTAAAAATGCAAAAATTAG

CTGTGCATGGTTGTGGGTGCCTGCAATCTCAGCTACTCGGGAGGCTGAAGCAGGAGAATC

GCTTGAAC

>5539 len=128 tot-seq=1

TGACACCAGCCAGTCTGACAGGTCAGAGGATGAGGGTGACTACCTGCCCAGCCAGTGTAA

TGCTACTTAGAGCCCCAAGTCCTGGGACAGGTGAGATCAGGAAACAGAAAGTGCAAAACC

ACCCCGAC

>5540 len=128 tot-seq=1

TGGAGCAACGCCGCGTGAGTGAAGAAGGTTTTAGGATAGTAAAGATATGTTGTTGGTGAA

GAAGGAGAGGGGTAGTAAATGAAATTTGTTTGAAGGTAATCAACTAGAAAGTCACGGCTA

ACTACGTG

>5541 len=128 tot-seq=1

TAGGGAATTTTTCACAATGGGCGCAAGCCTTATGAAGCAATGCCGCGTGAACGATGAAGG

TCTTATAGATCGTAAGAGCGTCGTGTAGGGAAAGAGTGTGCCAATGTGTAGATCTCGGTG

GTCGCCGT

>5542 len=128 tot-seq=1

CAGACCATCGGCCATTTGATAGAAGGGCAATGTCTCCGACTGCATGCCGAAGAAGCGCAC

CCGCGCTTCACAGTTCAGGCTCTTCGCCAGCGCCTGATAGCGAGGCTGATCCTTATCTTT

GCCGACCA

>5543 len=128 tot-seq=1

GCAGGAGGAGGAGAAAGAGGAGGAGGAGGAGCAGGAGGAGAAGGAGCAGGAGGAGAAGGA

GCAGGAGGAGGAGGAGCAGGAGGAAAAGGAACAGCAGGAGCAGGGCCAGACAGCCAGGGC

CCCTAGAC

>5544 len=128 tot-seq=1

TAGGGAATCTTCCACAATGGGCGCAAGCCTGATGGAGCAACACCGCGTGAGTGAAGAAGG

GTTTCGGCTCGTAAAACTCTGTTGTTGAAGAAGAACGTGCGTGAGAGGAAGCCCCGGCTA

ACTACGTG

>5545 len=128 tot-seq=1

GGGAGCAACGCCGCGGGAGGGAAGAAGGGGTTCGGATCGTAAAGCTCTGTTGTTGGTGAA

GAAGGATAGAGGTAGTAACTGGCCTTTTTTTGACGGTAATCAACCAGAAAGCCACGGCTA

ACTACGTG

>5546 len=128 tot-seq=1

TGCAGCGACGCCGCGTGAGGGATGGAGGCCTTCGGGTTGTAAAGTACTTTCAGCGGGGAG

GAAGGGAGTAAAGTGAATACCTTTGCTCATTGACGTTACCCGCAGAAGAAGCGCCGGCTA

ACTCCGTG

>5547 len=128 tot-seq=1

CCTGAACCAGCCAAGTAGCGTGAAGGATGACTGCCCTATGGGTTGTAAACTGCTTTTATA

TGGGGATAAAGTGCGTGACGTGTCATGCATTGCAGGTACCAAATAAATAAGGACCGGCTA

ATTCCGTG

>5548 len=128 tot-seq=1

TGGCTATGAGGAAGCAGAGAAAACTAATTTGGTAGAGACAGCATTAGAGAGTGCCCTCCA

AGAAGAAGGGTCTTGGGGAGGAAAAGAAATAGCCAGCAGGCGGTAATCCCCAGATCGGAA

GAGCGTCG

>5549 len=128 tot-seq=1

CCTGAACCAGCCAAGTAGCGTGCAGGATGACGGCCCTATGGGTTGTAAACTGCTTTTATG

TGGGGATAAAGTAGGTCACGTGTCATCTATTGCAGGTACCGCATGAATAAGGACCGGCTA

ATTCCGTG

>5550 len=128 tot-seq=1

TCTGAACCAGCCAAGTAGCGTGCAGGATGGCGGCCCTATGGGTTTTAAACTGCTTTTATA

TGGTGATAAAGTGGGGAACGTGTTCCCCCTTGCAGGTACCATATGAATAAGGACCGGCTA

ATTCCGTG

>5551 len=128 tot-seq=1

GAGGGGGAAGAAAGCACTAGGTGGCTAAGGGACTCTGGGCTCATTGACAGCCGGACAGCG

AAAGGAACAGGGGCTGGTTCGCAGCTTGAAAGATGGAGGCGGGAAGAGAAAGAGGGGGAA

GGTGGAAG

>5552 len=128 tot-seq=1

TGCAGCCATGCTGCGGGTAGGAAGAAGGCCTTCGGGTTGGAAAGTACTTTCAGCGGGGAG

GAAGGGAGTAAAGTTAATACCTTTGCTCCTTGACGTTACCCGCAGAAGCTGCACCGGCTA

ACCCCGTG

>5553 len=128 tot-seq=1

CCAGCAACGCCGCGTGAGGGATGACGGTTTTCGGATCGTAAAACTCTGTTGTTAGAGAAG

AACAAGGATGAGAAGAGAATGTTCATCCCTTGACGGTATCTAACCAGAAAGCCACGGCTA

ACTACGTG

>5554 len=128 tot-seq=1

CAGCAGCAGCAGCAGCAGCAGCAGCAGCAACAGCAACAGCAGCAGCAGCAGCAGCAGCAG

CAGCAGCAGCAGCAGCAGCAGCAGCAGCAACAGGCAGTGGCAGCTGCAGCCGTTCAGCAG

TCAACGTC

>5555 len=128 tot-seq=1

TATTGCACAATGGGGGAAACCCGGATGCAGCGACGCCGCGTGAGTGAAGAAGTATTTCGG

TATGTAAAGCTTTATCAGCAGGGAAGAAAGTGACGGTACCTGAAGAAGAAGCGCCGGCTA

ACTACGTG

>5556 len=128 tot-seq=1

TCAAGCAGAAGACGGCATACGAGATAAGTTGGTGACTGGAGTTCAGACGTGTTCTCTTCC

GATCTCCGACGGGAGGCAGCAGCCGGTGACTGAGTTCAGACGTGTGCTCTTCCGATCTCC

TACGGGAG

>5557 len=129 tot-seq=1

GGGAGGAGACCGTAGAGAGGAGAAAATTCAGAAGGACAGGGTCTTGCTAGGGCTGGTGGG

CTCAGGTTGAGAGATACTGACTTGGGAACAGAAAGGGTACATTGTAACTCTGAGCTTATG

TGTCCACAG

>5558 len=129 tot-seq=1

GCATCTGGGGTAGACCTTGCACAGGATAGGGCTGTTCCCTCCTGAAATGACCCTCTCTCT

ATGGGTGCCTTCTCCATATGAGATTGTCAGTACCACCCCAACATACCGTGGTCCAAGGTG

GTCCAGAGC

>5559 len=129 tot-seq=1

GAGAAGGGAGGGGATGCTACAGTTACACGTCATGCTTTAGAGGTTGACTGATTATCATTT

AGAATCACAGTGGAAACAAGTCTCCAAGTATATGTATGAGAGATTATCTAGACCTACATG

AACATCATT

>5560 len=129 tot-seq=1

TGACCCTGTAAAGCCCTATATACAAAGAAACTTTGAAATCAAAGATTATGTACCTGCCCA

AAGGTATAACAACCAAGTGAAGAAAGAGCGTTAACCTTGTCAAATTGTCATTAAGAACCA

AGATACTGT

>5561 len=129 tot-seq=1

TGGGGAATAGTGCACAATGGAGGAAACTCTGATGAAGAGACGCCGCGGGAAGGATGAAGT

ATTTCGGTAGGTAAACTTCTATCAGCAGGGAAGAAAATGACGGTACCTGACTAAGAAGCC

CCGGCTAAC

>5562 len=129 tot-seq=1

TGGAAATCCCAGGAATTGATGGACTAACAAGAGCCAGCGCCGCGGTAATCCTTAGATCGG

AAGAGCGTCGTGTAGGAAAGAGTGTGCCAATGTGTAGATCTCGGTGGTCGCCGTATCATT
AAAAAAAAA
>5563 len=129 tot-seq=1
CAATGGGCGAAAGCCTGATGGTGCGACGCCGCGTGGGGGAGGAAGGTCTTCGGATTGTAA
ACCCCTGTCATGTGGGAGCAAGACATTTGTTTGATAGTACCACAAGAGGAAGAGACGGCA
AACTCTGTG
>5564 len=129 tot-seq=1
CAGATGGCCAGATCAAATGGAGCCAGCAGAACCACAGGGCTCTGGAGCTGGACTCCGTGG
CCACAGGTCATGTGGAGGGAATCTGAATGGCCTGTCAGGCCCCTGCTGCCCTCTTGCTCC
TGATAACAC
>5565 len=129 tot-seq=1
ATGGAGCAACGCCGCGTGAGTGAAGAAGGGTTTCGGCTCGTAAAGCTCTGTTGTTGGTGA
AGAAGAACAAAAAAAATAACTGACCTTTGTTTGACGGTAATCAATTAGAAAGTCACGGCT
AACTACGTG
>5566 len=129 tot-seq=1
CGGAGCAATGCCGCGTGAGTGAAGAAGGCCTTCGGGTTGTAAAACTCTGTCCTTATTGAA
GAGAGGCAGTAGTGTAAATAATGCTATTGTAGGACGGTAAATAAGGAGGAAGCCCCGGCT
AACTACGTG
>5567 len=129 tot-seq=1
TCAGGAATGGATGTGAATGCAGATGGCACCCCTGCTCCCCAGCTCTGTCCTGGCCCTGAG
GTCCTCCTGATGCGCACCCCTCCCTACCTCCCGAATCCGAGTCATGGTAATTGGGGTCCA
GCCCCTTGT
>5568 len=129 tot-seq=1
CAGGTCAATCAGGCCCAAGGTTAGCACCATGGACTTGAGCTGGGCAGCTACAAACTCAAT
CCCTGCAGGGACACAAGCCAAGTCAGAACCCAAAACAGGGCAGCTGCCACAACCTACCCC
TTGTGAGTC
>5569 len=129 tot-seq=1
ATGGAGCAACGCCGCGTGAGTGATGAAGGTTTTCGGTTCGTAAAGCTCTGTTGTTGGTGA
AGAAGGATAGGGGTATTAACTGACCTTTATTTGACGGTAATCAACCAGAAAGTCACGGCT

AACTACGTG

>5570 len=129 tot-seq=1

CCGAGCAACGCCGCGTGAGTGAAGAAGGTTTTCGGATCGTAAAGCTCTGTTGTTAGAGAA

GAACGTTTGTGGGAGTGGAAAGTTCACCCTGTGACGGTAACTTACCAGAAAGGGACGGCT

AACTACGTG

>5571 len=129 tot-seq=1

GAGTGAGAAGGAGGAACTGGAGTGAGAAGCAGGAGCTCCTAACAATACAGGACTCTTTCG

AGGCCCTGTAATTGGAATGAGTCCACTTTAAATCCTTTAACGAGGATCCATTGGAGGGCA

AGTCTGGTG

>5572 len=129 tot-seq=1

CCCTGATGGTGCGACGTCGCGTGAGGGAAGACGGCCCTACGGGTTGTAAACCTCTTTTGT

CAGGGAGCAAGAGGCGGGTCGGGACCTGCTGTGAGAGTACCTGAAGAAAAAGCATCGGCT

AACTCCGTG

>5573 len=129 tot-seq=1

CCGCCACATGATGTCCCTGCTGGTAGGAAGCGCATGCACCATCCTGCTGGGAGCGGGAGC

ATTTTATCTGCTGGATTACATGAAAAAAGCCCCTATGGGCACTACCATCAATACCATCAA

GGAAGCCAT

>5574 len=129 tot-seq=1

CCGCAATGGGCGAAAGCCTGACGGAGCAACGCCGCGTGAGGGATGACGGCCTTCGGGTTG

TAAACCTCTTTCAGCACGGAAGAAGCGAAAGTGACGGTACGTGCAGAAGAAGCGCCGGCT

AACTACGTG

>5575 len=129 tot-seq=1

CCGAGCAACGCCGCGTGAGGGAAGAAGGTGTTCGGATCGTAAAGCTCTGTTGTGAGAGAA

GAACGAGTGTGAGAGTGGAAAGTTCACACTGTGACGGTAGCTTGCCAGAAAGGGACGGCT

AACTACGTG

>5576 len=129 tot-seq=1

TTTTTTCAAGCAGAAGACGGCATACGAGATTTATCCGTGACTGGATTCAGACGTGTGCTC

TTCCGATCTCCTACCGGAGGCAGCAGGGGGAGGAGGCTTGGAGGTGGTGGTGTTGAGTCC

TGTGATCCC

>5577 len=129 tot-seq=1

TAGGGAATCTTCCACAATGGGCGCAAGCCTGATGGAGCAACACCGCGTGAGTGAAGAAGG

GTTTCGGGCGTGAGAGTAACTGTTCACGCAGTGACGGTATCCAACCAGAAAGTCACGGCT

AACTACGTG

>5578 len=129 tot-seq=1

AGCATGACTTCCAGGAGTAGGTCATAAAGGCAATGCAGTTCCCACCTTAGTTTCTTGGAT

GGCTCACTCTGGGGGAACCCAGAGTCCATGCTGTGGGACACTCAGACAGCCCTGGGGAGA

GACCTCCCA

>5579 len=129 tot-seq=1

AAGTGGTTGATAAGCATTATAAGCATTCAAAAAAATGCTCTACTGTCCTCGACACTGATG

AAGAAAAATGCAAATTAAAGTAGAAACAAGATGCTATTCATCAGTGGTTCTTCATGTGTA

GTCCCTGAG

>5580 len=129 tot-seq=1

ATGCATCCGGGTGCAGGGCCGCTGAAGTCTTGCTGTTTGGCAGCTCCAGCAGGGACACCC

CCAGGACAACCCAAAGGCAACAAGAGGTCAGGGAGGGACCTCGCTTGTCTTGTTCACAGC

TGTAGGTGG

>5581 len=129 tot-seq=1

TTTCCCTTAAGTGGGTAGAAAGAGAAAGGGGAGCAAGAGAGACGCAAGGATTGGTGTCCA

TCTCAGAAACCGCCGCAGTCCTGAGACTTGCTAAAATTAGCTGCAATGCAGTTGTGCACA

ACTGCGGGG

>5582 len=129 tot-seq=1

GCCTGAACCAGCCAAGTAGCGTGAAGGATGACTGCCCTATGGGTTGTAAACTTCTTTTAT

AGGGGAATAAAATGAGCCACGTGTGGCTTTTTGTATGTACCCTATGAATAAGGATCGGCT

AACTCCGTG

>5583 len=129 tot-seq=1

GTGAAACCAAGCAGGTTTGCTGGTGCTGATTTTATGTATTCTCATTTCATCAAGAGTTGT

GTGGATGACAAAGAAGAACCTCTACCATACAGGATGTTCTTTTCCTAATCAGCTTTCTCT

GACCATTTG

>5584 len=129 tot-seq=1

TTTTTTTTTTCAAGCAGAAGACGGCATACGAGATTATTCGGTGACTGGAGTTCAGACGTG
TGCTCTTTCGATCTCCTCCGGGAGGCAGCAGCCTGAGGGGGTTTGGTTAGCATCAGGGCT
AGTGCAGAC
>5585 len=129 tot-seq=1
GAGGGAGGGAGGAGGCAGGTGCTAACCACAGATTGGTGAGGAGCAAAGACGCATGCTGGG
ACCCAGGGAAGCCTTTCCAGAGGGGACAAGAGTGGGTGCCCCAATTTCGCTGAAGCTTTG
GAGAGAGAG
>5586 len=129 tot-seq=1
CAGTTTTGAGAAGAAGCCAGACCTCCACGCTCTGCACAACAGCCTGAGCCCGGCCTACGG
TGGTGCTGAGTTTGCCGAGCTGCCCGGTCAGGCTGTTCCCACAGACACCCACCACCCCGC
TTCTCACCA
>5587 len=129 tot-seq=1
GCCTGAACCAGCCAAGTCGCGTGAGGGATGACGGTCCTACGGATTGTAAACCGCTTTAGG
CGGGGAGTAATGTGCGCTACGCGTAGAGTAGTGAGAGTACCCGCAGAATAAGCATCGGCT
AACTCCGTG
>5588 len=129 tot-seq=1
CAAGCAGAAGACGGCATACGAGATCGGTGTGTGACTGGAGTTCAGACGTGTGCTTTCCGA
TCTCCTATGGGAGGCAGCAGGAGAGAAAGAGTCTATTCTTCCCAGGCAATTCAGATACCC
ATCCGTGCT
>5589 len=129 tot-seq=1
CCGAGCAACGCCGCTTGAGTAAAGAAGGTTTTCGGATCGTAAAGCTCTGTTGTAAGAGAA
GAACTTGTGTGAGAGTGGAAAGTTCACACAGTGACTGTAACTTTCCAGAAAGGGACGGCT
AACTACGTG
>5590 len=129 tot-seq=1
CCGTGCCCGGTGCCGTACCAGAAATCACGCAGCCATGCGCTGCGGTTGCCGTCCCCGGCG
GCAATCTTGTCCAAATCGGTTTCCATATCGGCGGTGAAATCGAAATCCACCAGTCCCGGC
AGATTTTCT
>5591 len=130 tot-seq=1
GCATCAGACCCCAGACGGTCTCAGCCTCACAGGCCCTGGCCCAGGCTCTGGTTAAGCACG

AGGGGAGATGCTGGAGGGAGACGCAGGTGATGCCTGGGGCCTACCCCGGGAGCCCCCCTT

CCCCTGGACA

>5592 len=130 tot-seq=1

CATAAGAATTGCTTGAACCTGACAGGCAGAGGTTACAGTGAGCCGAGACTGTGCCACTGC

ACTCCAGCCTGGGTGACAGAACGATTTTCTGTCTCAACATCTGTATGGAGGAGACTGGAG

ATGACTGACC

>5593 len=130 tot-seq=1

GATGGAGCAACGCCGCTTGAGTGAAGAAGGGTTTCGGCTCGTAAAGCTCTGTTGGTAGTG

AAGCAATATAGATGTAGTAACTGGCCTTTATTTGACGGTAACTACTTAGAAAGTCACGGC

TAACTACGTG

>5594 len=130 tot-seq=1

AGAGGACCTGGGGGAGGACGAACAGAATCCGGCATGGAGGACAAGGCGGGGCCCCTCCTG

GGAAGGGAAGGGGAGAGGCCTCCCTGGTTCTGCACTGGCTACAGGCGCGTAGGCGGAGCC

GTTTTACAAA

>5595 len=130 tot-seq=1

TGAGGAATATTGGGCAATGGAGGCAACTCTGACCCAGCCATGCCGCGTGAGTGAGAAGGT

TTTCGAATTGTAACGCTCTTTCGGGTGTGAGGATGATGACGGTAACACCAGAAGAAGCCC

CGGCAAAATT

>5596 len=130 tot-seq=1

TGAGGAATATTGGTCAATGGGCGGAAGCCTGAACCAGCCAAGTAGCGTGAGGGAAGACTG

CCCTATGGGTTGTAAACCTCTTTTGTGCGGGGATAAAGTGTGGGACGTGTCCCATATTGC

AGGTACCGCA

>5597 len=130 tot-seq=1

AATTGGGGAGGGGCCACAAGGTGAAGGAAGCTCCTAGCTGATTTTGTAATAACTTTAACT

GAGCACAAACGTTCCTGAGCAGAATCAGTGGGGTTGGGGATTAACGGGAAGTGCAGATAA

GAACACAGAA

>5598 len=130 tot-seq=1

CTATTTCAGGAAACAAAAATTCATTACACACATCCTTATTGATTTTCCCTCTCCTTTGCT

TATTATTGAAATGCTTTTCCTTCCATCTCTTGAAGAAACTGTGTTGCAATTACTGGAAGC

TTTGCTCACC

>5599 len=130 tot-seq=1

CTACGGGCAGCCCTGATTGTTCGTAGACTCTGTATCTGAGAATCTCGCTTGCTAAGATTT

ATTTGTAATCCCCAAATAAATATCTGCCCTGCTTTCGGCAACAAATTCAAGTCACCTGAC

ACCCACTTTC

>5600 len=130 tot-seq=1

AGCCTGAACCAGCCAAGTAGCGTGCAGGATGACTGCCCTATGGGTTGTAAACTGCCTTTA

TGCGGGGATAAAGTGCGTGACGTGTCATTCATTGCATGTACCGCATGAATAAGGACCGGC

TAATTCCGTG

>5601 len=130 tot-seq=1

AATATTGCACAATGGGGGAAACCCTGATGCAGCAACGCCGCGTGAGTGAAGAAGTGTTTC

GGTACGTAAAGCTCTGTCAGCAGGGAAGAAAATGACGGTACCTGACCAAGAAGCCCCGGC

TAACTACGTG

>5602 len=130 tot-seq=1

TCTGGGATGCTCAAAGCACTGTGTATGTGCTGACTTTCCAGATGCCCAAAGAATGTTAAC

ATCTGCTCATGACGAGTGTTTTGAGAAAGTTAGCCAAAGCTGTGGCAGAACAATGCCTAG

GAAAGTTTCA

>5603 len=130 tot-seq=1

AGATCTCCAGGTGACTGGAGTTCAGACGTGTGCTCTGCCGATCTCCTACGGGAGGAAGCA

GTGATTAACCTTTAGCAATAAACGAAAGTTTAACTAAGCTATACTAACCCCAGGGTTGGT

CAATTTCGTG

>5604 len=130 tot-seq=1

CAGACGGCAGGCGGGAGCCCAGGGTAGAAGGCTGGGGAACGGAACCGTCTGACCAGTGCA

GGGAATGCATCTCAACACGAAGCAGGCCAAGGCGGCTCAGCTGGACTTAACTGTAACAAG

CTCACTTCCT

>5605 len=130 tot-seq=1

CAGTGGTGGGCTGGCATGTCTATACTGCTCTGAGCACGTGCACACCCAACTGGGTTATGA

CAGTGGCTGGGTGCAGCCACAACTTTGCTATGCCCAGCGCCGCGGTAATCCGGAGATCGG

AAGAGCGTCG

>5606 len=130 tot-seq=1

GCCCTGGGATGGAAGGAGGGACTAGAGAAGCCCCTCCTTCTCCTGCAGGGGAACAGGACT

CTGAACTTTGGCCAGGACACTTGAGGATGCCAGAGAACCCCAGCCTCTGAATGACCTCCT

GTGTCTCTGT

>5607 len=130 tot-seq=1

GGTCAGCCACAGGCAACTCTCACGGCTGCATGATCTTTGATGATGAATTGTTATCAACAC

AGGGCATGATGAAGTCCCGAGCTTCTTGCCTTATAAAATGGCCCTGATCACTTTCCAGGA

ACATTGCTAC

>5608 len=130 tot-seq=1

AGTGAAGTCTTTAAAATACTGAAAGAAAAAAGCATCATCCTGGAAGTCTACAACCTACAA

AAGTATCTTTCAAAATGTAGGCTAAAGAGAGACTTTTTAAAAAGGGAAACAAAACAGAGA

GAATTTATTG

>5609 len=130 tot-seq=1

GCAGACGGAGGCGAGGAGCGATTCCTTGGGCATCTTACGTCACTCTCCGGCCCCTGAGCA

GCTGTCTCGTTTAGGGAAAAGGTGTGTTCAGGCTGAATCTCTGTGGTCCCTCTTCTCCCT

GCTCCCATAC

>5610 len=130 tot-seq=1

GAGGGTCCAGGCCCTGCGGGACTCGGTAATTGGTCCCAGCCTCGCTGCTCTTGCAGCCGC

AGGGGCTGGGCTGGTGGGATCCTGGGAGGTCTGTGCCCAGGCAGTGGCTGGGAGGTCCTG

CCATCCCGGC

>5611 len=130 tot-seq=1

CTCAGAGCTTTCTCAATTAACCACTTCAAGTGCAACAGTATGTGCCATAAGAGAAAGCCG

CATATCCCGAATTAAAGGGCTCTTGATCAAAATTTAATAAACAAAAAAGGCCTTGATTTG

GGCTTTTTTC

>5612 len=130 tot-seq=1

GCAGGACCCTGCAGAAAGGGCCGCCAGGGACATCTGTCATCCTGGCCACACTTACGTCTG

TGGAAACTGATGGCTATAAGCCTCATAAATGTGATTTCACCAGTTAAGACTCCAGCCCCG

ATGGCACGAG

>5613 len=130 tot-seq=1

AGTCTGAACCAGCCAAGTAGCGTGAAGGATGACGGCCCTATGGGTTGTAAACTGCTTTTG

TATAAGAATAATGGGCATTACGTGTAATGTTTTGCATGTACCATTCGAATAAGGACCGGC

TAATTCCGTG

>5614 len=130 tot-seq=1

AGTCTGAACCAGCCAAGTAGCGTGAAGGATGACGGCTCTATGGGTTGTAAACTGCTTTTG

TATGGGAATAATGGGCATTACGTGTACTGTTTTGTATGTACCATATGAATAAGGACCGGC

TAACTCCGTG

>5615 len=131 tot-seq=1

TCTACTTCACAACGTTGGTTGGGTATACTGTATGACATCCAATAGTCCTATGAGTGGGGA

GTGAGGGTAATAGGGAAGATATAAAAAAGATGTAAGAGACAGTTATTGGAGGGCTGAAGG

CCTATACCACA

>5616 len=131 tot-seq=1

CAAGGAATGGGCTACAAACTGGGGAGTGGGGGCAGCCTGGAACAGTCAGAGTCCTACTCT

GAAGTCTTGCAGGCAGCAGCGGAAATAAACTGTGCATCAGGATAAGGGGGCCTGGGAGTT

TAGGGTACGTG

>5617 len=131 tot-seq=1

GATTGGGCCGAGGGGAAAAGTGTTAAGATGTCATTGGCACTCACCCAGGAAGGAAATCAG

GCCTGGTAGGAGGCTGCTCTCTCCAGCACAGATGCCCTGCTCCGCAGAGAAATGCAACCA

ATGGCCCTGTG

>5618 len=131 tot-seq=1

AAACAGGATAATTCCAGGGCTCTTGAAACATCTTTCCCTGCACATTAAATCAGGAGGGCT

GAGCTGGCTCAGAGCCTGCTTGGCCTGTGTGAAACCCTGGCTTCTCCGAACAACAGAGTT

CTAAACCACCA

>5619 len=131 tot-seq=1

TGGGGAATATTGCACAATGGGCGCAAGCCTGATGCAGCCATGCCGCGTGTATGAAGAAGG

CCTTCGGGTTGTAAAGTACTTTCAGCGGGGAGGAAAAAAATGACGTTACCCTCAGAAGAA

GCACCGGCTAA

>5620 len=131 tot-seq=1

TGATGGAGCAACGCCGCGTGAGTGAAGAAGGTCTTCGGATCGTAAAGCTCTGTTGTTAGA

GAAGAAGAAGAGTAAGAGTAACTGTTAATTGGATGACGGTATCTAACCAGAAAGTCACGG

CTAACTACGTG

>5621 len=131 tot-seq=1

ATATTACGGTGGTGAAAGAAACACCCATTGCGGTAGGTGAACGTATCTCTACAACGGCAT

CGCCAGAAACTAATCGCATCATCCGCGAGGAAACGATGAAAGCTAGACAAGATAAAAAAC

AAATCGTTGCA

>5622 len=131 tot-seq=1

GTTTAAGAGGCCAAAGAAGAGACCTAGAGCCAGCAAACAAGACACAGGGTTTTATTAGGG

GCTTACATACACGGAAGAGAGTCCAATGGTGGTGGGCTGGACAAGAGAACTGCCTTATGT

ACAGATATGGT

>5623 len=131 tot-seq=1

GTACCAGAGCCTTCTCCCTAACCCAGGTCCTCCCCACGACTGTGCCTGCACAGAACCACA

CAGGATCCCTGTTTATCTTCAGATCTTCAGCCTCTCTTGGATGCTGGCCTTGTCTGGTTC

TCTCCAGACAC

>5624 len=131 tot-seq=1

TGGGGAATATTGCACAATGGGGGGAACCCTGATGCAGCGACGCCGCGTGAGTGAAGAAGT

ATTTCGGTATGTAAAGCTCTGTCAGCAGGGACGAAAATGACGGTACCTGCAGAAGAAGCC

CCGGCTAACTA

>5625 len=131 tot-seq=1

CAGTCTAATACCAAAATGAAACTGGACCCTTACCTTACACCATATACACAATGAAAAAAA

GCAAACATTAGGCCAATATCCCCAATGAACATAGACATAAGAATCGTCAACAAAATAGTA

GCAAACTGAAT

>5626 len=131 tot-seq=1

CTGATGGAGCAACGCCGCGTGAGTGAAGAAGGGTTTCGGCTCGTAAAGCTCTGTTGGTAG

TGGAGACAGATAGAGGTAGTAACCGGCCTTTATTTGAGGTAATTACTTAGAAAGTCACGG

CTAACTACGTG

>5627 len=131 tot-seq=1

TGATGGAGCAACGCCGCGTGAGTGAAGAAGGTTTTCGGCTCGTAAAACTCTGTTGTTGGT

GAAGACGGTCAGGGGTAGTAACTGTCCTTTGTTTGACGGTAATCAATTAGAAAGGCACGG

CTAACTACGTG

>5628 len=131 tot-seq=1

TGGGGAATATTGCGCAATGGGGGAAACCCTGACGCAGCGACGCCGCGGTAATAACAAGAT

CGGAAGAGCGTCGTGTAGGGGAGTGTGCCAATGTGTAGATCTCGGTGGTCGCCGTATCAT

TAAAAAAAAAA

>5629 len=131 tot-seq=1

ACTCAGGTATGGATTTTAGAAAGATGTCTATATGGAGAATGGATTGGAAGAAATCAAGGT

TTATCTGTGCAAGATGAATAAAACAGGGCCCCCACCCTTAAGGAGCTGAAAGTCTAAAAG

GGGAAAAGATA

>5630 len=131 tot-seq=1

TGGCAGTTTTGAGAAGAAGCCAGACCTCCACGCTCTGCACAACAGCCTGAGCCCGGCCTA

CGGTGGTGCTGAGTTTGCCGAGCTGCCCAGCCAGGCTGTTCCCACAGACGCCCACCACCC

CACTCCTCACC

>5631 len=131 tot-seq=1

GGAGGGAGAAACGACGAGGGAGTGAAGAAGGGTGGCGGCGCGTAAAGCGCGGGGGGGGGG

GAAGAAGGACAGGGGGAGGAACTGACCTTGGTTTGACGGTAATCAATTAGAAAGTCACGG

CTAACTACGTG

>5632 len=131 tot-seq=1

GGAGGGAGAAAAGACGCGGGAGGGAAGAAGGGGGGCGGCGCGGAAAGCGAGGGGGGGGGG

GAAGAAGGACAGGGGGAGGAACGGACCGGTGTTTGACGGTAATCAATTAGAAAGTCACGG

CTAACTACGTG

>5633 len=131 tot-seq=1

ATACCCATAGGGAAGCCCTTCTTTCCCAAATTCCCACTTGAGCAGGGCATCCTGCGAGAG

CCGGGAAAAGGGTATGGAGAACATGAGGTTAGGGACAAGGGTTCAATTACTCTTTTGGGA

TGGAGTGAAAA

>5634 len=131 tot-seq=1

ATCTGATCCCAATCCCGACCCGGGGAGGTAGTGACGAAAAATAACAATACAGGACTCTTT

CGAGGCCCTGTAATTGGAATGAGTCCACTTTAAATCCTTTAACGAGGATCCATTGGAGGG

CAAGTCTGGTG

>5635 len=131 tot-seq=1

GAGCCTGAACCAGCCAAGTAGCGTGCAGGATGACGGCACTATGGGTTGTAAACTGCTTTT

AGAGGGGAATAAAGTGAGGGACGTGTCCCTTATTGCATGTACCATGCGAATAAGGGCCGG

CTAATTCCGTG

>5636 len=131 tot-seq=1

TGACGCAGCGACGCCGCGTGAGGGATGACGGTTTTCGGATCGTAAACCTCTGTCAGAATT

GAAGAAACTACATTGTGCTAATCAGCAGTGTACTGACGGTACCTTCAAAGGAAGCACCGG

CTAACTCCGTG

>5637 len=131 tot-seq=1

TGCGCACATGGCACCGTCAGGGTCTGTGCTGATCCTGGGTGCTCAGAGGGCCTGCAGCAG

CAGGTTTTGTACCAACTCTGCCGGCAACCCAGGTAGAGGCAGGCACGAGCACCTGCTAAA

CTCAGTTTCCA

>5638 len=132 tot-seq=1

GCGCGTAAATTACCCACTTTCGACCCGAAGAGGTAGTGACGACAAATAACTCTTGGCGCC

CCTCGGGGTGACTTGAAGGAATGATTTAGAAGTAAATTCTCTAAAGAAATCAATTGGAGG

GCAAGTCTGGTG

>5639 len=132 tot-seq=1

CCCTGATCCAGCAATGCCGCGTGTGAAGAAGGCCTTCGGGTTGTAAAGCACTTTTGTCCG

GAAAGAAATCGCCGTGGTTAATATCCGTGGCGGATGACGGTACCGGAAGAATAAGCACCG

GCTAACTACGTG

>5640 len=132 tot-seq=1

CCCGAGCCGCACCAAGAGGCCGCACTGTTGAAATTCTTCTGCCTGGAAGATAAATGGCCT

ACTTTACTCTCAAGTGTCAAGTAGAGAAAACAAAACAAAACAAAAATCCCTTCCAAGTGG

ATTTTCGGAGGA

>5641 len=132 tot-seq=1

CTGATCCAGCCATGCCGCGTGTGTGAAGAAGGCCTTTTGGTTGTAAAGCACTTTAAGCAG

TGAAGAAGACCTAACGGTTAATACCCGTTAGCGATGACATTAGCTGCAGAATAAGCACCG

GCTAACTCTGTG

>5642 len=132 tot-seq=1

CTGATGGAGCAACACCGCGTGAGTGAAGAAGGGTTTCGGCTCGTAAAACTCTGTTGTTGA

AGAAGAACGTGCGTGAGAGTAACTGTTCATTCGATGACGGTATCTAACCAGCAAGTCACG

GCTAACTACGTG

>5643 len=132 tot-seq=1

AGAGCCTGAACCAGCCAAGTAGCGTGCAGGATGACGGCCCTATGGGTTGTAAACTGCTTT

TATAAGGGAATAAAGTGGAGGTCGGGACCTCTTTTGCATGTACCTTATGAATAAGGACCG

GCTAAGTCCGTG

>5644 len=132 tot-seq=1

TGACCGAGCAACGCCGCGGGAGTGAAGAAGGGTTTCGGATCGTAAAGCTCTGTTGGTAGA

GAAGAACGTTGGGAGGAGTGGAAAATCTACCAAGTGGCGGTAACTAACCAGAAAGGGACG

GCTAACTACGTG

>5645 len=132 tot-seq=1

CTGATGGAGCAACGCCGCGTGAGTGAAGAAGGTCTTCGGATCGTAAAGCCCTGTTGTTAG

AGAAGAAGGTGCGTGAGAGTAACTGGTCACGTTTCGACGGTATCTAACCAGAAAGCCACG

GTTAACTACGTG

>5646 len=132 tot-seq=1

GCTCAGAAGAGTTCAAAACCTCAGTTCCAAGAGTCTGTACCTTATCTAAGACACAATAGC

ACATGGCTGAAGATTCTTCAGCAGAGGAGGGAAAGGGGAGTGGGTGTGAGGCCAGGGATC

CTGGAAAGTTGT

>5647 len=132 tot-seq=1

TAAGGATTTCCCTTTCATCTGCCAGATGAGCCCCCCAGGAGGACAATGGGTGCCCCACCT

GGACATGGGCAGGGGCTTTGAGGCTGCTGGCTGGCAACTGGGGTCGGTGGATGGAGTGGG

CATGGACTGGGG

>5648 len=132 tot-seq=1

CTGATGGAGCAACGCCGCGTGAGTGAAGAAGGGTTTCGGCTCGTAAAACTCTGTTGTTGG

AGAAGAACGTGAGTAAGAGTAACTGTTTACTCAGTGACGGTACCCAATCAGAAAGTCACG

GCTAACTACGTG

>5649 len=132 tot-seq=1

AGAGTCTGAACCAGCCAAGTAGCGTGCAGGATGACGGCCCTATGGGTTGTAAACTGCTTT

TATAAGGGAATAAAGTTAGTCTCGTGAGACTTTTTGCATGTACCTTTCGAATAAGCGCCG
GCTAACTACGTG
>5650 len=132 tot-seq=1
CTGATGGAGCAACGCCGCGTGAGTGAAGAAGGGTTTCGGATCGTAAAGCTCTGTTGTTGG
AGAAGAACGTGTGAGGGAGTAACTGGTCACGCAGTGACGGTATCCAACCAGAAAGTCACG
GCTAACTACGTG
>5651 len=132 tot-seq=1
AGTCTGATGGAGCAACGCCGCGTGAGTGAAGGTTTTCGGATCGTAAAGCTCTGTTGTTGG
TGAAGAAGCATAGAGGTAGTAACTGGCCTTTATTTGACGGTAATCAACCAGAAAGTCACG
GCTAACTACATG
>5652 len=132 tot-seq=1
TTTTTTCAAGCAGAAGACGGCATACGAGATCGTGATTGACTGGAGTTCAGACGTGTGCTC
TTCCGATCTCCTACGCGAGGCAGCAGCGACTGGGCTCCCTCAGGTTCTTGGCAGAAGCCA
GCTCCTTCTATG
>5653 len=132 tot-seq=1
TGGGGAGTATTGCACAATGGGCGCAAGACTGATGCAGCAACGCCGCGTGAGGGAAGACGG
TCTTCGGATTGTAAACCTCTGTCTTTGGTGAAGAAGAAAGGGACGGTAGCCAAGGAGGAA
GCCACGGCTAAC
>5654 len=132 tot-seq=1
CAACCGTGACTCAGGCGGAGCTGGGGCACCTGTCGGCCGAGGGTCAGGAGGGCCAGGCCA
CCACCATACCCATTGTGCTGACACAGCAGGAGCTGGCTGCCCTGGTGCAGCAGCAGCAGC
TGCAGGAGGCCC
>5655 len=132 tot-seq=1
CTGATGGAGCAACGCCGCGTGAGTAAAGAGGGTTTTCGGCTCGTAAGGCTCTGTTGTTGG
TGAAGAAGGACAGGGGTAGTAACTGACCTTTGTTTGACGGTAATCAATTAAAAAGTCACG
GCTAACTACGTG
>5656 len=132 tot-seq=1
TGGGGAATATTGCACAATGGAGGGAACCCTGATGCAGCAACTCTGCGTGTGTGAAAAAGG
TTTTCGGACTGTAAAACACTTTTAGTAGGGAAGAAAAAAATGACGGTACCTACAGAAGAA

GCAACGGCTAAA

>5657 len=132 tot-seq=1

TGCGCAATGGGGGAAAAAATGAAGAAGAGAAGAAGAGTGAGGGATGAAGGAATTAGGGGG

GGAAAAAGAGGGGGAGGGGGAGAAAGAGGTAGGGTGAGTGTAAATTTCGAATAAGCGCCG

GCTAACTACGTG

>5658 len=132 tot-seq=1

CTGATGGAGCAACGCCGCGTGAGTGAAGAAGGTTTTCGGCTCGTAAAACTCTGTTGTTGG

AGAAGAACGTGCGAGATAGTAACTGTTCACGCAGTGACGGTATTCAACCAGAAAGTCACG

GCTAACTACGTG

>5659 len=132 tot-seq=1

CATCCACAGGCCTGGGGGCCCAGGGCTGGTGCTGAATTGCCTGACTCCCACGCCCAGGGT

GGGAAGGTCACCTTGGGCTCTGAGTGGCAGTGTGCTGTGGTTTGCACTAGGCGGGGAACC

CTGCAAGGACAG

>5660 len=132 tot-seq=1

TTTACGGGTCACCCTGTGGGACCAAGTGTCTGAATCAATCAAGTCAAGATTCTCAGATCC

AGATTTCTGTACTGGATGCTCCACACCCAACTCCTGTCTCTTCCTGTCCTAATAAACAGT

GCCATTATCCAG

>5661 len=132 tot-seq=1

TTCTAGATGCATGCAGGCCCTACTCACCACAACCTGCTCTGGCACCATGCTGTTCACCAG

TCTCCCAAGGCCAAATCCGCTACCCCAGGAGGAAGTGCCCTCCCCTGACTATGATGTCAC

GCTGACAAGCCT

>5662 len=132 tot-seq=1

GTAGGAGGGGTAGTTCGGGAGACACACCAAGAATTTTTCCAGAACATTAGAGCAGCGGGA

CTCAGTCATGCTGCCGTGTGACGTTACAGGACAGAAAACTGTTGGTGACCAGTTTCCTGT

TAGAAGAGGGTT

>5663 len=132 tot-seq=1

TTTCATCAGCAATATTTCCTGATAGGCCAGATATGCTCGCAGTCATCCCAAGTAAAAGCA

AACAAATACTGACTCTAGGTCCACTGGAATACTAAAGAAGGCACTACAGAAATGAGTCAC

AGTGTCTCACTT

>5664 len=132 tot-seq=1

GTGTGTGCAGGGTGGAGGGTGTTTGGGGCTGTGGAACACCAGCCGTTCCCACATCTCTGC

TCTGTGTCTTCTGCAGGTGACGGAAGTACCGCCTCCTCCCGTCTGACGCCCCTCAGGGGA

CCCTGCATCGCT

>5665 len=132 tot-seq=1

ACGGCATACGAGATTCGCTTGTGACTGGAGTTCAGACGTGTGCTCTTACGATCTCTTACG

GGAGGCAGCAGAAGGCGATGGATTCCAAACTGCGGCGATCGGCAAGAGCCATGCAATTGC

GGTAGCAGTCGG

>5666 len=132 tot-seq=1

TGGGGAATTTTCCGCAATGGGCGAAAGCCTGACGGAGCAATGCCGCGTGGAGGTAGAAGG

CCCACGGGTCGTGAACTTCTTTTCCCGGAGAAGAAGGAATGACGTGGGGAATAAGCATCG

GCTAACTCTGTG

>5667 len=132 tot-seq=1

GAAGGAAGCTGAACCACCCCCCCAGAAGTCACACACAGCCATCCCTTCAGCCTCCTGTTG

CCGACACAGGGCAGCCTCATTCAGTACGGGACAGACACCAGGAGGCAGGGTCCCCCAGGG

CCATCCTGGAGG

>5668 len=132 tot-seq=1

CTGATGGAGCAACGCCGCGTGAGTGAAGAAGGGTTTCGGCTCGTAAAGCACTGTTGTTGG

TGAAGAAGGACATATGTAGTAACTGACCTTTGTTTGACGTTAATCAATTAGAAAGTCACG

GCTAACTACATG

>5669 len=132 tot-seq=1

CTGATGGAGCAACACCGCGTGAGTGAAGAAGGGTTTCGGCTCGTAAAGCTCTGTTGTTGG

TGAAGAACGTGCGTGATAGTAACTGTTCACTCAGTGACGGTAACCAACTAGAAAGTCACG

GCTAACTACGTG

>5670 len=132 tot-seq=1

ATGATGGAGAAAAAAAGAGTGAGTGAAGAAGGGTTTAGGATAGTAAAGATATGATGTTGG

AGAAGAAAGTGCGTGAGAGTAACCGTTCACGCAGTGACGGTATCCAACCAGAAAGTCACG

GCTAACTACGTG

>5671 len=132 tot-seq=1

GTGTGCAAATTGCCCACTCCGGACACGGGGAGGTAGTGATGAAAAATAACAGGAGTCTTT

TGAGGCCCTCTAATTGGAATGAGTCCAGTTTAAGTCCTTCCGCGAGGATCCATGGGAGGG

GCAAGTCTGGTG

>5672 len=132 tot-seq=1

AGACCAAGACTATAGTGACAAGCTAGATGGATGTGATCTCTGCTCCCATGGAGTCACAGT

CCTGTAGGGGGTGACGAAAACTGAATGAGCCATTGCAATCGATTTAGCACAATGGATGCC

ATTACATGGGAC

>5673 len=132 tot-seq=1

CGGAGGGAGCAACGCCGCGGGAGGGAAGAAGGTTTTCGGATCGGAAAGCTCTTTTGTTGG

TGAAGAAGGATAGAGGTTGTAACTGATCTTTATTTGACGGTAATCAACCAGAAAGTCACG

GCTAACTACGTG

>5674 len=132 tot-seq=1

GTCGCGTTCGATGACTCCACCCCCGTGCAGAGGCAGCTGGTGGACATCCGGTACTCCAAG

GACACGAGGAAGCAGTTCAAGCCGGGCCTGGCCTACGTGGGGAAGGTGGAGCTATCCTAC

CCCGATGGCAGC

>5675 len=132 tot-seq=1

GCAGGAGATATGACCTAGTGGGCTCGAAGGAGACAGGTCCAGGTGCCAAAGAGGAGGACT

GCAGGCCTACAGAGGGGTGGGGACAAACTGATGGGCAGACCTGGAGGCCGGCAAATTATT

CACAGCAGCCAG

>5676 len=132 tot-seq=1

CTGATGGAGCAACGCCGCGTGAGTGAAGAAGGGTTTCGGCTCGTAAAGCTCTGTTGTTAG

TGAAGAAAGATAGTGGTAGTAACTGTTCTTTATTTGACGGTAATTACTTAGAAAGTCACG

GCTAACTACGTG

>5677 len=132 tot-seq=1

TGACGGAGCAACGCCGCGTGAGTGAAGAAGGTCTTCGGATTGTAAAACTCTGTTATACGT

GACGAAAAAGGCGTATGCCAACAGTATCCGTCCGTAACTTTACCTTAATACAAAGCCACG

GCTAACTACGTG

>5678 len=132 tot-seq=1

CTGATGGAGCAACGCCGCGTGAGTGAAGAAGGTTTTCGGAGCGTAAAGCGCTGTGGCTGG

TGAAGAAGGATAGAGGTAGTAACTGGGCTTTATTTGACGGTAATCAACCAGAAAGTCACG

GCTAACTACGTG

>5679 len=132 tot-seq=1

CTGATGGAGCAACGCCGCGTGAGTGAAGAAGGGTTTCGGCTCGTAAAGCTCTGTTGTTGG

TGAAGAAGGACAATGGTAGTAACTGTCCTTTGTTTGACGGTAATCAATGAGAAAGCCACG

GCTAACTACGTG

>5680 len=132 tot-seq=1

CTGATGGAGCAACGCCGCGTGAGTGAAGAAGGGTTTCGGCTCGTAAAGCTCTGTTGTTGA

AGAAGAACGTTAGTGAGAGTAACTATTCTCGCTGTGACGGTATTCAACCAGAAAGTCACG

GCTAACTACGTG

>5681 len=132 tot-seq=1

GTTCTTATGGCCCTTGTACCTAAGGGTGGACAGGATCGGGGGGCTTGGTCCTCTACAGGG

GTGGGAGAGGAGGGAAGGGGATCCCCACGACCACAAGGACTCACTCGCCCAGCAGCTCCC

CTGTGTCTTTGT

>5682 len=132 tot-seq=1

GCGCGCAAATTGCCCACTCCTGACACAGGGAGGCAGTGACAAGAAATAACAATGCGGAGC

CTACGATTTCGCAATTGGAATGAGAACAATTTAAATCCCTTATCGAGGATCCATTGGAGG

GCAAGTCTGGTG

>5683 len=132 tot-seq=1

CTGATGGAGCAACGCCGCGTGAGTGAAGAAGGGTTTCGGGTCGTAAAGCTCTGTTGGTAG

TGAAGAAAGATAGATGTAGTAACTGGCCTTTATTGGAAGGTAATTACTGAGAAAGGCACG

GCTAACTACGTG

>5684 len=132 tot-seq=1

GCGCGTAAATTACCCAATCCTGACATAGGGAGGTAGTGACAATAAATCACAATGCGGAAC

TATTAGTTTTGCAATTGGATTGAGAACAATGTACAAATGTTATCGATAAACAATTGGAGG

GCAAGTCTGGTG

>5685 len=133 tot-seq=1

GAGTGGGAGTCAAGAGATACTTCAGTCAGAATTGATGAGAAACATCTGCTCTGCCATGCC

TATCAGGACAAGAAACAAGATGCACAACGGTGCCACTGGGGGCAGCCAGGCCAGTGAACT

GACGAACATTCAG

>5686 len=133 tot-seq=1

CTGACGGAGCAACGCCGCGTGAGTGAGGACGGGCTTCGGATTGTAAAGCTCTGTTAATCG

GGACGAATGGTGTGTGTGCAAAGAGGGCATAGACATGACGGTACCGGAATAGAAAGCCAC

GGCTAACTACGTG

>5687 len=133 tot-seq=1

TAGGGAATATTGCACAATGGAGGAAACTCTGATGCAGCAACACCGCGTGAGTGAAGAAGG

GTTTCGGCTCGTAAAGCTCTGTTGTTGGAGAAGAACGTATCTGAGAGTAACTGTTCAGGT

AGTGACGGTATCC

>5688 len=133 tot-seq=1

TGGGGAATATTGCACAATGGGGGAAACCCTGATGCAGCGACGCCGCGTGAGCGAAGAAGT

ATTTCGGTATGTAAAGCTCTATCAGCAGGGAAGATAATGACGGTACCCTGTGAGGAAGCC

CCGGCTAACTACG

>5689 len=133 tot-seq=1

CGACGACCCAGGCCTTGGTGTCAAATGGCTCTGCCCAAAGATGGCTCCTGAGCCCCAAGT

GGGGGCTTCAAGCAGGGCTGGGGAGGAGGCTACCCTTCTGGCCAGGCCAGGAATGAGCCC

CCTACCCCTGTCC

>5690 len=133 tot-seq=1

GTAGGTGGAGGTAGCCACTTAGGGCAGGGGAAGGTCCATTGTTGTTCACGCCTGGTTTCA

CTCCCAGGGCAATGTTGACACAGGAGCTCCAAATGCAATGGCGGTAATGCGGGGGTAATG

GGGGCAGAATGCA

>5691 len=133 tot-seq=1

GCGCGCAAATTACCCAATCCCGACACGGGGAGGTAGTGACAATACATAACAATATACGGC

TTTAACGTCGTATAATTGGAATGAGTACAATTTAAATCCCTTAACGAGGAACAATTGGAG

GGCAAGTCTGGTG

>5692 len=133 tot-seq=1

TCTGATGGAGCAACGCCGCGTGAGTGAAGAAGGTTTTCGGCTCGTAAAGCTCTGTTGTAA

GTGAAGAACGACAGGGGTAGTAACTGACCTTTGTTTGACGGTAATCAGTTAGAAAGTCAC

GGCTAACTACGTG

>5693 len=133 tot-seq=1

TGTTCTGCAATCCTATGTGAGCGACGAACATTGAGAACTTCGTAGCAGTGTTCTGATATT

CTATGTGAGAGCAAACACTCACAACCCAGCAGCACTGCTCTGGAATCGTATGTGAGGGAC

AACAACTCAGAAC

>5694 len=133 tot-seq=1

GGATATTGCACAATGGGGGGAACCCTGATGGAGCGACGCCGCGTGAGCGAAGAAGTATCT

CGGTATGTAAAGCTCTATCAGCAGGGAAGAAGAAATGACGGTACCTGAGTAAGAAGCCCC

GGCTAACTACGTG

>5695 len=133 tot-seq=1

TACTATATAAAGGTGAGTAAACGATGTTGCTGTTCTGCTGCTGTTGATGACATCAGTTCT

ATTAAAGTCAAGGTAAACAACTAAATGGTTATGAATTTTCTAAGTTTGGAGCTAATCCAC

AGATTTATCCTTG

>5696 len=133 tot-seq=1

TTGCACAATGGGCGAAAGCCTGATGCAGGGAGGCCGCGGGCGGGATGAAGGCTTTCGGGT

TGTAAACCGCTTTCAGCAGGGACGAGGCCGCAAGGTGACGGTACCTGCAGAAGAAGCCCC

GGCTAACTACGTG

>5697 len=133 tot-seq=1

AATATTGGGCAATGGGCGAAAGCCTGACCCAGCGACGCCGCGTGAAGGAAGAAGGCCCTC

GGGTTGTAAACTTCAGTCAACAGGGAAGAAACAAATGACGGTACCTGTGGAGGAAGCCAC

GGCTAACTACGTG

>5698 len=133 tot-seq=1

GAGGGGGTTTCGGGTGGCTGGCTGGCTGTCATTCATGGCTTTTCAAAACCCCAGACTCTC

CCTCGCCCACCTGAGTTTTAGCTTCACTATTTTCTCAGCCCCAGGATCTGGGTGTTTCAG

CAGAAAATTCTCA

>5699 len=133 tot-seq=1

GAGAGTCTGAACCAGCCAAGTAGCGTGCAGGATGACGGCCCTATGGGTGGTAAACTGCTT

TTGGTTGGGAATAATCGACATTACGTGTAAGGTTTTGCAGGTACCAGTCGAATAAGGACC

GGCTAATTCCGTG

>5700 len=133 tot-seq=1

CACCTTCCAAGAAAGCATCTGCTCTGTTTTCTCAGGTGACTTCACCAGCTTTCCCTTCCT

CCTCCTTCACCTCTCCTAAGGTTGACAAAGCCTGGATTTAGGTCAACCTCTTCAGTGGGT

CCTTCTGTTGTGG

>5701 len=134 tot-seq=1

GAGGATCAGAAGTTCAAGGTCTTCCTCAGCCACACAGCCAATTCAAAGCAGGATACCCGA

GACCTCGTCCCCGACAACCGCCCTAGCCCTTGCTTGAGACAGGCTCATTCACATCTCACA

AACCTACATCCACA

>5702 len=134 tot-seq=1

TCAGGAGAATAGAGTCTCTAGTCTGGGAAAGCAGGCTCACCTTAAGGCATGCCAAAGAGG

TAGGCAGACTCCGTTTTCTTTTCATGAGATACTACACTATACTTCCCAGCGATCACACCC

TCCCCCAGTACTCC

>5703 len=134 tot-seq=1

TGCCCTGCCATAGAAAAGCTGGCAAGACAGTGAGGCAGCAGGCCCACAGCGAGGCCCCTA

ACAAAGGCGGGGCGTTGGCAGGCAGCAGAAGAGGGGCGGGCTGAGATGGATGTGGCTTGG

AGCCTAACAGGCCA

>5704 len=134 tot-seq=1

GTGAGTTGGGCCTGAGCATGGGACAGCTGGCCCTACCACTCATCTCCCTGTGGTTGCTTA

GATGAGGGAAAGAGTCCCTTCTCCCCTGTCCTGGACCTGGGGTCATAGAGCAGAAGAGCT

GGCCCTGTCCTTCA

>5705 len=134 tot-seq=1

TGGGGAATATTGCACAATGGGGGAAACCCTGATGCAGCGAAGCCGCGTGAGCGATGAAGT

ATTTCGGTACAAAAGCTCTATCAGCAGGGAAGAAAATGACGGTACCTGACTAAGAAGCCC

CGGCTAACTACGTG

>5706 len=134 tot-seq=1

TGGGGAATATTGGGCAATGGGCGCAAGCCTGACCCAACGCCGCGTGAAGGAAGAAGGCTT

TCGGGTTGTAAACTTCTTTTAAGAGGGAAGAGCAGAAGACTGTACCTCTAGAATAAGCCA

CGGCTAACTACGTG

>5707 len=134 tot-seq=1

GGCTGTGCTCCTTCCCAAAGGCTTTAGAAGAGGATTCATTCCCTTGCTCATTGAGGTTGT

TGGCAGAATAAAGTTTCTGCAGTCACAGGGCTGAGTTCCCTGTTTCCTTGCTGGCTGTCG

GCTAAGGGCCATTC

>5708 len=134 tot-seq=1

TAGGGAATCTTCCACAATGGGCGCAAGCCTGATGGAGCAACACCGCGTGAGTGAAGAAGG

GTTTCGGCTCGTAAAACTCTGTTGTTGAAGAAGAACGTGCGTGAGAGTAACTGTTCACAC

TGTGACGGTATCTT

>5709 len=134 tot-seq=1

TGGGGAATCTTGCACAATGGGCGAAAGCCTGATGCAGCGACGCCGCGTGATTTAGAAGGC

CTTCGGGTTGTAAAAATCTTTTATATGGGAAGAAAATGACAGTACCATAAGAATAAGGTC

CGGCTAAATACGTG

>5710 len=134 tot-seq=1

TAGGGAATATTCCACAATGAGCGAAAGCTTGATGGAGCGACGCCGCGTGATTTAGAAGGC

CTTCGGGTTGTAAAAATCTTTTGTATGGGAAGAAAATGACAGTACCATACGAATAAGGAC

CGGCTAATTACGTG

>5711 len=134 tot-seq=1

TAGGGAATCTTCCACAATGGGCGAAACCCTGATGCAGCGACGCCGCGTGATTTAGAAGGC

CTTCGGGTTGTAAAAATCTTTTCTATGAGAAGAAAATGACAGTACCATAGGAATAAGGAC

CGGCTAATTACGTG

>5712 len=134 tot-seq=1

TTAGGAATCTTCGGCAATGGGGGAAACCCTGACCGAGCGACACTGCTTGAAGGATGAAGG

TCTTCGGATCGTAAACTTCTGAACCTGCGACGAAAAATGACGGTAGCAGGCTAATAGCAC

CGGCTAACTCCGTG

>5713 len=134 tot-seq=1

AGTCTGATGGAGCAACGCCGCGTGAGTGAAGAAGGGTTGCGGCTCGTAAAGCTCTGTTGT

TGGCGAAGAAGGACAGGGGTAGTAACTGACCTTTGTTTGAGGTAATCAACCAGAAAGTCA

CGGCTAACTACGTG

>5714 len=134 tot-seq=1

TGGGGGATATTGCACAATGGGGGGAACCCTGATGCAGCGACGCCGCGAGTGAAGAAGTAT

TTCGGTATGTAAAGCTCTATCAGCAGGGAAGAAGAAAGACGGTACCTGAGTAAGAAGCCC

CGGCTAACTACGTG

>5715 len=134 tot-seq=1

GCGCGCAAATTACCCAATCCTGACATGGGGAGGCAGTGACAATAAATAACAATACTGGGC

TCATCGAGTCTGGTAATTGGAATGAGTACAATCTAAATCCCTTAACGAGGATCCATTGGA

GGGCAAGTCTGGTG

>5716 len=134 tot-seq=1

GGCTTTTATGTAAGTGGTCTCTGGTCTTTGTCTGTTTGGTGGTGTACTTTTTTTTTTTAA

GCTACTGTCATTGCCCAGTCGTAGTTGAGCGTGTGTCCAGTTGCTTTATGTGTGGGCTTC

CTGGGGATGCCCTC

>5717 len=134 tot-seq=1

CAAGACACTGACCAAACATCTTCAGGAAACAAGCGAGTGAGCAGCTCCGCGGAACGTGAG

GCAGCAGGAGACTCACGGGGGTCTTTGCGTCCCCACCCCTCCGCGGACCCTGGATCTCCA

GGGACCCAGCCCCT

>5718 len=134 tot-seq=1

TGAGGAATATTGGTCAATAGGCGAGAGCCTGAACCAGCCACGTAGCGTGCAGGATGACGG

CCCTATGGGTTGTAAACTACTTTTATGTGGGGATAAAGTGCGCGACGTGTCGTGCATTGC

AGTTACCACATGAA

>5719 len=134 tot-seq=1

CTTAACTCTTTCTCTCTCTGGGCACAAGCGTGAGCCGTGCCATGCCGTGCTGTGCCGAAC

CCTGCCCTGGCTCCCCTCTGTCCATCTGCAAAATGGACGGCTCTGGTTATCCCTCTCCTC

TCTTTCTCTGGGTG

>5720 len=134 tot-seq=1

TGTTCTGGAATCCTATGCCAGGGACAAACACTCAGAAACCAGCAGTAGTGTTCTGGATTC

GTTTCTGAGGGACAAACATTCAGGACCTTGTAGCAGTGTTGTGGAATCCCATTTGAGGGA

CAAATACACTGAAC

>5721 len=134 tot-seq=1

GATTGAAAAAGACATCAACCTACAAGAACATAGAGGGAAGTTTAGCAGCAAAACAGCAAC

AATGTCTTGGAAGCCAGAAAGCAGATGAATAAATCTTAAATATCAGGGTAGATGTGAGAA

AATGACATAACAAG

>5722 len=134 tot-seq=1

TAGGGAATCTTCACAATGGGCGAAAGCCTGATGCAGCGACGCCGCGTGAGCGAAGAAGTA

TTTCGGTATGTAAAGCTCTATCAGAAGGGAAGAAAATGACGATACCTTACTAAGAAGCTC

CGGCTAAATACGTG

>5723 len=134 tot-seq=1

TGGGGAATTTTGCACAACGGGGGAAGCCCTGATGCAGCGACGCCGCGTGATTTAGAAGGC

CTTCGAGTTGTAAAAATCTTTTGTATGGGAAGAAAATGATAGTACCATACGAATAAGGAC

CGGCTAATTACGTG

>5724 len=134 tot-seq=1

CCCTGATGCAGCGACGCCGCGTGATTTAGAAGGCCTTCGGGTTGTAAAAATCTTTTGTAT

GGGAAGAACGAGTGTGAGAATGGAAAGTTCATGCTGTGGCGGTACTTAACAAGAAGGGGA

CGGCTAACTACGTG

>5725 len=134 tot-seq=1

AGCATGCCCTGCCCTGCTCCAGGCAGAAGGGTTGTAGGACTTAATCACGTTTCAGCTTGG

CTGTCGGGCTGTGAGTCACGGTTGCACTGCGATTATGTAAGCACGCAGGAATAGGTGGCA

TGACATATATGCTG

>5726 len=134 tot-seq=1

TGTTCTGCAATGCTATGTGAGGGACAAACATTCAAACAAGAGCAGGAATGTTCTGGAATC

CTATGTTATGGTCAAACATTCAGAATATTGTCTCAATTATCTGGAATTCTATGTGAGGGA

GAAACACTGTGAAC

>5727 len=134 tot-seq=1

CCCTGATGCAGCAACGCCGCGTGAGTGATGAAGGTTTTCGGCTCGTAAAACTCTGTTGTT

AAGGAAGAACACTTGAGGTAGTAACTGGCCTTGAGATGACGGTACTTAACCAGAAAGCCA

CGGCTAACTACGTG

>5728 len=134 tot-seq=1

GCGCACAAATTACCCACTCCCGACCCGGGGAGGTAGTGACGAAAAATAACAATACAGGAC

TCTTTCGAGGCCCTGTAATTGGAATGAGTCCACTTAATCCTTCAACGAGGATCCATTGGA

GGGCAAGTCTGGTG

>5729 len=134 tot-seq=1

GGACCCTGCTTCCCTAGGGGGAAGATGGGGCGCAGGGCAGGCTGTCCATCAAGGTGCTCC

AATTATGGGGATGGAGGGCCCTGGGGGAAGATCTGGCCAAAGCCCGTGGCCTCCCCCGCA

GCACGTGCACCCAC

>5730 len=134 tot-seq=1

GGAAGCACTGAGCAGGGGCCGAGTCAATCAGGGGTGTGTCTGAGGGACTGGGAGTCTGGG

CAGGGTGGGGCATGTGGCACAGCAGGGGGGCCTGGCAGCCCGGGAGGATAAAGGCAGAGT

GGGCAAGGGACGGA

>5731 len=134 tot-seq=1

AGGCGGAGGGAGCGACGCCGCGTGAGGGAAGAAGGGTTTCGGATCGTAAAGCTCTGTTGT

TGGTGAAGAAGGATAGAGGTAGTAACTGGCCTTTATTTGACGGTATCAACCAGAAAGTCA

CGGCTAACTACGTG

>5732 len=134 tot-seq=1

AGTATGTCCAAGAATTCTCACCTGGGAAGACAGACATTCCAGGGAAACACTGATATTTTT

GTTTTCTCCTCACTGGGAAAAAGGCAGCTGGCCTTCAGAAAACAACTTGCATGTACCTTA

TCTTCCCCACCCTC

>5733 len=134 tot-seq=1

AATAAGATGAAGTTGTACTAGAATATGGTGAACCCCAATCCAGTGATTGGCATCCTTATG

AGGAGAGGGAAATTTAGAGCCTGAGACACACGGAGGGAAGGCAGTGTGAAGACCCAGGAG

GATGCCCCGCGGTG

>5734 len=134 tot-seq=1

TGGGGAATATTGGACAATGGGCGAAAGCCTGATCCAGCGACGCCGCGTGAGGAAGAAGGT

CTTCGGATTGTAAACTCCTGTTGTTGGGGAAGATAATGACGGTACCCAACAAGGAAGTGA

CGGCTAACTACGTG

>5735 len=134 tot-seq=1

TGGGGAATATTGCACAATGGACGGAAGTTTGATGCAGCGACGCCGCGTGATTTAGAAGGC

CTTCGGGTTGTAAAAATCTTTTGTATGGGAAGAAAATGACAGTACCATACGAATAAGGAC

CGGCTAATTACGTG

>5736 len=134 tot-seq=1

TGCTTTGGAATCCTGTGTGAGCGACAAGGACTCAGGAATTCATAGCATTGTTCTGGAATT

CCATGTAAGAGGCACACACTGAGAACCCAGCAGCACTGTTCTGGAATCCTATGTGAGGGA

CAAAAACTCAGAAC

>5737 len=134 tot-seq=1

CGGGAGCCTGAACCAGCCAAGGAGCGTGCAGGAAGACGGCACTATGGGTTGTAAACTGCT

TTTATGCGGGGATAAAGGAGTACACGTGTGGGTTTTTGCAGGTATCGCATGAATAAGGAC

CGGCTAATTCCGTG

>5738 len=134 tot-seq=1

GGATCGTAACAGGAGAGTCCGGCAGGGCATTTTTACACACTGAGCAGTTGGATGGAAATG

GACGAAAAATTTCGCTGCTGTCCATTCTGCTAACCAATGTCATTAGTGTCCGGCGTTCTC

GGAAATCACTTCGT

>5739 len=134 tot-seq=1

TGGGGATATTGCACAATGGGGGAAACCCTGATGCAGCGACGCCGCGTGAGTGAAGGAGTA

TCTCGGTATGTAAAGCTCTATCAGCAGGGAAGAAAGTGACAGTACCTGGCTAAGAAGCTC

CGGCTAACTACGTG

>5740 len=134 tot-seq=1

CCTGACCGAGCAACGCCGCGTGAGTGAAGAAGGTCTTCGGATCGTAAAGCGCTGTTGTTA

AGGAAGAACGAGTGTGAGAGGGGAAAGTTCATACTGTGACGGTACTTAACCAGAAAGGGA

CGGCTAACTACGTG

>5741 len=134 tot-seq=1

TAGGGAATCTTCGGCAATGGGGAAACCCTGATGCAGCAACGCCGCGTGAGTGAAGAAGTC

ATTCGTGATGTAAAGCTCTATCAGCAGGGAAGAAAATGACGGTACCTGAGTAAGAAGCCC

CGGCTAACTACGTG

>5742 len=134 tot-seq=1

TGGGGAATATTGCACAATGGGGGAAACCCTGATGCAGCAACGCCGCGTGAGTGAAGAAGC

GCCTCGGCGCGTAAAGCTCTGTCAGCAGGGAAGAAAAAAGACGGTACCTGACCAAGAAGC

CCCGGCTAACTACG

>5743 len=134 tot-seq=1

GCCTGATCCAGCCATGCCGCGTGTGTGAAGAAGGGTTTCGGCTCGTAAAGCTCTGTTGGT

AGTGAAGAAAGATAGAGGTAGTAACTGGCCTTTATTTGACGGTAATTACTTAGAAAGTCA

CGGCTAACTACGTG

>5744 len=134 tot-seq=1

GTCTGATGGAGCAACGCCGCGTGAGTGAAGAAGGGTTTCGGCTCGTGAAGCGCTGTTGGT

AGTTAAGAAAGATAGAGGCAGTAACTGGCCTTTATTTGACGGTAATTACTTAGAAAGTCA

CGGCTAACTACGTG

>5745 len=135 tot-seq=1

CGTTAGAGCTGTTATATCATGTCCAGCTGAGAAAATAAGGACCGTGACAGGCAAAGCAGA

TACAAAATCAAGGTCTAGAAAACATAAGTAATGAAAGGCACCTGCTGCTGGGCAGTGGCA

GCCATGACTTTAATC

>5746 len=135 tot-seq=1

AGGTGTGAGTGGAATGGGGAGTAAGGGCAGCAGGACACCACATGACACTGAGGGAGGGGA

ACCGGCCAGGTCCTACAGGCACAGGACTGTAGGCTGGAGACAGACACCGTCAGATCTGTT

CTGAAAATAGTAGCC

>5747 len=135 tot-seq=1

TGGGGAATATTGCACAATGGGGGAAACCCTGATGCAGCGACGCCGCGTGAGTGAGGAAGT

AATTCGTTATGTAAAGCTCTATCAGCAGGGAAGAAAATGACGGTACCTGAAGAATAAGCA

CCGGCTAACTACGTG

>5748 len=135 tot-seq=1

TGGGGGATATTGGACAATGGGGGAAACCCTGATCCAGCGACGCCGCGTGAGCGATGAAGT

ATTTCGGTATGTAAAGCTCTATCAGCAGGGAAGAAAATGACGGTACCTGACTAAGAAGCA

CCGGCTAAATACGTG

>5749 len=135 tot-seq=1

TGGGGAATATTGCACAATGGGGGGAACCCTGATGCAGCAATGCCGCGTGGGTGATGGAGT

ATTTCGGTATGTAAAGCTCTATCAGCAGGGAAGATAATGACGGTACCTGACTAAGAAGCT

CCGGCTAAATACGTG

>5750 len=135 tot-seq=1

TTAGGAATCTTGGACAATGGGCGCAAGCCTGATCCAGCCATGCCGCGTGAGTGATGAAGG

CCTTAGGGTTGTAAAGCTCTTTCAGCGGGGAAGATAATGACGGTACCCGCAGAAGAAGCC

CCGGCTAACTTCGTG

>5751 len=135 tot-seq=1

TGGGGAATTTTGGACAATGGGCGCAAGCCTGATGCAGCGACGCCGCGTGAAGGAAGAAGT

ATTTCGGTATGTAAACTTCTATCAGCAGGGAAGAAAATGACGGTACCTGAGTAAGAAGCC

CCGGCTAACTACGTG

>5752 len=135 tot-seq=1

TAGGGAATCTTCCGCAATGGGCGAAAGCCTGACGGAGCAACGCCGCGTGAGTGAAGAAGT

ATTTCGGTATGTAAAGTTCTATCAGCAGGGAAGAGAATGACGGTACCTGAGTAAGAAGCC

CCGGCTAACTACGTG

>5753 len=135 tot-seq=1

TGGGGAATATTGCACAATGGGGGAAACCCTGGTGCAGCGACGCCGCGTGAGTGAAGAAGT

GTTTCGGTATGTAAAGCTCTATCAGCAGGGAAGAGAATGACGGTACCTGAGTAAGGAGCC

CCGGCTAACTACGTG

>5754 len=135 tot-seq=1

TGGGGAATTTTGGACAATGGGCGCAAGCCTGATGCAGCGACGCCGCGTGGAGGAAGAAGG

CCTTCGGATTGTAAACTCCTGTTGTTGAGGAAGATAATGACGGTACTCAACAAGGAAGTG

ACGGCTAACTACGTG

>5755 len=135 tot-seq=1

TGGGGAATATTGCACAATGGGGGAAACCCTGATGCAGCGACGCCGCGTGAGTGAAGAAGT

ATCTTGGTATGTAAACTTCTATCAGCAGGGAAGAAAATGACGGTACCTGAGTAAGAAGCC

CCGGCTAACTACGTG

>5756 len=135 tot-seq=1

TGGGGAATATTGGACAATGGGCGCAAGCCTGATCCAGCCATGCCGCGTGAGTGATGAAGG

CCTTAGGGCTGTAAAGCTCTTTCACCGGAGAAGATAATGACGGTATCCGGAGAAGAAGCA

CCGGCTAACTCCGTG

>5757 len=135 tot-seq=1

TGGGGAATATTGCACAATGGAGGAGACTCTGATGCAGCGACGCCGCGTGAGTGAAGAAGT

AATTCGTTATGTAAAGCTCTATCAGCAGGGAAGATAGTGACGGTACATGACTAAGAGGCT

CCGGCTAAATACGTG

>5758 len=135 tot-seq=1

TAGGGAATATTGCGCAATGGGCGAAAGCCTGACGCAGCGACGCCGCGTGAGTGACGAAGG

CCTTCGGGTCGTAAAGCTCTGTTGTAAGGGAAAATTATGATGGTACCTTATAAGAAAGGA

CCGGCTAACTTCGTG

>5759 len=135 tot-seq=1

CCAGTTTTGTGAAGTGGTTTTTTAGAAGGTGGAGTAAGTTGTTCTCACTGTTGTACGCTC

TTGGGTGAGCATCAAGGCAGTGAGAGCTGTGCTTCCTTGCACGCATCCTGCCCTCCTCAT

GACCTACGAGAGGCC

>5760 len=135 tot-seq=1

TGGGGAATATTGCGCAATGGGCGAAAGCCTGATGCAGCGACGCCGCGTGAAGGATGAAGT

ATTTCGGTATGTGAAGCTCTATCAGCAGGGAAGAAAATGACGGTACCTGACTAAGAAGCA

CCGGCTAAATACGTG

>5761 len=135 tot-seq=1

TGGGGAATATTGCACGATGGGGGAAACCCTGATGCAGCGACGCCGCGTGAGCGATGAAGT

ATCTCGGTATGTAAAGCTCTGTCAGCAGGGAAGAAAATGACGGTACCTGACTAAGAAGCA

CCGGCTAAATACGTG

>5762 len=135 tot-seq=1

TGGGGGATATTGCACAATGGGGGAAACCCTGATGCAGCGACGCCGCGTGGGTGAAGACGG

CCTTCGGGTTGTAAAGCCCTGTTGCAGAGGACGATAATGACGGTACTCTGTAAGAAAGCC

ACGGCTAACTACGTG

>5763 len=135 tot-seq=1

CGGGGAATATTGCATAATGGGGGAAACCCTGATGCAGCGACGCCGCGTGAGTGAAGAAGT

ATTTCGGTATGTAAAGCCCTATCAGCAGGGGAGAAAGTGACGGTACCTGAATAAGAAGCC

CCGGCTAACTACGTG

>5764 len=135 tot-seq=1

TGAGGAATATTGGTCAATGGGGGCAACCCTGACGCAGCAACGCCGCGTGCGGGACGAAGG

CCTTCGGGTTGTAAACCGCTTTCAGCAGGGAAGACTTTGACGGTACCTGCAGAAGAAGCT

CCGGCTAACTACGTG

>5765 len=135 tot-seq=1

TGAGGAATATTGGTCAATGGGGGAAACCCTGATGCAGCGACGCCGCGTGAAGGAAGAAGT

ATCTCGGTATGTAAACTTCTATCAGCAGGGGAGATAATGACGGTACCTGACTAAGAAGCC

CCGGCTAACTACGTG

>5766 len=135 tot-seq=1

TGGGGAATATTGCACAATGGGGGAAACCCTGATGCAGCGACGCCGCGTGAAGGATGAAGT

ATTTCGGTATGTAAACTTCTATCAGCAGGGAAGAAAATGACGTTACCCGCAGAAGAAGCA

CCGGCTAACTCCGTG

>5767 len=135 tot-seq=1

GGAAGCAGGGCACTGACTAAACTTCCAGGAGAGAGGGAGGAAGCCAGGGATGCAGAGTAG

AAGACAATGCTCAACTCAGTGTTTCATTCTGCATAACTCCTCACATGCCTGGCAGAGCAT

CTTCCAGAGATCCCC

>5768 len=135 tot-seq=1

TGGGGAATATTGGACAATGGGCGAAAGCCTGATCCAGCGACGCCGCGTGAAGGAAGAAGT

ATCTCGGTACGTAAACTTCTATCAGCAGGGAAGAGATTGACGGTACCTGACTAAGAAGCC

CCGGCTAACTACGTG

>5769 len=135 tot-seq=1

AAGACCAGAAAAGATCCAACCTAGCTCTGCTCCCTCCGTCAATCTGCCCAGCTCTTTATG

CAAAGATGGGGCTGAGGAGGGTCCACTCAGTGAATGGATAGCCACTACATATCTTATCCT

GGGCATCAGCCAAAA

>5770 len=135 tot-seq=1

TGGGGAATATTGCACAATGGGGGAAACCCTGATGCAGCGACGCCGCGTGAAGGATGAAGT

ATTTCGGTATGTAAACTTCTATCAGCAGGGAAGAAGATGACGGTACCTGACTAGAAAGCC

ACGGCTAACTACGTG

>5771 len=135 tot-seq=1

TGGGGGATATTGCACAATGGGGGAAACCCTGATGCAGCGACGCCGCGTGAGTGAAGAAGT

ATCTCGGTATGTAAAGCTCTATCAGCAGGGAAGAAAATGACGTTACCCGCAGAAGAAGCA

CCGGCTAACTCCGTG

>5772 len=135 tot-seq=1

TGGGGAATATTGCACAATGGGGGAAACCCTGATGCAGCGACGCCGCGTGAAGGAAGAAGT

ATCTCGGTATGTAAACTTCTATCAGCAGGGAAGATAATGACGGTACCTGAAGAATAAGCA

CCGGCTAACTACGTG

>5773 len=135 tot-seq=1

TAGGGAATCTTCCGCAATGGGCGAAAGCCTGACGGAGCAACGCCGCGTGGAGGAAGAAGG

TTTTCGGATTGTAAACTCCTGTCGTTAGGGACGATAATGACGGTACCTAACAAGAAAGCA

CCGGCTAACTACGTG

>5774 len=135 tot-seq=1

TGGGGAATATTGCACAATGGGGGAAACCCTGATGCAGCGACGCCGCGTGAGTGATGTAGT

ATTTCGGTATGTAAAACTCTATCAGCAGGGAAGATAATGACGGTACCTGACTAAGAAACA

CCGGCTAAATACGTG

>5775 len=135 tot-seq=1

TGGGGAATATTGCACAATGGGGGGAACCCCGATGCAGCGACGCCGCGTGAGTGAAGAAGT

ATTTCGGTATGTAAAGCTCTATCAGCAGGGAAGAAAGTGACGGTACCTGAATAAGAAGTC

CCGGCTAACTACGTG

>5776 len=135 tot-seq=1

TGGGGAATTTTGGACAATGGGGGCAACCCTGATCCAGCAACGCCGCGTGAGCGAAGAAGT

ATTTCGGTGTGTAAAGCTCTATCAGCAGGGAAGAAAATGACGGTACCTGACTAAGAAGCC

CCGGCTAACTACGTG

>5777 len=135 tot-seq=1

TGGGGAATATTGGGCAATGGACGAAAGCCTGATCCAGCAATGCCGCGTGAGTGAAGAAGG

CCTTAGGGTTGTAAAGCTCTTTTAGTAGGGAAGATAATGACGGTACCTACAGAAAAAGCA

CCGGCTAACTTCGTG

>5778 len=135 tot-seq=1

TGGGGAATTTTGGACAACGGGGGAAACCCTGATGCAGCGACGCCGCGTGAGTGAAGAAGT

AATTCGTTATGTAAAGCTCTATCAGCAGGGAAGAAAATGACGGTACCTGAGTAAGAAGCC

CCGGCTAACTACGTG

>5779 len=135 tot-seq=1

TGGGGGATATTGCACAATGGGGGAAACCCTGATGCAGCGACGCCGCGTGGAGGAAGAAGG

TTTTCGGATTGTAAACTCCTGTCGTTAGGGACGATAATGACGGTACCGGAAGAATAAGCA

CCGGCTAACTACGTG

>5780 len=135 tot-seq=1

TAGGGAATCTTCGGCAATGGGCGAAAGCCTGACCGAGCAACGCCGCGTGAGTGAAGAAGT

ATCTCGGTATGTAAAGCTCTATCAGCAGGGAAGAAAATGACGGTACCTGACTAAGAAGCC

CCGGCTAACTACGTG

>5781 len=135 tot-seq=1

TGGGGGATATTGCACAATGGGGGAAACCCTGATGCAGCAACGCCGCGTGAAGGAAGAAGG

TCTTCGGATTGTAAACTTCTGTCCTCAGGGAAGATAATGACGGTACCTGAGGAGGAAGCT

CCGGCTAACTACGTG

>5782 len=135 tot-seq=1

CAGAGAACCGGACAGCTTCTGGGACGGGCAGAAGCACAGAGCCGCTGAGGCAGCAGCCTG

GGCGTGCCACAGACAACCGGCCACCATCCGGACCAGAGGACAGGTGTCCGCCTGGCTTGG

GAGGCGGCCTCAGCC

>5783 len=135 tot-seq=1

TGGGGAATATTGCACAATGGAGGAAACTCTGATGCAGCGACGCCGCGTGAGTGATGAAGT

ATTTCGGTATGTAAAGCTCTTTCAGCAGGGAAGATAATGACGGTACCTGCATAAGAAGCC

CCGGCTAACTACGTG

>5784 len=135 tot-seq=1

GGGGGAATATGGCGAAAGGGGGGAAACCAGGAAGCAGCAACGACGAGGGAGGGATGAAGG

CAGTCGGGTTGGAAAACGCTGGCAGAAGGGAAGACATCGAAGGGACCTGCAGAAGAAGAC

CCGGCTAACTACGTG

>5785 len=135 tot-seq=1

TGGGGAATATTGCACAATGGGCGAAAGCCTGATGCAGCAACGCCGCGTGAGCGAAGAAGG

CCTTCGGGTTGTAAAGCTCTGTCCTTAGGGAAGATAATGACGGTACCTGAGGAGGAAGCC

CCGGCTAACTACGTG

>5786 len=135 tot-seq=1

TGGGGAATATTGCACAATGGGGGAAACCCTGATGCAGCGACGCCGCGTGGAGGAAGAAGG

TCTTCGGATTGTAAACTCCTGTCGTTGGGGACGATAATGACGGTACCCAACAAGAAAGCC

ACGGCTAACTACGTG

>5787 len=135 tot-seq=1

TGGGGAATATTGCACAATGGGGGGAACCCTGATGCAGCAACGCCGCGTGGGTGAAGAAGT
ATTTCGGTATGTAAAGCCCTGTAAGCAGGGAAGAAGGAGACGGTACCTGACCAAGAAGCC
CCGGCTAACTACGTG
>5788 len=135 tot-seq=1
TAGGGAATCTTCCACAATGGACGCAAGTCTGATGCAGCGACGCCGCGTGAGTGAAGAAGT
ATTTCGGTATGTAAAGCTCTATCAGCAGGGAAGATAATGACGGTACCTGACTAAGAAGCA
CCGGCTAAATACGTG
>5789 len=135 tot-seq=1
TAGGGAATCTTCGGCAATGGACGGAAGTCTGACCGAGCAACGCCGCGTGAGTGAAGAAGG
TTTTCGGGTTGTAAAAATCTTTTGTATGGGAAGAAAATGACAGTACCATACGAATAAGGA
CCGGCTAATTACGTG
>5790 len=135 tot-seq=1
AGTCTGATGGAGCAACGCCGCGTGGGTGAAGGAGGGCCTCGGCTCGTAAAGCTCTGTTGT
TGGTGAAGAAGGACGGGGGTAGTAACTGACCTTTGTTTGACGGTAATCAATTAGAAAGTC
ACGGCTAACTACGTG
>5791 len=135 tot-seq=1
TGAGGAATCTTCCACAATGGACGAAAGTCTGATGGAGCAACTCCGCGTGCAGGATGAAGG
CCTTCGGGTCGTAAACTGCTTTTATGAGTGAAGAATATGACGGTAACTCATGAATAAGGG
TCGGCTAACTACGTG
>5792 len=135 tot-seq=1
TGGGGAATTTTGCACAATGGGGGAAACCCTGATGCAGCGACGCCGCGTGAATGAAGAAGG
CCTTCGGGTTGTAAAATTCTGTCCTTAGTGAAGATAATGACAGTAACTAAGAAGCAAGCC
CTGGCTAAATACGTG
>5793 len=135 tot-seq=1
TGGGGAATATTGGACAATGGGGGAAACCCTGATCCAGCGACGCCACGTGAAGGAAGAAGT
ATCTCGGTATGTAAACTTCTATCAGCAGGGAAGATAATGACGGTACCTGACTAAGAAGCC
CCGGCTAACTACGTG
>5794 len=135 tot-seq=1
TGGGGGATATTGCACAATGGGGGAAACCCTGATGCAGCGACGCCGCGTGAGTGAAGACGG

CCTTCGGGTTGTAAAGCTCTGTCAACGGGGACGATAATGACGGTACCCGTGAAGGAAGCC

ACGGCTAACTACGTG

>5795 len=135 tot-seq=1

TGGGGAATATTGCGCAATGGGGGAAACCCTGACGCAGCAACGCCGCGTGCGGGATGAAGG

CCTTCGGGTTGTAAACCGCTTTCAGCAGGGAAGACATCGACGGTACCTTTCGAATAAGCG

CCGGCTAACTACGTG

>5796 len=135 tot-seq=1

TGGGGAATATTGGACAATGGAGGAAACTCTGATCCAGCAACGCCGCGTGCGGGATGAAGG

CCTTCGGGTTGTAAACCGCTTTCAGCAGGGAAGACATCGACGGTACCTGCAGAAGAAGCC

CCGGCTAACTACGTG

>5797 len=135 tot-seq=1

TGGGGAATATTGCGCAATGGGGGAAACCCTGACGCAGCGACGCCGCGTGCGGGATGAAGG

CCTTCGGGTTGTAAACCGCTTTCAGCAGGGAAGACATCGACGGTACCTAACCAGAAAGCC

ACGGCTAACTACGTG

>5798 len=135 tot-seq=1

TGGGGAATATTGCACAATGGGGGAAACCCTGATGCAGCGACGCCGCGTGAGCGAAGAAGG

CTTTCGAGTCGTAAAGCTCTGTCCTATGAGAAGATAGTGACGGTACCGAAAAAGCAAGCC

ACGGCTAACTACGTG

>5799 len=135 tot-seq=1

TGGGGAATATTGCACAATGGGCGCAAGCCTGATGCAGCAACGCCGCGCGAGCGAAGAAGT

ATTTCGGTATGTAAAGCTCTATCAGAAGGGGAGAAAATGACGGTACCTTACTAAGAAGCT

CCGGCTAAATACGTG

>5800 len=135 tot-seq=1

TGGGGAATATTGCACAATGGGCGAAAGCCTGGTGCAGCGACGCCGCGTGAGCGAAGAAGT

ATTTCGGTACGTAAAGCTTTATCAGAAGGGAGGAAAATGACGGTACCTTACTAAGAAGCT

CCGGCTAAATACGTG

>5801 len=135 tot-seq=1

TGGGGAATCTTGGACAATGGGGGAAACCCTGATCCAGCCATGCCGCGTGATCGATGAAGG

CCTTAGGGTTGTAAAGATCTTTCAGCTGGGAAGATAATGACTGTACCAGCAGAAGAAGCC

CCGGCTAACTCCGTG

>5802 len=135 tot-seq=1

TGGGGAATATTGGGCAATGGGCGAAAGCCTGACCCAGCGACGCCGCGTGAAGGAAGAAGG

CCTTCGGGTTGTAAACTTCTTTTATTAGGGACGATAGTGACGGTACCTAATGAATAAGCC

ACGGCTAACTACGTG

>5803 len=135 tot-seq=1

TGGGGAATATTGCGCAATGGGGGAAACCCTGACGCAGCGACGCCGCGTGCGGGATGAAGG

CCTTCGGGTTGTAAACCGCTTTTGACAAGCTTTCGGGTGAGTGTACCTTTCGAATAAGCG

CCGGCTAACTACGTG

>5804 len=135 tot-seq=1

TGGGGAATATTGGACAATGGGCAAAAGCCTGATCCGGCAATGCCGCGTGAATGATGAAGG

CCTTAGGGTTGTAAAGCTCTTTTACCCGGGATGATGATGACAGTACCGGAAGAATAAGCT

CCGGCTAACTCCGTA

>5805 len=135 tot-seq=1

TAGGGAATCTTCCACAATGGGCGGAAGTCTGACCGAGCAACGCCGCGTGAACGATGAAGG

TCTTCGGATCGTAAAGTTCTGTTGCAGGGGAAGATAATGACGGTACCCTGTGAGGAAGCC

CCGGCTAACTACGTG

>5806 len=135 tot-seq=1

TAGGGAATCTTCGGCAATGGACGGAAGTCTGACCGAGCAACGCCGCGTGAACGAAGAAGG

CTTTCGAGTCGTAAAGTTCTTTTATATGGGAAGATAATGACGGTACCATAAGAAAAAGCC

CCGGCTAACTACGTG

>5807 len=135 tot-seq=1

TGGGGAATCTTCCACAATGGGCGCAAGCCTGATGGAGCAACACCGCGTGAGTGAAGAAGG

CTTTCGAGTCGTAAAGTTCTTTTATATGGGAAGATAATGACGGTACCATAAGAAAAAGCC

CCGGCTAACTACGTG

>5808 len=135 tot-seq=1

TGGGGAATATTGCACAATGGGCGCAAGCCTGATGCAGCAACGCCGCGTGAACGATGAAGG

TCTTCGGATCGTAAAGTTCTGTTGCAGGGGAAGATAATGACGTTACCCGCAGAAGAAGCA

CCGGCTAACTCCGTG

>5809 len=135 tot-seq=1

TGGGGAATATTGCACAATGGGGGAAACCCTGATGCAGCGACGCCGCGTGAGCGAAGAAGT

ATTTCGGTATGTAAAGCTCTATCAGCAGGGAAGATAATGACGGTACCTTTCGAATAAGCG

CCGGCTAACTACGTG

>5810 len=135 tot-seq=1

TAGGGAATCTTCCACAATGGACGCAAGTCTGATGGAGCAACGCCGCGTGAGTGAAGAAGG

TTTTCGGATCGTAAAGCTCTGTTGTTGGTGAAGAAGGATAGAGGTAGTAACCAGAAAGGG

ACGGCTAACTACGTG

>5811 len=135 tot-seq=1

TAGGGAATCTTCCACAATGGACGCAAGTCTGATCCAGCCATGCCGCGTGAGTGATGAAGG

CCTTAGGGTTGTAAAGCTCTTTCACCGATGAAGATAATGACGGTAGTCGGAGAAGAAGCC

CCGGCTAACTTCGTG

>5812 len=135 tot-seq=1

TAGGGAATCTTCCACAATGGACGCAAGTCTGATGGAGCAACGCCGCGTGAGTGAAGAAGT

ATTTCGGTATGTAAAGCTCTATCAGTAGGGAAGATAATGACGGTACCTACATAAGAAGCC

CCGGCTAAATACGTG

>5813 len=135 tot-seq=1

TGGGGAATATTGGACAATGGGCGCAAGCCTGATCCAGCCATGCCGCGTGAGTGATGAAGG

CCTTAGGGTTGTAAAGCTCTTTCACCGGAGAAGATAATAACAGTATCCGGAGAAGAAGCC

CCGGCTAACTTCATG

>5814 len=135 tot-seq=1

TGGGGAATATTGGACAATGGGCGCAAGCCTGATCCAGCCATGCCGCGTGAGTGATGAAGG

CCTTAGGGTTGTAAAGCTCTTTCACCGATGAAGATAATGACGGTAATCAATTAGAAAGTC

ACGGCTAACTACGTG

>5815 len=135 tot-seq=1

TGGGGAATACTGTACAATGGGGGAAACCCTGATGCAGCGACGCCACGTGAACGAAGAAGG

TATTCGTATCGTAAAGTTCTGTCCTATGGGAAAATAATGACAGTACCATAGAAGAAAGCT

CCGGCTAAATACGTG

>5816 len=135 tot-seq=1

TGGGGAATATTGCACAATGGGCGAAAGCCTGATGCAGCGATGCCGCGTGAGGGAAGACGG

TTTTCGGATTGTAAACCTCTGTCTTGTGGGACGATAATGACGGTACCACAGGAGGAAGCC

ATGGCTAACTACGTG

>5817 len=135 tot-seq=1

TGGGGAATATTGCACAATGGGGGAAACCCTGATGCAGCAACGCCGCGTGAGCGATGAAGG

CCTTCGGGTCGTAAAGCTCTGTCCTAAGGGAAGATAATGACGGTACCTTAGGAGGAAGCC

CCGGCTAACTACGTG

>5818 len=135 tot-seq=1

TAGGGAATCTTCCACAATGGACGCAAGTCTGATGCAGCGACGCCGCGTGAGCGAAGAAGG

CTTTCGAGTCGTAAAGCTCTGTCCTATGAGAAGATAATGACGGTATCATAGGAAGAAGCC

CTGGCTAAATACGTG

>5819 len=135 tot-seq=1

TAGGGAATCTTCCACAATGGACGCAAGTCTGATGGAGCAACGCCGCGTGAGTGAAGAAGG

GTTTCGAGTCGTAAAGCTCTGTCCTATGGGAAGATAATGACGGTACCATAGAAGAAAGCC

CCGGCTAATTACGTG

>5820 len=135 tot-seq=1

TGGGGAATTTTGCACAATGGAGGAAACTCTGATGCAGCGACGCCGCGTGAACGAAGAAGG

TCTTCGGATTGTAAAGTTCTGTCCTGGGTGAAGATAATGACGGTAATCAATTAGAAAGTC

ACGGCTAACTACGTG

>5821 len=135 tot-seq=1

TAGGGAATCTTCCGCAATGGACGCAAATCTGACGGAGCAACGCCGCGTGAACGATGAAGG

TCTTCGGATCGTAAAGTTCTGTTGCAGGGGAAGATAATGACGGTACCCTGTGAGGAAGCC

CCGGCTAATTCCGTG

>5822 len=135 tot-seq=1

TGGGGAATATTGCACAATGGGGGAAACCCTGATGCAGCGACGCCGCGTGGAGGAAGAAGG

TCTTCGGATCGTAAAGTTCTGTTGCAGGGGAAGATAATGACGGTACCCTGTGAGGAAGCC

CCGGCTAACTACGTG

>5823 len=135 tot-seq=1

TGGGGAATATTGCGCAACGGGGGAAACCCTGACGCAGCGACGCCGCGTGAGTGAAGAAGT

AGTTCGCTATGTAAAGCTCTATCAGCAGGGAAGATAGTGACGGTACCTGACTAAGAAGCT

CCGGCTAAATACGTG

>5824 len=135 tot-seq=1

TGGGGGATATTGCACAATGGAGGAAACTCTGATGCAGCGACGCCGCGTGAAGGAAGAAGT

ATCTCGGTATGTAAACTTCTATCAGCAGGGAAGATAGTGACGGTACCCGACTAAGAAGCC

CCGGCTAACTAAGTG

>5825 len=135 tot-seq=1

TGGGGAATACTGCACAATGAGGGAAACCCTGATGCAGCGACGCCGCGTGAGCGGAGAAGT

ATTTCGGTATGTAAAGCTCTATCAGCAGGGAAGAAAATGACGGTACCTGACTAAGAAGCA

CCGGCTAAATACGTG

>5826 len=135 tot-seq=1

TGGGGAATATTGCACAATGGGGGGAACCCTGATGCAGCGACGCCGCGTGAGGGAAGAAGG

TTTTCGGATTGTAAACCTCTGTCTTGTGGGACGATAATGACGGTACCACAGGAGGAAGCC

ATGGCTAACTACGTG

>5827 len=135 tot-seq=1

TAGGGAATCTTCCACAATGGACGCAAGTCTGATGGAGCAACGCCGCGTGCGGGATGAAGG

CCTTCGGGTTGTAAACCGCTTTCAGCAGGGAAGACATCGACGGTACCTGCAGAAGAAGCC

CCGGCTAACTACGTG

>5828 len=135 tot-seq=1

TGGGGAATATTGCACAATGGGGGAAACCCTGATGCAGCGACGCCGCGTGAGCGATGAAGT

ATCTCGGTATGTAAACTTCTATCAGCAGGGAAGATAGTGACGGTACCTGACTAAGAAGCC

CCGGCTAGCTACGCA

>5829 len=135 tot-seq=1

TGGGGAATATTGCACAATGGGGGAAACCCTGATGCAGCGACGCCGCGTGGAGGAAGAAGG

TCTTCGGATTGTAAACTCCTGTTGTTGAGGAAGATAATGACGGTACCCGCAGAAGAAGCA

CCGGCTAACTCCGTG

>5830 len=135 tot-seq=1

TGGGGAATATTGCACAATGGGGGAAACCCTGATGCAGCGACGCCGCGTGGAGGAAGAAGG

TCTTCGGATTGTAAACTCCTGTTGTTGAGGAAGATAATGACGGTATTTAACCAGAAAGCC

ACGGCTAACTACGTG

>5831 len=135 tot-seq=1

TGGGGAATATTGCGCAATGGGGGAAACCCTGACGCAGCGACGCCGCGTGCGGGATGAAGG

TATTCGTATCGTAAAGTTCTGTCCTATGGGAAGATAATGACAGTACCATAGAAGAAAGCT

CCGGCTAAATACGTG

>5832 len=135 tot-seq=1

TGGGGAATATTGCGCAATGGGGGAAACCCTGACGCAGCAACGCCGCGTGATTGAAGAAGT

ATCTCGGTATGTAAAGCTCTATCAGCAGGGAAGAAAATGACGGTACCTGACTAAGAAGCC

CCGGCTAACTACGTG

>5833 len=135 tot-seq=1

TGGGGAATATTGCACAATGGGGGAAACCCTGATGCAGCGACGCCGCGTGAACGAAGAAGG

TATTCGTATCGTAAAGTTCTGTCCTATGGGAAGATAATGACAGTACTCAACAAGGAAGTG

ACGGCTAACTACGTG

>5834 len=135 tot-seq=1

TAGGGAATCTTCCACAATGGACGAAAGTCTGATGGAGCAACGCCGCGTGAGTGAAGAAGT

ACCTCGGTATGTAAAGCTCTATCAGCAGGGAAGAAAGTGACAGTACCTGACTAAGAAGCC

CCGGCTAACTACGTG

>5835 len=135 tot-seq=1

TGAGGAATCTTCCACAATGGGGGCAACCCTGATGGAGCAACGCCGCGTGCAGGATGAAGG

CCTTCGGGTTGTAAACTGCTTTTATTTGTGAAGAATATGACGGTAACAAATGAATAAGGG

TCGGCTAACTACGTG

>5836 len=135 tot-seq=1

TAAGGAATCTTCCACAATGGACGAAAGTCTGATGGAGCAACTCCGCGTGCAGGATGAAGG

CCTTCGGGTTGTAAACTGCTTTTATTTGTGAAGAATATGACGGTAACAAATGAATAAGGG

TCGGCTAACTACGTG

>5837 len=135 tot-seq=1

GCGCGCAAATTACCCAATCCCGACACGGGGAGGTAGTGACAATAAATAACAATATAGGGC

TCTATTGGGTCTTATAATTGGAATGAGTACAATTTAAATCCCTTAACGAGGAACAACTGG

AGGGCAAGTCTGGTG

>5838 len=135 tot-seq=1

TCGGGAATATTGCGCAATGGAGGAAACTCTGACGCAGTGACGCCGCGTGCAGGAAGAAGG

TTTTCGGATTGTAAACTGCTTTAGACAGGGAAGATGATGACAGTACCTGTAGAATAAGCT

CCGGCTAACTACATG

>5839 len=135 tot-seq=1

TGGGGGATATTGCACAATGGGGGAAACCCTGATGCAGCGACGCCGCGTGGAGGAAGACGG

CCTTCGGGTTGTAAACTCCTGTCTTCAGGGACGAAACTGACGGTACCTAAGGAGGAAGCA

CCGGCTAACTACGTG

>5840 len=135 tot-seq=1

TGGGGAATACTGCACAATGGGGGAAACCCTGATGCAGCGACGCCGCGTGAGTGAAGAAGT

ATTTCGGTATGTAAACTTCTATCAGCAGAGAAGAAACTGACGGTACCTGACTAAGAAGCA

CCGGCTAAATACGTG

>5841 len=135 tot-seq=1

TGGGGAATATTGCACAATGGGGGAAATCCTGATGCAGCGACGCCGCGTGAGCGATGAAGT

ATTTCGGTATGTAAAGCTCTATCAGCAGGGAAGAAAATGACGGTACCTGACTAAGAGGCC

CTGGCTAACTACGTG

>5842 len=135 tot-seq=1

TGGGGGATATTGCACAATGGGGGGAACCCTGATGCAGCGACGCCGCGTGAGTGAAGAAGT

CATTCGTGATGTAAAGCTCTATCAGCAGGGACGAAAATGACGGTACCTGACTAAGAAGCC

CCGGCTAACTACGTG

>5843 len=135 tot-seq=1

GCATTATAAATGTGGCCCCCAACTGCCTTTTCCTCCAACCTCACGCATTTACTGCCTCCC

TGCTGGTCTCCACCCACAGCCGCTCTTCCCGGGAACCTGGAGCTGCCCTCCCGGACCCCA

GGTGGTCCCCTCCTG

>5844 len=135 tot-seq=1

GCCAAGGTGTAATGTCCTACAGAGACCAATACAAGGAGGCTCTCAACATGCCAGCAGAAA

AGACAGAGGTGTCAGTTACTTACCGAACCTTCTCAAGTAGCTGGTGATAGTGCTGGAGCT

CCAGGGTGACATGGG

>5845 len=135 tot-seq=1

TGGGGAATATTGCGCAATGGGCGAAAGCCTGACGCAGCGACGCCGCGTGCGGGATGGAGG
CCCTCGGGTCGTAAACCGCTTTCGGCAGGGACGATTGTGACGGTACCTGCATAAGAAGCC
CCGGCTAAATACGTG
>5846 len=135 tot-seq=1
TAGGGAATTTTGCGCAATGGGCGAAAGCCTGACGCAGCAACGCCGCGTGAACGAGACGCC
CTTCGGGGTGTAAAGTTTTGTCAGTAGGGACGAAAGATGACGGTACCTACAGAGGAAGCA
CCGGCTAACTCCGTG
>5847 len=135 tot-seq=1
TGGGGAATCTTGGACAATGGGCGCAAGCCTGATCCAGCCATGCCGCGTGAGTGACGAAGG
CCTTAGGGTTGTAAAGCTCTTTTAGCAGGGAAGATAATGACGGTACCTGCAGAATAAGCC
CCGGCTAACTTCGTG
>5848 len=135 tot-seq=1
GCGCGCAAATTACCCAATCCCGACACGGGGAGGTAGTGACAATAAATAACAATATGGGGC
TCTTTCGGGTCTCATAATTGGAATGAGTACAATTTAAATCTCTTAACGAGGAACAATTGG
AGGGCAAGTCTGGTG
>5849 len=135 tot-seq=1
TGGGGAATATTGCGCAATGGGGGAAACCCTGACGCAGCAACGCCGCGTGAAGGATGAAGG
TTTTCGGATTGTAAACTTCTTTGATTGGGGACGATAATGACGGTACCCAAAAAACAAGCC
ACGGCTAACTACGTG
>5850 len=135 tot-seq=1
TGGGGAATATTGGACAATGGGGGCAACCCTGATCCAGCAATGCCGCGTGAGTGAAGAAGG
CCTTAGGGTTGTAAAGCTCTTTCGACGGGGACGATGATGACGGTACCCGTAGAAGAAGCC
CCGGCTAACTCCGTG
>5851 len=135 tot-seq=1
TGGGGAATATTGCGCAATGGGGGAAACCCTGATGCAGCGACGCCGCGTGAGCGAAGAAGG
CCTTCGGGTCGTAAAACTCTTTTATATGGGAAGATAATGACGGTACCATAAGAATAAGGA
CCGGCTAATTACGTG
>5852 len=135 tot-seq=1
TGGGGGATATTGCACAATGGAGGCAACTCTGATGCAGCAACGCCGCGTGAACGAAGAAGG

TATTCGTATCGTAAAGTTCTGTCCTATGGGAAGATAATGACAGTACCATAGAAGAAAGCT

CCGGCTAAATACGTG

>5853 len=135 tot-seq=1

TGGGGAATATTGCACAATGGACGAAAGTTTGATGCAGCGACGCCGCGTGAGCGATGAAGG

CTTTCGAGTCGTAAAGCTCTGTCCTATGGGAAGATAATGACGGTACCATAGAAGAAAGCC

CCGGCTAATTACGTG

>5854 len=135 tot-seq=1

TGGGGAATATTGCACAATGGACGAAAGTTTGATGCAGCGACGCCGCGTGAGCGAAGAAGG

CCTTCGGGTCGTAAAGCTCTTTTATATGGGAAGATAATGACGGTACCATAAGAAAAAGCC

CCGGCTAACTACGTG

>5855 len=135 tot-seq=1

TAGGGAATCTTCGGCAATGGACGGAAGTCTGACCGAGCAACGCCGCGTGAGTGAAGAAGG

TTTTCGAGTCGTAAAGCTCTGTCCTATGAGAAGATAATGACGGTATCATAGGAGGAAGCC

CTGGCTAAATACGTG

>5856 len=135 tot-seq=1

TGGGGAATTTTGCACAATGGAGGAAACTCTGACGCAGCGACGCCGCGTGAACGAAGAAGG

TCTTCGGATTGTAAAGTTCTGTCCTGGGTGAAGATAATGACGGTAACTTACCAGAAAGGG

ACGGCTAACTACGTG

>5857 len=135 tot-seq=1

TGGGGAATATTGCACAATGGAGGAAACTCTGATGCAGCGACGCCGCGTGAGCGATGAAGG

CTTTCGAGTCGTAAAGCTCTGTCCTATGGGAAGATAATGACGGTACCATATGAATAAGGA

CCGGCTAATTCCGTG

>5858 len=135 tot-seq=1

TGGGGAATATTGCGCAATGGGGGAAACCCTGACGCAGCGACGCCGCGTGAGCGAAGAGGG

CTTTCGAGTCGTAAAGCTCTGTCCTATGAGAAGATGATGACGGTATCATAGGAGGAAGCC

CTGGCTAAATACGTG

>5859 len=135 tot-seq=1

TGGGGAATATTGGGCAATGGGCGCAAGCCTGACCCAGCAACGCCGCGTGAAGGAAGAAGG

CTTTCGGGTTGTAAACTTCTTTTATCAGGGACGATAATGACGGTACCTGATGAATAAGCT

CCGGCTAACTACGTG

>5860 len=135 tot-seq=1

TGGGGAATCTTCCGCAATGGACGAAAGTCTGACGGAGCAACGCCGCGTGAGTGATGACGG

CTTTCGAGTCGTAAAGCTCTGTCCTATGGGAAGATAATGACGGTACCATAGAAGAAAGCC

CCGGCTAATTACGTG

>5861 len=135 tot-seq=1

TGGGGAATATTGGACAATGGAGGGAACTCTGATCCAGCAATTCTGTGTGTGTGAAGAAGG

TTTTAGGACTGTAAAACACTTTTAGTAGGGAAGATAATGACGGTACCGGGAGAATAAGCC

CCGGCTAACTCCGTG

>5862 len=135 tot-seq=1

TGGGGAATATTGCACAATGGAGGAAACTCTGATGCAGCGACGCCGCGTGAATGAAGAAGG

CTTTCGAGTCGTAAAATTCTGTCCTAGGGGACGATAATGACGGTACCCTAGGAGGAAGCC

CCGGCTAATTACGTG

>5863 len=135 tot-seq=1

TGAGGAATATTGGTCAATGGGGGGAACCCTGATGCAGCAACGCCGCGTGAGTGATGAAGG

TTTTCGGATCGTAAAGCTCTGTCTTTGGGGAAGATAATGACGGTACCCAAGGAGGAAGCC

ACGGCTAACTACGTG

>5864 len=135 tot-seq=1

TGGGGAATTTTGGACAATGGGGGCAACCCTGATGCAGCGACGCCGCGTGAACGAAGAAGG

TATTCGTATCGTAAAGTACTGTCCTATGGGAAGATAATGACAGTACCATAGAAGAAAGCT

CCGGCTAAATACGTG

>5865 len=135 tot-seq=1

TGGGGAATATTGCACAATGGGGGAAACCCTGATGCAGCGACGCCGCGTGGACGAGGAAGT

ACCTCGGTATGTAAAGTCCTATCAGCAGGGAAGAAAGTGACAGTACCTGACTAAGAAGCC

CCGGCTAACTACGTG

>5866 len=135 tot-seq=1

TGGGGAATCTTGCACAATGGGCGAAAGCCTGATGCAGCGACGCCGCGTGCGGGATGAAGG

CCTTCGGGTTGTAAACCGCTTTCAGCAGGGACGAAAATGACGGTACCTTACTAAGAAGCT

CCGGCTAAATACGTG

>5867 len=135 tot-seq=1

TGGGGAATATTGCACAATGGGGGGAACCCTGATGCAGCGACGCCGCGTGGGTGAGGAAGT

ACCCCGGTATGTAAAACCCTTTCGGCAGGGAAGAAAATGACGGTACCTGGCGAAGAAGCC

CCGGCTAACTACGTG

>5868 len=135 tot-seq=1

CGGAAGGCTGAACCAGCCAAGTCGCGTGAGGGAATAAGGCCCTACGGGTCGTAAACCTCT

TTTGTCGGGGAGCAAATTCCGCCACGTGTGGCGGAGTCGAGAGTACCCGAAGAAAAAGCA

TCGGCTAACTCCGTG

>5869 len=135 tot-seq=1

TGGGGGATATTGCACAATGGGGGAAACCCTGATGCAGCGACGCCGCGTGAGCGATGAAGT

ATTTCGGTATGTAAAGCTCTATCAGCAGGAAAGAAGATGACGGTACCTGACTAAGAAGCC

CCGGCTAAATACGTG

>5870 len=135 tot-seq=1

TGGGGAATATTGCACAATGGGCGCAAGCCTGATGCAGCGATGCCGCGTGGAGGAAGAAGG

TTTTCGGATTGTAAACTCCTGTCTTAAGGGACGATAATGACGGTACCTTAGGAGGAAGCT

CCGGCTAACTACGTG

>5871 len=135 tot-seq=1

TGGGGAATATTGCACAATGGGGGAAACCCTGATGCAGCGACGCCGCGTGAAGGATGAAGT

ATTTCGGTATGTAAAGCTCTATCAGAAGGGAAGAAAATGACGGTACCTTACTAAGAAGCT

CCGGCTAAATACGTG

>5872 len=135 tot-seq=1

TCGGGAATATTGCGCAATGGAGGAAACTCTGACGCAGTGACGCCGCGTATAGGAAGAAGG

TTTTCGGATTGTAAACTATTTTAGACAGGGAAGGAATTGACAGTACCTGTAGAATAAGCT

CCGGCTAACTACGTG

>5873 len=135 tot-seq=1

TGGGGAATATTGCGCAATGGGGGCAACCCTGACGCAGCAACGCCGCGTGAAGGATGACGG

TCTTCGGATTGTAAACTTCTTTAAGTGTGGAAGAAATTGACGGTACACACAGAATAAGCC

ACGGCTAACTACGTG

>5874 len=135 tot-seq=1

TGGGGGATATTGCACAATGGGGGGAACCCTGATGCAGCGACGCCGCGTGGGCGATGGAGT
ACCCCGGTACGTAAAGCCCTGTCAGCAGGGAAGACAATGACGGTACCTGACCAAGAAGCC
CCGGCTAACTACGTG
>5875 len=135 tot-seq=1
TGGGGAATATTGCACAATGGGCGAAAGCCTGACGCAGCGACGCCGCGTGAGCGAAGAAGT
ATTTCGGTATGCAAAGCCCTACCAGAAGGGAAGAAAATGACGGTACCTTACTAAGAAGCT
CCGGCTAAATACGTG
>5876 len=135 tot-seq=1
TGGGGAATATTGCGCAATGGGGGAAACCCTGACGCAGCGACGCCGCGTGCGGGATGAAGT
ATTTCGGTATGTAAAGCTCTATCAGCAGGGAAGAAGATGACGGTACCTGAATAAGAAGCC
CCGGCTAACTACGTG
>5877 len=135 tot-seq=1
TGGGGAATATTGCACAATGGGGGAAACCCTGATGCAGCGACGCCGCGTGGGTGAAGGAGT
ATTTCGGTATGTAAAGCCCTATCAGCAGGGAAGATAATGACGGTACCTGGCTAAGAAGCC
CCGGCTAACTACGTG
>5878 len=135 tot-seq=1
TAGGGAATATTGCACAATGGGGGCAACCCTGATGCAGCGACGCCGCGTGAGCGATGGAGT
ACTTCGGTATGTAAAGCTCTGTCAGCAGGGAAGACATTGACGGTACCTGACCAAGAAGCC
CCGGCTAAATACGTG
>5879 len=135 tot-seq=1
TGGGGAATATTGGACAATGGGGGAAACCCTGATCCAGCGACGCCGCGTGAGTGAAGAAGT
ATTTCGGTATGTAAACTTCTATCAGCAAGGAAGAAAATGACGGTACTTGACTAAGAAGCC
CCGGCTAAATACGTG
>5880 len=135 tot-seq=1
TGGGGAATATTGGACAATGGGGGAAACCCTGATCCAGCGACGCCGCGTGAGGGAAGAAGG
TTTTCGGATTGTAAACCTCTGTCTTAAGGGACGATAATGACGGTACCTTAGGAGGAAGCT
CCGGCTAACTACGTG
>5881 len=135 tot-seq=1
TGGGGAATATTGCACAATGGGCGAAAGCCTGATGCAGCAACGCCGCGTGAGTGATGAAGG

CCTTCGGGTTGTAAAGCTCTGTCTTCTGGGACGATAATGACGGTACCAGAGGAGGAAGCC

ACGGCTAACTACGTG

>5882 len=135 tot-seq=1

TGGGGAATATTGCGCAATGGGCGAAAGCCTGACGCAGCAACGCCGCGTGCGGGATGACGG

CCTTCGGGTTGTAAACCGCTTTCAGCAGGGACGAAACTGACGGTACCTGCAGAAGAAGCA

CCGGCTAACTACGTG

>5883 len=135 tot-seq=1

TGGGGAATATTGCACAATGGGGGAAACCCTGATGCAGCGACGCCGCGTGAGTGAAGAAGT

ATTTCGGTATGTAAAGCTCTATCAGCGGGGAAGAAGATGACAGTACCCGACCAAGAAGCC

CCGGCTAACTACGTG

>5884 len=135 tot-seq=1

TGGGGAATATTGCACAATGGGGGAAACCCTGATGCAGCGACGCCGCGTGAGCGAAGAAGC

GCTTCGGCGCGTAAAGCTCTATCAGCAGGGAAGAAAGCGACAGTACCTGACTAAGAAGCC

CCGGCTAACTACGTG

>5885 len=135 tot-seq=1

TGGGGGATATTGCACAATGGGGGGAACCCTGATGCAGCGACGCCGCGTGAGTGAAGAAGT

GCTTCGGCATGTAAAGCCCTGTCAGCAGGGAAGAAGGTGACGGTACCTGACCAAGAAGCC

CCGGCTAACTACGTG

>5886 len=135 tot-seq=1

TGGGGGATATTGCACAATGGGGGAAACCCTGATGCAGCAACGCCGCGTGTGGGAAGAAGG

TTTTCGGATTGTAAACCACTGTTCTCAGGGACGATAATGACGGTACCTGAGGAGAAAGCT

CCGGCTAACTACGTG

>5887 len=135 tot-seq=1

TGGGGAATATTGCGCAATGGGGGAAACCCTGACGCAGCCATGCCGCGTGAGTGATGAAGG

CCCTAGGGTTGTAAAGCTCTTTCAACGGTGAAGATAATGACGGTAACCGTAGAAGAAGCC

CCGGCTAACTTCGTG

>5888 len=135 tot-seq=1

TGGGGAATCTTGCGCAATGGGCGAAAGCCTGACGCAGCAACGCCGCGTGGGGGATGAAGG

CTTTCGGGTTGTAAACCCCTTTCAGCAGGGACGATTATGACGGTACCTGCAGAAGAAGGA

CCGGCCAACTACGTG

>5889 len=135 tot-seq=1

TGAGGAATATTGGACAATGGGCGAAAGCCTGATCCAGCAATACCGCGTGAGTGAAGAAGG

CCTTCGGGTTGTAAAGCTCTTTCAGCAGGGAAAATAATGATGGTACCTGCAAAAGAAGTC

CCGGCTAACTCCGTG

>5890 len=135 tot-seq=1

TGGGGAATATTGCACAATGGGGGGAACCCTGATGCAGCGACGCCGCGTGAGGGAAGAAGG

TATTCGTATTGTAAACCTCTGTCTTCGGGGACGATAATGACGGTACCCGAGGAGGAAGCA

CCGGCTAACTACGTG

>5891 len=135 tot-seq=1

TGGGGAATATTGGACAATGGGCACAAGCCTGATCCAGCCATGCCGCGTGAGTGATGAAGG

CCTTAGGGTTGTAAAGCTCTTTTGTCAGGGAAGATGATGACGGTACCTGAAGAATAAGCA

CCGGCTAACTTCGTG

>5892 len=135 tot-seq=1

TAGGGAATCTTCGGCAATGGGGGGAACCCTGACCGAGCAACGCCGCGTGAGCGAAGAAGG

CCTTCGGGTCGTAAAGCTCTTTTATATGGGAAGATAATGACGGTACCATAAGAAAAAGCC

CCGGCTAACTACGTG

>5893 len=135 tot-seq=1

TGGGGAATATTGGACAATGGGCGAAAGCCTGATCCAGCCATGCCGCGTGAACGATGAAGG

TCTTCGGATCGTAAAGTTCTGTTGCAGGGGAAGATAATGACGGTACCCTGTGAGGAAGCC

CCGGCTAACTACGTG

>5894 len=135 tot-seq=1

TGGGGAATATTGCACAATGGGCGCAAGCCTGATGCAGCGACGCCGCGTGAGGGATGGAGG

TATTCGTATCGTAAAGTTCTGTCCTATGGGAAGATAATGACAGTACCATAGAAGAAAGCT

CCGGCTAAATACGTG

>5895 len=135 tot-seq=1

TGAGGAATATTGCACAATGGGGGAAACCCTGATGCAGCAACGCCGCGCGAGTGAAGAAGT

ATTTCGGTACGTAAAGCCCTATCAGCAGGAAAGAAAATGACGGTACCTGACTAAGAAGCC

CCGGCTAACTACGTG

>5896 len=135 tot-seq=1

TGGGGAATATTGGACAATGGGCGCAAGCCTGATCCAGCCATGCCGCGTGAGCGATGAAGG

CCTTAGGGTTGTAAAGCTCTTTCACCGATGAAGATAATGACGGTAGTCGGAGAAGAAGCT

CCGGCTAAATACGTG

>5897 len=135 tot-seq=1

TGGGGGATATTGCACAATGGGGGCAACCCTGATGCAGCGACGCCGCGTGAGCGATGAAGT

ATTTCGGTATGTAAACTTCTATCAGCAAGGAAGAAAATGACGGTACTTGACTAAGAAGCC

CCGGCTAAATACGTG

>5898 len=135 tot-seq=1

TGGGGAATATTGCACAATGGGCGAAAGCCTGATGCAGCGACGCCGCGTGAGTGATGAAGG

CCTTAGGGTTGTAAAGCTCTATCAGAAGGGAAGAAAATGACGGTACCTTACTAAGAAGCT

CCGGCTAAATACGTG

>5899 len=135 tot-seq=1

TGGGGAATATTGCGCAATGGGCGAAAGCCTGACGCAGCGACGCCGCGTGAGGGATGAAGT

ATTTCGGTATGTAAAGCTCTATCAGCAGGGAAGAAAATGACGGTACCTGACTAAGAAGCC

CCGGCTAACTACGTG

>5900 len=135 tot-seq=1

TGGGGAATATTGCACAATGGAGGGAACTCTGATGCAGCGACGCCGCGTGAACGAAGAAGG

CTTTCGAGTCGTAAAGTTCTTTTATATGGGAAGATAATGACGGTACCTTACTAAGAAGCT

CCGGCTAAATACGTG

>5901 len=135 tot-seq=1

TGGGGGATATTGGACAATGGGGGGAACCCTGATCCAGCGACGCCGCGTGAGTGAAGAAGT

AATTCGTTATGTAAAGCTCTATCAGCAGGGAAGAAAATGACGGTACCTGAGTAATAAGCC

CCGGCTAACTACGTG

>5902 len=135 tot-seq=1

TAAGGGATATTGCACAATGGGGGAAACCCTGATGCAGCGACGCCGCGTGAGCGATGAAGT

ATCTCGGTATGTAAAGCTCTATCAGCAGGGAAGAAAGTGACAGTACCTGACTAAGAAGCC

CCGGCTAACTACGTG

>5903 len=135 tot-seq=1

TGGGGAATTTTGCGCAATGGGCGAAAGCCTGACGCAGCAACGCCGCGTGCGGGACGAAGG

CCTTCGGGTTGTAAACCGCTTTCAGCAGGGAAGAGAACGACGGTACCTGCAGAAGAAGCT

CCGGCTAACTACGTG

>5904 len=135 tot-seq=1

TGAGGAATATTGCACAATGGGGGAAACCCTGATGCAGCAACGCCGCGTGAGGGAAGACGG

TTTTCGGATTGTAAACCTCTGTCCTTAGTGACGATAATGACGGTAGCTAAGGAGGAAGCC

ACGGCTAACTACGTG

>5905 len=135 tot-seq=1

TGGGGGATATTGCACAATGGGGGGGACCCTGATGCAGCGACGCCGCGTGAGTGAAGAAGT

ATTTCGGTACGTAAAGCTCAATCAGCAGGGAAGAAAATGACGGTACCTGACTAAGAAGCC

CCGGCTAACTACGTG

>5906 len=135 tot-seq=1

TGGGGCATATTGCACAATGGGGGGAACCCTGATGCAGCGACGCCGCGTGGGCGAGGAAGT

ATTTCGGTATGTAAAGCTCTATCAGCAGGGAAGAAAGTGACGGTACCTGAAAAAGAAGCT

CCGGCTAAATACGTG

>5907 len=135 tot-seq=1

TGGGGAATCTTGGACAATGGGGGAAACCCTGATCCAGCCATGCCGCGTGAGTGAAGAAGG

CCTTCGGGTTGTAAAGCTCTTTTGTCAGGGACGATAATGACGGTACCTGAAGAATAAGCC

CCGGCAAACTTCGTG

>5908 len=135 tot-seq=1

TGAGGAATATTGGTCAATGGACGAGAGTCTGAACCAGCCAAGTAGCGTGCAGGATGACGG

CCCTATGGGTTGTAAACTGCTTTTGTTTGGGACTAATCGACATTACCATACGAATAAGGA

CCGGCTAATTACGTG

>5909 len=135 tot-seq=1

TGAGGAATATTGGTCAATGGGGGGAACCCTGATGCAGCGACGCCGCGTGGGTGAAGGAGC

ACCTCGGTGCGTAAAGCCCTATCGGCAGGGAAGAAACAGACGGTACCTGACTAAGAAGCC

CCGGCTAACTACGTC

>5910 len=135 tot-seq=1

TGAGGGATATTGGTCAATGGGGGAAACCCTGAACCAGCAACGCCGCGTGAGGGATGACGG

CCTTCGGGTTGTAAACCTCTGTCCTCTGTGAAGATAATGACGGTAGCAGAGGAGGAAGCT

CCGGCTAACTACGTG

>5911 len=135 tot-seq=1

TGGGGAATATTGCACAATGGGGGAAACCCTGATGCAGCGACGCCGCGTGAGTGAAGACGG

CCTTCGGGTTGTAAAGCTCTGTTGACGGGGACGATAATGACGGTACCCGTTAAGAAAGCC

ACGGCTAACTACGTG

>5912 len=135 tot-seq=1

TGGGGAATATTGGACAATGGGGGGTACCCTGATCCAGCCATGCCGCGTGAGTGATGACGG

CCTTCGGGTTGTAAAGCTCTTTTACACGGGAAGATAATGACGGTACCGTGAGAATAAGCA

CCGGCTAACTTCGTG

>5913 len=135 tot-seq=1

TGGGGGATATTGCACAATGGAGGCAACTCTGATGCAGCAACGCCGCGTGAGTGAAGAAGG

CTTTCGAGTCGTAAAACTCTGTCGTATGGGAAGAAAATGACGGTACCTTACTAAGAAGCT

CCGGCTAAATACGTG

>5914 len=135 tot-seq=1

TGGGGAATCTTCCGCAATGGACGAAAGTCTGATGCAGCGACGCCGCGTGAGTGAAGAAGT

ATTTCGGTATGTAAAGCTCTATCAGCAGGGAAGAAGATGACGGTACCTGAATAAGAAGCC

CCGGCTAACTACGTG

>5915 len=135 tot-seq=1

TGGGGGATATTGCACAATGGAGGCAACTCTGATGCAGCAACGCCGCGTGAGTGAAGAAGT

ATTTCGGTATGTAAAGCTCTATCAGAAGGGAAGAAAATGACGGTACCTTACTAAGAAGCT

CCGGCTAAATACGTG

>5916 len=135 tot-seq=1

TGGGGAATATTGCACAATGGGGGGAACCCTGATGCAGCAACGCCGCGTGAGTGGTGAAGG

TATTCGGATCGTAAAGCTCTGTCTGTGGGGAAGATAATGACGGTACCCAAGGAGGAAGCC

ACGGCTAACTACGTG

>5917 len=135 tot-seq=1

TGGGGAATATTGCACAATGGGGGAAACCCTGATGCAGCGACGCCGCGTGAAGGAAGACGT

ATTTCGGTATGTAAACTTCTATCAGCAAGGAAGATGATGACGGTACCCAAGGAGGAAGCC

ACGGCTAACTACGTG

>5918 len=135 tot-seq=1

TGGGGAATATTGCGCAATGGGGGAAACCCTGACGCAGCGACGCCGCGTGAGTGATGAAGG

TTTTCGGATCGTAAAGCTCTGTCTTTGGGGAGGATAATGACGGTACCCAAGGAGGAAGCC

ACGGCTAACTACGTG

>5919 len=135 tot-seq=1

TAGGGAATCTTCCACAATGGGCGCAAGCCTGATGGAGCAACACCGCGTGAGTGAAGAAGG

GTTTCGGCTCGTAAAGCTCTGTTGTTGGAGAAGATAATGACGGTACCCAAGGAGGAAGCC

ACGGCTAACTACGTG

>5920 len=135 tot-seq=1

TAGGGAATTTTCGTCAATGGGGGGAACCCTGAACGAGCAATGCCGCGTGAGTGATGAAGG

TTTTCGGATCGTAAAGCTCTGTCTTTGGGGAAGATAATGACGGTACCCAAGGAGGAAGCC

ACGGCTAACTACGTG

>5921 len=135 tot-seq=1

TGGGGAATATTGGACAACGGGCGCAGGCCTGATCCAGCCATGCCGCGTGAGTGATGAAGG

CCTTAGGGTTGTAAAACTCTTTCACCGATGAAGATAATGACGGTAGTCGGAGAAGAAGCC

CCGGCTAACTTCGTG

>5922 len=135 tot-seq=1

TGGGGAATATTGCACAATGGGGGGAACCCTGATGCAGCAACGCCGCGTGAGTGATGAAGG

TTTTCGGATCGTAAACCTCTGTCTTGAGGGACGATAATGACGGTACCTCAGGAGGAAGCT

CCGGCTAACTACGTG

>5923 len=135 tot-seq=1

TGAGGGATATTGGTCAATGGGGGAAACCCTGAACCAGCAACGCCGCATGAGGGAAGACGG

TCTTCGGATTGTAAACCTTTGTCCTCCGTGAAGATAATGACGGTAGCAGAGGAGGAAGCT

CCGGCTAACTACGTG

>5924 len=135 tot-seq=1

TGGGGAATCTTGGACAATGGGGGAAACCCTGATCCAGCCATTCCGCGTGGATGATGAAGG

CCTTAGGGTTGTAAAATCCTTTCAGCAGGGAAGATAATGACTGTACCTGCAGAAGAAGCC

CCGGCTAACTCCGTG

>5925 len=135 tot-seq=1

TGGGGAATATTGCGCAATGGGGGAAACCCTGATGCAGCAACGCCGCGTGAGTGATGAAGG

CCTTCGGGTCGTAAAGCTCTTTGATCAGGGACGATAATGACGGTACCTGAAAAACAAGCC

ACGGCTAACTACGTG

>5926 len=135 tot-seq=1

TCGAGAATCATTCACAATGGGGGAAACCCTGATGCAGCGACGCCGCGTGAAGGAAGAAGT

ATTTCGGTATGTAAACTTCTATCAGCAGGGAAGAAAATGACGGTACCTGAGTAAGAAGCC

CCGGCTAACTACGTG

>5927 len=135 tot-seq=1

TGGGGGATATTGCACAATGGGGGAAACCCTGATGCAGCAATGCCGCGTGAGGGAAGAAGG

TCTTCGGATTGTAAACCTAAGTAGCCAGGGACGATAATGACGGTACCTGGAGAGTAAGCT

CCGGCTAACTACGTG

>5928 len=135 tot-seq=1

TAGGGAATCTTCCGCAATGGGGGAAACCCTGATGCAGCGACGCCGCGTGGAGGAAGAAGG

TTTTCGGATTGTAAACTCCTGTCGTTAGGGACGATAATGACGGTACCTAACAAGAAAGCA

CCGGCTAACTACGTG

>5929 len=135 tot-seq=1

TGGGGAATATCGCACAATGGGGGAAACCCTGATGCAGCGACGCCGCGTGAGCGAGGAAGT

ACCTCGGTATGTAAAGCTCTGTCAGCAGGGAAGAAAATGACGGTACCTGACTAAGAAGCC

CCGGCTAACTACGTG

>5930 len=135 tot-seq=1

TGGGGAATATTGCACAATGGAGGAAACCCTGATGCAGCGACGCCGCGTGAGCGAAGAAGT

ATCTCGGTATGTAGAGCTCTATCAGCAGGGAGGATAATGACGGTACCTGACTAAGAAGCC

CCGGCTAACTACGTG

>5931 len=135 tot-seq=1

GGGCCTGTCCACACTGCTGTGGGCTATGAGGTCCTGACACACAGTTCCTGCTCAGTCAGT

GCTTGCAGACAAAGACACGAGAGAGAGAAGCGTGGAGCCCAGCACCTCTCCTCCGCCTGG

GATCGCCCTGAACCC

>5932 len=135 tot-seq=1

TGGGGAATTTTGGACAATGGGGGCAACCCTGATGCAGCGACGCCGCGTGAGTGAAGGAGT

ATTTCGGTATGTAAAGCTCTATCAGTAGGGAAGATAATGACGGTACCTACATAAGAAGCC

CCGGCTAAATACGTG

>5933 len=135 tot-seq=1

TGGGGAATATTGGACAATGGGCGCAAGCCTGATCCAGCAATGCCGCGTGGGTGAAGAAGG

TCTTCGGATTGTAAAGCCCTTTCGACAGGGACGATGATGACGGTACCTGTAGAAGAAGCC

CCGGCTAACTTCGTG

>5934 len=135 tot-seq=1

TGGGGAATATTGGACAATGGAGGGAACTCTGATCCAGCAATTCTGTGTGTGTGAAGAAGG

TTTTAGGACTGTAAAGCTCTATCAGCAGGGAAGAAGATGACGGTACCTGAATAAGAAGCC

CCGGCTAACTACGTG

>5935 len=135 tot-seq=1

TAGGGAATCTTCCACAATGGACGCAACTCTGATGCAGCGACGCCGCGTGAGTGAAGAAGT

ATTTCGGTATGTAAAGCTCTATCAGCAGGGGAGAAGATGACGGTACCTGAATAAGAAGCC

CCGGCTAACTACGTG

>5936 len=135 tot-seq=1

TAGGGAATCTTCCACAATGGACGCAAGTCTGATGGAGCAACGCCGCGTGAACGAAGAAGT

ACCTCGGTATGTAAAGTTCTATCAGCAGGGAAGAAAATGACGGTACCTGACTAAGAAGCC

CCGGCTAACTACGTG

>5937 len=135 tot-seq=1

TGGGGAATATTGCACAATGGACGGAAGTTTGATGCAGCGACGCCGCGTGCGGGATGAAGG

CCTTCGGGTTGTAAACCGCTTTCAGCAGGGAAGACATCGACGGTACCTGCAGAAGAAGCC

CCGGCTAACTACGTG

>5938 len=135 tot-seq=1

TGGGGAATATTGGACAATGGGCGAAAGCCTGATCCAGCAATGCCGCGTGAGTGATGAAGG

CCTTAGAGTTGTAAAGCTCTTTTACTGGGGATGATAATGACAGTACCCAGAGAATAAGCT

CCGGCTAACTCCGTG

>5939 len=135 tot-seq=1

AGTCTGATGGAGCAACGCCGCGTGAGTGAAGAAGGGTTTCGGCTCGTAAAGCTCTGTTGT

TGGTGAAGAAGGACAGGGATAGTAACTGTACTTTATTTGACGGTAGTCAATTAGAAAGTC

ACGGCTAACTACGTG

>5940 len=135 tot-seq=1

TGGGGAATCTTGCGCAATGGGGGGAACCCTGACGCAGCGACGCCGCGTGCGGGATGGAGG

CCTTCGGGTCGTGAACCGCTTTCAGCAGGGACGAGTCTGACGGTACCTGCAGAAGAAGCC

CCGGCTAACTACGTG

>5941 len=135 tot-seq=1

TGGGGAATCTTGCACAATGGGCGAAAGCCTGATGCAGCGACGCCGCGTGAACGAAGAAGG

TCTTCGGATTGTAAAGTTCTGTCCTGGGTGAAGATAATGACGGTAACTCAGGAGGAAGCC

CCGGCTAACTACGTG

>5942 len=135 tot-seq=1

GAAACAGGGCTGGACATGGAAATCCTCAATGGGGTGGAAATACTAGTGTGCAAAGATTAT

AATTTTAAGGGTAGCCTACGCGATTACCTGGTGTTAAATGCGTTACCGGGGATGCCTCCA

GCTATGGCATTTCTC

>5943 len=135 tot-seq=1

GGGGAGAGGAAGGTCTAAGCTTGGCAAGAAAATGAGAAAGGCTGTGGCTGGCTGGAGCTG

TGGGGAGTGTGTGGAGAGCAGTCGGAGAGTTGCCAGGGCCTGCTTAGCCTTGGTAAGGTC

GTTTATCTCTCTATT

>5944 len=135 tot-seq=1

TGAGGAATATTGGTCAATGGGCGAGAGCCTGATGCAGCGACGCCGCGTGAGCGAGGAAGG

CTTTCGAGTCGTAAAGCTCTGTCCTATGAGAAGATAATGACGGTATCATAGGAGGAAGCC

CTGGCTAAATACGTG

>5945 len=135 tot-seq=1

TGGGGAATTTTGGACAATGGGGGCAACCCTGATCCAGCCATGCCGCGTGAGCGAAGAAGG

CTTTCGAGTCGTAAAGCTCTGTCCTATGAGAAGATAATGACGGTATCATAGGAGGAAGCC

CTGGCTAAATACGTG

>5946 len=135 tot-seq=1

GCGCGTAAATTACCCAATCCTGAAACAGGGAGGTAGTGACAATAAATAACAATGCCTGGC

CTATCTAGGCTAGGCAATTGGAATGAGAACAATTTAAATCCCTTATCGAGGATCAATTGG

AGGGCAAGTCTGGTG

>5947 len=135 tot-seq=1

TAGGGAATCTTCCACAATGGACGCAAGTCTGATGGAGCAACGCCGCGTGAGTGAAGAAGG

TTTTCGGATCGTAAAGCTCTGTTGTTGGTGAAGAAGGATAGAGGAATCAACCAGAAAGTC

ACGGCTAACTACGTG

>5948 len=135 tot-seq=1

CCCAGACAGCTCCTCTGAGCAAGAGCTAAGGTAGAATGGGCTGAATAGGAAAAAACAAAG

CTAGTCCTGGGAATGGGCAACTTGCCATCTCACCATTCCCAAACCCCTAACCCTCCACGG

CCCAGCCACCAGGAG

>5949 len=135 tot-seq=1

TGGGGAATCTTGCCCAATGGGCGAAAGCCTGAGGCAGCAACGCCGCGTGTGGGATGAAGG

CCCTTGGGTTGTAAACCACTTTCAGCAGGGACGAAGATGACGGTACCTGCAGAAGAAGCC

CCGGCCAACTACGTG

>5950 len=135 tot-seq=1

TAGGGAATATTGCACAATGGGCGCAAGCCTGATGCAGCGACGCCGCGTGAGTGAAGAAGT

ATCTCGGTATGTAAACTTCTATCAGCAGGGAAGATAGTGACGGTACCTGACTAAGAAGCC

CCGGCTAACTACGTG

>5951 len=135 tot-seq=1

TGGGGAATATTGTACAATGGGCGAAAGCCTGATGCAGCGACGCCGCGTGAGCGAAGAAGT

ATTTCGGTATGTAAACTTCTATCAGCAGGGAAGAAAATGACGGTACCTGACTAAGAAGCC

CCGGCTAACTACGTG

>5952 len=135 tot-seq=1

TGAGGAATATTCCACAATGGGCGAAAGCCTGATGGAGCAATGCCGCGTGCAGGATGAAGG

CCCTCGGGTCGTAAACTGCTTTTATTAGAGAAGAATATGACGGTATCATAGGAGGAAGCC

CTGGCTAAATACGTG

>5953 len=135 tot-seq=1

TGGGGGATATTGCACAATGGGGGAAACCCTGATGCAGCGACGCCGCGTGGAGGAAGAAGG

TTTTCGGATTGTAAACTCCTGTCGTTAGGGACGATAATGACGGTACTCAACAAGGAAGTG

ACGGCTAACTACGTG

>5954 len=135 tot-seq=1

TGGGGAATATTGGACAATGGGCGCAAGCCTGATCCAGCTATGCCGCGTGAGTGAGGAAGG

CCTTAGGGTTGTAAAACTCTTTCAGTAGGGAAGATAATGACGGTACCTACAGAAGAAGTA

CCGGCAAACTCCGTG

>5955 len=135 tot-seq=1

CGGGGAATATTGCACAATGGGGGGAACCCTGATGCAGCAACGCCGCGTGAGTGAAGAAGT

ATTTCGGTATGTAAAGCTCTATCAGAAGGGAAGAAAATGACGGTACCTTACTAAGAAGCT

CCGGCTAAATACGTG

>5956 len=135 tot-seq=1

TAGGGAATATTCCACAATGAGCGAAAGCTTGATGCAGCGACGCCGCGTGAGCGAAGAAGT

ATTTCGGTATGTAAAGCTCTATCAGAAGGGAAGAAAATGACGGTACCTTACTAAGAAGCT

CCGGCTAAATACGTG

>5957 len=135 tot-seq=1

AATTCCTTGATGACGATTCAGCAGACATCACCTTTGATATGAGCGGGAGCCCTCGACTCC

CAAGGCATAGCTCTCTCATAGATGAAATGTTCAAGGGCTCGGCTGGTCACAGCCCCCTGG

CCACCCCATTGTCTC

>5958 len=135 tot-seq=1

TCGAGAATCATTCACAATGGGGGAAACCCTGATGGTGCGACGCCGCGTGGAGGAAGAAGG

TTTTCGGATTGTAAACTCCTGTCGTTAGGGACGATAATGACGGTACCTAACAAGAAAGCA

CCGGCTAACTACGTG

>5959 len=135 tot-seq=1

TGGGGAATATTGCACAATGGGCGAAAGCCTGATGCAGCAACGCCGCGTGAGCGATGAAGG

TTTTCGGATCGTAAAGCTCTGTTCTGAGGGAAGATAGTGACGGTACCTCAGGAGAAAGCC

CCGGCTAACTACGTG

>5960 len=135 tot-seq=1

TGGGGAATATTGGACAATGGGCGAAAGCCTGATCCAGCGACGCCGCGTGAGTGAAGAAGT

ATCTCGGTATGTAAACTTCTATCAGCAGGGAAGATAATGACGGTACCTGACCAAGAAGCC

CCGGCTAACTACGTG

>5961 len=135 tot-seq=1

TGAGGAATATTGGTCAATGGACGAGAGTCTGATCCAGCGACGCCGCGTGAGTGAAGAAGT

ATTTCGGTATGTAAAGCTCTATCAGCAGGGAAGAAAATGACGGTACCTGACTAAGAAGCC

CCGGCTAACTACGTG

>5962 len=135 tot-seq=1

TGGGGAATATTGGGCAATGGGCGAAAGCCTGACCCAGCAACGCCGCGTGCGGGATGAAGG

CCTTCGGGTTGTAAACCGCTTTCAGCAGGGAAGACATCGACGGTACCTGCAGAAGAAGCC

CCGGCTAACTACGTG

>5963 len=135 tot-seq=1

TGAGGAATATTGGACAATGGGCGCAAGCCTGATCCAGCAATACCGCGTGAGTGAAGAAGG

CCTTAGGGTTGTAAAGCTCTTTCAGCAGGAAAAATAATGATGGTACCTGCAGAAGAAGTT

CCGGCTAACTCCGTG

>5964 len=135 tot-seq=1

TGGGGAATATTGCACAATGGGCGCAAGCCTGATGCAGCAACGCCGCGTGAACGATGAAGG

TCTTCGGATCGTAAAGTTCTGTTGCAGGGGAAGATAATGACGGTACCTACAGAAGAAGCG

ACGGCTAAATACGTG

>5965 len=135 tot-seq=1

TGGGGAATATTGGACAATGGGGGAAACCCTGATCCAGCAATGCCGCGTGAATGAAGAAGG

CCTTAGGGTTGTAAAATTCTTTTGTTCGTGAAGATAATGACGGTAGCGGACGAATAAGCC

CTGGCTAACTCTGTG

>5966 len=135 tot-seq=1

TGGGAAATATTGGACAATGGGCGCAAGCCTGATCCAGCCATGCCGCGTGAGTGATGAAGG

TCTTAGGGTTGTAAAGCTTTTTCACCGATGAAGATAATGACGGTAGTCGGAGAAGAAGCC

CCGGCTAACTTCGTG

>5967 len=135 tot-seq=1

TGGGGAATATTGCACAATGGGCGAAAGCCTGATGCAGCAACGCCGCGTGAGTGAAGAAGT

ATCTCGGTATGTAAAGCTCTATCAGCAGGGAAGAAAATGACGGTACCTTTCGAATAAGCG

CCGGCTAACTACGTG

>5968 len=135 tot-seq=1

CTTCACTCCCATAGCTTGGCGGGAGGGAGTGGCAGCCAGTGGCATCTTCTCTCCCATTGT

TCAGCAAGCAAGAGGGAGGGTTACATCTTTTTACTCCAGCTGCCTGCAGCTCGGTGAGC

TGGAGGGAGTGGCAC

>5969 len=135 tot-seq=1

GCTACCAATGCCATCGGCTTTCGGCAGCGCGATCACTTCCACGTCTTCAGTCCAGAAAGA

GGTCGGCAGCGGTTCGACGTTGTGCCAGTAATCAACCGGCAGATAGAACCACAGTTTCGG

CTTGTTCACGCCATC

>5970 len=135 tot-seq=1

TGGGGAATATTGGACAATGGGCGAAAGCCTGATCCAGCAATGCCGCGTGAATGACGAAGG

CCTTAGGGTTGTAAAGTTCTTTTAGCAGGAAAGATAATGACGGTACCTGCAAAAAAAGCC

CCGGCTAACTCCGTG

>5971 len=135 tot-seq=1

GGCAGAACAGAGGGTACCAGTTTCCCAGGCCATGGTGTAAGGCCAAATTGTATCAGCCTC

AGTCTGACGCTTTTGAAGACCTTCGCTCCACCTTTCCCCAAGGCTAAAAATTGTCTGCCT

TTTGCAGGTGGTCAG

>5972 len=135 tot-seq=1

TGGGGAATATTGCACAATGGGGGAAACCCTGATGCAGCGACGCCGCGTGAGTGAGGAAGT

ATTTCGGTATGTAAAGCTCTATCAGCAGGGAAGAAAATGACGGTAATCAACCAGAAAGTC

ACGGCTAACTACGTG

>5973 len=135 tot-seq=1

TGGGGGATATTGCACAATGGGGGAAACCCTGATGCAGCAACGCCGCGTGAGGGAAGAAGT

TTTTCGGAATGTAAACCTCTATTCTTAGGGAAGATAATGACGGTAGCTAAGGAGAAAGCC

CCGGCTAACTACGTG

>5974 len=135 tot-seq=1

TGGGGAATATTGCACAATGGGGGAAACCCTGATGCAGCAACGCCGCGTGAGTGAAGAAGT

ATTTCGGTATGTAAAGCTCTGTCTTCAGGGACGATAATGACGGTACCTGAGGAGGAAGCC

ACGGCTAACTACGTG

>5975 len=135 tot-seq=1

TGGGGAATATTGGACAATGGAGGCAACTCTGATCCAGCAATTCTGTGTGTGTGAAGAAGG

TTTTAGGACTGTAAAACACTTTTAGTAGGGAAGATAATGACGGTACCTGACTAAGAAGCC

CCGGCTAACTACGTG

>5976 len=135 tot-seq=1

TGAGGAATATTGGTCAATGGGCGAAAGCCTGATGCAGCGACGCCGCGTGCGGGATGAAGG

CTTTCGAGTCGTAAAGCTCTGTCCTATGGGAAGATAATGACGGTACCATAGAAGAAAGCC

CCGGCTAATTACGTG

>5977 len=135 tot-seq=1

TGAGGAATATTGGTCAATGAGCGAAAGCCGGATGCAGCAACGCCGCGTGAGTGATGAAGG

CCTTCGGGTCGTAAAACTCTGTCCTCAAGGAAGATAATGACGGTACTTGAGGAGGAAGCC

CCGGCTAACTACGTG

>5978 len=135 tot-seq=1

TGGGGAATATTGCACAATGGGGGAAACCCTGATGCAGCGACGCCGCGTGAGCGATGAAGG

CTTTCGAGTCGTAAAGTTCTGTTGCAGGGGAAGATAATGACGGTACCCTGTGAGGAAGCC

CCGGCTAACTACGTG

>5979 len=135 tot-seq=1

TGGGGAATATTGCACAATGGGGGAAACCCTGATGCAGCGACGCCGCGTGAGCGAAGAAGG

CCTTCGGGTCGTAAAGCTCTTTTATATGGGAAGATAATGACGGTACCATACGAATAAGCA

TCGGCTAACTCCGTG

>5980 len=135 tot-seq=1

TGGGGAATATTGCACAATGGGCGCAAGCCTGATGCAGCAACGCCGCGTGAGCGATGAAGG

TTTTCGAATCGTAAAGCTCTGTCCTATGGGAAGATAATGACGGTACCATAGGAGGAAGCC

CCGGCTAACTACGTG

>5981 len=135 tot-seq=1

TGGGGAATATTGCACAATGGAGGAAACTCTGATGCAGCGACGCCGCGTGAACGATGAAGG

CTTTCGAGTCGTAAAGTTCTGTCCTTGGGGAAGATAATGACGGTACCCAAGGAGGAAGCC

CCGGCTAACTACGTG

>5982 len=135 tot-seq=1

TGGGGAATATTGCACAATGGGCGAAAGCCTGATGCAGCGACGCCGCGTGAGCGAAGAAGT

ATTTCGGTATGTAAAGCTCTGTCCTATGAGAAGATAATGACGGTATCATAGGAGGAAGCC

CTGGCTAAATACGTG

>5983 len=135 tot-seq=1

TGGGGAATCTTCCGCAATGGACGAAAGTCTGACGCAGCAACGCCGCGTGCGGGATGAAGG

CCTTCGGGTTGTAAACCGCTTTCAGCAGGGAAGACATCGACGGTACCTGCAGAAGAAGCC

CCGGCTAACTACGTG

>5984 len=135 tot-seq=1

TGGGGAATATTGCACAATGGAGGAAACTCTGATGCAGCGATGCCGCGTGAGGGAAGAAGG

TTTTCGGATTGTAAACCTCTGTGTACAGGGACGATAATGACGGTACCTGTTCAGAAAGCT

CCGGCTAACTACGTG

>5985 len=135 tot-seq=1

TGGGGAATATTGCACAATGGGCGAAAGCCTGACGCAGCGACGCCGCGTGAGCGAAGAAGT

ATTTCGGCATGTAAAGCTCTATCAGGAGGGAAGAAGATGACGGTACCTTACCAAGAAGCT

CCGGCTAAATACGTG

>5986 len=135 tot-seq=1

TGGGGAATATTGCACAATGGGCGAAAGCCTGATGCAGCGACGCCGCGTGAGCGAAGAAGT

ATTTCGGTATGTAAAGCTCTATCAGAAGGGAGGACATCGACGGTACCTGCAGAAGAAGCC

CCGGCTAACTACGTG

>5987 len=135 tot-seq=1

TGGGGAATATTGCACAATGGGGGAAAACCTGATGCAGCGACGCCGCGTGAGCGATGAAGT

ATTTCGGTATGTAAAGCTCTATCAGCAAGGAAGATAATGACGGTACTTGACTAAGAAGCC

CCGGCTAACTACGTG

>5988 len=135 tot-seq=1

TGGGGAATATTGCACAATGGGCGCAAGCCTGATGCAGCAACGCCGCGTGAACGATGAAGG

TCTTCGGATCGTAAAGTTCTGTTGCAGGGGAAGATAATGAGTGTACCTTTCGAATAAGCG

CCGGCTAACTACGTG

>5989 len=135 tot-seq=1

TGGGGAATATTGCGCAATGGGGGAAACCCTGACGCAGCGACGCCGCGTGCGGGATGACGG

TTTTCGGATTGTAAACCTCTGTCCTCTGTGAAGATAGTGACGGTAGCAGAGGAGGAAGCT

CCGGCTAACTACGTG

>5990 len=135 tot-seq=1

GCGCTAGCCTGAACCAGCCAAGTAGCGTGAAGGAGGAAGGCTCTATGGGTCGCAAACTTC

TTTTATAGAAGAATAAAGTGCAGTATGTATACTGGGTTGTATGTATTATATGAATAAGGA

TCGGCTAACTCCGTG

>5991 len=135 tot-seq=1

TGGGGAATATTGCACAATGGGGGAAACCCTGATGCAGCGACGCCGCGTGAAGGAAGAAGT

ATCTCGGTATGTAAACTTCTATAAGCAGGGAAGATAATGGCGGTACCTGACGAAGAAGCC

CCGGCTAACTACGTG

>5992 len=135 tot-seq=1

TGGGGAATATTGCACAATGGAGGGAACTCGGATGCAGCGATGCCGCGTGGAGGAAGAAGG

TTTTCGGATGGTAAACTCCTTTTATCAGGGACGATAATGACGGTACCTGAAGAAAAAGCT

CCGGCTAACTACGTG

>5993 len=135 tot-seq=1

TAGGGAATATTGCACAATGGAGGAAACTCTGATGCAGCGATGCCGCGTGAGGGAAGAAGG

TTTTCGGATTGTAAACCTCTGTCTTTGGGGACGGTAATGACGGTACCCAAGGAGGAAGCT

CCGGCTAACTACGTG

>5994 len=135 tot-seq=1

TGGGGAATATTGCGCAATGGGGGAAACCCTGACGCAGCGACGCCGCGTGAGTGAAGAAGG

CCTTCGGGTTGTAAAGCTCTGTCTTCAGGGACGATAATGACGGTACCTGAGGAGGAAGCC

ACGGCTAACTACGTG

>5995 len=135 tot-seq=1

TGGGGAATATTGCACAATGGGGGAAACCCTGATGCAGCGACGCCGCGTGCGTGAAGAAGT

AATTCGTTACGTAAAGCTCTACCAGCAGGGAAGAGAGTGACGGTACCTGAGTAAGAAGCC

CCGGCTAACTACGTG

>5996 len=135 tot-seq=1

CGACGAATATTCCGCAATGCGCGAAAGCGTGACGGAGCGATGCCGCGTGCAGGATGAAGC

GGCTCTGCCGTGTAAACTGCTGTCAGGGAACAGAACATACGATCATTCCCAAAGGAAGGG

CCTGCTAATCTCGTG

>5997 len=135 tot-seq=1

TAGGGAATCTTGCGCAATGGGCGAAAGCCTGACGCAACCATGCCGCGTGAATGATGAAGG

TCTTAGGATTGTAAAATTCTTTCAGCGGGGACGATAATGACGGTACCTGCAGAAGAAGCC

CCGGCTAACTTCGTG

>5998 len=135 tot-seq=1

TGGGGAATATTGCACAATGGGGGAAACCCTGATGCAGCAACGCCGCGTGAGTGATGAAGT

ACCTCGGTATGTAAAACTCTATCAGCAGGGAAGAAAATGACGGTACCTGACTAAGAAGCC

CCGGCTAACTACGTG

>5999 len=135 tot-seq=1

TGGGGAATGTTGGGCAATGGGCGAAAGCCTGACCCAGCAACGCCGCGTGAGGGAGGAAGG

GTTTCGGCTCGTAAACCTCTGTCCTATGGGACGAAGAAGTGACGGTACCATAGGAGGAAG

CCCCGGCTAACTACG

>6000 len=135 tot-seq=1

TGGGGAATATTGGACAATGGGCGCAAGCCGGATCCAGCCATGCCGCGTGAGTGACGAAGG

TCTTCGGATTGTAAAGCTCTTTTGGCGGGGACGATAATGACGGTACCCGCAGAATAAGCC

CCGGCTAACTTCGTG

>6001 len=135 tot-seq=1

GCGCGCAAATTACCCAATCCCGACACGGGGAGGTAGTGACAATAAATAACAATACCGGGC

TTTTTCAAGTCTGGTAATTGGAATGAGTACAATCTAAATCCCTTAACGAGGATCAATTGG

AGGGCAAGTCTGGTG

>6002 len=135 tot-seq=1

CTGAGAATCTTCCACAATGGACGAAAGTCTGATGGAGCGACATCGCGTGAGGGATGAAGG

CCTTCGGGTTGTAAACCTCGGAAATTATCGAAGAATGAGTGACAGTAGATAATGTAAGCC

TCGGCTAACTACGTG

>6003 len=135 tot-seq=1

TGGGGGATATTGCACAATGGGGGAAACCCTGATGCAGCGACGCCGCGTGAGGGAAGAAGG

TTTTCGGATTGTAAACCTCTGTCAGTGGGGACGATAATGACGGTACCCACGGAGGAAGCC

ACGGCTAACTACGTG

>6004 len=135 tot-seq=1

TGGGGGATATTGCACAATGGGGGAAACCCTGATGCAGCAACGCCGCGTGAAGGAAGAAGG

TCTTCGGATTGTAAACTTTTGTCCTTAGTGAAGATAATGACGGTAGCTAAGGGGGAAGCT

CCGGCTAACTACGTG

>6005 len=135 tot-seq=1

CCCTGACCGAGCAACGCCGCGTGAGTGAAGAAGGTTGTCGGATCGTAAAGCTCTGTTGTA

AGAGAAGAACGTGTGTGAGAGTGGAAAGTTCACTCAGTGACGGTAACGTGCCAGAAAGGG

ACGGCTAGCTACGTG

>6006 len=135 tot-seq=1

TGGGGAATATTGCACAATGGAGGAAACTCTGATGCAGCGACGCCGCGTGAGTGAAGAAGT

ATTTCGGTATGTAAAGCTCTATCAGCAGGGAAGAAAATGACGGTAACTTACCAGAAAGGG

ACGGCTAACTACGTG

>6007 len=135 tot-seq=1

TGGGGAATATTGCACAATGGGGGAAACCCTGATGCAGCGACGCCGCGTGAGCGAAGAAGG

CCTTCGGGTCGTAAAGCTCTTTTATATGGGAAGATAATGACGGTATCATAGGAGGAAGCC

CTGGCTAAATACGTG

>6008 len=135 tot-seq=1

TGGGGAATATTGCACAATGGACGGAAGTTTGATGCAGCGACGCCGCGTGGGCGAAGAAGG

CTTTCGAGTCGTAAAGCTCTGTCCTATGAGAAGATAATGACGGTATCATAGGAGGAAGCC

CTGGCTAAATACGTG

>6009 len=136 tot-seq=1

TGGGGAATATTGCACAATGGGGGAAACCCTGATGCAGCGACGCCGCGTGAGTGAAGAAGT

ATTTCGGTACGTAAAGCTCTGTTAGCAGGGAAGAGGAAGGACGGTACCTGCAGAGGAAGC

CACGGCCAACTACGTG

>6010 len=136 tot-seq=1

AATCTAATAAGAAACAGACAGTACACAATGAAGTCATCAAGATCTGGTGTCTGGTGCAAG

TAAGACTCGCCCCACAGGAGCTGGGGTCGTGGAAACAGAAGTGAAGAAATTGAGACATGG

AAACACAGGATAACAC

>6011 len=136 tot-seq=1

AAGCCTGAACGAGCGATGCCGCGGGAGGGAAGAAGGCCTGCGGGTCGTAAAGCGCTGTTG

CGGGGGAAAAAGGCAGCATCAGGAAATGGGTGCTGACGGATGGTGCCCCGCCAGAAAGT

CACGGCTAACTACGTG

>6012 len=136 tot-seq=1

TGGGGAATATTGCACAATGGGGGAAACCCTGATGCAGCAACGCCGCGTGAGTGAAGAAGT

ATTTCGGTATGTAAAGCTCCATCAGCAGGGAAGAAAAAGACGGTACCTGACGAATAAGC

CACGGCTAACTACGTG

>6013 len=136 tot-seq=1

TATGTATATGAACAAAGTAAATTGCAATAGCAGAAATAATTGACAAAATGTTGCTTGGTG

ATTTGCTGATATTCATAGACCATGAAAGCTTCCAATTTAATTATATATATGTTAGTAAAT

TAAGAAATATATCAAA

>6014 len=136 tot-seq=1

TGGGGAATTTTGGACAATGGGCGCAAGCCTGATCCAGCGACGCCGCGTGTGGGAAGACGG

TCCTCTGGATTGTAAACCACTGTCCCCAGGGACGAAAATGACGGTACCTGGGGAGGAAGC

TCCGGCTAACTACGTG

>6015 len=136 tot-seq=1

GCGCGCAAATTACCCATTCCCGGCACGGGGAGGTAGTGACGAAAAATAACGATACGGGAC

TCATCCGAGGCCCCGTAATCGGAATGAGCACACTTTAAATCCTTTAACGAGTATCCATTG

GAGGGCAAGTCTGGTG

>6016 len=136 tot-seq=1

TGGGGAATATTGCACAATGGGGGAAACCCTGATGCAGCGACGCCGCGTGAAGGATGAAGG

TTTTCGGATTGTAAACTTCTTTTATTAAGGACGAAACTTGACGGTACTTAATGAATAAGC

TCCGGCTAACTACGTG

>6017 len=136 tot-seq=1

TGGGGAACATTGCACAATGGGGGAAACCCTGATGCAGCGACGCCGCGTGAGTGAAGAAGT

ATTTCGGTATGTAAAGCTCTATCAGCAGGGAAGAAGAAGGACGGTACCTGAGTAAGAAGC

CCCCGCTAACTACGTG

>6018 len=136 tot-seq=1

CCTTAAGATAGTGACCTCTGCAGTTTGGTACTTTAAGGCAGTCAGTTTGTCAGCTTGCTA

GCTTTAGAGAATCCAGCCTAAATCCTTTTACCTGCAGACAGTCTGCCTAGCAGCTTGGTA

GCATAAGAGAGACAGA

>6019 len=136 tot-seq=1

TAGGGAATATTGGACAATGGGCGAAAGCCTGATCCAGCAATACCGCGTGAGTGATGAAGG
CTCTTTTGGTTGTAAAGCTCTTTCGTTGAGGAAGATTATGACGGTACTCAAAGAAGAAGT
CCCGGCTAACTCCGTG
>6020 len=136 tot-seq=1
AAGTCTGATGGAGCAACGCCGCGTGAGGGAAGAAGGGTTGCGGCTCGTAAAGCTCTGTTG
TTGGTGAAGAAGGACAGGGGGAGTAACTGACCTTGGTTTGACGGTAATCAAGTAGAAAGT
CACGGCTAACTACGTA
>6021 len=136 tot-seq=1
TGAGGAATAGGGGTCAATGGGAGAGAGCCTGAACCAGACAAGTAGCGAGCAGGATGACGG
CCCTATGGGATGTAAACTGCTTTTATATGGGAATAAAGTGAGGGACGTGTCCCTTATTGC
ATGTACCATACGAATA
>6022 len=136 tot-seq=1
TCGAGAATTTTGGGCAATGGGCGAAAGCCTGACCCAGCAATGCCGCGTGCGGGATGAAGG
TCTTCGGATTGTAAACCGCTGTCGAGGGGGACGAAATTTGACGGTACCCCTAGAGGAAGC
CACGGCTAACTACGTG
>6023 len=136 tot-seq=1
TGGGGAATATTGGGCAATGGGCGAAAGCCTGACCCAGCGACGCCGCGTGAGGGAAGAAGG
TCTTCGGATTGTAAACCTTAAACAACAGGGACGAAGAAGTGACGGTACCTGTTAGGAAGC
CACGGCTAACTACGTG
>6024 len=136 tot-seq=1
GCGCGCAAATTACCCACTCCCGGCACGGGGAGGTAGTGACGAAAAATAACGATACGGAAC
TCACATGAGGCTCCGTAATCGGAATGAGTACACTTTAAATCCTTTAACGAGGATCTATTG
GAGGGCAAGTCTGGTG
>6025 len=136 tot-seq=1
TGGGGAATATTGCTCAATGGGGGAAACCCTGAAGCAGCAACGCCGCGTGAAGGATGAAGG
CCTTCGGGTTGTAAACTTCTTTAATCAGGGACGAATTAATGACGGTACCTGAAGAATAAG
CCACGGCTAACTACGT
>6026 len=136 tot-seq=1
TGGGGAATATTGGGCAATGGGGGCAACCCTGACCCAGCAACGCCGCGTGAAGGAAGAAGG

CTTTCGGGTTGTAAACTTCTTTGACGAGGGACGAAACATGACGGTACCTCGAAAACAAGC

CACGGCTAACTACGTG

>6027 len=136 tot-seq=1

CACAGAGAGACACAGACAAACAGGAAGACAGTATCCCCTCGTGGTTAACATCACAGCTTC

TGTTTATTAAGCACCTAATGTGTTACAACTGCACAGATCCCATTGAACCATGGGCCCTCT

CCCTCACCTCCCCAAG

>6028 len=136 tot-seq=1

TGGGGAATATTGCGCAATGGGGGAAACCCTGACGCAGCAACGCCGCGTGGGGGATGAAGG

CCCTCGGGTTGTAAACCCCTTTCAGTAGGGACGAAATCAGACGGTACCTACAGAAGAAGC

CCCGGCCAACTACGTG

>6029 len=136 tot-seq=1

TGGGGAATCTTGCGCAATGGGCGAAAGCCTGACGCAGCAACGCCGCGTGCGGGATGAAGG

CCTTCGGGTTGTAAACCGCTTTCAGTGGGGACGAAACAAGACGGTACCCACAGAAGAAGC

CCCGGCCAACTACGTG

>6030 len=136 tot-seq=1

GGGAGCAGAGGTCTCCGCCCTGGTTCCACACAGGGCGCCTCTGCAGAATTGCACAGAAAT

CTCCAGAAATTCTGGCCTCCTGCTTATCTGGATGGAGTCTAGGATGGGATGTCCTGACAG

TCCTCAGTGATTCAAA

>6031 len=136 tot-seq=1

TAGGGAATCTTCCGCAATGGACGCAAGTCTGACGCAGCGACGCCGCGTGCGGGACGGAGG

CCTTCGGGTCGTAAACCGCTTTCAGCAGGGAAGAGAGAAGACTGTACCTGCAGAAGAAGC

CCCGGCTAACTACGTG

>6032 len=136 tot-seq=1

TAGGGAATCTTCCACAATGGACGCAAGTCTGATGGAGCAACGCCGCGTGGAGGATGACAC

TTTTCGGAGCGTAAACTCCTTTTATCAGGAAAGAATAATGACGGTACCTGATGAATAAGC

ACCGGCTAACTCCGTG

>6033 len=136 tot-seq=1

TGAGGAATCTTGCACAATGGGCGAAAGCCTGATGCAGCGACGCCGCGTGAGGGATGAAGG

CCTTCGGGTTGTAAACCTCTGTCAGTAGGGAAGAAGATTGACGGTACCTACTGAGGAAGC

TTCGGCTAACTACGTG

>6034 len=136 tot-seq=1

TGGGGAATATTGCACAATGGGGGAAACCCTGATGCAGCGACGCCGCGTGGAGGAAGAAGG

TCTTCGGATTAGAAACCCCAGTAGTCCATGTAGATCGGAAGAGCGTCGTGTAGGGAAAGA

GTGAGCCAATGTGTAG

>6035 len=136 tot-seq=1

AAGTCTGATGGAGCCACGCCGCGTGAGTGAAGAAGGGTTTCGGCTCGTAAAACTCTGTTG

TTGGTGAAGAAGTACAGGGGTAGTAACTGGCCTTTATTTGACGGTAATCAATTAGAAAGT

CACGGCTAACTACGTG

>6036 len=136 tot-seq=1

TGGGGAATCTTGGACAATGGGCGCAAGCCTGATCCAGCCATGCCGCGTGGATGACTGAAG

GCCCTAGGGTTGTAAAGTCCTTTCGACGGGGACGATAATGACGGTACCCGTAGAAGAAGC

CCCGGCTAACTTCGTG

>6037 len=136 tot-seq=1

TGGGGAATATTGGGCAATGGGCGCAAGCCTGACCCAGCAACGCCGCGTGAGGGAAGAAGG

TCTTCGGATTGTAAACCTTTTTGACAGGGGACGAAGCATGACGGTACCCTGAAAACAAGC

CACGGCTAACTACGTG

>6038 len=136 tot-seq=1

AAGCCTGACGGAGCAACGCCGCGTGAGTGAAGAAGGATTTAGGTTAGTAAAAATATGTTG

TTAGGGAAGAAAAAATGTATAGTAAATATATAAAAAAGAGAAGGTACCTAACCAGAAAGC

CACGGCTAACTACGTG

>6039 len=136 tot-seq=1

TGGGGAATATTGCACAATGGGCGAAATCTGATAGAGCTTTACATACCGAAATACTTCTTC

GCTCACGCGGCGTCGCTGCATCAGGCTTTGGAAGAAAATGACGGTACCTTACTAAGAAGC

TCCGGCTAAATACGTG

>6040 len=136 tot-seq=1

AAGTCTGATGGAGCAACGCCGCGTGAGGGAAGAAGGGTTTCGGCTCGTAAAGCTCTGTTG

TTGGTGAAGAAGGACAAGGGTAGTAACTGGCCTTGGTTTGACGGTAATCAAATAGAAAGT

CACGGCTAACTACGTG

>6041 len=136 tot-seq=1

TGGGGAATATTGCACAATGGGGGGAACTCTGACGCAGTGACGCCGCGTATAGGAAGAAGG

TCTTCGGATTGTAAACTATTGTCGTTAGGGAAGAAAAAGGACAGTACCTAAGGAGGAAGC

TCCGGCTAACTACGTG

>6042 len=136 tot-seq=1

TGAGGAATATTGCGCAATGGAGGAGACTCTGACGCAGTGACGCCGCGTATAGGAAGAAGG

TCTTCGGATTGTAAACTATTGTCGTTAGGGAAGAAAAAGGACAGTACCTAAGGAGGAAGC

TCCGGCTAACTACGTG

>6043 len=136 tot-seq=1

TGGGGGATATTGCACAATGGGGGGAACCCTGATGCAGCGATGCCGCGTGAAGGAAGAAGA

TTTTCGGATCGTAAACTTCTATCAGCAAGGAAGAAGAAGGACAGTACTTGACTAAGCAGC

CCCGGCTAACTACGTG

>6044 len=136 tot-seq=1

TGGGGAATATTGGGCAATGGGCGAAAGCCTGACCTAGCAACGCCGCGTGAAGGATGAAGG

TTTTCGGATTGTAAACTTCTGTCGCAGGGGACGAAGAATGACGGTACCCTGTAAGAAAGC

CCCGGCTAACTACGTG

>6045 len=136 tot-seq=1

TGGGGAATATTGCACAATGGGGGAAGCCCTGATGCAGCGACGCCGCGTGAGCGATGAAGT

ATTTCGGTATGTAAAGCTCTGTCAGCAGGGAAGAAGAATGACGGTACCTGACCAAGAAGC

CCCGGCTAACTACGTG

>6046 len=136 tot-seq=1

TGGGGAATATTGCACAATGAGGGGAACCCTGATGCAGCGACGCCGCGTGAGTGAAGAAGT

ATTTCGGTATGTAAAGCTCTATCAGCAGGGAAGAATTTAGACGGTACCTGACTAAGAAGC

CCCGGCTAACTACGTG

>6047 len=136 tot-seq=1

TGGGGAATATTGCACAATGGGGGAAACCCTGATGCAGCAACGCCGCGTGAGTGAAGAAGT

CATTCGTGATGTAAAGCTCTATCAGCAGGGGAAGAAAATGACGGTACCTTACTAAGAAGC

TCCGGCTAAATACGTG

>6048 len=136 tot-seq=1

TAGGGAATATTGCGCAATGGGGGAAACCCTGACGCAGCAACGCCGCGTGAGTGAAGACGT
ATCTCGGTATGTAAAGCTTTATCAGCGGGGAAGAAGGAAGACGGTACCCGACTAAGAAGC
CCCGGCTAACTACGTG
>6049 len=136 tot-seq=1
TGGGGGATATTGCACAATGGGGGAAACCCTGATGCAGCAACGCCGCGTGAGGGAAGAAGG
TCTTCGGATTGTAAACCTTTGTTTTGGTGACGAATAATGACGGTAGCCAAAGAGGAAGC
TCCGGCTAACTACGTG
>6050 len=136 tot-seq=1
TGAGGAATATTGGTCAATGGGCGGGAGCCTGAACCAGCCAAGTAGCGTGCAGGAAGACGG
CCCTATGGGTTGTAAACTGCTTTTATGCGGGGATAAAATGACGGTACCTTACTAAGAAGC
TCCGGCTAAATACGTG
>6051 len=136 tot-seq=1
TGGGGGATATTGCGCAATGGGGGCAACCCTGACGCAGCAACGCCGCGTGAAGGATGAAGG
TTTTCGGATTGTAAACTTCTTTTCTTGAGGACGAAAATTGACGGTACTCAAGGAATAAGC
TCCGGCTAACTACGTG
>6052 len=136 tot-seq=1
TGGGGAATTTTCCGCAATGGGGGCAACCCTGACGGAGCAATACCGCGTGAGGGAGGACGG
CTTTTGGGTTGTAAACCTCTTTTATCAGGGAAGAATTTTGACGGTACCTGATGAATCAGC
ATCGGCTAACTCCGTG
>6053 len=136 tot-seq=1
TGGGGGATATTGCACAATGGGGGGAACCCTGATGCAGCAACGCCGCGTGAGTGAAGAAGT
ATTTCGGTATGTAAAGCTCTATCAGCAGGGAAGAAAAAAGACGGTACCTGACTAAGAAGT
CCCGGCTAACTACGTG
>6054 len=136 tot-seq=1
AAGTCTGATGGAGCAACGCCGCGTGAGTGAAGAAGTGTTTCGGCTCGTAAAGCTCTGTTG
TTGGTTAAGAAGGACATGGGTAGTAACTGACCTTTATTTGACGGTAATCACTTAGAAAGT
CACGGCTAACTACGTG
>6055 len=136 tot-seq=1
CAGCAGGGGATCATGGGAAATGCCAAGTAAGCCAGCAACATGGTATCATGGGAAATGCCA

AGTAAGCCAGCAACATGGGATCATGGGAAATGCTAAGTAAACCAGCAACATGGGATCATA

GGGAATGCCAAGTAAA

>6056 len=136 tot-seq=1

ATGGACGCAAGTCTGATGGAGCAACGCCGCGTGAGTGAAGAAGGGTTTCGGCTCGTAAAG

CTCTGTTGTTGGTGAAGAAGGACAGGGGTACCTTTGTTTGACGGTAATCAATTAGAAAGT

CACGGCTAACTACGTG

>6057 len=136 tot-seq=1

AAGGCGGAGGGAGCAACGCCGCGGGAGGGAAGAAGGGGGGCGGAGCGGAAAGCGCGGGGG

GGGGGGAAGAAGGAGAGAGGGAGTAACTGGCCTTTATTTGACGGTAATCAACCAGAAAGT

CACGGCTAACTACGTG

>6058 len=136 tot-seq=1

TGGGGAATATTGCACAATGGGCGAAAGCCTGATGCAGCAACGCCGCGTGAGCGATGAAGG

CCTTCGGGTTGTAAAGCTCTGTCTTCGCTGACGAAACCTGACGGTAAGCGAGGAGGAAGC

CACGGCTAACTACGTG

>6059 len=136 tot-seq=1

CCACTGGGAAGCCCACCTGTGCTTTCCCCCATCTGGGTGGGGCGGCAGCAGAGACCCTGA

GAATCAGCGGTTATGGGAGCTGTGTGTTAGCTGTGTGTTATTGGCTTTGGCTTCAGCATG

TCCTGCCTAGGAGTCT

>6060 len=136 tot-seq=1

TGGGGAATATTGTACAATGGGGGAAACCCTGATGCAGCAACGCCGCGTGAAGGATGAAGG

TTTTCGGATTGTAGACTTCTTTTATTAAGGACGAAAAATGACGGTACTTAATGAATAAGC

TCTGGCTAACTACGTG

>6061 len=136 tot-seq=1

TAGGGAATATTGCACAATGGGCGAAAGCCTGATGCAGCAACGCCGCGTGTGCGATGAAGG

CCTTCGGGTCGTAAAGCACTTTTCTGGGAGATGAGAAAGGACAGTATCCCAGGAATAAGT

CTCGGCTAACTACGTG

>6062 len=136 tot-seq=1

TGGGGAATATTGGACAATGGAGGGAACTCTGATCCAGCAATTCTGTGTGTGTGAAGAAGG

TTTTAGGACTGTAAAACACTTTTAGTAGGGGAAGATAATGACGGTACCCTGTGAGGAAGC

CCCGGCTAACTACGTG

>6063 len=136 tot-seq=1

AAGCCGGAGGAAGCCAGGCCGCGGGGAGGAAGAAGGCCGGCGGGGGGGAAAGGACGGGCG

GCGGGGAGGAAGGGAGGAAAGGGAAGACCTTTACTCATTGACGTTACCCGCAGAAGAAGC

ACCGGCTAACTCCGTG

>6064 len=136 tot-seq=1

CGGTGACTAGGCCGGAATTCTCCTGGTTGATAAATGAGAGGAGCTGAGAAGCGCTGAGAT

GCCTGTTTGGAGCATCTGATCTGTAGAAGACGAATCTTCACTGTGCCATGGAGATGTTCT

GGGAGGGAGAGCCGGC

>6065 len=136 tot-seq=1

AAGAATGATGAAGAAATGAAGAGTGTATGAAGAAGGAATTAGGGTTGTAAAGTAATTTCA

GAGGGGAGGAAGGGAGTAAAGTTAATACCTTTGCTCATTGACGTTACCCGCAGAAGAAGC

ACCGGCTAACTCCGTG

>6066 len=136 tot-seq=1

TGGGGAATATTGCGCAATGGGGGCAACCCTGACGCAGCAACGCCGCGTGAAGGATGAAGG

TCTTCGGATTGTAAACTTCTGTTCTGAGGGGCGAACAAGGACGGTACCTCAGGAGGAAGC

CACGGCTAACTACGTG

>6067 len=136 tot-seq=1

TGGGGGATATTGCGCAATGGGGGAAACCCTGACGCAGCAACGCCGCGTGGAGGATGACGG

TTTTCGGATTGTAAACTCCTTTTATGGGGGACGAATCAAGACGGTACCCCATGAATAAGC

TCCGGCTAACTACGTG

>6068 len=136 tot-seq=1

GGAAGCTGGGACCAGCTGGGTGACAAACAGCCCATACCTTAGGCTCCTCCCTGGAGCCCA

GGGTGGCGTCCTAGGATGCTCCAGGCCTTGGACTCTGGGATCCTTCCTTCTGATGCCCAC

ATCACAGAGAGGTGGC

>6069 len=136 tot-seq=1

TGGGGAATATTGCACAATGGACGGAAGTTTGATGCAGCGACGCCGCGTGAGGGAAGAAGG

TTTTAGGATTGTAAACCTCTGTCTTCAGGGACGAAAAAAGACGGTACCTGAGGAGGAAGC

TCCGGCTAACTACGTG

>6070 len=136 tot-seq=1

TGGGGAATATTGCACAATGGGGGAAACCCTGATGCAGCAACGCCGCGTGAGTGAAGAAGC

GTTTCGGCGCGTAAAGCTCTGTCAGCGGGGAAGAAAGAAGACGGTACCCGACCAAGAAGC

CCCGGCTAACTACGTG

>6071 len=136 tot-seq=1

GAGGCCCAGGCACCCCCGCCCCAGGAGTGGCTCTGTCCATCCGGCGTCTTTCACACATTC

TTTCTTTCTCTGTCTCTCCTCCTCTCCTCCCACATCCTTCCTGTAGAGCTACTTGGTAAG

TCAAATGTCGGTCCCT

>6072 len=136 tot-seq=1

GGGGAATATTGGGCAATGGGGGGAACCCTGACCCAGCAACGCGGCGTGGAGGAAGAAGGG

TTTCGGATCGTAAACTCCTGTCCTTGGAGACGAGTAGAAGACGGTATCCAAGGAGGAAGC

CCCGGCTAACTACGTG

>6073 len=136 tot-seq=1

TGGGGAATATTGCGCAATGGGGGAAACCCTGATGCAGCGACGCCGCGCGAAGGAAGAAGT

ATTTCGGTATGTAAACTTCTATCAGCAGGGAAGAAAAAAGACGGTACCTGACTAAGAAGT

CCCGGCTAACTACGTG

>6074 len=136 tot-seq=1

AAGCCTGATGGAGCAACGCCGCGTGAGTGAAGAAGGTTTTCGGATCGTAAAACTCTGTTG

TTGAAGAAGAACGAGCGTGAGAGTAACTATTCACTCAGTGACGGTATTCAACCAGAAAGT

CACGGCTAACTACGTG

>6075 len=136 tot-seq=1

TTGAGGTCCAGGAGCTGCCGCCCACCCGGCTCCTCCTCTGGGTCCCCCAGCAGCACGGGG

ACCACACTGGAGAAGAGCTGTTTGCACCATTGCTCCGGCGGCGGCCACCTGCCTTCCGCC

TAGGGAGACAGAGCCG

>6076 len=136 tot-seq=1

GCGGGAGCCTGAACCAGCCAAGTCGCGTGAGGGAAGACGGTCCTACGGATTGTAAACCTC

CTTTGCCGGGGAGCAACGGGGTCCTTGCGAGGGCCCAATGAGAGTACCCGGAGAAAAAGC

ATCGGCTAACTCCGTG

>6077 len=136 tot-seq=1

TGGGGAATATTGCACAATGGGGGAAACCCTGATGCAGCGACGCCGCGTGAAGGAAGAAGT

ATCTCGGTATGTTAACTTCTATCAGCAGGGAGGAAAACAGACGGTACCTGACTAAGAAGC

CCCGGCTAACTACGTG

>6078 len=136 tot-seq=1

AAGTCTGATGGAGCAACGCCGCGTGAGGGAAGAAGGGTTTCGGCTCGTAAAGCTCTGTTG

GTAGTGAAGAAAGATAGAGGGAGGAACGGGCCTTTATTTGACGGTAATTACTTAGAAAGT

CACGGCTAACTACGTG

>6079 len=136 tot-seq=1

TAGGGAATCTTCGGCAATGGGGGCAACTCTGACGCAGTGACGCCGCGTGCAGGAAGAAGG

TTTTCGGATTGTAAACTGCTTTAGACAGGGAAGAAAAAAGACAGTACCTGTAGAATAAGC

TCCGGCTAACTACGTG

>6080 len=136 tot-seq=1

TGGGGAATATTGCACAATGGGGGGAACCCTGATGCAGCGACGCCGCGTGGGTGAAGAAGT

ATTTCGGTATGTAAAGCCCTGTCAGCAGGGAAGAAGAATGACGGTACCTGACCAAGAAGC

ACCGGCTAAATACGTG

>6081 len=136 tot-seq=1

CAGCTCTCACCTCTGCAAAACAGAGAACCCTGGCCAAGGGGGAGCAGGGTTGAAGATTAG

GGTAGAGGGTGGAATGGAGAAAGGATTTCTTTTGTGGCACAAAGAAGAAGGTAAATTGTT

TCTTCATCTCATTGTC

>6082 len=137 tot-seq=1

GGGGCCGTGTGTGTGCCTACAATGACCCAGGTCTACACCCCTTCTAGCACGGAGTCAGCA

CACATGCAACCATTCTGCTGGCTGGTACCCCACGGAAGGAACACAGGTGGCTTAGAGGTA

GATGCAGAGACCAGAGA

>6083 len=137 tot-seq=1

TGGGGAATATTGCGCAATGGGCGAAAGCCTGACGCAGCGACGCCGCGTGCGGGATGACGG

CCTTCGGGTTGTAAACCGCTGTCGGGAGGGACGAAGCTCTGACGGTACCTCCAAAGGAAG

CACCGGCTAACTCTGTG

>6084 len=137 tot-seq=1

GAGCTGGTGTCTTGAGTGAGGGGCCCTCAGAGCCAGTAGTTAACTGGGCAGTGGAGGGAG

GCATGTGGTTAGGCCTCAGGCCAGGTCTCCAGGAGACCTGAGAGATAGTTTGAGAGAGGA

GGACCATTCTGTGGAGC

>6085 len=137 tot-seq=1

TGGGGAATATTGCACAATGGGGGGAAACCCTGATGCAGCGACGCCGCGTGAGCGAAGAAG

TATTTCAGTATGTAAAGCTCTACCAGCAGGGAAGAAGAATGACGGTACCTGACTAAGAAG

CACCAGCTAAATACGTG

>6086 len=137 tot-seq=1

TAGGGAATTTTCGTCAATGGGGGAAACCCTGATGCAGCGACGCCGCGTGAGTGAGGAAGT

ATTTCGGTATGTAAAGCTCTATCAGCAGGGAAGAAGAAATGACGGTACCTGACTAAGAAG

CCCCGGCTAACTACGTG

>6087 len=137 tot-seq=1

CAAACTATTCTGTGATCTTGTTTCTCCTCTAAACTATGCTAAAACTACTGCTTACCCCAC

AGCCTCACCAGACTCCAGCAGAAACAGGCACCCAGGAAGCACTCTGGGTAAACACTTCCA

TTTACGGTGGCTGCACC

>6088 len=137 tot-seq=1

GGAAGAGGGAGAGGCACCAGCAGGAACTCAAAACTGCAGCAGCAGCAGCAGCAGCAGCAA

GAGGAGCAGAAGCACCAGAAGCAGCAACAGCAGAAGCAGCAGCAACAGCAGCAGAAGCAG

GGACAGAATCACCAGCA

>6089 len=137 tot-seq=1

TAGGGAATCTTCCACAATGGGCGAAAGCCTGATGGAGCAACGCCGCGTGGAGGTGGAAGG

CCTACGGGTCGTCAACTTCTTTTCTCGGAGAAGAAACAATGACGGTATCTGAGGAATAAG

CATCGGCTAACTCTGTG

>6090 len=137 tot-seq=1

TAGACTTGATTAAGCCTATAAGCGAATTACATGAAGATGTTGCAAATCTTGAATTATGCG

ATTTAATGACATGGCAACGCAAAGCGTATACAAAGTGGAAAAACAGCTTCGACGAGTGGC

TAAAATCCAAAGGAAAA

>6091 len=137 tot-seq=1

CAAGTCTGATGGAGCAACGCCGCGTGAGTGAAGAAGGGTTTCGGCTCGTAAAGCTCTGTT

GGTAGTGAAGAAAGATAGAGGTAGTAACTGTTCACGTTTCGACGGTATCTAACCAGAAAG

CCACGGCTAACTACGTG

>6092 len=137 tot-seq=1

CAACCCTGATCCAGCCATGCCGCGTGCAGGATGAAGGTCTTCGGATTGTAAACTGCTTTT

GTCAGGGACGAAAAGGGACGTGATAACACCACGTTCTGCTGACGGTACCTGAAGAATAAG

CACCGGCTAACTACGTG

>6093 len=137 tot-seq=1

CAAGTCTGATGGAGCAACGCCGCGTGAGTGGAGAAGGGTTTCGGCTCGTAAAGCTCTGTT

GTTGGTGAAGAAGGACGGGGGTAGTAACTGACCTTTGTTTGACGGTAATCAACCAGAAAG

TCACGGCCAACTACGTG

>6094 len=137 tot-seq=1

CGGGGAATATTGCGCAATGGGCGAAAGCCTGACGGAGCAACGCCGCGTGCGGGATGACGC

GTTTCGGCGTGTAAACCGCTGTTCGTGGGGACGAAGCACTGACGGTACCCACGGAGGAAG

GCCCGGCTAACTACGTG

>6095 len=137 tot-seq=1

TGGGGAATATTGGGCAATGGGCGCAAGCCTGACCCAGCAACGCCGCGTGAGGGAAGAAGG

TTTTCGGATCGTAAACCTCTGTTGTAGGGGACGAGGAAAAGACGGTACCCTACGAGGAAG

CTCCGGCTAACTACGTG

>6096 len=137 tot-seq=1

TGAGGAATATTGGTCAATGGGCGAGAGCCTGAACCAGCCAAGTAGCGTGCAGGATGACGG

CCCTATGGGTTGTAAACTGCTTTTATATGGGAATAATAATGACGGTACCAAAGGAATAAG

CACCGGCTAACTCCGTG

>6097 len=137 tot-seq=1

TGGGGAATCTTGCACAATGGGGGAAACCCTGACGCAGCGACGCCGCGTGAGGGAAGACGG

TCCTCTGGATTGTAAACCTCTGTCTTCGGGGACGAAAGAAGACGGTACCCGAGGAGGAAG

CCACGGCTAACTACGTG

>6098 len=137 tot-seq=1

TAGGGAATCTTCCACAATGGACGAAACTCTGATGCAGCCATGCCGCGTGTGTGAAGAAGG

CCTTCGGGTTGTAAAGCACTTTCGGAGGGGAGGAAAGAATGACGTTACCCTCAGAAGAAG

CACCGGCTAACTCCGTG

>6099 len=137 tot-seq=1

TGGGGAATATTGCGCAATGGGGGAAACCCTGACGCAGCGACGAAAAATAACAATACAGGA

CTCTTTCGAGGCCCTGTAATTGGAATGAGTCCACTTTAAATCCTTTAACGAGGATCCATT

GGAGGGCAAGTCTGGTG

>6100 len=137 tot-seq=1

TGGGGAATATTGCACAATGGGCGCAAGCCTGATGCGGCCATGCCGCGTGTGTGAAGAAGG

CCTTCGGGTTGTAAAGCACCTTCGGAGGGGAGGAAAAAATGACGTTACCCTCAGAAGAAG

CACCGGCTAACTCCGTG

>6101 len=137 tot-seq=1

TAGGGAATATTCCACAATGGGCGCAAGCCTGATGGAGCAACACCGCGTGAGTGAAGAAGG

GCTTCGGATTGTAAAGCTCTGTTGTTAGAGAAGAACGTTGGTAGGAGTGGAAAATCTACC

AAGTGACGGTAACTAAC

>6102 len=137 tot-seq=1

TAGGGAATATTCCACAATGAGCGAAAGCCTGACCCAGCAACGCCGCGTGAAGGAAGAAGG

CTTTCGGGTTGTAAACTTCTTTTAAGAGGGAAGAGCAGAAGACGGTACCTCTTGAATAAG

CCACGGCTAACTACGTG

>6103 len=137 tot-seq=1

TGGGGAATATTGCACAATGGGCGCAAGCCTGATGCAGCAACGCCGCGTGAGGGATGACGG

CCTTCGGGTTGTAAACCTCTTTTAGTAGGGAAGAAGCCTTCGGGTGACGGTACCTGCAGA

AAAAGCACCGGCTAACT

>6104 len=137 tot-seq=1

TGGGGAATATTGGGCAATGGAGGAAACTCTTACCCAGCAATGCCGCGTGAGTGATGAAGG

CCCTATGGGTTGTAAAGCTCTTTGATTAGGAACGAAACAATGACGGTACCTAAAGAACAA

GCCCCGGCTAACTATGT

>6105 len=137 tot-seq=1

AAAGGGAGGTCAGAGAAATTGGAAGCTTGTAAAGGATTCAGCCTGCCATTGCTGGTTTTG

ACAATGGAGGAAGGGGGACCTCTAGAACCTGAGAGCAACCCCCAACCAATAGCCATCAAG

GAAACAGGACCTAAGTC

>6106 len=137 tot-seq=1

CAGTATTCGCCTTAGACGCAGCAGAAGAATCCGCTTCAGCATTGTTAACCTCAACTCTCA

AAGCAGTCTTATCAACCGAAGCACTCTGAGAATCAGTAGTCTCAAAACCAGCAGCAGAAC

CAGAACCAACACCAGAA

>6107 len=137 tot-seq=1

TGGGGGATATTGCGCAATGGGGGCAACTCTGACCCAGCAACGCCGCGTGGAGGAAGAAGG

TTTTCGGATTGTAAACTCCTGTCCTTGGAGACGAGTAGAAGACGGTATCCAAGGAGGAAG

CCCCGGCTAACTACGTG

>6108 len=137 tot-seq=1

CAAGCCTGATGGAGCAACGCCGCGTGAGTGAAGAAGGGTTTCGGCTCGAAAAGCTCTGTT

GTTGATGAAGAAGGACAGGGGTAGTAACTGGCCTTTGTTTGACGGTGATCAATTAGAAAG

TCACGGCTAACTACGTG

>6109 len=137 tot-seq=1

GCGCGCAAATTACCCAATTCCGGCACGGAGAGGTAGTGACGAAAAATAACGATACGGGGA

CTCTTCTGAGCCTCCGTAATCGGAATGAGTTCGCTGTAAACACGCGGGCGAGGATCCATT

GGAGGGCAAGTCTGGTG

>6110 len=137 tot-seq=1

TGGGGAATATTGGACAATGGAGGAAACTCTGATCCAGCAATTCTATGTGTGCGAAGAAGG

TTTTAGGACTGTAAAACACTCTTAGTAGGGAAGAAAAAATGACGGTACCTACAGAAGAAG

CAACGGCTAAATACGTG

>6111 len=137 tot-seq=1

TGGGGGATATTGCACAATGGGGGAAACCCTGATGCAGCAATGCCGCGTGAAGGAAGAAGG

TCTTCGGATTGTAAACTTCTGTTTTTCGTGACGAAGAAGTGACGGTAGCGAAAAAGAAAG

CTCCGGCTAACTACGTG

>6112 len=137 tot-seq=1

TGAGGAATTTTCCGCAATGGGCGAAAGCCTGACGGAGCAATGCCGCGTGAAGGATGAAGG

CCTGTGGGTTGTAAACTTCTTTTCTCAGAGAAGAATTTCTGACGGTATCTGAGGAATAAG

CATCGGCTAACTCTGTG

>6113 len=137 tot-seq=1

TAGGGAATCTTCCACAATGGACGCAAGTCTGACGGAGCAATGCCGCGTGGAGGTAGAAGG

CCCACGGGTCGTGAACTTCTTTTCCCGGAGAAGAAGCAATGACGGTATCTGGGGAATAAG

CATCGGCTAACTCTGTG

>6114 len=137 tot-seq=1

TGGGGAATATTGCGCAATGGGGGAAACCCTGACGCAGCAACGCCGCGTGAAGGAAGAAGG

TTTTCGGATTGTAAACTTCTGTTCTTAGGGAAGAAAAAACGACGGTACCTAAGGAGCAAG

CCACGGCTAACTACGTG

>6115 len=137 tot-seq=1

TGGGGAATATTGGGCAATGGGCGCAAGCCTGACCCAGCAACGCCGCGTGAAGGAAGAAGG

CTTTCGGGTTGTAAACTTCTTTTAATGGGGAAGAGCAGAAGACTGTACCCATAGAATAAG

CCACGGCTAACTACGTG

>6116 len=137 tot-seq=1

TGGGGAATATTGGGCAATGGGCGCGAGTCTGACCCAGCAACGCCGCGTGAAGGAAGAAGG

CTTTCGGGTTGTAAACTTCTTTTAAGGGGGAACAGCAGAAGAGGGTACCCCTTGAATAAG

CCACGGCTAACTACGTG

>6117 len=137 tot-seq=1

TGGGGGATATTGCACAATGGGGGAAACCCTGATGCAGCGACGCCGCGTGAGTGAAGAAGC

AATTCGTTGCGTAAAGCTCTATCAGCAGGGAAGAAGAAATGACGGTACCTGAATAAGAAG

CCCCGGCTAACTACGTG

>6118 len=137 tot-seq=1

TGGGGAATATTGGGCAATGGACGCAAGTCTGACCCAGCAACGCCGCGTGAAGGAAGAAGG

CTTTCGGGTTGTAAACTTCTTTTAACAGGGAAGAGAAGAAGACGGTACCTGTTGAATAAG

CCACGGCTAACTACGTG

>6119 len=137 tot-seq=1

TGGGGGATATTGGACAATGGGGGGAACCCTGATCCAGCGACGCCGCGTGAGTGAAGAAGT

ATTTCGGTATGTAAGGCTCTGTCAGCAGGGAAGAAATCATGACGGTACCTGACCAAGAAG

CCCCGGCTAACTACGTG

>6120 len=137 tot-seq=1

CAAGCCTTATGAAGCAATGCCGCGTGAGTGAAGAAGGGTTTCGGCTCGTAAAGCTCTGTT

GGTAGTGAAGAAAGATAGAGGTAGTAACTGGCCTTTATTTGACGGTAATTACTTAGAAAG

TCACGGCTAACTACGTG

>6121 len=137 tot-seq=1

TGGGGAATATTGGACAATGGGGGGAACCCTGATCCAGCGACGCCGCGTGAAGGAAGAAGG

CTTTCGGGTTGTAAACTTCTTTTGACAGGGAAGAGCAGAAGACGGTACCTGTCGAATAAG

CCACGGCTAACTACGTG

>6122 len=137 tot-seq=1

TGGGGAATATTGGGCAATGGGCGCAAGCCTGACCCAGCAACGCCGCGTGAAGGAAGAAGG

CTTTCGGGTTGTAAACTTCTTTTAAGGGGGACGAACAAATGACGGTACCCCTTGAATAAG

CCACGGCTAACTACGTG

>6123 len=137 tot-seq=1

TGGGGAATATTGCGCAATGGACGAAAGTCTGACGCAGCGACGCCGCGTGAGTGAAGAAGT

ATCTCGGTATGTAAAGCTCTACCAGCAGGGAAGAAGAAATGACGGTACCTGAGTAAGAAG

CCCCGGCTAACTACGTG

>6124 len=137 tot-seq=1

TGGGGAATATTGGGCAATGGGCGAAAGCCTGACCCAGCGACGCCGCGTGAAGGAAGAAGG

TCTTCGGATTGTAAACTTTTGTCAACAGGGAAGAAGAAGTGACGGTACCTGTTAAGAAAG

CCACGGCTAACTACGTG

>6125 len=137 tot-seq=1

TGGGGGATATTGGACAATGGGGGAAACCCTGATCCAGCGACGCCGCGTGAGTGAAGAAGT

ATTTCGGTATGTAAAGCTCTGTCAGCAGGGAAGAAAGAAATGACGGTACCTGGGGAGGAA

GCTCCGGCTAACTACGT

>6126 len=137 tot-seq=1

TGGGGAATTTTCGGCAATGGGGGGAACCCTGACCGAGCAACGCCGCGTGAAGGAAGAAGG

CTTTCGGGTTGTAAACTTCTTTTAAGAGGGAAGAGCAGAAGACGGTACCTCTTGAATAAG

CCACGGCTAACTACGTG

>6127 len=137 tot-seq=1

TGGGGAATATTGGGCAATGGAGGAAACTCTGACCCAGCAACGCCGCGTGAAGGAAGAAGG

CTTTCGGGTTGTAAACTTCTTTTGACAGGGAAGAGCAGAAGACGGTACCTGTCGAATAAG

CCACGGCTAACTACGTG

>6128 len=137 tot-seq=1

TAGGGAATCTTCCACAATGGGCGAAAGCCTGATGGAGCAACACCGCGTGAAGGAAGAAGG

CTTTCGGGTTGTAAACTTCTTTTGACAGGGAAGAGCAGAAGACGGTACCTGTCGAATAAG

CCACGGCTAACTACGTG

>6129 len=137 tot-seq=1

TGGGGAATATTGCGCAATGGGGGAAACCCTGACGCAGCAACGCCGCGTGCGGGACGAAGG

CGTCCGCGTCGTAAACCGCTTTCAGCGGGGAACACCAATCGAGGGTACCCGCAGAAGAAG

CGCCGGCTAACTACGTG

>6130 len=137 tot-seq=1

TGGGGGATATTGCGCAATGGGGGCAACCCTGACGCAGCAATACCGCGTGAGTGAAGAAGG

TTTTCGGATCGTAAAGCTCTGTTATTGGGGAAGAAGCAGTGACGGTACCCAATGAGTAAG

TCCCGGCTAACTACGTG

>6131 len=137 tot-seq=1

TGGGGAATATTGCACAATGGGGGAAACCCTGACCCAGCAACGCCGCGTGAAGGAAGAAGG

CTTTCGGGTTGTAAACTTCTTTTAAGAGGGAAGAGAAGAAGACGGTACCTCTTGAATAAG

CCACGGCTAACTACGTG

>6132 len=137 tot-seq=1

TGGGGAATATTGCGCAATGGACGAAAGTCTGACGCAGCGACGCCGCGTGAGTGAAGAAGT

ATTTCGGTACGTAAAGCTCTATCAGCAGGGAAGAAAAAAGGACGGTACCTGAGTAAGAAG

CCCCGGCTAACTACGTC

>6133 len=137 tot-seq=1

TGGGGAATATTGGGCAATGGGCGGAAGCCTGACCCAGCGACGCCGCGTGAAGGAAGAAGG

CTTTCGGGTTGTAAACTTCTTTTCTAAGGGACGAATAGATGACGGTACCTTAGGAATAAG

CCACGGCTAACTACGTG

>6134 len=137 tot-seq=1

GAACCATGACCGAGCAAAGCCGCGTGAGTGATGAAGGTAGGAGGAGAGGAAAAAGAGGGG

AGGAGGGAAGAACAGAGGGGGAAGGAACGGGGCACAGAGGGACGGGACCGAAGCAGAAAG

ACACGGCGAACGACGGG

>6135 len=137 tot-seq=1

TGGCAGAGAGAAGGGTGAGCGGCTGTGTCAGTGGTGGCCCCAGGCACCTGTGCTCTCAGC

TGCTGACCCTGGGCAACTTTGTCCTTAACTAACAGCTAAGAATCTCTAGTCATGGGAAAC

TGCAGTAACAGCACAGG

>6136 len=137 tot-seq=1

TAGGGAATTTTCCGCAATGGGCGAAAGCCTGACGGAGCAACGCCGCGTGAGTGATGACGG

CCTTCGGGTTGTAAAGCTCTGTTCTTAGGGAAGAAGTTCTGACGGTACCTAAGGAGAAAG

CCCCGGCTAACTACGTG

>6137 len=137 tot-seq=1

TGGGGAATCTTCCGCAATGGACGAAAGTCTGACGGAGCAACGCCGCGTGAGTGATGACGG

TCTTCGGATTGTAAAGCTCTGTTATTGGGGAAGAAAACATGACGGTACCCAATGAGGAAG

TCCCGGCTAACTACGTG

>6138 len=137 tot-seq=1

ACTCCTCTGAGAGATAGGAACTCTATCTTCCTCATTTGCCAGACTGGGAAGTCAAGGTGG

ACATCCTGTCTTGGCCAGACAAATGTCTCCTGCAGGACCCAGCTCCAGTGTGATTGCTTG

GGTTTTTTCCTGGTTAC

>6139 len=137 tot-seq=1

TAGGGAATCTTCCACAATGGACGCTGAGTGAAGAAGGTTTTCGGATCGTAAAGCTCTGTT

GTTGGTGAAGAAGGATAGAGGTAGTAACTGGCCTTTATTTGACGGTAATCAACCAGAAAG

TCACGGCTAACTACGTG

>6140 len=137 tot-seq=1

TAGGGAATCTTCGGCAATGGACGGAAGTCTGACCCAGCAACGCCGCGTGAAGGAAGAAGG

CTTTCGGGTTGTAAACTTCTTTTGTCAGGGAAGAGTAGAAGACGGTACCTGACGAATAAG

CCACGGCTAACTACGTG

>6141 len=137 tot-seq=1

TGGGGAATATTGCACAATGGGGGAAACCCTGATGCAGCAACGCCGCGTGAGCGAAGAAGG

CCTTTGGGTCGTAAAGCTCTGTCCTTGGGGAAGAAAAAATGACGGTACCCAAGGAGGAAG

CCCCGGCTAACTACGTG

>6142 len=137 tot-seq=1

TGGGGGATATTGCACAATGGGGGAAACCCTGATGCAGCAACGCCGCGTGTGGGAAGAAGG

TTTTCGGATTGTAAAGCTCTGTTATTGGGGAAGAAAACATGACGGTACCCAATGAGGAAG

TCCCGGCTAACTACGTG

>6143 len=137 tot-seq=1

CTTCATCGCATCCAGTTGGTTAATCCCCTCTCTGTCAATCACCTGCATACAAGCTGAAAA

AACTATAAATTCACATTTCTGGAGCCACATGGTCAAAATAGCACTTTATATTCAGTGTGA

AATTCTTAAAATGAAAC

>6144 len=137 tot-seq=1

TGGGGAATATTGGACAATGGACGCAACTCTGGTCCAGCAACTCTGTGTGTGTGAAGAAGG

TTTTAGGACTGTAAAACACTTTTAGTAGGGAGGAAAAAATGACGGTACCTACAGAAGAAG

CGACGGCTAAATACGTG

>6145 len=137 tot-seq=1

GGACGAGAGTCTGAACCAGCCAAGTAGCGTGCAGGATGACGGCCCTATGGGTGGTAAACT

GCTGTTGTTTGGGAATAATCGACAGTACGTGTAATGGTTTGCATGTACCATTCGAAGAAG

GACCGGCTAATTCCGTG

>6146 len=137 tot-seq=1

TGGGGAATATTGCGCAATGGCCGAAAGGCTGACGCAGCGACGCCGCGTGTGGGATGAAGG

TCTTCGGATTGTAAACCACTGTCGGGAGGGACGAAGCTCTGACGGTACCTCCAAAGGAAG

CACCGGCTAACTCTGTG

>6147 len=137 tot-seq=1

TGGGGAATATTGCACAATGGGCGAAAGCCTGATGCAGCGACGCGGCGTGAGGGATGACGG

TTTTCGGATTGTAAACCTCTGTCTTCAGTAACGAATCAATGACGGTAACTGAGGAGGAAG

CCACGGCTAACTACGTG

>6148 len=137 tot-seq=1

CCATTTCTGGCCAGACTGGGCTAGGGGACAGGGAGGGGCCCGCACCCAGGATAGCTTGAT

GCCAAGCTCACTTTCCCAAGCAGGCCTTACCGGTGAACAGGAACAGCAGGGTCCAGCCGA

GCTGTTCCCAGTCACCA

>6149 len=137 tot-seq=1

TCGTACTACATTCCTGCCACTGAGGAGGATAGGGCCACCTTCAGTGATGGCAGCCTAGGC

TGGTGGCTAGGGAACACCTGCCCTGCTCTTGCCTCAGCCCCATTGGCAGCAGCCCTCGCC

TTCACTGCATTTTAGTC

>6150 len=137 tot-seq=1

TGGGGAATATTGCGCAATGGGGGAAACCCTGACGCAGCGACGCCGCGTGCGCGAAGAAGT

ATTTCGGTATGTAAAGCTCTATCAGCAGGGAAGAAAAAATGACGGTACCTGACTAAGAAG

CCCCGGCTAACTACGTG

>6151 len=137 tot-seq=1

TAGGGAATCTTCGGCAATGGGGGCAACCCTGACCGAGCAACGCCGCGTGAAGGAAGAAGG

CTTTCGGGTTGTAAACTTCTTTTAAGGGGGAAGAGCAGAAGACGGTACCCCTTGAATAAG

CCACGGCTAACTACGTG

>6152 len=138 tot-seq=1

TCGAGAATATTCGACAATGGACGAAAGTCTGATCGAGCGACGCCGCGTGCAGGATGAAGG

CCTTCGGGTTGTAAACTGCGGTAGCTAGGGAGGAACTTCGGTGACTGTACCTAGCGGAAA

GTGGTTGGTAACTACGTG

>6153 len=138 tot-seq=1

TTCAGTCCCTTAGCAGCTCCAGACCAGTCCTAGACTGGCAATCAGCACACATGAACTGGC

AGCTCTGTCCAGGTAGATGGTACACATGAACTGGCAGCTTCAGACTAGTCCTTTCAGCAG

GCAGACACCAGGCATTAA

>6154 len=138 tot-seq=1

TGGGGAATATTGCACAATGGGCGAAAGCCTGATGCAGCGACGCCGCGTGAGTGAAGAAGT

ATTTCGGTATGTAAAGCTCTATCAGCAGGGAAGAAAGAAGTGACGGTACCTGCATAAGAA

GCCCCGGCTAATTACGTG

>6155 len=138 tot-seq=1

TGGGGAATATTGGCCAATGGAGGAAACTCTGAACCAGCAACGTCGCGTGAATGATGAAGG

TCTTCGGATTGTAAAGTTCTCTTCTAAGGGAAGAAGATAGTGACGGTACCTTAGGAATAA

GCCTCGGCTAACTACGTG

>6156 len=138 tot-seq=1

TAGGGAATCTTCGGCAATGGGGGCAACCCTGACCGAGCAACGCCGCGTGAGTGAAGAAGG

TTTTCGGATTGTAAAACTCTTTAAGTGGGGACGAAGAAAGTGACTGTACCCACAGAATAA

GCCTCGGCTAACTACGTG

>6157 len=138 tot-seq=1

GGGCAGGGAGGAAGGGAGTGGAGAATCAGCTGATGGGAGGTAATTCCCTAGAAAAAAATG

GCTGTGGAGGTTAGGGGAATGTTTTAAAGTACTAGGGGGGAAAAGGTGAGAAACGTGGTT

CTATCTACTGGAATGTAT

>6158 len=138 tot-seq=1

TGGGGAATATTGCACAATGGGCGCAAGCCTGATGCAGCCATGCCGCGTGAAGGAAGAAGG

CCTTCGGGTTGTAAACTTCTTTTCTTAGGGACGAAGAAAGTGACGGTACCTAAGGAATAA

GCCACGGCTAACTACGTG

>6159 len=138 tot-seq=1

GGGAAACCCTGATGCAGCGACGCCGCGTGAGTGAAGAAGGTCTTCGGATCGTAAAGCTCT

GTTGTAAGTGAAGAACGGCTCATAGAGGAAATGCTATGGGAGTGACGGTAGCTTACCAGA

AAGCCACGGCTAACTACG

>6160 len=138 tot-seq=1

TATTACAATTTAGGAAGGGCAGAGGGAAGATCCCACTTACCTCACAGGACCTGCTCGGGA

CCTCTGTTTGAGAGTGGTGGCTTGGCAGCCTTTAGGTGAGGGGCTGGGAGAGAATTGCAA

AGCCACCTCTATTTGCCT

>6161 len=138 tot-seq=1

TGGGGGATATTGGACAATGGGGGAAACCCTGATCCAGCGACGCCGCGTGAGTGAAGAAGT

ATTTAGGTATGTAAAGATCTATCAGCAGGGAAGAAGGAAATGACGGTACCTGAGTAAGAA

GCCCCGGCTAACTACGTG

>6162 len=138 tot-seq=1

TGGGGAATATTGCACAATGGGCGAAAGCCTGATGCAGCGACGCCGCGTGAAGGATGAAGT

ATTTCGGTATGTAAAGCTCTATCAGCAGGGAAGAAAGAAATGACGGTACCTGAGTAAGAA

GCCCCGGCTAACTACGTG

>6163 len=138 tot-seq=1

GCAAGCCTGATGCAGCCATGCCGCGTGTATGAAGAAGGCCTTCGGGTTGTAAAGTACTGT

GTGCGTTGAGGAAGGGAGTAAATGTAATACCCTTGCTCATTGACGTTACCCGCAGAAGAA

GCACCGGCTAACTCCGTG

>6164 len=138 tot-seq=1

GCTTGAGACCTGTCACTGTCTGTGGTTTTAGCCTGGGGTCTTCCCAGCGGTGGAAGCCTT

TAGGGGCAGCTAAAGCAGGGAAGGTGAGGGACCTGAGAAGCCTTAGGTGACAGCCAGCCA

CAGAACCAGGCTCGCCAG

>6165 len=138 tot-seq=1

TGGGGAATATTGCACAATGGGGGAAACCCTGATGCAGCGACGCCGCGTGAAGGAAGAAGT

ATCTCGGTATGTAAACTTCTATCGACAGGGAAGAAAGAAATGACGGTACCTGAATAAGAA

GCCCCGGCTAACTACGTG

>6166 len=138 tot-seq=1

TGGGGAATATTGCACAATGGGCGCAACTCTGACCCAGCAACGCCGCGTGAATGATGAAGG

TCTTCGGATTGTAAAGTTCTTTTCTAAGGGAAGAAGAAAGTGACGGTACCTTAGGAATAA

GCCTCGGCTAACTACGTG

>6167 len=138 tot-seq=1

TGGGGAATATTGCACAATGGGGGAAACCCTGATGCAGCAACGCCGCGTGAAGGAAGAAGG

TTTTCGGATCGTAAACTTCTATCAATAGGGAAGAAACAAATGACAGTACCTAAAGAAAAA

GCCACGGCTAACTACGTG

>6168 len=138 tot-seq=1

TGGGGAATATTGCACAATGGGCGCAAGCCTGATGCAGCAATTCTGTGTGCACGATGAAGT

TTTTCGGAATGTAAAGTGCTTTCAGTTGGGAAGAAGAAAGTGACGGTACCAACAGAAGAA

GTGACGGCTAAATACGTG

>6169 len=138 tot-seq=1

TGGGGAATATTGCACAATGGGCGAAAGTCTGATGCAGCAACGCCGCGTGAGCGAAGAAGG

TCTTCGGATCGTAAAGCTCTGTCCTTGGGGAAGAAGAAAATGACGGTACCCTTGGAGGAA

GCCCCGGCTAACTACGTG

>6170 len=138 tot-seq=1

AAAGTCTGACGCAGCAATACCGTGTGAGGGATGAAGCATTTCGGTGTGTAAACCTCTGTC

GATGATGAATAAAGCATTCGAGAGTGAAAAGCTCAAATAAAGAAGGTAATCATGAAGGAA

GCCACAGCAAAAAAAATG

>6171 len=138 tot-seq=1

TAGGGAATATTCCACAATGAGCGAAAGCTTGATGGAGCGACACAGCGTGCACGATGAGGG

TCTTCGGATTGTAAAGTGCTGTTATAAGGGAAGAAAAAAATGACGGTACCTACAGAAGAA
GCAACGGCTAAATACGTG
>6172 len=138 tot-seq=1
TGGGGAATATTGGACAATGGAGGAAACTCTGATCCAGCAATTCTGTGTGTGTGAAGAAGG
TTTTAGGACTGTAAAACACTTTTAGTAGGGACGAAGAAAGTGACGGTACCTGTAGAACAA
GCCACGGCTAACTACGTG
>6173 len=138 tot-seq=1
TTGAACAGATAAGAACCCTTCGTGATTGTGAAATGGGAGAGTTCCCTGGCTCCCCCCTCG
CAGGATGTGCAACAAGGGTGTGGCTCTCTGTTCAGCCACTGCGAGCTCAAACCCCTTACG
AGATGGGGAGCGTGCAGA
>6174 len=138 tot-seq=1
TGGGGAATCTTCCGCAATGGACGAAAGTCTGACGGAGCAACGCCGCGTGAGTGAAGACGG
TCTTCGGATTGTAAACTGCTTTTATGCGGGGATAAAGGAGTCCACGTGTGGGCTTTTGCA
GGTACCGCATGAATAAGG
>6175 len=138 tot-seq=1
TGGGGAATCTTCCGCAATGGACGAAAGTCTGACGGAGCAACGCCGCGTGAGTGAAGAAGG
TCTTCGGATTGTAAAGCTCTGTTATACGGGACGAAAAAAATGACGGTACCTACAGAAGAA
GCAACGGCTAAATACGTG
>6176 len=138 tot-seq=1
AAGTGAGCGTGTGGCTCCGACATTGCGGGAGGAGGATCTATGTACCAGGTGGGTGGGGGA
ATGAGGGGGCCTTAGAGATCATACAGGCCCCAGAATCTAAACGGAGGCTGATGGAGATGA
TCCTCTGGGCTTCCACTC
>6177 len=138 tot-seq=1
AAAACAGAGTTGTAACACTTGAGGTCTGTCACTACCAAAGCTCTTTCCAGAGCCACTATT
TTCCCTGGCAGGCAGTGGAGCTGATCAGATGAACAAGCAGCCATAGTGTGCTGCCTCATG
TGACACCTGCCTCTCTGA
>6178 len=138 tot-seq=1
TATTGGGAGGGGAAGCTGGGGAGAAGTCAAAATTACAAAGAGAGCCATTTCTGGTGCTTT
TTCATGGCTAGGTTCAAGTTCACCTGCACTGAGGACCACAGCATCCTAATCCATGACAAT

GACCTTGTGTTCCCTATT

>6179 len=138 tot-seq=1

TGGGGAATCTTCCGCAATGGACGAAAGTCTGACGGAGCAACGCCGCGTGAGTGAAGACGG

CCTTCGGGTTGTAAAGCACTTTCGGAGGGGAGGAAAAAAATGACGTTACCCTCAGAAGAA

GCACCGGCTAACTCCGTG

>6180 len=138 tot-seq=1

TGGGGAATATTGCGCAATGGGGGAAACCCTGACGCAGCCATGCCGCGTGTGTGAAGAAGG

CCTTCGGGTTGTAAAGCACTTTCGGAGGGGAGGAAAAAAATGACGTTACCCTCAGAAGAA

GCACCGGCTAACTCCGTG

>6181 len=138 tot-seq=1

TAGGGAATCTTCGGCAATGGACGGAAGTCTGATGGAGCCATGCCGCGTGTGTGAAGAAGG

CCTTCGGGTTGTAAAGCACTTTCGGAGGGGAGGAAAAAAATGACGTTACCCTCAGAAGAA

GCACCGGCTAACTCCGTG

>6182 len=138 tot-seq=1

TGGGGAATTTTGGACAATGGGGGAAACCCTGATCCAGCCATGCCGCGTGCGGGATGAAGG

CCTTCGGGTTGTAAAGCACTTTCGGAGGGGAGGAAAAAAATGACGTTACCCTCAGAAGAA

GCACCGGCTAACTCCGTG

>6183 len=138 tot-seq=1

GGAAGGAAAAGCTAGTTTAGTTGGGTTTTCTGTTCCTTGTAACTGAAAGCATCCTCTTGG

GCAGTCACTGCTAAGAGAAGTCCAGATCTCGCTCCCATTTTAGATCTGGGAATCCCGCTG

CTTTGGCAGGAGCCCATC

>6184 len=138 tot-seq=1

TGGGGAATTTTCCGCAATGGGCGAAAGCCTGACGGAGCAATACCGCGTGAGGGAAGAAGG

CTCTTGGGTTGTAAACCTCTTTTCTTGGGGAAGAAAAAAATGACGGTACCCAAGGAATCA

GCATCGGCTAACTCCGTG

>6185 len=138 tot-seq=1

TGGGGAATATTGCACAATGGAGGAAACTCTGATGCAGCAACGCCGCGTGAACGAAGAAGG

CTCTTGGGTCGTAAAGTTCTGTTATGAGGGAAGAAGAAAGTGACGGTACCTCAGGAGAAA

GCCCCGGCTAACTACGTG

>6186 len=138 tot-seq=1

TGGGGAATCTTGCACAATGGGCGAAAGCCTGATGCAGCAACGCCGCGTGAGCGAAGAAGG

CCTTTGGGTCGTAAAGCTCTGTCGGTAGGGAAGAAGGAAGTGACGGTACCTACCGAGGAA

GCCCCGGCTAACTACGTG

>6187 len=138 tot-seq=1

AATCTTCCACAATGGACGATTGTCTGATGGAGCAACGCCGCGTGAGTGAAGAAGGTTTTC

GGCTCGTAAAGCTCTGTTGTTGGTGAAGAAGGACAGAGGTAGTAACTGGCCTTTATTTGA

CGGTAATCAATTTGCTCG

>6188 len=138 tot-seq=1

TGGACGAGAGTCTGAACCAGCCAAGTAGCGTGCAGGATGACGGCCCTATGGGTTGTAAAA

TGATTTTGTTTGGGAATAATAGAAATTAAGTGTAATGTTTTGCATGTACCATTCGAATAA

GGACCGGCTAATTACGTG

>6189 len=138 tot-seq=1

GGTGTGGCTATGTAGCATCAGCAGGAGGCATCCTGGGCAGCTCAGACCCGGAGGGCTGTC

CGGAGAGCCAGGTGAGGAGACAGGCAGGGGCCAGGCAGCTCGGGAGTCCGGGAAGCAGAA

AGTGCCTGATGAAAACCC

>6190 len=138 tot-seq=1

TGGGGAATATTGGGCAATGGGCGCAAGCCTGACCCAGCAACGCCGCGTGAGGGAAGAAGG

CCTTCGGGTTGTAAACCTCTGTCCTATGGGAAGAAGAAAGTGACGGTACCATAGGAGGAA

GCTCCGGCTAACTACGTG

>6191 len=138 tot-seq=1

GCTGATCCCAGGCTCTGGAGCTAGTCTTGGGTTCAAATCCCAGCTCCAGCAGTTACCAGC

TGTGTGCCCTTTGACACGTTTCCTGATCTCTCAAGTTTCCTATCAAGGTTTTTGCTCTGT

CAAGCTGTTTCCTCAAAC

>6192 len=138 tot-seq=1

TAGGGAATCTTCGGCAATGGACGGAAGTCTGACCGAGCAACGCCGCGTGAGTGAAGAAGG

CCTTCGGGTTGTAAAACTCTTTGGACAGGGACGAAGAAAGTGACGGTACCTGTAGAACAA

GCCACGGCTAACTACGTG

>6193 len=138 tot-seq=1

TAGGGAATCTTCGGCAATGGACGGGAGTCTGACCGAGCAACGCCGCGTGAGTGAAGAAGG

TTTTAGGACTGTAAAACACTTTTAGTAGGGAAGAAAAAAATGACGGTACCTACAGAAGAA

GCAACGGCTAAATACGTG

>6194 len=138 tot-seq=1

TGGGGAATATTGGGCAATGGAGGAAACTCTGACCCAGCAATGCCGCGTGAATGAAGAAGG

TCTTCGGATTGTAAAGTTCTTTAGTTAGGGAAGAATAAAATGACGGTACCTAAAGAATAA

GCCCCGGCTAACTACGTG

>6195 len=138 tot-seq=1

GAGGACTACAGAGTGACGGTGTCACACTGCGAGAGTCTGCCACTTGGGCAGGGAGCCTCC

CTGGAGCCACGCAGGAAGAGCAGGAGTTTCCAAGAGAACAAGACAGAACTCCGCATAGCC

TGATCCCTTGTGCCAAAC

>6196 len=138 tot-seq=1

TGGGGAATCTTCCGCAATGGACGAAAGTCTGACGGAGCAATTCTGTGTGTGTGAAGAAGG

TTTTAGGACTGTAAAACACTTTTAGTAGGGAAGAAAAAAATGACGGTACCTACAGAAGAA

GCAACGGCTAAATACGTG

>6197 len=138 tot-seq=1

TGGGGAATATTGCACAATGGGGGAAACCCTGATCCAGCAATTCTGTGTGTGTGAAGATGG

TTTTAGGACTGTAAAACACTTTTAGTAGGGAAGAAAGAAATGACGGTACCTACAGAAGAA

GCGACGGCTAAATACGTG

>6198 len=138 tot-seq=1

TAGGGAATCTTCCACAATGGACGCAAGTCTGATGGAGCAACGCCGCGTGAGTGAAGAAGG

GTTTCGGCTCGTAAAGCTCTGTTGTTGGTGAAGAAGGAAATGACGGTACCTGACCAAGAA

GCCCCGGCTAACTACGTG

>6199 len=138 tot-seq=1

TGGGGAATATTGCACAATGGGCGCAAGCCTGATGCAGCCATGCCGCGTGTATGAAGAAGG

CCTTCGGGTTGTAAAGTACTTTCAGTTGGGAAGAAGTCAGTGACGGTACCAACAGAAGAA

GCGACGGCTAAATACGTG

>6200 len=138 tot-seq=1

TGGGGAATATTGCACAATGGGCGCAAGCCTGATGCAGCAACGCCGCGTGAAGGAAGAAGG

CCCTCGGGTTGTAAACTTCTTTTAACAGGGACGAAGAAAGTGACGGTACCTGTTGAATAA

GCCACGGCTAACTACGTG

>6201 len=138 tot-seq=1

TGGGGAATATTGGACAATGGAGGGAACTCTGACCCAGCAACGCCGCGTGAGTGATGAAGG

CCTTCGGGTTGTAAAACTCTTTGGACAGGGACGAAGAAAGTGACGGTACCTGTAGAACAA

GCCACGGCTAACTACGTG

>6202 len=138 tot-seq=1

TGGGGAATATTGGGCAATGGGCGAAAGCCTGACCCAGCGACGCCGCGTGAGGGAAGAAGG

TCTTCGGATTGTAAACCTCTGTTGACAGGGAAGAAGAAAGTGACGGTACCTGTCGAGGAA

GTCACGGCTAACTACGTG

>6203 len=138 tot-seq=1

TGGGGAATATTGCACAATGGGGGAAACCCTGATGCAGCGATGCCGCGTGGAGGAAGAAGG

TTTTCGGATTGTAAACTCCTGCCTTAAAGGAAGAAGTCAGTGACGGTACTTTAGGAGGAA

GCTCCGGCTAACTACGTG

>6204 len=138 tot-seq=1

TTGGGAATATTGGACAATGGAGGAAACTCTGATCCAGTGACGCCGCGTGAAGGAAGAAGG

TCTTCGGATTGTAAACTTATTTTATCAGGGAAGAAGAAAGTGACAGTACCTGAAGAAAAA

GGACCGGCAAACTACGTG

>6205 len=138 tot-seq=1

TGAGGAATATTGGTCAATGGGCGCGAGCCTGAACCAGCGACGCCGCGTGAGTGAAGAAGT

ATTTCGGTATGTAAAGCTCTATCAGCAGGGAAGAAAGAAATGACGGTACCTGACCAAGAA

GCCCCGGCTAACTACGTG

>6206 len=138 tot-seq=1

TGGGGAATATTGGGCAATGGGCGCAAGCCTGACCCAGCAACGCCGCGTGAAGGAAGAAGG

CTTTCGGGTTGTAAACTTCTTTGACCAGGGAAGAAACAAATGACGGTACCTGGAAAACAA

GCCACGGCTAACTACGTG

>6207 len=138 tot-seq=1

TGGGGGATATTGCACAATGGGGGAAACCCTGATGCAGCGACGCCGCGTGAGTGAAGAAGT

ATTTCGGTATGTAAAGCTCTATCAGCAGGGAAGAAAGAAATGATAGTACCTGAAGAAAAA

GCATCGGCTAACTCCGTG

>6208 len=138 tot-seq=1

TGGGGAATATTGGGCAATGGGCGCAAGCCTGACCCAGCAACGCCGCGTGAAGGAAGAAGG

TTTTCGGATTGTAAACTTCTTTTATGAGGGACGAAACAAATGACGGTACCTCATGAATAA

GCCACGGCTAACTACGTG

>6209 len=138 tot-seq=1

TGGGGAATATTGCACAATGGGGGAAACCCTGATGCAGCAACGCCGCGTGAAGGACGAAGG

CTTTCGGGTTGTAAACTTCTATCGATGGGGAAGAAAAAAATGACGGTACCCAAGTAAGAA

GCCCCGGCTAACTACGTG

>6210 len=138 tot-seq=1

TCGAGAATTTTTCACAATGGGGGAGACCCTGATGGAGCGACGCCGCGTGGAGGATGAAGG

CCCTTGGGTTGTAAACTCCTGTCATAAGGGAACAAGAAAGTGATAGTACCTTAAGAGGAA

GAGACGGCTAACTCTGTG

>6211 len=138 tot-seq=1

TGGGGAATATTGGACAATGGGGGGAACCCTGATCCAGCGACGCCGCGTGAGTGAAGAAGT

AATTCGTTATGTAAAGCTCTATCAGCAGGGAAGAAGAAAGTGACGGTACCTGACTAAGAA

GCCCCGGCTAACTACGTG

>6212 len=138 tot-seq=1

TGGGGAATATTGCGCAATGGGGGAAACCCTGACGCAGCGACGCCGCGTGAAGGAAGAAGG

CTTTCGGGTTGTAAACTTCTTTTCTCGGGGACGAAGCAAGTGACGGTACCTGAGGAATAA

GCCACGGCTAACTACGTG

>6213 len=138 tot-seq=1

TGGGGAATATTGGGCAATGGGGGAAACCCTGACCCAGCAACGCCGCGTGAAGGATGAAGG

CTTTCGGGTTGTAAACTTCTTTGATCAGGGACGAAACAAATGACGGTACCTGAAGAACAA

GCCACGGCTAACTACGTG

>6214 len=138 tot-seq=1

TGGGGAATATTGGGCAATGGGCGAAAGCCTGACCCAGCAACGCCGCGTGAGTGATGAAGG

CCTTCGGGTTGTAAAACTCTTTGGACAGGGACGAAGAAAATGACGGTACCTTACTAAGAA

GCTCCGGCTAAATACGTG

>6215 len=138 tot-seq=1

TGGGGAATATTGCACAATGGGGGAAACCCTGATGCAGCGACGCCGCGTGAGTGAAGAAAT

ATTTCGGTATGTAAAGCTCTGTCAGCAGGGAAGAAAGCAATGACGGTACCTGACCAAGAA

GCCCCGGCTAACTACGTG

>6216 len=138 tot-seq=1

TGGGGAATATTGGGCAATGGAGGCAACTCTGACCCAGCAACGCCGCGTGAGCGATGAAGG

TCTTCGGATTGTAAAGCTTTTTAAGCGGGGACGAAGAAAGTGACTGTACCCGCAGAATAA

GCCTCGGCTAACTACGTG

>6217 len=138 tot-seq=1

TGGGGAATCTTGCACAATGGGCGAAAGCCTGATGCAGCGACGCCGCGTGGGCGAAGAAGG

CCTTTGGGCCGTAAAGCCCTGTCGGGAGGGAAGAAGGAAGCGACGGTACCTCCCAAGGAA

GCCCCGGCTAACTACGTG

>6218 len=138 tot-seq=1

TGGGGGATATTGCACAATGGGGGGAACCCTGATGCAGCGACGCCGCGTGAGGGAAGACGG

CCTTCGGGTTGTAAACCTCTGTCGCAGGGGACGAAGGAGATGACGGTACCCTGTGAGGAA

GCCACGGCTAACTACGTG

>6219 len=138 tot-seq=1

TGGGGGATATTGGACAATGGGGGAAACCCTGATCCAGCGACGCCGCGTGAGTGAAGAAGT

ATTTCGGTATGTAAAGCTCTGTCAGCAGGGAAGAAAGAAATGACGGTACCAAAAGAACAA

GCCCCGGCTAACTACGTG

>6220 len=138 tot-seq=1

GGAGCGAGAGCCGGAACCAGCCAAGGAGCGGGCAGGAGGACGGCCCGAGGGGGGGGAAAC

TGCTTTTATAAGGGAATAAAGTGAGAGTCGTGACTCTTTTTGCATGTACCTTATGAATAA

GGACCGGGTAATTCCGTG

>6221 len=138 tot-seq=1

TGGGGAATCTTCCGCAATGGACGAAAGTCTGACGGAGCAACGCCGCGTGAAGTATGAAGG

CCTTCGGGTTGTAAACTTCTTTGATCAGGGAAGAAACAAATGACGGTACCTGAAAAACAA

GCCACGGCTAACTACGTG

>6222 len=138 tot-seq=1

TGGGGAATATTGCACAATGGGGGAAACCCTGATGCAGCAACGCCGCGTGAGCGAAGAAGG

CCTTTGGGTCGTAAAGCTCTGTCCTCAGGGAAGAAGGAAGTGACGGTACCTGAGGAGGAA

GCCCCGGCTAACTACGTG

>6223 len=138 tot-seq=1

TGGGGAATATTGCACAATGGGCGAAAGCCTGATGCAGCGACGCCGCGTGAGCGAAGAAGG

TTTTAGGACTGTAAAACACTTTTAGTAGGGAAGAAAAAAATGACGGTACCTTACTAAGAA

GCTCCGGCTAAATACGTG

>6224 len=138 tot-seq=1

TGGGGGATATTGCACAATGGAGGCAACTCTGATGCAGCAACGCCGCGTGAGTGAAGAAGG

CTTTCGAGTCGTAAAACTCTGTCGTATGGGAAGAAAAAAATGACGGTACCTACAGAAGAA

GCAACGGCTAAATACGTG

>6225 len=138 tot-seq=1

TAGGGAATCTTCCGCAATGGGCGAAAGCCTGACGGAGCAACGCCGCGTGAAGGAAGAAGG

CTTTCGGGTTGTAAACTTCTTTTGTCAGGGACGAAGCAAGTGACGGTACCTGACGAATAA

GCCACGGCTAACTACGTG

>6226 len=138 tot-seq=1

TGGGGAATATTGGACAATGGAGGCAACTCTGATCCAGCAATTCTGTGTGTACGAAGAAGG

TCTTAGGATTGTAAAGTACTTTTAGTTGGGAAGAAGAAAGTGACGGTACCAACAGAAGAA

GCGACGGCTAAATACGTG

>6227 len=138 tot-seq=1

TGGGGGATATTGGACAATGGGGGGAACCCTGATCCAGCGACGCCGCGTGAGTGAAGAAGT

ATTTCGGTATGTAAAGCTCTATCAGCAGGGAAGAAGAAAATGACGGTACCTGACTAAGAA

GCACCGGCTAAATACGTG

>6228 len=138 tot-seq=1

CTTTGACAATGGTCCCAACACCCACATTTAACAAGAGGCAATTTGCTCAGGGTTTTATTC

CCACAATAAATCTTACCATTTTCTGCTCCTTAGCCTGCCTGTCTTCTCTTCTCTGTATTA

CACTAACAGCTCCTTTTC

>6229 len=138 tot-seq=1

TGGGGAATATTGGGCAATGGGGGGAACCCCGACCCAGCAACGCCGCGTGAGGGAAGAAGG

TTTTCGGATTGTAAACCTCTGTCCTCAAGGACGAAGGGAGTGACGGTACTTGAGGAGGAA

GCCCCGGCTAACTACGTG

>6230 len=138 tot-seq=1

TTGCACAATGGGGAAAACCCTGATGCAGCGACGCCGCGTGGGTGAAGGAGCACTTCGGTG

CGTAAAGCCCTATCGGCAGGGAAGAAAAGGGGGAAACCCCAGACGGTACCTGACTAAGAA

GCCCCGGCTAACTACGTG

>6231 len=138 tot-seq=1

TGGGGAATATTGGGCAATGGGCGCAAGCCTGACCCAGCAACGCCGCGTGAAGGATGAAGG

TTTTCGGATTGTAAACTTCTTTAGTCAGGGACGAAAGAAATGACGGTACCTGAAGAATAA

GCCACGGCTAACTACGTG

>6232 len=138 tot-seq=1

GGAAATGAATAGATTACACAGGCCCAGTCAGGACTGTGGCTCCAACCCCGTTAGAAGGAA

GAAGCAGGGCATTGTGATGAGGCCGCCACTGCCTGCTCAGCACAGTGGGCACAAAATGGC

CCAAAACATGTGTCAAGC

>6233 len=138 tot-seq=1

GCAAGTCTGATGGAGCAACGCCGCGTGAGTGAAGAAGGGTTTCGGCTCGTAAAACTCTGT

TGTTAGTGAAGAAGGACAAAGATAGTAACTGACCTTTGTTTGACGGTAATCAATTAGAAA

GTCACGGCTAACTACGTG

>6234 len=138 tot-seq=1

CGGGGAATTTTGGACAATGGGGGCAACCCTGATCCAGCAATTCTGTGTGTGTGAAGAAGG

TTTTAGGACTGTAAAACACTTTTAGTAGGGAAGAAAAAAATGACGGTACCTACAGAAGAA

GCAACGGCTAAATACGTG

>6235 len=138 tot-seq=1

TGGGGAATTTTGGACAATGGGGGCAACCCTGATCCAGCCATGCCGCGTGCAGGATGAAGG

TCTTCGGATTGTAAACTGCTTTTGTCAGGGAAGAAAAAAATGACGGTACCTACAGAAGAA

GCAACGGCTAAATACGTG

>6236 len=138 tot-seq=1

TGGGGGATATTGCACAATGGGCGCAAGCCTGATGCAGCAACGCCGCGTGAGGGAGGACGG

TCTTCGGATTGTAAACCTCTGTCCTTGGGGAAGAAACAAATGACGGTACCCTTCGAATAA

GCGCCGGCTAACTACGTG

>6237 len=138 tot-seq=1

TGGGGAATATTGCACAATGGGGGAAACCCTGATGCAGCAACGCCGCGTGTGGGAAGAAGG

TTTTCGGATTGTAAACCACTGTTCTTAGCGAAGAAACAAATGACGGTAACTAAGAAGAAA

GCTCCGGCTAACTACGTG

>6238 len=138 tot-seq=1

TGAGGAATATTGGTCAATGGGCGTAAGCCTGAACCAGCAATTCTGTGTGCACGATGAAGT

TTTTCGGAATGTAAAGTGCTTTCAGTTGGGAAGAAAAAAATGACGGTACCAACAGAAGAA

GTGACGGCTAAATACGTG

>6239 len=138 tot-seq=1

GAAAGTCTGATGGAGCAACGCCGCGTGAGTGAAGAAGGTTTTCGGATCGTAAAACTCTGT

TGTTAGAGAAGAACATGTGTGAGAGTAACTGTTCATGCAGTGACGGTATCTAACCAGAAA

GCCACGGCTAACTACGTG

>6240 len=138 tot-seq=1

AATGAAAGGGGCCAGGAGGTGACCCCAGGCTGGCCTCTGCCCTCACGGACAAAGCTGCTT

CCCCACGGCAGGTGGGAGTCGCTGCCCTTCTGCTGGCAGTGAGTCCCACCTGGAGTCTTG

CTAAGCCCTTCCTAACTA

>6241 len=138 tot-seq=1

TGGGGAATATTGCACAATGGGGGAAACCCTGATGCAGCGACGCCGCGTGAGTGAAGAAGT

ATCTCGGTATGTAAAGCTCTATCAGCAGGGAAGAAAAAAATGACGGTACCTACAGAAGAA

GCAACGGCTAAATACGTG

>6242 len=138 tot-seq=1

CAGTGGGGAATATTGCACAATGGGGGAAACCCGGATGCAGCGACGCCGCGTGAGCGAAGA

AGGTTTTCGAATCGTAAAGCTATGTACTATGGGAAGATAATGACGGTACCATAGGAGGAA

GCCCCGGCTAACTACGTG

>6243 len=138 tot-seq=1

TGGGGAATCTTGCACAATGGGCGAAAGCCTGACGCAGCGACGCCGCGTGCGGGATGAAGG

CCTTCGGGTTGTAAACCGCTTTCAGCAGGGACGAAGAAAGTGACGGTACCTGTAGAACAA

GCCACGGCTAACTACGTG

>6244 len=138 tot-seq=1

TGAGGAATATTGGTCAATGGACGAGAGTCTGAACCAGCAACGCCGCGTGAGTGATGAAGG

CCTTTGGGTTGTAAAACTCTTTGGACAGGGACGAAGAAAGTGACGGTACCTGTAGAACAA

GCCACGGCTAACTACGTG

>6245 len=138 tot-seq=1

TGGGGAATATTGGGCAATGGAGGCAACTCTGACCCAGCAACGCCGCGTGAATGATGAAGG

TCTTCGGATTGTAAAGTTCTGTGATGGGGGATGAACACAATGACGGTACCCCAATAGCAA

GCCACGGCTAACTACGTG

>6246 len=138 tot-seq=1

TGGGGAATATTGGACAATGGGCGCAAGCCTGATCCAGCAACGTCGCGTGAAGGAAGAAGG

TCTTCGGATTGTAAACTTCTTTAGACAGGGATGAAAGAAATGACAGTACCTGTAGAATAA

GCCACGGCTAACTACGTG

>6247 len=138 tot-seq=1

TGGGGAATCTTGCACAATGGGCGAAAGCCTGACCCAGCAACGCCGCGTGAAGTATGAAGG

CCTTCGGGTTGTAAACCTCTTTGATCAGGGAAGAAACAAATGACAGTACCTGAAAAACAA

GCCACGGCTAACTACGTG

>6248 len=138 tot-seq=1

CAACAACAAAAAAACAAAGCACTTAATTTTCTTCAGAATAAATGTTGCATTCGTAAAATA

GCAAGATGGTATAAAAAGTACCAACCAATAAAAAGAATTTGGAAAAAAAAAAAGAATGAG

GGTTTAAAAAAAAATCTG

>6249 len=138 tot-seq=1

GCAAGTCTGATGGAGCAACGCCGCGTGAGTGAAGAAGGGTTTCGGCTCGTAAAGCTCTGT

TGTTGGGGAAGAAGGGCAGGGGTAGTAACTGGCCTTTGTGTGACGGTAATCAATGAGAAA

GTCACGGCTAACTACGTG

>6250 len=138 tot-seq=1

CGATGGCTGGGAGTTCTTCTTTGACTTGATTGATAGGCCAGTTGAAGCAGTGGAGTATAT

TACCCTCCTGGATGCCTGAAGGCAGTCCGTAGTTCTGCGCCGACCCTACGGGAGTTGCCA

CAGCCAGCGACATCATCG

>6251 len=138 tot-seq=1

TGGGGAATATTGGGTAATGGGCGCAAGCCTGACCCAGCAACGCCGCGTGAAGGAGGAAGG

CCCTCGGGTTGTAAACTTCTTTTGTCAGGGACGAAGAAAGTGACGGTACCTGACGAATAA

GCCACGGCTAACTACGTG

>6252 len=138 tot-seq=1

TGGGGAATATTGGGCAATGGGCGCAAGCCTGACCCAGCAACGCCGCGTGAGGGAAGAAGG

CTTTCGGGTTGTAAACTTTTATTCTGAGGGACGAAGAAAGTGACGGTACCTCAGGAATAA

GCCACGGCTAACTACGTG

>6253 len=138 tot-seq=1

TGGGGAATATTGCGCAATGGGGGAAACCCTGACGCAGCAACGCCGCGTGAGGGAAGAAGG

TTTTCGGATTGTAAACCTCTGTCCTAAGGGAAGAAGGAAGTGACGGTACCTTAGGAGGAA

GCCCCGGCTAACTACGTG

>6254 len=138 tot-seq=1

GAGGCTGTTGAAAGCCAGGTGGGATGGGAGGCCACAGAGTCAAAGCTGTGTTTCCACATT

GTAGCTGGGGATGCTTAGCAGACACACAGACTGCGCCAGCCCAAACACTCGAAGAAGCTC

TTTCTGACCACATGTTGG

>6255 len=138 tot-seq=1

TGGGGAATATTGGGCAATGGGCGCAAGCCTGACCCAGCAACGCCGCGTGAAGGAAGAAGG

CCCTCGGGTTGTAAACTTCTTTTATTCGGGACGAAACAAATGACGGTAACTTACCAGAAA

GGGACGGCTAACTACGTG

>6256 len=138 tot-seq=1

TCTGCACCAAGATCATCCAAAATCAGAAGATCGTAGGCATTCAGACTGGCAAGAAAGTCA

GCACGCTCAGAACCGAACAGTCCGGTCATACGATTCAAAATCGTCGGGAAATTTGTCATC

AGAACCGGCACGTCCTGC

>6257 len=138 tot-seq=1

TGGGGAATATTGCACAATGGGCGCAAGCCTGATGCAGCAACGCCGCGTGAGTGAAGAAGG

CCTTCGGGTTGTAAAACTCTGTCCTATGGGAAGAAACAAATGACGGTACCATAGGAGGAA

GCCCCGGCTAACTACGTG

>6258 len=138 tot-seq=1

TGGGGAATTTTACGCAATGGGCGAAAGCCTGACGTAGCGACACCGCGTGAGCGAAGAAGC

CCTTTGGGGTGTAAAGCTCTGTCGGCTGGAACGAAAAAAATGACGGTACCAGCAAAGGAA

GCATCGGCTAACTACGTG

>6259 len=139 tot-seq=1

TGGGGAATCTTGGACAATGGGCGAAAGCCCGATCTAGCAATATCGCGTGAGTGAAGAAGG

GCAATGCCGCTTGTAAAGCGCTTTCGTTGAGTGCACGATCATGACAGGACTCGAGGAAGA

AGCCCCGGCTAACTCTGTG

>6260 len=139 tot-seq=1

GACGCACAGCCGGGATGATGCCTCTCAGTCTGCCTTGGACTTGGTCCCTCGTGAGAAAGG

AGAAGGAAGAAGAAGAGAGAACCAGGACCAGGGGGCTGCGGGACTCATGGAAGCAGGGAT

GGGTCTCTCTATGGCAGAC

>6261 len=139 tot-seq=1

TGGGGAATCTTGGACAATGGGCAAAATCCCGATCCAACAATATCGCGTGAGTGAAGAAGG

GCAATGCCGCTTGCAAAGCTCTTTCGTCGAGTGCACGATCATGACAGGACTCAAGGAGGA

AGCCCCGGCTAACTCCGTG

>6262 len=139 tot-seq=1

CTGAGTGAAGGTCAAACCCAGCCAGTGCTACCATAATTCATGGTGTAGGAGGCTACTCTG

GGCCAGGTTGCTTGGAAATCCAAGCCCAGGGTCCTGGAAGGGCAAACTCAAGGGCAACAG

TGGTGCAGAGTAGGAACCC

>6263 len=139 tot-seq=1

AACTTAGGAAATTAGTCTGAACAGGTGAGAGGGTGCGCCAGAGAACCAGACAGCTTCTGG

GACAGGCGGAAGCACAGAGCCGCTGAGGCAGCACCCTTGGCGGGCCGCAGACAGCCGGCC

ACTGTCCGGACCAGAGGAC

>6264 len=139 tot-seq=1

TGGGGAATATTGCACAATGGGGGGAACCCTGATGCAGCGACGCCGCGTGAGTGAAGAAGG

GCAATGCCGCTTGTAAAGCTCTTTCGTCGAGTGCGCGATCATGACAGGACTCGAGGAAGA

AGCCCCGGCTAACTCCGTG

>6265 len=139 tot-seq=1

ATGGAGGCAACTCTGACCCAGCCATGCCGCGTGGAGGATGAAGGCCCTCTGGGTTGTAAA

CTTCTTTTACAGGGGACGAAACCTTTCTATTCTTAGGAAGTTGACGGTACCCTGGGAATA

AGCACCGGCTAACTCCGTG

>6266 len=139 tot-seq=1

TAGGGAATCTTCCGCAATGGGCGAAAGCCTGATCCAGCAATATCGCGTGAGTGAAGAAGG

GCAATGCCGCTTGTAAAGCTCTTTCGTCGAGTGAGCGCAAATGACAGGACTCGAGGAAGA

AGCCCCGGTTAACTCCATG

>6267 len=139 tot-seq=1

CGCAAGCCTGATGGAGCAACACCGCGTGAGTGAAGAAGGGTTTCAGCTCGTAAAGCTCTG

TTGTTGGAGAAGAACGTGCGGGATAGTAACTGTTCACGGAGTGACGGTAACCAACCAGAA

AGTCACGGCTAACTACGTG

>6268 len=139 tot-seq=1

CGACGACGAAATGAGCAGAGAGGAAGCTGGATTTCAGGCTGTACATCTCCAGCAGGTGAC

AATGATGGCTACGGAAACCTCCAGCAGCCGCGTAATTATGAGATCGGAAGCGTCGTGTAG

GGAAAGAGTGTGCCCATGT

>6269 len=139 tot-seq=1

TGCCCACGCCAAGACAGGCAGCAAGGACAGCAGCACACAGACTGACAAGAGTGCCGAGCT

CTTCTGGCCCAGCATGGCCTCCCTTCCCAGCCGCGGCCGGCTGAGCACGACCCCTGCTCA

CAGCCCCGTCCTGAAACAC

>6270 len=139 tot-seq=1

TGGAGGTTTACCAGCCCAGACAGCTAAATAATCCTTCAATCCTTGACTTTCAGAGGAAAC

CGCGGGGGTCCATCCTGATTCGATTATTCATCCAAAACAGACTTCAGGGCTTTGGGACAG

CTCAGTTCCACAGGGTCAT

>6271 len=139 tot-seq=1

CGCAAGTCTGATGGAGCAACGCCGCGTAAGTGAAGAAGGGTTTCGGCTCGTAAAGCTCTG

TTGTTGGTGAAGAAGGACAGGCGTCCTAACTGACCTTTGTTCGACGGTAATCAATTAGAA

AGTCACGGCTAACTACATG

>6272 len=139 tot-seq=1

TAGGGAATCTTCCACAATGGACGAAAGTCTGATGGAGCAACGCCGCGTGAATGAAGAAGG

CCTTCGGGTCGTAAAATTCTGTTGTCAGAGAAGAACGTGCGTGAGAGTACCTTTCGAATA

AGCGCCGGCTAACTACGTG

>6273 len=139 tot-seq=1

CGCAAGCCTGATGGAGCAACACCGCGTGAGTGAAGAAGGGTTTCGGCTCGTAAAGCTCTG

TTGGTAGAGAAGAACGTATCAGAGAGTAACTGGTCAGGAAGTGACGGTATCCAACCAGAA

AGTCACGGCTAACTACGTG

>6274 len=139 tot-seq=1

GGTTGCAGTAAGCCAAGATCGCCCCACTGCATTCCAGCCTGGGTGACAAAGGTAGACTCC

ATCTCGAACACATGCTGTCCACACCTTCTCCCATAGGTAGCTCTTCTTCATCTACAGGGC

AAACTCCAAGCTTCTTGGC

>6275 len=139 tot-seq=1

CAATACAGTTTTGAAAACTCCCTGATCTCCCACAAAAAAAGACAAAATAACTAAGTAGCA

AAATCAAACACCCAACAATATTGAGTGACAAAGTACCTTCTCAACAATCCTCAAACTACA

GGAGGTTGAAGACAAACCA

>6276 len=139 tot-seq=1

GGAAACCCTGATCCAGCCATGCCGCGTGCAGGATGAAGGCCTTCGGGTTGTAAACTGCTT

TTGTACGGAACGAAAAGGCGCTCTCTAATACAGGGCGCACATGACGGTACCGTAAGAATA

AGCACCGGCTAACTACGTG

>6277 len=139 tot-seq=1

GGACTCTGTGGGTTCAATATCCCCCCAGTAGTAATATCAAAAACAACCTTAACCAACTCA

TCTTAAATACAGAAAAGTTATTACATTTATACAAAATAATACCCTCACAGCAGAAACAGT

GCACAAATCAGTACATGTA

>6278 len=139 tot-seq=1

TGGGGGATATTGCACAATGGGCGGAAGCCTGATGCAGCGACGCCGCGTGAGGGAAGACGG

TCCTCTGGATTGTAAACCTCTGTCGGAAGGGACGAAGAACGTGACGGTACCTTCCGAGGA

AGCTCCGGCTAACTACGTG

>6279 len=139 tot-seq=1

ACGGCGCCGGAAGGCCACAGCGCAGGCGCGACTCCGGCCTCCTTCCCGAGAGAAGGGGGG

ACCCTCCCCGCCCTCGTCCGACGAGCACCCGCGCCTGTTACCTGCAGCGCAGCCGGCGCG

GTCCAGTGAAAGCGCCTCT

>6280 len=139 tot-seq=1

TAGGGAATCTTCGGCAATGGACGGAAGTCTGACCGAGCAACGCCGCGTGAGTGAAGGCCT

TCGGGTTGTAAACCGCTTTTGATTGGGAGCAAGCCTTCGGGTGAGTGTACCTTTCGAATA

AGCGCCGGCTAACTACGTG

>6281 len=139 tot-seq=1

CCTGGGTTCTAACAAGTCTGCCAGCAAGCTCCTATGCATCTCAAGTCCGGGAACCATGCA

CCAGCAGCCGCGGTAAACTAAGATCGGAAGAGCGTCGTGTAGGGAAAGAGTGGGCCAATG

TGTAGATCTCGGTGGTCGC

>6282 len=139 tot-seq=1

CGAGGAGGAAGGTGTCCCCCGTGGAGGCAGCGGACCTGAACCTGGGGGGAGGTCCTGGCC

AACGAGGGATCACTAGGCTTTTACAGTACGATGATTTCCGTTTCTACTGGGAACACGATG

TAACAGCTGCAACAAGGCA

>6283 len=139 tot-seq=1

TGAGGAATATTGGACAATGGGTGGAAGCCTGATCCAGCCATCCCGCGTGAAGGAAGACGG

TTCTATGGATTGTAAACTTCTTTTATACAGGGATAAACCTACCCTCGTGAGGGTAGCTGA

AGGTACTGTATGAATAAGC

>6284 len=139 tot-seq=1

CGCAAGGCGGAGGGAGCAACGCCGCGGGAGGGAAGAAGGGTGGCGGCGCGTAAAGCTCTG

GGGTTGGTGAAGAAGGACAGGGGTAGTAACTGACCTTTGTTTGACGGTAATCAATTAGAA

AGTCACGGCTAACTTCGTG

>6285 len=139 tot-seq=1

TGGGGAATATTGGACAATGGACCAAGAGTCTGATCCCGCAATTCTGTGTGCACGATGAAG

TTTTCCGGAATGTAAAGTGCTTTCAGTTGGGAAGAAAGAAATGACGGTACCAACAAAAGA

AGTGACGGCTAACTACGTG

>6286 len=139 tot-seq=1

CGAAAGTCTGAGGGAGCAACGCCGCGTGAGTGAAGAAGGGTTTCGGCTCGTAAAGCTCTG

GTGGTAGTGAAGAAAGATAGAGGGAGTAACTGGCCTTTATTGGACGGTAATTACTTAGAA

AGTCACGGCTAACTACGTG

>6287 len=139 tot-seq=1

CGCAAGTCTGATGGAGCAACGCCGCGTGAGTGAAGAAGGGTTTCGGATCGTAAAGCTCGG

TTGTTGGGGAAGAAGGAGAGAGGTAGTAACTGGCCTTTATTTGACGGTAATCAACCAGAA

AGTCACGGCTAACTACGTG

>6288 len=139 tot-seq=1

CGCAAGTCTGATGGAGCAACGCCGCGTGAGTGAAAAAGTTTTTCGGATCGTAAAGCTCTA

TTGTTGGTGAAGAAGGATAGATGTAGTAACTGGCCTTTATTTGACGTTAATCAACCAGAA

AGTCACGGCTAACTACGTG

>6289 len=139 tot-seq=1

CCTTGTCAGTTTCCAGGTGGCCTTTGTCCCCAAGATGGGGCAGGTTCCAGTGGGGAGGTG

TGTGTGTGGGAGGCTGATACAGGAAGATTCCAGAAAGGAGGCCCTCAGGTTGCTGTGGTG

GGGAGTGGGGAGTGGGGAT

>6290 len=139 tot-seq=1

TGGGGAATATTGGACAATGGGCCGAGAGTCTGATCCAGCAATTATGTGTGCACGATGAAG

TTTTTCGGAATGTAGAGTGCTTTCAGTTGGGAAGAAAGAAATGACGGTACCAACAGAAGA

AGTGACGGCTAAATACGTG

>6291 len=139 tot-seq=1

CGCAAGCCTGATCCAGCCATGCCGCGTGAGTGAAGAAGGCCTTCGGGTTGTAAAGCTCTT

TCAGACGGAAAGAAATCTCCCAGGATAATACCTTGGGAGGATGACGGTACCGTAAGAAGA

AGCACCGGCTAACTACGTG

>6292 len=139 tot-seq=1

TGGGGAATATTGGACAATGGACCAAAAGTCTGATCCAGCAATTCTGTGTGTTTGATGAAG

GCCTTCGGGTTGTAAAGAACTTTAAGTAGGGAAGAAGAAAGTGACGGTACCTACAGAATA

AGCGACGGCTAAATACGTG

>6293 len=139 tot-seq=1

TGGCTGTGAGGAAGCAGAGAAAATTAATTTGGTAGAGACAGCATTAGAGAGTGCCCTCCA

AGAAGAAGGGTCTTGAGGAGGAAAAGAAATAGCCAGCAGCCGCGGTATGTAGAGATCGGA

AGAGCGTCGTGTAGGGAAG

>6294 len=139 tot-seq=1

GAAGCATAGAGTGTCAGGGCCTCGGTCCTCATTAAGGTCCAGATGAAATTGATCATCCGG

CACCATGCCAGGATTTCTCCTAGGTTCTGCTGAGGCAGAAGCAAAAGGAAAATAAAATAG

GATGTCATGTTCCCCAGCC

>6295 len=139 tot-seq=1

TGGGGAATCTTGCGCAATGGGGGAAACCCTGACGCAGCGACGCCGCGTGCGGGATGAAGG

CCTTCGGGTAAACCGCTTTCAGCAGGGACGAGGCCGCAAGGTGACGGTACCTGCAGAAGA

AGCTCCGGCTAAATACGTG

>6296 len=139 tot-seq=1

CGCAAGCCTGATGGAGCAACACCGCGTGAGTGAAGAAGGGTTTCGGCTCGTAAAGCTCTG

TTGTTAGAGAAGAACGTGAGTGATAGTAACTGTTCACGCAGTGACGGTAACCACCCAGAA

AGTCACGGCTAACTACGTG

>6297 len=139 tot-seq=1

CGCAAGTCTGATGGAGCAACGCCGCGTGAGTGAAGAAGGTTTTCGGATCGTAAAGCACTG

TTGTTGAAGAAGAAGGATAGAGGTAGTAACTGGCCTTTAATTGACGGTAATCAACCAGAA

AGTCACGGCTAACTACGTG

>6298 len=139 tot-seq=1

CCCCTGGAGGCCCGTGCAGGCCTCTGAGACCACCAGCTCAGGGAGGGCTTCCCTCCAGGC

AGGGCACACCGTGCACCTGCGGCATCCGGCAGGGCCAGCCCAAGGACAAATGGCGCCCCC

TTACCCACCGGTGACTTAC

>6299 len=139 tot-seq=1

CGTGTGGGGAATATTGCACAATGGGCGAAAGCCGGATGCAGCGACGCCGCGTGAGCGAAG

AAGTATTTCGGTAGGTAAAGCTCCATAAGAAGGGAAGAAAATGACGGTACCTTACTAAGA

AGCTCCGGCTAAATACGTG

>6300 len=139 tot-seq=1

CGCAAGTCTGATGGAGCAACGCCGCGTGAGTGAAGAAGGGTTTCGGCTCGTAAAGCTCTG

TTGTTGTTGAAGAATGACAGGTGGAGTAACGGACCTTTGTTTGACGGTAATCAATTAGAA

AGTCACGGCTAACTACGTG

>6301 len=139 tot-seq=1

CGAAAGCCTGATGGAGCAACGCCGCGTGGGTGAAGAAGGGTTTCGGCTCGTAAAGCTCTG

TTGGTAGTGAAGAAAGATAGAGGTAGTAGCTGGCCTTTATTTGACGGTAATTACTTAGAA

AGGCACGGCTAACTACGTG

>6302 len=139 tot-seq=1

GGGGAATATTGCACAATGGGCGCAAGCCTGATGCAGCGACGCCGCGTGGGGGATGACGGC

CTGCGGGTTGTAAACTCATTTCGCAACCGACGAAGCTTTTGTGACGGTAGGTTGAGAAGA

AGCACCGGCTAACTACGTG

>6303 len=139 tot-seq=1

TAGGGAATCTTGGACAATGGGCGCAAGCCTGATCCAGCAATATTGCGTGAGTGAAGAAGG

GCAATTCCGCTCGTAAAGCTCTTTCGTCGAGTGCGCGATCATGACATGACTCGAGGAAGA

AGCCCCGGCTAACTCCGTG

>6304 len=139 tot-seq=1

CGAAAGTCTGATGGAGCAACGCCGCGTGAGTGAAGAAGGGTTTCGGCTCGTAAAGCTCTG

TTGGTAGGGAAGAAAGAGAGAGGGAGTAACTGGTCTGTATTTGACGGTAATTACTGAGAA

AGTCACGGCTAACTACGTG

>6305 len=140 tot-seq=1

GGAGCAAAGTGGAGCGCCTGGGCCGCGCGTTCCGGAGCCGCGTGCGGCGACTGCGGGAGC

TCAGCGGCAGCCTGGAGCTCGTCTTCCTGGTGGACGAGTCGTCCAGCGTGGGCCAAACCA

ACTTCCTCAACGAGCTCAAG

>6306 len=140 tot-seq=1

CCAGACCATCTATACCTGCCTCCTCAGATGATCTGAAAAGATGTGGCGGGGTTTCCAAAG

ACAAAGGAACTGGCTTTCCAGAAGATGGAACCAGGAGAAACTGGCCATACTTAGAGTGTG

CTTGTCTGACATGGGAAATC

>6307 len=140 tot-seq=1

TGGGGAATATTGCACAATGGGCGCAAGCCTGATGCAGCCATGCCGCGTGTATGAAGAAGG

CCTTCGGATTGTAAACCCCTGTCATGTGGGAGCAAATTAAAAAGATAGTACCACAAGAGG

AAGAGACGGCTAACTCTGTG

>6308 len=140 tot-seq=1

TCCATCTACACAGGCATCCCTCAGACTCACCAGTGTGTCCTTGTTGCTGTAATGGACCAC

CCAGCCCTCCCGCAGGGTGGTGCTGGATTTCCGGGTCGTGTGTAGCACAGACTGTACCAC

GCGCAGCAGGGGGATGTATC

>6309 len=140 tot-seq=1

TGGGGAATATTGGACAATGGGCGAAAGCCTGATCCAGCCATGCCGCGTGTGTGAAGAAGG
TCTTAGGATTGTAAACCCCTGTCATGTGGGAGCAAATTAAAAAGATAGTACCACAAGAGG
AAGAGACGGCTAACTCTGTG
>6310 len=140 tot-seq=1
GACACAGGCAGTGTCCTCTAGATGAGGCCCTCTCCTTCCCTTGCTCATAGCTAAGGCCCA
GCTGAAGCTACCACCAATTTGACTAGAGCCAGGGCTGGCCTTTTCCCTACCATCCTGTGT
CCTTGAGGGCAAGTCTGGTG
>6311 len=140 tot-seq=1
AGAGACCTGTCCATGTTCACTACACATTCCACAATGCGCTACGGCCCCAGTCCAGCAACC
CAACAGCTTGCTTCTCCTAGCTGTTTGCTTATCTGGTTTCACAACAGAGAAGGGTGGGTC
TGTGGCCTCCAGAACAGGGC
>6312 len=140 tot-seq=1
CAAACACACACACACACACACAGTGGATCAGTGTAGAGTGCTCATTATTGTCTTCTTTTGGC
CTGGAAAGCCAGGGAGTGGCTGAGGTGTGTGACTCAATCGCATCACCTGTTATTCTAACT
CTTCTTTCCTTCTGTTCCAA
>6313 len=140 tot-seq=1
CAAAAAAGGGACTGCAGGCGGAGAGCAATAGTGTTTCCCTCTGTCAGCAGAACAGGGGAG
CAGGGTGGTGGCAGCGGCTGCTCAGGAGAGCGGTCACCACGGCTGCCTTTATTACCCACC
ATCAAACAGCAATTTCTTCC
>6314 len=140 tot-seq=1
TGGGGAATATTGCACAATGGGCGCAAGCCTGATGCAGCCATGCCGCGTGGATGAAGGCCT
TCGGGTTGTAAACCGCTTTTGATTGGGAGCAAGCCTTTTGGGTGAGTGTACCTTTCGAAT
AAGCGCCGGCTAACTACGTG
>6315 len=140 tot-seq=1
TGGGGAATATTGGACAATGGGCGGAAGCCTGATCCAGCAACGCCGCGTGAGGGATGACGG
CCTTCGGGTTGTGAACCTCTTTCAGCACAGACGAAGCGAAAGTGACGGTATGTGCAGAAG
GAGGACCGGCCAACTACGTG
>6316 len=140 tot-seq=1
TGGGGAATATTGCACAATGGGCGGAAGCCTGATGCAGCAACGCCGCGCGCGGGATGACGG

CCTTCGGGTTGTAAACCGCTTTCAGCAGGGAAGAAGCGCAAGTGACGGTACCTGCAGAAG

AAGTACCGGCTAACTACGTG

>6317 len=140 tot-seq=1

TAGGGAATCTTCCGCAATGGGCGAAAGCCTGACGCAGCGACGCAGCGTGCGGGATGACGG

CCTTCGGGTTGTAAACCGCTTTCAGCAGGGAAGAAGCGCAAGTGACGGTACCTGCAGAAG

AAGTACCGGCTAACTACGTG

>6318 len=140 tot-seq=1

TGGGGAATATTGGACAATGGGCGAAAGCCTGATCCAGCAACGCCGCGTGAGGGATGACGG

CCTTCGGGTTGTAAACCTCTTTCAGCGCAGACGAAGCGAAAGTGACGGTATGCGCAGAAG

AAGCACCGGCCAACTACGTG

>6319 len=140 tot-seq=1

TGGGGAATATTGCACAATGGGCGAAAGCCTGATACAGCGACGCCGCGTGCGGGATGACGG

CCTTCGGGTTGTAAACCGCTTTCAGCTCTGACGAAGCTTTTGTGACGGTAGGAGCAGAAG

GAGCACCGGCTAACTACGTG

>6320 len=140 tot-seq=1

TAGGGAATCTTCCACAATGGGCGCAAGCCTGATGCAGCAACGCCGCGTGCGGGATGACGG

CCTTCGGGTTGTAAACCGCTTTCAGTAGGGACGAAGCTAACGTGACGGTACCTACAGAAG

AAGGACCGGCTAACTACGTG

>6321 len=140 tot-seq=1

TGGGGAATCTTCCGCAATGGACGAAAGTCTGACGGAGCAACGCCGCGTGAGTGAAGACGG

CCTTCGGGTTGTAAACCTCTTTCAGCAGGGACGAAGCGCAAGTGACGGTACCTGCAGAAG

AAGCACCGGCTAACTACGTG

>6322 len=140 tot-seq=1

CGCAAGTCTGATGGAGCAACGCCGCGTGAGTGAAGAAGGGTTTCGGCTCGTAAAGCTCTG

TTAATCGGGACGAATGGTCCTTGTGTGAATAATGCAAGGATTTGACGGTACCGGAATAGA

AAGCCACGGCTAACTACGTG

>6323 len=140 tot-seq=1

TGGGGAATCTTGCACAATGGGCGAAAGCCTGATGCAGCGACGCCACGTGCGGGAAGAAGG

CCCTCGGGTCGTAAACCGCTTTCAGCAGGGACGAGGCCGAAAGGTGACGGTACCTGCAGA

AGAAGCCCCGGCTAACTACG

>6324 len=140 tot-seq=1

TGGGGAATATTGCGCAATGGGGGAAACCCTGACGCAGCGACGCCGCGTGAGGGATGACGG

CCTTCGGGTTGTAAACCTCTGTTAGCATCGAAGAAGCGAAAGTGACGGTAGGTGCAGAGA

AAGCGCCGGCTAACTACGTG

>6325 len=140 tot-seq=1

TGGGGAATTTTGGACAATGGGCGCAAGCCTGATCCAGCAATGCCGCGTGCGGGAAGAAGG

CCTTCGGGTTGTAAACCGCTTTCGCCTGTGACGAAGCGTGAGTGACGGTAATGGGTAAAG

AAGCACCGGCTAACTACGTG

>6326 len=140 tot-seq=1

TGGGGAATATTGCACAATGGGCGCAAGCCTGATGCAGCGACGCCGCGTGGGGGATGACGG

CCTTCGGGTTGTAAACCTCTTTCAGTACCGACGAAGCGTAAGTGACGGTAGGTACAGAAG

AAGCACCGGCCAACTACGTG

>6327 len=140 tot-seq=1

TGGGGAATATTGCACAATGGGGGAAACCCTGATGCAGCGACGCCGCGTGGAGGAAGAAGG

TCTTCGGATTAGAAACCCCTGTAGTCCTTCAAGATCGGAAGAGCGTCGTGTAGGGAAAGA

GTATGCCAATGTGTAGATCT

>6328 len=140 tot-seq=1

CGGAAGTCTGACCGAGCAACGCCGCGTGAGGGAAGAAGGGTTTCGGATCGTAAAGCTCTG

TTGTTAGAGAAGAACGGTGGTAGGAGTGGAAAATCGACCAAGTGACGGTAACGAACCAGA

AAGGGACGGCTAACTACGTG

>6329 len=140 tot-seq=1

TGGGGAATCTTGCACAATGGGCGAAAGCCTGATGCAGCGACGCCGCGTGCGGGATGAAGG

CCTTCGGGTTGTAAACCTCTTTCAGTACCGACGAAGCGCAAGTGACGGTAGGTACAGAAG

AAGCACCGGCCAACTACGTG

>6330 len=140 tot-seq=1

TGGGGAATATTGCACAATGGGCGCAAGCCTGATGCAGCAACGCCGCGTGAGTGAAGAAGT

ATTTCGGTATGTAAAGCTCTATCGGCAGGGAAGAAAGGCCCTTAACAGGGAGATGACGGT

ACCTGACTAAGAAGCCCCGG

>6331 len=140 tot-seq=1

TGGGGAATATTGGGCAATGGAGGAAACTCTGACCCAGCGACGCCGCGTGAGGGATGAAGG

CCTTCGGGTTGTAAACCTCTTTCAGCAGGGAATAAGCGAAAGTGAAGGTACCTGCAGAAG

AAGCACCGGCTAACTATGTG

>6332 len=140 tot-seq=1

TGGGGAATCTTCCGCAATGGACGAAAGTCTGACGGAGCAACGCCGCGTGAGTGAAGACGG

CCTTCGGGTTGTAAACCGCTTTCGCCTGTGACGAAGCGTGAGTGACGGTAATGGGTAAAG

AAGCACCGGCTAACTACGTG

>6333 len=140 tot-seq=1

TGGGGAATATTGGACAATGGAGGTAACTCTGATCCAGCAATTCTGTGTGTGTGTGAAGAA

GGTTTTAGGACTGTAAAACACTTTTAGTAGGGAAGAAAAAAGTGACGGCACCTACAGAAG

AAGCAACGGCTAAATACGTG

>6334 len=140 tot-seq=1

CTCAACTCCCCCATACCACTCTGTGCTTTTCCCCTGGCCCTCCTGCCACTAAGACTGCAT

GTAACTAAGTTTCTCCAGGATGCGTCCCCTAGATCAGAGAACCAGGAATTGGCAAACTAC

AGCCTGCAAATCAAATCCAG

>6335 len=140 tot-seq=1

TGAGGAATATTGGTCAATGGGCGCTAGCCTGAACCAGCCAAGTAGCGTGAGGGATGACGG

CCTTCGGGTTGTAAACCTCTTTCAGCAGGGACGAAGCGCAAGTGACGGTACCTGCAGAAG

AAGCACCGGCTAACTACGTG

>6336 len=140 tot-seq=1

TGGGGAATATTGCGCAATGGTAAACCCTGACGCAGCGACGCCGCGTGCGGGATGAAGGCC

TTCGGGTTATAAACCGCTTTTGATTGGGAGCAAGCCTTCGGGTGAGTGTACCTTTCGAAT

AAGCGCCGGCTAACTACGTG

>6337 len=140 tot-seq=1

GACAAAACTCAGTCAGCTTGGTGGTGACACAAGCCAGGTCACTTAGGAGGGTCACCAGTT

TGTAGCTGCCTTGAGAAACATGGTCTTGCTGCACTGGGCTCAGTGGAGGCGAGCGGTTTG

TGCACTGTTATCCAGACAGG

>6338 len=140 tot-seq=1

TGGGGAATATTGCACAATGGGCGCAAGCCTGATGCAGCGACGCCGCGTGGGGGATGACGG

CCTTCGGGTTGTAAACCTCTTTCAGCGCCGACGAAGCGAAAGTGACGGTAGGCGCAGAAG

AAGCACCGGCCAACTACGTG

>6339 len=140 tot-seq=1

TGGGGAATATTGCGCAATGGGCGGAAGCCTGACGCAGCGACGCCGCGTGGGGGATGACGG

CCTTTGGGTTGTAAACCTCTTTCGGCCCCGACGAAGCGTGAGTGACGGTAGGGGTAGAAG

AAGCACCGGCCAACTACGTG

>6340 len=140 tot-seq=1

TAGGGAATCTTCCACAATGGGCGAAAGCCTGATGGAGCAACGCCGCGTGGGTGAAGAAGG

TCTTCGGATTGTAAACCCCTGTCATGTGGGAGCAAATTAAAGAGATAGTACCACAAGAGG

AAGAGACGGCTAACTCTGTG

>6341 len=140 tot-seq=1

TGGGGAATATTGCACAATGGGCGCAAGCCTGATGCAGCGACGCCGCGTGAGGGATGGAGG

CCTTCGGGTTGTAAACCTCTTTTATCGGGGAGCAAGCGAGAGTGAGTTTACCTGCAGAAG

AAGCCCCGGCTAACTACGTG

>6342 len=140 tot-seq=1

TGGGGAATCTTCCGCAATGGACGAAAGTCTGACGGAGCGACGCCGCGTGAGGGATGGAGG

CCTTCGGGTTGTAAACCTCTTTTATCGGGGAGCAGGCGAGAGTGAGTTTACCCGTTGAAT

AAGCACCGGCTAACTACGTG

>6343 len=140 tot-seq=1

TAGGGAATCTTCGGCAATGGGCGAAAGCCTGACCGAGCAACGCCGCGTGAATGAAGAAGG

CCTTCGGGTTGTAAACCCCTGTCATGTGGGAGCAAATTAAAAAGATAGTACCACAAGAGG

AAGAGACGGCTAACTCTGTG

>6344 len=140 tot-seq=1

TGAGGAATATTGGTCAATGGGCGCAAGCCTGATGCAGCGACGCCGCGTGAGGGATGGAGG

CCTTCGGGTTGTAAACCTCTTTTATCGGGGAGCAAGCGTGAGTGAGTTTACCCGTTGAAT

AAGGACCGGCTAATTCCGTG

>6345 len=140 tot-seq=1

TGAGGAATATTGGTCAATGGGCGAGAGCCTGATGCAGCGACGCCGCGTGAGGGATGGAGG

CCTTCGGGTTGTAAACCTCTTTTATCGGGGAGCAAGCGAGAGTGAGTTTACCCGTTGAAT

AAGCACCGGCTAACTACGTG

>6346 len=140 tot-seq=1

AAGGGGAGAGAGAAGGAAAAAGACCAGGAGCGGGCAGGAGGACGGCCCGAGGGGGTGTAA

ACTGCTTTTATATGGGAATAAAGTGAGGGACGTGTCCCTTATTGCATGTACCATACGAAG

AAGGACCGGCTAATTCCGTG

>6347 len=140 tot-seq=1

GGGGATATTGCGCAATGGGGGAAACCCTGACGCAGCGACGCCGCGTGCGGGATGAAGGCC

TTCGGGTTGTGAGCCGCGTTTGATTGGGAGCAAGCCTTCGGGTGAGTGTACCTTTCGAAT

AAGCGCCGGCTAACTACGTG

>6348 len=140 tot-seq=1

CGGGGAATCTTGCACAATGGGCGAAAGCCTGATGCAGCGACGCCGCGTGCGGGATGAAGG

CCTTCGGGTTGTAAACCGCTTTCAGCAGGGACGAAGCGCAAGTGACGGTACCTGCAGAAG

AAGCACCGGCTAACTACGTG

>6349 len=140 tot-seq=1

TGGGGAATCTTCCGCAATGGGCGCAAGCCTGACGGAGCAACGCCGCGTGAGGGATGGAGG

CCTTCGGGTTGTAAACCTCTTTTATCGGGGAGCAAGCGAGAGTGAGTTTACCCGTTGAAT

AAGCACCGGCTAACTACGTG

>6350 len=140 tot-seq=1

TGGGGAATTTTGCACAATGGGGGAAACCCTGATGCAGCGACGCCGCGTGAGGGATGGAGG

CCTTCGGGTTGTAAACCCCTTTTATCGGGGAGCAAGCGAGAGTGAGTTTACCCGTTGAAT

AAGCACCGGCTAACTACGTG

>6351 len=140 tot-seq=1

GGAGTTGGAGATAAAGGACACAGGGCTCCTGCCCTCAGCATGTGCACTGTTCATGGTGCT

GGAGAGCAGGTGGGGATGAGGCTCCCTCTCTTGGATCTGTGAATGCAGCATCCCATGCAT

GACTGAGTCCTACAGAAAGT

>6352 len=140 tot-seq=1

TGGGGAATTTTGCACAATGGGGGAAACCCTGATGCAGCAACGCCGCGTGCGGGATGACGG

CCTTCGGGTTGTAAACCGCTTTCAGCAGGGACGAAGCGCAAGTGACGGTACCTGCAGAAG

AAGCACCGGCTAACTACGTG

>6353 len=140 tot-seq=1

TAGGGAATCTTCCACAATGGACGCAAGTCTGATGGAGCAACGCCGCGTGAGGGATGACGG

CCTTCGGGTTGTAAACTCCTTTCACCATCGACGAAGGTTTTCTGACGGTAGATGGAGAAG

AAGCACCGGCTAACTACGTG

>6354 len=140 tot-seq=1

TGGGGAATCTTCCGCAATGGGCGAAAGCCTGATGCAGCGACGCCGCGTGAGGGATGACGG

CCTTCGGGTTGTAAACCTCTTTCAGCAGGGACGAAGCGCAAGTGACGGTACCTACAGAAG

AAGCAACGGCTAAATACGTG

>6355 len=140 tot-seq=1

TCTCAACGAACAGTACCGCACTCTCATCATCGTTCAAACTGAGCCCGTTCGGAAAATAGA

GCTCCGACAAAACAACGGTAACTTCACCGTTTGGATCACGACGCAACAGCCGTCCTGATG

GTCTGTGCTCGATGAACGCT

>6356 len=140 tot-seq=1

ACGCAAGTCTGATGGAACAACGCCACGTGAGTGAAGAAGGTTTTCGGATCGTAAAGCTCT

GTTGTTGGTTAAGAAGGATAGAGGTAGTAACTGGCCTTTATTTGGCGGTAATCAACCAGA

AAGTCACGGCTAACTACGTG

>6357 len=140 tot-seq=1

TGGGGAATATCGCACAATGGACGGAAGTTTGATGCAGCGACGCCGCGTGAGGGATGACGG

CCTTCGGGTTGTAAACCTCTTTCAGCAGGGACGAAGCGCAAGTGACGGTACCTGCAGAAG

AAGCACCGGCTAACTACGTG

>6358 len=140 tot-seq=1

TGGGGAATATTGGACAATGGGCGAAAGCCTGATCCAGCAACGCCGCGTGAGGGATGAATG

CCTTCGGGTTGTAAACCTCTTTCGCCTGCGACGAAGCGAGAGTGACGGTAGCAGGTAAAG

AAGCACCGGCCAACTACGTG

>6359 len=140 tot-seq=1

GGGTAGGGGGAGCAGAGCAACTGTGCGACTCCAGGAGATGGGATGTGGGCTCAGTGGAGG

CCGTTGCAGGGGACCAGGTAGGCATCTGGACCTTCTGGCTAGGCCAGCTTCAAGCTCGAG

TTGCGGGGAGGCCTCTCCTA

>6360 len=140 tot-seq=1

CCCCAGACCCAGGGGGCCTGGAGCCACATCCTCCCCACCAGCCAGCAGCTTGGCATCAGG

TCCATGGGATCTGTGAACAAGAAAAGGAGAGGGGATAGGCTGGGAGGATAGAACTCCCAG

GAGCCTGAGGAAGGCGATCC

>6361 len=140 tot-seq=1

TGAATCAATCTCAGTGGGAGGTTCATAGTGAGAGGGAAGATGGAACACCTGAAACCAAAG

GGGGAAAAACAAACTGCAAATACATATATATTTTCTTTGCTAACATTACTTGGTAATTAG

ACAATTACAGGAAACAGCTT

>6362 len=140 tot-seq=1

TGGGGAATATTGCACAATGGGCGCAAGCCTGATGCAGCGACGCCGCGTGGGGGATGAAGG

CCTTCGGGTTGTAAACTCCTTTCAGCCATGACGAAGCGTAAGTGACGGTAGTGGTAGAAG

AAGCACCGGCTAACTACGTG

>6363 len=140 tot-seq=1

GCCCCTTGTGGCCTCACCCTGTTCCCACCGCGCCCCTTGCCCACAGCTTCACCCTCCCCA

GAGCTGGTCCGGTACTCTACGCTCAACTCTGAGCACTTCCCACAGCCCACACAGCAGATC

AAGAATATTGTCAGGCAGTA

>6364 len=140 tot-seq=1

TGGGGAATATTGCGCAATGGGGGAAACCCTGACGCAGCAACGCCGCGTGAGGGACGACGG

CCTTCAGGTTGTAAACCTCTTTTAGCAAGGAAGAAGCGAAAGTGACGGTACTTGCAGAAA

AAGCGCCGGCTAACTACGTG

>6365 len=140 tot-seq=1

TAGGGAATCTTCCACAATGGACGCAAGTCTGATGGAGCAACGCCGCGTGGGGGATGAAGG

CCTTCGGGTTGTAAACTCCTTTCGCTAGGGACGAAGCGCAAGTGACGGTACCTGGAGAAG

AAGCACCGGCTAACTACGTG

>6366 len=140 tot-seq=1

TGGGGAATATTGCGCAATGGGGGGAACCCTGACGCAGCCATGCCGCGTGAGGGATGACGG

CCTTCGGGTTGTAAACCTCTTTCAGCAGGGACGAAGCGCAAGTGACGGTACCTGCAGAAG

AAGCACCGGCTAACTACGTG

>6367 len=140 tot-seq=1

CGGCATACGAGATCGGTGTGTGACTGGAGTGCAGACGTGTGCTCTTCCGATTCCTACGGG

AGGCAGCGGTAGGCCTTTCTAACAAACAAGCACGTACACCCCCTGAGTCTGTAAAATACC

AAAACAATGACGACAGCACC

>6368 len=140 tot-seq=1

CCCTCACCTTGGCCCGAGCAGCAGCCCTGGTCTCGGCGTCCATCCAGTCCAGCTCCTCCA

GGCGCTGGCCCAGGGTGTACTTGATGTCTTCCACTAGCTGCTGCACCTGCAGGGTCAGGG

GTCAGGGAGCAAGGGTCAAC

>6369 len=140 tot-seq=1

TGAGGAATATTGGTCAATGGACGAAAGCCTGATGCAGCGACGCCGCGTGAGGGATGACGG

CCTTCGGGTTGTAAACCTCTTTCAGTAGGGACGAAGCGCAAGTGACGGTACCTGCAGAAG

AAGCACCGGCTAACTACGTG

>6370 len=140 tot-seq=1

CATGCGTGGCGCTGAGGATGCCCTGAGAGACCAGTGGAGAAGCAGGGGTGCGGCCTGGAG

TTCCCATCACCCTCACAATACCTTCTGCTCGTACTGCTGCTTCATGGACCGGAAATGCTG

GTCCCATTGCTTGTTCACTT

>6371 len=141 tot-seq=1

TGGGGAATAGGGCACAATGGGGGGAAGCCTGATGCAGACATGCCGCGAGTATGAAGAAGG

CCTTCGGGTGGTAAAGTACTTTCAGCGGGGAGGAAGGGAGTAAAGTTAATACCTTTGCTC

ATTGACGTTACCCGCAGAAGA

>6372 len=141 tot-seq=1

TGGGGAATATTGCACAATGGGCGGAAGCCTGATGCAGCAACGCCGCGTGCGGGATGACGG

CCTTCGGGTTGTAAACCGCTTTCACCATCGACGAAGCTTTTTGTGACGGTAGGTGGAGAA

GAAGCACCGGCTAACTACGTG

>6373 len=141 tot-seq=1

TAGGGAATCTTCGGCAATGGACGGAAGTCTGACCGAGCAACGCCGCGTGGGGGATGACGG

CCTTCGGGTTGTAAACTCCTTTCGCTAGGGACGAAGCTTTTTGTGACGGTACCTAGATAA

GAAGCACCGGCTAACTACGTG

>6374 len=141 tot-seq=1

GGCCGTATTCCCTCTGAAGGCGCCAGAGAAGGGTCCATCTTTGCCCTCCCAGCTTCCGGC

GGTCACCAGCAGGCCTTAGCGTTTGCTGGCGCAGCTGCATGGCTCTGATCTCTGCCTCTG
TCTTCACAGGGATTTCTTCCC
>6375 len=141 tot-seq=1
TGGGGAACCTTCCGCAATGGGCGCAAGCCTGACGGAGCAATGCCGCGTGAGTGAAGAAGG
CCTTCGGGTTGTAAACCGCTTTTGATTGGGAGCAAGCTTTCGGGTGAGTGTACCTTTCGA
ATAAGCGCCGGCTAACTACGT
>6376 len=141 tot-seq=1
CGGAAAGGGGGACGCAGAGAAAGGCAGCCTCGGCGCACCGCGGCCAGGGAGGCAGAGGCC
CCTCGCGGCGCCGGCTCTGGAGACCTAAACTCCGCCGGGGCTGTGCCCAGGGCGCGTCTG
AGCGTCGCCGCCTTCACCCTC
>6377 len=141 tot-seq=1
TGGGGAATATTGCACAATGGGCGCAAGCCTGATGCAGCGACGCCGCGTGGGGGATGACGG
CCTTCGGGTTGTAAACTCCTTTCGCAACCGACGAAGCCCTTGTGGTGACGGTAGGTTGAG
AAGAAGCACCGGCTAACTAGG
>6378 len=141 tot-seq=1
GACGAAAGTCTGATGGAGCAACGCCGCGTGAGTGAAGAAGGTTTTCGGATCGTAAAGCTC
TGTTGTTGGTGAAGGGGAGGGGAGGTAGTAACAGGCCTTTATTTGACGGTAATCAACCAG
AAAGTCACGGCTAACTACGTG
>6379 len=141 tot-seq=1
GAGATGGGGAAGGTATCCAGGGCCCCCTCAGCCGCTGCCCCGCTTGGGCAGATCCCGCAT
GCTTTTCTGCACAGCCATTTTGCATTCCTGCCTGCATTCAGTAGATAAACAAAGAAAGAG
CAGGTCCCTGGCATGCAGAGC
>6380 len=141 tot-seq=1
TGAGGAATATTGGTCAATGGTCGGGAGACTGAACCAGCCAAGCCGCGTGAGGGAATGAAG
GTCTTCGGATTGTAAACCCCTGTCATGTGGGAGCAAATTAAAAAGATAGTACCACAAGAG
GAAGAGACGGCTAACTCTGTG
>6381 len=141 tot-seq=1
GAGACAAGCTCAGGAGCTTGAATGCCAGACCAGAGTACTTGGGCCACAGTCTGGAGACAG
TGGGAGACTGGGGAAGGTATGGCGGGGGCGCTGCACAAGGTCACATAGAAGCCAAAGCTT

GGGGAGAAAGAGCTGCCATCC

>6382 len=141 tot-seq=1

TCTGAGCTGCGCTAAAAGGATAATTAATTGGAACGAAAACACCAGCTCCAGCTCCTCTGA

TCTGTTCCTCTCAGAGAATTCACAGTTGTCTTCCTCCAAGCCCCTCCCCCCTCCCAGACT

CCAGGCTAATTCCTTCAGATG

>6383 len=141 tot-seq=1

TGAGGAATATTGGTCAATGGGAGAGATCCTGAACCAGCCAAGCCGCGTGGGGGAATGAAG

GTCTTCGGATTGTAAACCCCTGTCATGTGGGAGCAAATTAAAAAGATAGTACCACAAGAG

GAAGAGACGGCTAACTCTGTG

>6384 len=141 tot-seq=1

TGGGGAATATTGCGCAATGGGGAAACCCTGACGCAGCGACGCCGCGTGCGGGATGAGGGC

CTTCGGGTTGTAGACCGCTTTTGATTGGGAGCAAGCCCTCGGGTGAGTGTACCTTTCGAA

TAAGCGCCGGCTAACTACGTG

>6385 len=141 tot-seq=1

TGAGGAATTTTTCACAATGGACGAAAGTCTGATGGAGCAACGCCGCGTGGGGGATGACGG

CCTTCGGGTTGTAAACTCCTTTCGACAGGGACGAAGCTTTTTGTGACGGTACCTGTATAA

GAAGCACCGGCTAACTACGTG

>6386 len=141 tot-seq=1

TGCCTTTGGCTGGGAGGAGGGATGGGGCATTCACAGGAGCAGGCCGGGGTGGACAGCGCC

CAATGCCACAAGTACAGTGGGTGGGCACCAGCAAAGTCCTCTTCCAATGCCTTCAGCTTC

TCAGGGAAGGAGGGTGCAAGG

>6387 len=141 tot-seq=1

GCGAAAGCCTGACGGAGCAACGCCGCGTGAGTGAAGACGGCCTTCGGGTTGTAAAGCTCT

GTGATTCGGGACGAAAGATAAGCAGACAAATAATCTGCATAAGAGACGGTACCGAAAAAG

CAAGCCACGGCTAACTACGTG

>6388 len=141 tot-seq=1

GAGGGTTCTACGTAAAACGTCGGTCCAGAGTTCAAACCCCAAACTAATAGTTTTGGATTT

CCCCAAGTACTACCCACTTAATATGTCACCAGGAAAGACCCATCACACACATATATGGAA

GGAAAAATTAAAGTTTCTCCT

>6389 len=141 tot-seq=1

CAAAAATATACCAGGACCATGCTAGGAGTTCACTCTCCCTGTCAGGATAGGTAGGACCAA

AGAAATAACTCTCAGTTAGCTTTTAGACCATTTCCAGAAATATCAGTGACATGGCATCAT

CAACTTTTACTTCACTTCACC

>6390 len=141 tot-seq=1

CTGGAACACAAGTCTCCCCACTGAGAAACTTGACCCCTCATTCTCAACCCCAGTCTCAGA

GCAATACTGAGGTAGTGCCTATGGGTCCAGGCAGACTCATGGAGGCAGAGGCCAAGGCCA

CACCCAGTCAGTAATAATCAC

>6391 len=141 tot-seq=1

GAACCTGAGGCTGAGCTGGCTCCTAGCCAGGACCCTCCGAGCTGCTGCCCTGCAGCACCA

CGGCCTGCCGAAGCACCCCTTCAGCCCAGTGCAGCCACAGCCGGCCTGGTGCACATCCTG

CCCCGGTTTTTTTTTTTTTAA

>6392 len=141 tot-seq=1

TGCAGTGAGCCGAGATCGCGCCACTGCACTCCAGCCTGGGCGACAGAGCAAGACTCCGTC

TCAAAAACAAAAACAAAAACAAAAAACCACTTTAAGGAACTGCCGAACTGCTTTCCAAAG

TGGTTGCACCATTTCACATTC

>6393 len=141 tot-seq=1

TGCGGGTAGCGGTCTAGGAACTAGGCATGGGGCAGGCCCACGTCACACTGCACTGAGGGA

GGGCCCGTCCTGTCAGGCAGGCTCCTGAATCCACACCTCCACCACTTCAGAGGCCTCATT

TCCTCAGTCCTCACAGTAACC

>6394 len=141 tot-seq=1

TGGGGAATATTGCACAATGGGCGCAAGCCTGATGCAGCGACGCCGCGTGGGGGATGACGG

CCTTCGGGTTGTAAACTCCTTTCGCCAGGGACGAAGCGTTTTGTGACGGTACCTAACCAG

AAAGTCACGGCTAACTACGTG

>6395 len=141 tot-seq=1

TGGGGAATATTGCGCAATGGGGGAAACCCTGACGCAGCGACGCGGGGGGCTGGATGAAGG

CCTTCGGGTTGTAAACCGCTTTTGATTGGGAGCAAGCCTTCGGGTGAGTGTACCTTTCGA

ATAAGCGCCGGCTAACTCCGT

>6396 len=141 tot-seq=1

CCGCCACATCGGCGGCGGCCACGGACAGCTTCCCGTCCGCCACCTCATGGATAGCCAGGG
TCACAGGTTTGACATCCAGCTTCTCCCCCATATCCTCTGCCTCCTGGGCGATCTGGCGGG
CCCGGCGGGCCACCACATTCA
>6397 len=141 tot-seq=1
TTTTTTTTTTCAAGCAGAAGACGGCATACGAGATCGGTGTGTGACTGGAGTTCAGACGTG
TGCTTTTCCGATCTCCTACGGGAGGCAGTAGCCAGCCTGAGAAGGGAGGGAGTGTTCTAC
TCTCCTGGAAAAGAGCTGAGG
>6398 len=142 tot-seq=1
TTTTTTTTTTCAAGCAGAAGACGGCATACGAGATAAGGCCGTGACTGGAGTTCAGACGTG
TGCTCTGATCTCCTACGGAGGCAGCAGGAGGCTTTGCTGATCAACTTCCCGCCAACTGAG
AAACAGAGAAAGGAGGCCAGAG
>6399 len=142 tot-seq=1
CTGGTTGGCGAAGGCAAAGACCAAGTCGGTGAGGGGGCCAAAGCCAGCGTTGTGCATCTG
AGTCCGGTTCAGGGTCAGTCCATCTGAGAAGGTCATTGTGTCTTGCTCAGGCGTGTACCG
CGTGCAGATTCGCAGAATCTGG
>6400 len=142 tot-seq=1
CAGCTACCTAGCTAGTTCCAGGCTACCCTGGTCTATGTGAGATCCTGTCTCACAAAAACA
AGAACAAACAAATAAACAAAAAACCTATATGTATACACCTATATGTATATGTATATGTAT
ATGTATATGTATATGTATATGT
>6401 len=142 tot-seq=1
ATGCATAAACTCAACAATGCCTTCCAGGCACTGCGCGAGGTCATCCCGCACGTGCGGGCT
GACAAGAAGCTCTCCAAGATCGAGACCCTCACGCTGGCCAAGAACTATATCAAGTCGCTG
ACCGCCACCATACTTACTATGT
>6402 len=142 tot-seq=1
TGGGGAATATTGCACGATGGGCGCAAGCCTGATGCAGCAACGCCGCGTGCGGGATGAAGG
CCTTCGGGTTGTAAACCGCTTTCAGCAGGGACGAGGCCGCAAGGTGACGGTACCTGCAGA
AGAAGCCCCGGCTAACTACGTG
>6403 len=142 tot-seq=1
TGGGGAATTTTGGACAATGGGCGAAAGCCTGATCCAGCAATGCCGCGTGTGTGAAGAAGG

CCTTTGGGTTGTAAGCCGCTTTTGATTGGGAGCAAGCTTTCGGGTGAGTGTACCTTTCGA

ATAAGCGCCGGCTAACTACGTG

>6404 len=142 tot-seq=1

TGGGGAATATTGCGCAATGGGGGAAACCCTGACGCAGCGACGCCGCGTGCGGGATGAAGG

CCTTCGGGTTGTAAACCGCTTTCAGCAGGGACGAGGCCGCAAGGTGACGGTACCGTAAGA

GAAAGCCACGGCTAACTACGTG

>6405 len=142 tot-seq=1

TGGGGAATATTGGACAATGGGGGCAACCCTGATCCAGCCATGCCGCGTGTGTGAAGAAGG

CCCTCGGGTTGTAAACCGCTTTTGATTGGGAGCAAGCCTTCGGGTGAGTGTACCTTTCGA

ATAAGCGCCGGCTAACTACGTG

>6406 len=142 tot-seq=1

GGGGACTGCAGGAAACTCTGTATGGCTTTGTGCAGAATCCCTCTCTAGCATTCAGTCCCA

ACCCAGAGGAAAACCAGTCTCTTGCCCCGCTGCTGGTGGAAGAAGAGGAGGAGAAGAAAA

ATGATGATGAGGAGGAGTATGA

>6407 len=142 tot-seq=1

ATCACCTGAGGTCAGGAGTTCGAGACAAAAACCAACATGGAAAAATCCCCTCTCTACTAA

AAATACGAAATTAGCCAGGCGTGGTGGCACATGCCTGTAATCCCAGCTGCTCAGGAGGCT

GATGCAAGAGAATCGCTTGAAC

>6408 len=142 tot-seq=1

GTGATGGGAGGAGAAGAGATGCAGGCAGAGAGTGAGTCCAGCAGGCTGGGCAGGAGGAGG

GAGGCCATGCCGTGGACCGGGCCAGGGTTTGTATATACAGTTGCCTCTCACTCAGTCCCT

CACTAAGAAGCCCAGTGTGGTG

>6409 len=142 tot-seq=1

TCGGGAATTTTGCACAATGGCCGAAAGGCTGATGCAGCAACGCCGCGTGGGGGAAGAAGG

CCTTCGGGTTGTAAACCGCTTTTGATTGGGAGCAAGCTTTCGGGTGAGTGTACCTTTCGA

ATAAGCGCCGGCTAACTACGTG

>6410 len=142 tot-seq=1

TAGGGAATCTTCCACAATGGACGCAACCCTGACGCAGCGACGCCGCGTGCGGGATGAGGG

CCTTCGGGTCGTAAACCGCTTTTGATTGGGAGCAAGCTTTCGGGTGAGTGTACCTTTCGA

ATAAGCGCCGGCTAACTACGTG

>6411 len=142 tot-seq=1

TAGGGAATCTTCGGCAATGGACGCAAGTCTGAACCAGCCAAGTAGCGTGCAGGATGAAGG

CCTTCGGGTTGTAAACCGCTTTTGATTGGGAGCAAGCTTTCGGGTGAGTGTACCTTTCGA

ATAAGCGCCGGCTAACTACGTG

>6412 len=142 tot-seq=1

AGCAGACCAGCACTGGTGCCACAGCTCCAATAGAGGCCACAGTCGCAGTGCCTAGGAGCA

GTAAGATTTTTTCACTTCCTCTCGCCAGATGGGTCTCATTGCCAGCTTCCAGCATAGTGG

TCCTGCTTCTGCCTGAAACTGG

>6413 len=142 tot-seq=1

TGGGGGATATTGCACAATGGACGCAAGTCTGATGCAGCGACGTCGTGTGGGGGATGAAGG

CCTTCGGGTTGTAAACCGCTTTCAGCAGGGACGAGGCCGCAAGGTGACGGTACCTGCAGA

AGAAGCCCCGGCTAACTACGTG

>6414 len=142 tot-seq=1

TGGGGAATCTTCCGCAATGGACGAAAGTCTGACGGAGCAACGCCGCGTGAGTGATGACGG

CCTTCGGGTTGTAAACCGCTTTTGATCGGGAGCAAGCCTACGGGTGAGTGTACCTTTCGA

ATAAGCACCGGCTAACTACGTG

>6415 len=142 tot-seq=1

TAGGGAATTTTCGGCAATGGGCGAAAGCCTGACCGAGCAACGCCGCGTGAGTGATGACGG

CCTTCGGGTTGTAAACCGCTTTTGATTGGGAGCAAGCTTTCGGGTGAGTGTACCTTTCGA

ATAAGCACCGGCTAACTACGTG

>6416 len=142 tot-seq=1

TAGGGAATCTTCGGCAATGGGGGCAACCCTGACCGAGCAACGCCGCGTGAGTGAAGAAGG

CCTTCGGGTTGTAAACCGCTTTTGATTGGGAGCAAGCTTTCGGGTGAGTGTACCTTTCGA

ATAAGCGCCGGCTAACTACGTG

>6417 len=142 tot-seq=1

TAGGGAATCTTCGGCAATGGGGGCAACCCTGATGCAGCGACGCCGCGTGCGGGATGAAGG

CCTTCGGGTTGTAAACCGCTTTCAGCAGGGACGAGGCCGCAAGGTGACGGTACCTGCAGA

AGAAGCCCCGGCTAACTACGTG

>6418 len=142 tot-seq=1

AGGGGAATATTGCGCAATGGAGGAAACCCTGACGCAGCGACGCCACGTGCGGGATGAAAG

CCTTCGGGTTGTAAACCGCTTTTGATTGGGAGCAAGCCTTCGGGTGAGTGTACCTTTCGA

ATAAGCGCCGGCTAACTACGTG

>6419 len=142 tot-seq=1

TTGACCTCACCCTGGCCCTCCTCACCCCCGCGCAGGTCCCTCCACGAGCTGTCCCGGGAG

ATGGGGCGCGAGGTCTGCACGCCGGCGTGCCGCACGCCGGCCCCGTGCTGCCGGCACTTG

CTCAGCAGCTCCTCGTACTTCT

>6420 len=142 tot-seq=1

CACTACTTGGACAAATAGATAATTCTGGGACTGGAGTGAAAAATCAAACTTAAGGTAGAG

TATTCTGTAAAGCTAGAAAGTTAAAAAGTGCTTTATGAACAACTGAAAGAGACATGTCAG

AGGAAGCCAACCTGAAAGAGTT

>6421 len=142 tot-seq=1

TGGGGAATATTGCACAATGGGCGCAAGCCTGATGCAGCGACGCCGCGTGCGGGATGACGG

CTTTCGGGTTGTAAACCGCTTTCAGCAAGGAAGAAGCTTAACTGTGACGGTACTTGCAGA

AGAAGCGCCGGCTAACTACGTG

>6422 len=142 tot-seq=1

TGGGGAATATTGCGCAATGGGGGGAACCCTGACGCAGCGACGCCGCGTGCGGGATGAAGG

CCTTCGGGTTGTAAACCGCTTTTGATTGGGAGCAAGCCTTTTTGGTGACGGTACCTGCAG

AAGAAGCCCCGGCTAACTACGT

>6423 len=142 tot-seq=1

TGGGGAATATTGCGCAATGGGGGAAACCCTGACGCAGCGACGCCGCGTGCGGGATGAAGG

CCTTCGGGTTGTAAACCGCTTTTGATTGGGACGAGGCCGCAAGGTGACGGTACCTGCAGA

AGAAGCCCCGGCTAACTACATG

>6424 len=142 tot-seq=1

TGGGGAATATTGCACAATGGGCGAAAGCCTGATGCAGCGACGCCGCGTGGGGGATGAAGG

CCTTCGGGTTGTAAACTCCTTTCGCTAGGGACGAAGAGTTACATTGACGGTACCTGGAGA

AGAAGCACCGGCTAACTACGTG

>6425 len=142 tot-seq=1

TGGGGAATATTGCACAATGGACGGAAGTCTGATGCAGCAACGCCGCGTGCGGGATGACGG

CCTTCGGGTTGTAAACCGCTTTCAATTGTGACGAAGCTTTTTTGTGACGGTAGCAGTAGA

AGAAGCACCGGCTAACTACGTG

>6426 len=142 tot-seq=1

TAGGGAATCTTCCACAATGGACGAAACCCTGACGCAGCGACGCCGCGTGCGGGATGAAAG

CCTTCGGGTTGTAAACCGCTTTTGATTGGGGGCAAGCTTTCGGGTGAGTGTACCTTTCGA

ATAAGCGCCGGCTAACTACGTG

>6427 len=142 tot-seq=1

TGGGGAATATTGCACAATGGGCGGAAGCCTGATGCAGCAACGCCGCGTGCGGGATGACGG

CCTTCGGGTTGTAAACCGCTTTCAGCAGGGACGAGGCCTTATGGTGACGGTACCTGCAGA

AGAAGCTCCGGCTAAATACGTG

>6428 len=142 tot-seq=1

TGGGGAATATTGCACAATGGGCGGAAGCCTGATCCAGGAACGCCGCGTGCGGGATGACGG

CCTTCGGGTTGTAAACCGCTTTCGGCAGGGACGAGGCCGCAAGGTGACGGTACCTGCAGA

AGAAGCCCCGGCTAACTACGTG

>6429 len=142 tot-seq=1

TAGGGAATCTTCGGCAATGGACGGAAGTCTGACCGAGCAACGCCGCGTGAGTGAAGAAGG

CCTTCGGGTTGTAAACCGCTTTCAGCAGGGACGAGGCCGCAAGGTGACGGTACCTGCAGA

AGAAGCCCCGGCTAACTACGTG

>6430 len=142 tot-seq=1

TGGGGAATATTGCCCAATGGGGGAAACCCTGACGCAGCGACGCCGCGTGCGGGATGAAGG

CCTTCGGGTTGTAAACCGCTTTTGGTTGGGGGCAAGCTTTCGGGTGAGTGTACCTTACGA

ATAAGCGCCGGCTAACTACATG

>6431 len=142 tot-seq=1

TGGGGAATCTTGCACAATGGGCGAAAGCCTGATGCAGCGACGCCGCGTGCGGGATGAAGG

CCTTTGGGTTGTAAACTCCTTTCAGTACAGAAGAAGCTTTATTGTGACGGTATGTGCAGA

AGAAGCGCCGGCTAACTACGTG

>6432 len=142 tot-seq=1

CTGGTGTGGAGTTCTGGGAGCAGGTAGGCTATGGGTGAGTACTGGTAGGATCTAGACGGA

TAGGGTGAGAGTAGAGTGGGAGGGCCAGGGAGTGCCCTCAGAGCAGGAGAAAGGGCACAA

GCAGAGGCGGTGGCAGGAACTC

>6433 len=142 tot-seq=1

TGGGGAATATTGCGCAATGGGGTAAACCATGACGCAGCGACGCCGCGTGCAGGATGAAGG

CCTTCGGGTTGTAAACCGCTTTTGATTGAGAGCAAGCCTTCGGGTGAGTGTACCTTTCGA

ATAAGCGCCGGCTAACTACGTG

>6434 len=142 tot-seq=1

TGGGGAATATTGGGCAATGGGCGCAAGCCTGACCGAGCAACGCCGCGTGAACGAAGAAGT

TATTCGTAATGTAAAGTTCTTTTATCAGGGAAGAAAGAGGAATTTGACGGTACCTGAGGA

ATAAGCTCCGGCTAACTACGTG

>6435 len=142 tot-seq=1

TAGGGAATCTTCCACAATGGACGCAAGTCTGATGGAACAACGCCGCGTGAGTGAAGAAGG

CCTTCGGGTTGTAAACCGCCTTTGATTGGGAGCAAGCCTTCGGGTGAGTGTACCTTTCGA

ATAAGCGCCGGCTAACTACGTG

>6436 len=142 tot-seq=1

TAGGGAATTTTTCACAATGGGCGCAAGCCTTATGAAGCAATGCCGCGTGAGTGAAGAAGG

GTTTCGGGTTGTAAACCGCTTTTGATTGGGAGCAAGCTTTCGGGTGAGTGTACCTTTCGA

ATAAGCGCCGGCTAACTACGTG

>6437 len=142 tot-seq=1

TGGGGAATATTGCACAATGGGCGGAAGCCTGATGCAGCGACGCCGCGTGAGGGATGACGG

CCTTCGGGTTGTAAACCTCTTTCAGTACGGAAGAAGCCTTTGGGTGACGGTACGTGCAGA

AGAAGCGCCGGCTAACTACGTG

>6438 len=142 tot-seq=1

TAGGGAATCTTGCACAATGGGCGCAAGCCTGATGCAGCGACGCCGCGTGAGGGATGGAGG

CCTTCGGGTTGTAAACCGCTTTTGATCGGGAGCAAGCCTTCGGGTGAGTGTACCTTTCGA

ATAAGCACCGGCTAACTACGTG

>6439 len=142 tot-seq=1

TGGGGAATATTGCACAATGGGCGCAAGCCTGATGCAGCGACGCCGCGTGCGGGATGACGG

CCTTCGGGTTGTAAACCGCTTTTGATCGGGAGCAAGCCTTCGGGTGAGTGTACCCTGTGA

GGAAGCCCCGGCTAACTACGTG

>6440 len=142 tot-seq=1

CAATGGGCGGGAGCCTGAACCAGCCAAGTCGCGTGAGGGAAGACGGTCCTACGGATTGTA

AACCTCTTTTGCCGGGAAGCAAGCTGTGCCACGTGTGGCGCAGCTGAGAGTACCCGGAGA

AAAAGCATCGGCTAACTCCATG

>6441 len=142 tot-seq=1

TAGGGAATTTTCGTCAATGGGCGCAAGCCTGAACGAGCAATGCCGCGTGCGGGATGAAGG

CCTTCGGGTTTTAAACCGCTTTTGATTGGGAGCAAGCCTTCGGGTGAGTGTACCTTTCGA

ATAAGCGCCGGCTAACTACGTG

>6442 len=142 tot-seq=1

TAGGGAATTTTTCACAATGGGCGCAACCCTGACGCAGCGACGCCGCGTGCGGGATGAAGG

CCTTCGGGTTGTAAACCGCTTTTGATTGGGAGCAAGCCTTCGGGTGAGTGTACCTTTCGA

ATAAGCGCCGGCTAACTACGTG

>6443 len=142 tot-seq=1

TAGGGAATTTTCGTCAATGGGGGGAACCCTGAACGAGCAATGCCGCGTGAGCGAAGAAGT

TATTCGTAATGTAAAGTTCTTTTATCAGGGAAGAAAGAGGAATTTGACGGTACCTGAGGA

ATAAGCTCCGGCTAACTACGTG

>6444 len=142 tot-seq=1

TGGGGAATATTGGGCAATGGGCGCAAGCCTGACCCAGCAACGCCGCGTGAAGGAAGAAGG

CTTTCGGGTTGTAAACCGCTTTCAGCAGGGACGAGGCCGCAAGGTGACGGTACCTGCAGA

AGAAGCCCCGGCTAACTACGTG

>6445 len=142 tot-seq=1

TGGGGAATCTTCCGCAATGGACGAAAGTCTGACGGAGCAACGCCGCGTGAACGATGACGG

CCTTCGGGTTGTAAACCGCTTTTGATTGGGAGCAAGCCTTCGGGTGAGTGTACCTTTCGA

ATAAGCGCCGGCTAACTACGCG

>6446 len=142 tot-seq=1

TGGGGAATATTGGACAATGGAGGCAACTCTGACGCAGCGACGCCGCGTGCGGGATGAAGG

CCTTCGGGTTGTAAACCGCTTTTGATTGGGAGCAAGCCTTCGGGTGAGTGTACCTTTCGA

ATAAGCGCCGGCTAACTACGTG

>6447 len=142 tot-seq=1

TGGGGAATCTTGCGCAATGGGGGGAACCCTGACGCAGCGACGCCGCGTGCGGGACGGAGG

CCTTCGGGTTGTAAACCGCTTTCAGCAGGGACGAGGCCGCAAGGTGACGGTAACTGCAGA

AGAAGCCCCGGCTAACTACGTG

>6448 len=142 tot-seq=1

TGGGGAATATTGCGCAATGGGGGAAACCCTGACGCAGCGACGCCGCGTGCGGGATGAAGG

CCTTCGGGTTGTAAACCGCTTTCAGCAGGGACGAGGCCGCAAGGTGACGGTACCTTACTA

AGAAGCTCCGGCTAAATACGTG

>6449 len=142 tot-seq=1

TGGGGAATATTGCACAATGGGCGAAAGCCTGATGCAGCGACGCCGCGTGCGGGATGAAGG

CCTTCGGGTTGTAAACCGCTTTTGATTGGGAGCAAGCCTTCGGGTGAGTGTACCTTTCGA

ATAAGGACCGGCTAATTCCGTG

>6450 len=142 tot-seq=1

GGTCCCAGAGGAGTTGCTTGGAAGAGGGTCCTAAGGCCAGTCATCAGAGAAGGAATACCT

GGGGTGGAGATTCCAGGAAGATACACACATCACCTGGTCCACAGCCTCTCCAGCCTTACT

TTCCAACACTGGAGTTATTTGA

>6451 len=142 tot-seq=1

CGAGATATACCTGTGACTGGAGTTCAGACGTGTGCTATTCCGATTCCTACGGAGGCAGCA

GGCACACAATAAATGGCATTTCACAGCCATGTGGGAAAGACCACATGCCAGGTGGGACAC

CTGGGGAGGCTGGGAAACTCGC

>6452 len=142 tot-seq=1

CGATTCATTGCTGCGTTTCTGAATAGGTTGCCACTGGTAAGTGATTTCCATGGGAACATG

GCTGGCGTAACGAGGATGCGGCCACAGGTCGTGGGGGAAGGGAAGGGAAGCAGGCTCTGC

TCACCTCTGCAGCCCCTCTGCC

>6453 len=142 tot-seq=1

CTGAGGATGGCGGAAACTGCCTCTCCAGCAGCACCCGGGAAACTCAGCACTGCCGTGTTC

ACATGGCACCTGTTCATTCCTCTCCAGCAGCACCCAGGAAACTCATCACTGCCGTGTTCA

CATAGCACCTGTTCATTCCTCT

>6454 len=142 tot-seq=1

GGGCGAAAGCCTGACGGAGCAACGCCGCGTGAGTGATGAAGGTCTTAGGATCGTAAAGCT

CTGTTATTAGGGAAGAACAAACGTGTAAGTAACTGTGCACGTCTTGACGGTACCCAATCA

GAAAGCCACGGCTAACTACGTG

>6455 len=142 tot-seq=1

TGGGGAATATTGGGCAATGGGCGAAAGCCTTACCCAGCAATGCCGCGTGCGGGATGAAGG

CCTTCGGGTTGTAAACCGCTTTTGATTGGGAGCAAGCTTTCGGGTGAGTGTACCTTTCGA

ATAAGCGCCGGCTAACTACGTG

>6456 len=142 tot-seq=1

GCAGGGGGGAAGAGGGGACAAGGGAGGAAACGCGGAGCCAGCAAGGCGGGGGGGGGGAAG

AAGGGGGGAGGACGGGAAAACACTTTGAGTAGTGAAGAAAAAAATGACGGTACCTTCAGA

AGAAGCAACGGCTAAAGACGTG

>6457 len=142 tot-seq=1

CCTCCTTTACAGATACAGATGTCAAGACAGATGAACAGGTAAGAGGGAAAAGGCTCCACG

CTGGGGGCTGCTTGTGTCCCCCTGGGTCAGTCCAGCCTGACAAGGGGCCTTATCATTAAA

GCCATGTCAGTCTACACCAGCC

>6458 len=142 tot-seq=1

TAAAGAATATTGCGCAATGGGGGAAACCCTGACGCAGCGACGCCGCGTGCGGGATGAAGG

CCCTCGGGTTGTAAACCGCTTTTGATTGGGAGCAAGCCTTCGGGTGAGTGTACCTTTCGA

ATAAGCGCCGGCTAACTACGTG

>6459 len=142 tot-seq=1

TCGAGAATCTTCGGCAATGGGCGCAAGCCTGACCGAGCGACGCCGCGTGGAGGATGAAGG

CCTTCGGGTTGTAAACTCCTGTCGAGGGGGAAGAAGCCGCAAGGTTGACTGATCCCTGGA

GGAAGCACGGGCTAAGTTCGTG

>6460 len=142 tot-seq=1

TGGGGAATCTTGCGCAATGGGGGAAACCCTGACGCAGCGACGCCGCGTGCGGGATGAAGG

CCTTCGGGTTGTAAACCGCCTTCAGCAGGGACGAGGCCGCAAGGTGACAGTACCTGCAGA

AGAAGCTCCGGCTAAATACGTG

>6461 len=142 tot-seq=1

TGGGGAATATTGCACAATGGACGGAAGTTTGATGCAGCGACGCCGCGTGGAGGGTGTAGG

CCTTCGGGTTGTAAACCGCTTTTGATTGGGAGCAAGCCTTCGGGTGGGTGTACCTTTCGA

ATAAGCGCCGGCTAACTACGTG

>6462 len=142 tot-seq=1

TGGGGAATATTGCACAATGGGGGGAACCCTGATGCAGCGACGCCGCGTGAGTGAAGAAGT

ATTTCGGTATGTAAAGCTCTATCAGCAGGGACGAGGCCGCAAGGTGACGGTACCTGCAGA

AGAAGCCCCGGCTAACTACGTG

>6463 len=142 tot-seq=1

TGGGGAATATTGCACAATGGGCGCAAGCCTGATGCAGCGACGCCGCGTGGGGGATGACGG

CCTTCGGGTTGTAAACTCCTTTCGACAGGGACGAAGCTTTATTGTGACGGTACCTGTAGA

AGAAGTACCGGCTAACCACGTG

>6464 len=142 tot-seq=1

CTCCTCCCACCTGCAAAGCAATCCTGGCACCCAGTCTGGCCAGGGAGTATGGTTGGCAGT

ACTGGGTGTCTAGGTTTCCCAGTCAGCAATCCAGCCCAGCTGCAAAGCACAGATGGCAAC

CATGCCCAGGCAGGGAACCAAG

>6465 len=142 tot-seq=1

TGAGGAATATTGGTCAATGGGCGTAAGCCTGAACCAGCCAAGTCGCGTGCGGGATGAAGG

CCTCCGGGTTGTAAACCGCTTTTGATTGGGAGCAAGCCTTCGGGTGAGTGTACCTTTCGA

ATAAGCGCCGGCTAACTACGTG

>6466 len=142 tot-seq=1

GGGCGCAAGCCTGATGAAGCCAGGCCGCGTGCATGAAGAAGGCCTTCGGGTTGTAAAGTA

CTTTCAGCGGGGAGGAAGGCGACAGGGTTAATAACCCTGTCGATTGACGTTACCCGCAGA

AGAAGCACCGGCTAACTCCGTG

>6467 len=142 tot-seq=1

GGACGCAAGTCTGATGGAGCAACGCCGCGTGAGTGAAGAAGGGTTTCGGCGCGTAAAGCT

CTGTTGTTGGTGAAGAAGGACAGGGGGAGTAACTGACCTTGGGGTGACGGTAATTAATTA

GAAAGTCACGGCTAACTACGTG

>6468 len=142 tot-seq=1

GGACGAAAGTCTGATGGAGCAACGCCGCGTGAGTGAAGAAGGGTTTCGGCTCGTAAAGCT

CTGTTGGTAGTGAAGAAAGAGAGAGGTAGTAGCGGGCCTTTATTTGACGGTAATTAGTGA

GAAAGGCACGGCTAACTACGTG

>6469 len=142 tot-seq=1

GGACGAAAGTCTGATGGAGCAACGCCGCGTGAGTGAAGAAGGGGTTCGGCTCGTAAAGCT

CTGTTGGTAGTGAAGAAAGATAGATTTAGTAGCTGGCCTTTATTTGACGGTAATTACGTA

GAAAGTCACGGCTAACTTCGTG

>6470 len=142 tot-seq=1

GGACGAAAGTCTGATGGAGAAACGCCGCGTGAGTGAAGAAGGGTTTCGGCTCGTAAAGCT

CTGTTGGTAGTGAAGAAAGAGAGAGGTAGTGACTGGCCTTGATTTGACGGTATTTAATTA

GAAAGTCACGGCTAACTACGTG

>6471 len=142 tot-seq=1

TGAGGAATATTGGTCATGAACCAGCCAAGTAGCGTGCAGGATGACGGCCCTATGGGTTGT

AAACTGCTTTTATGTGGGGATAAAGTGCGTGACGTGTCATGCATTGCAGGTACCACATGA

ATAAGGACCGGCTAATTCCGTG

>6472 len=142 tot-seq=1

TGGGGAATATTGCACAATGGGCGCAAGCCTGATGCAGCGACGCCGCGTGAGGGATGGAGG

CCTTCGGGTTGTAAACCTCTTTTGTTTGGGAGCAAGCCTTCGGGTGAGTGTACCATTCGA

ATAAGGACCGGCTAATTCCGTG

>6473 len=143 tot-seq=1

CAGTTGGGCTGCCTGGGGGCAGAGAGGCTGTACCCAAGCAGGGCCAGTGAACATGGAGGG

AGGCTCAGAGGACACCAGCATTATTTTTGGCTGTATTTTCTTTGTATTTTAGGATTACTG

CCTTTATCCATCTTCATTGCTAA

>6474 len=143 tot-seq=1

TGGGGAATTTTGCACAATGGAGGAAACTCTGATGCAGCGACGCCGCGTGCGGGATGAAGG

CCTTCGGGTTGTAAACCGCTTTTGATTGGGAGCAAGCCTTTTGGGTGAGTGTACCTTTCG

AATAAGCGCCGGCTAACTACGTG

>6475 len=143 tot-seq=1

GTCAATGGGCGCAAGCCTGAACCAGCCAAGTAGCGTGAAGGATGACTGCCCTATGGGTTG

TAAACTTCTTTTATACGCGAACAACCGCAGGTACGTGTACCTGTCTGCGTGTAGCGTATG

AATAAGCATCGGCTAACTCCGTG

>6476 len=143 tot-seq=1

CAGGGAATCTTCCGCAATGGGCGAAAGCCTGACGCAGCGACGCCGCGTGCGGGATGAAGG

CCTTCGGGTTGTAAACCGCTTTTGATTGGGAGCAAGCCTTTTGGGTAAGTGTACCTTTCG

AATAAGCGCCGGCTAACTACGTG

>6477 len=143 tot-seq=1

GCCTGCTCTGCCAACACCAGGCTGGAGGCAGGTTTTAGGGAAGGCTGGGCTTTCCCGGCC

TCAGAGCTGCACCCATGCTACCTGTGAGCTTCTGGGGCTTCCTGGAGCCTCGAGATGGCG

TGGAAAGGCCAGGGTCGTCGGTG

>6478 len=143 tot-seq=1

TGGGGAATATTGCACAATGGGCGCAAGCCTGATGCAGCCGTGCCGCGTGTGTGAGGAAGG

CCTTCGGGTTGTAAACCGCTTTTGATTGGGAGCAAGCCTTTTGGGTGAGTGTACCTTTCG

AATAAGCGCCGGCTAACTACGTG

>6479 len=143 tot-seq=1

TGAGGAATATTGCGCAATGGGGGAAACCCTGACGCAGCGACGCCGCGTGAAGGATGAAGG

TCTTCGGATTGTAAACCGCTTTTGATTGGGAGCAAGCCTTTTGGGTGAGTGTACCTTTCG

AATAAGCGCCGGCTAACTACGTG

>6480 len=143 tot-seq=1

TGGACGCAAGTCTGATGGAGCAACGCCGCGTGAGTGAAGAAGGGTTTCGGATGGAAAAGC

TCTGTTGTTGGTGAAGAAGGACAGGGGTAGTAACTGACCTAAAAGAGACGGTAATCAATT

AGAAAGTCACGGCTAACTACATG

>6481 len=143 tot-seq=1

TGGGGAATCTTGCACAATGGGGGGAACCCTGACGCAGCGACGCCGCGTGCGGGATGAAGG

CCTCCGGGTTGTAAACCGCCTTTGATTGGGAGCAAGCCTTTTTGGTGAGTGTACCTTTCG

AATAAGCGCCGGCTAACTACGTG

>6482 len=143 tot-seq=1

TGGGGAATATTGCACAATGGGGGAAACCCTGATGCAGCGACGCCGCGTGGGGGATGAAGG

CCTTCGGGTTGTAAACCCCTTTCAGTAGGGAACAAGGCCATGATTGTTGTTGTGGTTGAG

GGTACTTGCAGAAGAAGCGCCGG

>6483 len=143 tot-seq=1

TGGGGGAAACCCTGACGCAGCGACGCCGCGTGCGGGATGAAGGCCTTCGGGTTGTAAAGT
ACTTTCAGCGGGGAGGAAGGGAGTAAAGTTAATACCTTTGCTCATTGACGTTACCCGCAG
AAGAAGCACCGGCCAACTCCGTG
>6484 len=143 tot-seq=1
GGACGGAAGTCTGACCGAGCAACGCCGCGTGAGTGAAGAAGGTGTTCGGATCGTAAAGGT
AGGTTGTTAGAGAAGAACGTTGGTAGGAGTGGAAAATCTACCAAGTGACGGTAACTAACC
AGAAAGGGACGGCTAACTACGTG
>6485 len=143 tot-seq=1
TGGGGAATTTTGGACAATGGGGGAAACCCTGATCCAGCCATGCCGCGTGCGGGATGAAGG
CCTTCGGGTTGTAAACCGCTTTTGATTGGGGGCAAGCCCTTTGGGTGAGTGTACCTTTCG
AATAAGCGCCGGCTAACTACGTG
>6486 len=143 tot-seq=1
GGGGTTGCTCTCTAGGCAGGCACAGCTGTACTGGCTGGGGCACCCCAGGGTTCCAGCAGC
GGGAGGGAAGGGTGCAAGCTGAGGGCTGGGCGGGCCTGCCGAGAGCCTGTGGTGGTTAAC
CCCTTCCGGAACGCAGTCCACAG
>6487 len=143 tot-seq=1
TAGGGAATTTTTCACAATGGGCGCAAGCCTTATGAAGCGACGCCGCGTGCGGGATGAAGG
CCTTCGGGTTGTAAACCGCTTTTGATTGGGAGCAAGCCTTTTGGGTGAGTGTACCTTTCG
AATAAGCGCCGGCTAACTACGTG
>6488 len=143 tot-seq=1
ATCAGAGTGAATAGTAGATGTGATAACAGAAGCAAGAGGTTAGAATAATGTGGCCATGAG
CTAAAGAACGCAGCTAAGGAATGTGGCTTCTAGAGGTTGGAAGAGGTAAGAAATGGATTA
TTTCGTAGAGCCTCCAGAATGAA
>6489 len=143 tot-seq=1
TGGGGAATTTTGGACAATGGGCGCAAGCCTGACGCAGCGACGCCGCGTGCGGGATGAAGG
CCTTCGGGTTGTAAACCGCTCTTGATTGGGAGCAAGCCTTTTGGGTGAGTGTACCTTTCG
AATAAGCGCCGGCTAACTACGTG
>6490 len=143 tot-seq=1
GGACGGAAGGCGGACCGAGCAACGCCGCGGGAGGGAAGAAGGTGTTCGGATCGTAAAGCT

CTGTTGTAAGAGAAGAACGAGTGTGAGAGTGGAAAGTTCACACTGTGACGGTATCTTACC

AGAAGGGGACGGCTAACTACGTG

>6491 len=143 tot-seq=1

TAGGGAATCTTCCACAATGGGCGCAAGCCTGATGCAGCGACGCCGCGTGCGGGATGACGG

CCTTCGGGTTGCAAACCGCTTTTGACTGGGAGCAAGCCTTTTGGGTGAGTGTACCTTTCG

AATAAGCGCCGGCTAACTACGTG

>6492 len=143 tot-seq=1

TGAGGAATATTGGTCAATGGACGCAAGTCTGAACCAGCCAAGTAGCGTGCAGGATGACGG

CCCTATGGGTTGTAAACCGCTTTTGATCGGGAGCAAGCCTTCGGGTGAACGTACCTTTCG

AATAAGCACCGGCTAACTACGTG

>6493 len=143 tot-seq=1

GGGGGCAACCCTGAACGAGCAACGCCGCGGGAGTGAAGAAGGTTTTCGGATCGTAAAGCT

CTGTTGTAAGCCAAGAACGAGTGTGAGAGTGGAAAGTTCACTCTGTGACGGTAGCTTACC

AGAAAGGGACGGCTAACTACGTG

>6494 len=143 tot-seq=1

GGAAGAAAGACTGACGGAGCAACGCCGCGTGAGTGAAGACGGTCTTCGGATTGTAAAGCG

CTGTTATACGGGACGAATGGAAAGGTGGTAAATAGCCATCATGAGTGACGGTACCGTAAG

AGAAAGCCACGGCTAACTACGTG

>6495 len=143 tot-seq=1

TTTTAAAAAATATGGGCATCTTCTTTTGCGCTGGCATGAGTCAGCACTCTTCCCCACCCC

TCCGGCCCTGTGCCTGATGACTTGGGCTTGGGGGATGTAGTTGGTGGAAAAGCAACACTC

TTTTTCTGGATTTCCAAAGGGTC

>6496 len=143 tot-seq=1

GGAAGAAAGTATGAAGGAGAAAAGAAGAGTGAGTGAAGAAGGTCTTAGGATTGTAAAGCT

ATGTTATACGGGACGAACGGCAAGGTGGTAAATAGCCATCATGAGTGACGGTACCGTAAG

AGAAAGCCACGGCTAACTACGTG

>6497 len=143 tot-seq=1

CTGTAGAGGCAGGGAGGAGCTGGGGAGACCAGAGGGAGGCACTGCTGGTGGCTTCTTCTC

TTCTCTGCCCCGGCTCACCTTTACTTTATTATCAAACACCTCCTGCCACACCACATAGCC

CTTGCCATAAGAAGAGACGATGT

>6498 len=143 tot-seq=1

GGTGGACTTCCTGGAGGCATGAGAGAACTTTCACCTAAGAAAATGACTGAGGCCTACTTA

CTGGAGCCTGTCCTAATAAAGGAAGCTCGGCCTCGCCTCTGGGCAGGCTCGCAGGGGGCA

TTACCAGGCCTGTCCTCTCTCCC

>6499 len=143 tot-seq=1

CCTGGAAGCTGGTGTTTGACCCCAGAGCCCACAGTCAGACCTCTGCCCCAGGGCTCCTTT

CTCTGTGCGGCGGTGGGATCTTGGTCTGGCAAGGGTGGGGGCTGACCCAGAGAGCCTGCT

GTGAGGGAGCCCCCAGATAGTAA

>6500 len=143 tot-seq=1

GTCAATGGCCGGGAGGCTGTACCAGCCAAGTCGCGTGAGGGAAGACGGTCCTATGGATAG

TAAACCTCTTTGGTCAGGGAGAAAAATCTGCCACGTGTGGCAGGATGAGAGTACCTGAAG

AAAAAGCATCGGCTAACTCCGTG

>6501 len=143 tot-seq=1

TGAGGAATATTGGTCAATGGTCGGGAGACTGAACCAGCCAAGCCGCGTGCGGGATGAAGG

CCTTCGGGTTGTAAACCGCTTTTGATTGGGAAGCAAGCCTTCGGGTGAGTGTACCTTTCG

AATAAGCGCCGGCTAACTACGTG

>6502 len=144 tot-seq=1

CTGAGTCCTGTGTTCCCCCTTACACCCTGGTGACTGGAGGCTTCCCCCAAGGTCAGAACT

GAGGCTGGCCCTCCCCTCCTCTGCAGAGTTGCACATTCCCAGCCACCCCCAGCTGTAGGA

AAGTTCCCAGCCTGGCCTGGGCTC

>6503 len=144 tot-seq=1

CACAGCTGTCAGGACTCCAGAGCAGTATTACCAGACTTTCAGCCCGAGCTCCAGCCACTC

CCCTGCACGGTCTGTGGGTCGCTCCCCTTCCTATAGCTCCACCCCGTCACCACTGATGCC

CAATCTGGAGAACTTCCCCTATAG

>6504 len=144 tot-seq=1

GAAACACAGAGAGAAATAGACTGAACCTGGCCGTGAGGGGAGGAGAAGTGGAGCAGAGAG

ATAGAGAGGAGTGGGGAGGGAGGCTGGGAGGCATATCAAAAATAGTCCGGTTATAAGGAG

AATGAGTAAGTAGGGGAGGGAAGC

>6505 len=144 tot-seq=1

TGGGGAATCTTGCACAATGGGCGAAAGCCTGATGCAGCGACGCCGCGTGAGGGATGAAGG

CCTTCGGGTTGTAAACCTCTTTCAGTAGGGAAGCAGCCCTTCGGGGTGACGGTACCTGCA

GAAGAAGCGCCGGCTAACTACGTG

>6506 len=144 tot-seq=1

TGAGGAATATTGGTCAATGGACGAGAGTCTGAACCAGCGACGCCGCGTGCGGGATGACGG

CCTTCGGGTTGTAAACCGCTTTTGACTGGGAGCAAGCCCTTCGGGGTGAGTGTACCTTTC

GAATAAGCACCGGCTAACTACGTG

>6507 len=144 tot-seq=1

GAAAATTTGAATTTTGAAAACTTACAATACAAGGAGACACTCCATCAATGATTGACAGAA

ACTGGTGGATAAATACCCCAGCTCCCTCTCTCCTCAGACAGTATAACTCTGAGGCATGTG

GTCTACCTTGTTTCCCAGAGGTTC

>6508 len=144 tot-seq=1

GCAGCAGTGGGGAATATTGCACAATGGGGGAAACCCTGATGCAGCGACGCCGCGTGAACG

AAGAAGGTATTCGTATCGTAAAGTTCTATCAGCAGGGAAGAAGAAATGACGGTACCTGAC

TAAGAAGCACCGGCTAAATACGTG

>6509 len=144 tot-seq=1

TGACGAATATTGGCCCACCGACGCAAGTCTGAACCAGCCAAGTAGCGTGCAGGATGACGG

CCCTATGGGTTGTAAACTGCTTTTATATGGGGATAAAGTGGGGAACGTGTTCCCTTTTGC

ATGTACCATATGAATAAGGATCGG

>6510 len=144 tot-seq=1

TGGGGAATATTGCACAATGGGCGGAAGCCTGATGCAGCGACGCCGCGTGAGGGATGACGG

CCTTCGGGTTGTAAACCTCTTTCAGTGAGGAACAAGGCCTTCGGGTTGAGGGTACTCGCA

GAAGAAGCGCCGGCTAACTACGTG

>6511 len=144 tot-seq=1

ATGGACGCAAGTCTGATGGAGCAACGTCGCGTGAGTGAAGAAGGGTTTCGGCTCGTAAAG

CTCTGCTGTTGGTGAAGAAGTGCATTTTTAGTAACTGACCTTTGTTTGACGGTAATCAAT

TAGAAAGTCACGGCTAACTACGTG

>6512 len=144 tot-seq=1

ATCATGAGGTCAAGAGATCGAGACCATCCTGGCCAACATGGTGAAACCCCGTCTCTACTA
AAAATACAAAAATTAGCCGGGCGTGGTGGCATGCATCTGTAGTCCCAGCTACTCAGGAGG
CTGAGGCAGAAGAATCGCTTGAAC
>6513 len=144 tot-seq=1
ACCGGTGAAGACCAGCAAACCATCCTTGACATTGCCGAACATTGCAAACAGCAATACGAA
GACCAGAAGCAACGACACAGGGACGACGATTTGCAATCGCTGTGTCGCGGACTGCAGGTT
TTCAAATGTGCCACCCCAGCTCGT
>6514 len=144 tot-seq=1
TTGTGCCTGTAGACCCAGCCAAGAGGAAGAGGTGGGCCTGGAGAAGCCCCCATGAGGCAG
AGGTTGGGCCTGTAGACGCTGACAGGAGGCAGGAGCTGGGCCTGGACAGGTCAACTTGAG
GAGATTTTGGGCCTTCATAGGCCA
>6515 len=144 tot-seq=1
TTTAGCAGTTAAATGCAACATCCAGGAATAAGGTTATTTCTGTCTTCTCACATTGACAAT
TCTCATTATGTTGACTTTCATTCTCAGCCTAGGTGCCTCTTGATTCCAGAGTAATTTACA
CAGCTCCACTTAGCATATCCTTAC
>6516 len=144 tot-seq=1
TCAGGGCAGCTTCACTGAGGAGGTGACCTTGGAGCAAGCCTTACAGGGGAGCATGTGAGC
AGGCAGGTGTGTGGGGATGTGCATTCCAGGAGAAGGAACAGCAAAGAGCAAAGTCCCCAG
AGCAGCAGCAAGCCCAGCCTCCAC
>6517 len=144 tot-seq=1
GAAGTGCAGGGCCGAGGGGTGCTGTGCAGGAAGGAGGAGGGACATGTGGGGTGCTTCGCT
GGTAACCCCCCTTCTGGAGTGACCTCCTGGTGTGGCCGCTTTGGATCCTGCTACTTTCAT
TTTAAAAGTTGGCAGCATGTTGGG
>6518 len=144 tot-seq=1
AGGGCTTTCTGTAGGTCTGGGGGAGTAATGTGACCTCGTGGGGTGTTGGGAGGATCTACC
AGGCAAAGCGCCTCCAGAATGCAGCAGAGGGCTTGCTGTAGGTCTGGGGGAGTAATGTGA
TCTAACTCATGGGGTACTGGGAGG
>6519 len=144 tot-seq=1
TGGGGAATATTGCGCAATGGGGGAAACCCTGACGCAGCGACGCCGCGTGCGGGATGACGG

CCTTCGGGTTGTAAACCGCTTTTGACTGGGAGCAAGCCCTTCGGGGTGAGTGTACCTTTC

GAATAAGCACCGGCTAACTACGTG

>6520 len=144 tot-seq=1

GGAGTGTGTCTGGGGTGAGGTGCCAGCTGAGAGCAGGACCCCAGGACCCCAGGCCTAAGC

CCTGCTCTTGCTGGAGGCTGGGGCGGATTTCACCGCTGGAGGGAAACCGTGCCTCTGTGA

CGTGTGAGGGATGTTTTCTAGTCT

>6521 len=144 tot-seq=1

GCAGGCTCTTCCCGAAGCTGCTTCTGTTCACACATGTGCTTCCAAACTCTCCCACTTCTC

AGGAAACTCCCCTAATTGTGCCTGTGGGTCCAGGACACTGTCCCATGAGGGGGCCATGGC

AGGGTCTCCCCGGACGGTATGACT

>6522 len=144 tot-seq=1

GTCACATACCTGGAATCACCTGCTTTTGGCCAAGTTCTATAACCAGAGGGTCTCTGGTCA

GGGAGGTGTCAATAATACGTCCATCTACCAAGCTTCCCTGGGGGGAGAGAGCATCAAGAG

CATACATCTAGCACCCTCTCCCTC

>6523 len=144 tot-seq=1

CACACGTGTCTTCTCCCCAGGTACGCTGGCAGCTCCACATAGAAAACCTCACACCTCCCA

TAAGGACAGCGGGCCTCCCCTCCTGGGACCCCTCCCTTGGCTTCTCCCCAGCCCTGCTCC

TAGAAGGGAGGCTTGGAGCAGGAA

>6524 len=144 tot-seq=1

ATGGGGGCAACCCTGACCGAGCAACGCCGCGTGAGTGAAGAAGGTCTTCGGATCGTAAAG

CACTGTTGTAAGTGAAGAATGCCATATAGAGGAAATGCTATGTGGGTGACGGTAGCTTAC

CAGAAAGCCACGGCTAACTACGTG

>6525 len=144 tot-seq=1

GGATGAGTTCAAGGGCAGGACCCATAGCTGGGAATGCCACAGAAGACACAGTTCAGAGGA

AGAGTGGCTGCACAAGGAAAGAAGGCAGACTCCCGAGTTCTCTTGAAAACCTTTTCATAG

TAGACACTGAGTGGGAAGGGCCAG

>6526 len=144 tot-seq=1

AGGGAACGTCGCCACTAAGGCCTTGCACAGAAAGAGTTTTGGGGACCAACAGGGGGCCAG

TGTGGCTGAATATAGCCAGTAGGAGGAAGAAGGTTCTGGAAGGGGCCAGGCCATGTAAGC

ACGGCAGATTGTTCTCATTTAATT

>6527 len=144 tot-seq=1

CCTACCATGACCTCGGAATGGAGGGACCTCGTGCCATTCACCACATAACAAGTGGGAAAA

TTCTTACGGCTGATGCACGACTAAAGAAATGGTTTTCGCCCGACCAAATCACCATTATGA

AGGAAGCTGTGGAAGATCATAGAG

>6528 len=144 tot-seq=1

TAGGGAATCTTCCACAATGGACGCAAGTCTGATGCAGCGACGCCGCGTGCGGGATGACGG

CCTTCGGGTTGTAAACCGCTTTTGACTGGGAGCAAGCCCTTCGGGGTGAGTGTACCTTTC

GAATAAGCACCGGCTAACTACGTG

>6529 len=144 tot-seq=1

TTGGGGCGACCAGGTGAGCTGGCCCTTGTGGGAGGATGTAACTGACTCCTGAGCCTGGCG

AGCCAGGCAGCCCCTCGCCAACATCCCCACCCCTACCTCTCCAGCCCCCCCGCATTCCCT

GATCCTCCCATCCGCTCCCCTGAC

>6530 len=144 tot-seq=1

TGAGCCATGATCTCACCACTGCGCTCCACCCTGGCAACAGAGCGAGACCCTGACTCAAAT

AATAATAAAACTTAGTCTTTGAAATATCATGCACATTTTACACCTGCAATCCATCACAAT

TTAGACCAGCCACATTTCAAATGC

>6531 len=144 tot-seq=1

TAGGGAATCTTCGGCAATGGGGGCAAGCCTGATGCAGCGACGCCGCGTGCGGGATGACGG

CCTTCGGGTTGTAAACCGCTTTTGACTGGGAGCAAGCCCTTCGGGGTGAGTGTACCTTTC

GAATAAGCACCGGCTAACTACGTG

>6532 len=144 tot-seq=1

TGGGGAATATTGCACAATGGGCGGAAGCCTGATGCAGCGACGCCGCGTGGGGGATGACGG

CCTTCGGGTTGTAAACTCCTTTCGCCATTGACGAAGCCCTTTTGGGTGACGGTAGGTGGA

GAAGAAGCACCGGCTAACTACGTG

>6533 len=145 tot-seq=1

CCCCCACCTCGGGCCCCCACCCTGTCCCCGTCCTCCTCCTGGTTGCCCTGAGTTTAGAAG

AGCAGCCGCTGCCGCCACCACCACTCCGGAGGGCACCAGGGCTGCTGTCCAGGGAGGGAC

AGTAGCAGTGAGGCTCTGGCCAGTC

>6534 len=145 tot-seq=1

GAAAGGAGGACAAGGCAGCAGATGAAGGAAAAAAAAATGACACGAGGACAGGACCACTCC

TAGATATGGGTGGAGGAGGGGGTTTATTTTAGATAAGAGGGAAAACACAGTCAGAGGCAT

TTGGAAGGGTCCAGTGTGAGCATGG

>6535 len=145 tot-seq=1

AGAGTGAGGTAATGTCCAAACAATGACTGGCCCCACTTGAGATTCATCCCATGGGCAAGA

ACCAATCTCTGACTCTATTAGTGATAGATACTCTGTTATACTTGCAGACAGAACCCTAGC

ATAACTGTCTTCTTATAGTCTCCAC

>6536 len=145 tot-seq=1

GCAGGGTATGCTGTAGACAAAGCTCATTCCTGGGGGAGGCGAGGGGTGGAGGGAGGGGGG

CAGCATCTTGAGTTGCTCACTCCTCAAGGTTCTAGGACCTGCTTTCTTGCTATGACCATG

GAGGGCAGAGAGCGCGCACTGATGC

>6537 len=145 tot-seq=1

GAGGAGGAGGAGGAAGAAGAGGAGGAGGAGGAGCAGCAGCAGCAGCAGCAGCAGCAGCAG

CAGCATCACGTGTGCACGAGTTTAAGGCCAGCCTAAGCAACACATGGAGAGCCTCACAAA

TGAAAGCTATGGTCACTATGTCCTG

>6538 len=145 tot-seq=1

GGGTAGTCAATGTTTCAGTGCCTTCCACTTAGCGAAGTGGGGGGTTTGATGGGCATCTGT

CTGAACTGGCCTCCTAGTCCTGGATTTCTGGAAGCCACTGTCCTTTTACTTTCCTCTCCT

TGATGGCAGAAGGCAGAAAGTGGGC

>6539 len=145 tot-seq=1

TGAGGAATATTGGTCAATGGGCGAGAGCCTGAACCAGCCAAGTAGCGTGCAGGAAGACGG

CCCTATGGGTTGTAAACTGCTTTTATGCGGGGATAAAGTGCGCGACGTGACGGTACCTGC

AGAAGAAGCCCCGGCTAACTACGTG

>6540 len=145 tot-seq=1

TGGGGAATATTGCACAATGGGCGCAAGCCTGATGCAGCGACGCCGCGTGCGGGATGACGG

CCTTCGGGTTGTAAACCGCTTTTGATCGGGAGCAAGGCACTTTGTGTTGAGTGTACCTTT

CGAATAAGCACCGGCTAACTACGTG

>6541 len=145 tot-seq=1

TGGGGAATCTTCCGCAATGGACGAAAGTCTGACGGAGCAACGCCGCGTGAGTGATGACGG

CCTTCGGGTTGTAAACCTCTTTTGTTAGGGAGCAAGGCACTTTGTGTTGAGTGTACCTTT

CGAATAAGCACCGGCTAACTACGTG

>6542 len=145 tot-seq=1

TAGGGAATATTGCGCAATGGACGGAAGTCTGACGCAGCGACGCCGCGTGAGCGATGAAGG

CCTTCGGGTCGTAAAGCTCTGTCCTTGGGGAAGAAGGTCTTCGGACTTGACGGTACCCAA

GAAGAAAGCCCCGGCTAACTACGTG

>6543 len=145 tot-seq=1

GCATGCAAATTACCCACTCCCGACCCGAAGAGGTAGTGACGAAAGTTTTCGGATCGTAAA

GCTCTGTTGTTGGTGAAGAAGGATAGAGGTAGTAACTGGCCTTTATTTGACGGTAATCAA

CCAGAAAGTCACGGCTAACTACGTG

>6544 len=145 tot-seq=1

AATGGACGAAAGTCTGGCGGAGCAACGCCGCGTGAGTGAAGAAGGATTTCGGTTCGTAAA

ACTCTGTTGTTAAGGAAAAAAAAAAGAATAGTAACTAAAAACAAAAGAGACGGTACCTAA

CCAGAAAGCCACGGCTAACTACGTG

>6545 len=145 tot-seq=1

TGGGGAATATTGCGCAATGGGGGAAACCCTGACCCGGGGAGGTAGTGACGAAAAATAACA

ATACAGGACTCTTTCGAGGCCCTGTAATTGGAATGAGTCCACTTTAAATCCTTTAACGAG

GATCCATTGGAGGGCAAGTCTGGTG

>6546 len=145 tot-seq=1

TGGGGAATATTGCACAATGGGGGAAACCCTGATGCAGCAACGCCGCGTGAGGGATGGAGG

CCTTCGGGTTGTAAACCTCTTTTGTTAGGGAGCAAGGCATTTTGTGTTGAGTGTACCTTT

CGAATAAGCACCGGCTAACTACGTG

>6547 len=145 tot-seq=1

CAATGGGCGAGTGCCTGACGCAGCGACGCCGCGTGTGGGATGAAGGTCTTCGGATTGTAA

ACCACTGTCGGGAGGGACGAAGGCGCGGGGTTAACTGCCGCCGCGATTGACGGTACCTCC

AAAGGAAGCACCGGCTAACTCTGTG

>6548 len=145 tot-seq=1

TGAGGAATATTGGTCAATGGGCGAGAGCCTGAACCAGCCAAGTCGCGTGAGGGATGAATG

GCCTATGGCCTGTAAACCTCTTTTGTCAAGGAAGAATAAGGATGACGAGTCATTCGATGC

CAGTACTTGACGAATAAGCATCGGC

>6549 len=145 tot-seq=1

CAAAACTAGCAGACCACCAGCTCCATGACCACCATCCGGCTCAGGCAGATCTTTACAGTG

CAGCCTCAGAAGAGCCAAACAGGGAAGCTGCAGGTTGGGTAAGTTCAGCAGTTCGCACTG

TCTAGATCCAGCCACAGGAGCAAAT

>6550 len=145 tot-seq=1

TGGTCAATGGACGAGAGTCTGAACCAGCCAAGTAGCGTGCAGGACGACGGCCCTATGGGT

TGTAAACTGCCTTTATGTGGGGATAAAGTGCGTGACGGGTCATGCATTGCAGGTATCACA

TGAATAAGGACCGGCTAATTCCGTG

>6551 len=145 tot-seq=1

CAATGGGGGCAACCCTGATCCAGCAATGCCGCGTGTGTGAAGAAGGCCTGCGGGTTGTAA

AGCACTTTCAGAGGGGAAGAAAAAATCGGATCTAACACGTCCGATCTTGACGTTACCCTT

AGAAGAAGCACCGGTTAACTCCGTG

>6552 len=146 tot-seq=1

TGTGGATGAATAGTTGGATCACGGAAGAAAACAGGTACGAAGGAACAAAAAGTGCCAAAA

ATGGAGCCTTTGTAGAAGGATGCACAAGAGTAGCCATGACAGGAATCCTAGCTGCTCTTT

TCTTGGTCCCTTGCAACCTAACATCT

>6553 len=146 tot-seq=1

TAGGGAATAGACCGCAATGGGAGAAAGCCTGACGGAGAAACGCCGCGAGAGTGATGAAGG

TCTTCGGATAGTAAAACTCTGCTATTAGGGAAGAACAAATGTGTAAGTAACTATGCACGT

CTTGACGGTACCTAATCAGAAAGCCA

>6554 len=146 tot-seq=1

AGGGATCAGCTGGATGAATGGAAGATGTCAGCAGCAGATGTACTGGTTAGTTCAACGGGG

ACAAAATTCCATGAACACGCCACACGGCCTCTAACTTTCAAGAAGGGCACAGTGATAGGG

TGAATTTGCAGGAACCAGGGCAGCAG

>6555 len=146 tot-seq=1

AAATAGGATTCAATAAAGTTTAATTTTTATACCAGCATAATTAAATTAACAAACTGAACA

AAATTTGTCACACACACAGAAAAGTCTCATGTGTGTGTAACAGAAAATGTCTCAAGGCCA

GTGCTGCAGCGAGCAGGCTGCCCCAT

>6556 len=146 tot-seq=1

TGGGGAATAGAGGACAATGGGGGAAAGCCTGATCCAGACATGCCGCGAGTCTGAAGAAGG

CCTTCGGGTGGTAAAGGACTTTTGTCAGGGAAGAAAAGGACAGGGTTAATACACCTGTCT

GATGACGGTACCTGAAGAATAAGCAC

>6557 len=146 tot-seq=1

TAAGAAAGAAAAGGTCGGCTGGAGTTAAGAGTAAAAATAAATTCAAATTGGGACTTTCAT

GAGGCAAATCGACTTTCAACTGCACAAATAACTCCTTTGTTAGGTTTTCCGAGTAGCCGC

TAATGTCACCAAGAGCTGTTTACAGC

>6558 len=146 tot-seq=1

CGGCATACGAGATAAGGCCGTGACTGGAGTTCAGACGTGTGCTTTCCGATCTCCTACGGG

AGGCAGCGGGGGAGTGGGGGGTGTGTCTCCTGGGTCAGCTTTCATGTGAAGAGAGATCAC

CTCTGTCCTCACTTAGGCCAGACCAG

>6559 len=146 tot-seq=1

GAAAGAAGATTGAAACATACTTCTTCCTAAAGGGCCACACACACTCCAACAAGACCACAC

CTCCTAATAGTGGCATTCCCTATGGACCTACGGGGCCATTTCCATTCAAACCACCACACT

TGGTTTGGTGCCATCTGAGCACTTTG

>6560 len=146 tot-seq=1

TAGGGAATTTTCGTCAATGGGGGGAACCCTGAACGAGCAATGCCGCGTGTGTAAAGAAGG

TCTTCGGATCGTAAAGCACTGTTGTAAGTCAAGAACGGGTGTGAGAGTGGAAAGTTCACA

CTGTGACGGTAGCTTACCAGAAAGGG

>6561 len=146 tot-seq=1

GTTAAGAGACATAGATGCACACTGGTGCCTCATTCATTCTCACTTCAGACACTGTGCGGA

ACATCTAAGCATAGCTTGAGTGGGGGCAGCTGCACCGCTTCAGCCACAAGCACGCAGCAC

AGGCAAGTTCATTCATACAAAAGACT

>6562 len=146 tot-seq=1

CGTGAGACGTGGGTGACCCAGAGAGAAGCGGGCTGGGGAGAGGCCCTTATGGACCACGTG

GAGGGAGGGATCAGTGTGTCCCGTGGGCTGGCAGCCTTGCCAGGTCCTGAAGATGCCCAC

AATAGTGCATGCTCAGCTGGGAGGCC

>6563 len=146 tot-seq=1

GGGGCAGTGGGCTCAGTGGGGCAGGCAGCCACGATTCCCTTGATCCTTGAAGGGCTGGCG

CACGCCAGCATCTCTTATCCCCCCTCCCTGGGGCCAGGAGATGTGTAGGTTCAAGAACCA

GACGTTGAATCTTCTTGTAGTCCTTC

>6564 len=146 tot-seq=1

CAATGGGCGCAAGCCTGATGGAGCAACACCGCGTGAGTGAAGAAGGGTTTCGGCTCGTAA

AACTCTGTTGTTGGAGAAGAAGGACAGGGGTAGTAACTGACCTTTATTTGACGGTAATCA

ACCAGAAAGTCACGGCTAACTACGTG

>6565 len=146 tot-seq=1

CAATGGGCGCAAGCCTGATGGAGCAACACCGCGTGAGTGAAGAAGGGTTTCGGCTCGTAA

AGCTCTGTTGTTGGAGAAGAACGTATCTGAGAGTAACTGTTCACGTTTCGACGGTATCTA

ACCAGAAAGCCACGGCTAACTACGTG

>6566 len=146 tot-seq=1

GAGCCTTGGGCAGCAGCAGAACAGACCATGTGTGTTCAGGGAGTGGCACCTGGTGTGCCT

GGAGTCAGGCTGGAAAGTGTGAAGTGACTAGTGAGGAAGCTGCACACATGGGCTGGCACC

GATTGCAAAGGGGTTTCATACCAAAC

>6567 len=146 tot-seq=1

CAGACCCCCTCCCCTGCATAGAGGGCCTGGGAGATATCCAAGCAGCTACAGCCTATGGGG

CATGGTGGAGACTCACACCTCAGTGGCAATGGTGGAGAAAAAGGTTTCTGTCATGCACTG

CCGGACCACAGGCTCCGTGTCCTGCA

>6568 len=146 tot-seq=1

CAAGGGACGCAAGGCGGAGGGAGCAACGCCGCGGGAGGGAAGAAGGTTTTCGGATCGGAA

AGCTCTGTTGTTGGTGAAGAAGGATAGAGGTAGTAACTGGCCTTTATTTGACGGTAATCA

ACCAGAAAGTCACGGCTAACCACGTG

>6569 len=146 tot-seq=1

CAATGGGCGAAAGCCTGATCCAGCCAGGCCGCGTGTGTGAAGAAGGGCTTCGGAGTGTAA

AGCACGGTAAGTTGGGAGGAAGGGTGGTAGATTAATACTCTGCAATTTTGACGTTACCGA

CAGAATAAGCACCGGCTAACTCTGTG

>6570 len=146 tot-seq=1

TTTCAAGCAGAAGACGGCATACGAGATAACACTGTGACTGGAGGTTCAGACGTGTGCTTT

TCCGATCTCCTACGGGGGCAGCAGACACCAGGCCTACATGGACCACCTGAAGATATTTTC

GAACATGAAATCAACAGTCCCTCAAC

>6571 len=146 tot-seq=1

CCCAGGAGGCAAAGGGTGCAGTGAACTGAGATCACGCCATGGCACTCCAGCCCAGGTGAC

AGTGCAAGACTCAATCTCAAAAAAAAAAAAAAATGAAAAAATAAATTAATTAAAAATAAA

TACCCCTCTATCACAAATGGACAGAT

>6572 len=146 tot-seq=1

AATGGACGAAAGTCTGACGGAGCAACGCCGCGTGAGTGAAGACGGTCTTCGGATTGTAAA

GCTCTGTAATACGAGAAAAACGGCAAGGTGATAAATAGCCATCATAAATAACAAAGAAAA

AAGAGAAAGCCACGGCTAACTACGTG

>6573 len=146 tot-seq=1

CAATGGACGCAAGTCTGATGGAGCAACGCCGCGTGAGTGAAGAAGGTTTTCGGATCGTAA

AGCTCGGTTGTTGGTGAAGAAGGATAGAGTTAGTAACGGGCCTTTATATGACGGTAATCA

ACCAGAAAGTCACGGCTAACTACGTG

>6574 len=146 tot-seq=1

CAGAGAGGGATGGGTGCATTTGAGCTCAATCTCTAGTATCAAAGCTCAGCCGAACCTGGA

GAAGAAGCCACCCAGGGACGAATTCCACTGCTGAGATCATCCAGGACTTAGCTCTCTGCC

CACTCCCTTTAGAGTTAGACCCTGAC

>6575 len=146 tot-seq=1

CCGCAGCCACAGGGGCAGCCGGCGCATATGGGCGCCGAAGTCCTTCCAGGAGCCTGCTAC

CGTCCGTTCCAGCAGCCATACCACCAGAAACGCCGGTATCAGCGCCAGCAAAATGGCAAA

TATTCCGTCCTGTCCCAATATATCCG

>6576 len=146 tot-seq=1

CAATGGACGAAAGTCTGATGGAGCAACGCCGCGTGAGTGAAGAAGGTTTTCGGCTCGTAA

AGCTCTGTTGTTAGTGAAGAAAGATAGAGGGAGTAACTGGTCTTTATTTGACGGTATTTA

CTTAGAAAGTCACGGCTAACTACGTG

>6577 len=146 tot-seq=1

GAAGAAGTCCCCCACCAGCGCCACCAACAGCACGATCCCGGCGCGCGATACAGAGACCGG

CATTCCGGTCAGCAACAGGTAAAACAGTGCCGCCGCAGCGCGCAGCAAAATCAGCGGCTT

ATAAAATTTCCATTTGCGGCCAAAAC

>6578 len=147 tot-seq=1

AAAACAGTAGTGAAGCAGACCCACAGCCCATTTTATGGGAACGGTGACTCCCTGTCCCTG

CTCCGGCTGCCTGAACATGAGGGCATTCCTGTTGAAGTGACCGTCAACCTTCCATGTTTG

CTTCTGCCTAAAGCCACTCCAGTCACA

>6579 len=147 tot-seq=1

GGAGGCAGCAGTGGGGGAGATTGCACAAGGGGGGGAACCCGGAGGAAGCGACGCAGAGGG

GGGGAAGGAGCGGGGCGGCGCGGAAAGACCTAGCGGAAGGGAAGAAGGAGGAAGGGACCT

GACTAAGAAGACCCGGCTAACTACGTG

>6580 len=147 tot-seq=1

GAGAGGGTGCACCACAGAACCGGACAGCTTCTGGGTCAGGCGGAAGCACAGAGCCGCTGA

GGCAGCACCCTTTGCAGGCTGCAGACAGCCAGCCACCGTCCGGACCAGAGGACAGGTGTC

CACCTGGCTTGGGAGGCGGCCTCAGCC

>6581 len=147 tot-seq=1

CATGGGTGGAGAACACCAGCAAGGAGGGCCCTCAGAGGGACTGCCTGTGCCATTGAAAGA

GGCAATCAGGAAGCCATTTTCTATCCAGTCACTTCTGTTCTTACCATTTAAGTTATCATC

TTTTAATGTCTAAAACCACCTAAAATA

>6582 len=147 tot-seq=1

TTTTTTTTTTCAAGCAGAAGACGGCATACGAGATGCCTAAGTGACTGGAGTTCAGACGTG

TGCTCTGTTGTTAAGGAAGAACGAGTGTGAGAATGGAAAGTTCATGCTGTGACGGTACTT

AACCAGAAAGGGACGGCTAACTACGTG

>6583 len=147 tot-seq=1

AGGCAGCAGTGGGGAATAGTGGACAAGGGAGGGAACTCTGATCCAGCAATTCTGGGGGGG

TGAAGAAGGTTTTAGGACTGTAAAACAATTTTAGTAGGGAAGAAAAAAATGACGGTACCT

ACAGAAGAAGCAACGGCTAAATACGTG

>6584 len=147 tot-seq=1

TGGGGAATATTGCACAATGGGCGAAAGCCTGATGCAGCGACGCCGCGTGAGGGATGACGG

CCTTCGGGTTGTAAACCTCTTTCAGTAGGGAAGAAGCCCCTTTTGGGGGTGACGGTACTT

GCAGAAGAAGCGCCGGCTAACTACGTG

>6585 len=147 tot-seq=1

TGAGGAATATTGGTCAATGGACGAGAGTCTGAACCAGCCAAGTAGCGTGCAGGATGACGG

CCCTATGGGTTGTAAACTGCTTTTATAAGGGAATAAAGTGGGAGTCGTGACTCCTTTTGC

ATGTACCTTATGAATAAGGACTGGCTA

>6586 len=147 tot-seq=1

TTAGGAATCTTCCCCAATGGACGAAAGTCTGAGGGAGCGACGCCGCGTGAGGGATGAAGG

TTTTCGGATCGTAAACCTCTGAATCAGGGACGAAAGGGCCTTCGGGCAGATGACGGTACC

TGAGTAATAGCACCGGCTAACTCCGTG

>6587 len=147 tot-seq=1

CAATGGACGAAAGTCTGACGGGGCAACGCCGCGTGAGTGAAGAAGGTCTTCGGATTGTAA

AGCTCTGTTATACGGGACAAAAAAGACGGATGCCGACAGTATCCGTCCGTGACTGTACCG

TAAGAGAAAGCCACGGCTAACTACGTG

>6588 len=147 tot-seq=1

CCGGTGAGGGCAGAGGCCGGTGGCCCAGGCGTGCGACGCAGGGGGCTCAGTGGGGGTGGG

AGAGGTGGGCTGATGGTCCCCGTGGTCGGTGGAGGTGAAGAAGTGAAGTCTGGGAAGATT

TTGGATACTATTCCAATCTCATGGGAG

>6589 len=147 tot-seq=1

GCAATGGGCGAAAGCCTGACGGAGCAACGCCGCGTGAGTGATGAAGGTCTTCGGATCGTA

AAACTCTGTTATTAGGGAAGAACAGATATATAAGTAACTGTGCACAACTTGACGGTACCT

AATCAGAAAGCCACGGCTAACTACGTG

>6590 len=147 tot-seq=1

GCAATGGGCGAAAGCCTGACGGAGCAACGCCGCGTGAGTGATGAAGGTCTTCGGATCGTA

AAACTCTGTTATTAGGGAAGAAAATATGTGTAAGGAAAGGTGAACATCTTGACGGTACCT

AATCAGAAAGCAACGGCTAACTACGTG

>6591 len=147 tot-seq=1

TGGGGGATATTGCACAATGGGGGAAACCCTGATGCAGCGACGCCGCGTGAGGGATGACGG

CCTTCGGGTTGTAAACCTCTTTCAGTTGGGAAGAAGTCCCCTTTGGGGGTGACGGTACCT

TCAGAAGAAGCACCGGCTAACTACGTG

>6592 len=147 tot-seq=1

CACAAGCCCACTTTCCCACTCCCCTGCTGGACTCCATCCGCTGGTTCATGTCCCACCCCT

GCTCAGCAGCTGGGCTCGCAGCTCTCCTTAACCACCGACCCCAGATTGAGATCTCCATCT

ACGAGGACCGGTGCAGCAGTGGCAGCT

>6593 len=147 tot-seq=1

CAAGAGAGGGAGAGAAGCCTCCCAGAGCGTATGTTTTTGTCTACATGAGAAGGATGGTGA

CAGGGGCATCACCAGCACCGCGGTAATCAATAGATCGGAAGAGCGTCGTGTAGGGAAGAG

TGGGCCAATGTGTAGATCTCGGTGGTC

>6594 len=147 tot-seq=1

TTAGAATTCAATCAGAGAAGCAGAACCATATAAGTGATATAAAATATTTATTATAGGGAT

GAGATCTCACGCAATTGTGGGACCTTGGTGAGTGTATAGGTAAGTCTGTTGCTCCTGTGT

CTAGTGCTGGGCCTGAAGTCAGCAAGG

>6595 len=147 tot-seq=1

TTAGGAATCTTCCCCAATGGACGAAAGTCTGAGGGAGCGACGCCGCGTGAGGGATGAAGG

TTTTCGGATCGTAAACCTCTGAATCAGGGACGAGAGACGCGGAAGCGGGATGACGGTACC

TGAGTAATAGCACCGGCTAACTCCGTG

>6596 len=147 tot-seq=1

ACAATGGACGCAAGTCTGATGGAGCAACGCCGCGTGAGTGAATAATTTTTTCGGATCGTA

AAGCTCTGTTGTTGGTGAAGAAGGATAGAGGTAGTAACTGGCCTTTATTTGACAATAATC

AACCATAAATTCACTTCTAACTACGTG

>6597 len=147 tot-seq=1

GGTATTCTGAGTCCTACAGAGAGGGACCTGCGGGGCCTGCCCCACAGGCACGTGGGGCTC

CTGCTGGGGCTGTCAAGGACCCCCGGGCACTGAGAGGACATCTGGGCCCTTGGGCAGCCA

GGCTGACGGGTTCCCTGCACACACGAT

>6598 len=148 tot-seq=1

TGACTGCCCTCAGAAAGACCCAACAAGCAGCACAAAGAGTCAGATATAGATATTTGCACT

CAACCAATAGACAGAAGCTGCCGACCCCTGTGGTTGAATTAGAGAAAAGCTGGAAGAAGC

TGAGAAGAAGGAAGACCCTATAGGAGGA

>6599 len=148 tot-seq=1

CACAATGGACGAAATGCTGATGGAGCAACGCCGCGTGAGTGAATAAGGGTTTCGGCTCGT

AAATCTCTGTTTGTAGTGAAGAAATATAGAGGTAGTATCTGGCTTTTATTTTACGGTAAT

TACTTATAAAGTCACGGCTAACTACGTG

>6600 len=148 tot-seq=1

CAAAACCTACCTATGGGGTAACATAGTTCCTCTAATAAGGCCACACTTCCCAATAGTGCC

ACTCCCTATGAGCCACACATTTAAACACAAGAGTCCATGGGGGCCATTCCTAGTCAGAGC

ACCACACAAGGTGACTCACAGTCTCATG

>6601 len=148 tot-seq=1

ACCTTGGTGTAAATCACTTGAGATGAAGTAATAGAATGTAGACATGGCCAGCTTTGCATG

ATTAAAAACAAAGCAAAACAACAACATCAAAAGCAAACAGCTCCCACAAAGCAATGGGTT

TCTTCCTGTGTGGCTATTGCTTGCACTT

>6602 len=148 tot-seq=1

TCAATGGACGAGAGTCTGAACCAGCCAAGTAGCGTGAAGGATGAAGGTTCTATGGATTGT

AAACTTCTTTTATAAAGGAAGAACGGCGGCTACAGGAAATGGTAGCCGAGTGACGGTACT

TTATTAGAAAGCCACGGCTAACTACGTG

>6603 len=148 tot-seq=1

CTGGCCGGCTGCAGCGCCTGCGGGATGCTCACGAGGCACAGCAGTACTACCTGGATGCAG

GCGAGGCCGAAGCCTGGATCAGCGAGCAGGAGCTATATGTGTTCTCTGATGAACCCCCTA

AGGTACACGGGAGGGGGGGCAGAGTCCG

>6604 len=148 tot-seq=1

TTAGAGGGTGCGCCAGAGAACCGGACAGCTTCTGGGATGGGCAGAAGCACAGAGCCACTG

AGGCAGCAGCCTGGGCGGGCCGCAGACAACCGGCCACCATCCGGACCAGAGGACAGGTGT

CCGCCTGGCTTGGGAGGCGACCTCAGCC

>6605 len=148 tot-seq=1

TTTGTGGTCTCAGGCATTCACTGGGGGTGTCTTAGAATGTTATCTACCATAGTGAATGGG

GAACTACTGCACTTATTTGGTTGTAACACAACACAGCCTCTCTAGCTCTTCAGGTAAAAC

TTTGCAGTTTAGAAGAAAACACCCTATC

>6606 len=148 tot-seq=1

TAGGGAATAGGCGGCAATGGGGGGAACCCTGACCGAGAAACGCCGCGAGAGTGAAGAAGG

TTTTCGGATAGTAAAGCTCTGTTGTTAAGGAAGAACGAGTGTGAGAATGGAAAGTTCATG

CTGTGACGGTACTTAACCAGAAAGGGAC

>6607 len=148 tot-seq=1

ACGAGAGGCAGCAGTGGGGAACTGTGCACAATGGGGGAAACCCTGAGGCAGCGACGCCGC

GGGATTTAGAAGGCCTTCGGGTTGTAAAAATCTTTTGTATGGGAAGAAAATGACAGTACC

ATACGAATAAGGACCGGCTAATTACGTG

>6608 len=148 tot-seq=1

TGGGGAATATTGCGCAATGGGGGAAACCCTGACGCAGCGACGCCGCGTGCGGGATGAAGG

CCTTCGGGTTGTAAGAGAAGAACGTGTGTGAGAGTGGAAAGTTCACACAGTGACGGTAAC

TTACCAGAAAGGGACGGCTAACTACGTG

>6609 len=148 tot-seq=1

TGAAAATGCAGGGACCCACCTGGAATCAGGAAAGACACACTGCCACTGCAGATGGGTGAC

TTTCAAGCGACTGCTGCCTTTACATCTGGGCACATAGTAACTGGACGCGAGCCCCGCGGA

GTAGCCCGCTGAACAAGCAGTGATGCAG

>6610 len=148 tot-seq=1

GCAATGGGGGAAACCCTGACGCAGCGACGCCGCGTGCGGGATGAAGGCCTTCGGGTTGTA

AAGCTCTGTTAATCGGGACGAATGGTCCTTGTGTGAATAATGCAAGGATTTGACGGTACC

GGAATAGAAAGCCACGGCTAACTACGTG

>6611 len=148 tot-seq=1

AGCAGTGGGGAATTGTGCACAATGGGGGAAACCCTGACGCAGCGACGCCGCGTGAGGGAT

GAAGGCCTTCGGGTTGTAAACCGCTTTTGATTGGGAGCAAGACTTTTGGGTGAGTGTACC

TTTCGAATAAGCGCCGGCTAACTACGTG

>6612 len=148 tot-seq=1

CACAATGGACGCAAGTCTGATGGAGCAACGCCGCGTGAGTGAAGGAGGTTTTCGGATAGT

AAAGATATGTTGTTGGTGAAGAAGGAGAGAGGGAGTAAATGGAATTTATTTGACGGTAAT

CAACCAGAAAGTCACGGCTAACTACATG

>6613 len=148 tot-seq=1

GCCAGTGGGGAAAGCACCAAGGCTTGCACATCAGGCCAGACCACCGGACCCCCGACCGAA

AAGTTATAGGCAGTAGACCCCGTCGGAGTCGATACGATTAAACCGTCCGCCCCGTACTCG

GAAACCGCTTCTCCATCAACGCCAAAAG

>6614 len=148 tot-seq=1

CACAATGGACGAAAGTCTGATGGAGCAACGCTGCGTGAGTGAAGAAGGGTTTCGGCTCGT

AAAGCTCTGTTGGTAGTGAAGAAGGATAGAGGTAGGAACCGGCCTTTATTTGACGGTAAT

TACTTAGAAAGTCACGGCTAACTACGTG

>6615 len=148 tot-seq=1

GTGCAGAGTTCAGAGTCAGGCTGAGCCCACATCCCTGAAAGAAGGGGCTCTCGGCCAGGG

CTCCTCCTGCTTTCTCACTGTGGGGATTGATCGGGTTGGGGACCAATTTCCCATTTCATT

TTCCTCTAACGCTTCCTCTCCCCCTTCC

>6616 len=148 tot-seq=1

AGGTATGGATAACCTGTGACCGAGCTGTAGCCACTAATATAGACTCCAAAAAGAGGAAAG

CATTTTACTTCCGACCAAGACTAAGGAAATGAGGATGTGACAAAAAGGGGAAATTTTGAA

GAAATTTCTTTGGCGCTTCTGTTGCTCT

>6617 len=148 tot-seq=1

GGTTCTTGTTTGGACCCTGCCCCTGGGCCATGGCCAGGTGACCATGGCTACATTGCCAAA

CCTCTGACTGCCACAGCTGCAGACTGAGAGGGTGGGTCTGAGTCCCCACAATGTCTGAAG

CTGCCCCTGGGATTCTCAGGCCAACCTG

>6618 len=148 tot-seq=1

TGAGCCAAGATCGGGCCACTGCACTCAAGCCTGGGCAACAGAGCGAGACTCTGGGTCAGA

ACAAATGAAAAACCAGCACCAGCATGAAGAGCCTGTGTATTGCCTGGGGTACTTTGCTGC

CCTTGGGCAGAATCTGCATCCCTCCCAG

>6619 len=148 tot-seq=1

ACAATGGGAGCAAGCCTTATGAAGCAATGCCGCGTGAACGAAGAAGGTCTTAGAGATTGT

AAAGTTCTTTTATATGAGAAGAAACGCTAAAATAGGAAATGATTTTAGTTTGACTGTACC

ATTTGAATAAGGATCGGCTAACTACGTG

>6620 len=148 tot-seq=1

TGGGGGCTTAGACCAAGGGGAGCTCCGGAGCTTGAAGGTGCACATCCCCACACCCCGCTC

GTGCCTGTGGGCTCCAGGAAGGTGGGCATCCGGTCACTCTAGCTCTGCATTCCCGTACCT

CAGCCTGGCGCCTGGCACACAGTGGCTC

>6621 len=148 tot-seq=1

CACAATGGACGAAAGTCTGATGGAGCAACGCCGCGTGAGTGAAGAAGGTTTTCGGCTCGT

AAAGCTCTGTTGGTAGTGAAGAAGGATAGAAGTAGTAACTGGCCTTTATTTGACGGTAAT

TACTTAGAAAGTCACGGCTAACTACGTG

>6622 len=148 tot-seq=1

CACAATGGACGAAAGTCTGATGGAGCAACGCCGCGTGAGTGAAGAAGGGTTTCGGGTCGT

AAAGCTCTTTTGGTAGTGAAGAAAGATAGAGGGAATAACTGGCCTTTATTTGACGGTAAT

TACTGAGAAAGTCACGGCTAACTACGTG

>6623 len=148 tot-seq=1

CACAATGGACGAAAGTCTGATGGAGCAACGCCGCGTGAGTGAAGAAGGGTTTCGGCTCGT

AAAGCTCTGTTGGTATTTAAGAAAGATAGGGGTAGTAACTGGCCTTTATTTGACGGTAAT

TACTTAGAAAGTCACGGCTAACTACGTG

>6624 len=149 tot-seq=1

GTGACAACCAAAGAATGGAGAAATTGGAGAAAACCAACAGCAGCTTGCGCAAACAGAACC

GTGACCTCCTGGAGCAGTTGCAGGTAGAGCATATTTGCCGAGCGGCTTCCCGAGCCTCTG

TGTAGTCGCTCATTAACTCACCAAAGGCA

>6625 len=149 tot-seq=1

ACGGGAGGCAGCAGGGGGGAATATGGCAAAAGGGGGGAAACCAGGAGGCAGCGACGACGA

GGGAGAGAAGAAGGAGGTCGGTATGGAAAGCTCTAGCAGAAGGGAAGAAAATGAAGGGAC

CTGACTAAGAAGACCCGGCTAAATACGTG

>6626 len=149 tot-seq=1

ACGGGAGGCAGCAGGGGGGAATATGGCAAAAGGGGGGGAACCAGGAGGCAGCGACGACGA

GGGGGGGAGGAAGGAAGTCGGTATGGAAAGCCCTAGCAGAAGGGAAGAAAATGAAGGGAC

CTGACTAAGAAGACCCGGCTAACTACGTG

>6627 len=149 tot-seq=1

ACTGTTCCCCAATTATTCCCTGATTGGTCAACTAAAAAGCTGATTAACCAATGACTAAGC

AGGGGAGAGAATAGAGCTAGATTTCCTCCTAGTCAGGGGAATGGAGAAAGGCGAGAAGAG

GAAGTTGGGATTCCCCTTGGGGAGAAAGA

>6628 len=149 tot-seq=1

CAGCAGCAGCAGCAGCAGCAGCAGCAGCAGCATCACCACCACCAGCAGCAGCAGCAGCAG

CATCACCACCACCATCACCACCACCACCATCACCAGCAGCAGCAGCAGCAGCAGCAGCAG

CAGCATCACCACCACCATCACCACCATCA

>6629 len=149 tot-seq=1

CTGTCCACACCAGGCACTGTGTGGTATTTGGTCACTAGTGCCTGCCCTGCCAGGACCATG

TGCTGGGTGGAGGTAAAGGTATCCTCAGTCAGGCTTGGCGGCAGAGCCTGAATGAGGGAC

TGAGAAACATGGTCCTCCGCTCAGCAGCC

>6630 len=149 tot-seq=1

TGGGGAATCTTCCGCAATGGGCGAAAGCCTGACGGAGCAACGCCGCGTGAGTGATGACGG

CCTTCGGGTTGTAAAGCTCTGTGATCGGGGACGAACGGACAGGAAGCTAATAACTTCCTG

TAGTGACGGTACCCGAATAGCAAGCCACG

>6631 len=149 tot-seq=1

ACGGGAGGCAGCAGTGGGGAATATTGCAAAATGGGCGAAAGCATGATGCAGCGACGACGA

GTGAGAGAAGAAGTATTTCGGTATGCAAAGCTCTATCAGAAGGGAAGAAAATGAAGGTAC

CTTACTAAGAAGATCCGGCTAAATACGTG

>6632 len=149 tot-seq=1

TGGGGAATATTGCGCAATGGGGGAAACCCTGACGCAGCGACGCCGCGTGAGTGAAGAAGT

ATTTCGGTATGTAAAGCTCTATCAGCAGGGAAGAAAGGCTCGTAAGAGAGATGATGGTAC

CTGACTAAGAAGCCCCGGCTAACTACGTG

>6633 len=149 tot-seq=1

CCACAATGGACGCAAGTCTGATGGAGCAACGCCGCGTGAGTGAAGAAGGTCTTCGGATCG

TAAAACTATGTTGTTAGAGAAGAAAAAGAGTAAGAGTAAATGTTAATTCGATGACGGTAT

CTAACCAGCAAGTCACGGCTAACTACGTG

>6634 len=149 tot-seq=1

CCACAATGGACGCAAGTCTGATGGAGCAACGCCGCGTGAGTGAAGAAGGGTTTCGGCTCG

TAAAGCTCTGTTGTTAAAGAAGAACGTGAGGAAGAGTAACTGATTACCGTGTGACGGTAT

TTAACCAGAAAGCCACGGCTAACTACGTG

>6635 len=149 tot-seq=1

CGCAATGGACGAAAGTCTGACGGAGCAACGCCGCGTGAGGGAGGACGGCCTTCGGGTTGT

AAAGCTCTGTTAATCGGGACGAATGGTCCTTGTGGGAATAATGCAAGGATTTGACGGTAC

CGGAATAGAAAGCCACGGCTAACTACGTG

>6636 len=149 tot-seq=1

CCGCAATGGGCGAAAGCCTGACGGAGCAACGCCGCGTGAGTGATGAAGGTCTTCGGATCG

TAAAACTCTGTTATTAGGGAAGAACATACGTGCAAGTAACTAGGCACGTCTTGACGGTAC

TTAATCAGAAAGCCACGGCTAACTACGTG

>6637 len=149 tot-seq=1

CCACAATGGACGAAAGTCTGATGGAGCAACACCGCGTGAGTGAAGAAGGTTTTCGGATCG

TAAAGCTCTGTTGTTGGTGAAGAGGGATAGAGGTAGTAACTGGCCTTTATTTGCCGGTAA

TCAACCAGAAAGTCACGGCTAACTACGTG

>6638 len=149 tot-seq=1

TAGGGAATCTTCCACAATGGACGCAAGTCTGATGGAGCAACGAAGAAGGTTTTCGGATCG

TAAAGCTCTGTTGTTGGTGAAGAAGGATAGAGGTAGTAACTGGCCTTTATTTGACGGTAA

TCAACCAGAAAGTCACGGCTAACTACATG

>6639 len=149 tot-seq=1

CCGCAATGGACGAAAGTCTGACGGAGCAACGCCGCGTGAGTGAAGACGGCCTTCGGGTGG

TAAAGCTCTGTGATTCGGGACGAAAGGCCATATGGGAAGAAGATATGGAAATGACGGTAC

CGAAAAAGCAAGCCACGGCTAACTACGTG

>6640 len=149 tot-seq=1

CCACAATGGACGCAAGTCTGATGGAGCAACGCCGCGTGAGTGAAGAAGGGTTTCGGCTCG

TAAAGATATGTTGGTAGTGAAGAAAGATAGAGGTAGTAAATGGAATTTATTTGACGGTAA

TTACTTAGAAAGTCACGGCTAACTACGTG

>6641 len=149 tot-seq=1

TGGGGAATATTGCACAATGGGGGCAACCCTGATGCAGCAACGCCGCGTGAGTGAAGAAGT

ATTTCGGCATGTAAAGCTCAGTTGTTGGTGAAGAAGGACAGGGGTAGTAACTGACCTTTG

TTTGACGGTAATCAATTAGAAAGTCACGG

>6642 len=149 tot-seq=1

TGGAGGAGGTAGCAAGAGAGATAGCCATGTGAGGATGCAAGGGAAAGGCATCCAGGAAGA

GGGAACAGCATGTGCAAAGATCCTGCGGCACTGAGGCCAGGGGACGGAGCAGAAGAGGAA

CGGGGTTTGATGGTTGATAAAACATCCCC

>6643 len=149 tot-seq=1

AGTATCCCGTTCCTAGAGGTCCTAGAGGGCATTGGGGCTCACCAGAGAATCCAGGGTGCA

GACTGGAGGCTTGGACTGACAGATGAAGTTGTGGAACTCTGGAAAACAAGAGCTTGAGGG

GCCGGGCGCAGTGGCTCACGCCTGTAATC

>6644 len=149 tot-seq=1

TTTTTTTTTTCAAGCAGAAGACGGCATACGAGATAACACTGTGACTGGAGTTCAGACTGT

GCTCTTCCGATCTCCACGGGAGGCAGCAGCCTGGGTTCTGACAAGTCTGCCAGCAAGCTC

CTATGCATCTCAAGTCCGGGAACCATGCA

>6645 len=149 tot-seq=1

CCAATCAGAGGTGAAGCTTTCATCCTGGGGTCCTCATGCAGCCCCCAGATCCTTTCCACC

ATTTAAAAATATGGAGATATCTATAAAGTTCGGATTTACACCTTCTTCTGAGAACTGAGA

GACCTTTCAACACAGGCTCACCATGGTGC

>6646 len=149 tot-seq=1

TGGGGGATATTGCACAATGGAGGAAACTCTGATGCAACGACGCCGCGTGAGTGAAGAAGT

ATTTCGGTATGTAAAGCTCTATCAGCAGGGAAGAAAGGCTCGCAAGAGAGATGACGGTAC

CTGAGTAAGAAGCTCCGGCTAAATACGTG

>6647 len=149 tot-seq=1

CTCCATTTGTTATCCAGTGCTTTGAATTTTAGGATCTCAGCTCACATGAGTGAAGCTGAT

ATTTTGTCCTGGCTTCCCAGGGAGTCTCCAGCGGTGACTCCTTTCCTGGGTTGTGTGACC

AATAACCCGGTCCAGCTCACCATTCCCTA

>6648 len=149 tot-seq=1

TCAAGCAGAAGACGGCATACGAGATTTGACTGTGACTGGAGTTCAGACGTGGCTCTTCCG

ATCTCCTACGGGAGGAAGCAGGCCAGGAACCTGGGCTGGGACTAATTATTGCTGAAGAGA

GAACAGGGCCAAGTGTCCCTCATGTCCCC

>6649 len=149 tot-seq=1

CGGGAGGCAGCAGGCGCGCAAATTACCCACTCCCGACCCGGGGAGGTAGTGACGAAAAAT

AACAATACAGGACTCTGTCGAGGCCCTGTAATTGGAATGAGTCCACTTTAAGGCCTGTAA

CGAGGATCCATTGGAGGGCAAGTCTGGTG

>6650 len=149 tot-seq=1

GGGAAGGAAGTGCTCCTTGGCCCCCCACCATGTGCAGACAAGTGGAGGAGGCAGTGGAGT

CTTAGGAGCGGGGCTGCCCACCTGCTAAAGGATGAGAGAGGTTCAAGAGGAGGGGGTTCA

GGGAATTGGGATGGCAGGGGGCATCAGGC

>6651 len=149 tot-seq=1

CGGGAGGCAGCAGGCGCGCAAATTACCCACTCCCGACCCGGGGAGGTAGTGACGAAAAAT

AGCAATTCAGGACTCTGTCGAGTCCCTGTAATTGGAATGAGTCCACTTTAAATCCGGTAA

CGAGGATCCATTGGAGGGCAAGTCTGGTG

>6652 len=149 tot-seq=1

TGCTGTGTTGTTCTCTCCCAGACCGAATAGGACTGTCCTGTGTAATCACTAGGATATTGT

GGAAATTTAAAAGTGTGACTTTGAAGGCGAGGTTATAAAAGACGTGGTGGTTTTCATTTT

GCCATCTTGGATTACATGCTCTGAGGATG

>6653 len=149 tot-seq=1

CCGCAAAACAACAACCAGCCAAATGTAATACTGCCATTGCCGGCTGATTTTGTTTTGCAA

ACACTGTTTTCCAAAAAGTAAAGCCAGAATAAGTAAACTGCCGGAAAAAGACATGGACAG

GAAAATCTTCAAAACTGTGTTCATTGCTG

>6654 len=149 tot-seq=1

TAGGGAATCTTCGGCAATGGGGGCAACCCTGACCGAGCAACGCCGCGTGAGTGAAGAAGG

TTTTCGGATCGTAAAGCTCTGTTGTAAGAGAAGAACGTGTGTGAGAGTGGAAAGTTCACA

CAGTGACGGTAACTTACCAGACAGAACTA

>6655 len=149 tot-seq=1

AACAGCACTCAGGACCCCATCACTTTAATACAACTGCGGTTGCGTCACATGGTTTCTGTG

CTGGCATGTTCATCTCCTCACCCTCCTGCATGTGGCTGAGTGGCTTTGGTGCCCGGGAGG

CATCCCTCCCATCCTCTCACAGCCTGGCC

>6656 len=150 tot-seq=1

TACGGGAGGCAGCAGTGGGGAATATTGCACAAGGGGGGAAACCCGGATGCAGCGACGCCG

CGGGAGGGAAGAAGTATTTCGGTATGTAAAGCTCTATCAGCAGGGAAGAAAATGACGGGA

CCTGATTAAGAAGCCCCGGCTAACTACGTG

>6657 len=150 tot-seq=1

TCATTTTTACTTACTGAGCCATCTTTCTGGCTCCAAAAGAAGGAATTTGATCCTTTGGTT
TTGGTGTTTATAGGGTTGCTCATCCCCAATGGTGGTAAAGTTTGCCGACACCCAGAAGGA
CAAAGAACAGAAGAGAATGGCCCAGCAGCT
>6658 len=150 tot-seq=1
TGGGGGATATTGCACAATGGGGGAAACCCTGATGCAGCGATGCCGCGTGAGGGATGAAGG
TTCTATGGATCGTAAACCTCTTTTATAAGGGAATAAAGTGCGGGACGTGTCCTGTTTTGT
ATGTACCTTATGAATAAGGATCGGCTAACT
>6659 len=150 tot-seq=1
CTGGAGTGAGTAGGTGCATGTGTTGTGTCTCCCCAGGAACAGTTTTGTCACACAACACAC
TGCTAGAATGTGCATGGATGTAGGACCATCTACTTGATCATGGGTAACCTCCAAAGGACA
GCATCCTTGAAAAAAGCTGACTCTCTCTCT
>6660 len=150 tot-seq=1
AACAGAGCAGAGACTGAAGGAAAGGCTATCCAGAGACCTGCTTAACTTGGGATCTATCCC
AAGTGCAGGCACCAAACCCGGACACTGTTATTCATGCCATGTTGTACTTGCAGACAGGAG
CCTGGCATGACTGTACTTTGAAAGGTTCTA
>6661 len=150 tot-seq=1
TGGGGGATATTGCACAATGGGCGCAAGCCTGATGCAGCGACGCCGCGTGGGGGATGAAGG
CCTTCGGGTTGTAAACTCCTTTTGCTCTGAACAAGGCGCACTATTGGGTGTGTTGAGTGT
AGGGGTTGATTAGCGCCGGCTAACTACGTG
>6662 len=150 tot-seq=1
AACACAGGAGAGGGCGGAGACGCAGTGCTCACTTGCCACCTGACAGAGGCACACACGTGC
ACACAGAGTCACAGGCAGAGAGAGGCATCGCACACTCAGCCTGTGAGCCACACGCATCAG
AACCACCCGGAACACACACACATACCTACT
>6663 len=150 tot-seq=1
TGGGGAATTTTGCACAATGGAGGAAACTCTGATGCAGCGACGCCGCGTGAGTGAAGAAGG
GTTTCGGCTCGTAAAGCTCTGTTGTTGGTGAAGAAGGACAGGGGTAGTAACTGACCTTTG
TTTGACGGTAATCAATTAGAAAGTCACGGC
>6664 len=150 tot-seq=1
CTACGGGAGGCAGCAGTGGGGAATGTTGCACAATGGGGGAAACCATGATGCAGCGACGCC

GCGTGATTTAGAAGGCCTTCGGGTTGTAAAAATATGTTGGATGGGAAGAAAATGACAGTA

CCATACGAATAAGGACCGGCTAATTACATG

>6665 len=150 tot-seq=1

AATCTTCCACAATGGACGCAAGTCTGATGGAGCAACGCCGCGTGAGTGAAGAAGGGTTTC

GGCTCGTAAAGCTCTGTTGTTGGTGAAGAAGGGGTAGTGACTGACCTTTGTTTGATGGTA

ATCAATTAGAAAGTCACGGCTAACTACGTG

>6666 len=150 tot-seq=1

TAGGGAATCTTCGGCAATGGACGGAAGTCTGACCGAGCAACGCCGCGTGAGTGAAGAAGG

TTTTCGGATCGTAAAACTCTGTTATTAGGGAAGAACATATGTGTAAGTAACTGTGCACAT

CTTGGCGGTACCTAATCAGAAAGCCACGGC

>6667 len=150 tot-seq=1

CCCTGGAGGCAGCATCTCTTTGGTGTGGACACCCTTGGACACATGGGCTCTCACCCCGGG

AGGCCTGGGACAGTCTGCCCCCTCATCTCTCCTTGGCTCATGGCCTCCTCCTTCCCACTC

TGTAGGACTTCCCGCACATCTCTGAGTTCC

>6668 len=150 tot-seq=1

AATAACACTGAACATCTATGATGTATCAGGCACCATTCTATGTGCTTTACATGTATCACA

TTATTTAACATAGCAATCCTGTGAGAGGTTCTAGTGCCATCCTCAGCACTTTTGCTGACT

CCAGGCTTCTTGCTTTGGAGGCAGTGATGT

>6669 len=150 tot-seq=1

GGTGAAGAGGGAGGAGACTGGAGCTTGTGTGTGAGGGTGGCTGGGGACCTGACGGGGGGC

GGCACAGGCCGAGAGGCCACCAGCTGCACCCCCTCCTTTGCCCCGAGACTGTGGGTGCTC

CAGGCAGAGACAGCCCCGATGGGACTCATG

>6670 len=150 tot-seq=1

AATTTTCGGCAATGGGGGAAACCCTGACCGAGCAACGCCGCGTGAGTGATGACGGCCTTC

GGGTTGTAAAGCTCTGTTGTAAGAAAGGATAAATTAGTAGAGAATATTAGTCAGACATTA

TCTTACCAGAAAGCCACGGCTAACTACGTG

>6671 len=150 tot-seq=1

TCCACAATGGACGAAAGTCTGATGGAGCAACGCCGCGTGAGTGAAGAAGGGTTGCGTCTC

GTAAAGCTCTGTTGGTAGTGAAGAAAGATAGAGGTAGTAACTGGCCTTGATTTGACGGTA

ATTAATTAGAAAGTCACGGCTAACTACGTG

>6672 len=150 tot-seq=1

TCCACAATGGACGAAAGTCTGATGGAACGACGCCGCGTGAGTGAAGAAGGTTTTCGGCTC

GTAAAGCTCTGTTGGTAGTGAAGAAAGATAGAGGTAGTAACTGGCCCTTATTTGACGGTA

ATTACTTAGAAAGTCACGGCTAACTACGTG

>6673 len=150 tot-seq=1

CTCCACAATGGACGAAAGTCTGATGGAGCAACGCCGCTTGAGTGAAGAAGGGTTTCGGCT

CCTAAAGCTCCGTTGGTAGTGAATAAAGATAGAGGTAGTAACTGGCCTTTATTTGACGGT

AATTACTTAGAAAGTCACGGCTAACTACGT

>6674 len=151 tot-seq=1

TGGGGAATATTGGACAATGGGCGCAAGCCTGATCCAGCCATGCCGCGTGAGTGATGAAGG

CCTTAGGGTTGTAAACTGCTTTTGTTTGGGAATAATCGACATTACGTGTAATGTTTTGCA

TGTACCATTCGAATAAGGACCGGCTAATTCC

>6675 len=151 tot-seq=1

ACAGGGCCTTGTTGAGATTGAGCTGCCCTGTAGCATCACAGGCAGAAAACGCAGAGGAGA

TCCTCTGTAAATGCAGAGAGGAAATGCAGCAAGGTGTCAGACTGCACCTGAGGCTTAGGA

GGCAGCCTGCAGAGGACTGACAATGGACAAA

>6676 len=151 tot-seq=1

TGAGGAATCTTGGTCAATGGACGAGAGTCTGAACCAGCCACGTCGCGTGCAGGATGACGG

CCCTCTGGGCTGTAAACTGCTTTTATAAGGGAATAAAGTGAGTCTCGTGAGACTTTTTGC

ATGTACCTTATGAATAAGGACCGGCTAATTC

>6677 len=151 tot-seq=1

TGGGGAATATTGCACAATGGGGGAAACCCTGATGCAGCGACGCCGCGTGAGTGAAGAAGT

ATTTCGGTATGTAAAGCTCTATCAGCAGGGAAGAAAAAGAACCAAGGTTCTTCGGACGGT

ACCTGAGTAAGAAGCCCCGGCTAACTACGTG

>6678 len=151 tot-seq=1

CAAACCCAGGTAAGTCTAATGCCTGCCCCAGCTCTGAGGTCCAAAGTGTGTGCCTATCTC

ACCTGGAAGACACCCTGGGCATCGGCTGTGACAGCTGTGTGCAGGGGAGTGCGGCCTGAG

TGGTCCTGGGCATTGGTGTCTGCCCCAGCAT

>6679 len=151 tot-seq=1

GGACACAAGGGAAGAAAGGAGGAGGGAGAAACGACGAGGGAGGGAAGAAGGGGGGAGGAG

AGGAAAGCGCGGGGGGGGGGGAAGAAGGAGAGAGGGAGGAACGGGCCTTGATTTGACGGT

AATCAACCAGAAAGTCACGGCTAACGACGTG

>6680 len=151 tot-seq=1

CTTCCTTTCTATCCCCATTCTCATCCCCTCCCTCTTTTCCAAGACCAGAGCCAGAAGAGG

GCAGGATTGAGAGGACAACTCACACAGGATACTTCCCTACTGGTCCACCTATGCACCTTT

GGAGTCTGGCTCTCTGTCCTGCTCAGAGGTA

>6681 len=151 tot-seq=1

AGAAGACTGCAGACTTCCTGGCAGTCCTTGGAAGGCATTCGACTGTGACTGAGTGAGGAA

GGGTCCGGAGTGGGGAACGGGGGTGATCCGCCTCAGATGGAACAGGGAACCCTCCAGCCA

CCGGGTGGAGAAACGGACCATGAAGTCAAAG

>6682 len=151 tot-seq=1

TGGGGAATATTGGGCAATGGGCGAAAGCCTGACCCAGCAACGCCGCGTGAGTGATGAAGG

CCTTCGGGTTGTAAACTGCTTTTGTTTGGGAATAATCGACATTACGTGTAGTGTTTTGCA

TGTACCATTCGAATAAGGACCGGCTAATTCC

>6683 len=151 tot-seq=1

GAAAATAAATTAGGTTTGCCTTAGGCCTCACAATCATTCGCGACCACTCAGAGGCTGTAA

CTTCCAGGCCTGGTTTGCTGATGGGGTCATGCTCTTTGTGACTGGTAATGAGCTGGACAA

CCTGCCCACTTAACTCAAGAGGCCACCAGCA

>6684 len=151 tot-seq=1

TTTTTTTTTTCAAGCAGAAGACGGCATACGAGATCCAACCGTGACGGGAGTTCAGACGTG

TGCTCTTCCGATCTCCTAGGAGGCAGCAGACACCAGGCCTACATGGACCACCTGAAGATA

TTTTCGAACATGAAATCAACAGTCCCTCAAC

>6685 len=151 tot-seq=1

TGGGGAATATTGCACAATGGGCGCAAGCCTGATGCAGCGACGCCGCGTGAGGGATGAAGG

CCTTCGGGTTGTAAACCTCTTTCAGTAGGGAAGAAGCCTGTCTTTTGGGTGGGTGACGGT

ACCTGCAGAAGAAGCGCCGGCTAACTACGTG

>6686 len=151 tot-seq=1

TGGGGAATATTGGACAATGGGCGGAAGCCTGATCCAGCAACGCCGCGTGAGGGATGACGG
CCTTCGGGTTGTAAACCTCTTTCATTACCGACGAAGCTCAGTGTTGATCTGGGTGACGGT
AGGTGGAGAAGAAGCACCGGCCAACTACGTG
>6687 len=152 tot-seq=1
TGAGGAATATTGCGCAATGGAGGAAACTCTGACGCAGCGACGCCGCGTGAAGGATGAAGG
CCCTCTGGGTTGTAAACTTCTGTAGAGAGGGAAGAATTTCTCGCTCCGCGAGGTTGACGG
TACCTCTAAAGTAAGGGTCGGCTAACTACGTG
>6688 len=152 tot-seq=1
GTTGTTTGTTTTTTATTTCTCAAGACAGGGTTTCTCCGTGTAGCCCTGGCTGTGCTGGAA
CTCACTCTGTAGTCCAGGCTGGCCTTGTACTCACAGAGATCTGCCTTCCTCCCCTTCCCA
AGTGCTGGGATTAAAGGCATGCGCCCTCATGT
>6689 len=152 tot-seq=1
TGGGGAATATTGCACAATGGGGGAAACCCTGATGCAGCGACGCCGCGTGAAGGATGAAGT
ATTTCGGTATGTAAAGCTCTATCAGCAGGGAAGAAAAAAAGTCCACAGGACTTTGGACGG
TACCTGACTAAGAAGCCCCGGCTAACTACGTG
>6690 len=152 tot-seq=1
TCTCCTGTCCCTCTTGCTTACTCTTAGCCCATAAATTTCCATGTTCTGAGCTGCCCTAAG
GAGATTCTCAGGTGATAATGAACTGAGGGCTGTGAGGAAGGGAGCACAGCCAGCAGCCAG
GGAGAAGCTGAGACCTGCAGACCCTCATGATT
>6691 len=152 tot-seq=1
CTTCTATGAGTGCATCCCTAACTGATCCTCAGCTCACAGGAAAATGAGCATCCCCATCCA
TTGGCCTCTAGCATAAACCAGGACCCTTTCCCTCCATCGTCATTCATGAAAACCTCCAAG
CCCCTACCCCGTCAATAAAAGAGTTTGTCATT
>6692 len=152 tot-seq=1
GGAATCTTCCACAATGGACGCAAGTCTGAACCAGCCAAGTAGCGTGAAGGATGACTGCCC
TATGGGTTGTAAACTTCTTTTATACGGGAATAAAGTGAGGCACGTGTGCCTTTTTGTATG
TACCGTATGAATAAGGATCGGCTAACTCCGTG
>6693 len=152 tot-seq=1
TGGGGAATCTTGCGCAATGGTCTGACGACTGACGCAGCGACGCCGCGTGTGGGAGGACGC

CCTTCGGGGTGTAAACCACTGTTGCCCGGGACGAACCGCTGGTTTCGACCAGCCTGACGG

TACCGGGTGAGGAAGCACCGGCTAACTCCGTG

>6694 len=152 tot-seq=1

CGCAAGCCTGATGGAGCAACGCCGCGTGAGTGAAGAAGGGTTTCGGCTCGTAAAGCTCTG

TTGTTGGAGAAGAACGTGTGTGAGAGTAACTGTTCACGCAGCGACGGTAACCACCCAGAA

AGTCACGGCTAACTACGTGGTAAAGCTCTGTT

>6695 len=152 tot-seq=1

GCAACTGCAAAAGCCACAGGATAAAGAAAGTAGCAACGACGATAAGTACAGAACTGAGGT

TTGTACCGAACGCCAGCGAACCAAGCAGATCTTTCATCTTCGCATTCTCTTTGGTACGGA

CGGCGAAGATACCTATAATAGAAAGGATAATC

>6696 len=152 tot-seq=1

CACAATCATCCTGATATCATATATGTCAGTCATGAAAAGCCCAACAACATATCGGTTGAC

GACATAAGGACACAGCTCAACAACGATATAGTGATAAAGCCGTACAGCAGTAAATACAAG

ATTTACATAGTGGATGAGGCTGAAAAAATGAA

>6697 len=152 tot-seq=1

TGGGGGATATTGCACAATGGACGCAAGTCTGATGCAGCGACGTCGTGTGGGGGATGAAGG

CCTTCGGGTTGTAAACTCCTTTCGCCTGCGGAAAAGGCAGGGTTTTGGCCTTGTTGATGG

TAATCAATTAGAAAGTCACGGCTAACTACGTG

>6698 len=152 tot-seq=1

TGGGGAATATTGCACAATGGGCGCAAGCCTGATGCAGCGACGCCGCGTGGGGGATGACGG

CCTTCGGGTTGTAAACCTCTTTCAGCCTCGACGAAGCTGCGGGTTTTCTCGTGGTGACGG

TAGGGGCAGAAGAAGCACCGGCCAACTACATG

>6699 len=152 tot-seq=1

TGGGGGATATTGCACAATGGAGGAAACTCTGATGCAGCGACGCCGCGTGAGTGAAGAAGG

TTTTCGGATCGTAAAGCTCTGTTGTAAGAGAAGAACGTGTGTGAGAGTGGAAAGTTCACA

CAGTGACGGTAACTTACCAGAAAGGGACGGCT

>6700 len=152 tot-seq=1

TGAGGAATATTGGTCAATGGACGAGAGAATGCAGCCAAGTAGCGTGCAGGATGACGGCCC

TATGGGTTGTAAACTGCTTTTATAAGGGAATAAAGTGAGTCTCGTGAGACTTTTTGCATG

TACCTTATGAATAAGGACCGGCTAATTCCGTG

>6701 len=152 tot-seq=1

TGAACAGGTTGAGGGAGGCCAGGCCACTGGGCAGCGTCACATCGGCTGTGAAGAGATAGG

CCTGTGTGGGGTGAGTAGTCCTAGCCCTTCCTCCCCTGCCAATCACATCTCCCGGTGCCC

AGCCATGAAATTAACCTGGTGACATTTCCAGC

>6702 len=152 tot-seq=1

TAGGGAATATTGCACAATGGGGGAAACCCTGATGCAGCCATGCCGCGTGTGTGAAGAAGG

GTTTCGGCTCGTAAAGCTCTGTTGTTGGTGAAGAAGGACAGGGGTAGTAACTGACCTTTG

TTTGACGGTAATCAATTAGAAAGTCACGGCTA

>6703 len=152 tot-seq=1

GAGGGGCTCGGCGTTGCAGGAGATTGAGGCTGAGGGAGCTTGAGGGAGATGAAGGGGGCA

GCTCAGAAGCCCAGAGGGACCTGAGGGCTCTGGGGAAGCAGAGGGGAAAGGAGCCAGTAG

AACGGACAGGACGAGGCCAGGCAGAGCTGGGA

>6704 len=152 tot-seq=1

TGGGGAATATTGCACAATGGGGGAAACCCTGATGCAGCGACGCCGCGTGAGTGAAGAAGT

ATTTCGGTACGTAAAGCTCTATCAGCAGGGAAGAAAAAAAGTCCTTTGGACTTTGGACGG

TACCTGACCAGAAAGGGACGGCTAACTACGTG

>6705 len=152 tot-seq=1

TGAGGAATATTGGTCAATGGACGCAAGTCTGAACCAGCCAAGTAGCGTGCAGGGTGTAGG

CCTTCGGGTTGTGAACTCCTTTTTCTCGCGAAAAAGGCACAGTTTTGGCTGTGTTGATGG

TAGTGGGGGAAGAAGCGCCGGCTAACTACGTG

>6706 len=152 tot-seq=1

TGAGGAATATTGGTCAATGGGCGAGAGCCTGAACCAGCCAAGTCGCGTGAAGGATGACTG

TCTTATGGATTGTAAACTTCTTTTGTAGGGGAATAACGGACGGCACGTGTGCCGTAGTGA

ATGTACCCTACGAATAAGCATCGGCTAACTCC

>6707 len=152 tot-seq=1

TGGGGAATATTGCGCAATGGACGAAAGTCTGACGCAGCGACGCCGCGTGTGGGATGAAGC

CTTTCGGGGTGTAAACCACTGTTGCCCGGGACGAAACTCCTTTTTCGAGAGGATTGACGG

TACCGGGTGAGGAGGCACCGGCTAACTCTGTG

>6708 len=152 tot-seq=1

ACAGGGGTTCTGAGGAAGGCAAGGGGACTGGGTGTTTGAAATCAGGATGGTCTCAGCCGA

TCCAGACTATTAGGTGGCTGTGTGCTTATCATTGTTTAGGAAGCAATCCTTGTACCCCCA

GGTCATGCATGTTGCTGGAATCTCTGTCCACA

>6709 len=152 tot-seq=1

TAGGGAATCTTCCACGATGGACGCAAGTCTGATGGAGCAACGCCGCGTGAGTGAAGAAGG

TCTTCGGATCGTAAAACTCTGTTGTTAGAGAAGAACGGAAGAGCGTCGTGTAGGGAAAGA

GTGTGCCAATGTGTAGATCTCGGTGGTCGCCG

>6710 len=152 tot-seq=1

GAAAATACTTACGGGATGATTAAGGAGTTCTTGCCAGATGTACCGGAGGATGCCGTTATG

TATCTGGTCAGTACCACTGCATTGGATGCGAGATGGAGCGAGGTTTACTATACTTCTTCG

CAGGGGAACTTTACGAAAGAAGATGGAACGGT

>6711 len=152 tot-seq=1

CCGCTTGTTGTTGCGCTTACTCAGGCGAAGGGCTTGAGTATTGTGCATGAAACGGTTTAC

GAGAATCGTTTTGGTTTCACTAAGCCTCTTGTTCAAATGGGCGCTATGGTTCAGCTGTAT

CGTGAGTGCTTGGGTTCTCTTCCTTGCCGCTT

>6712 len=152 tot-seq=1

GGAATATTGGTCAATGGACGCAAGTCTGAACCAGCCATGCCGCGTGCAGGATGACGGCTC

TATGAGTTGTAAACTGCTTTTGTATGAGGGTAAAATCGGGTACGTGTACCTGACTGAAAG

TAGCATACGAATAAGGAGCGGCTAACTCCGTG

>6713 len=152 tot-seq=1

TGGGGAATATTGCACAATGGAGGGAACTCTGATGCAGCGACGCCGCGTGAGTGAAGAAGT

ATTTCGGTATGTAAAGCTCTATCAGTAGGGAAGATAATGACGGTACCTACATAAGAAGAT

CGGAAGAGCGTCGTGTAGGGAAGAGTGTGCCA

>6714 len=152 tot-seq=1

GCAAGAGCGGGGTGATGAGTTTGTGTTTGGATGTGGTGAATTGGAGGAGGCCGTCCAGGC

CATTACCAGCACCGCGGTAATGACAAGATCGGAAGAGCGTCGTGTAGGGAAAGAGCGTGC

CAATGTGTAGATCTCGGTGGTCGCCGTATCAT

>6715 len=152 tot-seq=1

TGGGGAATATTGCGCAATGGGGGAAACCCTGACGCAGCGACGCAGCGTGCAGGATGACGG

CCCTATGGGTTGTAAACTGCTTTTGTTGGGGAATAACTGACATTACGTGTAATGTTTTGC

ATGTACCCTTCGAATAAGGACCGGCTAATTCC

>6716 len=152 tot-seq=1

TGGGGAATTTTGGACAATGGGGGCAACCCTGATCCAGCCATGCCGCGTGCAGGGTGTAGG

CCTTCGGGTTGTGAACTCCTTTTTCTCGCGAAAAAGGCACAGTTTTGGCTGTGTTGATGG

TAGTGGGGGAAGAAGCGCCGGCTAACTACGTG

>6717 len=152 tot-seq=1

TGAGGAATATTCCACAATGGGCGAAAGCCTGATGGAGCAATGCCGCGTGCAGGATGAAGG

CCTTCGGGTTGTGAACTCCTTTTTCTCGTGAAAAAGGCATGCTTTTGGGTGTGTTGATGG

TAGCGGGGGAAGAAGCGCCGGCTAACTACGTG

>6718 len=152 tot-seq=1

TGGGGAATCTTCCGCAATGGACGAAAGTCTGAACCAGCCAAGTAGCGTGCAGGATGACGG

CCCTATGGGTTGTAAACTGCTTTTATGCGGGGATAAAGTGCGCGACGTGTCGCGCATTGC

AGGTACCGCATGAATAAGGACCGGCTAATTCC

>6719 len=152 tot-seq=1

CCTTGTAGGTAATATCTGTCTCTTCATCACTGCTGTTCTAAAAGATGGCCCCAAAAGTTC

AGTCTCTGTAAAATGTGTTCTAAATTAGTGGCCCCTTAGGGATTCTGGGGCTATTTCCCC

TAGACAGAGGTCAAGGGACTGGCTGGACTGGT

>6720 len=152 tot-seq=1

ATATCATTGTGACCATGGGGCATTCAGGCTACCAAGCCCAGGAGGGATCGAGTACACAGA

CTGCGGATTGTGGCTCCCCAAGAGGAAATAGCATGCTTTACCGAAAGGAGTGACAACAGA

TGCTGAGAGTCCAAGACCAGCATGCACCTGCC

>6721 len=152 tot-seq=1

TGAGGAATATTGCGCAATGGGCGAAAGCCTGACGCAGCGACGCCGCGTGGGGGATGAAGG

TCTTCGGATTGTAAACCCCTTTCGGGAGGGAAGACGGTTGCATACAAGTGCAACGGACGG

TACCTCCAGAAGCAGCCACGGCTAACTTCGTG

>6722 len=152 tot-seq=1

TGGGGAATCTTCCGCAATGGACGAAAGTCTGACGGAGCAACGCCGCGTGGAGGGTGTAGG

CCTTCGGGTTGTGAACTCCTTTTTCTCGTGAAAAAGGCATGCTTTTGGGTGTGTTGATGG

TAGCGGGGGAAGAAGCGCCGGCTAACTACGTG

>6723 len=152 tot-seq=1

TGGGGAATATTGCGCAATGGGGGAAACCCTGACGCAGCGACGCCGCGTGGAGGGTGTAGG

CCTTCGGGTTGTGAACTCCTTTTTCTCGTGAAAAAGGCATGCTTTTGGGTGTGTTGATGG

TAGCGGGGGAAGAAGCGCCGGCTAATTCCGTG

>6724 len=152 tot-seq=1

TGGGGAATTTCGCACAATGGGCGCAAGCCTGATGCAGCGACGCCGCGTGAGGGATGGAGG

CCTTCGGGTTGTGAACCTCTTTCGCTCATGGTCAAGCCGCGGATGTGTCCGTGGTGAGGG

TAGTGGGTAAAGAAGCGCCGGCTAACTACGTG

>6725 len=152 tot-seq=1

TGGGGAATATTGCACAATGGGCGAAAGCCTGATGCAGCGACGCCGCGTGAGTGAAGAAGT

ATTTCGGTATGTAAGGCTCTATCAGCAGGGAAGAAATACTGACCTTACGGTCAGCAGACG

GTACCTGACTAAGAAGCCCCGGCTAACTACGT

>6726 len=152 tot-seq=1

AAAGTCTGATGGAGCAATACCACGTGAACGATGAAGGTCTTCTGAGGGTAAAGTTCTTTA

GTTTAGGAAAAAAAGCGCGCTAGGAAATGAGCGCGCCTTGATGGTACTGATTGAATGAGT

GACAGATAACTATGTGCCAGCAGCCTGCGGAA

>6727 len=152 tot-seq=1

TGAGGAATATTGGTCAATGGGCGGAAGTTTGATGCAGCGACGCCGCGTGGAGGGTGTAGG

CCTTCGGGTTGTGAACTCCTTTTTCTCGTGAAAAAGGCATGCTTTTGGGTGTGTTGAAGG

TAGCGGGGGAAGAAGCGCCGGCTAACTACGTG

>6728 len=153 tot-seq=1

TAGGGAATTTTCGTCAATGGGGGAAACCCTGATGCAGCGACGCCGCGTGAGTGAAGAAGT

ATTTCGGTATGTAAAGCTCTATCAGCAGGGAAGAAATAGCGACCTTTGGGTCGCCAGACG

GTACCTGATTAAGAAGCCCCGGCTAACTACGTG

>6729 len=153 tot-seq=1

TAGGGAATTTTCGTCAATGGGCGCAAGCCTGAACGAGCGATGCCGCGTGAGTGAAGAAGT

ATTTCGGTATGTAAAGCTCTATCAGCAGGGAAGAAATAGCGACCTTTGGGTCGCCAGACG

GTACCTGATTAAGAAGCCCCGGCTAACTACGTG

>6730 len=153 tot-seq=1

TGGGGAATTTTGGACAATGGGCGCAAGCCTGATCCAGCCATGCCGCGTGAGTGAAGAAGT

ATTTCGGTATGTAAAGCTCTATCAGCAGGGAAGAAATAGCGACCTTTGGGTCGCCAGACG

GTACCTGATTAAGAAGCCCCGGCTAACTACGTG

>6731 len=153 tot-seq=1

CCTTTTTAAAAGGGCAGCTCACTAAAAATACAGCTTAGCTTAAAGAAAGAAAAGACTTGA

GGGCAGAGGTTCCTATTCTCTTTATGAAAACACTGTGCATGAGAGGCCAGAGCCTCTTAG

GGTAGCCACCGCCCTAGAGTGACCAGCGCTCAT

>6732 len=153 tot-seq=1

TCCTGCCCGAATGGTGTTACTCTTCCCTTTCTTTTGTCTCAGTGCTCACGATAACGACGT

GAAAGAAGCAAACAAGCAGAGGGGACAGTCAGCTCCCCAGTCGACACAGCTGACTGTGTT

CCAGACAGCGCAGCCCTCCCGGCCGGTTCACTT

>6733 len=153 tot-seq=1

TCTTCCGCAATGGGCGAAAGCCTGACGGAGCAACGCCGCGTGAGTGATGAAGGTCTTCGG

ATCGTAAAGCACTTTCAGTGAGGAAGAACGCCTGGTGGTTAATACCCATCAGGAAAGACA

TCACTCACAGAAGAAGCACCGGCTAACTCCGTG

>6734 len=153 tot-seq=1

TAATACGGGAGGCAGCAGTGGGGAATATTGCAAAATGGGGGAAAAAGTGATGAAGAGAAG

ACGCGTGAGAGAAGAAGTATTTCGGTATGGAAAGCTCTATCAGCAGGGAAGATAATGACG

GTACCTGACTAAGAAGCCCCGGCTAACTACGTG

>6735 len=153 tot-seq=1

TAGGGAATCTTCCACAATGGACGCAAGTCTGATGGAGCAACGCCGCGTGAGTGAAGAAGG

TCTTCGGATCGTAAAACTCTGTTGTTAGAGAAGAACACGAGTGAGAGTAACTGTTCATTC

GATGACAGTACCATAGAAGAAAGCTCCGGCTAA

>6736 len=153 tot-seq=1

GAGCAGGAAATCAACATATTAAAGGAGAAAAATTAAATGATCATGGCGATATCTGCTTGT

GGCAGAAACAATGCCATGTGTTCACTAAATTATTTTTTCTCTTCTTTTTCTTTTTTTTGT

AGATACACAGTTAAATTACATTTCCCAACCTCC

>6737 len=153 tot-seq=1

TGGGGAATCTTCCGCAATGGACGAAAGTCTGACGGAGCAACGCCGCGTGAGGGATGGAGG

CCTTCGGGTTGTAAACCTCTTTCAGTAAGGGTGAAGGCTCTCTTTTGTGGGGGGTTGACA

GTACTTGCAGAAGAAGCGCCGGCTAACTACGTG

>6738 len=153 tot-seq=1

TGGGGAATTTTGCACAATGGGGGAAACCCTGATGCAGCGTGAGTGAAGAAGGTTTTCGGA

TCGTAAAGCTCTGTTGTAAGAGAAGAACGAGTGTGAGAGTGGAAAGTTCACACTGTGACG

GTATCTTATCAGAAAGGGACGGCTAACTACGTG

>6739 len=153 tot-seq=1

TTTTTTTTTCAAGCAGAAGACGGCATACGAGATGACTGAGTGACTGGAGTTCAGACGTG

TGCTCTTCCGATCGAGGCAGCAGAGACTGAAGGACAATGGGCTTTGGTGGACAACCCAGA

TATCCCTGCACAGGCAGGGTGTACACAACTACA

>6740 len=153 tot-seq=1

TGGGGAATATTGCACAATGGGCGCAAGCCTGATGCAGCGACGCCGCGTGGGGGATGAAGG

CCTTCGGGTTGTAAACTCCTTTCATCATCGACGAAGGGCTCACTTGTTGTGGGTTTGACG

GTAGGTGGAGAAGAAGCACCGGCTAACTACGTG

>6741 len=153 tot-seq=1

CGGCAGGGTCGAAGGTAATGGAGCCAGACAGGTAATGCAGCGCCAGGTTAAGCCCGGTGC

TGGCTTTGTTAAGGTCCTGTATCTCTTCGTCTGGTTCGGTACGCCCAAAGGCTTCATCAA

GGTTTGCAAAAACAATTTCTGGCACCCGCAGGC

>6742 len=153 tot-seq=1

CCTGCCCGCGGCTCTGTGGCCAACTGGGGTCCAATGGCCAAGAGCCATGGGGGATGGGGA

AGTGCTCAAGGCCAAAGGCTCAAAGGCCAACACGCCCAGGCCACAATGCAGTGCCTGGGG

GCTACAGTGCCCACGGCCACCTCACAAGGCCCT

>6743 len=153 tot-seq=1

TACAGGCTGTGTAAAATAGTGCCTGGGAATATTCAGGGGGCAGTGATGGCTGAGGGAACT

CTGCATCTCATCATTAGGAAGAAAAAGAGGCAGCCACCGTGGGAGGAAGAAGCAGTACGC

AGGAATTGGACAGAAGCCAACCGAGCACTGTCA

>6744 len=153 tot-seq=1

TAATAAGGGAGGAAGAAGTGGGGAATATTGAAAAATGGGGGAAAAAATGATGAAGAAAAG

CCGAGTGAGTGATGAAGGTTTTCGGATCATAAAGCTCTGTCGTTTGGGAAGATAATGACG

GTACCCAAGGAGGAAGCCACGGCTAACTACGTG

>6745 len=153 tot-seq=1

TGGGGAATATTGCGCAATGGGGGAAACCCTGACGCAGCGACGCCGCGTGCGGGATGGAGG

CCTTCGGGTTGTAAACCGCTTTTGTCCAAGGGCAAGGCGCGGTCTTTGGCCGTGTTGAGT

GGATTGTTCGAATAAGCACCGGCTAACTACGTG

>6746 len=153 tot-seq=1

CGTACGGATCACAAGGTCAGGAGTTCAAGACCAGCCTGGCCAGCATGGTGAAACCCCATC

TCTACTAAAATTACCAAAAAATTAGCCGGGCATGGTGGCACGCACCTGTAGTCCTAGCTA

CTCAGGAGGCTGAGGCAGGAGAACTGCTTGAAC

>6747 len=153 tot-seq=1

GTTCTAAGAGGGAAGTCAATTCAAACAAATATTTCCTGCACGCCTTGATGCTGGGCATAA

ACCAAGTAGAATTATCACATTTACAGAGCTTAATCTAGAAGAAGAGATAAATTAAAACAA

GTAAAGAGACAAATAAATAATTGCAAATTGCGT

>6748 len=153 tot-seq=1

TGGGGAATATTGCACAATGGGGGGAACCCTGATGCAGCGACGCCGCGTGAGTGAAGAAGC

ATTTCGGTGCGTAAAGCTCTGTCAGCGGGGAAGAAAGAGCATCTGAAAGGATGCCGGACG

GTACCCGACCAAGAAGCCCCGGCTAACTACGTG

>6749 len=153 tot-seq=1

CAGGCGGATCACCTAAGGTCAGGAGTTCGAGACCAGCCTGGCCAACATGGTGAAACCCTG

TCTCTACTAAAAATACAAAAATTAGCTGGGCGTGGTGGCAGGCGCCTGTAATCCCAGCTA

CTTAGGAGGCTGAGGCAGGAGACTCACTTGAAC

>6750 len=153 tot-seq=1

CCCCGCCGCCACCGTCATGGATCAGCCACAGTTCAGCGGGGCGCCCCGCTTTCTCACCCG

GCCCAAGGCCTTCGTGGTGTCGGTGGGCAAGGACGCCACCCTCAGCTGCCAGATCGTGGG

TAATCCCACGCCACAGGTGAGCTGGGAGAAGGA

>6751 len=153 tot-seq=1

TGAACCAGACAGGTCTCTGCACTCACAGAACTGATATTGCTGTGGGAGAGACAGATGATA

AATACCTCAACCAATAAACAGGGTATTCTGGATAGTGATAGGTGCCAGGAAAAACGTACA

ACAGGACAACGGCATAGAGCTGTGCCGTCCTAT

>6752 len=153 tot-seq=1

TGGGGAATATTGCACAATGGGGGAAACCCTGATGCAGCAACGCCGCGTGAGTGATGACGG

CCTTCGGGTTGTAGAGTTCTTTCGGTATTGAGGAAGGTTGATGTGTTAATAGCACATCAA

ATTGACGTTAAATACAGAAGAAGCACCGGCTAA

>6753 len=153 tot-seq=1

CACAGGGGCTCGGGCCCACGCTGGCTGTCCACACCCAGGGCCACGCCTCCATGACGGTGC

CCTCCACCACATCCCCTCTCGTGTGTGCTGGTGCCACCACGGGGGCCCAGGGACCTTTTT

GCTCTACTTCTCTTTCAAACTTGCAGCCCTGTG

>6754 len=153 tot-seq=1

TGGGGAATATTGCACAATGGGGGAAACCCTGATGCAGCGACGCCGCGTGAGTGAAGAAGT

ATTTCGGTATGTAAAGCTCTATCAGCAGGGAAGAAAACGCGTCGCAAGGCGCGCGTGACG

GTACCTGACTAAGAAGCACCGGCTAAATACGTG

>6755 len=154 tot-seq=1

GTGCTTAAACTTGGAACCACACTGCTTTCAGTACACTCAATGGATCAGATTTTACATGGT

CCCGAAAGACCTTCCTCCTAATTATAGTGAATGATGCAGCTATCACTAAACTGGATGATC

ATTACCTAAATATTTAATGCCCACAGAATTCTCC

>6756 len=154 tot-seq=1

CAACAGGGAAGAGGAGCCAGTTCCCACAGAAGTTGGCTACTTCTGCTCACAGCACATCCT

GTGTGTCTATATCCATTCCTGGCAGGAAATGGCTAAGTCCAGTGTGAAGGAAATTTGGAA

GTATATTCCTTCTCAGGCAGGAATTCTGAGGCGC

>6757 len=154 tot-seq=1

TTACGCAGACACTGACAAAAGCTTGTTGCTCGGTACCTTTTGGCATTATCAAGAAGAATA

AGCATGCTCGCGCCTTCATCATCTTGAAAGCTCTCGTAGTGATGGCGATCAGCATTGCCA

ATCAAGTAATCAAAGACAGCCGTGTCAATGATGT

>6758 len=154 tot-seq=1

CACTGGCCAGAGGGTACACACCACAGAATAAAAATGAAAATGACATGGTCACATTATGAA

GTAAAAACACAAATTTACACAAAGTTCCAAAACAAGAATTTCACACTTCAGTAGGCTCTT

TCAAATCATATTTCTAGTTACAACCCCATTACCA

>6759 len=154 tot-seq=1

GTGGAGGAAGGGGGAGTTTGGCCAGCTCTAAGGTCTTTCTAGATCATCCCATACATTGCT

GGGGACCTCCAAGGGACAGACAGTGTCTTCATCAGGTCCTTTAAGGACACAGGTTCCTGT

GCATTGATGGCATTAGTGCTGAAGCCCAATTGCC

>6760 len=154 tot-seq=1

TAAGGAATATTGGTCAATGGACGCAAGTCTGAACCAGCCATGCCGCGTGGAGGATGAAGG

CCCTCTGGGTTGTAAACTTCTTTTATGGGGGACGAAATCACTTATTCTTAAGTGTCTGAC

GGTACCCCAGGAATAAGCACCGGCTAACTCCGTG

>6761 len=154 tot-seq=1

TAGGGAATATTGGGCAATGGTCGGAAGACTGACCCAGCCACGCCGCGTGCAGGAAGAAGG

CCTTATGGGTTGTAAACTGCTTTTATCTGGGAAGAAAAAGTCCCTGCGGGGAAAATTGCC

GGTACCAGATGAATAAGCACCGGCTAACTCCGTG

>6762 len=154 tot-seq=1

TAGGGAATCTTCCACAATGGACGCAAGTCTGATGGAGCAACGCCGCGTGAGTGAAGAAGG

GTTTCGGGTTGTAAACTGCTTTTACGCGGGGATAAAGTGCGTGACGTGTCTCGCATTGCA

GGTACCGCGTGAATAAGGACCGGCTAATTCCGTG

>6763 len=154 tot-seq=1

TGGGGAATCTTCCGCAATGGACGAAAGTCTGACGGAGCAACGCCGCGTGAGTGAAGACGG

TCTTCGGATTGTAAACTGCTTTTACGCGGGGATAAAGTGCGTGACGTGTCTCGCATTGCA

GGTACCGCGTGAATAAGGACCGGCTAATTCCGTG

>6764 len=154 tot-seq=1

TAGGGAATATTGGGCAATGGCCGAGAGGCTGACCCAGCCATGCCGCGTGCAGGAAGAAGG

CCTTCTGGGTTGTAAACTGCTTTTATATAGGAAGAAAACGCCCCTGCGGGGGTAACTGAC

GGTACTATATGAATAAGCACCGGCTAACTCCGTG

>6765 len=154 tot-seq=1

TGAGGAATATTGGTCAATGGTCGAGAGACTGAACCAGCCATGCCGCGTGAAGGTGACGGC

CCTATGGGTCTTAAACTTCTTTTGTAAGGGAGCAATAAGTCTTACGCGTAGGATGATGAG

AGTACCTTACGAATAAGCATCGGCTAACTCCGTG

>6766 len=154 tot-seq=1

ATCTTCCACAATGGGCGCAAGCCTGATGGAGCAACACCGCGTGAGTGAAGAAGGGTTTCG

GCTCGTAAAGCTCTGTTGTTGGAGAAGAACGTATCTGAGAGTAACTGTTCATTCGATGAC

GGTATCTAACCAGCAAGTCACGGCTAACTACGTG

>6767 len=154 tot-seq=1

TGGGGAATCTTCCGCAATGGACGAAAGTCTGACGGAGCAACGCCGCGTGAGTGATGACGG

CCTTCGGGTTGTAAACTGCTTTTATAAGGGAATAAAGTGAGTCTCGTGAGACTTTTTGCA

TGTACCTTATGAATAAGGACCGGCTAATTCCGTG

>6768 len=154 tot-seq=1

TGAGGAATACTGGACAATGGGCGGAAGCCTGATCCAGCCATGCCGCGTGAAGGACTAAGG

CCCTACGGGTCGTAAACTTCTTTGACGCAGAGCAATAAGGTCTACGCGTAGACCGATGAG

AGTATGCGTCGAATAAGCATCGGCTAACTCCGTG

>6769 len=154 tot-seq=1

TAGGGAATCTTCCACAATGGACGCAAGTCTGATGGAGCAACGCCGCGTGAGTGAAGAAGG

TCTTCGGATCGTAAACTTCTTTTATATAAGAATAAAGTGCAGTATGTATACTGTTTTGTA

TGTATTATATGAATAAGGATCGGCTAACTCCGTG

>6770 len=154 tot-seq=1

TAGGGAATCTTCGGCAATGGACGGAAGTCTGACCGAGCAACGCCGCGTGAGTGGAGAAGG

TTTTCGGATCGTAAACTTCTTTTATATAAGAATAAAGTGCAGTATGTATACTGTTTTGTA

TGTATTATATGAATAAGGATCGGCTAACTCCGTG

>6771 len=154 tot-seq=1

TTTTTTCAAGCAGAAGACGGCATACGAGATTATAGCGTGACTGGAGTTAGACGTGTGCTC

TTCCGATCTACTACGGAGGCAGCAGTGATTAACCTTTAGCAAGAAACGAAAGTTTAACTA

AGCTATACTAACCCCAGGGTTGGTCAATTTCGTG

>6772 len=154 tot-seq=1

TGGGGAATATTGCACAATGGGCGAAAGCCTGATGCAGCGACGCCGCGTGAGCGAAGAAGT

ATTTCGGTATGTAAAGCTCTATCAGAAGGGAAGAAAGTGCGTGACGTGTCGCGCATCGCA

GGTACCGCGTGAATAAGGACCGGCTAATTCCGTG

>6773 len=154 tot-seq=1

TGAGGAATATTGGTCAATGGGCGAGAGCCTGAACCAGCCAAGTAGCGTGCAGGATGACGG

CCCTATGGGTTGTAAACTGCTTTACGCGGGGATAAAGTGAGGGACGTGTCCCTTATTGCA

TGTACCATACGAATAAGGACCGGCTAATTCCGTG

>6774 len=154 tot-seq=1

TGGGGAATTTTGGACAATGGGGGCAACCCTGAACCAGCCAAGTAGCGTGCAGGATGACGG

CCCTATGGGTTGTAAACTGCTTTTATGTGGGGATAAAGTGCGTGACGTGTCATGCATTGC

AGGTACCACATGAATAAGGACCGGCTAATTCCGT

>6775 len=154 tot-seq=1

TGGGGAATATTGGACAATGGAGGGAGCCTGAACCAGCCAAGTAGCGTGCAGGAAGACGGC

CCTATGGGTTGTAAACTGCTTTTATGCGGGGATAAAGGAGTCCACGTGTGGGCTTTTGCA

GGTACCGCATGAATAAGGACCGGCTAATTCCGTG

>6776 len=154 tot-seq=1

TGGGGAATATTGCACAATGGGGGAAACCCTGATGCAGCAACGCCGCGTGCAGGAAGACGG

CCCTATGGGTTGTAAACTGCTTTTATGCGGGGATAAAGGAGTCCACGTGTGGGCTTTTGC

AGGTACCGCATGAATAAGGACCGGCTAATTCCGT

>6777 len=154 tot-seq=1

TAGGGAATATTCCACAATGAGCGAAAGCTTGATGGAGCGACACAGCGTGCACGGTGAAGG

TCTTCGGATTGTAAACTGCTTTTATATGGGGATAAAGTGGGGAACGTGTTCCCTTTTGCA

GGTACCATATGAATAAGGACCGGCTAATTCCGTG

>6778 len=154 tot-seq=1

GAAGAGCCCGGGAGGAGGGGGTGAGGGGTCGAGGTGTGGGGCGCTGCTTGACCCAGACGC

CAGGGCCCTGGGAGGAGGATGAGGGGGACAGAGCAGATCTTGGGCGAAAGGGGAGGTTCC

CGCAGGCTCAGTAGTCGCCGCAGTTCCAGCATGG

>6779 len=154 tot-seq=1

AAACAGACGATGAAGGTTTGATAAGGGGTGGCAGGAGAAACTGAGGCAGCACCTGCCTGC

CCCCTGGCCCCAGGGCATAAAGGGCGGGGAACACAAAGAAACACGGACATAGAGTCCCTG

CTCCTGGTGGCTGGTGGAGCCTGGGAGGGAGGGG

>6780 len=154 tot-seq=1

TGAGGAATATTGGTCAATGGACGAGAGTCTGAACCAGCCAAGTAGCGTGCAGGATGACGG

CCCTATGGGTTGTAAACTGCTTTTGTTTGGGATAAAGTGCGTGACGTGTCATGCATTGCA

GGTACCACATGAATAAGGACCGGCTAATTCCGTG

>6781 len=154 tot-seq=1

CGGCATCACCTGGGAGCCTCTTAGAAAAGCTCCACCCAGAGCCACTGAATTTGCATTTCA

CAAGTTCCACATGAGGCTCACACACATATAAGCATTTGGGAATCCCTACTCTAGACTTGA

GATTTTAAGCTATTTTATCCTATTTATCCTGTCC

>6782 len=154 tot-seq=1

TAGGGAATATTGGTCAATGGATGCAAGTCTGAACCAGCCATGCCGCGTGCAGGAAGAAGG

CCCTATGGGTTGTAAACTGCTTTTGCCAGGGGATAAAATGGGCATGCGTGCCTAATTGAA

GGTACCTGGTGAATAAGCCACGGCTAACTACGTG

>6783 len=154 tot-seq=1

GGGAGAAACTCTTTGTTGCCTCAAGCGATGGGGTTGATTCGTGGCATGCACAGTGGTCTG

AGTTCTCTGCACAGCCCCAGTGAGGCAGAGGCCAAGATGGGCTGGACCAGACCAGTCAAG

CCTGCCTCCAAATCCCTCAATGGCAGGCACAAGC

>6784 len=155 tot-seq=1

TGGGGAATATTGGACAATGGGCGAAAGCCTGATCCAGCCATGCCGCGTGAGGGAAGACGG

CCCTACGGGTTGTAAACCTCTTTTGTCAGGGAGCAAAGAACGCCACGAGTGGCGTCTCGA

GAGTACCTGAAGAAAAAGCATCGGCTAACTCCGTG

>6785 len=155 tot-seq=1

TGGGGAATATTGCACAATGGGCGCAAGCCTGAACCAGCCAAGTCGCGTGAGGGAAGACGG

CCCTACGGGTTGTAAACCTCTTTTGTCAGGGAGCAAAGAACGTCACGTGTGGCGTCTCGA

GAGTACCTGAAGAAAAAGCATCGGCTAACTCCGTG

>6786 len=155 tot-seq=1

TGGGGAATATTGGTCAATGGCCGAGAGGCTGAACCAGCCAAGTCGCGTGAGGGAAGACGG

CCCTACGGGTTGTAAACCTCTTTTGTCTGGGAGCAAAGAACGCCACGAGTGGCGCCTCGA

GAGTACCTGAAGAAAAAGCATCGGCTAACTCCGTG

>6787 len=155 tot-seq=1

TGAGGAATATTGGTCAATGGTCGGGAGACTGAACCAGCCAAGCCGCGTGAGGGAGGAAGG

TACAGAGTATCGTAAACCTCTTTTGTCAGGGAACAAAGACTGGGACGCGTCCCCGGATGA

GTGTACCTGAGGAGGAAGCCACGGCTAACTACGTG

>6788 len=155 tot-seq=1

TAGGGAATTTTCGTCAATGGGCGCAAGCCTGAACGAGCGATGCCGCGTGAGAGAAGACGG

CCCTACGGGTTGTAAACCTCTTTTGTCAGGGAGCAAAGAACGCCACGAGTGGCGTCTCGA

GAGTACCTGAAGAAAAAGCATCGGCTAACTCCGTG

>6789 len=155 tot-seq=1

TCAGGCCAAATAGAATGCAGTTAGTAACAGGCCAGAGCAGAGTTTCTTCACAAGTTTTCC

TAGACCATTAGCAGGAGAATTACCTGAGAACTTGCTAGAAATGAAAATTCTCAGGGTCCA

CCTCAGACTTGCTAAATTAGAAACCCAGGATAGGC

>6790 len=155 tot-seq=1

TGGGGAATATTGCACAATGGGCGCAAGTCTGAACCAGCCATGCCGCGTGCAGGAAGACGG

CTCTATGAGTTGTAAACTGCTTTTGTACTAGGGTAAACGCTTCTACGTGTAGGAGCCTGA

AAGTATAGTACGAATAAGGATCGGCTAACTCCGTG

>6791 len=155 tot-seq=1

CATACACACACACACACACACACACACACACACACACACACAGAGAGAGAGAGAGAGGGG

GGGCAACATCGCAATATTCCCCCCAAAGGGCTTGCCCCCAATGTCTTAAATTCTCTAGGT

CTCACCTCCGAAGAGTCCCACAACTTCCCAATAGC

>6792 len=155 tot-seq=1

TGAGGAATATTGGTCAATGGGCGTAAGCCTGAACCAGCCAAGTCGCGTGAGGGATGAAGG

TTCTATGGATCGTAAACCTCTTTTATACGGGAATAAACGAATCCACGTGTGGATTTTTGC

ATGTACCGTATGAATAAGGATCGGCTAACTCCGTG

>6793 len=155 tot-seq=1

TGAGGAATATTGGTCAATGGACGAGAGTCTGAACCAGCCAAGTAGCGTGAAGGATGACTG

CCCTATGGGTTGTAAACTTCTTTTATATGGGAATAAACGAATCCACGTGTGGATTTTTGC

ATGTACCGTATAAATAAGGATCGGCTAACTCCGTG

>6794 len=155 tot-seq=1

TTCTTATACAGACCAGGGAGCCAGAATCAGCAGTAAGGGAACTCCCTCTTGGCTGTCTGC

CCTTGCCACCCTACAGCACTCCCCAAGCCATCCCTCAGTCTCTCCCACTATTCCACAGGC

ACACAATCTGTTACGTAAGGACAGAAGTTCTGAGG

>6795 len=155 tot-seq=1

TGAGGAATATTGGTCAATGGACGCAAGTCTGAACCAGCCATGCCGCGTGCAGGATGAAGG

TGCTATGCATTGTAAACTGCTTTTGTACGAGGGTAAATGCAGGTACGTGTACCTGTTTGA

AAGTATCGTACGAATAAGGGTCGGCTAACTCCGTG

>6796 len=155 tot-seq=1

TGAGGAATATTGGTCAATGGACGCAAGTCTGAACCAGCCATGCCGCGTGCAGGAAGACGG

CTCTATGAGTTGTAAACTGCTTTTGTATAAGGGTAAACCTCGGGACGTGTCCCGAGCTGC

AAGTATTATACGAATAAGGATCGGCTAACTCCGTG

>6797 len=155 tot-seq=1

TGAGGAATATTGGTCAATGGACGCAAGTCTGAACCAGCCATGCCGCGTGCAGGAAGACGG

CTCTACGAGTTGTAAACTGCTTTTGTACAAGGGTAAACCTACTTACGTGTAAGTAGTTGA

AAGTACTGTACGAATAAGGATCGGCTAACTCCGTG

>6798 len=155 tot-seq=1

TCCATTGGTTTACAGCAAGTGACGACACCTCAGGCGAGCTAACGAGTCATGGTGTCTACG

CAGTGCAGGTGGCTGGGATGTGGCTAAGTCAGAGATAATGAGAAAGTCAGGTGCAGGTCA

TAAGACCTGGCAGCTGTCCCGGGTGCCATCTTGTG

>6799 len=155 tot-seq=1

GGCTGTGTCCCAGGCAGCATAAGACTGTAACTCTTAAGCATTTGGGTCTAAGATGAGGAA

GCATGGTTCCTGAATGTGCATCTTCTTCCCGTGCCTTCACAAGCCCGGAGATGAGTGAAT

TAGAGCGGCAGCGCAGGGGCGGGAGTGTAGACTAA

>6800 len=155 tot-seq=1

TGAGGAATATTGGTCAATGGCCGAGAGGCTGAACCAGCCAAGTAGCGTGAAGGATGACGG

CCCTACGGGTTGTAAACTTCTTTTATAAGGGAATAAAGTGCACCACGCGTGGTGTTTTGT

ATGTACCTTATGAATAAGCATCGGCTAACTCCGTG

>6801 len=155 tot-seq=1

TGAGGAATATTGGTCAATGGGCGCAAGCCTGAACCAGCCATGCCGCGTGCAGGATGACGG

TCCTATGGATTGTAAACTGCTTTTGTACGGGAAGAAACACTGCTTCGTGAAGCAGCTTGA

CGGTACCGTAAGAATAAGGATCGGCTAACTCCGTG

>6802 len=155 tot-seq=1

TAGGGAATCTTGGGCAATGGGCGAGAGCCTGACCCAGCCATGCCGCGTGCAGGAAGAAGG

CTTTCTGAGTCGTAAACTGCTTTTGACAGGGAAGAATAAGCTGAACGTGTTCAGTGATGA

CGGTACCTGCAGAATAAGCACCGGCTAACTCCGTG

>6803 len=155 tot-seq=1

TGAGGAATATTGGACAATGGGTGAGAGCCTGATCCAGCCATCCCGCGTGAAGGACGACGG

CCCTATGGGTTGTAAACTTCTTTTATACTGGGATAAACCTACTTACGTGTAAGTAGCTGA

AGGTACAGTATGAATAAGCACCGGCTAACTCCGTG

>6804 len=155 tot-seq=1

AATCTTCCACAATGGACGCAATTCTGATGGAGCAACTCCGCGTGAGTGATGAAGGGTTTC

GGCTCGTAAAGCTCTGTTGTTGGTGAAGAAGGACAGGGGTAGTAACTGACCTTTGTTTGA

CGGTAATCAATTAGTCCGTCACGGCTAACTACGTG

>6805 len=155 tot-seq=1

TAAGGAATATTGGTCAATGGGCGCAAGCCTGAACCAGCCATGCCGCGTGCAGGAAGACGG

CCCTCTGGGTTGTAAACTGCTTTTGTACGGGAATAAACCTCGCTACGTGTAGCGAGCTGA

ATGTACCGTAAGAATAAGGATCGGCTAACTCCGTG

>6806 len=155 tot-seq=1

TAAGGAATATTGGTCAATGGGCGGAAGCCTGAACCAGCCACGCCGCGTGCAGGAAGAAGG

CCCTATGGGTTGTAAACTGCTTTTATACGGGAAGAACGGTCCACCTGCGGGTGGGATTGA

CGGTACCGTAGGAATAAGCATCGGCTAACTCCGTG

>6807 len=155 tot-seq=1

TGGGGAATCTTCCGCAATGGACGAAAGTCTGACGGAGCAACGCCGCGTGCAGGATGACGG

CCCTATGGGTTGTAAACTGCTTTTACGCGGGGATAAAGTGCGTGACGTGTCTCGCATTGC

AGGTACCGCGTGAATAAGGACCGGCTAATTCCGTG

>6808 len=155 tot-seq=1

TGAGGAATATTGGTCAATGGACGTAAGTCTGAACCAGCCAAGTAGCGTGCAGGATGACTG

CCCTATGGGTTGTAAACTGCTTTTATGCGGGGATAAAGTCAGCCACGTGTGGCTGTTTGC

AGGTACCGCATGAATAAGGACCGGCTAATTCCGTG

>6809 len=155 tot-seq=1

TGAGGAATATTGGTCAATGGGCGCAAGCCTGAACCAGCCAAGTAGCGTGCAGGACGACGG

CCCTATGGGTTGTAAACTGCTTTTATGCGGGGATAAAGTATGGGACGTGTCCCATTTTGT

AGGTACCGCATGAATAAGGACCGGCTAATTCCGTG

>6810 len=155 tot-seq=1

TGAGGAATATTGGTCAATGGGCGATAGCCTGAACCAGCCAAGTAGCGTGCAGGATGACGG

CCCTATGGGTTGTAAACTGCTTTTGTACGGGAATAAAGTGAGCTACGTGTAGTTTATTGC

ATGTACCGTACGAATAAGGACCGGCTAATTCCGTG

>6811 len=155 tot-seq=1

TGGGGAATATTGCGCAATGGGGGAAACCCTGACCGAGCAACGCCGCGTGAGTGATGACGG

CCTTCGGGTTGTAAAGCTCTGTTGTAAGGGAAGAACGGTAGATAGAGAATATCTAAGTGA

CGGTACCTTACCAGAAAGCCACGGCTAACTACGTG

>6812 len=155 tot-seq=1

TAGGGAATCTTCGGCAATGGGCGCTAGCCTGAACCAGCCAAGTAGCGTGCAGGACGACGG

CCCTATGGGTTGTAAACTGCTTTTATGCGGGGATAAAGTCACCCACGTGTGGGTGTTTGC

AGGTACCGCATGAATAAGGACCGGCTAATTCCGTG

>6813 len=155 tot-seq=1

AATCTTCCACAATGGACGCAAGTCTGATGGGGCAACGCCGCGTGAGTGAAGAAGGGTTTC

GGCTCGTAAAGCTCTGTTGTTGGTGAAGAAGGACAGGGGTAGTAACTGACCTTTGTTTGA

CGGTAGTCGGAGAAGAAGCCCCGGCTAACTTCGTG

>6814 len=155 tot-seq=1

TAGGGAATCTTCCACAATGGACGCAAGTCTGAACCAGCCAAGTAGCGTGCAGGATGACGG

CCCTCTGGGTTGTAAACTGCTTTTAGTTGGGAATAAAAAAGAGGACGTGTCCTCTATTGT

ATGTACCTTCAGAAAAAGGACCGGCTAATTCCGTG

>6815 len=155 tot-seq=1

TGAGGAATATTGGTCAATGGTCGGAAGACTGAACCAGCCATGCCGCGTGCAGGAAGAATG

CCTTATGGGTTGTAAACTGCTTTTATATGGGAAGAATAAGGTGTACGTGTACATTGATGA

CGGTACCATATGAATAAGCATCGGCTAACTCCGTG

>6816 len=155 tot-seq=1

TAGGGAATTTTCGTCAATGGGGGGAACCCTGAACGAGCCAAGTAGCGTGCAGGATGACGG

CCCTATGGGTTGTAAACTGCTTTTATATGGGGATAAAGTGGGGAACGTGTTCCCTTTTGC

AGGTACCATATGAATAAGGACCGGCTAATTCCGTG

>6817 len=155 tot-seq=1

TGAGGAATATTGGTCAATGGGCAAGAGCCTGAACCAGCCAAGTAACGTGAAGGATGACTG

CCCTATGGGTTGTAAACTTCTTTTATAAAGGAATAAAGTCGGGTATGCATACCCGTTTGC

ATGTACTTTATGAATACGGATCGGCTAACTCCGTG

>6818 len=155 tot-seq=1

TAGGGAATATTCCACAATGAGCGAAAGCTTGATGGAGCGACACAGCGTGCAGGATGACGG

CCCTATGGGTTGTAAACTGCTTTTATAAGGGAATAAAGTGAGTCTCGTGAGACTTTTTGC

ATGTACCTTATGAATAAGGACCGGCTAATTCCGTG

>6819 len=155 tot-seq=1

TGAGGAATATTGGTCAATGGGCGTGAGCCTGAACCAGCCAAGTAGCGTGCAGGATGACGG

CCCTATGGGTTGTAAACTGCTTTTATACGGGGATAAAAGGGTGAACGTGTTCTCCTTTGC

AGGTACCGTATGAATAAGGACCGGCTAATTCCGTG

>6820 len=155 tot-seq=1

TGAGGAATATTGGTCAATGGGCGAGAGCCTGAACCAGCCAAGTCGCGTGCAGGAAGACGG

CCCTATGGGTTGTAAACTGCTTTTTCAGCAGGGTAAAGTGATTCACGTGTGGGTCATTGC

AAGTATGCTGAGAATAAGCATCGGCTAACTCCGTG

>6821 len=155 tot-seq=1

TGAGGAATATTAGACAATGGGCGGAAGCCTGATCCAGCCATGCCGCGTGAAGGACTAAGG

CCCTACGGGTCGTAAACTTCTTTTGACGCAGAGCAATAAGATCTACGTGTGGACCGATGA

GAGTATGCGTCGAATAAGCATCGGCTAACTCCGTG

>6822 len=155 tot-seq=1

TGAGGAATATTGGTCAATGGACGAGAGTCTGAACCAGCCAAATCGCGTGAAGGAAGACTG

CCCTACGGGTTGTAAACTTCTTTTGTACAGGAATAAAAGACATTACGTGTAATGTATTGC

ATGTACTGTACGAATAAGCATCGGCTAACTCCGTG

>6823 len=155 tot-seq=1

TGAGGAATATTGGTCAATGGGCGAGAGCCTGAACCAGCCAAGTAGCGTGCAGGATGACGG

CCCTACGGGTTGTAAACTGCTTTTGCTGGAGAATAAAACGCCCCACGCGTGGGGCCTTGC

ATGTATCCAGTGAATAAGGACCGGCTAATTCCGTG

>6824 len=155 tot-seq=1

TAGGGAATATTGGGCAATGGACGAGAGTCTGACCCAGCCATGCCGCGTGCAGGAAGAAGG

CTTTCTGAGTCGTAAACTGCTTTTGACAGGGAAGAATAAGGCGTACGTGTACGCTGATGA

CGGTACCTGCAGAATAAGCACCGGCTAACTCCGTG

>6825 len=155 tot-seq=1

TGAGGAATATTGGTCAATGGACGCAAGTCTGAACCAGCCATGCCGCGTGCAGGATGACGG

CTCTACGAGTTGTAAACTGCTTTTGTATGAGGGTAAACCTAGGTACGTGTACCTAGCTGA

AAGTACCATACGAATAAGGATCGGCTAACTCCGTG

>6826 len=155 tot-seq=1

TGAGGAATATTGGACAATGGGCGAGAGCCTGATCCAGCCATGCCGCGTGCAGGAAGACGG

CCCTATGGGTTGTAAACTGCTTTTGTACGGGAAGAAACACCCCCTCGTGAGGGGGCTTGA

CGGTACCGTAAGAATAAGGATCGGCTAACTCCGTG

>6827 len=155 tot-seq=1

TGAGGAATATTGGTCAATGGGCGAGAGCCTGGACCAGCCAAGTAGCGTGCAGGATGACGG

CCCTATGGGTTGTAAACTGCTTTTATAAGGGAATAAAGTGAGCTACGTGTAGCTTTTTGC

ATGTACCTTATGAATAAGGACCGGCTAATTCCGTG

>6828 len=155 tot-seq=1

TGAGGAATATTGGTCAATGGGCGGAAGCCTGAACCAGCCATGCCGCGTGACGGAATACGG

CCCTATGGGTCTTAAACGTCTTTGGTCCCGGAGCAATAAGAGCCACGTGTGGCTCGATGA

GAGTACGGGAAGAACAAGCATCGGCTAACTCCGTG

>6829 len=155 tot-seq=1

TAGGGAATTTTCGGCAATGGGGGGAACCCTGACCGAGCAACGCCGCGTGAGTGAAGAAGG

TCTTCGGATTGTAAAACTCTGTTGTAAGGGAAGAACGGTGAGATGCATAATCTCAAGTGA

CGGTACCTTACCAGAAAGCCACGGCTAACTACGTG

>6830 len=155 tot-seq=1

AATCTTCCACAATGGACGCAAGTCTGATGGAGCAACGCCGCGTGAGTGAATAAGGGTTTC

GGCTCGTAAAGCTCTGTTGTTGGTGAAGAAGGACAGGGGTAGTAACTGACCCCCGTTTGA

CGGTAAGCAAGTAGAAAGTCACGGCTAACTACGTG

>6831 len=155 tot-seq=1

TGAGGAATATTGGTCAATGGGCGGAAGCCTGAACCAGCCAAGTCGCGTGAAGGAAGAAGG

TATTATGTATTGTAAACTTCTTTAGCAGTGGAAAAATAAGGGGGTCGTGACCCCCGATGC

TAGTACACTGAGAATAAGGATCGGCTAACTCCGTG

>6832 len=155 tot-seq=1

CAAGCCCAGAGGTAGGGGACTCCAAGGTTGGTTGATCCAGGGCTTAACGATGTCATCAAA

GACCCAGGTTCTTTCTGTCTCGGCACCTCTGTCTGCAGGGCCAGCTTCATCCTAAGCCAG

ATTGTTCTTGTCTTGATTACAAGTTGGCTGCTGGG

>6833 len=155 tot-seq=1

ATGGAGCACCTTGGCTTCCTTCCTGCAGATCCGCCCACCTCAAACTTTCTAGCAGTTTCG

TCTTCATCCTACCCACCATTGTGGGAGCTGAGGGGGACATCTTTTGGAAAAGGGCCCAGA

GTTGTTAAGTTCCTGTTTCCTTGGAGGTTTTATCT

>6834 len=155 tot-seq=1

TAAGGAATATTGGTCAATGGGGGCAACCCTGAACCAGCCATGCCGCGTGCAGGATGACTG

CCCTATGGGTTGTAAACTGCTTTTGCCGGGGAATAAACCCCTCCACGAGTGGAGGGCTGA

ATGTACCCGGAGAATAAGGATCGGCTAACTCCGTG

>6835 len=155 tot-seq=1

TAAGGAATATTGGTCAATGGAGGCAACTCTGAACCAGCCATGCCGCGTGCAGGAAGACGG

CCCTATGGGTTGTAAACTGCTTTTATGTCGGAATAAACCTATTTACGTGTAAATAGCTGA

ATGTACGGCAAGAATAAGGATCGGCTAACTCCGTG

>6836 len=155 tot-seq=1

TGAGGAATATTGGTCAATGGACGCAAGTCTGAACCAGCCAAGTAGCGTGCAGGATGACGG

CTCTAAGGGTTGTAAACTGCTTTTATATGGGGATAAAGTGGGGAACGTATTCTCCTTTGC

AGGTACCATATGAATAAGGACCGGCTAATTCCGTG

>6837 len=155 tot-seq=1

AATATTCCACAATGAGCGAAAGCTTGATGGAGCGACGCCGCGTGAGTGAAGAAGGGTTTC

GGCTCGTAAAGCTCTGTTGTTGGTGAAGAAGGACAGGGGTAGTAACTGACCTTTGTTTGA

CGGTAATCAATTAGAAAGTCACGGCTAACTACGTG

>6838 len=155 tot-seq=1

GATCTTCCACAATGGACGCAAGTCTGATGGAGCAACGCCGCGTGAGTGAAGAAAGTTTTC

GGATCGTAAAGCTCTGCTGTCGGTGAAGAAGGATAGAGGTAGTGACTGGCCTTTATTTGA

CGGTAATCAACCAGAAAGTCACGGCTAACTACGTG

>6839 len=155 tot-seq=1

TGAGGAATATTGGTCAATGGGCGAGAGCCTGAACCAGCCAAGTAGCGTGCAGGACGACTG

CCCTATGGGTTGTAAACTGCTTTTATAGGGGAATAAAGTGAGGCACGTGTGCCTTTTTGT

ATGTACCCTATGAATAAGGACCGGCTAATTCCGTG

>6840 len=155 tot-seq=1

TGAGGAATATTGGTCAATGGACGGAAGTCTGAACCAGCCAAGCAGCGTGCAGGATGACGG

CCCTATGGGTTGTAAACTGCTTTTATGTGGGAATAAAGTGAGGGACGTGTCCCTTTTTGT

AGGTACCACATGAATAAGGACCGGCTAGTTCCGTG

>6841 len=155 tot-seq=1

TGGGGAATTTTGGACAATGGGGGCAACCCTGATCCAGCCATGCCGCGTGCAGGATGACGG

CCCTATGGGTTGTAAACTGCTTTTATATGGGGATAAAGTGGGGAACGTGTTCCCTTTTGC

AGGTACCATATGAATAAGGACCGGCTAATTCCGTG

>6842 len=155 tot-seq=1

TAGGGAATCTTCCACAATGGACGCAAGTCTGATGGAGCAACGCCGCGTGAGGAAGGAAGG

TACAGAGTATCGTAAACCTCTTTTGTCAGGGAACAAAGGCGGGGACGTGTCCCCGGATGA

GTGTACCTGAAGAAAAAGCATCGGCTAACTCCGTG

>6843 len=155 tot-seq=1

TGGGGAATATTGCGCAATGGGGGAAACCCTGACGCAGCGACGCCGCGTGCAGGAAGACGG

CCCTATGGGTTGTAAACTGCTTTTATGTGGGGATAAAGTGCGCGACGTGTCATGCATTGC

AGGTACCACATGAATAAGGACCGGCTAATTCCGTG

>6844 len=155 tot-seq=1

TGGGGAATCTTCCGCAATGGACGAAAGTCTGACGGAGCAACGCCGCGTGAGGGAAGACGG

TCCTATGGATTGTAAACCTCTTTTGTCAGGGAGCAAAAACTGCCACGCGTGGCAGGATGA

GAGTACCTGAAGAAAAAGCATCGGCTAACTCCGTG

>6845 len=155 tot-seq=1

TGAGGAATATTGGTCAATGGGCGCGAGCCTGAACCAGCCAAGTCGCGTGAGGGAAGACGG

TCCTATGGATTGTAAACCTCTTTTGCCGGGGAGCAAGCTGTGCCACGTGTGGCAGGATGA

GAGTACCTGAAGAAAAAGCATCGGCTAACTCCGTG

>6846 len=155 tot-seq=1

TGAGGAATATTGGTCAATGGCCGGGAGGCTGAACCAGCCAAGTCGCGTGAGGGAAGACGG

CCCTACGGGTTGTAAACCTCTTTTGCCGGGGAGCAAAGGGCGGGATGTGTCCCGCGTCGA

GAGTACCCGGAGAAAAAGCATCGGCTAACTCCGTG

>6847 len=155 tot-seq=1

TGAGGAATATTGGTCAATGGGCGCTAGCCTGAACCAGCCAAGTAGCGTGAAGGATGAAGG

CTCTATGGGTTGTAAACTGCTTTTACGCGGGGATAAAGTGCGTGACGTGTCTCGCATTGC

AGGTACCGCGTGAATAAGGACCGGCTAATTCCGTG

>6848 len=155 tot-seq=1

TGGGGAATATTGGGCAATGGGCGAAAGCCTGACCCAGCAACGCCGCGTGAGTGATGACGG

CCCTATGGGTTGTAAACTGCTTTTATATGGGAATAAAGTGAGGGACGTGTCCCTTATTGC

ATGTACCATACGAATAAGGACCGGCTAATTCCGTG

>6849 len=155 tot-seq=1

TGAGGAATATTGGTCAATGGCCGGAAGGCTGAACCAGCCAAGCCGCGTGAGGGAAGACGG

CCCTACGGGTTGTAAACCTCTTTAGCCGGGGAACAAGGCGCGGGACGAGTCCCGCTGTGA

GCGTACCCGGAGGATAAGCATCGGCTAACTCCGTG

>6850 len=155 tot-seq=1

TGAGGAATATTGGTCAATGGACGCAAGTCTGAACCAGCCATGCCGCGTGCAGGATGACGG

CTCTATGAGTTGTAAACTGCTTTTGTATATGGGTAAATGTAGGTACGTGTACCTATTTGA

AAGTAATATACGAATAAGGATCGGCTAACTCCGTG

>6851 len=155 tot-seq=1

TGAGGAATATTGGTCAATGGGCGAGAGCCTGAACCAGCCAAGTAGCGTGCAGGACGACGG

CCCTATGGGTTGTAAACTGCTTTTATGTGGGGATAAAGTGCGTGACGTGTCATGCATTGC

AGGTACCACATGTATAAGCTCCGGCTAACTACGTG

>6852 len=155 tot-seq=1

TGAGGAATATTGGTCAATGGGCGAGAGCCTGAACCAGCCAAGTAGCGTGCAGGATGACGG

CCCTATGGGTTGTAAACTGCTTTTATGCGGGGATAAAGTGCGTGACGTGTCATGCACTGC

AGGTACCGCATGAATAAGCGCCGGCTAACTACGTG

>6853 len=155 tot-seq=1

TGAGGAATATTGGTCAATGGGCGCAAGCCTGAACCAGCCATGCCGCGTGCAGGAAGAAGC

ATCTATGGTGTGTAAACTGCTTTTATACAGGAAGAAACACCCCCACGTGTGGGGGCTTGA

CGGTACTGTAAGAATAAGGACCGGCTAACTCCGTG

>6854 len=155 tot-seq=1

TGAGGAATATTGGTCAATGGACGGAAGTCTGAACCAGCCAAGTAGCGTGCAGGATGACGG

CCCTATGGGTTGTAAACTGCTTTTGTATGGGGATAAAGTTAGGGACGTGTCCCTATTTGC

AGGTACCGTAAGAGAAAGCCACGGCTAACTACGTG

>6855 len=155 tot-seq=1

TGGGGAATCTTGCACAATGGGCGAAAGCCTGATGCAGCGACGCCGCGTGCGGGATGAAGG

CCTTCGGGTTGTAAAGCTCTGTTGTAAGGGAAGAACGGTAGATAGAGAATATCTAAGTGA

CGGTACCTTACCAGAAAGCCACGGCTAACTACGTG

>6856 len=155 tot-seq=1

TGAGGAATATTGGACAATGGGCGCAAGCCTGATCCAGCCATGCCGCGTGCAGGATGACGG

TCCTATGGATTGTAAACTGCTTTTGCACAGGAAGAAACAACTCTACGTGTAGAGTCTTGA

CGGTACTGTGAGAATAAGGATCGGCTAACTCCGTG

>6857 len=155 tot-seq=1

TGAGGAATATTGGTCAATGGGCGAGAGCCTGAACCAGCCAAGTAGCGTGAAGGATGACTG

CCCTATGGGTTGTAAACTTCTTTTATAAGGGAATAAAGTGAGGGACGTGTCCCTTTTCGT

ATGTACCTTATGAATAAGGATCGGCTAACTCCGTG

>6858 len=155 tot-seq=1

TGGGGAATATTGCACAATGGACGGAAGTTTGATGCAGCGACGCCGCGTGCAGGATGACGG

CCCTATGGGTTGTAAACTGCTTTTACGCGGGGATAAAGTGCGTGACGTGTCGCGCATCGC

AGGTACCGCGTGAATAAGGACCGGCTAATTCCGTG

>6859 len=155 tot-seq=1

TGGGGAATCTTCCGCAATGGACGAAAGTCTGAACCAGCCAAGTAGCGTGCAGGATGACGG

CCCTATGGGTTGTAAACTGCTTTTATACGGGGATAAAGTTAGGGACGTGTCCCTATTTGC

AGGTACCGTATGAATAAGGACCGGCTAATTCCGTG

>6860 len=155 tot-seq=1

TGAGGAATATTGGTCAATGGCCGGGAGGCTGAACCAGCCAAGTCGCGTGAGGGAAGACGG

TCCTATGGATTGTAAACCTCTTTTGTCAGGGAGCAAAAACTGCCACGTGTGGCAGGATGA

GAGTACCTGACTAAGAAGCCCCGGCTAACTACGTG

>6861 len=155 tot-seq=1

TGAGGAATATTGGTTAATGGACGAGAGTCTGAACCAGCCAAGTTGCGTGCAGGATGACGG

CCCTATGGGTTGTAAACTGCTTTTATGTGGGGATAAAGTGAGCCTCGTGAGGCTTTTTGC

ATGTACCTTATGAATAAGGACCGGCTAATTCCGTG

>6862 len=155 tot-seq=1

TGAGGAATATTGGTCAATGGACGAGAGTCTGAACCAGCCAAGTAGCGTGCAGGATGACGG

CCCTATGGGTTGTAAACTGCTTTTATAAGGGAATAAAGTGAGAGTCGTGACTCGTTTTGC

ATGTACCATTCGAATAAGGACCGGCTAATTCCGTG

>6863 len=155 tot-seq=1

TGAGGAATATTGGTCAATGGACGAGAGTCTGAACCAGCCAAGTAGCGTGAAGGATGACTG

CCCTATGGGTTGTAAACTTCTTTTATACGGGAATAAAGGGCGGTATGCATACCGCGTTGC

ATGTACCGTATGAATAAGGATCGGCTAACTCCGTG

>6864 len=155 tot-seq=1

TGAGGAATATTGGTCAACGGACGCAAGTCTGAACCAGCCAAGTAGCGTGCAGGATGACGG

CCCTATGGGTTGTAAACTGCTTTTATATGGGGATAAAGTGCGTGACGTGTCACGCATTGC

AGGTACCGCATGAATAAGGACCGGCTAATTCCGTG

>6865 len=155 tot-seq=1

TGAGGAATATTGGTCAATGGGCGAGAGCCTGAACCAGCCAAGTAGCGTGCAGGATGACGG

CCCTATGGGTTGTAAACTGCTTTTATAAGGGAATAAAGTGAGAGTCGTGACTCTTTTTGC

ATGTACCTTTCGAATAAGCGCCGGCTAACTACGTG

>6866 len=155 tot-seq=1

TGAGGAATATTGGTCAATGGACGCAAGTCTGAACCAGCCAAGTAGCGTGCAGGATGACGG

CCCTATGGGTTGTAAACTGCTTTTATAAAGGAATAAGGTGGGTCTCGTGAGACTTTTTGC

ATGTACCTTATGAATAAGGACCGGCTAATTCCGTG

>6867 len=155 tot-seq=1

TGAGGAATATTGGTCAATGGACGAGAGTCTGAACCAGCCAAGTAGCGTGCAGGATGACGG

CCCTATGGGTTGTAACCTGCTTTTATAAGGGAATAAAGTGAGCCTCGTGAGGCTTTTGC

ATGTACCTTATGAATAAGCATCGGCTAACTCCGTG

>6868 len=155 tot-seq=1

TGGGGAATATTGCGCAATGGGCGGAAGCCTGATCCAGCCATGCCGCGTGAAGGACTAAGG

CCCTACGGGTCGTAAACTTCTTTTGACGCAGAGCAATAAGGTCCACGTGTGGACCGATGA

GAGTATGCGTCGAATAAGCATCGGCTAACTCCGTG

>6869 len=155 tot-seq=1

TGGGGAATATTGCACAATGGGCGAAAGCCTGATGCAGCGACGCCGCGTGAGCGAAGACGG

CCCTATGGGTTGTAAACTGCTTTTATGCGGGGATAAAGGAGTCCACGTGTGGGCTTTTGC

AGGTATCGCATGAATAAGGACCGGCTAATTCCGTG

>6870 len=155 tot-seq=1

TGGGGAATATTGCACAATGGAGGGAACTCTGATGCAGCCAAGTAGCGTGCAGGAAGACGG

CCCTATGGGTTGTAAACTGCTTTTATGCGGGGATAAAGGAGTCCACGTGTGGGCTTTTGC

AGGTATCGCATGAATAAGGACCGGCTAATTCCGTG

>6871 len=155 tot-seq=1

TGAGGAATATTGGTCAATGGGCGCTAGCCTGAACCAGCCAAGTAGCGTGCAGGATGACGG

CCCTATGGGTTGTAAACTGCTTTTATGCGGGGATAAAGTCACCCACGTGTGGGTGTTTGC

AGTTACCATACGAATAAGCATCGGCTAACTCCGTG

>6872 len=155 tot-seq=1

TAGGGAATTTTTCACAATGGGCGCAAGCCTTATGAAGCAATGCCGCGTGAACGATGAAGG

TCTTATAGATTGTAAAGTTCTTTTATATGGGAATAAAGTGAGGGACGTGTCCCTTATTGC

ATGTACCATACGAATAAGGACCGGCTAATTCCGTG

>6873 len=155 tot-seq=1

TGAGGAATATTGGTCAATGGACGTGAGCCTGAACCAGCCAAGTCGCGTGAAGGAAGAAGG

TTCTATGGATTGTAAACTTCTTTTATACGGGAATAAAGTGCGCCACGTGTGGTGTTTTGT

ATGTACCGTATGAATAAGCATCGGCTAACTCCGTG

>6874 len=155 tot-seq=1

TGAGGAATATTGGTCAATGGTCGGAAGACTGAACCAGCCAAGCCGCGTGAAGGATGAAGG

TACTTAGTATCGTAAACTTCTTTTGTCAGGGAGCAACGGACGCCACGTGTGGCGTTATGA

GTTTACCTGAAGAAAAAGCATCGGCTAACTCCGTG

>6875 len=155 tot-seq=1

CAGTGGTGAAAGATCAGTGTTATGAAGTGCAGCCAGAAGGGCAGACAACAGGAAGGACGT

GAGATCTGAGATGGTTGTTGGGATGGGATGGGTTAGGGAGTTCAGGAGGGAGAGCTAGAG

AGTTTGGATCTGTCCAGGGTACTGGGAGCGAGGGA

>6876 len=155 tot-seq=1

TGAGGAATATTGGTCAATGGTCGCAAGACTGAACCAGCCATGCCGCGTGCAGGATGACGG

TCCTATGGATTGTAAACTGCTTTTGTACGGGAAGAAACACTCCTACGTGTAGGGGCTTGA

CGGTACCGTAAGAATAAGGATCGGCTAACTCCGTG

>6877 len=155 tot-seq=1

TGAGGAATATTGGTCAATGGCCGGAAGGCTGAACCAGCCAAGTAGCGTGAAGGATGAAGG

TTCTATGGATTGTAAACTTCTTTTATACGCGAATAAAACCACCTACGTGTAGGTGCTTGT

ATGTACCGTATGAATAAGCATCGGCTAACTCCGTG

>6878 len=155 tot-seq=1

TGAGGAATATTGGTCAATGGGCGAGAGCCTGAACCAGCCAAGTAGCGTGCAGGATGACGG

CCTTATGGGTTGTAAACTGCTTTTATGCGAGGATAAAGTTACCCACGTGTGGGTGTTTGC

AGGTATCGCATGAATAAGGACCGGCTAATTCCGTG

>6879 len=155 tot-seq=1

TGGGGAATCTTCCGCAATGGGCGGGAGCCTGAACCAGCCAAGTAGCGTGCAGGAAGACGG

CCCTATGGGTTGTAAACTGCTTTTATGCGGGGATAAAGGAGTCCACGTGTGGGCTTTTGC

AGGTACCGCATGAATAAGGACCGGCTAATTCCGTG

>6880 len=155 tot-seq=1

TGGGGAATATTGCGCAATGGGGGAAACCCTGACCGAGCAACGCCGCGTGAGTGATGAAGG

TCTTCGGATTGTAAAGCTCTGTTGTAAGGGAAGAACGGCATCTATAGAATAGGTGAGTGA

CGGTACCTTACCAGAAAGCTACGGCTAACTACGTG

>6881 len=155 tot-seq=1

TGGGGAATATTGGACAATGGAGGGAACTCTGATCCAGCAATTCTGTGTGTGTGATGACGG

CCCTATGGGTTGTAAACTGCTTTTATATGGGAATAAAGTGAGGGACGTGTCCCTTATTGC

ATGTACCATACGAATAAGGACCGGCTAATTCCGTG

>6882 len=155 tot-seq=1

TGAGGAATATTGGTCAATGGGCGCTAGCCTGAACCAGCCAAGTAGCGTGAAGGATGACTG

CCCTATGGGTTGTAAACTTCTTTTATACGGGAATAAAGTGAGGCACGTGTGCCTTTTTGT

ATGTACCGTATGAATATGGATCGGCTAACTCCGTG

>6883 len=155 tot-seq=1

TGGGGAATATTGCACAATGGGCGGGAGCCCGAACCAGCCAAGTAGCGTGCAGGATGACGG

CCCTATGGGTTGTAAACTGCTTTTATATGGGAATAAAGTGAGGGACGTGTCCCTTATTGC

ATGTACCATATGAATAAGGACCGGCTAACTCCGTG

>6884 len=155 tot-seq=1

TGAGGAATATTGGTCAATGGGCGCAAGCCTGAACCAGCCAAGTAGCGTGCAGGATGACGG

CCCTATGGGTTGTAAACTGCTTTCAGTCGGGAATAACTCGTCGGTCGTGACCGACGTTGC

AGGTACCGTCATAAGAAGGACCGGCTAATTCCGTG

>6885 len=155 tot-seq=1

TGAGGAATATTGGTCAATGGGCGGGAGCCTGAACCAGCCAAGTAGCGTGCAGGAAGACGG

CCCTATGGGTTGTAAACCTCTTTAGGCGGGGAGTAATGTGCTCTACGCGTAGAGTAGTGA

GAGTACCCGCAGAATAAGCATCGGCTAACTCCGTG

>6886 len=155 tot-seq=1

TGGGGAATATTGGACAATGGGCGGAAGCCTGATCCAGCAACGCCGCGTGAGGGATGACGG

CCTTCGGGTTGTAAACCTCTTTCAGTGCCGACGAAGCCTTGCCTTTTGGGGGTGGGGTGA

CGGTAGGTGCAGAAGAAGGACCGGCCAACTACGTG

>6887 len=155 tot-seq=1

TGAGGAATATTGGTAAATGGAAGAAAGTATGAAAAAGAAAAGTAGAGTGAAGGATGAAGG

AAATATGGGTTGTAAAATGATTTTATATGGGGATAAAGTGGGGAACGTGTTCCCTTTTGC

AGGTACCATATGAATAAGGACCGGCTAATTCCGTG

>6888 len=155 tot-seq=1

TGGGGAATATTGCACAATGGGGGAAACCCTGATGCAGCGACGCCGCGTGAAGGATGACTG

CCCTATGGGTTGTAAACTTCTTTTATACGGGAATAAAGTGAGGCACGTGTGCCTTTTTGT

ATGTACCGTATGAATAAGGATCGGCTAACTCCGTG

>6889 len=155 tot-seq=1

CAGCAGGGCAGGACTGCTAGGCGTCGTCACTGCAGTGGTCTGGAGCAGTCAGCAGGGGGC

GCCTGTCTCGGGCCATGCTCTCCCACTCCTTCTTCACCATGACGTACAGCCTCATTGCTG

CCTGGGCATCCTGAATCTAGACGACATGAAACATC

>6890 len=155 tot-seq=1

TGGGGAATATTGGACAATGGAGGGAACTCTGATCCAGCCAAGTAGCGTGCAGGATGACGG

CCCTATGGGTTGTAAACTGCTTTTATATGGGGATAAAGTGGGGAACGTGTTCCCTTTTGC

AGGTACCATATGAATAAGGACCGGCTAATTCCGTG

>6891 len=155 tot-seq=1

GCAGGATCCTTGACCATCACTCCCTGAGTTCAGCCTCACTGCAGCGGTCTCGGCAGAGGG

CAGCTCTCTTTCCTTCATCTGCTCAAGCCAGAACCCTGGAGTTTCCTTGATGTTTCTCTC

CCTCACACTCCATGTTCACTCCGTCCTCAGTACAG

>6892 len=155 tot-seq=1

TGAGGAATATTGGTCAATGGACGGAAGTCTGAACCAGTCAAGTAGCGTGCAGGATGACGG

CCCTATGGGTTGTAAACTGCTTTTATACGGGGATAAAGTGAGCCACGTGTGGCTTATTGC

GGGTACCGTATGAATAAGGACCGGCTAATTCCGTG

>6893 len=155 tot-seq=1

TGAGGAATATTGGTCAATGGGCGAGAGCCTGAGCCAGCCAAGTCGCGTGAAGGATGAAGG

ATCTATGGTTCGTAAACTTCTTTTATAAGGGAATAAAGTGCGGCACGTGTGCTGTTTTGT

ATGTACCTTATGAATAAGGATCGGCTAACTCCGTG

>6894 len=155 tot-seq=1

CTGGGTTAGAAACAACGGGAGGAGGGCTGGTTTCAGAGGGTATTTGGGAAGTTACAGGGC

TGCCTGTTGGCCATTCAGGTGGATGTGTGCTACTAACGAAGTCAATTTCAGCTGGAATTC

TGGAGGGAAGTCCAGCCTGGTGGTATCAATTCGGG

>6895 len=155 tot-seq=1

TGAGGAATATTGGTCAATGGGCGGAAGCCTGAACCAGCCATGCCGCGTGCAGGAAGACGG

CTCTATGAGTTGTAAACTGCTTTTATATGGGAATAAAGTTTTCCACGTGTGGAATTTTGT

ATGTACCATATGAATAAGGATCGGCTAACTCCGTG

>6896 len=155 tot-seq=1

TAGGGAATTTTCGGCAATGGGCGAAAGCCTGACCGAGCAACGCCGCGTGCAGGATGACGG

CCCTATGGGTTGTAAACTGCTTTTATATGGGGATAAAGTGGGGAACGTGTTCCCTTTTGC

AGGTACCATATGAATAAGGACCGGCTAATTCCGTG

>6897 len=155 tot-seq=1

TGGGGAATCTTCCGCAATGGACGAAAGTCTGACGGAGCAACGCCGCGTGAGTGAAGACGG

CCCTATGGGTTGTAAACTGCTTTTATGTGGGGATAAAGTGCGTGACGTGTCATGCATTGC

AGGTACCACATGAATAAGGACCGGCTAATTCCGTG

>6898 len=155 tot-seq=1

TGAGGAATATTGGTCAATGGACGAGAGTCTGAACCAGCCAAGTAGCGTGCAGGATGACGG

CCCTATGGGTTGTAAACTGCTTTTGTTTGGGAATAAGGTGAGGGACGTGTCCCTTATTGC

ATGTACCATACGAATAAGGACCGGCTAATTCCGTG

>6899 len=155 tot-seq=1

TGAGGAATATTGGTCAATGGACGCAAGTCTGAACCAGCCAAGTAGCGTGCAGGACGACGG

CCCTCCGGGTTGTAAACTGCTTTTAGTTGGGAATAAAGTGCAGCTCGTGAGCTGTTTTGT

ATGTACCATCAGAAAAAGGACCGGCTAATTCCGTG

>6900 len=155 tot-seq=1

TGAGGAATATTGGTCAATGGACGAGAGTCTGAACCAGCCAAGTAGCGTGCAGGATGACGG

CCCTATGGGTTGTAAACTGCTTTTATGCGGGGATAAAGGAGTCCACGTGTGGATTTTTGC

ATGTACCATTCGAATAAGGACCGGCTAATTCCGTG

>6901 len=155 tot-seq=1

TGAGGAATATTGGTCAATGGACGAGAGTCTGAACCAGCCAAGTAGCGTGCAGGATGACGG

CCCTATGGGTTGTAAACTGCTTTTGTTTGGGAATAATCGACATTACGTGTAGGTGTTTGC

AGTTACCATACGAATAAGCATCGGCTAACTCCGTG

>6902 len=155 tot-seq=1

TAAGGAATATTGGTCAATGGACGGAAGTCTGAACCAGCCATGCCGCGTGCAGGAAGACGG

CCCTATGGGTTGTAAACTGCTTTTATACGGGAACAAACGTCGGTACGTGTACCGGTCTGA

GTGTACCGTAAGAATAAGGATCGGCTAACTCCGTG

>6903 len=155 tot-seq=1

TGAGGAATATTGGTCAATGGACGAGAGTCTGAACCAGCCAAGTCGCGTGAAGGACGACGG

TTCTATGGATTGTAAACTTCTTTTGTGCTAGAGTAAAGTCAGGTACGTGTACCTGATTGC

AAGTATAGTACGAATAAGCATCGGCTAACTCCGTG

>6904 len=155 tot-seq=1

TGAGGAATATTGGTCAATGGGTGAGAGCCTGAACCAGCCAAGTAGCGTGCAGGATGACGG

CCCTATGGGTTGTAAACTGCTTTTACGCGGGGATAAAGTGCGTGACGTGTCTCGCATTGC

AGGTACCTTACTAAGAAGCTCCGGCTAAATACGTG

>6905 len=155 tot-seq=1

TGAGGAATATTGGACAATGGGCGGAAGCCTGATCCAGCCATGCCGCGTGCAGGATGAAGC

ATCTATGGTGTGTAAACTGCTTTTGTACGGGAAGAAAACAAGCTACGTGTAGCTTACTGA

CGGTACCGTAAGAATAAGGATCGGCTAACTCCGTG

>6906 len=155 tot-seq=1

TGAGGAATATTGGTCAATGGACGCAAGTCTGAACCAGCCATGCCGCGTGCAGGAAGACGG

CTCTATGAGTTGTAAACTGCTTTTGTACGAGGGTAAACTCGGATACGTGTATCCGTTTGA

AAGTATCGTACGAATAAGGATCGGCTAGCTCCGTG

>6907 len=155 tot-seq=1

TGAGGAATATTGGTCAATGGGTGAGAGCCTGAACCAGCCAAGTAGCGTGCAGGATGACGG

CCCTGTGGGTTGTAAACGGCTTTTACGCGGAGATAAAGTGCGTGACGTGTCCCGCATTGC

AGGTACCGCGTGAATAAGGACCGGCTAATTCCGTG

>6908 len=155 tot-seq=1

TTTTTTTTTCAAGCAGAAGACGGCATACGAGATCCAACCGTGACTGGAGTGCAGACGTGT

GCTCTTCCGATCTCCTACGGGAGGCAGCAAGGAAAATCTTGAAGAAGGGGCAGAGCTTTC

AGCTCTTAAGTGAAGTTAAAGTGGAAGATGGAGAC

>6909 len=155 tot-seq=1

TGGGGAATATTGCGCAATGGGGGAAACCCTGAACCAGCCAAGTAGCGTGAAGGATGAAGG

CTCTATGGGTCGTAAACTTCTTTTATATGGGAATAAAGTATTCCACGTGTGGAATTTTGT

ATGTACCATATGAATAAGGATCGGCTAACTCCGTG

>6910 len=155 tot-seq=1

TGGGGAATATTGCGCAATGGGGGAAACCCTGACGCAGCGACGCCGCGTGCGGGATGAAGG

TTCTATGGATCGTAAACCTCTTTTATAAGGGAATAAAGTGTGGGACGTGTCCTGTTTTGT

ATGTACCTTATGAATAAGGATCGGCTAACTCCGTG

>6911 len=155 tot-seq=1

TGGGGAATATTGCGCAATGGGGGAAACCCTGAACCAGCCAAGTAGCGTGAAGGATGACTG

CCCTATGGGTTGTAAACTTCTTTTATATGGGAATAAAACAGGGTATGCATACCCTCTTGT

ATGTACCATATGAATAAGGATCGGCTAACTCCGTG

>6912 len=155 tot-seq=1

TTTTTTTTCAAGCAGAAGACGGCATACGAGATAAGTTGGTGACTGGAGTGCAGACGTGTG

CTCTTCCGATCTCCTACGGGAGGCGCAGTGATAAATATTAAGCAATAAACGAAAGTTTGA

CTAAGTTATACCTCTTAGGGTTGGTAAATTTCGTG

>6913 len=155 tot-seq=1

TGAGGAATATTGGTCAATGGGCGTTAGCCTGAACCAGCCAAGTAGCGTGAAGGATGAATG

CTCTATGGGTTGTAAACTTCTTTTATATAAGAATAAAGTGCAGTATGTATACTGTTTTGT

ATGTATCTTATGAATAAGGATCGGCTAACTCCGTG

>6914 len=155 tot-seq=1

TGAGGAATATTGGTCAATGGTCGGGAGACTGAACCAGCCAAGCCGCGTGAGCGAGGAAGG

TACAGAGTATCGCAAACCTCTTTTGTCAGGGAACAAAGACGGGGACGCGTCCCCGGATGA

GTGTACCTGAATAAAAAGCATCGGCTAACTCCGTG

>6915 len=155 tot-seq=1

TGGGGAATATTGCGCAATGGGGGAAACCCTGACGCAGCCAAGTAGCGTGAAGGATGACTG

CCCTATGGGTTGTAAACTTCTTTTATACGGGAATAAAGTGAGGCACGTGTGCCTTTTTGT

ATGTACCGTATGAATAAGGATCGGCTAACTCCGTG

>6916 len=155 tot-seq=1

TGGGGAATATTGCGCAATGGGGGAAACCCTGACGCAGCAATTCCGCGTGTGTGAAGAAGG

CCTTCGGGTTGTAAAGCACTTTAGTGAGGGAAGAAACAGCTTGGCCACACCAAGCCTTGA

CGGTACCTCAAGAATAAGCACCGGCTAACTCTGTG

>6917 len=155 tot-seq=1

AGGGGTCACTAGGAGGTCAGAGGTCACAAGGAGGTCAAGGGTCATGGACAGGTCATTGGG

CACCCTGTGAATCAGGGGTTGATCAGACATATGACCCCAAAATGGGGAAGGGGGCACTCC

TATGGTGGGCACAGAAAGGCCCAAGGGCCCAAGAG

>6918 len=155 tot-seq=1

TGAGGAATATTGGACAATGGGTGCAAGCCTGATCCAGCCATCCCGCGTGCAGGAAGACGG

CCCTATGGGTTGTAAACTGCTTTTATACAGGAATAAACCTATTCACGTGTGAGTAGTTGA

AGGTACTGTAAGAATAAGCATCGGCTAACTCCGTG

>6919 len=155 tot-seq=1

TGAGGAATATTGGTCAATGGGCGAGAGCCTGAACCAGCCAAGTAGCGTGCAGGATGACGG

CCCTACGGGTTGTAAACTGCTTTTGCGGGAGAATAACCGGCGCCACGGGTGGCGCCCCGC

ATGTATCCCGCGAATAAGGACCGGCTAATTCCGTG

>6920 len=155 tot-seq=1

TGAGGAATATTGGTCAATGGGCGAGAGTCTGAACCAGCCAAGTAGCGTGCAGGATGAAGG

CTCTATGGGTTGTAAACTGCTTTTATATGAGAATAATCGACAGTACGTATAATGTTTTGC

ATGTACCATTCGAATAAGGACCGGCTAATTCCGTG

>6921 len=156 tot-seq=1

CAATCAGCCCTTCTGATCACCTCACGCAGAGTAGAATTTAATCCCCCGCCCTGCAGGCTC

TTCTCCATACACACTAGGCTCCCTTTTGATGTTCTTTGTATCTTCCTTTCTAACCAAGTC

GCCCTTTAGCAGCTCTGAAAGTGCCTCCTGTCACCT

>6922 len=156 tot-seq=1

TGAGGAATATTGGTCAATGGTCGGGAGACTGAACCAGCCAAGTCGCGTGAGGGAAGACTG

CCCTATGGGTTGTAAACCTCTTTTGTCAGGGAGCAATTGGCGCTACGTGTAGCGTTATCG

AGAGTACCTGAAGAAAAAGCATCGGCTAACTCCGTG

>6923 len=156 tot-seq=1

CGTCATGTACTCCCAGTCAGTCTCATCGGCTTGTTATCCTGGCATGGTTAGCAACAATAG

AAACACACAAGAATATGATCCTACAGACTACTAACAAGAGGTGGTGGTGGGTGTCACAAA

TCCGTCAAGCAAATCTCAAATTCTTACCAGTTCTTA

>6924 len=156 tot-seq=1

AATCTTCCACAATGGGCGCAAGCCTGATGGAGCAACACCGCGTGAGTGAAGAAGGGTTTC

GGCTCGTAAAACTCTGTTGTAAGAGAAGAACGTGTGTGAGAGTGGAAAGTTCACACAGTG

ACGGTAACTTACCAGAAAGGGACGGCTAACTACGTG

>6925 len=156 tot-seq=1

TGGGGAATCTTGCACAATGGGCGAAAGCCTGATGCAGCGACGCGTGAGTGAAGAAGGTTT

TCGGATCGTAAAGCTCTGTTGTTGGTGAAGAAGGATAGAGGTAGTAACTGGCCTTTATTT

GACGGTAATCAACCAGAAAGTCACGGCTAACTACGT

>6926 len=156 tot-seq=1

TGAGGAATATTGGTCAATGGGCGAGAGCCTGAACCAGCCAAGTCGCGTGAAGGAAGACTG

CCCGCAAGGGTTGTAAACTTCTTTTGTATGGGATTAAAGTCGTCTACGTGTAGACGTTTG

CAGTTACCATAAGAAAAAGCCCCGGCTAACTACGTG

>6927 len=156 tot-seq=1

TGAGGAATCTTGGTCAATGGGCGCAATGCCTGAACCAGCCAAGTAGCGTGAAGGAAGACT

GCCCTTAGGGTTGTAAACTTCTTTTATAAGGGAATAACGATAGCAACGAGTTGCTATTTG

CATGTACCTTACGAATAAGGATCGGCTAACTCCGTG

>6928 len=156 tot-seq=1

CAGTTTTCTCCAGCTGGTTGCTAAAGAGGAAGTCAGAGAAATTCAGAGCATGAGAGATTT

GCCTTGAGGGATGTTCTCTGTTGCTGAGATGGGGGGGCCATGAGGCAAGAACCTGAGAGC

AGCCTCTAGGAGCTGAGTGCCAGCCCCAGCAAACAG

>6929 len=156 tot-seq=1

TGAGGAATATTGGTCAATGGGCGGAAGCCTGAACCAGCCAAGTCGCGTGAGGGAATAAGG

CCCTATGGGTCGTAAACCTCTTTTGTCGGGGAGCAAAGGCGTCCACGTGTGGGCGCAAGG

AGAGTACCCGAAGAAAAAGCATCGGCTAACTCCGTG

>6930 len=156 tot-seq=1

TGGGGAATATTGCACAATGGGCGCAAGCCTGATGCAGCCATGCCGCGTATATGAAGAAGG

CCTTCGGGTTGTAAAGTACTTTCAGCGGGGAGGGAGTAAAGTTAATACCTTTGCTCATTG

ACGTTACCCGCAGAAGAAGCACCGGCTAACTCCGTG

>6931 len=156 tot-seq=1

GAATCTTCCACAATGGACGCAAGTCTGATGGAGCAACGCCGCGTGAGTGAAGAAGGTCTT

CGGATCGTAAAGCTCTGTTGTTAGAGAAGAACGCGAGTGGGAGTAACTGTCCTTTCGATG

ACGGTAACTAACCAGCAAGTCACGGCTAACTACGTG

>6932 len=156 tot-seq=1

TGAGGAATATTGGTCAATGGCCGGAAGGCTGAACCAGCCAAGTCGCGTGAGGGATGACGG

CCCTACGGGTTGTAAACCTCTTTTGTCGGGGAGCAAATTCCGCCACGCGTGGCGGAGTCG

AGAGTACTCGAAGAAAAAGCATCGGCTAACTCCGTG

>6933 len=156 tot-seq=1

TGAGGAATATTGGTCAATGGGCGAAAGCCTGAACCAGCCAAGTAGCGTGCAGGATGACGG

CCCTATGGGTTGTAAACTGCTTTTAGGCGGGGGTAAAGTGCCGGACGTGTTCCGGTATTG

CAAGTACCGCCAGAAAAAGGACCGGCTAATTCCGTG

>6934 len=156 tot-seq=1

GCAAAAGCCAGATCATACAAGCCTTGCCAGACCCAAATTGGAGAGCCCAGTGGGTTCGTG

ACACTGTTCTGGAGACTCTAGAAGCTACAGACATGGCTCAGACCTAGTCCCTGCCCTCCA

GCAGCAGACAGCAACCAGTGGGATATTAGGCAAGTG

>6935 len=156 tot-seq=1

CGGGAGGCAGCAGTGGGGAATATTGCGCAATGGGGGAAACCCTGCAGAAGCGACGCCGCG

TGCGGGATGAAGGCCTTCGGGTTGCAAACCGCTTTTGATTGGGAGCAAGCCTTTTGGGTG

AGGGTACCTTTCGAATAAGCGCCGGCTAACTACGTG

>6936 len=156 tot-seq=1

TAGGGAATATTGCGCAATGGGGGAAACCCTGACGCAGCAACGCCGCGTGAAGGAAGACTG

CCCGCAAGGGTTGTAAACTTCTTTTGTATGGGATTAAAGTCGTCTACGTGTAGACGTTTG

CAGTTACCATACGAATAAGCATCGGCTAACTCCGTG

>6937 len=156 tot-seq=1

TGGGGAATCTTCCGCAATGGGCGAAAGCCTGACGGAGCAACGCCGCGTGAAGGAAGACTG

CCCGCAAGGGTTGTAAACTTCTTTTGTATGGGATTAAAGTCGTCTACGTGTAGACGTTTG

CAGTTACCATACGAATAAGCATCGGCTAACTCCGTG

>6938 len=156 tot-seq=1

TGAGGAATATTGGTCGACGGACGAGAGTCTGAACCGGCCAAGTCGCGTGAAGGAAGACTG

CCCGCAAGGGTTGTAAACTTCTTTTGTATAGGATTAAAGTCGTCTACGTGTAGACGTTTG

CAGTTACCATACGAATAAGCATCGGCTAACTCCGTG

>6939 len=156 tot-seq=1

AAGGGAGGAAGAAGTGGGGAATATTGCGCAATGGGGGAAACCCTGACGCAGAGAAGAAAA

GTGAGGGATGAAGGAATTTGGGGGGGAAAAAGAGGGGGAGGGGGAGAAAGAATTAGGGTG

AGTGTACCTTTCGAATAAGCGCCGGCTAACTACGTG

>6940 len=156 tot-seq=1

TGGGGAATATTGCACAATGGGCGCAAGCCTAAACCAGTCAAGTCGCGTGAGGGAAGACGG

TCCTATGGATTGTAAACCTCTTTTGCCGGGGAGCAAGCTGTGCCACGTGTGGCGCAGCTG

AGAGTACCCGGAGAAAAAGCATCGGCTAACTCCGTG

>6941 len=156 tot-seq=1

TGGGGAATCTTCCGCAATGGACGAAAGTCTGACGGAGCAACGCCGCGTGAGTGAAGACGG

CCTTCGGGTTGTAAAGCTCTGTGATTCGGGACGAAAGGCCATATGTGAATAATATGTGGA

AATGACGGTACCGAATAAGCGCCGGCTAACTACGTG

>6942 len=156 tot-seq=1

TGGGGAATATTGGACAATGGGCGAAAGCCTGATCCAGCCATGCCGCGTGTGTGAAGAAGG

TCTTCGGATTGTAAAGCACTTTAAGTTGGGAGGAAGGGCAGTTACCTAATACGTGATTGT

TTTGACGTTAATCAATTAGAAAGTCACGGCTAACTA

>6943 len=156 tot-seq=1

TGGGGAATATTGGACAATGGGGGGAACCCTGATCCAGCGACGCCGCGTGAGGGAAGACGG

TCCTATGGATTGTAAACCTCTTTTGCCGGGGAGCAAGCTGTGTCACGTGTGACACAGCTG

AGAGTACCCGGAGAAAAAGCATCGGCTAACTCCGTG

>6944 len=156 tot-seq=1

TGGGGGATATTGGTCAATGGGCGCGAGCCTGAACCAGCCAAGTCGCGTGAGGGAAGACGG

TCCTATGGATTGTAAACCTCTTTTGCCGGGGAGCAAGCTGCGCCACGTGTGGCGCAGCTG

AGAGTACCCGGAGAAAAAGCATCGGCTAACTCCGTG

>6945 len=156 tot-seq=1

TGAGGAATATTGGTCAATGGGCGAGAGCCTGAACCAGCCAAGTCGCGTGAAGGATGACGG

CCCTACGGGTTGTAAAATTCTTTTGTCAGGGAGCAATTGAGTCCACGTGTGGGCTTAGCG

AGAGTACCTGAGTAAGAAGCCCCGGCTAACTACGTG

>6946 len=156 tot-seq=1

TGAGGAATATTGGTCAATGGAGGAAACTCTGAACCAGCCAAGTCGCGTGAAGGAAGACGG

TTCATAAGCATTGTAAACTTCTTTTGTAGAGGGGTAAAGGTGCGTACGTGTACGCATTTG

CAAGTACTCTACGAATAAGTATCGGCTAACTCCGTG

>6947 len=156 tot-seq=1

TGAGGAATATTGGTCAATGGGCGAAAGCCTGAACCAGCCAAGTCGCGTGAGGGAAGACGG

TCCTATGGATTGTAAACCTCTTTTGTCAGGGAGCAAAGCCATCCTTGCGAGGGTGGAAGG

AGAGTACCTGAAGAAAAAGCATCGGCTAACTCCGTG

>6948 len=156 tot-seq=1

TAGGGAATCTTCGGCAATGGACGGAAGTCTGACCGAGCAACGCCGCGTGAGTGAAGAAGG

TTTTCGGATTGTAAAGCACTTTAAGTTGGGAGGAAGGGCATTAACCTAATACGTTAGTGT

TTTGACGTTACCGACAGAATAAGCACCGGCTAACTC

>6949 len=156 tot-seq=1

ATAAGGGAGGAAGAAGTGGGGAATATTGAAAAATGGGAGGAAGAATGATGAAGAGAAGAA

GAGTGGGGGATGAAGGCCTTCGGGTTGTAAACTCCTTTCGACAGGGACGAAGCGTAAGTG

ACGGTACCTGTATAAGAAGCACCGGCTAACTACGTG

>6950 len=156 tot-seq=1

TAGGGAATCTTCCACAATGGGCGAGAGCCTGAACCAGCCAAGTCGCGTGAAGGAAGACTG

CCCGCAAGGGTTGTAAACTTCTTTTGTATGGGATTAAAGTCGTCTACGTGTAGACGTTTG

CAGTTACCATACGAATAAGCATCGGCTAACTCCGTG

>6951 len=156 tot-seq=1

TGGGGAATTTTGGACAATGGGGGCAACCCTGATCCAGCCATGCCGCGTGCGGGAAGAAGG

CCTTCGGGTTGTAAACCGCTTTTGTCAGGGACGAAAAGGTGCGGGTTAAGAGCTAGCACT

GATGACGGTACCTGAAGAATAAGCACCGGCTAACTA

>6952 len=156 tot-seq=1

TGAGGAATATTGGTCAATGGAGGCAACTCTGAACCAGCCATGCCGCGTGCAGGATGACGG

TCCTATGGATTGTAAACTGCTTTTATACGGGAAGAAAAAAGATCACGAGTTGGTTTACTG

ACGGTACCGTAAGAATAAGGATCGGCTAACTCCGTG

>6953 len=156 tot-seq=1

TAGGGAATCATCGGCAATGGGGGAAACCCTGACCGTGCAATGCCGCGTGAGTGATGAAGG

CCTTATGGGTTGTAAAGCTCTGTTGCGAAGGAAGAATAGCCTAGATAGAATCTAGGTCTG

ACGGTACTTCGCCAGAAAGTCACGGCTAACTACGTG

>6954 len=156 tot-seq=1

TGAGGAATATTGGTCAATGGGCGGAAGCCTGAACCAGCCAAGTCGCGTGAGGGAAGACGG

TCCTATGGATTGTAAACCTCTTTTGCAGGGGAGCAAAGCCGGCCTCGTGAGGCCGGAAGG

AGAGTACCCTGAGAAAAAGCATCGGCTAACTCCGTG

>6955 len=156 tot-seq=1

ACGGGAGGCAGCAGTGGGGAATATTGCGCAATGGGGGGAACCCTGACCGAGAAACGCCGC

GTGAACGAAGAAGTAATTCGTTATGTAAAGGTCTTTTATTGGGGAAGAAAGCGCAAGTTG

ACGGTACCCAAGGAATAAGCTCCGGCTAACTACGTG

>6956 len=156 tot-seq=1

GAATCTGTAATGAGATAGGCAGCGAAGGGGTTTGGCATTCAGTCCTGGGTGGAAGAATGG

TAAAGAAACTCTTTTCGGTATTTAAAGAGAACAGGGTAATGTTGGTAGGCATAACATTTT

CCAATAAAGATTCCTTAACAATGACGTTAGGGCCTT

>6957 len=156 tot-seq=1

CCCCAACCTTGCTTCTAGCTTTCTGAATATTGAACAGGCAAAGGCCAGCACAGACAGCAT

GAGACAGACATCTCTGACCCCATAGAGTCTCGGGCATTCACCTCCTAATGCTATGAGTAA

ACCAGCGCTGCCATCAGAACGTCTTGGCTATTGGCA

>6958 len=157 tot-seq=1

GGAGCTGCTGGCTCTGAATCTGGCTTGGGAAGGCTGTTGAATCTGGACACAGAGCTGCGC

ACCAATCCTTTAGGGATATAGGAGAGGCTCTCCCGCCGACGGATGCTGAAGCCAGCATCA

TGGTGCTCTGCATTTTCATAGTAGTGCTTAATTTTGT

>6959 len=157 tot-seq=1

TGGTCCAGTGCTTTGTGTAGGACATTTGGAGAATGAACGATAGAGCCCGCATTTAAGATG

TCGAGAGTTGGTACCAAGACCTAAACCCAAAGAAAACTATTTCATTGACTGGGGGGAGGG

GGATTCTTTAACTCCTCAAATTGTTGATTAATTTTCT

>6960 len=157 tot-seq=1

TGGGGAATATTGCACAATGGGCGAAAGCCTGATGCAGCGACGCCGCGTGAAGGATGAAGT

ATTTCGGATCGTAAAGCTCTGTTGTTAAGGAAGAACGAGTGTGAGAATGGAAAGTTCATA

CTGTGACGGTACTTAACCAGAAAGGGACGGCTAACTA

>6961 len=157 tot-seq=1

GGGACCACTAGTGCTCACACGGCAGGGAACCACTTCTACTCTTTGGCCTGTGCTTCTCAG

CAGTATCTTTAGCGTCTACTCTGCCAAAGGACACAGGAAATGCTCTCTCTGAGTCTCCCA

GCCAAGTCTTACTTTACTTGACTGGCTCAACCACCGC

>6962 len=157 tot-seq=1

GAATCTTCGGCAATGGACGGAAGTCTGACCGAGCAACGCCGCGTGAGTGAAGAAGGTTTT

CGGATCGTAAAACTAAGTTATTAAAGAAGAACGTTGGTAGGAGTGGAAAAACAACCAAGT

GACGGTAACTAACCAGAAAGGGACGGCTAACTACATG

>6963 len=157 tot-seq=1

CTGTCAATCGCATGTCCCCCAAGCCTCCATCAATGTGATGGCCAGGGACTTGTGTGGGTC

ACAGACTCAAACCTGAACTTCTGCCGAAGATATCAAGAAGGAGAAGCCCTCTATTTACTG

AAGTCTCTGACTGTAATAACTGAGTGAATTGATATCT

>6964 len=157 tot-seq=1

GTCAGAAAAACCCGGAAGGAAGGAAGCTGGACAGACAGACAGTAGCTGATCCTAGCTTCA

TTGCTCCAGAGAGACACCCTCCAGACTCCTAGACTTCAAGGGACACTATCCTTCTCAGAA

GGAGAGAGCAGCCCTCTGTCTCTTCTGAAACCAAATT

>6965 len=157 tot-seq=1

GAATCTTCCACAATGGACGCAAGTCTGATGGAGCAACGCCGCGTGAGTGAAGAAGGGTTT

CGGCTCGTAAAGCTCTGTGATTCGGGACGAAAGATAAGCAGACGAATAATCTGCATAAGT

GACGGTACCGAAAAAGCAAGCCACGGCTAACTACGTG

>6966 len=157 tot-seq=1

TGGGGAATATTGCACAATGGGGGAAACCCTGATGCAGCGACGCCGCGTGGAGGAAGAAGG

TCTTCGGATTGTAAACTCCTGTTGGTGAAGAAGGACAGGGGTAGTAACTGACCTTTGTTT

GACGGTAATCAATTAGAAAGTCACGGCTAACTACGTG

>6967 len=157 tot-seq=1

ATGCCCAGGGAGACCCAAGAAAGAGTCAGGTTAGGGAGCAGTGAAAGTGAGGAGGGAAGA

CAATTCTGTGAACTCTGTAACTCTTAAAATTTTTGAAAACTCCATCGTTAAACAACTTTT

AAAAGAAATAACTAAATTTTCAAATGAGTAAGCAGTG

>6968 len=157 tot-seq=1

CTCCGGTCCCACCCTTCAGGGTTCTCTCAAGGCCAGCCCTGGGCCAGGTGGGAGCCACCC

TGACCCTTAACCCTGTGACACTGCACCTATCCAAGACCAGGTCCTGTGAAGCCACAAGGG

CAGGGGCTCCTGTGCCTGATGAAACCCGTAACCCTCC

>6969 len=157 tot-seq=1

AGGCGGAGCTTGTAGTGAGCCAAGATCGTGCCACTGCACTCCAGCCTGGGTGACAGCGAG

ACTCTGTCTCAAAAAAAAAAGAAAAAAGAAAAAAAGAAAGAAAAGAAATAAAAATAAATA

AAAAGATGGCCAGGCGCAGTGGCTCACACCTTTAATC

>6970 len=157 tot-seq=1

CGTCCCCTCCTCTCCCCAGCCGCAAGTGCTGGGACACTCGGTAGTAGAAACCGGCCATGG

TGCCTGTATTTACCCCATAGGCATCCGCAAATGCTATAAACCAGGGCTTCCCCTCCCCAC

CCCCACTCCACTCTGAGCACTGGTTGTTAAATATTCA

>6971 len=157 tot-seq=1

AGGGAGAGGACGCCTATGAATCTGAACACACCCACCATGCTGGAACACTTGACCGCCCAC

CCAGACTCCGCAAATCACAGGCCCAGCCTGAGGCCGTAGCTGGGGACCGATCCCATTAGT

AGGAGGCCACGCTGAGAGCCAGGTTTGGCCCCTGGAG

>6972 len=157 tot-seq=1

TAGGTTGTTTCACTGAGAATTATGAACAAGATAGTTATCCCTGGAGAGCTTATCTAGGAA

GGGGTATCCTGGACAGGGCTGAAATTTCTGCTAGTAAAATGATTGTAGGCTCAATGTATC

CAAGAGACTTCATTAGCTCTCCTCATTTGCTGGTATT

>6973 len=157 tot-seq=1

AAGGAGACATCAAATGGTGCAGCCTGCAGTTCCCAGCAGTGACTCTCAATGGGACTGCAT

GCATTACCATGGTTCCTGCAGGAACTACTGCCCAGCTGATTGTCAGTGGAAGTGTGGACA

CAGGCTATCCTCATTTCTCCTACCCTTATCTGGTTTC

>6974 len=157 tot-seq=1

GAATCTTCCACAATGGACGAAAGTCTGATGGAGCAACGCCGCGTGAGTGAAGAAGGGTTG

CGGCTCGTAAAGCTCTGTTGTAAGAGAAGAACGTGTGTGAGAGTGGAAAGTTCACACAGT

GACTGTAACTTTCCAGAAAGGGACGGCTAACTACGTG

>6975 len=158 tot-seq=1

TAGGGAATTTTCGTCAATGGAGGGAACTCTGAACGAGCAATGCCGCGTGAGTGAAGAAGG

CCTTCGGGTCGTAAAGCTCTGTTGCGGGGGAAAAAAGGCAGCATCAGGAAATGGGTGCTG

ACTGATGGTGCCCCGCCAGAAAGTCACGGCTAACTACG

>6976 len=158 tot-seq=1

TGGGGAATATTGCACAATGGGCGAAAGCCTGATGCAGCAACGCCGCGTGAACGATGAAGG

CCCTCGGGTCGTAAAGTTCTGTTGTAAGGGAAGAACAAGTGCCGCAGGCAATGGCGGCAC

CTTGACGGTACCTTGCGAGAAAGCCACGGCTAACTACG

>6977 len=158 tot-seq=1

TTCAGACTCTGCAGTGAGGAAGAGCAGGGCAGGGTTATTCCTGTCCAGGCCTCAGTGGGA

TCTTATAAACTTGGACTCCTCAGGGATAAGGTGCTCAGAGAGCCAAGTGCACCCACATGA

GGAGGTCTACATGGAGTACTCACAAGGCAAAGCTTGCC

>6978 len=158 tot-seq=1

TAGGGAATCTTCCGCAATGGGCGAAAGCCTGACGGAGCAACGCCGCGTGAGTGATGAAGG

TCTTTGGATCGTAAAACTCTGTTGTTAGAGAAGAACAAGGATGAGAGTAACTGTTCATCC

CTTGACGGTATCTAACCAGAAAGCCACGGCTAACTACG

>6979 len=158 tot-seq=1

TGGGGAATTTTGGACAATGGGCGCAAGCCTGATCCAGCAATGCCGCGTGTGAAGAAGGCC

TTCGGGTTGTAAAGCACTTTTGTCCGGAAAGAACACCTCCTGGTTAATACCCGGGGGGGA

TGACGGTACCGAAAGAATAAGCACCGGCTAACTACGTG

>6980 len=158 tot-seq=1

GAAGCAACGCGAAGAACCTTACCAAGTCTTGACAGCCCTCTGACCGGAACTTAACCGTTC

CTTTCCTTCGGGACAGAGGAGACAGGTGGTGCATGGTTGTCGTCAGCTCGTGTCGTGAGA

TGTTGGGTTAAGTCCCGCAACGAGCGCAACCCCTATCC

>6981 len=158 tot-seq=1

TGGGGAATATTGCACAATGGGCGCAAGCCTGATGCAGCCATGCCGCGAGTGATGAAGGTC

TTCGGATCGTAAAACTCTGTTATTAGGGAAGAACAAATGTGTAAGTAACTATGCACGTCT

TGACGGTACCTAATCAGAAAGCCACGGCTAACTACGTG

>6982 len=158 tot-seq=1

TGGGGAATATTGCACAATGGGCGAAAGCCTGATGCAGCGACGCGTGAGTGAAGACGGCCT

TCGGGTTGTAAAACTCTGTGATTCGGGACGAAAGATAAGCAGACGAATAATCTGCATAAG

TGACGGTACCGAAAAAGCAAGCCACGGCTAACTACGTG

>6983 len=158 tot-seq=1

GGGAATTTTGCACAATGGGGGAAACCCTGATGCAGCGACGCCGCGTGAGTGAAGAAGGTT

TTCGGATCGTAAAGCTCTGTTGTTAGAGAAGAAAGTGCGTGAGAGTAACTGGTCACGTTT

CGACGGTATCTAACCAGAAAGCCACGGCTAACTACATG

>6984 len=158 tot-seq=1

TGGGGAATATTGGACAATGGGGGGAACCCTGATCCAGCCATGCCGCGTGTGTGAAGAAGG

CCTTTTGGTTGTAAAGCACTTTAAGCGAGGAGGCTACCGAGATTAATACTCTTGGATAGT

GGACGTTACTCGCAGAATAAGCACCGGCTAACTCTGTG

>6985 len=158 tot-seq=1

TGGGGAATCTTGCACAATGGGCGAAAGCCTGATGCAGCGACGCGTGAGTGATGACGGTCT

TCGGATTGTAAAGCTCTGTTAATCGGGACGAATGGTCGGTGTGCAAATAGTGCATGGACA

TGACGGTACCGGAATAGAAAGCCACGGCTAACTACGTG

>6986 len=158 tot-seq=1

TGAGGAATATTGGTCAATGGACGCAAGTCTGAACCAGCCATGCCGCGTGCAGGATGAAGG

TCTTCGGATTGTAAACTGCTTTTGTCAGGGAAGAAAAGGTCTGTGTTAATACCATAGACT

GCTGACGGTACCTGAAGAATAAGCACCGGCTAACTACG

>6987 len=158 tot-seq=1

TGGGGAATCTTGCACAATGGGCGAAAGTCTGACCGAGCAACGCCGCGTGAGTGAAGAAGG

TTTTCGGATCGTAAGGCTCTGTTGTAAGAGAAGAACGAGTGTGAGAGTGGAAAGTTCACA

CTGTGACGGTATCTTACCAGAAAGGGACGGCTAACTAC

>6988 len=158 tot-seq=1

TAGGGAGTATTCGGCAATGGGCGCAAGCCTGACCGAGCAACGCCGCGTGAATGATGAAGA

TCTTCGGATTGTAAAATTCTGTGATTAGAGAAGAATGACGTAGTGAGGAAATGCATTACG

TTTGACGGTATCTATTTAGAAAGCCACGGCTAACTACG

>6989 len=158 tot-seq=1

CGGGGAATTTTGGACAATGGGCGAAAGCCTGATCCAGCAATGCCGCGTGAGTGAAGAAGG

CCTTCGGGTTATAAAGCTCTTTTGTCAGGGAAGAAACGGTGAAGGCTAATATCCTTCGCT

AATGACGGTACCTGAAGAATAAGCACCGGCTAACTACG

>6990 len=158 tot-seq=1

GATCCCCTCCTCTCCTTCCATCTACACCCAGGCTCTCCCAGCCTCTCCTCTAGTGGCCTG

CTGCTGTGCAGGACACGGTCCTGTCTCATCTGCACGGTGGCCTCAGCCTCCTAAAGGGTC

TCCCTGCCCCTCTATGTCCTGTGCTGGCCACGTTCCAC

>6991 len=158 tot-seq=1

TTTTTTTTTCAAGCAGAAGACGGCATACGAGATACCTCAGTGACTGGAGTCAGACGTGTG

CTCTTCCGATCTCCTACGGGAGGCAACAGATAAGGAGAAGGCTCGTCCTCCAGAACAGAG

GCAGCAGCTCCAGCAGCCCCAGGGGGCTCTAGCCACAG

>6992 len=158 tot-seq=1

TTTTTCAAGCAGAAGACGGCATACGAGATACCTCAGTGACTGGAGTGCAGACGTGTGCTC

TTCCGATCTCCTAAGGGAGGCAGCAGTAGGCCTTTCTAACAAACAAGCACGTACACCCCC

TGAGTCTGTAAAATACCAAAACAATGACGACAGCACCA

>6993 len=158 tot-seq=1

TGGGGAATATTGCACAATGGGCGAAAGCCTGATGCAGCAACGCCGCGTGAGCGATGAAGG

CCTTCTGATTGTAAAGTTCTTTTATTTAGGAAAAAAAGCGCGCTAGGAAATGAGCGCGCC

TTGATGGTACTAATTGAATAAGTGACAGCTAACTATGT

>6994 len=158 tot-seq=1

CGTCTTCAACAGCAATGGTTGCAGCTGGTTCGCACATAGATCGTCAAGAATTTTGGCTAC

CCGAAAACGCAGAAACGATTTGTACTCCAGCGCCTGCACGGGCCACTCGGCAACCTGCTC

AAGAAACTGTTTGGTCGTCTGTTCGGTGAAGGTGAGTT

>6995 len=158 tot-seq=1

TCCCCTCCCGTGCAGCATGTCTAACACAGAACTCAGAATCGTCCCCAAACCAACTCTCCC

CTCTGCGTTTTCCTTCTTGTTGTTGGCACCATAATTATCTGCGTTTCGGGCTCAGAAATG

GAGCGCTCTTCACTTCCCCTCCCCACCATTCCCTCCTC

>6996 len=158 tot-seq=1

TAGGGAATTTTCGGCAATGGGGGAAACCCTGACCGAGCAACGCCGCGTGAGTGAAGAAGG

TTTTCGGATCGTAAAACTCTGTTGTTAGAGAAGAACAAGGATGAGAGTAACTGTTCATCC

CTTGACGGTATCTAACCAGAAAGCCACGGCTAACTACG

>6997 len=158 tot-seq=1

TTGTGGGGTAGGAGGAGGGGAGGGACTCTGGGAATTGTGGTCGTTCACTTAACTCTTAGT

GTGAATAAGGATTCTGAGGCTTATGCAGGTCAGGAAAAGGGGAGAGAGGATCAGGTATGT

GGTCTTCTGCGGGGTAGGAATGGCTGGGAGAAGGGAGA

>6998 len=158 tot-seq=1

TGGGGAATATTGCGCAATGGGGGAAACCCTGACGCAGCGACGCGTGAGTGAAGAAGGTTT

TCGGATCGTAAAGCTCTGTTGTTAGAGAAGAACGTTGGTAGGAGTGGAAAATCTACCAAG

TGACGGTAACTAACCAGAAAGGGACGGCTAACTACGTG

>6999 len=158 tot-seq=1

TTTTTTTTTTCAAGCAGAAGACGGCATACGAGATAAGTTGGTGACTGGAGTTCAGACGTG

TGCTCTTCCGATCCCCTACGTGGGGCAGCAGTGACAAAAATTAAGCCATAAACGAAAGTT

TGACTAAGTTATATTAATTAGGGTTGGTAAATCTCGTG

>7000 len=158 tot-seq=1

ACAGACACCTCATACAGGAGAGCTCTGGCTGGCATCTGGTGGGTGCCCCTCTAGGACGAA

GCTTCCAGAGGAGGGAACAGGCAGCAATTACTGCTATTCTGCAACTTCTGCTGGTGATAC

CCAGGCAAACAGGGTCTAGAGTGGACCTCCAGCAAACT

>7001 len=159 tot-seq=1

TGGGGAATTTTGCACAATGGGGGAAACCCTGATGCAGCGACGCCGCGTGATTTAGAAGGC

CTTCGGGTTGTAAAGGACTTTTGTCAGGGAAGAAAAGGCTGTTGCTAATACCAACAGCTG

ATGACGGTACCTGAAGAATAAGCACCGGCTAACTACGTG

>7002 len=159 tot-seq=1

CAGGTGGATTTCTGTGAGTCAGTCCAGCCTGGTCTACAGCGTGAGTCCCAAGACAGCCAG

AGCTACATTGAAAGACCCTGTCTGGGGTGGGGGAAAAAAAAGTGTTGATTTACTTCATAT

AAATTGCTTTTAGGTTTTGCATTCCTTAGTCTTATAAAC

>7003 len=159 tot-seq=1

TGGGGAATTTTGCACAATGGGGGAAACCCTGATGCAGCGACGCCGCGTGATTTAGAAGGC

CTTCGGGCTGTAAAAATCTTTTGTATGGGAAGAAGGACAGGGGTAGTAACTGACCTTTGT

TTGACGGTAATCAATTAGAAAGTCACGGCTAACTACGTG

>7004 len=159 tot-seq=1

TAGGGAATCTTCCACAATGGGCGAAAGCCTGATGGAGCAACGCCGCGTGGGTAAAGAAGG

TCTTCGGATCGTAAAACCCTGTTGTTAGAGAGGAAAGTGCATAGAGTAACTGTTATGCGT

GTGACGGTATCTAACCAGAAAGCCACGGCTAACTACGTG

>7005 len=159 tot-seq=1

TGGGGAATTTTGCACAATGGGGGAAACCCTGATGCAGCGACGCCGCGTGATTTAGAAGGC

CTTCGGATCGTAAAGCTCTGTTGTTAGAGAAGAACGTTGGTAGGAGTGGAAAATCTACCA

AGTGACGGTAACTAACCAGAAAGGGACGGCTAACTACGT

>7006 len=159 tot-seq=1

TAGGGAATCTTCCACAATGGGCGCAAGCCTGATGGAGCAACGCCGCGTGAGTGAGGAAGG

CCCTCGGGTCGTAAAGCTCTGTTGCCAGGGAAGAATAAGCATATGTTAACTGCATATGCG

ATGACGGTACCTGAGAAGAAAGCCCCGGCTAACTACGTG

>7007 len=159 tot-seq=1

TGGGGAATTTTGCACAATGGGGGAAACCCTGATGCAGCGACGCCGCGTGATTTAGAAGGC

CTTCGGGTTGTAAAGTGCTGTTATAAGGGAAGAACATTTGCAATAGGAAATGATTGCAGA

CTGACGGTACCTTGTCAGAAAGCGATGGCTAACTATGTG

>7008 len=159 tot-seq=1

TGGGGAATATTGCACAATGGGCGAAAGCCTGATGCAGCGACGCCGCGTGAGGGATGACGG

CCCTCGGGTTGTAAAGCACTTTCAGTGAGGAAGAACGCCTGGTGGTTAATACCCATCAGG

AAAGACATCACTCAAGAAGAAGCACCGGCTAACTCCGTG

>7009 len=159 tot-seq=1

TCGGGAATTTTGGGCAATGGGCGAAAGCCTGACCCAGCAACGCCGCGTGAAGGATGAAGT

TTTTCGGATCGTAAAACTCTGTTATTAGGGAAGAACAAATGTGTAAGTAACTATGCACGT

CTTGACGGTACCTAATCAGAAAGCCACGGCTAACTACGT

>7010 len=159 tot-seq=1

TAGGGAATCTTCCACAATGGGCGCAAGCCTGATGGAGCAACACCGCGTGAGTGAAGAAGG

GTTTCGGCTCGTAAGCTCTGTTGTTGGTGAAGAAGGACAGGGGTAGTAACTGACCTTTGT

TTGAGGGTAATCAATTAGAAAGTCATGGCTAACTACGTG

>7011 len=159 tot-seq=1

TGGGGAATTGTTCGCAATGGGCGCAAGCCTGACGACGCAACGCCGCGTAGAGGATGAAGG

TCTTCGGATTGTAAACTCCTGTTGATCGGGACGAAAAGCTTCGACCTAACACGTCGGAGA

CTGACGGTACCGGTTGAGGAAGCCACGGCTAACTCTGTG

>7012 len=159 tot-seq=1

TGGGGAATTTTGCACAATGGGGGAAACCCTGATGCAGCGACGCCGCGTGATTTAGAAGGC

CTTCGGGTTGTAAAGTACTTTCAGCGGGGAGGAAGGGAGTAAAGTTAATACCTTTGCTCA

TTGACGTTACCCGCAGAAGAAGCACCGGCCAACTCCGTG

>7013 len=159 tot-seq=1

TGGGGAATTGTTCACAATGGGCGCAAGCCTGATGACGCAACGCCGCGTGGAGGATGAAGG

TCTTCGGATTGTAAACTCCTGTTGCTCGGGACGAAATGCTCCGACCTAATACGTCGGAGT

TTGACGGTACCGAGTGAGGAAGCCCCGGCTAACTCTGTG

>7014 len=159 tot-seq=1

TAGGGAATCTTCCACAATGGGCGCAAGCCTGATGGAGCAACGCCGCGTGAGTGAGGAAGG

CTTTCGGGTCGTAAAGCTCTGTTGCCAGGGAAGAATAAGGATGTGTTAACTGCACATTCG

ATGACGGTACCTGAGAAGAAAGCCCCGGCTAACTACGTG

>7015 len=159 tot-seq=1

TGGGGAATATTGGACAATGGGCGCAAGCCTGATCCAGCCATGCCGCGTGAGTGATGAAGG

CCTTCGGGTCGTAAAGCTCTGTGGGGAGGGACGAACCGCTTTTGGTGCATAGCCAAGAGC

ATGACGGTACCTCCTTAGCAAGCACCGGCTAACTTCGTG

>7016 len=159 tot-seq=1

TGGGGAATATTGCGCAATGGGGGAAACCCTGATGGAGCAATACCACGTGAACGATGAAGG

TCTTCTGATTGTAAAGTTCTTTTATTTAGGAAAAAAAGCGTGCTAGGAAATGAGCGCGCC

TTGATGGTACTAATTGAATAAGTGACAGCTAACTATGTG

>7017 len=159 tot-seq=1

GAGAGAAAATCTCTGCTCTTCTAACTGCAGTCCCTCTCCATGCATTGCCTGGCCTCAGGA

TTGCTCTCTGGGTGATCTTGGGAGAGAGCTGGGGTCTTTTTACTAGGTACATTGTAGCTC

CATTAGGATATTCTAGAATGTTTTACTAGTTTTCTCATT

>7018 len=159 tot-seq=1

TGGGGAATTGTTCACAATGGGCGCAAGCCTGATGACGCAACGCCGCGTGGAGGATGAAGG

TCTTCGGATTGTAAACTCCTGTTGCCCGGGACGAAAAGCTTCGACCTAACACGTCGAGGT

CTGACGGTACCGGGTGAGGAAGCCCCGGCTAACTCTGTG

>7019 len=159 tot-seq=1

TGGGGAATTTTGGACAATGGGGGCAACCCTGATCCAGCAATGCCGCGTGAGTGAAGAAGG

CCTTCGGGTTGTAAAGCTCTTTTGTCAGGGAAGAAACACCGGCTCTAACACAGTCCGGGA

ATGACGGTACCTGAAGAATAAGCACCGGCTAACTACGTG

>7020 len=159 tot-seq=1

TGGGGAATCTTGCGCAATGGGCGAAAGCCTGACGCAGCGACGCCGCGTGAGTGATGAAGG

CCTTCGGGTTGTAAAGCTCTGTGGGGAGAGACGAATAAGTGCCGGCTAATACCTTGCATG

ATGACGGTATCTCCTTAGCAAGCACCGGCTAACTCTGTG

>7021 len=159 tot-seq=1

TAGGGAATCTTCCACAATGGACGAAAGTCTGATGGAGCAATGCCGCGTGAGTGATGAAGG

ATTTCGGTTCGTAAAACTCTGTTGCGAGGGAAGAACAAGTGTAAAGTAACTACTTACACC

TTGACGGTACCTCGTTAGAAAGCCACGGCTAACTACGTG

>7022 len=159 tot-seq=1

ACGTAAAGGCGGAGAGGCGTGAGCAAGCCCGGTGGGGCAGGGGTTAGCAGTGGAATTCGT

CAAGGACCGCTTCTCAGAGAGGGCTAGTAGTGAGCCTGACTTGGGAGCAGTGGCTGGACA

TTTGAGGGAATGACACGAACCAAGGCACAGAGAGAGGAA

>7023 len=159 tot-seq=1

TAGGGAATCTTCCGCAATGGGCGAAAGTCTGACGGAGCAACGCCGCGTGAGTGAAGAAGG

TTTTCGGATCATAAAACTCTGTTGTGAGGGAAGAACAAGTACCAGCTAACTACTGGTACC

TTGACGGTACCTCACCAGAAAGCCACGGCTAACTACGTG

>7024 len=159 tot-seq=1

TAGGGAATCTTCGGCAATGGACGGAAGTCTGACCGAGCAACGCCGCGTGAGTGAAGAAGG

TTTTCGGATCGTAAAGCTCTGTTGTCGGGGACGAACGTGGAGACGGCGAATAGCCGTTTC

CAATGACGGTACCTGACGAGGAAGCCACGGCTAACTACG

>7025 len=159 tot-seq=1

GAAGTGAGGAATATGGGTAAATGGGAGAGATAATGAAAAAGAAAAGCCGCGTGAGGGAAG

ACGGCCCTATGGGTTGGAAACCTCTTTTGTCGGAGAACAAAACCCGGGACGTGTCCCGGA

CTGCGTGTATCCGAAGAAAAAGCATCGGCTAACTCCGTG

>7026 len=159 tot-seq=1

TAGGGAATCTTCGGCAATGGACGAAAGTCTGACCGAGCAACGCCGCGTGAGTGAAGAAGG

TTTTCGGATCGTAAAACTCTGTTGTCAGAGAAGAACAAGGATAAGAGTAACTGCTTATCC

CCTGACGGTATCTGACCAGAAAGCCACGGCTAACTACGT

>7027 len=159 tot-seq=1

TGGGGAATCTTGCGCAATGGGCGAAAGCCTGACGCAGCGACGCCGCGTGAGCGATGAAGG

CCTTCGGGTTGTAAAGCTCTGTGGGGAGGAAAGAATAAGGGTTGGCTAATATCCAGCTCG

ATGACGGTACCTCCTTAGCAAGCACCGGCTAACTCTGTG

>7028 len=159 tot-seq=1

TGGGGAATATTGCACAATGGGGGAAACCCTGATGCAGCAACGCCGCGTGAGTGATGAAGG

CCTTCGGGTTGTAAAGTACTTTCAGTCGTGAGGAAGGGGGTATCGTTAATAGCGGTATTC

TTTGACGTTAGCGACAGAAGAAGCACCGGCTAACTCCGT

>7029 len=159 tot-seq=1

ATCACTAACTCTGAAGCTCGCGCTCGGTGGCTTCCTGAAATTTCCCCTTCCAACTTCCCA

CCAGTCAGCCTACTCTCCACCTCCCTGAGAAGCAAGCTCAGCAGCCCATCTCTTGAGAAG

GTGACTTGAAAGTGGGAGAAAGTCAAGAAAGAGTCTAGG

>7030 len=159 tot-seq=1

CTGACATGAAGCTGGGGTGTCTTCTGCCTGCTGAGAGTTGGGGAGGTGGCAAGGTCAGAG

GCTTGAGGGAAGTGAAGTGGGCCTCACTCAACTGGAGCCTGGCCTGCTCTGGGACAAATT

GTTCAGGTTCCAAACTGACTCAGAGAACATTCCCCACCT

>7031 len=159 tot-seq=1

TGGGGAATTTTGCACAATGGGGGAAACCCTGATGCAGCGACGCCGCGTGATTTAGAAGGG

TTTCGGCTCGTAAAGCTCTGTTGTTGGTGAAGAAGGACAGGGGTAGTAACTGACCTTTGT

TTGACGGTAATCAATTAGAAAGTCACGGCTAACTACGTG

>7032 len=159 tot-seq=1

GACGGCAGGGACAGAGCTCAGGAGGCACGCAGCGTTCCTCCTAGCAGAGGCCAGGGACAA

AGGGCTCCATGGAGGACTGCCTGGTGCCCCCTCCCTGCCAGTTACACCCAGCCAGGGACC

AGGGCAGGGCACTGGCTAGCGGAGCCCCTCCCAGACATA

>7033 len=159 tot-seq=1

GGGGAATTTTGGACAATGGGGGCAACCCTGATCCAGCCATGCCGCGTGCATGATGAAGGT

CTTCGGATTGTAAACTACTTTTATCAGGGAAGAAAAGGTCTATGTTAATACCATAGTCTG

CTGACGTTACCTGAAGAATAAGCACCGGCTAACTACGTG

>7034 len=159 tot-seq=1

TGAGGAATATTGGTCAATGGGCGCTAGCCTGAACCAGCCAAGTAGCGTGCAGGATGACGG

CCCTATGGGTTGTAAAGCTCTGTGATTCGGGACGAAAGGCCATATGTGAATAATATATGG

AAATGACGGTACCGAAAAAGCAAGCCACGGCTAACTACG

>7035 len=159 tot-seq=1

TAGGGAATATTCCACAATGAGCGAAAGCTTGATGGAGCGACACAGCGTGCACGATGAAGG

TCTTCTGATTGTAAAGTTCTTTTATTTAGGAAAAAAAGCGCGCTAGGAAATGAGCGCGCC

TTGATGGTACTAATTGAATAAGTGACAGCTAACTATGTG

>7036 len=159 tot-seq=1

TTTTTTTTTTCAAGCAGAAGACGGCATACGAGATTATTCGGTGACTGGAGTTCAGACGTG

TGCTTTTCCGATCTCCTACGGGGGCAGCAGATAAGGAGAAGGCTCGTCCTCCAGAACAGA

GGCAGCAGCTCCAGCAGCCCCAGGGGGCTCTAGCCACAG

>7037 len=159 tot-seq=1

CTATCGCACCACTGCACTCCAGCCTGGGCAACAAGAGCGAAACCCTCAAAAAAAAAAAAA

TCTGTGAAGGAAGGTCAAGAAGGAAAGAGGAAACACACACACATACACACACATGCACAC

ACACACATAAAACATTCATGAACATTAGGTAGTTACATG

>7038 len=159 tot-seq=1

TGGGGAATTTTGGACAATGGGCGCAAGCCTGATCCAGCCATGCCGCGTGCGGGAAGAAGG

CCTTCGGGTTGTAAACCGCTTTTGGCAGGAACGAAACGGCGATTCCTAATACGAGTCGTT

AATGACGGTACCTGCAGAATAAGCACCGGCTAACTACGT

>7039 len=159 tot-seq=1

TGGGGAATCTTGCGCAATGGGCGAAAGCCTGACGCAGCAACGCCGCGTGAGGGAAGAAGG

CTTTCGGGTTGTAAACCTCTTTCAGGAGGGACGAAGCTTCCGAGGTTAATAGCCTACGGG

GTGACGGTACCTCCAGAAGAAGCCCCGGCTAACTACGTG

>7040 len=160 tot-seq=1

TGGGGGATATTGCACAATGGGGGAAACCCTGACCGAGCAACGCCGCGTGAGGGAAGAAGT

ATTTCGGTATGTAAACCTCTGTTATAAAGGAAGAACGGTATGAATAGGAAATGATTCATA

AGTGACGGTACTTTATGAGAAAGCCACGGCTAACTACGTG

>7041 len=160 tot-seq=1

TAGGGAATCTTCCACAATGGACGAAAGTCTGATGGAGCAACGCCGCGTGAACGAAGAAGG

TCTTCGGATTGTAAAGTTCTGTTATAGAGGAAGAACGGTGTACGTAGGAAATGACGTACA

AGTGACGGTACTCTGTGAGGAAGCCACGGCTAACTACGTG

>7042 len=160 tot-seq=1

TAGGGAATTTTCGTCAATGGGGGAAACCCTGAACGAGCAATGCCGCGTGAGTGAAGAAGG

CCTTCGGGTCGTAAAGGACTTTTGTCCGGGAAGAAAAGCGCGACGTTAATACTATTGCGT

GCTGACGGTACCGGAAGAATAAGCACCGGCTAACTACGTG

>7043 len=160 tot-seq=1

TAGGGAATCTTCGGCAATGGGCGAAAGCCTGACCGAGCAACGCCGCGTGAATGAAGAAGG

CCTTCGGGTCGTAAAGCTCTGTTGCGGGGGAAAAAAGGCAGCATCAGGAAATGGGTGCTG

ACTGATGGTGCCCCGCCAGAAAGTCACGGCTAACTACGTG

>7044 len=160 tot-seq=1

TAGGGAATTTTCGTCAATGGGCGCAAGCCTGAACGAGCGATGCCGCGTGAGTGAAGAAGG

CCTTCGGGTTGTAAAGGACTTTTGTCCGGGAAGAAAAGCGCGATGTTAATACCATTGCGT

GCTGACGGTACCGGAAGAATAAGCACCGGCTAACTACGTG

>7045 len=160 tot-seq=1

TGGGGGATATTGCACAATGGGGGGAACCCTGATGCAGCGACGCCGCGTGGGCGATGGAGT

GCTTCGGGTTGTAAAGGACTTTTGTCCGGGAAGAAAAGCGCGATGTTAATACCATTGCGT

GCTGACGGTACCGGAAGAATAAGCACCGGCTAACTACGTG

>7046 len=160 tot-seq=1

TGGGGAATATTGCACAATGGGGGAAACCCTGATGCAGCAACGCCGCGTGAGTGAAGAAGG

TTTTCGGGTCGTAAAGCTCTGTTGCGGGGGAAAAAAGGCAGCATCAGGAAATGGGTGCTG

ACTGATGGTGCCCCGCCAGAAAGTCACGGCTAACTACGTG

>7047 len=160 tot-seq=1

CTGCGCTCGGACATTGGGGTAAAGCTTGGGAGAGTTAAGAGGGGGCTGTTTGCTGGGATG

AAACTGTCCACAGCTCGGCTGGGCCATCTTCTTACTCAGGCTGACCCTAATTAAACATGG

AGCAGACAGACTGGCTCCCAAGAAGATTGGCTATGTGTCC

>7048 len=160 tot-seq=1

TGGGGGATATTGCACAATGGGGGGAACCCTGATGCAGCGACGCCGCGTGGGTGAAGAAGT

ATTTCGGTATGTAAAGTACTTTCAGCGGGGAGGAAGGGAGTAAAGTTAATACCTTTGCTC

ATTGACGTTACCCGCAGAAGAAGCACCGGCTAACTCCGTG

>7049 len=160 tot-seq=1

TAGGGAATCTTCCGCAATGGGCGAAAGCCTGACGGAGCAACGCCGCGTGAGTGATGAAGG

TCTTCGGATCGTAAAGGACTTTTGTCAGGGAAGAAAAGGCTGTTGCTAATATCGACAGCT

GATGACGGTACCTGAAGAATAAGCACCGGCTAACTACGTG

>7050 len=160 tot-seq=1

TGGGGAATATTGCACAATGGGGGAAACCCTGATGCAGCGACGCCGCGTGAGTGAAGAAGT

ATTTCGGGTTGTAAAGGACTTTTGTCAGGGAAGAAAAGGCTGTTGCTAATACCAACAGCT

GATGACGGTACCTGAAGAATAAGCACCGGCTAACTACGTG

>7051 len=160 tot-seq=1

TGGGGAATATTGCACAATGGGGGAAACCCTGATGCAGCAACGCCGCGTGAGTGAAGAAGG

TTTTCGGATTGTAAAGTTCTGTTATAGAGGAAGAACGGTGTACGTAGGAAATGACGTACA

AGTGACGGTACTCTGTGAGGAAGCCACGGCTAACTACGTG

>7052 len=160 tot-seq=1

TGGGGAATCTTGCACAATGGGCGAAAGCCTGATGTAGCGACGCCGCGTGCGGGATGAAGG

CCCTCGGGTCGTAAAGTTCTGTTGTAAGGGAAGAACAAGTGCCGCAGGCAATGGCGGCAC

CTTGACGGTACCTTGCGAGAAAGCCACGGCTAACTACGTG

>7053 len=160 tot-seq=1

TGGGGAATTTTGGACAATGGGCGCAAGCCTGATCCAGCCATGCCGCGTGTCTGAAGAAGG

CCTTCGGGTTGTAAAGGACTTTTGTCAGGGAAGAAAAGGCTGTTGCTAATATCGACAGCT

GATGACGGTACCTGATTAAGAAGCCCCGGCTAACTACGTG

>7054 len=160 tot-seq=1

TGGGGAATTTTGGACAATGGGCGCAAGCCTGATCCAGCCATGCCGCGTGTCTGAAGAAGG

CCTTCGGGTTGTAAAGGACTTTTGTCAGGGAAGAAAAGGCGATCTCTAATACAGGTCACT

CATGACGGTACCTGAAGAATAAGCACCGGCTAACTACGTG

>7055 len=160 tot-seq=1

TGGGGGATATTGCACAATGGGGGAAACCCTGAACGAGCAATGCCGCGTGAGTGAAGAAGG

TCTTCGGATCGTAAAGCTCTGTTGTAAGTGAAGAACGGCCCATAGAGGAAATGCTATGGG

AGTGACGGTAGCTTACCAGAAAGCCACGGCTAACTACGTG

>7056 len=160 tot-seq=1

TGGGGAATTTTGGACAATGGGCGCAAGCCTGATCCAGCAATGCCGCGTGAGTGAAGAAGG

CCCTCGGGTTGTAAAGCTCTTTTGTCAGGGAAGAAAAGGCTGTTGCTAATACCAACAGCT

GATGACGGTACCTGAAGAATAAGCACCGGCTAACTACGTG

>7057 len=160 tot-seq=1

TAGGGAATTTTCGTCAATGGGGGAAACCCTGAACGAGCAACGCCGCGTGAGTGAAGAAGG

CCCTCGGGTTGTAAAGCACTTTCAGTGAGGAAGAACGCCTGGTGGTTAATACCCATCAGG

AAAGACATCACTCACAGAAGAAGCACCGGCTAACTCCGTG

>7058 len=160 tot-seq=1

TGGGGAATATTGCACAATGGGGGAAACCCTGATGCAGCGACGCCGCGTGGAGGAAGAAGG

TCTTCGGATTGTAAAGCACTTTAAGTTGGGAGGAAGGGCAGTAAGCTAATACCTTGCTGT

TTTGACGTTACCGACAGAATAAGCACCGGCTAACTTCGTG

>7059 len=160 tot-seq=1

TGGGGAATTTTGGACAATGGGGGAAACCCTGATCCAGCCATCCCGCGTGAGTGAAGAAGG

CCCTCGGGTTGTAAAGCTCTTTTGTCAGGGAAGAAAAGGTGATCTCTAATACAGGTCACC

CATGACGGTACCTGAAGAATAAGCACCGGCTAACTACGTG

>7060 len=160 tot-seq=1

TAGGGAATTTTCGGCAATGGGGGAAACCCTGACGGAGCAACGCCGCGTGAGTGATGAAGG

TCTTCGGATCGTAAAACTCTGTTATCAGGGAAGAACAAATGTGTAAGTAACTGTGCACAT

CTTGACGGTACCTGATCAGAAAGCCACGGCTAACTACGTG

>7061 len=160 tot-seq=1

TGGGGAATATTGGACAATGGGGGCAACCCTGATCCAGCCATGCCGCGTGTGTGAAGAAGG

TCTTCGGATTGTAAAGCACTTTAAGTTGGGAGGAAGGGCAGTTGCCTAATACGTAACTGT

TTTGACGTTACCGACAGAATAAGCACCGGCTAACTTTGTG

>7062 len=160 tot-seq=1

TGGGGAATATTGGACAATGGGGGAAACCCTGATCCAGCCATGCCGCGTGTGTGAAGAAGG

CCTTCGGGTTGTAAAGGACTTTTGTCAGGGAAGAAAAGGACAGGGTTAATACCCCTGTCT

GATGACGGTACCTGAAGAATAAGCACCGGCTAACTACGTG

>7063 len=160 tot-seq=1

TGGGGAATATTGGACAATGGGCGAAAGCCTGATCCAGCCATGCCGCGTGAGTGAAGAAGG

TCTTCGGATCGTAAAGCTCTGTTGTAAGTGAAGAACGGCTCATAGAGGAAATGCTATGGG

AGTGACGGTAGCTTACCAGAAAGCCACGGCTAACTACGTG

>7064 len=160 tot-seq=1

TGGGGAATCTTCCGCAATGGGGGAAACCCTGATCCAGCCATCCCGCGTGTGCGATGAAGG

CCTTCGGGTTGTAAAGCACTTTTGGCAGGAAAGAAACGTCATGGGTTAATACCCCGTGAA

ACTGACGGTACCTGCAGAATAAGCACCGGCTAACTACGTG

>7065 len=160 tot-seq=1

TGGGGAATATTGGGCAATGGACGCAAGTCTGACCCAGCAACGCCGCGTGAAGGAAGAAGG

CTTTCGGGTTGTAAAGCACTTTCAGTGAGGAAGAACGCCTGGTGGTTAATACCCATCAGG

AAAGACATCACTCACAGAAGAAGCACCGGCTAACTCCGTG

>7066 len=160 tot-seq=1

TAGGGAATCTTCGGCAATGGGGGGAACCCTGACCGAGCAACGCCGCGTGTCTGAAGAAGG

CCTTCGGGTTGTAAAGGACTTTTGTCAGGGAAGAAAAGGACAGGGTTAATACCCCTGTCT

GATGACGGTACCTGAAGAATAAGCACCGGCTAACTACGTG

>7067 len=160 tot-seq=1

TGAGGAATATTGGTCAATGGGCGAGAGCCTGAACCAGCCAAGTCGCGTGGGTGAAGAAGG

CCCTCGGGTTGTAAAGCTCTTTTGTCAGGGAAGAAAAGGTGATCTCTAATACAGGTCACT

CATGACGGTACCTGAAGAATAAGCACCGGCTAACTACGTG

>7068 len=160 tot-seq=1

CCTGGTTCTTGTGCTGGTGGTGAGGTTAATTTTTGGCATTTTACTCTGAGGGAGAAGGAA

TGAAGAGATATCTGGTTGGGTCCAGACTGAACCGGGTCATCATGTGGTGGGAGGGGAATT

GAGGATGTGATGGAGGATAGAGGGGCAGGTCAAGAGATCA

>7069 len=160 tot-seq=1

TGGGGAATATTGCACAATGGGGGAAACCCTGATGCAGCGACGCCGCGTGAAGGATGAAGG

TCTTCGGATTGTAAAGCACTTTAAGTTGGGAGGAAGGGCAGTTACCTAATACGTGATTGT

TTTGACGTTACCGACAGAATAAGCACCGGCTAACTCTGTG

>7070 len=160 tot-seq=1

TGGGGAATATTGGACAATGGGCGCAAGCCTGGTCCAGCCATGCCGCGTGAGTGATGAAGG

CCCTAGGGTTGTAAAGCTCTTTCAGCGGGGAGGAAGGGAGTAAAGTTAATACCTTTGCTC

ATTGACGTTACCCGCAGAAGAAGCACCGGCTAACTCCGTG

>7071 len=160 tot-seq=1

GGCACTGTAGTGCCAGCTGAGGGGTCACTATCACACCTGCACCTTTATTTAAAATTAGGA

TGCTAAGATTCCAAATCGGGTTTTACAGTTTTGTAAGCCCTACTGAGTACTACTCAGCTA

CACCCACCACAACCATAAATTAATCCCACATTTTGTCCCT

>7072 len=160 tot-seq=1

TAGGGAATCTTCCGCAATGGGCGAAAGCCTGACGGAGCAACGCCGCGTGAGTGATGAAGG

TCTTCGGATCGTAAAGCTCTGTTGTAAGTGAAGAACGGCTCACAGAGGAAATGCTATGGG

AGTGACGGTAGCTTACCAGAAAGCCACGGCTAACTACGTG

>7073 len=160 tot-seq=1

TAGGGAATTTTCGTCAATGGGGGGAATCCTGAACGAGCAATGCTGCGTGTATGAAGAAGG

CCTTCGGGTTGTAAAGTACTTTCAGCGGGGAGGAAGGGAGTAAAGTTAATACCTTTACTC

ATTGACGTTACCCGCAGAAGAAGCACCGGCTAACTCCGTG

>7074 len=160 tot-seq=1

TGGGGAATATTGCACAATGGGGGAAAGCCTGACGGAGCAACGCCGCGTGAGTGATGAAGG

TCTTCGGATCGTAAAACTCTGTTATCAGGGAAGAACAAATGTGTAAGTAACTATGCACGT

CTTGACGGTACCTAATCAGAAAGCCACGGCCAACTACGTG

>7075 len=160 tot-seq=1

TAGGGAATCTTCCGCAATGGGCGAAAGCCTGATCCAGCCATGCCGCGTGTGTGAAGAGGG

TCTTCGGATTGTAAAGCACTTTAAGTTGGGAGGAAGGGCAGTTGCCTAATACGTAACTGT

TTTGACGTTACCGACAGAATAAGCACCGGCTAACTCTGTG

>7076 len=160 tot-seq=1

TAGGGAATCTTCGGCAATGGACGAAAGTCTGACCGAGCAACGCCGCGTGAGTGAAGAAGG

TTTTCGGATTGTAAAGCACTTTAAGTTGGGAGGAAGGGCAGTAAATTAATACTTTGCTGT

TTTGACGTTACCGACAGAATAAGCACCGGCTAACTCTGTG

>7077 len=160 tot-seq=1

TAGGGAATCTTCGGCAATGGACGGAAGTCTGACCGAGCAACGCCGCGTGAGTGAAGAAGG

TTTTCGGGTTGTAAAGGACTTTTGTCAGGGAAGAAAAGGCTGTTGCTAATATCGACAGCT

GATGACGGTACCTGAAGAATAAGCACCGGCTAACTACGTG

>7078 len=160 tot-seq=1

TGGGGAATATTGGGCAATGGAGGAAACTCTGACCCAGCAACGCCGCGTGGAGGAAGAAGG

TTTTCGGGTTGTAAAGTACTTTCAGCGGGGAGGAAGGGAGTAAAGTTAATACCTTTGCTC

ATTGACGTTACCCGCAGAAGAAGCACCGGCTAACTCCGTG

>7079 len=160 tot-seq=1

TGGGGAATATTGGACAATGGGGGGAACCCTGATCCAGCCATGCCGCGTGTGTGAAGAAGG

CCCTCGGGTTGTAAAGGACTTTTGTCAGGGAAGAAAAGGCTGTTGCTAATATCAATAGCT

GATGACGGTACCTGAAGAATAAGCACCGGCTAACTACGTG

>7080 len=160 tot-seq=1

TGGGGAATTTTGGACAATGGGCGCAAGCCTGACGGAGCAACGCCGCGTGAGTGATGAAGG

GTTTCGGCTCGTAAAACTCTGTTATTAGGGAAGAACAAATGTGTAAGTAACTGTGCACAT

CTTGACGGTACCTAATCAGAAAGCCACGGCTAACTACGTG

>7081 len=160 tot-seq=1

TGGGGAATATTGGGCAATGGAGGAAACTCTGACCCAGCAACGCCGCGTGGAGGAAGAAGG

TTTTCGGATCGTAAACTGCTTTTGTCAGGGACGAAAAGGGATGCGATAACACCGCATTCC

GCTGACGGTACCTGAAGAATAAGCACCGGCTAACTACGTG

>7082 len=160 tot-seq=1

TGGGGAATATTGCGCAATGGGGGGAACCCTGACGCAGCCATGCCGCGTGAATGAAGAAGG

CCTACGGGTTGTAAAGGACTTTTGTCCGGGAAGAAAAGCGCGATGTTAATACCATTGCGT

GCTGACGGTACCGGAAGAATAAGCACCGGCTAACTACGTG

>7083 len=160 tot-seq=1

TGGGGAATATTGCGCAATGGGGGGAACCCTGACGCAGCCATGCCGCGTGAATGAAGAAGG

CCTTAGGGTTGTAAAGCTCTTTCGGCCGGGAAGAAATCGCATAGGCTAATACCCTGTGTG

GATGACGGTACCGGAATAAGAAGCACCGGCTAACTACGTG

>7084 len=160 tot-seq=1

TAGGGAATCTTCCACAATGGACGAAAGTCTGATGGAGCAACGCCGCGTGAATGAAGAAGG

CCTTCGGGTTGTAAAATTCTGTTATAAGGGAAGAAAGGTGATAGGAGGAAATGACTATCA

ATTGACGGTACCTTACGAGAAAGCCACGGCTAACTACGTG

>7085 len=160 tot-seq=1

TGAGGAATATTGGTCAATGGACGAGAGTCTGATGCAGCCATGCCGCGTGTATGAAGAAGG

CCTTCGGGTTGTAAAGTACTTTCAGCGGGGAGGAGGGGAGTAAAGTTAATACCTTTGCTC

ATTGACGTTACCCGCAGAAGAAGCACCGGCTAACTCCGTG

>7086 len=160 tot-seq=1

TGGGGAATTTTCCGCAATGGGCGAAAGCCTAATCCAGCCATGCCGCGTGTGTGAAGAAGG

TCTTCGGATTGTAAAGCACTTTAAGTTGGGGGGAGGGGCATTAACCTAATACGTTAGTGT

TTTGACGTTACCGACAGAATAAGCACCGGCTAACTCTGTG

>7087 len=160 tot-seq=1

TGGGGAATTTTGGACAATGGGCGAAAGCCTGATCCAGCCATGCCGCGTGTCTGAAGAAGG

CCTTCGGGTTGTAAAGGACTTTTGTCAGGGAAGAAAAGGGCGGGATTAATACCCCTGTCT

GATGACGGTACCTGACTAAGAAGCCCCGGCTAACTACGTG

>7088 len=160 tot-seq=1

TGAGGAATATTGGTCAATGGGCGTAAGCCTGAACCAGCCAAGTCGCGTGTGTGAAGAAGG

TCTTCGGATTGTAAAGCACTTTAAGTTGGGAGGAAGGGCAGTAAGTTAATACCTTGCTGT

TTTGACGTTACCAACAGAATAAGCACCGGCTAACTTCGTG

>7089 len=160 tot-seq=1

TAGGGAATCTTCGGCAATGGACGAAAGTCTGACCGAGCAACGCCGCGTGAGTGAAGAAGG

TTTTCGGATCGTAAAACTCTGTTGTTAGGGAAGAACAAGTACCGGAGTAACTGCCGGTAC

CTTGACGGTACCTAACCAGAAAGCCACGGCTAACTACGTG

>7090 len=160 tot-seq=1

TGGGGAATATTGCACAATGGGCGAAAGCCTGATGCAGCAACGCCGCGTGAGGGAAGACGG

TTTTCGGATCGTAAAACTCTGTTATTAGGGAAGAACAAATGTGTAAGTAACTATGCACGT

CTTGACGGTACCTAATCAGAAAGCCACGGCTAACTACGTG

>7091 len=160 tot-seq=1

TGGGGAATCTTGGACAATGGGCGAAAGCCCGATCCAGCAATATCGCGTGAGTGAAGAAGG

CCTTCGGGTTGTAAAGTACTTTCAGCGGGGAGGAAGGGAGTAAAGTTAATACCTTTGCTC

ATTGACGTTACCCGCAGAAGAAGCACCGGCTAACTCCGTG

>7092 len=160 tot-seq=1

TGAGGAATATTGGTCAATGGACGAGAGTCTGAACCAGCCAAGTAGCGTGAGTGAAGAAGG

TTTTCGGATCGTAAAACTCTGTTGTTAGAGAAGAACAAGGACGTTAGTAACTGAACGTCC

CCTGACGGTATCTAACCAGAAAGCCACGGCTAACTACGTG

>7093 len=160 tot-seq=1

TGGGGAATATTGCACAATGGGGGAAACCCTGATGCAGCGACGCCGCGTGAGCGAAGAAGG

CCTTCGGGTTGTAAAGTTCTTTCGGTATTGAGGAAGGTTGATGTGTTAATAGTACATCAA

ATTGACGTTAAATACAGAAGAAGCACCGGCTAACTCCGTG

>7094 len=160 tot-seq=1

TAGGGAATCTTCGGCAATGGGCGAAAGCCTGACCGAGCAACGCCGCGTGAATGAAGAAGG

CCTTCGGGTTGTAAAGCACTTTTGTCGGGAAAGAAAACCTCTGCCCTAATACGGTGGGGG

GATGACGGTACCGGAAGAATAAGCACCGGCTAACTACGTG

>7095 len=160 tot-seq=1

TGGGGAATTTTGGACAATGGGCGCAAGCCTGATCCAGCCATTCCGCGTGCAGGATGAAGG

CCCTCGGGTTGTAAACTGCTTTTGTACGGAACGAAACGGCTCTGGTTAATACCTGGGGCT

AATGACGGTACCGTAAGAATAAGCACCGGCTAACTACGTG

>7096 len=160 tot-seq=1

TGGGGAATCTTCCGCAATGGACGAAAGTCTGACGGAGCAACGCCGCGTGAGTGATGACGG

CCTTCGGGTTGTAAAATTCTGTTATAAGGGAAGAAAGGTGATAGGAGGAAATGACTATCA

ATTGACGGTACCTTATGAGAAAGCCACGGCTAACTACGTG

>7097 len=160 tot-seq=1

TAGGGAATCTTCGGCAATGGGCGAAAGCCTGACCGAGCAACGCCGCGTGAATGAAGAAGG

CCTTCGGGTTGTAAAATTCTTTCGGTATTGAGGAAGGTTGATGTGTTAATAGCACATCAA

ATTGACGTTAAATACAGAAGAAGCACCGGCTAACTCCGTG

>7098 len=160 tot-seq=1

TGGGGAATATTGCACAATGGGCGCAAGCCTGATGCAGCCATGCCGCGTGTATGAAGAAGG

CCTTCGGGTTGTAAAGTACTTTTGTCCGGAAAGAAAACGCCGTGGTTAATACCCGTGGCG

GATGACGGTACCGGAAGAATAAGCACCGGCTAACTACGTG

>7099 len=160 tot-seq=1

TAGGGAATTTTCGTCAATGGGGGAAACCCTGAACGAGCAATGCCGCGTGTGTGAAGAAGG

CCTTTTGGTTGTAAAGCACTTTAAGTGGGGAGGAAAAGCTTATGGTTAATACCCATAAGC

CCTGACGTTACCCACAGAATAAGCACCGGCTAACTCTGTG

>7100 len=160 tot-seq=1

TGGGGAATATTGCACAATGGGGGAAACCCTGATGCAGCGACGCCGCGTGTGTGAAGAAGG

CCTTCGGGTTGTAAAGCACTTTTGTCGGGAAAGAAAACCTCTGCCCTAATACGGTGGGGG

GATGACGGTACCGGAAGAATAAGCACCGGCTAACTACGTG

>7101 len=160 tot-seq=1

TGGGGAATATTGGACAATGGGCGCAAGCCTGATCCAGCCATACCGCGTGGGTGAAGAAGG

CCTTCGGGTTGTAAAGCCCTTTTGTTGGGAAAGAAATCCAGCCGGCTAATACCTGGTTGG

GATGACGGTACCTGTTTAGAAAGCTCCGGCTAACTACGTG

>7102 len=160 tot-seq=1

TGAGGAATATTGGTCAATGGGCGATGGCCTGATCCAGCAATGCCGCGTGTGTGAAGAAGG

CCTTCGGGTTGTAAAGCACTTTTGTCCGGAAAGAAAACCTCCTGGTTAATACCTGGGGGG

GATGACGGTACCGGAAGAATAAGCACCGGCTAACTACGTG

>7103 len=160 tot-seq=1

TGGGGAATCTTGGACAATGGGCGAAAGCCCGATCCAGCAATATCGCGTGTGTGAAGAAGG

TCTTCGGATTGTAAAGCACTTTAAGTTGGGAGGAAGGGCATTAACCTAATACGCTAGTGT

TTTGACGTTACCGACAGAATAAGCACCGGCTAACTTCGTG

>7104 len=160 tot-seq=1

TAGGGAATCTTCGGCAATGGGCGAAAGCCTGACCGAGCAACGCCGCGTGAATGAAGAAGG

CCCTTTGGTTGTAAAGCACTTTAAGTGGGGAGGAAAAGCTTATGGTTAATACCCATAAGC

CCTGACGTTACCCACAGAATAAGCACCGGCTAACTCTGTG

>7105 len=160 tot-seq=1

TGGGGAATTTTGGGCAATGGGGGCAACCCTGATCCAGCAATGCCGCGTGTGTGAAGAAGG

TCTTCGGATTGTAAAGCACTTTAAGTTGGGAGGAAGGGCATTAACCTAATACGTTAGTGT

TTTGACGTTACCGACAGAATAAGCACCGGCTAACTTCGTG

>7106 len=160 tot-seq=1

TGGGGAATTTTCCGCAATGGGCGAAAGCCTGACGGAGCAATGCCGCGTGGAGGTGGAAGG

CCTACGGGTCGTAAAGGACTTTTGTCAGGGAAGAAAAGGCTGTTGCTAATATCGACAGCT

GATGACGGTACCTGAAGAATAAGCACCGGCTAACTACGTG

>7107 len=160 tot-seq=1

TCGAGAATCATTCACAATGGGGGCAACCCTGATGGTGCGACGCCGCGTGAAGGAAGAAGT

AATTCGTTATGTAAACTTCTGTCATAGAGGAAGAACGGTGGATATAGGGAATGATATCCA

AGTGACGGTACTCTATAAGAAAGCCACGGCTAACTACGTG

>7108 len=160 tot-seq=1

TGGGGAATTTTGGACAATGGGCGCAAGCCTGATCCAGCCATGCCGCGTGTCTGAAGAAGG

CCTTCGGGTTGTAAAGGACTTTTGTCAGGGAAGAAAAGGACAGGGTTAATACCCCTGTCT

GATGACGGTACCTGAGGAATAAGCATCGGCTAACTCTGTG

>7109 len=160 tot-seq=1

TAGGGAATCTTCCGCAATGGGCGAAAGCCTGACGGAGCAACGCCGCGTGAGTGATGAAGG

TCTTCGGATCGTAAAACTCTGTTATTAGAGAAGAAAGTGCGTGAGAGTAACTGTTCACGT

TTCGACGGTATCTAACCAGAAAGCCACGGCTAACTACGTG

>7110 len=160 tot-seq=1

TGGGGAATATTGGACAATGGGCGAAAGCCTGATCCAGCCATGCCGCGTGTGTGAAGAAGG

TCCTCGGGTTGTAAAGCTCTTTTGTCAGGGAAGAAAAGGTGATCTCTAATACAGGTCACT

CATGACGGTACCTGAAGAATAAGCACCGGCTAACTACGTG

>7111 len=160 tot-seq=1

TAGGGAATCTTCCACAATGGGCGAAAGCCTGATGGAGCAACGCCGCGTGGGTGAAGAAGG

TCTTAGGGTTGTAAAGTTCTTTCGGTATTGAGGAAGGTTGATGTGTTAATAGTACATCAA

ATTGACGTTAAATACAGAAGAAGCACCGGCTAACTCCGTG

>7112 len=160 tot-seq=1

TGGGGAATTTTGGACAATGGGCGCAAGCCTGATCCAGCCATGCCGCGTGTCTGAAGAAGG

CCTTCGGGTTGTAAAGGACTTTTGTCAGGGAAGAAAAGGGCGGGGTTAATACCATTGCGT

GCTGACGGTACCGGAAGAATAAGCACCGGCTAACTACGTG

>7113 len=160 tot-seq=1

TGGGGAATTTTGGACAATGGGCGCAAGCCTGATCCAGCAATGCCGCGTGAGTGAAGAAGG

CCCTCGGGTTGTAAAGTACTTTCAGCGGGGAGGAAGGCGTTGTGGTTAATAACCACAGCG

ATTGACGTTACCCGCAGAAGAAGCACCGGCTAACTCCGTG

>7114 len=160 tot-seq=1

TGGGGAATATTGCACAATGGGCGCAAGCCTGATGCAGCCATGCCGCGTGTATGAAGAAGG

CCTTCGGGTTGTAAACTGCTTTTGTACGGAACGAAAAAGCTTCTCCTAATACGAGAGGCC

CATGACGGTACCGTAAGAATAAGCACCGGCTAACTACGTG

>7115 len=160 tot-seq=1

TGGGGAATATTGCACAATGGGCGCAAGCCTGATGCAGCCATGCCGCGTGTATGAAGAAGG

CCTTCGGGTTGTAAAGTACTTTCAGCGGGGAGGAAGGCGATAAGGTTACTACTTTGCTGT

TTTGACGTTACCGACAGAATAAGCACCGGCTAACTCTGTG

>7116 len=160 tot-seq=1

TAGGGAATCTTCGGCAATGGGGGCAACCCTGATGCAGCCATGCCGCGTGTATGAAGAAGG

CCTTCGGGTTGTAAAGTACTTTCAGCGGGGAGGAAGGCGATAAGGTTAATAACCTTGTCG

ATTGACGTTACCCGCAGAAGAAGCACCGGCTAACTCCGTG

>7117 len=160 tot-seq=1

TAGGGAATCTTCCGCAATGGACGAAAGTCTGACGGAGCAACGCCGCGTGAGTGAAGAAGG

TTTTCGGATCGTAAAACTCTGTTGTCAGGGAAGAACAAGTACGGGAGTAACTGCCCGTAC

CTTGACGGTACCTGGCCAGAAAGCCACGGCTAACTACGTG

>7118 len=160 tot-seq=1

GGCGGGGAGAATGCCCTCCACTGCCCCCTCCCCAACCTTGACTCGATTGCTCTTCTCCTC

TGGGGACAGCGATGTGGTTAGGAAAGGCAGCATTCCCAAGGGCGCTTCTGTGGCTGCATC

TGCCTCTTCCCTTGCTCCCCCCTGCCCTTTCTGGTAAGTG

>7119 len=160 tot-seq=1

TAGGGAATCTTCCACAATGGACGCAAGTCTGATGCAGCCATGCCGCGTGAGTGAAGAAGG

TTTTCGGATCGTAAAGCTCTGTTGTAAGAGAAGAACGAGTGTGAGAGTGGAAAGTTCACA

CTGTGACGGTATCTTACCAGAAAGGGACGGCTAACTACGT

>7120 len=160 tot-seq=1

TAGGGAATCTTCGGCAATGGACGGAAGTCTGACCGAGCAACGCCGCGTGAGTGAAGAAGG

CCTTCGGGTTGTAAAGCACTTTCAGCGGGGAGGAAGGCGGTGAGGTTAATAACCTCATCG

ATTGACGTTACCCGCAGAAGAAGCACCGGCTAACTCCGTG

>7121 len=160 tot-seq=1

TGGGGAATATTGCACAATGGGCGCAAGCCTGATGCAGCCATGCCGCGTGTATGAAGAAGG

CCTTCGGATCGTAAAACTCTGTTGTTAGAGAAGAACACGAGTGAGAGTAACTGTTCATTC

GATGACGGTATCTAACCAGCAAGTCACGGCTAACTACGTG

>7122 len=160 tot-seq=1

TAGGGAATCTTCCACAATGGACGCAAGTCTGATGGAGCAACGCCGCGTGAGTGAAGAAGG

TCTTCGGATCGTAAAACTCTGTTGTTAGAGAAGAACACGAGTGAGAGTAACTGTTCATTC

GATGACGTTACCCGCAGAAGAAGCACCGGCTAACTCCGTG

>7123 len=160 tot-seq=1

TAGGGAATCTTCCACAATGGACGCAAGTCTGATGGAGCAACGCCGCGTGAGTGAAGAAGG

TCTTCGGATCGTAAAACTCTGTTGTTAGAGAAGAAAGTGCGTGAGAGTAACTGTTCATTC

TATGACGGTATCTAACCAGCAAGTCACGGCTAACTACGTG

>7124 len=160 tot-seq=1

TGGGGAATATTGCACAATGGGCGCAAGCCTGATGCAGCCATGCCGCGTGTATGAAGAAGG

TTTTCGGATCGTAAAACTCTGTTGTTAGAGAAGAACAAGGACGTTAGTAACTGAACGTCC

CCTGACGGTATCTAACCAGAAAGCCACGGCTAACTACGTG

>7125 len=160 tot-seq=1

TAGGGAATCTTCGGCAATGGGGGGAACCCTGACCGAGCAACGCCGCGTGAGTGAAGAAGG

TTTTCGGATCGTAAAGCTCTGTTGTTAAAGAAGAACACGTATGAGAGTAACTGTTCATAC

GTTGACGGTATTTAACCAGAAAGTCACGGCTAACTACGTG

>7126 len=160 tot-seq=1

TGGGGAATTTTGGACAATGGGGGCAACCCTGATCCAGCCATGCCGCGTGCGGGAAGAAGG

TTTTCGGATCGTAAAGCTCTGTTGTTGGTGAAGAAGGATAGAGGTAGTAACTGGCCTTTA

TTTGACGGTAATCAACCAGAAAGTCACGGCTAACTACGTG

>7127 len=160 tot-seq=1

TGGGGAATCTTGCACAATGGGCGAAAGCCTGATGCAGCGACGCCGCGTGTATGAAGAAGG

CCTTCGGGTTGTAAAGTACTTTCAGCGGGGAGGAAGGGAGTAAAGTTAATACCTTTGCAC

ATTGACGTTACCCGCAGAAGAAGCACCGGCTAACTCCGTG

>7128 len=160 tot-seq=1

TGGGGAATATTGCACAATGGGGGAAACCCTGATGCAGCGACGCCGCGTGAGCGATGAAGG

CTTTCGAGTCGTAAAGCTCTGTTGTTGGTGAAGAAGGATAGAGGTAGTAACTGGCCTTTA

TTTGACGGTAATCAACCAGAAAGTCACGGCTAACTACGTG

>7129 len=160 tot-seq=1

TGGGGAATTTTGGACAATGGGCGAAAGCCTGATCCAGCAATGCCGCGTGTAGGATGAAGG

CCCTCGGGTTGTAAACTACTTTTGTACGGAACGAAAAGACTCTTTCTAATAAAGAGAGTC

CATGACGGTACCGTAAGAATAAGCACCGGCTAACTACGTG

>7130 len=160 tot-seq=1

TGGGGAATATTGGACAATGGAGGCAACTCTGATCCAGCAATTCTGTGTGTGTGAAGAAGG

TTTTCGGATCGTAAAGCTCTGTTGTTGGTGAAGAAGGATAGAGGTAGTAACTGGCCTTTA

TTTGACGGTAATCAACCAGAAAGTCACGGCTAACTACGTG

>7131 len=160 tot-seq=1

TGGGGAACATTGCACAATGGGCGGAAGCCTGATGCAGCGACGCCGCGTGGGGGATGACGG

CCTTCGGGTTGTAAAGCTCTGTTGTTGGTGAAGAAGGACAGGGGTAGTAACTGACCTTTG

TTTGACGGTAATCAATTAGAAAGTCACGGCTAACTACGTG

>7132 len=160 tot-seq=1

TAGGGAATCTTCCACAATGGACGAAAGTCTGATGGAGCAACGCCGCGTGAGTGAAGAAGG

TTTTCGGATCGTAAAGCTCTGTTGTTGGTGAAGAACGTGCGTGAGAGTAACTGGCCTTTA

TTTGACGGTAATCAACCAGAAAGTCACGGCTAACTACGTG

>7133 len=160 tot-seq=1

TAGGGAATCTTCCGCAATGGACGCAAGTCTGACGGAGCAACGCCGCGTGAGTGAAGAAGG

ATTTCGGTTCGTAAAACTCTGTTGTTAGAGAAGAAGGACAGGGGTAGTAACTGACCTTTG

TTTGACGGTAATCAATTAGAAAGTCACGGCTAACTACGTG

>7134 len=160 tot-seq=1

TAGGGAATCTTCCACAATGGACGCAAGTCTGATGGAGCAACGCCGCGTGAGTGAAGAAGG

GTTTCGGGTTGTAAAGCACTTTCAGTGAGGAAGAACGCCTGGTGGTTAATACCCATCAGG

AAAGACATCACTCACAGAAGAAGCACCGGCTAACTCCGTG

>7135 len=160 tot-seq=1

TAGGGAATCTTCCGCAATGGGCGAAAGTCTGACGGAGCAACGCCGCGTGAGTGAAGAAGG

ATTTCGGTTCGTAAAGCTCTGTTGTTAGGGAAGAATGATTATGTAGTAACTATACATAGT

AGAGACGGTAACTAACCAGAAAGGGACGGCTAACTACGTG

>7136 len=160 tot-seq=1

TAGGGAATCTTCGGCAATGGACGGAAGTCTGACCGAGCAACGCCGCGTGAGTGAAGAAGG

TTTTCGGATCGTAAAGCTCTGTTGTTAGAGAAGAATGACTGTGTAGTAACTATACACAGT

AGAGACGGTACCTAACCAGAAAGCCACGGCTAACTACGTG

>7137 len=160 tot-seq=1

TGGGGAATCTTCCGCAATGGACGAAAGTCTGACGGAGCAACGCCGCGTGAGTGATGACGG

TCTTCGGATTGTAAAACTCTGTTGTTAGAGAAGAACAGCGCATAGAGTAACTGTTATGCG

TGTGACGGTATCTAACCAGAAAGCCACGGCTAACTACGTG

>7138 len=160 tot-seq=1

TAGGGAATCTTCCACAATGGACGAAAGTCTGATGGAGCAACGCCGCGTGTGTGATGAAGG

GTTTCGGCTCGTAAAACACTGTTATAAGAGAAGAACGTCAGTGAGGGTAACTATTCATTG

AGTGACGGTATCTTATCAGAAAGGGACGGCTAAATACGTG

>7139 len=160 tot-seq=1

TAGGGAATCTTCCACAATGGACGCAAGTCTGATGGAGCAACGCCGCGTGAGTGAAGAAGG

GTTTCGGATCGTAAAGCTCTGTTGTAAGTGAAGAACGGCTCATAGAGGAAATGCTATGGG

AGTGACGGTAGCTTACCAGAAAGCCATGGCTAACTACGTG

>7140 len=160 tot-seq=1

TGGGGAATCTTGCGCAATGCGCGAAAGCGTGGCGCAGCAACGCCGCGTGCGGGAAGACGG

CCTTCGGGTTGTAAACCGCTTTCAGTTGGGACGAAGCTTCGCCGGTTAATAGCCGGCTGG

AGTGACGGTACCTTCACAAGAAGCCCCGGCTAACTACGTG

>7141 len=160 tot-seq=1

TGGGGAATCTTGCACAATGCGCGAAAGCGTGATGCAGCAACGCCGCGTGCGGGAAGAAGG

CCTTAGGGTTGTAAACCGCTTTCAGTTGGGACGAAGCTTCAGTCGTTAATAGCGGTTTGG

AGTGACGGTACCTTCAGAAGAAGCGCCGGCTAACTACGTG

>7142 len=160 tot-seq=1

TGGGGAATTTTGCACAATGGGGGAAACCCTGATGCAGCGACGCCGCGTGATTTAGAAGGC

CTTATAGATTGTAAAGTTCTTTTATATGGGAAGAAACGCTAAAATAGGAAATGATTTTAG

TTTGACTGTACCATTTGAATAAGTATCGGCTAACTATGTG

>7143 len=160 tot-seq=1

CAAGGAATTTTCCGCAATGGGCGCAAGCCTGACGGAGCAACGCCGCGTGAGTGAAGAAGG

TTTTCGGATCGTAAAGCTCTGTTGTTAAGGAAGAACGAGTGTGAGAATGGAAAGTTCATG

CTGTGACGGTACTTAACCAGAAAGGGACGGCTAACTACGT

>7144 len=160 tot-seq=1

TGGGGAATTTTGGACAATGGGCGCAAGCCTGATCCAGCAATGCCGCGTGCGGGAAGAAGG

CCTTCGGGTTGTAAACCGCTTTTGGCAGGAACGAAACGGTGTCCGCTAATACCGGGCGCT

AATGACGGTACTTAACCAGAAAGGGACGGCTAACTACGTG

>7145 len=160 tot-seq=1

TGGGGAATTTTGCACAATGGGGGAAACCCTGATGCAGCGACGCCGCGTGATTTAGAAGGC

CTTCGGGTTGTAAAGCTCTGTTAATCGGGACGAAAGGCCTTCTTGCGAATAGTGAGAAGG

ATTGACGGTACCGGAATAGAAAGCCACGGCTAACTACGTG

>7146 len=160 tot-seq=1

TGGGGAATCTTGCACAATGGGCGAAAGTCTGACGGAGCAACGCCGCGTGAGTGAAGAAGG

TTTTCGGATCGTAAAACTCTGTTGTAAGAGAAGAACAAATTGTAGAGTAACTGCTACAGT

CTTGACGGTATCTTACCAGAAAGCCACGGCTAACTACGTG

>7147 len=160 tot-seq=1

TGGGGAATATTGGACAATGGAGGAAACTCTGATCCAGCAATTCTGTGTGAGTGAAGAAGG

ATTTCGGTTCGTAAAACTCTGTTGTTAGAGAAGAACAGCGCATAGAGTAACTGTTATGCG

TGTGACGGTATCTAACCAGAAAGCCACGGCTAACTACGTG

>7148 len=160 tot-seq=1

TAGGGAATCTTCCGCAATGGACGAAAGTCTGACGGAGCAACGCCGCGTGAGTGAAGAAGG

ATTTCGGTTCGTAAAACTCTGTTGTTAGGGAAGAAAAAATGTATAGTAACTATATACAAA

AGAGACGGTACCTACAGAAGAAGCCCCGGCTAACTACGTG

>7149 len=160 tot-seq=1

TGGGGAATATTGGACAATGGAGGAAACTCTGATCCAGCGACACAGCGTGCACGATGAAGG

TCTTCGGATTGTAAAGTGCTGTTATAAGGGAAGAACATTTGCAATAGGAAATGATTGCAG

ACTGACGGTACCTTGTCAGAAAGCGATGGCTAACTATGTG

>7150 len=160 tot-seq=1

TGAGGAATATTGGTCAATGGGCGCAAGCCTGACGGAGCGATGCCGCGTGAGTGAAGAAGG

CCTTCGGGTTGTAAAACTCTGTCCTCATCCAAGAGAGAGACCTGTAGTAACTGCCAGGTC

TAGGACGGTAGATGAGGAGGAAGCCCCGGCTAACTATGTG

>7151 len=160 tot-seq=1

TGGGGAATATTGGACAATGGGCGCAAGCCTGATCCAGCCATGCCGCGTGTGTGAAGAAGG

CCTTCGGGTTGTAAAGCACTTTCAGCGAGGAGGAAGGGGTTGTAGTTAATAGCTGCAATT

TTTGACGTTACTCGCAGAAGAAGCACCGGCTAACTCCGTG

>7152 len=160 tot-seq=1

TGAGGAATATTGGACAATGGGTTAGCGCCTGATCCAGCCATCCCGCGTGTATGAAGAAGG

CCTTCGGGTTGTAAAGTACTTTCAGCGGGGAGGAAGGGAGTAAAGTTAATACCTTTGCTC

ATTGACGTTACCCGCAGAAGAAGCACCGGCTAACTCCGTG

>7153 len=160 tot-seq=1

TGGGGAATTTTGGACAATGGGCGAAAGCCTGATCCAGCAATGCCGCGTGTGTGATGAAGG

CCTTCGGGTTGTAAAGCACTTTTGGTGGGAACGAAACGGTCCGGGTTAATACCCTGGACT

AATGACGGTACCCGCAGAATAAGCACCGGCTAACTACGTG

>7154 len=160 tot-seq=1

TGGGGAATATTGCACAATGGGCGCAAGCCTGATGCAGCCATGCCGCGTGTATGAAGAAGG

CCTTCGGGTTGTAAAGCACTTTTGTCCGGAAAGAAATCCTTGGCTCTAATACAGTCGGGG

GATGACGGTACCGGAAGAATAAGCACCGGCTAACTACGTG

>7155 len=160 tot-seq=1

TAGGGAATCTTCCACAATGGGCGCAAGCCTGATGGAGCAACACCGCGTGAGTGAAGAAGG

GTTTCAGCTCGTAAAGCTCTGTTGTTGGAGAAGAAGGACAAGGGTAGTAACTGACCTTTG

TTTGACGGTAATCAATTAGAAAGTCACGGCTAACTACGTG

>7156 len=160 tot-seq=1

TAGGGAATCTTCCACAATGGGTGCAAACCTGATGGAGCAATGCCGCGTGAGTGAAGAAGG

TCTTCGGATCGTAAAGCTCTGTTGTTAGTGAAGAAGGACAGGGGTAGTAACTGACCTTTG

TTTGACGGTAATCAATTGGAAAGTCACGGCTAACTACGTG

>7157 len=160 tot-seq=1

TGGGGAATATTGGACAATGGGGGGAACCCTGATCCAGCAATGCCGCGTGTGTGAAGAAGG

CCTGCGGGTTGTAAAGCACTTTCAGTAGGGAGGAGGGCTGATAGGTTAAGAGCTAATTAG

TTGGACGTTACCTACAGAAGAAGCACCGGCTAACTCCGTG

>7158 len=160 tot-seq=1

TGGGGAATATTGCGCAATGGGGGAAGCCTTATGAAGCAATGCCGCGTGAACGATGAAGGT

CTTATAGATTGTAAAGTTCTTTTATATGGGAAGAAACGCTAAGATAGGAAATGATTTTAG

TTTGACTGTACCATTTGAATAAGTATCGGCTAACTATGTG

>7159 len=160 tot-seq=1

TAGGGAATATTGCACAATGGAGGAAACTCTGATGCAGCCATGCCGCGTGTGTGAAGAAGG

CCTTCGGGTTGTAAAGCTCTGTTGGTAGTGAAGAAAGATAGAGGTAGTAACTGGCCTTTA

TTTGACGGTAATTACTTAGAAAGTCACGGCTAACTACGTG

>7160 len=160 tot-seq=1

TAGGGAATATTGCACAATGGAGGAAACTCTGATGCAGCCATGCCGCGTGTGTGAAGAAGG

TCTTCGGATCGTAAAGCTCTGTTGTAAGTGAAGAACGGCTCATAGAGGAAATGCTATGGG

AGTGACGGTAGCTTACCAGAAAGCCACGGCTAACTACGTG

>7161 len=160 tot-seq=1

TAGGGAATCTTCGGCAATGGACGGAAGTCTGACCGAGCAATGCCGCGTGAGTGAGGAAGG

TCTTCGGATCGTAAAGCTCTGTTGTAAGAGAAAAACGGCACTCATAGGGAATGATGAGTG

AGTGATGGTATCTTACCAGAAAGTCACGGCTAACTACGTG

>7162 len=160 tot-seq=1

TGGGGAATATTGCACAATGGACGCAAGTCTGATGGAGCAACGCCGCGTGAGTGAAGAAGG

CCTTTGGGTTGTAAAGTACTTTCAGCGGGGAGGAAGGGAGTAAAGTTAATACCTTTGCTC

ATTGACGTTACCCGCAGAAGAAGCACCGGCTAACTCCGTG

>7163 len=160 tot-seq=1

TGGGGTATATTGCACAATGGAGGGAACTCTGATGCAGCGACGCCGCGTGAACGAAGAAGG

CTTTCGGATCGTAAAACTCTGTTGTTAGAGAAGAACAAGGACGTTAGTAACTGAACGTCC

CCTGACGGTATCTAACCAGAAAGCCACGGCTAACTACGTG

>7164 len=160 tot-seq=1

TAGGGAATCTTCCACAATGGGCGCAAGCCTGATGGAGCAACACCGCGTGAGTGAAGAAGG

ATTTCGGTTCGTAAAACTCTGTTGTTAGAGAAGAACAGCGCATAGAGTAACTGTTATGCG

TGTGACGGTATCTAACCAGAAAGCCACGGCTAACTACGTG

>7165 len=160 tot-seq=1

TAGGGAATCTTCCACAATGGGCGCAAGCCTGATGGAGCAACACCGCGTGAGTGAAGAAGG

GTTTCGGCTCGTAAAACTCTGTTGTTGGAGAAGAACGTGCGTAAGAGTAACTGAACGTCC

CCTGACGGTATCTAACCAGAAAGCCACGGCTAACTACGTG

>7166 len=160 tot-seq=1

TAGGGAATATTGCACAATGGAGGAAACTCTGATGCAGCCATGCCGCGTGTGTGAAGAAGG

CCTTCGGGTTGTAAACCGCTTTTGTCAGAGACGAAAAGGGACGTACGAATAATACGTTCC

GCTGACGGTATCTGAAGAATAAGCACCGGCTAACTACGTG

>7167 len=160 tot-seq=1

TAGGGAATCTTCCACAATGGACGCAAGTCTGATGGAGCAACGCCGCGTGAGTGAAGAAGG

TTTTCGGATCGTAAAACCCTGTTGTTAGAGAAGAAGGATAGAGGCAGTAACTGGCCTTTA

TTTGACGGTAATCAACCAGAAAGTCACGGCTAACTACGTG

>7168 len=160 tot-seq=1

TAGGGAATCTTCCACAATGGGCGCAAGCCTGATGCAGCAACGCCGCGTGAACGATGAAGG

TCTTCGGATCGTAAAGTTCTGTTGTTAGAGAAGAAAGTGCGTGAGAGTAACTGTTCACGT

TTCGACGGTATCTAACCAGAAAGCCACGGCTAACTACGTG

>7169 len=160 tot-seq=1

TGGGGAATATTGCACAATGGGGGAAACCCTGATGCAGCGACGCCGCGTGAACGAAGAAGG

TATTCGTATCGTAAAGTTCTGTTGTTGGTGAAGAAGGATAGAGGTAGTAACTGGCCTTTA

TTTGACGGTAATCAACCAGAAAGTCACGGCTAACTACGTG

>7170 len=160 tot-seq=1

TGGGGAATCTTCCGCAATGGACGAAAGTCTGACGGAGCAACACCGCGTGAGTGAAGAAGG

GTTTCGGCTCGTAAAGCTCTGTTGTTGGAGAAGAACGTATCTGAGAGTAACTGTTCAGGT

AGTGACGGTATCCAACCAGAAAGTCACGGCTAACTACGTG

>7171 len=160 tot-seq=1

TGGGGAATATTGCGCAATGGGGGAAACCCTGACGCAGCGACGCCGCGTGGGTGAAGAAGG

TCTTCGGATCGTAAAACCCTGTTGTTCGAGAAGAAAGTGCGTGAGAGTAACTGTTCACGT

TTCGACGGTATCTAACCAGAAAGCCACGGCTAACTACGTG

>7172 len=160 tot-seq=1

TGGGGAATATTGCACAATGGGGGAAACCCTGATGCAGCGACGCCGCGTGAGCGAAGAAGT

ATTTCGGCTCGTAAAACCCTGTTGTTAGAGAAGAAAGTGCGTGAGAGTAACTGTTCACGT

TTCGACGGTATCTAACCAGAAAGCCACGGCTAACTACGTG

>7173 len=160 tot-seq=1

TAGAGAATCTTCCACAATGGGCGCAAGCCTGATGGAGCAACGCCGCGTGGGTGAAGAAGG

TTTTCGGATCGTAAAACCCTGTTGTTGGAGAAGAACGTGCGTGAGAGTAACTGTTCACGC

AGTGACGGTATCCAACCAGAAAGTCACGGCTAACTACGTG

>7174 len=160 tot-seq=1

TAGGGAATCTTCCACAATGGACGCAAGTCTGATGGAGCAACGCCGCGTGAGTGAAGAAGG

TTTTCGGATCGTAAAGCTCTGTTGTAAGAGAAAAACGACATTCATAGGGAATGATGAGTG

AGTGATGGTATCTTACCAGAAAGTCACGGCTAACTACGTG

>7175 len=160 tot-seq=1

TAGGGAATATTGCACAATGGAGGAAACTCTGATGCAGCCATGCCGCGTGTGTGAAGAAGG

CCTTCGGATCGTAAAGCTCTGTTGTAAGAGAAAAACGGCACTCATAGGGAATGATGAGTG

AGTGATGGTATCTTACCAGAAAGTCACGGCTAACTACGTG

>7176 len=160 tot-seq=1

TAGGGAATCTTCCACAATGGAGGGAACTCTGATGCAGCGACGCCGCGTGAATGAAGAAGG

CTTTCGAGTCGTAAAGCTCTGTTGGTAGTGAAGAAAGATAGAGGTAGTAACTGGCCTTTA

TTTGACGGTAATTACTTAGAAAGTCACGGCTAACTACGTG

>7177 len=160 tot-seq=1

TAGGGAATCTTCCACAATGGACGCAAGTCTGATGGAACAACGCCGCGTGAGTGAAGAAGG

GTTTCGGCTCGTAAAGCTCTGTTGTTGGTGAAGAAGGACAGGGGTAGTAACTGACCTTTG

TTTGACGGTATCTAACCAGAAAGCCACGGCTAACTACGTG

>7178 len=160 tot-seq=1

TGGGGAATATTGCACAATGGGCGCAAGCCTGATGCAGCAACGCCGCGTGAACGATGAAGG

TCTTCGGATCGTAAAACTCTGTTGTTAGAGAAGAACAAGGACGTTAGTAACTGAACGTCC

CCTGACGGTATCTAACCAGAAAGCCACGGCTAACTACGTG

>7179 len=160 tot-seq=1

TGGGGAATATTGCACAATGGGCGCAAGCCTGATGCAGCAACGCCGCGTGAACGATGAAGG
GTTTCGGCTCGTAAAACTCTGTTGTTGGAGAAGAACGTGCGTAAGAGTAACTGTTTACGC
AGTGACGGTATCCAACCAGAAAGTCACGGCTAACTACGTG
>7180 len=160 tot-seq=1
TAGGGAATATTGCACAATGGAGGAAACTCTGATGCAGCCATGCCGCGTGTGTGAAGAAGG
CCTTCGGGTTGTAAAACCCTGTTGTTAGAGAAGAAAGTGCGTGAGAGTAACTGTTCACGT
TTCGACGGTATCTAACCAGAAAGCCACGGCTAACTACGTG
>7181 len=160 tot-seq=1
TGGGGAATATTGCACAATGGGCGCAAGCCTGATGCAGCGACGCCGCGTGAGCGAAGAAGT
ATTTCGGTATGTAAAGCTCTGTTGTTGGTGAAGAAGGATAGAGGCAGTAACTGGCCTTTA
TTTGACGGTAATCAACCAGAAAGTCACGGCTAACTACGTG
>7182 len=160 tot-seq=1
TAGGGAATCTTCGGCAATGGGGGGAACCCTGACCGAGCAACGCCGCGTGAGTGAAGAAGG
TTTTCGGATCGTAAAGCTCTGTTGTTGGAGAAGAACGTGCGTGAGAGTAACTGTTCACGC
AGTGACGGTATCCAACCAGAAAGTCACGGCTAACTACGTG
>7183 len=160 tot-seq=1
TGGGGAATATTGCACAATGGGCGAAAGCCTGATGGAGCAACGCCGCGTGGGTGAAGAAGG
TCTTCGGATCGTAAAGCTCTGTTGTTGGTGAAGAAGGATAAAGGTAGTAACTGGCCTTTA
TTTGACGGTAATCAACCAGAAAGTCACGGCTAACTACGTG
>7184 len=160 tot-seq=1
TGGGGAATATTGCACAATGGGGGAAAGCCTGATGGAGCAACGCCGCGTGGGTGAAGAAGG
TCTTCGGATCGTAAAACCCTGTTGTTAGAGAAGAAGGATAGAGGCAGTAACTGGCCTTTA
TTTGACGGTAATCAACCAGAAAGTCACGGCTAACTACGTG
>7185 len=160 tot-seq=1
TGGGGAATCTTCCGCAATGGACGAAAGTCTGACGGAGCAACGCCGCGTGAGTGAAGAAGG
GTTTCGGCTCGTAAAACTCTGTTGTTGGAGAAGAACGTGCGTAAGAGTAACTGTTTACGC
AGTGACGGTATCCAACCAGAAAGTCACGGCTAACTACGTG
>7186 len=160 tot-seq=1
TAGGGAATTTTTCACAATGGGCGCAAGCCTTATGAAGCAATGCCGCGTGGGTGAAGAAGG

TCTTCGGATCGTAAAACCCTGTTGTTAGAGAAGAAAGTGCGTGAGAGTAACTGTTCACGT

TTCGACGGTATCTAACCAGAAAGCCACGGCTAACTACGTG

>7187 len=160 tot-seq=1

TGGGGAATATTGCACAATGGGGGAAACCCTGATGCAGCGACGCCGCGTGAGCGAAGAAGT

ATTTCGGTATGTAAAGCTCTGTTGTTGGAGAAGAACGTGCGTGAGAGTAACTGTTCACGC

AGTGACGGTATCCAACCAGAAAGTAACGGCTAACTACGTG

>7188 len=160 tot-seq=1

TAGGGAATCTTCGGCAATGGACGGAAGTCTGACCGAGCAACGCCGCGTGAGTGAAGAAGG

TTTTCGGATCGTAAAGCTCTGTTGTAAGTGAAGAACGGCTCATAGAGGAAATGCTATGGG

AGTGACGGTAGCTTACCAGAAAGCCACGGCTAACTACGTG

>7189 len=160 tot-seq=1

TAGGGAATCTTCCACAATGGGCGAAAGCCTGATGGAGCAACGCCGCGTGGGTGAAGAAGG

GTTTCGGCTCGTAAAGCTCTGTTGTTGGTGAAGAAGGACAGGGGTAGTAACTGACCTTTG

TTTGACGGTAATCAATTAGAAAGGGACGGCTAACTACGTG

>7190 len=160 tot-seq=1

TGGGGAATATTGCACAATGGACGCAAGTCTGATGGAGCAACGCCGCGTGAGTGAAGAAGG

TTTTCGGATCGTAAAGCTCTGTTGTTGGTGAAGAAGGATAGAGGCAGTACCTGGCCTTTA

TTTGACGGTAATCAACCAGAAAGGGACGGCTAACTACGTG

>7191 len=160 tot-seq=1

TGGGGAATTTTGGACAATGGGGGAAACCCTGATCCAGCCATGCCGCGTGCGGGATGAAGG

CCTTCGGGTTGTAAAGTACTTTCAGCGGGGAGGAAGGGAGTAAAGTTAATACCTTTGCTC

ATTGACGTTACCCGCAGAAGAAGCACCGGCTAACTCCATG

>7192 len=160 tot-seq=1

TAGGGAATCTTCCACAATGGGCGAAAGCCTGATGGAGCAACGCCGCGTGGGTGAAGAAGG

GTTTCGGCTCGTAAAACTCTGTTGTTGAAGAAGAACGTGCGTAAGAGTAACTGTTCACGC

AGTGACGGTATTCAACCAGAAAGTCACGGCTAACTACATG

>7193 len=160 tot-seq=1

TGGGGAATATTGCACAATGTGCGCAAGCCTGATGGAGCAACACCGCGTGAGTGAAGAAGG

GTTTCGGCTCGTAAAACTCTGTTGTCGGAGAAGAACGTGCGTAAGAGTAACTGTTTACGC

AGTGACGGTATCCAACCAGAAAGTCACGGCTAACTACATG

>7194 len=160 tot-seq=1

TGGGGAATATTGCACAATGGAGGGAACTCTGATGCAGCGACGCCGCGTGAACGAAGAAGG

CTTTCGGCTCGTAAAACTCTGTTGTTGGAGAAGAACGTGCGTAAGAGTAACTGTTTACGC

AGTGACGGTATCCAACCAGAAAGCCACGGCTAACTACGTG

>7195 len=160 tot-seq=1

TGGGGAATATTGGACAATGGGCGCAAGCCTGATCCAGCCATGCCGCGTGAGTGATGAAGG

CCTTAGGGTTGTAAAGTTCTTTTATATGGGAAGAAACGCTAAGATAGGAAATGATTTTAG

TTTGACTGTACCATTTGAATAAGTATCGGCTAACTATGTG

>7196 len=160 tot-seq=1

TAGGGAATCTTCCGCAATGGACGCAAGTCTGACGGAGCAACGCCGCGTGAGTGAAGAAGG

GTTTCGGCTCGTAAAACTCTGTTGTTAAGGAAGAATGACACATGGAGTAACTGCCATGTG

TGTGACGGTACTTAACCAGAAAGCCACGGCTAACTACGTG

>7197 len=160 tot-seq=1

TAGGGAATCTTCGGCAATGGGCGCAAGCCTGACGGAGCAATGCCGCGTGAGTGAAGAAGG

CCTTCGGGTTGTAAAACTCTGTCCTCATCCAAGAGAAGGAAAGGTAGTAACTGGCCTTTC

CAGGACGGTGGATGAGGAGGAAGCCCCGGCTAACTATGTG

>7198 len=160 tot-seq=1

TGGGGAATTTTGGACAATGGGCGCAAGCCTGATCCAGTAATGCCGCGTGCAGGATGAAGG

CCTTCGGGTTGTAAACTGCTTTTGTACGGAACGAAAAGCTCTGGGCTAATACCTCGGAGT

CATGACGGTACCGTAAGAATAAGCACCGGCTAACTACGTG

>7199 len=160 tot-seq=1

TAGGGAATCTTCCGCAATGGACGCAAGTCTGACGGATCAACGCCGCGTGTGTGAAGAAGG

TCTTCGGATCGTAAAGCACTGTTGTTAGAGAAGAACGACCGGTAGAGTAACTGTTACCGG

AGCGACGGTATCTAACCAGAAAGCCACGGCTAACTACGTG

>7200 len=160 tot-seq=1

TGGGGAATATTGGACAATGGGCGCAAGCCTGATCCAGCCATGCCGCGTGGGTGAAGAAGG

CCTTCGGGTTGTAAAGCCCTTTTGTTGGCAAAGAAAAGCTTGCGGTTAATACCCGTGAGT

CATGACGGTACCCAAAGAATAAGCACCGGCTAACTTCGTG

>7201 len=160 tot-seq=1

TGGGGAATATTGCACAATGGGGGAAACCCTGATGCAGCGACGCCGCGTGAACGAAGAAGG

TATTCGTATCGTAAAGTTCTGTTGGTAGTGAAGAAAGATAGAGGTAGTAACTGGCCTTTA

TTTGACGGTAATTACTTAGAAAGTCACGGCTAACTACGTG

>7202 len=160 tot-seq=1

TGGGGAATATTGCACAATGGGGGAAACCGTGATGCAGCCATGCCGCGTGTGTGAAGAAGG

CCTTCGGGTTGTAAAGCACTTTCAGTAGGGAGGAAAGGGTAAGGTTTAATACGCCTTATC

TGTGACGTTACCTACAGAAGAAGGACCGGCTAACTCCGTG

>7203 len=160 tot-seq=1

TGGGGAACTTTGGACAATGGGCGCAAGCCTGATCCAGCAATGCCGCGTGCTGGATGAAGG

CCTTCGGGTTGTAAACTGCTTTTGTACAGAACGAAAAGTCTTGGGTTAATACCCTGGGAT

CATGACGGTACCGTAAGAATAAGCACCGGCTAACTACGTG

>7204 len=160 tot-seq=1

TAGGGAATCTTCCACAATGGACGCAAGTCTGATGGAGCAACGCCGCGTGAGTGAAGAAGG

CCTTCGGGTTGTAAAGCACTTTTGTCCGGAAAGAAATCGCGCTGGTTAATACCTGGCGTG

GATGACGGTACCGGAAGAATAAGCACCGGCTAACTACGTG

>7205 len=160 tot-seq=1

TAGGAAATCTTCCACAATGGACGCAAGTCTGATGGAGCAACGCCGCGTGAGTGAAGAAGG

TTTTCGGATCGTAAAACTCTGTTGTAAGAGGGGAACAAATTGTAGAGTAACTGCTACAGT

CTTGACGGTATCTTACCAGAAAGCCACGGCTAACTACGTG

>7206 len=160 tot-seq=1

TAGGGAATTTTCGGCAATGGGGGCAACCCTGACCGAGCAACGCCGCGTGAGTGAAGACGG

CCTTCGGGTTGTAAAACTCTGTTGTAAGAGAAGAACAAATTGTAGAGTAACTGCTACAGT

CTTGACGGTATCTTACCAGAAAGCCACGGCTAACTACGTG

>7207 len=160 tot-seq=1

TGGGGAATCTTCCGCAATGGGCGAAAGCCTGACGGAGCAACGCCGCGTGAGTGAAGACGG

CCTTCGGGTTGTAAAGCTCTGTTGTTGGTGAAGAAGGACAGGGGTAGTAACTGACCTTTG

TTTGATGGTAATCAATTAGAAAGTCACGGCTAACTACGTG

>7208 len=160 tot-seq=1

TAGGGAATTTTCGTCAATGGGGGGAACCCTGAACGAGCAATGCCGCGTGAGTGAGAAAGG

TCTTCGGATCGTAAAACTCTGTTGTTAGAGAAGAACAGCGCATAGAGTAACTGTTATGCG

TGTGACGGTATCTAACCAGAAAGCCACGGCTAACTACGTG

>7209 len=160 tot-seq=1

TGGGGAATATTGCACAATGGGCGCAAGCCTGATGCAGCGACGCCGCGTGAGGGATGGAGG

CCTTCGGGTTGTAAACTGCTTTTGTCAGGGACGAAAAGGACCGTGTTAATACCATGGTCT

GCTGACGGTACCTGAAGAATAAGCACCGGCTAACTACGTG

>7210 len=160 tot-seq=1

TGGGGAATATTGCACAATGGGGGAAACCCTGATGCAGCGACGCCGCGTGAAGGAAGAAGT

AATTCGTTATGTAAACTTCTGTCATAGAGGAAGAGCGGTGGATATAGGGAATGATATCCA

AGTGACGGTACTCTATAAGAAAGCCACGGCTAACTACGTG

>7211 len=160 tot-seq=1

TGGGGAATCTTCCGCAATGGACGAAAGTCTGACGGAGCAACGCCGCGTGAGTGATGACGG

CCTTCGGGTTGTAAACTGCTTTTGTCAGGGACGAAAAGGACCGTGTTAATACCATGGTCT

GCTGACGGTACCTGAAGAATAAGCACCGGCTAACTACGTG

>7212 len=160 tot-seq=1

TGGGGAATATTGGACAATGGGGGGAACCCTGATCCAGCCATGCCGCGTGTGTGAAGAAGG

CCTTATGGTTGTAAAGCACTTTCAGCGGGGAGGAAGGGAGTAAAGTTAATACCTTTGCTC

ATTGACGTTACCCGCAGAAGAAGCACCGGCTAACTCCGTG

>7213 len=160 tot-seq=1

TGAGGAATATTGGTCAATGGACGAGAGTCTGAACCAGCCAAGTAGCGTGCAGGATGAAGG

CCTTCGGGTTGTAAACTGCTTTTGTCAGGGACGAAAAGGACCGTGTTAATACCATGGTCT

GCTGACGGTACCTGAAGAATAAGCACCGGCTAACTACGTG

>7214 len=160 tot-seq=1

TGAGGAATATTGCACAATGGAGGAAACTCTGATGCAGCGACGCCGCGTGAAGGATGAAGT

ATTTCGGTATGTAAAGTGCTGTTATAAGGGAAGAACATTTGCAATAGGAAATGATTGCAG

ACTGACGGTACCTTGTCAGAAAGCGATGGCTAACTATGTG

>7215 len=160 tot-seq=1

TGGGGAATATTGCACAATGGGCGAAAGCCTGATGCAGCAACGCCGCGTGAGCGATGAAGG

CCTTCGGGTCGTAAAGCTCTGTTGTTAAAGAAGAACGTGGGTAAGAGTAACTGTTTACCC

AGTGACGGTATTTAACCAGAAAGCCACGGCTAACTACGTG

>7216 len=160 tot-seq=1

TGGGGAATATTGCACAATGGGGGAAACCCTGATGCAGCGACGCCGCGTGAGTGAAGAAGT

ATCTCGGTATGTAAAGCTCTGTTGTTAAAGAAGAACGTGGGTAAGAGTAACTGTTTACCC

AGTGACGGTATTTAACCAGAAAGCCACGGCTAACTACGTG

>7217 len=160 tot-seq=1

CAAGCAGAAGACGGCATACGAGATATTGGCGTGACTGGAGTTCAGACTGTGCTCTTCCGA

TCTCCTAGGGAGGCAGCAGCCAACGGCTTGACTTCTCTGGCCTCTGAGGCCACAACTGGG

CACTCCCTGCTTTCTCTCCATCTCTCACATCCACGGTCCC

>7218 len=160 tot-seq=1

TGGGGAATATTGGGCAATGGGCGCAAGCCTGACCCAGCAACGCCGCGTGAAGGAAGAAGG

CTTTCGGGTTGTAAAGTACTTTCAGCGGGGAGGAAGGGAGTAAAGTTAATACCTTTGCTC

ATTGACGTTACCCGCAGAAGAAGCACCGGCTAACTCCATG

>7219 len=160 tot-seq=1

TGGGGAATCTTCTGCAATGGACGAAAGTCTGACGGAGCAACGCCGCGTGAGTGATGACGG

CCTTCGGGTTGTAAAGCTCTGTTAATCGGGACGAATGGTCCTGTGTGAATAATGCAAGGA

TTTGACGGTACCGGAGTAGAAAGCCACGGCTAACTACGTG

>7220 len=160 tot-seq=1

CTAAGAATATTCCGCAATGGGCGAAAGCCTGACGGAGCGACGCCGCGTGGATGATGAAGG

CCGGAAGGTTGTAAAGTCCTTTTATGACAGAGGAATAACCGGGGCAGGGAATGGCACCGG

GGTGACTGTAGGTCATGAATAAGCACCGGCTAATTACGTG

>7221 len=160 tot-seq=1

CAGGGAATCTTCCACAATGGACGAAAGTCTGATGGAGCAACGCCGCGTGAGTGAAGAAGG

TTTTCGAATCGTAAAGCTCTGTTGTTGGTGAAGAAGGATAGAGGTAGTGACTGGCCTTTA

CTTGACGGTAATCAACCAGAAAGTCACGGCTAACTACGTG

>7222 len=160 tot-seq=1

TAGGGAATCTTCCACAATGGACGAAAGTCTGGTGGAGCAACGCTGCGTGAGTGAAGAAGG

TTTTCGGATCGTAAAGCTCTGTTGTTGGTGAAGAAGGATAGAGGCAGTAACTGGCCTTTG

TTTGACGGTAATCAATTAGAAAGTCACGGCTAACTACGTG

>7223 len=160 tot-seq=1

TAGGGAATCTTCCACAATGGACGAAAGTCTGATGGAGCAACGCCGCGTGAGTGAAGAAGG

TTTTCGGATCGTAAAACTCTGTTATCAGGGAAGAACAAACGTGTAAGTAACTGTGCACGT

CTTGACGGTACCTGATCAGAAAGCCACGGCTAACTACGTG

>7224 len=160 tot-seq=1

TAGGGAATTTTCGGCAATGGGGGACACCCTGACCGAGCAACGCCGCGTGAATGATGAAGT

ATTTCGGTATGTAAAATTCTTTTGTAGAGGACGAATGGCACTATCAGGAAATGGGTAGTG

AATGACGGTACTTTACGAATAAGCTCCGGCTAACTACATG

>7225 len=160 tot-seq=1

TAGGGAACCTTCCACAATGGACGAAAGTCTGATGGAGCAACGCCGCGTGAGTGTAGAAGG

TCTTCGGATCGTAAAGCTCTGTTGTTGGTGAAGAAGGATAGAGGTAGTAACCGGCCTTTA

TTTGACGGTAATCAACCAGAAAGTCACGGCTAACTACGTG

>7226 len=160 tot-seq=1

TAGGGAATTTTCGGCAATGGGGGGAACCCTGACCGAGCAACGCCGCGTGAGTGAGGAAGG

CCTTCGGGCTGTAAAGCTCTGTTGCAGAGGAAGAACGGGAGAGCGAGGAAATGCGCTTTT

CGTGACGGTACTCTGCGAGGAAGCCACGGCTAACTACGTG

>7227 len=160 tot-seq=1

TGGGGAATATTGGACAATGGGCGAAAGCCTGATCCAGCCATGCCGCGTGTGTGAAGAAGG

CTCTAGGGTTGTAAAGCACTTTCAGTGGGGAGAAAGGTCGTTCACTTAATACGTGAATGA

ATTGATGTTACCCACAGAAGAAGCACCGGCTAACTTCGTG

>7228 len=160 tot-seq=1

TAGGGAATCTTCCACAATGGGCGCAAGCCTGATGGAGCAACGCTGCGTGTGTGATGAAGG

GTTTCGGCTCGTAAAACACTGTTATAAGAGAAGAACGGCACTGAGAGTAACTGTTCAGTG

TGTGACGGTATCTTACCAGAAAGGAACGGCTAAATACGTG

>7229 len=160 tot-seq=1

TAGGGAATTTTCGGCAATGGGGGAAACCTTGACCGAGCAATGCCGCGTGAATGATGAAGG

TCTTCGGATCGTAAAGTTCTGTTGTTAAGGAAGAACTATGGGATTAGGAAATGAGTTCCA

TTTGACGGTACTTAACTAGAAAGCCCCGGCTAACTATGTG

>7230 len=160 tot-seq=1

TGAGGAATTTTTCACAATGGACGAAAGTCTGATGGAGCAATACCACGTGAGTGAAGAAGG

TTTTCGGATCGTAAAGCTCTGTTGTTGGTGAAGAAGGATAGAGGTAGTAACTGGCCTTTA

TTTGACGGTAATCAACCAGAAAGTCACGGCTAACTACGTG

>7231 len=160 tot-seq=1

TGGGGAATATTGCGCAATGGGGGAAACCCTGACGCAGCGACGCCGCGTTCGGGATGAAGG

CCTTCGGGTTGTAAAGCTCTGTTGTTAGAGAAGAACGTGCGTGAGAGCAACTGTTCACGC

AGTGACGGTATCTAACCAGAAAGTCACGGCTAACTACGTG

>7232 len=160 tot-seq=1

TAGGGAATCTTCCGCAATGGACGCAAGTCTGACGGAGCAACGCCGCGTGAGTGAAGAAGG

TTTTCGGATCGTAAAACTCTGTTGTCGGAGAAGAACACGTAAGCGAGTAACTGCGCTTAC

CATGACGGTACCCGACCAGAAAGCCACGGCTAACTACGTG

>7233 len=160 tot-seq=1

TAGGGAGTATTCGGCAATGGGGGAAACCCTGACCGAGCAACGCCGCGTGAATGATGACGG

CCTTCGGGTTGTAAAATTCTGTGGCGAGGAACGAATGGCAAGGAGAGGAAATGCTCTTTG

TTTGACGGTACCTCGCTAGAAAGCCACGGCTAACTACGTG

>7234 len=160 tot-seq=1

TGGGGAATTTTGGACAATGGGCGCAAGCCTGATCCAGCCATTCCGCGTGCAGGATGAGGG

CCTTCGGGTTGTAAACTGCTTTTGTACGGAACGAAAAGGCTTTCTCTAATACAGGGAGTC

CATGACGGTACCGTAAGAATAAGCACCGGCTAACTACGTG

>7235 len=160 tot-seq=1

TAGGGAATTTTCGTCAATGGGGGAAACCCTGAACGAGCAATGCCGCGTGAGTGAAGAAGG

TTTTCGGATCGTAAAGCTCTGTTGTTGGTGAAGAAGGATAGAGGTAGTAACTGGCCTTTA

TTTGACGGTAATCAACCAAAAAGTCACGGCTAACTACGTG

>7236 len=160 tot-seq=1

TCGAGGATCATTCGCAATGGGGGGAACCCTGACGATGCGACGCTGCGTGGAGGATGAAGG

CCCTCGGGTCGTAAACTCCTGTCATGACGGAAAAGGAGAGGGGACCCAACACGTCCCTTA

TTTGATGGTACGTCAAGAGGAAGAGACGGCTAACTCTGTG

>7237 len=160 tot-seq=1

TCGAGGATCATTTGCAATGGGCGAAAGCCTGACAATGCGACGCTGCGTGGAGGATGGAGG

CCCTCGGGTTGTAAACTCCTGTCATGGGGGAAGAGGCGGACCGGCCCAATACGCCGGCGA

GTTGACTGTACCCCAAGAGGAAGCGACGGCTAACTCTGTG

>7238 len=160 tot-seq=1

TTAGGAATATTCGTCAATGGGGGAAACCCTGAACGAGCAATGCCGCGTGAGTGATGAAGG

TCTTTGGATTGTAAAACTCTGTTGTATGGAAAGAATGGCTAAAATAGGAAATGATTTTAG

TTTGACGGTACCATGCCAGAAAGCCCCGGCTAACTACGTG

>7239 len=160 tot-seq=1

TGGGGAATATTGGGCAATGGGCGCAAGCCTGACCCAGCAACGCCGCGTGAAGGAAGAAGG

CTTTCGGGTTGTAAAGCTCTGTTGTTGGTGAAGAAGGACAGGGGTAGTAACTGACCTTTG

TTTGACGGTAATCAATTAGAAAGTCACGGCTAACTACGTG

>7240 len=160 tot-seq=1

TAGGGAATCTTCCACAATGGACGCAAGTCTGATGGAGCAACGCCGCGTGAGTGAAGAAGG

GTTTCGGCTCGTAAAGCTCTGTTGTTGGAGAAGAACGTGCGTGAGAGTAACTGACCTTTG

TTTGACGGTAATCAATTAGAAAGTCACGGCTAACTACGTG

>7241 len=160 tot-seq=1

GCGGCTGTACCTTCCGGACGCAGGGTCAGGGAGTCACCGTTGCGATCTTCAAAGGTGTAC

ATCTCTTTTTCGACGATGTCGGTCACTTCACCGATGGAGCGCTTGAACAGCTCGGTGAAC

TCGACGATCGGCATGCGGATCTGTTTGTAACCGTAGTTAT

>7242 len=160 tot-seq=1

TGGGGAATATTGGGCAATGGGCGGAAGCCTGACCCAGCCACGCCGCGTGAGTGATGAAGG

CCTTCGGGTTGTAAAGCTCTGTGGGAAGGGGCGAAAAACTGCGAAGAGAATATCTAGCAG

GTTGACGGTACCTTCTTAGCAAGCACCGGCTAACTCTGTG

>7243 len=160 tot-seq=1

TAGGGAATCTTCCGCAATGGACGCAAGTCTGACGGAGCAACGCCGCGTGTGTGAAGAAGG

TCTTCGGATCGTAAAGCACTGTTATTAGAGAAGAACACCCTGTAGAGTAACTGGCCTTTA

TTTGACGGTAATCAACCAGAAAGTCACGGCTAACTACGTG

>7244 len=160 tot-seq=1

TGGGGAATTTTGGACAATGGGGGCAACCCTGATCCAGCCATCCCGCGTGTGCGATGAAGG

CCTTCGGGTTGTAAAGCACTTTTGGCAGGAAAGAAACGGCGCCGGCTAATACCTGGCGCT

AATGACGGTACCTGCAGAATAAGCACCGGCTAACTACGTG

>7245 len=160 tot-seq=1

TGGGGAATATTGCACAATGGTCGAAAGCCTGATGCAGCAACGCCGCGTGAGTGATGAAGG

CCTTCGGGTCGTAAAGCTCTGTTGTTGGTGAAGAAGGACAGGGGTAGTAACTGACCTTTG

TTTGACGGTAATCAATTAGAAAGTCACGGCTAACTACGTG

>7246 len=160 tot-seq=1

TAGGGGATCTTCCACAATGGACGCAAGTCTGATGGAGCAACGTCGCGTGAGTGAAGAAGG

GTTTTGGCTCGTAAAGCTCTGTTGCTGGTGAAGAAGGACGGGGGTAGTAACTGACCTTTG

TTTGACGGTAATCAATTAGAAAGTCGCGGCTAACTACGTG

>7247 len=160 tot-seq=1

CAGTGAGCTGAGATTGCGCCATTGCACTCTAGTCTGGGTGACAAAGCAAGACTCCGTCTC

AAAAAAAAAAAAGAAAAGAAAGAAAATGTATGGAGAACAGTGCAAATTGTAAAACTGCAT

GCAAACCTTAGTTACTAGTTAATAATGCTCTCTGATATAC

>7248 len=160 tot-seq=1

CAGAGGTTGCAGTGAGCCGAGATCGTGCCACTGCACTTCAGCCTGGGGGACAGAGCAAGA

CTCCATTTCAAAAAATAAAAAATAAACATAAATAAAAAATAAAAACTGCATCGCCCAGCC

TCCTTTACAGAAATGGACAGCCATGTGACTAAGATCTGGA

>7249 len=160 tot-seq=1

TGGGGAATATTGGACAATGGGCGAAAGCCTGATCCAGCCATGCCGCGTGTGTGAAGAAGG

CCTTATGGTTGTAAAGCACTTTAAGTGGGGAGGAAAAGCTTATGGATAACACCCATAGGT

TCTGACGTTACCCACAGAATAAGCACCGGCTAACTCTGTG

>7250 len=160 tot-seq=1

TGGGGAATCTTGCACAATGGGCGAAAGCCTGATGCAGCGACGCCGCGTGCGGGATGAAGG

CCTTCGGATCGTAAAACTCTGTTGTAAGAGAAGAACAAATTGTAGAGTAACTGCTACAGT

CTTGACGGTATCTTACCAGAAAGCCACGGCTAACTACGTG

>7251 len=160 tot-seq=1

TGGGGAATCTTGCACAATGGGCGAAAGCCTGATGCAGCGACGCCGCGTGAGTGAAGAAGG

GTTTCGGCTCGTAAAACTCTGTTGTTGAAGAAGAACGTGCGTGAGAGTAACTGTTCACGC

AGTGACGGTATTCAACCAGAAAGTCACGGCTAACTACGTG

>7252 len=160 tot-seq=1

TGGGGAATATTGGACAATAGGCGCAAGCCTGATCCAGCCATGCCGCGTGAGTGAAGAAGG

CCTTCGGGTTGTAAAGCTCTTTTGTCCGGAAAGAAAAGCTGCCGGTTAATACCCGGTGGT

TATGACGGTACCGGAAGAATAAGCACCGGCTAACTTCGTG

>7253 len=160 tot-seq=1

TAGGGAATTTTTCACAATGGGCGCAAGCCTTATGAAGCAATGCCGCGTGAACGATGAAGG

TTTTCGGATCGTAAAGCTCTGTTGTTGGTGAAGAAGGATAGAGGTAGTAACTGGCCTTTA

TTTGACGGTAATCAACCAGAAAGTCACGGCTAACTACGTG

>7254 len=160 tot-seq=1

TGGGGAATATTGCGCAATGGGCGCAAGTCTGACGGAGCAATGCCGCGTGAGTGAAGAAGG

CCTTCGGGTTGTAAAACTCTGTCCTCATCCAAGAGAAGGAAAGGTAGTAACTGGCCTTTC

CAGGACGGTAGATGAGGAGGAAGCCCCGGCTAACTATGTG

>7255 len=160 tot-seq=1

TGGGGAATATTGCACAATGGGCGCAAGCCTGATGCAGCCATGCCGCGTGTATGAAGAAGA

CCTTCGGGTTGTAAAGTACTTTCAGCGGGGAGGAAGGGAGTAAAGTTAATACCTTTGCTC

ATTGACGGTAATCAATTAGAAAGTCACGGCTAACTACGTG

>7256 len=160 tot-seq=1

TAGGGAATCTTCCACAATGGACGAAAGTCAGATGGAGCAACGCCGCGTGAGTGAAGAAGG

TTTTCGGATCGTAAAGCTCTGTTGGTAGTGAAGAAAGATGGAGGTAGTAACTGGCCTTTA

TTTGACGGTAATTACTTAGAAAGTCACGGCTAACTACGTG

>7257 len=160 tot-seq=1

TGGGGAATTTTGGACAATGGACGCAAGTCTGATCCAGCCATTCCGCGTGCAGGATGAAGG

CCCTCGGGTTGTAAACTGCTTTTGTACAGAACGAAAAGGTCCTGGTTAATACCTGGGGCT

CATGACGGTACTGTAAGAATAAGCACCGGCTAACTACGTG

>7258 len=160 tot-seq=1

TCGGGAATATTGCACAATGGAGGAAACTCTGATGCGGTGACGCCGCGTATAGGAAGAAGG

TTTTCGGATCGTAAAGCTCTGTTGTTGGTGAAGAAGGATAGAGGTAGTAACTGGCCTTTA

TTTGACGGTAATCAACCAGAAAGTCACGGCTAACTACGTG

>7259 len=160 tot-seq=1

TCGAGAATCTTTCGCAATGGGCGCAAGCCTGACGAAGCGACGCCGTGTGAGCGAAGAAGG

CCTTCGGGTTGTAAAGCTCTTTCGCTAGGGAACAAGAGAGATAGGATAACACCCTATTGA

TTTGATGGTACTTGGTAAAGAAGCACCGGCTAACTCCGTG

>7260 len=160 tot-seq=1

TGGGGAATTTTGGACAATGGGCGCAAGCCTGATCCAGCCATGCCGCGTGAGTGAAGAAGG

CCTTCGGGTTGTAAAGCTCTTTCGGCAGGAACGAAATGGTTCGTGCTAATACCACGGACT

GATGACGGTACCTGAAGAAGAAGCACCGGCTAACTACGTG

>7261 len=160 tot-seq=1

TGGGGGATATTGCACAATGGAGGCAACTCTGATGCAGCAACGCCGCGTGAGTGAAGAAGG

CCTTCGGGTTGTAAAACTCTGTCCTCATCCAAGAGAAGGGAAGGTAGTAACTGACCTTTC

CAGGACGGTAGATGAGGAGGAAGCCCCGGCTAACTATGTG

>7262 len=160 tot-seq=1

TGGGGAATATTGCACAATGGACGAAAGTTTGATGCAGCGACGCCGCGTGGAGGGTGTAGG

CCTTCGGGTTGTAAAGTACTTTCAGCGGGGAGGAAGGGAGTAAAGTTAATACCTTTGCTC

ATTGACGTTACCCGCAGAAGAAGCACCGGCTAACTCCGTG

>7263 len=160 tot-seq=1

TGGGGAATATTGCGCAATGGGGGAAACCCTGACGCAGCGACGCCGCGTGAGTGAAGAAGG

CCTTCGGGTTGTAAAACTCTGTCTTCATCCAAGAGAAGGGAAGGTAGTAACTGACCTTTC

CAGGACGGTAGATGAGGAGGAAGCCCCGGCTAACTATGTC

>7264 len=160 tot-seq=1

TAGGGAATCTTCGGCAATGGACGAAAGTCTGACCGAGCAACGCCGCGTGAGTGAAGAAGG

TTTTCGGATCGTAAAACTCTGTTGTTAGAGAAGAACAAGGACGTTAGTAACTGAACGTCC

CCTGACGGTAATTACTTAGAAAGTCACGGCTAACTACGTG

>7265 len=160 tot-seq=1

TAGGGAATCTTCCGCAATGGACGCAAGTCTGACGGAGCAACGCCGCGTGAGTGAAGAAGG

ATTTCGGTTCGTAAAACTCTGTTGGTAGTGAAGAAAGATAGAGGTAGTAACTGGCCTTTA

TTTGACGGTAATTACTTAGAAAGTCACGGCTAACTACGTG

>7266 len=160 tot-seq=1

TAGGGAATTTTTCACAATGGGCGCAAGCCTTATGAAGCAATGCCGCGTGTGTGAAGAAGG

CCCTCGGGTTGTAAAGCACTTTCAGTGAGGAAGAACGCCTGGTGGTTAATACCCATCAGG

AAAGACATCACTCACAGAAGAAGCACCGGCTAACTCCGTG

>7267 len=160 tot-seq=1

TAGGGAATCTTCCGCAATGGACGCAAGTCTGACGGAGCAACGCCGCGTGAGTGAAGAAGG

ATTTCGGTTCGTAAAACTCTGTTGTTAGAGAAGAACAGCGCATAGAGTAACTGTTATGCG

TGTGACGGTACCTGCAGAAGAAGCCCCGGCTAACTACGTG

>7268 len=160 tot-seq=1

TGGGGAATATTGCGCAATGGGGGAAACCCTGACGCAGCGACGCCGCGTGAGTGAAGAAGG

ATTTCGGTTCGTAAAGCTCTGTTGTTAGGGAAGAATGATTGTGTAGTAACTATACACAGT

AGAGACGGTACCTAACCAGAAAGCCACGGCTAACTACGTG

>7269 len=160 tot-seq=1

TGGGGAATATTGCACAATGGGCGCAAGCCTGATGCAGCAACGCCGCGTGTGTGAAGAAGG

TCTTCGGATCGTAAAGCACTGTTGTTAGAGAAGAACGACCGCTAGAGTAACTGTTAGCGG

AGTGACGGTATCTAACCAGAAAGCCACGGCTAACTACGTG

>7270 len=160 tot-seq=1

TCGAGGATCATTTGCAATGGGCGAAAGCCTGACAATGCGACGCTGCGTGGAGGATGAAGG

CCTTCGGGTTGTAAACTCCTGTCATAAGGGAACAGGCGAGGATACCCAATACGTATCCGA

GTTGAGTGTACTTTAAGAGGAATTGGCGGCTAACTCTGTG

>7271 len=160 tot-seq=1

TGGGGAATTTTGGACAATGGGCGCAAGCCTGATCCAGCAATGCCGCGTGAGTGAAGAAGG

CCTTCAGGTTGTAAAGCTCTTTTGTCAGGGAAGAAACGGGATTGGATAATACCTAGTCCT

AATGACGGTACCTGAAGAATAAGCACCGGCTAACTACGTG

>7272 len=160 tot-seq=1

TGGGGAATTTTGGACAATGGGCGCAAGCCTGATCCAGCCATGCCGCGTGTCTGAAGAAGG

CCTTCGGGTTGTAAAGGACTTTTGTTCGGGAGGAAATCCCGCTGGTTAATACCTGGCGGG

GATGACAGTACCGGAAGAATAAGCACCGGCTAACTACGTG

>7273 len=160 tot-seq=1

TGGGGAATATTGGACAATGGGGGAAACCCTGATCCAGCCATGCCGCGTGTGTGAAGAAGG

CCTTTTGGTTGTAAAGCACTTTAAGCAGTGAAGAAGACTCTTCGGTTAATACCCGGAGAC
GATGACATTAGCTGCAGAATAAGCACCGGCTAACTCTGTG
>7274 len=160 tot-seq=1
TGAGGAATATTGGTCAATGGGCGCTAGCCTGAACCAGCCAAGTAGCGTGTATGAAGAAGG
CCTTCGGGTTGTAAAGTACTTTCAGCGGGGAGGAAGGGAGTAAAGTTAATACCTTTGCTC
ATTGACGTTACCCGCAGAAGAAGCACCGGCTAACTCTGTG
>7275 len=160 tot-seq=1
TGGGGAATATTGGACAATGGAGGTAACTCTGATCCAGCAATTCTGTGTGTGTGAAGAAGG
TTTTAGGACTGTAAAGTTCTTTTATATGGGAAGAAATGCTAAAATAGGAAATGATTTTAG
TTTGACTGTACCATTTGAATAAGTATCGGCTAACTATGTG
>7276 len=160 tot-seq=1
TGGGGAATATTGCACAATGGGCGCAAGCCTGATGCAGCCATGCCGCGTGTATGAAGAAGG
CCTTCGGGTTGTAAAGTACTTTCAGTCGGGAGGAAGGTGGTAAGGTTAATACCTTTGCTC
ATTGACGTTACCCGCAGAAGAAGCACCGGCTAACTCCGTG
>7277 len=160 tot-seq=1
TGGGGAATATTGCACAATGGGGGAAACCCTGATGCAGCAACGCCGCGTGAGTGATGAAGG
TTTTCGGATCGTAAAGTACCTTCAGCGGGGAGGAAGGGAGTAAAGTTAATACCTTTACTC
ATTGACGTTACCCGCAGAAGAAGCACCGGCTAACTCCGTG
>7278 len=160 tot-seq=1
ACAGTTCCATGTGAATTTGTTTAATGTAAATACATCCTCTATTTATCTTTGGCTGTTAAT
AAATTCATGCATTATTAGAAAATCCCTCATACCAGCAGACCCGCTGAGTTAGATCAGTTC
GTTTTAAGAGATTTCCTGTTGGCAGGATTCAAAGCCCACA
>7279 len=160 tot-seq=1
TAGGGAGTTTTCGGCAATGGGGGAAACCTTGACCGAGCAACGCCGCGTGAGTGATGAAGG
TCTTCGGATCGTAAAGCTCTGTTACGAGGGACGAAGGACCGGCTCAGGAAATGGGGCCGG
TTTGACGGTACCTCGCCAGAAAGTGACGGCTAACTACGTG
>7280 len=160 tot-seq=1
TCGAGGATCATTTGCAATGGGCGAAAGCCTGACAATGCGACGCTGCGTGGAGGATGAAGG
CCTTCGGGTTGTAAACTCCTGTCATGGGGGAACAGGCGAGGGGACCCAACACGTCCCTTA

GTTGAGTGTACCCCAAGAGGAAGCGACGGCTAACTCTGTG

>7281 len=160 tot-seq=1

TTAGGAATATTCGTCAATGGAGGAAACTCTGAACGAGCAATGCCGCGTGAGTGATGAAGG

TCTTTGGATTGTAAAACTCTGTTGTATGTGAAGAACGACTAAATAAGGAAATGTATTTAG

AGTGACGGTAGCATACCAGAAAGCCCCGGCTAACTACGTG

>7282 len=160 tot-seq=1

TAGGGAATCTTCCACAATGGACGCAAGTCTGATGGAGCAACGCCGCGTGAGTGAAGAAGG

GTTTCGGCTCGTAAAGCTCTGTTGTTGGAGAAGGGGGACAGGGGGAGTAACTGACCTTTG

TTTGACGGTAATCAATTAGAGAGTCACGGGGAACTACGTG

>7283 len=160 tot-seq=1

TTTTTTTTTTCAAGCAGAAGACGGCATACGAGATTTATCCGTGACTGTAGTTCAGACGTG

TGCTCTTCCGATCTCATACGGGAGGCAGCAGGAAGGAGAAAGAGGGGGTGGTGGGGACAG

TGACAGAGAAGGATTGTGCCACAGATGGTGACATAGAGAG

>7284 len=160 tot-seq=1

TAGGGAATTTTCGTCAATGGGGGGAACCCTGAACGAGCAATGCCGCGTGAGTGAAGAAGG

CCTTCGGGTCGTAAAGCTCTGTTGTAAGAGAAAAACGGTCATTATAGGGAATGATAATGA

AGTGATGGTATCTTACCAGAAAGCCACGGCTAACTACGTG

>7285 len=160 tot-seq=1

TGGGGAATTTTGGACAATGGGGGCAACCCTGATCCAGCAATGCCGCGTGAGTGAAGAAGG

CCCTCGGGTTGTAAAGCTCTTTTGTCAGGGAAGAAACGGGTGAGGCTAATATCCCCATCT

AATGACGGTACCTGAAGAATAAGCACCGGCTAACTACGTG

>7286 len=160 tot-seq=1

TGGGGAATATTGCACAATGGGGGAAACCCTGATGCAGCCATGCCGCGTGTGTGAAGAAGG

CCTTCGGGTTGTAAAGCACTTTCAGCGAGGAGGAAAGGGTAGTGGCTAATATCCGCTACC

TGTGACGTTACTCGCAGAAGAAGCACCGGCTAACTCCGTG

>7287 len=160 tot-seq=1

TGGGGAATTTTGGACAATGGGCGAAAGCCTGATCCAGCCATGCCGCGTGAGTGAAGAAGG

CCTTCGGGTTGTAAAGCTCTTTCAGTCGGAAAGAAAAGGCGCCGGATAATACCTGGCGCT

GATGACGGTACCGACAGAAGAAGCACCGGCTAACTACGTG

>7288 len=160 tot-seq=1

TAGGGAATTTTCGGCAATGGAGGAAACTCTGACCGAGCAACGCCGCGTGAATGAAGAAGT

ATTTCGGTATGTAAAGTTCTTTTATTAAGGAAGAATGTGTTTTATAGGAAATGATAGAAC

AGTGACGGTACTTAATGAATAAGCCCCGGCTAACTATGTG

>7289 len=160 tot-seq=1

TGGGGAATATTGGACAATGGGGGCAACCCTGATCCAGCCATGCCGCGTGTGTGAAGAAGG

CCTTCGGGTTGTAAAGCACTTTCAGCGAGGAAGAACGCCAGGGAATTAATACCTCCCTGG

AAAGACATCACTCGCAGAAGAAGCACCGGCTAACTCCGTG

>7290 len=160 tot-seq=1

TAGGGAATCTTCCACAATGGGCACAAGCCTGATGGAGCAACACCGCGTGAGTAAAGAAGG

GTTTCGGCTCGTAAAGCTCTGTTGTTGGAGAAGAGCGTGTGCGAGAGTAACTGTTCACGC

AGTGACGGTATCCAACCAGAAAGTCACGGCTAACTACGTG

>7291 len=160 tot-seq=1

TGGGGAATATTGGACAATGGGCGAAAGCCTGATCCAGCCATGCCGCGTGTGTGAAGAAGG

TCTTCGGATTGTAAAGCTCTGTTGGTAGTGAAGAAAGATAGAGGTAGTAACTGGCCTTTA

TTTGACGGTAATTACTTAGAAAGTCACGGCTAACTATGTG

>7292 len=160 tot-seq=1

TAGGGAATCTTCCACAATGGACGCAAGTCTGATGGAGCAACGCCGCGTGAGTGAAGAAGG

GTTTCGGGTTGTAAAGCACTTTCAGTAAGGAGGAAAGGTTAAGTTTTAATAGAGCTTAGC

TGTGACGTTACTTACAGAAGAAGCACCGGCTAACTCCGTG

>7293 len=160 tot-seq=1

TGGGGAATATTGCACAATGGGCGCAAGCCTGATGCAGCCATGCCGCGTGTGTGAAGAAGG

CCTTCGGGTTGTAAAGCTCTGTTGTTGGAGAAGAACGTGCGTGAGAGTAACTGTTCATGC

AGTGACGGTATCCAACCAGAAAGTCACGGCTAACTACGTG

>7294 len=160 tot-seq=1

TGGGGAATATTGGACAATGGGCGCAAGCCTGATCCAGCCATGCCGCGTGAGTGATGAAGG

CCCTAGGGTTGTAAAGCTCTTTCAGCGGGGAGGAAGGCGGTGAGGTTAATAACCTCATCG

ATTGACGTTACCCGCAGAAGAAGCACCGGCTAACTCCGTG

>7295 len=160 tot-seq=1

TAGGGAATCTTCGGCAATGGACGGAGGTCTGACCGAGCAACGCCGCGTGAGTGAAGAAGG
TTTTCGGATCGTAAAGCTCTGTTGTTAGAGAAGAACGTGCGTGAGAGCAACTGTTCACGC
AGTGACGGTATCTAACCAGAAAGTCACGGCTAACTACGTG
>7296 len=160 tot-seq=1
TGGGGAATATTGCACAGTGGGCGCAAGCCTGATGCAGCGACGCCGCGTGCGGGATGACGG
CCTTCGGGTTGTAAAACTCTGTCCTCATCCAAGAGAGAGACCTGTAGTAACTGCCAGGTC
TAGGACGGTAGATGAGGAGGAAGCCCCGGCTAACTATGTG
>7297 len=160 tot-seq=1
TAGGGAATCTTCGGCAATGGGCGCAAGCCTGATGCAGCGACGCCGCGTGCGGGATGAAGG
CCTTCGGGTTGTAAACCGCTTTTGTCAGAGACGAAAAGGGATGTACGAATAATACGTTCC
GCTGACGGTATCTGAAGAATAAGCACCGGCTAACTACGTG
>7298 len=160 tot-seq=1
TAGGGAATTTTCGTCAATGGGGGCAACCCTGAACGAGCAATGCCGCGTGAGCGAAGAAGG
TCTTCGGATTGTAAAGCTCTGTTGAAGGGGAAAAAGAGCGGAGAGAGGAAATGGTCTCCG
ATTGATATTACCCTTCGAGGAAGTCACGGCTAACTACGTG
>7299 len=160 tot-seq=1
TAGGGAATTTTCGTCAATGGGCGCAAGCCTGAACGAGCAATGCCGCGTGAACGAGGAAGG
CCTTCGGGTTGTAAAGTACTTTCAGCGGGGAGGAAGGGAGTGAGGTTAATAACCTTATTC
ATTGACGTTACCCGCAGAAGAAGCACCGGCTAACTCCGTG
>7300 len=160 tot-seq=1
TAGGGAATCTTCGGCAATGGGGGGAACCCTGACCGAGCAACGCCGCGTGAGTGAGGAAGG
TCTTCGGATCGTAAAGCTCTGTTGTAAGAGAAAAACGGCGCTCATAGGGAATGATGAGTG
AGTGATGGTATCTTACCAGAAAGTCACGGCTAACTACGTG
>7301 len=160 tot-seq=1
TCGAGAATCATTCACAATGGGGGAAACCCTGATGGAGCAACGCCGCGTGGGTGAAGAAGG
TCTTCGGATCGTAAAACCCTGTTGTTAGAGAAGAAAGTGCGTGAGAGTAACTGTTCACGT
TTCGACGGTATCTAACCAGAAAGCCACGGCTAACTACGTG
>7302 len=160 tot-seq=1
TGAGGAATATTGGTCAATGGGCGCAAGCCTGAACCAGCCATGCCGCGTGCAGGAAGACGG

CTCTATGAGTTGTAAACTGCTTTGTCAGGGAAGAAAAGGTCTGTGTTAATACCATAGACT
GCTGACGGTACCTGAAGAATAAGCACCGGCTAACTACGTG
>7303 len=160 tot-seq=1
TGGGGAATATTGCACAATGGGCGAAAGCCTGACGCAGCGACGCCGCGTGAGGGATGAAGG
TTTTCGGATCGTAAACCTCTGTCGGAAGGGAAGAAAGTGCGTGGTGCTAATCAGCCGCGT
ATTGACGGTACCTTCAAAGGAAGCACCGGCTAACTCTGTG
>7304 len=160 tot-seq=1
TGGGGAATATTGCACAATGGGCGCAAGTCTGACGGAGCAACGCCGCGTGAGTGATGACGG
TCTTCGGATTGTAAAGCTCTGTTAATCGGGACGAATGGTTTGTGTGCAAATAGTGCATAG
ACATGACGGTACCGGAATAGAAAGCCACGGCTAACTACGT
>7305 len=160 tot-seq=1
TAGGGAATCTTCCACAATGGGCGCAAGCCTGAACGAGCAATGCCGCGTGAACGAGGAAGG
TCTTCGGATCGTAAAGTTCTGTTGAAGGGGAAAAAGAGCGGAGAGAGGAAATGCTTTCTG
ATTGATATTACCCTTCGAGGAAGTCACGGCTAACTACGTG
>7306 len=160 tot-seq=1
TGGGGAATCTTCCGCAATGGACGAAAGTCTGACGGAGCAACGCCGCGTGAGTGATGACGG
TCTTCGGATTGTAAACTGCTTTTGTCAGGGAAGAAAAGGTCTGTGTTAATACCATAGACT
GCTGACGGTACCTGAAGAATAAGCACCGGCTAACTACGTG
>7307 len=160 tot-seq=1
TGGGGAATATTGCGCAATGGGGGAAACCCTGACGCAGCGACGCCGCGTGCGGGATGAAGG
TCTTCGGATTGTAAACTGCTTTTGTCAGGGACGAAAAGGGATGCGATAACACCGCATTCC
GCTGACGGTACCTGAAGAATAAGCACCGGCTAACTACGTG
>7308 len=160 tot-seq=1
TAGGGAATCTTCCACAATGGACGCAAGTCTGATGGAGCAACGCCGCGTGAGTGAAGAAGG
ATTTCCGTTCGTAAAACTCTGTTGTTAGAGAAGAACAGTGCATAGAGTAACTGTTATGCG
TGTGACGGCATCTAACCAGAAAGCCACGGCTAACTACGTG
>7309 len=160 tot-seq=1
TAGGGAATCTTCCGCAATGGACGCAAGTCTGACGGAGCAACGCCGCGTGAGTGAAGAAGG
ATTTCGGTTCGTAAAACTCTGTTGTTAGAGAAGAACACGAGTGAGAGTAACTGTTCATTC

GATGACGGTATCTAACCAGCAAGTCACGGCTAACTACGTG

>7310 len=160 tot-seq=1

TAGGGAATCTTCCACAATGGACGCAAGTCTGATGGAGCAACGCCGCGTGAGTGAAGAAGG

TCTTCGGATCGTAAAACTCTGTTGTTAGAGAAGAACACGAGTGAGAGTAACTGTTCATTC

GATGACGGTACCGGAATAGAAAGCCACGGCTAACTACGTG

>7311 len=160 tot-seq=1

TGGGGAATATTGCACAATGGGCGCAAGCCTGATGCAGCAACGCCGCGTGAACGATGAAGG

TCTTCGGATTGTAAACTGCTTTTGCCAGGGACGAAAAGGGATGCGATAACACCGCATTCC

GCTGACGGTACCTGAAGAATAAGCACCGGCTAACTACGTG

>7312 len=160 tot-seq=1

TGGGGGATATTGCACAATGGAGGCAACTCTGATCCAGCCATGCCGCGTGCAGGACGAAGG

TCTTCGGATTGTAAACTGCTTTTGTCAGGGAAGAAAAGGTCTGTGTTAATACCATAGACT

GCTGACGGTACCTGAAGAATAAGCACCGGCTAACTACGTG

>7313 len=160 tot-seq=1

TAGGGAATTTTCGGCAATGGGGGAAACCCTGACCGAGCAACGCCGCGTGAATGATGAAGT

ATTTCGGTATGTAAAATTCTTTTATAGAGGACGAATGTTACTAATAGGAAATGATTAGTA

AGTGACGGTACTTTATGAATAAGCCCCGGCTAACTACGTG

>7314 len=160 tot-seq=1

TGGGGAATATTGCACAATGGGCGAAAGCCTGATGCAGCGACGCCGCGTGAGCGAAGAAGT

ATTTCGGTATGTAAAGCTCTATCAGAAGGGAAGAAAAGGTCTGTGTTAATACCATAGACT

GCTGACGGTACCTGAAGAATAAGCACCGGCTAACTACGTG

>7315 len=160 tot-seq=1

TGGGGGATTTTGGACAATGGGGGCAACCCTGATCCAGCCATGCCGCGTGCAGGATGAAGG

TCTTCGGATTGTAAACTGCTTTTGTCAGGGAAGAAAAGGTCTGTGTTAATACCATAGACT

GCTGACGGTACCTGAATAGAAAGCCACGGCTAACTACGTG

>7316 len=160 tot-seq=1

TGGGGAATATTGCGCAATGGGGGAAACCCTGACGGAGCAACACCGCGTGAGTGAAGAAGG

GTTTCGGCTCGTAAAGCTCTGTTGTTAGAGAAGAACGTGCGTGAGAGCAACTGTTCACGC

AGTGACGGTATCTAACCAGAAAGTCACGGCTAACTACGTG

>7317 len=160 tot-seq=1

TAGGGAATCTTCCACAATGGGCGCAAGCCTGATGGAGCAACACCGCGTGAGTGAAGAAGG

GTTTCGGCTCGTAAAGCTCTGTTGTTGAAGAAGAATGTGCAAGAGAGTAACTGTTCTTGT

ATTGACGGTATTCAACCAGAAAGTCACGGCTAACTACGTG

>7318 len=160 tot-seq=1

TGAGGAATATTGGTCAATGGCCGAAGGGCTGAACCAGCCAAGTCGCGTGAGTGATGAAGG

TCTTCGGATCGTAAAACTCTGTTATTAGGGAAGAACATATGTGTAAGTAACTGTGCACAT

CTTGACGGTACCTAATCAGAAAGCCACGGCTAACTGCGTG

>7319 len=160 tot-seq=1

TGGGGAATATTGCACAATGGGCGCAAGCCTGATGCAGCCATGCCGCGTGTATGAAGAAGG

CCTTCGGATCGTAAAACTCTGTTATTAGGGAAGAACATATGTGTAAGTAACTGTGCACAT

CTTGACGGTACCTAATCAGAAAGCCACGGCTAACTACGTG

>7320 len=160 tot-seq=1

TAGGGAATCTTCCGCAATGGGCGAAAGCCTGACGGAGCAACGCCGCGTGTATGAAGAAGG

CCTTCGGGTTGTAAAGTACTTTCAGTCGTGAGGAAGGGGGTATCGTTAATAGCGGTATTC

TTTGACGTTAGCGACAGAAGAAGCACCGGCTAACTCCGTG

>7321 len=160 tot-seq=1

TAGGGAATCTTCCGCAATGGGCGAAAGCCTGATCCAGCAATGCCGCGTGCAGGATGAAGG

CCTTCGGGTTGTAAACTGCTTTTGTACGGAACGAAAAAGCTCCTTCTAATATAGGGGGCC

CATGACGGTACCGTAAGAATAAGCACCGGCTAACTACGTG

>7322 len=160 tot-seq=1

TAGGGAATCTTCCACAATGGGTGCAAACCTGATGGAGCAATGCCGCGTGAGTGAAGAAGG

ACTTCGGTTCGTAAAGCTCTGTTGTTAGAGAAGAACAAGGATAGGAGTAACTGTCTATCC

CTTGACGGTACCTAACCAGAAAGTCACGGCTAACTACGTG

>7323 len=160 tot-seq=1

TGGGGAATATTGGACAATGGGCGCAAGCCTGATCCAGCCATACCGCGTGGGTGAAGAAGG

CCTTCGGGTTGTAAAGCCCTTTTGTTGGGAAAGAAATCTTTCAGGCTAATACCCTGGGAG

GATGACGGTACCCAAAGAATAAGCACCGGCTAACTTCGTG

>7324 len=160 tot-seq=1

TGGGGAATCTTCCGCAATGGACGAAAGTCTGACGGAGCAACGCCGCGTGAGTGAAGAAGG

TCTTCGGATTGTAAAGTTCTTTTATATGGGAAGAAACGCTAAAATAGGAAATGATTTTAG

TTTGACTGTACCATTTGAATAAGTATCGGCTAACTATGTG

>7325 len=160 tot-seq=1

TGGGGAATCTTCGGCAATGGACGGAAGTCTGACCGAGCAACGCCGCGTGAGTGAAGAAGG

TTTTCGGATCGTAAAGCACTTTAAGTTGGGAGGAAGGGTTGTAGATTAATACTCTGCAAT

TTTGACGTTACCGACAGAATAAGCACCGGCTAACTCTGTG

>7326 len=160 tot-seq=1

TGGGGAATCTTCCGCAATGGACGGAAGTCTGGCCGAGCAACGCCGCGTGAGTGAAGAAGG

TTTTCGGATCGTAAAGCTCTGTCGTGAGAGAAGAACGAGTGTGAGAGTGGAAAGTTCACA

CTGTGACGGTATCTTACCAGAAAGGGACGGCTAACTACGT

>7327 len=160 tot-seq=1

TGGGGAATTTTGGACAATGAGCACAAGCCTGATCCAGCAATGCCGCGTGAGTGAAGAAGG

CCTTCGGGTTGTAAAGCTCTTTTGTCAGGGAAGAAACGGCTGTGGGTAATATCCATGGCT

AATGACGGTACCTGAAGAATAAGCACCGGCTAACTACGTG

>7328 len=160 tot-seq=1

TGGGGAATCTTGCACAATGGGCGAAAGCCTGATGCAGCGACGCCGCGTGCGGGATGAAGG

GTTTCGGCTCGTAAAGCTCTGTTGTTGGTGAAGAAGGACAGGGGTAGTAACTGACCTTTG

TTTGACGGTAATCAATTAGAAAGTCACGGCTAACTACGTG

>7329 len=160 tot-seq=1

TAGGGAATTTTCGTCAATGGGGGGAACCCTGAACGAGCAATGCCGCGTGAGTGAAGAAGG

TCTTCGGATCGTAAAGCTCTGTTGTAAGAGAAAAACGGTCTCCATAGGGAATGATGGAGA

AGTGATGGTATCTTACCAGAAAGCCACGGCTAACTACGTG

>7330 len=160 tot-seq=1

TGGGGAATTTTGGACAATGGGGGCAACCCTGATCCAGCAATGCCGCGTGAGTGAAGAAGG

CCTTCGGGTTGTAAAGCTCTTTTGTCAGGGAAGAAAAGGGCTGGGTTAATACCCTGGTTT

CATGACGGTACCTGAAGAATAAGCACCGGCTAACTACGTG

>7331 len=160 tot-seq=1

TGGGGAATATTGCGCAATGGGGGAAAGTCTGACGGAGCAACGCCGCGTGAGTGATGAAGG

TCTTCGGATTGTAAAACTCTGTTGTCAGGACGAATGTACTGATCTATAATACATTTCGGT

ATTGACGGTACCTGACGAGGAAGCCACGGCTAACTACGTG

>7332 len=160 tot-seq=1

TGGGGAATTTTGCACAATGGGGGAAAGTCTGACGGAGCAACGCCGCGTGAGTGATGACGG

TCTTCGGATTGTAAAGCTCTGTTAATCGGGACGAATGGTCGGTGTGCAAATAGTGCATGG

ACATGACGGTACCGGAATAGAAAGCCACGGCTAACTACGT

>7333 len=160 tot-seq=1

TGGGGAATATTGCACAATGGGCGCAAGCCTGATGCAGCCATGCCGCGTGAATGAAGAAGG

CCTTAGGGTTGTAAAGTTCTTTCGGTATTGAGGAAGGGGTGTGTGCTAATAGTACACATC

ATTGACGTTAAATACAGAAGAAGCACCGGCTAACTCCGTG

>7334 len=160 tot-seq=1

TGGGGAATATTGCACAATGGGCGCAAGCCTGATGCAGCGACGCCGCGTGTGTGAAGAAGG

TCTTCGGATTGTAAAGCACTTTAAGTTGGGAGGAAGGGCAGTAAGTTAATACCTTGCTGT

TTTGACGTTACCAGCAGAATAAGCACCGGCTAACTTCGTG

>7335 len=160 tot-seq=1

TGGGGAATATTGCACAATGGGCGCAAGCCTGATGCAGCGACGCCGCGTGAGTGAAGAAGG

GTTTCGGCTCGTAAAGCTCTGTTGTTAAAGAAGAACACGTATGAGAGTAACTGTTCATAC

GTTGACGGTATTTAACCAGAAAGTCACGGCTAACTACGTG

>7336 len=160 tot-seq=1

TGGGGAATATTGCACAATGGGCGCAAGCCTGATGGAGCAACACCGCGTGAGTGAAGAAGG

GTTTCGGCTCGTAAAGCTCTGTTGTTGGAGAAGAACAAGGACGTTAGTAACTGAACGTCC

CCTGACGGTATCTAACCAGAAAGCCACGGCTAACTACGTG

>7337 len=160 tot-seq=1

TAGGGAATTTTCGTCAATGGGGGGAACCCTGAACGAGCAATGCCGCGTGAGTGAGGAAGG

TCTTCGGATCGTAAAGCTCTGTTGTTGGAGAAGAACGTGCGTGAGAGTAACTGTTCACGC

AGTGACGGTATCCAACCAGAAAGTCACGGCTAACTACGTG

>7338 len=160 tot-seq=1

TAGGGAATTTTCGTCAATGGGGGGAACCCTGAACGAGCAACACCGCGTGAGTGAAGAAGG

GTTTCGGCTCGTAAAGCTCTGTTGTTGGAGAAGAACGTGCGTGAGAGTAACTGTTCACGC

AGTGACGGTATCCAACCAGAAAGTCACGGCTAACTACGTG

>7339 len=160 tot-seq=1

TAGGGAATCTTCGGCAATGGACGAAAGTCTGACCGAGCAACGCCGCGTGAGTGAAGAAGG

TTTTCGGATCGTAAAACTCTGTTGTTAGAGAAGAACAAGGATGAGAGTAACTGTTCACGC

AGTGACGGTATCCAACCAGAAAGTCACGGCTAACTACGTG

>7340 len=160 tot-seq=1

TAGGGAATCTTCCACAATGGGCGCAAGCCTGATGGAGCAACACCGCGTGAGTGAAGAAGG

GTTTCGGCTCGTAAAGCTCTGTTGTTAAAGAAGAACACGTATGAGAGTAACTGTTCACGC

AGTGACGGTATCCAACCAGAAAGTCACGGCTAACTACGTG

>7341 len=160 tot-seq=1

TAGGGAATCTTCGGCAATGGACGAAAGTCTGACCGAGCAGCGCCGCGTGAGTGAAGAAGG

CCTTCGGGTCGTAAAATTCTGTTGTCAGAGAAGAACGTGCGTGAGAGTAACTGTTCACGT

ATTGACGGTATCTGACCAGAAAGCCACGGCTAACTACGTG

>7342 len=160 tot-seq=1

TAGGGAATTTTCGGCAATGGGGGAAACCCTGACCGAGCAACGCCGCGTGAGTGAAGAAGG

CCTTCGGGTTGTAAAGCTCTGTTGTAAGGGAAGAACGATAGTTAGAGGGAATGCTAGCTA

TATGACGGTACCTTACCAGAAAGCCACGGCTAACTACGTG

>7343 len=160 tot-seq=1

TGGGGAATATTGGACAATGGAGGCAACTCTGATCCAGCAATTCTATGTGTGTGAAGAAGG

TCTTCGGATTGTAAAGTGCTGTTATAAGGGAAGAACATTTGCAATAGGAAATGATTGCAG

ACTGACGGTACCTTGTCAGAAAGCGATGGCTAACTATGTG

>7344 len=160 tot-seq=1

TGAGGAATATTGGTCAATGGACGCAAGTCTGATGGAGCAACGCCGCGTGAGTGGAGAAGG

GTTTCGGCTCGTAAAGCTCTGTTGTTGGTGAAGAAGGACAGGGGTAGTAACTGACCTTTG

TTTGACGGTAATCAATTAGAAAGTCACGGCTAACCACGTG

>7345 len=160 tot-seq=1

TGGGGAATTTTGCACAATGGGCGCAAGCCTGATGCAGCGACGCCGCGTGCGGGATGAAGG

CCTTCGGGTTGTAAAACTCTGTCCTCATCCAAGAGAAGGAAAGGTAGTAACTGACCTTTC

CAGGACGGTAGATGAGGAGGAAGCCCCGGCTAACTATGTG

>7346 len=160 tot-seq=1

TGGGGAATCTTCCGCAATGGGCGCAAGCCTGACGGAGCAATGCCGCGTGAGTGAAGAAGG

CCTTCGGGTTGTAAAACTCTGTCCTCATCCAAGAGAAGGGAAGGTAGTAACTGACCTTTC

TAGGACGGTACCCTGTGAGGAAGCCCCGGCTAACTACGTG

>7347 len=160 tot-seq=1

TGGGGAATCTTCCGCAATGGGCGCAAGCCTGACGGAGCAATGCCGCGTGAGTGAAGAAGG

CCTTCGGGTTGTAAAACTCTGTCCTCATCCAAGAGAAGGAAAGGTAGTAACTGACCTTTG

TTTGACGGTAATCAATTAGAAAGTCACGGCTAACTACGTG

>7348 len=160 tot-seq=1

TGGGGAATCTTCCGCAATGGACGAAAGTCTGACGGAGCAACGCCGCGTGAGTGAAGACGG

CCTTCGGGTTGTAAAGCTCTGTGATTCGGGACGAAAGGCCATATGTGAATAATATATGGA

AATGACGGTACCTGCAGAAGAAGCCCCGGCTAACTACGTG

>7349 len=160 tot-seq=1

TAGGGAATTTTCGGCAATGGGGGAAACCCTGACCGAGCAACGCCGCGTGAGTGATGACGG

CCTTCGGCTCGTAAAGCTCTGTTGTTGGTGAAGAAGGACAGGGGTAGTAACTGACCTTTG

TTTGACGGTAATCAATTAGAAAGTCACGGCTAACTACGTG

>7350 len=160 tot-seq=1

TAGGGAATCTTCCGCAATGGGCGAAAGCCTGACGGAGCAACGCCGCGTGAGTGAAGACGG

CCTTCGGGTTGTAAAACTCTGTCCTCATCCAAGAGAAGGGAAGGTAGTAACTGACCTTTC

TAGGACGGTAGATGAGGAGGAAGCCCCGGCTAACTATGTG

>7351 len=160 tot-seq=1

TGGGGAATATTGCACAATGGAGGGAACTCTGATGCAGCGACGCCGCGTGAACGAAGAAGG

CTTTCGAGTCGTAAAGCTCTGTTGTTGGTGAAGAAGGACAGGGGTAGTAACTGACCTTTG

TTTGACGGTAATCAATTAGAAAGTCACGGCTAACTACGTG

>7352 len=160 tot-seq=1

TAGGGAATCTTCGGCAATGGGGGCAACCCTGACCGAGCAACGCCGCGTGAGTGAAGAAGG

TTTTCGGATCGTAAAACTCTGTTGTTAGAGAAGAACAAGTGCTAAAGTAACTGTTAGCGC

CTTGACGGTATCTAACCAGAAAGCCACGGCTAACTACGTG

>7353 len=160 tot-seq=1

TGGGGAATCTTCCGCAATGGGCGCAAGCCTGACGGAGCAATGCCGCGTGAGTGAAGACGG
CCTTCGGGTTGTAAAACTCTGTCCTTGCCCAAGAGAGAGGATGGTAGTAACTGACCATCC
TAGGACGGTAGGCAAGGAGGAAGCCCCGGCTAACTATGTG
>7354 len=160 tot-seq=1
GTAAGAATATTCCGCAATGGGGGGAACCCTGACGGAGCGACGCCGCGTGAACGAAGAAGG
CCGGAAGGTTGTAAAGTTCTTTTCTGTCCGAGGAATAAGTGTAGGAGGAAATGCCTGCAT
GGTGACGGTAGGGCAGGAATAAGCACCGGCTAATTACGTG
>7355 len=160 tot-seq=1
TGGGGAATATTGCACAATGGGCGAAAGTCTGATGGAGCGACGCCGCGTGAATGATGAAGG
CCGTGAGGTTGTAAAGTTCTTTTCGGGAGGAGGAATGAAGTTATCAGGGAATGGATGACA
GATGACGAGAATCCCGGAATAAGCCCCGGCTAACTACGTG
>7356 len=160 tot-seq=1
TGGGGAATATTGCACAATGGGCGCAAGCCTGATGCAGCCATGCCGCGTGTATGAAGAAGG
CCTTCGGGTTGTAAAGTACTTTCAGTCAGGAGGAAGGTGGTGAGCTTAATACGCTCATCA
ATTGACGTTACTGACAGAAGAAGCACCGGCTAACTCCGTG
>7357 len=160 tot-seq=1
TGGGGAATATTGCGCAATGGGGGGAACCCTGACGCAGCCATGCCGCGTGAATGAAGAAGG
CCTTCGGGTTGTAAAGCTCTGTTGTTGGTGAAGAAGGACAGGGGTAGTAACTGACCTTTG
TTTGACGGTAATCAATTAGAAAGTCACGGCTAACTACGTG
>7358 len=160 tot-seq=1
TAGGGAATCTTCCACAATGGACGCAAGTCTGATGGAGCAACGCCGCGTGAGTGAAGAGGG
TTTTCGGATCGTAAAGCTCTGTTGTTAGGGAAGAATGATTGTGTAGTAACTATACACAGT
AGAGACGGTACCTAACCAGAAAGCCACGGCTAACTACGTG
>7359 len=160 tot-seq=1
TGGGGAATCTTGCGCAATGGGCGAAAGCCTGACGCAGCCATGCCGCGTGGATGATGAGGG
TCTTAGGATTGTAAAGCTCTGTTGTTGGTGAAGAAGGACAGGGGTAGTAACTGACCTTTG
TTTGACGGTAATCAATTAGAAAGTCACGGCTAACTACGTG
>7360 len=160 tot-seq=1
TAGGGAATCTTCCGCAATGGACGAAAGTCTGACGGAGCAACGCCGCGTGAGTGATGAAGG

TTTTCGGATCGTAAAACTCTGTTGTTAGGGAAGAACAAGTAGCATAGTAACTGATGTTAC

CTTGACGGTACCTAACCAGAAAGCCACGGCTAACTACGTG

>7361 len=160 tot-seq=1

TGGGGAATATTGCGCAATGGGGGAAACCCTGACGCAGCCATGCCGCGTGCAGGATGAAGG

TCTTCGGATTGTAAACTGCTTTTGTCAGGGAAGAAAAGGTCTGTGTTAATACCATAGACT

GCTGACGGTACCTGAAAAATAAGCACCGGCTAACTACGTG

>7362 len=160 tot-seq=1

TGGGGAATATTGGACAATGGGCGCAAGCCTGATCCAGCCATGCCGCGTGTGTGAAGAAGG

CCTTCGGGTTGTAAAGCACTTTTGTCCGGAAAGAAAAGCTTCGGGTTAATAACCCGGAGT

TATGACGGTACCGGAAGAATAAGCACCGGCTAACTTCGTG

>7363 len=160 tot-seq=1

TGGGGAATTTTGGACAATGGGCGCAAGCCTGATCCAGCAATGCCGCGTGAGTGAAGAAGG

CCTTCGGGTTGTAAAGCTCTTTTGTCAGGGAAGAAACGGTGGGAGCTAATATCTCCTACT

AATGACGGTACCTGAAGAATAAGCACCGGCTAACTACGTG

>7364 len=160 tot-seq=1

TGGGGAATCTTCCGCAATGGACGAAAGTCTGACGGAGCAACGCCGCGTGAGTGAAGACGG

CCTTCGGGTTGTAAAGCTCTGTGATTCGGGACGAAAGGCCATATGTGAATAATATATGGA

AATGACGGTACCCAAGGAGGAAGCCACGGCTAACTACGTG

>7365 len=160 tot-seq=1

TGGGGAATATTGCACAATGGGCGCAAGCCTGATGCAGCCATGCCGCGTGTATGAAGAAGG

CCTTCGGGTTGTAAAGTACTTTCAGCGAGGAGGAAAGGTTGATGCCTAATACGTATCAGC

TGTGACGTTACTCGCAGAAGAAGCACCGGCTAACTCCGTG

>7366 len=160 tot-seq=1

GTAAGAATATTCCGCAATGGACGGAAGTCTGACGGAGCGACGCCGCGTGCGCGAAGAAGG

CCGGAAGGTTGTAAAGCGCTTTTTAGGTTGAGGAAGAACACTGAGAGGAAATGCTTGGTG

GGTGACTGAAGACTTGGAATAAGCACCGGCTAATTACGTG

>7367 len=160 tot-seq=1

TAGGGAATCTTCCACAATGGGCGCAAGCCTGATGGAGCAACACCGCGTGAGTGAAGAAGG

GTTTCGGCTCGTAAAGCTCTGTTGTTAAAGAAGAACGTGGGTGAGAGTAACTGTTCACCC

AGTGACGGTATTTAACCAGAAAGCCACGGCTAACTACGTG

>7368 len=160 tot-seq=1

TGGGGAATCTTCCGCAATGGACGAAAGCTTGATGGAGCGACACAGCGTGCACGATGAAGG

TCTTCGGATTGTAAAGTGCTGTTATAAGGGAAGAACATTTGCAATAGGAAATGATTGCAG

ACTGACGGTACCTTGTCAGAAAGCGATGGCTAACTATGTG

>7369 len=160 tot-seq=1

TGGGGAATATTGGACAATGGGCGAAAGTCTGACCGAGCAACGCCGCGTGAGTGAAGAAGG

TTTTCGGATCGTAAAACTCTGTTGTTAGAGAAGAATGGTCGGCAGAGTAACTGTTGCCGG

CGTGACGGTATCCAACCAGAAAGCCACGGCTAACTACGTG

>7370 len=160 tot-seq=1

TCGAGAATTTTTCTCAATGGGGGAAACCCTGAAGGAGCGACGCCGCGTGGAGGATGAAGG

TTTTCGGATTGTAAACTCCTGTCATTGGTGAACAAGAACTTGTGGTTAACTGCCATGGGT

TTCGATAGTAGCCGAAGAGGAAGGGACGGCTAACTCTGTG

>7371 len=160 tot-seq=1

TGAGGAATATTGGCCAATGGGCGCAAGCCTGACGGAGCAATGCCGCGTGAGTGAAGAAGG

CCTTCGGGTTGTAAAACTCTGTCCTCATCCAAGAGAAGGGAAGGTAGTAACTGACCTTTC

CAGGACGGTAGATGAGGAGGAAGCCCCGGCTAACTATGTG

>7372 len=160 tot-seq=1

GGCTCATGTTTCAGTGAATTTTCAGCTGACACCATGCCTTCAGGCCCATGGGATCTTTCT

GTGCCCCAGCGAGGCCCTGCCTGCCTCACCAGATGAGGTGAGCCCATCCCTGGTCACCTG

GTGGGATCCAAAATCGGATCTGAAGAGGAGTACTGAGACC

>7373 len=160 tot-seq=1

TGGGGAATTTTGGGCAATGGGCGAAAGCCTGATCCAGCCATGCCGCGTGCAGGATGAAGG

CCTTCGGGTTGTAAACTGCTTTTGTACGGAACGAAACGGTCTTTTCTAATACAGAAGGCT

AATGACGGTACCGTAAGAATAAGCACCGGCTAACTACGTG

>7374 len=160 tot-seq=1

TGGGGAATATTGGACAATGGGGGAAACCCTGATCCAGCGACGCCGCGTGTGTGAAGAAGG

CCTGCGGGTTGTAAAGCACTTTTAGTGGGGATGAAAAGTTCAGGGCTAATACCCCTGGAT

CTTGACCTAACCCACAGAAAAAGCACCGGCTAACTCTGTG

>7375 len=160 tot-seq=1

TGGGGAATCTTGCGCAATGCACGAAAGTGTGACGCAGCAACGCCGCGTGCGGGAAGAAGG

TCTTCGGATCGTAAACCGCTTTCAGTTGGGACGAGGGTCAGCTGGTGAATAGCCAGTCTG

ACTGACGGTACCTTCAGAAGAAGCCCCGGCTAACTACGTG

>7376 len=160 tot-seq=1

TGAATGAGGCTGACCCCTCCGTGCCCTCATGGGAGAGACAACCCAAGGACATATCACCAC

ACAAACCAGCTACAGGCTGGGATCTAACCCTGGACTCAGGAGAGCAGCACCAGGAGCCCC

AGCCTGGCCTGGGGATCAGGGAGGGCTTCCTGGAGGAGGC

>7377 len=160 tot-seq=1

TGGGGAATCTTCCGCAATGGACGAAGGTCTGACGGAGCAACGCCGCGTGAGTGAAGACGG

CCTTCGGATTGTAAAGCTCTGTGATTCGGGACGAAAGGCCATATGTGAATAATATGTGGA

AATGACGGTACCGGAAAAGCAAGCCACGGCTAACTACGTG

>7378 len=160 tot-seq=1

TGGGGAATCTTCCGCAATAGACGAAAGTCTGACGGAGCAACGCCGCGTGAGTGAAGACGG

CCTTCGGGTTGTAAAGCTCTGTGATTCGGGACGAAAGGCCATATGTGAATAATATATGGA

AATGACGGTATCATAGGAGGAAGCCCTGGCTAAATACGTG

>7379 len=160 tot-seq=1

TGGGGAATTTTGCACAATGGGGGAAACCCTGATGCAGCGACGCCGCGTGATTTAGAAGGC

CTTCGGGTTGTAAAGCTCTGTTATACGGGACGAACGGCAAGGTGGTAAATAGCCATCATG

AGTGACGGTACCGTAAGAGAAAGCCACGGCTAACTACGTG

>7380 len=160 tot-seq=1

TAGGGAATCTTCGGCAATGGACGAAAGTCTGACCGAGCAACGCCGCGTGAGTGAAGAAGG

TTTTCGGATCGTAAAACTCTGTTGTTAGAGAAGAACAAGGACGTTAGTAACTGGCCTTTA

TTTGACGGTAATCAACCAGAAAGTCACGGCTAACTACGTG

>7381 len=160 tot-seq=1

TAGGGAATCTTCCACAATGGACGCAAGTCTGGTGGAGCAACGCCGCGCGAGTGAAGAAGG

TTTTCGGATCGTAAAGCTCTGTTGTTGGTAAAGAAGGATAGAGGCAGCAACTGGCCTTTA

TTTGACGGTAATCAACCAGAAAGTCACGGCTAACTACGTG

>7382 len=160 tot-seq=1

TGGGGAATATTGGACAATGGAGGGAACTCTGATCCAGCAATTCTGTGTGTGTGAAGAAGG

TTTTAGGACTGTAAAGTACTTTCAGCGGGGAGGAAGGCGACAGGGTTAATAACCCTGTCG

ATTGACGTTACCCGCAGAAGAAGCACCGGCTAACTCCGTG

>7383 len=160 tot-seq=1

TGGGGAATATTGCACAATGGGCGCAAGCCTGATGCAGCCATGCCGCGTGTATGAAGAAGG

CCTTCGGGTTGTAAAGTACTTTCAGCGGGGAGGAAGGCGGTGGAGTTAATAGCTTCACCG

ATTGACGTTACCCGCAGAAGAAGCACCGGCTAACTCCGTG

>7384 len=160 tot-seq=1

TAGGGAATCTTCCGCAATGGGCGAAAGCCTGACGGAGCAACGCCGCGTGAGTGATGAAGG

TCTTCGGATCGTAAAACTCTGTTGTTAGGGAAGAACAAATTTGTTAGTAACTGAACAAGT

CTTGACGGTACCTAACCAGAAAGCCACGGCTAACTACGTG

>7385 len=160 tot-seq=1

TGGGGAATATTGCACAATGGGCGAAAGCCTGATGCAGCGACGCCGCGTGAGTGAAGAAGG

TTTTCGGATCGTAAAACTCTGTTGTTAGAGAAGAACAAGTGCTAGAGTAACTGTTAGCGC

CTTGACGGTATCTAACCAGAAAGCCACGGCTAACTACGTG

>7386 len=160 tot-seq=1

TGGGGAATATTGCGCAATGGGGGAAACCCTGACGCAGCGACGCCGCGTGCGGGATGAAGG

CCTTCGGGTTGTAAAGTACTTTCAGCGGGGAGGAAGGGAGTGAGGTTAATAACCTCATTC

ATTGACGTTACCCGCAGAAGAAGCACCGGCTAACTCCGTG

>7387 len=160 tot-seq=1

TAGGGAATCTTCCACAATGGACGAAAGTCTGATGGAGCAACGCCGCGTGAGTGATGAAGG

TTTTCGGATCGTAAAACTCTGTTGTAAGGGAAGAACAAGTACGTTAGGAAATGAACGTAC

CTTGACGGTACCTTATTAGAAAGCCACGGCTAACTACGTG

>7388 len=160 tot-seq=1

TGGGGAATATTGCGCAATGGGGGAAACCCTGACGCAGCGACGCCGCGTGCGGGATGAAGG

CCTTCGGGTTGTAAAGGACTTTTGTCAGGGAAGAAAAGGCTGTTGCTAATATCGACAGCT

GATGACGGTACCTGAAGAATAAGCACCGGCTAACTACGTG

>7389 len=160 tot-seq=1

TGAGGAATATTGGTCAATGGACGAGAGTCTGAACCAGCCAAGTAGCGTGAGTGAAGAAGG

GCTTCGGCTCGTAAAACTCTGTTGTTGAAGAAGAACGTGCGTGAGAGTAACTATTCACGC

AGTGACGGTATTCAACCAGAAAGTCACGGCTAACTACGTG

>7390 len=160 tot-seq=1

TGGGGAATATTGCGCAATGGGGGAAACCCTGACGCAGCGACGCCGCGTGTGTAAAGAAGG

TCTTCGGATCGTAAAGCACTGTTGTAAGTGAAGAATGCCACATAGAGGAAATGCTATGTG

GGTGACGGTAGCTTACCAGAAAGCCACGGCTAACTACGTG

>7391 len=160 tot-seq=1

TAGGGAATCTTCGGCAATGGGGGCAACCCTGACCGAGCAACGCCGCGTGAGTGAAGAAGG

TTTTCGGATCGTAAAGCTCTGTTGTAAGAGAAGAAAGATAGAGGTAGTAACTGGCCTTTA

CTTGACGGTAATTACTTAGAAAGTCACGGCTAACTACGTG

>7392 len=160 tot-seq=1

TAGGGAATCTTCGGCAATGGGGGCAAGCCTGATGCAGCCATGCCGCGTGTATGAAGAAGG

CCTTCGGGTTGTAAAGTACTTTCAGCGGGGAGGAAGGGAGTGAGGTTAATAACCTCATTC

ATTGACGTTACCCGCAGAAGAAGCACCGGCTAACTCCGTG

>7393 len=160 tot-seq=1

TAGGGAATCTTCGGCAATGGGGGCAACCCTGACCGAGCAACGCCGCGTGTATGAAGAAGG

CCTTCGGGTTGTAAAGTACTTTCAGCGGGGAGGAAGGGAGTGAGGTTAATAACCTTATTC

ATTGACGTTACCCGCAGAAGAAGCACCGGCTAACTCCGTG

>7394 len=160 tot-seq=1

TAGGGAATCTTCCACAATGGACGAAAGTCTGATGGAGCAACGCCGCGTGAGTGAAGAAGG

GTTTCGGCTCGTAAAGCTCTGTTGGTAGTGTCGAAAGTTGGAGGTAGTAACTGGCCTTTC

TTTGACGGTAATTACTTAGAAAGTCACGGCTAACTACGTG

>7395 len=160 tot-seq=1

TAGGGAATCTTCCACAATGGACGAAAGTCTGATGGAGCAACGCCGCGTGAGTGAGGAAGG

GTTTCGGCTCGTAAAGCTCTGTTGGTAGTGAAGAAAGATAGAGGGAGTAACGGGCCTTGA

TTTGACGGTAATTACTGAGAAAGTCACGGCTAACTACGTG

>7396 len=160 tot-seq=1

TGGGGAATTTTGGACAATGGGGGCAACCCTGATCCAGCCATGCCGCGTGTATGAAGAAGG

CCTTAGGGTTGTAAAGTACTTTTGTTAGGGAAGAAAAGTCAGCTGTGAATAATGGCTGAT

ACTGACGGTACCTAAAGAATAAGCACCGGCTAACTACGTG

>7397 len=160 tot-seq=1

TGGGGAATTTTGGACAATGGGCGCAAGCCTGATCCAGCCATTCCGCGTGAGTGAAGAAGG

CCTTCGGGTTGTAAAGCTCTTTCGCAAGGGAAGAAAGATTACCCTCTAACATAAGGTAAT

TATGACGGTACCTTGATAAGAAGCACCGGCTAACTACGTG

>7398 len=160 tot-seq=1

TGGGGAATATTGGACAATGGGCGAAAGCCTGATCGAGCCATGCCGCGTGTGTGAAGAAGG

TCTTCGGATTGTAAAGCACTTTAAGTTGGGAGGAAGGATAGTAAATTAAAACTTTGCTGT

TTTGACGTTACCGAAAGAATAAGCACCGGCTAACTCTGTG

>7399 len=160 tot-seq=1

TGGGGAATATTGCACAATGGGGGAAACCCTGATGCAGCCATGCCGCGTGTGTGAAGAAGG

CCTTCGGGTTGTAAAGCACTTTCAGCGAGGAGGAAAGGGTGGAAGCTAATACCTTTCATC

TGTGACGTTACTCGCAGAAGAAGCACCGGCTAACTCCGTG

>7400 len=160 tot-seq=1

TGGGGAATTTTGGACAATGGGCGCAAGCCTGATCCAGCAATGCCGCGTGGGTGAAGAAGG

CCTTCGGGTTGTAAAGCTCTTTTGTCAGGGAAGAAACGGGAGAAGCTAATATCTTTTCCT

AATGACGGTACCTGAAGAATAAGCACCGGCTAGCTACGTG

>7401 len=161 tot-seq=1

GGTTGACAGATGGGGATAGGAGAGCGAGAGGCACTGTTTTCATGGGGGATCAGATTGTTC

AATGAGAGGGAAACACCTGAGGAAAAATGTTGAGGAAAGATGTTGGGAGAGGTGAAGGCA

GAAAGGCTTCCTTCAATCATATGCCACCTTGTGTCTCCTTC

>7402 len=161 tot-seq=1

TTAGGGATATTCGTCAATGGGGGAAACCCTGAACGAGCAATGCCGCGTGAAGGAAGAAGG

TCCTATGGATTGTAAACTTCTGTTGTAAGGGAAGAAAGAGTAGAAGAGGAAATGCTTCTA

AAGTGACAGTACCTTACGAGAAAGCTCCGGCTAACTACGTG

>7403 len=161 tot-seq=1

TGGGGAATATTGGACAATGGGCGAAAGCCTGATCCAGCCATGCCGCGTGTGTGAAGAAGG

CCTTCGGGTTGTAAAGCTCTGTTAATCGGGACGAAAGGCCTTCTTGCGAATAGTTAGAAG

GATTGACGGTACCGGAATAGAAAGCCACGGCTAACTACGTG

>7404 len=161 tot-seq=1

TCTCAGTACAGAACACCTGGTGGGCACATCTGATGGGCTGAGTCCTGAAAACATGCAGTC

TTGCTGCTGATCAGGCAGCTTTGCAAGTTAGAGAACAGAGAGGTGGTGGGGGGACCTTGT

GGGTGAGGAAGGCCTGTCTTCAGTAATGGGAGCAGTTAGGG

>7405 len=161 tot-seq=1

CTTCTTCATGGAAGGGGTGTCACATGCAACCAGGGAATGATGACTTGTCCCCTTTAAGTC

CTGCCACTATTCCAGTAGTGGGACATCTTGCCTGCTTGGTTGCTGTTGCAGTTTGTGCAA

TTCATAGTTAGCTGGGATAGACCACTGATGCTGTTTCTCCT

>7406 len=161 tot-seq=1

TGGGGAATATTGGACAATGGGGGGAACCCTGATCCAGCCATGCCGCGTGTGTGAAGAAGG

CCTTCGGGTTGTAAAGCACTTTAATTTGGGAGGAAGGGCTTACGGTTAATAACCGCTAAG

TTTTGACGTTACCAAAAGAATAAGCACCGGCTAACTTCGTG

>7407 len=161 tot-seq=1

TGGGGAATATTGGGCAATGGAGGAAACTCTGACCCAGCAACGCCGCGTGGAGGAAGAAGG

TTTTCGGATCGTAAAGCTCTGTTGTAAGAGAAGAACGAGTGTGAGAGTGGAAAGTTCACA

CTGTGACGGTATCTTACCAGAAAGGGACGGCTAACTACGTG

>7408 len=161 tot-seq=1

TGGGGAATCTTCCGCAATGGGCGCAAGCCTGACGGAGCAACGCCGCGTGAGTGAAGAAGG

GTTTCGACTCGTAAAGCTCTGTTGTCGGGGACGAACGTAGAGGTCATGAACAATGACTTC

TAATGACGGTACCTGACGAGGAAGCCACGGCTAACTACGTG

>7409 len=161 tot-seq=1

TAGGGAATCTTCCGCAATGGGCGAAAGCCTGACGGAGCAACGCCGCGTGAGTGATGAAGG

TCTTCGGATCGTAAAGCTCTGTTAATCGGGACGAATGGTCGGTGTGCAAATAGTGCATGG

ACATGACGGTACCGGAATAGAAAGCCACGGCTAACTACGTG

>7410 len=161 tot-seq=1

TGGGGAATATTGCACAATGGGGGAAACCCTGATGCAGCGACGCCGCGTGGAGGAAGAAGG

TCTTCGGATCGTAAAGCCCTGTTGTTAAGGAAGAACGAGTGTGAGAATGGAAAGTTCATA

CTATGACGGTACTTAACCAGAAAGGGACGGCTAACTACGTG

>7411 len=161 tot-seq=1

AAGGCTTAGAAGTACCCAGCCTTTGAGGAAGCCAAAGCATTTTAAAATTAAAACCTGAGG

ATATCTGTAGGGGATTGGATCTACTCTTAGTTGGGAATCTGCATGTCCAGACACTGAAGG

TCCTTCCTTGTCTGTGGTTTTTTGATCTATCAATAAAGGTA

>7412 len=161 tot-seq=1

TAGGGAATCTTCGGCAATGGGGGCAACCCTGACCGAGCAACGCCGCGTGAGTGATGACGG

CCTTCGGGTTGTAAAGCTCTGTTAATCGGGACGAAAGGTCCTCTTGCGAATAGTTAGAGG

AATTGACGGTACCGGAATAGAAAGCCACGGCTAACTACGTG

>7413 len=161 tot-seq=1

TGAGGAATATTGGTCAATGGGCGCAGGCCTGAACCAGCCAAGTAGCGTGAGTGAAGAAGG

TTTTCGGATCGTAAAGCTCTGTTGTAAGTCAAGAACGAGTGTGAGAGTGGAAAGTTCACA

CTGTGACGGTAGCTTACCAGAAAGGGACGGCTAACTACGTG

>7414 len=161 tot-seq=1

TGGGGAATATTGCACAATGGGGGAAACCCTGATGCAGCGACGCCGCGTGAGCGAAGACGG

CCTTCGGGTTGTAAAGCTCTGTTAATCGGGACGAAAGGTCCTCTTGCGAATAGTTAGAGG

AATTGACGGTACCGGAATAGAAAGCCACGGCTAACTACGTG

>7415 len=161 tot-seq=1

TGAGGTACTAAGCTTGCAGCTCGAATGCTTTATGACTACTACCTCATCTCTAATATCTTA

TCTGGGAAGTGGTTGTGCTGTCCCCTAAAAAATTCTGCATAAAACCTAGAGAAGTTTGTG

CATTAAAACAGGTTTGTGTGTGCCAGGGTATTTTATATAAT

>7416 len=161 tot-seq=1

TGGGGAATCTTGGACAATGGGCGAAAGCCCGATCCAGCAATATCGCGTGAGTGAAGAAGG

GCAATGCCGCTTGTAAAGCTCTTTCGGTATTGAGGAAGGTTGATGTGTTAATAGCACATC

AAATTGACGTTAAATACAGAAGAAGCACCGGCTAACTCCGT

>7417 len=161 tot-seq=1

TGGGGAATATTGCGCAATGGGGGCAACCCTGACGCAGCCATGCCGCGTGAATGAAGAAGG

TTTTCGGATCGTAAAGCTCTGTTGTAAGTCAAGAACGAGTGTGAGAGTGGAAAGTTCACA

CTGTGACGGTAGCTTACCAGAAAGGGACGGCTAACTACGTG

>7418 len=161 tot-seq=1

TGGGGAATTGTTCGCAATGGGCGCAAGCCTGACGACGCAACGCCGCGTGGAGGATGAAGG

TCTTCGGATTGTAAACTCCTGTTGATCGGGACGAACAACCACCGGGATAACACCCCGGAG

GTCTGACGGTACCGATTGAGGAAGCCCCGGCTAACTCTGTG

>7419 len=161 tot-seq=1

TGGGGAATATTGCACAATGGGCGCAAGCCTGATGCAGCAACGCCGCGTGAACGATGACGG

CCTTCGGGTTGTAAAGTTCTGTTATATGGGACGAACAGGGCATCGGTTAATACCCGGTGT

CTTTGACGGTACCGTAAGAGAAAGCCACGGCTAACTACGTG

>7420 len=161 tot-seq=1

TGGGGAATCTTCCGCAATGGGCGCAAGCCTGACGGAGCAACGCCGCGTGAGTGAAGAAGG

GTTTCGACTCGTAAAGCTCTGTTGTCGGGGACGAATGTGGAGATGGTGAATAACCATTTT

CAATGACGGTACCTGACGAGGAAGCCACGGCTAACTACGTG

>7421 len=161 tot-seq=1

TGAGGAATTTTTCACAATGGACGAAAGTCTGATGGAGCAACGCCGCGTGAGTGAAGAAAG

TTTTCGGATCGTAAAGCTCTGTTGTAAGAGAAGAACGAGTGTGAGAGTGGAAAGTTCACA

CTGTGACGGTATCTTACCAGAAAGGGACGGCTAACTACGTG

>7422 len=161 tot-seq=1

TTAGGAATATTCGTCAATGGGGGAAACCCTGAACGAGCAATGCCGCGTGAGTGATGAAGG

TCCTTTGGATTGTAAAACTCTGTTGTTAAGGAAAAATGATAAGAATAGGAAATGATTCTT

ATTTGATGGTACTTTTCAAGAAAGCCACGGCTAACTACGTG

>7423 len=161 tot-seq=1

TAGGGAATCTTCCACAATGGGCGAAAGCCTGATGGAGCAACGCCGCGTGGGTGAAGAAGG

TCTTCGGATCGTAAAGCTCTGCTGTAAGAGAAGAACGTGTGTGAGAGTGGAAAGTTCACA

CAGTGACGGTAACTTACCAGAAAGGGACGGCTAACTACGTG

>7424 len=161 tot-seq=1

TAGGGAATCTTCGGCAATGGGGGCAACCCTGACCGAGCAACGCCGCGTGAGTGAAGAAGG

TTTTCGGATCGTAAAGCTCTGTTGTAAGAGAAGAACGTGTGTGAGAGTGGAAAGTTCACA

CAGTGACGGTATTCAACCAGAAAGTCACGGCTAACTACGTG

>7425 len=161 tot-seq=1

TAGGGAATATTGCGCAATGGGGGAAACCCTGACGCAGCAACGCCGCGTGAGTGAGGAAGG

TCTTCGGATTGTAAAACTCTTTTATTAGGGAAAAATGTCTAGGTGGCTAATATCCATCTA

GAGTGATGGTACCTAAGGAATAAGCACCGGCTAACTTCGTG

>7426 len=161 tot-seq=1

TAGGGAATCTTCGGCAATGGGGGCAACCCTGACCGAGCAACGCCGCGTGAGTGAAGAAGG

TTTTCGGATCGTAAAGCTCTGTTGTAAGAGAAGAACGTGTGTGAGAGTGGAAAGTTCACA

CAGTGACGGTACCTGCAGAAGAAGCCCCGGCTAACTACGTG

>7427 len=161 tot-seq=1

TAGGGGATCTTCCGCAATGGGCGAAAGCCTGACGGAGCAACGCCGCGTGAGTGAAGAAGG

ATTTCGGTTCGTAAAGCTCTGTTGTTAGGGAAGAACGTTGGTAGGAGTGGAAAATCTACC

AAGTGACGGTAACTAACCAGAAAGGGACGGCTAACTACGTG

>7428 len=161 tot-seq=1

TGGGGGATCTTCCGCAATGGACGAAAGTCTGACGGAACAACGCCGCGTGAGTGATGACGG

TCTTCGGATTGTAAAGCTCTGTTAATCGGGACGAATGGTCGGTGTGCAAATAGTGCATGG

ACATGACGGTAACTAACCAGAAAGGGACGGCTAACTACGTG

>7429 len=161 tot-seq=1

TAGGGAATCTTCGGCAATGGACGGAAGTCTGACCGAGCAACGCCGCGTGAGTGAAGAAGG

TTTTCGGACCGTAAAGCTCTGTTGTAAGAGAAGAACGAGTATGAGAGTGGAAAATTCATA

CTGTGACGGCATCTTACCAGAAAGGGACGGCTAACTACGTG

>7430 len=161 tot-seq=1

TAGGGAGTTTTCGGCAATGGGCGAAAGCCTGACCGAGCAACGCCGCGTGAGTGATGAAGG

TCTTCGGATCGTAAAGCTCTGTTGTTAGAGAAGAACGTTGGTAGGAGTGGAAAATCTACC

AAGTGACGGTAACTAACCAGAAAGGGACGGCTAACTACGTG

>7431 len=161 tot-seq=1

AGGATGAGATGGATGGCATCACTGACTCAAGGGATGTGAATTTGAGCAAATTCTAGGACA

TGGTGAAGGACAGGGAAGCCTGGTGTGCTGCAGTCCATGGGTCGCAGAGTTGGACACGAC

TTAGCGCCTGAACAACCAGGGGAGGAGAGCAGAGTAAAAGA

>7432 len=161 tot-seq=1

TGAGGAATTTTTCACAATGGACGAAAGTCTGATGGAGCAATACCACGTGAACGATGAAGG

TCTTCGGATCGTAAAGCTCTGTTGTTAAGGAAGAACGAGTGTGAGAATGGAAAGTTCATG

CTGTGACGGTACTTAACCAGAAAGGGACGGCTAACTACGTG

>7433 len=161 tot-seq=1

TAGGGAATTTTTCACAATGGGCGCAAGCCTTATGAAGCAATGCCGCGTGAACGATGAAGG

TCTTATAGATCGTAAAGTGCTGTTATAAGGGAAGAACATTTGCAATAGGAAATGATTGCA

GACTGACGGTACCTTGTCAGAAAGCGATGGCTAACTATGTG

>7434 len=161 tot-seq=1

TAGGGAATTTTTCACAATGGGCGCAAGCCTTATGAAGCAATGCCGCGTGAACGATGAAGG

TCTTATAGATTGTAAAGTTCTTTTATATGGGAAGAAACGCTAAAATAGGAAATGATTTTA

GTTTGACGGTACCTTGTCAGAAAGCGATGGCTAACTATGTG

>7435 len=161 tot-seq=1

TGGGGAATCTTCCGCAATGGACGAAAGTCTGACGGAGCAACGCCGCGTGAGTGATGACGG

TCTTCGGATTGTAAAACTCTGTCATACGGGACGAAAAAGGCGGATGCCAACAGTATCCGT

CCGTGACGGTACCGTAAGAGAAAGCCACGGCTAACTACGTG

>7436 len=161 tot-seq=1

TAGGGAATCTTCCGCAATGGACGAAAGTCTGACGGAGCAACGCCGCGTGAGTGAAGAAGG

ATTTCGGTTCGTAAAACTCTGTTATACGGGACGAAAAAGGCGGATGCCAACAGTATCCGT

CCGTGACGGTACCGTAAGAGAAAGCCACGGCTAACTACGTG

>7437 len=161 tot-seq=1

TGGGGAATATTGGACAATGGAGGAAACTCTGATCCAGCAATTCTGTGTGTGTGAAGAAGG

TTTTAGGACTGTAAAGCTCTGTTAATCGGGACGAATGGTCGGTGTGCAAATAGTGCATGG

ACATGACGGTACCGGAATAGAAAGCCACGGCTAACTACGTG

>7438 len=161 tot-seq=1

TAGGGAATTTTCGGCAATGGGCGAAAGCCTGACCGAGCAACGCCGCGTGAGTGATGACGG

CCTTCGGGTTGTAAAGCTCTGTTATACGGGACGAAGAAGACGGATGCCAACAGTATCCGT

CCGTGACGGTACCGTAAGAGAAAGCCACGGCTAACTACGTG

>7439 len=161 tot-seq=1

TGGGGAATATTGCACAATGGGGGAAACCCTGACCGAGCAACGCCGCGTGAGTGAAGAAGG

TTTTCGGATCGTAAAACTCTGTTGTTAGAGAAGAACAAGGATGAGAGTAGAATGTTCATC

CCTTGACGGTATCTAACCAGAAAGCCACGGCTAACTACGTG

>7440 len=161 tot-seq=1

TGGGGAATATTGGACAATGGAGGCAACTCTGATCCAGCAATTCTGTGTGTGTGAAGACGG

TCTTCGGATTGTAAAGCTCTGTTATACGGGACGAACGGCAAGGTGGTAAATAGCCATCAT

GAGTGACGGTACCGTAAGAGAAAGCCACGGCTAACTACGTG

>7441 len=161 tot-seq=1

TAGGGAATATTGCACAATGGGCGAAAGCCTGATGCAGCAACGCCGCGTGAGTGAAGAAGG

CCCTTGGGTCGTAAAGCTCTTTAGCCTGGAAAGAAGTGTCCATGAGTGAATAATTCATGG

ATGTGACGGTACCAGGAGAATAAGGTCCGGCTAATTTCGTG

>7442 len=161 tot-seq=1

TAGGGAATCTTCCACAATGGACGCAAGTCTGATGGAGCAACGCCGCGTGTGTGAAGAAGG

CCTTTTGGTTGTAAAGCACTTTAAGCGAGGAGGAGGCTACTTAGATTAATACTCTAGGAT

AGTGGACGTTACTCGCAGAATAAGCACCGGCTAACTCTGTG

>7443 len=161 tot-seq=1

TAGGGAATCTTCGGCAATGGACGGGAGTCTGACCGAGCAACGCCGCGTGAGTGAAGAAGG

TTTTCGGGTCGTAAAGCTCTGTTGTTAAGGAAGAACGAGTGTGAGAATGGAAAGTTCATG

CTGTGACGGTACTTAACCAGAAAGGGACGGCTAACTACATG

>7444 len=161 tot-seq=1

TAGGGAATCTTCCACATTGGGCGAAAGCCTGATGGAGCAACGCCGCGTGGGTGAAGAAGG

TCTTCGGATCGTAAAGCTCTGTTGTTAAGGAAGAACGAGTGTGAGAATTGAAAGTTCATG

CTGTGACGGTACTTAACCAGAAAGGGACGGCTAACTACGTG

>7445 len=161 tot-seq=1

TGGGGAATCTTCCGCAATGGACGAAAGTCTGACGGAGCAACGCCGCGTGAGTGAAGAAGG

TCTTCGGATCGTAAAACCCCGTTGTTAGAGAAGAACGTTGGTAGGAGTGGAAAATCTACC

AAGTGACGGTAACTAACCAGAAAGGGACGGCTAACTACGTG

>7446 len=161 tot-seq=1

TAGGGAATCTTCCACAATGGGCGAAAGCCTGACCGAGCAACGCCGCGTGAGTGAAGAAGG

TTTTCGGATCGTAAAGCTCTGTTGTTAGAGAAGAACGTTGGTAGGAGTGGAAAATCTACC

AAGTGACGGTAACTAACCAGAAAGCCACGGCTAACTACGTG

>7447 len=161 tot-seq=1

TGGGGAATCTTCCGCAATGGACGAAAGTCTGACGGAGCAACGCCGCGTGAGTGAAGACGG

CCTTCGGGTTGTAAAGCTCTGTGATCCGGGACGAAAGAGCCTGAGGTTAATAGCCTAAGG

AAGTGACGTTACCCTCAGAAGAAGCACCGGCTAACTCCGTG

>7448 len=161 tot-seq=1

TGGGGAATCTTCCACAATGGGCGCAAGCCTGATGGAGCAACACCGCGTGAGTGAAGAAGG

TTTTCGGATCGTAAAGCTCTGTTGTTAGAGAAGAACGTTGGTAGGAGTGGAAAATCTACC

AAGTGACGGTAACTAACCAGAAAGTCACGGCTAACTACGTG

>7449 len=161 tot-seq=1

TAGGGAATATTGCACAATGGAGGAAACTCTGATGCAGCCATGCCGCGTGTGTGAAGAAGG

CCTTCGGGTTGTAAAGCTCTGTTAATCGGGACGAATGGTTTGTGTGCAAATAGTGCATAG

ACATGACGGTACCGGAATAGAAAGCCACGGCTAACTACGTG

>7450 len=161 tot-seq=1

TGGGGAATCTTCCGCAATGGGCGAAAGCCTGACGGAGCAACGCCGCGTGAGTGATGACGG

CCTTCGGGTTGTAAAGCTCTGTGATCGGGGACGAACGGTCATTGGACGAATACTCCAGTG

AAGTGACGGTACCCGAATAGCAAGCCACGGCTAACTACGTG

>7451 len=161 tot-seq=1

TAGGGAATCTTCGGCAATGGACGGAAGTCTGACCGAGCAACGCCGCGTGAGTGAAGAAGG

TTTTCGGATCGTAAAGCTCTGTTGTAGGAGAAGAACGTGTGTGAGAGTGGAAAGTCCACA

CAGTGACGGTAACTTACCAGAAAGGGACGGCTAACTACGTG

>7452 len=161 tot-seq=1

TGGGGAATCTTCCGCAATGGACGGAAGTCTGACGGAACAACGCCGCGTGAGTGAAGACGG

CCTTCGGGTTGTAAAGCTCAGTGATCCGGGACGAAAGAGCCTGAGGTTAATAGCCTAAGG

AAGTGACGGTACCGGAAAAGCAAGCCACGGCTAACTACGTG

>7453 len=161 tot-seq=1

TGGGGAATCTTCCGCAATGGACGAAAGTCTGACGGAGCAACGCCGCGTGAGTGAAGACGG

CCTTCGGGTTGTAAAGCTCTGTGATCCGGGACGAAAGAGCCTGAGGCTAATAGCCTAAGG

AGGTGACGGTACCGGAAAAGCAAGCCACGGCTAACTACATG

>7454 len=161 tot-seq=1

TAGGGAATCTTCCGCAATGGACGAAAGTCTGACGGAGCAACGCCGCGTGAGTGAGGAAGG

CCTTCGGGTCGTAAAGCTCTGTTGTGAGGGAAGAAGCGGTGCCGTTCGAATAGGGCGGTA

CCTTGACGGTACCTCACCAGAAAGCCACGGCTAACTACGTG

>7455 len=161 tot-seq=1

TTAGGGATCTTCGTCAATGAGGGAAACCTTGAACGAGCAATGCCGCGTGAATGATGACGG

TCATTATGATTGTAAAATTCTGTTGTTAGGGATGAATTGTATATATAGGAAATGATATAT

GCTTGACAGTACCTTTCCAGAAAGCTCCGGCTAACTACGTG

>7456 len=161 tot-seq=1

TTAGGAATATTCGACAATGGGGGAAACCCTGATCGAGCAATGCCGCGTGAGTGATGAAGG

TCTTTATGATTGTAAAACTCTGTTGTTAAGGAAGAATAGTCTAAATAGGTAATGATTTAG

ATCTGACGGTACTTTTCAAGAAAGCTCCGGCTAACTACGTG

>7457 len=161 tot-seq=1

TGAGGAATTTTTCACAATAGACGAAAGTCTGACGGAGCAACGCCGCGTGAGTGAAGAAGG

TCTTCGGATTGTAAAACTCTGTTATACGGGACGAAAAAGACGGATGCCAACAGTATCCGT

CCGTGACGGTACCGTAAGAGAAAGCCACGGCTAACTACGTG

>7458 len=161 tot-seq=1

TGAGGAATATTGGTCAATGGACGCAAGTCTGACGGAGCAACGCCGCGTGAGTGAAGAAGG

ATTTCGGTTCGTAAAACTCTGTTGTTAAGGAAGAACGAGTGTGAGAATGGAAAGTTCATG

CTGTGACGGTACTTAACCAGAAAGGGACGGCTAACTACGTG

>7459 len=161 tot-seq=1

TGGGGAATCTTCCGCAATGGACGAAAGTCTGACGGAGCAACGCCGCGTGAGTGATGACGG

TCTTATAGATTGTAAAGTTCTTTTATATGGGAAGAAACGCTAAGATAGGAAATGATTTTA

GTTTGACTGTACCATTTGAATAAGTATCGGCTAACTATGTG

>7460 len=161 tot-seq=1

TGGGGAATATTGCGCAATGGGGGAAACCCTGACGGAGCAACGCCGCGTGAGTGAAGAAGG

CCTTCGGGTTGTAAAGCTCTGTTATTCGGGAAGAAAGATTCGTTTGTGAACAATGAACGG

AAGTGACGGTACCGAAAGAGAAAGCCACGGCTAACTACGTG

>7461 len=161 tot-seq=1

TGGGGAATTTTGGACAATGGGGGCAACCCTGATCCAGCCATGCCGCGTGCAGGATGAAGG

CCTTCGGGTTGTAAAGCTCTGTTAATCGGGACGAATGATCCTTGTGTGAATAATGCAAGG

ATTTGACGGTACCGGAATAGAAAGCCACGGCTAACTACGTG

>7462 len=161 tot-seq=1

TGGGGAATATTGCACAATGGGGGAAACCCTGATGGAGCAACGCCGCGTGAGTGATGACGG

CCTTCGGGTTGTAAAGCTCTGTTAATCGGGACGAATGGTCCTTGTGTGAATAATGCAAGG

ATTTGACGGTACCGGAATAGAAAGCCACGGCTAACTACGTG

>7463 len=161 tot-seq=1

TGGGGAATATTGCACAATGGGCGCAAGCCTGATGCAGCGACGCCGCGTGAGGGATGGAGG

CCTTCGGGTTGTAAAGCTCTGTTATTCGGGAGGAAAGATTCGTTTGTGAATAATGAACGG

AAGTGACGGTACCGAAAGAGAAAGCCACGGCTAACTACGTG

>7464 len=161 tot-seq=1

TGGGGAATATTGCACAATGGGCGCAAGCCTGATGCAGCGACGCCGCGTGAGGGATGGAGG

CCTTCGGGTTGTAAAGCTCTGTTAATCGGGACGAATGGTCCTTGTGTGAATAATGCAAGG

ATTTGACGGTACCGGAATAGAAAGCCACGGCTAACTACGTG

>7465 len=161 tot-seq=1

TGGGGAATATTGGGCAATGGGCGCAAGCCTGACCCAGCAACGCCGCGTGAGTGATGACGG

CCTTCGGGTTGTAAAGCTCTGTTAATCGGGACGAATGGTCCTTGTGTGAATAATGCAAGG

ATTTGACGGTACCGGAATAGAAAGCCACGGCTAACTACGTG

>7466 len=161 tot-seq=1

TGGGGAATTTTCCGCAGTGGACGAAAGTCTGACGGAGCAACGCCGCGTGAGTGATGACGG

CCTTCGGGTTGTAAAGCTCTGTTAATCGGGACGAATGGTCCTTGTGTGAATAATGCAAGG

ATTTGACGGTACCGGAATAGAAAGCCACGGCTAACTACATG

>7467 len=161 tot-seq=1

TAGGGAATATTCCACAATGAGCGAAAGCTTGATGGAGCGACACAGCGTGCACGATGAAGG

TCTTCGGATCGTAAAGCTCTGTTGTAAGAGAAGAACGTGTGTGAGAGTGGAAAGTTCACA

CAGTGACGGTAACTTACCAGAAAGGGACGGCTAACTACGTG

>7468 len=161 tot-seq=1

TGGGGAATCTTCCGCAATGGACGAAAGTCTGACGGAGCAACGCCGCGTGAGTGGTGACGG

CCTTCGGGTTGTAAAGCTCTGTTAATCGGGGAGGAAGGGAGTAAAGTTAATACCTTTGCT

CATTGACGTTACCCGCAGAAGAAGCACCGGCTAACTCCATG

>7469 len=161 tot-seq=1

TAGGGAATCTTCGGCAATGGGGGCAACCCTGACCGAGCAACGCCGCGTGAGTGATGACGG

CCTTCGGGTTGTAAAGCTCTGTTAATCGGGACGAATGGTCCTTGTGTGAATAATGCAAGG

ATTTGACGGTACCGGAATAGAAAGCCACGGCTAACTACGTG

>7470 len=161 tot-seq=1

TAGGGAATCTTCCACAATGGACGAAAGTCTGATGAAGCAATGCCGCGTGTGTGAAGAAGG

CCTTATGGTTGTAAAGCACTTTAAGCGAGGAGGAGGCTACTGAGACTAATACTCTTGGAC

AGTGGACGTTACTCGCAGAATAAGCACCGGCTAACTCTGTG

>7471 len=161 tot-seq=1

TGGGGAATCTTCCGCAATGGGCGCAAGCCTGACGGAGCAATGCCGCGTGAGTGAAGAAGG

TTTTCGGATCGTAAAGCTCTGTTGTAAGAGAAGAACGTGTGTGAGAGTGGAAAGTTCACA

CAGTGACGGTAACTTACCAGAAAGGGACGGCTAACTACGTG

>7472 len=161 tot-seq=1

TTAGGAATATTCGTCAATGGGGGAAACCCTGAACGAGCAATGCCGCGTGAGTGATGAAGG

TCTTAATGATTGTAAAACTCTGTTGTGAAGGAAGAATAGCCATCATAGGTAATGATGATG

GTGTGACGGTACTTCACCAGAAAGCCCCGGCTAACTACATG

>7473 len=161 tot-seq=1

TTAGGAATATTCGTCAATGGGGGAAACCCTGAACGAGCAATGCCGCGTGAAGGATGACGG

TCCTATGGATTGTAAACTTCTGTTGTTAGGGAAGAACGACCTGTGTAGGAAATGAAACAG

GAGTGACGGTACCTTTCAAGAAAGCTCCGGCTAACTACGTG

>7474 len=161 tot-seq=1

TAGGGAATCTTCCGCAATGGACGAAAGTCTGACGGAGCAACGCCGCGTGAGCGAAGAAGG

TCTTCGGATTGTAAAGCCCTGTTGTCAGGGAAGAACAAGTACCGTTCGAATAGGACGGTA

CCTTGACGGTACCTGACCAGAAAGCCACGGCTAACTACGTG

>7475 len=161 tot-seq=1

TGGGGAATTTTTCGCAATGGGGGAAACCCTGACGAAGCAACGCCGCGTGGAGGATGAAGG

CCTTCGGGTCGTAAACTCCTGTCGACCGGGACGAACGCTATTGGGCCCAATACGCCCGAT

AGTCGACTGTACCGGTGGAGGAAGCCACGGCTAACTCTGTG

>7476 len=161 tot-seq=1

CTAAGAATATTCCGCAATGGGCGAAAGCCTGACGGAGCGACGCCGCGTGGACGATGAAGG

CCGGAAGGTTGTAAAGTCCTTTTATGACAGAGGAATAATGAAAGGAGTGGAAAGCCTTTC

AGATGACTGTAGGTCATGAATAAGCACCGGCTAATTACGTG

>7477 len=161 tot-seq=1

TAGGGAATCTTCCACAATGGACGCAAGTCTGATGGAGCAACGCCGCGTGAGTGAAGAAGG

TTTTCGGATCGTAAAGCTCTGTTGTTAGAGAAGAACAAGTACCGTTTGAATAAGGCGGTA

CCTTGACGGTACCTAACGAGAAAGCCCCGGCTAACTACGTG

>7478 len=161 tot-seq=1

TAGGGAATCTTCGGCAATGGACGAAAGTCTGACCGAGCAACGCCGCGTGAGTGAAGAAGG

TTTTCGGATCGTAAAACTCTGTTGTTAGAGAAGAATGTTGATTAGAGTGGAAAATTAATC

AAGTGACGGTATCTAACCAGAAAGGGACGGCTAACTACGTG

>7479 len=161 tot-seq=1

CCGACAGACACCTCGTACAGGAGAGCTCTGGCTGGCATCTGGCGGGTGCCCCTCTGGGAT

GAAGCTTCCAGAGGAAGGAACAGGCAGCAATCTTTGCTGTTCTGCAGTCTCTGCTGGTGA

TACCCAGGTAAACAGGGTCTGGAGTAGACCCCCAGCAAACT

>7480 len=161 tot-seq=1

TGGGGAATCTTCCGCAATGGGCGCAAGCCTGACGGAGCAACGCCGCGTGAGTGAAGAAGG

TCTTCGGATCGTAAAGCTCTGTTGACGGGGACGAACGTGCGAAGGGTGAATAATCCTTTG

CAATGACGGTACCTGTCGAGGAAGCCACGGCTAACTACGTG

>7481 len=161 tot-seq=1

TAGGGAATCTTCGGCAATGGACGGAAGTCTGACCGATCAACGCCGCGTGAGTGAAGAAGG

TTTTCGGATCGTAAAGCTCTGTTGTAAGAGAAGAACGAGTGTGAGAGTGGAAAGTTCACA

CTGTGACGGTACCTGCAGAAGAAGCCCCGGCTAACTACGTG

>7482 len=161 tot-seq=1

TGGGGAATATTGCGCAATGGGGGAAACCCTGACGGAGCAACGCCGCGTGAGTGATGAAGG

TCTTCGGATTGTAAAGCTCTGTTAATCGGGACGAAAGAGTTCTGTGTGAATAATGCAGAA

AAGTGACGGTACCGGAATAGAAAGCCACGGCTAACTCCGTG

>7483 len=161 tot-seq=1

CATCAGAGCGGTGATTTCGTTAGAGTCCAGCGCGAGGGCTTTGTCGATCATTGCGCGAGT

CTGGGCGGTCATATGCTGGCTGGCCTGGTAATAAAGCACCGTCGCCAGCGCCGCATACAG

TTCAGCGTTCTCACCACGTAGTTGCAACGCCTGACGGTACG

>7484 len=161 tot-seq=1

TGGGGAATATTGCACAATGGGCGCAAGCCTGATGCAGCCATGCCGCGTGTATGAAGAAGG

CCTTCGGGTTGTAAACCGCTTTTGATTGGGGAGGAAGGGAGTAAAGTTAATACCTTTGCT

CATTGACGTTACCCGCAGAAGAAGCACCGGCTAACTCCGTG

>7485 len=161 tot-seq=1

TGGGGAATTTTGGACAATGGGGGAAACCCTGATCCAGCCATGCCGCGTGCGGGATGAAGG

CCTTCGGGTTGTAAAGCTCTGTTATACGGGACGAAAAAGACGGATGCCAACAGTATCCGT

CCGTGACGGTACCGTAAGAGAAAGCCACGGCTAACTACGTG

>7486 len=161 tot-seq=1

TAGGGAATCTTCCACAATGGACGCAAGTCTGATGGAGCGACGCCGCGTGAGTGAAGAAGG

GTTTCGGCTCGTAAAGCTCTGTTGTTGGTGAAGAACGTTGGTAGGAGTGGAAAATCTACC

AAGTGACTGTAACTAACCAGAAAGGGACGGCTAACTACGTG

>7487 len=161 tot-seq=1

TGAGGAATATTGGTCAATGGGCGAAAGCCTGATGCAGCGACGCCGCGTGAGTGAAGAAGG

TCTTCGGATTGTAAAGCTCTGTTATACGGGACGAAAAAGACGGATGCCAACAGTATCCGT

CCGTGACGGTACCGTAAGAGAAAGCCACGGCTAACTACGTG

>7488 len=161 tot-seq=1

TAGGGAATCTTCCGCAATGGACGAAAGTCTGACGGAGCAACGCCGCGTGAACGATGAAGG

TTTTCGGATCGTAAAGTTCTGTTGTTAGGGAAGAACACGTACCGTTCGAATAGGGCGGTA

TCTTGACGGTACCTAACGAGAAAGCCCCGGCTAACTACGTG

>7489 len=161 tot-seq=1

TTAGGAATATTCGGCAATGGGGGAAACCCTGACCGAGCAATGCCGCGTGTGAGATGAAGG

TCCTTCGGATTGTAAATCACTTTTATTTGGGAAGAACTGTAAGCAGAGGAAATGATGCTT

ACTTGACGGTACCTTTTGAATAAGCCCCGGCTAACTACGTG

>7490 len=161 tot-seq=1

TAGGGAGTTTTCGGCAATGGGGGAAACCCTGACCGAGCAACACCGCGTGAGTGATGAAGG

TCTTCGGATTGTAAAACTCTGTTGTGTGGAAAAAATGACAGGTAGAGTGGAAAGCTACTT

GTTTGATGGTACCACACCAGAAAGCCACGGCTAACTACGTG

>7491 len=161 tot-seq=1

TCGAGAATCATTCACAATGGGGGAAACCCTGATGGTGCGACGCCGCGTGGGGGAATGAAG

GTCTTCGGATTGTAAACTTCTGTTGTTAGGGAAGAATGACCTATATAGGAAATGATATAG

GAGTGACGGTACCTTTCAAGAAAGCTCCGGCTAACTACGTG

>7492 len=161 tot-seq=1

CCACCCTACTCCCAGCAACCACCGTCCCAGACCCCTCATGCCCAACCTTCGTATCAGCAG

CAGCCACAGTCTCAACCACCACAGCTCCAGTCCTCTCAGCCTCCATACTCCCAGCAGCCA

TCCCAGCCTCCACATCAGCAGTCCCCGGCTCCATACCCCTC

>7493 len=161 tot-seq=1

TGGGGAATCTTCCGCAATGGACGAAAGTCTGACGGAGCAGCGCCGCGCGAGTGAAGAAGG

TCTTCGGATTGTAAAACTCTGTTATACGGGACGAAAAAGGCGGATGCCGACAGTATCCGT

CCGTGACGGTACCGTAAGAGAAAGCCACGGCTAACTACGTG

>7494 len=161 tot-seq=1

TAGGGAATCTTCGGCAATGGGGGGGACCCTGACCGAGCAACGCCGCGTGAGTGAAGAAGG

TTTTCGGATCGTAAAGCTCTGTTGTTAAGGAAGAACGGGAGTAGGAGTGGAAAGCCTACT

TTGTGACGGTACTTAACCAGAAAGGGACGGCTAACTACGTG

>7495 len=161 tot-seq=1

TGGGGAATCTTGCACAATGGGCGAAAGCCTGATGCAGCGACGCCGCGTGAGTGAAGACGG

CCTTCGGGTTGTAAAGCTCTGTGATCCGGGACGAAAGAGCCTTGGGTAAATAGCCTAAGG

AAGTGACGGTACCGGAAAAGCAAGCCACGGCTAACTACGTG

>7496 len=161 tot-seq=1

TGGTTATAAGGTGGGTACTGGGAAGAAGGAAGCTGGAAGTGATGGTCTTCGTTTTTATGG

GAACAAAATGCATTTACATCTCAATGTTAACCACCCTGTTAACTGACCTCTCACATCCCA

ATACTATCTCTCTCTTTTTCTCTTTACCCAGTATTGAATTC

>7497 len=161 tot-seq=1

TAGGGAATCTTCGGCAATGGGGGGAACCCTGACCGAGCAACGCCGCGTGAGCGAAGAAGG

CCTTCGGGTTGTAAAGCTCTGTTATTCGGGAAGAAAGATTCGTTTGTGAACAATGAACGG

AAGTGACGGTACCGAAAGAGAAAGCCACGGCTAACTACGTG

>7498 len=161 tot-seq=1

TAGGGAATATTGCACAATGGAGGGAACTCTGATGCAGCGACGCCGCGTGAGTGAAGAAGG

TTTTCGGATCGTAAAGCTCTGTTGTTAAGGAAGAACGAGTGTGAGAATGGAAAGTTCATG

CTGTGACGGTACTTAACCAGAAAGGGACGGCTAACTACGTG

>7499 len=161 tot-seq=1

TGGGGAATATTGCACAATGGGCGCAAGCCTGATGCAGCGACGCCGCGTGAGGGATGGAGG

CCTTCGGGTTGTAAACCTCTTTTAATCGGGACGAATGGTCGGTGTGCAAATAGTGCATGG

ACATGACGGTACCGGAATAGAAAGCCACGGCTAACTACGTG

>7500 len=161 tot-seq=1

TAGGGAATTTTCGTCAATGGGGGGAACCCTGAACGAGCAATGCCGCGTGAGTGAGGAAGG

TCTTCGGATCGTAAAGCTCTGTTGTTAAGGAAGAACGAGTGTGAGAATGGAAAGTTCATG

CTGTGACGGTACTTAACCAGAAAGGGACGGCTAACTACGTG

>7501 len=161 tot-seq=1

TGAGGAATATTGGTCAATGGACGGAAGTCTGAACCAGCCAAGTAGCGTGCAGGATGACGG

CCCTATGGGTTGTAAACTGCTTTTGTCAGAGACGAAAAGGGACGTACGAATAATACGTTC

CGCTGACGGTATCTGAAGAATAAGCACCGGCTAACTACGTG

>7502 len=161 tot-seq=1

TGGGGAATATTGGACAATGGAGGAAACTCTGATCCAGCAATTCTGTGTGAGTGAAGACGG

CCTTCGGGTTGTAAAACTCTGTGATTCGGGACGAAAGATAAGCAGACGAATAATCTGCAT

AAGTGACGGTACCGAAAAAGCAAGCCACGGCTAACTACGTG

>7503 len=161 tot-seq=1

TGGGGAATCTTCCGCAATGGACGAAAGTCTGACGGAGCAACACCGCGTGAGTGATGACGG

TCTTCGGATTGTAAAGCTCTGTTAATCGGGACGAATGGTTTGTGTGCAAATAGTGCATAG

ACATGACGGTACCCTGTGAGGAAGCCCCGGCTAACTACGTG

>7504 len=161 tot-seq=1

TGGGGAATCTTCCGCAATGGACGAAAGTCTGACGGAGCAACGCCGCGTGAGTGATGACGG

TCTTCGGATTGTAAAGCTCTGTTAATCGGGACGAATGGCGTAGCGGTCAATACCCGTTAC

GAGTGACGGTACCGTAAGAGAAAGCCACGGCTAACTACGTG

>7505 len=161 tot-seq=1

TGGGGAATATTGCACAATGGGCGCAAGCCTGATGCAGCAACGCCGCGTGAGTGAAGAAGG

GTTTCGACTCGTAAAGCTCTGTTGTCGGGGACGAACGTGGAGACGGCGAATAGCCGTTTC

CAATGACGGTACCTGACGAGGAAGCCACGGCTAACTACGTG

>7506 len=161 tot-seq=1

TGGGGAATCTTCCGCAATGGACGAAAGTCTGACGGAGCAACGCCGCGTGAGTGATGACGG

TCTTCGGATTGTAAAGTCCTGTTATATGGGACGAACAGGACATCGGTCAATACCCGGTGT

CTTTGACGGTACCGTAAGAGAAAGCCACGGCTAACTACGTG

>7507 len=161 tot-seq=1

TGGGGAATTTTGGACAATGGGGGCAGCCCTGATCCAGCCATGCCGCGTGCAGGATGAAGG

TCTTCGGATTGTAAAGCTCTGTTAATCGGGACGAATGGTCGGTGTGCAAATAGTGCATGG

ACATGACGGTACCGGAATAGAAAGCCACGGCTAACTACGTG

>7508 len=161 tot-seq=1

TGGGGAATCTTCCGCAATGGACGAAAGTCTGACGGAGCAACGCCGCGTGAGTGATGACGG

TCTTCGACTCGTAAAGCTCTGTTGTCGGGGACGAACGTGGAGACGGCGAATAGCCGTTTC

CAATGACGGTACCTGACGAGGAAGCCACGGCTAACTACGTG

>7509 len=161 tot-seq=1

TAGGGAATCTTCCACAATGGACGAAAGTCTGATGGAGCAACGCCGCGTGAATGAAGAAGG

CCTTCGGGTCGTAAAGCTCTGTTAATCGGGACGAATGGTCGGTGTGCAAATAGTGCATGG

ACATGACGGTACCGGAATAGAAAGCCACGGCTAACTACGTG

>7510 len=161 tot-seq=1

TTAGGAATATTCGTCAATGGGGGAAACCCTGAACGAGCAATGCCGCGTGAAGGAAGAAGG

TCCTATGGATTGTAAACTTCTGTTGTAAAGGAAGAACGACCTAAGTAGGAAATGACTTAG

GAGTGACGGTACTTTATTAGAAAGCTCCGGCTAACTACGTG

>7511 len=161 tot-seq=1

TGGGGAATATTGCACAATGGGCGCAAGCCTGATGCAGCAACGCCGCGTGAACGATGAAGG

TCTTCGGATCGTAAAGTTCTGTTAATCGGGACGAATGGTTTGTGTGCAAATAGTGCATAG

ACATGACGGTACCGGAATAGAAAGCCACGGCTAACTACGTG

>7512 len=161 tot-seq=1

TAGGGAATATTCCACAATGAGCGAAAGCTTGATGGAGCGACACAGCGTGCACGATGAAGG

TCTTCGGATCGTAAAGCTCTGTTGTTAGAGAAGAACGTTGGTAGGAGTGGAAAATCTACC

AAGTGACGGTAACTAACCAGAAAGGGACGGCTAACTACGTG

>7513 len=161 tot-seq=1

TAGGGAATCTTCGGCAATGGACGAAAGTCTGACCGAGCAACGCCGCGTGAGTGAAGAAGG

TTTTCGGGTTGTAAAACTCTGTTATACGGGACGAATGTAGGCATGGTTAATACCCATGCG

TAGTGACGGTACCGTAAGAGAAAGCCACGGCTAACTACGTG

>7514 len=161 tot-seq=1

TGGGGAATCTTGCACAATGGGCGAAAGCCTGACCGAGCAACGCCGCGTGAGTGAAGAAGG

TTTTCGGATCGTAAAACTCTGTTGGTAGAGAAGAACGTTGGTGAGAGTGGAAAGCTCATC

AAGTGACGGTAACTACCCAGAAAGGGACGGCTAACTACGTG

>7515 len=161 tot-seq=1

TGGGGAATATTGCACAATGGAGGGAACTCTGATGCAGCGACGCCGCGTGAGTGAAGACGG

CCTTCGGGTTGTAAAACTCTGTGATTCGGGACGAAAGATAAGTAGACGAATAATCTGCAT

AAGTGACGGTACCGAAAAAGCAAGCCACGGCTAACTACGTG

>7516 len=161 tot-seq=1

TGGGGGATATTGCACAATGGAGGCAACTCTGATGCAGCAACGCCGCGTGAGTGATGACGG

CCTTCGGGTTGTAAAGCTCTGTTAATCGGGACGAATGGTCTTTGTGTGAATAATGCAAAG

ATTTGACGGTACCGGAATAGAAAGCCACGGCTAACTACGTG

>7517 len=161 tot-seq=1

TGGGGAATCTTCCGCAATGGGCGCAAGCCTGACGGAGCAATGCCGCGTGAACGAAGAAGG

TCTTCGGATTGTAAAGTTCTGTCCTTATCGAAGAGAGGGTATAGAGTGAAAAATGATATA

CTAGGACGGTAGATGAGGAGGAAGCCCCGGCTAACTACGTG

>7518 len=161 tot-seq=1

TTAGGAATATTCGACAATGGGGGAAACCCTGATCGAGCAATGCCGCGTGAAGGATGAAGG

TCCTATGGATTGTAAACTTCTGTTGTTAGGGAAGAATGACCTATATAGGAAATGATATAG

GAGTGACGGTACCTTTCAAGAAAGCTCCGGCTAACTACGTG

>7519 len=161 tot-seq=1

TGGGGAATCTTCCGCAATGGACGGAAGTCTGACGGAGCAACGCCGCGTGAGTGATGACGG

CCTTCGGGTTGTAAAGCTCTGTTAATCGGGACGAAAGGCCTTCTTGCAAATAGTGAGAAG

GATTGACGGTACCCAAGGAGGAAGCCACGGCTAACTACGTG

>7520 len=161 tot-seq=1

TAGGGAATTTTGCACAATGGAGGCAACTCTGATGCAGCGACGCCGCGTGAAGGATGAAGG

TTTTCGGATCGTAAAGCTCTGTTGTTAAGGAAGAACGAGTGTGAGAATGGAAAGTTCATA

CTGTGACGGTACTTAACCAGAAAGGGACGGCTAACTACGTG

>7521 len=161 tot-seq=1

TAGGGAATCTTCGGCAATGGGGGGAACCCTGACCGAGCAACGCCGCGTGAGTGAAGAAGG

TTTTCGGATCGTAAAGCTCTGTTGTTAAGGAAGAACGAGTGTGAGAATGGAAAGTTCACA

CTGTGACGGTATCTTACCTGAAAGGGACGGCTAACTACGTG

>7522 len=161 tot-seq=1

TAGGGAATCTTCCGCAATGGGCGAAAGCCTGACCGAGCAACGCCGCGTGAGTGAAGAAGG

TTTTCGGATCGTAAAGCTCTGTTGTAAGAGAAGAACGGGTACGAGAGTGGAAAATTCATA

CTGTGACGGTATCTTACCAGAAAGGGACGGCTAACTACGTG

>7523 len=161 tot-seq=1

TAGGGAATCTTCGGCAATGGACGCAAGTCTGACCGAGCAACGCCGCGTGAGTGAAGAAGG

TTTTCGGATCGTAAAACTCTGTTGTTAGAGAAGAACAAGGATGAGAGTGGAAAGTTCACA

CTGTGACGGTATCTTACCAGAAAGGGACGGCTAACTACGTG

>7524 len=161 tot-seq=1

TGGGGAATATTGGGCAATGGGCGCAAGCCTGACCCAGCAACGCCGCGTGAAGGAAGAAGG

TTTTCGGATCGTAAAACTCTGTTGGTAGAGAAGAACGTTGGTGAGAGTGGAAAGCTCATC

AAGTGACGGTAACTACCCAGAAAGGGACGGCTAACTACGTG

>7525 len=161 tot-seq=1

TGGGGAATATTGCACAATGGGCGAAAGCCTGATGCAGCGACGCCGCGTGAGGGATGACGG

CCTTCGGGTTGTAAACCTCTGTTATACGGGACGAAAAAGACGGATGCCAACAGTATCCGT

CCATGACGGTACCGTAAGAGAAAGCCACGGCTAACTACGTG

>7526 len=161 tot-seq=1

TAGGGAATTTTTCACAATGGGCGCAAGCCTTATGAAGCAATGCCGCGTGAACGATGAAGG

TCTTATAGATTGTAAAGTTCTTTTATATGGGAAGAAACGCTAAAATAGGAAATGATTTTA

GTTTGACTGTACCATACGAATAAGGACCGGCTAATTCCGTG

>7527 len=161 tot-seq=1

TAGGGAATCTTCGGCAATGGACGGAAGTCTGACCGAGCAACGCCGCGTGAGTGAAGACGG

CCTTCGGGTTGTAAAACTCTGTGATTCGGGACGAAAGATAAGCAGACGAATAATCTGCAT

AAGTGACGGTACCGAAAAAGCAAGCCACGGCTAACTACGTG

>7528 len=161 tot-seq=1

TGGGGAATATTGGACAATGGGGGGAACCCTGATCCAGCCATGCCGCGTGTGTGAAGAAGG

CCTTTTGGTTGTAAAGCACTTTAAGCGAGGAGGAGGCTCCCAGTATTAATACTACTGGAG

AGTGGACGTTACTCGCAGAATAAGCACCGGCTAACTCTGTG

>7529 len=161 tot-seq=1

TGGGGAATTTTGGACAATGGGGGCAACCCTGATCCAGCCATGCCGCGTGCAGGATGAAGG

TCTTCGGATTGTAAAACTCTGTTGTCAGGGACGAATGTACTGATTTACAATACACTTCGG

TGTTGACGGTACCTGACGAGGAAGCCACGGCTAACTACGTG

>7530 len=161 tot-seq=1

TGGGGAATCTTCCGCAATGGACGAAAGTCTGACGGAGCAACGCCGCGTGAGTGAAGACGG

CCTTCGGGTTGTAAAGCTCTGTGATCCGGGACGAAAGAGCCTGAGGTGAATAGCTTAAGG

AAGTGACGGTACCGGAGTAGAAAGCCACGGCTAACTACGTG

>7531 len=161 tot-seq=1

TGGGGAATCTTCCGCAATGGACGAAAGTCTGACGGAGCAACGCCGCGTGAGTGATGACAG

TCTTCGGATTGTAAAGCTCTGTTAATCGGGACGAATGGTTTGTGTGCAAATAGTGCATAG

ACATGACGGTACCTGACGAGGAAGCCACGGCTAACTACGTG

>7532 len=161 tot-seq=1

TAGGGAATCTTCCACAATGGACGCAAGTCTGATGGAGCAACGCCGCGTGAGTGATGAAGG

TCTTCGGATTGTAAAACTCTGTTGTCAGGGACGAATGTACTAATCTATAATACATTTCGG

TATCGACGGTACCTGACGAGGAAGCCACGGCTAACTACGTG

>7533 len=161 tot-seq=1

TGGGGAATCTTCCGCAATGGACGAAAGTCTGACGGAGCAACGCCGCGTGAGTGATGAAGG

TCTTCGGATTGTAAAACTCTGTTGTCAGGGACGAATGTACTGATCTATAATACACTTCGG

TATTGACGGTACCGGAATAGAAAGCCACGGCTAACTACGTG

>7534 len=161 tot-seq=1

GTGGGGAATATTGCACAATGGGCGCAAGCCTGATGCAGCCACGCCGCGTGTATGAAGAAG

GCCTTCGGGTTGTAAAGTACTTTCAGCGAGGAGGAAGGTGCTGAGGTTAATAACCTCAGC

AATTGACGTTACTCGCAGAAGAAGCACCGGCTAACTCCGTG

>7535 len=161 tot-seq=1

TAGGGAATCTTCCACAATGGACGAAAGTCTGATGGAGCAATACCATGTGAACGATGAAGG

TCTTTTTGATTGTAAAGTTCTTTTATATGGGAAGAAACGCTAAGATAGGAAATGATTTTA

GTTTGACTGTACCATTTGAATAAGTATCGGCTAACTATGTG

>7536 len=161 tot-seq=1

TGGGGAATCTACCGCAATGGACGAAGGTCTGACGGAGCAACGCCGCGTGAGTGATGACGG

CCTTCGGGTTGTAAAGCTCTGTTAATCGGGGCGAATGGTCTTTGTGTAAATAATGCAAAG

ATTTGACGGTACCGGAATAGAAAGCCACGGCTAACTACGTG

>7537 len=161 tot-seq=1

TCGAGAGGCTTCGGCAATGGGGGCAACCCTGACCGAGCGACGCCGCGTGGGTGATGAAGA

TTCTCGGATCGTAAAACCCTGTCAGGTGGGATGAAAAATCATTCGGTTAACACCCGAGTG

ACATGACAGTACCACCAGAGGAAGCTCCGGCCAACTCCGTG

>7538 len=161 tot-seq=1

CATGACCCCCAGTCCCAGGCTCCGGGGTTACACTGCCTTAGTTCCACTGTACTCCTGTGT

GACCTTGTGGATGCTAACAGTACCTGCTAATAGGGTTGTTATGGGAATGATAAGAACTAA

AGTAGATAAATGACTTTGGGCAGTGCCAGGCACATGTTAAG

>7539 len=161 tot-seq=1

TGAGGAATATTCCACAATGGGCGAAAGCCTGATGGAGCAATGCCGCGTGAGTGAAGACGG

CCTTCGGGTTGTAAAACTCTGTGATTCGGGACGAAAGATAAGCAGACGAATAATCTGCAT

AAGTGACGGTACCGAAAAAGCAAGCCACGGCTAACTACGTG

>7540 len=161 tot-seq=1

TAGGGAATATTCCACAATGAGCGAAAGCTTGATGGAGCGACACAGCGTGCACGATGAAGG

TCTTCGGATCGTAAAGCTCTGTTGTTAAGGAAGAACGAGTGTGAGAATGGAAAGTTCATG

CTGTGACGGTACTTAACCAGAAAGGGACGGCTAACTACGTG

>7541 len=161 tot-seq=1

TGGGGAATCTTCCGCAATGGACGAAAGTCTGACGGAGCAACGCCGCGTGAGTGTAGAAGG

TCCTCGGATTGTAAAACCCTGTTATACGGGACGAAAAAGGCGGATGCCAACAGTATCCGT

CCGTGACGGTACCGTAAGAGAAAGCCACGGCTAACTACGTG

>7542 len=161 tot-seq=1

TGGGGAATATTGCACAATGGAGGGAACTCTGATGCAGCGACGCCGCGTGAGTGAAGAAGG

TCTTCGGATTGTAAAACTCTGTTATACGGGACGAAAAAGACGGATGCCAACAGTATCCGT

CCGTGACGGTACCGTAAGAGAAAGCCACGGCTAACTACGTG

>7543 len=161 tot-seq=1

TGGGGAATATTGGACAATGGAGGGAACTCTGATCCAGCAATTCTGTGTGTGTGAAGAAGG

TTTTAGGACTGTAAAACTCTGTTATACGGGACGAAAAAGGCGGATGCCAACAGTATCCGT

CCGTGACGGTACCGTAAGAGAAAGCCACGGCTAACTACGTG

>7544 len=161 tot-seq=1

GAGATTACGTTGTGACTGGAGTCAGACGTGTGCTCTTCCGATCTCCTACGGGAGTCAGCA

GTGGGGAATATTGGACAATGGAGGCAAATCTGATCCAGCAATTCTGTGTGTGTGAAGAAG

GTTTTACGGTACCTACAGAAGAAGCGACGGCTAAATACGTG

>7545 len=161 tot-seq=1

TGGGGAATTTTGGACAATGGGGGCAACCCTGATCCAGCAATGCCGCGTGTGTGAAGAAGG

CCTTCGGGTCGTAAAGCTCTGTTGTAAGAGAAGAACGTGTGTGAGAGTGGAAAGTTCACA

CAGTGACGGTAACTTACCAGAAAGGGACGGCTAACTACGTG

>7546 len=161 tot-seq=1

CCTTGGCGCTTCGCCGGGCATGCACATCTTCAGAGGGCCGCGCAATTCGCGCTGTACCAC

GCCAAGCTCTTCCGCCGTGGAGAGCAAGGTGTCGAGCAAGGGCTTTGCGCGTTCGTAGAG

CAGCCGGCCGGCTTCCGTCATGCGCAGATGGCGGGTACTTC

>7547 len=161 tot-seq=1

TGGGGAATCTCCCGCAGTGGACGAAAGTCTGACTGAGCAACGCCGCGTGAGTGAAGAAGG

TCTTCGGATTGTAAAACTCTGTTATACGGGACGAAAAAGGCGGATGCCAACAGTATCCGT

CCGTGACGGTACCGTAAGAGAAAGCCACGGCTAACTACGTG

>7548 len=161 tot-seq=1

TGGGGAATCTTGCGCAATGGGCGAAAGCCTGACGCAGCCATGCCGCGTGAATGATGAAGG

TCTTAGGATTGTAAAGCTCTGTTATACGGGACGAACGGCAAGGTGGTAAATAGCCATCAT

GAGTGACGGTACCGTAAGAGAAAGCCACGGCTAACTACGTG

>7549 len=161 tot-seq=1

TGGGGAATCTTCCGCAATGGGCGAAAGCCTGACGGAGCAACGCCGCGTGAGTGAAGACGG

CCTTCGGGTTGTAAAGCTCTGTTATTCGGGAAGAAAGAGTCGCTTGTGAACAATGAGCGA

AAGTGACGGTACCGAAAGAGAAAGCCACGGCTAACTACGTG

>7550 len=161 tot-seq=1

TGGGGAATCTTCCGCAATGGGCGCAAGCCTGACGGAGCAACGCCGCGTGAGTGAAGAAGG

GTTTCGACTCGTAAAGCTCTGTTGTCGGGGACGAATGTCAGGACTCAGAATATGGGTTCT

GAATGACGGTACCTGACGAGGAAGCCACGGCTAACTACGTG

>7551 len=161 tot-seq=1

TGGGGAATATTGCACAATGGGCGCAAGCCTGATGCAGCGACGCCGCGTGCGGGATGAAGG

CCTTCGGGTTGTAAAGCTCTGTTATACGGGACGGACGGCAAGGTGGTAAATAGCCATCAT

GAGTGACGGTACCGTAAGAGAAAGCCACGGCTAACTACGTG

>7552 len=161 tot-seq=1

TGGGGAATATTGCACAATGGGCGAAAGTCTGACCGAGCAACGCCGCGTGAGTGATGAAGG

TTTTCGGATCGTAAAACTCTGTTGTTAGAGAAGAACAAGTACCGTTTGAATAAGGCGGTA

CCTTGACGGTACCTAACGAGAAAGCCCCGGCTAACTACGTG

>7553 len=161 tot-seq=1

TGAGGAATATTGGTCAATGGGCGAGAGCCTGAACCAGCCAAGTAGCGTGCAGGATGACGG

CCCTATGGGTTGTAAAACTCTGTCCTCATCCAAGAGAAGGGAAGGTAGTAACTGACCTTT

CCAGGACGGTAGATGAGGAGGAAGCCCCGGCTAACTATGTG

>7554 len=161 tot-seq=1

TGGGGAATATTGGACAATGGGGGGAACCCTGATCCAGCCATGCCGCGTGTGTGAAGAAGG

CCTTATGGTTGTAAAGCACTTTAAGTGAGGAGGAGGCTCTTCTAGTTAATACCTAGGATG

AGTGGACGTTACTCGCAGAATAAGCACCGGCTAACTCTGTG

>7555 len=161 tot-seq=1

TGAGGAATATTGGTCAATGGACGCGAGTCTGAACCAGCCAAGTAGCGTGAAGGATGACTG

CCCTATGGGTTGTAAACTTCTTTTATATGGGGATAAAGTGGTCCACGTGTGGACTTTTGT

ATGTACCATATGAATAAGGATCGGCTAACTCCGTGCCAGCG

>7556 len=161 tot-seq=1

TGGGGAATATTGGGCAATGGAGGAAACTCTGACCCAGCAACGCCGCGTGAGTGAAGAAGG

TTTTCGGATCGTAAAGCTCTGTTGTTAGAGAAGAACGTTGGTAGGAGTGGAAAATCTACC

AAGTGACGGTAACTAACCAGAAAGGGACGGCTAACTACGTG

>7557 len=161 tot-seq=1

TAGGGAATCTTCCACAATGGGTGCAAACCTGATGGAGCAATGCCGCGTGAATGAAGAAGG

TCTTCGGATCGTAAAGCTCTGTTGTAAGAGAAGAACGTGTGTGAGAGTGGAAAGTTCACA

CAGTGACGGTAACTTACCAGAAAGGGACGGCTAACTACGTG

>7558 len=161 tot-seq=1

TGGGGAATATTGCACAATGGGGGAAACCCTGATGCAGCGACGCCGCGTGGGCGAGGAAGT

ATTTCGGATCGTAAAGCTCTGTTGTAAGTCAAGAACGAGTGTGAGAGGGGAAAGTTCACG

CTGTGACGGTAGCTTACCAGAAAGGGACGGCTAACTACGTG

>7559 len=161 tot-seq=1

TGAGGAATATTGGTCAATGGGCGTAACCCTGACCGAGCAACGCCGCGTGAGTGAAGAAGG

TTTTCGGATCGCAAAGCTCTGTTGTAAGAGAAGAACGTGTGTGAGAGTGGAAAGTTCACA

CAGTGACGGTAACTTACCAGAAAGGGACGGCTAACTACGTG

>7560 len=161 tot-seq=1

TAAGGAATATTCCACAATGGGGGAAACCCTGATGGAGCAATACCGCGTGAGTGAAGAAGG

TTTTCGGATCGTAAAGCTCTGTTGTAAGAGAAGAACGTGTGTGAGAGTGGAAAGTTCACA

CAGTGACGGTAACTTACCAGAAAGGGACGGCTAACTACGTG

>7561 len=161 tot-seq=1

TGGGGAATTGTTCACAATGGGCGCAAGCCTGATGACGCAACGCCGCGTGGGGGATGAAGG

TCTTCGGATCGTAAACCCCTGTCGAATGGGACGAAAGACTCGCGAGTTAATACTTCGCGG

GAGTGACGGTACCGTTAAAGGAAGCCACGGCTAACTCTGTG

>7562 len=161 tot-seq=1

TAGGGAATATTGCGCAATGGAGGAAACTCTGACGCAGCGACGCCGCGTGGGTGATGAAGG

CCTTCGGGTCGTAAAGCCCTGTCGGAAGGGAAGAAACACGGTATGGCTAACATCCATACC

GCTTGACGGTACCTTTAAAGGAAGCACCGGCTAACTACGTG

>7563 len=161 tot-seq=1

TAGGGAATCTTCGGCAATGGGGGCAACCCTGACCGAGCAACGCCGCGTGAGTGAAGAAGG

TTTTCGGATCGTAAAGCTCTGTTGTAAGAGAAGAACGTGTGTGAGAGTGGAAAGTTCACA
CAGTGACGGTAATGGGTAAAGAAGCACCGGCTAACTACGTG
>7564 len=161 tot-seq=1
TGGGGGATATTGGACAATGGGGGAAACCCTGATCCAGCGATGCCGCGTGAGGGAAGAAGG
TTTTCGGATTGTAAAGCTCTGTTGTAAGAGAAGAACGTGTGTGAGAGTGGAAAGTTCACA
CAGTGACGGTAACTTACCAGAAAGGGACGGCTAACTACGTG
>7565 len=161 tot-seq=1
TAGGGAATCTTCGGCAATGGGGGAAAGCCTGATCCAGCCATGCCGCGTGTGTGAAGAAGG
CCTTTTGGTTGTAAAGCACTTTAAGCGAGGAGGAGGCTACTTGGATTAATACTCTAGGAT
AGTGGACGTTACTCGCAGAATAAGCACCGGCTAACTCTGTG
>7566 len=161 tot-seq=1
TGAGGAATATTGGTCAATGGACGAGAGTCTGAACCAGCCAAGTAGCGTGAGTGAAGACAG
CCTTCGGGTTGTAAAACTCTGTGATTCGGGACGAAAGATAAGCAGACGAATAATCTGCAT
AAGTGACGGTACCGAAAAAGCAAGCCACGGCTAACTACGTG
>7567 len=161 tot-seq=1
TGGGGAATCTTCCGCAATGGACGAAAGCCTGACGGAGCAACGCCGCGTGAGTGAAGAAGG
TCTTCGGATTGTAAAACTCTGTTACACGGGACGAGAAAGGCGGATGCCAACAGTATCCGT
CCGTGACGGTACCGTAAGAGAAAGCCACGGCTAACTACGTG
>7568 len=161 tot-seq=1
TGGGGGATATTGCACAATGGGGGAAACCCTGACGCAGCGACGCCGCGTGAGCGAAGAAGG
CCTTCGGGTCGTAAAGCTCTGTTGTAAGTCAAGAACGAGTGTGAGAGTGGAAAGTTCACA
CTGTGACGGTAGCTTACCAGAAAGGGACGGCTAACTACGTG
>7569 len=162 tot-seq=1
GAAAGCCCCAGGAGTCTCCTCCATAAGGGTGCAGAGTCGGTATTGACAGATAACATTCCT
TCAACACCCCACTCCCACCCCCCAGCACAGGCCTTTTTCGCTCTTCCTAGCGGCATTCCT
AGCATCCAATCTGCTCCTGTAAAAAAGGACAGATTGGACCGG
>7570 len=162 tot-seq=1
TCTGTAGACCTGGCTGGCTTCTAACTCAGAGAACTATGTGCCTCTGCCTCCTGAGTGCTG
ACTGAGTGTATGTTGGGATGAATGGATGTCCTGGAATTCTACTTTACTCTCTGAGACTAT

TCTTCTGTTGTGGAGACAGATATAAAAAATAGGGGTTTCTGT

>7571 len=162 tot-seq=1

CATTAAGTAAAACCTCGTTTAAGAAATGGATAGTAAAGGCCCAGGGGAGTGGATGAAGCC

GCAACTGATTTGGAGGGGTCCTGGCCCAACTGAGACAAGACATGACAGCCAACAGTCACT

ATGCCTCACCGCCGTTTAGTAGCTGGTATTCCACACTGTAGC

>7572 len=162 tot-seq=1

CATCCCGAGGGCTGGGAACATTGGGAGACAAGAGTGAGAGGAAATGCAATCTGCATTTTC

CTTTTCCTACTTCTTGCTTTGACAGGAAGTGCCCTCTCTGTTCTGACAAATCGAACCCTT

TGACAGTGTGAGCTGAAAGAAATAATTCCTTCTATGTAGCTG

>7573 len=162 tot-seq=1

TGGGGAATATTGCACAATGGGCGCAAGCCTGATGCAGCCATGCCGCGTGTATGAAGAAGG

CCTTCGGGTTGTAAAGTACTTTCAGCGGGGAGGAAGGGAGTAAAGTTAGTAACTGGCCTT

TATTTGACGGTAATTACTTAGAAAGTCACGGCTAACTACGTG

>7574 len=162 tot-seq=1

ACCAGCCTCCTAAAGAGCCTACATGTGGGCAAGTACCTCCTCCACTTACCCTGTGCCCAG

GCTGTGACCGGCCAAAGAGCACCTCGGAGACGTCCACACCATCAAAGCGCCGTCCTTGAG

GTAAGCTGGCCTGGGCCAGGGCTACCACAGTTGGAAAAATGT

>7575 len=162 tot-seq=1

TGGGGAATATTGGACAATGGACCAAAAGTCTGATCCAGCAATTCTGTGTGCACGATGAAG

TTTTTCGGATCGTAAAGCTCTGTTGTTAGAGAAGAACGTTGGTAGGAGTGGAAAATCTAC

CAAGTGACGGTAACTAACCAGAAAGGGACGGCTAACTACGTG

>7576 len=162 tot-seq=1

TGGGGAATATTGCACAATGGGCGCAAGCCTGATGCAGCCATGCCGCGTGTATGAAGAAGG

CCTTCGGGTTGTAAAGTACTTTCAGCGGGGAGGAAGGGAGTAAAGTTAGTCTCGTGAGAC

TTTTTGCATGTACCTTATGAATAAGGACCGGCTAATTCCGTG

>7577 len=162 tot-seq=1

TGAGGAATATTGGTCAATGGGCGAGAGTCTGAACCAGCCAAGTAGCGTGCAGGATGACGG

CCCTATGGGTTGTAAAGCTCTGTTAATCGGGACGAATGGTTTTTGTGTGAATAATGCAAA

GATTTGACGGTACCGGAATAGAAAGCCACGGCTAACTACGTG

>7578 len=162 tot-seq=1

CCCGGGCCAGCAGCTCCCAGTGTGACCGGACAAAAACACGTAGGGGCAGGGACGGTCACC

ACCCCCAGGGACACAACCCCTGGTCTTGGACCAGTAGAGGACACGGAGGGTTCAGACCCC

TCCTCAGACCCTCCCCACATATGAAGCAGCGGCTAACCAACT

>7579 len=162 tot-seq=1

AAACCAAGGAGCATCTAGAGAGCCAGCAAACCACCAGAGCTTGCGGACAGGCCTTCAGAA

GGAACCAATCCTGCTGACACCTTGATCTTGAACTTTTAGCCTCCAGAGCTGTGAGGCCAT

ACATTCCTGTTGTTTAAGCTCCCCAGTTTGTGGTACTGTGTT

>7580 len=162 tot-seq=1

GAGATAACAGAGATAATTTCAACAAAATACAACTTGGCTAAGATAGAGTTATGAAAAGTA

GAGAGACTCTTAGGTCAACATGAAAGTTCCTGAAGTTGCCAGTACCCAAGAGGAATCTTG

AATGATCAGCAAGATTTATAGCGGCTAGAAGAATATTTATTC

>7581 len=162 tot-seq=1

CGGGCCTGCCGCTCAAGCAGACTGCGGATTCGCTCGGAGCGTCCTGTCTGCAGGGCCAGC

AGCTCCTCTTCCACCTGGGGCATCCGGAACAGAGGTTAGAGTCGGGGGCAGGAGCCCACC

CTCCCTCCCCAGTCCTGGAGGCTGGGCCCCTTACCCGCTGCT

>7582 len=162 tot-seq=1

TTTTTTTTTTCAAGCAGAAGACGGCATACGAGATCGACAAGTGACTGGAGTTCAGACGTG

TGCTCTCCGATCTCCTATGGGAGGCAGCAGATACTCGGAAAGCCAGCGGGGACCCCGTGT

CTTCTGACTCACTGCCATGGCCGACGCCAGACCTCCTTACTC

>7583 len=162 tot-seq=1

TTTCAAGCAGAAGACGGCATACGAGATCGGTGTGTGACTGGAGTTCAGACGTGTGCGCTT

CCGATCTCCTATGGGAGGCAGCAGGATTACACACATGGAAGTGGAGAAGGCAATGTGGTG

ATGGAGACAGATTGGAGTGATGTGGCCACAAGCCAAGGACTG

>7584 len=162 tot-seq=1

TGAGGAATATTGGTCAATGGACGAGAGTCTGAACCAGCCAAGTAGCGTGCAGGATGACGG

CCCTATGGATCGTAAAGCTCTGTTGTAAGAGAAGAACGAGTGTGAGAGTGGAAAGTTCAC

ACTGTGACGGTATCTTACCAGAAAGGGACGGCTAACTACGTG

>7585 len=163 tot-seq=1

TAAGACTTGAACCACAGTGAGCCGCCATCACACATTCAAAGGGGCTGTCATCCAAAGGAC

AGATGAGGAATTCTGGGGAGCTTGCTGTGCAGAACCCAGACCATCACACTACTAAGGAGG

ATGGGAAATGAGGCAGTCCCATCCGTGACAGTCTTCACATCTC

>7586 len=163 tot-seq=1

AGGTAAGCCACTAGCCTTTTTCAAGGTCTCAAAGTCTGAGTAAGGACTCCTCTGGTGACC

CCTGCTCTCCCTTTTACATCCACAAAACACCTCCCCTTCCTTGGTTAGGGGGTACAACAG

GGCAGCTAAGGTCGCCAACCCAGGTGTCTAGAGTCTGTAGAAG

>7587 len=163 tot-seq=1

TCCCTCAGCCTGCACGGAGGTGTGGCGGGAGAGGCACAGGCAGCAACTGCGGCTGCGCGC

CGCGCTTGTGGGCCAGCTAGAGTTCCGGGTGGGAGTGGGTTTGGCAGCCCCGGGCAGCCG

GCTGCCCCAACGGCCCCAGGCAGTGAGGGGCTTAGCACCTGGA

>7588 len=163 tot-seq=1

GCACAGGCTTTTCTGGAGGGGAGGCTGGTGATGGCTCGGGGGCTTCCCAGCCAGCCCTGT

TACTTACTTGTCTGCTGACTATCCTGTCCATGGGGTGTCCCAGCCCCACTCCCTCCTCCC

TCAGACCCAGGAATCCAGGCTCCCAGCCCCTCCTCTCTCAGAC

>7589 len=163 tot-seq=1

GGAAGAAAATGCCACAAAGGACATTTATTAGTAAGGAAGAAAAGCAAGCACCAGGATTTA

AGGCAGGAAGAGATAGGCAAACTCTACTGTTCTGTGCAAATGCAGTCGGTTTGCTGCCCT

TAGCCATAAAGCTGCTAATCCCTGAGCCTCAAAGGGAAAAAAC

>7590 len=163 tot-seq=1

TAGGGAATCTTCCACAATGGACGAAAGTCTGATGGAGCAACGCCGCGTGAGTGAGGAAGG

TTTTCGGATCGTAAAGCTCTGTTGTTGGTAGTGAAGAAAGATAGAGGTAGTAACTGGCCT

TTATTTGACGGTAATCAACCAGAAAGTCACGGCTAACTACGTG

>7591 len=163 tot-seq=1

TAGGGAATCTTCCACAATGGACGAAAGTCTGATGGAGCAACGCCGCGTGAGTGAAGAAGG

TTTTCGGATCGTAAAGCTCTGTTGTTGGTAGTGAGGAAAGATAGAGGTAGTAACTGGCCT

TTATTTGACGGTAATTACTTAGAAAGTCACGGCTAACTACGTG

>7592 len=163 tot-seq=1

CCTGGCCCTTCCCTGCATGCACAGGAGGGAGAGAGAGGTGGAGAGGTGGGTTGGGGTGCC

TGGCACATCCCAGAGTGGGGGACCCACCATGCCCCTCCTCTTGCATAATCGAGGGAGTTG

GCACTCTTGCTGCTCAAGAGCTCCAGGCCAGCCCAGGGAAGTC

>7593 len=163 tot-seq=1

TACACGCGTGTGCCCGCATGCACATGTGTGTGGTCCACAGGACTCTCCAGGCACGAGGGG

GAAGGGGTGGTCCTTGGGCCTGGAGAAGGCTCAAGGCTCAGGAGCCCCGGGATGAAGGTA

GCAGCACCACAAGGGGCATGGAGGGGGCACTCGCCAGACCCAT

>7594 len=163 tot-seq=1

TGAGGAATATTGGTCAATGGGCGAGAGCCTGAACCAGCCAAGTCGCGTGAAGGAAGACTG

CCCGCAAGGGTTGTAAAGCTCTGTTATACGGGACGAAAAAGACGGATGCCAACAGTATCC

GTCCGTGACGGTACCGTAAGAGAAAGCCACGGCTAACTACGTG

>7595 len=163 tot-seq=1

TTTTTTTTTTCAAGCAGAAGACGGCATACGAGATATTCTCGTGATTGGAGTTCAGACGTG

TGCTCTTCCGATCTCCTACGGGCGGCAGCAGTAGGCCTTTCTAACAAACAAGCACGTACA

CCCCCTGAGTCTGTAAAATACCAAAACAATGACGACAGCACCA

>7596 len=163 tot-seq=1

CCCGCCGACGTCTCCGCGTCCCCCGGATTCCAGGTTCGCGACCCCCGCCGTCTTCGCGCC

TCCGGGACTCTAGCAGCGGTCTCCGCGACCCCCTAGGATCCAGCCACTGTACCCGTGCTC

CCCAAACCCAGGCTCCAGCCGTCGTCTCCGCGCTCCCGGGGAT

>7597 len=163 tot-seq=1

TCCATTCAGTCAGTTGCGGGGTTTAGAATTTTATGTTTGGTTTACAGAGTAAATATGAGG

TGAGGGCTAAAAGCACATTTTCTGAGTCCCAAATCAAGATACATGGGCTAACTAGGGATT

TTTGTGCCATTTTCAGATGTGAGACGCTGTCTTGGAGGCATGG

>7598 len=163 tot-seq=1

TAGGGAATCTTCCGCAATGGGCGAAAGCCTGACGGAGCAACGCCGCGTGAGTGATGAAGG

TCTTCGGATCGTAAAACTCTGTTATTAGGGAAGAACAAATGTGTAAGAGTGGAAAGTTCA

CACAGTGACGGTAACTTACCAGAAAGGGACGGCTAACTACGTG

>7599 len=164 tot-seq=1

TGGGGAATAGAGCACAATGGGAGAAAGCCTGATGCAGAGACGCCGCGAGAGCGAAGAAGT

ATTTCGGTAAGTAAAGCTCTATCAGAAGGGAAGAAAATGACGGTACCTTACTAAGAAGCT

CCGGCTAAATACGTGCCACAGCCGCGGTCATCCCCAGATCGGAA

>7600 len=164 tot-seq=1

CACTAGAGCAAGCGAAAGGCATGAAACAGATTCTCCCCTAGAGCTGTAAGAGGCAGTGTG

GCCCTGGCAACACCTTGCTTTAGGACTTCTAACCTCTAGAATAATGAGATAATGAGAGAG

TATATTTCTGTTGGTTTCGGCCATGCGGTTTGTGGTAACTGGTT

>7601 len=164 tot-seq=1

GCAGTAGGGAATCTTCCACAATGGACGTAAGTCTGATTCCTCCACGCCGCGTGAGTGAAG

AAGGTTTTCGGATCGTAAAGCTCTGTTGTTGGTGAAGAAGGATAGAGGTAGTAACTGGCC

TTTATTTTCCTGTGATCAACCAGAAAGTCACGGCTAACTACGTG

>7602 len=164 tot-seq=1

AGCAGCGAGGAATTTTTCACAATGGACGAAAGTCCGATGGAGCAATTCCACGTGAACGAT

GAAGGTCTTCTGATTGTAAAGTTCTTTTATTTAGGAAAAAAAGCGCGCTAGGAAATGAGT

GCGCCTTGATGGTACCAATTGAATAAGTGACAGCTAACTATGTG

>7603 len=164 tot-seq=1

CCAATCGCCTCCTACCAGGCCCCACCTCCAACATTAGGGATTACAATTCAACATGAGATT

TGGTGGGGGACACAGGTCCAACCTATATCAATAGGCCCACAATCCCAATGCACAATCCCC

AACCTGGAAGAGCTCCAAAAACTCAGAGGGTTTTTGTAACCCAC

>7604 len=164 tot-seq=1

TAGGGAATATTGCACAATGGGGGAAACCCTGATGCAGCGACGCCGCGTGAGCGAAGAAGG

CCTTCGGGTCGTAAAGCTCTTTTATATGGGAAGATAATGACGGTACCATAAGAAAAAGCC

CCGGCTAACTACGTGCCAGCAGCCGCGGTATCATTAGATCGGAA

>7605 len=164 tot-seq=1

TAGGGAATCTTCCACAATGGACGAAAGTCTGTTGGAGCAATACCATGTGAACGACGAAGG

TCTTTTTGATTGTAAAGTTCTTTTATGAGGGATAACAACTGATAGTACCTCATGAATAAG

TGACAGCAAACTATGTGCCAGCAGCCGCGAATCCGGAGATCGGA

>7606 len=164 tot-seq=1

GGTGGTGGGGCCTGGAAGCTGGGAGGGGTGCTGGGCCTGCTGGGGTGGGATCAGTGGGAG

AGCTGATTGTTTTGTCTCCTTCTGAGCACAAGAGTAATCTGTAACCCTTCCACAGCCTGT

GATGCGGGCCTGGAGCTCTGCACTGAGCTGCATGTTATCTGCAC

>7607 len=164 tot-seq=1

CAGGGTTTGAAGGGATTGACTCCAATTTTGAGAGGTTTTACTTTTGACAAAATGCAAACA

GCATTGCATGCTACAGGGAAATCTTTAACGAAAGGAAGGGTTAATCAATGTGTCAAGCTT

CATTGTTGTCTTATTTTAAGAAATTGCCAGAGCCACGCTAACCT

>7608 len=164 tot-seq=1

CGAAGTTCACTCTTTTCCCCACCTCCACTATCTCCAATAAACTCTCAATGTAAAGTTAAA

CTGATTGGGCTGTGGGTCAGTTGCATTACATTTAAATAAGCACAGAGAACATCAAAAACT

TCAGGAAACACAGTGCAGATATCTGCAGCTACCCTTTGATAAAC

>7609 len=164 tot-seq=1

TGGATTACCTGAGTGGGCCCCAAATCCAATGACAGATGCCATAAGAGTGAGGCAGGCAGA

GATTAGACAGAAGAGAACTCACAGAGACACACAGGGAGAAAGCAACATGAAGATGGAGGC

AGAGATTGGAGTGATGTGTCTACAAGCCAAGAACACAAAGGATC

>7610 len=164 tot-seq=1

GGAGCCAATAAAAATAATTAATTAATTTCATTTTGGAAAAAAAAAAATCCTCTCTTCCTG

ATAAACAGAACAGGGCAACAACATATTGTTCAGTGAGTTATGGTGGCTAAAATCTCACTC

TGAAAATTATTTGTCATGGCAACGGGTGAGTGTCACCCCTGAAG

>7611 len=165 tot-seq=1

CAAAGTCATGCTGCGACATTTAGGTCCAGTGTCTTTTTAACAAGATGAGGCATCCAACCT

CATGGCCAGGATCCTCAGAGTGTCACCTCTGTGGTCCTGTATCCAAAGTTGCCGCCTGTA

TCTTCCACTAGGTTTTCTTGTAAGAAGGGTGCTAAAATAACCTGG

>7612 len=165 tot-seq=1

GGGACTCTTAGCTGAATGAATTATTAAATGGGCTCTCAGAGGGAATAGATTGCTGGGGGA

TTGCTTTTAATGGATATATTTGTTCCTATGTGACCTATCCACCACGAGGTGAGCCACGTT

GCTCTGCCATGTTTTGCACAATACTTTGCCGCACCTTGCACAGAC

>7613 len=165 tot-seq=1

GACTTGGGAAGCTCTTTCTGCACCAGGCCAGTTCTGTTCCCACAGGACTCCTTGCTAGAA

ATAGCTCAGAAGAGAGTGATTCTGGAAACTTCTCAGCTCCGCTACAGAATCCAGGACTAG

ATGAAGAAAGGTCTGAGTGGCCAGATGTGGCACAGGCCTGTCATT

>7614 len=165 tot-seq=1

CAGGGAAGCCCAAAGTGGGCAGTGGGGCAACCCTGACCGAGCAACGCCGCGTGAGTGAAG

AAGGTTTTCGGATCGTAAAGCTCTGTTGTAAGAGAAGAACGTGTGTGAGAGTGGAAAGTT

CACACAGTGACGGTAACTTACCAGAAAGGGACGGCTAACTACGTG

>7615 len=165 tot-seq=1

AGAAGTAGGGAATATTAAAAAATGGACGAAAGTCTGATGGAGCAACGCCGCGTGAGTGAA

GAAGGTTTTAGGATAGTAAAGATATGTTGTTGGTGAAGAAGGAGAGAGGTAGTAAATGGA

ATTTATTCGACGGTAATCAACCAGAAAGTCACGGCTAACTACATG

>7616 len=165 tot-seq=1

AAGCCTCTGAATAATGTCCACAAGACTAGAGAAGCCCCTTGGAGCCTGCAGCACCTTCAG

AACTGAATTTCAATAAATCCAGAGGGGGCTTAAGATTTGTGTGAATATCTAAACTGTGCT

GACAGCTAAAGGAGAAAGGGGTAGGGAGGGAAAATGATCTGTTCG

>7617 len=165 tot-seq=1

GGATGAGCCCTTGGACATCACAGCCCTGATGGCTGCCATCCAGCACGGGCACGAGGCCGT

GGTGCGTCTACTGATGGAGTGGGGCGCGGACCCCAACCACGCAGCCCGGACCGTGGGCTG

GAGCCCGCTGATGCTGGCCGCACTCACTGGGCGGCTTGGAGTGGC

>7618 len=165 tot-seq=1

GCCCGACACGACCTCCACAACCTCGGCCGCAGCAGGCATCGATCAGGGGCCTCTGCAGCG

TGCTTGGCCACTGCGTGGTGGCAGGAAGACTGGACCCTCGATTCACTTTGCTTCTGGACG

TCCTGGTGGCTAATGAGATGGTAGCATTTCCTCCACTGGTAAGCA

>7619 len=165 tot-seq=1

TACCACGATTTTCGTCTATTCTGACATACTGAAATATATCCTCCACACACAGTTCATCAT

TGACTGCCGTAATCACTGCATTTATTTCATTTTCCAGCCGGATATCACCGACTTTTATTG

GCAAAATATCGCTCTCATTGTACTGATAGACAAAAATATCGCCCT

>7620 len=165 tot-seq=1

GTCAGGCAGTCTGATAGATGTCGACCCAGTGCCAGCATGGCACATCGCGGCCCTCCCCAC

CTGGGCTAGAGCTGGGACTGGGGAAAGCCTTCCCACAGAGATGGCACCTGATTGGCATCC

TAATCATGATCAGTAGTCAGCTGGGGTGGAGGATTTTAGGAAATG

>7621 len=165 tot-seq=1

AACTAAGGTACATTCCTAGGCTTGTACCATAAAACAAGGAGACAACTGAGAGGTGAGATG

CACAGGTGGAAAATGCCTTGTACTTCCCCTGAGCTGATGAGATTGCACGTATGGAGGAAA

CTATCTTAGAGTAAGAGTAAAGGATTCCAGTGAGGGGACCACCGC

>7622 len=165 tot-seq=1

GGAGTTCAGACGTGTGCTCTGCCGATCTCCGACGGGAGGCAGCAGCACGTGCAAAGGCCC

TAAGGAATAAGGAGGCACTCACATTGAGAAGCCCAGTCTGGCCAGAGTGCAGAGGGTGAA

AGGCCTTGGCTGAATGAGATGTGAGGTGGGAAAGGGCAGTGTGGG

>7623 len=165 tot-seq=1

TGGGGAATATTGCACAATGGGGGGAACCCTGATGCAGCGACGCCGCGTGAGCGAAGAAGG

CCTTCGGGTCGTAAAGCTCTTTTATATGGGAAGATAATGACGGTACCATAAGAAAAAGCC

CCGGCTAACTACGTGCCAGCAGCCGCGGTTTCGCAGATCGGAAGA

>7624 len=165 tot-seq=1

TAAATAGAGACAGAGACCCACAGAGCACAGGGCCCACATCACCATGGGAACCCAGATGAA

GAAAGTTCAGGGAGAATGCTCCCAAGCCTTTGAGACTCAAGACTCCTAAACCAAAAAGAC

CTCGAGGGGTCATTCAGTTCAGTCTTCTGCCTCCAGACTGGACAG

>7625 len=165 tot-seq=1

TTACAAACCACGTGTCTCATGCCTAGGGGTTGTGGGCAAACTCTACCAGTCATGCAGTCT

GGAATTTTCTCTGGTTAACCTTCAGTCTCCTGGGATACAGCCTAGTGATGGGGAAAGGGA

TGGACTTTGAAATCAAAACCAGTATGGAGCTCCAGCTCTGATTTA

>7626 len=165 tot-seq=1

TGTTGTAACGAAGTCGGGATGGCACGAATAATCATTTTAAGAATTTTAGTAACGGTAATA

ATAATGTTAATTAAACCGCATAAGAACACCATGCACATTGCATGCTGCCAACTAAAGCCT

AAGCCTTTTACGACTGTGAACGTAAAGAATGCATTTAAGCCCATA

>7627 len=166 tot-seq=1

GACAACGCCTGATCCACCCCTTTGGCTCAGAAGGGGGCCACAAAATCTCCTTTAAGGAGG

TTTTTAACATGATAATCTCAGAGACTCTTTTGGGCAGGTGAGGAAGGACAAGCATTCACA

CATAGAAAGCTGGTAATGGGCTGTTAAGAGGACAATACCTTCTATT

>7628 len=166 tot-seq=1

GCTCAGGAATGTTCCTCTCCACCTTCTGCGTGAATTACCAGCAGGACATTGGGCTGAAAA

CGTTGACTTTTGTCCCATTGCTTAGGGCTGTCTGTGCTGTTCTTTTCATTTCTGAGTAAA

GTTCTTTCTTGCTAATTCAGAGACCACACTACTGCTGATGTGGCTA

>7629 len=166 tot-seq=1

AGTAGGAAGCACCAGTAATATGTCCCCCTATCTAGACAAAAATTGCACTGGCAGGATCTG

TCTGATTTAACTATTTTCAAACACTGGAGTCCATTAAGTTTTGCAACTTCCAGGGGAAGT

TTTGAATAGTCAACTGCATTAATTTTGATCAATTTCAGCTCTTAGC

>7630 len=166 tot-seq=1

CCTGCCAGCAGGTCATGTAGGACTTTGTAGGCACCATAAGGTCTCAGGCTTTTCCACTGG

GTGAACGGGAGCTGCACGCACAGGAGGGACACGGTCTGACTGACATTTCCAGTGCGGTTT

TATCAGCACTGAAACCATCCCCCTTTAGCCCTTAAGGCCCACACCC

>7631 len=166 tot-seq=1

AATTGCTTGAACCCAGGAGGCAGAGGTTGCAGTGAGCCGAGATTGTGCCACTGCACTCTA

GCCTGGGCAACAAGAGCGAAACTCCATCTCAAAAAAAAAAAAAGAAAAGAAAGAAAGAAA

GAAAACATGAATGAATGGCCGGGCACTATGGCTCACACTTGTAATC

>7632 len=166 tot-seq=1

TTTTTTTCAAGCAGAAGACGGCATACGAGATCAAGTGGTGACTGGAGTTCAGACGTGTGC

TCTTCCGATTTCCTACGGAAGGCAGCAGAAGGGTAGAAGAAAGAGAAATAATTTCTACCA

AGCTTATGTCCAGTTCACAAGATGTATTTTGCAAATGTGTCATCTC

>7633 len=166 tot-seq=1

GACACTGCACTTGCCTCAGCCCCATCCTGACTCCCCGTGGATCATCGTGCATGCAGCAGC

TGTGACCCCCAGAGGCCTCTAGTTCAGCATAAGCTGAGGCAAAGGGGGCCCCCAGGTTCC

CTCTACTGGTGTGAAGCCCAGCCGGCAAGGGGACTGGGGATCGGCG

>7634 len=166 tot-seq=1

TGGGGAATATTGGGCAATGGGGGAAACCCTGACCCAGCAACGCCGCGTGAGGGAAGAAGG

TTTTCGGATTGTAAACCTCTGTCCTATGGGAAGAAGAAAGTGACGGTACCATAGGAGGAA

GCCCCGGCTAACTACGTGCCAGCAGCCGCGTAATATGGAGATCGGA

>7635 len=166 tot-seq=1

GGCCCTTCGGAGGAGCGGATGGAAGGCCTTCTCAGGTAAAGGGCAGAGCACCACACGCCT

TTCTGACCACCACCCCCAGGGCCCAGCCGCTTCCAGGGACCAAGACAAGAGGCCCCTGTG

GTCTTCTAGGTGAGCTGTAGTCAGCCTCTGTCGCTTCTGAATAATC

>7636 len=166 tot-seq=1

CCATATCAGAAACCTGAGACCCAAGGGGCTACTATCGGGGGAACAGGCGGTCAGCCAACC

CTGATAAGAGGTGCCTGCCCGGAGTGTCTCACAAACACCCTGTCATCAGAGGGCTGTGCT

GGGTCACGAGGCAGTCAATCCACTGCCATGCAGGGCTGGGGGTTGT

>7637 len=166 tot-seq=1

GCTGGGGGTGGGGACGGAGGCAAATGAGAGACAGGGTAAGATCTGAGCTGGTTCCCGGGC

TCAGGCTGGAGTTCATGGCAGCTCACTCCAGCAGCAGGGTCGGGGCCCATGGGCAGGGAT

GGGTGGAAGGATTTTTCCAGCCTCACTGAGAGCAGGTCTTGGCCTG

>7638 len=167 tot-seq=1

ATGTTTTTCTAGGAAGGTGGTGCCTCTCTTCCACAGCTTCTCGTTTTGACTCTGGTACTT

AGACTGGAGTTGCTTCATCTGGTTCTGGAATTCTTGAACGGCAGTGGTTTCCACTGCAGG

GGTATGAAAGAACCAAAATAGTCCCACTGCAAGGATGGCAACAAGTC

>7639 len=167 tot-seq=1

GAGGCAGCAGAAGAGATGGGAGGTGGGGTTTAATGTTTGGGGGGAAACATCTCAATGAGG

GTGTCTTTTTAGAGGGCTCATCTTGACCCCTTCTTTTCTGTGCCTACCTCTAGGCCACCA

TATGGTGAGCTGACCTTCCTCAGGCTCCCTTACCCTTGATGCCCTTC

>7640 len=167 tot-seq=1

GGGTGCTGTGTAAATCTGTGCTGAGGCTCAGGTGGCTCGCACCCAGAGTCAATAGTCTGC

GATGCCGAACCCAAGAGGAATGCCTTTTATCCGATTGTTTAGGGTCCAGGCTTCCCGTGC

GCGTTCTGTGGACTCCACGGGAAATTGGCGATGCCGAGATGTTAATG

>7641 len=167 tot-seq=1

CTCCCTGAATGTTCCCTATTTCATCTAAAACCTTGGTATTGCATCACACACTTGGTACTC

CAAGTCCAGATAGTGTATAGGTTCCATTTATGAAATCAATGAACATTGTCAGCATTTTAA

AAAGCTTATGTAAATGACCGCCCCACCATAAGATATATAGAAAAAGG

>7642 len=167 tot-seq=1

GAGAGGGAATGGAAATTGGTGGAAGAAGCATCAGGAGACATAGGACAGGGGAGTCTCCTG

CTAGTCTATGGGGTGACCCTAGCTGAGACTCCGATCAGCCGGGGGTACAGAGACTTAAGG

GGCCACATCCTGTAGCCAGGAGGGAGTTCAGTGGAGGGAAGGGGATA

>7643 len=167 tot-seq=1

GGCCTTGGAGCAGCTGCATCTATAGGCAGGGAGCAGAGAACAATTGAAGATTGTGCCACT

CAGTTTGTTTTCTTTACCTGGTGCTAGACTTGGTATACCTGCCAGGGAATGTCACTGCCC

ATAGTGGGCACATCCTCCTCCTTCAGTTAATGCATGCAAGGTAACTG

>7644 len=167 tot-seq=1

GGGGAGAAGAATGGAGGACATTGAGTTTTGGGGAAGAAAGGGTAGCTGCAGGTGGGGGCT

AGGGCTGCTGAACACAGAAGCCACTGCCCTGTCCACCTCTGTCCACAGTACAGGTCCTTC

AGACACTTACACAGTCTTGGCTCCTGTGTTCCCTGCAGGACGCTGGT

>7645 len=167 tot-seq=1

GGAGGCAGCAGGGAGGAAGAGGGGGCAATGGGCGAGAGCCTGAACCAGAAAAGTCGCGTG

AGGGAAGACGGTCCGAGGGAGGGGAAACCGCGGGGGTCGGGGAGCAAAGAGGCCTCGGGA

GGGCAAAAGTGAGAGGACCCGAAGAAAAAGAATCGGCTAACTCCGTG

>7646 len=167 tot-seq=1

CTCCAAGCAACCACACTAAGGAAGCCCAAGGCCAGAGTGCCCCCAGCCCATTTTCCCAAC

TAGTGGGTCCCATTTTCTTTATCACTTAGTTGAGAGTCGGCTCTGTTCCAGACCGGCAGC

CTAGAGCAGTGGGCTGGAGCAAAGAGATGCAGCTGAGCTAGAGCAAC

>7647 len=167 tot-seq=1

CTAGAAAAGTGCCCACATTTTCGATTCTAAAACAGTTTCAAGGTTGGTTGAATGTCGGCA

AACACTTCAGGGAAAGCCTGTTTTTTGAGGTGACCCTGTAGGATACTCATGGAATGTGAA

GGCTTGCCTATAAAACAGCAAGGTTTTCTGCGACATGGAAACACCAG

>7648 len=167 tot-seq=1

GATAGAGGTGAACTATAAGGGGAACTTTATCCCCTATGTCCCAGCACATACCTACGCTAC

GATGCTCCAGCTTGATGAGCCTATCCAGACTTCTTGGCTCAAGGGGGTACTTGCTTCTGT

AGAGCTACGTGGCAATGGCCCGATCTACTGGGACTCGGAGAATATGC

>7649 len=167 tot-seq=1

TAGAAGCTGTTCCCCATCATCCCCCAGGCCCCATCCTGGGAGCCCCGTCACCAACCACCT

TTCTCTTTAGTCCTCAGCTGGCCTCGATATTGTCACCAAGTAAACTTTGGGGTCAACAAA

CATGGGTAGGAACCAACATTAGCACGGTCAGCAAATGCGCCCCTGCT

>7650 len=167 tot-seq=1

TAAATCTACAGGGAGAGATAGAGTCCAATACAATGATAGTTGAGAACTTCAACATCCTAC

TGTCAGCATTGGACAGTTCATCTAGACATAAAATCAACAAAGAAACATTAGACTTAAGCT

GCACTTTAGACCAAATGGACCTAACAGATATTTTCAGAATATTTCAT

>7651 len=167 tot-seq=1

ATTCTGTTCTTCCCTCACCCTCTGACTCCACAACCAGGTCCATCTGGCTGCACATTCCCA

GCACCTGCTGTCTATCTGCATGCCACAGAAACCCTGAGCCGGGTGCGCTGGCTGAGTTGG

AACTGGGTCCTGGGGATGAGGCTAACCAGGCATTGCGTGGATCTCAC

>7652 len=167 tot-seq=1

GATAATCTGCCGGAACTGTTCCAATCCGACACCGAGCAGCTTTTCTACAGCGCTGTCCAC

CCCCTTTGAAGTGATATGGGCTTTTTCCCTTCCTTCTTCATCTATTTCATAAAAGTCCGC

TTTTCCTTTATGCTCTACAGGCTTGCTCCTGTCACCCTTCTGCCGCT

>7653 len=167 tot-seq=1

AGGTGGTGGGGGGGATGACGGAAGCAACCGGGTATGCGGCCTTGCATCTTAGCCCTGCCC

CTAACTCCCATGTGACCTTGAGCACTCCCCTCCCCATCTGCGGGCAGGCAAGGGGCTCGG

GATGCCGCTATAGGGCTCAACCCCACTGGGCTAGAAACAACCAAGGC

>7654 len=167 tot-seq=1

AATTGGTACATGGCTTAATACTATCTACTACTATGACTCTCCTTGCTTTAATTGGCTTCC

AGGAAAATTTCATGAGCCTTTGTGGTTGTGGTTTAGTTAAATACACACATTTACCTAGAG

GCAGGCTGGGGGAGGAGAGCAGAGTGCTTTCACTGACTATCCAACTC

>7655 len=167 tot-seq=1

TGAGGAATATTGGTCAATGGACGAGAGTCTGAACCAGCCAAGTAGCGTGCAGGATGACGG

CCCTATGGGTTGTAAACCGCTTTTGATTGGGAGCAAGCCTTCGGGTGAGTGTACCTTTCG

AATAAGCGCCGGCTAACTACGTGCCAGCAGCCGCGTAATACTTAGAT

>7656 len=167 tot-seq=1

CATTTCTCAAAAGAAGACATACAAACAGCCAACAAATCTATTTTTAAAAGCTCAACATCA

CTAATTATCAGAGAAAATGCAAATTGAAACCACAATGAGATACCATCTCATACCAGTCAG

AATGGTCATTACTACAAAGTTAAAAAAATAAAAATAAAATAAAATAA

>7657 len=167 tot-seq=1

CAAAAACAGGCTCAAGAAGCCGAGGGCGATAGCGGTGAGCAGCCAGCGTACCCAGGCGGC

CTCGGTGATGGCGGCGTTTTTCTGGATCAGCGGCCCGCGGCCAAACTGTTGCGGAATGGG

CGGCTCGCCACCATGGTCGGGCACGGCGTGGCCAATGTCTGGGGAGG

>7658 len=168 tot-seq=1

CCAGGTGCTGCATGTCCCGCCAGGTGAGGTTCTTGCTGCAAAGGAGAGAACGTCTGGGGT

CAGGCTTTCTGGCATGTGGCTGCAGAAAACAGCTGCCTGACAGGGAGCATGGGTGGGACA

GGCTGTTGCTTGCCCACTTACTTGGCCTCCAGGGTGAGAGCAATGATA

>7659 len=168 tot-seq=1

GATCCAGCATATGAAGCCAATGTCAAAAATAGTGAGACCTTAAAATACAGTAGGGCGGGT

GTGTGTGTGTAGGGGGGTGACCCAGCTGATAAAGCTCTGCTGTACAAAAAGGGCTGTGTT

TGACCTCTGCACTCGCATTCAAAGCCAGGTAAGGGTACACACTCACAA

>7660 len=168 tot-seq=1

AGGGGAACAAGTGTCTACAGGGATAAGACTTGGTCTCATGAGATATTTGCGGTTGTTGTA

TAATAATAAAACCTTTACCTGTCTTAAATCTAAGTCACTTTGCTTCAGTAGATGACCTGC

CTTCTGAGTTCCTCTGAGGTGAGAAACTGTTTAGCACCTCACCCTAAG

>7661 len=168 tot-seq=1

GAGTGCACCCAGGAAGACATGGAAGGTGGTGGAAAGGTTGTGTTAATCTGCATGGGCTTT

CCACTGGGGGGTCATTGTGCAAATGGAAGTTACTGTTCATCAGCCTTCAAAGGTGTGGCT

TTTTGCCAGATACAACCTTTTGTTCGTTCTCAGACTCTCCCAACTGTA

>7662 len=168 tot-seq=1

CAGACAGATATGGTTAAGGGAGTTGTGCCCTCTGACCTTTCAACCCTTTGATCCTCTCAC

AGCAGGTGGCCTGAAGCCCTTCCTGGGCATCTCCCTCTCTTCCGACACTGTTGATCCTTG

CCGTGCAATAAGCCTGACCCTATACAGGTGACTCACGTGCGCTGATCA

>7663 len=168 tot-seq=1

AAAGGACAGTCAACCCTAAGGAAGGCCAGTGGTGGGTTCCACAAACCAGTGGAAATCCCA

GTGCTCTCGAGTCTGCCCTTCCAGACTTTCTTTCCAGTGTGTGTGAACTCTTTGCCTGGA

GGGTGGGAAGGGGCTAGCCCTCTAGGAGGCCCTAGGACCAGCCTAACC

>7664 len=168 tot-seq=1

GCTCCAAAATACCTGTATCCAATTTGTATTATTCCCCCAGCTCTCAGGGACAAGACCAGT

CCCCCAGCGTGGTGGTCAGCACGGAAGCTCCACCTTCTGGGTGGAGGTGCCATCCTAACC

ATCCAGCCAGGCCACCCACAACCCGAGAATCAGGGAGAAAGTCCCTCC

>7665 len=168 tot-seq=1

TCTCTTGAGCCAGACCCCAGACCCTGGCTCTGCCTCTTACCAGGTGTTTGAGCTGACACA

AATTATTTAACCTCCTTGTGCCTCAGTTCCTCATCTGTAAAACCAGAAAACAGGAATACT

TACCTTCATGGGGCGGCTATGAGTACTCACTGAGCAAATTCATGACAC

>7666 len=168 tot-seq=1

AGTATGTTTCATACGTGTTGAACTGTGTAAAGTTACCTGAGAGAGTGGGGTTAACCTCAA

AAGAAATAGAAAACATGGCCTGTTCCATGGATGACTTCAGTCTTATTGGGAGATCTAACA

TTCATTGCTGAAACCAAGAGCAGTGATTGTCCAAGTATGGTCCCTGGA

>7667 len=168 tot-seq=1

CCACTGTCTCCCATCTCCCATGGCCTCCTTGGCCCCCAACTCAAGCCTTGAACTCCACTG

CCCCAGCCCCTGGGCTGAGGCATGAACACCCCCTCTGGGTCCAGTCAGAGCCCCCGGTGC

CAGGGATGGGGAGGCATAGGGCTCTTGGCCGCCAGCCGCGTGAAGGTG

>7668 len=168 tot-seq=1

TGTGGGGAGGGCATGGGCAGGCTGGTGCATGTCAGTGGGGGTCACTCTGCTGCAGCACTC

TGATAATCAGGTGTGGTCCACTAATGCAGGAGCTATGATGCAGGCCCTCAGAGGTACCTG

GGGGTTGCACTGCAAGTAGGTGTGGTCAGACTGGGGCCCCAGGAGAAG

>7669 len=168 tot-seq=1

GTGAACGGAAAAAACCGTCTTTACGATAAATTACGGCAACATAAGGTTCTTTTTGGCCGT

GGGCCAATTTACTGATCAGTTTCGGGGCGGCTTCAGCTTTAATGCCCGATTTTTCAGCTA

ATTTACAAAATTTGTCGATTTCTCTGAAGGCCGGATCATAGTAAGCGG

>7670 len=168 tot-seq=1

TCACTATAAATGTCCCTGAAATGCAGCTGATGGGAGCTGTACCCACCAGGCCCTTCGTTG

CAGACAATAAAAGCCACCTCTATACCTGATAGAGGAAAGGAGAGTGTTGGAAAGAGGTCA

GAAGAACTAATGAGTTTCCCAGAGTATGTGGGAGAGGTCAGTAGAGAC

>7671 len=169 tot-seq=1

TTAGGATGCTAAGACAAGAGCTGAGTTCCACAAATGAGTTCCAGGGTAGAGCCCCTGCCT

CTGTGGTTAAGAACTATGAAAAGCACCAGTTGAGCTTCTTCTGGTGCTGTGTCACAAACA

TCCTGAACTCACTGAGTCACAGTGGACAAGACAAACAGCCAAATGACTT

>7672 len=169 tot-seq=1

CACGCACATACATGTACACACACAGGCATACACACCATCATATGGACTGAGTAGAAAAGG

GAGAGATAATGTAATTATATTATAATTTCAAAAACAAAAAGTAACTTTTAAAAAGTTGAT

TAAAAGAAAGGAAGGTACATTCTGAATAAGAGAAAGAAATAGCTCAGCC

>7673 len=169 tot-seq=1

TTGGCCATCAGTAGCTGCCTCATGAGAGTTTAGGACTTCGTGGTCCCAGGACTGGCTTAG

TGTATGGGATAGGTGTACTCTGTGGTATTCCTATGTTGCCCAGAGCACTCATGGGCCAGT

TGTTATGCTTGCAAGTCCACATATAGAGAATGACAACAAAAGTCCTGGC

>7674 len=169 tot-seq=1

CCTTCCTCTCCAGGCTCAACCTCCTCAACACCTCAGAACTGAGGCCTTAGGTCTGCTCCT

CCCTGCGCCCACTTCAGCTAGGGCCCGTGTATCACTCAGAACCAAAAGAAACCCGTCAGC

GCCCACAGGCGCCATCCTTGTTCCATCCAAACAGGGCCAAAGTCAACAC

>7675 len=169 tot-seq=1

TCTGCTGTGCTTTTATCAAAGAAAATGTCTATCACAGCAGCAACCTCTTTTAGAAGTGTG

TGTGTGTGTCCCCGTTTTCTTTATTCCTTCGCCTCTCTCCCATTAATTCATTAATTTTGT

AAGTCTCTACTAGAGTCTTAAATTCTCCCAAACAATAGGGTAGACAACC

>7676 len=169 tot-seq=1

CTTGTCCTACTTCACAGGTGCGCAAATGGCAGTTGGGTTGGTGAGTGGGGGTGCCCACTG

GGGGGCGAAGTGACCTTCTTCAGGACATAGAAGGACCAGGCCTCTAGCCCAAGTAGACTC

CGAAGCCTGTGCTCTTGACCTCTGTCTTACACCGCCCACATGGCATGTC

>7677 len=169 tot-seq=1

GGGAGTATAGTATAGCATCTCTGCACCTAGCTGTGTCTGAGGCCACCGCTGGCCTTGGGT

AACAGCGACGATCCTGCCCCTGGAGCTGGACCACGGGAGGGTCCGCGCCCTTCTCCCATG

CAGTGAAGTCAGCCAGCAGTGGCCAGAACAGGCTCCAGCCCTCCGCCCA

>7678 len=169 tot-seq=1

TCCTCGATTTCAACTTCTCAGAGAAACTGTGTTGGCACCACCTATTAGGGTCATCTCTGC

TCATGCTCCTTCTGAGTCGCCTCATGCTGAACAGCTCAGGAATAAAGGAAAGAACAAAAT

ATCAACTGAGCAAAATGGTGAGAGACCTCCCTGGCAAAGATCAGGCCCC

>7679 len=169 tot-seq=1

AGGATGGATTTGAGCCTGGCTGTGTGCGGTCAGGGCACCTCCCATACGCGAGGGGGTGGA

TCAGTCTCCTGGGGTTGCTGTGACAAATGACCATGAACTCAGTGGCTGAAAACAAGACAC

ACATTCGCTCACAAGCAGTCTCGTATCAAGGTGTCAGAAGGGCCACGCT

>7680 len=169 tot-seq=1

AAAGGGGATCCTGCCACAAAAAAAGGCTCCACCTGGGAAGTGTTTTCCTTGGAGAGAGGG

GCAGTCCTCAGCACTGGTGCCTGTCTCCAGGATATCTGTTTGTATTCCCTCTACCTCCAT

CAGAGGCATCATGAGGCTCTACTGAAGAGCTCCACCCCTTCAGGTAGCA

>7681 len=169 tot-seq=1

TAGTCTGGAATCCTTTGTGAGGGACAAATAAACAGAACCCAGTAGCAGTGTTCTAAAGTC

CCTTGTGAGGGAAAAATATTCAGACCTTCGTAGCAGTGTTCTGGAATCCTATGTGAGGGA

AAAACATTTAGACCCTCGTAGCAGAGTCCTGGAATCCTATATGAGGGAC

>7682 len=169 tot-seq=1

GTATCGCTCCTCCAGACACATCGGTGGAGTTGGCAGAACGGTCTGTACCGTAGATAGGCA

CACCATCGATGACATAAAGGGGGTTGTTGTCTCCGATGATGGAGCTTAAACCACGAATGG

TCAAGACGTTAGCAGAGCCCAAAGCACCCGATGATGTGGTCATGAAAAC

>7683 len=169 tot-seq=1

GACTAGGGAAGCCTTCCAGGAAAGGTGATGCCTGAGCTGAGTTGTTTTTCTTTTGAGTTT

GGAACATTAATTTAATCAGAGGAAAACAAGGGACATGAAAAGGGCAAGGAAATAAGAAAA

TGAGCTGAGTTTTGAAGGCAATGATCAGATGAAGTGAGAGAAGAATATT

>7684 len=169 tot-seq=1

CTGCAATTTCACCCTTTCTCCCCTTGTCTGCACAGAGTAGTTGAGGAAGACAGTTGTCCC

ATTTACCTTTAACACCTGCTCAGCACCTGCTTCTCCCTTCCAACCTCCCCCACACCAGAG

AAAGGCCCCCAGCACTGACCTAGGAGCCATGAGCCAACACCTGCACCGG

>7685 len=169 tot-seq=1

TGGGGGATATTGGACAATGGGGGAAACCCTGATCCAGCGATGCCGCGTGAGGGAAGAAGG

TTTTCGGATTGTAAACCTCTGTGGACAGGGACGATAATGACGGTACCTGTTTAGAAAGCC

ACGGCTAACTACGTGCCAGCAGCCGCGTAATCTCGAGATCGGAAGTGGG

>7686 len=169 tot-seq=1

GATGTAGTGGTTAAAAGCATGGGCTGTAGAATCTGACCATCTGGGTTTAAACCCAACTTT

TATCAACTCTGCAAGGTGGGGAAAATAACCTACCGTCTCTAGGGCTGTCGTGATAATTGA

GTGAATAAAGATTCACCCAGGTGGTGGCTGCCCTGTCGTAAGCACTGTG

>7687 len=170 tot-seq=1

TCATCTGGGAAGAACAGGACTGGACTGCCAGAGGAGGCTGGATTTGCGGTGGTGGTATCA

CATGCTAACTGGGAGAACGTTGGGTTACCTTAAATTACCATCCATGCATGCCTCTTCTCT

GTGTCCTGGGCCTTATAGTTCAGAGTGCCCCAAACAAGTGTTCAGCAAAC

>7688 len=170 tot-seq=1

TGTGGCCAGGAGGAGGGGGTGATGAGAAGGCTCCCCTCCTACCCTGGGCCAACTTTCTCA

GGGTCCAGGAAGCCCGAGGGAATTCTGAACTTTCCCAGCAGCCCGCGGTAATAGCTAGAT

CGGAGAGCGTCGTGTAGGGAAAGAGTGTGCCAATGTGTAGATCTCGGTGG

>7689 len=170 tot-seq=1

AGCAACTGTTTGTTTTCCATTTCAACCTGGCAATTGATTGATTCAGAGAGGTCTTTTTAT

AAACAGGCAGTGGTGGGCTAACTATCTTTTCATTGTTTGTTTCTTATTCTAGCTACCCGA

CCACTGGGAAAAAGCTGTCATCACAGTGCAGCGACCTGGAAGGATTCCAA

>7690 len=170 tot-seq=1

CGGAGCTGCTGGGAGTGGGAGTGACTCCCCCACCTCGGGCCCCCACCCTGTCCCTGTCCT

CTTCCCGCTTGCCCTGAGTTTAGAAGAGCAGCCGCTGCCACCACTGCCACTCGGGAGGGC

ACCAGGGCTGCTGGCTAGGGAGGGACAGGGCAGGGAGGCTCTGGCCAGTC

>7691 len=170 tot-seq=1

GTGGAGTTGGGAGATGTCATCACTCAGGATTTGGGAAGGGTAAGTGCATAGAGTGAGAAG

AGAGAGAGGTTGAGGAGTGGATACTGGGAAAAGGGCATTTGAGGTTGGGCAAAGGAAGAG

GATGGAGAGAGAGGAAGAGAGTGGTGAAACTGAGGTGGGGTTGTCATGTC

>7692 len=170 tot-seq=1

CCTATGAACTCATTTTGACCTGTGTTAAAGCCAACTCAACATGGCCTGAGAATGACTTCG

TACTTCTTGAGACCTTGTGTATGAACTGCAACCTAGCTTAATAGGTAGGCAAGGCTGAAA

ACTTAACTTAGGAGTGTGTGTCTGTAACAATAGCTGAGTCTTGGCCAATC

>7693 len=170 tot-seq=1

TAGAGGTAGCAGGAGTGTTTCAGGTACGGACGCTCGTTGAAGTGTCGTTCAAATGAACGG

CTCAAGAAACTCATGTCGTCTTTCTGTAACTCCGGCTTGTAGCGTTCCTTGATGAACCAG

TCCTGCTTATGCACGACGCAGAAGTCCGGCAGTACCTTGGTAGCCTTGCA

>7694 len=170 tot-seq=1

GCAGAGCCGCTGGTTATTGAAACACCAGAGCCAGCTACAGAGGGCAGCATAAGAGTATAT

GATTATGACGGCTGCTGTATTTATGCGTATTACGGAGAAATCAAGATAAACAGCGACGGC

AGGGACGGAAAGCAGATTGATATTGAATGTGCGGGGTATTTAGAGGGATA

>7695 len=170 tot-seq=1

AACGGGAGGCAGCAGTGAGGAATATTGGTCAATGGACGAGAGTCCGAACCAGCCAAGTAG

CGTGCAGGATGACGGCCCTATGGGTTGTAAACTTCTTTTGTTTGGGAATAATCTACAGTA

CGTATAATGTTTTGCATGTACCATTCGAATAAGGACCGGCTAATTCCGTG

>7696 len=171 tot-seq=1

AACTTAGGAAATTAGCCTGAACAGGTGAGAGGGTGCGCCAGAGAACCGGACAGCTTCTGG

GACAGGCGGAAGCACAGAGCTGCTGGGGCAGCACCCTTGGCGGGCCGCAGACAGCCGGCC

ACCGTCCGGACCAGAGGACAGGTGTCCTCCTGGCTTGGGAGGCAGCCTCAG

>7697 len=171 tot-seq=1

CTTAGGAAATTAGTCGGAACAGGTTAGAGGGTGCACCAGAGAACCGGACAGCTTCTGGGA

CATGCGGAAGCACAGAGCCACTGAGGCAGCAGCCTGGGCGGGCCACAGACAACCGGCCAC

CATCCGGACCAGAGGACAGGTGTCCACCTGGCTTGGGAGAAGGCCTCAGCC

>7698 len=171 tot-seq=1

CTTTCTCCAGTCAAGCTCTTTAGGCCCACCTCCTGCCTCGCGGTGGCCTGCACAGACCCA

GCTCTGGCTTGAGAAGAGCCGCTGCAGGCCCCACTCCTGCCTCCCAGGGGACTGTCCAGG

CCCAGCTCTTGCCTCACGGCGCCTTCCCAGGGCCCAGTTCCTGCCTGCCTC

>7699 len=171 tot-seq=1

GGAAGCCTGGAGGCAGGCGCAGAGCCTCTGGGCCTGGGTGCCTACCCCTGCCCACCCCCA

GGGCCTCAGGCCACAGGAGACCACCTAAAGCAGGAGCCGGGAGTAGGGCCCAGGCTGGGC

TGGCACAGCTGGGCCTGTGAGGGGTGGAGGGGCCGCTGGGGAAGGGACTTA

>7700 len=171 tot-seq=1

AGGGGAGAGGCTGGCTGGGCACTTGGCTCAGGAGGCTGGAGATCTGGATCCCTTCCAGCT

CCTCCTCCTGGCTGTGCAACTTGGCCTTGTTACTGAACGTCTCTGAGCCACATCTGTAGA

GTGGAGACGGCATTGGACCCATATCACTGTGTCATGTGGGGCTGGTAAGAT

>7701 len=171 tot-seq=1

GGAGGCAGCAGTAGGGAATCTTCCACAATGGACGCAAGTCTGATGGAGCAACGCCGCGTG

AGTGAAGAAGGGTTTCGGATCGTAAAGCTCTGTTGTTGGAGAAGAACACGAGTAAGAGTA

ACTGTTCATTCGATGACGGTAGCTAACCAGAAAGTCACGGCTAACTACGTG

>7702 len=171 tot-seq=1

TGTCATGATGGCCGAGTCTATGTCTCCAGTCTGGTTCTAAAAGCCATATTTTAGGGTCAT

CTATGGTTTTTGGTTGCCAGTGTTTCTCCTCTTCTCCTTTTCAAGTTTTGAGTTCTGGAA

TATGAAATCAACTTTCAAAATATAACAGTATCAATACTGCAATTATTACTG

>7703 len=171 tot-seq=1

GACGCACATCCTGAGGCCTGGTGGGAGGCCGGGCCGCCCGTGCTCACCAGCTCGTTGGCG

TGCTTGGACCAGGCCAGATTGCACACTTGGGAGCCCGTGTCGATACACTGCAGTGGTTGT

CCTGTCAGCGTGTTCCAGAAGCGGATACAGCGGTCAGCTGTGCCGCCCCCC

>7704 len=171 tot-seq=1

TGGCAGTGTTGGTGGGGAACAGACTGCCAGCATCCATGAGCACATTTCAGCCAGTGATAG

TGTTGGTGTAGGAGAGCAGGGCTGCTAGCACCCGTGTGTGCATTTACACTATTGGTGGTG

TCATTGCAAAGGCAGTGGACAGGGACACTGGTGTCTGTGCACTCATTTACA

>7705 len=171 tot-seq=1

GCCATCCTCTAATTACATGTGTGGATCCTCTGGCAACATACCCCCATCCTCCAGGAGTCA

CCTCATGAACATAAACTCAGGTATAGTGGAAAAGGGCTTGCTGTGAATAACAAAAGATGC

TCCTTTTCCACCAATCATTCAGAAAACTCCAAAGGTTTTAGAAGCACCGTG

>7706 len=171 tot-seq=1

AGGGCTGATCTAGGACCCCGGGTACCTGAGGCCTTGTTTCGAGGCGAGAGGGCTGGGCTG

GCTGGAGCCTGGCCCAAGCTAGGAAGAGGGCTGCCTTGGCATGACCTCTTCTCTCCTCCT

CGACAGGCCTGTCCTTCGCAGCTTACCATCAGTCACTCCCTGTTGGAGCCT

>7707 len=171 tot-seq=1

ACCACACAGACCTCAGCTCAAATCCAGGCTCCATCACTGTGTGACTTTAGAAAAATGACC

ACCCTCTCTGGGACTCAGTTTTCCCACATGGAAGATGAGGATACCAATTTCACATAATTT

ATTGGTAAGCTGTAAAGTGCAGTGCACTTAAGGAGGCCCTACCCTATCCCC

>7708 len=172 tot-seq=1

GATCCCATCTTTTACTACAAGGGTGGAGCAGATGGCGAGGGTCGGGAGTTAGGTGAGGCT

GTAAGCCTTCTAAGCCTACCCCACTGATACACTTTTCCAGCACGGCTGTACCTCCTAAAC

CTGCCCCCAAAGTACCACCAACTGGGCATCAAATGTTCTAATGTCCAAATGT

>7709 len=172 tot-seq=1

GGGAAGAGTGAAACTGACAACATAGCAGGGGCCTATAGTTGTGTGGGAGGCACCTTCTAG

AACTAGGGTCCTGCCTGGGGGTTTGGGGACCTGGAATCATTTGAGGTAATGGCTGTCGGC

AACCTGGTCTGCCCAATTCCACCTGCTTCTTATATATGCAGTCCAAATGTTG

>7710 len=172 tot-seq=1

TGCTCTTTCTGCTCCCATGTGGTATTGATGTCATTGGTACGATTTTGCTTGGTTCAGTCA

AGTGAAGTATTTGTGTGTGCATCTGTCACATCAAATCTGGATTCAAACTCCAACATGGGG

CCTCTCTATTTCTGCAACAATGTCTTCATTTTCACACAATGGCCCAAAAAGC

>7711 len=172 tot-seq=1

CTACATGGCTTGGAGAGAGAATCTGTTCACCTCGGGAAGAAAGAGCACTGTGGGACTTCG

CAGTGAACTCAGTGCTGCCCTGTCATGACAGAAAGCAACACTTGGCAGAAATCAGCTAAC

ACCCACGTAGGGAACATTTAGATCAGCCCTAGCCAGAGAGGAATTGCCCATC

>7712 len=172 tot-seq=1

CAGACTCCCAGGCTGAAGGCTCCCCTCAGAAGAAGCCAGCTTCTCGGAAGCAAACAGTGA

AGGTTTCTCCTGACAAGTTTCCCAAGGGCTCCAATGACCCAAGGGGATGCCGCCCCAGGA

CGACCTGTCCCTGCACCAAGCACAGCATCCTGGGCTGCTTACCCATGGGAAA

>7713 len=172 tot-seq=1

AGCGAGACCTTGCACATTCCGGGAAACCAGACCCCCATTCTAATCGCTGCTGCTGGTACT

GCGCATTCCAGGGCGCCGGGGACATGGGCACAGAGGGGCCTGTGCGACTGGATTTCCTCG

AATTCTGCAAAGAAAATGCACAGGAAGGACAATGGCATTGCTAAGTGGTGGC

>7714 len=172 tot-seq=1

GCCGACGCTTCTCAAGTCCTGCTGCCTGTCGGGGACACCTGCACACGCCCCTCAAAGCCA

CACTCAATTGTGCCTGTGTGGGAAGCTTCTCCAAAGCACCCTCAGCAGGAACATTCCACC

CTGCAGGCAAGCGCCTCTGCTCCTCAGCTTCACATGTTCGTGGAGGAACCTA

>7715 len=172 tot-seq=1

TTTTTTTTTCAAGCAGAAGACGGCATACGAGATCGACAAGTGACTGGAGTTCGGACGTGT

GCTCTTCCGATCTCCTACGGAGGCAGCAGACATTTGCATAAGTAACAAGGAGTCAAATGG

TAATCCCCAAAACAATGGGGAAAATGTCTTCAGGACATGTCAGAAACATTTG

>7716 len=173 tot-seq=1

AACTTAGGAAATTAGTCTGAACAGGTTAGAGGGTGCGCCAGGGAAACGGACAGCTTCTGG

GACGGGCGGAAGCACAGAGCCGCTGAGGCAGTACCCTTTGCAGGCTGCAGACAGCCGGCC

ACCGTCCAGACCAGAGGACAGGTGTCCGCCTGGCTTGGGAGGCAGCCTCAGCC

>7717 len=173 tot-seq=1

CCCCATCAAGGTGGAGACAAATGACACATCTCAGTGTCCTTTTAAAGTCCAGCTTAAGTG

AAGACTTAGATCAATAGTTCTGCCTTGAGAGCCAAGCCTGCATGGTGCTCTGTCTCACCC

CTGCCCCTCTCCTTTCAAGACCATATCTCCCTCCAAGTTCAAATTCTTGCCAG

>7718 len=173 tot-seq=1

AACTTAGGAAATTAGTCTGAACAGGTGAGAGGGTGCGCCAGAGAACCGGACAGCTTCTGG

GACAGGTGGAAGCACAGAGCCGCTGAGGCAGCACCCTTGGTGGGCCACAGACAGCCGGCC

ACCATCCTGACCAGAGGACAGGTGTCCGCCTGGCTTGGAAGGCAGCCTCAGCC

>7719 len=173 tot-seq=1

GTGTGCGGGCCGGTTTCCTTCCCTTCCCTCTTTTAAGCCATTTTTTGTTGATGTACCCAG

GTTTTGTAAAACTGGTGGCAGAGTATTACGTAAAATGTAAAATTCTCCTTTCAATTGTGT

TTGGGTGCACAATTCCGGGATACCTTCCTTTTTCTTGCAAAAGTTAAAATACA

>7720 len=173 tot-seq=1

AACTTAGGAAATTAGTCTGAACAGGTTAGAGGGTGCGCCAGAGAACCGGACAGCTTCTGG

GACAGGCAGAAGCACAGAGCTGCTGAGGCAGCAGCCTGGGCGGGCCGCAGATAACCGGCC

ACCATCCGGACCAGAGGACAGGTGTCCGCCTGGCTCGGGAGGCGGCCTCAGCC

>7721 len=173 tot-seq=1

GATCCATCAATGACTTCTTTGCAGCCCTAGCCAAACAATCAAAAGCAAATAGTCAGCTTC

AAAAATCAATGTTTCGTGAGATGATGAGCCAACCAGTGAGTTCTCGTGAGGTGGGATGGA

AAGGAGAACTAAGAGGGAAAACAGAAACATTTTTTTCATACTACCCACCAAAG

>7722 len=173 tot-seq=1

TTGTTGACGAAGACAGAATTTTAACCATGGAACTGTTTATATTACTTACTTTCAAGTCCC

TGTAACAGAGGCAAGTTTTTTGTTTGTTTTTGTTTTTGTTTATTTTGCTTCCAGGAGTCA

GAGGGCTCAGCCCATTGCAGTGTGTGAAACAGACTGAGTAAAGAGATCACAAC

>7723 len=173 tot-seq=1

TGGGGAATATTGCACAATGGGGGAAACCCTGATGCAGCGACGCCGCGTGGAGGAAGAAGG

TCTTCGGGTTGTAAACTCCTGTTGTTGAGGAAGATAATGACGGTACTCAACAAGGAAGTG

ACGGCTAACTACGTGCCAGCAGCCGCGGTTCCTGAGATCGGAAGAGCGTCGTG

>7724 len=173 tot-seq=1

TCCTTGGAAATATATTTAGAATCTGACATCTTCTCACCATCTCACCTGACTTCCCTAGAC

CAGTCCACGAATCTATCTCACATGCACTATTGCAATAAACTGCCAACCAACCAGCTTTCC

TGCTTTTACTGTCTCCCCTGTCCCCACTCCCAATCCATCCTCCATTAAGCAGC

>7725 len=173 tot-seq=1

AAACTATTCCAATCAATAGAAAAAGAGGGAATCCTGCCTAACTCATTTTATGAGGCCAGC

ATCATCCTGATACCAAAGCCTGGCAGAGACACAACAAAAAAAGAGAATTTTAGACCTATA

TCCCTGATGTACATTGACACAAAAATCCTCAATAAAATACTGGCAAACCGAAC

>7726 len=173 tot-seq=1

CACGATCAGCTGGCGGCAAAGGGAGGTAATGCTGGAGTATACGCCGTTGCCCAGGGCCTG

GAAGCTGGCGCCCAGGCCAATTCCCACCGCTGCCAGGGGGAAGTGGAGCGCCACGATCCG

CAGGGCGGAAATGCCCATTTTGCGCATCTCCTCGCCGGCCTCAAACAGGTCCA

>7727 len=173 tot-seq=1

CCTGTCAAGAGCACCAGAGGAGACCCCCCAAGACAAGGCCATGTGGGTGGCGGGGTGAGC

CCCAGAGTAGCCGGGCCAGGCAGCTCCTTTCTCTCTAAGAGGCCATCTACAGAGTGGGAG

AGGACCCCCGCCATCCCTGCTGAGGGCATCAGGTCATCAGGGCCAGCTAGCCA

>7728 len=173 tot-seq=1

CGGGGACTCACAGCAAGTACAAACTTCTGGTCGATGTACTTCTTGGCTATGTTTGGCTGG

AAATCAGGAGACTCAAGGAAACGTAAGAAGAACTCATACACGAGCTATAGGGGAAAAGGC

ACGCCACTGAGGGGATGTTCCCAAGGACTTGCAGGCCTCCAAGTGCCCCACTC

>7729 len=173 tot-seq=1

CCAGGGGAGTTGATGTGGAAGATGCTCTAAGAAGCATCCCCAATTAGCAAGCCTCAGCCA

GAAACTACCTACAGGGAGCAGGAGGCCCTAGCGGGAGGCAGCCCACGCCTCATTATTCCT

GTAGCAGGAAGGGACGTCCAGACGGGACTCCAGAGGTGATGGAAAGTGGGGGA

>7730 len=173 tot-seq=1

TGCAGATGCGTCCCCAAAACTTTAGCTCATGCAATGTCTTACGCCCCAAGGAATGGGTAG

TGAAACACACTGGGGAGGCTGCCCTAGGGACAAAGGGAGCCAAAGCATGTACTCTCCAGA

GCCTGAGAGCTGCCTGCCTGGGGCTGTTGCCACTGACAGCAAATCCTTACCCC

>7731 len=173 tot-seq=1

CCGAAGGGAGGCAGCAGGGAGGAAGAGGGGGCAAGGGGCGCGAGCCGGAACCAGCCAAGG

CGCGGGAGGGAAGACGGGCCGATGGATTGTAAACCTCTTTTGCCGGGGAGCAAGCTGTGC

CACGTGTGGCGCAGCTGAGAGTACCCGGAGAAAAAGCATCGGCTAACTCCGTG

>7732 len=173 tot-seq=1

TTCCATCATCAGTTCCGCAACATATTGCGGGGCATGGTAGCTCCTCGCCTTCACTGCTTT

ATTCGTCACTGGATCATAATGATCATTATCACTGTGCGTAATCAGTACAGCATCTGGTGA

CGGTATCTCACCTGGAACTGCCGGAGGTTCTATCAGCAAGGGCATATCGAATC

>7733 len=173 tot-seq=1

CAGCGAGGGGTGCCTCCCGGATCTCATGGCCATCTGGGAATACCCAAGACAGACCCAGAA

CGTGCAGCTGATGACCAGTCCTCCAAGCTGGTGGTCTCACCGGAGCCACACACCTCCTTC

TCAGCCAGCAGCCGCGGTATGCGTAGATCGGAAGAGCGTCGTGTAGGGAAAGA

>7734 len=173 tot-seq=1

CAGGTGAGAACAGGGAGGAAATCTGGGGCTACTCCGTCACTTTTGGCCTGGGTGACTGGG

TGGACATTGATGCCAGGAACAGAGAAGAAATAAAGAAAATGTTAGAGGTGTTTGGAAGAA

AAGATAATCAATTGCACTACCACATGTTGGCTCTGAGGGAGTTGCAGAGTTCT

>7735 len=174 tot-seq=1

TCAGGTCACAGAGCTAGAGCACTGGTCAAGGAAACATGCTACCTCACTGCTTTTCTGACT

GTCCCACCCTAATCCTGCCCCACCAATCCCGGGATACCCAGACATGGCTGGGCCGGTTCC

TGCCATCTGGAGTCCTCCATTCCACACTCAAAGGTGCCTCGTCAACCGTGAGTA

>7736 len=174 tot-seq=1

GGCTATGTGTCCTCCTGGGATTGGTCAGTCTGCCGGCACTTTAAGGGGAGGGGTTGCACC

GGAGGGGTTGGCGCCAGATTTGGGCTAGCACCTGTGCAGTGGTGTTGTTTACGGTGACGA

CGGTGTACCAGAGACCGACGCCATCTTTTAGGAGCCAGCTGCCTACTTAGGTCT

>7737 len=174 tot-seq=1

AGTAATCTCCTCTCTGGACCAAGTCAATTAAAGAGATGTTGACTCTCATACATGGGCTTA

GGGCCATGAGTACAATCATGGGTTCATACCAGCACGGAGGTCTCAGAGCAAATTCTGACT

CTTGTGAATAGCATATAAAGCCCTTGAATAGTAGAGAGACTGTCCTCATAGGGC

>7738 len=174 tot-seq=1

CACCAGCTCCCCTTCCGCCATAAGTGAAAGCCACCTGAGGCCCTCACCAGAAGCAGAAGC

TGGCACCACCACACTTCTTGTACAGTCTGCAGAACCATGAGCCAATTAACCCTTTCTTTA

TAAATTACCCAGCCTCAGGCATTTCTTTATAGCAACACAGACTAAGACACCATC

>7739 len=174 tot-seq=1

GAGAGCAGCTACATCTGTAGCTTCTCCTGGGTGGTAAGACTTGCAGCCAGGTCCAGCTTG

GTGACCTGGAACTGCTCTGCGTGTGCCCCAGCCTGCTGTCCTGATATTATGGTGCAGTGG

GACCCTCTCTGCTCCACCCCCAGGCAGAAATCCAGGCATGTGTAGGACCCTGGA

>7740 len=174 tot-seq=1

GGAGTAGAGAACTTAGAGGGGAAACTAGCCCTCAGGCTCCCCCTGAGAAGACCGTCCACT

TGAGAAAAGCAACCTTGGAGGAGCCTTTGTGCACATAGAACCTACTGGATTAAGCAGCTG

GGAGACAGCCAAGGAGTAGGGGACAAAGACGGCTACATTGTTAGCACTGAATGT

>7741 len=174 tot-seq=1

AGCAGGAGGGAGAAGATGAGATGACATGAGATGGGGGAGGAAGAAGGGGCCAGGTCATGG

AAATGAGGAAAATGTGTATGCCAAGTGTGAAGGGACAGTGCTCAGAATTCCAAGCCCTAA

CTCTCTCAGCCCCATGTCCTTTCCACTTCATATCACTGTGGCTTAAACCCCCAC

>7742 len=174 tot-seq=1

TTGCTGCTTAGGATGGGGGCAAGTCTGCTTTCCTCTGTGTCTACTTTCACATGTGTAGTA

GTTTTGGTGCAGGAGTGGGTTGCTGGAGGGTGTGGGGCTGACAGACTCTGTGCCTGCCAA

AGGTCCAACAACAATGGTGGTGCAGCAGGGGAAGGCAAGGAAGGTGCACTTATG

>7743 len=174 tot-seq=1

CCCCCATGATTTAATTACCTCCCACTGGGTTCCTCCCATGGGAATTGTAGGAGTTACAAT

TCAAGATGAAATTTGGGTGAGGACACAGCCAAACCATATCACCATGCCTTGGGCCACTGC

TCACACCCCCAGGCCCTCCTTTCTTCCTCTGACCCTCAGCCTTCCTACCCCTCC

>7744 len=174 tot-seq=1

GGGGGGCCGGAGGGGTAGGTGGGCGGCACAGCTGGGGACTGAGGGTGCTGGTTGCTGTGG

ACAGGCTTGGAGCCGTTTTTGGCTGGAGACTGCGGGTGGGAGAGAGCAGAGGGTCAGGAC

CCAGTGGGCCAGCTGGTCTCCCTCACCACCCCCACCTCAGGCTCCATCTTTGTC

>7745 len=175 tot-seq=1

CCTCTGGAAGAGCAGGCAGTGTCTTAACCGCTAAGCCATCTCTCCAGCCCCTTGTTCTTC

AACATCATGAAGGCTCGCAGGGCCTTCAAGGGACTGAATAATAGTTCTAAGACAGTCCCA

CAGCGGGACATCCTGGGTCCTACATGTGCAGGGGTAGAAGGGTCAAGTATGACCT

>7746 len=175 tot-seq=1

AACCTTGAGAAATATACCTTTGGTTTGCCTCAGAAAAGTAACACATATTGCACTGTTAAG

GTTTATAAAATTGGCATAAAACAATGTTGTAACATCACGATACCCGTTTTAAGAGTTCAG

TAACTAACAGGAGCCCACAGGACATACACAGAACTGCGGATGCTATCACACTTAG

>7747 len=175 tot-seq=1

CCTCTTCTGCTGCTGAGACTTTTGCAACAGCTATCCTAGTCATGGGGTCCTTCCAGTCTG

CTCCTCCAACTATCTATTTTCAAGAAGGCAGATATTCATTTATTAAAGTATAAATCATGT

CAGATAATTACTCTCTTCAGAATGAGCATTACTCATCATGCCAGTCACTCTAAGT

>7748 len=175 tot-seq=1

AGGTGTGAGATGATGACTAGAAGACGGAGAAGATGGCTATGGCAATGACATTGCATAACT

GCTGTCGCTGCGGCATGCATCCTCCCATACCCCCTGTGTTTGGTGTCAGCTGGGGTGAGC

TCAACCCTGGACTTGGATGAATCAGTGCAGGGATTCTCAAAGTGTGATCCCTGGA

>7749 len=175 tot-seq=1

TGGGGAATATTGCACAATGGGCGCAAGCCTGATGCAGCAACACCGCGTGAACGATGAAGG

TCTTCGGATCGTAAAGTTCTGTTGCAGGGGAAGATAGTGACGGTACCCTGTGAGGAAGCC

CCGGCTAACTACGTGCTAGCAGTCGCGGTAATAACCAGATCGGAAGAGCGTCGTG

>7750 len=175 tot-seq=1

CAGCAGAGTGGCAGATGATGCAGCCAAGCCTTCCCTCATACCTGACCTGGTGGCTCCATC

CAGCACTTCCTTCCAGACCTTCCAGGGAAACTTCAGGAAGCTTCTTGGGTGCCCCACCTG

TACTTGCACTGCCCAACATCACCCCAGCTCATACATCTAGGCACTCCCTATGATC

>7751 len=175 tot-seq=1

CTGAGTTGTGAAACCGGGTAGCTTGGGAGGTGCAGAGGCTCAGAATCTGAGTTCAAATGA

GGCAGACTAGGTTCCATCCCTGGGGGTAGGCTCCAGGGCCCCACATGGAGATGAGGCACA

AGGACAAGCAGGGGCGAGGAGGTTCCTTGCAATGAGGAGAGTCCAGGGCTCACCC

>7752 len=175 tot-seq=1

GGAGGTATTTGCGGGGGAGGTCCAGCAGCTGTCTTTCCTAAGACAGGGACACATGGGCCT

GGTTATTCCTCTTGTCACATGTGGAACGGTAGGAGATGGAAGACGGAGACAGAACAAGCA

AAGGAGGGCCCTGGGCACAGAGGTCTGTGTGTGTAGCCATCCAAGCCACTGGACC

>7753 len=176 tot-seq=1

CAGGAGGCAGAAGCAGGCGGATTTCTGAGTTCGAGGCCAGCCTGGTCTACAGAGTGAGTT

CCAGGACAGCCAGAGCTATACAGAGAAACCCTGTCTTGAAAAACCAAAACCAAAACCAAC

CAACCAAACAACCAACCAAAAAGCATGGTGATCATCCCGGGACAGCTGCATTCCCA

>7754 len=176 tot-seq=1

GCCACCACAGGAGACCCTTCTGTGAGCTGGCCTGAGCTCAGGCACATGCTGACCAGTAAG

CTCAGTGTTTGAGCTGTTGGAGTCTGGGGTAGAGAAAGGGTGGCTCTGGGGTCCCTTCCT

CTGCCCCCTTTCACCTGGGTACACCCTGGGTCTTTTCCTCAAAAGCCCAGCCCAGC

>7755 len=176 tot-seq=1

CCACAGCATGGTGTTCATTCATGGTGCTGAGGCCAAAAGTACCACACAGTGAGGGCGTTT

GTTTGGGATGCCCTGCTACTGTCTTCCCCGTGTCTGATCAGAATAACAGTGGCTATTACA

CTTTCTGTTTCCTTAGCACAAAGGTCTATTGCAGATGAAACTATGTATACTAGGTG

>7756 len=176 tot-seq=1

ATAGTTCATTCTTATTTCATGGGTTAGGAATGCTGCCTGCTAACCTTGAACTATTTTCCC

TTCCTGAGATATGGCCCAACAAGCTGTAGCAGGCAGAACCACTGCTGATAATCACACAAT

CTTATAGAGGTTCTGCCTCCCTAGAAATCAGGTTTCACCTGCAGCCCAGGAATTCT

>7757 len=176 tot-seq=1

CACCCACGCACACATTCATCCAAGACCTACTTCCTACTATGGAGCACTGCGCTATGTTCT

GGGAACACAGAAATGACCAAGCTAGGTCATCAGCTCCCGCCTTCAGACCATTTGTCTGGA

GGGCATGAAACAGGTGGTACATGTAAGGGAGGATTGCAGCCGCTCACAAGCTGTAG

>7758 len=176 tot-seq=1

GGAGATGGGTGATCAAATCCACCAATCTTATCAATAAATGAGCTGAGGATGTAACTCAGA

GTTAGAGCACTTGCCTGGTGTGCTCCAGACCCTAACTTCAATCCTTGACACCCAAAACGA

AACCATAGAATTGGACAGAGATAATTTTTATGTCTGAGAGATCCTATAACAAAGTA

>7759 len=176 tot-seq=1

TCAAATATTATTATAATTAGCTTTTAGTAAATTATGTTTTGTGTATTTGTGTATGCATGT
AGAGGCCAGATATCAGTGTCAAGACCTTGGGTGGCTTCCTCAGTCCCTTGCAGCCTTATT
GCTTGAGACAGTCTCTCTTGGGTCCTGAGGCTCAGCAATTCATCTAGACCAGTTGG
>7760 len=176 tot-seq=1
AGGAAGCCTGAGTTGATGTGGGTAAAGGATGTTTGTAACAGAGGAAACCAAAAGTGCAAA
TGTCTTGCGTTGAGAGCATGATGGTTTTGATGAAAATATTTAATGGGTGTTTGATGAAAA
TGAAGGAGAACGATCAGTTTAGAAAAAAATAAAAGGTGAGATACATATCAAATCGC
>7761 len=176 tot-seq=1
TAGGGAATCTTCCGCAATGGACGAAAGTCTGACGGAGCAACGCCGCGTGAGTGATGAAGG
ATTTCGGTTCGTAAAGCTCTGTTGTTAGGGAAGAACAAGTGCCGTTCAAATAGGGCGGCA
CCTTGACGGTACCTAACCAGAAAGCCACGGCTAACTACGTGCCAGAAGCCGAGGAA
>7762 len=176 tot-seq=1
TAAGGGAGGAAGAAGTAGGGAATATTCGGCAATGGACGGAAGTCTGACCGAGCAACGCCG
CGTGAGTGAAGAAGGTTTTAGGATAGTAAAGATATGTTGTTAGAGAAGAAAGGGGGGAGG
AGTGGAAAATATAAAAAGTGACGGTAACTAACCAGAAAGGGACGGCTAACTACGTG
>7763 len=176 tot-seq=1
TAGTGAGGTGCAGAGAGCATTTCTGTGGTCTTGGGAGAGAGAGAGCCAGCAACTGTGAGA
CATTGAACTTAGTGCTGCCTTTATTATAGCAGAAAGCAAAACTAGACAAATTCAGTTGAT
GCCCACTCATGTAGGGAGCATTTAAACTAGCCCTAGCAAGAGGAAAGTTGCCCACC
>7764 len=176 tot-seq=1
GGGTTTCCTCAGCAACCCTGCATGGGGCCAGCACTCAAGCCACCAGGCCACCTTTGTGAG
ATCTGGGAAGAAATGCCCCTGGGAACAGAGGCAGCCTTGCCTGAGGGTACTGACCTGGTG
AGTGCAGAGTGAGCTGGATTTCAGCCCACCTGCCCTTCACCTGTGTGTGTGTCTGTCC
>7765 len=176 tot-seq=1
TGAGGGGGACAGCAGCCACGGGGATGGCTCTGAGTTCGACGATGAAGGGATGCAGAGCGA
TGACCCGGGCGCCTACAGGAGCGAGAGGGGCAACTCCCGAATAAGCGATGCCGGCCTCAA
GTCAAGAGAATCGAAAGGGGACGATGAGGAGCAGGCAAGCACCCAGGATTCCCATG
>7766 len=176 tot-seq=1
GCCCCGCGGCCTGGCGGCCCTTCTGCTGCAGGCTGCCCAGTTGCCCGAGTGCTGTGTCCA

CCTGGCCACTCCTCTGCCTGAGCTGTCACCCTTCCTCTCCCTAGGGTATGCACCACGTGG
TCCTTGACGGGCACCTGGAGGCCCCGAGCTGCCCCCTGGCTCCCAGGGACCCTGGC
>7767 len=176 tot-seq=1
CAGTGGGCAGCTGAGCCAATGCTAACAGCTGCCTTGTTATGGGAACTGAAGAAAATGAAA
CCACCCAATTGTTAATGTCACTGTTGGTCACACCTTTTGTTACGGTAAGCTGACAGCATT
TGTCACTGAAGCGGTAGGACTCCTAGCGCAGTGCTCTTCCCACTGTATAACTGTTC
>7768 len=176 tot-seq=1
GACGGGAGGCAGCAGGAGGGAAGCGGCGGCAAGGGGGGCAACCCGGACCGAGCAACGCCG
CGTGAGTGAAGAAGGTTTTCGGATCGTAAAGCTCTGTTGTAAGAGAAGAACGTGTGTGAG
AGTGGAAAGTTCACACAGTGACGGTAACTTACCAGAAAGGGACGGCTAACTACGTG
>7769 len=177 tot-seq=1
TGCTGACCTGTCAACATGCTGGAAAAGAGCATGGCTGGGAAGTAGTGGTGGAAGTAGAGG
ATCCGGCCCATCAAGAAGAAAGGGAAGTAATGGAGCATCCATCCCAGGAGCAGCTGGCCA
CCTCCTCGCAGCAGCACCTTGGTCAGCCCTGTGCAGCCCAATCAGCACAACCCAATC
>7770 len=177 tot-seq=1
CAAGGATGCTGTGCCCGAGTACTTCAACTCAAACCACACGGAGACGAGCTTTGACGGGCG
CTCTGATGACAAGGGCCCGGAACCCGAGGGGCTGACACTGGGGACGATCGGCGATAAGAC
GTACGCTTTCATTGGCTTGGAGCGCATCGGTGGCATCATGGTGTACGACATCACGAA
>7771 len=177 tot-seq=1
GCGCGCGCATAAAACTAGAAAAACTAGAAAAACTAGAACTCTGGCTCTTCGTCTGATCCA
CCAAAATCGGAGCTAGCTCCAAACGTTGCAAATCCGCCGTTTGATGGAGCAGAAGCACTC
CACGGGTCTTCAACGTTTACGCCCTGATTAGCGCCATTTTGCGCATTCATTCCAGCA
>7772 len=177 tot-seq=1
GGAAGACAAGAGGCCACCAACCTCAGGGAGAGGCTTATAGGGGGCAGGCTGCAGCATGAC
CTTGAGCAAGATACCCTCATCTATAAGCCCAACAAGGGTCTGGTTGCCAGGGACAGCACC
GTGAGGGAACCTCACGGCTGCAGCTCTGGGTCAGACGTGGCCTGGGGCCAGCTCTGA
>7773 len=177 tot-seq=1
AATGACCCTGATCTACTTTAGTTGTCACAGTAGTTAATGTCATTTCAAAATAAGGGAATA
TGTAGTAATAAGAGCTAACATTTATTGAGTGCTTACTATGTTCCAAGAATTGTGCCAAGC

ACTTACTATACTCATGTGCTCATTTGATTATCTCCATACCTTGTGAAGAATGATAAG

>7774 len=177 tot-seq=1

CAAATATTATGAAATCTAGACGGAGAGATAGAGACCAATATAATGATAGTTGAGCACTTC

AATATCCTACTCTCAGCATTGGACAGTTCATCTAGATATAAAATCAACAAAGAAACATTA

GATTTAAGCTGCACTTTAGACCAAATGGACCTAACAGTTATTTTCAGAATATTTCAT

>7775 len=177 tot-seq=1

CGGAATCCTTTGAAATGAGCTGAATAATAGCAATGTTTGTGGGGAAGGATCCTCATTTTA

CCCCAATTCCTATTACCACTGTTTCCTTTCCTATGCTTTTCTCTTCAGATTGCCTCTTTG

CTCTGGAATCTGTAACATCCTTTCTTTGTGAAATTATTTTTCTCTCTCTCTTTTTAG

>7776 len=177 tot-seq=1

GGGGCCTGGGCAGTGATGGCCACAGGGCTCCCTGCTCAGAGGGACCAGTGTGAGGGCCCC

AAGGAGGTGAAGAGACTCCCAAACAGGTGGCCCCAGACCCCACAGGGCCCAGCACTGAGC

TGTGTTGGGTGGGGCGGCGGCCACCCCACATTGCCTTGCTCACAGCTCCACGCGGTG

>7777 len=177 tot-seq=1

CTTCGTAGACTACCGTCGGCGCGGTGGTGATCAGGTCGAGATCGTATTCACGCTCCAGAC

GCTCCTGAATGATCTCCATGTGCAGCAGGCCGAGGAAGCCGCAGCGGAAGCCGAAGCCCA

GCGCGGTAGAGCTTTCCGGCTCGTAGAACAGAGAGGCATCGTTAAGGCTCAGTTTGC

>7778 len=177 tot-seq=1

TGGGGAATATTGCACAATGGACGGAAGTTTGATGCAGCGACGCCGCGTGGAGGGTGTAGG

CCTTCGGGTTGTGAACTCCTTTTTCTCGTGAAAAAGGCATGCTTTTCGGTATGTAAAGCT

CTATCAGAAGGGAAGAAAATGACGGTACCTTACTAAGAAGCTCCGGCTAAATACGTG

>7779 len=177 tot-seq=1

GATACGGTAGTGACTGGAGTTCTACGGGAGGCAGCAGTGGGGAATATTGCGCAATGGGCG

AAAGCCTGACGCAGCGACGCCGCGTGCGGGATGGAGGCCTTCGGGTTGTAAACCGCTTTT

ATCGGGGAGCAAGCGAGAGTGAGTGTACCCGTTGAATAAGCACCGGCTAACTACGTG

>7780 len=177 tot-seq=1

CATTTTACAGCACATAGGAGGGAGCAAACCTTGCAAAAATGGATCGATAGAGACTGCTTA

TTGAAAGAACCTTGAACAAGCACATGTGTAAGGATTGAGAATGTGGTGTCTGAACTGGCA

GCATGTGCCCAGAGCAGCACAGACCTCCCAGGAAAATGAAGAGAGGAAATCGAGGTC

>7781 len=177 tot-seq=1

TCTGTGGGGAAGGAAATTCCTACTACGTGCCACATTTAGGGCTTGTAACAGTCCGGTCTG

TGAACTGGGAGCTGGGTTAAGGGGAAAATGTTGGCTATGTTCTCATACACAGCAAGTTAC

ATTTTAAGTTAAAATTCAAAGCACAGCACACGTGAGCATGCCAAGGCCCTTCTCACT

>7782 len=177 tot-seq=1

CCCAGTCCCACCCCATCTCCACCATTAATGCCCTTTTTCACTAAGGACCCTATTCTGAGC

AGTGACCCCCAATCCCAACACAACCTCACCAGTAACACTTGCTCCCACCTCTTGTCATTA

GTAACCCCAACTCCCACCACAGCCCCTCATTCTAGAAGTGACCCATCCCCACCCCGT

>7783 len=178 tot-seq=1

CAATAATTGGAGACACTCAGGGAGCAAGGCACATAGAACTCTGAAGACAAAAACCAGGTA

CGGACAGAGAGCAGCCCATTATTTAAATTTCCCCTGGCTGACAGATTGGAGAGTGAGTGG

GCCAGACGCCGCTGGGAAGAGATGAAAAGACTTCATTTCCTTCCTGCAGATAAACATA

>7784 len=178 tot-seq=1

GACTGTTAGAAGCAATACATGTACATTTTTTAAAAAATGGAGCAAAACACAAGCTTGGGT

TGCAGCTCAGTTGGTAGAGTTACTGGATAGTGAGAGTAAAGACCTGGGTTTGGGGTTGGT

GAGATGGCTCAGCAGGTAAGAGCACTGGCTGCTCTTCCAAAGGGCCCAAGTTCAAATC

>7785 len=178 tot-seq=1

GTGTGCATCCCTTCACTGAATAGCTTGTGAGGGGAACCCACTCTCTACAGCACAATTTCA

GGATGGCACCAGCTCTAGGTAGAGCAGGGGCTTTATATTCGTTGTCCATACGTCCCAGGG

AACTCTAACGACTCCCCTTCCCCACTGTCCCGCACGTCCAGCCTCCCATCGTCTCCGT

>7786 len=178 tot-seq=1

TGGGGAATATTGCACAATGGGGGAAACCCTGATGCAGCGACGCCGCGTGGAGGAAGAAGG

TCTTCGGATTGTAAACTCCTGTTGTTGAGGAAGATAACGACGGTACTCAACAAGGAAGTG

ACGGCTAACTACGTGCCAGCAGCCAATAACAAGATCGGAAGAGCGTCGTGTATGGGGA

>7787 len=178 tot-seq=1

CCACTTAAGTGTCCTTGCTCAGAAGGCTTCCTGAACCCCGGCAGCTTCCTGCTCTCTGGA

CCAGCTTCCCCTGCAGAAGGATTGGTCTACTTACCGAAGGTCCAGGTTCCCTGGGCAGAA

CGAGCTCTGGTGCACTCAGAGGGCACAGGGGTTTGTGTCCTCTGGCAGCGTTCCCATC

>7788 len=178 tot-seq=1

CTTGGCCGTGGCAATCACCATAAAGGGAAATATACACTGCAAAGTCAGGTATGAGCTGAG

CACCCAAGAGGATTAGTAGTTCAAGGACTGCCTTGGCTATGTAGTGAGTTTGAGGTCAGG

CTGGGCTACATGGGACCCTGTCTACGAAATCCTGAACCAACTACCACCACCACCACCA

>7789 len=178 tot-seq=1

ATGTTGGGGAGGATGTGGAGAAAGAGGAACACTCCTCCATTACTGGTGGGATTGCAAGCT

GGTACCATCACTCTGGAAATCAGTCTGGCAGTTCCTCAGAAATTTGGACATAGTACTACA

TGAAGACCGAGCAATACCTCTCCTGGGCATATACCCAGATATTCCAACTGGTAAGAAG

>7790 len=178 tot-seq=1

CCTCTATAAGTAGCTTAGGCTGGCCTTGAACTCCTGACCCTCCTACATTCTCTTCCCAGG

TGCTGGTATTACAGAAAACATTCTTTTAGAGAAAAACCAATGCTCGGCTGGGGAGGTGGC

TCAGTGATTAAGGGCACATAGTGCTCTTTCAGAATTTCAGAGGTCCTGAATTCAATTC

>7791 len=178 tot-seq=1

TTCTGTCCAGGGAATTCCATCAGGGGAACGGGTGGGTCACAATAGCCACGGGCTCATCCA

CAGTGCCACAGTGCACAGGGTACGTGGGCAAGGGCTGCACCTGTCAGCACCCTAGGCACA

CCCCGAGTCTCCAGGCAGGGGTGTATGCACAGCCCTTCACAGTGGCAGCTCCATACCC

>7792 len=178 tot-seq=1

CAGGGTCTCCTTTGCTCCCCTGCGGGGGCATCCCTACCTTGCCTTCACTTCCTGGACATC

TGCTTCCTGGTTTTTTTGCTCCTTCTCGTTTTTCACAGCAGCTGCACTTCTGTTCTTCCC

CTCTGGCTTCTTGAGGTCTTCAGGCTCCCCTTGTTCATTTTTACCGACTTTTTCTTCA

>7793 len=178 tot-seq=1

TTTTTTTTTTCAAGCAGAAGACGGCATACGAGATAGTGGGGTGACTGGAGTTCAGACGTG

TGCTCTGCCGATCTCTACGGGAGGCAGCAGTAGGGAATCTTCCACAATGGACGCAAGTCT

GATGGAGCAAGCCTTTTGGGTGAGTGTACCTTTCGAATAAGCGCCGGCTAACTACGTG

>7794 len=178 tot-seq=1

GGGTGATGTTGTTTCAGTTCTGAGTCCAGGAAGCAAGAGGCCAAGTGTGCTTCTGCCAGC

ACTTTGGGGTTACTATCCAACCCTCATAATGCTACCCACTGGAGGATGAGAGACTGTGTG

GAGGAGAGAGGAGCTACCACAGCCAAGACCATGCAAGATCCAGAAGCCTCCAGCCGAC

>7795 len=178 tot-seq=1

GGAAACAAAGGTACTGCACGTTCCATCCAGATGATTCAGCAAAAAGTACCCAAAAGACAA

AAGGCTGCAGACAGTCTACATCTGGAGGACAGAGCGACAGACTTCGGTTCCAGGATCAGG

GGAAGGAGCAACCTGGCTGTCCAGGTGCACAGGCAGAAAGCTGGCATTTGGCAGCCTT

>7796 len=179 tot-seq=1

ATGAGTACAACTCAGACTGTTATGCTCAGCATCATGCACAAAGGACCAGGACGGGTAACT

TCTCTTCAAGACTCTGACATGACTTCTTTTCTCACAGTTCCTTCAACCATAGCTGGTAA

ATCTTGACTCCCTTCAACTCTTCTAGTCCTATCCATACCTACTCTGGTCACAGCCATGG

>7797 len=179 tot-seq=1

GGCTCCTGGCCACCTCTCCTGCTTACATCCTTCTCTCCATCCATCTGCTTGTGTATTTGG

GCTCCTTTTGAGAGTATATGAAAATAAGATCTGTAGACAGACTAAGTTTGGAAAAATCCA

GTGTGTTTTATGATCATTAAAGTCATTTTCTGCCTTAAAAAAAATTAAGTAAATCTAAC

>7798 len=179 tot-seq=1

GAATAACAGGAGAAGTGGGGCTATGCCCAGCTGAGCCTTGGTCAGAAGTTGAGTTTAGTT

GCCTGGAACCCAGCTGTGGAACTTTCTATTTAGGAATGAGGGCCAGGAATGAGGGCCAGA

GAGCAGAAAGGAGCCAAAGAGGTGGGCAGGGTAGCCAGACAGACCCACCACATAGGATG

>7799 len=179 tot-seq=1

AAGGCCAGGCAGGTGGGCAGGTTTTTGGGTCCCTGGGCAGCCAGCATGGTTTGAGTGATG

GCATCAATAGCGGTGGGAAAACACTCTGGGTCCCAAGTGATATGTGCTGATGTTGGCTAT

GACTGTTATAAGCTGAATTGGCCAGTCTCTGAGCCCATAAGTAGCATATACATGTAGGT

>7800 len=179 tot-seq=1

CATCACATGGCTGGGTGAGACTGTCTCCCGGGCCCTCTTCTCACTGTGAGCCCCATGAGC

AGGGGGAGCAGTGACCCCGTGACAGTCCCCGGCTGTCAGCTCCAGTCTTGGCAGAAGTCT

GCCTCACACCCTCGCTCAGGGCTTTCTCTGTCCTGTCTGAAGCAAAAGCCCCATGTGAA

>7801 len=179 tot-seq=1

CGTTTGCATCTCCACCTCGTCGTGGTGAGCCGCGATGGCGTTGCAGATATCCGGCTTCTC

CTTATATTTCTCGGCCAGCTTGGCGCCATACAGCGCGTGGGGCAGCTCGCTCTCCTCATC

AGGCACCTTTCCGATGTCGTGCAAGAGTCCGGCGCGTTTCGCCTTCTTGGGGTTGAGCC

>7802 len=179 tot-seq=1

GCAAGGAGAACAGATTGGGCAGTTACAACCACAGGAGAGTAAAACAAATAGTTCCATCAG

GGTGAATTTTTGAGTGAACTTTCTTAGAAAACACTTGTTAACAGCTTTAAAAGATAGTTT

ATTGGAAGATAGTTTATTGGAAAATATGCAGCAGGAAAATTAATGTGAAATAGACTCTT

>7803 len=179 tot-seq=1

CCAGTAGGGGCTGATAGACACCTTATACAGGAGAGCTCTGGCTGGCATCTGGCAGGTGCC

CCTCTGGGACAAAGCTTCTGGAGGAAAGATCAGGCAGCAATCTTTGCTGTTCTGCAGGCT

CTGCTGGTGACACCCAGGCAAACAGCATTGGGAGTGGACCTCCAGCAAACTCCAGCAGA

>7804 len=180 tot-seq=1

CAAGCCAGGCCAGGTGGAACGAGCAGAGGTCCTAAAAGTCAACCACAACCCAACAACCCA

ACAACCACACGAAGGCTCACAGCCATGTTCAGCTATAGTGTGCTCACATACATAAAATAA

AGAAGTAAATCTTTAAAAAAAGGAAAAGAAAACGCACAAAGTCATTCAAAACTGTAGTCT

>7805 len=180 tot-seq=1

GAAGCTCAGAACTGTTATCATGCTGCTCAGACAATATGTTCTTTAAGATGGGTAGAGAGA

GGGTCAGATGGTGCTGATACTGTTCTGGTGCCAGAGAAGCCACTGGATCGGTGTGGTCAT

CAGCATAGTCTCACCAGGTGCCCAAGGCAGAGGACACTCCAATCTCCCATCTTCAGTGTG

>7806 len=180 tot-seq=1

GCTGGAGTCTGAGGATATAAGGAACCAAATGGAACTGTCCTGGGACCCTCACTGCACTGG

TCACTTTCCTAGATGACACATGCCCTCTTGTTCCCTCAGCAGGACATCTGGGCAAAGTAG

TCCCTTCCTTCAATAGACTCACGGAAGTTATCTGCTTCTCTGGATGTGTGATGCATTAAA

>7807 len=180 tot-seq=1

GAATGGGAAACAGAATAGACCCTCACCCCACATCAGGAAAAAAAGCAGTGGTCCCAGCAC

TGGGAATCTCTTAGGTTGGCACAGACAACAGTCGTCAGAGGCACACAGGAGCTGGTTTCC

TCCCGTTTGCTAGGAACCCCAGGCTCCTACGGGCCATGGTACCCTCCATGCTGGAAGAGC

>7808 len=180 tot-seq=1

GCAAGAAACTATTGTCACTAGGTTCCAGATGAGATGTTGCTCAGCTTCATGAAGGCTTGT

TGCCCTGGTGTAGAGGTGTACTTACCAGGTGGCCTCCTGCTGTCAGCTCCTTTATCTGAG

GTGTGCTTCAGCAGTAGAAAGCCACCTGACCAGGTCCGCTCTTCTTAGAACAGTTAGCAT

>7809 len=180 tot-seq=1

GTGACAGACAATCAGTAAGTGGCAGATATTAGCTCACTGCAACCCCAGTACCCTCGACCT

ATGGGTGCACAGTACGGTCCTCACGATAGCTATAATTACTCGCTTTCTGGAGTTGCCTCT

ACTTCCTCGATTGCACAGGTGCAGTGGGAGCATTGACTAATAAGAGCAGCCAGCAGATTC

>7810 len=180 tot-seq=1

CTGCCCTCCTATCTAATTCCCTGACTCGGCAACCTCAGCCACTGTCCCCACAGGCCTTTT

ACTCTGAGACCTTGGGCACACTACCCCAGCTCACAGCTCTCTACTGTTGCCAATGTGTGG

TTTGTTTGTTTAGCTCCCTCCTTCTCTTCACAAGGAAGACAGGAGAAGACCCCTGCTCCC

>7811 len=180 tot-seq=1

TGGGGAATATTGCACAATGGGGGAAACCCTGATGCAGCGACGCCGCGTGGAGGAAGAAGG

TCTTCGGATTGTAAACTCCTGTTGTTGGGGAAGATAATGACGGTACCCAACAAGGAAGTG

ACGGCTAACTACGTGCCAGCAGCCGTAATACGGAGATCGGAAGAGCGTCGTGTTGGGGAA

>7812 len=180 tot-seq=1

TGGGCTGCAGGGCGCTGCGGGGGACCCTCCATCAGGCACGCTCCCTTTTGTGATGCTCGG

TGGATGCACCAAAGGAGCAACATCCCTCAAAAAAACTTTTTTTTTTAAGTCTTCATTTT

GACCTTGAACCCTGATGTCATGGCCACCTCCAGGGAGTCAGCTGTGAGAGCAGTCATTTC

>7813 len=180 tot-seq=1

TCCAAAAGTTTGACAAATGCTGCATATGAAAACTCTTCATGGAAATTCAAAAAGCATACT

TGCCAACAACAGTTTCTGAGAAGCCAAAGGGAAGGATAGATAAGCCTCTTTAGCTTCATC

TTACCCAGACTTTCCCAGGCTTCTTTGATTATCAAACCCATTTTTCATAACACATATTAG

>7814 len=180 tot-seq=1

CAGGTGGCAGGATAGGGCAGGGGAGCGAAACCCAGGGCGCCCTTGGGAGGAGCCCAAGGA

CGGAGGTTGTCTTCTCCAGGCTCCAACCTCCAGAACCAGGAGGTCTGGGGTTCCCCCAAA

GCCCCCAGCCTCAGTTTACCCCCAGTGACCAGACGGTGCTGCACAGACCACTTTATTTGA

>7815 len=180 tot-seq=1

TGGGTCAGGCTTTCAGGTGGGTTTATGTGCCTCTGGGTGGTGTGCACAGCATTGGTGATG

GCAGTTGTGATGTTAGGTCAACCCTCAGGCCTGCAGGCAGCACATACTGGCACCAGCAGT

GGCCACAGCAGACTGGCTGATTTAGTCCCCAGGCCCTCAGGTGATATGTGCATGTTGGTG

>7816 len=180 tot-seq=1

ATCCTTAAACCCCACGTGCCTTACATCAGGACTAATCCTGGACACAGGCTGGAAAGCATA

GCCTACAGGTGGTGAGGCAGGAGGAGTGGGCTCAGCTGTGAGAAGGAGAAGGAAATATGG

CTGAAAACTAGATAGGGGTCTTGAAATCACACCGAGGATTTGGGCCTTTGCTGCTATCTG

>7817 len=180 tot-seq=1

GTCTTGTCTGGTTGGGCAGGAGACCTGACCACACAGGTCAGGCCTCACAGGTCCTTCCCC

GACAGAGGAGACCCCGTTTGAGGTGGGGATCCGAGTGCAGATCCACAGCCAGGAGGAGCC

GCCCATCATCGATCAGCTGGGCTTGGGGGTGTCCCCGGGCTACCAGACCTTTGTTTCTTG

>7818 len=180 tot-seq=1

AGAGGAATATTCGTCAATGGGCGAGAGTCTGAACCAGCCAAATAGCGTGCAGGATGACGG

CCTTACGGGCTGTAAACTGCTTTGGTTTGGGAATAATCGACATTACGTGTAATGTTTTGC

ATGTACCATTCCAATAAGACCCGGCTAAGTCCGTGCCAGCAGCCGCGTAATCGATAGATC

>7819 len=180 tot-seq=1

TGGGGAATATTGCGCAATGGGGGAAACCCTGACGCAGCCATGCCGCGTGAATGATGAAGG

TCTTAGGATTGTAAAATTCTTTCACCGGGGACGATAATGACGGTACCCGGAGAAGAAGCC

CCGGCTAACTTCGTGCCAGCAGCCGCGGTATAAGCAGATCGGAAGCGCGTCGTGTAGGGA

>7820 len=180 tot-seq=1

ACTTTGACTATACATGGAGCTACAGTGATGCTCCCTGAGCAAATGGGCAGAATTCAGACT

TGGCCACATGAATTTCCCATTTCCTCTGTCCTCCTCCCACCTGAAAACTTGTTCTGCCTT

TGTCTCCCCTCTGCCTAAGTACCCTGGGACTTTAGTTCTCTAGGCTGATCCTATAAACTG

>7821 len=181 tot-seq=1

TGGGGAATATTGCACAATGGGGGAAACCCTGATGCAGCGACGCCGCGTGGAGGAAGAAGG

TCCTCGGATTGTAAACTCCTGTTGTTGAGGAAGATAATGACGGTACTCAACAAGGAAGTG

ACGGCTAACTACGTGCCAGAGCCGCGGTAATGTAAAGATCGGAAGAGCGTCGTGTGGGGA

A

>7822 len=181 tot-seq=1

TGTTTAAGGGCAGGATCTGGTTTGCTTTAGAATAGAGTTTTGACAAGTCGTTTGAACTTT

TCATATTCCCTCGATGAGAATAATAATAGTTTCAACCTCATAAGGATGCTCTAGGGATTT

AAACAGATAAAGTTTAGAAAACGTCTGATACGGAGGATGGAACCAAACAAGCTCCCAGTA

C

>7823 len=181 tot-seq=1

TGGGGAATATTGCACAATGGGGGAAACCCTGATGCAGCGACGCCGCGTGGAGGAAGAAGG

TCTTCGGATTGTAAACTCCTGTTGTTGAGGAAGATAATGACGGTACTCAACAGAGAAGTG

ACGGCTAACTACGTGCCAGCAGCCGCGGTTAATCAATAGATCGGAAGAGCGTCGTGTTGG

G

>7824 len=181 tot-seq=1

TGGGGAATTTTCCGCAATGGGCGAAAGCCTGACGGAGCAATGCCGCGTGAAGGTAGAAGG

CCTACGGGTCATGAACTTCTTTTCCCGGAGAAGAAGCAATGACGGTATCCGGGGAATAAG

CATCGGCTAACTCTGTGCCAGCAGCCGCGGTATACCAAGATCGGAAGAGCGTCGTGTATG

G

>7825 len=181 tot-seq=1

CAGCAGCAGCAAGTCTCAACCAACACCCTAATTACAGCGTAGCCAACAGAAAAGCTGTAA

CATTTATATGATGTGTCTTCAGGTGCCCGGAGTCACTGGTTACAAATGGGAGTGATCCAC

ACCTCTGGGAAGTCTTAACAATGTCACAGCCTTTTAAAAATATGCCCTTTTGTTTCAACA

A

>7826 len=181 tot-seq=1

TTGTGTCCCCACATAGGGCACAATGTAGTTAGTCAAGACAACCTAATGCTACATCTTTCC

ATTAGTGCAGTCTAGGTTCCACTCCCAGGAGAGCAGGGTCAGCTCCAGCTCTCTGGGCCT

TTGGGGGAGGAGCTGGTGGGTTCAAGTTTTCAGAGCTGCTGAATTCACCTGGACCCAGAG

G

>7827 len=181 tot-seq=1

GCAGGCACACCAAGCTAGTAAATATCAGAGACTCAGGCACTTTTAATCTTTCTTTGTCAT

TATTCTCAGGTTGTAGTTTTTATCCTTAAAGTCACCTCATGAGCACAACATAATTGCTAG

AGTGAAATCTATCACATCAATATTCCAGGCAAGAAGAAGGAAGAACAAAAGGATATCACT

C

>7828 len=181 tot-seq=1

TTCGGCACAACCATGTCTTAGACCCGTCAAACTGCTACGGAAAGGACTCAGTTATCAAAG

TACGTTCCTGTCAATTCACAAATATTTCATCATCCCACTGTGTAAGAAGTACTATAATTG

TACTATGAGAGAGACTCAAAAGAAATAGATTGTATGGTCTCCGCCTTAGAAAGACTTATA

C

>7829 len=181 tot-seq=1

TTTCCAAAGCCATATGGAATGGGTTTTGTCATTCATTATTTTGGATTCTCAGGGTGGTAT

TCCCCTCACACACTCCTCACTCCCACCCCTGGGATAATCAAGAGTCTAATATTGACTGAA

AGGAGGCCTTTGTAGAAAAGCTAATGAAGTCATATGGAGTTGCAGGGCTCTGCTGCCTTC
C
>7830 len=181 tot-seq=1
ATCCAGAACGGAAAAGTAGTGTGGACCTATAACAAGGTAAAAGGATATGTAGAAACAAAG
CCTTTGGTTACAACAATCAAGTAATCTTCGGTGCATGGGACAATACACTTTACTCGCTGA
ATCAAAAGAACGGTCATGAGAATTGGCGTTGGACGGGCAATCTCACTCGAATGCACTTTT
C
>7831 len=181 tot-seq=1
TGGGGGATATTGGACAATGGGGGAAACCCTGATCCAGCGATGCCGCGTGAGGGAAGAAGG
TTTTCGGATTGTAAACCTCTGTGGACAGGGACGATAATGACGGTACCTGTTTAGAAAGCC
ACGGCTAACTACGTGCCAGCAGCCGCGGTATACATAGATCGGAAGAGCGTCGTGTAGGGA
A
>7832 len=182 tot-seq=1
TTCAAGAATGTGCCGGCAGCTGATCAATCCTGGAAGCTGGTGTCTCAGCTGGACTTCAGT
GTATGCTGGAAGCCTGAAGGAGGCAGCTCTAATGTCAGTGAAAGAACAGACCTGCCAGCG
AGAGTGAGGGCAAGCAGGCACAGAGAGCAAGCTTCAGTCTTTGCTCATATTTATAGAGGT
TG
>7833 len=182 tot-seq=1
AAAAAAAAAAAAAAAAGGAAAAAAGGAGGGAGGGAGGAAGGAAGGAAGGATGGGAGGGAG
AGAGGAAGGGAGGATGGAAAGGAGGAAGGGAGGGAAGGGGGAGGGAGAGAGGAGAGAGAG
ACAGAGACAGACAGAGACAGAGACAGGCAGAGACAGAAAGGAAAAAGACCCTAATGCTGT
CC
>7834 len=182 tot-seq=1
TATTATCAGGGGTACAAAACTCTTAGATGTGGGGGAAAGCACAGCCTCCCCAAGCCTGGT
GTGACTGAAGGCCTCTCTCCAGGCAAGGACCAGCAGGGCTCAGCGAGGCAGGTTCTGTAG
TGACATTTGCATCAGGGAAGAGACTGGCAGAGGTCAGTGTCCCATTCTGGTAAACCCAGC
TC
>7835 len=182 tot-seq=1
TGGGGAATATTGCACAGTGGGGGAAACCCTGATGCAGCGACGCCGCGTGGAGGAAGAAGG

TCTTCGGATTGTAAACTCCTGTTGTTGAGGAAGATAATGACGGTACTCAACAAGGAAGTG

ACGGCTAACTACGTGCCAGCAGCCGCGGTAATCAGGAGATCGGAAGAGCGTCGTGTGGG

GA

>7836 len=182 tot-seq=1

CCCCCAGTTCTAAGGCCTATGACTTCGTGTCCCCTGTACCTCACCATACTTGAACCTCCA

TTCTAAAGATGGGATCCTTAAGCATGTACCAGATGCCTATCACACAACCAAACAGCCAAA

TACAAGTCCTGGCTTGCTCCAAACTACCACGACATCGCTCTTCTGAGCCTCGCTGAGCAC

GT

>7837 len=182 tot-seq=1

ATCCAGCTGTGGGTCTGTAGATGTATGGGAAGCATACATCTAAGAAAGCTGACTGACGGA

AGTACTGTTGGTAGTCTGCCTTGATTCTTACAGGTGACTCTTCTACAGGGGCATGGTGGT

CAGCAACACGTGTTTTATCAACAGAGCGTTGATCTCATCCCCTAAGTGACCCTTACCAAC

AG

>7838 len=182 tot-seq=1

GAGCGCCGACGGGAGGCAGCAGTAGGGAAGCGGCCACAAGTGACGCAAGGAGGATGGAGC

AAAGAAGAGGGAGTGAAGAAGGGTTTCGGAGAGGAAAGATCTGGGGGGAGTGAAGAAAGA

GAGAGGTAGTAAAGGGCATTGATTTGAAGGGAATTACTTAGAAAGGCACGGCTAACTACG

TG

>7839 len=182 tot-seq=1

TGTTCTGGAATCGTTTGTGATGGAAAAAGATTTAGACACTCGTAGCAGTGTCCTGGGATC

CTGTGTGAGGGACAAACACTCAGAAACTGGTATCAGTGTTCTGGAACCCTATGTGAGGGA

CAAACATTCAGAACCCAGCGGCAGTGTTCTGGAATCCTATGTGAGGGACAAACACTCAGA

AC

>7840 len=182 tot-seq=1

TGGGGAATATTGGACAATGGGCGCAAGCCTGATCCAGCCATGCCGCGTGAGTGATGAAGG

CCTTAGGGTTGTAAAGCTCTTTCACCGGAGAAGATAATGACGGTATCCGGAGAAGAAGGA

TAGAGGTAGTAACTGGCCTTTATTTGACGGTAATCAACCAGAAAGTCACGGCTAACTACG

TG

>7841 len=182 tot-seq=1

GATTTGGAGACTGGATAATAGAGGTGAGGGAGAGAGGAGATATAATTCTCAGGTTTCTGG

CTTGGTTGCCTGGATTAGTTTATTGGTGTGATGTGAGAGAGAGATTACTGATAGAGGAAA

AAGGTAGATGAGTTTAGTTTAAAGCATCTTAACTTTGAAGTGCCTGAGAGATATCCAGCT

AT

>7842 len=182 tot-seq=1

TGGGGAATATTGCACAATGGGGGAAACCCTGATGCAGCGACGCCGCGTGGAGGAAGAAGG

TCTTCGGATTGTAAACTCCTGTTGTTGAGGGAGATAATGACGGTACTCAACAAGGAAGTG

ACGGCTAACTACGCGCCAGCAGCCGGGTAATAGGTAGATCGGAAGAGCGTCGTGTTGGGG

AA

>7843 len=182 tot-seq=1

TGGCTGAGTGCCAGCAGGTGCTGGCCTCTCTCCTGGGCTCAGCAGGGCAGGTCCTGGCTG

GCCCAAAGCTTCGTCCTCTGGAAGCCTCCTTCACCATCCTGTCGTACAAGCACCTGCTCT

TCCCTCCCTGCCACACGCTCGCTTTGGGCTCACTTGGCCAACCTCCAAGTTCCCAGCCAA

AC

>7844 len=182 tot-seq=1

TAGGGAATCTTCCACAATGGGCGAAAGCCTGATGGAGCAACGCCGCGTGAGTGAAGAAGC

ATCTCGGTATGTAAAGCTCTGTCAGCAGGGAAGAAAATGACGGTACCTGACCAAGAAGCC

CCGGCTAACTACGTGCCGGCAGCCGCGATAATGCCTAGATCGGAAGAGCGTCGAGTAGGG

AA

>7845 len=182 tot-seq=1

TGGGGAATATTGCACAATGGGGGAAACCCTGATGCAGCGACGCCGCGTGAGCGAAGAAGG

CCTTCGGGTCGTAAAGCTCCTTTATATGGGAAGATAATGACGGTACCATAAGAAAAAGCC

CCGGCTAACTACGTGCCAGCAGCCCGGTAATATAAAGATCGGAAGAGCGTCGTGTATGGG

GA

>7846 len=182 tot-seq=1

TGAGGAATATTGGTCAATGGGCGAGAGCCTGTACCAGCCAAGTAGCGTGCAGGACGACGG

GCCTATGGGTTGTAAACTGCTTTTATGTGGGGATAAAGTGCGCGACGTGTCGTGCATTGC

AGGTACCACATGAATAAGGACCGGCTAATTCCGTGCCAGCAGCCGCGTAATACCAAGATC

GG

>7847 len=182 tot-seq=1

TGGGGAATATTGCACAATGGGGGAAACCCTGATGCAGCGACGCCGCGTGGAGGAAGAAGG

TCTTCGAATTGTAAACTCCTGTTGTTGGGGAAGATAATGACGGTACCCAACAAGGAAGTG

ACGGCTAACTACGTGCCAGCAGCGCGGTAATGAGAAGATCGGAAGAGCGTCGTGTTGGGG

AA

>7848 len=182 tot-seq=1

TGGGGAATATTGCGCAATGGGGGAAACCCTGATGCAGCGACGCCGCGTGAAGGAAGAGGG

TCTTCGGATTGTAAACTCCTGTTGTTGAGGAAGATAATGACGGTACTCAACAAGGAAGTG

ACGGCTAACTACGTGCCAGCAGCCGCGGTATCCAAAGATCGGAAGAGCGTCGTGTTGGGG

AA

>7849 len=182 tot-seq=1

TGGGCATCATTGCCACTTCCCAGAGGCCCCAAGGGGTCATAAAGTCCAACAAGCTGTCTC

AACAAAGCTGTTTCCAAATCCTCTGGGACAGGATGGCTGTCCAGAGAGGCCCTGGCTGAA

TGGCCTTCGAGTCTGAGTGCCTCTCAAAGGGAGGATCAGGGTCTCGGTTCCTCTTGCCCC

TG

>7850 len=182 tot-seq=1

TCCGCCGTAGTGTTCTTGGTGATGTAACACGAATTCTTCCCGTGAACCAGCAACAGCCGT

CTGATATTTGTCCAACCAAATCTGGTAGTCAGTGCGCAGCCTCCCCCGATGCTGATGCGC

TGGGGCTCCCAGGTTGTCTTCATCAAGATGAATCAAGGGGTCAATCTTACCCAGCTCGTG

CC

>7851 len=183 tot-seq=1

TAGGAGTGGTGCAGATCACCACAACCAGTTTATGCAGTGCTGGGGATTGAACCCTGGGCG

TTGCACATGCTGGGCAAGTACTCTGCCAAGAGAGCTAAGTCTGTAGACCTCAAAGTGAAG

ATTAACCCTGCCAGTGGGTTAATCTTCCTTCAAAGGTAGCTTGGACAAGAACAGCGAGTG

GTT

>7852 len=183 tot-seq=1

GCAGGCAGATCTCTGTGAGTTTGAGGACAGTTACAAAGTCCTCATGGTCAGTTCCCCGAA

ACACAACAATCACTCAGCGCTAACCTAGCACATCTTCTTACCCTTGGAACCCCCACACCA

TCATAGAATTTCTAAACCACTCCCACAGGCCATGCAGCTCAACCACCAGCCTTAGATACT

TCT

>7853 len=183 tot-seq=1

CTGTTCTGCTAGTCTGGCTAGCCAGTTTCCTTCAGGAATACTCTGACTTTATTTCGCTGT

GCTGGGATTACAGGCAGGCAATGGTACTGTGTTAGTTTGAATAAAAATGGCCCCCGCTGG

ACTGGAGAGATGGCTCAGCTGTTAAGAGAACTGACTGCTCTTCCAGAGGTCCTGAGTTCA

ATT

>7854 len=183 tot-seq=1

CTGGCTTAGGCTCTTGAAATCCCAAAGCCCACCCCCAGTGACACACTTCCTCCAACAAGG

CCACACCCACCCAAGAGGCCACACCCACCCAAGAGGTCATACCTCCATCCTTATCAGTAG

TGCCATTCCCTGGTGACCAAGCCATTCAAATAGATGAGTCTATGAGGGCCCTTCCTATTC

ATC

>7855 len=183 tot-seq=1

TGGACCAGGTAGGTGGGCAGGTTCTCAAACCGTTGGGCAGTGTTCATGGCATGGGTGATA

GCAATCGAAGTGGTGGGAAAACCCTCTGAAACCCAAGTAGTCAGTCAGCACTGGTGTTGG

AACTGGCTGTGATGGGTTGGTTGGCCTTGTCTCCAAACCTACAGGTGGGTCGTGTAGGTG

GGT

>7856 len=183 tot-seq=1

CCAACAATGAGAAAGCTGATTCCCACCAGAGAACTGGAAAAGAACTGAACAGCACACGGG

CACTTCTCGAGACTTCTCCAGACAGGCCGGGTCCGGAGGCTCGAGGGGTCCCTGAAAGGC

TGTAGATTCCATGACCCACATCCCTGTGCTACCCTAGCAGACCCACTTCCTAAGGAATTG

CAC

>7857 len=183 tot-seq=1

TGGGGAATATTGCACAATGGGCGCAAGCCTGATGCAGCGACGCCGCGTGAGGGATGGAGG

CCTTCGGATCGTAAAGCTCTGTCTTTGGGGAAGATAATGACGGTACCCAAGGAGGAAGCC

ACGGCTAACTACGTGCCAGCAGCCGGGTAATCAATAGATCGGAAGAGCGTCGTGTAGGGA

AAG

>7858 len=183 tot-seq=1

GCTCCAGGGAGTCGGGGGTGGCCTTGGGCAGGGTTTCTTCACTACGAGGGGTGTCCCCCA

AAGAGCCATGAACTGTAGAGGAAGAGAAAAAGTTCAGAGCTAAGGGCTCAGGAGATCCTG

TGTTTAGGGAAGGTGACGGTCCAATTGGGGCCCGTTTTAGCTGCACTCACCTCTCTCGGT

GGC

>7859 len=183 tot-seq=1

GTCAGACGGGTGCTCTTCCGATATAATAAGGGAGGCAGAAGTGGGGAATATTGCGCAATG

GGGGAAAAAATGAAGAAGCGACGCAGCGTGCGGGATGAAGGCCTTCGGGTTGTAAACCGC

TTTTGATTGGGAGCAAGCCTTCGGGTGAGTGGACCTTTCGAATAAGCGCCGGCTAACGAC

GTG

>7860 len=183 tot-seq=1

TGGCCCTTCTGTGGGCCTGCCATCAGGGAGGATGGTGCCACTCTCAGTGGGAGCTGCATA

GGCAAGCAGCTGTGGGTCACACGGTTTGCTCATGCCTTAGTCCCACAGAAGCTTGCAGCA

ATTGTGGTGATATTTGTCCTTGGGGCATGTAAAAGCGCCCAGTCTACACTCTCCCTCCTT

GTT

>7861 len=183 tot-seq=1

GCAGGTGGGAGGCGCTTACTTCCCTCGTGGCTGGCCTTGACCCCCCACACTGCTACAAGT

TTCAACAAGACATGCTTACCAGCAAAGGAAGGACCCAGGTCCCCAACAAGGGCAGGCACT

GGGGCTGAGCACCCCTCCCTCTCTCGATCTGGGAATCTGAGTTCAAGGACTCCCAGGCTC

TGT

>7862 len=183 tot-seq=1

TGACTGGAGTTCAGACGTGTGCTTTCCGATCTCTACGGGAGGCAGCAGTGGGGAATATTG

CACAATGGGGGAAACCCTGATGCAGCGACGCCGAGTGAGGGAAGAAGGTTTTCGGATTGT

AAACCTCTGTCCTTGGTGAAGATAATGACGGTAGCCAAGGAGGAAGCTACGGCTAACTAC

GTG

>7863 len=183 tot-seq=1

AATGGGCACAGAGTGTCTGCTGGCGTGGGAGCAGAGCCAGCTTTGGACAGGTTCAAATCC

CTGGTTGTCACCTCCTACAGGGTTATGTAGGGGCAGTCACACCCCTACTCCATACAAAGA

GAAGAGCCCTGTCTTGTGGAGTCAGCTGGGGCCTGCAGCCCTGAGCACACCTCTTGAGTC

ACC

>7864 len=183 tot-seq=1

GTGGCGGGTACGACCGGAGCTCTGGCGGGTCCTACAGAGATAGCTACGACAGTTATGGTA

AGTGGCGCTCCAAGGGCTGGTGGTGGGAGTTCAGTGCGCCTCAGTGCACTCTGCCAGCCT

CGATCAGAGCTGGTTACTCTGCTGACCCTGTAAGGGACAAGATTGTCCTCTCATGTCTCG

GTG

>7865 len=184 tot-seq=1

AGGGAGAGGCTGATGTGAGATTCTGAGCCTTAAAATTCGCAGCTTTCCCGCGTGGGGTTT

TAGGAACATCCATCTCCCTTTCTTCTCTTTCCCCTACTGAATTCCTCCCACAAAATGATG

GGGCCTCTCTTCCATTTTTTTTTTTAAGTAACAGGTCTGTGTCTTCACTGTTTTCTACTT

TATG

>7866 len=184 tot-seq=1

CCAATGAAGGCCCAAAGAATCTAGTAGTTGTTTAGTTCACAAGGCTGGATGTTTCATCTG

CTCTCTAATATAAGCCAGAAGCCTGAAAAAAGTGGGGTCTGATGCCAGTGAAGACTTGCC

AGCGAGAGTGAGGGCAAGCAGGCAAAGAGCAAGCTGCCTTCTTCTGTGTCCCCTGTGTAA

GTGG

>7867 len=184 tot-seq=1

AGATGCTCAGGGGTGAGGAAGGAACACATGCTCTTACAGAAGACCTGGGTTCATTCTGGA

ACCCACAATAAGTGGCTCACAGCTGCCTGGAACCCCAGCTCCAGGGGATCTTCACCCTCT

CCTGGCCTCTAGAGGCCCGCACACATACTCTGCCGTACACGCCCGCAGTCGCTCACACAG

ATGT

>7868 len=184 tot-seq=1

ATACCGGCCAGACAGGCAGAGCGCAGAGGAGTGCGCCCCAGAGATGAAAAGAGAAACGAC

AGTGCCCGGAGCACCCGTTTCGTGGTTCTTCAGCCCAAATCAGAGGCTGGGACAGCAGGA

GGGGGAAGAGACCTGGACCCAGGGCTGGGCATGAGGCAAGGACAGCAGAGAGCTGAGTGC

AACG

>7869 len=184 tot-seq=1

GGGAAAAGACACATGGAAGCAAGAAGATACGCACTGAGCTGCGCAGGACCTGGGACAGTT

TGAAGGCCTCTCTCCTTTGGGCCTCACCTTTCTCAGGGAAGCCATAGAGAAATCGGCCTA

GGACAAGTCAGAGAGAAAAGGGAAACTGCCACTTATGAGGCAGAATCCACGTCCTGTTAC

TTCC

>7870 len=184 tot-seq=1

GGAGACGGCAGGACAGAGATTTCAGGAAGACCCCTGACTGGCAAATATCACTCTCATGAT

CTCAGGGCCTCAAATGCATTATGACATTGAATTTGTCCTCTTCTGTCACCATCTTTTAAT

ATGGGCATTTAAAGCCTCATTAACTTGGCTCTTAACATCCTAGAAACCAAGGAGCTTGGA

TTGC

>7871 len=184 tot-seq=1

AATCGCTTGAACGCGGGAGGCAGAGGTTGCAGTGAGCCGAGATTGTGCCACAATACTCCA

GCCTTGGCAACAGAGCGAGACTCCGTCTCAAAATAAAATAAAATAAAATAAAAAAATAAA

GCCACAATGAGCTCTTACTGGAGTGCCCTAATGATTCTTGAACCACTCTTGGAAAAACAG

ATCT

>7872 len=184 tot-seq=1

GTGTGTGGCAGACAGTGCGTGGCTTGGTGTGGTGTTGATGCTCGCCCTGAGGATGGGAAG

GGCAGATCCATGACATTTTTCCAAGAAAGAAAGAGGAGAGCCGGGGGTGCTTGTGCCAGG

GGTTGGGGAAACTGGTTCTGATTGGGCCTGGACCAGCATCTCATGCCTCACCCCCAACCT

GGGC

>7873 len=184 tot-seq=1

CCGATCTCCGACGGGAGGCAGAAGGAGGGAAGCGGAAAAAAGGGAAGAAAGGAGGAGGGA

GAAAAGACGCGGGAGGGAAGAAGGGGGGCGGAGCGGAAAGCGAGGGGGGGGGGGAAGAAG

GAGAGAGGGAGGAACGGGCCGGTATTTGACGGTAATCAACCAGAAAGGCACGGCTAACTA

CGTG

>7874 len=185 tot-seq=1

CATGGACTTGGAGGAATTCCTGTCAGAGAATGGCATCCCCCCGAGTCCGTCGCAGCACGA

CCACAGCCCTCACCCCCCTGGTTTGCAACCAGCTTCCTCCACGGCGCCCTCGGTCATGGA

TCTCAGCAGCCGGGCCACAGCGCCCCTCCACCCGGGCATCCCGTCTCCGAACTGTATGCA

GAGCC

>7875 len=185 tot-seq=1

TGGGGAATCTTGGACAATGGGCGAAAGCCCGATCCAGCAATATCGCGTGAGTGAAGAAGG

GCAATGCCGCTTGTAAGGCTCTTTCGTCGAGTGCGCGATCATGACAGGACTCGAGGAAGA

AGCCCCGGCTAACTCCGTGCCAGCAGCCGCCGGTAATAATCAGATCGGAAGAGCGTCGTG

TATGG

>7876 len=185 tot-seq=1

CATAGGAGATATCCTGATTTGAGATCAGCCTGGGTATATAGTGAGTTTCAGAACAGCAAA

AGGCTACATAGAGAGGCTCTGTTACAAAACAAAACAAAACAAAACAAAACAAAACAAAAT

GGCTGGAGAGATGGCTCTGCAGTTAAGAGCAGTGACTCCTCTTCTGAAGGTCCTGAGTTC

AATTC

>7877 len=185 tot-seq=1

GATGGATAATGATCAGAGGCCCGGGCTGGGACCCAGAGCAGAGTGCAGGCAAACCCACTG

CAGCTAGCTAGAGCTTGAGCTGCTCCTCCAGACCCAGTCCTTCTGCTTCTCCTCCCCACT

GCCCGCCCCCCCAGACCAAGGCGCGGGGTTTCGCTTCGCAGTGGAAAGTGTGAGGGGGCT

GATTC

>7878 len=185 tot-seq=1

TGGGGAATATTGCACAATGGGGGGAAACCCTGATGCAGCGACGCCGCGTGAGCGAAGAAG

GTTTTCGAATCGTAAAGCTCTATCAGAAGGGAAGAAAATGACGGTACCTTACTAAGAAGC

TCCGGCTAAATACGTGCCAGCAGCCGCCGGTAATGGGGAGATCGGAAGAGCGTCGTGTTG

GGGAA

>7879 len=185 tot-seq=1

TGAGGAATATTGGTCAATGGGCGCGAGCCTGAACCAGCCAAGTAGCGTGCAGGACGACGG

CCCTATGGGTTGTAAACTGCTTTTATGCGGGGATAAAGGAGTCCACGTGTGGATTTTTGC

AGGTACCGCATGAATAAGGACCGGCTAACTCCGTGCCAGCAGCCGCGGTAAAGATCGGAA

GAGCG

>7880 len=185 tot-seq=1

TGAGGAATATTGGTCAATGGACGAGAGTCTGAACCAGCCAAGTAGCGTGAAGGATGACTG

CCCTATGGGTTGTAAACTTCTTTTATATGGGAATAAAATGTTCCACGTGTGGGATTTTGT

ATGTACCATATGAATAAGGATCGGCTAACTCCGTGCCAGCCCGCGGTAATAGCTAGATCG

GAGGA

>7881 len=185 tot-seq=1

ACACAGTTTCAAATCCCCGGCTCTTTCTTCAAAACACCAGGGTGTTAGGAACAGACCCTG

GATTAACACAGCACTCAAAAAGGTGCCTGAGAGGTGCAGTTATTCAACCACAAGGCCGAT

ACCCAGAAGGAAAATCAGCACAGTGAACTCTCTACCTTTCAATGGATACTTCCAGTCCCC

GTTAC

>7882 len=185 tot-seq=1

TCAAGGTCAAGCAAAGCTTCATTTGACCTGGGCGTGAAAGAATGTGCAGGACTGTGGTAA

GCAAAGGAGACAGGTGGCATTTCAAGCTTCAGGAATAGAATGAACAGGGAGTTGGAGGAA

AGAAAACACAAAATGGCCTGTTACAATAGTTAAGGCAAGAGATGGGGAAACCTTCAACCT

GGTCA

>7883 len=185 tot-seq=1

GAAGAGCGAAGAAGGGAGGCAGCAGGAGGGAAGCGGAAGAAAGGGGCGAAAGCCGGACGG

AGCAACGCCGCGGGAGTGAGGAAGGTCTTCGGATCGTAAAACTCTGTTATTAGGGAAGAA

CATATGTGTAAGTAACTGTGCACATCTTGACGGTACCTAATCAGAAAGCCACGGCTAACT

ACGTG

>7884 len=185 tot-seq=1

GGGTTCAGTGCTCAACCTAAATTTGAAAATCATGGAGCTGATGAAAACCCTCATCTCACA

GATGAAGACACCGGTTGCTTAGAGACATGATGGGCTTGTCCGTGTTCATGGGGCCAATGA

AGAACAGAGCCACACCGTGAACAGCATCTTGCACTAGGCCTGGGAGATTTTGCTATTTAC

TAACT

>7885 len=185 tot-seq=1

CGAGGGAGGGCTGGGGGCCATAACTCTCCCTGGCATCTGGCAGAATCCAGTTCTTTCCGC

TGCTGGGTGATGTCACCTATTGTTGAAGGCTATGCTTTTTATAGCTTCTGCCGCAGCTAG

GGAGGGCGGCTGGCAGGCGGCAGGGAAGGGCGCTATCGGTTTCCAAGCCTCGTGCTCCCA

GCCCT

>7886 len=185 tot-seq=1

TTCAGAAACGTGGTCAGATTTGAAGTTGAATCTGAGGGTTTGAGTTAAGGAAATAAGCTT

AAGGAGACAAGATCATTCACCACAATGTCACACAGGCAGAGATCAGATACAAATCAGAGA

ATGCCAGAGGTGTGATAAAATAGTTGGTCAATTGGGGCCAGGCACAGTGGCTCATGCCTG

TAATC

>7887 len=185 tot-seq=1

AAATAGCCGTACAAATGGTGAAGCAAAACAAAGCCGATGCACTCATGAAAGGCTTCGTTA

ATACCGATGTTTTGCTAAAAGCAGTGCTCCATAAAGAACACGGAATTTTGCCAAAAGGGA

ATGTTTTGACACACATTACAGCAGCAAAAATAAACGATTATCCCAAGATTTTATTCTTCA

CAGAC

>7888 len=185 tot-seq=1

TCCAGTTATGAGCAGAGACTCTGGGCTTTCACAGAAGGTAGCCTAGCCCTGAGAGGAACT

ACCGAGTAGGCCCATGGTCCTTAATGGCTGTGAAAATGATGACCAGTATCCAGTATCTGC

TGCTCCACCTTGACGCGTGACTTGTTCGAAGACAAGAACTGCCATCTGGCCATATGTCTC

AAGAG

>7889 len=185 tot-seq=1

GGAGCCAAGGACCAAGAAGGACTGAGGTACAGTCATTCTGCATCCAAAGGCTTAAATTGT

AAGGGACTGGCTTTACTCTGGCTGTTTCCGGAAAGGCAGGCCCAGCCAGCCCTCCCGTCT

CTCTCTCTGACAGCCAACCTCACATGTGCCTCCCTGGGAGCACCTGCTCCGAGCTGTATC

AGCCC

>7890 len=186 tot-seq=1

TCTGACAGACAGGGGGCAGAATAGCTCAGTAAAAAAAAACTTCTCTGAAGGGCCAACATT

CCTAAAATAGACCTTTGCTTGACCTCAGGCGTAGAGGTCCTAGTGCCAAGGCCCTAAGAA

AGCAGCTCATCTCATGTTTAAGACTGGGCTAGAAGGTGTCTATGGGGCAGGCCCACACCT

TGGTGG

>7891 len=186 tot-seq=1

CACCTGCAAAGCTTTTGAACCTAGTTTGTTCCTGTCTGAAAAAATGCAGGGACAAAAATG

GAGCAGAGACTGAAGAAATGACTGACCAGTGACTGGCTCAACAGGCACCAATCTCTGACA

CTATTACTGATTCTATGTTGTGCTTGCAGAAAGGAGGCTTGCTTGACTGTCCTCTGAAAG

GCTCTA

>7892 len=186 tot-seq=1

GCATTGCAATTCACAACAGTAGCAAAAAGACAGTTATGAAGTAGCAACGAAAGTAATTGT

ACAATTGTGACATGAGGAGCTGTATGAAAGGATCAGAGTGTTCGGGCGGTTGAGAACTGC

TGCTCTGGTGTAGAGGACGTGAAGACATGGATGCTGAGGACAGGCCTCTCCAGAGAACCC

AGCCAG

>7893 len=186 tot-seq=1

TGTCATCCTATGCTCTTGGACAGAGAGAGCATGATGAACAGAGGAGCATGTGGCAGAGGC

TGCTCCTCTCTTGGTGGACAGGAAGTGAGAATGAGAGATTGGCCAGGACCAGTCCAACCT

TCAAAGTCTTGCTTTTTCCAACTATGTTCAATCTCTTGACATTTTTTAGAACCTCCCAAA

ACATTG

>7894 len=186 tot-seq=1

TCTGCCACTAACTGACCTCCAGTTTACTCACACTTCACATCCCCAGGCCCAAGGAAGACT

TTCAGGGTCTCCAATTCCATGGCCTTCCTGCTTTCTGCTAGATACTAGACTGTTCAAACT

CCTGCCCTTAGTGAAGTTCTCCGTTTTTCACCGCAGAGGTCTAAGTGGGTAAAGTCGATT

GTGAAC

>7895 len=186 tot-seq=1

TGGGGAATTTTGCACAATGGAGGAAACCCTGATGCAGCGACGCCGCGTGATTTAGAAGGC

CTTCGGGTTGTAAAAATCTTTTGTATGGGAAGAAAATAACAATACAGGACTCTTTCGAGG

CCCTGTAATTGGAATGAGTCCACTTTAAATCCTTTAACGAGGATCCATTGGAGGGCAAGT

CTGGTG

>7896 len=186 tot-seq=1

GACCTACAAGGGTAGAGAGGTGCGCTGCAATGATGCCAACGAAGAAGGTCATGGACGCAA

AGCGCAAGCCACAAGAGGTGGCGGAAGGGCTGCAGGTAGACGTGCGGAGGGCGGACGCAA

GAATGGAAAAAATGCGAAACGACCCTCTTATCAGCAGGAAGACACTGGCGACTGGCGACA

GTTTTT

>7897 len=186 tot-seq=1

TTCTTGTTCTGCCCACCAGATATACCTGCAGACCTAGTGTATCCTCTCGCCCAGAACACA

GGACATCTTATACAGGGGGCTCTATCTACAGGGGGCTCTATGCTTTGACAGGAACTGTGA

ACCTATGGGCAGCGGATTTTCAATTCATGTTTGTAGCTCTCATTACAAATGTCCTGCCTC

AGGCAC

>7898 len=186 tot-seq=1

CCGGGAAGGAGCGGAGATGCCGGATCGGACAGGCTTCAACCAGGAGAATCCGGCTTGAGA

GAAAGATGGCTTCCTCCACATCATGGGTGATAAAGAGGACCGTCTTTTTTTCTGCCCGGC

ACTGTTCCAAAAGCCACTCCCGCATGGAGAGCCGTGTCAGGTAATCCAGCGCCGAAAAAG

GTTCAT

>7899 len=186 tot-seq=1

CAGAGTTCTAGAATCCTATGTGAGGGAGAAACACTCAGAACCCAGCAACAGTGTTCTGGA

ATCCTTTGTGAGTGACAAACTTTCTCAACCTCGTAGCAGTGTTCCGGAACGCTATGTGAG

AGACAAACACTCTTAACCGACCAGCAGTGTTCTGTAATTCCTATGTGAAGGACAAACACT

CAGAAC

>7900 len=186 tot-seq=1

ACTGATTTCATCTTGATCGTCCAGGTAATCACTCCAGCCAACACTATAAGTCCCTTCTTA

GCCTTTTGACTTAAAGGTAGGAGGAGCTCAAAGTGTCCAGGTGGCAAACTTAACTTCCAG

TTTAATGGAATCATTGTTGTGTCTCCTGGTGGCAGCATTCCTCCCTTTGGAACCAAGACC

TCTAGG

>7901 len=186 tot-seq=1

TTCAGATATAATAAGGGAGGCAGCAGTGGGGAATATTGAAAAATGGGAGCAAGAATGATG

CAGACATGCCGCGTGTGTGAAGAAGGCCTTCGGGTTGTAAAGCACTTTCAGCGGGGAGGA

AGGCGATAAGGTTAATAACCTTGTCGATTGACGTTACCCGCAGAAGAAGCACCGGCTAAC

TCCGTG

>7902 len=186 tot-seq=1

TCCGATATAATAAGGGAGGAAGAAGTAGGGAATATTAGGAAATGGAAGGAAGTATGAAAG

AGAAACGCAGCGTGAGTGAAGAAGGTTTTCGGATCGTAAAGCTCTGTTGTTAGAGAAGAA

CGTTGGTAGGAGTGGAAAATCTACCAAGTGACGGTAACTAACAAGAAAGGGACGGCTAAC

TACGTG

>7903 len=186 tot-seq=1

AAAAGCCTTAGTTTTACATTTTAGTGGAAACTGTTAGCAGTAGGAGGATTTGCTTTGTGT

GTGTGTGTGTCTGTCTGCTTTGCCTTATTTTGGTTCTGACCATACCACTGCTATCTCAGT

CTTAGAGGCTGATTTTGTGGCCCAGAGTGTTTCGTTCAGATATCTTTGTGTTGAGGTGAT

ATTGAG

>7904 len=186 tot-seq=1

AGCAGTAGATGGGCCAGTGTGGGGCCTGGCGTGGGGACGAGGTGCCTCCTGCCCCGGAGG

CGCCTTCTCCGGCCACCTCCTCTCACTGGCTGCTGAGCATCTACACTGAGCTGGGCACGG

CCTCATCGAGCCTCCAAGACAGGGTGAGAAGTGAGCAGAGCAGAGCAAATGGTGCTGCAT

GAACCC

>7905 len=186 tot-seq=1

CTTTTGGCTTCGAATGCTGTGAACCAAAACGTAAATATCCTTCTGGCCAAGAGAAGAAGG

TGTAGATACACGGAAACAAGAGGGCTGCAAGACACTCTCTTGACCTTGTGATGTACCTGG

AAGCAGGTTAATATTGGCGCTTTCCAAGCTTCTGGGTAAAGCGATCCACACACCTGGGTC

CACAGC

>7906 len=186 tot-seq=1

CACTGGGTGGAGAAGGGGAAAAGAGACCCCAGGAGGGGAACAGCCTACCCCTCTCCTGTC

TGAGATTCTCTCTACCAAGCTCCCAGCCACCGAGAGCTGCTGCAGGCCCTCGGGCCAGAG

TCCGCCTGCGAGAACCTGCCCTGACCTGCAGCGGGCCGAAGATGTCCCTGGACCTGGCCT

GCATCT

>7907 len=187 tot-seq=1

TGGGCAGCAGTTTCTTTCGGATGAGGCTGCTTAGCAGAATGTCTCCCCGCACAGCCTTTT

GCCAGTCTGTGCAGACCTCCCCAATGTGCTCGTCAAAGGCCCCTTGTAGCTCATCAAAGC

CATCCATGAGGAAGAGGATCCTGGAAGGCTTGCGCAGGATCTTGCACACTGGGGGGTTTG

GGTCAGG

>7908 len=187 tot-seq=1

GCAAACACAGAACAAAGGCTTGGTGCTGAGCCCCTGATCTGGCCATCATGTATCTCAGGG

CTGGGCTGCAAGCTAGTGTTTGCTCATCTGGAAGCAATATTGTAGTAACGTTAGACGGCT

CGAATACGGCATATTCTCTGAATCCGTGAACTCTACAGTGAGGAATATGGATTTAATGGA

TAATCTC

>7909 len=187 tot-seq=1

CAGCCTATCTGGCTTCAGCTTGGTTTTGTCCTAGAGCTAGATGAAAGGGGGAGGAGAAAG

GCAGGTCCCCCATTCTTCTAACTGACTCTGCAGCTCAGCTCTCCAGCCAGACTTAAATGC

CTGGAACAAGGACAATGGCAGGCCATGATCCTGGGAAAAGTGAAGCCATCACCTCCTTAG

CCAAGGT

>7910 len=187 tot-seq=1

CAACTTGTTTATAAAACCATCTTGCAGATTTTTCTAAACAAACTCATTCTGATCTTTCTG

AATTTACCAAGATAAAGTGAAACAAATAAATGCCTGCATATTGAAAAATGCAGGGGGTGC

AGGGCTGGAGAGATAGCTTAGCAGTTAAGAACACTGACTGTTCTTCCAGAGTTCCTGAGT

TCAACTC

>7911 len=187 tot-seq=1

TTGCCCCAAAACAATGGGCCTGAAACCCATCGTGGCCTGAAACCTCTAACGCGGTGAGCC

AAAGCAAAGGGTTGTCCCCTGCTCGCTCTTCTCTGGAGACCTCATTGGTGACTGCTTGGC

CCTTCTTTGCTCCGCTCAGTCCTAAGGAGCTCCACCCCGCAGTACTGTCTTCTCATCCTC

CTTCAAA

>7912 len=187 tot-seq=1

GGATGGGGCAACACTGACGAACACCCAAAGCCAAACAGGGTCAGGCGTTAAGAGTCCTAC

CAAGGTCAGGGCGTCTGCGCCTGCAGACTGCGCTCCAGTACGCTGGTGCGTCCAGACGGG

CTTGCACCCCAGAGCATCCCTTCCCTTGACCGCAGTCTAGGCAAAGGCAGCACCTCGCCG

GGAGACG

>7913 len=187 tot-seq=1

AGACAGTAATGGGGGATGTAATATGAGCTGTCTCGGTTTTGAACACAGTAGATGCTGGGA

TGCAAGTTTTGGTTGAAATTGAATAGAAGATAGGATCTACTCTACAGTCAGCACTAGGTC

TAGACGCTGCCCTGACCACACTGCCTCCACAGATTTCACACTGTCGGAAAAAACACTTGC

TCTGGTT

>7914 len=187 tot-seq=1

AATGTGTAAGTGGAGGAGTGAGAATTTCACCCTAGTTTGTTTGGTTTTGAGCCTATGTTC

CTAACTGCTACCATGATGTGGTCTCCAACTGTAGTGGTCTTGCTCATTGCTTCTCTACCT

GACAGTGGTATGTGAACAACACTTCTCTGTAGACGCCATAGTTAGACCACAGAAGTCAGA

TCTTTTT

>7915 len=187 tot-seq=1

GCAATTGTGCTGCTCAGGGTACTTTGCTCCTGGACCGCTGACCCCCCTGAGCCAGGCTCA

GGAAGGTAAAAAGCATTCAGCTCACTAGCGTCTAACTTAACACAGCACACCTCAAAATAG

GGGAGCCTGGTCACTATAAGCCACCTCCTCCCTTACCTGGAAGACCCACCTCTGGGTCAC

ATCACAG

>7916 len=187 tot-seq=1

GAAACTGTGACCACCCCAGGGCCGAAGGGACAAGGGAAGGAAATAGTGTCACCAGCCAGT

GGGAGAGGGAGCACCGAGCAGGAGAGATACAGCCATGGTCAGGGCTGTGGTGGCAGAACA

GGGTGGGGACAGGATAAGAAAAACCCCAACCCTGCCACCTCCTGTTCTTGGGATTTGCCC

AGTGGCC

>7917 len=187 tot-seq=1

AATGTATTCAGATCTTTCTTACGGCATTTTTCATAAAGCCTTATAGTTACTCAAGGAAAG

ATCCTTGAGGCCAGGGACTATCTTTCTATTAGGATCCATCTTGGTATTATCCTCAGTACC

CAATGTAGAGCCTTGGACTTAGCAGGCCTTCTGTTAATCCAGTGGTTCTCAAAGTGTGCT

CTTAGGA

>7918 len=187 tot-seq=1

TGGGGAATATTGCACAATGGGCGGAAGCCTGATGCAGCGACGCCGCGTGAGGGATGACGG

CCTTCGGGTTGTAAACCTCTTTCAGCAGGGACGAAGCGTAAGTGACGGTACCTGCAGAAG

AAGGATAGAGGTAGTAACTGGCCTTTATTTGACGGTAATCAACCAGAAAGTCACGGCTAA

CTACGTG

>7919 len=187 tot-seq=1

GGTCCTGCGTCCTCCGCGGGAGTGTTGGCGGCGGGGGTCGCTCTGCGCGGGGACACTTGG

CAGAGGGGGCGTGCGGCCTCTCTAGGAAGCTTTCTCGGAGCCCCAGACACTGGCGAGGCG

CCGCGCCCCCTCTTCTGGATTCCGGCGCGCCCCCTGAACCCGGGAGCTACGAGCGGAGTC

GCTTCTC

>7920 len=187 tot-seq=1

TGTGACTGGAGTTCAGACGTGTGATATTAAGATATAATAAGGGAGGAAGAAGTGGGGAAT

ATTGAAAAATGGGAGAAAGAATGATGAAGAGAAGAAGAGTGAGAGAAGAAGTATTTAGGT

ATGTAAAGATATATAAGAAGGGAAGAAAATGACGGTACCTTACTAAGAAGCTCCGACTAA

ATACGTG

>7921 len=187 tot-seq=1

CTCTTAACATGCCTCCTTTGTCCCCAACCCCATCCTGTTTCCCAGCTCCAACCCTCTGCA

CAAAGTCAGGAAGGACGAGATTCATGAGCCTGTTATTTTCTCATCCCCAACACCCCTACT

CCCATACCCCAACCTCCCACTGTTCCTTCACCTTCAGGGACTCAGGAATGCTCCTTGCTA

AAGATTT

>7922 len=187 tot-seq=1

CGTGACTGGAGTTCAGACGTGTGCTCTTCCGATATAATAAGGGAGGAAGAAGGAGAGAAA

ATTAAAAAATAAAGAAAAGGGGAGGTAGTGAAGAAAAATAAAAATAAAGGAATCTTTCGA
GGCACTGTAATTGGAATGAGTCCACTTTAAATCCTTTAACGAGGATCCATTGGAGGGCAA
GTCTGGT
>7923 len=187 tot-seq=1
AGAGGTTAAAGACTTCATTCATTTGGCATCTCAACAAGTGTTTCAGTAGCTCCTTTGGCC
GGATGCTCTCCTGGGTGCTTAGAATACGATAAGCAGGGTCCCTCCCCTCCTGGAGTGACA
GTCTAATGGGGACAGAGGTAGGTCCAGGCGCTGGCCCGCCTGGCTTGTGGTTTTTCTACA
GCAGTAA
>7924 len=187 tot-seq=1
CCCCCATAAAGCACTGTGAAAATCAAGTCTGGGTCCAAATGATCAGCGACAGAGAACTAA
AATGCCATCAAAAGCCCTTTCCGTGATCCCAGCACAAACATGGATGTGAACTGTGCTCTC
ACTACAGAGGCTCAAAGCCCATCTTCCAGGCCCTGTTCTTCCACCACATGTGGTAGAACT
CTCTTCC
>7925 len=187 tot-seq=1
TGGGGAATATTGCACAATGGGGGAAACCCTGATGCAGCGACGCCGCGTGAGCGAAGAAGG
CCTTCGGGTCGTAAAGCTCTTTTATATGGGAAGATAGTGACGGTACCATAAGAAAAAGCC
CCGGCTAACTACGTGCCAGCAGCCGGGTAATTTTAAGATCGGAAGAGCGTCGTGGGAAAG
AGTGTAG
>7926 len=188 tot-seq=1
TAGTGGGAACAGTCAATACCTGAGGCAAACCAAGAGATATAGATGTAAGATACCAACAAA
GTATAGGGAAAAAATAAAGCAGAAGGAAACAAGGACCAGAAACTCCAGAGACCCGGAGGA
AAAAAGAAGCTAGGTGATACCAGTACAAAGCTAAGGAGTCTGGTAGTGGTGGTACTTCGT
CTTTAATC
>7927 len=188 tot-seq=1
TTGGGAATTTTCGTCAATGGGGGAAACCCTGAACGAGCAATGCCGCGTGAGTGAAGAAGG
CCTTCGGGTCGTAAAGCTCTGTTGTAAGAGAAAAACGGTAATGATAGGGAATGATCATTA
AGTGATGGTATCTTACGAGGAAGCCACGGCTAACTACGTGCCAGCAGCCGCGGTATCGAT
AGATCGGA
>7928 len=188 tot-seq=1

CTAGTGACACCCACAGGACACCCAGGATGTTACCACCCTGGCCAAGGAAAAGGAGGAGGG

GGAGGAATGGGAGGAGCTCATCCGTGAGGGATCCTAGCATCATAACCTCTTCTGGAGGAA

GGCATGAGATCCCAACCCGGCCGAGTGGTATTCTATCAACCCTACATGGAAAACCCCACA

GGACATCC

>7929 len=188 tot-seq=1

CTAATGGCTAGTTCTTAAAACCTATTTCCCCTGACTTCTGCTCCCACTGCACCCCATTCC

CAGCTTGCAGCAATGACATGTCACTTCTGTATGGATGAGATATCAAGTCCAAACTGGAGG

TTTCTCCATGCAAACAGAATAACCAAAGACATCCAACAGGCCAGCTCTCTCAGCTCCAAA

AGCAGATA

>7930 len=188 tot-seq=1

CCTCAGGGCCCTACTGTGTCTTCCCTGGATCCTAATAAATGAAACTTGCATTTAAATACA

AACTGCTCAGCTGAGGCTGAAAGCCAATTAGCCTTAAGAACAGGCTGTTCTGATTTTAAC

AACCTGTCCCTTCTCCTTTTCCAACTTCTGATTCATTCCTCAGCTTAGCAGAGGAGGCCA

GATGTCTC

>7931 len=188 tot-seq=1

TTTTTCAAGCAGAAGACGGCATACGAGATACGAATGTGACTGGAGTTCAGCGTGTGCTCT

TCCGATCTCCTACGGGACGCAGCGACGCCGCGTGCGGGATGAAGGCCTTCGGGTTGTAAA

CCGCTTTTGATTGGGAGCAAGCCTTTTGGGTGAGTGTACCTTCCGAATAAGCGCCGGCTA

ACTACGTG

>7932 len=188 tot-seq=1

CGTGAGTTACCTCTGACTGCTGTCGCTGCGCAGTTTAATTTCAGCTGGCTAGAATCCCAG

CTGCTCTGAGAGGCCATATTCCCTTGCTGGCTGCAGTGAGGAGCTGGGTTTGTCTCTGGT

GCCTCCTGGTCCTTGTTACCTGCCGCTGGAAGGTGAGAGAGTATCTCACCTGGGACTAGC

AGAGGAAC

>7933 len=188 tot-seq=1

ACCTTGGTTGATAATCGTCATTTCTTAGGAATAAAGTAGACTGTAGAATTTTTTTTAGCA

GAAAGGAAACCCAAAGATAATTCTAGTGCAAATCCCTCACTTTATAGAGCAGAAGCTCAA

GTCCCAGAGGAACAAGTGGCTTGAACGAACATCGGAATGTTAGGGGCTGGATTTGTACCC

TCCTGGTG

>7934 len=188 tot-seq=1

CAGGTGTCTGGGCCAGAACAGCCCCTCTCCTGAGTTCCCAAAGGCCCACGCTGGCTCCAG

GACCCCAGGGGCTATTAGGAAACAAGAAACTGGCACAGGTGCCCACCCCCCTCCACCTCC

TAAGTGGTCCCCTTTTCCCCCAAAGTTAAACTTGCCAAAACAGCTGACTAGAAGGGCAGC

CTGGATAT

>7935 len=188 tot-seq=1

TAGGTGTTTAGTGAACGGGCTATGGAGGCTGCCCCACTCACTACTTCCTTTCTAGGGGAG

GCCGACTTCAAATGCGTGCACACACACAAACAGGATGGCCTGATGCAGACCAGCTGGTCT

AGAAAGGCCTTCACGGCCTCAGGGAGCCTGGGAGGCGGCCTGGGGGAGACCATGAAACAG

CAGATACA

>7936 len=188 tot-seq=1

CCTAAGAATATGTTTCTACAGTCACCCAAAGCAACTGGACATCCCCTTGGTATCAAGTAG

ACATGAGTTCAAGTCCTTGTTCAACCCCCAACCCACTCCATGAGAGCCTAAGCTGGTCCA

TCCTCAGAGTGGGCATCAGAGCACATATGCCTCAGAGCAGTTACAAGATCAGAGCGCCAA

CTCCTGCC

>7937 len=188 tot-seq=1

AATCACCTGAGTCCAGGAAGTTGAGGCTGCAGTGAGCTGTGATCGTGCCACTGCACTCTA

GCCTGGGTGACCGAGTGAGACCCTGTCTCAAAAACAGAACAAACGAACAAACAAACAAAC

AAACAAACAAAACCATGGGAGTGCAGATATCCCTTCCATGTACTGATTTTTTTCTTTTGG

ATATACTT

>7938 len=188 tot-seq=1

CAAGTGTGATGGAGTTCAGACGTGCCTCTTCCGATCTCTACGGGAGGCAGCAGTGGGGAA

TATTGCACAATGGGGGAAACCCTGATGCAGCGACGCCGCGTGAAGGAAGAAGTATCTCGG

TATGTAAACTTCTATCAGCAGGGAAGATAATGACGGTACTTGAGGAGGAAGCCCCGGCTA

ACTACGTG

>7939 len=188 tot-seq=1

AACAAAATGAGCAATTTGTCAGTGAAAAACTCTAGAGTGACTAAGAGGACCAGCCCTGCA

AAATCCATACCTGGGGAATGAAGATGAAGCAGTTGATTGTAGTTGTACAAACAAAGATCC

CTCCAGATGTGAGTCCAAAGACCAGGTTGGGCCAGGAATGCAAGTATCTGGTGACTACAA

AAAGCAGC

>7940 len=188 tot-seq=1

GCAAAAAGAGAATGAGGAAGACACAAAAGTGGAAACTCCTGATAAAACCATGAGATCTCG

TGAGACTTATTCACTACCATGAGAACAGTATGGGGGAAACTGCCCCTGTGATTCAATTGT

CTCCCACAGGGTCCCTCCCACAACACGTGGGAATTATGGGAGTACAATTCAAGATGAGAT

TTGGGTGG

>7941 len=188 tot-seq=1

CGGACAGACACGGCGTGGATCTTGCGCTGGACTTTGTGTATGCCCTGGCATATCTTGGAA

GCCCTGGTTCTCAATAGCCGGGAAAGGCCTGGAATGGTCCGAAAAGAGCTGGGCCTCTGC

TGCACTGCCATGGAAGCATTTGTGTTAGCTATTTCCCTGGGTACTGGGGTGACAGCCATG

GGCACTCA

>7942 len=188 tot-seq=1

GCGGCCGAGCGCCGACGGGAGGCAGCAGGAGGGAAGCGGCCACAAGGGACGCAAGGCGGA

GGGAGCAACGCCGCGTGAGTGAAGAAGGTTTTCGGATCGTAAAGCTCTGTTGTTGGTGAA

GAAGGATGGAGGTAGTAACTGGCCTTTATTTGACGGTAATCAACCAGAAAGTCACGGCTA

ACTACGTG

>7943 len=188 tot-seq=1

GAATGGCAAAGAGATGCCTTCACGGTGTTGATGGTGTGCGCCAGCCACCCATGCACATGA

CCTGGTGTTTCTGTCGTGGAACTGGAGATGCGGCCTTAGGTGCTGACCGCTGAGCCACAC

AGGTGGGGCTCACTTTCTCACCTATCAAGAAATATACATGGGAAACCCACAGAGCCCTGC

CTCCTGGC

>7944 len=188 tot-seq=1

GAGACTCTGTCTCAAAAAAACCAACAACAAAGAGAGGCAGGGCACGGTGACTCACGCCTG

TAATCCCAGCACTTTGGGAGGCCGAGGCAGGCAGATCATCTGAGGTTAGGAGCTCCAGAC

TAGCCTGGCCAACATGGTGACCTCTTCTCTACTAAAAATTAGCCGGGCGGTGGCGCACGC

TTGTAGTC

>7945 len=189 tot-seq=1

CCAGCTGTAATCTAAACCGGTCCCAGCCAGAGTTCTCTGGAGTGCAAAGCTCTGGAATCC

CTGGAAATCACATTCTCTGTGGATGCTACCAAGAGCTCGAAGCCCTATCTTCCATGTGGT

TGCTTGCTTGAACCTCAACGACATTCCCTCTCTTGACCTGATCCCTGAGCTGACAATGTC

ACCGGAGGC

>7946 len=189 tot-seq=1

GAACAGAAACAGGCAGACACACGTGGACAGGGCAAGGCTGCGGGCGAAGAAAGGAGAAAG

AGAAACGGAAGTAGACCAGTAAGATAAGGAGAGAAACGGCTTGTGGTGACCACAGGATGC

TGTGTTTAACAAGGGCCAGAGAGCTGTTAAGGGCACTGGATGCTCTTCCAGAGGACCCTG

GTTCAATTC

>7947 len=189 tot-seq=1

CGGCATACGAGATACCTCAGTGACTGGAGTTCAGACGTGTGCTGGAGGCAGCAGTGGGGA

ATCTTGCGCAATGGGCGAAAGCCTGACGCAGCCATGCCGCGTGGATGATGAAGGTCTTAG

GATTGTAAAATCCTTTCACCGGGGACGATAATGATGGTACCCGGAGAAGAAGCCCCGGCT

AACTTCGTG

>7948 len=189 tot-seq=1

ATGTAATTAAGGATTTTGAGATGACATCATCCTGAATTCAGGGTGGGTCTAAATCCAATG

GCTGGTTTCCTTATGAGAGACAGAAGAGGCAGATTTGAGACACAGAGAAACATAGAGAAG

AAGGCCATGTGAGGACAGAGGCAGAGATTGGAATGACTCCCAGCTACAAGCCCGGGAACA

CCAAGATTG

>7949 len=189 tot-seq=1

CTGGTCCCAGCTGTGTCCCCGTGTCTCTGTTTGCTGAGTAAGTCATGTCACCAATCTGCA

ATGCGAGGAGGTGGATGGGAGGCTCTCCCTCCTTTTTGGCCTGGGTGGTGAGTAACCTAT

GCAGGAAAGTCTTCCGGGGTTGTAGTCAAGCCATCCCCGAGTTGAATATCCCATCAGCCA

GTACAGAAA

>7950 len=189 tot-seq=1

TTGTTATTCTTAGTATTGGTCCACAAAAGTTATATTCACATTCTAGCTTTGATGCCTCTT

TCCTGGGATTAAATGAGCTGAAAGACCTATGTGAACTGTAGAGAAGACCAGGGGCTCAGC

CCAGCCCAGCCCAGCCCCTCAGGGCTCTGTGCTCTTCTAGGGATGGATGGTTTTGGTAAA

GAACAGACG

>7951 len=189 tot-seq=1

AAGCAGGGATGGCCAAGTGGATTAAGGACGGCCAAGCGCTCACCTGAGGATTTTGTCACA

GGAGGCCGGGAAGAGGCCAGGCCCAGTGGACTGGGCAGGAACAAACAAGGCATCAATAGG
GGCTGAGTCGGGAGTGCAGGCCAGGCCCGAGTGCAAAGAACACCTTCAAGAAGCTTGGCT
AGAAGGAGG
>7952 len=189 tot-seq=1
AACCAAAGACCGTTTGGTTTTTCAGGAGGAAGCGGGTGTCCCCAGGCTTTGGCACCCTCC
ATCAGAAACGCCACTGCGTGTCCAAAGCCTCAGGCTTTCTTGGGAGCAGCTTTCCGTGAT
GGGAGGGACAACCCACCAGCTGTCACCACTCAGCGCAGAGGCAGGCAGGTTCCCAGGGCA
CTGCCTGGG
>7953 len=189 tot-seq=1
CTGAGGTTCTGCACTCTGCAGGTTTGACCCAACCTAGTCCCTGAGCAGTGTTTCCAGTCT
ATCATGCTGGGAATGAGCCTGGCATTCTGGAATCAGCCTTCTCTGGAGTGCCCACCATGT
TTCTCTAGTTTGGGTAATGAGCATCGGCTTGAGTCTATGGATCAGCACTCAGTGTTCCCC
CACCCCAGC
>7954 len=189 tot-seq=1
GGGTTTGGAAGGAGTAGTTAAGTACTGCGAGGCCTGCGTTCTCATGACCAAAGTTCACAA
ATGCCCATTTCCTTCTTTCTTTCTTTTTCTTTTTTTTAATATTTAAAAATCTTTATGTGT
CTATTAACACCTTTTGGAAATTTCCATCCCTTTCAAACTATGTTCCAGTCAAACAAAACA
AAGTGTGGC
>7955 len=189 tot-seq=1
GGGGGGGGACGGGAGGGCAGACGGGGGCGCGCCGAGCGCCGACGGGAGGCAGCAGGGGGG
AGAGGGCACAAGGGGGGAAACCCGTATGCAGCAACGCCGCGTGAGTGATGAAGGTTTTCG
GATCGTAAAGCTCTGTCTTTGGGGAAGATAATGACGGTACCAAAGGAGGAAGCCACGGCT
AACGACGTG
>7956 len=189 tot-seq=1
ATATTAAGATCTCCTACGGGAGGCAGAAGTAGGGAATCTTCCGCAATGGGAGAAAGACTG
AAGGAGCAACGACGCGTGAGTGATGAAGGTCTTCGGATCGTAAAACTCGGTTATTAGGGA
AGAACATATGTGTAAGTAACGGTGCACATCTTGACGGTACCTAAGCAGAAAGCCACGGCG
AACTACGTG
>7957 len=189 tot-seq=1

TGAGAAACACCAATATTAAAGAAGCTGGAAAACCAAGAGGAACCTGCATAGAGAAGGCTA
GGAATGGTGAAAACTACACTGTGGACCTAAGAGCAGTGGGTGAGCCAGAAACTGCTGCAG
AATTTGGAAGGAGGTGAATAATTGGTGATGCCAGCGGGGCCACACTCACAGGAGCCTGCC
ATCCTATTC
>7958 len=189 tot-seq=1
AGGACATCATCCTGGAGCAACCCATACCCCCAGGGAGCTCTGACCTCAGACTTCCCAAGG
GACTTCTGCACCTCCTTCAGGAATTCCAGCCGGTACTCAGCTTCCTCCAGGTGGCCTTCT
AAGATATGACACAAGATGATCCCTGTAAATGGGACAAAACAATGATGAGCAGCTAACACT
GAAGCACAG
>7959 len=189 tot-seq=1
AATTGCAACTTATATCTTAATATGTTTATAGCAGGCAAAATAAATCATTACTCTTCCAGG
GTAGAAGGTGTCAACTTGCCACCAAGTATCTCCTTGATGTCCTTGAAATCCTCCTTCTTT
TACCCTTAGTCTTTATGCTATTTAATCATCTTCAGCTACTTTAAAATCATATTTTCTAAA
GCATGGTCT
>7960 len=189 tot-seq=1
TCATGCAACAAGCCGTCAGGCTATTGGTTTTCAAGATGCACGACTAATACCGTGGCTTTC
TGTAAACAAAAATGTTGTTTTCGGCATGGAAGGAAGCAAGCAAGATCTTAAGAATATTGC
GCATTTAGCGCTTTGCCAAATGCGCCTTGAAGGCTACGATAACAAGTGGCCTTCTGAGCT
TTCTGGAGG
>7961 len=189 tot-seq=1
GCGGGGACAGAAGTAGTTTATCACTGACGTTGATTAGAATTGCTGGTGCATCACTGTTAG
TCAGTTTATTATTGTTTGTAATTTTTCTGTAGATGATATGACCTCTTATTGTACCCATGT
GCACCATTCCTATGGCCCCGTCCTTGGGGACACCACTGCTTGGGAGTTAAGATGGTGGAG
ATCTGGACC
>7962 len=189 tot-seq=1
TGAGGAATTTTGCACAATGGACGAAAGTCTGATGGAGCAATGCCACGTGAACGATGAAGG
TCTTCAGATTGTAAAGTTCTTTTATTTAGGAAAAAAAGCGTGCTAGGAAATGAGCGCGCC
TTGATGGTACTGATTGAAGTAGTGACAGCTAACTATGTGCCAGCAGCCGCGGTATAGAGA
GATCGGAAG

>7963 len=190 tot-seq=1

CCAATGCCAAGGCTATTAGGGAGTGTTGCTTTCATGCTTACTCTTTTTGACGTGGTTAGC

CTGCCTCCTTATAGTATCTAGGGTCATAAGCCTAGAGGTGGCACTGCCCACAGAGATGGG

CGCCCCCACATAAATCTTCAATCAAAATGCCCTACAGGCTTGCCCACAGTCAACCTGTGG

AGAATTTTTC

>7964 len=190 tot-seq=1

ACTCTAAAGCAGCATGACTCTCACTGGGACCCAGTGCCCATTTAGGGCACTTTAGGGAGG

GTAGGGAGAAACCTGATGGAGAGAGTGCTGGCGGGCCCTGGGGTGGAGGGACCACTGGGC

TCACAGGCAGCTGGGGCGGCCTAGGGAAGGATCAGAAGCTGAGATCTTCAGAGCAGGCTG

CCGCTAACAA

>7965 len=190 tot-seq=1

AATCCCGCAGGAATTTTCACCACTGGCGTTGTGTAAAATGGGACTCCGGACAAACGTAGC

ACTATGGGAACCACTGAATCTTTAAAGTTTCCAAATAAAAGGAATATGAGCATTTTTACC

TCTTGGCTCGACTCATCAAAGTGCTGGCTTGGGTTTGCCAGTCACTAAATCATAAATCTG

CCAGGCTACC

>7966 len=190 tot-seq=1

AAAGAATATCTTAAATATTTGTTATTTCAAGTGAGTGGGATGAAGTATAATGCTATCAAA

ATCGGTGCTGTATAAAAACACAAGTCACTGGTGTTTCATCTAAATGCAAAAAGGAATGGG

TGATCACTGTCTATACACAAAGACTTTCAAGCAACACAGAGACAAAGATGAATAATAATT

ACCTTAGCAG

>7967 len=190 tot-seq=1

TTTGCAGGTGTAATTAGTTAAGATGAGATTATACTGGATTAGGGTGGGCCCTGACCCAAT

GGGTGGTGTCCTTATAAGAAGAGAAAGCAGAGACACAGAGACACGCAGGGAGAATGCCAC

GTGAGGACAGAGTCGGAGACTGCAATGATGTGTCTCCAGGCCAAGAAATGCCAAGGACTG

CCGGCCACCA

>7968 len=190 tot-seq=1

CCCGCCTCCTCCCCACCACCTGCGTGCAGCTGACCGAGGAGAAGAACCTGCTACAGGAAC

AGCTGCAGGCAGAGACAGAGCTGTATGCAGAGGCTGAGGAGATGCGGGTGCGGCTGGCGG

CCAAGAAGCAGGAGCTGGAGGAGATACTGCATGAGATGGAGGCCCGCCTGGAGGAGGAGG

AAGACAGGGG

>7969 len=190 tot-seq=1

CACAGGGGCATCTGCCAGCTCCCCCACTGTGGTTGGCATGATTTGGGGCAAACACTGTTG

TGATGAGAAGTAGATGGATTGAGAGATTCCAAGCTGCCTCTCTATCCTCCAGATGCCTGG

TGCCCTGAAGGGAGCCCTAGTGCTGAGTGTAACTGGAGCAGCCTCAGCCCAGCCAGGACT

AGACCAGGGA

>7970 len=190 tot-seq=1

AGAGAAAGAGAAAGAAAGCAAGAAAGCAAGAAGAAAGAAAAGAAAGAAAGAAAGAAAGAA

AGAGAAGGGAAAGAAGGGAGGAAGGAAGGAAGGACCTGAAAAAGTGATTGTATTTGGGAG

GAGGGCATTCCTGGCAGAGCACACAGCAAGTGCAAGGGCCCTGGGGTGGGAGTGCGCCTG

CCAGGCGAGG

>7971 len=190 tot-seq=1

GTGTGCTCTTCCGATCTCCTTCGGGAGGCAGCAGTGAGGAATATTGGGCAATGGGCGAGA

GCCTGAACCAGCCAAGTCGCGTGAAGGAAGACTGCCCGCAAGGGTTGTAAACTTCTTTTG

TATGGGATTAAACTCACCTACGTGTAGGGGTTTGCAGTTACCATTCGAATAAGCATCGGC

TAACTCCGTG

>7972 len=191 tot-seq=1

CCAGGGCTGTCATTTCAGTGCCTGTCTGGTCCAAGTCAAGGTCTTGTTTCTTCCCATGGT

GGGAGCTCCAGAGGTGAGTCCTAAGCCACAGGGAGAAGCGGCAGCAGCTTAAGGCACCTT

GTCTGTTCCCCTTCCTCTCATTGTTTCTAAAAGACACCGATAGCCGGGCGTGGTGGCGCA

CACCTTTAATC

>7973 len=191 tot-seq=1

GGACCTCTTCCTTAAATTCTGTGGATTTTGCCCTGCCCCTTGGGGGGCCAACTTGATGGT

AATCCAGCCAAGGAAGAAGGGTCAAACAGATCATCCTACAGAGCCCACTTGGCCAGTTGC

TTTCTGGGACACTTGGCAGCAAGGAGACGGTTTCAGGGTTAGGCAAGCATGGATGCGTTC

CTACTTGTGCA

>7974 len=191 tot-seq=1

CTGTCTCTAAAGAATCCACAACAAGCAATAGACTTTCAACTGTATCCAAAAGAAATCTGC

TAACAGTGTGCAGTCCAGCTGGGGATGCAGCTCAGCTGGTAAAGTATTTGTCAAGCGTGC

GCAAAGCCCTGAGTTCCGTGCCTGGCAAACACATAAGCCAGGAGTGGGGGAGGGTGGACA

CACCTGTGATC

>7975 len=191 tot-seq=1

CCTGCCCCTTAGCCTTATCTATGGTCCAGACCCCTTTATCATCAGCAACAAGGCTTGGGC

CCGTGGCTCCCCGTGCAGGCCGCACCTGAGGGGCTTGCGCTGGATGCACACCCGCTGGGC

CAGGCCGCTCAGAAAGGCAGGTGGACTCTCCTGCACCACATGCTGCACGCGCAGACGCCA

CTTGACATACT

>7976 len=191 tot-seq=1

ATACTAGAGGGGATTTCTCTCAGACCCACTCAGCTGGGGTTAGCCTTCCCTGACAGGCCT

TAACCTTTTTATAACCTTGTATATCTGAAAATAGGATCCTCTCCCCAACTCCACTCCCCT

GGCCCTGTACCATATCACCAACTGCTAGTGGATTCTGGCTCAGGTTTGGGATCACCATGC

TCTCATGGGAC

>7977 len=191 tot-seq=1

CAGTGTTCTGGAATCCTGTGTGAGGAACAATCATTCAGACACTCGTAGCTGTGTTCTGGA

ATTCCGTGTGATGGACAAACATTCAGACCCTCATAGCAGTGTTCTGGAAACCTATGTGAG

GGACAAACATTCAGACCACAGCAGGCATGTTCTGGAATCCTCTGTGAGGAAGAAACATTC

AGACCCTCGTA

>7978 len=191 tot-seq=1

GATATCCCGCAATTTTCCAACCGGAGCAGCCACAATCGGCAGCCCCAAGCTCATGGCTTC

TTGGGCGATGAAACCCCGCGACTCCCACTCCGAAGTAATCACCAACACGCTCGCTGCCTG

CAACCAGGTATCCAAGGACTTGCGCCACCCCAGGAAAGATACTTGTGCGTCGGTGCCGTA

AGAACGCAAAC

>7979 len=191 tot-seq=1

CACCATGACCCCATCTCGCAGTGCTCACATCCTTCCACTCCACCCCCAAGCCCAAAGGTC

CAGCAGCCAGTTAGACAGCATCAACCAAGGCCCAGAGCAACAGGAGGAAGCCTGTATTCC

AGACGGTTTGGCTGATGCTCTCAGTAAAGGTGACTCTGATAGAACTCAGAGGGAAAAGTA

AACCCTCTTCT

>7980 len=191 tot-seq=1

CTGGCCAAGGCTGGAGCTGGCTCCCTCAGCTTGTGGGGAGGAGTGGAGGGAGAGGTGTGG

GCGGGAACAGGCAATGCCTGCCTGCTTGGGGGCCAACTAGAGTTCTGGGTGGGCGTGGGC

TTGGCAGCCCCTCACTCGGAGCCGCTGGCCGGCCCCCCCTGCCCCAGGTAGTAAGGGGCT

TAGCACCTGGG

>7981 len=191 tot-seq=1

TGAGAGGTCCCCTCCAGAACAGTATGTGGTTCCAAACTCCTGAGGGAGCGGGGGCAGACC

GCAAGGAAGTAGATGAGAACCTGAATGAGGCTCTGTGGGGAGAGAGGATGTTCCTCAGCC

CTCACAAGGACATGTCAGTTTCACTTGAGATTAGGTCCTGGCCTTGGCTGGGCAGCCTGC

CAGGCAGGTTC

>7982 len=191 tot-seq=1

CCAAGTGACTGGAGTTCAGACGTGTGATATTAAGATATAATAAGGGAGGAAGAAGGAGAG

AAAATTAAAAAATAAAGAAAAGGGGAGGTAGTGAAGAAAAATAAAAATAAAGGAATATTT

AGAGGAGATGTAATTGGAATGAGTAAACTTTAAATCCTTTAACGAGGATCCATTGGAGGG

CAAGTCTGGTG

>7983 len=191 tot-seq=1

GCCGCGGCGCTGCACCTGGTATTCGGACTGCGGTTGCGACTTGACGTAGCCCTGGTGCGA

CAGGGTCACGATCATCTCCTGCGGCGCAATCAGATCCTCGCGGTTCAGGTTCAGAGCAAA

TTCGCTGATCTCGGTGCGGCGGGCGTCGCCGTACTGCTCCTTGATCGCCAGCAGCTCCTC

GCGGATGACCT

>7984 len=191 tot-seq=1

GAGTGGCCACAGCCCCCCTGCAGGCGGCTGCCTCCACCTGTTCTGAATGCCCCTACGCCA

CACCCTCAAGCCCTGAGCAGGAAGGAGCAGCCATTGTGCATTTCAAGGCATGTCCCACAT

CTGTAGTTTGCAGAGAGCTCACCATATTCCAGGCAATATTGGGAAATGGACAAGCTGGTG

CTTGCCCTCTA

>7985 len=191 tot-seq=1

CCACTGTGTGCTCGGTGCTGACCTGCAGGCTTCCCACTGCTGGTCAGAAGCACATACTCA

GTGGAAGATCATCTGGAAAGCTTTGCTGAGCCCACCCTTGCGCTTACCCAGAATTCACAG

CAAGGCCAAACAAGCCCCACACCCATCCCTATTTAACTGTTCATGCCTCTCCAGGTATTC

CCAACGCCAGC

>7986 len=191 tot-seq=1

GAGGGAATCTTCCACAATGGACGCAAGTCTGATGGAGCAACGCCGCGTGAGTGAAGAAGG

TTCTCTGAGAGTAAAGCTCTGTTTTTGGTGAAGAAGGATAGAGGTAGTAACTGGCCTTTA

TTTGACGGTAAGCAACCAGTAAGTCACGTCTAACTACGTGCCAGCAGCCGCCGGTAATCA

TTAGATCGGAA

>7987 len=191 tot-seq=1

TTCTCATGCCAGGCCTGCCTGCAACAGCAGTGCCCAGTCTGGTTTGTTCGGCTTGTGGAA

CTGTTTCTTCCCCATCTCTAGAGAGCTGAAGCTGATTTCCTATCAAACACCCGCTTTGCT

GTCAGATGACAAAGCCTGTGGTTTGCCCACATTCTGGGTCCTGAGATTTGAGAGGCTCAG

ATGAGACACAG

>7988 len=192 tot-seq=1

TGATGGTTTCTTGGCATGTTCCCCTGGTAACTTGGAAGGTCTGTTGACTTCTTTATGAAG

TTTCACTGTTCTTCAGCAAGTGTTGCCAGTCCTGGCTGCCTGCCACCTGTCCCAGCTGCC

TGCCACCTGTTCCAGCTGCCTGCTGTCTATCCCAGCTGCCCTGCCACTTGTCCCAGCTGC

CTGCCAACTGTC

>7989 len=192 tot-seq=1

TGGGGAATATTGCACAATGAGGGAAACCCTGATGCAGCGACGCCGCGTGGAGGAAGAAGG

TCTTCGGATTGTAAACTCCTGTTGTTGAGGAAGATAATGACGGTACTCAACAAGGAAGTG

ACGGCTAACTACGTGCCAGCAGCCGCGGGATCGGAAGAGCGTCGTGTAGGGAAAGAGTGT

GCCAATGTGTAG

>7990 len=192 tot-seq=1

TATAAACACCTTGTTATTAATGACTGGAGCTAAGACATCCAATGGAAAGGGAAAGAATTA

GCATCAAAGATTTTATGGAAGGCAGGTTTATGACCAAAAGAACTCTTTAATATCATCCTA

AGTAAGGTAACCCAGACCCAAAAAGATATGAATGTCAGGAACTTAGCATAATTGTACTCT

GAGAGGCTCGAC

>7991 len=192 tot-seq=1

TCGAGGGCTCCTCTCCTAGGTGAACAGTGTGCCTCCAAACACCAAACTAACAACCCATCT

CTAACAATCCCTTTGTAAAAGCTTGTCTTCCACAGGATAGTCACGGAGTGGAAGATAATT

AGGCAAGGTTTTCATCAATTCCATTTCCTCTCTTTTTAAAGAGGGGAGAGGCAGCTGGGT

AGCCATCTCACC

>7992 len=192 tot-seq=1

TGGAAGCCCATGCCTCCCTGGCGAGGTGACTTCCTCTTCTATCCCCTGGCTCTGCCTGTA

CTCCGTCATCCACCCTCCTATTTATCCATCCACCCATCCCTCCATTCACTTATTCATTCC

TTCCTTTATACATGCAAGCACTGTTCATTCTGTGCTATACTAGAGCCATGCAAGGCTGTG

GGGAAGCAAAAT

>7993 len=192 tot-seq=1

ATTGTGGGCCCAGTGGGCATGCAGGCAGCTGCGCTCCCAGAATCTCCACACAGAACTCAC

ACCTTCCACTAAAAGGGGCAGGGACCCACGAGGAATGATTGGGAATGAGCCTGATCCGGT

AAGAACATCGAACTCAAAAAGAAATTAACTTTGAATTACAAAACTGCAAAGTAGGGACCC

AACTCCAGGCAC

>7994 len=192 tot-seq=1

GGACGGGGCGGGAGGGAGATCGTAAAGGGCGAGCGCCCTCTCCCGGCCGCGGAACGGGCA

GGTGATCTGTAAGCCTCCCCGCTGGTCTTGGAGTGCTTGGGTGCCCACCTCCATCACGCT

CCATCTCTCAATTCGGTTTGAACCCGGTTTTCTCCCACGCTCCCTCTCCAGGCGAGGCCA

GCACCAGGTCCC

>7995 len=192 tot-seq=1

CCTGTGTTCTCGATGAGCATGTGCTGCGGTGGCCGGAACCTTGCCTGGCACCAGCGGGGA

TGTGCAAAGGCCCTGGGGGAAAAGGCAGAGAGGCTGGCTCTGGACTCGGACAGGATGTGA

TGGTCACCCTCCCTCTTGGTCCTGGAGCCGCATTCCCCGTGTGGGTGCCTCTGCAGGGTC

ACAGGATAGCAG

>7996 len=192 tot-seq=1

TAGGGAATCTTGCACAATGGGCGCAAGTCTGATGGAGCAACGCCGCGTGAGTGAAGAAGG

TTTTCTGAGCGTAAAGCTCTGTCATTGGTGAAGAAGGATAGAGGTAGTAACTGGCCTTTA

TTTGACGGTAATCAACCAGCAAGTCACGGCTAACTACGTGCCAGCAGCCGCGTAATTCAG

AGATCGGAAGAG

>7997 len=192 tot-seq=1

GAGGGTCACGGGCCAGGGAAGGGCCTCCTGCTCTGGCTGTGGTGTGCCAAGTCGGCGGGG

AGCAGGGTGTCCAGGGCTGTTTGCTCTAACCAGCCTGCCTCTCCTCCCTGCAGAGTTACG

AAGTGAGGAGTGTCCTTGGAAAGGAGGTGGGGCCGTTAACCTGTTTTGTCCGGTGCATCA

CCACCCACCCGA

>7998 len=193 tot-seq=1

AAGATCCAGTTGCCAAGAATGGACCTCATATTCCTTGAGTTAGGAGAGATGCCTGGCTTC

TTTGCCACGCCTGCCAGCCTACATTAGGCGAGCCTTGGGCCTCCTGCATGCAGCAGGGGA

AATTAGAGCCAATCAAGGCGATTTCAAGGGCACTGCTCCTTCCCAGCAGCCTGTGACCAT

GCCTGTTAACCAG

>7999 len=193 tot-seq=1

AGCAGATGCAACATTATTCCCCAGTCAAGATGTTTAAATTATGTTTTGTTTTGTCTTCCA

TTAAGTGCAAGTGAATGTGTTCATATGTAAAATATCTCTTGCTGAATGTGGACAGTTTAC

ACACTTAACACATATTCTTTCTAAAATTACTCTGTACATAATAAGCCAGGCATGGTTGTG

CATGCCTGTAATT

>8000 len=193 tot-seq=1

CGAGTTTAGGATTTTGTCCTGTTTACTGTCATGTACACAGTTTCTATGCTACCACATGGA

GCAGCTCATGGATTTGATGGTAAGAACAAAAAGCTTTGATTCCCATTAAGATCTCTAAAA

GTGGGGACTGCAGAGATGGCTCAGTGGTTAAGAGCACTAACTGCTCTTCCAGAGGATGCA

TGCAGGCCAATTC

>8001 len=193 tot-seq=1

AGGCCTGTGGGCAAACAGTGATGAGAACGTTTAAGGGTGTGTGGAAACAGACAATAAGGA

TGGAGCTAAACACTAAACACAAGATGAAGAGCACCCCTTTCTTTCATGACGGACAACAAA

ATTCTAGCACCTCTGAGTAGCAGAGGCTGTATACTGTAGGACCTCCAGAGGCCGCATGTC

AGTGAGGAACGTT

>8002 len=193 tot-seq=1

TAAGACCATGACCCTCACCAGAGTGCAAGCCCAGACATTACCCTGGAGCCAAGTGGCAGC

TGAGGACACTCACATCAATATGGCCCATGGTGGCAACATTGCCCAGGAACATCAGCATGT

CTTCAGGCTGCAGCACAAACAGATATCCACATGGCCTTAAATGCCAGCTCAGTTCATGGC

CATCAACACAGAC

>8003 len=193 tot-seq=1

CACAGGGAACACTTGACATAAAGGATTTTCTGTGAGTCACGATCACTACTTGCACACAGA

GGGGAGTCATGCACTTAGGAAAAGGCAAGTAACTGAAGTGATATGAAAGTATTTGAGAGC

ACACATTTCCACTGAGACTCTGGACTTCCCTGTCCTATAGCTCCTTCTCCCCAACACTCC

TATACATTAGTAT

>8004 len=193 tot-seq=1

GGGCCTGATTGACGGGCCAAGAGGCCTGTTGAAGCCGACCCTCTGGCAGGTGAAAGCCAA

TGGGAGAGGGGCAAGAGGAGCCTCTCGTGGTCAACCCCCCGCCGAGGAGCTCCGCGGGAG

TAGGCCCCGGGAGAGAGTGGGATAAGGAGTAACTTTCTCCCGGCACTCCTGCCCGAGGAA

GCCTGAGAGAGCC

>8005 len=193 tot-seq=1

ATAAAGTGGTGAATGGAGTTAAGAAGTGTGATATTAAGATATAATAAGGGAGGAAGAAGT

GGGGAATTTTGAAAAATGGGGGAAAAAATGATGGAGAGAAGAAGAGTGATTTAGAAGGCC

TTCGGGTTGTAAAAATCTTTTGTATGGGAAGAAAATGACAGTACCATACGAATAAGGACC

GGCTAATTACGTG

>8006 len=193 tot-seq=1

GTGATATTAAGATATAATAAGGGAGGAAGAAGTAGGGAATATTAGGAAATAGAAGAAAGT

ATGAAAGAGAAAAGAAGAGTGAGTGAAGAAGGTTTTAGGATAGTAAAAATATGTTGGTAG

AGAAGAACGTTGGTGAGAGTGGAAAGCTCATCAAGTGACGGTAACTACCCAGAAAGGGAC

GGCTAACTACGTG

>8007 len=193 tot-seq=1

CCCATCAGTCTGATCCAGAACTTAAGCCTTTGCCCACTGAGCTCCAGTTCTACATTTCTA

TGGCTTAAAAACATGTAGATATTCAAAATCCTAAATATGATGGAAGGAAGATCATCAGTA

AGCAGCTTATTCAAAGGTTATGGGGACTGAAGACAAACGGAAGAGTAAATTAAAGTGAAA

AAACATAGATGGG

>8008 len=193 tot-seq=1

CTGCCTTGAGGTCCTGGGGGCAGTTCCACGTGGGTGTGTCTGGCAGGACATGGCAGTCCC

TGGCACTCACTCTGAGAGGTGTCATTTCCTCCAATCCTTCTCCTGTCCCCTGCAGTCCCA

GCAGGGCAGAGGCTTCTAAGAGAGCGGAAGCACTAGACGTGTCCGCAAAGGGAAGCCTTG

TAAGCTTCAAGCC

>8009 len=193 tot-seq=1

CAGGAGGGCCAGGGGGTCCAGCATTTCCCTGGATGAGGATAGGAGGGGCTGTCAGACTCC

AGGGGGCTCTGGTGCATCATTGGGTCCTCAGTCAGCCCCACCATCCTTCTGGCAGCCCCC

ACCCAGCACCCCCAACCTAGAGCAGTGGACTCTGCTGCAGAGACTTACAGAGGGGCCAGG

AGGACCGACTCGG

>8010 len=193 tot-seq=1

AAGGGTAGTGTATGCAGAGGATTTTATTGGGTGATGGCTCTCAGCAACATGGGAGCTGGA

AAGGGGATTGTGTGGGAAGAAGGTGATCTTTCCCTGAAGCCGCACTGTCTGAAGTTAGCT

GCATCTATCCATAGTCACCAACGCTCAGTTGCTGCTTCTCTGCTCACTGCTCAGCCTCTT

GTATCCCCAACTC

>8011 len=193 tot-seq=1

AACATGTTACTGTAGTAGACCTACTTCGTAATGATTTAGGATTAAATGCCAATGATATAA

GAGTGGTAAAATATAGATATATAAATAAAATAGATACTTTTCAACGTTCTATTTTGCAAG

TCAGCAGCGAAATAAAAGGTACACTTGAAGATGATTGGCAAACACGATTGGGGGATATTA

TTTTATCTATGTT

>8012 len=193 tot-seq=1

GCAAGGTGAAACCACTAGGCAGTTACACTTATCTCAGAGAAGTGAAAATTCACTGTGCCC

CCCTAAATAATAGCCCCATAATGATGTACACATCCTAATCCACAGAATTTGGGAACACAT

TACTTCACATGGCAGAGGCTTTTTGCAGAGGTGATTAAATTAGGTGGATTAATGAAGCCA

AAAGCAAGAAATG

>8013 len=193 tot-seq=1

GACAGCCGAGGGTAGAAGCCACCGCACAGCATCTCTCCCCCACTCAGCAGCTCCAGCTGG

GACATCATCCTCCTGTCTCTCCTTTTCAGGCGCTTCGGGGGGTTCCCATTCAGGCACCTT

CTCCTCCTTGCTCCGCTCCCTTCGTTTCTTTCCCCAAACTTAGCATCTCCTTCAAAGAAG

CCCAGAGCCACGG

>8014 len=193 tot-seq=1

TCAAATGATCCATTGGCATCAGCCTCCCAAAGCGTCGGAATTACAAGCATGAGCCACTGC

GTCCAGCCCAGATGCTTAGCAGAGCATCCCATGTGACTGCTGTGGCAAGAACATATTTGG

GGACCACTGACCAACGCACAGGCTGTTCCCCAGCCTCGCAGTATCACAGGCCTGAGCCCC

GTCTCCCTTATGT

>8015 len=193 tot-seq=1

AATACGAAGTGCAGGGAAAGACGCCAAGCACAAGGTCACATATCGCCAGGCCACGTGTCG
CCAGGCCACGTGTCACCAGGCCACGTGTCATCAGGCACCACTGATGTGATGTGTCCTGAA
AGGCAAGCCCATGGAGATGGGAAGTGGAGGAGTGGCAGCGGGGGTGAGGAGGCTGGGGCG
GTGGGAATGACTG

>8016 len=194 tot-seq=1

CCAAGCAGAGTGTTTGCTCTGAGTGCTGAGGGTCTAAAACTGGGAATGGAGTCCATGCTG
ATTAGACCCCCTAAGCTGTGGATCATTATGCACCATGCACATCCTGATCCTGGGTAGCAG
CACCCTCTCTCAAGCCCCTACAAGGCTGGAGCACTACCTCTGTGGGGGAGCTTACACTCA
GCGATGTCCCCTGG

>8017 len=194 tot-seq=1

AAGTCCTGTAGACCGCAGAAACCCAGGATGGCTGCATGGAGAAGAGGAGGAATCACCATG
CCTCTCCCACTTCATTCCCCTAATTAAGGTCAGCTCAGAACCAGAAGGAACTTCTCCCTG
TGGAGGAAAGGTAAGTAGACGAATAATCTGCATAAGTGACGGTACCGAAAAAGCAAGCCA
CGGCTAACTACGTG

>8018 len=194 tot-seq=1

TTCCAGTGAGCCAAGATCACACCACTGCACTCCAGCCTAGGTGACACAGCGAGACTCTGT
CTCAAAAAAATAAAAATAAAATAAAATAAATAAATAAATAAATAAATAAATAAATATGCT
GAGAGGCTCACATACTTCTTGTCACAATAAAGACCTATAATCATATAGGAGACGGGGAAA
AAAAACTAAATCAT

>8019 len=194 tot-seq=1

TGTGACTGAGGGATGCCCACCCAGGGGATTACCTGGCTGGGGCTGAAGCAGAGGGTGGGG
CGGAGCACCCGAGGTACCACCGGCTAGGCCTGGGGCAGAGTGATCCAGTGGAAGGTTCTG
AATGGAAGAGAGATGGGCTCCTCTGCCTCCTGTAGCTGTCAGGGGCTGTCACAGGCAGCC
CTGCCCAGCCGGCC

>8020 len=194 tot-seq=1

GGAGACCGTTCTTCTTCCCGTGAGGAACATGCTCCAGGGTCCGGAGTCTACGGAAAAATA
GGAGGGTTTGAGAAAGTGAAACCCGTTCATTTTCTGTGGAGCAGAATCAGCAAATGCTGT
CTCGGGGGGCCCTGTGGGCCTTGGGAAGCATTTTGGACTAAACCAGAGGATAATGAGACA
CCACTGGAGGTTTA

>8021 len=194 tot-seq=1

GTCATTGGGGTCACTCCCAGCTGATGGCTCTTTCCTCAGCGAAGTGGAAGGTGAGGACCT

CTGCTGGGCGTGAAGGTGGCAGGGAAAGTCTGAGCAGAGTGAAGGAAGTCTGAAATGGGT

CCCATGAAGCAAGGCAGATTTCCTGAAGGGGCCGAGAAGCATCGTGGGCAGGCCGAGGCC

TCTGAAGGCTGCGG

>8022 len=194 tot-seq=1

GCAATCTGGGTGACCCGCTGATGACACTCACACCAGAAGCCCGTCTGTGCTGAAGCAAAT

CAGCGCCAGCACTGCCAGCCATTGGCATCTGTCGAAGGGTGTGTCTGTGTTGTCTCAGTC

TGGTGCCCACAAAAGCCAACCTTGAGATGATGACTCAAGGGCACATGCATGTTTTGAGGA

GGTGACGCCCATGA

>8023 len=194 tot-seq=1

CCTAAAGAAAGCTTAGGTCTGAAACAAGTTATGTTCATCTGCATGATTTACTCCATGTAG

ATTCCAGGCAGTTGCCAGACAGGAGCAGGAATTGCACTCAGTGCCAGCTGGCCCTGGGGT

CTGCAGTGAGGCAGAAATCAATTGAACTGGATTCTTGTAACCCTCTCTTTCAATGGGGCC

TGTCCCCAGCCAGA

>8024 len=195 tot-seq=1

ATCCAAACGGATAGATCTACAAAACCGTCCCAGACACACCTAAGGCTCAGACAACACTCC

AGTAGACCAGGCAATAAGACTGTAAGAGGCTCAGGGAGTTTGCAGTGAGATTATGACTCC

TCGGAATGTCACAAATGACACCCATAAAAGTCTCACCAACGTGACTGCACAAATAGGAGC

TGAACAAGGACACCA

>8025 len=195 tot-seq=1

ATAGATGCCCCTTTGCCCTGTTGCTGTTCTTGGGTGTTGCTGTAGAGCTACTCAACTATT

AGAGCAGTGGTTCTCAAACCTGTCTCTCTGTTTAAGATAAAAATAAACGGGGTGGAGAAG

ACAGGCTAAAGGAAGCCATAGGAAGATATTCCTGGTAAAAAGATAGCTGCAGCCTTTGAG

ATCCGCCCTTGATTT

>8026 len=195 tot-seq=1

GCCATCTGCTTCATGGCACTTGCTGGCCAGGAAAGTCAGTGACTCTCCATTTCCTCTTGA

CCAAATCTGAATTCCTTTTCCTAGCATGGGCGGTCTTGGGCTATTGGGACCCACCCTACC

CCGGCTATATTTTCCACAGCTGCTCAACACACACACCCTACCTTAGGTTGGGTCCTCTTC

GCTTTGCTTCCTGAA

>8027 len=195 tot-seq=1

GCATGGCTATAGGCTTATATTTTTCTAAGCCTACTCTAATAAGTGTTTTCAAATCCCTCC

TAAACCACCACCCACCAGAGGTAGTAGAAAGGCAAGGTTAATAGGGCAAAGGAGGATGTG

GACCTGTTTGGAAAGAGTTCTTTGGAGTGAATCCAATCTGCCTTGTCAGGATATCGGCAG

CTCAGTTCACACAAA

>8028 len=195 tot-seq=1

GTCTAGGGTGCAGGAGCTTATCCCGTTTAGCAGGAGCTTAGCGTTTCCAGGTAGAGGGAG

GGAGGATTACAAGGGGATGTTGAGCCTTACTGGATGGAGCACTTAGGCATTTGTATCTGA

CTGAACAGGAAATTGGGAGCTGTTAATGTGATTGAACAGGTGAGTGACTCAACAGGTGCT

CGCTTTTCGATGACT

>8029 len=195 tot-seq=1

TAGACCAGGAGTTAGGATAGCTCTCCAGGGCTGAATGCCCCGTCAGTTCTCTTGTCCTGG

CTCTGTGTGACCTTGGGCCAGTCACTTCCCCTCTCTGGGACTCAGGAGCAAAAGACAGAA

GCAAGACATACATGCCTTCTGCAAGCTCTGGACTATGCTTCTCCCTACAGCACATTGGAA

GCATGGAGGTCTCCG

>8030 len=195 tot-seq=1

CAGGACTGTTACTCCAGCAAGTTCTTGAAGCACTGGGACCACCTCACCCAGGTCAAGAAG

CCAGTCATCGCTGCTGTCAATGGCTATGCCGTGAGTGTTGCTGCCAAGAGTCTCCTCCTC

TCGTGGTTGAAGGGACCGTGCTGTGCATACACAGCATAACAGATTTTTATTTTGAAGAGG

CCTTTAGTTCCCAGG

>8031 len=195 tot-seq=1

ATAAGAGAGTGACTGGAGTTCAGACGTGTGCTCTTCCGATATAATAAGGGAGGAAGAAGG

AGAGAAAATTAAAAAATAAAGAAAAGGGGAGGTAGTGAAGAAAAATAAAAATAAAGGAAT

ATTTAGAGGAAATGTAATTGGAATGAGTCCACTTTAAATCCTTCCGCGAGGATCCATTGG

AGGGCAAGTCTGGTG

>8032 len=195 tot-seq=1

ATAAATAAAGAGATGTTCAACATTAGTGTAGGCCGGGCGTGGTGGCTCACGCCTGTAATC

CCAGCACTTTGGGAGGCCAAGGCGGGCGAATCGCCTGAGGTCAGGAGTTTGAGACTGGCC

TGACCAATATGGTAAAACCCCATCTCTACTAAAAATACAAAAATTAGCCAGGCATGGTGG

TGGGCACCTGTAGTC

>8033 len=195 tot-seq=1

CATGCTTCAGATTCTCCCATCCTTCAAGGCTCCATTCTCCATAGTCTGGTTCTGCCGGGG

TGTCCTCTGTAGCCCTGATCCTCAAACGTCATCGAGCCTCCTGAGCAATGCCAACCCGAT

GTCTCACTGATTCTCTTTCGCTCTTCTCTCATGTTTAGGAAGATCCCTCCTGCGGCAGAT

AGCCCCCTGCCCTCG

>8034 len=196 tot-seq=1

GGGCAGGAAATGGGGTTCGAGAGGCCTAAACAGGTCCTCAGCCACATAAAGTAACAGGCT

GTGACCTACTGAGGCCACAGCGACAGCCACAAGGGAAGAAGGCTGGGAAAACACTAATGG

GCAAGAAAAGATCCTAAACCAGTAACAAGGAGGTCCGGCAGCCCAAGACTCAGCTGCCCT

TCCTTCCTTAACAGCT

>8035 len=196 tot-seq=1

TATTTACACGCAATAGCAATATATGAGGAAACGCGTAACAGAATGCTTGGGGGAGAAAGA

GAGAATGCAAATGTTACTGGAAGCTCTAGGCACAACACCGGGTTTGTTGAAGACCTGTCT

GAGTTTTACAAGCCGGTTTACTCTGTCTGCTACCCCAGTATCATTCTGCTACCTCATATG

GGCTGTCTCAGAGTCT

>8036 len=196 tot-seq=1

AGTGGCAAGTGCTGAAGCAGATGGCTGTTGACAGCGTGAACCCAGGAGCTGCAGACGAGG

TTGCCCTGGAGACATGCTCAGCTTTCTCAGAATCTGAATGGAGAGCTACCCCGCTAATGG

ATGAAATCATCCGTTAAAGAACAGGTAAGGGGAAAGTCCACAGCATACAGCAATAAGGAG

TAAATGAGGCAGAAAA

>8037 len=196 tot-seq=1

AACATTAAAAAAAAAAGAAAGAAAAAGCATCCTCTACCCCTGGTTCAAGCAGGCATCTTG

GGAGACAGGCAGAGCTCTGTGGTGTGTGGTGGGCAAGCCAATCAAATAGAGAATTTAAGG

GAGCTATGGATATGGAAGGAGACTGGACTCAAACAGTACAAGACAGAACCTCACAATTGC

TGGGTTCTGTGGGGTT

>8038 len=196 tot-seq=1

CAAAGTGTTAACTCTGAGAATGAAGGGGCAAATAAAAAAGGACACTTGTAAGCAGACCCT

CCATTTGGGAGAACCTTAATTACAGGTTGTTAGTTGCAGTGGAAACCCAGGCTGGGTGGC

CCTCCAGCTCATTGTCTTGCCCCTTTTCTCTCTCCCTGGGTGGGTCGCATGGGTCAGAGA

TTTGGCCCCACCCAAC

>8039 len=196 tot-seq=1

GGTTGTGAGATAACAGGGAGGAGTGGGAACCAATTGGAAACCACGGGCCACCTGCCAGCT

TCCCCATCTAGGGGCGGGTCTGTGCCCTGGCCTCAGTTCTGATTTGTCTGAATGTCTTGG

AAGAGTGCTTTGATCTGCAAGATGAGGGAGGGCCAGAAGTGGCCATCAGACCCCTAGTCC

TGAACAGTAAGAGCTC

>8040 len=196 tot-seq=1

TAAATCCCATTTCTGCTTAAATCACTATGTCAGTTTGCTTTTGCCTACACCATATCACCT

AAAATCAAGTAGTGTAAAACAATCACCATTTATCTACCATGGGCCATTATTCTGGGCTGG

GCTTGGCTGGGGCTGCCTGATGTAGGATGATCTTAGCTGGGATGGCTCATTTCTGCCCCA

TGTAGCCTGTCACCCT

>8041 len=196 tot-seq=1

GGGAATATGAGGCTGGAGAGGAGTCCGACGAGATGGAGAGGGTGGGGGAAACAAGACTGT

GGCCCAGGAGGGCCCCGTGAGGACCACTCCACCCCAGAAGTTGGAATGAGATTTTATGGG

GCTCAAAGTCAATTGAATGAATGAATGAATTTTGTCCCATCTGAAAATGCAAAAACTGAG

CTCCCCCTGCCTGGCA

>8042 len=196 tot-seq=1

AAAGGAGGAAAAGGCTTCCAAGTACAAGTCTCTATGTCTACATGTGCATGTAAAGAAAGC

GATGGCAGAGGAAGGACAACGTTGAGGGTTAGGGCAAGGGACAAAGACTGACATATGCGT

GATCTCTTAGGGGAAAGGAGTAGAGCAATTTGGAACTGAGTTTTCCCTGGCCCAACCATC

AGGAATTCAATTAGTG

>8043 len=196 tot-seq=1

TTCAGACGGGGGCGCGGCCGAGAGCCGACGGGAGGCAGCAGGGAGGAAGAGGGGCCAAGG

GACGGAAGGCGGAACCAGCCAAGGAGCGGGCAGGAGGACGGCCCGAGGGGTTGTAAACTG

CTTTTGTATGGGGATAAAGTTAGGGACGTGTCCCTATTTGCAGGTACCATACGAATAAGT

ACCGGCTAATTCCGTG

>8044 len=196 tot-seq=1

GGGGGGACGGGAGGTCAGACGGGGGCGCGGCCGAGAGAAGAAGGGAGGCAGAAGGGGGGA

AGAGGGAGAAAGGGGGGAAAAAAGGAAGAAGCGACGCAGCGGGCGGGAGGAAGGCCGGCG

GGGGGGAAACCGCGGGGGAGGGGGAGCAAGCCTTCGGGTGAGTGGACCTTTCGAATAAGC

GCCGGCTAACGACGTG

>8045 len=196 tot-seq=1

GGGGATAGGGTGTGGCCAGGAATGTGGAGTGGGCCCAGGGTCCCAGGGGCTGGGATCTCT

CTCCTTGTCCATGGCACATGCCTAGGCTTGGTCAGCATGTTGATGTATCTACGGAGCTCA

GCTGTGTACTGGGCCATCTGCTCCGGTGTGGTATTGTCCCCTGGGTACAATGGCTCCAAC

GGGGCTCCCCGGGCAC

>8046 len=196 tot-seq=1

CCAGGCTCAGCAGCTGGACGAGAGTGCAAGGATTCAAATCCAAGGAGGGGTGAATTCAGG

GCCTGTTCTGAGCCTGTACTCTCTGCTCTCTATCAGCTACCTCTGGAGGCTGCCTTAAAG

CAGCTTTCCCCTCTCCTTTTCTTCGCAACCCTAGGAGGAAGGTAACGAGTTAAAATCAGC

TGGCATCGGAGGCCCT

>8047 len=196 tot-seq=1

AGCAGCAGTGTCCATGTATGCACATGCACCAGTAATTGCAGTGTAATGGAATCTACCTGC

ATGTATATGGGGGTGGCAGTGCAGTGGCACAGCAGAGTTAGCATGCACACTTGGGTACTG

GTGGCAGTGGGGTGGTGGAGACCACATGTGTCCACTTGTCCAGTGATGGCATGGCAGTGA

GGTCTGTGCATATGCA

>8048 len=196 tot-seq=1

TGAGGAATATTGGTCAATGGACGAGAGTCTGAACCAGCCAAGTAGCGTGAAGGATGACTG

CCCTATGGGTTGTAAACTTCTTTTATATGGGAATAAAACGTTCCACGTGTGGAATTTTGT

ATGTACCATATGAATAAGGATCGGCTAACTCCGTGCCAGCAGCGTTAGATCGGAAGAGCG

TCGTGTAGGGAGAGAG

>8049 len=196 tot-seq=1

CACCATGTTAGCCAGGATGGTCTTGATCTCCTGACCTTGTGATCCACCCATCTCGGCCTC

CCGAAGTGCTGGGATTACAGGCGTGAGCCACCGCGCCCGGCCTGAGAATCACCCCATTTT

ACGGTTGGGAGAATCGAGGCTCAGTGGAGCAGGTGTACTCTCCCAGGTGACACAGCAAGA

GACCCGGGTTTTAAGG

>8050 len=196 tot-seq=1

TAAGGAAGATAGCTGAAATACGTCAGCTTGTGAGAGGGTCAAGGAGAGACATGAAGTGGA

AGGGGATAGAATGCGTTGGAGGCACGGCAGTGCCACGTGGGTGTCTGGAAGGGACTTGCC

GAGCTGGCAGGTGAAGACACAACTGCAAGAAATGAGTGAGTGAGCCATGCGTTCACCAAG

GGAAGAGCCTCCAGCA

>8051 len=197 tot-seq=1

TAGCCATCCTTTGTACAAAAAACAAAAAACAAAAACCCCCTTGACCTTTGAGAGAAAACC

CCTAAGACTACTCGTATTGAGGCGATGTGGCTGATGCCAACAAGAACTTCACAAGGGCCC

TGACAGGGGAGACCCTCACCCTTGAGGTGGAGTCTATCACTGAGACTGTCCGGACCCAGA

CCCAAGACCCTGTCTGA

>8052 len=197 tot-seq=1

CCGGGACCCTCTCTTAAAAACCAGAAGCAGCAAGGAAATGGTACAAACGCTGAGAGTGTG

GCTGAGCTGTGGCGTGGGTAAGGCTCTGGACCCAACCCCTAGCACTGAACAAGAAAAGAC

AACTGAGATCTGGGCCGCAGCGACTGCCTGTCTTCAATGGAAGCACAGTCAGCCCTGACC

TTTTCACCTTTTCAGTG

>8053 len=197 tot-seq=1

GGGTTTCTAGTGTTCATCTTTGGGAGTTACTACACTTACCCATTGCCTCTTCCCACTTCC

CAGAAGTGCATCCCATTAGTTAGTTCTGAAGTTCATGTGTACAGTCCCTTCACCGCAAGT

GTCACCAAGCCACCTACAACTGGATATGACACTTGTGCTCCCTGTCCTGTTAAGTTACCC

AGAGTTTGTTTCTAGAG

>8054 len=197 tot-seq=1

TGATACGTTTGAAGACACGTTGTTGCCCGTATTCTGGAGGTTCCTGGTGTTCATACCAGC

ATTCTGTGAACAGCTCAGAAACCAAAAAGGAAATCAACCAGGCTAGAGTGCACAAGTCCT

CACGAGGACCTCTAGTGGCTACCCAGTAAGCCCTGGACTTACTGTGGAAAGTGCTCCACC

CTGGCAAGGGATTTCAG

>8055 len=197 tot-seq=1

CCTCACCTGGGAGCTCATTCAGTCAGTTCAGTCGCTCAGTCATTTCTGATTCTTTGCAAC

CCCATGAATTGCAGCACGCTAAGCTTCCCTGTCCATCACCAACTCCCGGAGCTTGCTCAA

ACTCATGTCCATCGAGTTGGTGATGCCATCCAGCCATCTCATCCTCTGTCATCCCCTTCT

CCCGCCTTCAACCTTTC

>8056 len=197 tot-seq=1

TGGGGAATTTTGCACAATGGGCGCAAGCCTGATGCAGCGACGCCGCGTGAGGGATGAAGG

CCTTCGGGTTGTAAACCTTTTTCGCTCATGGTCAAGCCGCAGCTTTGGGTTGTGGTGAGG

GTAGTGGGTAAAGAAGCGCCGGCTAACTACGTGCCAGAGCCGCGGTAATCCCAAGATCGG

AAGAGCGTCGTGTATGG

>8057 len=197 tot-seq=1

TGGGGAATATTGCACAATGGGCGCAAGCCTGATGCAGCGACGCCGCGTGCGGGATGACGG

CCTTCGGGTTGTAAACCGCTTTTGATCGGGAGCAAGCCTTCGGATCGTAAAGCTCTGTTG

TTAAGGAAGAACGAGTGTGAGAATGGAAAGTTCATGCTGTGACGGTACTTAACCAGAAAG

GGACGGCTAACTACGTG

>8058 len=197 tot-seq=1

TTCAGATGATCAGTGTAGCCTTACAAATTGTGGCATTGAATCTCTGCAGTCATAGGCGTC

CTTCAGCGCTGGTACAACAAACCCAACAGATGGCCCCCAAGCCGTGCCTAGGATGGGAGG

GAACCTGGGAGATCAACCTAATTCCCAGGAAGTGGTGTGGGCTCCAGGGCATAAGAGCCC

CATGACATACACAGGAG

>8059 len=197 tot-seq=1

AAGTTTTTTGTTGTTGTTGTTGTTGTTGTTTTGTTTTTGTTTGTTTTTCTCTTGTTCAAG

GCTAATTCCTTGATTCCATTTTTGTTGTTGTCTTCTGTTTGTTTGTTTGTTTTTTGGTTT

GATCATGCAATGGCATGATCTCAGCTCACTGCAACCTCCACCTCCCAGGTTCAAGCAATT

CTCCTGCCCCAGCCTCC

>8060 len=197 tot-seq=1

GGGCATACGCACACACATGCACGCTCCCATCAGAGAATGAAAGGAACGTGATTCTCCACC

CTGATAACACCCAGGGGCTCTGCCAGCTTTGAGATGCTCTCATTTGGCACTTAGGAAGGT

TTTCAGACCACCAAGGCATTCTTTTAAAGTAAACAGACAGTAAACAGACAGTAAAGGTGG

CCCGGTGAGTGAGTTGG

>8061 len=197 tot-seq=1

TCAGACTTGCGTCCATTGACCAATATTCCTCACTGCTGCCTCCCGTAGGAGATCGGAAGA

GCACACGTCTGAACTCCAGTCAAGTAGCGTGCAGGATGACGGCCCTATGGGTTGTAAACT

GCTTTTATATGGGGATAAAGTGGGGAACGTGTTCCCTTTTGCAGGTACCATATGAATAAG

GACCGGCTAATTCCGTG

>8062 len=197 tot-seq=1

TAGGGAAGTTTTCACAATGGGCGCAAGCCTTATGAAGCAGTGCCGCGTGAACGATGAAGG

TCTTATAGATTGTAAAGTTCTTTTATATGGGAAGAAACGCTAAGATAGGAAATGATTTTA

GTTTGACTGTACCATTTGAATAAGTATCGGCTAACTATGTGCCAGCAGCCCGCGGTAATC

ATAAGATCGGAATAGGG

>8063 len=197 tot-seq=1

CAAAGCATGCGAAGAAGGCGTAGGGTCCCCAGCAGAAGCAGAATGCCAGGACCATCACCA

CCACCATGCGCGTCACTTCCTTCTCTGCCTTCTGGGTGGATTCAGACTCTTTCTGCTGCT

TTGCCACCTAAGGGAGACAGGGATAGTGAGTCGGGGGTTGGAGGGCAGCAAGAGGCAGGT

GACCCAGGCATAGGGAG

>8064 len=197 tot-seq=1

TTGCTGCAGAGGAGTGAGGAGAGCTGCCTGGCAACGCAACGCATCGCTGTGCATAAACTT

TGTTTACAGCCAAGGCTGGAGTAAAGGAACAGATGGCAAGAAAATCGGATTTAAAGAAAG

GAAGGATGTGCTTGCATCTGGGAGCTCGGTAAATCTCTTGGCTTCTGGTGTGCGTACAAG

GGTGACGTCTAGCTACC

>8065 len=198 tot-seq=1

GGAGTTCAGACGTGTGCTCTTCCGATTCCTACGGAGGCAGCAGTGAGGAATATTGGTCAA

TGGGCGAGAGCCTGAACCAGCCAAGTAGCGTGCAGGACGACGGCCCTATGGGTTGTAAAC

TGCTTTTATGTGGGGATAAAGTGCGTGACGTGTCATGCATTGCCGGTACCCAATGAATAA

GGACCGGCTAATTCCGTG

>8066 len=198 tot-seq=1

TAGGGAATATTGGGCAATGGAGGCAACTCTGACCCAGCCATGCCGCGTGCAGGATGACGG

CCTTCTGGGTTGTAAACTGCTTTTATCAGGGAAGAAAACGCTCCTGCGGGGGTAACTGAC

GGTACCTGAGGAATAAGCACCGGCTAACTCCGTGCCAGCAGCCGCGTAATCCCAAGATCG

GAAGAGCGTCGTGTATAG

>8067 len=198 tot-seq=1

ACAAGTTAGGGAAGAATGACTTTCAAACCATTCGGGGTTTTGAGTGAAGCCACTGGGAGA

AGGGTTTAGTGGGAAGAGACACTGGGGAGGACTGTGTGGTTTGAGATGGTGGCTGGATCC

ATGCACTTGCATCTGGCTTTTCTTCCTAAGGAGACAGTGTGTTTTCTGAAAGCCGCTCCT

GTCTTGGCAACTGGGTAC

>8068 len=198 tot-seq=1

TGAGGAATATTGGTCAATGGGCGAGAGCCTGAACCAGCCAAGTAGCGTGCAGGATGATGG

CCCTATGGGTTGTAAACTGCTTTTATATGGGGATAAAGTGGGGAACGTGTTCCCTTTTGC

AGGTACCATATGAATAAGGACCGGCTAATTCCGTGCCAGCAGCCGCGAATATTCAGATCG

GAAGAGCGTCGTGTTGAG

>8069 len=198 tot-seq=1

GCAGCGAGGGAGGAGGAGAGGCCCCAGGTGAACGGAGGGGCTTGTCCAGGCCGGCAGCAT

CACCAGAGCCCAGGGCAGGGTCAGCAGAGCTGGCCGTAGGGCCCTCCTCTCAGCCAGGAC

CAAGGACAGCAGGTGAGCCGGGAGCAGAGCAGCGAGGGTGAGTGTGGCAGCAGGACAGAA

GGGTGGAAGCCAAGGAGC

>8070 len=198 tot-seq=1

CTCCCAAATGGCCTGTTCCAGACACCTAGTAACTGGCAGACACAACCAGATGGAACCATT

GCCAGGCCCTCTCCTGAGAGCAGGAGAAGGAGGGAGTGCAAACCCCTCCTGGCTGAAGTT

TGCTCTTGGCTTGATTTTTTCCAAGTCCAGCCCTGCCTTGAGTGGGCTGGGGCTCGGGCA

CCCACCCAGTTTCTACCT

>8071 len=198 tot-seq=1

GGGAGAGAAGGAGAAAGAAGGAAGAACAAGGCATCTGGAAATTAGTTCTTTCTGGCAAGA

TTTTAGTTTCACTCTGAAAACTCTTTTTTAGAGCTCAAATGGCTTACTCTCCTTAAAGCT

GGGTCTCCTTAAAGAATCCATCCTTGTCCAGGGCATTATAGGGCAGCGCAAGTGTCAGCT

CTCCACAATGGAGTCAGC

>8072 len=198 tot-seq=1

GACAGCCCTGATGAAGCTCGGCTACTGCCCCCACTTCACAGAGGGTGACACCAAGGCTCA

GGACGGCAAAGGCACTTATCTGAGGTCCTAAAGCCAGGAAGCGGCAGAGCCGGAGTTGTT

GTCAGGCCATGTGGAATGTGCTGTCATCCTTAAAACCCAATAGTCCTAGGCTGGGTGTGG

TGGCTCACACCTGTAATC

>8073 len=198 tot-seq=1

CCCTTGGTTCCTTCTCCCCAATTCCTGTGACCAGGAGCTCTCCTGCCCAGGGGGTAAAGA

ACTGGAAGAGGAAGAGGAGGCCTCCCCCTCAGCCTCAGCCCCGTTCATGGAGGGAGATGA

AAGCTTGTCATGTTCCCTTTGAAAGCGCTGGCAGAGCCTCAGTAGGCCCTGATTGCTGGG

CGCTGCCTCCCCTGGGAT

>8074 len=199 tot-seq=1

GAAGACTCCAGTTGTGGGCAAGAGAGCCACACTGATCACCAAGGAGGGCATACAGGTGTG

ACATTGATGAGACCTTTCAGATCTTGGTTGCTAGGGGGACAAGAACTCTAGGTGTTACGT

GTCACACACTTTGCTCAAATCACTCAAAACAGTGACAGACAGAAGACTTCCTCCCAGATG

CAGCACCCTCAGCTCCCAC

>8075 len=199 tot-seq=1

GGGGCTTGGGGACCAATGCCTGTGGTGGGAGGTGTGGGTCTTGGGTTCCTGGCTCGGGAA

GCATTTGGGAGCTTTCCTGAGATTTAGAGAATGCCTCCTCCTTGTCTCCTTTCTTCTTCC

TCTGGTCATTTCTTTTCCCAATCAGCCAAGCCCACCAGTTTCCACCTTGCTTCTCCACAG

GTCTTTGGCTGGTTGACTG

>8076 len=199 tot-seq=1

AGGTGTGGGGATCAGATCTCTGGATTCACCTTGTCATGTTCTGCCTTTGCACAGCCTCCT

CGGGGGGGTCTCTAAGCATGGCTAGAGTTAATAACTTTATACTGGGAGTCCTCGAACAAA

AGTGATTCCTACCTATCGTTGGAAAGGGAGTGCATTCCAGCAGGGCAGGTGAAGAGCATG

AAGTCTCAGGTACACGGAC

>8077 len=199 tot-seq=1

TTGGCAAATGTGCCTGAGGGGGTGGGGAGGTCATACCTTCAAGCACTGTGCTGACCCATG

AGGGAGGAAGGAGGAAACTCAGCTGTTATTATCTTTTCTCCTGAACTTCCTGATGTTTCT

GGAAAAGGAGGCCCTGGGAGGGGCAGATCTCTCAGTTGCTAGGCTCCAAATAAATAACTG

AATAATTAAGGACAAAGAC

>8078 len=199 tot-seq=1

GCCGCTCGATATCATACTCACCAGTGATTCAAACATGATGCTCTCCCCTAATGCCGGGCA

CGATCATACTCAACTCATCGGAACAGTAAAGTACAAAATAGCAGCACTTTGCGCCGATCG

GCTATTTTTCATCATGCTGTTGAACCCTACTCCATACCCAATTCTTTTAGCTTACGCGTC

AGGGTATTACGTCCCCATC

>8079 len=199 tot-seq=1

CAGACGTGTGCTCTTCAGATCTAATAAGGGAGGAAGAAGTAGGGAATATTACGCAATGGG

AGAAAGCATGACGGAGCAACGCCGCGTGAGTGATGAAGGTCTTCGGATCGTAAAACTCTG

TTATTAGGGAAGAACAGATGTGTAAGTAACTGTGCACATCTTGACGGTACCTAATCAGAA

AGCCACGGCTAACTACGTG

>8080 len=199 tot-seq=1

AGCAGCAGACTGATCGCCGCACCTGGATGTAGGCTACGGGGTGCAGCACAGCCCAGGCAG

CAAGCCCAGACTCCTGGGTGCTGGCAGCGCTGAGCGGAGCCTCAGCCCTACAGGCACACA

GCCCTGCAGGGCTCCTGTGCCATAGGGTGGCAGGCAGAAGCTACCGCGAAAAGGCCAAGG

GCCTGACCTGTCCACTGGT

>8081 len=199 tot-seq=1

GTCCTGGGGAAACCCAGCGTTCTAACCTGCTTCATCTCATCACACCGGCTCATTTGGGGT

CAGCACTGCTGTGTGTCCCCCCTCCTGAGAGCAAGGAGTGAGGCTTCCCCAGGCTGGTCT

GTTCCCCGGGACAGTGCAGCCATCACAGCTGCAGCCCACATTCCCAGAGAATATTTTCAA

AGCACGTTGCCTAGGGGAG

>8082 len=199 tot-seq=1

TGAGGAATATTGGTCAATGGTCGAGAGACTGAACCAGCCAAGTCGCGTGAAGGAAGACGG

CTCTATGAGTTGTAAACTTCTTTTGTACAGGAGTAAAGTGAGACACGCGTGTTTTATTGC

AAGTACTGTACGAATAAGCATCGGCTAACTCCGTGCCAGCAGCCGCGGTATCTCGAGATC

GGAAGAGCGTCGTGTAGGG

>8083 len=199 tot-seq=1

TACTAACTGTACTCACTACGCCCTGGTCACTTTATAGCACAAGTCCGAGGCAAGGAAAGA

AGTTTTACAAACAAAACTGGTCAACTGTTCTATCTTCTTACTCCTGGAAAACAATAAAGG

TCCACTTTCAAACCATTTGGACGCACTTGCTCCTTCTAAAGGGTTAGGGAGTCCCGGCAG

ATAGCGTACCCCTCCCCGC

>8084 len=199 tot-seq=1

GTCTGTCTTAAAAGTTCGGGGCTTAACCCCGTGATGGGATGGAAACTGCCAACCTAGAGT

ATCGGAGAGGAAAGTGGAATTCCTAGTGTAGCGGTGAAATGCGTAGATATTAGGAAGAAC

ACCAGTGGCGAAGGCGACTTTCTGGACGAAAACTGACGCTGAGGCGCGAAAGCCAGGGGA

GCGAACGGGATTAGATACC

>8085 len=199 tot-seq=1

ACGAGATTCATGTGTGACTGGAGTTCAGACGTGTGCTCTTCAGATATAATAAGGGAGGAA

GAAGTGGGGAATATTGAAAAATGGGGGAAAAAATGATGAAGAGAAGAAGAGTGAGAGAAG

AAGGATTTAGAGTAGTAAAGATATGTCCTATGAGAAGATAATGACGGTATCATAGGAGGA

AGCCCTGGCTAAATACGTG

>8086 len=199 tot-seq=1

GCCACCTCCTGGGGCTGACACTGGCCGGGGCCTGCATTTCTGAGGGCGGGGCGTGCTTTG

ACATCCTGAAAGGGTTGCAAAACCCATCACAGAGAACCCCAAGAAGGGAGACTCTGCCAC

CGAGACCCCGTGTGCCCCGCAGGGTCTGCGCCGTGTGCTCTCTGGCTCTTCATGGAAGCC

TTTCCCAGCCCTGGTCGGG

>8087 len=200 tot-seq=1

CCCCACAGGCAGAATGCTACCCTTGGGAACCATGGAGACACATTACTATGAGCACACCCA

GTAGATACAACCACATACCGATTTCCATTCCTTATATAAAAACAAACAAGAATAAGGGGA

AGTGGCCAAGCGTCCTTAACAGGGGGGTTGATAAACTGTGGGTACAAAACCGCAGGGACA

GTGACTTGTATGTGAGAAGT

>8088 len=200 tot-seq=1

GAGGCAAGGTCTGTTAAGGATTCACTCATCAAGGATGAGAACTGTAAACGATTCTGGTTG

TCATCCTGACTGGAGTAGGGGGTGGGGTGACTAGAGAGTTGGCAAAGCACACATACTCTT

GTGTATCTGTGACTTGCTTCCAGGGAACAGTGGTAGTGTGTCAGTGGACTGAGATCAGGA

TAGCTCTTCTGAGTCACAAT

>8089 len=200 tot-seq=1

CCAGCTTCCTGAATTTCCATGTTTTGGTGTCTGCCTGACCTATTAACACACATACCCCTG

AGGCAGACAGCACATGTTGATGCATATGTGTGCATCAGAGAGCACATGTCAGGTTGCAGA

ATCTGACTGAGACAGCTTGGAGTCTTAACATTTCTGAACCTGGGGACAGCTATGAGCCGT

GAGCTGTGGACGAGGGATGC

>8090 len=200 tot-seq=1

CATCTTGATACAAGGTTAGATGGCCAGGGAACACAGAGAGGGAAAAGCTGATTTGTTTTG

GTGGGTGGAAGTCTTGCAGGCGTCCCGGAAGAGGTCATATTCCCTATCAACAAGATTCCT

GACATGTATATAAAATAGCCTCCCCGCAGCACCAGCACATATCTGAAACCCTTCAAACTT

TGGCTATCATGACAAAGCTC

>8091 len=200 tot-seq=1

AGATCAAGGAAGAATGGTGATTCTTACTTTCTTTTGGCTCTATGGTAAGTATCTGGTTGT

AAAGTCTGAGGGCATCCTAAGGGAAAATATTGTGTCCAGTGTAACTGTGGTTTTGATCCT

ATAGTTTTTTCTTTCTTTTTTTTTCTTCAGTGCGTCAGAATCTAGCTATCAAGTTGAAAT

AGGTTAATCTGATCTTATCA

>8092 len=200 tot-seq=1

GGCCTAAAGTGTAGTAGGAGGGTACTCTGGGGGGCTAGAGGGTATGGGTCAGCGGTGGTC

TGGCGACCAGGGGATGGTGGTGAGGAAAAGCAACCTGAAGGGATCCAGAGGGTTCGGCAG

TCATATGCAGGGTGTCGGGTGGCTGCCTTGTGGGAGAAGGTGGGTTTCCGTGAGCAAGGG

AGCCTGACATACAGAAAAAG

>8093 len=200 tot-seq=1

AGCCCTTCTCAACTTGACTGTACATAAGACACACTTGAGAACCTTTAAAAACGACCTCTG

ACCCTGTAACCACCTCAAATTAAATTAGAATCTCGGGTCTCAAGCATCAGTAATTTGTGA

AATCTCCCCAAGTGATTCTAATGTGCTCCCAGGGAGGCAAACTGCTGCTAGAGCCCAGCT

CCTCAAAGTGCAGCCTGGGA

>8094 len=200 tot-seq=1

GTTGCTCTGGGGGTTGCACCCCATTCCCCAGTGCACTTGTGGGGGCTGTGGGGTCCCCAG

CAGGAGCATGCCCTGTGACTCCTGCAGGGCAATCAGAGTCATCCCCTACACTGCTCAGAC

GTGGAGATGGGCAGGGCCTTGCCTGAGGGTGGAGGAGCAGAATAGACAGAGACCTGGTGT

AGCCTGGGACCTGCCCTCTG

>8095 len=200 tot-seq=1

TTTAGCTTGTGATGTGATCATAAAGTGAGTCACATCTGCCAGGCGCAGTGGCTCACGCCT

GAAATCCCAGCACTTTGGGAGGCTGAGGTGGGTGGATCATGAGGTCAGGAGATCAAGACC

ATCCTGGCTAACACGGTGAAAGCCCATCTCTACCAAAACTACAAAAAATTAGCTGGGCAT

GGTGGCAGACACCTGTAGTC

>8096 len=200 tot-seq=1

TAGGGAATTTTGCACAATGGACGCAAGCCTGATGAAGCAATGCCGCGTGAACGATGAAGG

TCTTATAGTTTGTAAAGTTCTTTTATATGGGAAGAAACGCTAAGATAGGAAATGATTTTA

GTTTGACTGTAGCATTTGGATAAGTATCGGCTAACTATGTGCCACAGCCGCGGTAATTTG

AAGATCGGAAGAGCGTCGTG

>8097 len=200 tot-seq=1

AGTCCTGCCCACCCTGGTGCCAGCCTGCCCTTTCAGCACCCTCTCCCCACGCTAGCTCTG

ATGCCACAGTGTTTTTGTACCTGCTGCTTCCTCTGCCATAATGCTCTTCCCTTTCCGTCT

TGCCTGCCTGTCCTTCAGGACGCTGCTCAAATGTTTCTCTTCTGTAAAGTTTCCCTTGAC

TCCCAAGGGCAGAACACAAC

>8098 len=200 tot-seq=1

CCAAGGTAACACGGCCAGTAGATGAGAAGCCAGGATTTGAAGCTGTCTGGTCCCAGGGTT

CAGGCCAGGCAGAGGGAGGACCCGGCAAGGGAAGATGCGCCCCTGCCCTGGAGTCTGGGA

ATGACACGCTTCTCTTGCCTCTCAGGGAGAACCTCAATACTCAAGTCATTCCAGCAGCAA

CACCCTCTCCAGCAACGCAT

>8099 len=201 tot-seq=1

TCTATGGAAGATAGATGTGTTTAATCCACTCTGAAAAAAATAATAGCCCTTGTGGAATGC

CACTTCCTCGGCGCAAGACATTCTCGTCTGGTATTTCTGTCTCTATTAGTGTGGCTACAA

ACCCCTAGACTGAGCTGTCGCGGAGCTCCTACTGTGGCGAGTCCCCCTTGTCACCACAGC

AAGGCAAGGAGCTCAGCCCCT

>8100 len=201 tot-seq=1

CAGTGCCCTTCACCATTGGTGTGAGGGGTGTCTGTAAGAGCTGGGAAGCAGAACAGACAA

TGGGAGGGAGGAGCTGCTCTAAGAGCTATGACAAAGTAGAAGTCTGAAGTGGTCCATGGC

CTGCAAGCAAGGGTTCCTGATGACGGGACCAGGCTCTCCCACACTGCCTTTTACTTCTCC

AGATTCCTGGATCACAAAACG

>8101 len=201 tot-seq=1

TGAGGAATATTGGTCAATGGGCGAGAGCCTGAACCAGCCAAGTAGCGTGCAGGAAGACGG

CCCTATGGGTTGTAAACTGCTTTTATGTGGGGATAAGGTGCGCGACGTGTCGTGCATTGC

AGGTACCACATGAATAAGGACCAGCTAATTCCGTGCCAGCAGCGCGGTAATCCCCAGATC

GGAAGAGCGTCGTGTTGAGGA

>8102 len=201 tot-seq=1

AACAGGAAATTAGATATAAAACCCACAACTTTCTAAGGAGGGTAGGCCCTGTTAACAGGA
GATTTTTTTGTGGGGGCAGTGGGGTTAGATACAAAGTCATGAACCTCAATATAATTAATG
GCCTGTCACAGTTCTGAGAGTTTTCAGTAGTGGGTGGAGTGTACAAATTTGAAGGTAGAA
GCTGGAAGACACTTAAACTTC
>8103 len=201 tot-seq=1
GAGACACTGAGACGGGGCCCCAGCTCCAGGTCAAGTCCCATGTGTGATCTGTCAGGCTCG
ACCTTAGGGGATGACAAGGGGGTGCCTGGCAGGTCACACAGGGGCCAATGCCCTACTCAC
AGCAGACCTCGTGCCCAAACCGTGCTTCCCAGTTCCTTGGAGGGGCCACGGTCCAGGACT
CAGAAGGCAGAACCGAGGCCA
>8104 len=201 tot-seq=1
TGACTGAAGTGGCCGAGCACATGGGAAGTGTGGCTGGCAACGGATGCATGCTGTCCAGGG
GCGTCGTATCACTTCTGCCATGGGGCTCCTCTCTGAGGGTGTTCTCCAGCTCACCAGGCT
CAGAGGGCAGAGAAGAACTCTCCAGGCAGAGCCAAGAGCATGCGCCAGGTTGGGCAGGGA
ATGGAAGGCAGCCCAGGTACG
>8105 len=201 tot-seq=1
GACCCTTTCTGGAATGAGGGCCTTATGACCCTCAAACAGGCTAGAGTTCGGCTATGGGAA
GGTGAAAGGAGAGCGGGAGTAGGTCAGAGAGAGAGATTCTGTTTACTCAAGAGAGTACGC
AGAAGGGTGATGGGAGTTATGAGCCAGGAACCATGGGTGGAAACCTATTGTGTGTATATA
TCTATGTCATCGATATCTATT
>8106 len=201 tot-seq=1
CGTATGGTGAGAAAACAGGAACAGAGCTGGGCAGCTCTGCTGGGTCATCTTGGGCCTTAG
CAGGAGGTTGGAGGGACAGTGACGGCTTAGGCCACATCCCCTACAGGCACCCTTGGGCCT
AGTAGGCAGAGGGGCTACGGCCATGTATTGGTAGCACAAGGCGATGGTCAGAAGGTCAAC
CATGTGACCCTCATCTCTTTT
>8107 len=201 tot-seq=1
AGGTAGCCGGTGGGGAGCCTTCCGCCCCGGATGGCCGCGTGGGCCACCGGCAGAAAAAGG
CAGCACAGAGCCATGGCGGGCAGAAACCACAGGTGGTAATGTCCCTTAACAAGGTCAGAA
AAAAATTGATATGTATTGCCAAAATTCCCGGCGGCCAGCCGGGAGCCTAAGGCATAGACC
AGCGACCAGAAGAAGAACAGG

>8108 len=201 tot-seq=1

TGGGGAATATTGCACAATGGGCGCAAGCCTGATGCAGCCATGTCGCGTGTATGAAGAAGG

CCTTCGGGTTGTAAAGTACTTTCAGCGAGGAGGAAGGCGTTAAGGTTAATAACCTTGGCG

ATTGACGTTACTCGCAGAAGAAGCACCGGCTAACTCCGTGCCAGCAGCCGCCGGTAATCA

AAAGATCGGAAGAGCGTCGTG

>8109 len=202 tot-seq=1

GTCCGGGTGACGCCAGGGAGCCAGGGGCGGCGAGGTCACGCGTGAAGACCACGCCAGCCC

AGCCCACGTGTGCGCCAGATTTAAAAGTTGGTGGCGCGCGGAGATGGTCACTTTGGTGTC

TGAGCTCAGCAAGAGGAGGCGCACAGGACACGCTGAGGGACAGCTAAAACACCCGCACAA

CACAGGGTACCAGGTCCCCAGT

>8110 len=202 tot-seq=1

TGAGGAATATTGGTCAATGGGCGAGAGCCTGAACCAGCCAAGTAGCGTGCAGGAAGACGG

CCCTATGGGTTGTAAACTGCTTTTATGTGGGGATAAAGTGCGTGACGTGTCATGCATTGC

AGGTACCACATGAATAAGGACAGGGGTAGTAACTGACCTTTGTTTGACGGTAATCAATTA

GAAAGTCACGGCTAACTACGTG

>8111 len=202 tot-seq=1

CGAGGAATATTAGTCAATGGACGGAAGTCTGAACCAGCCAAGTAGCGTGCAGGATGACGG

CTCTATGGGCTGTAAACTGCTTTTGTATGGGGATAAAGTTAGGGACGTGTCCCTATTTGC

AGGTACCATACGAATAAGGACCGGCTAATTCCGTGCAAGCAGCCGCGGTAATAAAAAGAT

CGGAAGAGCGTCGTGTAGGGAA

>8112 len=202 tot-seq=1

TGGGGAATCTTCCGCAATGGACGCAAGTCTGAACCAGCCAAGTAGCGTGCAGGATGACGG

CCCTATGGGTTGTAAACTGCTTTTATATGGGGATAAAGTGGGGAACGTGTTCCCTTTTGC

AGGTACCATATGAGTAAGGACCGGGGTAGTAACTGACCTTTGTTTGACGGTAATCAATTA

GAAAGTCACGGCTAACTACGTG

>8113 len=202 tot-seq=1

TGGGGAATATTGCACAATGGGCGCAAGCCTGATGCAGCAACGCCGCGTGAACGATGAAGG

TCTTCGGATCGTAAAGTTCTGTTGCAGGGGAAGGTAATGACGGTACCCTGGGAGGAAGCC

CCGGCTAACTACGTGCCAGCAGCCGCGGTATGGACAGATCGGAAGAGCGTCGTGTAGGAA

AGAGTGTCTTGTGGGGAATATT

>8114 len=202 tot-seq=1

CTGGAGTTCAGACGGGGGCGCGGCCGAGAGCCGACGGGAGGCAGCAGGGAGGAAGAGGGG

GCAAGGGGCGAGAGGCGGAACCAGCCAAGGAGCGGGCAGGAGGACGGCCCGAGGGGTTGT

AAACTGCTTTTATATGGGAATAAAGTGAGGGACGTGTCCCTTATTGCATGTACCATACGA

ATAAGGACCGGCTAATTCCGTG

>8115 len=202 tot-seq=1

AAAGCCCTGGGGTGTGCTGAGTTTTGTGTTGGCCGGGCCACTGAGATGATGCAACTCCAC

TCTGCATTGTGCAATGAGATGAAAGGGCCACCAGGGAGGGCGCACACACAGGGGCTTTGC

ATCAGCCACACATTGAACTGGGTCACAATCAGTTATACACACAGACAGTCTAAGATTCAG

AGGAAAAGGAAGACATAAAACT

>8116 len=202 tot-seq=1

CATTTCCTCTCGTTTTTCTTTTCTATTTATTGGGACATAATTGACATATAGTGAAATGCA

CAGATCTCGAGTGTATAGTTGGGTGAGTTTTGATAAATACAGACACTTGTATAAGCCATT

CCCTTGGTATAGAACGTTTGCGTCACCCCAAAAGGCTCCCCCACCCCCTTCCCAGCCAAT

TCCCCACTTGCTCTCCTCCCTC

>8117 len=202 tot-seq=1

TTCTTAACCTTGAGCCAAAGGATCAATTGATGGGCTTCAGAGGGACTGAGTCCCTTGAAT

TCGGTGCAAAAAAATTTTTATTCCAGTGGAAAGGCACATAGATTGAGAATGGCCGCAAGA

GGCTCTAAGTCCCCTGAGGGTAGCACAATGTCTTACCCATTTGTGTCCCCAGAGCCTAAC

ACAGGACTTGGCAGAAACAAGG

>8118 len=202 tot-seq=1

GATCCGGAAGGTAGTGTCCACATAGATATTTCTACTCCTTGGGACTCCTGAAGCCACTAT

ATGCTGAGCACTGCTAGCCCCAAAATCCAGGGAAATGTTTTAGAGGTGAGAGGATCAATT

GGGAGTGAGGGGAGTCAAATCCTAGAACCTTTTCCTGCCTTGGAGAGCTGTCCAGGTATT

CTATCTTTCTCTGTCCTCCCTA

>8119 len=202 tot-seq=1

CAAAGACCCAGGCAAGAAAAATGCCAGGCCCAGGGAAAAGCTCCGGGGGGATGGCGACAT

GATGATAATCAGCTTCCAGAGGATGTTCTCCCAGAAGGACCTGGAGATCGAGGAGCGCCA

CAAGGGGCACAAGGAGAGGATGAAGCAGATGGAGAAGCTGAGGCACCAGTCCAGAGACCC

CAACCTCAAGGAGAGGGCGAAG

>8120 len=202 tot-seq=1

AGGTGCCACACACGTGTCAACTGAAAATCCAGCAGAGACCCATGCCAGATGTCAGGGAAG

CCCAAGGAGCGATGGAGGGAGAGGAGGAGGACTTCCAGGGGTGGCGAATTGTGCTTTTCT

TGAAGGATGAACAGTTCATCAAGGGGTGGGGAAAGGCATCCTGGCAAAGTCCCAGGTGCC

CCCCAAAAGTCTCCGTTACTGC

>8121 len=202 tot-seq=1

GAGGGGTGAAGCTGCTAGCTCAAGGTCACCAGCCCCTTGGGGGCAGAGCTGGAACTTGAA

CCCAGGTTTTCTGATACCAACTATAGCGTGCTGTGCCTCTTCCTCATTCACAACACAGGC

ACACAGAATGTAAGGGGCACCAGGCTGGCCTCTTCACCCTGGGCCATTAAACTAGGCTGT

CCTCACTCCCATCCCCATGAGG

>8122 len=202 tot-seq=1

CCCCCCGCCTCGGCCTCCCAGAGTGCTAGGATTACAGGCGTGAGCCACAGTGCCCGGCCG

ATACATGGCTCTTAATATGTAACAGGCATTGTTATCGAGCATTTGACGGTGCTCATTTCA

GACAAAGAAAACGAGGCTCAGAGAAATTAACTAACTTTCTTAAATCACAGAGCCACTAAG

TAATGAAGTGTGAATTCAAACC

>8123 len=203 tot-seq=1

GCGAGGGCTGCAGTGAGCACAGGGCAGCACCTTGGAGGTGCGGTGCGGGTTTCCAGGGCA

TCCGTGGAGGGCAGCAGCAGGCACATGCAAGGAGCAAGCGTGCTGTGTGTGTATCCATGA

TTCATATGCTCACCCCCAGGATCTGACAGGTCTTCAGACCGACCAGGTCTCTAATCTCCT

CACAGCCTTGCCAGGCAGGCGGA

>8124 len=203 tot-seq=1

GGGCAAATCTCCACCAAATTGGATTCACCTGTATCCTGGTGCACCTGCGCAGCTCTCAAG

ATGTTTGTGGCTTATATGAGGAAGTCAGGTGCAAGTCATATGACTTAACTGCAGTCCCTG

GCGCCTTTGGGACTGCCGCCACACCTGCTCCTAACATCTCCCCCTTTTTCTTTTCTGGCA

AAGAGAATGTCTGTAGATAGCCC

>8125 len=203 tot-seq=1

GAGAAAAGGAAGTAAAGTTTACCTATGGGTTACGCAGCCGTGCCGGACCCGGCAGGGGCA

CTTGCCAAGGGCCTGGCATTTTCTATAGACTGGTCTTAAATCATGTGATTTTGTCCTAAG

CTTGGGTAGGATAAAAATCACACAGGAGAGCACAGACGCAGTGTGAGGTAACCTGCCAGA

AGCCACTTAGCTCAGGAGAGACC

>8126 len=203 tot-seq=1

ACGCACATGCCTATGGCAACAACATGGTGGAGTGAGGCTGTTGGTGAGTTTACATTGGTA

AAGACCTGTCTGCTAAAGCTCTCCAATGGTTAGAGAGTTCTGCCAGTGAACGAGCTATGG

TGGTGGTCCCCAAGATGTGCCATGATTGAGCATCCAAGTCTGTGTTGCAAGTGGATACAG

AGAAGCAGGAACCTTAGGAGAGA

>8127 len=203 tot-seq=1

AAAGTTCCAGCCAGAAAGGTACAAACTTTGGAAAGCTGGGAAGGACAACACAGTTATTGA

CCATACTCTGCCCACGCCAGAAGCAGCTGAGTTTCTTAAGGAGAGTGAACTGCCTCCAAG

AGCTGGCAACGAGGAGGAGTGCCCAGAGGAGGACATGGAAGGGGTGGAGGATGGAGAGGA

AGGAGACCTGAAGACAAGGTAAC

>8128 len=203 tot-seq=1

CTACCCTTCTCTCTGGGGACTTCATCCCAGGGAGAAATCAGAGTTCTGTCCATAGAACTC

CAGCTGGAGTTTCTAAAATTCTGATGGGGAGGCCCTGTCCAGTGAGGATGGATGGATTTT

GGTCTCACTTAAAGAAGCAGCCTGGCCACGATCAGTCACAACAGCTGTACTGTGTTATAG

GGGACTCCTCCTGGTCCCTGGTG

>8129 len=203 tot-seq=1

TTTTTGCAATATCCAGATGAACACACTGAGGCACAGAGATGCAAACTGACTTGTCTAGGA

CCACACAGCTTGTGAGTGACAAAGCTGAGATTTTAACCCAGGCCTGTTAAAGCTTCATAT

AAGTTGTGGGCATTTTGGTGCAGGAGGCAGAATGCATTAATGTGCCTGTCATCTTCCTTC

CCTAGGGCTGAAAGTCCTCAGCC

>8130 len=203 tot-seq=1

CTCAAACTCTCTTAACGGATGGGAAACTGATGCTCCGACGACAGGGTAGAGACTCCTCAG

CGTCAACCCGCTTTCGGCGGCAGGACAGAAACTGGGCCTGGGCCTGGGCCTGAGATGGTT

CCTTCCTACACCCTTCTCCAGGGTACCGTGTCCACCTCTAAGCCTCTGGACCCTCTGACA

TGTAACCTGTGTCCCCTCCCCAA

>8131 len=203 tot-seq=1

CCAGCAGGTAAGGCTGGCCATGGATGTAGAAAGGAGAGCACTTTTCAGAGAGGGTTTCTG

AGATGGACTTGGGACCACTGGGATGGAAAGGGCTTCAGAGGTTCATTAAGTGCCCAAGGA

AAGCAAGGCTCCCTTAACACATTCTGCAGGGACAAAATCAGCCTGGGGGTCCCTGCAGGT

TAAGCATCAATTTACCATATGAG

>8132 len=203 tot-seq=1

AGAAGGGTATGATTTCAATGGTAAAGGCAAAGGGGAACTGTGTTCTGGGCTGAAGGAGCA

GTGTAAGCAAAGATACAGAAGTGTGAAAACTGGGGGCAGGTGCATAGAATGAGCAGCCCA

ACATTCCTGGAGTTAAATGTGGTATAAAAGAGAGAGGGGCTCCAGAGAAGGCTTTCAATG

CTGGGGCAGGAATCCACCTCCCT

>8133 len=203 tot-seq=1

CTTTAATCATCAGGGGTGCACAGGGCGGTAGGCACTAGACAACTGCATACACCCCCAACC

CCCAGGATGTTTAACCTGAACCAAAGAACATCCTCGGGGAGCCTAGGAGACCCTCTGAAA

GCTTGTACCCTCAGCCACGCGACTCCCGGCAAGGACCAAGGTGAAGTCCGAAGCTGAAGC

CAAACCAGCCCCAAAGGCTAAAC

>8134 len=203 tot-seq=1

AAAGTGTAAATAGGAATAGAAAAGGCTTGGAAGGACGGGAGAAGGGGGAGGTATTCTGGT

TTATGAGTACATAAATAAGTACTTGGAAGCCTAGAAGTGCCTGGAATAGCTGATGTGAGT

TGAAGCATTGAGGGCATTAAAGAATAATAATTTGAGCCAGAGTCAAACTAGGCTTGATTC

TTCAGGCCTGATATTGTTTCATG

>8135 len=203 tot-seq=1

CACCTTCCCTGGCACCAGGTCCCATCCTTCTCTGCAAAATAGCTCTTTCTGTAGGATGTT

ACCTTTCCTGCAACAGTGGACCTCAGCCTCAGCTATGCAGCTTTGAAAAGTGCTTATGCT

TGGGAACCACCCCAGAATTTTGGATTTAATTGATCTAGGACGAGGCCCAGATAGGGCAAG

CTTAAATGGCTCTCCTGCTGATT

>8136 len=203 tot-seq=1

GCGTCTGCTGTGAAAGGCCGGGGCTTAACCCCGTGTATTGCAGTGGGTACGGGCAGACTA

GAGTGCAGTAGGGGAGACTGGAATTCCTGGTGTAGCGGTGGAATGCGCAGATATCAGGAG

GAACACCGATGGCGAAGGCAGGTCTCTGGGCTGTAACTGACGCTGAGAAGCGAAAGCATG

GGGAGCGAACAGGATTAGAAACC

>8137 len=203 tot-seq=1

CAATCTCTGTTTTGAAACACAGGGAGCCCCGCTGTGCGGAGATCCAGGCACCTTTAATTC

AGCAAGAGGGGCTGAAAAACCCTCCACATTTTGGAAGAAGGATCCAAACCCCTGTTTTAG

GTAAAGAGAGCCCCCAACGTTTTGGGGGATGGTCTGAAGGCCGCCATTTGTAGGGACACA

GAGCCCTGGTGTGCAGGTCTGGG

>8138 len=203 tot-seq=1

TGCAAGTAACTACAGGCAAAGATTCAATGTGAGGAGTGGCTGGGGCAGTAAGATAGGTCC

AGAGGAAATGCTCTGAATTTAGACTTCCGGGCTCATCCTGCAGGCTTGCTGGAGTGAGTC

CCTGTGTTTCTATTTGCCCTCTGAGTTTTCAGGGCTGAAATTGTAGGAGGGTGTGTACCC

ATCTTGTAGGCCATTCTGTCTAC

>8139 len=203 tot-seq=1

TTTATGAAACACAAAATGTAAAAATAATTGTATAGGTAAATATTTTATAAGTAAGTCTAT

TTTATACATTTACGTTTATGTTTAGATTGGTATTTATTATTATTTACATTTACCTATAAA

ATAAAACATGGTTTAGATGTAAATTTTGTAGGTAAATTTTTAGGTAAATATAAGAGCCTA

CATGATGGTCCAGACTCCACCAT

>8140 len=204 tot-seq=1

CAAGCCTAGCCGAACAACCATGACAAAAATATAAAACAACCAAACAAACAGAAAAACAGG

AAAACCCAGGTTCAGCTACAGACTCTGTCTCACGCATGTGTATAGCCTTATTTCCTTATA

CACATGCATATACACGTATATGTAACACACACAATTCATATTTTAAACTATAACCTAAAT

AGTAAATACTTCAGCACAGTCATG

>8141 len=204 tot-seq=1

GGGAGGAGGGATGGGAGAGGGGAACCTAATCTGCTATTAGATAAGGGAAAAGGACTGAAG

CCCTAAGGGCCAGCAGAAAGAATGTAACAGGCAACCTCGGGAGGTAGGAGGCTGGGAGCC

ACCCTCCAGAATTCATCAGAGATTTGGAAGGTGAGAGACTCTCAGGACCCAAAGGGAGGG

ACCTTAAATGAAATGCCTGACAGT

>8142 len=204 tot-seq=1

GGGCAAATCTCCACCAAATTGGATTCACCTGTATCCTGGTACACCTGCGCAGCTCTCAAG

ATGTTTGTGTCTTATATGAGGAAGTCAGGTGCAAGTCATATGACTTAGCTGCAGTACCTG

GCACCTTTGGGACTGCCGCCACACCTGCTCCTCACATCTCCCCCTTTTTTCTTTCTTGGC

AGAGAGAATGTCTGTAGATAGCCC

>8143 len=204 tot-seq=1

ATATGGTACCGTGTTTCTGGAAGAAAAAAAGAGGAAGGGACTTTGTAACATTATCTGGCT

CAGAGAAGAATGGCTTCTATAGAGCAACAGCAAAAGCCACAGAGCTGGAGCAGATGCTGC

TTCACTGGGTGCTCACTCTCTGTTTGCCCAGAAAAGGCAACCGTAAAGGCAACCGTAAAG

GCAACCATAAAGGGTCTTCCCTCC

>8144 len=204 tot-seq=1

TGAGGCAGAGGCATCGAAGGTTGGGTCTATGCCTCTGTAGGGCATGATTTTCTACTAGCA

GCCAGAAGTCTCTGCCCAATGTTGCTAGCTGTTGGAGTGGTGGAGGACAGAAGAGGGGGG

AAGGAAGCACTGGAAGTAGAGTGTTCCATATCCTAGGAATATGTTTGTAGCACACAGCAC

TGCTAGCCTTTACTTTTTAAGTCC

>8145 len=204 tot-seq=1

GAGCTAGGGCCCAACTACAGATTAAAGAAGGAAGCAGGACTCACAGTCTCTCCAGCTTTG

GCAGGTGCTGGAAGGATTCAGGGTCCAGCGTTTCTATCTGATTAAAGTGCAGGTACCTAG

GGGATAAAACAGCCACACAATGTGAATGACTTACCGACCTTTGCCAACCCAGATTTTGGG

CTCCAAAGTTTTCCCGCACTTCGT

>8146 len=204 tot-seq=1

GCTCAGGAGGACTGAAGGGCCACATCAAGTGCTGCTCAGAGATCACATGGGGTGCGGATG

GAAAGAGACTTAATATCAGCTACACTGGAGACATTAGCCAAGTGTGGGCTCAGTTCAGAA

ACTTGGGAGACCCAGTTAGAAGGAGCTGAAAAGCTCAAGAGAATTTCGGAGAGAGACAGG

AAATGCAATTAGTTAAGAAGTTTA

>8147 len=204 tot-seq=1

TCTGGTGGGCAGAGGAAGGGAGATCACAGGTCCATGGAAAGAGCCAGTCGTTATCTATGT

AAAACAACCAAGTCCATGTATACAGCCTCTCCATGGAACCTTCCTGTCCAGCTCCCTGCC

TCATTCACATACTCATACTCATCTCCTTTTCTAGATGCACCCACACCATCCACACCCACA

TTATCCCCAAGTGCATCATCCTCA

>8148 len=204 tot-seq=1

TGGGGAATATTGCGCAATGGGGGAAACCCTGACGCGGCGACGCCGCGTGCGGGATGAAGG

CCTTCGGGTTGTAAAACTCTGTTGTAAGAGAAGAACAAATTGTAGAGTAACTGCTACAGT

CTTGACGGTATCTTACCAGAAAGCCACGGCTAATTACGTGCCAGCAGCCGGGGTATTAAA

AGATCGGAAGAGCGTCGTGTTGGG

>8149 len=204 tot-seq=1

AAGCGTCCCAGGAGGAAGCGCCCAGCCATAAATGGAGAGAGGAGATTCGGTAAGACCAAG

TAATGGTCCTTCAGTGGGAGAGGCAGTCAGCTGGAATTCACAGGCCAGCTTCCAGATCGA

TACTTCTGTGCTGATTTGCTGAAGAGGCTTTTATGAAGTTTAAGCAGAGTTCATTGTTAA

ATACTCTTATGTACAAAGGAGCTG

>8150 len=204 tot-seq=1

CCCCAGAGGAAACGGAAGTCGTATCTGTCCGGACGGAAGCAGGAAGCGGGAGCGTAGGGC

CACGCCTGCGGCGCTGCTGGTTGAGGCTGTGTGGGTGGGGGACGGGCCGAGGCGATGGCG

GAGAAGTTTGACCACCTAGAGGAGCACCTGGAGAAGTTCGTGGAGAACATTCGGCAGCTC

GGCATCATCGTCAGTGACTTCCAG

>8151 len=204 tot-seq=1

TTAGTAAAGGTACAGTGTGGCCAGATGCGGGGTGTTTGACCCTCAAAGTGGCGCGTGTGA

CTATCTCGCGCGTTCCGGGGAGGTATAGGAAGCGCGCACACCCCTCGAAGCCACGGAACA

AGACACGGCTCAACACTGCACACCTTCGAAGATCGGCGGCAGCAGAGCGGAGCCCGACAG

GACTGGGCCATGCGGACCCAGCAG

>8152 len=204 tot-seq=1

GAGTGGGGGCCACTTTTGAGGAGGGAGTTGTTCCAAGACTATGCTGATGCCTGAACAACT

TGGAGGGAGGAAGGGCAGGCATTGGGACTGATTGTCATGAGAGGGGTTGCTCTGGGCCGG

CAGCCAACCAGAACATGAGGCCACTGACCTCAAGAACAAGAAGACTGTGCTGGAAGGAGC

AAAGGTGCCCAGAGAGAAGCCACA

>8153 len=204 tot-seq=1

TGAGGAATATTGGTCAATGGACGAGAGTCTGAACCAGCCAAGTAGCGTGAAGGATGACTG

CCCTATGGGTTGTAAACTTCTTTTATATGGGAATAAAGTATTCCACGTGTGGGATTTTGT

ATGTACCATATGAATAAGGATCGGCTAACTCCGTGCCAGAGCCGCGGTGATAGAAAGATC

GGAAGAGCGTCGTGTAGGAAAGAG

>8154 len=205 tot-seq=1

TGGGGAATTTTGGACAATGGGGGAAACCCTGATCCAGCCATCCCGCGTGTATGATGAAGG

CCTTCGGGTTGTAAAGTACTTTTGGCAGAGAAGAAAAGGTACCTCCTAATACGAGGTACT

GCTGACGGTATCTGCAGAATAAGCACCGGCTAACTACGTGCCAGCAGCGGTAATACCGAG

ATCGGAAGAGCGTCGTGTTGGGGAA

>8155 len=205 tot-seq=1

TGGGGAATATTGCACAATGGGCGCAAGCCTGATGCAGCCATGCCGCGTGTATGAAGAAGG

CCTTCGGGTTGCAAAGTACTTTCAGCGGGGAGGAAGGCGATAAGGTTAATAACCTTGTCG

ATTGACGTTACCCGCAGAAGAAGCACCGGCTAACTCCGTGCCAGCAGCCGGGTAATTCGG

AGATCGGAAGAGCGTCGTGTGGGGA

>8156 len=205 tot-seq=1

GCCTGAGGGACCCACAGAGATGCGAGGGTCCACCGGGCTGGAGGGAGGGGAGGCGACAGG

TCGAAGATGAGGTGAGGCAGCGAGCCCAGGCCTGCAGTTCCAGGAAGGACCCCTGCAGCA

CCTGCAACCAGGCAAGCTGGGATCCACCCACTGTCACTAGGAGGGAAAAGAGGAACCAGA

AAGTTCTCATACAATGGAAAACTTC

>8157 len=205 tot-seq=1

GGCATACGAGATCAAGTGGTGACTGGAGTTCAGACGTGTGTTCTTCCGATCTCCTACGGG

AGGTAGCAGGGGGGAATATTGCACAAGGGGGGAAACCCGGATGCAGCGACGCCGCGTGGG

CGACGAAGCGCTCAGGCGCGGAAAGCCCTGTCAGCAGGGAAGAAAAAAGACAGTACCTGA

CCAAGAAGCCCCGGCTAACTACGTG

>8158 len=205 tot-seq=1

CTCTAGAAGCTGGACAAGAAAGGGAACAGATTCTTTCTATAAGGGTCATCAGCAAGGGAA

CCTAATCTCCCCCAAGAGCCTCCAGAAGACACACAGCCTTGCCAATCTTGATTTTGGCCC

CATAAACCATTTCAGATTTCTAACCTCCAGAACTATGAGAGAATACATTTGCATTGTTAA

GCCACTGAGTTTGTGGTAATTTGTT

>8159 len=205 tot-seq=1

TGAGGAATATTGGTCAATGGGCGTGAGCCTGAACCAGCCAAGTAGCGTGCAGGAAGACGG

CCCTATGGGTTGCAAACTGCTTTTGTGCGGGGATAAAGGAGTCCACGTGTGGATTTTTGC

AGGTACCGCATGAATAAGGACCGGCTAATTCCGTGCCAGCAGCCGCGTAATGTCTAGATC

GGAAGAGCGTCGTGTAGGGAAAGAG

>8160 len=205 tot-seq=1

TGAGGAATATTGGTCAATGGGCGAGAGCCTGAACCAGCCAAGTAGCGTGCAGGACGACGG

CCCTATGGGTTGTAAACTGCTTTTATATGGGGATAAAGTGGGGAACGTGTTCCCTTTTGC

AGGTACCATATGAATAAGGACCGGCTAATTCCGTGCCAGCAGCGCGGTAATCACTAGATC

GGAAGAGCGTCGTGTAGGGAACGAG

>8161 len=205 tot-seq=1

CAGTGACTGGAGTTCAGACGTGTGCTCTTCCGACTCCTCGGGACAGCAGTGAGGAATATT

GGTCAATGGGCGAGAGCCTGAACCAGCCAAGTCGCGTGAAGGAAGACTGCCCGCAAGGGT

TGTAAACTGCTTTTGTATGGGATTAAAGTCACCTACGTGTAGGTGTTTGCAGTTACCATA

CGAATAAGCATCGGCTAACTCCGTG

>8162 len=205 tot-seq=1

GAGGGAGGATCTAGACACAAGTGTTCTCTTAGGCCAAAAACAAATTTGTTCTCAATGAAA

GTGGAAGGATGGAAGCAAGGAAGTAGGCTATGGAACAGAAATGAGCAAACTTTTTTTTTT

TTAACAGCAATGTTCATCGATTATAAGGGCACCTTTTATGCAGTGGTTAAGAATGCTGGG

GAGTCACACTGCCTGGGATTACAGT

>8163 len=206 tot-seq=1

TAGGGAATTTTCGTCAATGGGCGCAAGCCTGAACGAGCGATGCCGCGTGAGTGAAGAAGG

CCTTCGGGTCGTAAAGCTCTGTTGCGGGGGAAAAAAGGCAGCATCAGGAAATGGGTGCTG

ACTGATGGTGCCCCGCCAGAAAGTCACGGCTAACTACGTGCCAGCAGCCGCCGGTAATGC

CAAGATCGGAAGAGCGTCGTGTATAG

>8164 len=206 tot-seq=1

AATGGGAAAAGTAAGATGTGGAAGTAAGCAGAACTTATTGAGGAAACGTTGTGATGGCCC

CTCTGGTGCGTTCCATGTGGACATCTATTCTGTTTCATCCTACCCATACCCCAGGAAGGG

GAAGACATCAGTTGGAGATGGCCACCAAATCTCTGTGCCAGCGACATGGAGAGAGTGTCT

GGGAGGAGAGTCAGGATTCAAAAGAC

>8165 len=206 tot-seq=1

TGGGGGATGGGCTGAGTATGACCAAAGTACATCCATGTGTGACAATGCCATGAAGGGACC

CAACACTTAATAAATTAATGTATGCTAATAAAAGTTAAAGTGAAGTAAACAGTAAACTTA

GGTTGCAGGTTATAAAGAGTCGAGGGAACAACAGTAAAATCCGGAGTATTCCGAGACAGA

CATAGAGCCTAAAAGGACCTAACAAG

>8166 len=206 tot-seq=1

TATATACAACACTGATCATTTTTTTAAAATCCTGGCAATATCCCAAGCTCTGGTACCTCT

AGATTGGACTACCTACTTAGAAGATTATTTACCCAAGTTTTGTTTTTAATCTAAAAAGGA

ATGGGCCTTTTATTTATAAATATATATGTTGGCTTTAGTTTCTGTAACCTGCCCTTTCAA

AGAAAATAATGAGCGGAGAGGTATTG

>8167 len=206 tot-seq=1

GGTGGATGTAATAACCTGCAAAGCCTACAAGGTTGATGAGAAGAAAGGCGGAAGAGAAAC

GAAGATAATTCTTACGATCACCTTTTATATATAGATATATGCTGAATCCAATGGGCACAG

GCACCCAACAAAGATAGAAAAAGCCTGCCAAGAGGTCGGCGACAGTGTGATGGTGAAGTG

CAAAAAACTCGTTGGGTGTCACGGTG

>8168 len=206 tot-seq=1

GCAACAAGAGCAATTGGATGATGCTTTGTGGTTTAATGAAGGACAGGACTTTCACCTACA

TTAACTCTTTTGGATCACCTGCTCCACCTACCATAAGAGGCTGATTCTCACAACAGCCTC

TTATGGTAGGTGGAGCAGGTGATACAATCTTTATTTTACATAAAGATTACAGGGCTCAGA

GAACTGAGGGACCTACCCAAGGTTGC

>8169 len=206 tot-seq=1

CAACTACAGCGGAAAGAGAGGCAAATGCATTGAGAGGCAGCTCCCAGCCATAAGGTGTCA

CTGCTGTTTGAAAGTTGATTCAATCAATATTAATAACATGATGCCATCACCGAAGATTTA

CTGATACATTGAACACTTACTGTGGGCCAAAGCTGTAGCCAAGTGCTTTATAAGCGTTAT

CTAATTTTATCCCCAGCAGCCGCGGT

>8170 len=206 tot-seq=1

AAACAAACAAACAAACAAACAAACGAACAAACAAAAATCCAGTTAAGTATATGTTCAGGC

TAGGCAGTAGCTATTTTGAAAACTCAGGACTAAAGGAATTCCTGGGAGTATGTATAGGAT

GATTAGAGTTGCAGTTTCCATTGTCATGCAGTAGAACATCTTGCGGGGGTAGGTACACAA

TGGTGAGCCATCAGCAGGCAACATTG

>8171 len=206 tot-seq=1

ATCATGGGGCTTCTCAGCCTCCATATTGCATGAGCCAATTCCTTATTTTATATATGTTTT

GATGATTCTGTTTCTCTGGAGAGCCCTGACTACACAGGGGTAGAAAATAATGGAGGAAAC

CCACTGAATAGCATGTTCAAAGAAGGCCTCTCTGGGTTGGTGCAAAGCATTAAGTGAATA

AGGTAGATTAAAGAGTGAAAGAGGCC

>8172 len=206 tot-seq=1

GGAGCAAAGCAGGAGAGCCTGGGCGAGCGCACGAAGGGTGAGGCTCCCATCCCGGGGTCT

CCTCCGCCTCTGTGCCTGGTCACGGTATGAGGTATTACCCACAGAAAACGGGGGCCAGAA

CCTGACCTCTAGCCTGCCCCACCCTGGGCACAGCTTCTCACCACAGGAAAACTCCACCAC

GCGCTCAGCTTCCACCCCAGCACAGG

>8173 len=207 tot-seq=1

TGGGGAATATTGGACAATGGGGGGAACCCTGATCCAGCCACGCCGCGTGTGTGAAGAAGG

CCTTATGGTTGTAAAGCACTTTAAGCGAGGAGGAGGCTACTGAGACTAATACTCTTGGAT

AGTGGACGTTACTCGCAGAATAAGCACCGGCTAACTCTGTGCCAGCAGCCGCGGAATACT

CAGATCGGAAGAGCGTCGTGTAGGGAA

>8174 len=207 tot-seq=1

TGTTGTTGTTGTTGCTAAGCAAACAGACAATTTTACGGAGAGAATGAATAAGATCCATTT

CTTCAGAGCCACTTGTAGGATAAGGCAATGGGGGTACAATCCCTAACCCTATGCCAAAGG

TGGAGGTGGAGAAAGGGTCTAGTTCCCTGGAGGAACTAGAATGGTACAGTGACTCAGGAG

GCAGTGGGGGAGGAAATGACGAACTTG

>8175 len=207 tot-seq=1

AAGAGATAAAAATGTGAATGGAGGTAAGAAGTGGGAGAGGAAGATATAATAAGGGAGGAA

GAAGGGGGGAAGAGGGAGAAATGGGGGAAAAAATGAAGAAGAGAAGAAGAGTGAGGGATG

AAGGAATTCGGGTTGTAAACCGCTTTTGATTGGGAGCAAGCCTTTTGGGTGAGTGTACCT

TTCGAATAAGCGCCGGCTAACTACGTG

>8176 len=207 tot-seq=1

CTGGAGTTCAGACGTGTGCTCTTCCGATCTCCTACGGGAGGCAGCTGTATGTCTTCTTCC

ACAATGGACGCAAGTCTGATGGAGCAACGCCGCGTGAGTGAAGAAGGTCTTCGGATCGTA

AAACTCTGTTGTTAGAGAAGACCACGAGTGAGAGTAACTGTTCATTCGATGACGGTATCT

AACCAGCAAGTCACGGCTAACTACGTG

>8177 len=207 tot-seq=1

CAGGTGATAATCAAATAAGGCTGAGAACCAGGCAGTATGGTGGTTGGAAAAGAGGCAGAG

AGGGTCAGATGATTCTCAGGGGGTCTGGGCATGTTTGAGAATGGAGAAAAAAATAAATTA

AAATATTGATGACCACCAAATGAGACCAGTAGGAAATGGCTGGAAACATTTCAAGAAACT

GGAAATGAAAGGTCACCCAATCAAAGC

>8178 len=207 tot-seq=1

GTGACTGGAGTTCGGACGTGTGCTCTTCCGATCTCCTACGGGAGGCAGCATTGAGGAATA

TTGGTCAATGGGCGAGAGCCTGAACCAGCCAAGTCGCGTGAAGGAAGACTGCCCGCAAGG

GTTGTAAACTGCTTTTATGCGGGGATAAAGGAGTCCACGTGTGGATTTTTGCAGGTACCG

CATGAATAAGGACCGGCTAATTCCGTG

>8179 len=207 tot-seq=1

GATGCGATAGTGACTGGAGTTCAGACGTGTGCTCTTCCGATCAGGCAGCAGTGAGGAATA

TTGGTCAATGGGCGAGAGCCTGAACCAGCCAAGTCGCGTGAAGGAAGACTGCCCGCAAGG

GTTGTAAACTTCTTTTGTATGGGATTAAAGTCACCTACGTGTAGGTGTTTGCAGTTACCA

TACGAATAAGCATCGGCTAACTCCGTG

>8180 len=207 tot-seq=1

AAGAGATGGACATTTTCCATCTCTTCTCATTCACACACACTTCTTTTCATACAAACACAC

TTCTTTTCACACACACACACACACACACACACACACACCTCTTCTCATTCTATACTGCCC

AATGCACCAAATGGACGGCAGTGGCCCCACTAATGAGAGCCCACAGAACACTAAGCCCAA

GTGCTCCACAAGTTCTGAGTAATGCCT

>8181 len=208 tot-seq=1

TAAGAGGACAAAGGCATTCAGCTTATACGATGAGAATGTCCCCCAACGGGATCTGATGTT

TGAACATATTGTCCTAGTTAGGGTAGAATTATCACCTATATCCTTGCTGGAGAAAGTGTC

CCTTGGGGTGGGATTTGAGGTCTCAAACACTCATGCCATCCCCAGTTTTTTCTGTCAGTT

TCCCACGTGAAGCACAAGATACGAGGTC

>8182 len=208 tot-seq=1

GCTGTATAGACTTTGATGGTGACTGGAGACATCTACCCAAAGGGACCCATGGACTCTGGA

AGCCCAGCCAGGCCTTATCTGTACCTTGGAGAATGCAGCCTTGTAGGTTCTGGGCAGGCC

CTGTTTATCCGGAAGCAGAGGGAAAAAAATCTCACCCAACTGGTTTGCCTGGCCCCTGGT

CCTGGGCTGTGCCTGGACGGGCATGCAC

>8183 len=208 tot-seq=1

GGAAAGATGTCATCTCACCATTGCCTACACAACCGTGTGTGCGTGTGTGTGTGTGTGTGT

GTGTGTGTGTGTGATGTGTGCACACACATCCACAGGCATTCACACCTGGGATGCTCTATG

CTCTATGCAGGCCTACTACCTGCCCACAGTCCTGGGTGTGCAGGACTCAATCTGCCATCT

TTGTTCTGGTTTGGATGGAGTTCTACCC

>8184 len=208 tot-seq=1

GATCGCCAGCGTATCTTTTTTGCTCATTCCCAGGGCGCTACAGGACTGCCACTGACCTTC

TGGGATCGCACGAATTGCACCGTAAAACAGCTGCGTGGTATACGCCGCACTGTTCAGCGA

TAACGCAATCAGCGCACATAACCACGGTTCTGACAACAAATGCCACAGTGCCGGATACTC

CTGCAAAGTCGGAAACTGGCCCGGCCCG

>8185 len=208 tot-seq=1

TGGGGAATATTGGACAATGGGGGGAACCCTGATCCAGCCATGCCGCGTGTGTGAAGAAGG

CCTTATGGTTGTAAAGCACTTTAAGCGAGGAGGAGGCTACTGAGACTAATACTCTTGGAT

AGTGGACGTTACTCGCAGAATAAGCACCGGCTAACTCTGTGCCAGCACCGCGGTAATCAC

GAGATCGGAAGAGCGTCGTGTATGGGGA

>8186 len=208 tot-seq=1

AGAGGGCAGAATGATGGGAAGCTGAGCTATGGGGCAGTCACAACAGTAGCCTTAGCCCAC

CCATAGAGCTTGAACTGGGTGGCCCTGCAGAGGCAGGGATCAGGTCTTTTAACCCAACAC

TGACTGCTGGCTGCCCTTGGCCAAGTGGCTGTCTTCAGCTCATGGCCACCCCCTGAGGAA

CTGGGCAGAGTTCTTAGCTGCCAGCACC

>8187 len=208 tot-seq=1

ATTTCTGACAGCAAAAATAGCCTGCTTCAGCTTTCAGTGGGGATTGATTAAAACCAGCAA

ACGAGAAAAACCCCAAACAAACATCTCATAGCAAAGATGGATGTGAGCCTGCAGGGGAAG

AGGCTGCTTTTCTAGGGACAATGAAAGGTGCTGTGGACTCAGAAGGGACACAGCACCAGA

CACTGCGCCTTGCCTTGAAGAACAGGAG

>8188 len=208 tot-seq=1

ACTGTGACTGGAGTTCAGACGGGGGAGCGGCCGAGCGCAGACGGGAGGCAGCAGGGAGGA

AGAGGGGGCAAGGGACGGGAGGCGGAACCAGCCAAGGAGCGGGCAGGAGGACGGCCCTAG

GGGTTGTAAACTGCTTTGTTTGGGAATAATCGACATTACGTGTAATGTTTTGCATGTACC

ATTCGAATAAGGACCGGCTAATTCCGTG

>8189 len=208 tot-seq=1

GCAGTCCCAGAGAACATCTAATTGGTCTGTCAGGGTTAGAATAAGCCCATCCTGATGGCA

GGCACTGGGAGTCGGGGAGAGGCAGTTTGCAAACACCAAGTTAAGGTGCTGTTGCCAGAG

TGATGACAAATGGATCCTGTGCACAGGGCCGGGTGCAATGACAAAGGAACTGAACCGAGA

TGACACTTAGCAGCAACACGAACACGGA

>8190 len=208 tot-seq=1

GACGGCATACGAGAGGAGAGCGGGACTGGAGGGCAGACGGGGGCGAGGAAGAGAGAAGAA

GGGAGGCAGAAGGAGAGAAAAGGAAAAGCGACAGAAAAGGGGAGGGAGGGACGAAAAAGA

AAAAGACAGGACGCGGGCGAGGCCCGGGAAGGGGAAGGAGGCCACTGTAAATCCTTGAAC

GAGGAGCCAGGGGAGGGCAAGGCGGGTG

>8191 len=208 tot-seq=1

CAGGGATGCCCAGTGGAAGAAGATCAATGGCCGGGCTGTGTGAGGAGTGGCTGACCGTGC

AAACAAGCCCTGGACAAAGAAATCTGTGAAGAGTTGAAGGGAGCCTCCTGGGAGCAGGGG

AGCAGCTCCTGCCTGGAACCCCGGGGCGACGGACGGGCAGTCGAGGCTCTCAGGGAAGGA

GTTCCGCTGCTCTGACCCTCCGCACTGG

>8192 len=208 tot-seq=1

CCGTCTCTGGGAGCTTCAGCGTAATCGTAATTTCCAGGCCAGTCGCCGTCTTGAAGGCGT

CGAGATGCCTTTAGTCACTCTTACTGCCGCAGAGGCTTTAACGCGCCTTGAAGCGCTGAG

GAGGCACTATGAGCGATAAATTCGGCGAAGGGCGCGATCCGTATCTTTATCCAGGCCTTG

ATATCATGCGTAGCCGGCTGAACATCCG

>8193 len=209 tot-seq=1

CCAGTATGGCTCATAGGGAGTTTCTGAACCACAGAGTGGGTAGCCGAGGGCGGCAAAGAG

CAGCAGAGGCTTGACCAAGACTGGAAAATGAGGTTCCGAGGGAGGGAGAAAGGTTGGCCT

TGGCTTTCCTGGCAGGGTGGGGGCTGAGGCCATCTGCCAGCACACCCGAGGATTGCCATA

TTGTCATTATTTGAGATTCACAAAGACTT

>8194 len=209 tot-seq=1

GGAATCTCTGCGATGGGGCAGCCAGCCAGCCTGGGATCCGAGCCTATGCTGAGCACAGAG

GGCTTGAATCTACCTTTGAGACCATACATTGAAGTCAGGCGTACTCACTGTGGTTGGGGC

ACATGTGGACTTAAGAGGGAAGATATTGAGAACTTGTAGAGCCCAAGAAGGGTCAGGGAC

AAGTCCCTTTCCTTTGTTCACTGGTATAT

>8195 len=209 tot-seq=1

GAGAAATTTTAGCAGCAGGTGTGATCTCACTCCTACTTGGAACTCGATGTGGTCAACCAC

ATAAAAATTAGGAGTAGAAGAGATGTGACCTGAGACCGGGGATTGATCCTACTGGGGATG

GGAGACTGTAAACCAGTGGGTCAATCAGTACAAGTTGGCAGCCTTGTCTCTTCACCTGCA

TATACCGCATCCATCTCTAAACAGTGCAC

>8196 len=209 tot-seq=1

TTAAGTAAAAGATTATCCAGACGGATGTAATCTAATCCCATAAACTCTTAAAAGCCTCTT

CTGCTGGTGGCAGAAGAGAAAAGAAGTTACAGAGCTGTGACAGAAAGGGAAGTCAGAGAT

TTTCAAAGAATGAGAAGGATTTGACAGGCCATTGCTGGCCTGAAGATGGAGGGGATCATA

TGGATGGCTTGATAAGGAAATAAATTATG

>8197 len=209 tot-seq=1

AGGAGTGGACAAAGGCTGGACTCACATCTCCTTCTCTTGGTGGAGTTTGCAGGCCTCTTG

CTGGAGGCTTTCCAACTGCTGCTGAGCATCCTGGAGCTCCCAGGAAGTCCGCTCCAGCTC

CCGGGCCAGCTCCTGGTTAGTGAGTTTCAGCTTGGTATTCTCAGCCTTGGTCTCATGCTT

GTCATGATGCAGGGCTGTGCACTGACCTT

>8198 len=209 tot-seq=1

CTAATGGCATGCCCTTGCTTGGAGACCAGATTGCTGTGTTGTAATTAATTAGGATGTGGT

AGCACTGGGGTGGGCGGGCCCCTAATTTAGGAGGAATGCTGTCCTCATACAAAGAACACC

ACATGGAGAGACAGACTCACAGTGGGAATGCCAAGAGGGCATGAGGCAGAGACGGGGTCT

CCCTGCAAGCCAAGGAAGGCCAGAGGTCA

>8199 len=209 tot-seq=1

GGTGTGAGTTGGGAGTAAGGAGGGGGACTAAGGGATTTGCTATCAAAAGATCTGGGTTTA

TGGCCTTTCTGTGCCACTAAGTCAATATGTTGGTCAGGTAGAGCCTCTGTTTACTCATCT

GTACAATAGGTATGGCACGGCTATGAGGCTCCCATCAGATACAGTCCAAAGGGCTCTGGG

AGACTTCCCAATCCACTTCCCTCCTTGCT

>8200 len=209 tot-seq=1

CTACAAACAATAAGATTTCAGGTATATTATTTAGGTGGCAGAATATAAAATTAATATACA

GAAATCCATAGAATTTATCAAAACAACAACCAGTTAAATGACATAATGGAAACTAGGATG

TTGTTTACTATAACAGCAAAAATATAAAATGCTAAAATTATACTTAATAAAAAGTTGATC

TCATACAAGTAGAGATTAGAGTAGTGGTT

>8201 len=209 tot-seq=1

GATTGCTTGAGCCCAGTTTGAGGCTGCAGTGAACTGTGATGGTGCCACTGTAGTGCAGCC

TGGGCAACAGAGCAATGCTCTGTCCCTAAACATTAAAAATGAATCAATGAATGAATGAAA

TGAGGTGGTCAGAAAAGGACAAATATTGTCCATTTATATGAAGTACATATAGTAGTCAAA

TTCAGAGACAGAAAGTAGAATGGTAGTTG

>8202 len=210 tot-seq=1

TAGCTGATAAGACAGTCTGATGATGAAATTGTGGTAACCCAGGTGGAGATGGGTCTGATG

TCCAGCTCTTCTCCCCACTTCTACTCAAGGTCCCAGGATGGAGCTGACAGAGCCCTTACC

GAGCGCAGCCGTGCAGAAGGAGGAACAGGAATTGTTGGATCGAACTTTCTTCTCTTGGGC

TGAGTTCAGCCGCTTCTTCGACAAGTGGTG

>8203 len=210 tot-seq=1

TGTGCCAGGCATGACTGGCTAAGCAGACCACAGAGCTGTAAAGGCTCTAGTGACGTGCAC

ATCACATCTCTATTTTAATTTGCCTTGGCAATCTACCCCAACATAGTGAAGTTCTTGTGT

GACACGCTACCACTGTATCTCCCAAAACCAGCAGACACAAACATCATACAAAAAAATCGA

TCCAGAGGAGGGTGGGCTCATAAGTGATGG

>8204 len=210 tot-seq=1

GAACGTAGCTGGGAGAAGGAAGAGTAAGACAGACAACCGGTTAGGAAAGAATTCTGTTTA

TTTTCAGAATCCCTTGGGCTGCATGTGGAGTCTTTTGGCCATTAACTGCTTGAGTCACCT

CCCCTGTGCCTCCTGGTTCTGAGAGGTGGCTGCTGTTTTCTCCAAGGAGTTGGGAGGCGT

TGGGAGGATGACAAGATGTTGGAGCTGTTG

>8205 len=210 tot-seq=1

GAAATTATGATGTTGGTTGGTTGCTTCTAGCTTCTCTGGATTAAACTGACAAAGGAAAAA

AAATGAGCTCCATGATAAAACTTAACCAATATCGAGCATCTCCTAATACCACAAAGAACA

GCAATGAATTCAGTGATGAAACTGACCAACTTCAGTCTCACATAAATGTTCTAAAGGTTT

CTAAGTATACCCTGGAAGAGAATCTTCTCT

>8206 len=210 tot-seq=1

AATGAACAAGGGCAGCATCCTCCTCTTCCTCTTCCCCTCTTGCTCAGACTACAGGAGGAT

GCTGCCCTTGTTCATTCTGCTTCCTATCCTTGATCCTTGGGTCTTTGAAAGGCAGCTCAA

GTTCAGCTGCGGCTCACCCTGCCTGGTTAGGACCTAGGATCCTTATCCTTCCCTCTGCCA

CCCTGCATGACCGGCCTCTGTCACCACCAT

>8207 len=210 tot-seq=1

TGGGGAATATTGCACAATGGGCGCAAGCCTGATGCAGCCATGCCGCGTGTATGAAGAAGG

CCTTCGGGTTGTAAAGTATTTTCAGCGGGGAGGAAGGCGATAAGGTTAATAACCTTGTCG

ATTGACGTTACCCGCAGAAGAAGCACCGGCTAACTCCGTGCCAGCAGGCGGTAATTTCTA

GATCGGAAGAGCGTCGTGTAGGGAAGAGTG

>8208 len=210 tot-seq=1

TAGGGAATTTTTCACAATGGGCGCAAGCCTTATGAAGCAATGCCGCGTGTGTGAAGAAGG

CCTTCGGGTTGTAAAGCACTTTCAGCGGGGAGGAAGGCGTTAAGGTTAATAACCTCATCG

ATTGACGTTACCCGCAGAAGAAGCACCGGCTAACTCCGTGCCAGCAGCCGGGTAATCTAA

AGATCGGAAGAGCGTCGTGTAGGGAAAGAG

>8209 len=210 tot-seq=1

CCAAGTGACTGGAGTTCAGACGTGGGCGCGGCCGAGCGCCGACGGGAGGCAGCAGGGAGG

AAGAGGGGGCAAGGGGCGCGAGCCGGAACCAGCCAAGGAGCGGGAAGGAGGAAGGCGCGA

GGGGTCGTAAACTTCTTTTATATAAGAATAAAGTGCAGTATGTATACTGTTTTGTATGTA

TTATATGAATAAGGATCGGCTAACTCCGTG

>8210 len=210 tot-seq=1

GCCACCTTCTTCCTCTCCCCACCGCCCCCTCTCCCCTGCAACACGTCATCTGACAAGTCA

GTGCGATCTCACTGGAGGTGCATCTCACAGGAACGCGGGGTCACAGCCTCCTGCACACAC

TCCATGCTGCACAGCAAGGTGCACGTGTCCCTCAGAGCCCCAGACACCATCCCCCACTCA

CCCAGAAGCCCAAGTGATTCCCAACAGCCC

>8211 len=210 tot-seq=1

AGGGGAGGGAGAGCTGATCTGTACAGGGGGCAGCCAGTGAGCAGGAAATGCCCCAGCACC

GACAGGAAGGAGGGCCGCCTTCTGACACAGGGCGGGCTGGAGACATCCCAGGTGACAGTG

ATGATGACGGGGTAGCTACCACTTACTGAAGGCCTCCCGCATGCCATGCACTGAACTGTG

GCTCACCCACATGCTCTCATTTAACCTCAG

>8212 len=210 tot-seq=1

CCGGTCTCCTAATCTGCGTGGGCTGACCAACAAGGAGGTCCGCTCCGCAGAGGAGACTCA

GGTGGATGCTCGCTGTGGCGGTCCCCAGCGAGGGTTGTTGGCACAGCTTCTAAGGGACAG

ACAGCCTAGTTCAAGGTCCTACTCAGAGTACATCTCAGCCTGCAAGCCATTAGAGGGGAG

GAAGTTCGCTTCGGATGCAGGGAGAGACTT

>8213 len=210 tot-seq=1

CGTGGATTAAGCGTGTTGTGAAATGTAGACGCTCAACGTCTGACTTGCAGCGCGAACTGG

TTCACTTGAGTGTGCGCAGCGCAGGCGGAATCCGTGGTGTAGCGGTGAAATGCTTAGATA

TCACGAAGAACTCCGATTGCGAAGGCAGCTTGCGGGAGCACAACTGACGCTGAAGCTCGA

AAGTGCGGGTATCAAACAGGATTAGAAACC

>8214 len=210 tot-seq=1

GGCACAGCATGGAGGGAGGGCGCAGGCCAGGGTGGGCCATGGTGGTGCAACAGGTGCAGC

TGGGTGAGGCTCTTCTGCTTAGCCAGGCGCATGGCAGCTGGGGAGCTGCAGGGCAGACCT

AGCTCCTGGCAGAGCAGGGCTGTGAGCACCAGGCAGTCATCCCAGTAGGAGAAGCAGCCA

TCTAGCTTGAGGCCGCGAGCCCGCACCAAC

>8215 len=211 tot-seq=1

GGGTTCTGTGTGCTGTTTGCTTTGGCCAGAGGTTCAAAGTTCTGTAGCTGGTGTTGGGCT

TCCATCCTGGCTTTTCACCTCCTCTGATTCCAAGCTATTTCCCCTTTAATACCAGTGCAT

TAGGAGCTCTGCATTTCACCTGGCTCTGGGTAGAAGGGAACACAGTTGGGTAGTGGCACT

ATGGTACAGAGGAAATGTCAGAGACGGGAGT

>8216 len=211 tot-seq=1

TGGGGAATATTGCACAATGGGCGCAAGCCTGATGCAGCCATGCCGCGTGTATGAAGAAGG

CCTTCGGGTTGTAAAGTACTTTCAGCGGGGAGGAAGGCGATAAGGTTAACAACCTTGTCG

ATTGACGTTACCCGCAGAAGAAGCACCGGCTAACTCCGTGCCAGCAGCCGCGGAGCAGCC

GCGGTAATGCAAAGATCGGAAGAGCGTCGTG

>8217 len=211 tot-seq=1

TGGGGAATATTGCACAATGGGCGCAAGCCTGATGCAGCCATGCCGCGTGTATGAAGAAGG

CCTTCAGGTTGTAAAGTACTTTCAGCGGGGAGGAAGGCGATAAGGTTAATAACCTTGTCG

ATTGACGTTACCCGCAGAAGAAGCACCGGCTAACTCCGTGCCAGCAGCCGCGGTAGCGGT

AATCCAGAGATCGGAAGAGCGTCGTGTTGGG

>8218 len=211 tot-seq=1

GCTGGGCCTGGGCTGGTGTCTGGGGCAGGGGTGACATTGTTAAGAGCTTCCCCTTTACCC
CAGTGCTCAGGGGCAACCCAGGACAGAGCAGATCATCCCTGGGCCCAGCCATAGGGAGTG
TGAGGCTTGGCGGTCACCCCCGATGTGGGTTGACCCCAGGGGTATGGCCTCTGGTGGCTC
CATGCAGAGCCTCCTCCCAAGGCAGAGGATC
>8219 len=211 tot-seq=1
TAGGGAATCTTCCACAATGGGCGAAAGCCTGATGGAGCAACGCCGCGTGGGTGAAGAAGG
TCTTCGGATCGTAAAACCCTGTTGTTAGAGAAGAAAGTGCGTGAGAGTAACTGTTCACAT
TTCGACGGTATCTAACCAGAAAGCCACGGCTAACTACGTGCCAGCAGCCGCGGGCGGTAA
TACCCAGATCGGAAGAGCGTCGTGTAGGGAA
>8220 len=211 tot-seq=1
TGTAGCAGGTGTGAGAGCGTCAAAGCAAAGCCACCATGACTGTGGTGCTGCTGAGGACCT
GAGGCAAGTCCCTTTCTTCTGCACCTCATTTTGCTCAATTGCAAAGTGCGGAGAATATGT
AGGGAATTTAGTCCAGTGGTTGACATGTGGAAAACCACCAGAAACACGCATGGAGATGTT
TAATTTTGGAAAGAGTCTACCCTGAGCTAGT
>8221 len=211 tot-seq=1
CTGACGTGGAGATGAAGCTTCCTGCTGGGCACATGCACATGCCAGTTTCTTTTCCTAATA
GGTCCCCACTGGGTGCTGGCTGCATCAACTGAACATCAGAGAACTAGGAAAGTTCACAAA
CACTGAGCAGTAGTATCTAAAGCAGTTTAAAAATAAAGCCAAAAGGAAGACCAAAAAATC
CCTCATCACTGAGCGGAGACAAATCTACATA
>8222 len=211 tot-seq=1
CTCTTCCGATCTCCTACGGGAGCAGCAGTTGCAGTGGGCAGAGATGGCACACAGTTGCAC
TCTAGCCCAGGCAACAGAGCAAGACTCCGTCAAAAAATAAAGGTGGTCTATCTTGGTAGG
CTATTTTTTCCCATTCCTTTGGTGGGAGATCAGTCTTCGCTCAGGGACTGTATTGTCTGT
GCCCAGGAGCATCTCCAGTTTACAGGCTTCC
>8223 len=211 tot-seq=1
ACCGCACTGGATGGTGGTGGGGATTTCCATAGCTCTCCGGCGTTTGTAACGCAGCTCACT
GGACGGGGGCTGGTTCCAGTTGGCAGAGAGGTAGCCAAACTCATCCCAGAACTTGATGAT
TCCCCTCTGGGCTGGAAAGCAAACACCGATACATCATGTTAGGAGCCAGCCTCTCTCTCC
CTACATTCAGGTGAAGAGAAAAGGCAGAGGC

>8224 len=211 tot-seq=1

AGAAGTCAATCTCTTCATTCTGTCACCATAGCTTTGCTGTCCTATTCCACCTGGTACTCT

TTCTTCAGCAACCAGTCACCTTGTGCTTTTCTCATATCCATTGAGTGTTAAACTCATGGA

TGCCTTTCCACAGCAGTGCTCTGTGATTTAGGTCTCTCATTTCCTTTTGGCTCTGATGAA

TGAACATGTCTTAGGCTCCATTCTTTCTCAG

>8225 len=211 tot-seq=1

TGAGGAATATTGGTCAATGGACGAGAGTCTGAACCAGCCAAGTAGCGTGAAGGATGACTG

CCCTATGGGTTGTAAACTTCTTTTATATGGGAATAAAATGTTCCACGTGTGGGATTTTGT

ATGTACCATATGAATAAGGACCGGCTAACTCCGTGCCAGCAGCCGCGGGCAGATCGGAAG

AGCGTCGTGTAGGGAAAGTGAGGAATATTGG

>8226 len=212 tot-seq=1

AGGGTTCGACCCTGTGGCCTTGCTGGGGAACGGGGAAAGCCAGCGGTGGATCTTTAAAGA

GACCCCATGAAGGAATGCACAAACACCCAGAGAACTGAGCCTGGCAAGCTCTCAGCGCTT

GCCCTACTTTCTCCTATGCCATTAATTGATTAAAATTTTAAGACAAATTATTAAGCCACT

GCCTCATGCTGTTTTCAATGCTTAGTCTCTAG

>8227 len=212 tot-seq=1

CAGCAGCCGCTCTGCCAGAGCCGCGGACTTGCTTGTGCTACAACTGTCTTCCTCACTCTG

AAGGAGCTGGGGTGGGGATGCTGGAGGAAATATACTTTAGAATTATTTTTCTTCCTGAGC

CGCCGGGAAGGCCGACTGTCTTTTGACATGCTAGTTCATTTTTTTCTTTCAGTTTTGCCA

GGGGACAAGAAACAAATGTTCTCTCTGTTCAC

>8228 len=212 tot-seq=1

TGGGGAATCTTGGACAATGGGCGAAAGCCCGATCCAGCAATATCGCGTGAGTGAAGAAGG

GCAATGCCGCTTGTAAAGCTCTTTCGTCGAGTGCGCGATCATGACAGGACTCGAGGAAGA

AGCCCCGGCTAACTCCGTGCCAGCAGCCGGTAATTCGTAGATCGGCAGAGCGTCGTGTAG

GGAAAGAGTGTGCCAATGTGTAGATCTCTGGG

>8229 len=212 tot-seq=1

GAGAATCACTTGAACCTGAGAGGCAGAGGTTGCAGTGAGCTGCGATCGCACCACTGCACT

CCAGCCTTGGCGACAGAGTGAGACCCTGACTCAATAAATAAATAAATAGCTATTGGATAC

TGTTTCTTTTTTTTAGGGACAGGGTCTCTCTATTGCCCAGGCTGGAGTGCCATGGCATGA

TCCCTGCCGACTGCAGCCTCAAGCCATCCTCC

>8230 len=212 tot-seq=1

TGCTCATGTGAACATGGAGCGCTCACCCCAGCCCCTCAGCACAGCCAGGGGGCCTTGGGG

TACACACCCTCCTTCCCTGGGGCCGCCAGCACCTCCTCTGCCCTATCCCGGATGGGGCCT

GGGGGTCTGCCCAGGGTGCGAAACTGGAATCTATGCTGAAACACCTAAGTGCCCAGGAGG

TGCCCCCATGGCCCAGGAGTGACACGGCTCCC

>8231 len=212 tot-seq=1

TGGGGAATATTGCACAATGGGGGAAACCCTGATGCAGCAACGCCGCGTGAGTGAAGAAGT

CATTCGTGATGTAAAGCTCTATCAGCAGGAACGAGACAAGACGGTACCTGACTAAGAAGC

CCCGGCTAACTACGTGCCAGCAGCCGGCGGTAATGATTAGATCGGAAGAGCGTCGTGTAG

GGAAAGAGTATGCCAATGTGTAGATCTCGGTG

>8232 len=212 tot-seq=1

GATCGCCAGCGTATCTTTTTTGCTCATTCCCAGGGCGCTACAGGACTGCCACTGACCTTC

CGGGATCGCGCGAATGGCACCGTAAAACAGCTGCGTGGTATACGCTGCACTGTTCAGCGA

CAACGCAATCAGCGCACATAACCACGGTTCTGACAACAGATGCCACAGTGCCGGATACTC

CTGCAACGTCGGGAACTGACCCGGTCCGTAAT

>8233 len=212 tot-seq=1

GCAGCACCAGTCTACATTTTTATAAAATGACAAGGGAACACATTATGTATTTCCTTGTGC

TAGTGGCCCTTTGTGGGACAAACAGAGGGCTTCTATAAAGCTTAGGAAGTATGATTTTAA

TCACCGGGCAAATTCATAGGTGGAGGCTTGAGCATTTCCTTTATCTCTGTACCAGAATTT

CCCAGGTATACACTCCCCAGCATGTCATCAGC

>8234 len=212 tot-seq=1

TTAAATGTGTCACATTGAAAGCTCCATTACAGTTGAATGTAAAATTTGCCTCTTAAAAAA

AATAACAAAGATGATTGTGTGAACCTTCCTTTGCCAGACCCCTTCAGATTAGGAGCTCTT

GGGGCTGCCCAGCAGGGTCTGAGACACCGAAACCTCCTGCTTTCTGAGCAGCTGTTTCTG

GTGGCCAGGCGGTGGGAAGGCCATCAGAGGCC

>8235 len=212 tot-seq=1

TGGTGGCCCTGGGGACTTCCTTCCCTTTCTTTCTCCACAGGAAAGAGGAGGGGCTAGAGG

TGCTGAAGGAGCAGAATTGGGAGCTGCCCCTGCAGGATGGTGAGGGCCTCTGGACCTGAG

GGTCCCTGCTGTGACCATCAAGCAGTGGGGAAGGGTTTGGGAGCCCTGGGCTTCCCTGAA

GTGCTATGTTTGCCCTCTAGAACCTCTGTACC

>8236 len=212 tot-seq=1

CCAGTGCGGAGCCTAGGGATGGGCTGGGGCTTCTCAGGGTGCATCTGCTTCACTGCCATG

ACTGACTTGACACAATTTCTGTACTTCATAAGGACTTGTCTCCTAAGAGTTACAGTTCCT

GCAGTGAAGGTCTGTTATTAAGACTGTGAATGTGGAAAACCTTTAATAAAGTCGATCGTT

ATTTTAGAATAAACTTTTAAAAGAAAAATGTC

>8237 len=212 tot-seq=1

AGAGCCTGGGATGCTTCCATCCATGGACATCTATAATTGGAGGATGGCTGCACTCTATTT

TGAATAACTGAGTCATCAGCCTCTTGAAGTCATGGAGCTACCTCAGATGACTCCTGCGAG

TCTTTCCAATGCCTGCTCCTTAAAACTCTATTGCTTAGAGAAGAAATGGTTTAACGGAGA

CTCTCATAAGCCAGTCTAGATCAATATCTGTC

>8238 len=212 tot-seq=1

CTACACAGATGCATGGCCTTGCAAGAGTACATGGCAGGTTTAGAACGACTAAAGCAGTTT

AGAATCCCAGCTGCTTTCCTACTAGGTTTTACCAATAGGAGGCACTAGAATGAGCTGGAG

AGTGAGAGAAGCAGTAAGTTGATGTCTCCTGTTGTCCCTGTTTCTCAAGAGTCATGGGCA

GCTGGCTCCAGCCTTTCCATGCCCCTTGGGTT

>8239 len=213 tot-seq=1

AGGAGATGAGCTCAGAGGAATTTGTTTGCTAAAGAAGCTGGCATTGTGACCTGCAGCTTA

TGGGCAGCAGTGGAAGACTTAGGAAGAGGAAGGTGGCTATAGCCATCATTTAGTATATTT

AATAAAAGGTAAGTAAAAAGAGAATCTTCCTTTAGTGTTTCAGAACTTTCAAAACATCTG

AGAACCTCCAGATGCTGTAAAATCACCACCACC

>8240 len=213 tot-seq=1

TATCTTCAAAGAGGGTCTTCGACAATCCTTGATCAAAGACCTTGCGGTTCTAAGCACAGA

GTTGGCCTTCCTCCTCCTCTCCCGTTTCCACCCTCTCCTGCAAGCAGCCGAGTGTCCTCA

CAGTGCGGCCATAGAAGCTGGGTGGGAGGAGGAGGCCTCATGCTGGGAAGGCCCTTCCCT

TCTCCTCCTCAATGTCCTGCTCCAGGTGAGTGC

>8241 len=213 tot-seq=1

TAGGGAATTTTTCACAATGGGCGCAAGCCTTATGAAGCAATGCCGCGTGAACGATGAAGG

TCTTATAGATTGTAAAGTTCTTTTATATGGGAGGAAACGCTAAGATAGGAAATGATTTTA

GTTTGACTGTACCATTTGAATAAGTATCGGCTAACTATGTGCCAGCAGCCGGTAATTGGC

AGATCGGAAGAGCGCGTGTAGGGAAAGAGTTAG

>8242 len=213 tot-seq=1

GAGAAACTGAGGAAGAGCTTCATGTCCAGGTACCTGGTATTGACCCAGGGTGAGGGTGAG

ACAGAGATGAGGGGCTCTGGGACCCCCCATCCCCTAGAAGGTAGTCCTCAGATCATGGAT

ACCTCCTTCCCACCTTCAGAGAGGGAGAGAGGATGGTCTGGGATGCAAGAGATAGCCTTT

GGAGTGGACATGTAATGGTGGGCGAGGGAAGAA

>8243 len=213 tot-seq=1

GATCTATGTGAAATAAATGCCTGCTCCAGTGTGGGGAGCAGGGAAGAAAACTGTGACTGG

CCTTCCAGGTCATCCAGAACCCTCCCACGGTTTCTCCAAGCACGCTCTGGGACCACTGGC

AGTGGGATCCCTTAGCGGGACAGGGCAGGCTGGTAAAATGCAGATCTCTTGGCCATATCC

AGACCCACTCAGAGAGGCTCGCTGAGAGTGGGG

>8244 len=213 tot-seq=1

TGTGGGAGCCACTTTCTGGGCTGGCCAAGGCTGGAGCCCGCCCCCTCAGCTTGCAGGGAG

GTGTGGAGGGAGAGGCGCGAGCAGGAAGCAGGGCTGCGTGCCGCGCTTGCGAGTCAGCTG

GAGTTCCGGGTGGGCGTGGGCTTGGCGGGCCCCACACTCAGAGCAGCCGGCCAGCCCTGC

TGGCCCCGGGCAATGAGGGACTTAGCACCCGGG

>8245 len=213 tot-seq=1

GGAGCCACAGTACAAATCCCAAGAGTATCCCTCAGATGTTCTTGGGTCCAAGTCCTGGGT

AACAGGCTCACATCCTTGCAAAAGGGGAAGTGTGGGTTTTCCTTTGGGGGCCTAAAGGGT

CTGTGGTCACTGAGAAGTCCTCACAGCCTCCAAAGGCAGAGCTGCAGGAGGGAAGTTGCA

GTAGCAGCAGATGAGGCTGGGAGAGAGGCAAAG

>8246 len=214 tot-seq=1

GACAAGTCTCTTAGTGATGTTTAAGGAGCAATGGGGCAGTCAGTACAGGGAAGCAAAAGG

GCAAACAATGCGATTTGCAAAAGCAGGGTGGACTCCTGAGGACTGATATTTCATTCCTTT

GTAATAATAGATCCACCACAGAGCCTGGCTGACTCATCTGCCAAATGCTTCTCTAGAGAG

AAAGAAAGGTCACCAGGGCAAGCCAGCAGCCGCG

>8247 len=214 tot-seq=1

CACACACAAATAGAATAAAAAGTTTAAAGTAATGATTAATTATCGAAGTGGCAGGCACCC

TTACGTTATTTTTCTATTCACTCTCTCTAGGAAGTAGAGGTTATTTGCAGGACTTAGAAG

TTTAGGTGACGGAGCTAATCAGAACCCTATGCTCACATTGATATCATCAAGCAGTAGATG

ATAACACAACAGCCTATAAAGGCCTGGAGTACAG

>8248 len=214 tot-seq=1

TAGGGAATTTTTCACAATGGGCGCAAGCCTTATGAAGCAATGCCGCGTGAACGATGAAGG

TCTTATAGATTGTAAAGTTCTTTTATATGGGAAGAAACGCTAAAATAGGAAATGATTTTA

GTTTGACTGTACCATTTGAATAAGTATCGGCTAACTATGTGCCAGGCCGCGGTAATTCCG

AGATCGGAAGAGCGTCGTGTAGGGAAAGAGTTAG

>8249 len=214 tot-seq=1

TAGGGAATTTTTCACAATGGGCGCAAGCCTTATGAAGCAATGCCGCGTGAACGATGAAGG

TCTTATAGATTGTAAAGTTCTTTTATATGGGAAGAAACGCTAAGATAGGAAATGATTTTA

GTTTGACTGTACCATTTGAATAAGTATCGGCTAACTATGTGCCAGCAGCCGCAATCTACA

GATCGGAAGAGCGTCGTGTAGGGGAAAGAGTTAG

>8250 len=214 tot-seq=1

TTTCAAATAGGATATGTTTCCGTTCATAACTACCTTCGTTCGGAACTTTTGCTTGTTCAA

ATAATCACACAAGTTGCGCACAAACAGCGCACAAATAAAATCACGCCCGCACTATGGCGA

GCGTGAATACTTAAATCAAATTTTCAGTTTTGTTATTCAGTTTTGTTTCAAAGCCGCCAA

ACGGCGAGCATTGGCCACAGCGTACAAGCTAATA

>8251 len=214 tot-seq=1

GAGACAATGAAGCTGATAAGGAGAAATGGAATCAGCTGAGAAAGGGCTTAAGTCATCTAA

AGAGTGTGGACTTTATCCCAGGCAATGGGAACCAGCCAGTGTCCCTCAGAAGAAGCACTT

CTAACATTAAGATACTTCCTGTGTGGAACTATTTCACCAGGCACTACAAAACATTTAGCA

TCTTGGCCAGGCACGGAGGCTCACGCCAATAACC

>8252 len=214 tot-seq=1

GACGGCAGACGAGATCTCTCTGTGACTGGAGTTCAGAAGGGGGAGAGGAAGAGAGAAGAA

GGGAGGAAGAAGGGGGGAAGAGGGAGAAAGGGGGGAAAAAAGGAAGAAGAGAAGAAGAGG

GCGGGAGGAAGGCCGGCGGGGGGGAAAACGCGGGGGAGGGGGAGCAAGCCGGCGGGGGAG

GGGACCGGTCGAATAAGCGCCGGCTAACTACGGG

>8253 len=214 tot-seq=1

ACTGTGATTTTCAGTATTTGAGAGGTTCGTTATTTTAGACTTTAGAGATTTTGACCTTCA

GGGATTTTGATCTTTTGAGATTTCAACATTCAGGATTATAGCATTTGGGATGGTGTCTCT

CAGCATTATGAGCCAAACAGTATGAATAACGAGCTAGAGTCCCCTAGAAAAAAATCTGTG

ATTCATCACTGTAGCAGGACCAGAATACCCTTCG

>8254 len=215 tot-seq=1

GCAGTAACTTTAGGGCCAAGAGAGTGTGATATCCCGAGTGAGGCCTTCTGGGAGCCTCTC

TGAGTGAGTTACTCAAGCTTTTGAGGAGGGATTTCCAAATAATATATGCAGCCACAAAGA

TCAAGGATCAGAAATACTTCTGTAACAGATAGTGGGTATAGCTATATACTGGGTAACAAA

ATGTTCCTCTTAGGGACAGAGGGCATCCAACTGTA

>8255 len=215 tot-seq=1

TAGGGAATTTTTCACAATGGGCGCAAGCCTTATGAAGCAATGCCGCGTGAACGATGAAGG

TCTTATAGATTGTAAAATTCTTTTATATGGGAAGAAACGCTAAAATAGGAAATGATTTTA

GTTTGACTGTACCATTTGAATAAGTATCGGCTAACTATGTGCTAGCAGCCGCGGTAATTC

ACAGATCGGGAGCGTCGTGTAGGGAAAGAGTGTGC

>8256 len=215 tot-seq=1

CTAATTTGGGACTGATGCCGGTTCGTTACATGGATAATATGATTATTTACGCGATGGCGG

GCTACTTTGCTTGTATTAGCAAGGCGCCATTTACAGCAATTCTCTTGATTACTGAGATGG

TGGGGTCGCTGGCCTTTTTGATGCCGCTTGCCATTGTGGCTGTGATCGCTTACTTAGTTG

TTGATAGTCTGGGTGGTGCACCGGTGTACATGGCG

>8257 len=215 tot-seq=1

GCAAGAGAGAGCTTGTGCAGGGGAACTCCCATTTATAAAACATCAGATCTCATGAGACTT

GTTCACTATCATGAGAACAGTATGGGGGAAACCATCCCCATGATTCAATTATCTCCACCT

GGCTCCCTCCTTGACATGTGGGGATTATTAAACTTCAAGGTGAGATTTGGATGGGGACAC

AGCCACACCATATCAAGGAGAGAGGCCCAGGTCAG

>8258 len=215 tot-seq=1

GAGCAAACTCTATGCAGGCCCCATGGCAGCATCCAGGTGGGGTGCCTGCAATTCCGGAAG

GCCCAGAGGGTGTGTTACAGTGCTTTCAGCTCTGCCATCTGCAGACAGCTTAAGTGTTAA

CAGCTCAGTGGGCCCATAGCTACCCTCTGCCAACTAAGGCAAAGAGCCAGTGTGACAGCC

TCCTGTATCTGCACTCATGGCTCCCAAGCTCTTGT

>8259 len=215 tot-seq=1

CAGGGGACGGGAGGGCAGACGGGGGAGATTAAGATATAATAAGGGAGGAAGAAGTAGGGA

ATATTAGGAAATGGAAGGAAGTATGAAAGAGAAAAGAAGAGTGAGTGAAGAAGGTGGGAG

GAGCGGAAAGCGCGGGGGTAAGAGAAGAACGAGTGGGAGAGGGGAAAGGGCACACGGGGA

CGGGAGCGGACCAGAAAGGGACGGCGAACGACGTG

>8260 len=215 tot-seq=1

CAGCATCATACAGCTGGTTTCTGAAACACCTTTTCTGCCCTCCAAGATCCTCTGACATGA

TCAACATTCCTGCATCATCAAGACAAACCTGAAAAGAGCTGTTCTTTGTTCTTTTTGGTG

GAAAGTGATGAGGTCCTTCCCGTTTGCACTGCCGTGTGGGTTATTTCCACTCTAGGTAAC

TTCCGCCTTCAGGTACTCCCCATACGTCTAATGGC

>8261 len=215 tot-seq=1

CTCGTCCAGGGCCACACAGCAGAAGGCCCGTGTCTGCTCAATGACTGAAGATACGCGGCT

CTTTTCTTGATTCTCCATTCATTCGTTGATTCACTCACTCACTGTAACACACGCACATAT

CCTCTGGGTACTCACTATTCCATTGCACAGCAACCTGAAGGCTTCCAGCTAAGCTTGAGC

ACCTGAGGACCAGCGACTCTGCCTGGGGGCTTCAT

>8262 len=216 tot-seq=1

CACTCCTTTTTAGGAGTCGTTAGAGTGCTAGACAAAGTGCTCTTGGCCGCTTATTGGTTT

AATAAATAGAACAAACCCTTGAGGACAAAAGAGAACAGACTTTCTCTTAAAAGGCTTGTG

CAAGACTAGGGAATGGTGCTTCCTGCATTGAGTAAATTGATTGTACCCTTCCTCGACTCC

CATTCGCAGAAGTTGGAGCTGAGCAACCAAGTGTCC

>8263 len=216 tot-seq=1

CTATTACTTCAGTCAACCTGTGAACAAGATTGGCATATTTTTTATATTTAATAAGCCTTA

TATCAACCATTTATCTCAGCTTTACAAGAGTCTGAAACATAGCAGAAATTATTCTCGTAG

TATGAAAAACAAGTCTTAAAAGCAATTTATCCATGGAGCTAGAGATAGCCCTGAGGTTAA

GAGCACTTGTTCTTACAGAGAACCAGAGTTCAGTTC

>8264 len=216 tot-seq=1

CAAGTGACTGTGTGCAGCTTGGTGTGTGGACAATAAGCATCCTCCTTGTTTGGTTGTATG

TTTCTCAGCTATGGGATTATTGACGTTTCATGGCGTGAAGCAACTGTACAGAGGTTGCAC

ACACTGAGTATATTTGTGGTTTCTTCAGTTCCACACCATACTCGGCATATGACCCTGAGA

CATATACAGGCCACTGGAAGCAACTGACTGTTCGTA

>8265 len=216 tot-seq=1

TGGGGAATATTGCACAATGGGCGCAAGCCTGATGCAGCCATGCCGCATGTATGAAGAAGG

CCTTCGGGTTGTAAAGTACTTTCAGCGGGGAGGAAGGCGATAAGGTTAATAACCTTGTCG

ATTGACGTTACCCGCAGAAGAAGCACCGGCTAACTCCGTGCCAGCAGCCGCGGTATGTAA

AGATCGGAAGAGCGTCGTGTAGGGGAAGAGGGTGCC

>8266 len=216 tot-seq=1

TCAACGTGACTGGAGTTCAGACGTGTGCTCTTCCGATATAATAAGGGAGGCAGCAGTAGG

GAATCTTAAAAAATGGACGAAAGTATGATGGAGCAAAGCCGCGTGAGCGAAGAAGGTTTT

CGGATCGTAAAGCTCTGTTGTTGGTGAAGAAGGATAGAGGTAGTAACTGGCCTTTATTTG

ACGGTAATCAACCAGAAAGTCACGGCTAACTACGTG

>8267 len=216 tot-seq=1

CGGTAGTGACTGCAGTTCAGACGTGTGCTCTTCCGATATAATAAGGGAGGCAGCAGTGGG

GAATATTGAACAATGGGCGCAAGAATGATGCAGCAATGCCGCGTGTGTGAAGAAGGCCTT

CGGGTTGTGAAGCACTTTCAGCGGGGAGGAAGGCGGTGAGGTTAATAACCTCATCGATTG

ACGTTACCCGCAGAAGAAGCACCGGCTAACTCCGTG

>8268 len=216 tot-seq=1

CCAGCTTCAGGGGGCTTCTTTGAATAGGAAAGGGGAATGCCAAGGGGCAGAAGAAGAGGA

AAGAAAAGAGGGCAGTTTGATCTGGTGATGTGATTTGTATCTTGGGGACAGCTGTACAGA

AATACACAGGAAATGGGACCCTGCCTCGAACCTTTCTGTGTTCAGAAAGGTCAGTAGTGA

CCTACATGTAGGCAGGTGACCAGGTAGCTGCTCACA

>8269 len=216 tot-seq=1

TCCTACAAGGTGCATTTGCTACTGCATTTTAAAACATTCAGATACAACTCACCTACCAGC

AATTGTATCATTTTAAAGTGTATGATTTGGGGGTTTTGGGTATATTCACATGGTTGTGCA

ACCATCGCCACTATGTAATTCCAGAATATCTTCATCACCTCCAAAGAAACCTCGTGCCCA

TTAGCAGTCTCTCCCCAGCCCTGGATGCCCCAGCCC

>8270 len=216 tot-seq=1

CTCATCTCTCTGCCAGAGCAATCTGATTGACAATCATCAATCAGGAAGGACTTCAGAGGT

CTCGTGGCCCCTTTACTACAGAGACTTGCCACAGCCACCTAGCCGGCGAGTGACGGCCTG

GACCCTCCCCATTTGCCAGGTCTAAGGACATTTATTAGAAAGGACACTGCTCACCACTGG

CCCTGGACACCTCTGAGACCACTGGGCCTGTCACGT

>8271 len=216 tot-seq=1

GCTAAGCATTGGCATCGTATGCAGGTTGAGTACGTGAAGTCTTAAAGATGAGAGAGTTGG

AATTTGGGATATACTGCGGCTTGTGCGGCGGTTGAGGACCAGGAAGGCGAGAAAATGTTA

CCAGCAAAGTTCCGTGGATTCGCATCACGCGTGGGTCGTCTTGCGTCGCGCGCTTTGGTC

TGGCGTAAGACCAGCGCTAACGACGCCGTTCGTACT

>8272 len=216 tot-seq=1

GGTGGGATCCAGATGCCTGCAGCTGGACTCATTTGTGTTTCTGGATCTCTTGTCTTCCCA

CCACTTCTTTGGACTTGCAAACATCGGGTCACATTTGGAACAAGGATACTGGCAGACAAA

CCATGTATCCTGAGTAAGAAGGAAAGCTGGGGCAGGTCCCTGCTCTTCCAAGGCCACCTT

GTGTCCATCCCCACACCTCTCCTAGACCATCTCCTT

>8273 len=217 tot-seq=1

TAGGGAATCTTCCACAATGGGCGAAAGCCTGATGGAGCAACGCCGCGTGGGTGAAGAAGG

TCTTCGGATCGTAAAACCCTGTTGTTAGAGAAGAAAGTGCGTGAGAGTAACTGTTCACGT

TTCGACGGTATCTAACCGGAAAGCCACGGCTAACTACGTGCCAGCAGCCGCGGAGAAAGA

TCGGAAAGCGTCGTGTAGGGAAAGAGTGTGCCAATGT

>8274 len=217 tot-seq=1

TAGGGAATTTTTCACAATGGGCGCAAGCCTTATGAAGCAATGCCGCGTGAACGATGAAGG

TCTTATAGATTGTAAAGTTCTTTTATATGGGAAGAAACGCTAAGATAGGAAATGATTTTA

GTTTGACTGTACCATTTGAATAAGTATCGGCTAACTATGTCCCAGCAGCCTAATTAACAG

ATCGGAAGAGCGTCGTGTAGGGAAAAGTGTGCTAGGG

>8275 len=217 tot-seq=1

CCGCCTCCCTGTGTCCCAGCACTGGGGAAACACAGTTCTGACTGCTCTGAGGAGCTTGTT

TGGTGAGCCCATTGCGCTGTTCCTCTCCGTTCCTGGCGAGCCTTGACACCAGTACCCTCA

TCTACAATGACACCCAAACTTCCACCTTGGCCTACAGTATCATGTAAGCAATTTGGAAAT

TCCTTCCAAAATAAGTTTCTAAAGCCAAGATCCCCAT

>8276 len=217 tot-seq=1

GGCAATCTTCTGTGTCACTAAGAATCAATTCTTTTTCTCCATTGTTTGTTGTTAGAAAAA

CAAGATGCCAAAATCCAAACAAAACCAGGAACGAGGTGGTTCTGGAGCTACCGCACAGCA

GCAGGCAGACTGACCACACTCCCGACTGGGAGTATGCAGCCGACTGCACCGTCTTGTCCT

TTTCCGCTACACACACACACACACAAGTGAAGAGGGTGT

>8277 len=217 tot-seq=1

TGTGCTCTTCAGTTACAGAGGGCTATAAGTAGCAAGGGAAGACAGCAGAAAAGCACTATG

GTTTTACATACAATCATCCGTTTGTAAAGGGAAATCCGATATGATTGATACTTCTTAGGG

TCATCTGCATATAATTCCTCAAAATACTGAAAAAACAGGCACAATGGATATTTGTTTTTG

TCTGTGTGTTTGAGGGAAATGCTATCTTTCAGCAAAA

>8278 len=218 tot-seq=1

GGTGTTATTTCCCTCCAGAGCTGAGCCAACCATTACAGACATAAGTACAATAGTTCTGTG

GTTCTTTAGTGAAATGTTGAGTAGAAGGTGGCATTTGAAAGACAATCCCCAATCATGCCA

GGTTGGGAAGCAGTGATTTGCAGACGTTCTAATAGCACAAACAGGAATGTAGGCTATGGA

TTTCACCCCGGGTCTGTTGGACTCTGATGGCTTACATA

>8279 len=218 tot-seq=1

GCGCGCGGGGACGGGAGGGCAGACGGGGGAGAGGAAGAGAGAAGAAGGGAGGAAGAAGGA

GGGAAGAGGAAAAAAGGGGAGAAAGAAGGAGGGAGAAAAAAAGAGGGAGGGAAGAAGGGG

GGAGGCGAGGAAAGCGCGGGGGGGGGAGAAGAACGGGGGGGAGAGGAACGGGGCACGCAG

GGACGGGAGCCAACCAGAAAGGCACGGCGAACGACGGG

>8280 len=218 tot-seq=1

CCTGGCTGAGGGAGGGGTGTCCGCCATTGCTGAGGCTTGAGTAGGTAAACAAAGCAGCTA

GGAAACTCGAACTGGGTGGAGCCCACCACAGCTCAACAAGGCCTACTGCCTCTAGACTCC

ATCTCTGTGGGCAGGACATAGCTCAAAAAAAGGCAGCAGACAACTTCTGCAGACTTAAAC

GTCTCTGTCTGACAGCTCTGAAGAGAGCAGTGGTTCTC

>8281 len=219 tot-seq=1

ACACCTCCACAATGCAGCTCGGAAACAGAATTTCTATAGATCTGCTGTCCTTTTTTTAAA

ATCACATAGAAGAGTTTGTACAAATCTCATTTGTTTTCACTTCCTTCATTTTTGTGTCTT

AACACATTATTTTGAGGAAAATGCCTCTGAATTTTATCCTTGCTCTTCAATTACGGTTGA

ACAGGAACTGTTAATTGAGTTTAGCCACTCAAAAGAAGA

>8282 len=219 tot-seq=1

ATTAACTCGTGACTGGAGTTCAGACGTGTGCTCTTCCGATATAATAAGGGAGGCAGCAGT

AGGGAATCTTCCAAAATGGACGAAAGTCTGATGGAGCAACGCAGCGTGAGTGAAGAAGGG

TTTAGGATAGTAAAGCTCTGTTGGTAGTGAAGAAAGATAGAGGTAGTAACTGGCCTTTAT

TTGACGGTAATTACTTAGAAAGTCACGGCTAACTACGTG

>8283 len=219 tot-seq=1

TTCTGGGTTTTTTGTTGGTTTTAAACACATTAGGTTCTAAGAAATGTTGCTGTGGGACCC

TTTAACATAGTTTAACACAGAAGGGAGAAAACTGTTTCCTTTCTCACCTAGCAACTCACT

GCTGCTGTGCTATTACAACTTAGCAAGGATTGAGAGCACTGGTAGCTGGAGGGGCTAGCA

AGGATCTGTACCTGGTGTAAAACATGAACATAGCAGAGG

>8284 len=219 tot-seq=1

TCGCCAGGGGACGGGAGGGCAGACGGGGGAGAGGAAGAGAGAAGAAGGGAGGAAGAAGGA

GGGAAGAGGAGGAAAGGGAAGGAAGGAGGAAAGAGAAAAGAAGAGGGAGGGAAGAAGGGG

GGCGGAGCGGAAAGCGCTGGGGGAAGAGAAGAACGAGTGGGAGAGGGGAAAGGGCACACG

GGGCCGGGAGCGTACCAGAAAGGGACGGCTAACTACGTG

>8285 len=219 tot-seq=1

GGGGTCAGCAGGGAAAGGGCGTGCGGGCCAGGCTGTGGCCTGCTATGACCCGTGCCTCTC

TCCCCCACAGGCCGGCTGGATCCTGCAGGCAGCTGTAGCCGTTCCCTGGCCAGGTAATGC

CTTTGGGCTGGGCAGTGGGTGGGCTCCCTGCTCGCCCTCAGGCTCACGCACCTCTCTCCC

CGTCTGCAGCCGGGCAGTGCAGCGCAGCCTGGCCATCAT

>8286 len=220 tot-seq=1

GCAGGCAGACAGAGATGGGAAAGGGAGAGAGGGAGGACACACTATAATTTAGCTCATAGT

TCCCAAATGTCAATGACTTCAGTTGTGACTTGTCAGTGACTCATATACTGTCACTAGACT

CCAGCTTGTCTTCTCAGCATGTCTGGTTCTATTCTCAAGTGTCCCTTCAAGATCAGAGGA

TGGCAGCAGCCACAGTCCTTCTGTCTTCTCAGTTTATAAT

>8287 len=220 tot-seq=1

CGGCAGCGGCAGCAGCAGCATCCTAGCCTGACATTTAAACTTCCAAGTGATTTCCTTGGC

TTCTCAGTTTCTACATCTGTGCAGGCTTCTACTGGCTGGCTCCTGCTGCCAGCTACACTC

TCAGATCTGTGCATGAGACTTACCCAACCTAGCCTTCAGAGCTAGCTCATCTAGCTTCTC

CCAGTTGGCCTGAAGGGCTCACGTGCTGCAGCCCATGACC

>8288 len=220 tot-seq=1

TCTGGCAGGGGGAGGGGCGTCTGCCATTGCTGAGGCTTGAGTAGGTAAACAAAGTGGCCG

GGGAAGCTTGAACTGGGGGGAGCCCACCGCAGCTCACCAAGACCTGCTGCCTCTGTAGAC

TCCACCTCTGGTGGCAGGGCATAGCAGAACAAAAGGCAGCAGAAACTTCTGCAGACTTAA

ACATCCCTGTCTGACAGCTCTGAAGACAGCAGTGGTTCTC

>8289 len=220 tot-seq=1

GACAGGGGACCCAGGGCTCACAGGGGCTTTCCGAATGATGGTCACAGGATTAATGTTCAC

AAGATAATGCCTGGGATGCCGGTGCTTGGGCACAGGAACCTTATGTCTGTTATGCTCGTT

GCTGTTTCCCTAGAGTGGCACTCACCAGTCCCCCAGGGAGTGTCTGGTGGGAGACTAGGA

GGCAAGAGACTCAAGGGTAAAATGAGATAGAGACAAAATC

>8290 len=220 tot-seq=1

GTGCGTAGGTGGGCTGTTAAGTCCGCGGTGAAAGTTTGTCGCTTAACGATAAAATTGCCG

TTGAAACTGGTAGTCTTGAGTATAGATGAAGTAGGCGGAATGCGTGGTGTAGCGGTGAAA

TGCATAGAGATCACGCAGAACTCCGATTGCGAAGGCAGCTTACTAAGGTATAACTGACAC

TGAAGCACGAAAGCGTGGGTATCAAACAGGATTAGAAACC

>8291 len=220 tot-seq=1

GACTCGGTGGAGGATGGACAGAAGGAGGAGTCTAGGCCAAGGGCCTCACCCCTGGCTGAA

GAATCGCTGGGGCTCAGGAGCCACAGATGCTCGGATCCCGCCTCCAGGGTTCCCAATCTC

ATGGGACACTGGTAGTCAAACCACCCCGGATGGCTCTGATGCGCAGCCAAGGCTGTGCGT

GACTCTGGGGCCAGGGCTAGTGGCTGTCACTAAGGGCCAC

>8292 len=221 tot-seq=1

GATCCTCTTCTTCCTCTCAGAATCTGGGAGGAGCTCAAAGTGGGGGGAGGGGGGAGGGGC

TGCTGCAAGTGTAAGTCCTCAGGAGCCCTCCACAGACAAAGCAGAACTGTCTGACCTGGT

GGAGGCTCCTTGCCAGCCACCAGCGCCGCTTAGAGAAAGATGAGAGCAGGGATATAGGGT

GCTAGCCACCGCCAGAGCCGCGGAAATTTCTAGATCGGAAG

>8293 len=221 tot-seq=1

TCCACGTTAGTGATCAGAATAAGACCTAAAGAGTGAAGCACAACTTGCAGGAATGCTGAA

ATCAAAATTGCTTTTTTCTAGATGTTTTGATTGCTAAAACCTGTGTGATTTCACGAAGCT

TAACTTTGCTCATTGGCATGGGGTTGGTGGCTCAGCTTTGCTAGTGTCTGTGCCCAAGCT

GAGTCGGCCCGCTGGTTTATCTACCGTGGACGTGTCCAGAT

>8294 len=221 tot-seq=1

ACGTGTGCTCTTCCGATCTCCTACGGGAGGCAGCGGGGTGGAGTGAGAGGCAAAATGGAC

TAGTCAAAACTGTCTGCAGATTCAATTATTCTACTAAACACGATGGGCAAATAGAAAACA

GCTAAGACGACACACATGGGCAGGCAGCATTCAGTGGGGAAAAGGGCCGTTCTCATCTCA

GCTCCCCTCCTTTCAGCCCTGCGTGCACTCGAGAAAGGAAC

>8295 len=221 tot-seq=1

TCCTTCTGAAATAAATTGTTTGCTTTACAGTGGAAGACATACCCTGTTCCTTTATTTTTC

TAAAGCAAATCCAATCCACTCTGAGCCTCCAGTAGGCAAGAAGTCTGAGATCATCACACC

ATGGGCTCCTTCATCCCTGCCCTGAGTGGGATGTGGGCCCTGGTGCTTCCTGCTGCCAGG

ATCTGGGGTGGCCTGGACTTTTGTCCGTCTCTTTTATGGGA

>8296 len=222 tot-seq=1

CAATGGAGGGCTGGGGCAGTGTTCTTCAGAAGGCAGTATATGCTTTGAATCAGCATTCAA

TATAAAGTACAGTTTCTCCCATAGCCAGGATTCATGGGTCCAGGAATCAAGGTGTGGAAA

AGGGAATAGTTCTACTTACTATCACTCCTAGTGATCCTCTAGAAAAATCTTCACTTCCTG

TCCCCACAACCCTAGGTTCTGCTTGCCTAGAAGTTTTGGTTC

>8297 len=222 tot-seq=1

AATTGCTTGAATCTGGAAGGCGGAGGTTGCAGTGAGCTGAGATTGTGCCACTGTGTGTGT

GTGTGTGTGTGTGTGTGTGTGTGTGTGTGGTGTATACACACACACACACATATATATCTCCC

ACATCTCCCACAAGATAAAGTCAATATCTGGTCCGCTTTGATCGTTTCCTCCCTGTCATC

ATTGCCCCTGTGTCCCTCCTGGCACCTCTGCCCAAGCCTGGT

>8298 len=222 tot-seq=1

GGGATGATGCTCAGGGTTGAGGAGGGTGGAGCGGGGACCCCTCTGAACCCCAGATGGCAC

CCAGCCCTGAGGAGAGGGAGGGGCTGCAGGCTGACCCTGGGGACCAAGGCCACTCACTGC

AGTGGCACGGTGGGAGGGGGCATCCTGTCCCCTCTGTACAGAGCCCATCCAGCACCCTCT

CCCTGCAAAGAGGACACCGACTTTGGGCAGGGGGCCATGTGT

>8299 len=222 tot-seq=1

CTGCTGATAAGGACATACCCAAGGCTGGGAAGAAACAGAGGTTTAATGGACTCACAGTTC

CAGGGGGCTGGGGAGGCCTCATAATCATGACAGAAGGCAAAGGCACCTCTTTCATGGCAG

TAGCAAGAAAGAATGAGAGAAGTGAAAGCGGAAACCCTTTTTAAAACCATCAGATCTTGT

GAGACTTATTCACTACCATGAGAACAGTATGGGGGAAACCGC

>8300 len=222 tot-seq=1

ATCCCTATTCATCCTGAATTGTAAGGGCTGGTTTGCTTGTGTCTACTCCCTTCCGATCTG

AGTGTTCCATGACGGCAAAGGCTGTGTCTGCTGGCTTACCATGGAACCCCATCGTACTCC

TAAACTACCTGGACACAGTAAGTATCCAACGAACACTTGAATGAGTGAGTGAGTAACAGA

AAGAATGAATAAACTTTGTAGCTAAGTCTCTTGCCTATTCTT

>8301 len=223 tot-seq=1

TGGCTGGACTGTAACTCTCAGACATCCTCTAGTCTGCACCACCCGATTGCTAGGATTAAA

TGTGTTCGCCACAGGCCTGGCCATCCTTGTCTTTTGATACAAAGTTTCTTGCTACACTTG

GCAGCGTCTTCCTGTCTCTACCTCCTCAGTTCTGGGAGAGCAGGCATGCGGTTACATTTT

AAGGGAGACAACTCAGGTCTTCACCTTTGCATCTCACTAAGAG

>8302 len=223 tot-seq=1

CGAGAGACGAATGTGACTGGAGTTCAGACGTGTGCTCTTCCGAGCGCCGACGGGAGGCAG

CAGTAGGGAAGCGGCCACAAGGGACGCAAGGAGGATGGAGCAAAGAAGATGGAGTGAAGA

AGGTTTTCGGAGAGGAAAGATCTGGGGGGGGTGAAGAAGGAGAGAGGTAGTAAAGGGCAT

TGATTTGAAGGGAATCAACCAGAAAGGCACGGCTAACTACGTG

>8303 len=223 tot-seq=1

TCATAGGCCATCACAGCAAGCAGGGAGGATTCAGTGCCCCCAGTGGCAAACACAAAGAAG

ACCTAAGCCATGCAGCCCTCTACTGAGATGGTTTTCTTCTCAGACAGGAGGTTCTCCAAC

AGCTTAGGTGCAGTGACAGAGGAATGGCAGAGATCCAAGAAGGACAATTGTCCCAGGAAG

AAGTACATGGGTGTGTGGAGGCAAGAATCAGCATTAATCACCA

>8304 len=223 tot-seq=1

TTCAAGCAGAAGACGGCATACGAGATCTGATCGTGACTGGAGTTCAGACGTGTGCTGGAG

GCAGCAGTGAGGAATATTGGTCAATGGGCGAGAGCCTGAACCAGCCAAGTCGCGTGAAGG

AAGACTGCCCGCAAGGGTTGTAAACTTCTTTTGTATGGGATTAAAGTCACCTACGTGTAG

GTGTTTGCAGTTACCATACGAATAAGCATCGGCTAACTCCGTG

>8305 len=223 tot-seq=1

CAAGCAGAAGACGGCAGACGAGAGCGCCAGGGGAAGGGAGGGAAGAAGGGGGAGAGGAAG

AGAGAAGAAGGGAGGAAGAAGGGGGGAAGAGGGAGAAAGGGGGGAAAAAAGGAAGAAGAG

AAGAAGCGGGCGGGAGGAAGGCCGGCGGGGTGGAAACCGCGTGGGAGGGGGAGCAAGCCG

GCGGGGGAGGGGACCGTGCGAAGAAGCGCCGGCTAACTACGTG

>8306 len=223 tot-seq=1

CCAGGGTGAGCCCTGCAGGGTGTCTCCTCCCTGATGACAAGTCCTCCTTGTCCTTGTGCA

GAACACCTCCAATCATAGGATACCTGCCTTTTGAAGTGCTGGGAACCTCAGGCTATGACT

ACTACCACATTGATGACCTGGAGCTCCTGGCCAGGTGTCACCAGCACCGTGAGTACCACT

GCCCAGCCCAGGCATGGGGGCCTTGCGTTCACTCCACTGGGGC

>8307 len=224 tot-seq=1

CTCTCCTTGTCTATCAGATGCTGACAAATCAGCCTACCTCATGGGGCTGAACTCAGCTGA

CCTGCTCAAGGGCCTGTGTCACCCTCGGGTGAAGGTGGGGAACGAGTATGTCACCAAGGG

GCAGAGTGTACAGCAAGTGTACTATTCCATCGGGGCACTGGCCAAGTCAGTGTACGAGAA

GATGTTCAACTGGATGGTGACACGCATCAACGCAACCCTGGAGA

>8308 len=224 tot-seq=1

GGATGCAAGACCTGAATAGAGGCCTGCGCTCATTGATTTGATTTGATTTTTCCTTCTAGA

TAACTGGCTCAAATACCTATTGTTTTCCCTTGATCTTAAGGCAGCATTTTCAGGACAAGC

AGGAGAGTCTCTACTTGCCTTCACCTCGGGCCATAAACCAGGGGACAGCACCACCTCCTG

GCCGGCAGAGATGTGGCGCCCGCTTGGTGTCCGGCTTGCAGAAC

>8309 len=224 tot-seq=1

GCTTAGAGGGTAAGAGCCACACTGCCTGGCTCTCTTGGCTTCCCTGTGCCTCTGTTTCTT

TATCTGTATAGTGTAAATAATAACAATCTGACTTAATAGGCTTCTTATGAGGGTTAAAGA

GAGAGGCATGTAAAACGTTAAAACTCGCCAGGCCCAAAGCAATGAATAGATATCGGCAGA

TGTGAAGATCATCTGTGCCCAGGCAAAAGCCAGGGATCTGGCTG

>8310 len=224 tot-seq=1

GGTGGTTGAGTTTGGCAACCACTTACCTTGATTCCATATAAAACGGTTACTTGGAGTGTA

TTGGTCATTCTGTTCCTGCTTCTCGGCCAACTTCCTGGCCAGAACACTGAGTTGCTGGGC

GTGGTGGGAAGGCGGGGAAAAGGAGAGGTTCGAGCCAACGTTTACGGGGGACTCCACTCT

GCCATTAGCCGTGGCTGCAGCATCGAGTTTGAGGGAACCAGCCT

>8311 len=224 tot-seq=1

GCTCCCGAGCAGCAGGCGGCGCTGCGCACACTGAACACCGAAGCGGCTACGCTGCAAAGT

CAGTTCCAGCAGCGACTGCTGGGAGCGGCAAAAAGCGGCGGCCTGGTGGTTGATTACAGG

CATCAACTGGCCGGCCTGAGCGATGAGGAGATAGCGGCGGCGGCTGACGCAGCGCGTGAA

AGAGGGCTTAGCGACCGCTGGTTGCTGACGCTGACCAATACCAC

>8312 len=224 tot-seq=1

GCAAGTGCGCGCCAGTGGGGGCCCACCTGCAGAAACTCTGTGTTAGGTGGAGTCTGCCAG

TGAATAAGCTATGGCAGTGGCCACTGGTATGTGGCTCTGCTAACTGCGGCCACATTACAG

GTTGGTGTGGCCAGGCAGAGACTCTGGGATAAACCAGCAGACAGAGGGGTGCTCAGATCA

AACTGCCTCTGTCCCACAGACAAGTTAGTCCTGCTTTATCCAGG

>8313 len=224 tot-seq=1

AAGGGCACGCAGGCGGTGACTTAAGTGAGATGTGAAAGCCCCGAGCTTAACTTGGGAATT

GCATTTCATACTGGGTCGCTAGAGTACTTTAGGGAGGGGTAGAATTCCACGTGTAGCGGT

GAAATGCGTAGAGATGTGGAGGAATACCGAAGGCGAAGGCAGCCCCTTGGGAATGTACTG

ACGCTCATGTGCGAAAGCGTGGGGAGCAAACAGGATTAGATACC

>8314 len=225 tot-seq=1

GAAGAGAGAGTCACTGGCCCTGGCTTGAGCTTTTGAAAACCTCAAAGCTCACCTGCATTG

ACATACTTCATCCAACAATGCCACTCCTCCTAATCCTTTCCAATAGTGCTACTCCCTGGT

GACCAGGCATTCAAATCTATGAGCCTAGGAAGGCCATTCTCATTTAAACCACCAGTTACT

TACTGAAGTGTGCCTTTGAAGGGTTTATCTTATCACCAGACCATT

>8315 len=225 tot-seq=1

TTCCCTGGCATCTCGTCCACGTAGGCACTGGTACATAATTTATAGACAGTGTACATTCCT

GTATCGTTGAGCTCGACTTTTGGCAGAGAACGGGGAAGCAGGTGACCTTGGTAATTAAAG

TCAGAATCAAAAAGAAATCCCAAATTAGTTCAAATTACACCTCAGGAATGCCCAGAGATG

CTCTCTTCCTCCTAGGCAACAACAGCTGTGCCCTCTCTCCTGAGG

>8316 len=225 tot-seq=1

TAGGGAATTTTTCACAATGGGCGCAAGCCTTATGAAGCAATGCCGCGTGAACGATGAAGG

TCTTATAGATTGTAAAGTTCTTTTATATGGGAAGAAACGCTAAAATAGGAAATGATTTTA

GTTTGACTGTACCATTTGAATAAGTATCGGCTAACTATGTGCCAGCAGGTAATCATAAGA

TCGGAAGAGCGTCGTGTAGGGAAAGAGGTCTTGTAGTGTAGATCT

>8317 len=225 tot-seq=1

CCCGAAAGCCGTTCCTATGGTGCAGCCTGTCAGCATGCCGCAGCGCAGACTGCACGTCAT

CACCATAAGAAGTGACAAAAGCCTGCCAACAGAAACGGCGTCAAAAATTTTCAGCCCGGA

CAGCGCCATGAGAAAGGCCGCCACCGTTATGAGGATGGAGCTTGCATGACGTATCTCCTC

GGCCTCGGTCGTATTCTCCCGTTCCGACAGCGCCTCGCGGAAAAA

>8318 len=225 tot-seq=1

ACAGCCGCCAAAATAACCAGCATGGTGTTGTAACATCCCCCCAGTGGGGGCTAGAATTCC

CCATGGTGACCTGTGACCTGCTCCCTGAGACAGGGGAGGCCAGGCAGGTCACGGTGGGGA

CGCAGCAGAACATTATTTCTCCATCTTGCTGTTTTCTAGCCTTGAGTTGGGGACATGAGT

GAGAACTGGGGGTTTTGAGGAGTAACCCTTGTGAACACATGTACT

>8319 len=225 tot-seq=1

GTGATGGGGACCATGAGGCTCCCCAGGGAGCCTGTGGGAGGGAGGCGCCTGGGCACAAGA

CCCAGGGACAAGGCTGCCAGTGTGCCCTGTGCCTTCTCTCCACAGCCGTGGTCTCCTCCT

CTGGGGGCTCGGGCTACAGCGGGGGCGGCGGCCTCTGCATGACCGGGGGCGGCTATGGCT

TTGGCAGCAGCGGCTTCAGCAGCAGCGGCGGTAGCTTCAGCTCCA

>8320 len=225 tot-seq=1

ACCAGAGAGGACGTCCCCAGCACCCCGAACGCTGCAATATTTTCATAAATGTAGAAAAAG

TAGTAGTGTTTCTCAAGCAACCTTTATTTACCCCCAGCTTTGCTTCTCTTTCTATGGAAG

GCTTCCTTATTGTCCCACGTGCTCCTGAATAATGCATGAGCCTCAGAGTCCGCCAGGCCA

GGAAGCACAAAGGCCCCGTGGTGGGGGCCCTTCCAGGCAGGCACC

>8321 len=225 tot-seq=1

AGAGTTGGAAGAGACATCTACAGATAAAATAAATAATCCAACCCTACTCATTACTATACA

TGAAGAAACTGACTTCCAGAGAAGTTGAAGGACATAAGTCACCCAACAATGGATGACCAG

AATCAGACTTTTCTCCAGTCCTCTTCTTGTTGGTTAATGGTAGGTTCCCTTTCTTCGTCC

CTAAGAAACTAGCTTTTCTTACACTTGTACCAACTCCTTTAATAG

>8322 len=226 tot-seq=1

AATCGCTGAAACCTGGGCGGCAGAGGTTGCAGTGAGCCAAGATTGTGCCACTGCCCTCCA

GCCTGGGCAACAAAGTGAGACTCCATCTCAAAAATAAAATAAAATAAAATAAGAAAAACC

TAAATTAGAACACTTGGAAAAGAGTTACTCTAAGACTCAATTTTTTACCTGTGAAGAAAA

AACTTCATCCCTGACCCTGGGTCCTGCTGATTCAAACGCAGTCAAG

>8323 len=226 tot-seq=1

CATACGATAGCGACGCGGGACGGGAGGGCAGACGGGGGCGAGGAAGAGAGAAGAAGGGAG

GAAGAAGGGAGGAAGGGGGAAAAAGGGACGAAAGGAGGAGGGAGAAAGACCAAGGGAACG

AGGAAGGGCGGCGGAGGGGAAAGGGCGGTTATTTAGGAAAAAAAGCGTGCTAGGAAAGGA

GCGCGCCTTGATGGTACTAATTGAATAAGGGACAGCGAACGATGTG

>8324 len=226 tot-seq=1

TTCAAAGCCAGCCTGGGTAACATAGCAAGACCCCCCCTCTCTAAAAAAAGCAAAAGCAAA

ACCAAAACAAAATGCTCAAGAGGACAAGGTTTGGGGAGCTTCCTAGTAGCTGAAGACCTG

GAGATTCCTGGAGGGCAGTGCGCCCAGGGAGGGCATGGAGGGTCCATGCTCCTTTTTCCA

TACCTCACCCTATGCATCTCTTCATCTGTAATATACTTTATAATCA

>8325 len=227 tot-seq=1

GTATCTTGAAGTGATAGGGGCTACAGTGAAGTTGCAGGGGTGGGTTCTGACGAAGGGTGA

TAGTGGATGGAGGCAGAGGTTTGGGAGACAGTTGGTTTGGCTTTGCTAATACCTTGCTGG

GATCTAAAGGGACCGTGGGTCCCATGAGGTCTCGATGGAGCAGTTGAATGGTAAACAGGA

GTCCCAGATCACAGCTAGAGATATAAAGTCTTAATCCCCATATCTTA

>8326 len=227 tot-seq=1

TTTTTTTTTTTCAAGCAGAAGACGGCATACGAGATGTAGCCGTGAATGGAGTTAAGAAGT

GTGATATTAAGATATAATAAGGGAGGAAGAAGTGGGGAATATTGAAAAATGGGAGAAAGA

ATGATGAAGAGAAGCCGAGTGAGCGAAGAAGTATTTCGGTATGTAAAGCTCTATCAGAAG

GGAAGAAAATGACGGTACCTTACTAAGAAGCTCCGGCTAAATACGTG

>8327 len=227 tot-seq=1

TCCCCAGTGCGCTCACGTCACTTGCTTTCATCTGTGGGAACTCCCAGGAAGGTGTGGGAG

GAGGAGGGTCGGCCAAGGGCACTCACATAGACGGCGAAGTCCTTGGGGGCGCTGCTGATG

TTGCCTGTTGGCGACAGCGTCTTAGGGATGTGCTCCAGAGTGAAGGCGGCTGGGTGGATC

ATCATGGAGAGCCTCACCACCAGGTACCCCTGGGAGCCTTTAAATGC

>8328 len=227 tot-seq=1

AGAAGACGGCATACGAGATTAGGCAGTGACTGGAGTTCAGACGTGTGCTCTTCCGATACG

GGAGGCAGCAGTGAGGAATATTGGTCAATGGGCGAGAGCCTGAACCAGCCAAGTCGCGTG

AAGGAAGACTGCCCGCAAGGGTTGTAAACTTCTTTTGTATGGGATTAAAGTCACCTACGT

GTAGGTGTTTGCAGTTACCATACGAATAAGCATCGGCTAACTCCGTG

>8329 len=227 tot-seq=1

CTCATATGTTGACAGCTCCCAATCCCCACTTCTGGAGCAGATTTTCCAGGGGGAAGGACA

TTACAGAGTCCGGATGCCTCTTCCATGATTCCAGCACCTCTAATGTTGTAAGGCCGTGTT

ATGAGCACCCTAAAGATGGCACTTCAGCACACGGTTCTCGAAATTCTGACCATTCTATCT

TCTTTTAAATCCCTCTGTTTTTCCAAATCCTAAAGCCCTGTGACACA

>8330 len=228 tot-seq=1

GAAGGGCCAACTGGGAAGGTAGACTAGGATAAGCAATTAAGGTGAATTGTCAGGTGACAA

TCTATTTCAGAAAGTTCAGATATTCGTTTTCTTATCACGACTAATGTGTCTAATCTCTCC

TACACAAGTTGGACCAAAGATTTAAAGATTTAAAGCCTGTGCTAACACTTCTAAAGGAAG

CCTAGCAAACAGGCAAGAGGCCAGGCATCATGGCCCATGCCTGTAATC

>8331 len=228 tot-seq=1

CAGTCCTCCTACTGTCACATACTGACCTTCCCGAATCCATGCCTACACTCTCGCCTTCTC

TCCCATTACAGAGGAAGACAAGCCTCTGTTCCTGTCTGAGGCAGCCTCTTTGCTTATACT

CTGGATTCCACTTCTCATCTACTCAAGGACTTTGTTCTCAAAGCACTCCACCTCCCACCT

TGCAGCATCCATTTCTCACTTTTCACACTTACTTGCATACAAAAATAT

>8332 len=228 tot-seq=1

CATAGTGATAGTAGTGGTGGGTTGGCAGGCACCCAAAACTCTGAGAGAGGGGAGCCCTTC

TCTCTAAGCAGAGGCATTGTGCTTCCAAGAGTGTGGGGCAAATCTCCATTGCTTTTTCTC

TCTGTCCTTCAGTCTGGCTCCAGATGCAGACACAGTCACAGGAAGGGCACAGCAGAAGAA

AGAAATTAAAGCCCCAGCTTTCTGGCTAGAGAACCAAAAAGGAAGGCC

>8333 len=228 tot-seq=1

GGAGGAAAGAGAAATGGGGGCTGTGGCTTTGTGTTCCTCTGGACTCTTCAAATGTGCAAA

GAAATAACTTAGCAAAGAGGATGCCAGGGAACAGAGGCTGGGCAGGTCAGGGTGTTTCCA

TTTTTGCAACCTCAGAGAGAAGCTGATGAATGAAATGGAGTGAATTCTCCTGGGAATACC

TGAGGTTGGCCATCACCCAGCTGTCCTCCCAGCACAGGAAACAGGCCC

>8334 len=228 tot-seq=1

TTTTTTTTTCAAGCAGAAGACGGCATACGAGATCTGATAGTGAATGGAGTTAAGAAGTGT

GATATTAAGATATAATAAGGGAGGAAGAAGTGGGGGATATTGAAAAATGGAGGAAAATAT

GATGAAGAAAAGAAGAGTGAGTGAAGAAGGCTTTCGAGTCGTAAAACTCTGTCGTATGGG

AAGAAAAAAATGACGGTACCATACAAGAAAGCCCCGGCTAACTACGTG

>8335 len=228 tot-seq=1

CATACGAGAGCGCCAGGGGACGGGAGGGCAGACGGGGGAGAGGAAGAGAGAAGAAGGGAG

GAAGAAGGAGGGAAGAGGAGGAAAGGGAAGGAAGGAGGAAAGAGAAAAGAAGAGGGAGGG

AAGAAGGGGGGAGGAGCGGAAAGCGCGGGGGTAAGAGAAGAACGAGTGGGAGAGGGGAAA

GGGCACACGGGGACGGGAGCGGACCAGAAAGGGACGGCGAACGACGTG

>8336 len=228 tot-seq=1

CGCTCTAGACTTCAGCCAGGGGCACAAATCCAACCTTTCCATTGAGCTCGAGGGCAGTAA

AGCAACAACAAAATCCTTCAATTTGGAGTCAGCTGGGCAGAGTGTTTCTTTCATTGAGAT

CGCTATGAAGGAGGCATACTGGAAGCTATTGGTGAACTCAAACATTTTCTATCTGGGAAA

GAACAATGCAAGGAAGACAGAAGAGAGCTGGGTCATACAATGCCAGAA

>8337 len=228 tot-seq=1

TGCAGTGAGTCGAGATCGCGCCACTGCACTCCAGCCTGGGCGACAGAGCGAAACTCCGTC

TCAAAAAAAAAAAAAAAAAAAAGAACTAAGGGTCCAACAGAGGCATCTCTGATCAAATCA

GAACCTTTTCATTTCACTCTGCCATACAAGATCATGCTTTGAGCCGTTGTTACTATGACG

TCTCGGAAAAGCGTTACAGAGAAACTTCATATTTATAATTTACTGTTG

>8338 len=228 tot-seq=1

ACCGCGGGAACAGCGGTTCGCAGGTAGAGAGCGGTTGCTCGGTCGCAAACAAGTCAAACC

TATTCAGGCTCGAAAAAGCCTTTTGCGCCTCTGTCAAGTGCAAAGCTGCCTGCATTTTTT

CAGCGGTCTCCGGCATAAATGGAGCAATATATATACTGACCAATCGCAGAACTTCACCTA

AATTGCGCAAAACCGCTGCCAGGCGTTCCTGCTTAGTCGGATCTTTAG

>8339 len=229 tot-seq=1

GTACGGAAGGAGAGGAAGCTGATCGCTGGGGGCTGGGAGAGCAATCCAGAGCCAGGGTGA

AGGGGGCAGCAGGGCAGGGCACTGAGGGGACGTGATCAGTCCCATAGGAAGAGGAGGAGG

AAGCCGGGGGGTCCCCGGTGGGTACCTGATGACCTCTCTGTGGCCATTCTTGGCAGCCAA

GTGCAGGGGCGTGGTGTAGTTGGGGTCGCACGGGTCCTTGGCCTCCCCC

>8340 len=229 tot-seq=1

CCGGCCAGGGCCATGAGCCCGGTGGAAATCCCCAGGATGCGCCGGGCCCCTTTCCGGTCA

TTCACTGCCGCCCGGCGGGAAATCAGGATGGACATGGCCGCCGGGACCCCGGCGGAGGAC

AGGGCTACCAGCAGCAGGTACAGGGGATAGGCGATCTGGTACAGCCCGATGCCCTCCCCG

CCCAGCAGCCGGGAAAGCAGGATCCGATTCACGGACCCCAGGATTTTTA

>8341 len=229 tot-seq=1

CACTTGAGCCCAGAAATTTGAGGCTGCAGTGAGTCATGATTGCGCCACTGTACCCCAGCC

TGGGTAACAGAGTGACACCCAGCCTTTTTATTTGTGGATAGCTCAAAGAAGTCATTTACA

AAATGCCTTTTGTTTTCAAAGCACAGCTTTTCATAAAAAGCAAGGCTCACATTTAATAGA

TGCTCTGGTACATAGGTGTGTGTTGAGAGCCGCTGGGGACTCAGGAAGC

>8342 len=229 tot-seq=1

TGGAGGCCTATTGCGAGTGCATGTTTGTGTGTGAGAGCATGCCCTCACGAGTGTGCAGGG

GTGTGTGGGGTGCGGTGTATACATGCACAGGTGTGTTCGTGTGTATGCAGGAGTGGGCGG

GTGCACAGACGTGCACCTGTGGGCAGGAGTGACCTCAGAGACTCCTTCCCGCAGATGCCT

CGGTCGCAGGGCCCCAGCCCGGCGTGGTTGGTGACCTCAGAGACTCCTT

>8343 len=230 tot-seq=1

GGTCATTCCTAAGAAGATCATAGAAGTGTCTCATTTAACTTAGAATTTTGTAAAAAGTAG

ATTGGTGGGCTATGGTACAGTGTGCAAGTCCAGAAACTATGCCTTATTTTAAAATAAAAA

ATTTCCCCTAATTTTAATGTAACATTTGTAAATTATGTTGTTGAAATTTAAAGCTGAACG

AAATAAGCATGGCGCATTGCCTTCAAAGCGCAGCATCCGTTCTTTAACGT

>8344 len=230 tot-seq=1

TGGTTTAGTCTTGCAGGGAATTCAGGGAAGCTTGGATTTGCTGAAGCTCTAGACGAGTTT

GGGCTGGGATATGGAAACAGCATGGAGCCGGGGCCCTTCTGCACGCTGGAGTCTGAGTAG

TTGCACCCTGGTGCAGCCATAGGTGTTCCCCACCTGGAGCACAGCTGTGGATAGAAGCTG

GGAGAGCTGTGGAGGGAAGAGGAGGAGGAGGAGGGAGTCTCAGGGCAGTC

>8345 len=230 tot-seq=1

GGTTTAAAGGGAGCGTAGGCTGGAGATTAAGTGTGTTGTGAAATGTAGACGCTCAACGTC

TGAATTGCAGCGCATACTGGTTTCCTTGAGTACGCACAACGTTGGCGGAATTCGTCGTGT

AGCGGTGAAATGCTTAGATATGACGAAGAACTCCGATTGCGAAGGCAGCTAACGGGAGCG

CAACTGACGCTTAAGCTCGAAGGTGCGGGTATCAAACAGGATTAGAAACC

>8346 len=230 tot-seq=1

AGCTACCAGGGCTGGTAGATAAAGACCACAGGGTAGGGGCAGGGATAGGCATATCTGAAT

TCAGTCTCTCCTTGGGCGGGGCTTGCTTCATTCAGCTCATGTGGAGGACGGGGTGTGGTT

CTCAGTCCAATGGAGTTATATTCCGAGGGTGATTATGGCTGCCTCTGCTTACTCATCCAG

GTTGCCAGGGAAGTAGGGGAAAGCCAGCAGTCACAGGCCTCACCCTGCTC

>8347 len=230 tot-seq=1

CCAAGGGAACGAGGGGCGGGGAGGGGAGGGTGTAGCCACCCAGGGGCTCCCACGCTGCCT

GAACAGCCAGCTGTCTACCCTGCAGCTGAAACAGCTGGAGCCAAGGGCCCTGGAAGACTA

TGATGGAGGGGCTCCCCGGCCCCCTGAGGCGTCTGCTGAATCCTGCAGGCAGCTCTGGCT

CTTTCTCTTCCAAAGCCAAGTCTCCTCCCTGGGCCCAAGGCCAAGTTTCC

>8348 len=231 tot-seq=1

TTGAGGTGAGCTTCGGAATGTGGCGGCATGCAGGACTTTCCACACTGTAGTCTAAGCCCT

TTCCTTTGCAGCAGCATTTTCCTGTACCAAGAGCTTTGCAATGTAAGCAACAAATGTCAG

TAGGAGCTACTAAACGTAGACCGAATCACTTAAAACTGGGTGCTATGGGTTGCAGTGATA

TTTCTCAGATTTTCATTACACTCTCTCCAGTACACACTGACCCAGATGGGC

>8349 len=231 tot-seq=1

TCAGAGCATTCCCAACCTTTCCTTTCAATGCTGTTCACAAAATAAAATGAGAAAGGATGC

CATCGGTCCCCAGCAAGGACACAAAGGTTTATATCTTATGTCACATTCAAGATCCTTGCC

TAAGGTGGACAGTTAAACTGCGTCATTTACAAAAATAATATGCTTTCAGCCGGCCTCAGC

TTTCTAAGATGAGTAAAACTCGAAAATGGAAACGGTTGATGCAATTTTAAA

>8350 len=232 tot-seq=1

GCAGGTCGTTGGGTCTTTGCAGACACTTAAGATCTTGGCTTCCTGTCCTAGGTGAGGAGG

AAGCCAGGATTTGCACCCAGGAGCAACATGCTGTGTGGCCAGTTGCTTCTCTGGAAATCA

GCCTCCACCACCTCTTCCCTGCCTCTGAGAAGGCTGGTGGAGGCCTGTCTTCCTGAGGGA

TCATTGCTTCTCGCCCTAGGTGTCTGCCCTAAGCGGCCGAGTGTTTGTAGAG

>8351 len=232 tot-seq=1

TGACTAGAACGTGGAAGACCTGGTGCTCAGGAAGCTCACAGTGTGGGACACCAAGTGTCA

GCAGTGGGTCTAGGAAGGGAGTCTGTCTTCTAGGAGGTTTGATAAGGGAGGGGCTTACGA

GGCAGTGACACTTGGGCAGAGACTGAAGTGAGGAAGCAGTCCTGTATATAGATGACTAGG

AGAAGACTGCAGACAGGGATTGGCCAATGAAAAGCCTGAGGCAGGAGTGTAC

>8352 len=232 tot-seq=1

TTGCAGTGAAACCCCGTCTCTACTAAAAATACAAAAAATTAGCCGGGCGTGGTGGCGGGC

ACCTGTAGTTCCAGCTACTCGGAGGCTGAGGCAGGAGAATGGCGTGAACCCGAGAGGCAG

AGCTTGCAGTAAGCTGAGATCGCGCCACTGCACTCCAACCTGGGCGACAGAGCGAGACGC

CGTCTCAAAAAAACCAAAAAACAAAAAAAAGAAGAAGAAGAAAGTATGTAAC

>8353 len=233 tot-seq=1

CAGTCTTACAGAATCCTTTGTGAGTTACTAACATTCAGAACCTCGTAGCAGTGTTCTGGA

ATCGTGTGTAAGGGACAAACTTTCTGAAACCAGCAGCAGTGTTTTGGAATCCCATGTGAG

GGAGAAACACTCAGAACCCAGCAGCAGGGTTCTGGAATCCTATGTGAGTGACAAACACTA

AGAAATCTGCAGCAGTTTTATGGAATCCTATGTGAGGGACAAACACTCAGAAC

>8354 len=233 tot-seq=1

TGGGTGTACAGGAACTCTCTGTACTTTGTGCTCAATTTTGCTGTGAACCTAAAACTGCTC

TAAAAAAGTCCATTTAAAAATTAAATCACATATACACACAGAGATATATTCAATGTTTAC

ATGGAATAGTTTAAAGAAAACCATACATTAAAGCTCCACAGTCATCAACATCCCTGGGAG

AAGTCACTGGGTAGGAGGTGGGAGAATGTAGAAACCCCAGGTAACACCAATGG

>8355 len=233 tot-seq=1

GTTCCTCCACAGGTCCTGTGGCTCACAATGCTCATGTCTGACTTGGCCTCACAGCTCCTG

GGACAGAACCTCTGAGAAGCTCTGGGAATGGCCAAAGTGGGTCCCCTAGGTTTTCTCTCC

CCCTTGTTGAAATGCCTGAGATTCCAGTATGATCATTTCACTCTTTGGGGAGAAGGTACT

GCTGACCCCTGATATAATGCACTGCCTCTGGGGTCATGCATGAAGCAATGAGT

>8356 len=234 tot-seq=1

GCACACAGGAGCAGGGACTCAGACTAGGTAGTCTACACCATGCTTTTCAAGAACAGAGAC

ATCGACTCTCCCATTTGGGCTTTTAGACTCCTGGGGGAAAAAAAGAGATTTCTACTTTGA

GTTTGGAAAGAAAAACAGACAAAACGGGTCATGAGCCAAGAAATGCAATATAAGGTCAAG

CCTATCTTGGAAACATTTCCCGTTGCATTATATTATTCTCTAGAGTAGACATCT

>8357 len=234 tot-seq=1

GAGGAGGGCCCTCGGGGTTCAGGCCCTCCAGGCACCCCATGCATGACAAACCGTCACACT

TGTGAAGTCGGAGTGGGGAAGCCCCCTCCCTGCCCTTTCAGGCAAGGGTGACCCTGAATA

ATGGTCTGTTCCTCCATGCCTCAGTTTCCTTGTCTGTAAAGGAGGCGACATCTACAATCT

AAAAGGACTGTGGGAATGTCAGCACAGTCAGCTCCTGCCCCGGGCTGGCCCCCA

>8358 len=235 tot-seq=1

ACTCTCTGACACTTCATTCCTGATTCTCTGTGCAGTTCTCTAGCAGCAACTCAAGGCCCA

TAAGTCAGGGAACCCCAACTTTGGCTGCATCTTCCCACCCCTGGAACAGTGTCTCAGCGC

TTCTCTGAAACCCAACAACACCTGACATGGAATAAGCCCAGTCAAGCTCTCCAGTGGGGC

GGAAGGCGGTCCCACACAGAACCAATGTTTCTAGGTTTTCTTGGAGGGCTTGGGA

>8359 len=235 tot-seq=1

GGGAAGGAAGGAGAAGTCCAGGATGTTTGGAAGCGCAGGGCATGGGTCTAGCTGGAGGCC

AGGCTTCACATGCAGACGTGATAAAAGATGAGGCTGAAGACAGAAGTCATAAGGACAGGC

CTCGTGGGCCAGCTTCAGGACGTGGTACTCCATTCTGACCAGGATGGGAGTGAGCAGCGT

CTGAAGGATTTTAGACAAGAGCACGGGGTCAGGTGTGCCCATCAGAAAGTTTGCT

>8360 len=236 tot-seq=1

GATCAGAAATTCAAGGTTATCCTTAGGTCCATGGTGAATTTGAACTGAACTTGGAATTTA

TGTCATTCTGCATCCAAAAAGGAATGCAGACCCCATAGCTCACAGTGCAAACCAGATGTC

TACCACCTGGTCAGGACACAGGGAAAATAAGCGGCTGCTTTGATTTAGAGATGCTTAGTA

GCCTGAGAAACGATAATTAAAGGTTCTGTGGATGTGTCCAGAAGTCCCTCCTCCGC

>8361 len=236 tot-seq=1

CATCATGATAGGGTGGTGTATTAGTCAGGGTTCTCTAGAGTCACAGAACTTATGGATAGT

CTCTATATAGTAAAAGAACTTATTGATGACTTACAGTCGGCAGCCCAATTCCCAACAATG

GTTCAGTCGCAGCTGTGAATGGAAGACCGAGGATCTAGCAGTTACTCAATCTCACACAGC

AAGCAGGAGAAGGAGCAAGAGCCAGACTCCCTTCTTTCAATGTCCTTATATTGTCT

>8362 len=237 tot-seq=1

GCTGGGCCACTTTCTGAGTCACAGAGGGGTGTGGGTGATGCCAGGAAGCCGAGCTGAAAA

TTGACCTTCTCCCTGAGGTCACTGCCAACACAGCGCTCATCACATGGCAAGCATTCGCCA

ATGGCTTAACCCGCTGCAAAGGAGAAGAGGGCAACTCGATGGGCATGTGATATGAAACTA

GCATTGGAAAATCCACCAGTCCCTCTCCCTAGCAAGAAGGACCAGCAGACAACATCC

>8363 len=238 tot-seq=1

ATGTGGCACACATATCAGAGTGCCTGGTGCTAATGAGTGAAGTAACTCAACAGAGGTCAC

ACAGCTAGTGAGAGCCAGGGCTGGGGTTCAAATTTAATAATTCTGGTAGCTAAGAGCATA

AGATTTAGGACCAATCTTGTATCTTGATTTCCTTACTTGTGAAATGGAGGTTGTCCTATC

TATCCTGCATCTCTGTGATGAGAAATAAATAATATTTGTAAAGCATTTAGTACAGCAC

>8364 len=239 tot-seq=1

CTGTGGACACATGGAGTCTGGCTGTTTGCTCAAGCAGCTTGTTTGTACTTTCTGGCCCTG

ATTGTGCCTGATCTCTAGTGTTCCACTTCTGTCGTATGCTTTTCATTTGGTTCGTCTGAA

GGAACAATGGCTTGCAGGCCAAATATGGATTATTTTTGTGAATAAAGTTTTATTGTAAGG

TAGCCACACCCTTTGTAATAATAATTCAAGTAATTATTGTCTACAGCAGCTTTGACACT

>8365 len=241 tot-seq=1

GCAAACAGCTTGTGTAAGGAAACTTCTGTTTTAAAAACCATCAGATTTCATGAGACTTAT

TCACTATCATGAGAAAAGCATGGGAAAGACCTGCCCTCATGATTTAATTACTTCCCACTA

GGTCCCTCCCACATGTGGGAATTCAAGATAAGATTTGGGTGGGGACAGAGCCAAACCATA

TCAATCCCCCCGGCCCCTCCCAAATCTCATGTCCTCATATTTCAAAACCAATCATGCCTC

C

>8366 len=241 tot-seq=1

GATGTGAGCAAATATTTGGGGGGTTCTTACTATGAGCCAGACATGGCATTCACAGGTAAG

AGCTGATCCTAACTCTCAAGTAACTCAGTTTCATTTTAAAAAGTCAAAAATACTCACAAT

AATTCATCATGAATACAGATGAGAGCAGCGTAACAGAGGCAGAGACCAACAGAGTGAGGA

GGAGAGATGGGCCGCTCTGTGGGCCTGGAAAGCTTGGCCACAGGGGCCTGCGCATGCCAT

G

>8367 len=241 tot-seq=1

GATTTTAACTTAGACTGCACCACCAGGAGGCACCCACTGAGGCATCAGAATCAGGAAGAG

GAGTGGCAGCCTCTATGCTTCCATCAGGAGCAGGCAGATGTGTGAGCTTCCACAGCCGGT

GGATATGGAGTGCGGGAAGCTGCCGCTTCCAAGGAGGTTCCTGCAGATCACCTGCGTTGG

TCCGCAGGTCGCTGAGATCATCAGCCAGCCATGTGTTCCTGCGATTCTTGACTTCCTGAA

A
